# Enantioselective Intermolecular C–H Amination Directed by a Chiral Cation

**DOI:** 10.1021/jacs.1c05206

**Published:** 2021-06-28

**Authors:** Alexander Fanourakis, Benjamin D. Williams, Kieran J. Paterson, Robert J. Phipps

**Affiliations:** Yusuf Hamied Department of Chemistry, University of Cambridge, Lensfield Road, Cambridge, CB2 1EW, United Kingdom

## Abstract

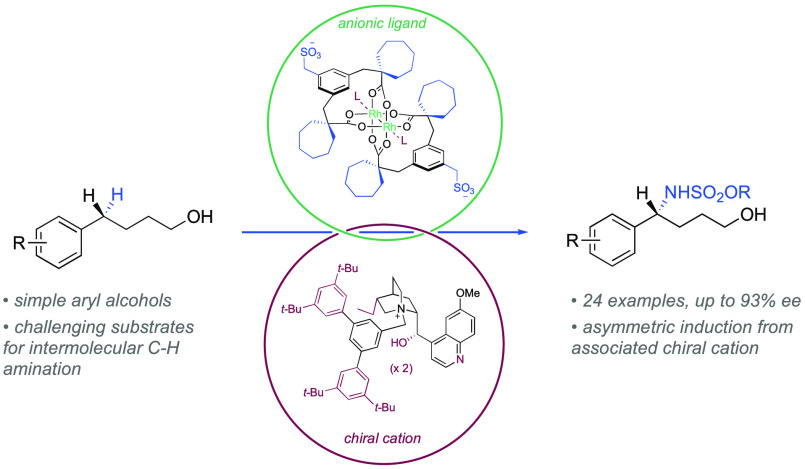

The enantioselective amination of C(*sp*^3^)–H bonds is a powerful synthetic transformation yet highly challenging to achieve in an intermolecular sense. We have developed a family of anionic variants of the best-in-class catalyst for Rh-catalyzed C–H amination, Rh_2_(esp)_2_, with which we have associated chiral cations derived from quaternized cinchona alkaloids. These ion-paired catalysts enable high levels of enantioselectivity to be achieved in the benzylic C–H amination of substrates bearing pendant hydroxyl groups. Additionally, the quinoline of the chiral cation appears to engage in axial ligation to the rhodium complex, providing improved yields of product versus Rh_2_(esp)_2_ and highlighting the dual role that the cation is playing. These results underline the potential of using chiral cations to control enantioselectivity in challenging transition-metal-catalyzed transformations.

The ability to form new C–N bonds in a direct and efficient manner is crucially important due to their ubiquity in organic molecules. Traditional disconnections are increasingly supplemented by methods which can use far less reactive C–H bonds, enabling powerful alternative retrosynthetic strategies.^1^ One of the most widely used and versatile involves the insertion of catalytically generated rhodium nitrenoids into C(*sp*^3^)–H bonds.^2^ The original catalysts used for this purpose were dirhodium tetracarboxylates (also referred to as paddlewheel complexes),^3^ and extensive development of this methodology has been undertaken by Du Bois and co-workers in particular.^2a^ This has culminated in the development of the versatile and robust strapped dicarboxylate catalyst Rh_2_(esp)_2_ which can perform rhodium-catalyzed C–H amination intermolecularly on benzylic, tertiary, and, in some cases, secondary alkyl C–H bonds (Figure 1a).^2a,4^ In many instances, C–H amination leads to the introduction of a new stereocenter and efforts to render Rh(II)-catalyzed C–H aminations enantioselective have been ongoing since its early development.^5^ Due to the ready availability of chiral carboxylic acids, their incorporation into paddlewheel complexes has constituted the main strategy, encompassing important contributions from Hashimoto,^6^ Müller,^7^ Davies,^8^ and Dauban,^9^ among others (Figure 1b, left). Additionally, Du Bois developed a chiral carboxamidate variant for enantioselective intramolecular amination (Figure 1b, middle).^10^ Despite these advances, as well as related ones employing alternative transition metals^11^ and enzymes,^12^ intermolecular C–H amination via nitrene transfer still remains extremely challenging to achieve asymmetrically. For rhodium dimers bearing chiral carboxylate ligands, the chiral information is located at a considerable distance from the reactive axial site. Although very successful for enantioselective carbene C–H insertions,^13^ for nitrene insertions the development of fundamentally different strategies is clearly warranted. Given that Rh_2_(esp)_2_ is the current state-of-the-art catalyst for nonenantioselective intermolecular C–H amination, the development of a chiral variant could be transformational but there are few structural opportunities to achieve this.^4b,14^ In a creative strategy, Bach and co-workers tethered the bridging aryl ring of (esp), through an alkyne linker, to a chiral lactam (Figure 1b, right).^15^ A dual hydrogen bonding interaction with the substrate permitted benzylic C–H amination with up to 74% *ee*.

**Figure 1:**
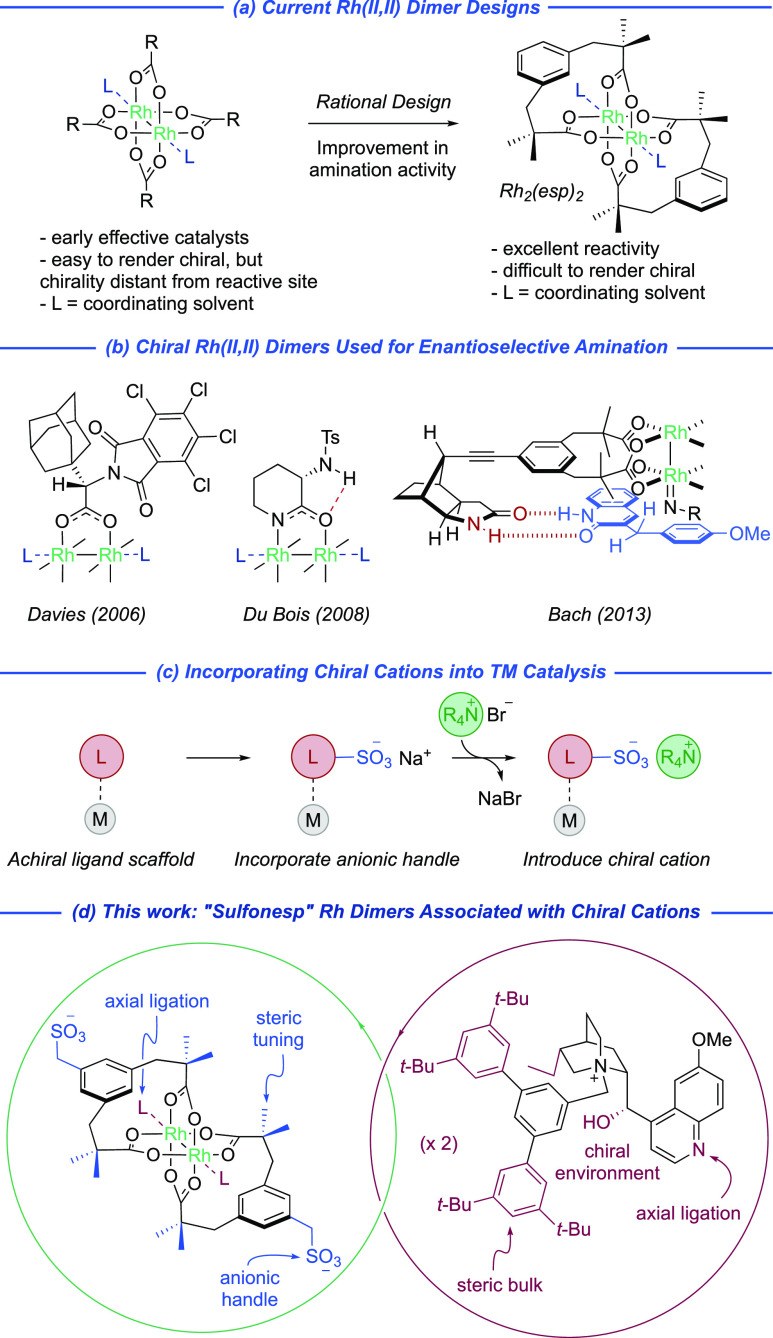
Background to enantioselective C–H amination using Rh dimers (a, b) and this work (c, d).

We recently outlined a strategy for inducing asymmetry in reactions that use ligand scaffolds which are particularly challenging to render chiral in the conventional manner. In our approach, the ligand is made anionic through the attachment of a sulfonate group which in turn allows association of a chiral cation with which to exert enantiocontrol (Figure 1c).^16^ This strategy provides an opportunity to unite privileged chiral cations with the diverse reactivity of transition metal complexes.^17^ In our first study a distally sulfonated bipyridine ligand was associated with a quinine-derived cation to impart enantiocontrol in iridium-catalyzed arene borylation.^18^ Quaternized cinchona alkaloids provide a well-defined chiral pocket with ample opportunity for attractive noncovalent interactions between the substrate and the rich functionality of the cation.^19^ Seeking to apply this strategy to C–H amination we first synthesized an (esp) analogue bearing a methylenesulfonate group on each bridging benzene ring. These anionic handles would then be used to associate with chiral cations to form a “sulfonesp” family of ion-paired catalysts (Figure 1d).

The “sulfonesp” scaffolds were readily synthesized in a three-step sequence comprising dialkylation using ester enolates, displacement of the remaining benzyl bromide with sodium sulfite, and ester hydrolysis to the corresponding diacid (Figure 2a). After assembly of the rhodium dimers, the bisligated complexes were isolated as the bistetrabutylammonium salts and it proved straightforward to introduce chiral cations via intermediate protonation using Amberlite IRC120 H. This accessed a series of “sulfonesp” scaffolds with varied geminal dialkyl substitution, both acyclic (**A**) and cyclic (**B**–**E**) (Figure 2b), a steric parameter on the ligand that we anticipated could be used to tune enantioselectivity. Notably, chirality is introduced in the final ion exchange, enabling rapid access to libraries of ion-paired catalysts.^20^ We initially synthesized the *gem*-dimethyl ligand scaffold (**A**) in combination with dihydroquinine-derived (**DHQ**-derived, **1**) and dihydroquinidine-derived (**DHQD**-derived, **2a**) cations that bore the specific bulky quaternizing benzyl group that had been optimal in our previous work (Figure 2c).^18^ Intriguingly, we observed a striking solution color change from green to red once the chiral cations were incorporated. This strongly suggested axial ligation of rhodium, with the quinoline nitrogen of the cations constituting the most likely ligand. UV–visible studies lent strong support to this hypothesis: comparing the λ_max_ of Rh_2_(**D**)_2_**·**(**2a**)_2_ (536 nm) with Rh_2_(esp)_2_ (655 nm) in 1,3-difluorobenzene suggests a significant difference in their respective HOMO–LUMO energy gaps (see Supporting Information (SI) for further details).^21^ While such binding could prevent nitrenoid formation if the binding in solution were too strong, we anticipated that a weaker, reversible interaction could actually be beneficial, potentially protecting the rhodium dimer from decomposition pathways and extending the catalyst lifetime, as has been shown in a number of recent studies.^4d,22^

**Figure 2:**
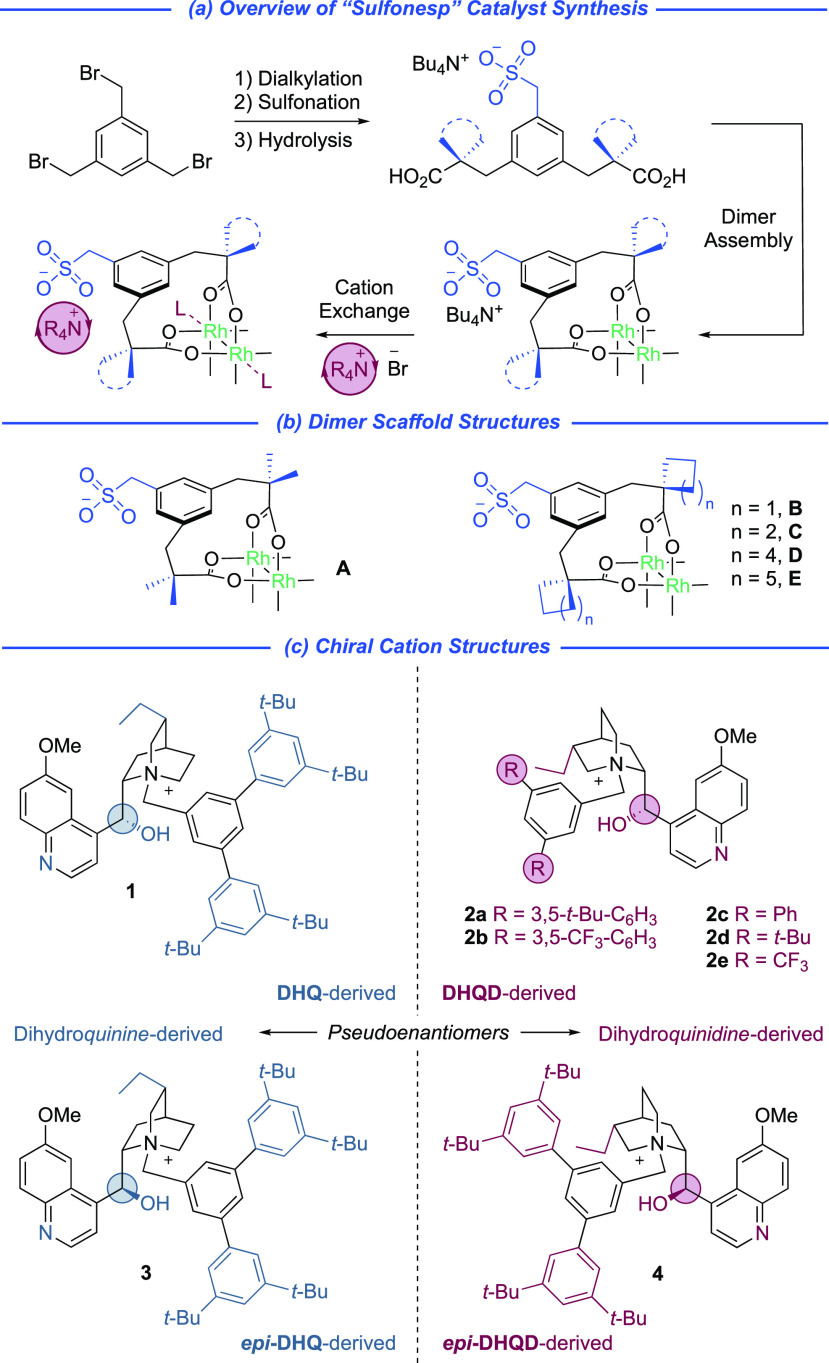
Synthesis (a) and systematic variation of ion-paired “sulfonesp” ligands on both ligand scaffold (b) and cation (c).

We began our investigations using 4-phenybutan-1-ol (**6a**) as a challenging test substrate (Table 1). This contains prochiral benzylic C–H bonds and a hydroxyl functionality that could feasibly hydrogen bond with the catalyst sulfonate group to provide organization at the transition state. The direct rhodium-catalyzed intermolecular amination of substrates containing unhindered primary alcohols has little precedent, yet the resulting chiral aminoalcohol derivatives could be of great synthetic utility, particularly since they are an oxidation away from γ-aminobutyric acids.^23^ Additionally, the transformation would give rise to products not currently accessible using Du Bois’ enantioselective intramolecular amination methodology involving cyclization of sulfamate esters en route to 1,3-amino alcohols.^10^ Although Rh_2_(esp)_2_ gave a very low yield (9%) on this substrate under the evaluation reaction conditions, we were pleased to observe that this increased significantly (43%) when Rh_2_(**A**)_2_**·**(**1**)_2_ was used. Further, a low but encouraging *ee* of 33% was measured (Table 1, entries 1 and 2). Remarkably, when using complex Rh_2_(**A**)_2_**·**(**2a**)_2_ containing the pseudoenantiomeric **DHQD**-derived cation, the *ee* increased drastically from 33% to −71% (entry 3). Such divergence in the enantiomeric excesses afforded by the pseudoenantiomers is intriguing but has been noted in other systems.^24^ This prompted us to evaluate another set of diastereomers of the cinchona alkaloid family, namely the *epi***-DHQ**-derived (**3**) and *epi***-DHQD**-derived (**4**) cations, in which the hydroxyl-bearing stereocenter is inverted on each. In these cases, the *ee* outcomes were poor (entries 4 and 5) so we continued optimization with the **DHQD**-derived cations. We were pleased to discover that switching the oxidant from PhI(OPiv)_2_ to iodosobenzene (PhIO) increased both conversion and *ee* (Table 1, entry 6). Despite the moderate yield, full conversion of starting material was observed along with a number of uncharacterized byproducts. A switch to the lower melting 1,3-difluorobenzene solvent enabled us to reduce the temperature to −25 °C, which in turn allowed for a more controlled reaction to give the product in an excellent 83% yield and −81% *ee* (entry 7). We next evaluated dimer scaffolds **B**–**E** to systematically explore steric changes near to the active site which we anticipated might lead to subtle variations in cation and substrate positioning in the enantiodetermining transition state (entries 8–11). This revealed that the cycloheptyl “sulfonesp” scaffold **D** provided both optimal yield (90%) and *ee* (−90%) in the complex Rh_2_(**D**)_2_·(**2a**)_2_. Finally, we returned to evaluate a selection of other quaternizing groups on the **DHQD** framework in conjunction with optimal scaffold **D**. Replacing the *t*-Bu groups at the periphery of the teraryl unit with CF_3_ (**2b**) or removing them completely (**2c**) was detrimental to the *ee* (entries 12 and 13), as was removing the outer two aryl rings of the teraryl unit (entries 14 and 15). Under the optimal conditions, the ion-paired catalysts greatly outperformed Rh_2_(esp)_2_, which delivered only a 17% yield (entry 16), underlining the importance of the chiral cation in improving reaction yield in addition to its pivotal role in enantioinduction. Indeed, when 4-phenylbutan-1-ol was subjected to the current state-of-the-art conditions for intermolecular amination using either PhOSO_2_NH_2_ or DfsNH_2_ as the aminating agents and Rh_2_(esp)_2_ as the catalyst, the desired product was not observed.^4d^ Only by use of the more reactive perfluorinated sulfamate ester **5** could ∼20% crude ^1^H NMR yield be obtained, emphasizing that **6a** is a challenging substrate for C–H amination (see SI).^25^

**Table 1:**
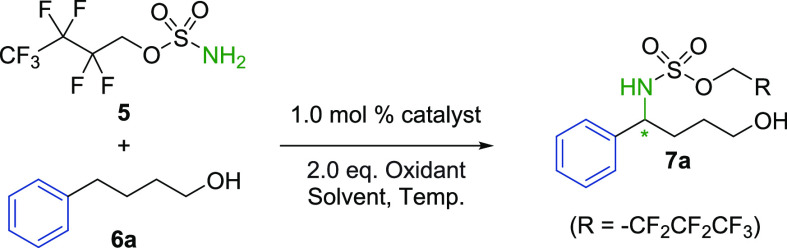
Reaction Optimization^a^

Entry	Catalyst	Oxidant	Temp (°C)	Solvent	Yield (%)	*ee*^b^ (%)
1	Rh_2_(**esp**)_2_	PhI(OPiv)_2_	–10	1,4-DFB	9	Rac.
2	Rh_2_(**A**)_2_·(**1**)_2_	PhI(OPiv)_2_	–10	1,4-DFB	43	33
3	Rh_2_(**A**)_2_·(**2a**)_2_	PhI(OPiv)_2_	–10	1,4-DFB	35	–71
4	Rh_2_(**A**)_2_·(**3**)_2_	PhI(OPiv)_2_	–10	1,4-DFB	13	Rac.
5	Rh_2_(**A**)_2_·(**4**)_2_	PhI(OPiv)_2_	–10	1,4-DFB	21	+26
6	Rh_2_(**A**)_2_·(**2a**)_2_	PhIO	–10	1,4-DFB	46	–87
7	Rh_2_(**A**)_2_·(**2a**)_2_	PhIO	–25	1,3-DFB	83	–81
8	Rh_2_(**B**)_2_·(**2a**)_2_	PhIO	–25	1,3-DFB	60	–81
9	Rh_2_(**C**)_2_·(**2a**)_2_	PhIO	–25	1,3-DFB	75	–87
10	Rh_2_(**D**)_2_·(**2a**)_2_	PhIO	–25	1,3-DFB	90^c^	–90^c^
11	Rh_2_(**E**)_2_·(**2a**)_2_	PhIO	–25	1,3-DFB	71	–86
12	Rh_2_(**D**)_2_·(**2b**)_2_	PhIO	–25	1,3-DFB	46	–53
13	Rh_2_(**D**)_2_·(**2c**)_2_	PhIO	–25	1,3-DFB	58	–76
14	Rh_2_(**D**)_2_·(**2d**)_2_	PhIO	–25	1,3-DFB	57	–82
15	Rh_2_(**D**)_2_·(**2e**)_2_	PhIO	–25	1,3-DFB	58	–74
16	Rh_2_(**esp**)_2_	PhIO	–25	1,3-DFB	17^c^	Rac.^c^

a Reactions performed on 0.1 mmol scale with respect to **6a** using 1.2 equivalents of **5**. Reaction concentration = 0.2 M. Yields determined by ^1^H NMR with reference to internal standard.

b *ee* determined by chiral SFC analysis of the crude reaction.

c Data corresponds to the isolated sample. DFB = difluorobenzene.

With the optimized conditions in hand we evaluated the tolerance to various arene substituents (Scheme 1). An ester at the *meta* position was well tolerated (**7b**) as were methyl groups at the *ortho* and *meta* positions (**7c**, **7d**). Substrates with fluorine atoms in all three positions also gave very high levels of enantioselectivity (**7e**–**7g**). With *meta*-substituted substrates (**7h**–**7l**), we were pleased to see that high enantioselectivity was maintained although in these cases, increasing the catalyst loading to 3.0 mol % was beneficial for conversion. Substitution at the *ortho* position with chlorine gave a reduced yield (**7m**) although the enantioselectivity remained high. Substitution at the *para* position gave a slightly reduced enantioselectivity in the case of methoxy (**7n**), but not chloride (**7o**). Finally, a range of disubstituted arenes (**7p**–**7v**) were compatible as well as naphthalene (**7w**) and thiophene (**7x**) containing substrates. For products **7q** and **7r**, a higher temperature was used for improved conversion and the solvent was switched from 1,3-difluorobenzene to 1,4-difluorobenzene since the latter had given a slightly improved enantioselectivity in initial reaction optimization (Table 1, entries 6 and 7).

**Scheme 1:**
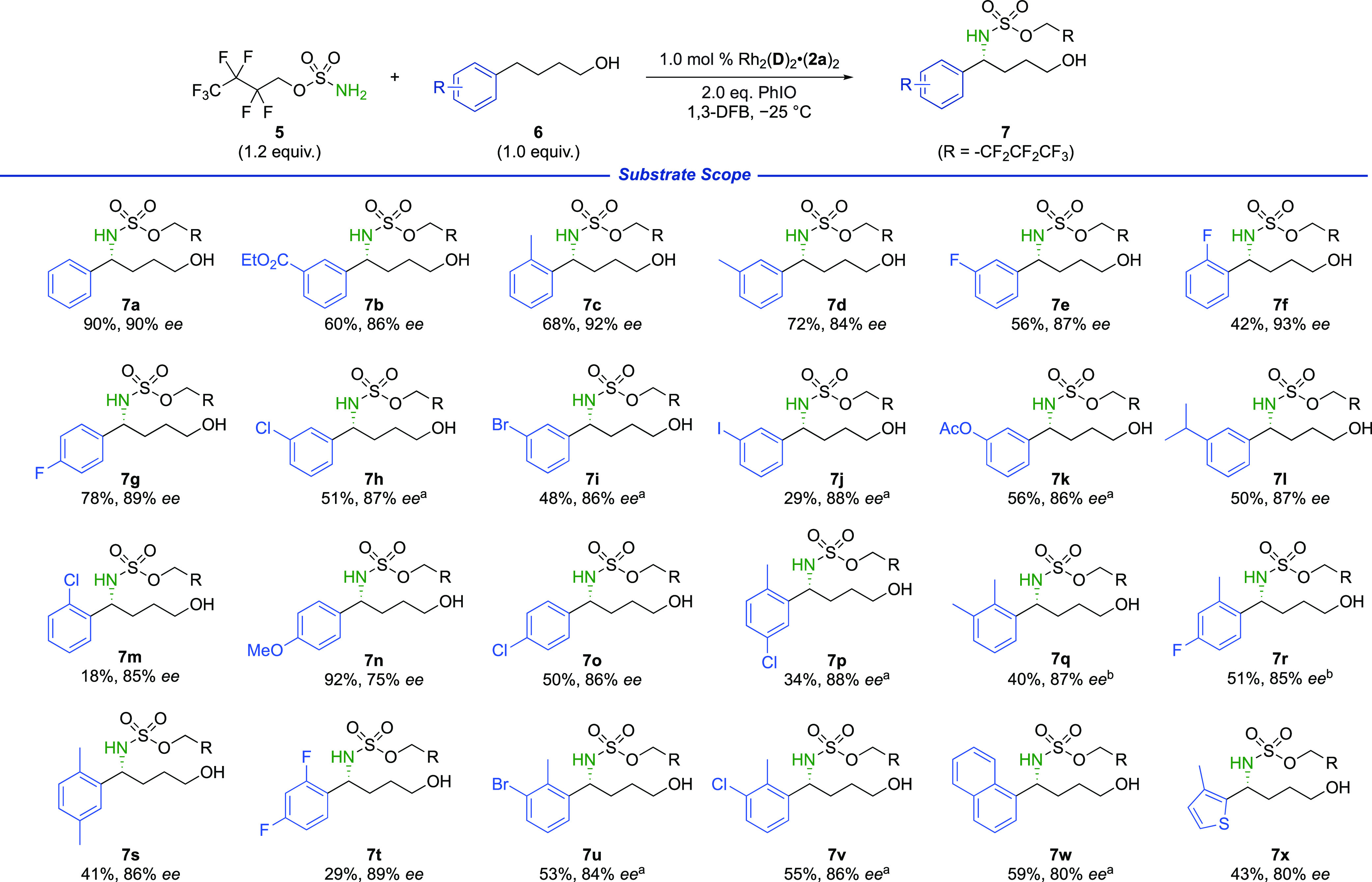
Reaction Scope Exploration Reaction performed with 3.0 mol % Rh_2_(**D**)_2_·(**2a**)_2_. Reaction performed at −10 °C using 1,4-DFB in place of 1,3-DFB.

Reaction product **7a** was readily transformed into protected 2-arylpyrrolidine **8** using Mitsunobu chemistry (Scheme 2a). *N*-Deprotection of **8** allowed assignment of the absolute stereochemistry of the products by comparison of the optical rotation of **9** with literature values (all other amination products were assigned by analogy). Our earlier observation that the precise diastereomer of the cinchona alkaloid scaffold used greatly impacted the enantioselectivity was curious, but also a practical limitation if the opposite product enantiomer is required. Assuming that the ethyl group in **DHQ**-derived **1** causes an unfavorable steric interaction at the transition state, we removed it by devinylation of quinine.^24^ We were pleased to find that the resulting catalyst Rh_2_(**D**)_2_**·**(**10**)_2_ gave the product *ent-***7a** with almost exactly the opposite sense of enantioinduction and with only a small reduction in yield (Scheme 2b). To probe the importance of the proposed hydrogen bonding between the substrate hydroxyl and the catalyst sulfonate, we evaluated the amination of phenylbutane (Scheme 2c, left). This showed drastically reduced reactivity and enantioselectivity suggesting that the attractive interaction is crucial for both outcomes. We also carried out the amination using Rh_2_(esp)_2_ in combination with **2a·Br** to examine the effect of severing the ionic link between ligand and cation. This resulted in poor enantioselectivity (19% *ee*, Scheme 2c, right). Interestingly, the yield was significantly improved compared with Rh_2_(esp)_2_ alone (51% vs 17%) which provides support for beneficial axial ligation by the quinoline of the cation, even when the cation is not associated with the ligand. Further support for this fortuitous benefit provided by the cinchona alkaloid-based chiral cations was provided by the poor yield (7%) obtained using an ion-paired ligand bearing tetrabutylammonium in place of the quinoline-containing chiral cation.

**Scheme 2:**
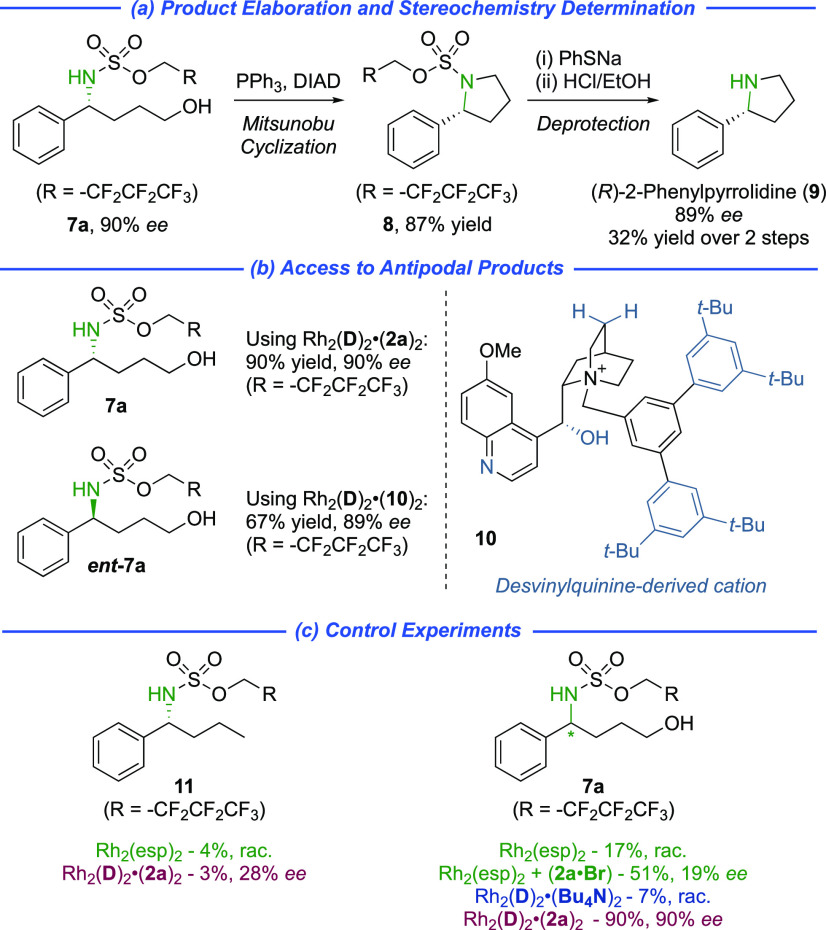
Practical Considerations and Control Experiments

We next tested substrate **12**, in which the hydroxyl is replaced by a carboxylic acid to evaluate its ability to interact productively with the catalyst. Here PhIO as the oxidant led to only racemic products, but switching to PhI(OPiv)_2_ afforded the product with an encouraging level of enantioenrichment (78% *ee*), albeit in low yield in these initial investigations (Scheme 3a). We also evaluated shorter (three carbon) and longer (five carbon) chain alcohols against our five “sulfonesp” scaffolds **A**–**E**, all using cation **2a**. This revealed that, for phenylpropanol, the same scaffold (**D**) that was optimal for phenylbutanol was best and cyclobutane-containing **B** was poorest (Scheme 3b, **14**). However, for the longer chain phenylpentanol, scaffold **B** was superior to all others (Scheme 3b, **15**). While still preliminary, these encouraging results provide a compelling demonstration that the modularity of our ion-paired “sulfonesp” ligands, in terms of both ligand scaffold and cation, will facilitate matching them with future substrates of interest. We also tested other functional groups in place of hydroxyl, which gave poor outcomes (see SI for details).

**Scheme 3:**
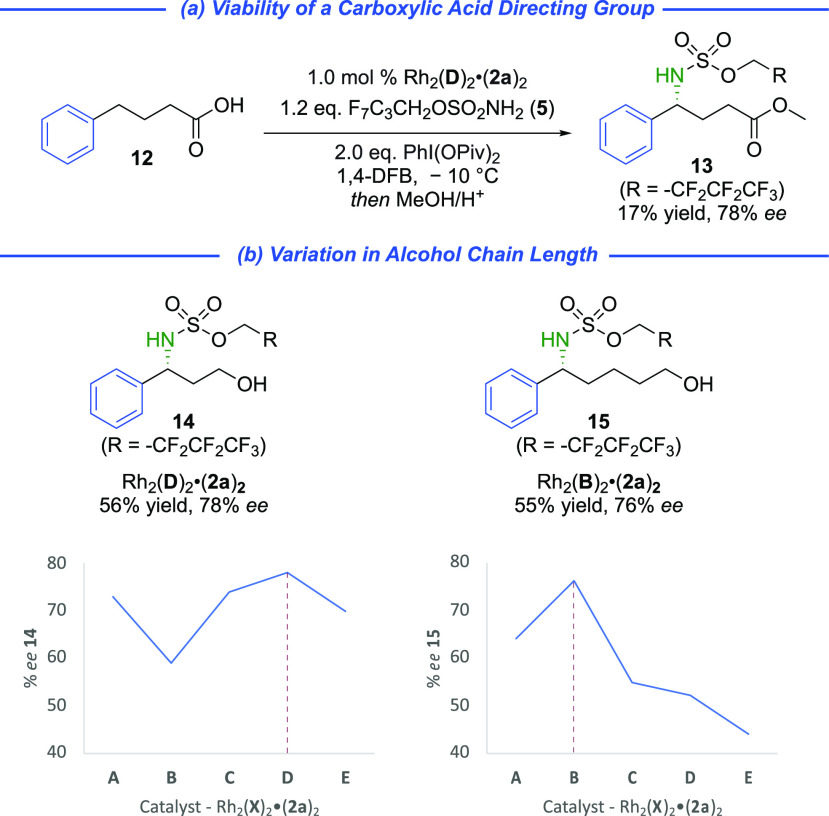
Further Substrate Exploration

In conclusion, we have developed a family of ion-paired chiral catalysts for rhodium-catalyzed C–H amination based on the (esp) ligand scaffold and have applied them successfully to the enantioselective intermolecular C–H amination of 4-arylbutanols. Furthermore, the optimal ion-paired catalyst also results in significantly improved yields compared with Rh_2_(esp)_2_. We believe that this is due to a combination of axial coordination by the chiral cation and a network of noncovalent interactions between ligand and substrate which promote the desired benzylic amination. These results form the basis of a catalyst design principle that we anticipate, with further development, should be applicable to intermolecular amination reactions of other challenging substrate classes. More broadly, this demonstrates the potential of using ion-paired ligands bearing chiral cations to tackle challenging transition-metal-catalyzed reactions.

## Supporting Information Available

Full experimental details, characterization data for compounds and details of UV–vis studies (PDF)
