# Supplementary material for: Enantioselective Intermolecular C–H Amination Directed by a Chiral Cation
Source: J Am Chem Soc. 2021 Jun 28;143(27):10070–6. doi: 10.1021/jacs.1c05206 (PMC8283762; doi:10.1021/jacs.1c05206)
Supplement: Supplementary file 1 — ja1c05206_si_001.pdf [file ja1c05206_si_001.pdf]

Enantioselective Intermolecular C-H Amination Directed by a Chiral Cation

Alexander Fanourakis, Benjamin D. Williams, Kieran J. Paterson and Robert J. Phipps*

Yusuf Hamied Department of Chemistry, University of Cambridge, Lensfield Road, Cambridge, CB2 1EW, United Kingdom.

Corresponding Author

*rjp71@cam.ac.uk

CONTENTS

General Experimental S3
General Protocols S6
Synthesis of Sulfonated Ligands and Achiral Rh(II,II) Dimers S11
Synthesis of Chiral Cation Bromide Salts S36
Synthesis of Chiral Rh(II,II) Dimers S55
Synthesis of Starting Materials for Enantioselective Intermolecular Amination S74
Enantioselective Intermolecular Amination Products S110
Unsuccessful Substrates for Enantioselective Intermolecular Amination S147
UV-Visible Spectroscopy Studies S150
Chiral SFC and Chiral HPLC Traces S153
NMR Spectra S184
References S470

General Experimental

Reaction Setup, Solvents and Reagents: All reactions were carried out under an inert argon or nitrogen atmosphere using standard Schlenk-septa techniques in heat gun-dried glassware unless otherwise stated. Reactions performed in 4.0 mL crimp-top vials that required cooling were placed in a Polar Bear Cub (by Cambridge Reactor Design) featuring a deep-welled heating block (IKA DB 5.2). All reagents were used as supplied from commercial sources without further purification unless otherwise stated. CuI was purified according to the procedure reported by Kauffman and Fang.[1] NaOH was finely ground. Tetrahydrofuran, Et_2O, MeOH, MeCN, CH_2Cl_2, *n*-hexane and toluene were purified by distillation on site under inert atmosphere *via* the following processes: tetrahydrofuran and Et_2O were pre-dried over sodium wire then distilled from calcium hydride and lithium aluminium hydride. MeOH, MeCN, CH_2Cl_2, *n*-hexane and toluene were distilled from calcium hydride. Iodosobenzene was routinely prepared on multi-gram scale according to the protocol described by Malcolmson and co-workers and stored in the dark at 3-4 °C.[2]

Chiral HPLC Analysis: Performed on a Shimadzu XR-LC system with DAICEL CHIRALPAK AD-H or IC columns (4.6 x 250 mm, 5.0 μm) in a mixed solvent system of hexane and ^{i}PrOH.

Chiral SFC Analysis: Performed on a Waters ACQUITY UPC2 System with YMC CHIRAL ART SB, SC, or SJ columns (4.6 x 250 mm, 3.0 μm), or DAICEL CHIRALPAK IG or IH columns (4.6 x 250 mm, 3.0 μm) in a mixed solvent system of supercritical CO_2 and MeOH or ^{i}PrOH. A system backpressure of 138 bar was used in all cases.

Chromatography: Analytical thin-layer chromatography was performed using precoated Merck glass backed silica gel plates (Silica gel 60 F254). Visualisation was by ultraviolet fluorescence (λ = 254 and 365 nm) and/or staining with potassium permanganate ($KMnO_4$) or Ceric Ammonium Molybdate (CAM). Flash column chromatography was performed using silica gel 60 (pore size: 60 Å, mesh: 40-63 μm) from Material Harvest®. All ratios of eluents are quoted as v/v. Silver nitrate impregnated upon silica gel was prepared according to the protocol described by Williams and Mander.[3,4]

High Resolution Mass Spectrometry (HRMS): Recorded on a Waters Micromass LCT Premier, a Waters Xevo G2-S or a Waters Vion IMS Qtof at the Department of Chemistry at the University of Cambridge. The ionisation method is noted—positive/negative electrospray ionisation (+/–ESI) or positive atmospheric solids analysis probe (ASAP+). Measured values are reported to 4 decimal places and are within ± 5 ppm of the calculated value. The calculated values are based on the most abundant isotope unless otherwise stated in the chemical formula. For ions bearing more than a single unit of charge, the masses reported as 'found' and 'required' are the mass/charge ratios.

IR Spectroscopy: Recorded on either a PerkinElmer Spectrum One FT-IR spectrophotometer sampling accessory or a Bruker ALPHA FT-IR Spectrometer, scanning from 4000-400 cm^{-1}. IR absorption maxima (ν_{max}) are reported in wavenumbers (cm^{-1}) of weak (w), medium (m) or strong (s) intensity and of broad (br) peak shape where appropriate.

NMR Spectroscopy: ^{1}H NMR spectra were recorded on 700 MHz TXO Cryoprobe, 600 MHz Bruker Avance DRX-600, 500 MHz Bruker DCH Cryoprobe, 400 MHz Bruker DPX-400 Dual, 400 MHz Avance III HD or 400 MHz Avance III HD Smart Probe spectrometers. Chemical shifts are reported in parts per million (ppm) and the spectra are calibrated to the resonance resulting from incomplete deuteration of the solvent ($CDCl_3$: 7.26 ppm; CD_3CN: 1.94 ppm, p; $(CD_3)_2CO$: 2.05 ppm, p; D_2O: 4.79 ppm; $(CD_3)_2SO$: 2.50 ppm, p; CD_3OD: 3.31 ppm, p; C_5D_5N (H–C–N): 8.74 ppm).[5,6] ^{13}C NMR spectra were recorded on the same spectrometers with complete proton decoupling. ^{13}C NMR experiments referenced as such were performed using a UDEFT sequence to increase the signal:noise ratio for ^{13}C signals of carbon nuclei along poly-fluorinated chains.[7] Chemical shifts are reported in ppm with the solvent resonance as the internal standard ($^{13}CDCl_3$: 77.16 ppm, t; $^{13}CD_3CN$: 1.32 ppm, sept; $(^{13}CD_3)_2CO$: 29.84 ppm, sept; $(^{13}CD_3)_2SO$: 39.52 ppm, sept; $^{13}CD_3OD$: 49.00 ppm, sept; $^{13}C_5D_5N$ (C–N): 150.35 ppm, t).[5,6] ^{19}F NMR spectra were recorded on 400 MHz Avance III HD or 400 MHz Avance III HD Smart Probe spectrometers. Chemical shifts are reported in ppm with $CFCl_3$ as the external standard ($CFCl_3$: 0.00 ppm). Data are reported as follows: chemical shift δ, multiplicity (s = singlet, d = doublet, t = triplet, q = quartet, p = pentet, sext = sextet, hept = heptet, br = broad, m = multiplet or combinations thereof (^{13}C and all other nuclides except ^{1}H are singlets unless otherwise stated)), coupling constants J, number of nuclides (signals for all other nuclides except ^{1}H refer to one nuclide unless otherwise stated), assignment. ^{1}H NMR spectra are

assigned as fully as possible, using ^{1}H-COSY, ^{1}H-NOESY, DEPT-135, HSQC and HMBC where appropriate to facilitate structural determination. Assignments either follow the numbering system shown on the structures or are described unambiguously. ^{1}H NMR signals are reported in ppm to 2 decimal places and all other nuclide signals to 1 decimal place. Coupling constants are reported in Hz to a maximum of 3 significant figures. For cinchona alkaloid-derived compounds the appearance and chemical shifts of the peaks in the NMR spectra can vary significantly depending on sample concentration and other factors. For spectra acquired in C_5D_5N the residual water peak is often visible at approximately 4.9 ppm in the ^{1}H NMR spectrum.

Optical Rotations: Measured in spectrophotometric grade $CHCl_3$ or C_5H_5N on a Perkin Elmer 343 Polarimeter using a sodium lamp (λ = 589 nm, D-line). $[\alpha]_D$ values are reported at the stated temperature, with concentration in g /100mL.

Naming and Numbering of compounds: Systematic names were generated by the computer program ChemDraw according to the guidelines specified by the IUPAC. However, the numbering on the structures does not correspond to the systematic name.

General Protocols

General protocol for the assembly of the achiral Rh(II,II) dimers from $Rh_2(TFA)_4$ (**GP1**)

A variant of the protocol described by Du Bois and co-workers was employed.[8] An oven-dried 2-necked round-bottomed flask was charged with $Rh_2(TFA)_4$ (1.0 equiv) and ligand (1.03 equiv.) and fitted with a reflux condenser. Chlorobenzene (approx. 0.02 M) was then added and the apparatus was evacuated until the solvent started to boil, following which it was backfilled with nitrogen. This was repeated a further nine times. The reaction mixture was then stirred at reflux for 6 hours. Following this, a further portion of ligand (1.03 equiv.) was added and the reaction mixture stirred at the same temperature for a further 6 hours. The reaction mixture was then allowed to cool and the solvent was removed under reduced pressure to dry the crude mixture directly onto silica gel. Purification by flash column chromatography (SiO_2, 0-100% v/v EtOH in $CHCl_3$) afforded the crude product. A ^{1}H NMR of the crude product often revealed an under-integration of the tetrabutylammonium cations relative to the dimer. As a result, ^{1}H NMR with an internal standard was used to determine the extra number of mmol (τ) of tetrabutylammonium cation required to ensure a 1:1.8 ratio of sulfonated dimer: tetrabutylammonium cation (90% of 1:2 to avoid excess tetrabutylammonium cation in the final product). Thus, the crude product was taken up in water (10 mL) following which the appropriate number of mmol of tetrabutylammonium hydrogensulfate (τ) and NaOH (τ) were added and the resulting biphasic mixture was shaken vigorously in a separating funnel, upon which the blue colour present in the aqueous layer was observed to move into the organic layer. The organic phase was reserved and the aqueous phase was extracted with a 3:1 mixture of $CHCl_3$/^{i}PrOH (2 x 10 mL). The combined organic layers were dried ($MgSO_4$) and the solvent removed under reduced pressure to afford the solvated product. Desolvation of the catalysts was achieved by heating the solvated products at 90 °C overnight under vacuum.

General protocol for the synthesis of quaternized cinchona alkaloids (**GP2**)

The protocol described by Phipps and co-workers was employed.[9] A crimp-top vial was charged with the cinchona alkaloid derivative (0.59 mmol, 1.0 equiv.) and the benzyl bromide

(0.59 mmol, 1.0 equiv.). The vial was sealed and evacuated and backfilled with nitrogen three times, following which dry THF (15 mL) was added. The reaction mixture was then heated at 75 °C overnight following which it was allowed to cool and the THF removed under reduced pressure. The crude residue was taken up in CH_2Cl_2 (20 mL) and washed twice with water (2 x 10 mL). The organic layer was dried ($MgSO_4$) and the solvent removed under reduced pressure. Purification by flash column chromatography afforded the title compounds.

General protocol for the synthesis of soluble chiral ion-paired Rh(II,II) dimers (**GP3**)

A sinter funnel was loosely packed with a pad of Amberlite® IRC120 H hydrogen form beads (approximately 6 cm column height) and equilibrated by flushing through with three column volumes of MeOH. Following this**,** a solution of the achiral rhodium dimer (0.041 mmol) in MeOH (4 mL) was loaded onto the pad and eluted with light suction. The eluent was then recycled through the pad a further four times. Following this, the eluent was evaporated to dryness and taken up in water (10 mL). Drops of 10% NaOH (aq.) were added until the solution was basic. A 3:1 mixture of $CHCl_3$/^{i}PrOH (20 mL) was added, followed by the chiral cation bromide salt (0.074 mmol). The biphasic mixture was stirred rapidly overnight during which the blue colour of the aqueous phase disappeared and a deep red colour was observed to move into the organic phase. The layers were separated and the aqueous phase extracted with a 3:1 mixture of $CHCl_3$/^{i}PrOH (3 x 20 mL). The organic fractions were dried ($MgSO_4$) and the solvent removed under reduced pressure. Desolvation of the catalysts was achieved by heating the solvated products at 90 °C overnight under vacuum.

General protocol for the synthesis of highly insoluble ion-paired chiral Rh(II,II) dimers (**GP4**)

A sinter funnel was loosely packed with a pad of Amberlite® IRC120 H hydrogen form beads (approximately 6 cm column height) and equilibrated by flushing through with methanol three times. Following this**,** a solution of the achiral rhodium dimer (0.041 mmol) in MeOH (approximately 4 mL) was loaded onto the pad and eluted with light suction. The eluent was then recycled through the pad and the procedure repeated a further four times. Following this, the eluent was evaporated to dryness and taken up in water (10 mL). Drops of 10% NaOH (aq.) were added until the solution was basic. A 3:1 mixture of $CHCl_3$/^{i}PrOH (20 mL) was added, followed by the chiral cation bromide salt (0.074 mol). The biphasic mixture was stirred rapidly overnight upon which an intractable red emulsion formed. The mixture was

evaporated to dryness and the crude solid residue placed on a sinter funnel and washed successively with water and plenty of ice-cold MeOH. The solid was then allowed to dry on the filter (washing with portions of Et_2O where necessary to remove traces of MeOH which were slowing the drying process), collected and desolvated by heating at 90 °C overnight under vacuum to afford the title compounds.

<u>First general protocol for Sonogashira coupling (**GP5**)</u>

A variant of the protocol described by Beak and co-workers was employed.[10] To a stirring solution of the aryl iodide (5.0 mmol, 1.0 equiv.), and but-3-ynol (1.25 equiv.), in Et_3N (30 mL) cooled to 0 °C were added freshly prepared CuI (2.0 mol %), Pd_2dba_3 (3.0 mol %) and Ph_3P (2.0 mol %) in sequence. The resulting mixture was stirred at 0 °C for 30 minutes and then allowed to warm to room temperature before heating to 70 °C overnight. The reaction mixture was then filtered through Celite®, eluting with plenty of $CHCl_3$. The filtrate was collected following which the solvent was removed under reduced pressure. Purification of the crude reside by flash column chromatography afforded the title compounds.

<u>Second general protocol for Sonogashira coupling (**GP6**)</u>

To a solution of aryl iodide (5.0 mmol, 1.0 equiv.) in MeCN (30 mL) at room temperature was added Et_3N (5.1 equiv.). Following this, $(Ph_3P)_2PdCl_2$ (4.5 mol %) and freshly prepared CuI (9.1 mol %) were added. The resulting mixture was stirred at room temperature for 20 minutes following which but-3-ynol (1.20 equiv.) was added and stirring continued at room temperature overnight. The solvent was then removed under reduced pressure. Purification of the crude reside by flash column chromatography afforded the title compounds.

<u>General protocol for alkyne hydrogenation (**GP7**)</u>

To a solution of the alkyne (5.0 mmol, 1.0 equiv.) in EtOH (30 mL) was added Pd/C (10 wt % loading Pd) (3.6 mol %). The reaction flask was then capped with a fresh suba seal following which it was evacuated and backfilled three times with hydrogen gas. The reaction mixture was then allowed to stir at room temperature overnight under two balloons of hydrogen. The reaction was then filtered through Celite®, eluting with plenty of EtOAc. The filtrate was collected, the solvent removed under reduced pressure and purification of the crude reside by flash column chromatography afforded the title compounds.

General protocol for the alkylation reaction of benzyl bromides (**GP8**)

A variant of the protocol described by Yang and co-workers was employed.[11] To a solution of the benzyl bromide (1.0 equiv.) in THF (5 mL) cooled to 0 °C was added dropwise allylmagnesium bromide solution (1 M Et_2O, 1.2 equiv.). The resulting mixture was then allowed to warm to room temperature and stirred overnight. The reaction was quenched *via* addition of NH_4Cl (sat. aq.), the aqueous layer extracted with CH_2Cl_2, and the combined organic extracts dried ($MgSO_4$), filtered through a pad of Celite®, and concentrated *in vacuo*. The crude was then used with no further purification.

General protocol for the alkylation reaction of benzyl chlorides (**GP9**)

A variant of the protocol described by Yang and co-workers was employed.[11] To a solution of the benzyl chloride (1.0 equiv.) in THF (5 mL) cooled to 0 °C was added dropwise allylmagnesium bromide solution (1 M Et_2O, 1.8 equiv.). The resulting mixture was then allowed to warm to room temperature and stirred overnight. The reaction was quenched *via* addition of NH_4Cl (sat. aq.), the aqueous layer extracted with CH_2Cl_2, and the combined organic extracts dried ($MgSO_4$), filtered through a pad of Celite®, and concentrated *in vacuo*. The product was then used with no further purification.

General protocol for the hydroboration of terminal alkenes (**GP10**)

A variant of the protocol described by Breinbauer and co-workers was employed.[12] To a solution of the terminal alkene (1.0 equiv.) in hexane (20 mL) cooled to 0 °C was added dropwise 9-BBN solution (0.5 M THF, 0.75 equiv.). The resulting mixture was then allowed to warm to room temperature and stirred overnight. 2.5 M NaOH (1.0 equiv.) and H_2O_2 (30% w/v in H_2O, 3.8 equiv.) were then added, and the reaction heated to 50 °C and monitored by TLC. Upon completion the reaction was allowed to cool to room temperature, the layers separated, and the organic layer sequentially washed with $Na_2S_2O_3$ (sat. aq.), water, and brine. The combined aqueous extracts were saturated with Na_2CO_3, filtered, and then re-extracted with Et_2O. **All** organic fractions were then combined, dried ($MgSO_4$), filtered, concentrated *in vacuo*, and the product isolated by flash column chromatography. (*Note: Yields were calculated relative to the limiting 9-BBN reagent*).

General protocol for the reduction of benzoic acids (**GP11**)

The protocol described by Chen, Vasdev, Liang and co-workers was employed.[13] To a solution of the benzoic acid (1.0 equiv.) in THF (35 mL) cooled to 0 °C was added dropwise BH_3.THF solution (1 M THF, 3.0 equiv.). The resulting mixture was allowed to warm to room temperature and stirred vigorously overnight. Upon completion the solution was cooled to 0 °C, quenched *via* dropwise addition of water, and the solvent removed *in vacuo*. The resulting residue was taken up in water and a small amount of brine, extracted with Et_2O, and the combined organic extracts washed with brine, dried ($MgSO_4$), filtered, concentrated *in vacuo*, and the product isolated by flash column chromatography.

General protocol for conversion of the benzyl alcohols to benzyl bromides (**GP12**)

To a solution of the benzyl alcohol (1.0 equiv.) in CH_2Cl_2 (30 mL) cooled to 0 °C was added dropwise PBr_3 (1.0 equiv.). The resulting mixture was stirred at 0 °C for a further 2 h, and then allowed to warm to room temperature and stirred until completion. The reaction mixture was then poured onto ice water, the pH adjusted to basic *via* the careful addition of $NaHCO_3$ (sat. aq.), and then extracted with CH_2Cl_2. The combined organic extracts were washed with brine, dried ($MgSO_4$), filtered, and concentrated *in vacuo*. The crude was then used with no further purification.

General protocol for intermolecular amination reactions (**GP13**)

Under an atmosphere of air, a 4.0 mL crimp-top vial was charged with the alcohol (0.1 mmol, 1.0 equiv.) and catalyst (1 mol %). A solution of 2,2,3,3,4,4,4-heptafluorobutyl sulfamate (**5**) (0.12 mmol, 1.2 equiv.) in 1,3-difluorobenzene (0.5 mL, 0.2 M) was then added *via* syringe. The vial was then cooled to – 25 °C over 10 minutes. Following this, iodosobenzene (0.2 mmol, 2.0 equiv.) was added in a single portion. The vial was then capped and the reaction mixture stirred at the same temperature overnight. Sat. aq. thiourea (1 mL) and $CHCl_3$ (1 mL) were then added and the biphasic mixture was stirred vigorously for 5 minutes at – 25 °C. The mixture was then allowed to warm to room temperature and stirred for a further 15 minutes. The layers were separated and the aqueous layer extracted with $CHCl_3$ (3 x 1 mL). The combined organic layers were dried ($MgSO_4$), filtered and concentrated. Purification by flash column chromatography afforded the title compounds.

Synthesis of Sulfonated Ligands and Achiral Rh(II,II) Dimers

Dimethyl 3,3'-(5-(bromomethyl)-1,3-phenylene)bis(2,2-dimethylpropanoate)

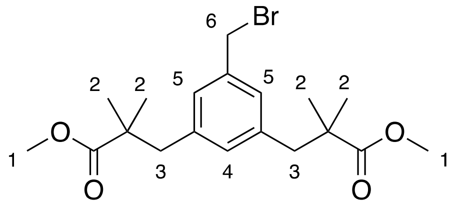

To a solution of $^{i}Pr_2NH$ (4.2 mL, 30.0 mmol) in THF (25 mL) at 0 °C was added *n*-BuLi (18.8 mL of a 1.6 M solution in hexanes, 30.0 mmol) *via* syringe pump over 15 minutes. The solution was stirred at 0 °C for 5 minutes following which methyl isobutyrate (3.8 mL, 33.0 mmol) was added dropwise over 5 minutes. The above solution was stirred for 10 minutes then added *via* syringe pump over 20 minutes to a solution of 1,3,5-tris(bromomethyl)benzene (5.35 g, 15.0 mmol) in THF (50 mL) at 0 °C. The reaction mixture was stirred at 0 °C for a further 20 minutes and then quenched by the addition of saturated aqueous NH_4Cl solution (30 mL). The THF was then removed under reduced pressure. The crude product was extracted with EtOAc (3 × 60 mL) and the combined organic layers were washed with brine (30 mL), dried ($MgSO_4$) and the solvent removed under reduced pressure. Purification by flash column chromatography (SiO_2, 0-10% v/v EtOAc in hexane) afforded the title compound as a colourless oil (2.47 g, 6.2 mmol, 41%).

***R_f* value** = 0.33 (10% v/v EtOAc in hexane);

^{1}H NMR (400 MHz, $CDCl_3$) δ 6.98 (d, J = 1.5 Hz, 2H, H-5), 6.79 (s, 1H, H-4), 4.42 (s, 2H, H-6), 3.66 (s, 6H, H-1), 2.81 (s, 4H, H-3), 1.16 (s, 12H, H-2) ppm;

^{13}C NMR (101 MHz, $CDCl_3$) δ 177.9, 138.3, 137.3, 132.4, 129.1, 51.9, 46.2, 43.8, 33.8, 25.1 ppm;

IR (neat, cm^{-1}): ν_{max} 2948 (w), 1729 (s), 1603 (w), 1456 (w), 1194 (m), 1124 (m), 983 (w), 857 (w);

HRMS (+ESI) found $[M+H]^+$ 399.1158, $[C_{19}H_{28}BrO_4]^+$ requires 399.1165, (δ = – 1.8 ppm).

Tetrabutylammonium (3,5-bis(3-methoxy-2,2-dimethyl-3-oxopropyl)phenyl)methanesulfonate

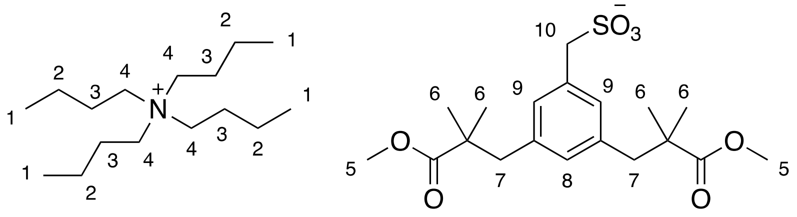

A variant of the protocol described by Phipps and co-workers was employed.[14] To a solution of dimethyl 3,3'-(5-(bromomethyl)-1,3-phenylene)bis(2,2-dimethylpropanoate) (1.06 g, 2.65 mmol) in a 2:3 mixture of acetone/water (13 mL) was added Na_2SO_3 (401 mg, 3.18 mmol). The resulting solution was heated to reflux for 2 hours and then allowed to cool to room temperature. The volatiles were removed under reduced pressure and the crude mixture was taken up in water (10 mL) and washed with Et_2O (10 mL). The aqueous layer was reserved, following which freshly ground NaOH (84.7 mg, 2.1 mmol), Bu_4NHSO_3 (720 mg, 2.1 mmol) and CH_2Cl_2 (30 mL) were added. The biphasic mixture was shaken vigorously in a separating funnel and the layers separated. The aqueous layer was extracted with CH_2Cl_2 (3 x 15 mL). The combined organic layers were then washed once with water (10 mL), dried ($MgSO_4$) and the solvent removed under reduced pressure to afford the title compound as a colourless oil (1.21 g, 1.88 mmol, 71%).

^{1}H NMR (400 MHz, $CDCl_3$) δ 7.06 (d, J = 1.5 Hz, 2H, H-9), 6.68 (br s, 1H, H-8), 3.98 (s, 2H, H-10), 3.65 (s, 6H, H-5), 3.18-3.23 (m, 8H, H-4), 2.77 (s, 4H, H-7), 1.54-1.62 (m, 8H, H-3), 1.36 (sext, J = 7.4 Hz, 8H, H-2), 1.13 (s, 12H, H-6), 0.96 (t, J = 7.2 Hz, 12H, H-1) ppm;

^{13}C NMR (101 MHz, $CDCl_3$) δ 178.3, 136.8, 134.4, 130.8, 130.4, 58.8, 57.9, 51.8, 46.2, 43.7, 25.0, 24.1, 19.8, 13.8 ppm;

IR (film, cm^{-1}): ν_{max} 2962 (m), 2874 (w), 1727 (s), 1602 (w), 1459 (m), 1386 (w), 1219 (s), 1191 (s), 1126 (s), 1034 (s), 883 (w), 856 (w);

HRMS (+ESI) *m/z* For cation found 242.2847, $[C_{16}H_{36}N]^+$ requires 242.2842, (δ = + 2.1 ppm).

HRMS (−ESI) *m/z* For anion found 399.1472, $[C_{19}H_{27}O_7S]^-$ requires 399.1483, (δ = – 2.8 ppm).

Tetrabutylammonium (3,5-bis(2-carboxy-2-methylpropyl)phenyl)methanesulfonate (**A•Bu$_4$N**)

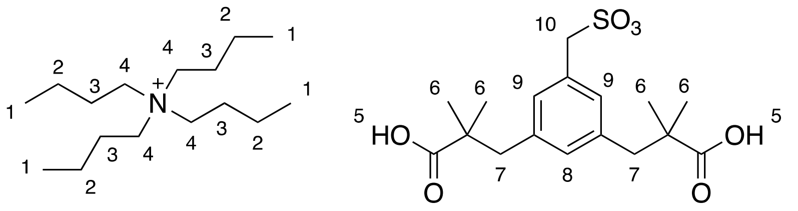

A round-bottomed flask fitted with a condenser was charged with tetrabutylammonium (3,5-bis(3-methoxy-2,2-dimethyl-3-oxopropyl)phenyl)methanesulfonate (2.49 g, 4.1 mmol) and freshly ground NaOH (1.13 g, 28.4 mmol). A 1:1 mixture of MeOH/H_2O (30 mL) was added and the reaction mixture stirred at 50 °C overnight. The MeOH was removed under reduced pressure and the aqueous layer was acidified with 3 M HCl and extracted with a 3:1 mixture of $CHCl_3$/^{i}PrOH (5 x 30 mL). The combined organic layers were dried ($MgSO_4$) and the solvent removed under reduced pressure. Extensive cycles of drying under vacuum and scratching the crude oil eventually afforded the title compound as an off-white amorphous solid (1.38 g, 2.2 mmol, 55%).

^{1}H NMR (400 MHz, MeOD) δ 7.11 (d, *J* = 1.1 Hz, 2H, H-9), 6.93 (s, 1H, H-8), 3.98 (s, 2H, H-10), 3.21-3.26 (m, 8H, H-4), 2.83 (s, 4H, H-7), 1.62-1.70 (m, 8H, H-3), 1.42 (sext, *J* = 7.5 Hz, 8H, H-2), 1.14 (s, 12H, H-6), 1.03 (t, *J* = 7.3 Hz, 12H, H-1) ppm;

^{13}C NMR (101 MHz, MeOD) δ 181.5, 138.8, 133.7, 132.2, 131.8, 59.5 (t, J_{C-N} = 2.9 Hz), 58.5, 47.0, 44.4, 25.5, 24.8, 20.7 (t, J_{C-N} = 1.5 Hz), 13.9 ppm;

IR (film, cm^{-1}): ν_{max} 2963 (br, s), 1717 (s), 1575 (s), 1475 (s), 1407 (s), 1351 (s), 1250 (s), 1171 (s), 1038 (s), 958 (s), 883 (s);

HRMS (+ESI) *m/z* For cation found 242.2846, $[C_{16}H_{36}N]^+$ requires 242.2842, (δ = + 1.7 ppm).

HRMS (−ESI) *m/z* For anion found 371.1161, $[C_{17}H_{23}O_7S]^-$ requires 371.1170, (δ = − 2.4 ppm).

Bis[rhodium tetrabutylammonium (3,5-bis(2-carboxy-2-methylpropyl)phenyl)methanesulfonate)] (Rh_2(**A**)$_2$•(**Bu$_4$N**)$_2$)

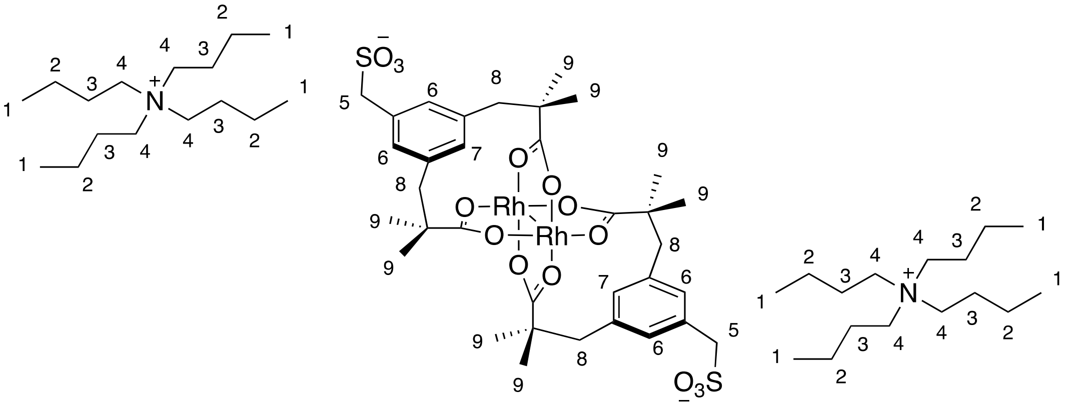

Prepared according to **GP1** on a 1.1 mmol scale with respect to $Rh_2(TFA)_4$ and using tetrabutylammonium (3,5-bis(2-carboxy-2-methylpropyl)phenyl)methanesulfonate (**A•Bu$_4$N**) as the ligand. The title compound was obtained as a green amorphous solid (1.37 g, 0.96 mmol, 88%).

^{1}H NMR (600 MHz, MeOD) δ 6.91 (s, 2H, H-7), 6.88 (s, 4H, H-6), 3.92 (s, 4H, H-5), 3.21-3.24 (m, 16H, H-4), 2.60 (s, 8H, H-8), 1.66 (p, J = 7.8 Hz, 16H, H-3), 1.41 (sext, J = 7.4 Hz, 16H, H-2), 1.03 (t, J = 7.2 Hz, 24H, H-1), 0.96 (s, 24H, H-9) ppm;

^{13}C NMR (101 MHz, MeOD) δ 196.9, 139.3, 132.6, 131.4, 131.3, 59.5 (t, $J_{C\text{-}N}$ = 2.8 Hz), 58.6, 47.9, 46.7, 26.3, 24.8, 20.7 (t, $J_{C\text{-}N}$ = 1.5 Hz), 13.9 ppm;

IR (neat, cm^{-1}): ν_{max} 2963 (s), 1585 (s), 1456 (w), 1404 (s), 1218 (m), 1162 (s), 1028 (s), 882 (w);

HRMS (+ESI) *m/z* For cation found 242.2838, $[C_{16}H_{36}N]^+$ requires 242.2842, (δ = − 1.7 ppm).

HRMS (−ESI) *m/z* For dianion found 472.0058, $[C_{34}H_{42}O_{14}Rh_2S_2]^{2-}$ requires 472.0069, (δ = − 2.3 ppm).

Diethyl 1,1'-((5-(bromomethyl)-1,3-phenylene)bis(methylene))bis(cyclobutane-1-carboxylate)

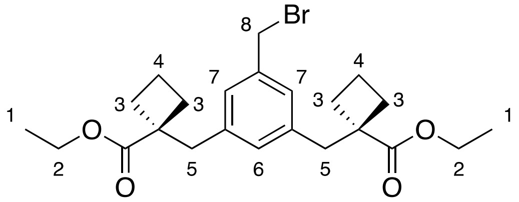

To a solution of $^{i}Pr_2NH$ (2.0 mL, 14.0 mmol) in THF (15 mL) at 0 °C was added *n*-BuLi (8.8 ml of a 1.6 M solution in hexanes, 14.0 mmol) *via* syringe pump over 10 minutes. The solution was stirred at 0 °C for 5 minutes following which ethyl cyclobutanecarboxylate (1.9 mL, 14.0 mmol) was added dropwise over 5 minutes. The above solution was stirred for 10 minutes and then added *via* syringe pump over 15 minutes to a solution of 1,3,5-tris(bromomethyl)benzene (2.50 g, 7.0 mmol) in THF (25 mL) at 0 °C. The reaction mixture was stirred at 0 °C for a further 20 minutes and then quenched by the addition of saturated aqueous NH_4Cl solution (30 mL). The THF was then removed under reduced pressure. The crude product was extracted with EtOAc (3 × 40 mL) and the combined organic layers were washed with brine (30 mL), dried ($MgSO_4$) and the solvent removed under reduced pressure. Purification by flash column chromatography (SiO_2, 0-9% v/v Et_2O in hexane) afforded the title compound as a colourless oil (674 mg, 1.5 mmol, 21%).

***R**$_f$* **value** = 0.19 (10% v/v Et_2O in hexane);

^{1}H NMR (500 MHz, $CDCl_3$) δ 6.99 (d, *J* = 1.3 Hz, 2H, H-7), 6.86 (s, 1H, H-6), 4.40 (s, 2H, H-8), 4.11 (q, *J* = 7.1 Hz, 4H, H-2), 3.03 (s, 4H, H-5), 2.38-2.44 (m, 4H, H-3a), 2.00-2.05 (m, 4H, H-3b), 1.84-1.89 (m, 4H, H-4a, H-4b), 1.21 (t, *J* = 7.1 Hz, 6H, H-1) ppm;

^{13}C NMR (126 MHz, $CDCl_3$) δ 176.7, 138.9, 137.5, 130.6, 128.3, 60.6, 48.7, 43.1, 33.9, 30.0, 15.7, 14.4 ppm;

IR (film, cm^{-1}): ν_{max} 2980 (m), 2941 (m), 2869 (w), 1721 (s), 1603 (w), 1445 (w), 1367 (w), 1324 (w), 1246 (w), 1200 (s), 1158 (m), 1096 (s), 1021 (w), 862 (w), 713 (w), 550 (w);

HRMS (+ESI) *m/z* found $[M+H]^+$ 451.1465, $[C_{23}H_{32}BrO_4]^+$ requires 451.1478, (δ = – 2.9 ppm).

Tetrabutylammonium (3,5-bis((1-(ethoxycarbonyl)cyclobutyl)methyl)phenyl)methanesulfonate

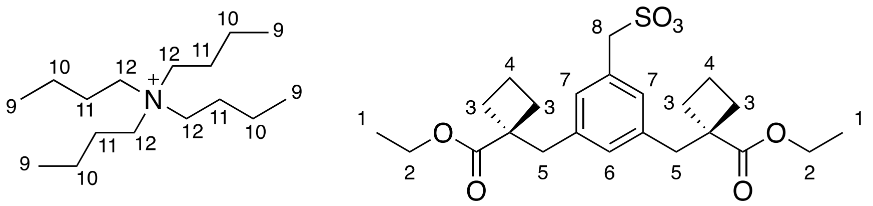

A variant of the protocol described by Phipps and co-workers was employed.[14] To a solution of diethyl 1,1'-((5-(bromomethyl)-1,3-phenylene)bis(methylene))bis(cyclobutane-1-carboxylate) (674 mg, 1.5 mmol) in a 2:3 mixture of acetone/water (15 mL) was added Na_2SO_3 (227 mg, 1.8 mmol). The resulting solution was heated to reflux for 3 hours and then allowed to cool to room temperature. The volatiles were removed under reduced pressure and the crude mixture was taken up in water (20 mL) following which freshly ground NaOH (48.0 mg, 1.2 mmol) and Bu_4NHSO_3 (407 mg, 1.2 mmol) were added and the aqueous layer extracted with CH_2Cl_2 (3 x 20 mL). The combined organic layers were dried ($MgSO_4$) and the solvent removed under reduced pressure to afford the title compound as a thick colourless oil (974 mg, 1.4 mmol, 94%).

^{1}H NMR (500 MHz, $CDCl_3$) δ 7.11 (d, *J* = 1.3 Hz, 2H, H-7), 6.76 (s, 1H, H-6), 4.12 (q, *J* = 7.1 Hz, 4H, H-2), 4.02 (s, 2H, H-8), 3.22-3.26 (m, 8H, H-12), 2.99 (s, 4H, H-5), 2.32-2.38 (m, 4H, H-3a), 2.01-2.06 (m, 4H, H-3b), 1.76-1.83 (m, 4H, H-4a, H-4b), 1.57-1.63 (m, 8H, H-11), 1.35-1.42 (m, 8H, H-10), 1.22 (t, *J* = 7.1 Hz, 6H, H-1), 0.98 (t, *J* = 7.3 Hz, 12H, H-9) ppm;

^{13}C NMR (126 MHz, $CDCl_3$) δ 177.1, 137.5, 134.0, 130.2, 128.9, 60.6, 58.9, 57.8, 48.8, 43.1, 29.8, 24.2, 19.8, 15.7, 14.4, 13.8 ppm;

IR (film, cm^{-1}): ν_{max} 2960 (m), 2874 (m), 1719 (s), 1602 (w), 1459 (m), 1325 (w), 1190 (s), 1095 (m), 981 (s), 798 (m), 760 (m), 685 (m), 609 (w);

HRMS (+ESI) *m/z* For cation found 242.2836, $[C_{16}H_{36}N]^+$ requires 242.2842, (δ = − 2.5 ppm).

HRMS (−ESI) *m/z* For anion found 451.1801, $[C_{23}H_{31}O_7S]^-$ requires 451.1796, (δ = + 1.1 ppm).

Tetrabutylammonium (3,5-bis((1-carboxycyclobutyl)methyl)phenyl)methanesulfonate (**B•Bu$_4$N**)

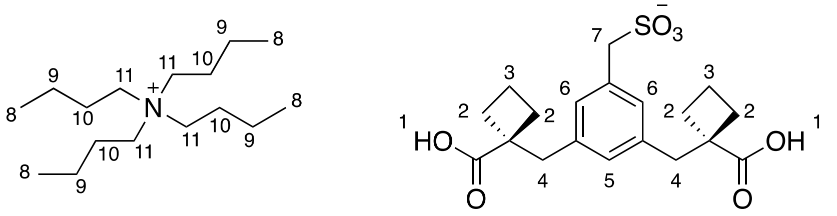

A round-bottomed flask fitted with a condenser was charged with tetrabutylammonium (3,5-bis((1-(ethoxycarbonyl)cyclobutyl)methyl)phenyl)methanesulfonate (974 mg, 1.4 mmol) and freshly ground NaOH (560 mg, 14.0 mmol). A 1:1 mixture of EtOH/H_2O (25 mL) was added and the reaction mixture stirred at reflux overnight. The volatiles were then removed under reduced pressure and the aqueous layer was acidified with 3 M HCl and extracted with a 3:1 mixture of $CHCl_3$/^{i}PrOH (3 x 20 mL). The combined organic layers were dried ($MgSO_4$) and the solvent removed under reduced pressure. Traces of ^{i}PrOH were removed through multiple azeotropes with $CHCl_3$ to afford the title compound as an off-white amorphous solid (713 mg, 1.1 mmol, 80%).

^{1}H NMR (500 MHz, CD_3CN/MeOH) δ 7.03 (d, *J* = 1.4 Hz, 2H, H-6), 6.91 (s, 1H, H-5), 3.82 (s, 2H, H-7), 3.06-3.09 (m, 8H, H-11), 3.03 (br s, 4H, H-4), 2.28-2.35 (m, 4H, H-2a), 1.98-2.05 (m, 4H, H-2b), 1.87-1.96 (m, 2H, H-3a), 1.77-1.85 (m, 2H, H-3b), 1.55-1.62 (m, 8H, H-10), 1.31-1.38 (m, 8H, H-9), 0.96 (t, *J* = 7.4 Hz, 12H, H-8) ppm (*Note: Drops of MeOH were added to the sample*

to improve solubility – MeOD was NOT used due to a persistent grease contaminant present in the commercial deuterated solvent at the time of synthesis);

^{13}C NMR (126 MHz, CD_3CN/MeOH) *δ* 179.0, 138.8, 135.1, 130.8, 130.1, 59.4 (t, J_{C-N} = 2.9 Hz), 58.3, 49.8, 44.0, 30.6, 24.4, 20.4 (t, J_{C-N} = 1.5 Hz), 16.0, 13.9 ppm (*Note: Drops of MeOH were added to the sample to improve solubility – MeOD was NOT used due to a persistent grease contaminant present in the commercial deuterated solvent at the time of synthesis*);

IR (neat, cm^{-1}): ν_{max} 2937 (br), 2872 (m), 1724 (s), 1602 (m), 1183 (s), 977 (s), 835 (m), 684 (m);

HRMS (+ESI) *m/z* For cation found 242.2842, $[C_{16}H_{36}N]^+$ requires 242.2842(*δ* = + 0.0 ppm).

HRMS (−ESI) *m/z* For anion found 395.1171, $[C_{19}H_{23}O_7S]^-$ requires 395.1170, (*δ* = + 0.3 ppm).

Bis[rhodium tetrabutylammonium (3,5-bis((1-carboxycyclobutyl)methyl)phenyl)methanesulfonate (Rh_2(**B**)$_2$•(**Bu$_4$N**)$_2$)

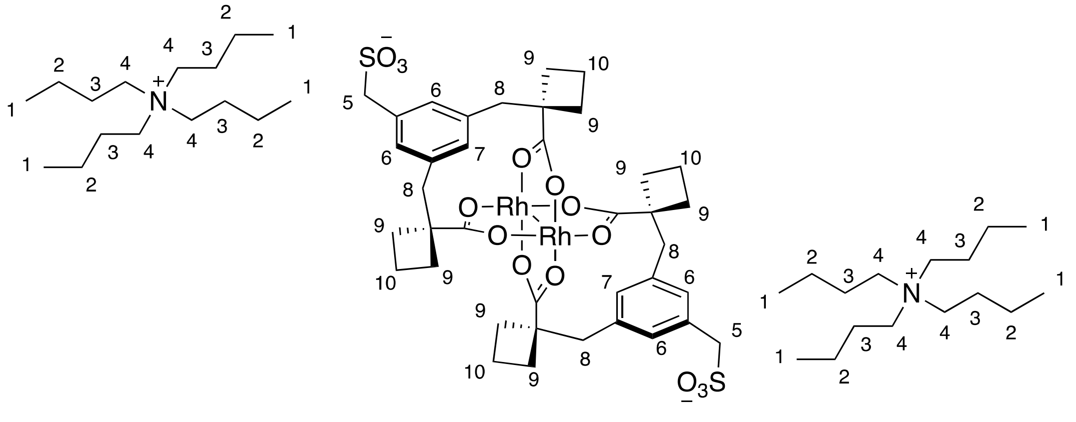

Prepared according to **GP1** on a 0.27 mmol scale with respect to $Rh_2(TFA)_4$ and using tetrabutylammonium (3,5-bis((1-carboxycyclobutyl)methyl)phenyl)methanesulfonate (**B•Bu$_4$N**) as the ligand. The title compound was obtained as a green amorphous solid (194 mg, 0.13 mmol, 49%).

^{1}H NMR (500 MHz, C_5D_5N) *δ* 7.45 (s, 4H, H-6), 7.36 (s, 2H, H-7), 4.28 (s, 4H, H-5), 3.44-3.48 (m, 16H, H-4), 3.03 (s, 8H, H-8), 2.37 (q, *J* = 10.2 Hz, 8H, H-9a), 1.82-1.89 (m, 8H, H-9b), 1.72-

1.81 (m, 20H, H-3, H-10a), 1.60-1.68 (m, 4H, H-10b), 1.35-1.42 (m, 16H, H-2), 0.92 (t, *J* = 7.4 Hz, 24H, H-1) ppm;

^{13}C NMR (126 MHz, C_5D_5N) δ 196.7, 138.3, 136.8, 130.8, 129.7, 59.7, 59.2, 52.1, 44.5, 31.2, 24.6, 20.5, 16.2, 14.2 ppm;

IR (neat, cm^{-1}) ν_{max} 2958 (m), 2936 (m), 2873 (m), 1576 (s), 1469 (w), 1441 (w), 1407 (s), 1263 (w), 1224 (s), 1118 (m), 1153 (s), 1030 (s), 879 (m), 760 (m), 719 (m), 606 (m);

HRMS (+ESI) *m/z* For cation found 242.2837, $[C_{16}H_{36}N]^+$ requires 242.2842, (δ = − 2.1 ppm).

HRMS (−ESI) *m/z* For dianion found 496.0063, $[C_{38}H_{42}O_{14}Rh_2S_2]^{2-}$ requires 496.0069, (δ = − 1.2 ppm).

Dimethyl 1,1'-((5-(bromomethyl)-1,3-phenylene)bis(methylene))bis(cyclopentane-1-carboxylate)

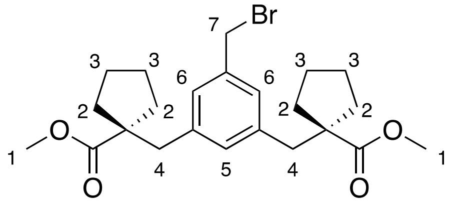

To a solution of ^{i}Pr$_2$NH (1.3 mL, 9.3 mmol) in THF (12 mL) at 0 °C was added *n*-BuLi (5.8 ml of a 1.6 M solution in hexanes, 9.3 mmol) *via* syringe pump over 15 minutes. The solution was stirred at 0 °C for 5 minutes after which methyl cyclopentanecarboxylate (1.19 g, 9.3 mmol) was added dropwise over 5 minutes. The above solution was stirred for 10 minutes and then added *via* syringe pump over 15 minutes to a solution of 1,3,5-tris(bromomethyl)benzene (1.66 g, 4.7 mmol) in THF (20 mL) at 0 °C. The reaction mixture was stirred at 0 °C for a further 20 minutes and then quenched by the addition of saturated aqueous NH_4Cl solution (30 mL). The THF was then removed under reduced pressure. The crude product was extracted with EtOAc (3 × 40 mL) and the combined organic layers were washed with brine (30 mL) dried ($MgSO_4$) and the solvent removed under reduced pressure.

Purification by flash column chromatography (SiO_2, 0-15% v/v Et_2O in hexane) afforded the title compound as white plates (909 mg, 2.0 mmol, 43%).

***R_f* value** = 0.19 (10% v/v Et_2O in hexane);

^{1}H NMR (400 MHz, $CDCl_3$) δ 6.96 (s, 2H, H-6), 6.79 (s, 1H, H-5), 4.40 (s, 2H, H-7), 3.64 (s, 6H, H-1), 2.89 (s, 4H, H-4), 2.04-2.07 (m, 4H, H-2a), 1.55-1.66 (m, 12H, H-2b, H-3a, H-3b) ppm;

^{13}C NMR (101 MHz, $CDCl_3$) δ 177.6, 139.2, 137.4, 131.3, 128.5, 55.7, 51.9, 43.9, 35.8, 33.9, 24.5 ppm;

IR (film, cm^{-1}): ν_{max} 2949 (s), 2870 (w), 2160 (w), 2012 (w), 1976 (w), 1722 (s), 1602 (m), 1450 (m), 1433 (m), 1340 (w), 1266 (w), 1192 (s), 1161 (s), 1107 (w), 1066 (w), 1020 (w), 982 (w), 861 (w), 714 (w);

HRMS (+ESI) *m/z* found $[M{-}Br]^+$ 371.2222, $[C_{23}H_{31}O_4]^+$ requires 371.2217, (δ = + 1.3 ppm).

Tetrabutylammonium (3,5-bis((1-(methoxycarbonyl)cyclopentyl)methyl)phenyl)methanesulfonate

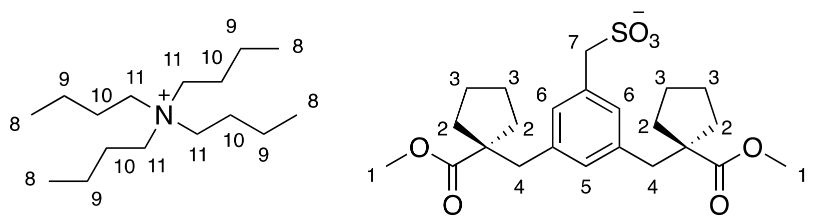

A variant of the protocol described by Phipps and co-workers was employed.[14] To a solution of dimethyl 1,1'-((5-(bromomethyl)-1,3-phenylene)bis(methylene))bis(cyclopentane-1-carboxylate) (897 mg, 2.0 mmol) in a 2:3 mixture of acetone/water (10 mL) was added Na_2SO_3 (301 mg, 2.4 mmol). The resulting solution was heated to reflux in a sealed crimp-top vial for 6 hours and then allowed to cool to room temperature. The volatiles were removed under a stream of compressed air and the crude mixture was taken up in water (10 mL) following which freshly ground NaOH (64.0 mg, 1.6 mmol) and Bu_4NHSO_3 (540 mg,

1.6 mmol) were added and the aqueous layer was extracted with a 3:1 mixture of $CHCl_3$/^{i}PrOH (3 x 20 mL). The combined organic layers were then washed once with water (15 mL), dried ($MgSO_4$) and the solvent removed under reduced pressure to afford the title compound as a thick oil (1.37 g, 2.0 mmol, quant.).

^{1}H NMR (400 MHz, $CDCl_3$) δ 7.04 (s, 2H, H-6), 6.68 (s, 1H, H-5), 3.99 (s, 2H, H-7), 3.64 (s, 6H, H-1), 3.19-3.24 (m, 8H, H-11), 2.85 (s, 4H, H-4), 1.97-2.06 (m, 4H, H-2a), 1.54-1.60 (m, 20H, H-2b, H-3a, H-3b, H-10), 1.32-1.41 (m, 8H, H-9), 0.96 (t, J = 7.3 Hz, 12H, H-8) ppm;

^{13}C NMR (101 MHz, $CDCl_3$) δ 177.9, 137.7, 134.3, 130.3, 129.3, 58.9, 57.9, 55.7, 51.8, 44.0, 35.6, 24.5, 24.2, 19.8, 13.8 ppm;

IR (neat, cm^{-1}): ν_{max} 2955 (s), 2874 (m), 2159 (w), 2030 (w), 1977 (w), 1723 (s), 1602 (w), 1455 (m), 1381 (w), 1341 (w), 1190 (s), 1065 (w), 1033 (s), 881 (m), 760 (w), 706 (w);

HRMS (+ESI) *m/z* For cation found 242.2854, $[C_{16}H_{36}N]^+$ requires 242.2842, (δ = + 5.0 ppm).

HRMS (−ESI) *m/z* For anion found 451.1801, $[C_{23}H_{31}O_7S]^-$ requires 451.1796, (δ = + 1.1 ppm).

Tetrabutylammonium (3,5-bis((1-carboxycyclopentyl)methyl)phenyl)methanesulfonate
(**C•Bu₄N**)

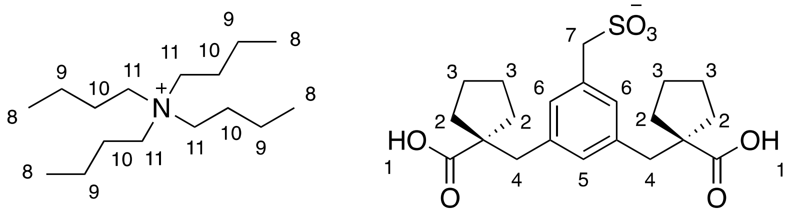

A round-bottomed flask fitted with a condenser was charged with tetrabutylammonium (3,5-bis((1-(methoxycarbonyl)cyclopentyl)methyl)phenyl)methanesulfonate (1.35 g, 1.9 mmol) and freshly ground NaOH (780 mg, 19.5 mmol). A 1:1 mixture of MeOH/H_2O (20 mL) was added and the reaction mixture stirred at 90 °C overnight. The MeOH was removed under reduced pressure and the aqueous layer was acidified with 3 M HCl and extracted

with a 3:1 mixture of $CHCl_3$/^{i}PrOH (4 x 20 mL). The combined organic layers were dried ($MgSO_4$) and the solvent removed under reduced pressure. Traces of ^{i}PrOH were removed through multiple azeotropes with $CHCl_3$ to afford the title compound as an off-white amorphous solid (934 mg, 1.4 mmol, 72%).

^{1}H NMR (400 MHz, MeOD) *δ* 7.10 (s, 2H, H-6), 6.96 (s, 1H, H-5), 3.96 (s, 2H, H-7), 3.21-3.25 (m, 8H, H-11), 2.92 (s, 4H, H-4), 2.01-2.04 (m, 4H, H-2a), 1.62-1.70 (m, 20H, H-2b, H-3a, H-3b, H-10), 1.41 (sext. *J* = 7.5 Hz, 8H, H-9), 1.02 (t, *J* = 7.3 Hz, 12H, H-8) ppm;

^{13}C NMR (101 MHz, MeOD) *δ* 181.1, 139.5, 133.9, 131.33, 131.31, 59.5 (*t*, $J_{C\text{-}N}$ = 2.9 Hz), 58.5, 56.6, 44.7, 36.5, 25.3, 24.8, 20.7 (*t*, $J_{C\text{-}N}$ = 1.5 Hz), 13.9 ppm;

IR (neat, cm^{-1}): ν_{max} 2957 (s), 2875 (m), 2537 (br), 2159 (m), 2013 (m), 1715 (s), 1437 (m), 1163 (s), 1036 (s), 887 (m), 707 (w);

HRMS (+ESI) *m/z* For cation found 242.2844, $[C_{16}H_{36}N]^{+}$ requires 242.2842, (*δ* = + 0.8 ppm).

HRMS (−ESI) *m/z* For anion found 423.1486, $[C_{21}H_{27}O_{7}S]^{-}$ requires 423.1483, (*δ* = + 0.7 ppm).

Bis[rhodium tetrabutylammonium (3,5-bis((1-carboxycyclopentyll)methyl)phenyl)methanesulfonate)] (Rh_2(**C**)$_2$•(**Bu$_4$N**)$_2$)

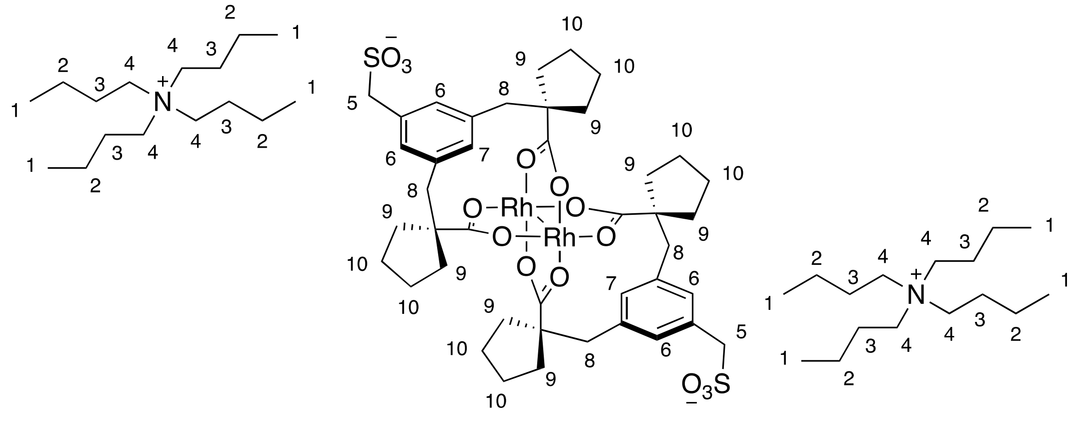

Prepared according to **GP1** on a 0.67 mmol scale with $Rh_2(TFA)_4$ and using tetrabutylammonium (3,5-bis((1-carboxycyclopentyl)methyl)phenyl)methanesulfonate

(**C•Bu4N**) as the ligand. The title compound was obtained as a green amorphous solid (646 mg, 0.42 mmol, 63%).

^{1}H NMR (500 MHz, DMSO-d_6) δ 6.77 (d, J = 1.2 Hz, 4H, H-6), 6.50 (s, 2H, H-7), 3.54 (s, 4H, H-5), 3.14-3.18 (m, 16H, H-4), 2.60 (s, 8H, H-8), 1.63-1.67 (m, 8H, H-9a), 1.49-1.59 (m, 24H, H-3, H-10a), 1.37-1.42 (m, 8H, H-9b), 1.27-1.34 (m, 24H, H-2, H-10b), 0.93 (t, J = 7.3 Hz, 24H, H-1) ppm;

^{13}C NMR (126 MHz, DMSO-d_6) δ 196.7, 137.0, 134.6, 128.8, 128.1, 57.8, 57.7, 57.5 (t, $J_{C\text{-}N}$ = 2.6 Hz), 44.3, 35.5, 24.1, 23.1, 19.2, 13.5 ppm;

IR (neat, cm^{-1}): ν_{max} 2957 (s), 2871 (m), 1575 (s), 1453 (w), 1400 (s), 1221 (m), 1190 (m), 1153 (s), 1032 (s), 764 (m), 721 (m), 708 (w), 609 (m);

HRMS (+ESI) *m/z* For cation found 242.2846, $[C_{16}H_{36}N]^+$ requires 242.2842, (δ = + 1.7 ppm).

HRMS (−ESI) *m/z* For dianion found 524.0383, $[C_{42}H_{50}O_{14}Rh_2S_2]^{2-}$ requires 524.0382, (δ = + 0.2 ppm).

Methyl cycloheptanecarboxylate

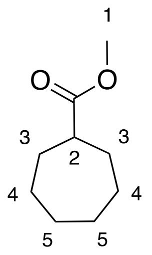

A microwave vial was charged with cycloheptane carboxylic acid (2.13 g, 15.0 mmol). Following this, methanol (15 mL) and c.H_2SO_4 (15 drops) were added. The vial was sealed and the resulting mixture was stirred at 60 °C for 2.5 hours. Following this, the solvent was removed under reduced pressure. The crude residue was taken up in EtOAc (40 mL) and washed with saturated aqueous $NaHCO_3$ (20 mL). The organic layer was then dried ($MgSO_4$)

and the solvent removed under reduced pressure to afford the title compound as a colourless oil (1.89 g, 12.1 mmol, 81%).

***R_f* value** = 0.44 (4% v/v Et_2O in hexane);

^{1}H NMR (400 MHz, $CDCl_3$) δ 3.65 (s, 3H, H-1), 2.48 (tt, J = 9.5 Hz, 4.4 Hz, 1H, H-2), 1.88-1.95 (m, 2H, H-3a), 1.42-1.75 (m, 10H, H-3b, H-4a, H-4b, H-5a, H-5b) ppm;

^{13}C NMR (101 MHz, $CDCl_3$) δ 177.7, 51.6, 45.1, 31.0, 28.4, 26.5 ppm;

IR (neat, cm^{-1}): ν_{max} 2925 (s), 2857 (m), 2159 (w), 2020 (w), 1732 (s), 1434 (m), 1376 (w), 1316 (w), 1251 (w), 1193 (m), 1156 (m), 1029 (w), 999 (w).

The NMR data is in agreement with that reported in the literature.[15]

Dimethyl 1,1'-((5-(bromomethyl)-1,3-phenylene)bis(methylene))bis(cycloheptane-1-carboxylate)

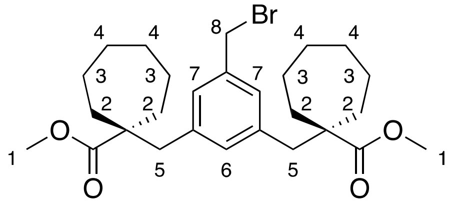

To a solution of $^iPr_2NH$ (1.3 mL, 9.3 mmol) in THF (12 mL) at 0 °C was added *n*-BuLi (5.8 ml of a 1.6 M solution in hexanes, 9.3 mmol) *via* syringe pump over 15 minutes. The solution was stirred at 0 °C for 5 minutes after which methyl cycloheptanecarboxylate (1.45 g, 9.3 mmol) was added dropwise over 5 minutes. The above solution was stirred for 10 minutes then added *via* syringe pump over 15 minutes to a solution of 1,3,5-tris(bromomethyl)benzene (1.66 g, 4.7 mmol) in THF (20 mL) at 0 °C. The reaction mixture was stirred at 0 °C for a further 20 minutes and then quenched by the addition of saturated aqueous NH_4Cl solution (30 mL). The THF was then removed under reduced pressure. The crude product was extracted with EtOAc (3 × 40 mL) and the combined organic layers were

washed with brine (30 mL) dried ($MgSO_4$) and the solvent removed under reduced pressure. Purification by flash column chromatography (SiO_2, 0-10% v/v Et_2O in hexane) afforded the title compound as a white amorphous solid (1.06 g, 2.1 mmol, 45%).

***R_f* value** = 0.25 (10% v/v Et_2O in hexane);

^{1}H NMR (400 MHz, $CDCl_3$) δ 6.91 (s, 2H, H-7), 6.69 (s, 1H, H-6), 4.39 (s, 2H, H-8), 3.66 (s, 6H, H-1), 2.77 (s, 4H, H-5), 2.00-2.04 (m, 4H, H-2a), 1.44-1.57 (m, 20H, H-2b, H-3a, H-3b, H-4a, H-4b) ppm;

^{13}C NMR (101 MHz, $CDCl_3$) δ 177.7, 138.3, 137.2, 132.2, 128.9, 51.7, 51.5, 46.7, 36.2, 33.9, 30.0, 23.5 ppm;

IR (neat, cm^{-1}): ν_{max} 2926 (s), 2863 (m), 2160 (w), 2007 (w), 1722 (s), 1601 (w), 1452 (s), 1431 (w), 1359 (w), 1334 (w), 1244 (w), 1209 (w), 1189 (s), 1170 (s), 1149 (s), 1061 (m), 985 (m), 890 (m), 870 (w), 839 (w), 806 (w), 774 (w), 758 (w), 711 (m);

HRMS (+ESI) *m/z* Found $[M-Br]^+$ 427.2844, $[C_{27}H_{39}O_4]^+$ requires 427.2843, (δ = + 0.2 ppm).

Tetrabutylammonium (3,5-bis((1-(methoxycarbonyl)cycloheptyl)methyl)phenyl)methanesulfonate

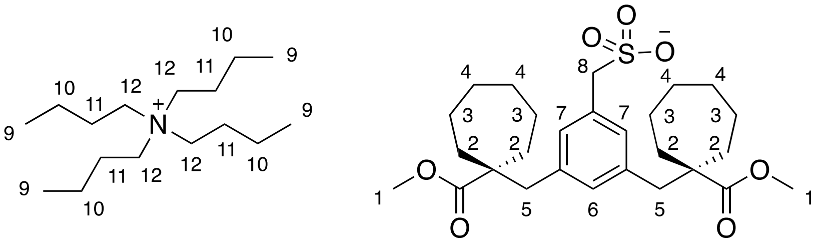

A variant of the protocol described by Phipps and co-workers was employed.[14] To a solution of dimethyl 1,1'-((5-(bromomethyl)-1,3-phenylene)bis(methylene))bis(cycloheptane-1-carboxylate) (1.05 g, 2.1 mmol) in a 2:3 mixture of acetone/water (10 mL) was added Na_2SO_3

(313 mg, 2.5 mmol). The resulting solution was heated to reflux for 6 hours and then allowed to cool to room temperature. The volatiles were removed under reduced pressure and the crude mixture was taken up in water (10 mL) following which freshly ground NaOH (68.0 mg, 1.7 mmol) and Bu_4NHSO_3 (577 mg, 1.7 mmol) were added and the aqueous layer was extracted with a 3:1 mixture of $CHCl_3$/^{i}PrOH (2 x 20 mL). The combined organic layers were then washed once with water (10 mL), dried ($MgSO_4$) and the solvent removed under reduced pressure to afford the title compound as a thick colourless oil (1.26 g, 1.7 mmol, 81%).

^{1}H NMR (500 MHz, $CDCl_3$) δ 6.99 (d, J = 1.4 Hz, 2H, H-7), 6.60 (s, 1H, H-6), 4.01 (s, 2H, H-8), 3.66 (s, 6H, H-1), 3.21-3.24 (m, 8H, H-12), 2.75 (s, 4H, H-5), 1.97-2.01 (m, 4H, H-2a), 1.56-1.62 (m, 8H, H-11), 1.42-1.54 (m, 20H, H-2b, H-3a, H-3b, H-4a, H-4b), 1.33-1.41 (m, 8H, H-10), 0.97 (t, J = 7.3 Hz, 12H, H-9) ppm;

^{13}C NMR (126 MHz, $CDCl_3$) δ 177.9, 136.9, 133.6, 130.7, 130.5, 58.9, 57.8, 51.7, 51.5, 46.9, 36.2, 30.0, 24.2, 23.6, 19.8, 13.8 ppm;

IR (film, cm^{-1}): ν_{max} 2927 (s), 2858 (w), 2159 (m), 2030 (m), 1976 (m), 1723 (s), 1602 (w), 1459 (m), 1221 (s), 1192 (s), 1034 (S), 886 (w), 730 (w);

HRMS (+ESI) *m/z* For cation found 242.2847, $[C_{16}H_{36}N]^+$ requires 242.2842, (δ = + 2.1 ppm).

HRMS (−ESI) *m/z* For anion found 507.2428, $[C_{27}H_{39}O_7S]^-$ requires 507.2422, (δ = + 1.2 ppm).

Tetrabutylammonium (3,5-bis((1-carboxycycloheptyl)methyl)phenyl)methanesulfonate
(**D•Bu₄N**)

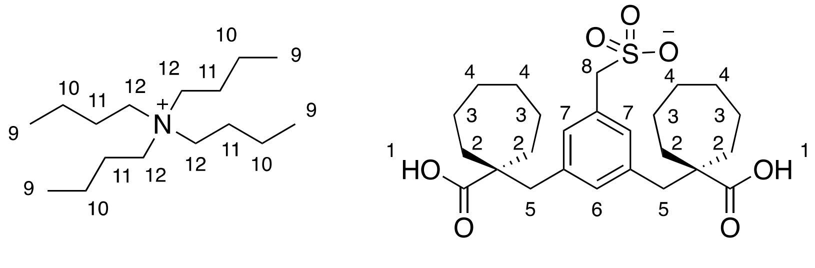

A round-bottomed flask fitted with a condenser was charged with tetrabutylammonium (3,5-bis((1-(methoxycarbonyl)cycloheptyl)methyl)phenyl)methanesulfonate (1.26 g, 1.7 mmol) and freshly ground NaOH (668 mg, 16.7 mmol). A 1:1 mixture of MeOH/H_2O (20 mL) was added and the reaction mixture stirred at 90 °C overnight. The MeOH was removed under reduced pressure and the aqueous layer was acidified with 3 M HCl and extracted with a 3:1 mixture of $CHCl_3$/^{i}PrOH (4 x 20 mL). The combined organic layers were dried ($MgSO_4$) and the solvent removed under reduced pressure. Traces of ^{i}PrOH were removed through multiple azeotropes with $CHCl_3$ to afford the title compound as an off-white amorphous solid (789 mg, 1.1 mmol, 64%).

^{1}H NMR (400 MHz, MeOD) δ 7.08 (s, 2H, H-7), 6.88 (s, 1H, H-6), 3.96 (s, 2H, H-8), 3.21-3.25 (m, 8H, H-12), 2.81 (s, 4H, H-5), 1.99-2.03 (m, 4H, H-2a), 1.62-1.70 (m, 8H, H-11), 1.46-1.59 (m, 20H, H-2b, H-3a, H-3b, H-4a, H-4b), 1.37-1.46 (m, 8H, H-10), 1.03 (t, *J* = 7.4 Hz, 12H, H-9) ppm;

^{13}C NMR (101 MHz, MeOD) δ 181.1, 138.6, 133.7, 132.3, 131.7, 59.5 (t, $J_{C\text{-}N}$ = 2.8 Hz), 58.5, 52.2, 47.5, 37.2, 31.1, 24.8, 24.6, 20.7 (t, $J_{C\text{-}N}$ = 1.5 Hz), 13.9 ppm;

IR (neat, cm^{-1}): ν_{max} 3474 (br), 2926 (s), 2858 (m), 2159 (w), 2028 (w), 1706 (s), 1602 (w), 1459 (m), 1380 (w), 1168 (s), 1150 (s), 1036 (s), 995 (m), 880 (m), 771 (w), 706 (w);

HRMS (+ESI) *m/z* For cation found 242.2849, $[C_{16}H_{36}N]^+$ requires 242.2842, (δ = + 2.9 ppm).

HRMS (−ESI) *m/z* For anion found 479.2118, $[C_{25}H_{35}O_7S]^-$ requires 479.2109, (δ = + 1.9 ppm

Bis[rhodium tetrabutylammonium (3,5-bis((1-carboxycycloheptyl)methyl)phenyl)methanesulfonate)] (Rh_2(**D**)$_2$•(**Bu_4N**)$_2$)

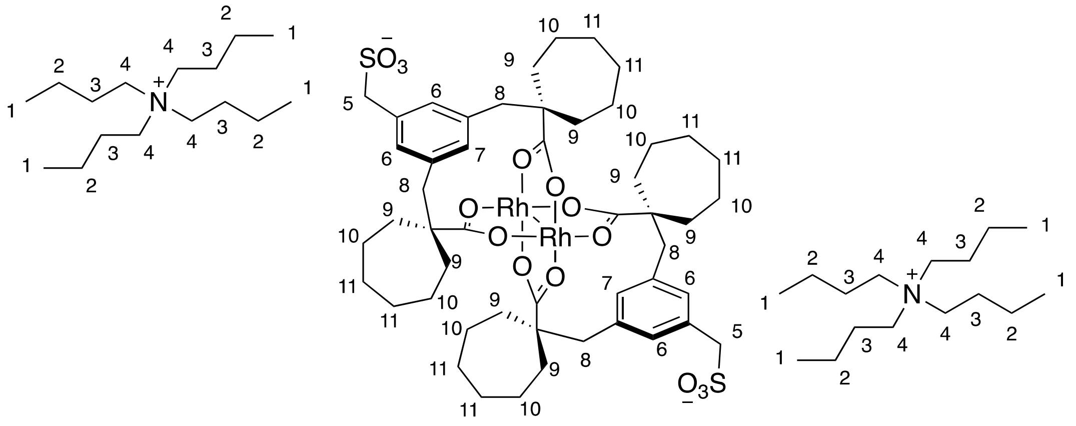

Prepared according to **GP1** on a 0.40 mmol scale with respect to $Rh_2(TFA)_4$ and using tetrabutylammonium (3,5-bis((1-carboxycycloheptyl)methyl)phenyl)methanesulfonate (**D•Bu$_4$N**) as the ligand. The title compound was obtained as a green amorphous solid (320 mg, 0.19 mmol, 49%).

^{1}H NMR (500 MHz, C_5D_5N) δ 7.38 (s, 4H, H-6), 7.30 (s, 2H, H-7), 4.37 (s, 4H, H-5), 3.42-3.45 (m, 16H, H-4), 2.79 (s, 8H, H-8), 2.05-2.07 (m, 8H, H-9a), 1.70-1.75 (m, 16H, H-3), 1.50-1.55 (m, 40H, H-9b, H-10a, H-10b, H-11a, H-11b), 1.31-1.41 (m, 16H, H-2), 0.90 (t, J = 7.3 Hz, 24H, H-1) ppm;

^{13}C NMR (126 MHz, C_5D_5N) δ 197.2, 137.9, 135.2*, 130.7, 130.5, 59.4, 58.8, 53.3, 47.1, 37.4, 31.4, 26.2, 24.2, 20.1, 13.9 ppm;

*obscured by the residual solvent peak but deduced from the HMBC.

IR (neat, cm^{-1}): ν_{max} 2958 (m), 2921 (s), 2854 (m), 1570 (s), 1456 (m), 1399 (s), 1235 (m), 1218 (s), 1191 (s), 1155 (s), 1033 (s), 881 (m), 761 (m), 738 (m), 720 (s), 706 (m), 605 (s);

HRMS (+ESI) *m/z* For cation found 242.2854, $[C_{16}H_{36}N]^+$ requires 242.2842, (δ = + 5.0 ppm).

HRMS (−ESI) *m/z* For dianion found 580.0988, $[C_{50}H_{66}O_{14}Rh_2S_2]^{2-}$ requires 580.1008), (δ = −3.4 ppm).

Methyl cyclooctanecarboxylate

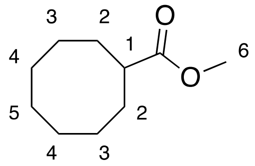

A variant of the protocol described by Shi and co-workers was employed.[16] To a stirred mixture of $Pd(OAc)_2$ (202 mg, 0.90 mmol) and Xantphos (521 mg, 0.90 mmol) in toluene (6.0 mL) were successively added *cis*-cyclooctene (3.9 mL, 30.0 mmol), formic acid (2.3 mL, 60.0 mmol), and acetic anhydride (0.57 mL, 6.0 mmol) *via* syringe. The flask was evacuated and backfilled with nitrogen ten times following which the reaction mixture was stirred at 70 °C overnight. The reaction mixture was then cooled to room temperature, diluted with CH_2Cl_2 (10 mL) and poured into 2.6 M NaOH (40 mL) in a separatory funnel. The contents were shaken and the layers separated, following which the aqueous layer was washed with CH_2Cl_2 (3 x 100 mL) and the organic washings discarded. The aqueous layer was subsequently acidified with 3 M HCl and extracted with CH_2Cl_2 (3 x 80 mL). The combined organics were dried ($MgSO_4$) and the solvent removed under reduced pressure. The crude residue was then dissolved in MeOH (60 mL) to which c.H_2SO_4 (1.5 mL) was added and the resulting mixture refluxed for 2 hours. The solvent was then removed under reduced pressure and the crude residue was taken up in Et_2O (100 mL) and washed with saturated aqueous $NaHCO_3$ (20 mL). The organic layer was then dried ($MgSO_4$) and the solvent removed under reduced pressure to afford the title compound as a colourless oil (3.04 g, 17.9 mmol, 60% over 2 steps).

***R$_f$* value** = 0.45 (5% v/v Et_2O in hexane);

^{1}H NMR (400 MHz, $CDCl_3$) δ 3.65 (s, 3H, H-6), 2.51 (tt, *J* = 9.2 Hz, 4.0 Hz, 1H, H-1), 1.84-1.91 (m, 2H, H-2a), 1.65-1.73 (m, 4H, H-2b, H-3a), 1.48-1.59 (m, 8H, H-3b, H-4a, H-4b, H-5a, H-5b) ppm;

^{13}C NMR (101 MHz, $CDCl_3$) δ 178.0, 51.6, 43.6, 28.9, 26.9, 26.3, 25.4 ppm;

IR (film, cm^{-1}): ν_{max} 2920 (s), 2854 (w), 1734 (s), 1470 (w), 1447 (w), 1434 (w), 1190 (m), 1166 (s), 1134 (w), 1051 (m).

The NMR data is in agreement with that reported in the literature.[17]

Dimethyl 1,1'-((5-(bromomethyl)-1,3-phenylene)bis(methylene))bis(cyclooctane-1-carboxylate)

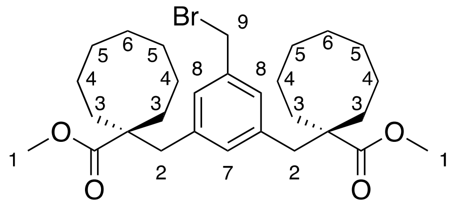

To a solution of ${}^{i}Pr_2NH$ (2.2 mL, 15.8 mmol) in THF (15 mL) at 0 °C was added *n*-BuLi (9.9 ml of a 1.6 M solution in hexanes, 15.8 mmol) *via* syringe pump over 10 minutes. The solution was stirred at 0 °C for 5 minutes after which methyl cyclooctanecarboxylate (2.69 g, 15.8 mmol) was added dropwise over 5 minutes. The above solution was stirred for 10 minutes and then added *via* syringe pump over 15 minutes to a solution of 1,3,5-tris(bromomethyl)benzene (2.82 g, 7.9 mmol) in THF (30 mL) at 0 °C. Transfer of the enolate was made quantitative with THF (4 mL). The reaction mixture was stirred at 0 °C for a further 20 minutes and then quenched by the addition of saturated aqueous NH_4Cl solution (30 mL). The THF was then removed under reduced pressure. The crude product was extracted with EtOAc (3 × 40 mL) and the combined organic layers were washed with brine (30 mL), dried ($MgSO_4$) and the solvent removed under reduced pressure. Purification by flash column chromatography (0-100% v/v CH_2Cl_2 in hexane followed by 100% EtOAc) afforded the title compound as a colourless oil (1.97 g, 3.7 mmol, 47%).

***R**$_f$* **value** = 0.23 (10% v/v Et_2O in hexane);

^{1}H NMR (400 MHz, $CDCl_3$) *δ* 6.87 (s, 2H, H-8), 6.64 (s, 1H, H-7), 4.38 (s, 2H, H-9), 3.64 (s, 6H, H-1), 2.75 (s, 4H, H-2), 1.91-1.96 (m, 4H, H-3a), 1.49-1.65 (m, 24H, H-3b, H-4a, H-4b, H-5a, H-5b, H-6a, H-6b) ppm;

^{13}C NMR (101 MHz, $CDCl_3$) δ 177.2, 138.3, 137.2, 132.0, 128.8, 51.8, 51.6, 44.6, 33.9, 30.7, 28.6, 25.3, 23.2 ppm;

IR (film, cm^{-1}): ν_{max} 2921 (s), 2853 (m), 1721 (s), 1476 (m), 1446 (m), 1264 (m), 1209 (m), 1193 (m), 1145 (w), 1113 (w), 1070 (w), 1023 (w), 734 (s), 715 (m), 703 (m);

HRMS (+ESI) *m/z* Found $[M+Na]^+$ 557.2246, $[C_{29}H_{43}BrNaO_4]^+$ requires 557.2237, (δ = + 1.6 ppm).

Dimethyl 1,1'-((5-(((2,2,2-trifluoroethoxy)sulfonyl)methyl)-1,3-phenylene)bis(methylene))bis(cyclooctane-1-carboxylate)

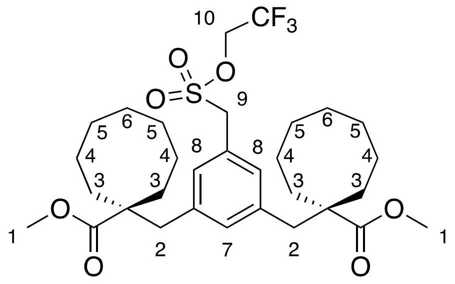

A variant of the protocol described by Maiti and co-workers was employed.[18] To a round-bottomed flask containing dimethyl 1,1'-((5-(bromomethyl)-1,3-phenylene)bis(methylene))bis(cyclooctane-1-carboxylate) (1.97 g, 3.7 mmol) and thiourea (281 mg, 3.7 mmol) was added EtOH (20 mL) and the reaction mixture heated to reflux for 3 hours. Following this, the reaction was allowed to cool and the solvent was removed under reduced pressure. The crude residue was suspended in MeCN (15 mL) and 2 N HCl (3.8 mL) was added. The mixture was then stirred at 0 °C for 15 minutes following which *N*-chlorosuccinimide (1.98 g, 15 mmol) was added to the suspension portion-wise in order to obtain a clear solution. The solution was then stirred for another 30 min at room temperature following which the MeCN was removed under reduced pressure. The remaining aqueous portion was extracted with EtOAc, dried ($MgSO_4$) and the solvent removed under reduced pressure to afford the sulfonyl chloride which was used directly in the next step with no further purification. A round-bottomed flask containing the crude sulfonyl chloride was evacuated and backfilled with argon and 2,2,2-trifluoroethanol

(0.27 mL, 3.7 mmol) was added. CH_2Cl_2 (20 mL) was then added and the reaction mixture cooled to 0 °C. Following this, Et_3N (0.71 mL, 5.1 mmol) was added dropwise under rapid stirring and the reaction mixture was allowed to warm to room temperature overnight. The reaction was then quenched with saturated aqueous NH_4Cl solution (10 mL) and the aqueous layer extracted with CH_2Cl_2 (4 × 20 mL). The combined organics were washed with brine (10 mL), dried ($MgSO_4$) and the solvent removed under reduced pressure. Purification by flash column chromatography (0-12% v/v acetone in hexane) afforded the product as white plates (715 mg, 1.2 mmol, 31%).

R

$\boldsymbol{R_f}$ **value** = 0.26 (12 % v/v acetone in hexane);

^{1}H NMR (500 MHz, $CDCl_3$) δ 6.90 (d, J = 1.1 Hz, 2H, H-8), 6.77 (br s, 1H, H-7), 4.35 (s, 2H, H-9), 4.11 (q, J = 8.0 Hz, 2H, H-10), 3.66 (s, 6H, H-1), 2.78 (s, 4H, H-2), 1.93 (dd, J = 15.2 Hz, 7.6 Hz 4H, H-3a), 1.50-1.54 (m, 24H, H-3b, H-4a, H-4b, H-5a, H-5b, H-6a, H-6b) ppm;

^{13}C NMR (126 MHz, $CDCl_3$) δ 177.0, 138.9, 133.4, 130.3, 126.3, 122.0 (q, $J_{C\text{-}F}$ = 277.8 Hz), 65.7 (q, $J_{C\text{-}F}$ = 38.1 Hz), 58.0, 51.8, 51.7, 44.3, 30.7, 28.5, 25.3, 23.2 ppm;

^{19}F NMR (376 MHz, $CDCl_3$) δ – 75.4 (s) ppm;

IR (film, cm^{-1}): ν_{max} 2922 (s), 2854 (w), 1722 (s), 1477 (w), 1447 (m), 1372 (m), 1283 (m), 1169 (s), 1070 (m), 1035 (s), 962 (m), 898 (w), 875 (w), 840 (m), 735 (m), 649 (w);

HRMS (+ESI) *m/z* Found $[M+Na]^+$ 641.2735, $[C_{31}H_{45}F_3NaO_7S]^+$ requires 641.2730 (δ = + 0.8 ppm).

Tetrabutylammonium (3,5-bis((1-carboxycyclooctyl)methyl)phenyl)methanesulfonate
($E{\bullet}Bu_4N$)

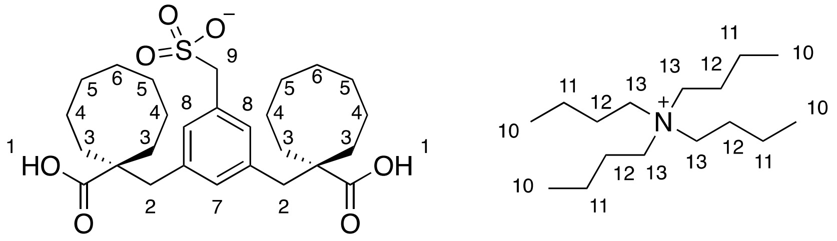

A round-bottomed flask fitted with a condenser was charged with dimethyl 1,1'-((5-(((2,2,2-trifluoroethoxy)sulfonyl)methyl)-1,3-phenylene)bis(methylene))bis(cyclooctane-1-carboxylate) (709 mg, 1.2 mmol) and freshly ground NaOH (460 mg, 12.0 mmol). A 1:1 mixture of MeOH/H_2O (40 mL) was added and the reaction mixture stirred at 90 °C overnight. ^{1}H NMR analysis showed no reaction so THF (20 mL) was added to aid dissolution of the starting material and the reaction mixture stirred at reflux for 24 hours following which a further portion of freshly ground NaOH (460 mg, 11.5 mmol) was added and the reaction stirred at the same temperature for a further 6 days. A final portion of NaOH (230 mg, 5.8 mmol) was then added and the reaction mixture stirred for one more night. The volatiles were then removed under reduced pressure and the aqueous layer was acidified with 3 M HCl. Then, a 3:1 mixture of $CHCl_3$/^{i}PrOH (30 mL) and Bu_4NHSO_3 (312 mg, 0.92 mmol) were added and the biphasic mixture shaken vigorously. The layers were separated and the aqueous layer was extracted with a 3:1 mixture of $CHCl_3$/^{i}PrOH (3 x 25 mL). The combined organic layers were dried ($MgSO_4$) and the solvent removed under reduced pressure. Traces of ^{i}PrOH were removed through multiple azeotropes with $CHCl_3$ to afford the title compound as an off-white amorphous solid (781 mg, 1.0 mmol, 90%). *In this case the ligand was obtained with a slight under-integration of the tetrabutylammonium cation.*

^{1}H NMR (500 MHz, DMSO-d$_6$) δ 6.85 (d, J = 0.8 Hz, 2H, H-8), 6.67 (s, 1H, H-7), 3.53 (s, 2H, H-9), 3.14-3.18 (m, 7H, H-13), 2.65 (s, 4H, H-2), 1.74-1.78 (m, 4H, H-3a), 1.42-1.59 (m, 32H, H-3b, H-4a, H-4b, H-5a, H-5b, H-6a, H-6b, H-12), 1.27-1.34 (m, 7H, H-11), 0.93 (t, J = 7.4 Hz, 10H, H-10) ppm;

^{13}C NMR (126 MHz, DMSO-d$_6$) δ 177.6, 136.3, 134.4, 129.9, 129.7, 57.7, 57.5 (t, J_{C-N} = 2.5 Hz), 50.3, 43.8, 30.1, 28.1, 24.8, 23.1, 22.6, 19.2, 13.5 ppm;

IR (film, cm^{-1}): ν_{max} 2923 (s), 2875 (s), 1719 (s), 1476 (m), 1151 (s), 1066 (w), 1033 (s), 875 (m), 836 (w), 807 (w), 731 (s), 702 (s), 571 (m), 520 (m);

HRMS (+ESI) *m/z* For cation found 242.2843, $[C_{16}H_{36}N]^+$ requires 242.2842, (δ = + 0.4 ppm).

HRMS (−ESI) *m/z* For anion found 507.2417, $[C_{27}H_{39}O_7S]^-$ requires 507.2422, (δ = − 1.0 ppm).

Bis[rhodium tetrabutylammonium (3,5-bis((1-carboxycyclooctyl)methyl)phenyl)methanesulfonate (Rh_2(**E**)$_2$•(**Bu$_4$N**)$_2$)

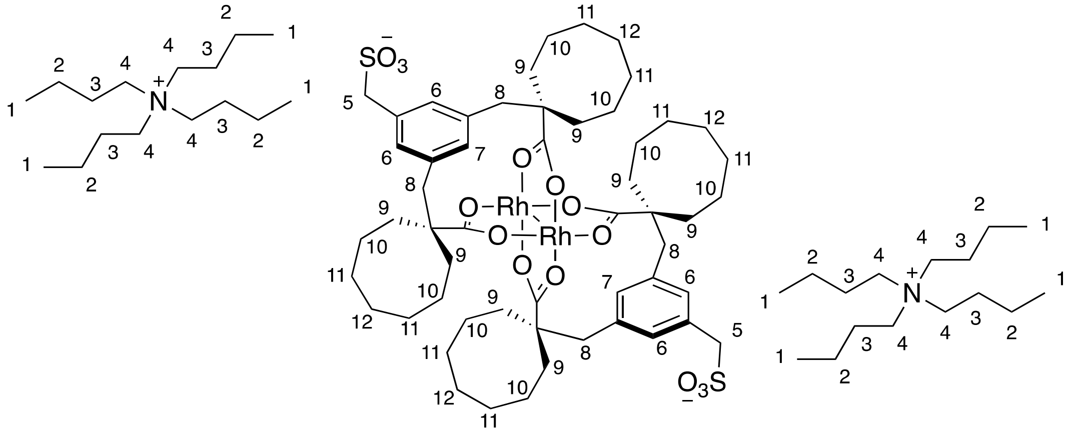

Prepared according to **GP1** on a 0.28 mmol scale with respect to $Rh_2(TFA)_4$ and using tetrabutylammonium (3,5-bis((1-carboxycyclooctyl)methyl)phenyl)methanesulfonate (**E•Bu$_4$N**) as the ligand. The title compound was obtained as a green amorphous solid (166 mg, 0.097 mmol, 35%).

^{1}H NMR (400 MHz, C_5D_5N) δ 7.34 (s, 4H, H-6), 7.22 (s, 2H, H-7)*, 4.30 (s, 4H, H-5), 3.42-3.46 (m, 16H, H-4), 2.76 (s, 8H, H-8), 2.09-2.15 (m, 8H, H-9a), 1.31-1.78 (m, 80H, H-2, H-3, H-9b, H-10a, H-10b, H-11a, H-11b, H-12a, H-12b), 0.90 (t, *J* = 7.3 Hz, 24H, H-1) ppm;

*obscured by one of the residual solvent peaks but deduced from the ^{1}H-COSY.

^{13}C NMR (126 MHz, C_5D_5N) δ 196.9, 138.0, 136.0†, 131.2, 130.9, 59.7, 59.1, 54.0, 45.4, 32.5, 29.4, 26.3, 24.6, 20.5, 14.3, 14.2 ppm;

†obscured by one of the residual solvent peaks but deduced from the HMBC spectrum.

IR (neat, cm^{-1}): ν_{max} 2919 (s), 2855 (s), 1579 (s), 1473 (w), 1461 (w), 1393 (s), 1353 (w), 1312 (w), 1300 (m), 1216 (w), 1191 (s), 1034 (w), 974 (s), 884 (m), 826 (m), 756 (m), 710 (m), 601 (s);

HRMS (+ESI) *m/z* For cation found 242.2848, $[C_{16}H_{36}N]^+$ requires 242.2842, (δ = + 2.5 ppm).

HRMS (−ESI) *m/z* For dianion found 608.1300, $[C_{54}H_{74}O_{14}Rh_2S_2]^{2-}$ requires 608.1321, (δ = − 3.5 ppm).

Synthesis of Chiral Cation Bromide Salts

(R)-((1S,2S,4S,5R)-5-Ethylquinuclidin-2-yl)(6-methoxyquinolin-4-yl)methanol (Dihydroquinine)

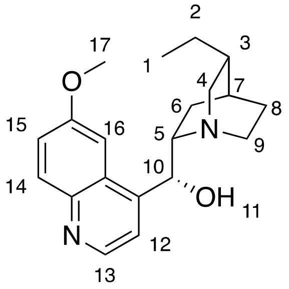

The protocol described by Phipps and co-workers was employed.[9] Quinine (10.0 g, 30.8 mmol) and ammonium formate (7.8 g, 124 mmol) were dissolved in MeOH (20 mL) and formic acid (3.5 mL, 92.8 mmol) was added. The mixture was heated to 55 °C and water (40 mL) was added until the reaction mixture became homogenous. The solution was cooled to room temperature and Pd/C (10 wt % loading Pd) (667 mg, 0.63 mmol) was added. The mixture was stirred at room temperature for 1 hour following which it was heated to 50 °C and stirred at the same temperature for a further 24 hours. The reaction mixture was then cooled to room temperature and formic acid (1.3 mL) was added with vigorous stirring. The mixture was then filtered through Celite® and the solvent partially removed. Aqueous NH_3 (35%, 50 mL) was added and the product extracted with CH_2Cl_2 (3 x 80 mL). The organic layers were washed with aqueous NH_3 (35%, 60 mL), water (2 x 60 mL), dried ($MgSO_4$) and the solvent removed under reduced pressure to afford the title compound as a pale-yellow powder (8.7 g, 26.6 mmol, 86%).

^{1}H NMR (400 MHz, $CDCl_3$) δ 8.68 (d, J = 4.5 Hz, 1H, H-13), 7.99 (d, J = 9.2 Hz, 1H, H-14), 7.51 (d, J = 4.5 Hz, 1H, H-12), 7.34 (dd, J = 9.2 Hz, 2.6 Hz, 1H, H-15), 7.25 (d, J = 2.7 Hz, 1H, H-16), 5.52 (d, J = 4.4 Hz, 1H, H-10), 3.91 (s, 3H, H-17), 3.35-3.43 (m, 1H, H-9a), 3.10-3.15 (m, 1H, H-5), 3.06 (dd, J = 13.5 Hz, 10.0 Hz, 1H, H-4a), 2.60-2.67 (m, 1H, H-9b), 2.38 (ddd, J = 13.4 Hz, 4.2 Hz, 2.6 Hz, 1H, H-4b), 1.67-1.77 (m, 3H, H-6a, H-7, H-8a), 1.36-1.54 (m, 3H, H-3, H-6b, H-8b), 1.21-1.29 (m, 2H, H-2a, H-2b), 0.81 (t, J = 7.3 Hz, 3H, H-1) ppm;

^{13}C NMR (101 MHz, $CDCl_3$) δ 157.9, 147.8, 147.7, 144.5, 131.8, 126.8, 121.6, 118.6, 101.5, 72.5, 59.9, 58.8, 55.8, 43.5, 37.7, 28.5, 27.9, 25.6, 21.9, 12.2 ppm;

IR (neat, cm^{-1}): ν_{max} 3175 (br), 2959 (s), 1620 (s), 1590 (w), 1508 (s), 1460 (m), 1432 (m), 1360 (w), 1325 (w), 1239 (s), 1226 (s), 1131 (w), 1080 (w), 1068 (w), 1027 (s), 913 (m), 858 (m), 831 (m);

$[\boldsymbol{\alpha}]_{\mathbf{D}}^{\mathbf{25.0}}$ = − 79.8 (c. 0.5, $CHCl_3$);

The spectroscopic data is in agreement with that reported in the literature.[9]

(S)-((1S,2R,4S,5R)-5-Ethylquinuclidin-2-yl)(6-methoxyquinolin-4-yl)methanol (Dihydroquinidine)

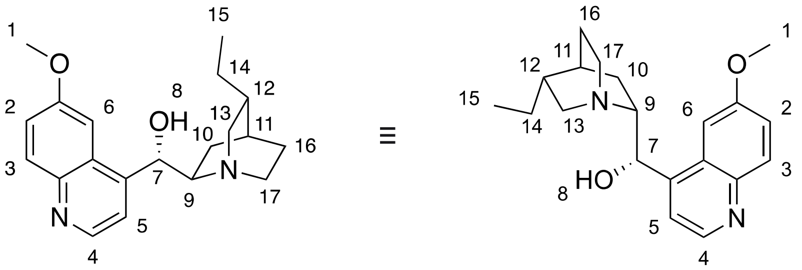

The protocol described by Phipps and co-workers was employed.[9] To a stirred solution of quinidine (3.24 g, 10.0 mmol), ammonium formate (2.52 g, 40.0 mmol) and MeOH (14 mL) in a two-neck flask fitted with a reflux condenser under air at room temperature was added dropwise formic acid (1.1 mL, 30.0 mmol) followed by water (7 mL). The reaction mixture was heated at 55 ^{o}C for 20 minutes and then allowed to cool to room temperature. Pd/C (10 wt % loading Pd, 222 mg, 0.21 mmol) was added and the reaction mixture was stirred at 50 °C for 16 hours. The reaction mixture was then allowed to cool to room temperature and formic acid (0.38 mL, 10.0 mmol) was added dropwise. The reaction mixture was filtered through Celite® and concentrated *in vacuo*. Aqueous NH_3 (35% NH_3, 20 mL) was added to the residue and the aqueous layer was extracted with CH_2Cl_2 (3 x 30 mL). The combined organic layers were dried ($MgSO_4$), filtered and concentrated *in vacuo* to afford the title compound as a white solid (3.22 g, 9.9 mmol, 99%).

^{1}H NMR (500 MHz, $CDCl_3$) δ 8.65 (d, J = 4.5 Hz, 1H, H-4), 7.96 (d, J = 9.2 Hz, 1H, H-3), 7.52 (d, J = 4.5 Hz, 1H, H-5), 7.30 (dd, J = 9.2 Hz, 2.7 Hz, 1H, H-2), 7.19 (d, J = 2.7 Hz, 1H, H-6), 5.59 (d, J = 4.1 Hz, 1H, H-7), 3.84 (s, 3H, H-1), 2.97-3.08 (m, 2H, H-9, H-13a), 2.82-2.92 (m, 2H, H-13b, H-17a), 2.70-2.77 (m, 1H, H-17b), 1.89–1.98 (m, 1H, H-10a), 1.69 (s, 1H, H-11), 1.44–1.54 (m, 2H, H-16a, H-16b), 1.36–1.44 (m, 3H, H-12, H-14a, H-14b), 1.10–1.16 (m, 1H, H-10b), 0.86 (t, J = 7.2 Hz, 3H, H-15) ppm;

^{13}C NMR (126 MHz, $CDCl_3$) δ = 157.8, 147.7*, 144.3, 131.7, 126.8, 121.6, 118.6, 101.4, 72.0, 60.0, 55.7, 51.3, 50.4, 37.5, 27.2, 26.4, 25.3, 21.2, 12.1 ppm;

*HMBC shows that this signal corresponds to two ^{13}C environments.

$[\alpha]_D^{25.0}$ = + 165.0 (c. 0.91, $CHCl_3$);

The spectroscopic data is in agreement with that reported in the literature.[19]

(1S,2S,4S,5R)-5-Ethyl-2-((R)-hydroxy(6-methoxyquinolin-4-yl)methyl)-1-((3,3'',5,5''-tetra-tert-butyl-[1,1':3',1''-terphenyl]-5'-yl)methyl)quinuclidin-1-ium bromide (**1•Br**)

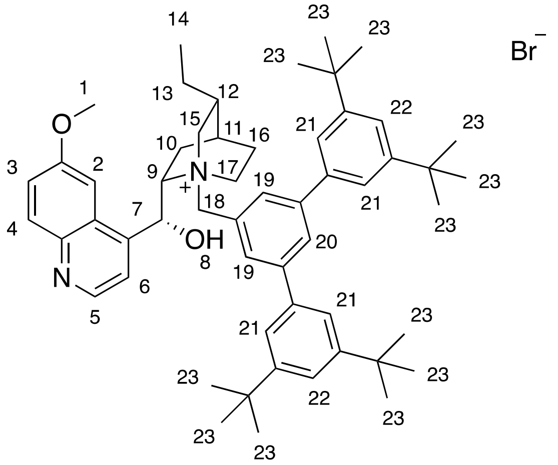

Prepared according to **GP2** on a 0.59 mmol scale with respect to dihyrdroquinine and using 5'-(bromomethyl)-3,3'',5,5''-tetra-*tert*-butyl-1,1':3',1''-terphenyl as the benzyl bromide.

Purification by flash column chromatography (SiO_2, 0-5% v/v MeOH in CH_2Cl_2) afforded the title compound as an off-white amorphous solid (352 mg, 0.40 mmol, 68%).

^{1}H NMR (500 MHz, $CDCl_3$) δ 8.78 (d, J = 4.5 Hz, 1H, H-5), 8.08 (d, J = 9.2 Hz, 1H, H-4), 7.98 (br s, 2H, H-19), 7.91 (t, J = 1.4 Hz, 1H, H-20), 7.80 (d, J = 4.5 Hz, 1H, H-6), 7.52 (t, J = 1.7 Hz, 2H, H-22), 7.49 (d, J = 1.7 Hz, 4H, H-21), 7.40 (dd, J = 9.3 Hz, 2.6 Hz, 1H, H-3), 7.25 (br s, 1H, H-2), 6.99 (d, J = 6.7 Hz, 1H, H-8), 6.87 (d, J = 7.0 Hz, 1H, H-7), 6.72 (d, J = 12.1 Hz, 1H, H-18a), 5.26 (t, J = 11.8 Hz, 1H, H-17a), 4.33 (d, J = 12.0 Hz, 1H, H-18b), 3.96 (s, 3H, H-1), 3.80 (t, J = 11.5 Hz, 1H, H-15a), 3.60-3.63 (m, 1H, H-9), 3.16-3.22 (m, 1H, H-17b), 2.66-2.70 (m, 1H, H-15b), 2.38-2.44 (m, 2H, H-10a, H-16a), 2.04 (br s, 1H, H-11), 1.80-1.86 (m, 1H, H-12), 1.67-1.71 (m, 1H, H-16b), 1.48-1.53 (m, 1H, H-10b), 1.40 (s, 36H, H-23), 1.30-1.36 (m, 2H, H-13a, H-13b), 0.84 (t, J = 7.4 Hz, 3H, H-14) ppm;

^{13}C NMR (126 MHz, $CDCl_3$) δ 158.3, 151.8, 148.0, 144.5, 144.4, 143.6, 139.5, 132.6, 131.4, 129.2, 127.5, 125.9, 122.6, 122.0, 120.9, 120.8, 101.9, 71.5, 64.8, 64.0, 63.3, 56.3, 51.2, 36.3, 35.2, 31.7, 26.6, 25.4, 24.5, 21.3, 11.4 ppm;

$[\boldsymbol{\alpha}]_{\mathbf{D}}^{\mathbf{25.0}}$ = − 103.6 (c. 1.0 $CHCl_3$);

The spectroscopic data is in agreement with that reported in the literature.[9]

Note: 5'-(bromomethyl)-3,3'',5,5''-tetra-tert-butyl-1,1':3',1''-terphenyl was prepared on a multigram scale using the procedure reported by Phipps and co-workers.[9]

(1S,2R,4S,5R)-5-Ethyl-2-((S)-hydroxy(6-methoxyquinolin-4-yl)methyl)-1-((3,3'',5,5''-tetra-tert-butyl-[1,1':3',1''-terphenyl]-5'-yl)methyl)quinuclidin-1-ium bromide (**2a•Br**)

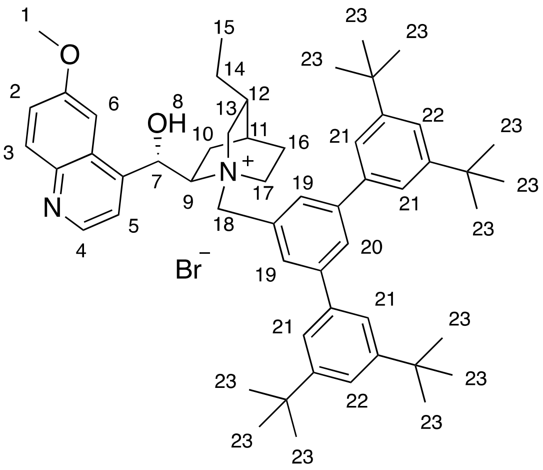

Prepared according to **GP2** on a 0.59 mmol scale with respect to dihydroquinidine and using 5'-(bromomethyl)-3,3'',5,5''-tetra-*tert*-butyl-1,1':3',1''-terphenyl as the benzyl bromide. Purification by flash column chromatography (SiO_2, 0-5% v/v MeOH in CH_2Cl_2) afforded the title compound as an off-white amorphous solid (337 mg, 0.39 mmol, 65%).

^{1}H NMR (500 MHz, $CDCl_3$) δ 8.70 (d, J = 4.6 Hz, 1H, H-4), 8.06 (d, J = 9.2 Hz, 1H, H-3), 7.93 (s, 2H, H-19), 7.86-7.87 (m, 2H, H-5, H-20), 7.52 (t, J = 1.7 Hz, 2H, H-22), 7.47 (d, J = 1.8 Hz, 4H, H-21), 7.34-7.36 (m, 2H, H-2, H-6), 7.09 (d, J = 5.8 Hz, 1H, H-8), 6.70 (d, J = 5.2 Hz, 1H, H-7), 6.29 (d, J = 12.3 Hz, 1H, H-18a), 4.88 (d, J = 12.2 Hz, 1H, H-18b), 4.47 (t, J = 9.3 Hz, 1H, H-13a), 3.95 (s, 3H, H-1), 3.84 (t, J = 9.2 Hz, 1H, H-9), 3.67-3.73 (m, 2H, H-13b, H-17a), 3.15 (q, J = 10.3 Hz, 1H, H-17b), 2.49 (t, J = 11.7 Hz, 1H, H-10a), 1.91 (br s, 1H, H-11), 1.75-1.84 (m, 2H, H-16a, H-16b), 1.55-1.71 (m, 3H, H-12, H-14a, H-14b), 1.40 (s, 36H, H-23), 1.00-1.06 (m, 1H, H-10b), 0.90 (t, J = 7.3 Hz, 3H, H-15) ppm;

^{13}C NMR (126 MHz, $CDCl_3$) δ 158.3, 151.8, 147.1, 144.4, 144.2, 143.5, 139.6, 131.6, 131.4, 129.2, 127.9, 126.2, 122.5, 122.1, 121.2, 120.9, 102.6, 69.3, 65.2, 64.2, 57.6, 56.3, 56.2, 36.4, 35.2, 31.7, 24.9, 24.7, 24.4, 21.5, 11.7 ppm;

$[\alpha]_D^{25.0}$ = + 100.9 (c. 0.37 $CHCl_3$);

The spectroscopic data is in agreement with that reported in the literature.[9]

Note: 5'-(bromomethyl)-3,3'',5,5''-tetra-tert-butyl-1,1':3',1''-terphenyl was prepared on a multigram scale using the procedure reported by Phipps and co-workers.[9]

(1S,2R,4S,5R)-5-Ethyl-2-((S)-hydroxy(6-methoxyquinolin-4-yl)methyl)-1-((3,3'',5,5''-tetrakis(trifluoromethyl)-[1,1':3',1''-terphenyl]-5'-yl)methyl)quinuclidin-1-ium bromide (**2b•Br**)

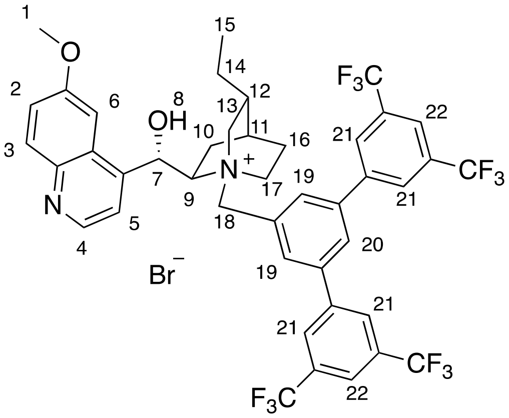

Prepared according to **GP2** on a 0.90 mmol scale with respect to dihydroquinidine and using 5'-(bromomethyl)-3,3'',5,5''-tetrakis(trifluoromethyl)-1,1':3',1''-terphenyl as the benzyl bromide. In this case the following procedural modification was used: upon cooling of the reaction mixture, first to room temperature and then to 0 °C, a precipitate formed. The precipitate was collected by filtration and washed on the filter with ice-cold THF and ice-cold Et_2O to afford the title compound as a white amorphous solid (661 mg, 0.72 mmol, 80%).

***R_f* value** = 0.50 (10% v/v MeOH in CH_2Cl_2);

^{1}H NMR (400 MHz, $CDCl_3$) δ 8.47 (d, *J* = 4.5 Hz, 1H, H-4), 8.27 (br s, 2H, H-19), 7.98 (br s, 4H, H-21), 7.86 (br s, 2H, H-22), 7.81 (d, *J* = 4.6 Hz, 1H, H-5), 7.78 (d, *J* = 9.2 Hz, 1H, H-3), 7.60 (br s, 1H, H-20), 7.44 (br s, 1H, H-6), 7.02 (dd, *J* = 9.3 Hz, 2.1 Hz, 1H, H-2), 6.83 (d, *J* = 5.7 Hz, 1H, H-8), 6.47 (br s, 1H, H-7), 6.11 (d, *J* = 12.3 Hz, 1H, H-18a), 6.01 (d, *J* = 12.1 Hz, 1H, H-18b), 4.40 (t, *J* = 9.8 Hz, 1H, H-13a), 4.20 (t, *J* = 10.7 Hz, 1H, H-17a), 4.10 (t, *J* = 9.2 Hz, 1H, H-9), 3.84 (s, 3H, H-1), 3.38 (t, *J* = 11.0 Hz, 1H, H-13b), 2.97 (q, *J* = 10.2 Hz, 1H, H-17b), 2.27 (t, *J* = 12.0 Hz,

1H, H-10a), 1.79-1.87 (m, 2H, H-11, H-16a), 1.70-1.75 (m, 1H, H-16b), 1.48-1.63 (m, 3H, H-12, H-14a, H-14b), 0.90-0.97 (m, 1H, H-10b), 0.85 (t, J = 7.2 Hz, 3H, H-15) ppm;

^{13}C NMR (101 MHz, $CDCl_3$) δ 158.2, 146.1, 143.8, 142.8, 140.9, 140.0, 132.2, 132.7 (q, $J_{C\text{-}F}$ = 33.5 Hz), 130.8, 130.5, 127.33, 127.30, 127.26, 126.2, 123.2 (q, $J_{C\text{-}F}$ = 273.0 Hz), 122.2-122.3 (m), 120.7, 103.1, 68.4, 66.7, 61.6, 57.2, 56.6, 56.5, 36.3, 24.7, 24.6, 24.2, 21.7, 11.5 ppm;

^{19}F NMR (376 MHz, $CDCl_3$) δ – 62.7 (s) ppm;

IR (neat, cm^{-1}): ν_{max} 3132 (br, w), 1623 (w), 1512 (w), 1462 (w), 1367 (s), 1277 (s), 1171 (s), 1119 (s), 1109 (s), 1081 (w), 1039 (w), 999 (w), 904 (s), 860 (w), 844 (w), 729 (s), 683 (w), 640 (w);

$[\alpha]_D^{25.0}$ = + 91.3 (c. 0.39, $CHCl_3$);

HRMS (+ESI) *m/z* For cation found 841.2648, $[C_{43}H_{37}F_{12}N_2O_2]^+$ requires 841.2658, (δ = – 1.2 ppm).

*Note: Both 5'-(bromomethyl)-3,3'',5,5''-tetrakis(trifluoromethyl)-1,1':3',1''-terphenyl and the salt (**2b•Br**) were synthesised by Dr Georgi R. Genov for which we are very grateful. The synthetic procedures and full characterisation for 5'-(bromomethyl)-3,3'',5,5''-tetrakis(trifluoromethyl)-1,1':3',1''-terphenyl have been previously reported.*[9]

[1,1':3',1''-Terphenyl]-5'-ylmethanol

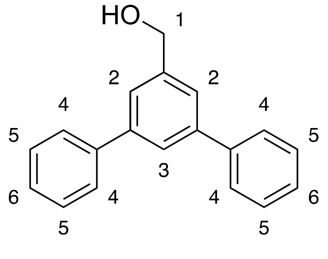

To a round-bottomed flask charged with 3,5-dibromobenzaldehyde (2.64 g, 10.0 mmol, 1.0 equiv.), phenyl boronic acid (2.80 g, 23.0 mmol, 2.3 equiv.), and

[1,1′-bis(diphenylphosphino)ferrocene] dichloropalladium(II) (1:1 complex with CH_2Cl_2; 245 mg; 0.30 mmol; 3 mol %) under an argon atmosphere was added degassed 1,4-dioxane (140 mL). 2 M Na_2CO_3 (11.0 mL, 22.0 mmol, 2.2 equiv.) was subsequently added, and the resulting mixture stirred under reflux for 16 h. Upon completion the solvent was removed *in vacuo,* and the resulting residue taken up in MeOH, the solution cooled to 0 °C, $NaBH_4$ (0.76 g, 20.0 mmol, 2.0 equiv.) added, and the reaction mixture stirred at room temperature for 1 h. The reaction was subsequently quenched *via* addition of 3 M HCl, the solution concentrated *in vacuo*, and CH_2Cl_2 (140 mL) added. The mixture was then washed with 3 M HCl, dried ($MgSO_4$), filtered, and concentrated *in vacuo*. Purification by flash column chromatography (SiO_2, 20% v/v EtOAc in hexane) afforded the title compound as an amorphous white solid (2.18 g, 8.4 mmol, 84%).

***R_f* value** = 0.25 (10% v/v EtOAc in petrol);

^{1}H NMR (400 MHz, $CDCl_3$) *δ* 7.75 (t, *J* = 1.8 Hz, 1H, H-3), 7.69 – 7.64 (m, 4H, H-4), 7.59 (d, *J* = 1.7 Hz, 2H, H-2), 7.50 – 7.45 (m, 4H, H-5), 7.42 – 7.36 (m, 2H, H-6), 4.83 (s, 2H, H-1), 1.92 (br s, 1H, OH) ppm;

^{13}C NMR (101 MHz, $CDCl_3$) *δ* 142.2, 141.9, 141.0, 128.8, 127.5, 127.3, 125.5, 124.7, 65.4 ppm;

IR (film, cm^{-1}): ν_{max} 3371 (br, m), 3059 (w), 3035 (w), 2927 (w), 1596 (m), 1576 (w), 1497 (w), 1456 (w), 1434 (m), 1409 (w), 1034 (m), 1020 (w), 866 (w), 759 (s), 697 (s);

HRMS (+ESI) *m/z* found $[M\text{-}OH_2]^+$ 243.1168, $[C_{19}H_{15}]^+$ requires 243.1168, (*δ* = + 0.0 ppm).

5'-(Bromomethyl)-1,1':3',1''-terphenyl

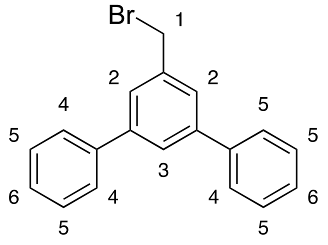

To an RBF charged with [1,1':3',1''-terphenyl]-5'-ylmethanol (2.00 g, 7.7 mmol, 1.0 equiv.) and CBr_4 (3.81 g, 11.5 mmol, 1.5 equiv.) was added CH_2Cl_2 (30 mL) and the resulting solution

cooled to 0 °C. To this was added dropwise a solution of PPh_3 (3.02 g, 11.5 mmol, 1.5 equiv.) in CH_2Cl_2 (15 mL), and the reaction mixture allowed to warm to room temperature and stirred overnight. The solvent was removed *in vacuo*, and purification by flash column chromatography (SiO_2, 0-10% v/v EtOAc in hexane) afforded the title compound as a white amorphous solid (1.65 g, 5.1 mmol, 66%).

^{1}H NMR (400 MHz, $CDCl_3$) δ 7.76 (t, J = 1.7 Hz, 1H, H-3), 7.69-7.64 (m, 4H, H-4), 7.62 (d, J = 1.7 Hz, 2H, H-2), 7.53-7.46 (m, 4H, H-5), 7.44-7.38 (m, 2H, H-6), 4.63 (s, 2H, H-1) ppm;

^{13}C NMR (101 MHz, $CDCl_3$) δ 142.5, 140.5, 138.8, 128.9, 127.7, 127.3, 126.8, 126.3, 33.5 ppm.

The spectroscopic data is in agreement with that reported in the literature.[20]

(1S,2R,4S,5R)-1-([1,1':3',1''-Terphenyl]-5'-ylmethyl)-5-ethyl-2-((S)-hydroxy(6-methoxyquinolin-4-yl)methyl)quinuclidin-1-ium bromide (**2c•Br**)

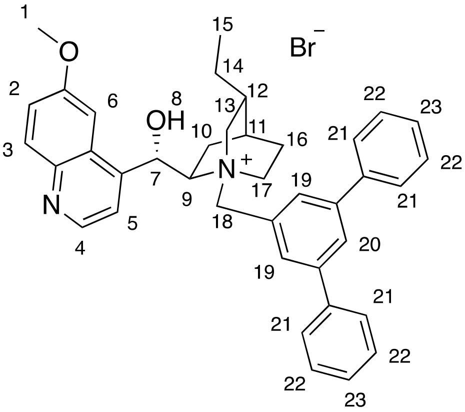

Prepared according to **GP2** on a 0.60 mmol scale with respect to dihydroquinidine and using 5'-(bromomethyl)-1,1':3',1''-terphenyl as the benzyl bromide. Purification by flash column chromatography (SiO_2, 0-14% v/v MeOH in CH_2Cl_2) afforded the title compound as an off-white amorphous solid (246 mg, 0.38 mmol, 63%).

***R_f* value** = 0.43 (10% v/v MeOH in CH_2Cl_2);

^{1}H NMR (500 MHz, $CDCl_3$) δ 8.47 (d, J = 4.2 Hz, 1H, H-4), 7.91 (s, 2H, H-19), 7.84 (d, J = 9.2 Hz, 1H, H-3), 7.78 (d, J = 4.5 Hz, 1H, H-5), 7.66 (s, 1H, H-6), 7.56 (s, 1H, H-20), 7.45-7.47 (m, 4H, H-21), 7.28-7.33 (m, 6H, H-22, H-23), 7.08 (dd, J = 9.2 Hz, 2.5 Hz, 1H, H-2), 7.05 (d, J = 5.6 Hz, 1H, H-8), 6.54-6.55 (m, 1H, H-7), 6.01 (d, J = 12.1 Hz, 1H, H-18a), 5.83 (d, J = 12.2 Hz, 1H, H-18b), 4.32 (t, J = 10.9 Hz, 1H, H-13a), 4.09 (q, J = 11.0 Hz, 2H, H-9, H-17a), 3.75 (s, 3H, H-1), 3.48 (t, J = 11.2 Hz, 1H, H-13b), 2.96 (q, J = 10.3 Hz, 1H, H-17b), 2.25 (t, J = 12.1 Hz, 1H, H-10a), 1.75-1.81 (m, 2H, H-11, H-16a), 1.67 (t, J = 10.5 Hz, 1H, H-16b), 1.46-1.56 (m, 3H, H-12, H-14a, H-14b), 0.89-0.94 (m, 1H, H-10b), 0.80 (t, J = 7.3 Hz, 3H, H-15) ppm;

^{13}C NMR (126 MHz, $CDCl_3$) δ 158.0, 147.4, 144.3, 142.7, 142.0, 139.3, 131.9, 131.1, 129.0, 128.6, 128.0, 127.3, 127.2, 126.5, 120.7, 120.2, 103.2, 68.1, 67.4, 62.2, 56.9, 56.4, 56.3, 36.3, 24.7, 24.6, 24.1, 21.9, 11.6 ppm;

IR (neat, cm^{-1}): ν_{max} 3182 (br), 2958 (w), 2361 (w), 2342 (w), 1620 (m), 1595 (m), 1507 (m), 1458 (m), 1433 (w), 1353 (w), 1317 (w), 1255 (w), 1239 (m), 1226 (m), 1177 (m), 1130 (w), 1078 (w), 1027 (m), 994 (m), 909 (m), 886 (m), 864 (m), 826 (s), 759 (s), 720 (s), 696 (m);

$[\boldsymbol{\alpha}]_{\mathbf{D}}^{\mathbf{25.0}}$ = + 125.5 (c. 1.1, $CHCl_3$);

HRMS (+ESI) *m/z* For cation found 569.3165, $[C_{39}H_{41}N_2O_2]^+$ requires 569.3163, (δ = + 0.4 ppm).

(1S,2R,4S,5R)-1-(3,5-Di-tert-butylbenzyl)-5-ethyl-2-((S)-hydroxy(6-methoxyquinolin-4-yl)methyl)quinuclidin-1-ium bromide (**2d•Br**)

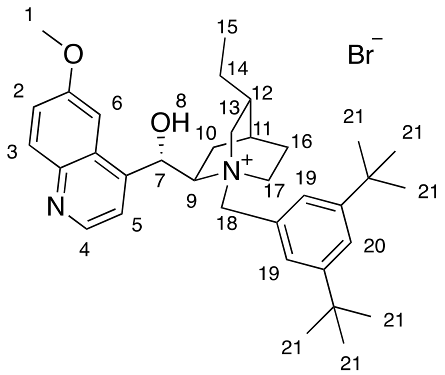

Prepared according to **GP2** on a 0.60 mmol scale with respect to dihydroquinidine and using 1-(bromomethyl)-3,5-di-*tert*-butylbenzene as the benzyl bromide. Purification by flash column chromatography (SiO_2, 0-14% v/v MeOH in CH_2Cl_2) afforded the title compound as a yellow amorphous solid (140 mg, 0.23 mmol, 38%).

***R$_f$* value** = 0.55 (10% v/v MeOH in CH_2Cl_2);

^{1}H NMR (400 MHz, $CDCl_3$) δ 8.69 (d, *J* = 4.3 Hz, 1H, H-4), 8.03 (d, *J* = 9.2 Hz, 1H, H-3), 7.75 (d, *J* = 4.4 Hz, 1H, H-5), 7.57 (s, 2H, H-19), 7.53 (s, 1H, H-20), 7.35 (dd, *J* = 9.2 Hz, 2.3 Hz, 1H, H-2), 7.26 (s, 1H, H-6), 6.97 (d, *J* = 6.3 Hz, 1H, H-8), 6.64 (d, *J* = 5.8 Hz, 1H, H-7), 6.06 (d, *J* = 12.0 Hz, 1H, H-18a), 4.69 (d, *J* = 12.4 Hz, 1H, H-18b), 4.40 (t, *J* = 9.7 Hz, 1H, H-13a), 3.94 (s, 3H, H-1), 3.69 (t, *J* = 9.2 Hz, 1H, H-9), 3.61 (br s, 1H, H-17a), 3.52 (t, *J* = 11.5 Hz, 1H, H-13b), 3.10 (q, *J* = 10.4 Hz, 1H, H-17b), 2.51 (t, *J* = 11.5 Hz, 1H, H-10a), 1.90 (br s, 1H, H-11), 1.79 (t, *J* = 7.0 Hz, 2H, H-16a, H-16b), 1.62-1.70 (m, 1H, H-12), 1.53-1.61 (m, 2H, H-14a, H-14b), 1.34 (s, 18H, H-21), 1.02-1.09 (m, 1H, H-10b), 0.86 (t, *J* = 7.3 Hz, 3H, H-15) ppm;

^{13}C NMR (101 MHz, $CDCl_3$) δ 158.1, 152.3, 147.9, 144.4, 143.3, 132.3, 128.4, 126.3, 126.1, 124.6, 120.8, 120.7, 102.3, 69.1, 65.0, 64.9, 57.3, 56.2, 56.1, 36.3, 35.2, 31.6, 25.1, 24.7, 24.4, 21.4, 11.5 ppm;

IR (film, cm^{-1}): ν_{max} 3175 (br), 2959 (s), 2872 (s), 2361 (s), 2342 (s), 1734 (w), 1718 (w), 1621 (m), 1595 (w), 1508 (m), 1473 (m), 1432 (m), 1395 (w), 1363 (w), 1240 (s), 1226 (m), 1204 (m), 1116 (w), 1078 (m), 1048 (m), 1028 (m), 995 (m), 908 (w), 865 (w), 827 (w);

$[\boldsymbol{\alpha}]_{\mathbf{D}}^{\mathbf{25.0}}$ = + 88.4 (c. 1.0, $CHCl_3$);

HRMS (+ESI) *m/z* For cation found 529.3793, $[C_{35}H_{49}N_2O_2]^+$ requires 529.3789, (δ = + 0.8 ppm).

(1S,2R,4S,5R)-1-(3,5-Bis(trifluoromethyl)benzyl)-5-ethyl-2-((S)-hydroxy(6-methoxyquinolin-4-yl)methyl)quinuclidin-1-ium bromide (**2e•Br**)

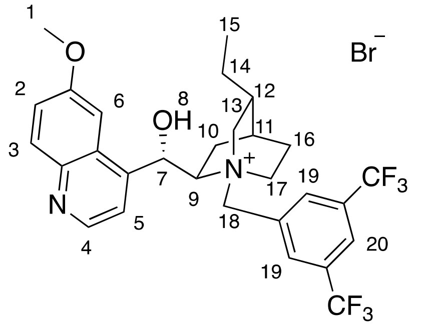

Prepared according to **GP2** on a 0.60 mmol scale with respect to dihydroquinidine and using 1-(bromomethyl)-3,5-bis(trifluoromethyl)benzene as the benzyl bromide. Purification by flash column chromatography (SiO_2, 0-12% v/v MeOH in CH_2Cl_2) afforded the title compound as an off-white amorphous solid (264 mg, 0.41 mmol, 69%).

***R**$_f$* **value** = 0.40 (10% v/v MeOH in CH_2Cl_2);

^{1}H NMR (400 MHz, $CDCl_3$) δ 8.50 (d, J = 4.5 Hz, 1H, H-4), 8.28 (s, 2H, H-19), 7.83 (s, 1H, H-20), 7.76 (d, J = 9.3 Hz, 1H, H-3), 7.74 (d, J = 4.6 Hz, 1H, H-5), 7.53 (d, J = 2.3 Hz, 1H, H-6), 7.02 (dd, J = 9.3 Hz, 2.5 Hz, 1H, H-2), 6.71 (d, J = 5.8 Hz, 1H, H-8), 6.41 (br s, 1H, H-7), 6.19 (d, J = 12.4 Hz, 1H, H-18a), 5.88 (d, J = 12.4 Hz, 1H, H-18b), 4.32 (t, J = 10.4 Hz, 1H, H-13a), 4.24 (t, J = 11.0 Hz, 1H, H-17a), 4.15 (t, J = 9.0 Hz, 1H, H-9), 3.78 (s, 3H, H-1), 3.13 (t, J = 11.0 Hz, 1H, H-13b), 2.68 (q, J = 10.1 Hz, 1H, H-17b), 2.20 (t, J = 12.8 Hz, 1H, H-10a), 1.44-1.82 (m, 6H, H-11, H-12, H-14a, H-14b, H-16a, H-16b), 0.87-0.93 (m, 1H, H-10b), 0.84 (t, J = 7.2 Hz, 3H, H-15) ppm;

^{13}C NMR (101 MHz, $CDCl_3$) δ 158.0, 147.2, 144.1, 142.3, 133.9-134.1 (m), 132.5 (q, J_{C-F} = 34.0 Hz), 131.8, 130.6, 126.1, 124.2-124.3 (m), 122.7 (q, J_{C-F} = 273.1 Hz), 120.5, 119.9, 103.2, 68.3, 67.1, 60.3, 56.7, 56.6, 56.5, 36.2, 24.6, 24.5, 24.2, 21.9, 11.5 ppm;

^{19}F NMR (376 MHz, $CDCl_3$) δ – 63.7 (s) ppm;

IR (neat, cm^{-1}): ν_{max} 3185 (w), 2964 (w), 2361 (w), 2342 (w), 1621 (m), 1508 (m), 1461 (m), 1432 (m), 1372 (m), 1277 (s), 1227 (w), 1172 (s), 1128 (s), 1027 (w), 903 (s), 864 (w), 827 (m), 785 (w), 711 (m), 682 (m);

$[\alpha]_D^{25.0}$ = + 82.3 (c. 1.1, $CHCl_3$);

HRMS (+ESI) *m/z* For cation found 553.2289, $[C_{29}H_{31}F_6N_2O_2]^+$ requires 553.2284, (δ = + 0.9 ppm).

(1S,2S,4S,5R)-5-Ethyl-2-((S)-hydroxy(6-methoxyquinolin-4-yl)methyl)-1-((3,3'',5,5''-tetra-tert-butyl-[1,1':3',1''-terphenyl]-5'-yl)methyl)quinuclidin-1-ium bromide (**3•Br**)

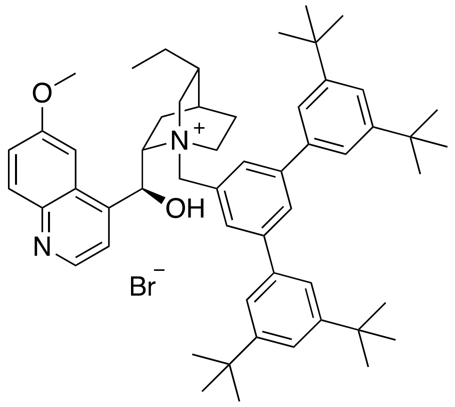

The compound (**3•Br**) was obtained as a gift from Dr Georgi R. Genov for which we are very grateful. The synthetic route and full characterisation data for this compound have been reported in the literature.[9]

(1S,2R,4S,5R)-5-Ethyl-2-((R)-hydroxy(6-methoxyquinolin-4-yl)methyl)-1-((3,3'',5,5''-tetra-tert-butyl-[1,1':3',1''-terphenyl]-5'-yl)methyl)quinuclidin-1-ium bromide (**4•Br**)

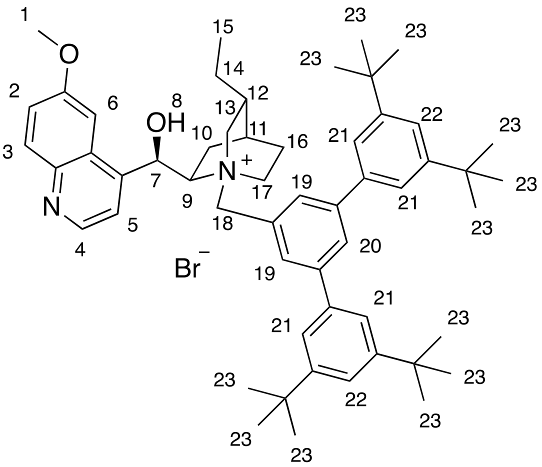

Prepared according to **GP2** on a 1.0 mmol scale with respect to *epi*-dihydroquinidine and using 5'-(bromomethyl)-3,3'',5,5''-tetra-*tert*-butyl-1,1':3',1''-terphenyl as the benzyl bromide. Purification by flash column chromatography (SiO_2, 2-5% v/v MeOH in CH_2Cl_2) afforded the title compound as a light-brown amorphous solid (389 mg, 0.45 mmol, 45%).

***Rf* value** = 0.46 (10% v/v MeOH in CH_2Cl_2);

^{1}H NMR (400 MHz, $CDCl_3$) 8.64 (d, *J* = 4.5 Hz, 1H, H-4), 7.89 (d, *J* = 9.2 Hz, 1H, H-3), 7.81-7.83 (m, 3H, H-19, H-20), 7.76 (d, *J* = 4.6 Hz, 1H, H-5), 7.63 (br s, 1H, H-6), 7.50 (t, *J* = 1.6 Hz, 2H, H-22), 7.44 (d, *J* = 1.7 Hz, 4H, H-21), 7.24 (dd, *J* = 9.2 Hz, 2.6 Hz, 1H, H-2), 6.86 (br s, 1H, H-8), 6.69 (d, *J* = 7.2 Hz, 1H, H-7), 5.59 (d, *J* = 13.2 Hz, 1H, H-18a), 5.15 (d, *J* = 12.8 Hz, 1H, H-18b), 4.59 (q, *J* = 8.6 Hz, 1H, H-9), 4.31 (t, *J* = 10.0 Hz, 1H, H-13a), 4.01 (s, 3H, H-1), 3.82-3.90 (m, 1H, H-13b), 3.67-3.73 (m, 1H, H-17a), 3.16-3.23 (m, 1H, H-17b), 1.89-1.96 (m, 1H, H-16a), 1.38-1.78 (m, 43H, H-10a, H-10b, H-11, H-12, H-14a, H-14b, H-16b, H-23), 0.84 (t, *J* = 7.3 Hz, 3H, H-15) ppm;

^{13}C NMR (101 MHz, $CDCl_3$) δ 158.6, 151.6, 147.7, 145.2, 144.5, 144.1, 139.6, 131.7, 131.3, 128.8, 128.6, 127.3, 122.5, 122.3, 122.0, 120.2, 101.5, 70.8, 67.6, 66.3, 57.0, 56.6, 55.3, 53.6, 36.4, 35.1, 31.6, 26.3, 24.8, 24.7, 11.5 ppm;

IR (neat, cm^{-1}): ν_{max} 2957 (w), 2522 (br), 2159 (w), 2023 (w), 1978 (w), 1620 (w), 1589 (m);

$[\alpha]_D^{25.3}$ = + 8.6 (c. 1.0, $CHCl_3$);

HRMS (+ESI) *m/z* For cation found 793.5672, $[C_{55}H_{73}N_2O_2]^+$ requires 793.5667, (*δ* = + 0.6 ppm).

Note: 5'-(bromomethyl)-3,3'',5,5''-tetra-tert-butyl-1,1':3',1''-terphenyl was prepared on a multigram scale using the procedure reported by Phipps and co-workers.[9] *Both epi-dihydroquinidine and the salt (**4•Br**) were synthesised and characterised by Dr Georgi R. Genov for which we are very grateful. The synthetic route*[21] *and characterisation data*[21–23] *for epi-dihydroquinidine have been reported in the literature.*

(R)-((1S,2S,4S)-5-Formylquinuclidin-2-yl)(6-methoxyquinolin-4-yl)methyl acetate

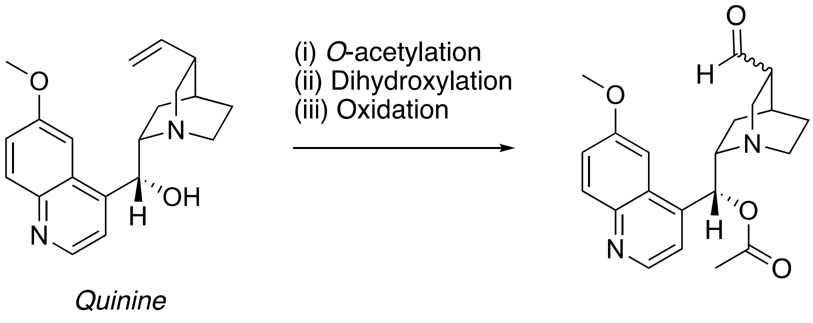

Prepared according to the protocol described by Hiemstra and co-workers.[24] Quinine (2.78 g, 8.6 mmol) was dissolved in a 1:1 mixture of Ac_2O/pyridine (30 mL). 4-Dimethylaminopyridine (170 mg, 1.4 mmol) was added and the resulting mixture was stirred at room temperature overnight. The mixture was evaporated to dryness in a well-ventilated fume hood under a stream of compressed nitrogen and the crude residue was dissolved in CH_2Cl_2 and washed with saturated aqueous $NaHCO_3$ solution. The aqueous layer was extracted with CH_2Cl_2 and the organic layers were combined, washed with brine, dried ($MgSO_4$) and the solvent removed under reduced pressure. The crude residue was dissolved in *tert*-butanol (40 mL) and added to a 3:5 solution of *tert*-butanol/water (160 mL) containing AD-mix α and methanesulfonamide (4.10 g, 43.1 mmol). The mixture was stirred at room temperature for 6 hours following which it was quenched with solid Na_2SO_3 (15.0 g). Water and CH_2Cl_2 were added and the layers were separated. The aqueous layer was thrice extracted with CH_2Cl_2 and the combined organic fractions dried ($MgSO_4$) and the solvent removed under reduced pressure. The crude residue was dissolved in a 1:1 mixture of acetone/water (40 mL) and

cooled to 0 °C. $NaIO_4$ (1.83 g, 8.56 mmol) was added and the resulting mixture stirred at room temperature overnight. The acetone was then removed under reduced pressure following which saturated aqueous $NaHCO_3$ and CH_2Cl_2 were added. The layers were separated and the aqueous layer extracted twice with CH_2Cl_2. The organic fractions were combined, dried ($MgSO_4$) and the solvent removed under reduced pressure. Purification by flash column chromatography (SiO_2, 0-40% v/v 80:20:3 CH_2Cl_2/MeOH/NH_4OH (aq.) in CH_2Cl_2) afforded the title compound as a white foamy solid and as an inconsequential mixture of diastereomers (2.14 g, 5.8 mmol, 68%).

***R**$_f$* **value** = 0.28 (40% v/v 80:20:3 CH_2Cl_2/MeOH/NH_4OH (aq.) in CH_2Cl_2);

^{1}H NMR (400 MHz, $CDCl_3$) δ 9.76 (s, 1H), 8.72-8.74 (m, 1H), 8.00-8.03 (m, 1H), 7.42 (br s, 1H), 7.36-7.39 (m, 1H), 7.32-7.33 (m, 1H), 6.49-6.59 (m, 1H), 3.96-3.98 (m, 3H), 3.25-3.38 (m, 3H), 2.73-2.92 (m, 2H), 2.43-2.55 (m, 2H), 2.10-2.15 (m, 3H), 1.47-1.83 (m, 4H) ppm; (*Note: Unassigned in order to avoid confusion between the diastereomeric protons within each diastereomer).*

^{13}C NMR (101 MHz, $CDCl_3$) δ 203.4, 203.1, 170.1, 169.9, 158.3, 158.2, 147.5*, 145.0, 144.9, 143.4, 143.1, 132.1, 132.0, 127.1, 126.9, 122.0*, 119.1, 118.6, 101.5*, 73.4*, 59.5, 58.6, 56.0, 55.9, 49.6, 49.4, 48.8, 48.4, 42.9*, 28.9, 27.1, 25.5, 23.7, 23.6, 22.5, 21.3, 21.2 ppm;

*Corresponds to two ^{13}C environments.

IR (film, cm^{-1}): ν_{max} 2940 (m), 2873 (w), 2834 (w), 2712 (w), 2362 (w), 2342 (w), 1741 (s), 1718 (s), 1620 (s), 1592 (w), 1508 (m), 1474 (m), 1456 (w), 1369 (m), 1303 (w), 1225 (s), 1184 (w), 1134 (w), 1027 (s), 962 (w), 917 (w), 853 (m), 831 (m), 733 (s);

$[\boldsymbol{\alpha}]_{\mathbf{D}}^{\mathbf{25.0}}$ = − 42.5 (c. 1.0, $CHCl_3$);

The spectroscopic data is in agreement with that reported in the literature.[25]

(R)-(6-Methoxyquinolin-4-yl)((1R,2S,4R)-quinuclidin-2-yl)methanol (Desvinylquinine)

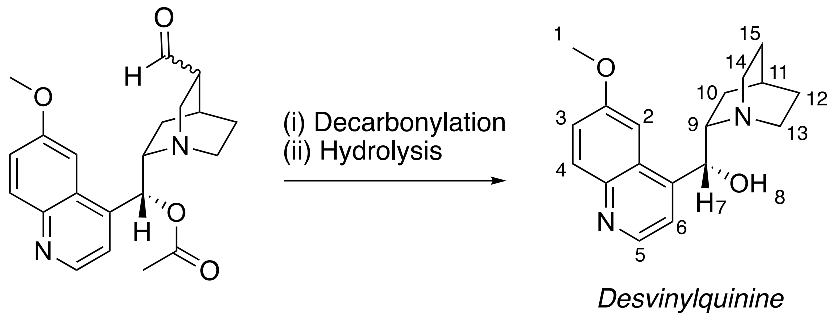

A variant of the protocol described by Hiemstra and co-workers was employed.[24] A 2-necked round-bottom flask was charged with (*R*)-((1*S*,2*S*,4*S*)-5-formylquinuclidin-2-yl)(6-methoxyquinolin-4-yl)methyl acetate (820 mg, 2.2 mmol) and diglyme (30 mL). The flask was evacuated and backfilled with nitrogen ten times. To the flask were added $[Rh(COD)Cl]_2$ (24.7 mg, 0.050 mmol) and 1,3-bis(diphenylphosphino)propane (90.0 mg, 0.22 mmol) under nitrogen atmosphere and another ten vacuum/nitrogen cycles were performed followed by a thorough sparging of the solution with nitrogen for 30 minutes. The resulting mixture was heated to 140 °C overnight. The solution was then allowed to cool to room temperature and diluted with water (200 mL), brine (20 mL) and CH_2Cl_2 (200 mL). The layers were separated and the aqueous layer was extracted with CH_2Cl_2 (2 x 100 mL). The volatiles were then removed under reduced pressure to afford a crude residue. The residue was dissolved in MeOH (30 mL) and cooled to 0 °C. K_2CO_3 (1.50 g, 10.9 mmol) was added and the mixture was allowed to warm to room temperature and stirred for 2 hours. The volatiles were then removed under a steam of air, the reaction quenched with water (80 mL), extracted with CH_2Cl_2 (3 x 100 mL), dried ($MgSO_4$) and the solvent removed under reduced pressure. Purification by flash column chromatography (0-55% v/v 80:20:3 CH_2Cl_2/MeOH/NH_4OH (aq.) in CH_2Cl_2) afforded the title compound as a pale-yellow solid (299 mg, 1.0 mmol, 45%).

***R_f* value** = 0.18 (40% v/v 80:20:3 CH_2Cl_2/MeOH/NH_4OH (aq.) in CH_2Cl_2);

^{1}H NMR (400 MHz, $CDCl_3$) δ 8.66 (d, *J* = 4.5 Hz, 1H, H-5), 7.95 (d, *J* = 9.2 Hz, 1H, H-4), 7.53 (d, *J* = 4.5 Hz, 1H, H-6), 7.29 (dd, *J* = 9.2 Hz, 2.6 Hz, 1H, H-3), 7.19 (d, *J* = 2.6 Hz, 1H, H-2), 5.68 (d, *J* = 3.2 Hz, 1H, H-7), 3.84 (s, 3H, H-1), 3.52-3.59 (m, 1H, H-13a), 3.18 (td, *J* = 9.0 Hz, 4.0 Hz, 1H, H-9), 2.83-2.96 (m, 2H, H-14a, H-14b), 2.72-2.80 (m, 1H, H-13b), 1.81-1.86 (m, 2H, H-10a, H-11), 1.60-1.66 (m, 1H, H-12a), 1.41-1.54 (m, 3H, H-12b, H-15a, H-15b), 1.29-1.35 (m, 1H, H-10b) ppm;

^{13}C NMR (101 MHz, $CDCl_3$) δ 157.9, 147.7, 147.5, 144.3, 131.7, 126.7, 121.7, 118.6, 101.3, 71.6, 59.9, 55.9, 50.9, 44.2, 26.3, 26.2, 25.4, 22.0 ppm;

IR (film, cm^{-1}): ν_{max} 3147 (br), 2937 (m), 2865 (m), 2361 (w), 1621 (m), 1591 (w), 1508 (m), 1471 (m), 1456 (w), 1431 (w), 1363 (w), 1328 (w), 1241 (s), 1227 (s), 1205 (w), 1182 (w), 1122 (w), 1084 (m), 1030 (s), 989 (w), 907 (s), 830 (m), 728 (s);

$[\boldsymbol{\alpha}]_{\mathbf{D}}^{\mathbf{25.0}}$ = − 116.0 (c. 1.0, $CHCl_3$);

HRMS (+ESI) *m/z* found $[M+H]^+$ 299.1756, $[C_{18}H_{23}N_2O_2]^+$ requires 299.1754, (δ = + 0.7 ppm).

(1R,2S,4R)-2-((R)-Hydroxy(6-methoxyquinolin-4-yl)methyl)-1-((3,3'',5,5''-tetra-tert-butyl-[1,1':3',1''-terphenyl]-5'-yl)methyl)quinuclidin-1-ium bromide (**10•Br**)

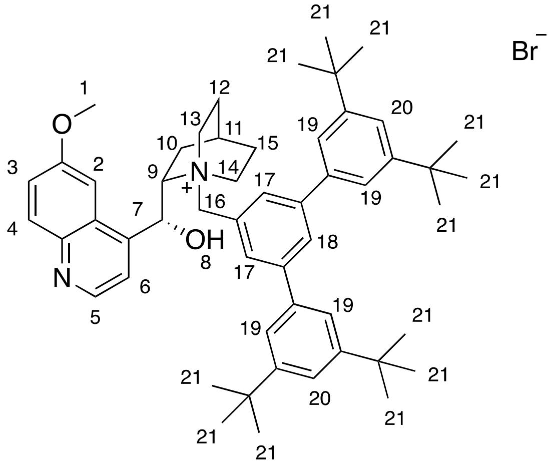

Prepared according to **GP2** on a 0.34 mmol scale with respect to desvinylquinine and using 5'-(bromomethyl)-3,3'',5,5''-tetra-tert-butyl-1,1':3',1''-terphenyl as the benzyl bromide. Purification by flash column chromatography (0-12% v/v MeOH in CH_2Cl_2) afforded the title compound as a white amorphous solid (219 mg, 0.26 mmol, 76%).

***R$_f$* value** = 0.39 (10% v/v MeOH in CH_2Cl_2);

^{1}H NMR (400 MHz, $CDCl_3$) δ 8.75 (d, J = 4.5 Hz, 1H, H-5), 8.06 (d, J = 9.2 Hz, 1H, H-4), 7.96 (br s, 2H, H-17), 7.89 (br s, 1H, H-18), 7.78 (d, J = 4.5 Hz, 1H, H-6), 7.51 (t, J = 1.7 Hz, 2H, H-20), 7.49 (d, J = 1.7 Hz, 4H, H-19), 7.38 (dd, J = 9.2 Hz, 2.5 Hz, 1H, H-3), 7.28 (d, J = 2.5 Hz, 1H, H-2), 7.03 (d, J = 6.8 Hz, 1H, H-8), 6.80 (d, J = 6.6 Hz, 1H, H-7), 6.57 (d, J = 12.2 Hz, 1H, H-16a), 5.14 (t, J = 11.6 Hz, 1H, H-14a), 4.54 (d, J = 12.1 Hz, 1H, H-16b), 3.96 (s, 3H, H-1), 3.79 (t, J = 8.7 Hz, 1H, H-9), 3.34-3.50 (m, 3H, H-13a, H-13b, H-14b), 2.45-2.49 (m, 1H, H-10a), 2.19-2.25 (m, 1H, H-15a), 2.15 (br s, 1H, H-11), 1.81-1.85 (m, 2H, H-12a, H-12b), 1.68 (t, J = 9.4 Hz, 1H, H-15b), 1.40 (s, 37H, H-10b, H-21) ppm;

^{13}C NMR (126 MHz, $CDCl_3$) δ 158.4, 151.8, 147.1, 144.4*, 143.4, 139.5, 131.7, 131.3, 129.2, 127.7, 126.1, 122.5, 122.1, 121.3, 120.9, 102.3, 70.4, 64.7, 63.9, 57.8, 56.4, 51.4, 35.2, 31.7, 26.1, 24.1, 24.0, 20.6 ppm;

*HMBC shows that this signal corresponds to two ^{13}C environments.

IR (neat, cm^{-1}): ν_{max} 3200 (br), 2953 (s), 2902 (m), 2866 (m), 2361 (m), 2324 (m), 1620 (m), 1589 (s), 1508 (m), 1474 (m), 1460 (m), 1421 (w), 1392 (w), 1361 (s), 1246 (s), 1226 (s), 1202 (w), 1064 (w), 1026 (m), 909 (w), 714 (s), 654 (w);

$[\alpha]_D^{25.0}$ = − 93.5 (c. 1.1, $CHCl_3$);

HRMS (+ESI) *m/z* For cation found 765.5390, $[C_{53}H_{69}N_2O_2]^+$ requires 765.5354, (δ = + 4.7 ppm).

Note: 5'-(bromomethyl)-3,3'',5,5''-tetra-tert-butyl-1,1':3',1''-terphenyl was prepared on a multigram scale using the procedure reported by Phipps and co-workers.[9]

Synthesis of Chiral Rh(II,II) Dimers

Bis[rhodium (1S,2S,4S,5R)-5-ethyl-2-((R)-hydroxy(6-methoxyquinolin-4-yl)methyl)-1-((3,3'',5,5''-tetra-tert-butyl-[1,1':3',1''-terphenyl]-5'-yl)methyl)quinuclidin-1-ium (3,5-bis(2-carboxy-2-methylpropyl)benzenesulfonate)] (Rh_2(**A**)$_2$•(**1**)$_2$)

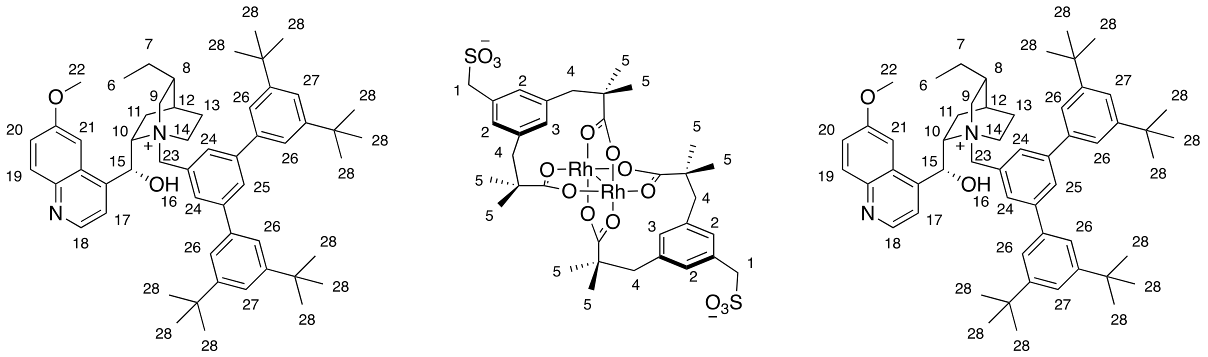

Prepared according to **GP3** on a 0.035 mmol scale with respect to Rh_2(**A**)$_2$•(**Bu_4N**)$_2$ and using (**1•Br**) as the chiral bromide salt. The title compound was obtained as a brown amorphous solid (83.7 mg, 0.033 mmol, 94%).

^{1}H NMR (500 MHz, DMSO-d$_6$) δ 8.84 (br s, 2H, H-18), 8.05 (br s, 2H, H-19), 7.91 (s, 2H, H-25), 7.79 (br s, 6H, H-17, H-24), 7.39-7.49 (m, 16H, H-20, H-21, H-26, H-27), 6.72-6.74 (m, 6H, H-2, H-15), 6.60 (s, 2H, H-16), 6.52 (s, 2H, H-3), 5.54 (d, *J* = 12.1 Hz, 2H, H-23a), 4.74 (br s, 2H, H-23b), 4.18 (br s, 2H, H-14a), 3.99 (s, 6H, H-22), 3.79-3.90 (br s, 2H, H-10), 3.55 (s, 6H, H-9a, H-1a, H-1b), 3.46 (br s, 2H, H-9b), 3.28 (br s, 2H, H-14b)*, 2.50 (s, 8H, H-4a, H-4b)†, 2.28 (br s, 2H, H-11a), 2.12 (br s, 2H, H-13a), 1.96 (br s, 2H, H-12), 1.89 (p, *J* = 8.2 Hz, 2H, H-8), 1.76 (t, J = 11.6 Hz, 2H, H-13b), 1.57 (br s, 2H, H-11b), 1.23-1.40 (m, 76H, H-7a, H-7b, H-28), 0.87 (br s, 24H, H-5a, H-5b), 0.77 (t, *J* = 7.3 Hz, 6H, H-6) ppm;

*obscured by the residual water peak but deduced from the ^{1}H-COSY.

†obscured by the residual solvent peak but deduced from the HMBC.

^{13}C NMR (126 MHz, DMSO-d$_6$) δ 196.5, 157.4, 151.0, 147.6, 144.2, 143.8, 143.1, 139.4, 136.5, 133.8, 131.6, 131.0, 129.7, 129.1, 128.5, 128.0, 125.4, 121.7, 121.6, 121.5, 120.3, 101.8, 68.8,

63.6, 62.0, 61.1, 57.7, 55.4, 51.0, 46.2, 45.6, 34.9, 34.7, 31.3, 25.5, 25.4, 24.8, 24.5, 19.9, 11.1 ppm;

IR (neat, cm^{-1}): ν_{max} 2957 (s), 1586 (s), 1509 (w), 1474 (m), 1459 (m), 1407 (m), 1361 (m), 1246 (s), 1172 (s), 1034 (s), 868 (s);

$[\alpha]_D^{25.0}$ = − 31.6 (c. 0.22, $CHCl_3$);

HRMS (+ESI) *m/z* For cation found 793.5702, $[C_{55}H_{73}N_2O_2]^+$ requires 793.5667, (δ = + 4.4 ppm).

HRMS (−ESI) *m/z* For dianion found 472.0066, $[C_{34}H_{42}O_{14}Rh_2S_2]^{2-}$ requires 472.0069, (δ = − 0.6 ppm).

Bis[rhodium (1S,2R,4S,5R)-5-ethyl-2-((S)-hydroxy(6-methoxyquinolin-4-yl)methyl)-1-((3,3'',5,5''-tetra-tert-butyl-[1,1':3',1''-terphenyl]-5'-yl)methyl)quinuclidin-1-ium (3,5-bis(2-carboxy-2-methylpropyl)benzenesulfonate)] (Rh_2(**A**)$_2$•(**2a**)$_2$)

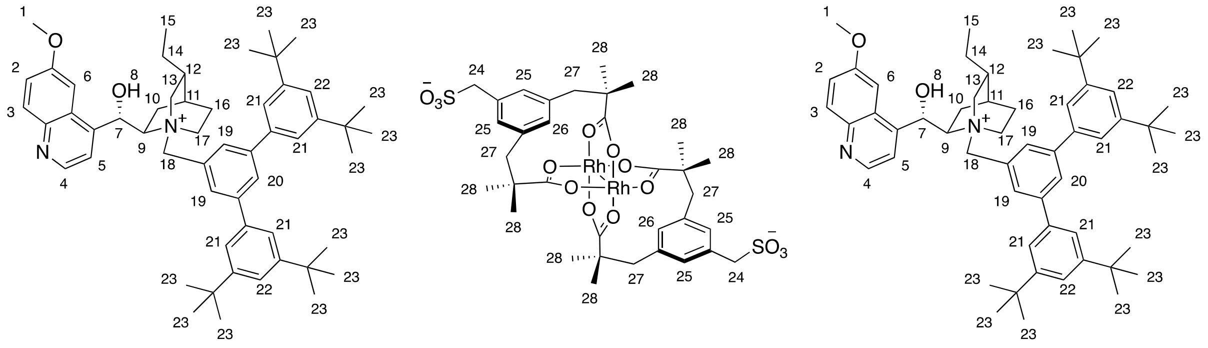

Prepared according to **GP3** on a 0.041 mmol scale with respect to Rh_2(**A**)$_2$•(**Bu_4N**)$_2$ and using (**2a•Br**) as the chiral bromide salt. The title compound was obtained as a brown amorphous solid (73.4 mg, 0.029 mmol, 71%).

^{1}H NMR (500 MHz, C_5D_5N) δ 9.07 (d, J = 4.5 Hz, 2H, H-4), 8.64 (br s, 2H, H-8), 8.54 (s, 4H, H-19), 8.37 (d, J = 9.2 Hz, 2H, H-3), 8.26 (br s, 2H, H-20), 8.06 (d, J = 4.5 Hz, 2H, H-5), 7.93 (d, J = 1.6 Hz, 8H, H-21), 7.90 (d, J = 2.1 Hz, 2H, H-6), 7.72 (br s, 4H, H-22), 7.53 (dd, J = 9.2 Hz, 2.5 Hz, 2H, H-2), 7.14-7.15 (m, 6H, H-25, H-26), 7.10 (br s, 2H, H-7), 6.06 (d, J = 12.2 Hz, 2H, H-

18a), 5.51 (d, J = 12.3 Hz, 2H, H-18b), 4.54 (t, J = 9.3 Hz, 2H, H-13a), 4.47 (t, J = 11.2 Hz, 2H, H-17a), 4.38 (d, J = 12.9 Hz, 2H, H-24a), 4.34 (d, J = 13.5 Hz, 2H, H-24b), 4.17-4.23 (m, 2H, H-9), 3.97 (s, 6H, H-1), 3.84 (t, J = 11.0 Hz, 2H, H-13b), 3.32 (q, J = 10.0 Hz, 2H, H-17b), 2.62 (t, J = 11.8 Hz, 2H, H-10a), 2.49 (d, J = 12.4 Hz, 4H, H-27a), 2.40 (d, J = 12.4 Hz, 4H, H-27b), 1.67 (br s, 2H, H-11), 1.58-1.59 (m, 2H, H-16a), 1.42-1.54 (m, 80H, H-12, H-14a, H-14b, H-16b, H-23), 1.06-1.12 (m, 2H, H-10b), 1.02 (s, 12H, H-28a), 1.01 (s, 12H, H-28b), 0.76 (t, J = 6.9 Hz, 6H, H-15) ppm;

^{13}C NMR (126 MHz, C_5D_5N) δ 197.2, 159.0, 152.5, 148.5, 145.7, 145.1, 145.0, 141.1, 138.7, 134.7, 133.0, 132.8, 131.2, 130.4, 130.2, 129.3, 127.3, 123.1, 122.9, 122.0, 121.8, 103.6, 69.4, 66.3, 64.1, 59.4, 57.4, 56.8, 56.5, 47.4, 46.3, 36.5, 35.7, 32.1, 26.5, 25.6, 25.1, 25.0, 21.8, 11.9 ppm;

IR (neat, cm^{-1}): ν_{max} 2953 (s), 2909 (s), 2874 (s), 1622 (w), 1585 (s), 1512 (m), 1473 (m), 1459 (m), 1407 (s), 1375 (m), 1360 (w), 1243 (s), 1170 (s);

$[\boldsymbol{\alpha}]_{\mathbf{D}}^{\mathbf{25.0}}$ = + 57.4 (c. 0.33, $CHCl_3$);

HRMS (+ESI) *m/z* For cation found 793.5676, $[C_{55}H_{73}N_2O_2]^+$ requires 793.5667, (δ = + 1.1 ppm).

HRMS (−ESI) *m/z* For dianion found 472.0069, $[C_{34}H_{42}O_{14}Rh_2S_2]^{2-}$ requires 472.0069, (δ = + 0.0 ppm).

Bis[rhodium (1S,2S,4S,5R)-5-ethyl-2-((S)-hydroxy(6-methoxyquinolin-4-yl)methyl)-1-((3,3'',5,5''-tetra-tert-butyl-[1,1':3',1''-terphenyl]-5'-yl)methyl)quinuclidin-1-ium (3,5-bis(2-carboxy-2-methylpropyl)benzenesulfonate)] (Rh_2(**A**)$_2$•(**3**)$_2$)

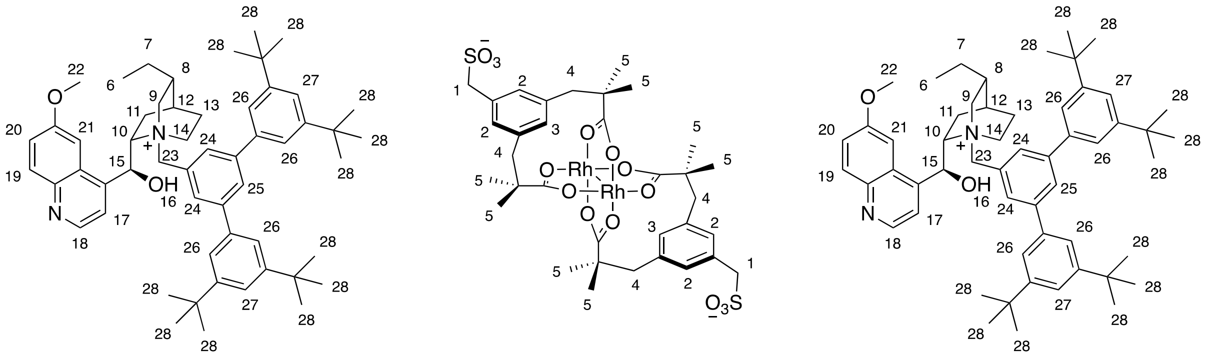

Prepared according to **GP4** on a 0.041 mmol scale with respect to Rh_2(**A**)$_2$•(**Bu_4N**)$_2$ and using (**3•Br**) as the chiral bromide salt. The title compound was obtained as a dark brown amorphous solid (84.1 mg, 0.033 mmol, 81%).

^{1}H NMR (500 MHz, C_5D_5N) δ 8.94 (d, *J* = 4.5 Hz, 2H, H-18), 8.57 (br s, 4H, H-24), 8.54 (d, *J* = 5.2 Hz, 2H, H-16), 8.36 (d, *J* = 9.2 Hz, 2H, H-19), 8.30 (s, 2H, H-25), 8.24 (d, *J* = 1.8 Hz, 2H, H-21), 7.90 (d, *J* = 1.6 Hz, 8H, H-26), 7.81 (d, *J* = 4.4 Hz, 2H, H-17), 7.72 (t, *J* = 1.6 Hz, 4H, H-27), 7.61 (d, *J* = 2.5 Hz, 2H, H-20), 7.31 (s, 4H, H-2), 7.22 (s, 2H, H-3)*, 6.93 (br s, 2H, H-15), 5.77 (d, *J* = 12.6 Hz, 2H, H-23a), 5.45 (br s, 2H, H-23b), 5.05 (br s, 2H, H-14a), 4.58 (br s, 2H, H-10), 4.45 (s, 4H, H-1a, H-1b), 4.22 (t, *J* = 11.0 Hz, 2H, H-9a), 4.07 (s, 6H, H-22), 3.80 (q, *J* = 12.3 Hz, 2H, H-14b), 3.22 (br s, 2H, H-9b), 2.56-2.62 (m, 8H, H-4a, H-4b), 2.22 (br s, 2H, H-13a), 1.74-1.78 (m, 4H, H-12, H-13b), 1.54 (br s, 4H, H-11a, H-8), 1.49 (s, 72H, H-28), 1.03 (s, 30H, H-5a, H-5b, H-11b, H-7a, H-7b), 0.69 (t, *J* = 7.3 Hz, 6H, H-6) ppm;

*obscured by the residual solvent peak but deduced from the ^{1}H-COSY spectrum

^{13}C NMR (126 MHz, C_5D_5N) δ 197.2, 159.5, 152.5, 148.7, 147.1, 146.1, 144.7, 141.1, 138.8, 134.5, 132.8, 132.7, 131.2, 131.0, 130.4, 129.0, 128.9, 123.5* 122.9†, 121.4, 103.2, 69.4, 69.0, 67.5, 63.6, 59.4, 56.8, 51.6, 47.6, 46.4, 36.1, 35.7, 32.1, 26.5, 26.3, 25.5, 25.4#, 11.7 ppm;

*obscured by residual solvent peak but deduced from the HSQC and HMBC.

†HSQC shows that this signal corresponds to two ^{13}C environments.

#corresponds to two ^{13}C environments (the second, overlapped environment becomes apparent in the DEPT-135).

IR (neat, cm^{-1}): ν_{max} 2955 (m), 2923 (w), 2866 (w), 2361 (w), 2341 (w), 1620 (s), 1585 (w), 1551 (w), 1507 (w), 1474 (m), 1457 (w), 1406 (m), 1360 (w), 1299 (w), 1246 (m), 1219 (m), 1171 (m), 1033 (s), 869 (m), 823 (w), 714 (s);

$[\boldsymbol{\alpha}]_{\mathbf{D}}^{\mathbf{25.0}}$ = − 22.7 (c. 0.15, $CHCl_3$);

HRMS (+ESI) *m/z* For cation found 793.5668, $[C_{55}H_{73}N_2O_2]^+$ requires 793.5667, (δ = + 0.1 ppm).

HRMS (−ESI) *m/z* For dianion found 472.0071, $[C_{34}H_{42}O_{14}Rh_2S_2]^{2-}$ requires 472.0069, (δ = + 0.4 ppm).

Bis[rhodium (1S,2R,4S,5R)-5-ethyl-2-((R)-hydroxy(6-methoxyquinolin-4-yl)methyl)-1-((3,3'',5,5''-tetra-tert-butyl-[1,1':3',1''-terphenyl]-5'-yl)methyl)quinuclidin-1-ium (3,5-bis(2-carboxy-2-methylpropyl)benzenesulfonate)] (Rh_2(**A**)$_2$•(**4**)$_2$)

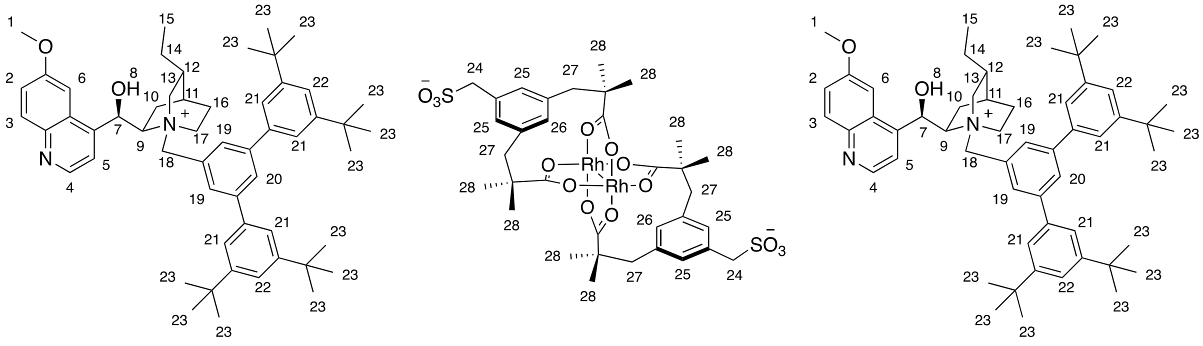

Prepared according to **GP4** on a 0.041 mmol scale with respect to Rh_2(**A**)$_2$•(**Bu_4N**)$_2$ and using (**4•Br**) as the chiral bromide salt. The title compound was obtained as a grey amorphous solid (60.6 mg, 0.024 mmol, 58%).

^{1}H NMR (500 MHz, C_5D_5N) δ 8.96 (d, *J* = 4.4 Hz, 2H, H-4), 8.40 (br s, 4H, H-19), 8.32 (d, *J* = 9.2 Hz, 2H, H-3), 8.23 (s, 2H, H-20), 8.13 (d, *J* = 2.3 Hz, 2H, H-6), 7.97 (d, *J* = 4.1 Hz, 4H, H-5, H-8), 7.90 (d, *J* = 1.4 Hz, 8H, H-21), 7.73 (d, *J* = 1.6 Hz, 4H, H-22), 7.56 (dd, *J* = 9.1 Hz, 2.4 Hz, 2H, H-2), 7.33 (s, 4H, H-25), 7.22 (s, 2H, H-26), 7.02 (br s, 2H, H-7), 5.86 (d, *J* = 13.2 Hz, 2H, H-18a), 5.57 (d, *J* = 13.3 Hz, 2H, H-18b), 4.74 (br s, 2H, H-9), 4.48 (s, 4H, H-24a, H-24b), 4.24-4.32 (m, 4H, H-13a, H-17a), 4.09 (t, *J* = 11.5 Hz, 2H, H-13b), 4.00 (s, 6H, H-1), 3.39 (q, *J* = 10.3 Hz, 2H,

H-17b), 2.58 (s, 8H, H-27a, H-27b), 1.81 (q, J = 10.2 Hz, 2H, H-16a), 1.30-1.60 (m, 82H H-10a, H-11, H-14a, H-14b, H-16b, H-23), 1.15-1.20 (m, 4H, H-10b, H-12), 1.04 (s, 24H, H-28), 0.75 (t, J = 7.3 Hz, 6H, H-15) ppm;

^{13}C NMR (126 MHz, C_5D_5N) δ 197.2, 159.3, 152.5, 148.9, 147.2*, 145.9, 144.7, 141.1, 138.9, 134.5, 132.8†, 131.2, 131.1, 130.4, 128.8#, 123.2, 123.0, 122.8, 121.6, 103.0§, 72.9, 66.6#, 59.4, 56.7, 56.6, 55.1, 47.6, 46.4, 36.4, 35.7, 32.0, 26.7, 26.6, 25.6, 25.3, 25.1, 11.8 ppm;

*weak signal but clearly visible in the HMBC.

†HSQC shows that this signal corresponds to two ^{13}C environments.

#HMBC shows that this signal corresponds to two ^{13}C environments.

§extremely weak signal but clearly visible in the HSQC

IR (neat, cm^{-1}): ν_{max} 2954 (s), 2924 (s), 2867 (m), 2361 (m), 2331 (w), 1621 (w), 1586 (s), 1508 (m), 1474 (m), 1458 (m), 1406 (s), 1360 (m), 1247 (s), 1222 (s), 1172 (s), 1130 (w), 1060 (w), 1033 (s), 868 (s), 829 (m), 779 (w), 765 (w), 714 (s);

$[\boldsymbol{\alpha}]_{\mathbf{D}}^{\mathbf{25.0}}$ = − 36.4 (c. 0.12, C_5H_5N);

HRMS (+ESI) *m/z* For cation found 793.5675, $[C_{55}H_{73}N_2O_2]^+$ requires 793.5667, (δ = + 1.0 ppm).

HRMS (−ESI) *m/z* For dianion found 472.0068, $[C_{34}H_{42}O_{14}Rh_2S_2]^{2-}$ requires 472.0069, (δ = − 0.2 ppm).

Bis[rhodium (1S,2R,4S,5R)-5-ethyl-2-((S)-hydroxy(6-methoxyquinolin-4-yl)methyl)-1-((3,3'',5,5''-tetra-tert-butyl-[1,1':3',1''-terphenyl]-5'-yl)methyl)quinuclidin-1-ium (3,5-bis((1-carboxycyclobutyl)methyl)phenyl)methanesulfonate] (Rh_2(**B**)$_2$•(**2a**)$_2$)

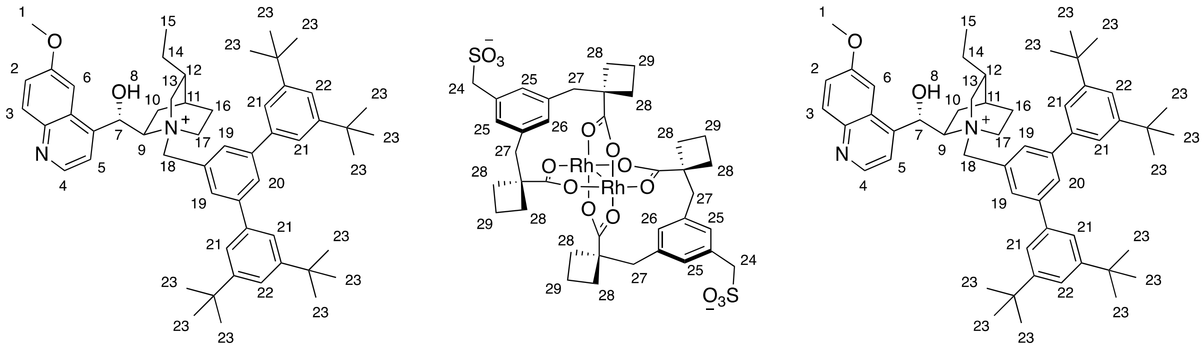

Prepared according to **GP3** on a 0.041 mmol scale with respect to Rh_2(**B**)$_2$•(**Bu_4N**)$_2$ and using (**2a•Br**) as the chiral bromide salt. The title compound was obtained as a brown powder (81.1 mg, 0.031 mmol, 77%).

^{1}H NMR (500 MHz, C_5D_5N) δ 9.11 (d, J = 4.5 Hz, 2H, H-4), 8.56 (s, 2H, H-8), 8.54 (s, 4H, H-19), 8.41 (d, J = 9.2 Hz, 2H, H-3), 8.26 (s, 2H, H-20), 8.06 (d, J = 4.3 Hz, 2H, H-5), 7.94 (d, J = 1.6 Hz, 8H, H-21), 7.86 (br s, 2H, H-6), 7.73 (d, J = 1.5 Hz, 4H, H-22), 7.56-7.59 (m, 2H, H-2), 7.17 (s, 4H, H-25), 7.13 (br s, 2H, H-7), 7.10 (s, 2H, H-26), 6.20 (d, J = 12.4 Hz, 2H, H-18a), 5.46 (d, J = 12.3 Hz, 2H, H-18b), 4.54 (t, J = 9.0 Hz, 2H, H-13a), 4.43 (t, J = 10.6 Hz, 2H, H-17a), 4.15-4.18 (m, 6H, H-9, H-24a, H-24b), 3.96 (s, 6H, H-1), 3.84 (t, J = 10.7 Hz, 2H, H-13b), 3.38 (q, J = 10.2 Hz, 2H, H-17b), 2.78 (d, J = 12.5 Hz, 4H, H-27a), 2.72 (d, J = 12.5 Hz, 4H, H-27b), 2.66 (t, J = 11.4 Hz, 2H, H-10a), 2.29 (br s, 8H, H-28a), 1.55-1.80 (m, 22H, H-11, H-16a, H-16b, H-28b, H-29a, H-29b), 1.38-1.55 (m, 78H, H-12, H-14a, H-14b, H-23), 1.09-1.15 (m, 2H, H-10b), 0.75 (t, J = 7.1 Hz, 6H, H-15) ppm;

^{13}C NMR (126 MHz, C_5D_5N) δ 196.5, 159.0, 152.5, 148.5, 145.7, 145.1, 145.0, 141.1, 138.3, 135.4, 132.9, 132.8, 130.5, 130.4, 129.9, 129.4, 127.3, 123.1, 122.8, 122.0, 121.7, 103.5, 69.6, 66.1, 64.4, 59.3, 57.5, 56.7, 56.4, 52.0, 44.3, 36.5, 35.7, 32.1, 31.2, 25.5, 25.2, 25.0, 21.8, 16.2, 11.9 ppm;

IR (neat, cm^{-1}): ν_{max} 2954 (s), 2866 (m), 1552 (s), 1537 (w), 1508 (w), 1475 (m), 1461 (m), 1406 (s), 1361 (w), 1311 (w), 1300 (w), 1241 (s), 1225 (s), 1173 (s), 1120 (m), 1033 (s), 868 (s), 827 (m), 760 (m), 713 (s), 654 (m);

$[\alpha]_D^{25.0}$ = + 67.9 (c. 0.15, $CHCl_3$)

HRMS (+ESI) *m/z* For cation found 793.5665, $[C_{55}H_{73}N_2O_2]^+$ requires 793.5667, (δ = – 0.3 ppm).

HRMS (−ESI) *m/z* For dianion found 496.0055, $[C_{38}H_{42}O_{14}Rh_2S_2]^{2-}$ requires 496.0069), (δ = – 2.8 ppm).

Bis[rhodium (1S,2R,4S,5R)-5-ethyl-2-((S)-hydroxy(6-methoxyquinolin-4-yl)methyl)-1-((3,3'',5,5''-tetra-tert-butyl-[1,1':3',1''-terphenyl]-5'-yl)methyl)quinuclidin-1-ium (3,5-bis((1-carboxycyclopentyll)methyl)phenyl)methanesulfonate)] (Rh_2(**C**)$_2$•(**2a**)$_2$)

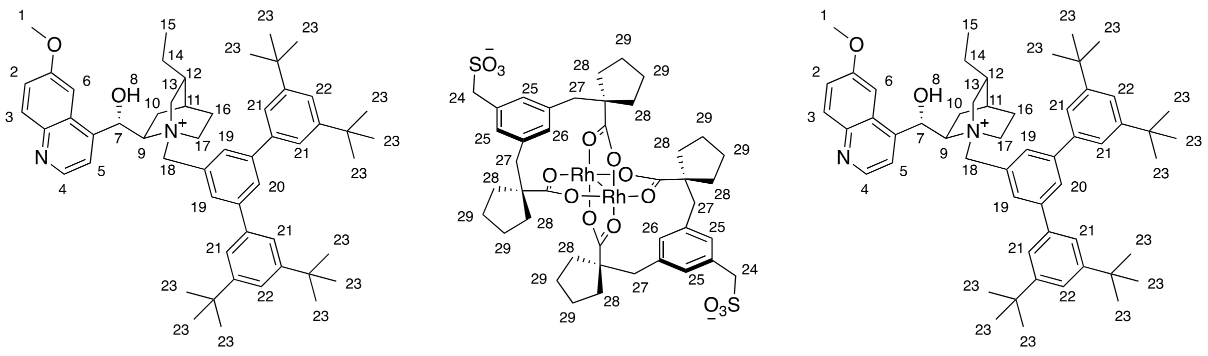

Prepared according to **GP3** on a 0.041 mmol scale with respect to Rh_2(**C**)$_2$•(**Bu$_4$N**)$_2$ and using (**2a•Br**) as the chiral bromide salt. The title compound was obtained as a grey amorphous solid (88.3 mg, 0.034 mmol, 83%).

^{1}H NMR (500 MHz, C_5D_5N) δ 9.07 (d, *J* = 4.5 Hz, 2H, H-4), 8.65 (d, *J* = 4.2 Hz, 2H, H-8), 8.55 (br s, 4H, H-19), 8.38 (d, *J* = 9.5 Hz, 2H, H-3), 8.26 (br s, 2H, H-20), 8.06 (d, *J* = 4.4 Hz, 2H, H-5), 7.94 (d, *J* = 1.8 Hz, 8H, H-21), 7.89 (d, *J* = 2.3 Hz, 2H, H-6), 7.73 (t, *J* = 1.7 Hz, 4H, H-22), 7.55 (dd, *J* = 9.2 Hz, 2.6 Hz, 2H, H-2), 7.17 (s, 6H, H-25, H-26), 7.09 (br s, 2H, H-7), 6.11 (d, *J* = 12.3 Hz, 2H, H-18a), 5.47 (d, *J* = 12.3 Hz, 2H, H-18b), 4.56 (t, *J* = 8.9 Hz, 2H, H-17a), 4.46 (t, *J* = 11.3 Hz, 2H, H-13a), 4.34 (d, *J* = 13.4 Hz, 2H, H-24a), 4.31 (d, *J* = 13.4 Hz, 2H, H-24b), 4.20 (t, *J* = 9.3 Hz, 2H, H-9), 3.98 (s, 6H, H-1), 3.83 (t, *J* = 10.9 Hz, 2H, H-13b), 3.34 (q, *J* = 10.5 Hz, 2H, H-17b), 2.60-2.64 (m, 6H, H-10a, H-27a), 2.54 (d, *J* = 12.5 Hz, 4H, H-27b), 1.97-1.99 (m, 8H, H-28a), 1.68 (br s, 2H, H-11), 1.58-1.62 (m, 2H, H-16a), 1.33-1.53 (m, 104H, H-12, H-14a, H-14b, H-16b, H-23, H-28b, H-29a, H-29b), 1.06-1.11 (m, 2H, H-10b), 0.75 (t, *J* = 7.1 Hz, 6H, H-15) ppm;

^{13}C NMR (126 MHz, C_5D_5N) δ 197.2, 159.0, 152.5, 148.5, 145.7, 145.1, 145.0, 141.1, 139.2, 135.2, 132.9, 132.8, 130.6, 130.5, 130.1, 129.3, 127.3, 123.1, 122.9, 122.0, 121.8, 103.6, 69.5, 66.3, 64.2, 59.4, 58.6, 57.5, 56.8, 56.5, 45.5, 37.0, 36.5, 35.7, 32.1, 25.6, 25.4-25.5 (m), 25.1, 25.0, 21.8, 11.9 ppm;

IR (neat, cm^{-1}): ν_{max} 2957 (s), 2871 (m), 1622 (w), 1579 (s), 1512 (m), 1451 (m), 1401 (s), 1362 (m), 1245 (s), 1226 (s), 1175 (s), 1125 (w), 1035 (s), 868 (s), 828 (w), 765 (w), 713 (s), 654 (m);

$[\boldsymbol{\alpha}]_{\mathbf{D}}^{\mathbf{25.0}}$ = + 39.6 (c. 0.13, $CHCl_3$)

HRMS (+ESI) *m/z* For cation found 793.5649, $[C_{55}H_{73}N_2O_2]^+$ requires 793.5667 (δ = − 2.3 ppm).

HRMS (−ESI) *m/z* For dianion found 524.0381, $[C_{42}H_{50}O_{14}Rh_2S_2]^{2-}$ requires 524.0382, (δ = − 0.2 ppm).

Bis[rhodium (1S,2R,4S,5R)-5-ethyl-2-((S)-hydroxy(6-methoxyquinolin-4-yl)methyl)-1-((3,3'',5,5''-tetra-tert-butyl-[1,1':3',1''-terphenyl]-5'-yl)methyl)quinuclidin-1-ium (3,5-bis((1-carboxycycloheptyl)methyl)phenyl)methanesulfonate)] (Rh_2(**D**)$_2$•(**2a**)$_2$)

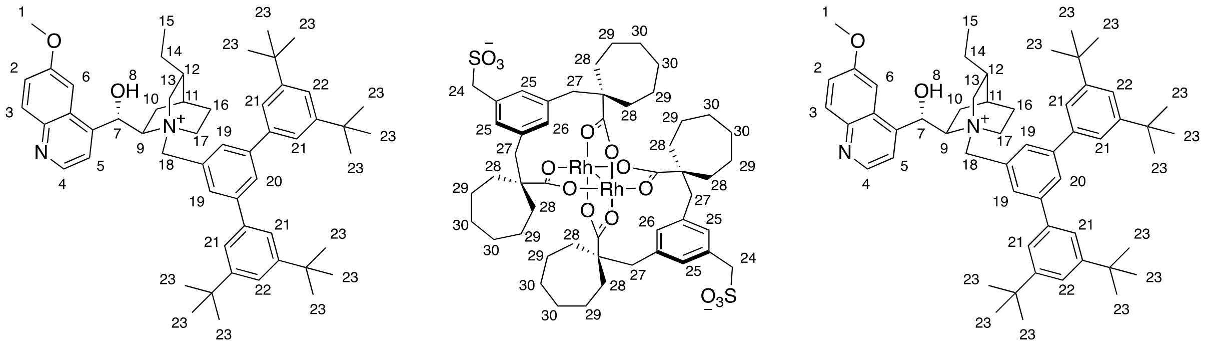

Prepared according to **GP3** on a 0.041 mmol scale with respect to Rh_2(**D**)$_2$•(**Bu_4N**)$_2$ and using (**2a•Br**) as the chiral bromide salt. The title compound was obtained as a brown amorphous solid (86.2 mg, 0.031 mmol, 76%).

^{1}H NMR (500 MHz, C_5D_5N) δ 9.05 (d, *J* = 5.5 Hz, 2H, H-4), 8.66 (d, *J* = 5.4 Hz, 2H, H-8), 8.53 (br s, 4H, H-19), 8.37 (d, *J* = 11.6 Hz, 2H, H-3), 8.28 (br s, 2H, H-20), 8.03 (d, *J* = 5.6 Hz, 2H, H-5),

7.94 (d, *J* = 1.9 Hz, 8H, H-21), 7.85 (d, *J* = 2.8 Hz, 2H, H-6), 7.73 (br s, 4H, H-22), 7.54 (dd, *J* = 11.6 Hz, 3.1 Hz, 2H, H-2), 7.17 (br s, 4H, H-25), 7.10 (br s, 2H, H-26), 6.97 (br s, 2H, H-7), 6.01 (d, *J* = 15.3 Hz, 2H, H-18a), 5.31 (d, *J* = 15.2 Hz, 2H, H-18b), 4.53-4.57 (m, 2H, H-13a), 4.41 (t, *J* = 14.8 Hz, 2H, H-17a), 4.27-4.34 (m, 4H, H-24a, H-24b), 4.19 (t, *J* = 11.4 Hz, 2H, H-9), 3.91 (s, 6H, H-1), 3.82 (t, *J* = 13.4 Hz, 2H, H-13b), 3.33 (q, *J* = 12.7 Hz, 2H, H-17b), 2.48-2.64 (m, 10H, H-10a, H-27a, H-27b), 1.96-2.01 (m, 8H, H-28a), 1.66 (br s, 4H, H-11, H-16a), 1.48 (br s, 120H, H-12, H-14a, H-14b, H-16b, H-23, H-28b, H-29a, H-29b, H-30a, H-30b), 1.04-1.10 (m, 2H, H-10b), 0.73 (t, *J* = 8.6 Hz, 6H, H-15) ppm;

^{13}C NMR (126 MHz, C_5D_5N) δ 197.4, 158.9, 152.5, 148.5, 145.6, 145.0*, 141.1, 138.3, 134.6, 133.0, 132.8, 131.1, 130.8, 130.4, 129.3, 127.3, 123.1, 122.9, 121.7†, 103.8, 69.6, 66.4, 64.1, 59.4, 57.5, 56.8, 56.3, 53.5, 47.1, 37.7, 36.4, 35.7, 32.1, 31.6, 25.5, 25.2, 25.0, 24.5, 21.8, 11.9 ppm;

*HMBC shows that this signal corresponds to two ^{13}C environments.

†HSQC shows that this signal corresponds to two ^{13}C environments.

IR (neat, cm^{-1}): ν_{max} 2958 (s), 1865 (m), 1622 (m), 1579 (s), 1553 (w), 1538 (w), 1509 (w), 1459 (m), 1397 (s), 1362 (m), 1300 (m), 1243 (s), 1225 (s), 1173 (s), 1130 (m), 1033 (s), 963 (w), 868 (s), 825 (m), 765 (w), 713 (s);

$[\boldsymbol{\alpha}]_{\mathbf{D}}^{\mathbf{25.0}}$ = + 29.7 (c. 0.18, $CHCl_3$);

HRMS (+ESI) *m/z* For cation found 793.5685, $[C_{55}H_{73}N_2O_2]^+$ requires 793.5667, (δ = + 2.3 ppm).

HRMS (−ESI) *m/z* For dianion found 580.0991, $[C_{50}H_{66}O_{14}Rh_2S_2]^{2-}$ requires 580.1008, (δ = − 2.9 ppm).

Bis[rhodium (1S,2R,4S,5R)-5-ethyl-2-((S)-hydroxy(6-methoxyquinolin-4-yl)methyl)-1-((3,3'',5,5''-tetra-tert-butyl-[1,1':3',1''-terphenyl]-5'-yl)methyl)quinuclidin-1-ium (3,5-bis((1-carboxycyclooctyl)methyl)phenyl)methanesulfonate (Rh_2(**E**)$_2$•(**2a**)$_2$)

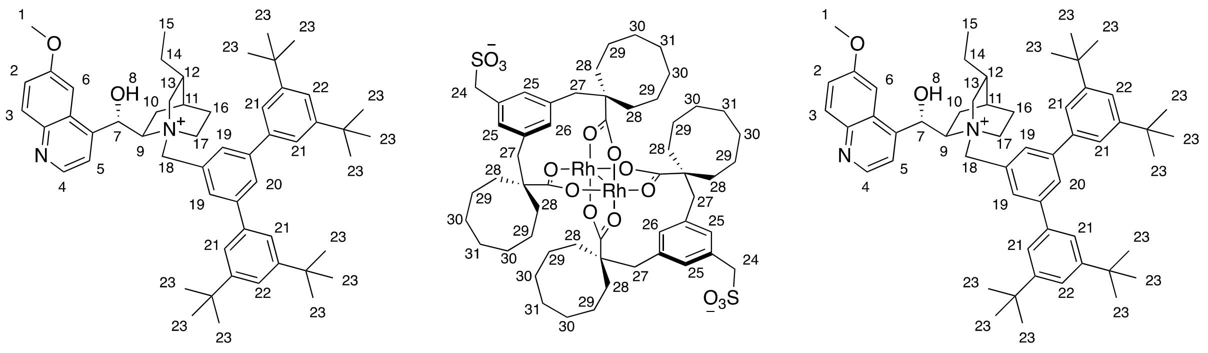

Prepared according to **GP3** on a 0.041 mmol scale with respect to Rh_2(**E**)$_2$•(**Bu$_4$N**)$_2$ and using (**2a•Br**) as the chiral bromide salt. The title compound was obtained as a light brown powder (81.8 mg, 0.029 mmol, 71%).

^{1}H NMR (500 MHz, C_5D_5N) δ 9.05 (d, J = 4.4 Hz, 2H, H-4), 8.71 (s, 2H, H-8), 8.53 (s, 4H, H-19), 8.38 (d, J = 9.2 Hz, 2H, H-3), 8.28 (s, 2H, H-20), 8.01 (d, J = 4.4 Hz, 2H, H-5), 7.94 (d, J = 1.6 Hz, 8H, H-21), 7.84 (d, J = 2.2 Hz, 2H, H-6), 7.74 (s, 4H, H-22), 7.54 (dd, J = 9.2 Hz, 2.5 Hz, 2H, H-2), 7.18 (s, 4H, H-25), 7.04 (s, 2H, H-26), 6.90 (s, 2H, H-7), 5.96 (d, J = 12.2 Hz, 2H, H-18a), 5.22 (d, J = 12.3 Hz, 2H, H-18b), 4.55 (t, J = 9.1 Hz, 2H, H-13a), 4.42 (t, J = 11.3 Hz, 2H, H-17a), 4.23-4.29 (m, 4H, H-24a, H-24b), 4.20 (t, J = 9.3 Hz, 2H, H-9), 3.90 (s, 6H, H-1), 3.81 (t, J = 10.6 Hz, 2H, H-13b), 3.33 (q, J = 10.2 Hz, 2H, H-17b), 2.60 (t, J = 11.4 Hz, 2H, H10a), 2.45-2.51 (m, 8H, H-27a, H-27b), 1.99-2.05 (m, 8H, H-28a), 1.28-1.66 (m, 132H, H-11, H-12, H-14a, H-14b, H-16a, H-16b, H-23, H-28b, H-29a, H-29b, H-30a, H-30b, H-31a, H-31b), 1.01-1.08 (m, 2H, H-10b), 0.73 (t, J = 7.1 Hz, 6H, H-15) ppm;

^{13}C NMR (126 MHz, C_5D_5N) δ 196.8, 158.9, 152.5, 148.5, 145.6, 145.0, 144.9, 141.1, 138.2, 134.6, 133.0, 132.8, 131.2, 131.1, 130.4, 129.3, 127.3, 123.1, 122.9, 121.7, 121.6, 103.9, 69.6, 66.5, 64.1, 59.3, 57.6, 56.3, 53.9, 45.0, 36.4, 35.7, 32.4, 32.1, 29.4, 26.2, 25.5, 25.2, 25.0, 24.5, 24.4, 21.8, 11.9 ppm;

IR (neat, cm^{-1}): ν_{max} 2952 (s), 2921 (s), 2860 (m), 1578 (s), 1473 (m), 1393 (s), 1362 (m), 1300 (w), 1245 (s), 1225 (s), 1174 (s), 1122 (m), 1033 (s), 868 (s), 825 (s), 782 (w), 712 (s), 655 (w), 623 (m);

$[\alpha]_D^{25.0}$ = + 53.4 (c. 0.15, $CHCl_3$);

HRMS (+ESI) For cation found 793.5669, $[C_{55}H_{73}N_2O_2]^+$ requires 793.5667, (δ = + 0.3 ppm).

HRMS (−ESI) For dianion found 608.1344, $[C_{54}H_{74}O_{14}Rh_2S_2]^{2-}$ requires 608.1321, (δ = + 3.8 ppm).

Bis[rhodium (1S,2R,4S,5R)-5-ethyl-2-((S)-hydroxy(6-methoxyquinolin-4-yl)methyl)-1-((3,3'',5,5''-tetrakis(trifluoromethyl)-[1,1':3',1''-terphenyl]-5'-yl)methyl)quinuclidin-1-ium (3,5-bis((1-carboxycycloheptyl)methyl)phenyl)methanesulfonate)] (Rh_2(**D**)$_2$•(**2b**)$_2$)

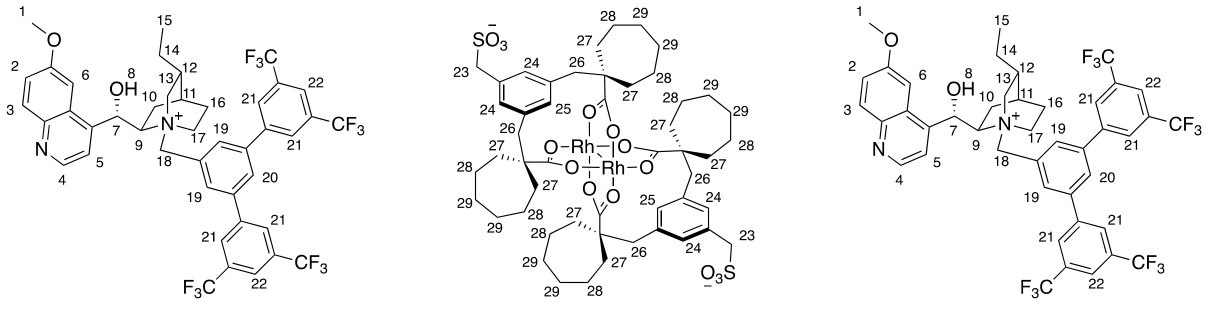

Prepared according to **GP4** on a 0.041 mmol scale with respect to Rh_2(**D**)$_2$•(**Bu$_4$N**)$_2$ and using (**2b•Br**) as the chiral bromide salt. The title compound was obtained as a dark brown amorphous solid (85.6 mg, 0.030 mmol, 73%).

^{1}H NMR (500 MHz, C_5D_5N) δ 9.05 (d, J = 4.0 Hz, 2H, H-4), 8.87 (s, 4H, H-19), 8.65 (s, 8H, H-21), 8.45 (s, 4H, H-8, H-20), 8.36 (d, J = 9.1 Hz, 2H, H-3), 8.15 (s, 4H, H-22), 8.06 (d, J = 4.3 Hz, 2H, H-5), 7.96 (s, 2H, H-6), 7.53 (d, J = 9.1 Hz, 2H, H-2), 7.17 (s, 4H, H-24), 7.08 (br s, 4H, H-7, H-25), 6.24 (d, J = 12.3 Hz, 2H, H-18a), 5.69 (d, J = 12.3 Hz, 2H, H-18b), 4.64 (t, J = 9.5 Hz, 2H, H-13a), 4.49 (br s, 2H, H-17a), 4.31-4.37 (m, 4H, H-23a, H-23b), 4.23 (t, J = 9.1 Hz, 2H, H-9), 4.01 (t, J = 10.9 Hz, 2H, H-13b), 3.95 (s, 6H, H-1), 3.52 (q, J = 9.7 Hz, 2H, H-17b), 2.56-2.60 (m, 6H,

H-10a, H-26a), 2.49 (d, J = 12.3 Hz, 4H, H-26b), 1.97-2.01 (m, 8H, H-27a), 1.69 (s, 2H, H-11), 1.48-1.65 (m, 50H, H-12, H-14a, H-14b, H-16a, H-16b, H-27b, H-28a, H-28b, H-29a, H-29b), 1.06 (br s, 2H, H-10b), 0.71 (t, J = 7.3 Hz, 6H, H-15) ppm;

^{13}C NMR (126 MHz, C_5D_5N) δ 197.4, 159.0, 148.4, 145.6, 144.7, 142.9, 140.6, 138.3, 134.7, 134.3, 133.0, 132.5 (q, $J_{C\text{-}F}$ = 33.1 Hz), 131.6, 131.2, 130.8, 129.2, 127.3, 124.4 (q, $J_{C\text{-}F}$ = 273.3 Hz), 122.5, 121.8, 121.4†, 104.1, 69.5, 66.6, 63.5, 59.2, 57.7, 57.1, 56.4, 53.6, 47.2, 37.7, 36.6, 31.7, 25.4, 25.0, 24.9, 24.5-24.6 (m), 21.9, 11.9 ppm;

†HSQC shows that this signal corresponds to two ^{13}C environments.

^{19}F NMR (376 MHz, C_5D_5N) δ – 62.0 (s) ppm;

IR (neat, cm^{-1}): ν_{max} 2927 (m), 2856 (m), 1622 (w), 1578 (m), 1459 (m), 1397 (m), 1370 (m), 1301 (w), 1277 (s), 1240 (w), 1171 (s), 1128 (s), 1033 (m), 963 (w), 883 (m), 844 (m), 824 (m), 763 (w);

$[\boldsymbol{\alpha}]_{\mathbf{D}}^{\mathbf{25.0}}$ = + 53.1 (c. 0.15, C_5H_5N);

HRMS (+ESI) *m/z* For cation found 841.2634, $[C_{43}H_{37}F_{12}N_2O_2]^+$ requires 841.2658, (δ = + 2.9 ppm).

HRMS (−ESI) *m/z* For dianion found 580.1009, $[C_{50}H_{66}O_{14}Rh_2S_2]^{2-}$ requires 580.1008, (δ = – 0.2 ppm).

Bis[rhodium (1S,2R,4S,5R)-1-([1,1':3',1''-terphenyl]-5'-ylmethyl)-5-ethyl-2-((S)-hydroxy(6-methoxyquinolin-4-yl)methyl)quinuclidin-1-ium (3,5-bis((1-carboxycycloheptyl)methyl)phenyl)methanesulfonate)] (Rh_2(**D**)$_2$•(**2c**)$_2$)

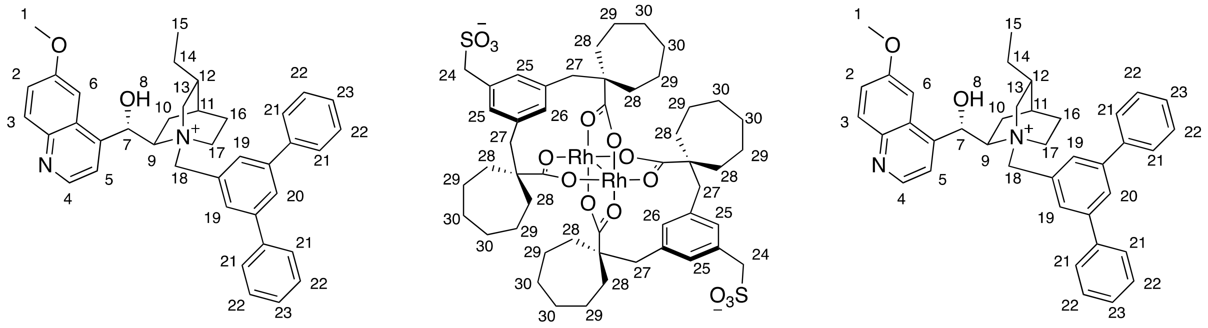

Prepared according to **GP4** on a 0.041 mmol scale with respect to Rh_2(**D**)$_2$•(**Bu_4N**)$_2$ and using (**2c•Br**) as the chiral bromide salt. The title compound was obtained as a dark brown amorphous solid (67.2 mg, 0.029 mmol, 71%).

^{1}H NMR (500 MHz, C_5D_5N) δ 9.10 (d, J = 4.5 Hz, 2H, H-4), 8.59 (d, J = 4.2 Hz, 2H, H-8), 8.42 (d, J = 1.4 Hz, 4H, H-19), 8.40 (d, J = 9.3 Hz, 2H, H-3), 8.13 (t, J = 1.4 Hz, 2H, H-20), 8.08 (d, J = 4.5 Hz, 2H, H-5), 7.99-8.01 (m, 8H, H-21), 7.92 (d, J = 2.5 Hz, 2H, H-6), 7.53-7.57 (m, 10H, H-2, H-22), 7.43 (t, J = 7.4 Hz, 4H, H-23), 7.08-7.09 (m, 8H, H-7, H-25, H-26), 5.96 (d, J = 12.4 Hz, 2H, H-18a), 5.44 (d, J = 12.4 Hz, 2H, H-18b), 4.49-4.53 (m, 2H, H-13a), 4.42 (t, J = 11.0 Hz, 2H, H-17a), 4.23-4.29 (m, 4H, H-24a, H-24b), 4.19 (t, J = 9.3 Hz, 2H, H-9), 4.00 (s, 6H, H-1), 3.75 (t, J = 10.1 Hz, 2H, H-13b), 3.30 (q, J = 10.3 Hz, 2H, H-17b), 2.61 (t, J = 11.6 Hz, 2H, H-10a), 2.55 (d, J = 12.4 Hz, 4H, H-27a), 2.47 (d, J = 12.4 Hz, 4H, H-27b), 1.95-1.98 (m, 8H, H-28a), 1.70 (s, 2H, H-11), 1.61-1.65 (m, 2H, H-16a), 1.57-1.61 (m, 2H, H-16b), 1.40-1.48 (m, 46H, H-12, H-14a, H-14b, H-28b, H-29a, H-29b, H-30a, H-30b), 1.04-1.09 (m, 2H, H-10b), 0.73 (t, J = 7.0 Hz, 6H, H-15) ppm;

^{13}C NMR (126 MHz, C_5D_5N) δ 197.4, 159.1, 148.5, 145.7, 144.9, 143.2, 140.7, 138.3, 134.5, 133.0, 132.5, 131.1, 130.7, 130.5, 129.9, 128.8, 128.4, 128.1, 127.3, 122.0, 121.7, 103.6, 69.4, 66.3, 64.1, 59.4, 57.6, 56.9, 56.6, 53.6, 47.2, 37.7-37.8 (m), 36.5, 31.7, 25.3, 25.1, 24.8, 24.5-24.6 (m), 21.8, 12.0 ppm;

IR (neat, cm^{-1}): ν_{max} 2919 (m), 2853 (w), 1622 (w), 1577 (s), 1511 (w), 1459 (m), 1397 (s), 1348 (w), 1243 (m), 1225 (s), 1173 (s), 1033 (s), 999 (w), 882 (w), 860 (w), 826 (w), 760 (s), 718 (m), 699 (s);

$[\boldsymbol{\alpha}]_{\mathbf{D}}^{\mathbf{25.0}}$ = + 33.0 (c. 0.15, C_5H_5N);

HRMS (+ESI) *m/z* For cation found 569.3172, $[C_{39}H_{41}N_2O_2]^+$ requires 569.3163, (δ = + 1.6 ppm).

HRMS (−ESI) *m/z* For dianion found 580.0996, $[C_{50}H_{66}O_{14}Rh_2S_2]^{2-}$ requires 580.1008, (δ = − 2.0 ppm).

Bis[rhodium (1S,2R,4S,5R)-1-(3,5-di-tert-butylbenzyl)-5-ethyl-2-((S)-hydroxy(6-methoxyquinolin-4-yl)methyl)quinuclidin-1-ium (3,5-bis((1-carboxycycloheptyl)methyl)phenyl)methanesulfonate)] (Rh_2(**D**)$_2$•(**2d**)$_2$)

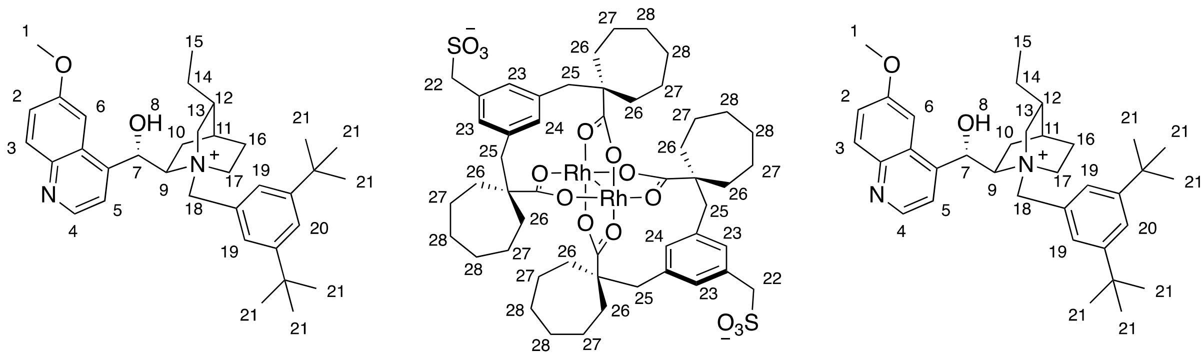

Prepared according to **GP3** on a 0.041 mmol scale with respect to Rh_2(**D**)$_2$•(**Bu_4N**)$_2$ and using (**2d•Br**) as the chiral bromide salt. The title compound was obtained as a brown amorphous solid (72.3 mg, 0.033 mmol, 79%).

^{1}H NMR (500 MHz, C_5D_5N) δ 9.08 (d, *J* = 4.5 Hz, 2H, H-4), 8.63 (d, *J* = 4.4 Hz, 2H, H-8), 8.39 (d, *J* = 9.2 Hz, 2H, H-3), 8.05 (d, *J* = 4.5 Hz, 2H, H-5), 7.96 (d, *J* = 1.6 Hz, 4H, H-19), 7.84 (d, *J* = 2.5 Hz, 2H, H-6), 7.73 (t, *J* = 1.6 Hz, 2H, H-20), 7.56 (dd, *J* = 9.3 Hz, 2.7 Hz, 2H, H-2), 7.09 (s, 6H, H-23, H-24), 6.97 (d, *J* = 2.9 Hz, 2H, H-7), 5.75 (d, *J* = 12.2 Hz, 2H, H-18a), 5.20 (d, *J* = 12.2 Hz, 2H, H-18b), 4.50 (t, *J* = 9.2 Hz, 2H, H-13a), 4.38-4.43 (m, 2H, H-17a), 4.21-4.27 (m, 4H, H-22a, H-22b), 4.14 (t, *J* = 9.4 Hz, 2H, H-9), 3.95 (s, 6H, H-1), 3.71 (t, *J* = 10.9 Hz, 2H, H-13b), 3.29 (q, *J* = 10.2 Hz, 2H, H-17b), 2.60 (t, *J* = 11.4 Hz, 2H, H-10a), 2.52 (d, *J* = 12.4 Hz, 4H, H-25a), 2.45 (d, *J* = 12.5 Hz, 4H, H-25b), 1.95-2.01 (m, 8H, H-26a), 1.65 (s, 2H, H-11), 1.42-1.62 (m, 86H, H-12, H-14a, H-14b, H-16a, H-16b, H-21, H-26b, H-27a, H-27b, H-28a, H-28b), 1.01-1.07 (m, *J* = 6.4 Hz, 2H, H-10b), 0.78 (t, *J* = 7.2 Hz, 6H, H-15) ppm;

^{13}C NMR (126 MHz, C_5D_5N) δ 197.4, 159.0, 152.5, 148.5, 145.6, 145.0, 138.3, 134.6, 133.0, 131.1, 130.8, 129.8, 128.5, 127.3, 124.5 121.9, 121.7, 103.7, 69.2, 66.2, 64.7, 59.4, 57.3, 56.6, 56.4, 53.6, 47.1, 37.7, 36.4, 35.6, 32.0, 31.7, 25.6, 25.1, 25.0, 24.4-24.5 (m), 21.7, 12.0 ppm;

IR (neat, cm^{-1}): ν_{max} 2923 (s), 2854 (m), 1723 (w), 1578 (s), 1512 (m), 1460 (s), 1363 (w), 1244 (s), 1223 (s), 1172 (s), 1120 (s), 1082 (s), 880 (w), 858 (w), 761 (w), 718 (s), 604 (s), 569 (s), 517 (s);

$[\boldsymbol{\alpha}]_{\mathbf{D}}^{\mathbf{25.0}}$ = + 17.9 (c. 0.29, C_5H_5N);

HRMS (+ESI) *m/z* For cation found 529.3798, $[C_{35}H_{49}N_2O_2]^+$ requires 529.3789, (δ = + 1.7 ppm).

HRMS (−ESI) *m/z* For dianion found 580.1015, $[C_{50}H_{66}O_{14}Rh_2S_2]^{2-}$ requires 580.1008, (δ = + 1.2 ppm).

Bis[rhodium (1S,2R,4S,5R)-1-(3,5-bis(trifluoromethyl)benzyl)-5-ethyl-2-((S)-hydroxy(6-methoxyquinolin-4-yl)methyl)quinuclidin-1-ium (3,5-bis((1-carboxycycloheptyl)methyl)phenyl)methanesulfonate)] (Rh_2(**D**)$_2$•(**2e**)$_2$)

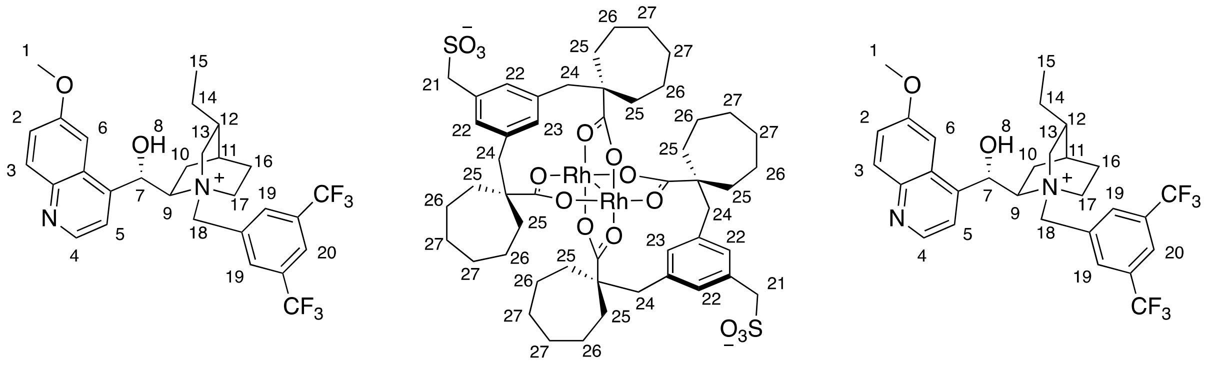

Prepared according to **GP4** on a 0.041 mmol scale with respect to Rh_2(**D**)$_2$•(**Bu_4N**)$_2$ and using (**2e•Br**) as the chiral bromide salt. The title compound was obtained as a brown amorphous solid (81.7 mg, 0.036 mmol, 88%).

^{1}H NMR (500 MHz, C_5D_5N) δ 9.08 (d, *J* = 4.5 Hz, 2H, H-4), 8.90 (br s, 4H, H-19), 8.72 (br s, 2H, H-8)*, 8.38 (d, *J* = 9.2 Hz, 2H, H-3), 8.16 (br s, 2H, H-20), 8.12 (d, *J* = 4.4 Hz, 2H, H-5), 8.04 (d, *J* = 2.3 Hz, 2H, H-6), 7.57 (dd, *J* = 9.8 Hz, 2.6 Hz, 2H, H-2), 7.10 (br s, 6H, H-22, H-23), 6.97 (br s, 2H, H-7), 5.82 (d, *J* = 12.5 Hz, 2H, H-18a), 5.68 (d, *J* = 12.5 Hz, 2H, H-18b), 4.57-4.62 (m, 4H, H-13a, H-17a), 4.32 (t, *J* = 9.5 Hz, 2H, H-9), 4.23-4.29 (m, 4H, H-21a, H-21b), 4.03 (s, 6H, H-1), 3.68 (t, *J* = 11.1 Hz, 2H, H-13b), 3.27 (q, *J* = 10.0 Hz, 2H, H-17b), 2.59 (t, *J* = 11.7 Hz, 2H, H-10a), 2.54 (d, *J* = 12.2 Hz, 4H, H-24a), 2.44 (d, *J* = 12.2 Hz, 4H, H-24b), 1.96-2.01 (m, 8H, H-25a), 1.68 (br s, 2H, H-11), 1.57-1.65 (m, 6H, H-12, H-16a, H-16b), 1.40-1.49 (m, 44H, H-14a, H-14b, H-25b, H-26a, H-26b, H-27a, H-27b), 1.02-1.08 (m, 2H, H-10b), 0.77 (t, *J* = 7.4 Hz, 6H, H-15) ppm;

*obscured by the residual solvent signal but deduced from the ^{1}H-COSY.

^{13}C NMR (126 MHz, C_5D_5N) δ 197.4, 159.2, 148.4, 145.7, 144.5, 138.4, 135.0*, 134.5, 133.0, 132.5, 132.5 (q, $J_{C\text{-}F}$ = 33.4 Hz), 131.1, 130.8, 127.5, 124.2*, 124.1 (q, $J_{C\text{-}F}$ = 273.3 Hz), 121.9, 121.8, 104.1, 69.4, 67.0, 62.0, 59.3, 57.3, 57.2, 56.7, 53.6, 47.1, 37.6, 36.4, 31.7, 25.5, 25.0, 24.8, 24.4-24.5 (m), 22.0, 12.0 ppm;

*obscured by the residual solvent signal but deduced from the HSQC.

^{19}F NMR (376 MHz, C_5D_5N) δ – 62.9 (s) ppm;

IR (neat, cm^{-1}): ν_{max} 2924 (m), 2855 (w), 1623 (w), 1580 (s), 1512 (w), 1460 (m), 1398 (m), 1374 (m), 1279 (s), 1174 (s), 1132 (s), 1034 (s), 903 (m), 859 (w), 827 (w), 770 (w), 711 (m), 682 (m);

$[\boldsymbol{\alpha}]_{\mathbf{D}}^{\mathbf{25.0}}$ = + 15.6 (c. 0.21, C_5H_5N);

HRMS (+ESI) *m/z* For cation found 553.2278, $[C_{29}H_{31}F_6N_2O_2]^+$ requires 553.2284, (δ = − 1.1 ppm).

HRMS (−ESI) *m/z* For dianion found 580.1021, $[C_{50}H_{66}O_{14}Rh_2S_2]^{2-}$ requires 580.1008, (δ = + 2.2 ppm).

Bis[rhodium (1R,2S,4R)-2-((R)-hydroxy(6-methoxyquinolin-4-yl)methyl)-1-((3,3'',5,5''-tetra-tert-butyl-[1,1':3',1''-terphenyl]-5'-yl)methyl)quinuclidin-1-ium (3,5-bis((1-carboxycycloheptyl)methyl)phenyl)methanesulfonate)] (Rh_2(**D**)$_2$•(**10**)$_2$)

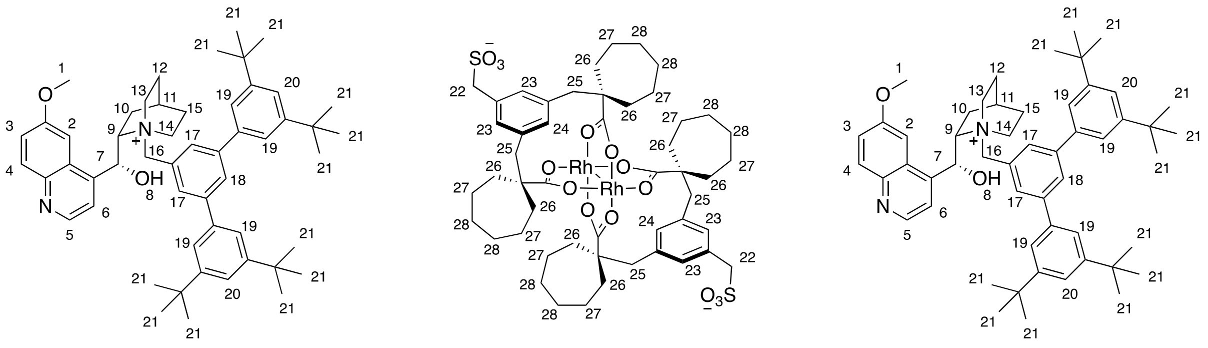

Prepared according to **GP3** on a 0.041 mmol scale with respect to Rh_2(**D**)$_2$•(**Bu$_4$N**)$_2$ and using (**10•Br**) as the chiral bromide salt. The title compound was obtained as a grey amorphous solid (77.8 mg, 0.029 mmol, 70%).

^{1}H NMR (500 MHz, C_5D_5N) δ 9.03 (d, J = 4.4 Hz, 2H, H-5), 8.45 (br s, 4H, H-17), 8.36 (d, J = 9.2 Hz, 2H, H-4), 8.23-8.24 (m, 4H, H-8, H-18), 7.94 (d, J = 1.5 Hz, 8H, H-19), 7.90 (d, J = 4.4 Hz, 2H, H-6), 7.74 (t, J = 1.5 Hz, 4H, H-20), 7.64 (d, J = 2.5 Hz, 2H, H-2), 7.51 (dd, J = 9.2 Hz, 2.6 Hz, 2H, H-3), 7.16 (br s, 4H, H-23), 7.09 (d, J = 5.3 Hz, 2H, H-7), 7.03 (s, 2H, H-24), 6.42 (d, J = 12.2 Hz, 2H, H-16a), 4.93 (d, J = 12.4 Hz, 2H, H-16b), 4.80 (t, J = 13.1 Hz, 2H, H-14a), 4.31 (s, 4H, H-22a, H-22b), 3.99-4.02 (m, 4H, H-9, H-13a), 3.82 (s, 6H, H-1), 3.51-3.56 (m, 2H, H-13b), 3.33-3.56 (m, 2H, H-14b), 2.45-2.53 (m, 10H, H-10a, H-25a, H-25b), 1.94-2.01 (m, 10H, H-26a, H-15a), 1.83 (br s, 2H, H-11), 1.60 (br s, 2H, H-12a), 1.39-1.51 (m, 116H, H-12b, H-15b, H-21, H-26b, H-27a, H-27b, H-28a, H-28b), 1.23 (t, J = 11.4 Hz, 2H, H-10b) ppm;

^{13}C NMR (126 MHz, C_5D_5N) δ 197.4, 158.9, 152.5, 148.5, 145.6, 145.4, 144.9, 141.1, 138.3, 134.3, 133.0, 132.7, 131.2, 130.7, 130.2, 129.4, 127.0, 123.1, 122.8, 121.7, 121.5, 103.2, 70.7, 64.9, 64.8, 59.2, 57.5, 56.2, 53.5, 51.7, 47.1, 37.6-37.7 (m), 35.7, 32.1, 31.7, 26.0, 24.6, 24.5, 24.3, 21.4 ppm;

IR (neat, cm^{-1}): ν_{max} 2951 (s), 2865 (m), 2361 (m), 2332 (m), 1621 (w), 1581 (s), 1508 (m), 1459 (s), 1394 (s), 1362 (s), 1241 (s), 1225 (s), 1174 (m), 1130 (w), 1065 (w), 1033 (s), 869 (s), 828 (m), 714 (s), 654 (m);

$[\boldsymbol{\alpha}]_{\mathbf{D}}^{\mathbf{25.0}}$ = − 55.0 (c. 0.15, $CHCl_3$);

HRMS (+ESI) *m/z* For cation found 765.5355, $[C_{53}H_{69}N_2O_2]^+$ requires 765.5354, (δ = + 0.1 ppm).

HRMS (−ESI) *m/z* For dianion found 580.1001, $[C_{50}H_{66}O_{14}Rh_2S_2]^{2-}$ requires 580.1008, (δ = + 1.2 ppm).

Synthesis of Starting Materials for Enantioselective Intermolecular Amination

2,2,3,3,4,4,4-Heptafluorobutyl sulfamate (**5**)

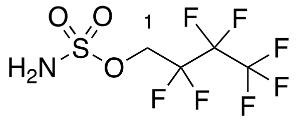

Prepared according to the protocol described by Du Bois, Sigman and co-workers.[26] To a stirred solution of chlorosulfonyl isocyanate (4.35 mL, 50.0 mmol, 2.20 equiv.) and MeCN (12 mL) at 0 °C was added dropwise formic acid (1.92 mL, 50.9 mmol, 2.24 equiv.). After 10 minutes, MeCN (20 mL) was added and the solution was allowed to warm to room temperature and stirred for a further hour. The reaction mixture was then cooled to 0 °C and a solution of 2,2,3,3,4,4,4-heptafluorobutan-1-ol (2.69 mL, 21.5 mmol, 1.00 equiv.) in *N*,*N*-dimethylacetamide (20 mL) was added dropwise. The reaction mixture was allowed to warm to room temperature and stirred for a further 2 hours. Water (20 mL) and EtOAc (40 mL) were then added and the layers separated. The aqueous layer was extracted with EtOAc and the combined organic layers were washed with water, aq. LiCl (10% w/v) and brine, dried ($MgSO_4$), filtered, and concentrated *in vacuo*. Purification by flash column chromatography (SiO_2, 20% v/v EtOAc in Hexane) afforded the title compound as a white gel (4.76 g, 17.0 mmol, 79%)

^{1}H NMR (400 MHz, $(CD_3)_2CO$) δ 7.24 (2H, br s, NH_2), 4.76 (2H, t, J = 13.6 Hz, H-1) ppm;

^{13}C NMR (126 MHz, $(CD_3)_2CO$) δ 118.5 (qt, $J_{C\text{-}F}$ = 287 Hz, 34.0 Hz), 114.5 (tt, $J_{C\text{-}F}$ = 257 Hz, 30.9 Hz), 112.8–106.2 (m), 64.5 (t, $J_{C\text{-}F}$ = 27.2 Hz) ppm;

^{19}F NMR (376 MHz, $(CD_3)_2CO$) δ − 82.8 (t, J = 9.5 Hz), − 122.2 - − 122.0 (m), − 129.1 - − 129.0 (m) ppm.

The spectroscopic data is in agreement with that reported in the literature.[26]

Ethyl 3-(4-hydroxybut-1-yn-1-yl)benzoate

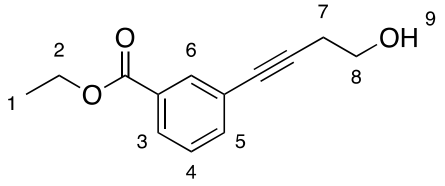

Prepared according **GP5** on a 10.0 mmol scale with respect to ethyl 3-iodobenzoate. Purification by flash column chromatography (SiO_2, 0-40% v/v EtOAc in hexane) afforded the title compound as an orange oil (2.00 g, 9.3 mmol, 92%).

***Rf* value** = 0.30 (30% v/v EtOAc in hexane);

^{1}H NMR (400 MHz, $CDCl_3$) δ 8.09 (br s, 1H, H-6), 7.96 (d, J = 7.8 Hz, 1H, H-3), 7.58 (d, J = 7.5 Hz, 1H, H-5), 7.37 (t, J = 7.8 Hz, 1H, H-4), 4.38 (q, J = 7.1 Hz, 2H, H-2), 3.83 (t, J = 6.2 Hz, 2H, H-8), 2.71 (t, J = 6.2 Hz, 2H, H-7), 1.64 (br s, 1H, H-9), 1.40 (t, J = 7.1 Hz, 3H, H-1) ppm;

^{13}C NMR (101 MHz, $CDCl_3$) δ 166.1, 135.9, 132.9, 130.8, 129.1, 128.5, 123.9, 87.5, 81.7, 61.4, 61.3, 24.0, 14.5 ppm;

IR (film, cm^{-1}): ν_{max} 3414 (br), 1717 (s), 1601 (w), 1579 (w), 1476 (w), 1430 (w), 1392 (w), 1368 (w), 1295 (s), 1226 (s), 1169 (w), 1105 (m), 1080 (w), 1046 (w), 1025 (m), 914 (w), 852 (w), 816 (w), 754 (m), 684 (w);

The NMR data is in agreement with that reported in the literature.[27]

Ethyl 3-(4-hydroxybutyl)benzoate (**6b**)

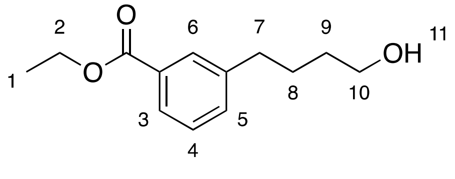

Prepared according to **GP7** on a 9.3 mmol scale with respect to ethyl 3-(4-hydroxybut-1-yn-1-yl)benzoate. Purification by flash column chromatography (SiO_2, 40% v/v EtOAc in hexane) afforded the title compound as a colourless oil (1.59 g, 7.2 mmol, 77%).

R

R_f value = 0.28 (40% v/v EtOAc in hexane);

^{1}H NMR (400 MHz, $CDCl_3$) δ 7.87 (br s, 2H, H-3, H-6), 7.32-7.38 (m, 2H, H-4, H-5), 4.37 (q, J = 7.1 Hz, 2H, H-2), 3.67 (t, J = 6.5 Hz, 2H, H-10), 2.70 (t, J = 7.4 Hz, 2H, H-7), 1.73 (p, J = 7.5 Hz, 2H, H-8), 1.57-1.64 (m, 2H, H-9), 1.43 (br s, 1H, H-11), 1.39 (t, J = 7.1 Hz, 3H, H-1) ppm;

^{13}C NMR (101 MHz, $CDCl_3$) δ 167.0, 142.7, 133.1, 130.7, 129.6, 128.4, 127.2, 62.9, 61.0, 35.6, 32.4, 27.6, 14.5 ppm;

IR (film, cm^{-1}): ν_{max} 3368 (br), 2935 (w), 2860 (w), 1715 (s), 1606 (w), 1587 (w), 1444 (m), 1392 (w), 1367 (m), 1275 (s) 1194 (s), 1106 (s), 1088 (m), 1058 (m), 1022 (m), 926 (w), 905 (w), 862 (w), 820 (w), 750 (s), 694 (m), 670 (w);

HRMS (+ESI) *m/z* found $[M+H]^+$ 223.1326, $[C_{13}H_{19}O_3]^+$ requires 223.1329, (δ = – 1.3 ppm).

4-(o-Tolyl)but-3-yn-1-ol

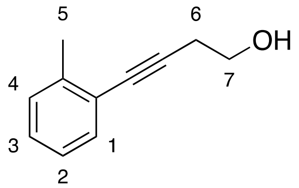

Prepared according to **GP5** on a 5.0 mmol scale with respect to 2-iodotoluene. Purification by flash column chromatography (SiO_2, 0-30% v/v EtOAc in petrol) afforded the title compound as a golden oil (303 mg, 1.9 mmol, 38%)

^{1}H NMR (400 MHz, $CDCl_3$) δ 7.39 (d, J = 7.5 Hz, 1H, H-1), 7.23–7.17 (m, 2H, H-3, H-4), 7.15–7.09 (m, 1H, H-2), 3.82 (t, J = 6.4 Hz, 2H, H-7), 2.73 (t, J = 6.4 Hz, 2H, H-6), 2.43 (s, 3H, H-5), 2.22 (br s, 1H, OH) ppm;

^{13}C **NMR** (101 MHz, $CDCl_3$) δ 140.1, 132.0, 129.4, 128.0, 125.6, 123.2, 90.4, 81.4, 61.4, 24.0, 20.8 ppm;

The spectroscopic data is in agreement with that reported in the literature.[28]

4-(o-Tolyl)butan-1-ol (**6c**)

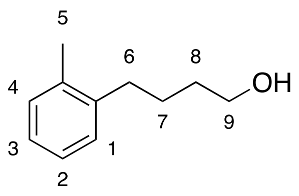

Prepared according to **GP7** on a 1.9 mmol scale with respect to 4-(*o*-tolyl)but-3-yn-1-ol. Purification by flash column chromatography (SiO_2, 0-35% v/v EtOAc in Hexane) afforded the title compound as a pale-yellow oil (247 mg, 1.5 mmol, 80%)

***R_f* value** = 0.60 (20% v/v acetone in $CHCl_3$);

^{1}H **NMR** (400 MHz, $CDCl_3$) δ 7.18–7.10 (m, 4H, H-1, H-2, H-3, H-4), 3.71–3.65 (m, 2H, H-9), 2.69–2.62 (m, 2H, H-6), 2.34 (s, 3H, H-5), 1.86 (s, 1H, OH), 1.73–1.62 (m, 4H, H-7, H-8) ppm;

^{13}C **NMR** (101 MHz, $CDCl_3$) δ 140.5, 135.9, 130.2, 128.8, 125.9*, 62.8, 33.0, 32.6, 26.4, 19.3 ppm;

* corresponds to two ^{13}C environments.

IR (film, cm^{-1}): ν_{max} 3321 (br, m), 2935 (m), 2863 (m), 1492 (m), 1459 (m), 1379 (m), 1157 (w), 1115 (w), 1058 (m), 1031 (m), 981 (m);

HRMS (+ESI) *m/z* found $[M+K]^+$ = 203.0837; $[C_{11}H_{16}KO]^+$ requires 203.0833, (δ = + 2.0 ppm).

4-(m-Tolyl)but-3-yn-1-ol

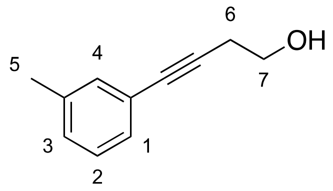

Prepared according to **GP5** on a 10.0 mmol scale with respect to 3-methyliodobenzene. Purification by flash column chromatography (SiO_2, 20% v/v EtOAc in Hexane) afforded the title compound as a dark red oil (381 mg, 2.4 mmol, 24%).

^{1}H NMR (400 MHz, $CDCl_3$) δ 7.25 – 7.16 (m, 3H, H-1, H-2, H-4), 7.11 (dtd, *J* = 7.2 Hz, 1.7 Hz, 0.8 Hz, 1H, H-3), 3.81 (q, *J* = 6.0 Hz, 2H, H-7), 2.69 (t, *J* = 6.2 Hz, 2H, H-6), 2.32 (s, 3H, H-5), 1.83 (t, *J* = 6.0 Hz, 1H, OH) ppm;

^{13}C NMR (101 MHz, $CDCl_3$) δ 137.9, 132.3, 128.9, 128.7, 128.2, 123.1, 86.0, 82.6, 61.2, 23.8, 21.2 ppm.

The spectroscopic data is in agreement with that reported in the literature.[29]

4-(m-Tolyl)butan-1-ol (**6d**)

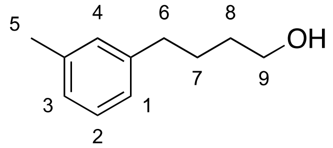

Prepared according to **GP7** on a 1.9 mmol scale with respect to 4-(*m*-tolyl)but-3-yn-1-ol. Purification by flash column chromatography (SiO_2, 0-10% v/v acetone in $CHCl_3$) afforded the title compound as a pale-yellow oil (136 mg, 0.83 mmol, 44%).

***R$_f$* value** = 0.39 (10% v/v acetone in $CHCl_3$);

^{1}H NMR (400 MHz, $CDCl_3$) δ 7.17 (t, *J* = 7.8 Hz, 1H, H-2), 7.04 – 6.92 (m, 3H, H-1, H-3, H-4), 3.66 (t, *J* = 6.3 Hz, 2H, H-9), 2.61 (t, *J* = 7.5 Hz, 2H, H-6), 2.33 (s, 3H, H-5), 1.73 – 1.57 (m, 4H, H-7, H-8), 1.35 (s, 1H, OH) ppm;

^{13}C NMR (101 MHz, $CDCl_3$) δ 142.3, 137.8, 129.2, 128.2, 126.5, 125.4, 62.9, 35.6, 32.4, 27.6, 21.4 ppm;

IR (film, cm^{-1}): ν_{max} 3364 (br), 3023 (w), 2936 (s), 2862 (m), 1609 (w), 1487 (w), 1457 (w), 1275 (w), 1259 (w), 1060 (m), 1036 (w), 910 (m), 781 (m), 734 (s), 699 (m), 447 (w), 435 (w), 426 (w);

HRMS *The compound did not ionise.*

4-(3-Fluorophenyl)but-3-yn-1-ol

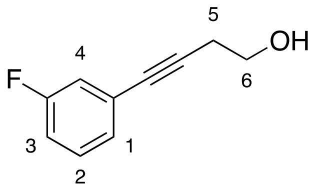

Prepared according to **GP5** on a 5.0 mmol scale with respect to 1-fluoro-3-iodobenzene. Purification by flash column chromatography (SiO_2, 0-30% v/v EtOAc in hexane) afforded the title compound as a golden oil (795 mg, 4.8 mmol, 97%)

***R$_f$* value** = 0.62 (20% v/v acetone in $CHCl_3$);

^{1}H NMR (400 MHz, $CDCl_3$) δ 7.28–7.22 (m, 1H, H-2), 7.18 (d, J = 7.7 Hz, 1H, H-1), 7.10 (d, J = 9.5 Hz, 1H, H-4), 7.00 (td, J = 8.5 Hz, 2.6 Hz, 1H, H-3), 3.82 (t, J = 6.3 Hz, 2H, H-6), 2.69 (t, J = 6.3 Hz, 2H, H-5), 1.83 (br s, 1H, OH) ppm;

^{13}C NMR (101 MHz, $CDCl_3$) δ 162.5 (d, $J_{C\text{-}F}$ = 246 Hz), 129.9 (d, $J_{C\text{-}F}$ = 8.7 Hz), 127.7 (d, $J_{C\text{-}F}$ = 3.1 Hz), 125.3 (d, $J_{C\text{-}F}$ = 9.5 Hz), 118.6 (d, $J_{C\text{-}F}$ = 22.7 Hz), 115.4 (d, $J_{C\text{-}F}$ = 21.2 Hz), 87.7, 81.4 (d, $J_{C\text{-}F}$ = 3.4 Hz), 61.2, 23.9 ppm;

^{19}F NMR (376 MHz, $CDCl_3$) δ – 114.1 (s) ppm;

IR (neat, cm^{-1}): ν_{max} 3356 (br, m), 2944 (w), 2886 (w), 2235 (w), 1687 (w), 1609 (m), 1579 (s), 1486 (m), 1431 (m), 1279 (m), 1263 (m), 1170 (m), 1150 (m), 1041 (s), 935 (m);

HRMS (+ESI) *m/z* found $[M+H]^+$ = 165.0707, $[C_{10}H_{10}FO]^+$ requires 165.0710, (δ = – 1.8 ppm).

4-(3-Fluorophenyl)butan-1-ol (**6e**)

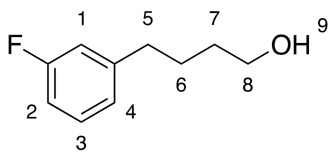

Prepared according to **GP7** on a 4.8 mmol scale with respect to 4-(3-fluorophenyl)but-3-yn-1-ol. Purification by flash column chromatography (SiO_2, 0-30% v/v EtOAc in petrol) afforded the title compound as a colourless oil (565 mg, 3.4 mmol, 70%).

***R_f* value** = 0.30 (30% v/v EtOAc in petrol);

^{1}H NMR (400 MHz, $CDCl_3$) δ 7.22 (q, J = 7.3 Hz, 1H, H-3), 6.95 (d, J = 7.6 Hz, 1H, H-4), 6.85-6.89 (m, 2H, H-1, H-2), 3.66 (t, J = 6.4 Hz, 2H, H-8), 2.64 (t, J = 7.5 Hz, 2H, H-5), 1.66-1.74 (m, 2H, H-6), 1.56-1.63 (m, 2H, H-7), 1.47 (s, 1H, H-9).

^{13}C NMR (101 MHz, $CDCl_3$) δ 163.1 (d, $J_{C\text{-}F}$ = 245.1 Hz), 145.0 (d, $J_{C\text{-}F}$ = 7.2 Hz), 129.8 (d, $J_{C\text{-}F}$ = 8.3 Hz), 124.2 (d, $J_{C\text{-}F}$ = 2.7 Hz), 115.3 (d, $J_{C\text{-}F}$ = 20.7 Hz), 112.7 (d, $J_{C\text{-}F}$ = 21.0 Hz), 62.8, 35.5 (d, $J_{C\text{-}F}$ = 1.6 Hz), 32.3, 27.4 ppm;

^{19}F NMR (376 MHz, $CDCl_3$) δ – 114.0 (s) ppm;

IR (film, cm^{-1}): ν_{max} 3329 (br), 2937 (m), 2862 (m), 1616 (m), 1589 (s), 1487 (s), 1448 (s);

HRMS (−ESI) *m/z* found $[M-H]^-$ 167.0877, $[C_{10}H_{12}FO]^-$ requires 167.0878, (δ = – 0.6 ppm).

4-(2-Fluorophenyl)but-3-yn-1-ol

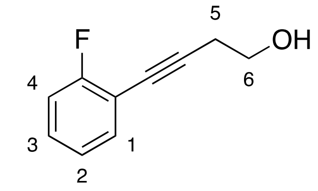

Prepared according to **GP5** on a 5.0 mmol scale with respect to 1-fluoro-2-iodobenzene. Purification by flash column chromatography (SiO_2, 0-30% v/v EtOAc in hexane) afforded the title compound as a golden oil (661 mg, 4.0 mmol, 81%)

***R_f* value** = 0.60 (20% v/v acetone in $CHCl_3$);

^{1}H NMR (400 MHz, $CDCl_3$) δ 7.40 (td, J = 7.8 Hz, 1.7 Hz, 1H, H-1), 7.31–7.23 (m, 1H, H-3), 7.11–7.01 (m, 2H, H-2, H-4), 3.83 (t, J = 6.2 Hz, 2H, H-6), 2.73 (t, J = 6.2 Hz, 2H, H-5), 1.97 (br s, 1H, OH) ppm;

^{13}C NMR (101 MHz, $CDCl_3$) δ 163.0 (d, J_{C-F} = 251 Hz), 133.7 (d, J_{C-F} = 1.5 Hz), 129.7 (d, J_{C-F} = 7.9 Hz), 124.0 (d, J_{C-F} = 3.7 Hz), 115.6 (d, J_{C-F} = 21.1 Hz), 112.0 (d, J_{C-F} = 15.7 Hz), 92.1 (d, J_{C-F} = 3.3 Hz), 76.0 (d, J_{C-F} = 1.1 Hz), 61.2, 24.2 ppm;

^{19}F NMR (376 MHz, $CDCl_3$) δ – 111.8 (s) ppm;

IR (film) ν_{max}/cm^{-1} 3363 (br, m), 2946 (w), 2887 (w), 2239 (w), 1685 (w), 1610 (w), 1573 (w), 1491 (s), 1449 (m), 1254 (m), 1214 (m), 1104 (m), 1040 (s), 943 (m);

HRMS (+ESI) *m/z* found $[M+H]^+$ = 165.0703; $[C_{10}H_{10}FO]^+$ requires 165.0710, (δ = – 4.2 ppm).

4-(2-Fluorophenyl)butan-1-ol (**6f**)

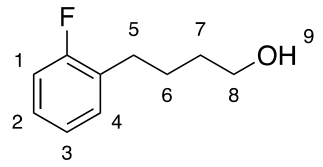

Prepared according to **GP7** on a 3.9 mmol scale with respect to 4-(2-fluorophenyl)but-3-yn-1-ol. Purification by flash column chromatography (SiO_2, 0-30% v/v EtOAc in hexane) afforded the title compound as a colourless oil (490 mg, 2.9 mmol, 75%).

***R_f* value** = 0.37 (30% v/v EtOAc in hexane);

^{1}H NMR (400 MHz, $CDCl_3$) δ 7.13-7.20 (m, 2H, H-2, H-4), 7.05 (t, J = 7.4 Hz, 1H, H-3), 7.00 (t, J = 9.2 Hz, 1H, H-1), 3.66 (t, J = 6.3 Hz, 2H, H-8), 2.68 (t, J = 7.3 Hz, 2H, H-5), 1.58-1.74 (m, 4H, H-6, H-7), 1.54 (s, 1H, H-9) ppm;

^{13}C NMR (101 MHz, $CDCl_3$) δ 161.3 (d, $J_{C\text{-}F}$ = 244.4 Hz), 130.7 (d, $J_{C\text{-}F}$ = 5.2 Hz), 129.2 (d, $J_{C\text{-}F}$ = 16.0 Hz), 127.6 (d, $J_{C\text{-}F}$ = 8.1 Hz), 124.0 (d, $J_{C\text{-}F}$ = 3.6 Hz), 115.3 (d, $J_{C\text{-}F}$ = 22.3 Hz), 62.8, 32.4, 28.8 (d, $J_{C\text{-}F}$ = 2.3 Hz), 26.4 ppm;

^{19}F NMR (376 MHz, $CDCl_3$) δ – 120.0 (s) ppm;

IR (film, cm^{-1}): ν_{max} 3327 (br), 2936 (m), 2864 (m), 1584 (w), 1491 (s), 1454 (m), 1227 (s), 1182 (w), 1227 (s), 1182 (w), 1109 (w), 1056 (m), 1030 (m), 982 (w), 937 (w), 845 (w), 751 (s), 723 (w);

HRMS *The compound did not ionise.*

4-(4-Fluorophenyl)but-3-yn-1-ol

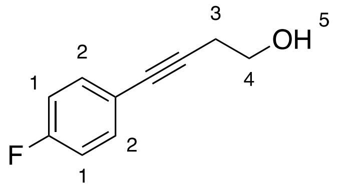

Prepared according **GP5** on a 5.0 mmol scale with respect to 4-fluoroiodobenzene. Purification by flash column chromatography (SiO_2, 10-35% v/v EtOAc in petrol) afforded the title compound as a brown oil (795 mg, 4.8 mmol, 97%).

***R_f* value** = 0.29 (30% v/v EtOAc in hexane);

^{1}H NMR (400 MHz, $CDCl_3$) δ 7.36-7.40 (m, 2H, H-2), 6.98 (tt, J = 8.7 Hz, 2.0 Hz, 2H, H-1), 3.81 (t, J = 6.2 Hz, 2H, H-4), 2.67 (t, J = 6.2 Hz, 2H, H-3). 1.83 (s, 1H, H-5) ppm;

^{13}**C NMR** (101 MHz, $CDCl_3$) δ 162.4 (d, J_{C-F} = 248.8 Hz), 133.6 (d, J_{C-F} = 8.3 Hz), 119.5 (d, J_{C-F} = 3.5 Hz), 115.6 (d, J_{C-F} = 22.0 Hz), 86.2 (d, J_{C-F} = 1.4 Hz), 81.5, 61.3, 23.9 ppm;

^{19}**F NMR** (376 MHz, $CDCl_3$) δ – 111.6 (s) ppm;

IR (film, cm^{-1}): ν_{max} 3336 (br), 2887 (w), 1673 (w), 1600 (m), 1505 (s), 1409 (w), 1220 (s), 1155 (m), 1092 (w), 1043 (s), 1014 (w), 834 (s), 810 (m);

The NMR data is in agreement with that reported in the literature.[30]

4-(4-Fluorophenyl)butan-1-ol (**6g**)

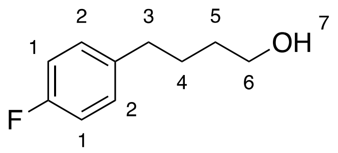

Prepared according to **GP7** on a 4.6 mmol scale with respect to 4-(4-fluorophenyl)but-3-yn-1-ol. Purification by flash column chromatography (SiO_2, 20-50% v/v EtOAc in petrol) afforded the title compound as a pale-yellow oil (515 mg, 3.0 mmol, 67%).

***R_f* value** = 0.31 (30% v/v EtOAc in hexane);

^{1}**H NMR** (400 MHz, $CDCl_3$) δ 7.11-7.14 (m, 2H, H-2), 6.96 (t, *J* = 8.5 Hz, 2H, H-1), 3.66 (t, *J* = 6.2 Hz, 2H, H-6), 2.62 (t, *J* = 7.4 Hz, 2H, H-3), 1.56-1.72 (m, 4H, H-4, H-5), 1.34 (s, 1H, H-7) ppm;

^{13}**C NMR** (101 MHz, $CDCl_3$) δ 161.3 (d, J_{C-F} = 243.1 Hz), 138.0 (d, J_{C-F} = 3.2 Hz), 129.8 (d, J_{C-F} = 7.7 Hz), 115.1 (d, J_{C-F} = 21.0 Hz), 62.8, 34.9, 32.3, 27.8 (d, J_{C-F} = 1.0 Hz) ppm;

^{19}**F NMR** (376 MHz, $CDCl_3$) δ – 118.9 (s) ppm;

IR (film, cm^{-1}): ν_{max} 3339 (br), 2935 (m), 2863 (m), 1600 (m), 1508 (s), 1459 (w), 1415 (w), 1218 (s), 1157 (m), 1099 (w), 1056 (m), 1031 (w), 983 (w), 934 (w), 831 (s), 758 (w), 701 (w);

The NMR data is in agreement with that reported in the literature.[31]

1-(But-3-en-1-yl)-3-chlorobenzene

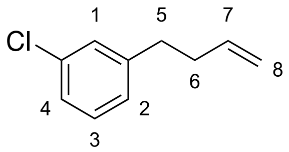

Prepared according to **GP8** on a 20.0 mmol scale with respect to 3-chlorobenzyl bromide. Crude title compound obtained as a straw-coloured oil and taken through with no further purification.

4-(3-Chlorophenyl)butan-1-ol (**6h**)

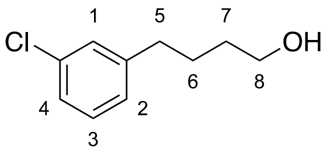

Prepared according to **GP10** on an 18.9 mmol scale with respect to 1-(but-3-en-1-yl)-3-chlorobenzene. Purification by flash column chromatography (SiO_2, 10-20% v/v EtOAc in hexane) afforded the title compound as a pale-yellow oil (1.02 g, 5.5 mmol, 37% over two steps).

***R_f* value** = 0.13 (20% v/v EtOAc in hexane);

^{1}H NMR (400 MHz, $CDCl_3$) δ 7.23 – 7.12 (m, 3H, H-1, H-3, H-4), 7.06 (d, J = 7.2 Hz, 1H, H-2), 3.66 (t, J = 6.4 Hz, 2H, H-8), 2.62 (t, J = 7.5 Hz, 2H, H-5), 1.76 – 1.54 (m, 4H, H-6, H-7), 1.51 (s, 1H, OH) ppm;

^{13}C NMR (101 MHz, $CDCl_3$) δ 144.3, 134.0, 129.5, 128.5, 126.6, 126.0, 62.7, 35.3, 32.2, 27.3 ppm;

IR (film, cm^{-1}): ν_{max} 3318 (br), 2936 (s), 2861 (m), 1596 (m), 1573 (m), 1475 (m), 1429 (m), 1207 (w), 1078 (s), 1059 (s), 1034 (m), 999 (w), 985 (w), 863 (m), 779 (s), 695 (s), 684 (s), 441 (w);

HRMS *The compound did not ionise.*

1-Bromo-3-(but-3-en-1-yl)benzene

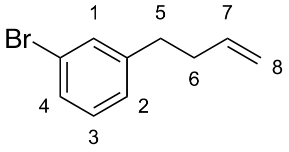

Prepared according to **GP8** on a 20.0 mmol scale with respect to 3-bromobenzyl bromide. Crude title compound obtained as a colourless oil and taken through with no further purification.

4-(3-Bromophenyl)butan-1-ol (**6i**)

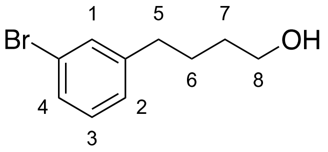

Prepared according to **GP10** on an 18.9 mmol scale with respect to 1-bromo-3-(but-3-en-1-yl)benzene. Purification by flash column chromatography (SiO_2, 10-20% v/v EtOAc in Hexane) afforded the title compound as a straw-coloured oil (980 mg, 4.3 mmol, 29% over two steps).

^{1}H NMR (400 MHz, $CDCl_3$) δ 7.37 – 7.28 (m, 2H, H-1, H-4), 7.17 – 7.07 (m, 2H, H-2, H-3), 3.65 (t, J = 6.4 Hz, 2H, H-8), 2.61 (t, J = 7.5 Hz, 2H, H-5), 1.74 – 1.54 (m, 4H, H-6, H-7), 1.51 (br s, 1H, OH) ppm;

^{13}C NMR (101 MHz, $CDCl_3$) δ 144.7, 131.4, 129.9, 128.9, 127.1, 122.4, 62.6, 35.3, 32.2, 27.4 ppm.

The spectroscopic data is in agreement with that reported in the literature.[12]

(3-Iodophenyl)methanol

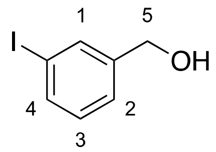

Prepared according to **GP11** on an 8.0 mmol scale with respect to 3-iodobenzoic acid. Purification by flash column chromatography (SiO_2, 10% v/v EtOAc in Hexane) afforded the title compound as a colourless oil (1.59 g, 6.8 mmol, 83%).

^{1}H NMR (400 MHz, $CDCl_3$) δ 7.72 (s, 1H, H-1), 7.61 (d, J = 7.8 Hz, 1H, H-4), 7.31 (d, J = 7.6 Hz, 1H, H-2), 7.09 (t, J = 7.8 Hz, 1H, H-3), 4.63 (s, 2H, H-5), 1.75 (s, 1H, OH) ppm;

^{13}C NMR (101 MHz, $CDCl_3$) δ 143.1, 136.6, 135.9, 130.3, 126.0, 94.5, 64.4 ppm.

The spectroscopic data is in agreement with that reported in the literature.[32]

1-(Bromomethyl)-3-iodobenzene

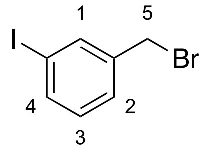

Prepared according to **GP12** on a 6.0 mmol scale with respect to (3-iodophenyl)methanol. Crude title compound obtained as an off-white amorphous solid and taken through with no further purification.

1-(But-3-en-1-yl)-3-iodobenzene

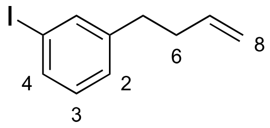

Prepared according to **GP8** on a 3.4 mmol scale with respect to 1-(bromomethyl)-3-iodobenzene. Crude title compound obtained as a pale-yellow oil and taken through with no further purification.

4-(3-Iodophenyl)butan-1-ol (**6j**)

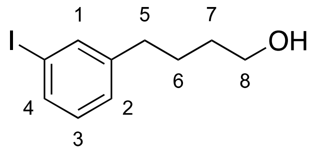

Prepared according to **GP10** on a 2.7 mmol scale with respect to 1-(but-3-en-1-yl)-3-iodobenzene. Purification by flash column chromatography (SiO_2, 10-40% v/v EtOAc in hexane) afforded the title compound as a pale-yellow oil (136 mg, 0.5 mmol, 11% over three steps).

***R_f* value** = 0.11 (20% v/v EtOAc in hexane);

^{1}H NMR (500 MHz, $CDCl_3$) *δ* 7.55 (t, *J* = 1.8 Hz, 1H, H-1), 7.52 (dt, *J* = 7.8, 1.5 Hz, 1H, H-4), 7.16 – 7.13 (m, 1H, H-2), 7.01 (t, *J* = 7.7 Hz, 1H, H-3), 3.66 (t, *J* = 6.4 Hz, 2H, H-8), 2.59 (t, *J* = 7.5 Hz, 2H, H-5), 1.73 – 1.55 (m, 4H, H-6, H-7), 1.40 (br s, 1H, OH) ppm;

^{13}C NMR (126 MHz, $CDCl_3$) *δ* 144.8, 137.4, 134.9, 130.1, 127.7, 94.5, 62.7, 35.2, 32.2, 27.4 ppm;

IR (film, cm^{-1}): ν_{max} 3353 (br, w), 2961 (w), 2963 (w), 2859 (w), 1562 (w), 1469 (w), 1418 (w), 1260 (s), 1031 (br, s), 862 (w), 790 (s), 692 (w), 658 (w);

HRMS *The compound did not ionise.*

3-Iodophenyl acetate

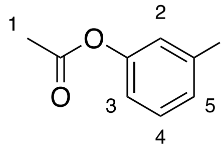

The protocol described by Ferreira and Costello was employed.[33] To a solution of 3-iodophenol (2.00 g, 9.1 mmol) in CH_2Cl_2 (18 mL) at 0 °C were sequentially added 4-dimethylamino pyridine (55.5 mg, 0.45 mmol), Et_3N (1.29 mL, 9.3 mmol), and acetic anhydride (1.03 mL, 10.9 mmol). The resulting mixture was allowed to warm to room temperature and stirred overnight. Following this, water (20 mL) was added and the layers were separated. The aqueous phase was extracted with CH_2Cl_2 (2 x 20 mL), and the combined organic fractions were washed with brine (20 mL), dried ($MgSO_4$) and the solvent removed under reduced pressure. Purification by flash column chromatography (SiO_2, 10% v/v EtOAc in petrol) afforded the title compound as a colourless oil (2.21 g, 8.4 mmol, 93%).

***R_f* value** = 0.47 (10% v/v EtOAc in hexane);

^{1}H NMR (400 MHz, $CDCl_3$) *δ* 7.56 (d, *J* = 7.0 Hz, 1H, H-5), 7.47 (br s, 1H, H-2), 7.06-7.12 (m, 2H, H-3, H-4), 2.28 (s, 3H, H-1) ppm;

^{13}C NMR (101 MHz, $CDCl_3$) *δ* 169.1, 151.0, 135.1, 130.9, 130.8, 121.3, 93.6, 21.1 ppm;

IR (film, cm^{-1}): ν_{max} 3062 (w), 1762 (s), 1577 (m), 1466 (m), 1419 (w), 1368 (s), 1181 (s), 1044 (m), 1009 (m), 997 (m), 921 (s), 893 (m), 868 (m), 827 (m), 772 (m), 753 (m), 696 (m), 668 (m), 647 (w);

The spectroscopic data is in agreement with that reported in the literature.[34]

3-(4-Hydroxybut-1-yn-1-yl)phenyl acetate

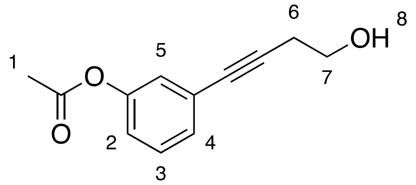

Prepared according **GP6** on a 3.6 mmol scale with respect to 3-iodophenyl acetate. Purification by flash column chromatography (SiO_2, 0-34% v/v EtOAc in petrol) afforded the title compound as a golden oil (683 mg, 3.3 mmol, 93%).

***R**$_f$* **value** = 0.25 (30% v/v EtOAc in hexane);

^{1}H NMR (400 MHz, $CDCl_3$) *δ* 7.25-7.30 (m, 2H, H-3, H-4), 7.14-7.15 (m, 1H, H-5), 7.02 (dt, *J* = 7.0 Hz, 2.3 Hz, 1H, H-2), 3.79 (q, *J* = 6.3 Hz, 2H, H-7), 2.67 (t, *J* = 6.3 Hz, 2H, H-6), 2.28 (s, 3H, H-1), 1.92 (t, *J* = 6.3 Hz, 1H, H-8) ppm;

^{13}C NMR (101 MHz, $CDCl_3$) *δ* 169.4, 150.5, 129.4, 129.3, 124.9, 124.8, 121.6, 87.5, 81.6, 61.2, 23.9, 21.2 ppm;

IR (film, cm^{-1}): ν_{max} 3408 (br), 2941 (w), 2884 (w), 1763 (s), 1739 (s), 1603 (m), 1577 (m), 1483 (m), 1426 (m), 1368 (m), 1197 (s), 1151 (s), 1042 (s), 1015 (s), 927 (m), 894 (m), 784 (m), 734 (w), 684 (s);

HRMS (+ESI) *m/z* found $[M+H]^+$ 205.0861, $[C_{12}H_{13}O_3]^+$ requires 205.0859, (*δ* = + 1.0 ppm).

3-(4-Hydroxybutyl)phenyl acetate (**6k**)

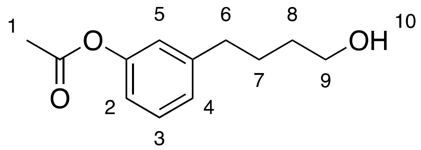

Prepared according to **GP7** on a 3.6 mmol scale with respect to 3-(4-hydroxybut-1-yn-1-yl)phenyl acetate. Purification by flash column chromatography (SiO_2, 0-25% v/v acetone in $CHCl_3$) afforded the title compound as a colourless oil (345 mg, 1.7 mmol, 46%).

***R**$_f$* **value** = 0.50 (20% v/v acetone in $CHCl_3$);

^{1}H NMR (400 MHz, $CDCl_3$) *δ* 7.28 (t, *J* = 7.6 Hz, 1H, H-3), 7.05 (d, *J* = 7.6 Hz, 1H, H-4), 6.90-6.91 (m, 2H, H-2, H-5), 3.65 (t, *J* = 6.4 Hz, 2H, H-9), 2.65 (t, *J* = 7.5 Hz, 2H, H-6), 2.29 (s, 3H, H-1), 1.67-1.74 (m, 2H, H-7), 1.57-1.64 (m, 2H, H-8), 1.37 (br s, 1H, H-10) ppm;

^{13}C NMR (101 MHz, $CDCl_3$) *δ* 169.7, 150.8, 144.2, 129.3, 126.1, 121.6, 119.0, 62.8, 35.5, 32.3, 27.3, 21.2 ppm;

IR (neat, cm^{-1}): ν_{max} 3381 (br), 2936 (m), 2861 (m), 1763 (s), 1739 (m), 1611 (w), 1587 (w), 1445 (m), 1369 (s), 1198 (s), 1141 (s), 1055 (m), 1014 (m), 937 (m), 910 (m), 881 (m), 788 (m), 754 (w), 693 (m), 669 (m);

HRMS (+ESI) *m/z* found $[M+Na]^+$ 231.0995, $[C_{12}H_{16}NaO_3]^+$ requires 231.0992, (δ = + 1.3 ppm).

4-(3-Isopropylphenyl)but-3-yn-1-ol

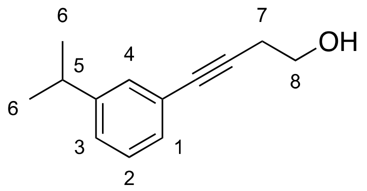

A solution of 1-bromo-3-isopropylbenzene (0.77 g, 3.9 mmol, 1.0 equiv.), $PdCl_2(PPh_3)_2$ (133 mg, 0.19 mmol, 5 mol %), CuI (74.0 mg, 0.39 mmol, 10 mol %), PPh_3 (102 mg, 0.39 mmol, 10 mol %) and *N*,*N*-dimethylformamide (10 mL) in a two-neck flask fitted with a reflux condenser was stirred at room temperature for 10 minutes. $^i Pr_2NH$ (5.0 mL) and but-3-yn-1-ol (0.58 mL, 7.7 mmol, 2.0 equiv.) were then added and the reaction mixture was refluxed for 16 h. The reaction mixture was then allowed to cool to room temperature and EtOAc (20 mL) was added. The reaction mixture was filtered through Celite® and the filtrate was washed with aq. LiCl (10% w/v) and brine, dried ($MgSO_4$), filtered and concentrated *in vacuo*. Purification by flash column chromatography (SiO_2, 10% v/v EtOAc in hexane) afforded the title compound as a golden oil (631 mg, 3.4 mmol, 86%).

***R$_f$* value** = 0.19 (20% v/v EtOAc in hexane);

^{1}H NMR (400 MHz, $CDCl_3$) δ 7.30 (s, 1H, H-4), 7.27 – 7.18 (m, 2H, H-1, H-2), 7.16 (dd, *J* = 6.8, 2.2 Hz, 1H, H-3), 3.82 (td, *J* = 6.3 Hz, 2.1 Hz, 2H, H-8), 2.87 (hept, *J* = 7.0 Hz, 2.0 Hz, 1H, H-5), 2.70 (td, *J* = 6.3 Hz, 2.0 Hz, 2H, H-7), 1.89 (br s, 1H, OH), 1.24 (dd, *J* = 7.0 Hz, 2.1 Hz, 6H, H-6) ppm;

^{13}C NMR (101 MHz, $CDCl_3$) δ 148.9, 129.7, 129.1, 128.3, 126.4, 123.1, 85.8, 82.9, 61.2, 34.0, 23.9* ppm;

*corresponds to two ^{13}C environments

IR (film, cm^{-1}): ν_{max} 3343 (br, m), 2960 (s), 2875 (m), 1600 (m), 1578 (w), 1482 (m), 1462 (m), 1425 (m), 1383 (w), 1364 (w), 1332 (w), 1191 (w), 1047 (s), 1019 (m), 925 (w), 891 (w), 847 (w), 794 (s), 699 (s), 475 (w);

HRMS (+ESI) *m/z* found [M+H]$^+$ 189.1281, $[C_{13}H_{17}O]^+$ requires 189.1274, (δ = + 3.7 ppm).

4-(3-Isopropylphenyl)butan-1-ol (**6l**)

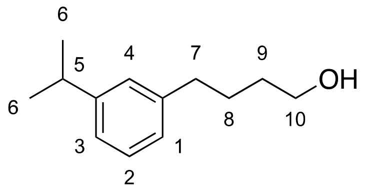

Prepared according to **GP7** on a 1.6 mmol scale with respect to 4-(3-isopropylphenyl)but-3-yn-1-ol. Purification by flash column chromatography (SiO_2, 10% v/v EtOAc in hexane) afforded the title compound as a clear oil (75.0 mg, 0.4 mmol, 26%).

^{1}H NMR (500 MHz, $CDCl_3$) δ 7.21 (td, *J* = 7.5 Hz, 0.8 Hz, 1H, H-2), 7.07 – 7.03 (m, 2H, H-3, H-4), 7.01 (dt, *J* = 7.4 Hz, 1.3 Hz, 1H, H-1), 3.67 (t, *J* = 6.4 Hz, 2H, H-10), 2.88 (hept, *J* = 6.9 Hz, 1H, H-5), 2.63 (t, *J* = 7.6 Hz, 2H, H-7), 1.75 – 1.59 (m, 4H, H-8, H-9), 1.50 (br s, 1H, OH), 1.25 (d, *J* = 6.9 Hz, 6H, H-6) ppm;

^{13}C NMR (126 MHz, $CDCl_3$) δ 148.9, 142.3, 128.2, 126.6, 125.8, 123.8, 62.9, 35.8, 34.1, 32.5, 27.6, 24.0 ppm;

IR (film, cm^{-1}): ν_{max} 3345 (br, m), 3022 (w), 2959 (s), 2934 (s), 2865 (m), 1605 (w), 1487 (w), 1461 (w), 1383 (w), 1363 (w), 1057 (m), 790 (w);

1-(But-3-en-1-yl)-2-chlorobenzene

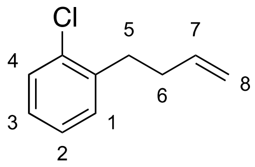

Prepared according to **GP8** on a 10.0 mmol scale with respect to 2-chlorobenzyl bromide. Crude title compound obtained as a pale-yellow oil and taken through with no further purification.

4-(2-Chlorophenyl)butan-1-ol (**6m**)

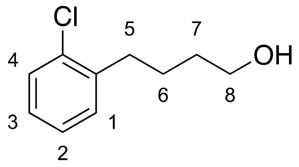

Prepared according to **GP10** on a 9.2 mmol scale with respect to 1-(but-3-en-1-yl)-2-chlorobenzene. Purification by flash column chromatography (SiO_2, 0-30% v/v EtOAc in hexane) afforded the title compound as a colourless oil (579 mg, 3.1 mmol, 41% over two steps).

^{1}H NMR (400 MHz, $CDCl_3$) *δ* 7.33 (dd, *J* = 7.7 Hz, 1.5 Hz, 1H, H-4), 7.24 – 7.10 (m, 3H, H-1, H-2, H-3), 3.69 (t, *J* = 6.2 Hz, 2H, H-8), 2.80 – 2.73 (m, 2H, H-5), 1.77 – 1.59 (m, 4H, H-6, H-7), 1.38 (s, 1H, OH) ppm;

^{13}C NMR (101 MHz, $CDCl_3$) *δ* 139.8, 133.9, 130.3, 129.5, 127.3, 126.7, 62.8, 33.3, 32.4, 25.9 ppm.

The spectroscopic data is in agreement with that reported in the literature.[35]

1-(But-3-en-1-yl)-4-chlorobenzene

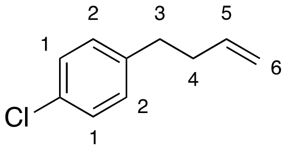

Prepared according to **GP8** on a 20.0 mmol scale with respect to 4-chlorobenzylbromide. Crude title compound obtained as a yellow oil and taken through with no further purification.

4-(4-Chlorophenyl)butan-1-ol (**6o**)

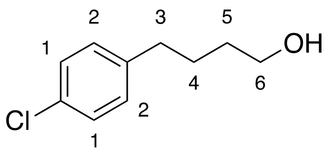

Prepared according to **GP10** on an 18.9 mmol scale with respect to 1-(but-3-en-1-yl)-4-chlorobenzene. Purification by flash column chromatography (SiO_2, 0-30% v/v EtOAc in hexane) afforded the title compound as a pale-yellow oil (956 mg, 5.2 mmol, 35% over two steps).

***R_f* value** = 0.27 (30% v/v EtOAc in Hexane);

^{1}H NMR (400 MHz, $CDCl_3$) δ 7.24 (d, *J* = 8.4 Hz, 2H, H-2), 7.11 (d, *J* = 8.3 Hz, 2H, H-1), 3.66 (t, *J* = 6.3 Hz, 2H, H-6), 2.62 (t, *J* = 7.5 Hz, 2H, H-3), 1.73 – 1.55 (m, 4H, H-4, H-5), 1.47 (s, 1H, OH) ppm;

^{13}C NMR (101 MHz, $CDCl_3$) δ 140.7, 131.5, 129.7, 128.4, 62.7, 35.0, 32.2, 27.5 ppm.

IR (film, cm^{-1}): ν_{max} 3340 (br, m), 2938 (s), 2869 (m), 1977 (w), 1562 (w), 1457 (m), 1437 (m), 1380 (w), 1062 (m), 997 (w), 773 (m), 713 (w), 578 (w), 460 (w);

HRMS *The compound did not ionise*

(5-Chloro-2-methylphenyl)methanol

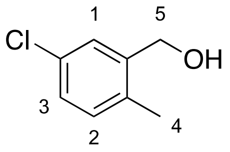

Prepared according to **GP11** on a 15.0 mmol scale with respect to 5-chloro-2-methylbenzoic acid. Purification by flash column chromatography (SiO_2, 0-5% v/v acetone in $CHCl_3$) afforded the title compound as an off-white solid (1.69 g, 10.8 mmol, 72%).

***R_f* value** = 0.22 (20% v/v EtOAc in Hexane);

^{1}H NMR (400 MHz, $CDCl_3$) δ 7.39 (s, 1H, H-1), 7.18 (d, *J* = 8.2 Hz, 1H, H-3), 7.10 (d, *J* = 8.0 Hz, 1H, H-2), 4.66 (s, 2H, H-5), 2.29 (s, 3H, H-4), 1.99 (s, 1H, OH) ppm;

^{13}C NMR (101 MHz, $CDCl_3$) δ 140.5, 134.0, 131.7, 131.5, 127.4, 127.1, 62.7, 18.0 ppm;

IR (film, cm^{-1}): ν_{max}/cm^{-1} 3307 (br, m), 2922 (w), 1485 (m), 1446 (m), 1404 (m), 1380 (w), 1210 (w), 1178 (m), 1128 (w), 1094 (m), 1035 (s), 1008 (m), 874 (s), 809 (s), 752 (w), 721 (w), 693 (w), 648 (m), 626 (w), 564 (w), 532 (m), 489 (w), 455 (w), 435 (w);

The ^{1}H NMR data is in agreement with that reported in the literature.[36]

2-(Bromomethyl)-4-chloro-1-methylbenzene

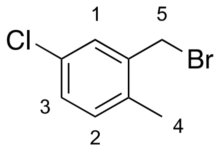

Prepared according to **GP12** on a 10.8 mmol scale with respect to (5-chloro-2-methylphenyl)methanol. Crude title compound obtained as a straw-coloured oil and taken through with no further purification.

2-(But-3-en-1-yl)-4-chloro-1-methylbenzene

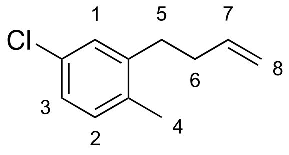

Prepared according to **GP8** on a 7.3 mmol scale with respect to 2-(bromomethyl)-4-chloro-1-methylbenzene. Crude title compound obtained as a straw-coloured oil and taken through with no further purification.

4-(5-Chloro-2-methylphenyl)butan-1-ol (**6p**)

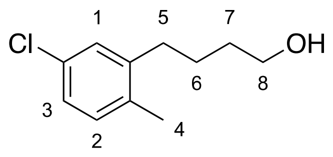

Prepared according to **GP10** on a 5.9 mmol scale with respect to 2-(but-3-en-1-yl)-4-chloro-1-methylbenzene. Purification by flash column chromatography (SiO_2, 10-30% v/v EtOAc in hexane) afforded the title compound as a straw-coloured oil (545 mg, 2.7 mmol, 34% over three steps).

***R_f* value** = 0.12 (20% v/v EtOAc in hexane);

^{1}H NMR (400 MHz, $CDCl_3$) δ 7.13 – 7.09 (m, 1H, H-1), 7.09 – 7.05 (m, 2H, H-2, H-3), 3.71 – 3.68 (m, 2H, H-8), 2.63 – 2.59 (m, 2H, H-5), 2.28 (s, 3H, H-4), 1.68 – 1.65 (m, 4H, H-6, H-7), 1.62 (br s, 1H, OH) ppm;

^{13}C NMR (101 MHz, $CDCl_3$) δ 142.4, 134.3, 131.4, 131.3, 128.6, 125.8, 62.7, 32.8, 32.5, 26.1, 18.8 ppm;

IR (film, cm^{-1}): ν_{max} 3329 (br, m), 2935 (m), 2865 (m), 1597 (w), 1570 (w), 1484 (m), 1458 (w), 1398 (w), 1380 (w), 1180 (w), 1124 (w), 1098 (w), 1055 (s), 1032 (m), 993 (w), 938 (w), 875 (m), 807 (s), 733 (w), 715 (w), 692 (w), 648 (m), 608 (w), 553 (w), 495 (w), 455 (w), 440 (w);

HRMS *The compound did not ionise.*

4-(2,3-Dimethylphenyl)but-3-yn-1-ol

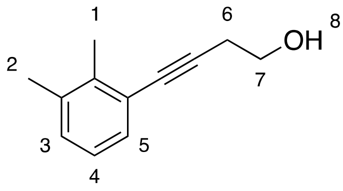

Prepared according **GP6** on a 5.0 mmol scale with respect to 1-iodo-2,3-dimethylbenzene. Purification by flash column chromatography (SiO_2, 0-34% v/v EtOAc in petrol) afforded the title compound as a golden oil (950 mg, quant.)

R

R_f value = 0.45 (30% v/v EtOAc in hexane);

^{1}H NMR (400 MHz, $CDCl_3$) δ 7.26 (d, J = 7.4 Hz, 1H, H-5), 7.08 (d, J = 7.2 Hz, 1H, H-3), 7.02 (t, J = 7.6 Hz, 1H, H-4), 3.83 (t, J = 6.3 Hz, 2H, H-7), 2.74 (t, J = 6.3 Hz, 2H, H-6), 2.38 (s, 3H, H-1), 2.27 (s, 3H, H-2), 1.92 (s, 1H, H-8) ppm;

^{13}C NMR (101 MHz, $CDCl_3$) δ 138.5, 136.8, 130.0, 129.7, 125.4, 123.3, 89.6, 82.1, 61.4, 24.1, 20.5, 17.6 ppm;

IR (neat, cm^{-1}): ν_{max} 3327 (br), 2940 (m), 2915 (m), 2884 (m), 2361 (w), 2334 (w), 1678 (w), 1580 (w), 1468 (m), 1457 (m), 1383 (m), 1335 (w), 1285 (w), 1249 (w), 1171 (w), 1039 (s), 939 (w), 827 (w), 770 (s), 713 (s);

HRMS (+ESI) *m/z* found $[M+H]^+$ 175.1118, $[C_{12}H_{15}O]^+$ requires 175.1117, (δ = + 0.6 ppm).

4-(2,3-Dimethylphenyl)butan-1-ol (**6q**)

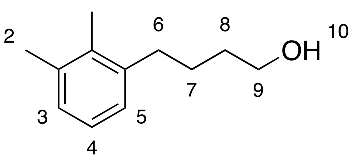

Prepared according to **GP7** on a 5.3 mmol scale with respect to 4-(2,3-dimethylphenyl)but-3-yn-1-ol with some procedural modifications: 12 mol % of Pd/C (10 wt % loading Pd) was used

and the reaction mixture was stirred at 60 °C for 48 hours. Purification by flash column chromatography (5-20% v/v EtOAc in hexane) afforded the title compound as a colourless oil (615 mg, 3.4 mmol, 65%).

***R_f* value** = 0.38 (30% v/v EtOAc in hexane);

^{1}H NMR (400 MHz, $CDCl_3$) δ 6.99-7.06 (m, 3H, H-3, H-4, H-5), 3.67-3.70 (m, 2H, H-9), 2.66-2.69 (m, 2H, H-6), 2.30 (s, 3H, H-2), 2.22 (s, 3H, H-1), 1.60-1.71 (m, 4H, H-7, H-8), 1.46 (s, 1H, H-10) ppm;

^{13}C NMR (101 MHz, $CDCl_3$) δ 140.5, 137.0, 134.5, 127.8, 127.0, 125.4, 63.0, 33.9, 32.8, 26.8, 20.8, 15.1 ppm;

IR (film, cm^{-1}): ν_{max} 3322 (br), 2935 (s), 2864 (s), 2362 (w), 2334 (w), 1587 (w), 1470 (s), 1383 (m), 1153 (w), 1508 (s), 1029 (s), 982 (m), 936 (w), 775 (s), 722 (s);

HRMS *The compound did not ionise.*

1-(But-3-en-1-yl)-4-fluoro-2-methylbenzene

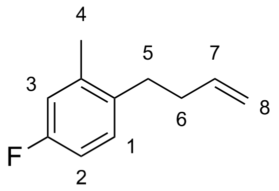

Prepared according **GP8** on a 5.0 mmol scale with respect to 4-fluoro-2-methylbenzyl bromide. Crude title compound obtained as a colourless oil and taken through with no further purification.

4-(4-Fluoro-2-methylphenyl)butan-1-ol (**6r**)

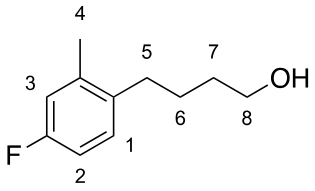

Prepared according to **GP10** on a 3.6 mmol scale with respect to 1-(but-3-en-1-yl)-4-fluoro-2-methylbenzene. Purification by flash column chromatography (SiO_2, 0-30% v/v EtOAc in hexane) afforded the title compound as a colourless oil (156 mg, 0.9 mmol, 23% over two steps).

***R_f* value** = 0.11 (20% v/v EtOAc in hexane);

^{1}H NMR (400 MHz, $CDCl_3$) *δ* 7.06 (dd, *J* = 8.3 Hz, 6.0 Hz, 1H, H-1), 6.86 – 6.77 (m, 2H, H-2, H-3), 3.71 – 3.65 (m, 2H, H-8), 2.62 – 2.56 (m, 2H, H-5), 2.28 (s, 3H, H-4), 1.62 (hept, *J* = 2.5 Hz, 4H, H-6, H-7), 1.42 (s, 1H, OH) ppm;

^{13}C NMR (101 MHz, $CDCl_3$) *δ* 161.0 (d, $J_{C\text{-}F}$ = 242.9 Hz), 137.9 (d, $J_{C\text{-}F}$ = 7.5 Hz), 136.1 (d, $J_{C\text{-}F}$ = 3.2 Hz), 130.0 (d, $J_{C\text{-}F}$ = 8.2 Hz), 116.7 (d, $J_{C\text{-}F}$ = 20.7 Hz), 112.3 (d, $J_{C\text{-}F}$ = 20.5 Hz), 62.8, 32.5, 32.3, 26.5, 19.4 (d, $J_{C\text{-}F}$ = 1.6 Hz) ppm;

^{19}F NMR (376 MHz, $CDCl_3$) δ – 119.4 (s) ppm;

IR (film, cm^{-1}): ν_{max} 3330 (br, m), 2936 (s), 2866 (m), 1611 (w), 1591 (w), 1498 (s), 1452 (w), 1380 (w), 1249 (m), 1188 (w), 1150 (m), 1105 (w), 1061 (m), 1033 (w), 1001 (w), 983(w), 954 (m), 862 (m), 815 (w), 723 (w), 700 (w), 582 (w), 450 (w);

HRMS *The compound did not ionise.*

2-(But-3-en-1-yl)-1,4-dimethylbenzene

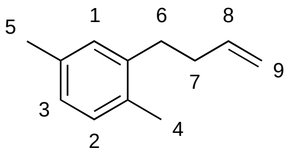

Prepared according to **GP9** on a 5.0 mmol scale with respect to 2,5-dimethylbenzyl chloride. Crude title compound obtained as a colourless oil and taken through with no further purification.

4-(2,5-Dimethylphenyl)butan-1-ol (**6s**)

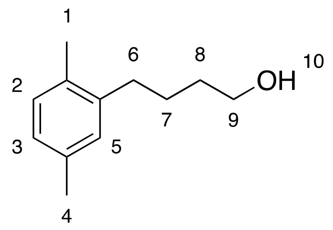

Prepared according to **GP10** on a 3.4 mmol scale with respect to 2-(but-3-en-1-yl)-1,4-dimethylbenzene. Purification by flash column chromatography (SiO_2, 0-30% v/v EtOAc in hexane) afforded the title compound as a colourless oil (163 mg, 0.9 mmol, 35%).

***R_f* value** = 0.42 (30% v/v EtOAc in hexane);

^{1}H NMR (400 MHz, $CDCl_3$) δ 7.03 (d, *J* = 7.6 Hz, 1H, H-2), 6.96 (br s, 1H, H-5), 6.93 (d, *J* = 7.6 Hz, 1H, H-3), 3.67-3.70 (m, 2H, H-9), 2.59-2.62 (m, 2H, H-6), 2.30 (s, 3H, H-4), 2.28 (s, 3H, H-1), 1.61-1.70 (m, 4H, H-7, H-8), 1.50 (s, 1H, H-10) ppm;

^{13}C NMR (101 MHz, $CDCl_3$) δ 140.4, 135.3, 132.7, 130.2, 129.7, 126.7, 63.0, 33.1, 32.8, 26.6, 21.1, 18.9 ppm;

IR (neat, cm^{-1}): ν_{max} 3324 (br), 2931 (s), 2863 (m), 2361 (w), 2334 (w), 1615 (w), 1503 (m), 1456 (m), 1378 (w), 1156 (w), 1119 (w), 1056 (s), 1033 (s), 994 (w), 939 (w), 880 (w), 806 (s);

HRMS *The compound did not ionise.*

4-(2,4-Difluorophenyl)but-3-yn-1-ol

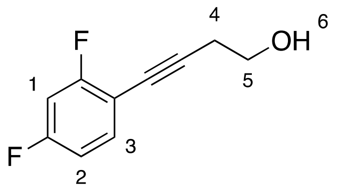

Prepared according **GP5** on a 5.0 mmol scale with respect to 2,4-difluoroiodobenzene. Purification by flash column chromatography (SiO_2, 0-25% v/v EtOAc in petrol) afforded the title compound as a golden oil (754 mg, 4.1 mmol, 83%).

***R_f* value** = 0.38 (30% v/v EtOAc in hexane);

^{1}H NMR (400 MHz, $CDCl_3$) δ 7.38 (q, J = 7.9 Hz, 1H, H-1), 6.82 (t, J = 8.3 Hz, 2H, H-2, H-3), 3.83 (t, J = 6.1 Hz, 2H, H-5), 2.72 (t, J = 6.2 Hz, 2H, H-4), 1.65 (s, 1H, H-6) ppm;

^{13}C NMR (101 MHz, $CDCl_3$) δ 163.3 (dd, $J_{C\text{-}F}$ = 253.2 Hz, 13.0 Hz), 162.5 (dd, $J_{C\text{-}F}$ 251.5 Hz, 11.4 Hz), 134.4 (dd, $J_{C\text{-}F}$ = 9.7 Hz, 2.8 Hz), 111.6 (dd, $J_{C\text{-}F}$ = 21.8 Hz, 3.8 Hz), 108.3 (dd, $J_{C\text{-}F}$ = 16.0 Hz, 4.0 Hz), 104.3 (t, $J_{C\text{-}F}$ = 25.1 Hz), 91.8 (dd, $J_{C\text{-}F}$ = 3.4 Hz, 1.8 Hz), 74.9 (d, $J_{C\text{-}F}$ = 1.1 Hz), 61.1, 24.1 ppm;

^{19}F NMR (376 MHz, $CDCl_3$) δ – 106.4 (d, J = 8.1 Hz), – 107.7 (d, J = 8.1 Hz) ppm;

IR (neat, cm^{-1}): ν_{max} 3371 (br), 2951 (w), 2892 (w), 1685 (w), 1610 (s), 1590 (s), 1503 (s), 1469 (w), 1424 (s), 1298 (w), 1265 (s), 1219 (w), 1187 (w), 1141 (s), 1098 (s), 1042 (s), 965 (s), 850 (s), 814 (m), 734 (m);

HRMS (+ESI) *m/z* found $[M+H]^+$ 183.0620, $[C_{10}H_9F_2O]^+$ requires 183.0616, (δ = + 2.1 ppm).

4-(2,4-Difluorophenyl)butan-1-ol (**6t**)

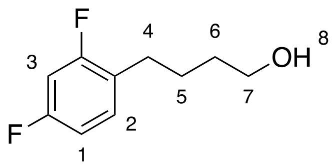

Prepared according to **GP7** on a 4.0 mmol scale with respect to 4-(2,4-difluorophenyl)but-3-yn-1-ol. Purification by flash column chromatography (SiO_2, 0-25% v/v EtOAc in hexane) afforded the title compound as a colourless oil (601 mg, 3.2 mmol, 81%).

***R_f* value** = 0.34 (20% v/v EtOAc in hexane);

^{1}H NMR (400 MHz, $CDCl_3$) δ 7.13 (q, J = 7.9 Hz, 1H, H-3), 6.73-6.80 (m, 2H, H-1, H-2), 3.66 (t, J = 6.4 Hz, 2H, H-7), 2.63 (t, J = 6.9 Hz, 2H, H-4), 1.56-1.70 (m, 4H, H-5, H-6), 1.44 (br s, 1H, H-8) ppm;

^{13}C NMR (101 MHz, $CDCl_3$) δ 161.5 (dd, J_{C-F} = 245.9 Hz, J_{C-F} = 11.9 Hz), 161.1 (dd, J_{C-F} = 247.0 Hz, 11.7 Hz), 131.1 (dd, J_{C-F} = 9.3 Hz, 6.8 Hz), 125.0 (dd, J_{C-F} = 16.3 Hz, 3.8 Hz), 111.0 (dd, J_{C-F} = 20.8 Hz, 3.8 Hz), 103.7 (t, J_{C-F} = 25.7 Hz), 62.8, 32.2, 28.3 (d, J_{C-F} = 1.9 Hz), 26.5 ppm;

^{19}F NMR (376 MHz, $CDCl_3$) δ – 114.0 (d, J = 6.6 Hz), – 114.7 (d, J = 6.5 Hz) ppm;

IR (film, cm^{-1}): ν_{max} 3351 (br), 2936 (m), 2867 (m), 1618 (m), 1601 (m), 1503 (s), 1460 (w), 1427 (w), 1274 (m), 1136 (m), 1100 (m), 1059 (br), 964 (s), 848 (s), 811 (w), 732 (w);

HRMS *The compound did not ionise.*

1-Bromo-3-(bromomethyl)-2-methylbenzene

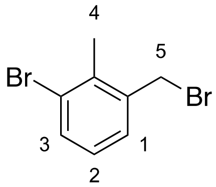

Prepared according to **GP12** on a 5.0 mmol scale with respect to (3-bromo-2-methylphenyl)methanol. Crude title compound obtained as an off-white amorphous solid and taken through with no further purification.

1-Bromo-3-(but-3-en-1-yl)-2-methylbenzene

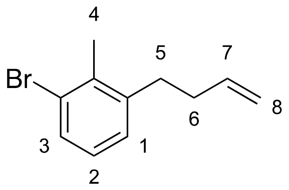

Prepared according to **GP8** on a 2.4 mmol scale with respect to 1-bromo-3-(bromomethyl)-2-methylbenzene. Crude title compound obtained as a golden oil and taken through with no further purification.

4-(3-Bromo-2-methylphenyl)butan-1-ol (**6u**)

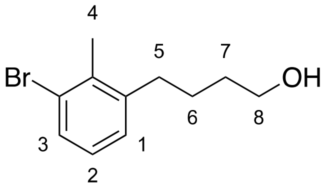

Prepared according to **GP10** on a 1.8 mmol scale with respect to 1-bromo-3-(but-3-en-1-yl)-2-methylbenzene. Purification by flash column chromatography (SiO_2, 10-30% v/v EtOAc in hexane) afforded the title compound as a colourless oil (111 mg, 0.5 mmol, 12% over three steps).

***R_f* value** = 0.11 (20% v/v EtOAc in hexane);

^{1}H NMR (400 MHz, $CDCl_3$) δ 7.39 (dd, *J* = 8.0 Hz, 1.3 Hz, 1H, H-3), 7.06 (dd, *J* = 7.6 Hz, 1.5 Hz, 1H, H-1), 6.95 (t, *J* = 7.7 Hz, 1H, H-2), 3.71 – 3.65 (m, 2H, H-8), 2.73 – 2.64 (m, 2H, H-5), 2.38 (s, 3H, H-4), 1.66 – 1.61 (m, 4H, H-6, H-7), 1.27 (br s, 1H, OH) ppm;

^{13}C NMR (101 MHz, $CDCl_3$) δ 142.6, 135.6, 130.4, 128.3, 126.9, 126.1, 62.8, 34.4, 32.5, 26.5, 19.1 ppm;

IR (film, cm^{-1}): ν_{max} 3340 (br, m), 2938 (s), 2869 (m), 1977 (w), 1562 (w), 1457 (m), 1437 (m), 1380 (w), 1062 (m), 997 (w), 773 (m), 713 (w), 578 (w), 460 (w);

HRMS (+ESI) *The compound did not ionise.*

(3-Chloro-2-methylphenyl)methanol

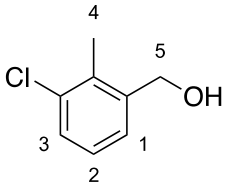

Prepared according to **GP11** on a 16.0 mmol scale with respect to 3-chloro-2-methylbenzoic acid. Purification by flash column chromatography (SiO_2, 0-5% v/v acetone in $CHCl_3$) afforded the title compound as an amorphous white solid (2.19 g, 14.0 mmol, 87%).

^{1}H NMR (400 MHz, $CDCl_3$) δ 7.32 (dd, J = 7.9 Hz, 1.4 Hz, 1H, H-3), 7.26 (dd, J = 7.6 Hz, 1.3 Hz, 1H, H-1), 7.13 (t, J = 7.8 Hz, 1H, H-2), 4.69 (s, 2H, H-5), 2.38 (s, 3H, H-4), 1.83 (s, 1H, OH) ppm;

^{13}C NMR (101 MHz, $CDCl_3$) δ 140.5, 135.1, 134.1, 128.7, 126.7, 126.0, 63.8, 15.3 ppm.

The ^{1}H NMR data is in agreement with that reported in the literature.[37]

1-(Bromomethyl)-3-chloro-2-methylbenzene

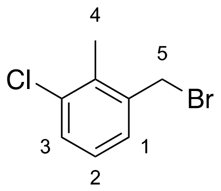

Prepared according to **GP12** on a 14.0 mmol scale with respect to (3-chloro-2-methylphenyl)methanol. Crude title compound obtained as a white amorphous solid and taken through with no further purification.

1-(But-3-en-1-yl)-3-chloro-2-methylbenzene

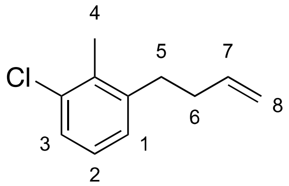

Prepared according to **GP8** on a 11.0 mmol scale with respect to 1-bromo-3-(bromomethyl)-2-methylbenzene. Crude title compound obtained as a pale-yellow oil and taken through with no further purification.

4-(3-Chloro-2-methylphenyl)butan-1-ol (**6v**)

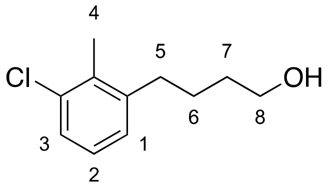

Prepared according to **GP10** on a 9.6 mmol scale with respect to 1-(but-3-en-1-yl)-3-chloro-2-methylbenzene. Purification by flash column chromatography (SiO_2, 0-40% v/v EtOAc in hexane) afforded the title compound as a colourless oil (920 mg, 4.6 mmol, 64%).

***R**$_f$* **value** = 0.31 (32% v/v EtOAc in hexane);

^{1}H NMR (400 MHz, $CDCl_3$) δ 7.20-7.22 (m, 1H, H-3), 7.03-7.06 (m, 2H, H-1, H-2), 3.67 (br s, 2H, H-8), 2.67 (br s, 2H, H-5), 2.36 (s, 3H, H-4), 1.64 (br s, 4H, H-6, H-7), 1.40 (br s, 1H, OH) ppm;

^{13}C NMR (101 MHz, $CDCl_3$) δ 142.7, 135.1, 134.0, 127.7, 127.1, 126.6, 62.9, 34.2, 32.6, 26.6, 16.0 ppm;

IR (neat, cm^{-1}): ν_{max} 3325 (br), 2936 (s), 2865 (m), 2361 (w), 2340 (w), 1595 (w), 1569 (w), 1457 (s), 1440 (s), 1379 (m), 1176 (w), 1136 (w), 1060 (s), 1007 (s), 937 (w), 841 (w), 777 (s), 714 (s), 645 (w);

HRMS *The compound did not ionise.*

1-(But-3-en-1-yl)naphthalene

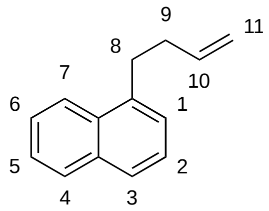

Prepared according to **GP9** on a 5.0 mmol scale with respect to 1-(chloromethyl)-naphthalene. Crude title compound obtained as a colourless oil and taken through with no further purification.

4-(Naphthalen-1-yl)butan-1-ol (**6w**)

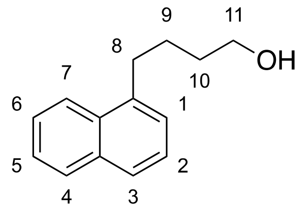

Prepared according to **GP10** on a 3.6 mmol scale with respect to 1-(but-3-en-1-yl)naphthalene. Purification by flash column chromatography (SiO_2, 0-40% v/v EtOAc in hexane) afforded the title compound as a yellow oil (302 mg, 1.5 mmol, 40% over two steps).

^{1}H NMR (400 MHz, $CDCl_3$) δ 8.06 (d, *J* = 8.2 Hz, 1H, H-7), 7.87 (d, *J* = 7.9 Hz, 1H, H-4), 7.73 (d, *J* = 8.1 Hz, 1H, H-3), 7.50 (p, *J* = 7.1 Hz, 2H, H-5, H-6), 7.41 (t, *J* = 6.6 Hz, 1H, H-2), 7.34 (d, *J* = 7.0 Hz, 1H, H-1), 3.68 (t, *J* = 5.6 Hz, 2H, H-11), 3.12 (t, *J* = 6.7 Hz, 2H, H-8), 1.92 – 1.64 (m, 4H, H-9, H-10), 1.50 (s, 1H, OH) ppm;

^{13}C NMR (101 MHz, $CDCl_3$) δ 138.4, 133.9, 131.9, 128.8, 126.6, 126.0, 125.8, 125.6, 125.5, 123.8, 62.8, 32.8, 32.8, 26.9 ppm.

The spectroscopic data is in agreement with that reported in the literature.[38]

4-(3-Methylthiophen-2-yl)but-3-yn-1-ol

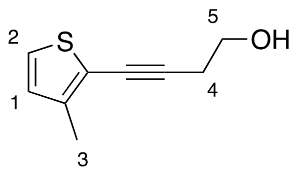

A solution of 2-bromo-3-methylthiophene (0.56 mL, 5.0 mmol, 1.0 equiv.), $PdCl_2(PPh_3)_2$ (175 mg, 0.25 mmol, 5 mol%), CuI (95.2 mg, 0.50 mmol, 10 mol%), PPh_3 (131 mg, 0.50 mmol, 10 mol%) and *N*,*N*-dimethylformamide (10 mL) in a two-neck flask fitted with a reflux condenser was stirred at room temperature for 10 minutes. $^{i}Pr_2NH$ (5.0 mL) and but-3-yn-1-ol (0.76 mL, 10 mmol, 2.0 equiv.) were then added and the reaction mixture was refluxed for 16 h. The reaction mixture was then allowed to cool to room temperature and EtOAc (20 mL) was added. The reaction mixture was filtered through Celite® and the filtrate was washed with aq. LiCl (10% w/v) and brine, dried ($MgSO_4$), filtered, and concentrated *in vacuo*. Purification by flash column chromatography (SiO_2, 0-30% v/v EtOAc in Hexane) afforded the title compound as a golden oil (764 mg, 4.6 mmol, 92%)

^{1}H NMR (400 MHz, $CDCl_3$) δ 7.07 (d, J = 5.1 Hz, 1H, H-2), 6.80 (d, J = 5.1 Hz, 1H, H-1), 3.81 (t, J = 6.3 Hz, 2H, H-5), 2.74 (t, J = 6.3 Hz, 2H, H-4), 2.29 (s, 3H, H-3), 1.97 (br s, 1H, OH) ppm;

^{13}C NMR (101 MHz, $CDCl_3$) δ 142.1, 129.2, 125.1, 118.7, 92.6, 75.4, 61.3, 24.3, 15.0 ppm.

The spectroscopic data is in agreement with that reported in the literature.[39]

4-(3-methylthiophen-2-yl)butan-1-ol (**6x**)

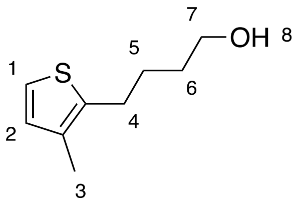

Prepared according **GP7** on a 4.5 mmol scale with respect to 4-(3-methylthiophen-2-yl)but-3-yn-1-ol with some procedural modifications: 20 mol % of Pd/C (10 wt % loading Pd) was used and the reaction mixture was stirred at 60 °C for 72 hours. Purification by flash column chromatography ($AgNO_3$-impregnated SiO_2, 5-30% v/v EtOAc in hexane) afforded the title compound as a yellow oil (132 mg, 0.78 mmol, 17%).

***R**$_f$* **value** = 0.56 (15% v/v acetone in $CHCl_3$);

^{1}H NMR (500 MHz, $CDCl_3$) *δ* 7.02 (d, *J* = 5.1 Hz, 1H, H-1), 6.78 (d, *J* = 5.1 Hz, 1H, H-2), 3.67 (t, *J* = 6.3 Hz, 2H, H-7), 2.76 (t, *J* = 7.1 Hz, 2H, H-4), 2.16 (s, 3H, H-3), 1.61-1.74 (m 4H, H-5, H-6), 1.36 (s, 1H, H-8) ppm;

^{13}C NMR (126 MHz, $CDCl_3$) *δ* 138.3, 132.7, 130.1, 121.1, 62.9, 32.4, 27.8, 27.7, 13.7 ppm;

IR (film, cm^{-1}): ν_{max} 3331 (br), 2931 (s), 2860 (s), 2361 (w), 2337 (w), 1742 (w), 1556 (w), 1454 (m), 1383 (w), 1332 (w), 1259 (w), 1232 (w), 1164 (w), 1057 (s), 1030 (m), 944 (w), 876 (w), 833 (w), 696 (s), 604 (m);

HRMS *The compound did not ionise.*

N-(4-phenylbutyl)acetamide

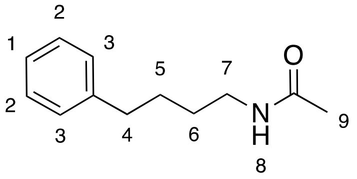

To a solution of 4-phenylbutylamine (0.79 mL, 5.0 mmol) in CH_2Cl_2 (10 mL) at 0 °C were successively added Ac_2O (0.52 mL, 5.5 mmol) and Et_3N (0.77 mL, 5.5 mmol) dropwise. The

reaction mixture was allowed to warm to room temperature and stirred for 36 hours. The reaction mixture was then diluted with CH_2Cl_2 (10 mL) and the organic layer washed with 3 M HCl (2 x 5 mL), saturated aqueous $NaHCO_3$ (5 mL) and brine (5 mL). The organic extracts were dried ($MgSO_4$) and the solvent removed under reduced pressure. Purification by flash column chromatography (SiO_2, 80-100% v/v EtOAc in hexane) afforded the title compound as a thick, colourless oil (696 mg, 3.6 mmol, 73%).

***R_f* value** = 0.20 (80% v/v EtOAc in hexane);

^{1}H NMR (400 MHz, $CDCl_3$) δ 7.26-7.29 (m, 2H, H-2), 7.15-7.19 (m, 3H, H-1, H-3), 5.72 (br s, 1H, H-8), 3.24 (q, J = 7.1 Hz, 2H, H-7), 2.62 (t, J = 7.5 Hz, 2H, H-4), 1.94 (s, 3H, H-9), 1.60-1.68 (m, 2H, H-5), 1.48-1.56 (m, 2H, H-6) ppm;

^{13}C NMR (101 MHz, $CDCl_3$) δ 170.2, 142.2, 128.5, 128.4, 125.9, 39.6, 35.6, 29.2, 28.8, 23.4 ppm;

IR (film, cm^{-1}): ν_{max} 3290 (br), 2929 (m), 2857 (m), 1647 (s), 1554 (s), 1453 (m), 1367 (m), 1294 (m), 1177 (w), 748 (m), 699 (m).

The spectroscopic data is in agreement with that reported in the literature.[40]

4-Phenylbutane-1-thiol

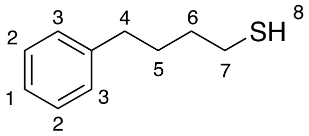

The protocol described by Wu and co-workers was employed.[41] To a solution of thiourea (381 mg, 5.0 mmol) in EtOH (10 mL) was added 1-bromo-4-phenylbutane (1.07 g, 5.0 mmol) and the reaction mixture heated under reflux for 4 hours. The reaction mixture was then allowed to cool and the EtOH was removed under reduced pressure. A solution of NaOH (280 mg, 7.0 mmol) in water (5 mL) was added to the residue and the reaction mixture was heated under reflux for 2 hours. The reaction mixture was then allowed to cool and acidified with aqueous

H_2SO_4 (15%). The crude product was then extracted with Et_2O, the organic extracts washed with water, dried ($MgSO_4$) and the solvent removed under reduced pressure. Purification through a plug of SiO_2, eluting with 60% v/v EtOAc in hexane afforded the title compound as an odoriferous, pale-yellow oil (694 mg, 4.2 mmol, 83%).

***R_f* value** = 0.67 (2% v/v EtOAc in hexane);

^{1}H NMR (400 MHz, $CDCl_3$) δ 7.31 (t, *J* = 6.9 Hz, 2H, H-2), 7.20-7.23 (m, 3H, H-1, H-3), 2.65 (t, *J* = 6.9 Hz, 2H, H-4), 2.57 (q, *J* = 7.0 Hz, 2H, H-7), 1.64-1.79 (m, 4H, H-5, H-6), 1.36 (t, *J* = 7.7 Hz, 1H, H-8) ppm;

^{13}C NMR (101 MHz, $CDCl_3$) δ 142.2, 128.5, 128.4, 125.9, 35.4, 33.6, 30.2, 24.6 ppm;

IR (neat, cm^{-1}): ν_{max} 3061 (w), 3025 (w), 2931 (m), 2855 (m), 1603 (m), 1495 (m), 1452 (m), 1267 (w), 1029 (w), 909 (w), 809 (w), 743 (s), 697 (s).

The NMR data is in agreement with that reported in the literature.[42]

Enantioselective Intermolecular Amination Products

<u>*Note on intermolecular amination reactions performed using $Rh_2(esp)_2$:*</u> In order to obtain racemic SFC/HPLC traces, racemic intermolecular amination reactions were run alongside the corresponding chiral ones. The racemic reactions were performed according to **GP13** using $Rh_2(esp)_2$ as the catalyst and on a 0.25 mmol scale with respect to the alcohol starting material. For products **7a**-**7x**, catalyst Rh_2(**D**)$_2$•(**2a**)$_2$ convincingly outperformed $Rh_2(esp)_2$ in terms of yield. Further, in many instances where $Rh_2(esp)_2$ was used, an inseparable side product was often obtained along with the desired intermolecular benzylic amination product after column chromatography in different solvent systems. The side product was most prevalent in cases where the benzylic amination product yield was especially low and could never be isolated fully pure. Analysis by ^{1}H NMR of both crude racemic reaction mixtures containing the impurity and isolated, contaminated benzylic amination products leads us to tentatively suggest that this side product likely arises due to oxidation of the unfunctionalized alcohol starting material to the corresponding carboxylic acid. Whilst the presence of the side product was frequently observed in varying amounts in the racemic reactions and is often visible as a contaminant in the HPLC/SFC traces, it was almost never observed in the enantioselective reactions.

2,2,3,3,4,4,4-Heptafluorobutyl (R)-(4-hydroxy-1-phenylbutyl)sulfamate (**7a**)

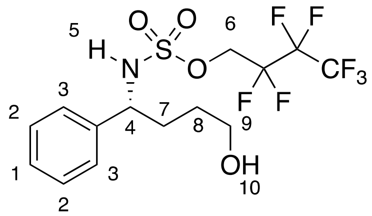

Prepared according to **GP13** using Rh_2(**D**)$_2$•(**2a**)$_2$ as the catalyst and 4-phenylbutan-1-ol (**6a**) as the starting material. Purification by flash column chromatography (SiO_2, 0-20% v/v

acetone in $CHCl_3$) afforded the title compound as a white amorphous solid (38.6 mg, 0.090 mmol, 90%, 90% *ee*).

***Rf* value** = 0.45 (50% v/v EtOAc in hexane);

^{1}H NMR (500 MHz, $CDCl_3$) δ 7.36 (t, *J* = 7.4 Hz, 2H, H-2), 7.29-7.32 (m, 3H, H-1, H-3), 6.20 (d, *J* = 4.8 Hz, 1H, H-5), 4.48 (q, *J* = 6.3 Hz, 1H, H-4), 4.18 (q, *J* = 12.7 Hz, 1H, H-6a), 4.11 (q, *J* = 12.7 Hz, 1H, H-6b), 3.66-3.73 (m, 2H, H-9a, H-9b), 2.00-2.05 (m, 1H, H-7a), 1.92-1.97 (m, 1H, H-7b), 1.89 (br s, 1H, H-10), 1.63-1.69 (m, 1H, H-8a), 1.57-1.62 (m, 1H, H-8b) ppm;

^{13}C NMR (126 MHz, $CDCl_3$) δ 140.6, 129.0, 128.3, 126.6, 117.5 (qt, $J_{C\text{-}F}$ = 288.0 Hz, 34.7 Hz), 113.2 (tt, $J_{C\text{-}F}$ = 258.5 Hz, 30.8 Hz), 105.9-111.0 (m), 64.0 (t, $J_{C\text{-}F}$ = 26.9 Hz), 62.4, 59.3, 33.9, 28.4 ppm;[7]

^{19}F NMR (376 MHz, $CDCl_3$) δ – 81.8 (t, *J* = 9.2 Hz), – 121.7 - – 121.8 (m), – 128.5 - – 128.6 (m) ppm;

IR (neat, cm^{-1}): ν_{max} 3137 (br), 2949 (w), 2159 (m), 2029 (w), 1978 (w), 1448 (m), 1352 (s), 1298 (w), 1225 (s), 1175 (s), 1130 (s), 1089 (w), 1046 (s), 999 (m), 953 (m), 913 (m), 803 (m), 754 (s), 694 (s);

$[\boldsymbol{\alpha}]_{\mathbf{D}}^{\mathbf{25.0}}$ = + 20.9 (c. 0.91, $CHCl_3$);

HRMS (−ESI) *m/z* Found [M−H]$^-$ 426.0623, $[C_{14}H_{15}F_7NO_4S]^-$ requires 426.0615, (δ = + 1.9 ppm);

Chiral SFC Analysis CHIRAL ART SC (CO_2:^{i}PrOH, 96:4, 2.5 mL min^{-1}, 40 °C) t_R = 12.6 (major), 15.3 (minor) minutes.

The opposite enantiomer (***ent*-7a**) was prepared according to **GP13** using Rh_2(**D**)$_2$•(**10**)$_2$ as the catalyst and 4-phenylbutan-1-ol (**6a**) as the starting material. Purification by flash column chromatography (SiO_2, 0-20% v/v acetone in $CHCl_3$) afforded 2,2,3,3,4,4,4-heptafluorobutyl (*S*)-(4-hydroxy-1-phenylbutyl)sulfamate as a white amorphous solid (28.8 mg, 67%, 89% ee).

In a control experiment **7a** was prepared according to **GP13** on a 0.25 mmol scale with respect to 4-phenylbutan-1-ol (**6a**), using Rh_2(esp)$_2$ as the catalyst and with the addition of (**2a•Br**) (2 mol %) as an additive (introduced into the reaction vial prior to the addition of the sulfamate ester stock solution). Purification by flash column chromatography (SiO_2, 0-20% v/v acetone in $CHCl_3$) afforded the title compound as a white amorphous solid (54.9 mg, 51%, 19% *ee*).

In a further control experiment, ***rac*-7a** was prepared according to **GP13** using Rh_2(**D**)$_2$•(**Bu$_4$N**)$_2$ as the catalyst and 4-phenylbutan-1-ol (**6a**) as the starting material. Purification by flash column chromatography (SiO_2, 0-20% v/v acetone in $CHCl_3$) afforded the title compound as a white amorphous solid (3.1 mg, 7%, racemic).

Ethyl (R)-3-(1-(((2,2,3,3,4,4,4-heptafluorobutoxy)sulfonyl)amino)-4-hydroxybutyl)benzoate (**7b**)

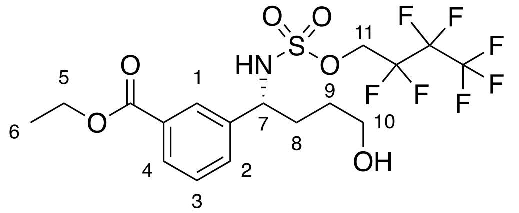

Prepared according to **GP13** using Rh_2(**D**)$_2$•(**2a**)$_2$ as the catalyst and ethyl 3-(4-hydroxybutyl)benzoate (**6b**) as the starting material. Purification by flash column chromatography (SiO_2, 0-20% v/v acetone in $CHCl_3$) afforded the title compound as a colourless oil (30.0 mg, 0.060 mmol, 60%, 86% *ee*).

***R$_f$* value** = 0.45 (20% v/v acetone in $CHCl_3$);

^{1}H NMR (500 MHz, $CDCl_3$) δ 8.02 (t, *J* = 1.4 Hz, 1H, H-1), 7.98 (dt, *J* = 7.7 Hz, 1.3 Hz, 1H, H-4), 7.50 (d, *J* = 7.7 Hz, 1H, H-2), 7.43 (t, *J* = 7.7 Hz, 1H, H-3), 6.83 (br d, *J* = 7.0 Hz, 1H, NH), 4.55 (q, *J* = 7.1 Hz, 1H, H-7), 4.38 (q, *J* = 7.1 Hz, 2H, H-5a, H-5b), 4.28 (q, *J* = 13.0 Hz, 1H, H-11a), 4.20 (q, *J* = 13.0 Hz, 1H, H-11b), 3.64-3.76 (m, 2H, H-10a, H-10b), 2.09 (br s, 1H, OH), 1.90-2.07 (m, 2H, H-8a, H-8b), 1.64-1.73 (m, 1H, H-9a), 1.54-1.63 (m, 1H, H-9b), 1.39 (t, *J* = 7.1 Hz, 3H, H-6) ppm;

^{13}C NMR (126 MHz, $CDCl_3$) *δ* 166.7, 141.5, 131.4, 131.2, 129.3, 129.0, 127.4, 117.5 (qt, J_{C-F} = 289 Hz, 33.4 Hz), 113.2 (tt, J_{C-F} = 258 Hz, 31.1 Hz), 105.4-111.1 (m), 63.9 (t, J_{C-F} = 27.2 Hz), 62.3, 61.5, 58.9, 34.1, 28.3, 14.4 ppm;[7]

^{19}F NMR (376 MHz, $CDCl_3$) *δ* – 80.9 (t, *J* = 9.2 Hz), – 120.7 - – 120.9 (m), – 127.5 - – 127.6 (m) ppm;

IR (film, cm^{-1}) ν_{max} 3271 (br, w), 2939 (w), 1697 (m), 1448 (m), 1370 (m), 1287 (m), 1226 (s), 1179 (s), 1123 (m), 1048 (m), 1015 (m), 959 (m);

$[\alpha]_D^{25.0}$ = + 28.2 (c. 0.91, $CHCl_3$);

HRMS (+ESI) *m/z* Found [M+H]$^+$ 500.0954, $[C_{17}H_{21}F_7NO_6S]^+$ requires 500.0972, (*δ* = – 3.6 ppm);

Chiral SFC Analysis CHIRAL ART SB (CO_2:MeOH, 97:3, 2.5 mL min^{-1}, 40 °C) t_R = 13.1 (major), 15.5 (minor) minutes.

2,2,3,3,4,4,4-Heptafluorobutyl (R)-(4-hydroxy-1-(o-tolyl)butyl)sulfamate (**7c**)

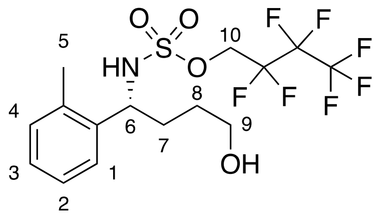

Prepared according to **GP13** using Rh_2(**D**)$_2$•(**2a**)$_2$ as the catalyst and 4-(*o*-tolyl)butan-1-ol (**6c**) as the starting material. Purification by flash column chromatography (SiO_2, 0-15% v/v acetone in $CHCl_3$) afforded the title compound as a white powder (30.2 mg, 0.068 mmol, 68%, 92% *ee*).

***R_f* value** = 0.36 (20% v/v acetone in $CHCl_3$);

^{1}H NMR (500 MHz, $CDCl_3$) *δ* 7.28 (d, *J* = 7.3 Hz, 1H, H-1), 7.22 (td, *J* = 7.4 Hz, 1.9 Hz, 1H, H-3), 7.21–7.15 (m, 2H, H-2, H-4), 6.22 (br d, *J* = 7.0 Hz, 1H, NH), 4.79 (q, *J* = 6.9 Hz, 1H, H-6), 4.13 (q, *J* = 12.9 Hz, 1H, H-10a), 4.01 (q, *J* = 12.9 Hz, 1H, H-10b), 3.70-3.76 (m, 1H, H-9a), 3.64-3.70 (m, 1H, H-9b), 2.39 (s, 3H, H-5), 1.85-2.01 (m, 3H, H-7a, H-7b, OH), 1.65-1.75 (m, 1H, H-8a), 1.56-1.65 (m, 1H, H-8b) ppm;

^{13}C NMR (126 MHz, $CDCl_3$) δ 139.2, 135.2, 131.0, 128.0, 126.8, 125.5, 117.5 (qt, $J_{C\text{-}F}$ = 287 Hz, 33.4 Hz), 113.2 (tt, $J_{C\text{-}F}$ = 258 Hz, 31.0 Hz), 105.4-111.1 (m), 63.9 (t, $J_{C\text{-}F}$ = 27.0 Hz), 62.4, 55.0, 33.5, 28.5, 19.2 ppm;[7]

^{19}F NMR (376 MHz, $CDCl_3$) δ – 81.8 (t, J = 9.2 Hz), – 121.7 - – 121.9 (m), – 128.5 - – 128.6 (m) ppm;

IR (film, cm^{-1}) ν_{max} 3305 (br, w), 2925 (w), 1454 (m), 1356 (m), 1295 (w), 1226 (s), 1179 (s), 1125 (m), 1052 (m), 1015 (m), 960 (m);

$[\alpha]_D^{25.0}$ = + 19.0 (c. 0.91, $CHCl_3$);

HRMS (−ESI) *m/z* Found [M−H]$^-$ 440.0774, $[C_{15}H_{17}F_7NO_4S]^-$ requires 440.0772, (δ = + 0.5 ppm);

HPLC Analysis CHIRALPAK IC (Hexane:^{i}PrOH, 96:4, 1.0 mL min^{-1}, 30 °C) t$_R$ = 14.9 (major), 22.7 (minor) minutes.

2,2,3,3,4,4,4-Heptafluorobutyl (R)-(4-hydroxy-1-(m-tolyl)butyl)sulfamate (**7d**)

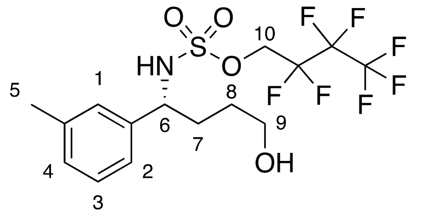

Prepared according to **GP13** using $Rh_2(\mathbf{D})_2\bullet(\mathbf{2a})_2$ as the catalyst and 4-(*m*-tolyl)butan-1-ol (**6d**) as the starting material. Purification by flash column chromatography (*Note:* two consecutive purification procedures were carried out: SiO_2, 0-25% v/v acetone in $CHCl_3$ followed by SiO_2, 0-50% v/v EtOAc in hexane) afforded the title compound as a colourless oil (31.9 mg, 0.072 mmol, 72%, 84% *ee*).

***R$_f$* value** = 0.38 (20% v/v acetone in $CHCl_3$);

^{1}H NMR (500 MHz, $CDCl_3$) δ 7.24 (t, J = 7.9 Hz, 1H, H-3), 7.06-7.14 (m, 3H, H-1, H-2, H-4), 6.14 (br d, J = 5.8 Hz, 1H, NH), 4.43 (q, J = 6.4 Hz, 1H, H-6), 4.17 (q, J = 13.1 Hz, 1H, H-10a), 4.08 (q, J = 13.0 Hz, 1H, H-10b), 3.63-3.74 (m, 2H, H-9a, H-9b), 2.34 (s, 3H, H-5), 1.97-2.06 (m, 1H, H-

7a), 1.87-1.96 (m, 1H, H-7b), 1.89 (br s, 1H, OH), 1.62-1.70 (m, 1H, H-8a), 1.54-1.62 (m, 1H, H-8b) ppm;

^{13}C NMR (126 MHz, $CDCl_3$) δ 140.6, 138.9, 129.1, 128.9, 127.4, 123.5, 117.5 (qt, $J_{C\text{-}F}$ = 288 Hz, 33.5 Hz), 113.2 (tt, $J_{C\text{-}F}$ = 258 Hz, 31.0 Hz), 105.7-111.4 (m), 63.9 (t, $J_{C\text{-}F}$ = 26.9 Hz), 62.4, 59.4, 33.8, 28.6, 21.4 ppm;[7]

^{19}F NMR (376 MHz, $CDCl_3$) δ – 80.9 (t, J = 9.2 Hz), – 120.8 - – 120.9 (m), – 127.5 - – 127.7 (m) ppm;

IR (film, cm^{-1}) ν_{max} 3536 (br, w), 3301 (br, w), 2925 (m), 1450 (m), 1356 (m), 1294 (m), 1227 (s), 1179 (s), 1125 (m), 1047 (m), 1014 (m), 960 (m);

$[\alpha]_D^{25.0}$ = + 20.5 (c. 0.91, $CHCl_3$);

HRMS (−ESI) *m/z* Found $[M-H]^-$ 440.0771; $[C_{15}H_{17}F_7NO_4S]^-$ requires 440.0772, (δ = – 0.2 ppm);

Chiral SFC Analysis CHIRAL ART SC (CO_2: ^{i}PrOH, 96:4, 2.5 mL min^{-1}, 40 °C) t_R = 12.6 (major), 15.0 (minor) minutes.

2,2,3,3,4,4,4-Heptafluorobutyl (R)-(1-(3-fluorophenyl)-4-hydroxybutyl)sulfamate (**7e**)

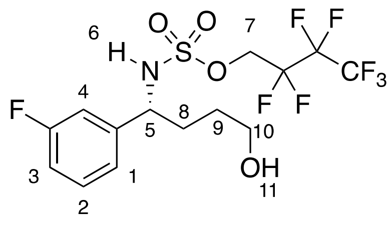

Prepared according to **GP13** using Rh_2(**D**)$_2$•(**2a**)$_2$ as the catalyst and 4-(3-fluorophenyl)butan-1-ol (**6e**) as the starting material. Purification by flash column chromatography (SiO_2, 0-20% v/v acetone in $CHCl_3$) afforded the title compound as a yellow oil (25.1 mg, 0.056 mmol, 56%, 87% *ee*).

***R_f* value** = 0.37 (20% v/v acetone in $CHCl_3$);

^{1}**H NMR** (500 MHz, $CDCl_3$) δ 7.30-7.35 (m, 1H, H-2), 7.08 (d, *J* = 7.7 Hz, 1H, H-1), 6.98-7.04 (m, 2H, H-3, H-4), 6.65 (d, *J* = 6.9 Hz, 1H, H-6), 4.48 (q, *J* = 6.9 Hz, 1H, H-5), 4.28 (q, *J* = 12.6 Hz, 1H, H-7a), 4.21 (q, *J* = 12.6 Hz, 1H, H-7b), 3.66-3.75 (m, 2H, H-10a, H-10b), 2.07 (br s, 1H, H-11), 1.89-2.03 (m, 2H, H-8a, H-8b), 1.56-1.74 (m, 2H, H-9a, H-9b) ppm;

^{13}**C NMR** (126 MHz, $CDCl_3$) δ 163.1 (d, $J_{C\text{-}F}$ = 247.1 Hz), 143.4 (d, $J_{C\text{-}F}$ = 6.6 Hz), 130.6 (d, $J_{C\text{-}F}$ = 8.2 Hz), 122.3 (d, $J_{C\text{-}F}$ = 2.0 Hz), 117.5 (qt, $J_{C\text{-}F}$ = 287.7 Hz, 33.7 Hz), 115.1 (d, $J_{C\text{-}F}$ = 21.2 Hz), 113.6 (d, $J_{C\text{-}F}$ = 22.2 Hz), 113.2 (tt, $J_{C\text{-}F}$ = 258.3 Hz, 31.2 Hz), 105.9-111.0 (m), 64.0 (t, $J_{C\text{-}F}$ = 27.1 Hz), 62.3, 58.7, 34.0, 28.1 ppm;[7]

^{19}**F NMR** (376 MHz, $CDCl_3$) δ – 81.9 (t, *J* = 9.2 Hz), – 113.1 (s), – 121.7 - – 121.8 (m), – 128.5 - – 128.7 (m) ppm;

IR (film, cm^{-1}): ν_{max} 3307 (br), 2925 (m), 1595 (w), 1454 (m), 1356 (s), 1228 (s), 1180 (s), 1126 (m), 1048 (m), 1015 (m), 960 (w), 915 (w), 791 (m), 760 (m), 699 (m);

$[\alpha]_D^{25.0}$ = + 17.9 (c. 1.0, $CHCl_3$);

HRMS (–ESI) *m/z* Found $[M-H]^-$ 444.0509, $[C_{14}H_{14}F_8NO_4S]^-$ requires 444.0521, (δ = – 2.7 ppm);

Chiral SFC Analysis CHIRAL ART SB (CO_2:^{i}PrOH, 96:4, 2.5 mL min^{-1}, 40 °C) t_R = 13.1 (major), 15.4 (minor) minutes.

2,2,3,3,4,4,4-Heptafluorobutyl (R)-(1-(2-fluorophenyl)-4-hydroxybutyl)sulfamate (**7f**)

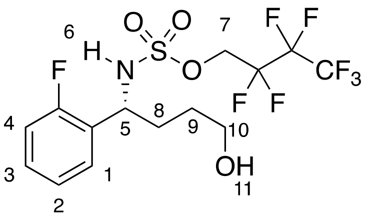

Prepared according to **GP13** using Rh_2(**D**)$_2$•(**2a**)$_2$ as the catalyst and 4-(2-fluorophenyl)butan-1-ol (**6f**) as the starting material. Purification by flash column chromatography (SiO_2, 0-15%

v/v acetone in $CHCl_3$) afforded the title compound as a yellow oil (18.5 mg, 0.042 mmol, 42%, 93% *ee*).

***Rf* value** = 0.36 (20% v/v acetone in $CHCl_3$);

^{1}H NMR (500 MHz, $CDCl_3$) δ 7.28-7.32 (m, 2H, H-1, H-3), 7.14 (tt, J = 7.6 Hz, 1.1 Hz, 1H, H-2), 7.05-7.09 (m, 1H, H-4), 6.24 (d, J = 8.1 Hz, 1H, H-6), 4.69 (q, J = 8.0 Hz, 1H, H-5), 4.28 (q, J = 12.7 Hz, 1H, H-7a), 4.22 (q, J = 12.7 Hz, 1H, H-7b), 3.66-3.74 (m, 2H, H-10a, H-10b), 2.03-2.10 (m, 1H, H-8a), 1.93-2.00 (m, 1H, H-8b), 1.80 (br s, 1H, H-11), 1.65-1.73 (m, 1H, H-9a), 1.54-1.62 (m, 1H, H-9b) ppm;

^{13}C NMR (126 MHz, $CDCl_3$) δ 160.4 (d, $J_{C\text{-}F}$ = 245.7 Hz), 129.9 (d, $J_{C\text{-}F}$ = 8.5 Hz), 128.8 (d, $J_{C\text{-}F}$ = 3.9 Hz), 127.7 (d, $J_{C\text{-}F}$ = 12.6 Hz), 124.7 (d, $J_{C\text{-}F}$ = 2.8 Hz), 117.5 (qt, $J_{C\text{-}F}$ = 287.5 Hz, 33.5 Hz), 116.1 (d, $J_{C\text{-}F}$ = 21.7 Hz), 113.2 (tt, $J_{C\text{-}F}$ = 258.4 Hz, 31.1 Hz), 105.9-111.0 (m), 64.0 (t, $J_{C\text{-}F}$ = 27.2 Hz), 62.3, 55.1, 32.8, 28.6 ppm;[7]

^{19}F NMR (376 MHz, $CDCl_3$) δ – 81.9 (t, J = 9.2 Hz), – 119.7 (s), – 121.8 - – 121.9 (m), – 128.5 - – 128.6 (m) ppm;

IR (film, cm^{-1}): ν_{max} 3303 (br), 2925 (w), 2358 (w), 2330 (w), 1493 (m), 1457 (m), 1373 (m), 1356 (w), 1229 (s), 1188 (s), 1126 (m), 1050 (m), 1016 (m), 960 (m), 916 (m), 837 (w), 803 (m), 760 (s);

$[\alpha]_D^{25.0}$ = + 20.1 (c. 1.0, $CHCl_3$);

HRMS (−ESI) *m/z* Found $[M-H]^-$ 444.0518, $[C_{14}H_{14}F_8NO_4S]^-$ requires 444.0521, (δ = – 0.7 ppm);

Chiral HPLC Analysis CHIRALPAK IC (Hexane:^{i}PrOH, 96:4, 1.0 mL min^{-1}, 30 °C) t_R = 17.6 (major), 26.9 (minor) minutes.

2,2,3,3,4,4,4-Heptafluorobutyl (R)-(1-(4-fluorophenyl)-4-hydroxybutyl)sulfamate (**7g**)

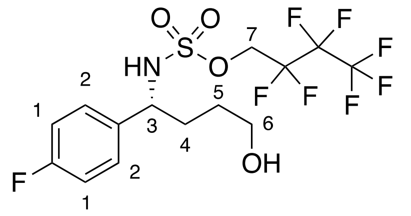

Prepared according to **GP13** using Rh_2(**D**)$_2$•(**2a**)$_2$ as the catalyst and 4-(4-fluorophenyl)butan-1-ol (**6g**) as the starting material. Purification by flash column chromatography (SiO_2, 0-20% v/v acetone in $CHCl_3$) afforded the title compound as an off-white powder (34.6 mg, 0.078 mmol, 78%, 89% *ee*).

***R**$_f$* **value** = 0.33 (20% v/v acetone in $CHCl_3$);

^{1}H NMR (500 MHz, $CDCl_3$) δ 7.27 (dd, J = 8.5 Hz, 5.2 Hz, 2H, H-2), 7.04 (t, J = 8.5 Hz, 2H, H-1), 6.54 (br d, J = 6.7 Hz, 1H, NH), 4.47 (q, J = 6.8 Hz, 1H, H-3), 4.25 (q, J = 12.9 Hz, 1H, H-7a), 4.16 (q, J = 12.9 Hz, 1H, H-7b), 3.69-3.75 (m, 1H, H-6a), 3.63-3.69 (m, 1H, H-6b), 2.16 (br s, 1H, OH), 1.94-2.04 (m, 1H, H-4a), 1.85-1.94 (m, 1H, H-4b), 1.52-1.71 (m, 2H, H-5a, H-5b) ppm;

^{13}C NMR (126 MHz, $CDCl_3$) δ 162.5 (d, $J_{C\text{-}F}$ = 247 Hz), 136.6 (d, $J_{C\text{-}F}$ = 3.2 Hz), 128.3 (d, $J_{C\text{-}F}$ = 8.2 Hz), 117.6 (qt, $J_{C\text{-}F}$ = 287 Hz, 33.3 Hz), 115.9 (d, $J_{C\text{-}F}$ = 21.8 Hz), 113.2 (tt, $J_{C\text{-}F}$ = 258 Hz, 31.1 Hz), 105.5-111.4 (m), 64.0 (t, $J_{C\text{-}F}$ = 27.1 Hz), 62.3, 58.6, 34.1, 28.3 ppm;[7]

^{19}F NMR (376 MHz, $CDCl_3$) δ – 80.9 (t, J = 9.2 Hz), – 114.1 (s), – 120.7 - – 120.9 (m), – 127.6 - – 127.7 (m) ppm;

IR (neat, cm^{-1}): ν_{max} 3539 (br, w), 3135 (br, w), 2954 (w), 1899 (w), 1605 (w), 1511 (m), 1484 (w), 1448 (m), 1355 (m), 1224 (s), 1177 (s), 1130 (s), 1099 (m), 1046 (s), 999 (s);

$[\alpha]_D^{25.0}$ = + 25.9 (c. 0.91, $CHCl_3$);

HRMS (−ESI) *m/z* Found $[M-H]^-$ 444.0519; $[C_{14}H_{14}F_8NO_4S]^-$ requires 444.0521, (δ = – 0.5 ppm);

Chiral SFC Analysis CHIRAL ART SC (CO_2:^{i}PrOH, 96:4, 2.5 mL min^{-1}, 40 °C) t_R = 9.4 (major), 11.1 (minor) minutes.

2,2,3,3,4,4,4-Heptafluorobutyl (R)-(1-(3-chlorophenyl)-4-hydroxybutyl)sulfamate (**7h**)

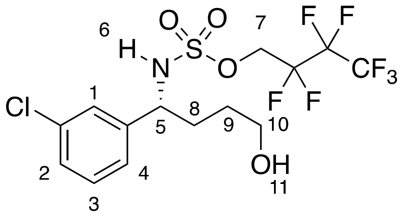

Prepared according to **GP13** using $Rh_2(\mathbf{D})_2•(\mathbf{2a})_2$ as the catalyst and 4-(3-Chlorophenyl)butan-1-ol (**6h**) as the starting material. In this case the catalyst loading was 3 mol % instead of 1 mol %. Purification by flash column chromatography (SiO_2, 0-20% v/v acetone in $CHCl_3$) afforded the title compound as a colourless oil (23.5 mg, 0.051 mmol, 51%, 87% *ee*).

***R_f* value** = 0.30 (20% v/v acetone in $CHCl_3$);

^{1}H NMR (500 MHz, $CDCl_3$) *δ* 7.27-7.32 (m, 3H, H-1, H-2, H-3), 7.18-7.20 (m, 1H, H-4), 6.45 (d, *J* = 6.4 Hz, 1H, H-6), 4.48 (q, *J* = 6.4 Hz, 1H, H-5), 4.32 (q, *J* = 12.2 Hz, 1H, H-7a), 4.26 (q, *J* = 12.2 Hz, 1H, H-7b), 3.68-3.77 (m, 2H, H-10a, H-10b), 1.92-2.04 (m, 2H, H-8a, H-8b), 1.80 (br s, 1H, H-11), 1.58-1.71 (m, 2H, H-9a, H-9b) ppm;

^{13}C NMR (126 MHz, $CDCl_3$) *δ* 142.9, 134.9, 130.3, 128.4, 126.8, 124.9, 117.5 (qt, $J_{C\text{-}F}$ = 287.4 Hz, 33.4 Hz), 113.2 (tt, $J_{C\text{-}F}$ = 258.3 Hz, 31.1 Hz), 105.9-111.1 (m), 64.0 (t, $J_{C\text{-}F}$ = 27.1 Hz), 62.4, 58.6, 34.2, 28.0 ppm;[7]

^{19}F NMR (376 MHz, $CDCl_3$) *δ* – 81.8 (t, *J* = 9.3 Hz), – 121.7 - – 121.8 (m), – 128.5 - – 128.6 (m) ppm;

IR (film, cm^{-1}): ν_{max} 3304 (br), 2927 (w), 1599 (w), 1577 (w), 1476 (w), 1437 (m), 1356 (m), 1296 (w), 1229 (s), 1182 (s), 1126 (m), 1048 (m), 1016 (m), 960 (m), 915 (m), 836 (w), 790 (m), 699 (m);

$[\alpha]_{\mathbf{D}}^{\mathbf{25.0}}$ = + 23.2 (c. 1.0, $CHCl_3$);

HRMS (–ESI) *m/z* Found $[M–H]^-$ 460.0214, $[C_{14}H_{14}ClF_7NO_4S]^-$ requires 460.0226, (*δ* = – 2.6 ppm);

Chiral SFC Analysis CHIRAL ART SB (CO_2:^{i}PrOH, 95:5, 2.5 mL min^{-1}, 40 °C) t_R = 12.5 (major), 14.7 (minor) minutes.

2,2,3,3,4,4,4-Heptafluorobutyl (R)-(1-(3-bromophenyl)-4-hydroxybutyl)sulfamate (**7i**)

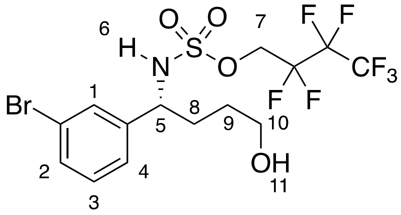

Prepared according to **GP13** using Rh_2(**D**)$_2$•(**2a**)$_2$ as the catalyst and 4-(3-bromophenyl)butan-1-ol (**6i**) as the starting material. In this case the catalyst loading was 3 mol % instead of 1 mol %. Purification by flash column chromatography (SiO_2, 0-20% v/v acetone in $CHCl_3$) afforded the title compound as white plates (24.2 mg, 0.048 mmol, 48%, 86% *ee*).

***R$_f$* value** = 0.42 (20% v/v acetone in $CHCl_3$);

^{1}H NMR (400 MHz, $CDCl_3$) δ 7.43-7.46 (m, 2H, H-1, H-2), 7.23-7.24 (m, 2H, H-3, H-4), 6.48 (br s, 1H, H-6), 4.47 (t, J = 6.8 Hz, 1H, H-5), 4.20-4.37 (m, 2H, H-7a, H-7b), 3.66-3.77 (m, 2H, H-10a, H-10b), 1.90-2.04 (m, 3H, H-8a, H-8b, H-11), 1.58-1.72 (m, 2H, H-9a, H-9b) ppm;

^{13}C NMR (126 MHz, $CDCl_3$) δ 143.1, 131.3, 130.5, 129.7, 125.3, 123.1, 117.5 (qt, $J_{C\text{-}F}$ = 287.4 Hz, 33.4 Hz), 113.2 (tt, $J_{C\text{-}F}$ = 258.4Hz, 31.1 Hz), 105.9-111.1 (m), 64.0 (t, $J_{C\text{-}F}$ = 27.1 Hz), 62.4, 58.6, 34.2, 27.9 ppm;[7]

^{19}F NMR (376 MHz, $CDCl_3$) δ – 81.8 (t, J = 9.3 Hz), – 121.6 - – 121.7 (m), – 128.4 - – 128.5 (m) ppm;

IR (film, cm^{-1}): ν_{max} 3312 (br), 2951 (w), 2926 (w), 2362 (w), 2342 (w), 1597 (w), 1573 (w), 1435 (w), 1356 (m), 1230 (s), 1183 (s), 1128 (m), 1048 (m), 1016 (w), 960 (w), 916 (m), 789 (w), 760 (w);

$[\alpha]_D^{25.0}$ = + 28.9 (c. 1.0, $CHCl_3$);

HRMS (−ESI) *m/z* Found [M−H]$^-$ 503.9726, [$C_{14}H_{14}BrF_7NO_4S$]$^-$ requires 503.9721, (δ = + 1.0 ppm);

Chiral SFC Analysis CHIRAL ART SB (CO_2:MeOH, 96:4, 2.5 mL min^{-1}, 40 °C) t_R = 9.8 (major), 11.2 (minor) minutes.

2,2,3,3,4,4,4-Heptafluorobutyl (R)-(4-hydroxy-1-(3-iodophenyl)butyl)sulfamate (**7j**)

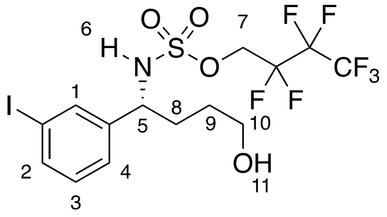

Prepared according to **GP13** using Rh_2(**D**)$_2$•(**2a**)$_2$ as the catalyst and 4-(3-iodophenyl)butan-1-ol (**6j**) as the starting material. In this case the catalyst loading was 3 mol % instead of 1 mol %. Purification by flash column chromatography (SiO_2, 0-20% v/v acetone in $CHCl_3$) afforded the title compound as a colourless oil (16.3 mg, 0.029 mmol, 29%, 88% *ee*).

***R$_f$* value** = 0.40 (20% v/v acetone in $CHCl_3$);

^{1}H NMR (400 MHz, $CDCl_3$) δ 7.63-7.65 (m, 2H, H-1, H-2), 7.26-7.28 (m, 1H, H-4), 7.10 (t, *J* = 7.7 Hz, 1H, H-3), 6.29 (d, *J* = 5.7 Hz, 1H, H-6), 4.45 (q, *J* = 6.7 Hz, 1H, H-5), 4.22-4.38 (m, 2H, H-7a, H-7b), 3.68-3.77 (m, 2H, H-10a, H-10b), 1.98 (q, *J* = 7.0 Hz, 2H, H-8a, H-8b), 1.62-1.68 (m, 3H, H-9a, H-9b, H-11) ppm;

^{13}C NMR (126 MHz, $CDCl_3$) δ 143.1, 137.3, 135.6, 130.7, 126.0, 117.5 (qt, J_{C-F} = 287.8 Hz, 33.4 Hz), 113.3 (tt, J_{C-F} = 258.4 Hz, 31.0 Hz), 106.2-111.5 (m), 94.8, 64.0 (t, J_{C-F} = 27.1 Hz), 62.4, 58.5, 34.2, 27.9 ppm;[7]

^{19}F NMR (376 MHz, $CDCl_3$) δ – 80.8 (t, *J* = 9.3 Hz), – 120.6 - – 120.7 (m), – 127.4 - – 127.5 (m) ppm;

IR (film, cm^{-1}): ν_{max} 3300 (br), 2926 (w), 1568 (w), 1450 (m), 1356 (s), 1296 (w), 1229 (s), 1182 (s), 1126 (m), 1050 (m), 1015 (m), 960 (m), 915 (m), 760 (w);

$[\alpha]_D^{25.0}$ = + 31.3 (c. 1.0, $CHCl_3$);

HRMS (−ESI) *m/z* Found $[M-H]^-$ 551.9583, $[C_{14}H_{14}F_7INO_4S]^-$ requires 551.9582, (δ = + 0.2 ppm);

Chiral SFC Analysis CHIRAL ART SB (CO_2:^{i}PrOH, 94.5:5.5, 1.25 mL min^{-1}, 40 °C) t_R = 45.4 (major), 50.2 (minor) minutes.

(R)-3-(1-(((2,2,3,3,4,4,4-Heptafluorobutoxy)sulfonyl)amino)-4-hydroxybutyl)phenyl acetate (**7k**)

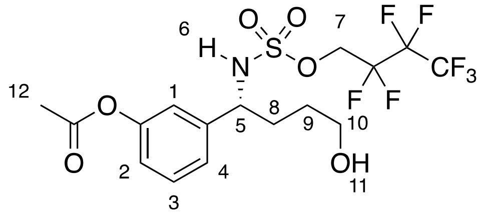

Prepared according to **GP13** using Rh_2(**D**)$_2$•(**2a**)$_2$ as the catalyst and 3-(4-hydroxybutyl)phenyl acetate (**6k**) as the starting material. In this case the catalyst loading was 3 mol % instead of 1 mol %. Purification by flash column chromatography (SiO_2, 0-20% v/v acetone in $CHCl_3$) afforded the title compound as a colourless oil (27.0 mg, 0.056 mmol, 56%, 86% *ee*).

***R*$_f$ value** = 0.49 (30% v/v acetone in $CHCl_3$);

^{1}H NMR (700 MHz, $CDCl_3$) δ 7.37 (t, *J* = 7.9 Hz, 1H, H-3), 7.17 (d, *J* = 7.7 Hz, 1H, H-4), 7.08 (br s, 1H, H-1), 7.03 (dd, *J* = 8.1 Hz, 1.9 Hz, 1H, H-2), 6.37 (d, *J* = 5.5 Hz, 1H, H-6), 4.51 (q, *J* = 6.3 Hz, 1H, H-5), 4.28 (q, *J* = 12.7 Hz, 1H, H-7a), 4.21 (q, *J* = 12.7 Hz, 1H, H-7b), 3.65-3.71 (m, 2H, H-10), 2.30 (s, 3H, H-12), 1.95-2.04 (m, 2H, H-8a, H-8b), 1.88 (br s, 1H, H-11), 1.64-1.68 (m, 1H, H-9a), 1.56-1.61 (m, 1H, H-9b) ppm;

^{13}C NMR (126 MHz, $CDCl_3$) δ 169.7, 151.1, 142.4, 130.1, 124.3, 121.3, 119.9, 117.5 (qt, J_{C-F} = 287.6 Hz, 33.6 Hz), 113.3 (tt, J_{C-F} = 258.4 Hz, 31.0 Hz), 105.9-111.0 (m), 64.0 (t, J_{C-F} = 27.2 Hz), 62.3, 58.7, 33.8, 28.2, 21.2 ppm;[7]

^{19}F NMR (376 MHz, $CDCl_3$) δ – 81.8 (t, *J* = 9.3 Hz), – 121.6 - – 121.7 (m), – 128.4 - – 128.5 (m) ppm;

IR (film, cm^{-1}): ν_{max} 3292 (br), 2926 (w), 2360 (w), 1767 (m), 1738 (m), 1612 (w), 1592 (w), 1448 (w), 1450 (w), 1372 (s), 1181 (s), 1126 (m), 1047 (m), 1015 (m), 958 (m), 914 (w), 801 (m), 759 (m), 701 (w), 533 (m);

$[\alpha]_D^{25.0}$ = + 22.4 (c. 1.0, $CHCl_3$);

HRMS (−ESI) *m/z* Found $[M-H]^-$ 484.0671, $[C_{16}H_{17}F_7NO_6S]^-$ requires 484.0670, (δ = + 0.2 ppm);

Chiral SFC Analysis CHIRAL ART SC (CO_2:^{i}PrOH, 96:4, 2.5 mL min^{-1}, 40 °C) t_R = 15.6 (major), 17.7 (minor) minutes.

2,2,3,3,4,4,4-Heptafluorobutyl (R)-(4-hydroxy-1-(3-isopropylphenyl)butyl)sulfamate **(7l)**

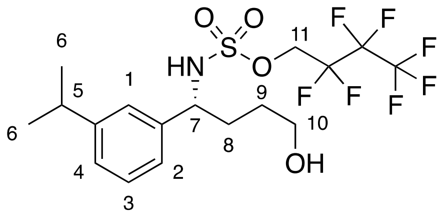

Prepared according to **GP13** using Rh_2(**D**)$_2$•(**2a**)$_2$ as the catalyst and 4-(3-isopropylphenyl)butan-1-ol (**6l**) as the starting material. Purification by flash column chromatography (SiO_2, 0-15% v/v acetone in $CHCl_3$) afforded the title compound as a white powder (23.7 mg, 0.050 mmol, 50%, 87% *ee*).

***R$_f$* value** = 0.44 (20% v/v acetone in $CHCl_3$);

^{1}H NMR (500 MHz, $CDCl_3$) δ 7.29 (t, *J* = 7.6 Hz, 1H, H-3), 7.18 (dt, *J* = 7.6, 1.5 Hz, 1H, H-4), 7.14 – 7.09 (m, 2H, H-1, H-2), 6.01 (d, *J* = 7.2 Hz, 1H, NH), 4.46 (q, *J* = 7.2 Hz, 1H, H-7), 4.18 – 4.09 (m, 1H, H-11a), 4.09 – 4.00 (m, 1H, H-11b), 3.75 – 3.64 (m, 2H, H-10a, H-10b), 2.90 (hept, *J* = 6.9 Hz, 1H, H-5), 2.07 – 1.90 (m, 2H, H-8a, H-8b), 1.76 (br s, 1H, OH), 1.72 – 1.55 (m, 2H, H-9a, H-9b), 1.24 (6H, d, *J* = 6.9 Hz, H-6) ppm;

^{13}C NMR (126 MHz, $CDCl_3$) δ 150.0, 140.6, 129.1, 126.4, 124.9, 123.9, 117.5 (qt, $J_{C\text{-}F}$ = 287.1 Hz, 33.4 Hz), 113.2 (tt, $J_{C\text{-}F}$ = 258.3 Hz, 31.2 Hz), 105.7–110.8 (m), 64.0 (t, $J_{C\text{-}F}$ = 27.0 Hz), 62.4, 59.5, 34.2, 33.9, 28.6, 24.0 (d, J = 3.3 Hz) ppm;

^{19}F NMR (376 MHz, $CDCl_3$) δ – 80.9 (t, J = 9.3 Hz), – 120.7 - – 120.9 (m), – 127.5 - – 127.6 (m) ppm;

IR (film, cm^{-1}): ν_{max} 3297 (br, w), 2965 (w), 1450 (w), 1356 (m), 1294 (w), 1229 (s), 1180 (s), 1126 (m), 1051 (m), 1016 (w), 958 (w), 915 (m), 832 (m), 799 (m), 759 (m), 598 (w), 557 (w);

$[\alpha]_D^{25.0}$ = + 13.8 (c. 1.1, $CHCl_3$);

HRMS (−ESI) *m/z* Found $[M-H]^-$ = 468.1074; $[C_{17}H_{21}F_7NO_4S]^-$ requires 468.1085, (δ = – 2.3 ppm);

Chiral SFC Analysis CHIRAL ART SC (CO_2: ^{i}PrOH, 95:5, 2.5 mL min^{-1}, 40 °C) t_R = 18.0 (major), 20.7 (minor) minutes.

2,2,3,3,4,4,4-Heptafluorobutyl (R)-(1-(2-chlorophenyl)-4-hydroxybutyl)sulfamate (**7m**)

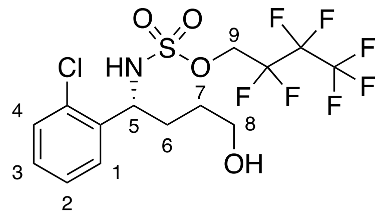

Prepared according to **GP13** using $Rh_2(\mathbf{D})_2\bullet(\mathbf{2a})_2$ as the catalyst and 4-(2-chlorophenyl)butan-1-ol (**6m**) as the starting material. Purification by flash column chromatography (SiO_2, 0-25% v/v acetone in $CHCl_3$) afforded the title compound as a pale-yellow oil (8.3 mg, 0.018 mmol, 18%, 85% *ee*).

***R_f* value** = 0.43 (20% v/v acetone in $CHCl_3$);

^{1}H NMR (500 MHz, $CDCl_3$) δ 7.36-7.40 (m, 2H, H-1, H-4), 7.29 (td, J = 7.5 Hz, 1.4 Hz, 1H, H-2), 7.23-7.25 (m, 1H, H-3), 6.48 (br d, J = 7.2 Hz, 1H, NH), 4.93 (q, J = 7.1 Hz, 1H, H-5), 4.26-4.34

(m, 2H, H-9a, H-9b), 3.69-3.77 (m, 2H, H-8a, H-8b), 2.02 (q, *J* = 7.1 Hz, 2H, H-6a, H-6b), 1.58-1.76 (m, 3H, H-7a, H-7b, OH) ppm;

^{13}C NMR (126 MHz, $CDCl_3$) δ 138.0, 132.3, 130.3, 129.3, 128.5, 127.4, 117.5 (qt, $J_{C\text{-}F}$ = 288 Hz, 33.5 Hz), 113.2 (tt, $J_{C\text{-}F}$ = 258 Hz, 31.1 Hz), 105.7-111.1 (m), 64.0 (t, $J_{C\text{-}F}$ = 27.2 Hz), 62.5, 56.6, 32.6, 28.1 ppm;[7]

^{19}F NMR (376 MHz, $CDCl_3$) δ – 81.8 (t, *J* = 9.2 Hz), – 121.7 - – 121.8 (m), – 128.4 - – 128.6 (m) ppm;

IR (film, cm^{-1}): ν_{max} 3301 (br, w), 2930 (w), 1708 (m), 1475 (m), 1444 (m), 1355 (m) 1294 (m), 1226 (s), 1180 (s), 1124 (m), 1049 (m), 1014 (m), 958 (m);

$[\alpha]_{D}^{25.0}$ = + 16.3 (c. 0.58, $CHCl_3$);

HRMS (−ESI) *m/z* Found [M−H]$^-$ 460.0225; $[C_{14}H_{14}ClF_7NO_4S]^-$ requires 460.0226, (δ = – 0.2 ppm);

HPLC Analysis CHIRALPAK IC (Hexane:^{i}PrOH, 96:4, 1.0 mL min^{-1}, 30 °C) t_R = 15.0 (major), 18.9 (minor) minutes.

2,2,3,3,4,4,4-Heptafluorobutyl (R)-(4-hydroxy-1-(4-methoxyphenyl)butyl)sulfamate (**7n**)

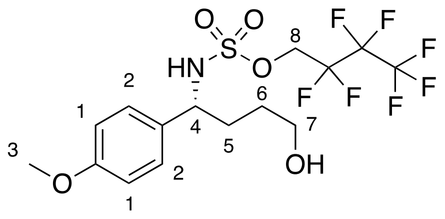

Prepared according to **GP13** using Rh_2(**D**)$_2$•(**2a**)$_2$ as the catalyst and 4-(4-methoxyphenyl)butan-1-ol (**6n**) as the starting material. Purification by flash column chromatography (SiO_2, 0-20% v/v acetone in $CHCl_3$) afforded the title compound as a beige powder (42.0 mg, 0.092 mmol, 92%, 75% *ee*).

***R$_f$* value** = 0.32 (20% v/v acetone in $CHCl_3$);

^{1}H NMR (500 MHz, $CDCl_3$) δ 7.19-7.24 (m, 2H, H-2), 6.87-6.91 (m, 2H, H-1), 6.15 (br d, 1H, J = 6.6 Hz, NH), 4.42 (q, J = 6.9 Hz, 1H, H-4), 4.19 (q, J = 13.1 Hz, 1H, H-8a), 4.10 (q, J = 13.0 Hz, 1H, H-8b), 3.79 (s, 3H, H-3), 3.62-3.73 (m, 2H, H-7a, H-7b), 1.96-2.06 (m, 1H, H-5a), 1.86-1.95 (m, 2H, H-5b, OH), 1.60-1.68 (m, 1H, H-6a), 1.51-1.60 (m, 1H, H-6b) ppm;

^{13}C NMR (126 MHz, $CDCl_3$) δ 159.6, 132.7, 127.9, 117.5 (qt, $J_{C\text{-}F}$ = 286 Hz, 33.5 Hz), 114.4, 113.2 (tt, $J_{C\text{-}F}$ = 259 Hz, 31.0 Hz), 105.5-111.4 (m), 63.9 (t, $J_{C\text{-}F}$ = 27.0 Hz), 62.4, 58.9, 55.4, 33.8, 28.6 ppm;[7]

^{19}F NMR (376 MHz, $CDCl_3$) δ – 81.8 (t, J = 9.2 Hz), – 121.6 - – 121.7 (m), – 128.4 - – 128.6 (m) ppm;

IR (film, cm^{-1}): ν_{max} 3527 (br, w), 3128 (br, w), 2962 (w), 1614 (w), 1516 (m), 1444 (w), 1345 (m), 1300 (m), 1226 (s), 1174 (s), 1128 (s), 1030 (s), 996 (m);

$[\alpha]_D^{25.0}$ = + 15.8 (c. 0.91, $CHCl_3$);

HRMS (+ESI) *m/z* Found $[M+Na]^+$ 480.0686; $[C_{15}H_{18}F_7NNaO_5S]^+$ requires 480.0686, (δ = + 0.0 ppm);

Chiral SFC Analysis CHIRALPAK IG (CO_2:^{i}PrOH, 95.5:4.5, 2.5 mL min^{-1}, 40 °C) t_R = 11.3 (major), 13.5 (minor) minutes.

2,2,3,3,4,4,4-Heptafluorobutyl (R)-(1-(4-chlorophenyl)-4-hydroxybutyl)sulfamate (**7o**)

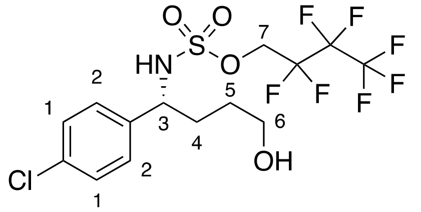

Prepared according to **GP13** using Rh_2(**D**)$_2$•(**2a**)$_2$ as the catalyst and 4-(4-chlorophenyl)butan-1-ol (**6o**) as the starting material. Purification by flash column chromatography (SiO_2, 0-20% v/v acetone in $CHCl_3$) afforded the title compound as a white powder (23.3 mg, 0.050 mmol, 50%, 86% *ee*).

***R_f* value** = 0.35 (20% v/v acetone in $CHCl_3$);

^{1}H NMR (500 MHz, $CDCl_3$) δ 7.34 (d, J = 8.2 Hz, 2H, H-1), 7.24 (d, J = 8.3 Hz, 2H, H-2), 6.47 (br d, J = 5.2 Hz, 1H, NH), 4.48 (q, J = 5.1 Hz, 1H, H-3), 4.18-4.35 (m, 2H, H-7a, H-7b), 3.64-3.76 (m, 2H, H-6a, H-6b), 1.87-2.03 (m, 3H, H-4a, H-4b, OH), 1.55-1.70 (m, 2H, H-5a, H-5b) ppm;

^{13}C NMR (126 MHz, $CDCl_3$) δ 139.3, 134.1, 129.1, 128.0, 117.5 (qt, $J_{C\text{-}F}$ = 288 Hz, 33.5 Hz), 113.2 (tt, $J_{C\text{-}F}$ = 258 Hz, 31.1 Hz), 105.6-111.5 (m), 64.0 (t, $J_{C\text{-}F}$ = 27.1 Hz), 62.4, 58.6, 34.1, 28.1 ppm;[7]

^{19}F NMR (376 MHz, $CDCl_3$) δ – 81.8 (t, J = 9.2 Hz), – 121.6 - – 121.8 (m), – 128.5 - – 128.6 (m) ppm;

IR (film, cm^{-1}): ν_{max} 3525 (br, m), 3302 (br, w), 3144 (br, w), 2927 (w), 1493 (m), 1448 (m), 1355 (m), 1298 (m), 1227 (s), 1177 (s), 1129 (m), 1092 (m), 1046 (m), 1013 (m), 957 (m);

$[\alpha]_D^{25.0}$ = + 34.4 (c 0.91, $CHCl_3$);

HRMS (−ESI) *m/z* Found [M−H]$^-$ = 460.0224; $[C_{14}H_{14}ClF_7NO_4S]^-$ requires 460.0226, (δ = – 0.4 ppm);

HPLC Analysis CHIRALPAK IC (Hexane:^{i}PrOH, 96:4, 1.0 mL min^{-1}, 30 °C) t_R = 12.7 (major), 19.4 (minor) minutes.

2,2,3,3,4,4,4-Heptafluorobutyl (R)-(1-(5-chloro-2-methylphenyl)-4-hydroxybutyl)sulfamate (**7p**)

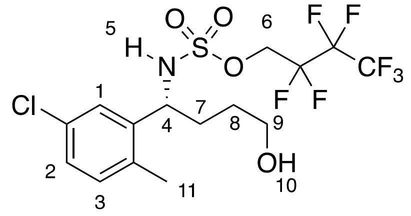

Prepared according to **GP13** using Rh_2(**D**)$_2$•(**2a**)$_2$ as the catalyst and 4-(5-chloro-2-methylphenyl)butan-1-ol (**6p**). In this case the catalyst loading was 3 mol % instead of 1 mol %. Purification by flash column chromatography (SiO_2, 0-19% v/v acetone in $CHCl_3$) afforded the title compound as a colourless oil (16.1 mg, 0.034 mmol, 34%, 88% *ee*).

***R_f* value** = 0.49 (20% v/v acetone in $CHCl_3$);

^{1}H NMR (500 MHz, $CDCl_3$) δ 7.28 (d, J = 2.2 Hz, 1H, H-1), 7.16 (dd, J = 8.2 Hz, 2.2 Hz, 1H, H-2), 7.09 (d, J = 8.2 Hz, 1H, H-3), 6.43 (d, J = 6.4 Hz, 1H, H-5), 4.73 (q, J = 6.9 Hz, 1H, H-4), 4.29 (q, J = 12.6 Hz, 1H, H-6a), 4.19 (q, J = 12.8 Hz, 1H, H-6b), 3.73-3.78 (m, 1H, H-9a), 3.67-3.72 (m, 1H, H-9b), 2.34 (s, 3H, H-11), 1.88-1.98 (m, 3H, H-7a, H-7b, H-10), 1.61-1.77 (m, 2H, H-8a, H-8b) ppm;

^{13}C NMR (126 MHz, $CDCl_3$) δ 141.2, 133.5, 132.4, 132.3, 127.9, 125.9, 117.5 (qt, $J_{C\text{-}F}$ = 287.0 Hz, 33.9 Hz), 113.2 (tt, $J_{C\text{-}F}$ = 259.0 Hz, 31.0 Hz), 105.9-111.0 (m), 64.0 (t, $J_{C\text{-}F}$ = 27.1 Hz), 62.4, 54.9, 33.5, 28.1, 18.7 ppm;[7]

^{19}F NMR (376 MHz, $CDCl_3$) δ – 80.9 (t, J = 9.3 Hz), – 120.8 - – 121.0 (m), – 127.5 - – 127.6 (m) ppm;

IR (film, cm^{-1}): ν_{max} 3295 (br), 2949 (w), 1600 (w), 1486 (w), 1453 (w), 1356 (m), 1294 (w), 1227 (s), 1178 (s), 1125 (m), 1046 (m), 1015 (m), 959 (m), 914 (m), 835 (w), 811 (m), 759 (m), 579 (w), 533 (m);

$[\alpha]_D^{25.0}$ = + 41.0 (c. 1.1, $CHCl_3$);

HRMS (−ESI) *m/z* Found $[M-H]^-$ 474.0384, $[C_{15}H_{16}ClF_7NO_4S]^-$ requires 474.0382, (δ = + 0.4 ppm);

Chiral SFC Analysis CHIRAL ART SB (CO_2:^{i}PrOH, 95:5, 1.25 mL min^{-1}, 40 °C) t_R = 27.0 (major), 29.7 (minor) minutes.

2,2,3,3,4,4,4-Heptafluorobutyl (R)-(1-(2,3-dimethylphenyl)-4-hydroxybutyl)sulfamate (**7q**)

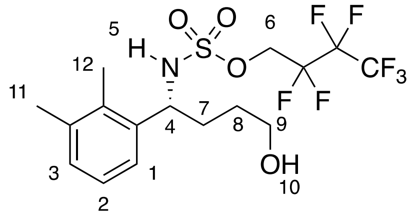

Prepared according to **GP13** using Rh_2(**D**)$_2$•(**2a**)$_2$ as the catalyst and 4-(2,3-dimethylphenyl)butan-1-ol (**6q**) as the starting material. In this case the reaction was run at – 10 °C instead of at – 25 °C and in 1,4-difluorobenzene instead of 1,3-difluorobenzene. Purification by flash column chromatography (SiO_2, 0-20% v/v acetone in $CHCl_3$) afforded the title compound as a white amorphous solid (18.2 mg, 0.040 mmol, 40%, 87% *ee*).

***R_f* value** = 0.46 (20% v/v acetone in $CHCl_3$);

^{1}H NMR (500 MHz, $CDCl_3$) δ 7.09-7.13 (m, 3H, H-1, H-2, H-3), 5.86 (d, *J* = 7.0 Hz, 1H, H-5), 4.88 (q, *J* = 7.1 Hz, 1H, H-4), 4.17 (q, *J* = 12.4 Hz, 1H, H-6a), 4.01 (q, *J* = 12.6 Hz, 1H, H-6b), 3.66-3.74 (m, 2H, H-9a, H-9b), 2.29 (s, 3H, H-12), 2.27 (s, 3H, H-11), 1.88-2.01 (m, 2H, H-7a, H-7b), 1.56-1.72 (m, 3H, H-8a, H-8b, H-10) ppm;

^{13}C NMR (126 MHz, $CDCl_3$) δ 138.9, 137.7, 133.9, 129.6, 126.1, 123.3, 117.5 (qt, *J* = 287.5 Hz, 33.4 Hz), 113.2 (tt, *J* = 258.2 Hz, 31.1 Hz), 105.9-111.0 (m), 63.9 (t, *J* = 26.9 Hz), 62.5, 55.3, 33.6, 28.5, 21.0, 14.7 ppm;[7]

^{19}F NMR (376 MHz, $CDCl_3$) δ – 80.9 (t, *J* = 9.2 Hz), – 120.8 - – 120.9 (m), – 127.6 - – 127.7 (m) ppm;

IR (film, cm^{-1}): ν_{max} 2950 (w), 1462 (w), 1355 (w), 1294 (w), 1228 (s), 1216 (s), 1179 (s), 1125 (w), 1104 (m), 1015 (m), 959 (m), 915 (m), 752 (s), 668 (m), 578 (m), 533 (m);

$[\boldsymbol{\alpha}]_{\mathbf{D}}^{\mathbf{25.0}}$ = + 7.9 (c. 1.1, $CHCl_3$);

HRMS (−ESI) *m/z* Found $[M-H]^-$ 454.0935, $[C_{16}H_{19}F_7NO_4S]^-$ requires 454.0928, (δ = + 1.5 ppm);

Chiral SFC Analysis CHIRAL ART SC (CO_2:^{i}PrOH, 96.5:3.5, 2.5 mL min^{-1}, 40 °C) t_R = 18.5 (major), 23.1 (minor) minutes.

2,2,3,3,4,4,4-Heptafluorobutyl (R)-(1-(4-fluoro-2-methylphenyl)-4-hydroxybutyl)sulfamate (**7r**)

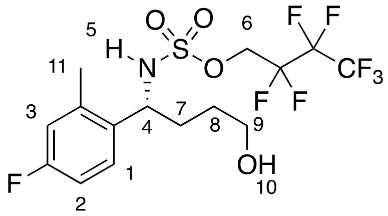

Prepared according to **GP13** using Rh_2(**D**)$_2$•(**2a**)$_2$ as the catalyst and 4-(4-fluoro-2-methylphenyl)butan-1-ol (**6r**) as the starting material. In this case the reaction was run at – 10 °C instead of at – 25 °C and in 1,4-difluorobenzene instead of 1,3-difluorobenzene. Purification by flash column chromatography (SiO_2, 0-20% v/v acetone in $CHCl_3$) afforded the title compound as a colourless oil (23.2 mg, 0.051 mmol, 51%, 85% *ee*).

R R_f **value** = 0.32 (20% v/v acetone in $CHCl_3$);

^{1}H NMR (700 MHz, $CDCl_3$) δ 7.25-7.27 (m, 1H, H-1), 6.92 (td, *J* = 8.2 Hz, 2.0 Hz, 1H, H-2), 6.88 (dd, *J* = 9.6 Hz, 2.0 Hz, 1H, H-3), 6.14 (d, *J* = 6.3 Hz, 1H, H-5), 4.75 (q, *J* = 6.9 Hz, 1H, H-4), 4.25 (q, *J* = 12.7 Hz, 1H, H-6a), 4.16 (q, *J* = 12.8 Hz, 1H, H-6b), 3.73-3.76 (m, 1H, H-9a), 3.68-3.71 (m, 1H, H-9b), 2.38 (s, 3H, H-11), 1.88-1.97 (m, 2H, H-7a, H-7b), 1.59-1.80 (m, 3H, H-8a, H-8b, H-10) ppm;

^{13}C NMR (126 MHz, $CDCl_3$) δ 162.0 (d, J_{C-F} = 246.6 Hz), 137.7 (d, J_{C-F} = 7.8 Hz), 135.0 (d, J_{C-F} = 2.6 Hz), 127.5 (d, J_{C-F} = 8.5 Hz), 117.5 (d, J_{C-F} = 21.1 Hz), 117.5 (qt, J_{C-F} = 287.3 Hz, 33.0 Hz), 113.6 (d, J_{C-F} = 21.3 Hz), 113.2 (tt, J_{C-F} = 258.2 Hz, 31.1 Hz), 105.9-111.0 (m), 63.9 (t, J_{C-F} = 27.0 Hz), 62.4, 54.6, 33.6, 28.2, 19.3 ppm;[7]

^{19}F NMR (376 MHz, $CDCl_3$) δ – 81.8 (t, *J* = 9.2 Hz), – 116.0 (s), – 121.7 - – 121.8 (m), – 128.5 - – 128.6 (m) ppm;

IR (film, cm^{-1}): ν_{max} 3304 (br), 2951 (w), 2928 (w), 1616 (w), 1592 (w), 1502 (m), 1454 (m), 1356 (m), 1283 (w), 1228 (s), 1180 (s), 1126 (s), 1049 (s), 1016 (m), 957 (m), 915 (m), 867 (w), 818 (m), 760 (m);

$[\alpha]_D^{25.0}$ = + 18.8 (c. 0.97, $CHCl_3$);

HRMS (–ESI) *m/z* Found $[M-H]^-$ 458.0682, $[C_{15}H_{16}F_8NO_4S]^-$ requires 458.0678, (δ = + 0.8 ppm);

Chiral SFC Analysis CHIRAL ART SC (CO_2:^{i}PrOH, 97:3, 2.5 mL min^{-1}, 40 °C) t_R = 14.3 (major), 16.4 (minor) minutes.

2,2,3,3,4,4,4-Heptafluorobutyl (R)-(1-(2,5-dimethylphenyl)-4-hydroxybutyl)sulfamate (**7s**)

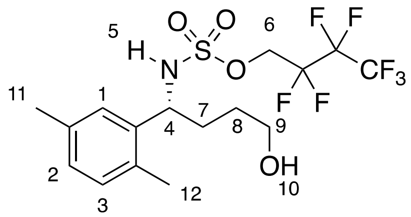

Prepared according to **GP13** using Rh_2(**D**)$_2$•(**2a**)$_2$ as the catalyst and using 4-(2,5-dimethylphenyl)butan-1-ol (**6s**) as the starting material. Purification by flash column chromatography (SiO_2, 0-20% v/v acetone in $CHCl_3$) afforded the title compound as a white amorphous solid (18.6 mg, 0.041 mmol, 41%, 86% *ee*).

***R_f* value** = 0.42 (20% v/v acetone in $CHCl_3$);

^{1}H NMR (500 MHz, $CDCl_3$) δ 7.04-7.06 (m, 2H, H-1, H-3), 7.00 (d, *J* = 7.7 Hz, 1H, H-2), 6.04 (d, *J* = 6.7 Hz, 1H, H-5), 4.75 (q, *J* = 7.2 Hz, 1H, H-4), 4.11 (q, *J* = 12.6 Hz, 1H, H-6a), 4.04 (q, *J* = 12.6 Hz, 1H, H-6b), 3.65-3.75 (m, 2H, H-9a, H-9b), 2.33 (s, 3H, H-12), 2.30 (s, 3H, H-11), 1.92-2.00 (m, 1H, H-7a), 1.85-1.92 (m, 1H, H-7b), 1.80 (br s, 1H, H-10), 1.57-1.73 (m, 2H, H-8a, H-8b) ppm;

^{13}C NMR (126 MHz, $CDCl_3$) δ 138.9, 136.3, 132.0, 130.9, 128.8, 126.1, 117.5 (qt, J_{C-F} = 287.5 Hz, 33.4 Hz), 113.2 (tt, J_{C-F} = 258.4 Hz, 31.0 Hz), 105.9-111.0 (m), 63.9 (t, J_{C-F} = 26.9 Hz), 62.4, 55.1, 33.5, 28.6, 21.1, 18.7 ppm;[7]

^{19}F NMR (376 MHz, $CDCl_3$) δ – 80.9 (t, *J* = 9.1 Hz), – 120.9 - – 121.0 (m), – 127.6 - – 127.7 (m) ppm;

IR (film, cm^{-1}): ν_{max} 3313 (br), 2925 (w), 1504 (w), 1452 (w), 1357 (m), 1295 (w), 1229 (s), 1182 (s), 1126 (m), 1050 (m), 1017 (m), 962 (w), 916 (w), 812 (w), 760 (w);

$[\alpha]_D^{25.0}$ = + 25.1 (c. 0.98, $CHCl_3$);

HRMS (−ESI) *m/z* Found [M−H]$^-$ 454.0924, $[C_{16}H_{19}F_7NO_4S]^-$ requires 454.0928, (δ = – 0.9 ppm);

Chiral SFC Analysis CHIRAL ART SC (CO_2:^{i}PrOH, 96.5:3.5, 2.5 mL min^{-1}, 40 °C) t_R = 13.1 (major), 14.6 (minor) minutes.

2,2,3,3,4,4,4-Heptafluorobutyl (R)-(1-(2,4-difluorophenyl)-4-hydroxybutyl)sulfamate (**7t**)

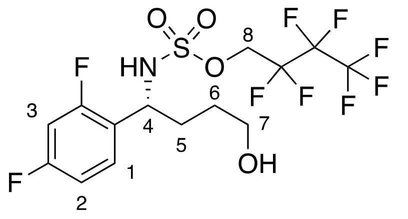

Prepared according to **GP13** using Rh_2(**D**)$_2$•(**2a**)$_2$ as the catalyst and 4-(2,4-difluorophenyl)butan-1-ol (**6t**) the starting material. Purification by flash column chromatography (SiO_2, 0-15% v/v acetone in $CHCl_3$) afforded the title compound as a pale-yellow oil (13.5 mg, 0.029 mmol, 29%, 89% *ee*).

***R_f* value** = 0.32 (20% v/v acetone in $CHCl_3$);

^{1}H NMR (500 MHz, $CDCl_3$) δ 7.26-7.32 (m, 1H, H-1), 6.86-6.91 (m, 1H, H-2), 6.80-6.86 (m, 1H, H-3), 6.22 (br d, *J* = 7.6 Hz, 1H, NH), 4.68 (q, *J* = 7.8 Hz, 1H, H-4), 4.25-4.41 (m, 2H, H-8a, H-8b), 3.68-3.76 (m, 2H, H-7a, H-7b), 1.91-2.10 (m, 2H, H-5a, H-5b), 1.54-1.73 (m, 3H, H-6a, H-6b, OH) ppm;

^{13}C NMR (126 MHz, $CDCl_3$) δ 162.7 (dd, J_{C-F} = 250 Hz, 12.3 Hz), 160.4 (dd, J_{C-F} = 249 Hz, 11.9 Hz), 129.7 (dd, J_{C-F} = 9.3 Hz, 6.3 Hz), 123.8 (dd, J_{C-F} = 12.8 Hz, 3.3 Hz), 117.5 (qt, J_{C-F} = 287 Hz, 33.5 Hz), 113.2 (tt, J_{C-F} = 258 Hz, 31.1 Hz), 111.8 (dd, J_{C-F} = 21.3 Hz, 2.8 Hz), 105.8-111.2 (m), 104.6 (t, J_{C-F} = 25.6 Hz), 64.0 (t, J_{C-F} = 27.2 Hz), 62.3, 54.6, 32.9, 28.3 ppm;[7]

^{19}F NMR (376 MHz, $CDCl_3$) δ – 80.9 (t, J = 9.2 Hz), – 110.1 (d, J = 7.7 Hz), – 114.7 (d, J = 7.7 Hz), – 120.8 - – 120.9 (m), – 127.5 - – 127.6 (m) ppm;

IR (film, cm^{-1}): ν_{max} 3306 (br, w), 2953 (w), 1620 (m), 1606 (m), 1506 (m), 1433 (m), 1355 (m), 1289 (m), 1227 (s), 1178 (s), 1125 (m), 1103 (m), 1044 (m), 1014 (m), 965 (s);

$[\boldsymbol{\alpha}]_{\mathbf{D}}^{\mathbf{25.0}}$ = + 22.3 (c. 0.87, $CHCl_3$);

HRMS (−ESI) *m/z* Found [M−H]$^-$ = 462.0429; $[C_{14}H_{13}F_9NO_4S]^-$ requires 462.0427, (δ = + 0.4 ppm);

HPLC Analysis CHIRALPAK IC (Hexane:^{i}PrOH, 96:4, 1.0 mL min^{-1}, 30 °C) t_R = 11.8 (major), 17.3 (minor) minutes.

2,2,3,3,4,4,4-Heptafluorobutyl (R)-(1-(3-bromo-2-methylphenyl)-4-hydroxybutyl)sulfamate (**7u**)

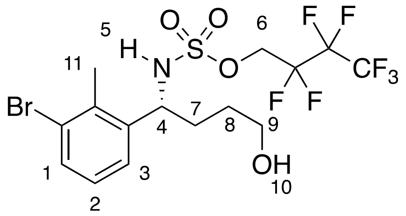

Prepared according to **GP13** using Rh_2(**D**)$_2$•(**2a**)$_2$ as the catalyst and using 4-(3-bromo-2-methylphenyl)butan-1-ol (**6u**) as the starting material . In this case the catalyst loading was 3 mol % instead of 1 mol %. Purification by flash column chromatography (SiO_2, 0-20% v/v acetone in $CHCl_3$) afforded the title compound as a pale-pink amorphous solid (27.7 mg, 0.053 mmol, 53%, 84% *ee*).

***R$_f$* value** = 0.37 (20% v/v acetone in $CHCl_3$);

^{1}H NMR (500 MHz, $CDCl_3$) δ 7.51 (dd, J = 7.9 Hz, 0.9 Hz, 1H, H-1), 7.27 (dd, J = 7.7 Hz, 0.6 Hz, 1H, H-3), 7.08 (t, J = 7.9 Hz, 1H, H-2), 6.50 (d, J = 6.4 Hz, 1H, H-5), 4.84 (q, J = 6.7 Hz, 1H, H-4), 4.26 (q, J = 12.4 Hz, 1H, H-6a), 4.19 (q, J = 12.4 Hz, 1H, H-6b), 3.72-3.77 (m, 1H, H-9a), 3.66-

3.71 (m, 1H, H-9b), 2.47 (s, 3H, H-11), 2.05 (br s, 1H, H-10), 1.89-1.93 (m, 2H, H-7a, H-7b), 1.60-1.73 (m, 2H, H-8a, H-8b) ppm;

^{13}C NMR (126 MHz, $CDCl_3$) δ 141.3, 134.8, 132.3, 127.6, 126.7, 125.0, 117.5 (qt, $J_{C\text{-}F}$ = 288.0 Hz, 32.8 Hz), 113.2 (tt, $J_{C\text{-}F}$ = 259.5 Hz, 31.1 Hz), 105.9-111.0 (m), 64.0 (t, $J_{C\text{-}F}$ = 27.1 Hz), 62.4, 55.9, 33.7, 28.1, 18.8 ppm;[7]

^{19}F NMR (376 MHz, $CDCl_3$) δ – 81.8 (t, J = 9.1 Hz), – 121.7 - – 121.8 (m), – 128.5 - – 128.6 (m) ppm;

IR (film, cm^{-1}): ν_{max} 3304 (br), 2942 (w), 1567 (w), 1439 (m), 1355 (m), 1295 (w), 1227 (s), 1178 (s), 1126 (s), 1047 (m), 1014 (m), 959 (m), 915 (m), 836 (w), 786 (m), 759 (m), 718 (m), 674 (w), 650 (w), 577 (m), 531 (m);

$[\boldsymbol{\alpha}]_{\mathbf{D}}^{\mathbf{25.0}}$ = + 15.7 (c. 1.0, $CHCl_3$);

HRMS (−ESI) *m/z* Found $[M-H]^-$ 517.9877, $[C_{15}H_{16}BrF_7NO_4S]^-$ requires 517.9877, (δ = + 0.0 ppm);

Chiral SFC Analysis CHIRAL ART SB (CO_2:MeOH, 96:4, 2.5 mL min^{-1}, 40 °C) t_R = 10.7 (minor), 13.3 (major) minutes.

2,2,3,3,4,4,4-Heptafluorobutyl (R)-(1-(3-chloro-2-methylphenyl)-4-hydroxybutyl)sulfamate (**7v**)

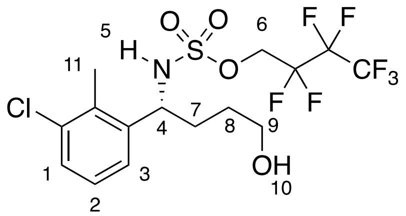

Prepared according to **GP13** using Rh_2(**D**)$_2$•(**2a**)$_2$ as the catalyst and 4-(3-chloro-2-methylphenyl)butan-1-ol (**6v**) as the starting material. In this case the catalyst loading was 3 mol % instead of 1 mol %. Purification by flash column chromatography (SiO_2, 0-20% v/v

acetone in $CHCl_3$) afforded the title compound as an off-white amorphous solid (26.2 mg, 0.055 mmol, 55%, 86% *ee*).

***R_f* value** = 0.36 (20% v/v acetone in $CHCl_3$);

^{1}H NMR (500 MHz, $CDCl_3$) δ 7.31 (dd, *J* = 7.8 Hz, 1.0 Hz, 1H, H-1), 7.22 (dd, *J* = 7.8 Hz, 1.0 Hz, 1H, H-3), 7.15 (t, *J* = 7.9 Hz, 1H, H-2), 6.50 (d, *J* = 6.7 Hz, 1H, H-5), 4.83 (q, *J* = 6.9 Hz, 1H, H-4), 4.26 (q, *J* = 12.7 Hz, 1H, H-6a), 4.17 (q, *J* = 12.8 Hz, 1H, H-6b), 3.73-3.77 (m, *J* = 5.4 Hz, 1H, H-9a), 3.66-3.71 (m, 1H, H-9b), 2.42 (s, 3H, H-11), 2.08 (br s, 1H, H-10), 1.85-1.96 (m, 2H, H-7a, H-7b), 1.59-1.73 (m, 2H, H-8a, H-8b) ppm;

^{13}C NMR (126 MHz, $CDCl_3$) δ 141.3, 135.6, 133.2, 128.9, 127.2, 124.3, 117.5 (q, $J_{C\text{-}F}$ = 287.4 Hz, 33.4 Hz), 113.2 (tt, $J_{C\text{-}F}$ = 258.3 Hz, 31.1 Hz), 105.6-111.0 (m), 64.0 (t, $J_{C\text{-}F}$ = 27.1 Hz), 62.4, 55.6, 33.6, 28.2, 15.5 ppm;[7]

^{19}F NMR (376 MHz, $CDCl_3$) δ – 81.8 (t, *J* = 9.2 Hz), – 121.7 - – 121.8 (m), – 128.5 - – 128.6 (m) ppm;

IR (neat, cm^{-1}): ν_{max} 3325 (br), 2936 (s), 2865 (m), 2361 (w), 2340 (w), 1595 (w), 1569 (w), 1457 (s), 1440 (s), 1379 (m), 1176 (m), 1136 (m), 1060 (s), 1007 (s), 937 (w), 841 (w), 777 (s), 714 (s), 645 (m);

$[\alpha]_D^{25.0}$ = + 17.7 (c. 1.0, $CHCl_3$);

HRMS (−ESI) *m/z* Found $[M-H]^-$ 474.0384, $[C_{15}H_{16}ClF_7NO_4S]^-$ requires 474.0382, (δ = + 0.4 ppm);

Chiral SFC Analysis CHIRAL ART SB (CO_2: ^{i}PrOH, 95:5, 2.5 mL min^{-1}, 40 °C) t_R = 14.8 (minor), 16.5 (major) minutes.

2,2,3,3,4,4,4-Heptafluorobutyl (R)-(4-hydroxy-1-(naphthalen-1-yl)butyl)sulfamate (**7w**)

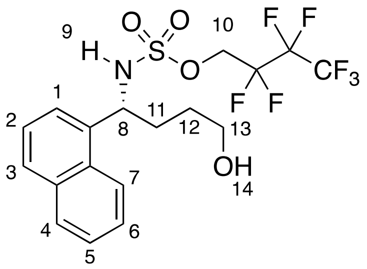

Prepared according to **GP13** using Rh_2(**D**)$_2$•(**2a**)$_2$ as the catalyst and 4-(naphthalen-1-yl)butan-1-ol (**6w**) as the starting material. In this case the catalyst loading was 3 mol % instead of 1 mol %. Purification by flash column chromatography (SiO_2, 0-20% v/v acetone in $CHCl_3$) afforded the title compound as a dark yellow oil (28.1 mg, 0.059 mmol, 59%, 80% *ee*).

***R$_f$* value** = 0.44 (20% v/v acetone in $CHCl_3$);

^{1}H NMR (500 MHz, $CDCl_3$) δ 8.08 (d, J = 8.5 Hz, 1H, H-7), 7.89 (d, J = 8.1 Hz, 1H, H-4), 7.82 (d, J = 8.1 Hz, 1H, H-3), 7.57 (t, J = 7.6 Hz, 1H, H-6), 7.54 (d, J = 7.1 Hz, 1H, H-1), 7.52 (t, J = 7.5 Hz, 1H, H-5), 7.48 (t, J = 7.6 Hz, 1H, H-2), 6.41 (d, J = 6.0 Hz, 1H, H-9), 5.37 (q, J = 6.2 Hz, 1H, H-8), 4.12-4.22 (m, 2H, H-10a, H-10b), 3.69-3.75 (m, 2H, H-13a, H-13b), 2.18 (q, J = 7.0 Hz, 2H, H-11a, H-11b), 1.85 (br s, 1H, H-14), 1.68-1.73 (m, 1H, H-12a), 1.61-1.67 (m, 1H, H-12b) ppm;

^{13}C NMR (126 MHz, $CDCl_3$) δ 136.2, 134.2, 130.4, 129.3, 128.9, 126.9, 126.1, 125.4, 124.1, 122.3, 117.5 (qt, J_{C-F} = 287.4 Hz, 33.4 Hz), 113.2 (tt, J_{C-F} = 258.4 Hz, 31.1 Hz), 105.8-111.0 (m), 64.0 (t, J_{C-F} = 27.0 Hz), 62.5, 55.4, 33.7, 28.3 ppm;[7]

^{19}F NMR (376 MHz, $CDCl_3$) δ – 80.9 (t, J = 9.3 Hz), – 120.8 - – 120.9 (m), – 127.6 - – 127.7 (m) ppm;

IR (film, cm^{-1}): ν_{max} 3301 (br), 3071 (w), 2947 (w), 1514 (w), 1434 (m), 1354 (m), 1295 (w), 1227 (s), 1178 (s), 1125 (s), 1047 (s), 1015 (m), 959 (m), 915 (m), 800 (s), 778 (s), 760 (s);

$[\alpha]_D^{25.0}$ = + 13.6 (c. 1.0, $CHCl_3$);

HRMS (−ESI) *m/z* Found [M−H]$^-$ 476.0773, $[C_{18}H_{18}F_7NO_4S]^-$ requires 476.0772, (δ = – 0.2 ppm);

Chiral SFC Analysis CHIRAL ART SB (CO_2:MeOH, 94:6, 1.25 mL min^{-1}, 40 °C) t_R = 22.3 (minor), 25.6 (major) minutes.

2,2,3,3,4,4,4-Heptafluorobutyl (R)-(4-hydroxy-1-(3-methylthiophen-2-yl)butyl)sulfamate (**7x**)

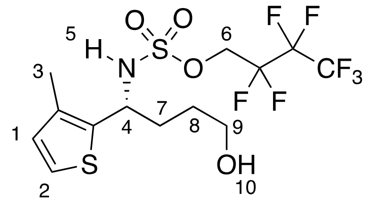

Prepared according to **GP13** using Rh_2(**D**)$_2$•(**2a**)$_2$ as the catalyst and using 4-(3-methylthiophen-2-yl)butan-1-ol (**6x**) as the starting material. Purification by flash column chromatography (SiO_2, 0-20% v/v acetone in $CHCl_3$) afforded the title compound as a brown oil (19.2 mg, 0.043 mmol, 43%, 80% *ee*).

R

R_f **value** = 0.37 (20% v/v acetone in $CHCl_3$);

^{1}H NMR (500 MHz, $CDCl_3$) δ 7.17 (d, *J* = 5.1 Hz, 1H, H-2), 6.79 (d, *J* = 5.1 Hz, 1H, H-1), 5.77 (d, *J* = 6.4 Hz, 1H, H-5), 4.85 (q, *J* = 7.1 Hz, 1H, H-4), 4.26 (q, *J* = 12.6 Hz, 1H, H-6a), 4.06 (q, *J* = 12.6 Hz, 1H, H-6b), 3.66-3.75 (m, 2H, H-9a, H-9b), 2.25 (s, 3H, H-3), 2.06-2.13 (m, 1H, H-7a), 1.91-1.99 (m, 1H, H-7b), 1.74 (br s, 1H, H-10), 1.62-1.70 (m, 2H, H-8a, H-8b) ppm;

^{13}C NMR (126 MHz, $CDCl_3$) δ 137.5, 135.7, 130.4, 123.7, 117.5 (qt, $J_{C\text{-}F}$ = 287.3 Hz, 33.4 Hz), 113.2 (tt, $J_{C\text{-}F}$ = 258.4 Hz, 31.1 Hz), 105.9-111.1 (m), 64.0 (t, $J_{C\text{-}F}$ = 26.9 Hz), 62.3, 52.9, 34.6, 28.8, 13.8 ppm;[7]

^{19}F NMR (376 MHz, $CDCl_3$) δ – 80.8 (t, *J* = 9.2 Hz), – 120.7 - – 120.8 (m), – 127.5 - – 127.6 (m) ppm;

IR (film, cm^{-1}): ν_{max} 3295 (br), 2944 (w), 1434 (m), 1356 (s), 1295 (w), 1226 (s), 1177 (s), 1124 (s), 1107 (s), 1046 (s), 1013 (s), 959 (m), 914 (s), 883 (w), 802 (w), 758 (s), 716 (m), 672 (w), 641 (w), 570 (m), 534 (s);

$[\alpha]_D^{25.0}$ = + 14.0 (c. 1.0, $CHCl_3$);

HRMS (−ESI) *m/z* Found $[M-H]^-$ 446.0344, $[C_{13}H_{15}F_7NO_4S_2]^-$ requires 446.0336, (δ = + 1.8 ppm);

Chiral SFC Analysis CHIRAL ART SC (CO_2:MeOH, 95.5:4.5, 1.25 mL min^{-1}, 40 °C) t_R = 20.0 (major), 23.9 (minor) minutes.

2,2,3,3,4,4,4-Heptafluorobutyl (R)-2-phenylpyrrolidine-1-sulfonate (**8**)

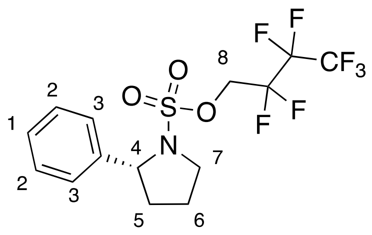

The protocol described by Chan and co-workers was employed.[43] A round-bottomed flask was charged with 2,2,3,3,4,4,4-heptafluorobutyl (*R*)-(4-hydroxy-1-phenylbutyl)sulfamate (**7a**) (150 mg, 0.35 mmol) and PPh_3 (110 mg, 0.42 mL) and evacuated and backfilled with nitrogen three times. THF (9.0 mL) was then added and the reaction mixture cooled to 0 °C. Diisopropylazidodicarboxylate (8.3 μL, 0.42 mmol) was added in a single portion and the reaction mixture was allowed to warm to room temperature overnight. The volatiles were then removed under reduced pressure and the crude residue was purified by flash column chromatography (SiO_2, 0-20% v/v EtOAc in hexane) to afford the title compound as a colourless oil (124 mg, 0.30 mmol, 87%).

***R_f* value** = 0.60 (20% v/v EtOAc in hexane);

^{1}H NMR (400 MHz, $CDCl_3$) δ 7.28-7.35 (m, 5H, H-1, H-2, H-3), 4.92-4.96 (m, 1H, H-4), 4.31-4.44 (m, 2H, H-8a, H-8b), 3.64-3.74 (m, 2H, H-7a, H-7b), 2.38-2.47 (m, 1H, H-5a), 1.95-2.10 (m, 3H, H-5b, H-6a, H-6b) ppm;

^{13}C NMR (126 MHz, $CDCl_3$) δ 141.8, 128.8, 127.8, 126.2, 117.6 (qt, $J_{C\text{-}F}$ = 288.6 Hz, 32.6 Hz), 113.5 (tt, $J_{C\text{-}F}$ = 255.3 Hz, 30.0 Hz), 106.1-110.9 (m), 64.9, 63.8 (t, $J_{C\text{-}F}$ = 27.6 Hz), 50.7, 36.0, 24.6 ppm;[7]

^{19}F NMR (376 MHz, $CDCl_3$) δ – 81.8 (t, J = 9.3 Hz), – 121.6 - – 121.7 (m), – 128.3 - – 128.4 (m) ppm;

IR (film, cm^{-1}): ν_{max} 2963 (m), 1496 (w), 1452 (w), 1379 (m), 1260 (s), 1225 (s), 1173 (s), 1050 (s), 1041 (s), 960 (w), 912 (w), 798 (s), 756 (m), 699 (m), 605 (m), 532 (m);

$[\alpha]_D^{25.0}$ = + 57.2 (c. 0.5, $CHCl_3$);

HRMS (+ESI) *m/z* Found [M+H]$^+$ 410.0655, $[C_{14}H_{15}F_7NO_3S]^+$ requires 410.0655, (δ = + 0.0 ppm)

(R)-2-Phenylpyrrolidine (**9**)

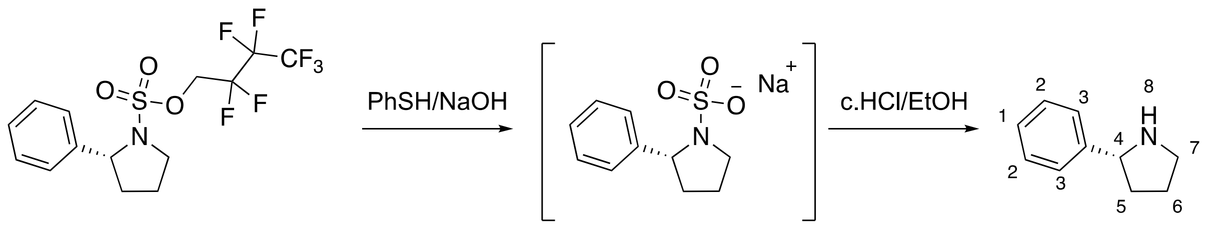

8 **9**

A crimp-top vial was charged with 2,2,3,3,4,4,4-heptafluorobutyl (*R*)-2-phenylpyrrolidine-1-sulfonate (**8**) (107 mg, 0.26 mmol) and freshly-ground NaOH (52.0 mg, 1.3 mmol). The vial was sealed and then evacuated and backfilled with nitrogen three times. MeCN (4.0 mL) and thiophenol (0.13 mL, 1.3 mmol) were added successively and the vial was heated at 90 °C for 50 h during which the intermediate *N*-sulfonate sodium salt formed as a white precipitate. The volatiles were then removed under a stream of compressed air and the crude residue was placed on a silica plug and washed by eluting with 20% v/v Et_2O in hexane. These first organic washings were discarded and the intermediate sodium salt was then obtained by flushing the plug with plenty of EtOH. The ethanolic flushes were collected and the solvent removed under reduced pressure to afford the intermediate sodium salt which was then

transferred to a crimp-top vial.* To the crimp-top vial were then added EtOH (1.5 mL) and c.HCl (1.5 mL). The vial was sealed and the resulting mixture stirred at 80 °C overnight. The reaction mixture was then allowed to cool to room temperature and the EtOH was removed under reduced pressure. The crude was diluted with water (10 mL) and washed with Et_2O (2 x 10 mL). The aqueous fraction was then made basic with 10% NaOH (aq.) and extracted with CH_2Cl_2 (4 x 20 mL). The CH_2Cl_2 extracts were combined, dried ($MgSO_4$) and the solvent removed under reduced pressure to afford the title compound as a yellow oil (12.1 mg, 0.082 mmol, 32% over two steps, 89% ee).

**Note: In a subsequent experiment, the deprotection to form the N-sulfonate salt was complete within 12 h and the sodium salt was collected by an improved protocol: The reaction mixture was allowed to cool to room temperature and then further in ice and the yellow supernatant was discarded. The remaining solid was triturated with ice-cold MeCN (2 x 4 mL) and then dried to afford the pure intermediate as a white powder which was immediately taken forward to the sulfonate deprotection step.*

^{1}H NMR (400 MHz, $CDCl_3$) δ 7.38 (d, J = 7.5 Hz, 2H, H-3), 7.32 (t, J = 7.3 Hz, 2H, H-2), 7.22-7.26 (m, 1H, H-1), 4.15 (t, J = 7.3 Hz, 1H, H-4), 3.41 (br s, 1H, H-8), 3.10-3.25 (m, 1H, H-7a), 3.00-3.06 (m, 1H, H-7b), 2.17-2.25 (m, 1H, H-5a), 1.82-2.01 (m, 2H, H-6a, H-6b), 1.68-1.78 (m, 1H, H-5b) ppm;

^{13}C NMR (101 MHz, $CDCl_3$) δ 143.7, 128.6, 127.2, 126.8, 62.8, 46.8, 34.2, 25.5 ppm;

$[\boldsymbol{\alpha}]_{\mathbf{D}}^{\mathbf{25.0}}$ = + 43.5 (c. 1.0, $CHCl_3$); [reported[44] $[\boldsymbol{\alpha}]_{\mathbf{D}}^{\mathbf{25.0}}$ = + 36.4 (c. 1.1, $CHCl_3$)]

Chiral HPLC Analysis CHIRALPAK AD-H (Hexane + 0.1% Et_2NH:^{i}PrOH, 98:2, 1.0 mL min^{-1}, 30 °C) t_R = 14.8 (major), 17.0 (minor) minutes.

The spectroscopic and spectrophotometric data are in agreement with that reported in the literature.[44]

2,2,3,3,4,4,4-Heptafluorobutyl (R)-(1-phenylbutyl)sulfamate (**11**)

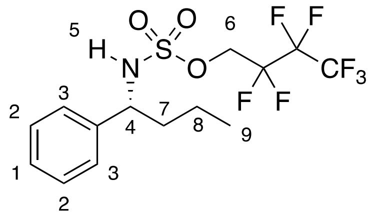

Prepared according to **GP13** using Rh_2(**D**)$_2$•(**2a**)$_2$ as the catalyst and using butylbenzene as the starting material. Purification by flash column chromatography (SiO_2, 0-20% v/v EtOAc in hexane) afforded the title compound as a colourless oil (1.2 mg, 0.0029 mmol, 3%, 28% ee).

***R_f* value** = 0.48 (20% v/v EtOAc in hexane);

^{1}H NMR (500 MHz, $CDCl_3$) δ 7.36-7.39 (m, 2H, H-2), 7.30-7.34 (m, 1H, H-1), 7.26-7.28 (m, 2H, H-3), 4.91 (d, J = 7.3 Hz, 1H, H-5), 4.45 (q, J = 7.4 Hz, 1H, H-4), 4.08-4.22 (m, 2H, H-6a, H-6b), 1.87-1.94 (m, 1H, H-7a), 1.75-1.83 (m, 1H, H-7b), 1.21-1.43 (m, 2H, H-8a, H-8b), 0.92 (t, J = 7.4 Hz, 3H, H-9) ppm;

^{13}C NMR (126 MHz, $CDCl_3$) δ 140.5, 129.1, 128.5, 126.7, 117.5 (qt, $J_{C\text{-}F}$ = 287.5 Hz, 33.6 Hz), 113.2 (tt, $J_{C\text{-}F}$ = 258.3 Hz, 31.0 Hz), 105.9-110.8 (m), 64.0 (t, $J_{C\text{-}F}$ = 27.2 Hz), 59.5, 39.0, 19.3, 13.7 ppm;[7]

^{19}F NMR (376 MHz, $CDCl_3$) δ – 81.8 (t, J = 9.1 Hz), – 121.6 - – 121.7 (m), – 128.4 - – 128.5 (m) ppm;

IR (film, cm^{-1}): ν_{max} 3298 (br), 2965 (w), 2938 (w), 2876 (w), 1497 (w), 1458 (w), 1355 (m), 1298 (w), 1227 (s), 1180 (s), 1125 (s), 1056 (m), 1035 (m), 1015 (m), 958 (m), 915 (s), 837 (w), 804 (m), 760 (s), 700 (s), 561 (m);

HRMS (−ESI) *m/z* Found $[M-H]^-$ 410.0682, $[C_{14}H_{15}F_7NO_3S]^-$ requires 410.0666, (δ = + 3.9 ppm);

Chiral SFC Analysis CHIRAL ART SB (CO_2:^{i}PrOH, 99.5:0.5, 1.25 mL min^{-1}, 40 °C) t_R = 17.4 (minor), 20.2 (major) minutes.

The racemic product was obtained by repeating the procedure above on 0.25 mmol scale with respect to butylbenzene and using $Rh_2(esp)_2$ as the catalyst. The racemic product was obtained in 4% yield.

Methyl (R)-4-(((2,2,3,3,4,4,4-heptafluorobutoxy)sulfonyl)amino)-4-phenylbutanoate (**13**)

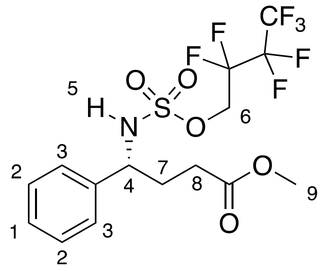

A 4 mL crimp-top vial under an atmosphere of air was charged with 4-phenylbutanoic acid (**12**) (16.4 mg, 0.1 mmol) and Rh_2(**D**)$_2$•(**2a**)$_2$ (2.7 mg, 1 mol %). A solution of 2,2,3,3,4,4,4-heptafluorobutyl sulfamate (33.5 mg 0.12 mmol) in 1,4-difluorobenzene (0.5 mL) was then added *via* syringe. The vial was then cooled to – 10 °C over 10 minutes. Following this, $PhI(OPiv)_2$ (81.3 mg, 0.2 mmol), was added in a single portion. The vial was then capped and stirred at – 10 °C overnight. Sat. aq. thiourea (1 mL) and $CHCl_3$ (1 mL) were then added and the biphasic mixture was stirred vigorously for 5 minutes. The mixture was then allowed to warm to room temperature and stirred for a further 15 minutes. The layers were separated and the aqueous layer extracted with $CHCl_3$ (3 x 1 mL). The combined organic layers were dried ($MgSO_4$), filtered and concentrated *in vacuo*. The crude residue was taken up in MeOH (2.5 mL), following which c.H_2SO_4 (3 drops) was added and the reaction mixture was stirred in a sealed 4.0 mL crimp-top vial at 75 °C under air for 2 hours. The reaction mixture was then allowed to cool to room temperature and concentrated *in vacuo*. $CHCl_3$ (3 mL) was added to the residue and the solution was filtered through $MgSO_4$, eluting with plenty of $CHCl_3$ and the eluent concentrated under reduced pressure. Purification by flash column chromatography

(SiO_2, 0-5% v/v acetone in $CHCl_3$) afforded the title compound as a colourless oil (7.7 mg, 0.017 mmol, 17%, 78% ee).

***R*F value** = 0.28 (30% v/v acetone in hexane);

^{1}H NMR (500 MHz, $CDCl_3$) δ 7.38 (tt, J = 7.3 Hz, 1.6 Hz, 2H, H-2), 7.31-7.35 (m, 1H, H-1), 7.28-7.30 (m, 2H, H-3), 5.83 (d, J = 7.3 Hz, 1H, H-5), 4.53 (q, J = 7.3 Hz, 1H, H-4), 4.22 (q, J = 12.1 Hz, 1H, H-6a), 4.15 (q, J = 12.1 Hz, 1H, H-6b) 3.69 (s, 3H, H-9), 2.38-2.48 (m, 2H, H-8), 2.20-2.27 (m, 1H, H-7a), 2.08-2.14 (m, 1H, H-7b);

^{13}C NMR (101 MHz, $CDCl_3$) δ 174.4, 139.9, 129.2, 128.6, 126.5, 117.8 (qt, J_{C-F} = 287.8 Hz, J_{C-F} = 33.3 Hz), 113.2, (tt, J_{C-F} = 258.5 Hz, J_{C-F} = 31.0 Hz), 105.6-109.0 (m), 64.0 (t, J_{C-F} = 27.1 Hz), 59.3, 52.3, 31.5, 30.7 ppm;[7]

^{19}F NMR (376 MHz, $CDCl_3$) δ – 81.8 (t, J = 9.0 Hz), – 121.7 - – 121.8 (m), – 128.4 - – 128.5 (m) ppm;

IR (film, cm^{-1}): ν_{max} 3286 (w), 1714 (m), 1440 (m), 1355 (m), 1226 (s), 1179 (s), 1124 (m), 1054 (w), 1015 (w), 959 (m), 913 (w), 835 (w), 802 (w), 759 (w), 740 (w), 700 (w);

$[\boldsymbol{\alpha}]_{\mathbf{D}}^{\mathbf{25.0}}$ = + 18.2 (c. 0.5, $CHCl_3$);

HRMS (−ESI) *m/z* Found [M−H]$^-$ 454.0576, $[C_{15}H_{15}F_7NO_5S]^-$ requires 454.0565, (δ = + 2.4 ppm);

Chiral SFC Analysis CHIRAL ART SC (CO_2:MeOH, 98.5:1.5, 2.5 mL min^{-1}, 40 °C), t_R = 5.6 (major), 6.9 (minor) minutes.

2,2,3,3,4,4,4-Heptafluorobutyl (R)-(3-hydroxy-1-phenylpropyl)sulfamate (**14**)

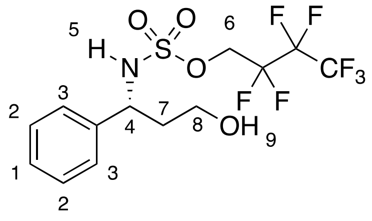

Prepared according to **GP13** using Rh_2(**D**)$_2$•(**2a**)$_2$ as the catalyst and using 3-phenylpropan-1-ol as the starting material. In this case the catalyst loading was 2 mol % instead of 1 mol %. Purification by flash column chromatography (SiO_2, 0-20% v/v acetone in $CHCl_3$) afforded the title compound as an off-white amorphous solid (23.0 mg, 0.056 mmol, 56%, 78% ee).

***R*F value** = 0.44 (50% v/v EtOAc in hexane);

^{1}H NMR (400 MHz, $CDCl_3$) δ 7.29-7.40 (m, 5H, H-1, H-2, H-3), 6.29 (br s, 1H, H-5), 4.75-4.78 (m, 1H, H-4), 4.16-4.31 (m, 2H, H-6a, H-6b), 3.75-3.84 (m, 2H, H-8a, H-8b), 2.11-2.19 (m, 1H, H-7a), 1.99-2.07 (m, 1H, H-7b), 1.82 (br s, 1H, H-9) ppm;

^{13}C NMR (101 MHz, $CDCl_3$) δ 140.0, 129.0, 128.3, 126.6, 117.5 (qt, J_{C-F} = 287.7 Hz, 33.5 Hz), 113.2 (tt, J_{C-F} = 258.3 Hz, 31.1 Hz), 105.3-111.8 (m), 64.0 (t, J_{C-F} = 27.0 Hz), 60.0, 58.1, 38.1 ppm;[7]

^{19}F NMR (376 MHz, $CDCl_3$) δ – 81.8 (t, J = 9.3 Hz), – 121.6 - – 121.7 (m), – 128.4 - – 128.5 (m) ppm;

IR (neat, cm^{-1}): ν_{max} 3501 (m), 3089 (br), 2163 (w), 1977 (w), 1476 (w), 1344 (s), 1223 (s), 1174 (s), 1129 (s), 1046 (s), 1014 (s), 959 (w), 916 (m), 888 (w), 867 (w), 807 (s), 759 (s), 698 (s), 672 (w);

$[\alpha]_D^{25.0}$ = + 32.1 (c. 1.0, $CHCl_3$);

HRMS (−ESI) *m/z* Found [M−H]$^-$ 412.0464, $[C_{13}H_{13}F_7NO_4S]^-$ requires 412.0459, (δ = + 1.2 ppm);

Chiral SFC Analysis CHIRALPAK IG (CO_2:MeOH, 97.5:2.5, 2.5 mL min^{-1}, 40 °C) indicated, t_R = 7.4 (minor), 8.1 (major) minutes

2,2,3,3,4,4,4-heptafluorobutyl (R)-(5-hydroxy-1-phenylpentyl)sulfamate (**15**)

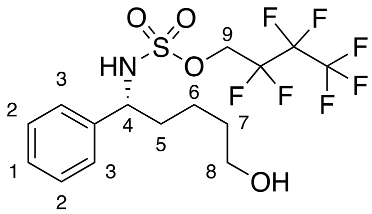

Prepared according to **GP13** using Rh_2(**D**)$_2$•(**2a**)$_2$ as the catalyst and 5-phenylpentan-1-ol as the starting material. Purification by flash column chromatography (*note*—two consecutive purification procedures were carried out: SiO_2, 0-25% v/v acetone in $CHCl_3$ followed by SiO_2, 0-50% v/v EtOAc in hexane) afforded the title compound as a colourless oil (24.2 mg, 0.055 mmol, 55%, 76% *ee*).

***R$_f$* value** = 0.24 (20% v/v acetone in $CHCl_3$);

^{1}H NMR (500 MHz, $CDCl_3$) δ 7.35-7.40 (m, 2H, H-2), 7.30-7.34 (m, 1H, H-1), 7.25-7.29 (m, 2H, H-3), 5.29 (br d, *J* = 7.4 Hz, 1H, NH), 4.45 (q, *J* = 7.4 Hz, 1H, H-4), 4.06-4.23 (m, 2H, H-9a, H-9b), 3.64 (t, *J* = 6.3 Hz, 2H, H-8a, H-8b), 1.91-2.01 (m, 1H, H-5a), 1.80-1.89 (m, 1H, H-5b), 1.67-1.71 (m, 1H, OH), 1.54-1.64 (m, 2H, H-7a, H-7b), 1.42-1.53 (m, 1H, H-6a), 1.30-1.39 (m, 1H, H-6b) ppm;

^{13}C NMR (126 MHz, $CDCl_3$) δ 140.5, 129.2, 128.5, 126.6, 117.5 (qt, J_{C-F} = 287 Hz, 32.9 Hz), 113.2 (tt, J_{C-F} = 258 Hz, 31.2 Hz), 105.4-111.3 (m), 64.0 (t, J_{C-F} = 27.1 Hz), 62.6, 59.7, 36.6, 32.0, 22.5 ppm;[7]

^{19}F NMR (376 MHz, $CDCl_3$) δ – 81.8 (t, *J* = 9.2 Hz), – 121.6 - – 121.8 (m), – 128.4 - – 128.6 (m) ppm;

IR (film, cm^{-1}): ν_{max} 3303 (br, w), 2934 (w), 2866 (w), 1457 (w), 1355 (m), 1229 (s), 1182 (s), 1126 (m), 1052 (m), 1016 (m), 961 (m);

$[\boldsymbol{\alpha}]_{\mathbf{D}}^{\mathbf{25.0}}$ = + 10.6 (c. 0.91 $CHCl_3$);

HRMS (+ESI) *m/z* Found $[M+Na]^+$ = 464.0724; $[C_{15}H_{18}F_7NNaO_4S]^+$ requires 464.073, (δ = – 2.8 ppm);

Chiral SFC Analysis CHIRAL ART SC (CO_2:MeOH, 97.5:2.5, 2.5 mL min^{-1}, 40 °C) t_R = 11.2 (major), 12.4 (minor) minutes.

Unsuccessful Substrates for Enantioselective Intermolecular Amination

Attempted intermolecular amination of a terminal amine substrate: When 4-phenylbutan-1-amine was subjected to **GP13** no intermolecular benzylic amination was observed.

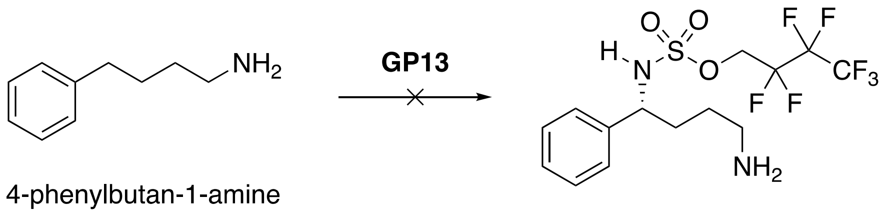

4-phenylbutan-1-amine

Intermolecular amination of an acetyl-protected terminal amine substrate: When *N*-(4-phenylbutyl)acetamide was subjected to **GP13** intermolecular benzylic amination was observed affording 2,2,3,3,4,4,4-heptafluorobutyl (4-acetamido-1-phenylbutyl)sulfamate in 44% isolated yield. However, the product was racemic. The characterisation data is presented below:

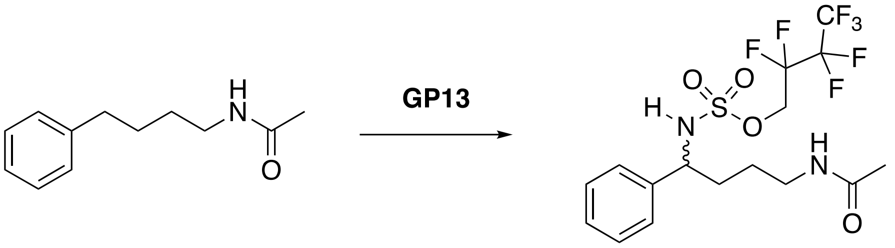

N-(4-phenylbutyl)acetamide

44%, racemic

2,2,3,3,4,4,4-Heptafluorobutyl (4-acetamido-1-phenylbutyl)sulfamate

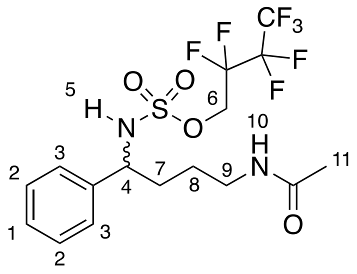

Prepared according to **GP13** using $Rh_2(\mathbf{D})_2\bullet(\mathbf{2a})_2$ as the catalyst and *N*-(4-phenylbutyl)acetamide as the starting material. Purification by flash column chromatography (SiO_2, 0-40% v/v acetone in $CHCl_3$) afforded the title compound as a colourless oil (20.6 mg, 0.044 mmol, 44%, racemic).

***R_f* value** = 0.58 (50% v/v acetone in $CHCl_3$);

^{1}H NMR (500 MHz, $CDCl_3$) δ 7.53 (d, *J* = 7.6 Hz, 1H, H-5), 7.26-7.35 (m, 5H, H-1, H-2, H-3), 6.11 (br s, 1H, H-10), 4.40 (q, *J* = 7.1 Hz, 1H, H-4), 4.13 (q, *J* = 12.8 Hz, 1H, H-6a), 3.94 (q, *J* = 12.8 Hz, 1H, H-6b), 3.22-3.34 (m, 2H, H-9a, H-9b), 1.92-2.01 (m, 4H, H-7a, H-11), 1.76-1.84 (m, 1H, H-7b), 1.64-1.73 (m, 1H, H-8a), 1.46-1.55 (m, 1H, H-8b) ppm;

^{13}C NMR (126 MHz, $CDCl_3$) δ 171.6, 141.2, 129.0, 128.2, 126.6, 117.5 (qt, $J_{C\text{-}F}$ = 287.4 Hz, 33.1 Hz), 113.2 (tt, $J_{C\text{-}F}$ = 258.2 Hz, 31.0 Hz), 105.8-111.2 (m), 63.7 (t, $J_{C\text{-}F}$ = 26.7 Hz), 59.6, 39.3, 33.8, 26.9, 23.1 ppm;

^{19}F NMR (376 MHz, $CDCl_3$) δ – 81.9 (t, *J* = 9.0 Hz), – 121.8 - – 121.9 (m), – 128.6 - – 128.7 (m) ppm;

IR (film, cm^{-1}): ν_{max} 1651 (s), 1538 (m), 1456 (m), 1355 (s), 1294 (w), 1228 (s), 1179 (s), 1124 (m), 1044 (m), 1014 (m), 960 (m), 906 (s), 835 (w), 802 (m), 759 (w), 727 (s), 700 (s);

HRMS (+ESI) *m/z* Found $[M+H]^+$ = 469.1031; $[C_{16}H_{20}F_7N_2O_4S]^+$ requires 469.1027, (δ = + 0.9 ppm);

Chiral SFC Analysis CHIRALPAK IG (CO_2:MeOH, 96.5:3.5, 2.5 mL min^{-1}, 40 °C) t_R = 9.5, 10.3 minutes.

Attempted intermolecular amination of a terminal thiol substrate: When 4-phenylbutane-1-thiol was subjected to **GP13** no intermolecular benzylic amination was observed. Instead, analysis of the reaction mixture by ^{1}H NMR and HRMS suggested the formation of a mixture of products arising from oxidation at sulfur under the reaction conditions.

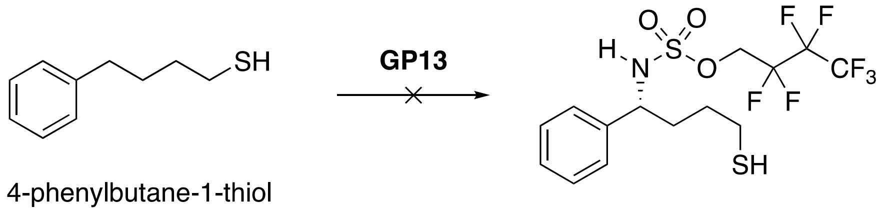

Attempted intermolecular amination at an allylic position: When hex-5-en-1-ol was subjected to **GP13** no intermolecular allylic amination was observed.

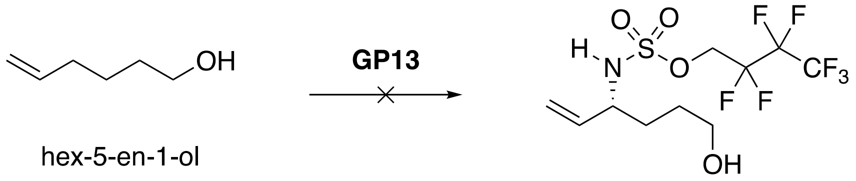

Attempted intermolecular amination at an inert C(*sp*^{3})-H bond: When pentan-1-ol was subjected to **GP13** no intermolecular amination was observed. Even when using an excess of pentan-1-ol (0.5 mmol in place of 0.1 mmol) no intermolecular amination was observed.

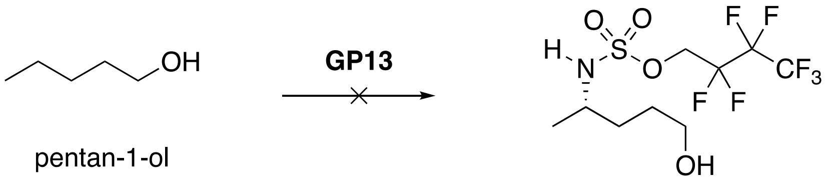

UV-Visible Spectroscopy Studies

UV-visible spectra were recorded on a PerkinElmer Lambda XLS UV-Vis spectrometer using a quartz cuvette with path length of 1 cm.

Sample 1: $Rh_2(esp)_2$ in 1,3-difluorobenzene: A 1 cm path length quartz cuvette was charged with 3.00 ml of 1,3-difluorobenzene, and a background spectrum of this solution was recorded. The 1,3-difluorobenzene was then removed, and 3.00 ml of a 2.0 mM stock solution of $Rh_2(esp)_2$ in 1,3-difluorobenzene was added. A UV-visible absorption spectrum of this solution was then recorded from 350 – 950 nm, with a λ_{max} of 655 nm observed.

Sample 2: $Rh_2(D)_2$•$(2a)_2$ in 1,3-difluorobenzene: A 1 cm path length quartz cuvette was charged with 3.00 ml of 1,3-difluorobenzene, and a background spectrum of this solution was recorded. The 1,3-difluorobenzene was then removed, and 3.00 ml of a 0.5 mM stock solution of $Rh_2(\mathbf{D})_2$•$(\mathbf{2a})_2$ in 1,3-difluorobenzene was added. A UV-visible absorption spectrum of this solution was then recorded from 350 – 950 nm, with a λ_{max} of 536 nm observed. *Note that in this case the absorbance maximum for the $\pi^* \longrightarrow \sigma^*$ transition appears as a shoulder on a higher energy band which may be due to overlap with the UV-visible spectrum of **2a** itself.*

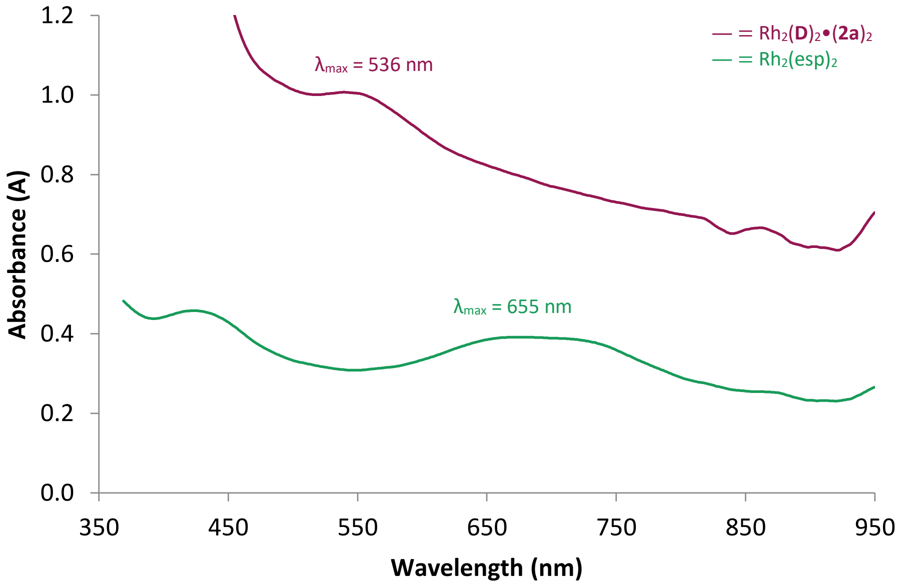

Figure S1. Superposition of the UV-visible absorption spectra of $Rh_2(esp)_2$ (green) and $Rh_2(\mathbf{D})_2$•$(\mathbf{2a})_2$ (purple) in 1,3-difluorobenzene.

***Sample 3: $Rh_2(esp)_2$ + 4-phenylbutan-1-ol (100 equiv.) in 1,3-difluorobenzene**:* A 1 cm path length quartz cuvette was charged with 3.00 ml of 1,3-difluorobenzene, and a background spectrum of this solution was recorded. The 1,3-difluorobenzene was then removed, and 3.00 ml of a 2.0 mM stock solution of $Rh_2(esp)_2$ in 1,3-difluorobenzene containing 4-phenyl-1-butanol (100 equiv.) was added. A UV-visible absorption spectrum of this solution was then recorded from 350 – 950 nm, with a λ_{max} of 612 nm observed.

Sample 4: $Rh_2(D)_2$•$(2a)_2$ + 4-phenylbutan-1-ol (100 equiv.) in 1,3-difluorobenzene: A 1 cm path length quartz cuvette was charged with 3.00 ml of 1,3-difluorobenzene, and a background spectrum of this solution was recorded. The 1,3-difluorobenzene was then removed. A 0.5 mM stock solution of Rh_2(**D**)$_2$•(**2a**)$_2$ and 4-phenyl-1-butanol (100 equiv.) in 1,3-difluorobenzene was prepared and sonicated for approximately 30 seconds to ensure complete dissolution of the components following which 3.00 mL of this solution was introduced into the cuvette. A UV-visible absorption spectrum of this solution was then recorded from 350 – 950 nm, with a λ_{max} of 548 nm observed.

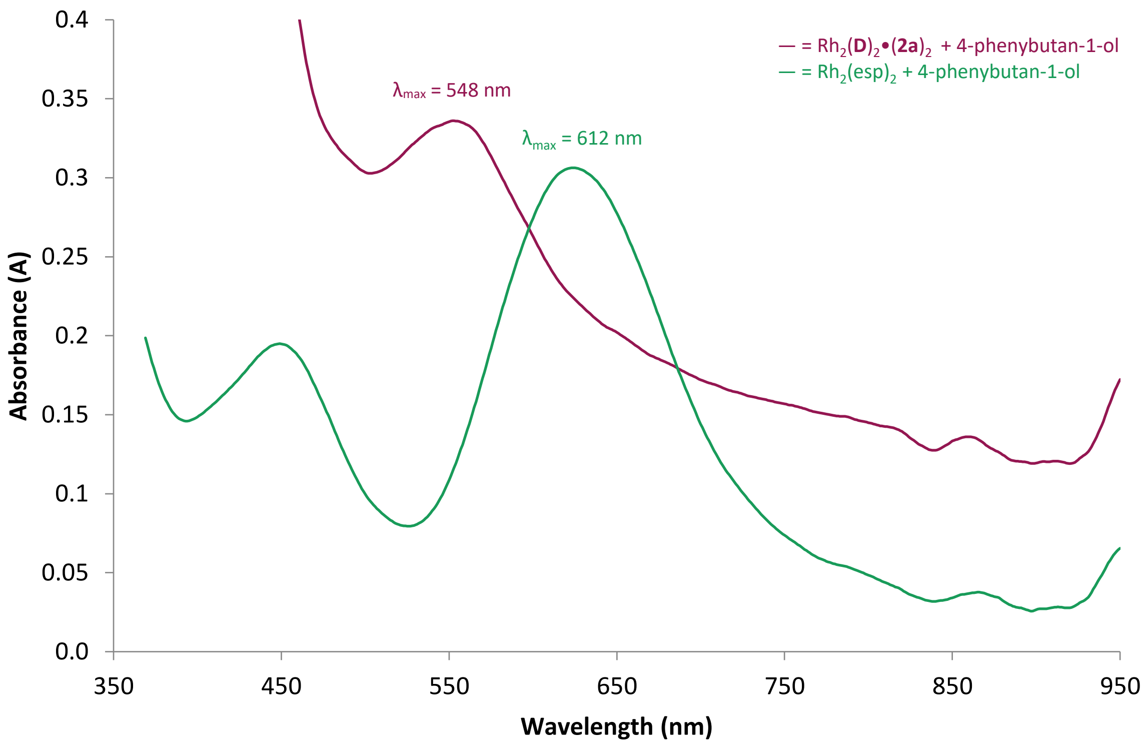

Figure S2. Superposition of the UV-visible absorption spectra of ($Rh_2(esp)_2$ + 100 equiv. of 4-phenylbutan-1-ol) (green) and (Rh_2(**D**)$_2$•(**2a**)$_2$ + 100 equiv. of 4-phenybutan-1-ol) (purple) in 1,3-difluorobenzene.

*Note: Due to solubility issues, the UV-visible absorption spectra of Rh_2(**D**)$_2$•(**2a**)$_2$ were recorded at 0.5 mM to ensure full dissolution of the dimer. When attempting to record the measurements using $Rh_2(esp)_2$ at 0.5 mM the absorbance was very weak so we elected to run UV-visible spectra for this species at the higher concentration of 2.0 mM.*

Analysis: The UV-visible spectrum of Rh_2(**D**)$_2$•(**2a**)$_2$ recorded in 1,3-difluorobenzene shows a higher energy $\pi^* \longrightarrow \sigma^*$ transition compared to that of $Rh_2(esp)_2$ recorded under the same conditions. This suggests axial ligation in the case of Rh_2(**D**)$_2$•(**2a**)$_2$ which we propose is from the quinoline moieties of the chiral cations.[45–47] (For reference, Berry and co-workers observed a λ_{max} of 520 nm for the complex $Rh_2(esp)_2(L)_2$ where L = axially-binding pyridine).[46]

Given that the alcohol substrates could also potentially act as axial ligands, we repeated the UV-visible measurements in the presence of a 100-fold excess of 4-phenylbutan-1-ol (reflecting the initial stoichiometry of the enantioselective intermolecular amination) to investigate any potential interaction between the amination substrates or products and the catalysts. For both catalysts, the curves appeared sharper with better-defined maxima for the $\pi^* \longrightarrow \sigma^*$ transitions. In the case of $Rh_2(esp)_2$ the λ_{max} was shifted to slightly higher energy (from 655 nm to 612 nm) which may suggest a small degree of axial binding by the alcohol. In the case of Rh_2(**D**)$_2$•(**2a**)$_2$ the λ_{max} was practically unchanged upon the introduction of the alcohol (from 536 nm to 548 nm) suggesting minimal change in the axial environment of the complex despite a large excess of a potential axial ligand.

Chiral SFC and Chiral HPLC Traces

2,2,3,3,4,4,4-Heptafluorobutyl (R)-(4-hydroxy-1-phenylbutyl)sulfamate (**7a**)

Chiral SFC Analysis: CHIRAL ART SC (CO_2: ^{i}PrOH, 96:4, 2.5 mL min^{-1}, 40 °C, 210 nm) indicated 90% *ee*, t_R = 12.6 (major), 15.3 (minor) minutes.

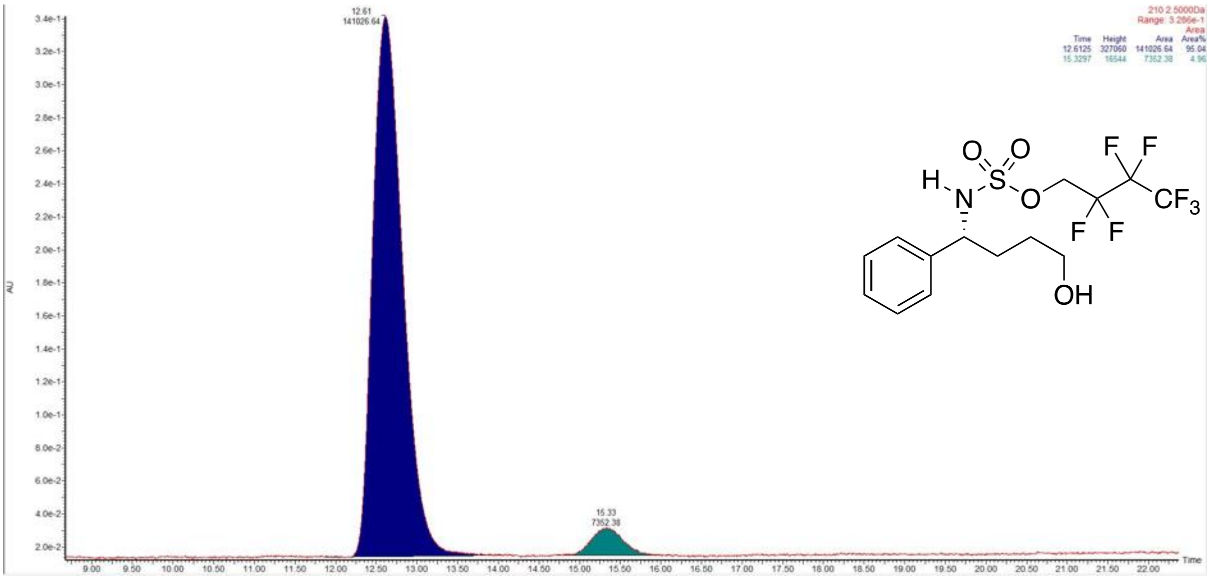

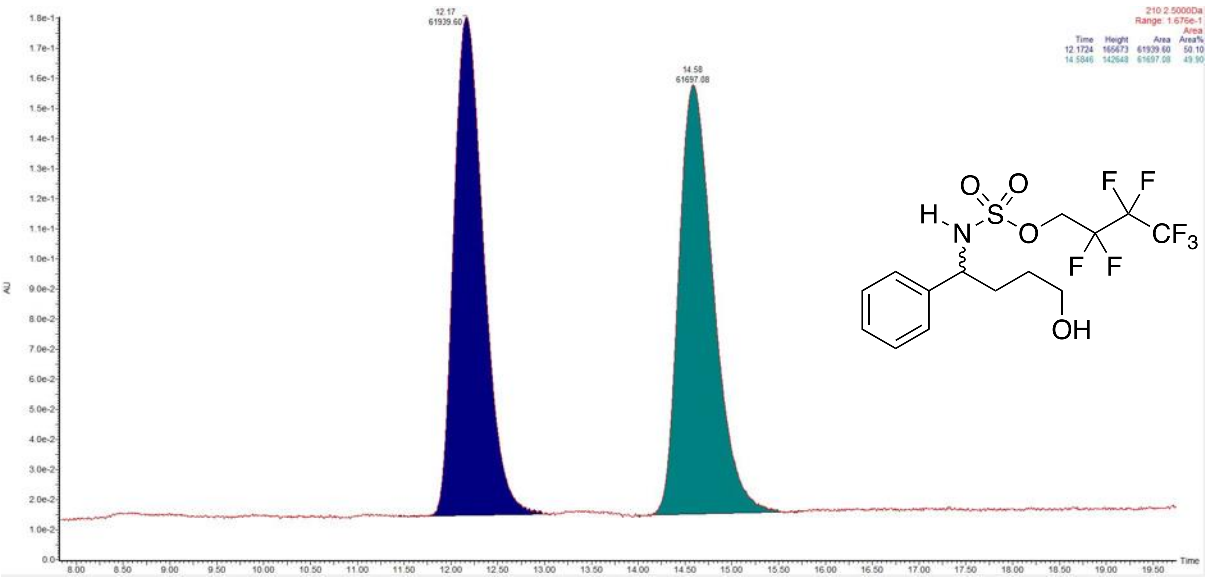

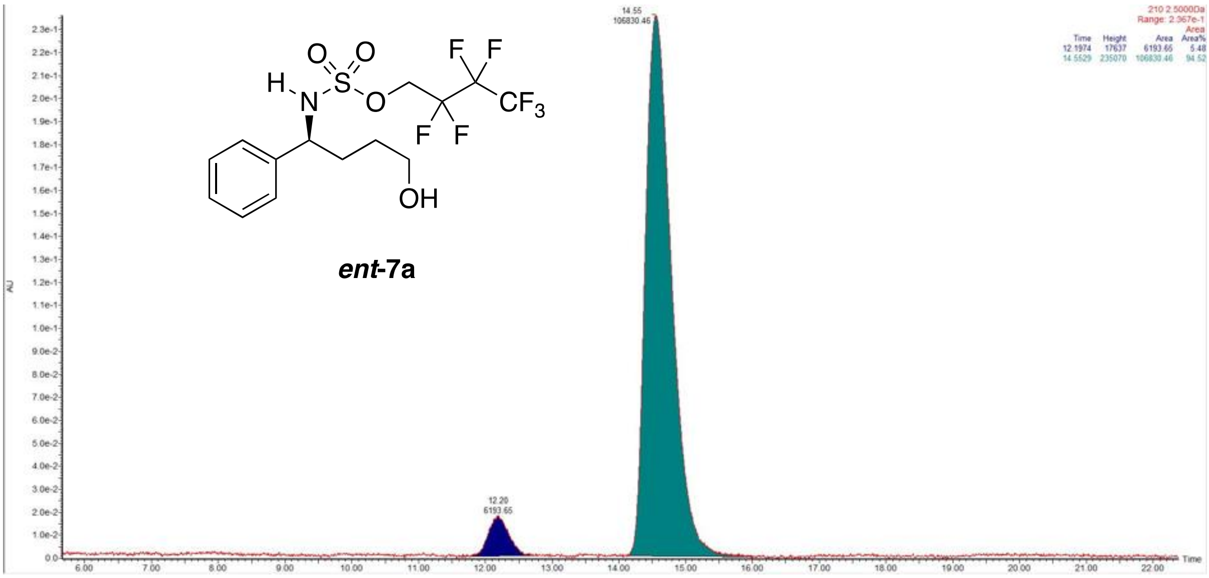

ent-7a
14.55
106830.46
12.20
6193.65
210 2.5000Da
Range: 2.367e-1
Area
Time Height Area Area%
12.1974 17637 6193.65 5.48
14.5529 235070 106830.46 94.52
AU
Time

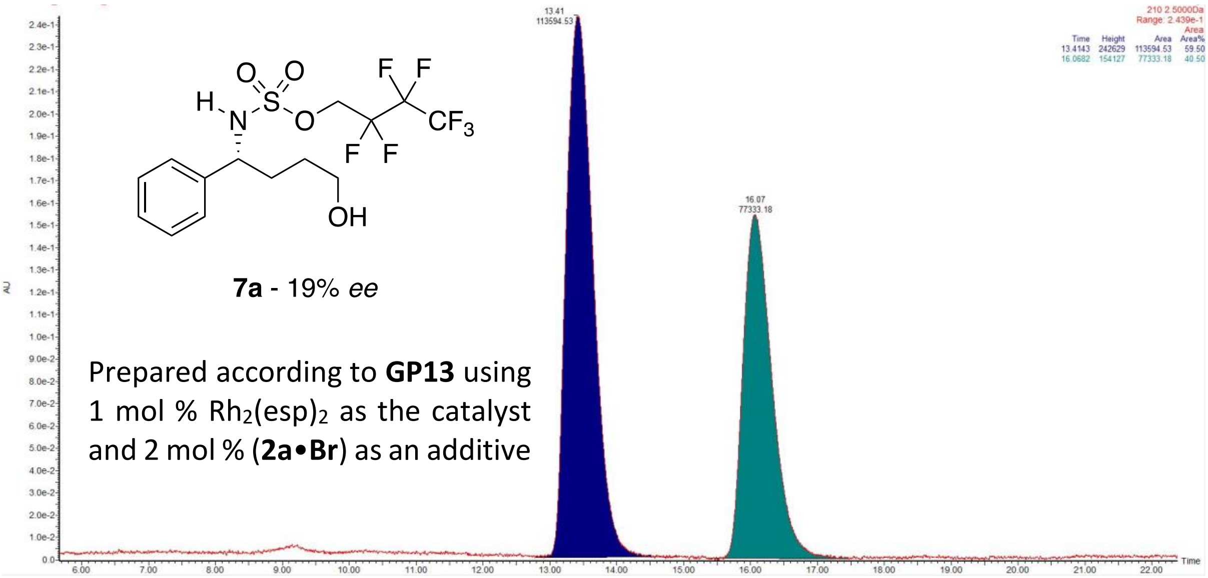

7a - 19% ee
Prepared according to GP13 using 1 mol % Rh2(esp)2 as the catalyst and 2 mol % (2a•Br) as an additive
13.41
113594.53
16.07
77333.18
210 2.5000Da
Range: 2.439e-1
Area
Time Height Area Area%
13.4143 242629 113594.53 59.50
16.0682 154127 77333.18 40.50
AU
Time

Ethyl (R)-3-(1-(((2,2,3,3,4,4,4-heptafluorobutoxy)sulfonyl)amino)-4-hydroxybutyl)benzoate (**7b**)

Chiral SFC Analysis CHIRAL ART SB (CO_2:MeOH, 97:3, 2.5 mL min^{-1}, 40 °C, 228 nm) indicated 86% *ee*, t_R = 13.1 (major), 15.5 (minor) minutes.

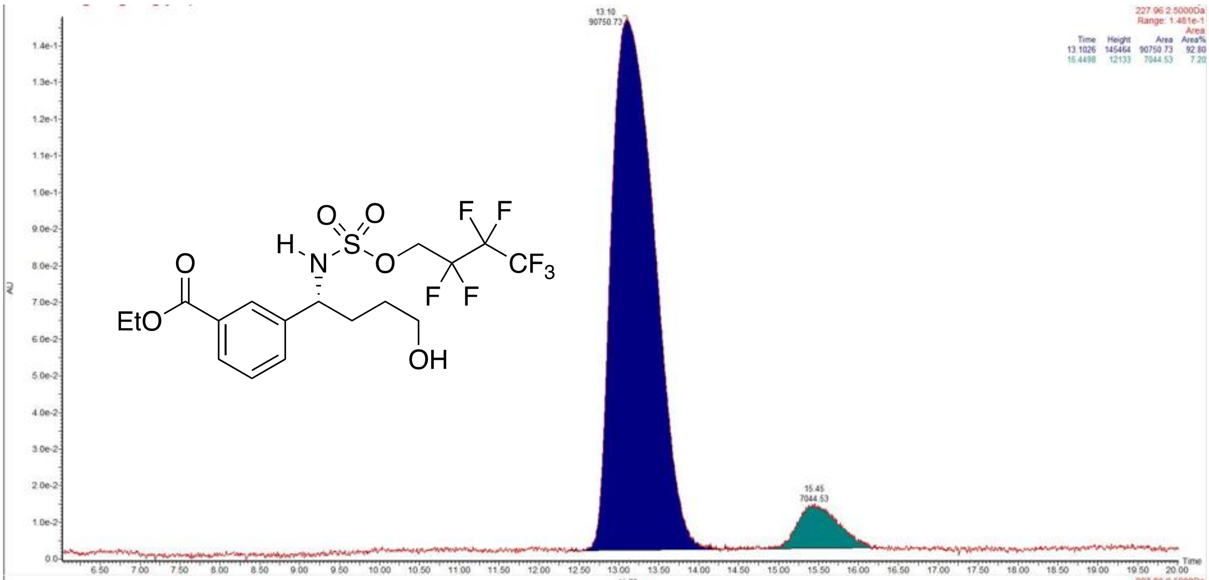

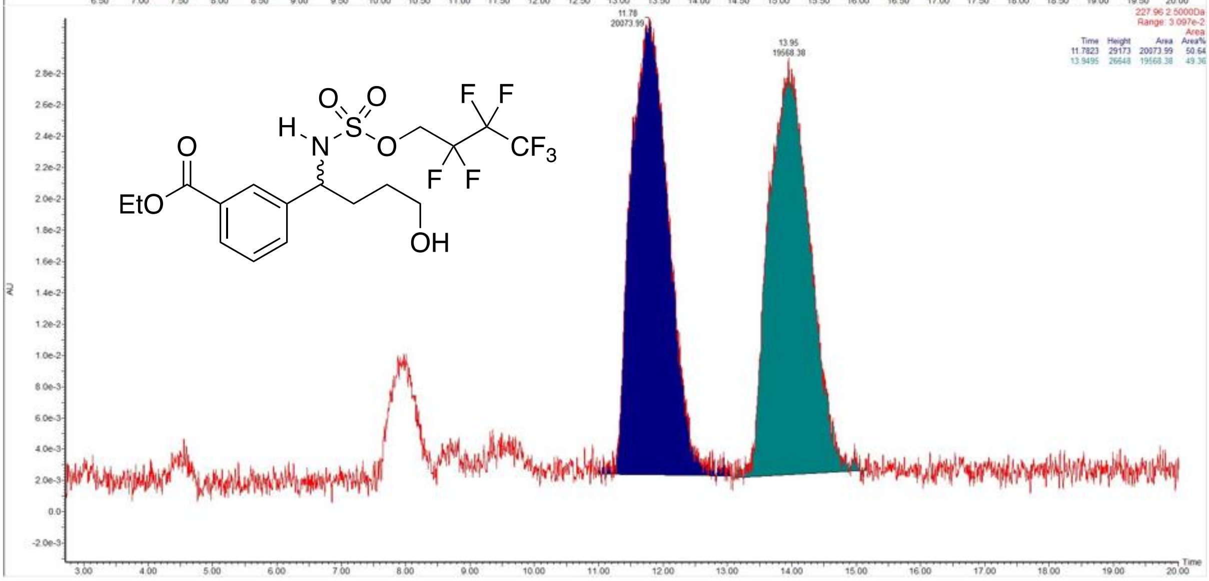

2,2,3,3,4,4,4-Heptafluorobutyl (R)-(4-hydroxy-1-(o-tolyl)butyl)sulfamate (**7c**)

Chiral HPLC Analysis CHIRALPAK IC (Hexane:^{i}PrOH, 96:4, 1.0 mL min^{-1}, 30 °C, 190 nm) indicated 92% *ee*, t_R = 14.9 (major), 22.7 (minor) minutes

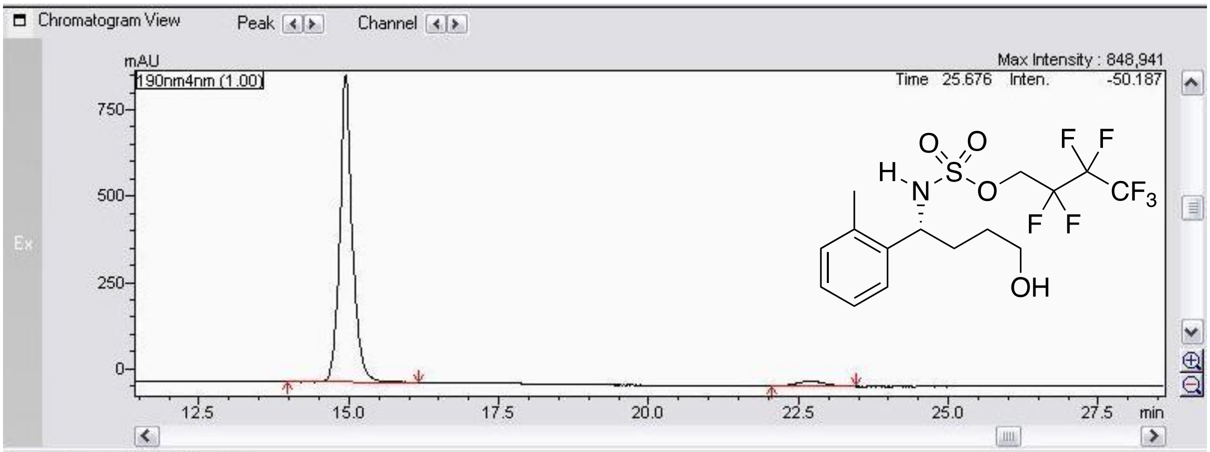

Results View - Peak Table

Peak Table | Compound | Group | Calibration Curve

Peak#	Ret. Time	Area	Height	Area%
1	14.949	12884567	887341	96.227
2	22.684	505154	15345	3.773
Total		13389721	902686	100.000

Extracted 190nm | Ch1 254nm

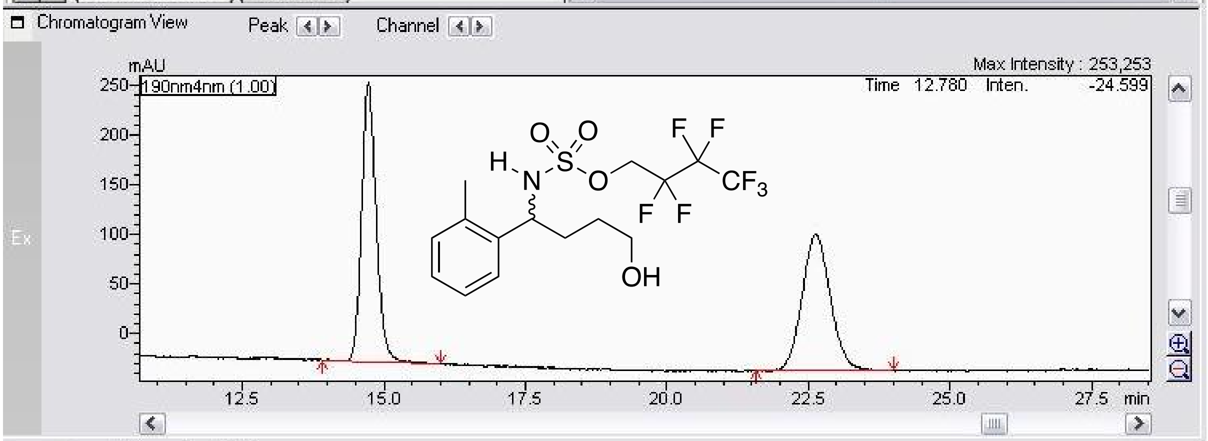

Results View - Peak Table

Peak Table | Compound | Group | Calibration Curve

Peak#	Ret. Time	Area	Height	Area%
1	14.724	4997588	281244	50.916
2	22.615	4817676	138511	49.084
Total		9815264	419755	100.000

Extracted 190nm | Ch1 254nm

2,2,3,3,4,4,4-Heptafluorobutyl (R)-(4-hydroxy-1-(m-tolyl)butyl)sulfamate (**7d**)

Chiral SFC Analysis CHIRAL ART SC (CO_2: ^{i}PrOH, 96:4, 2.5 mL min^{-1}, 40 °C, 208 nm) indicated 84% *ee*, t_R = 12.6 (major), 15.0 (minor) minutes.

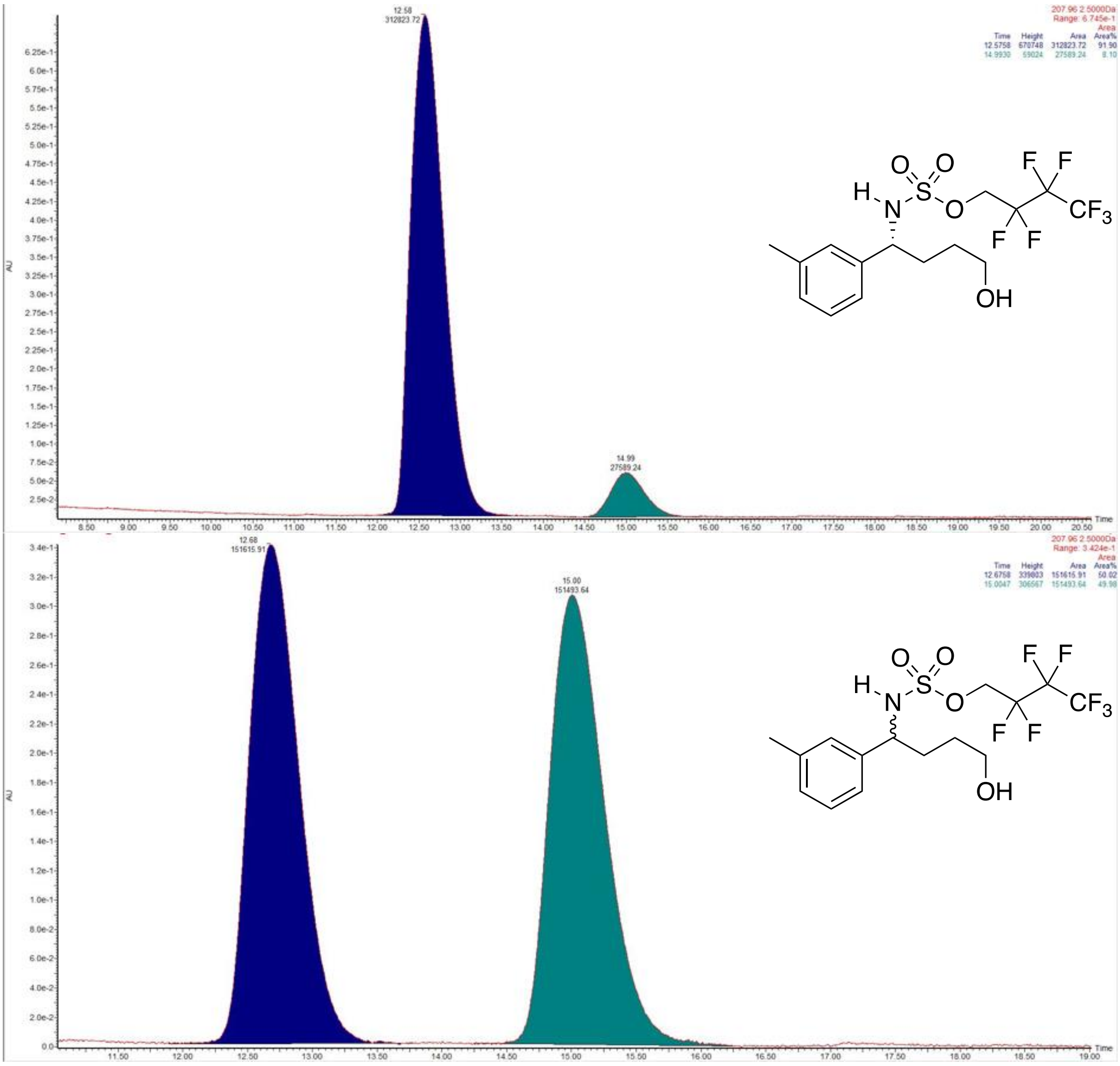

2,2,3,3,4,4,4-Heptafluorobutyl (R)-(1-(3-fluorophenyl)-4-hydroxybutyl)sulfamate (**7e**)

Chiral SFC Analysis CHIRAL ART SB (CO_2: ^{i}PrOH, 96:4, 2.5 mL min^{-1}, 40 °C, 210 nm) indicated 87% *ee*, t_R = 13.1 (major), 15.4 (minor) minutes.

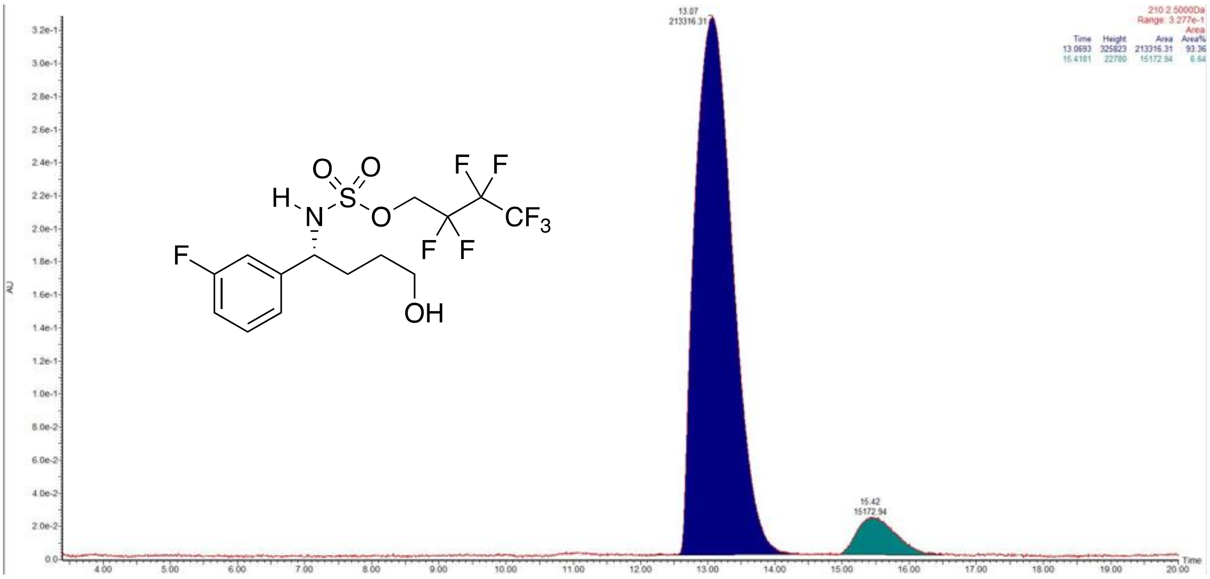

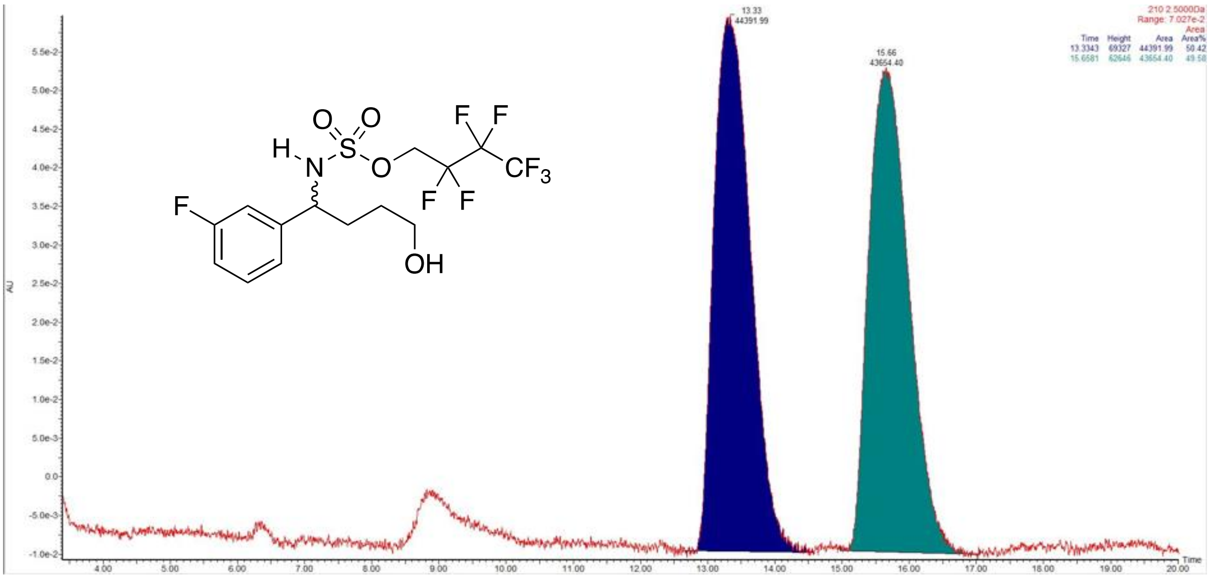

2,2,3,3,4,4,4-Heptafluorobutyl (R)-(1-(2-fluorophenyl)-4-hydroxybutyl)sulfamate (**7f**)

Chiral HPLC Analysis CHIRALPAK IC (Hexane:^{i}PrOH, 96:4, 1.0 mL min^{-1}, 30 °C, 190 nm) indicated 93% *ee*, t$_R$ = 17.6 (major), 26.9 (minor) minutes.

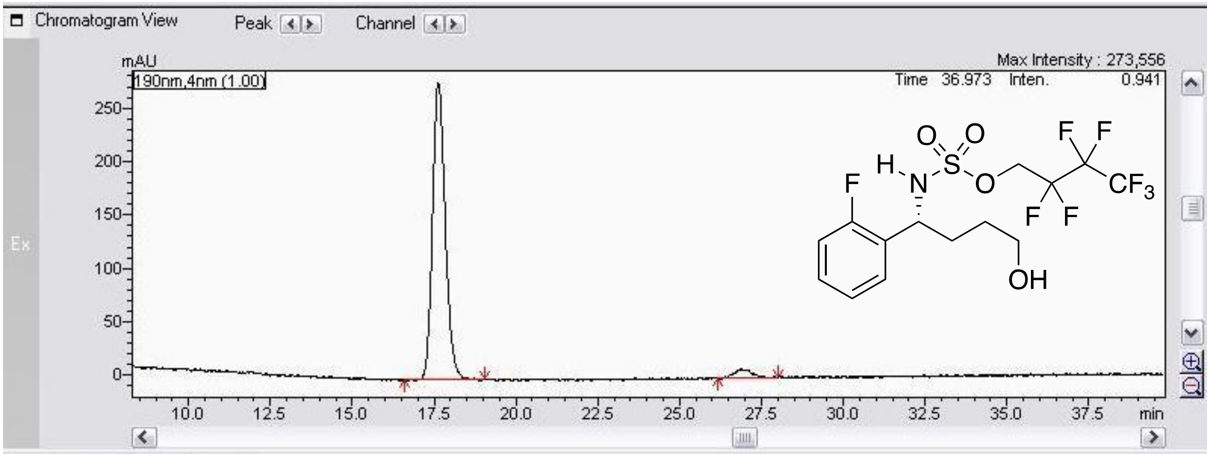

Results View - Peak Table

Peak#	Ret. Time	Area	Height	Area%
1	17.633	7503524	277700	96.622
2	26.869	262370	7629	3.378
Total		7765893	285329	100.000

Extracted 190nm / Ch1 254nm

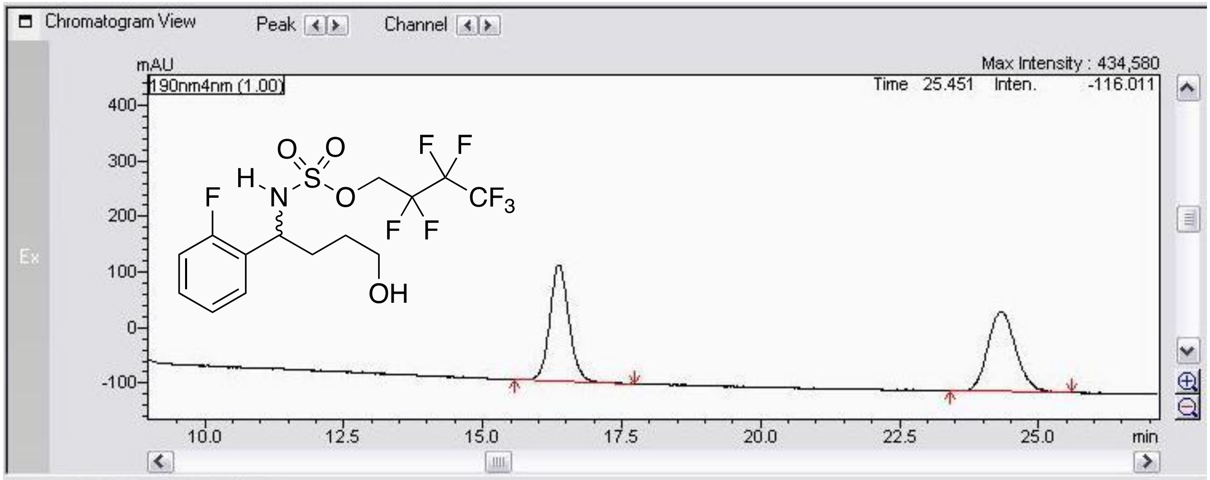

Results View - Peak Table

Peak#	Ret. Time	Area	Height	Area%
1	16.380	4999131	209051	49.792
2	24.320	5040915	144373	50.208
Total		10040046	353424	100.000

Extracted 190nm / Ch1 254nm

2,2,3,3,4,4,4-Heptafluorobutyl (R)-(1-(4-fluorophenyl)-4-hydroxybutyl)sulfamate (**7g**)

Chiral SFC Analysis CHIRAL ART SC (CO_2:^{i}PrOH, 96:4, 2.5 mL min^{-1}, 40 °C, 210 nm) indicated 89% *ee*, t_R = 9.4 (major), 11.1 (minor) minutes.

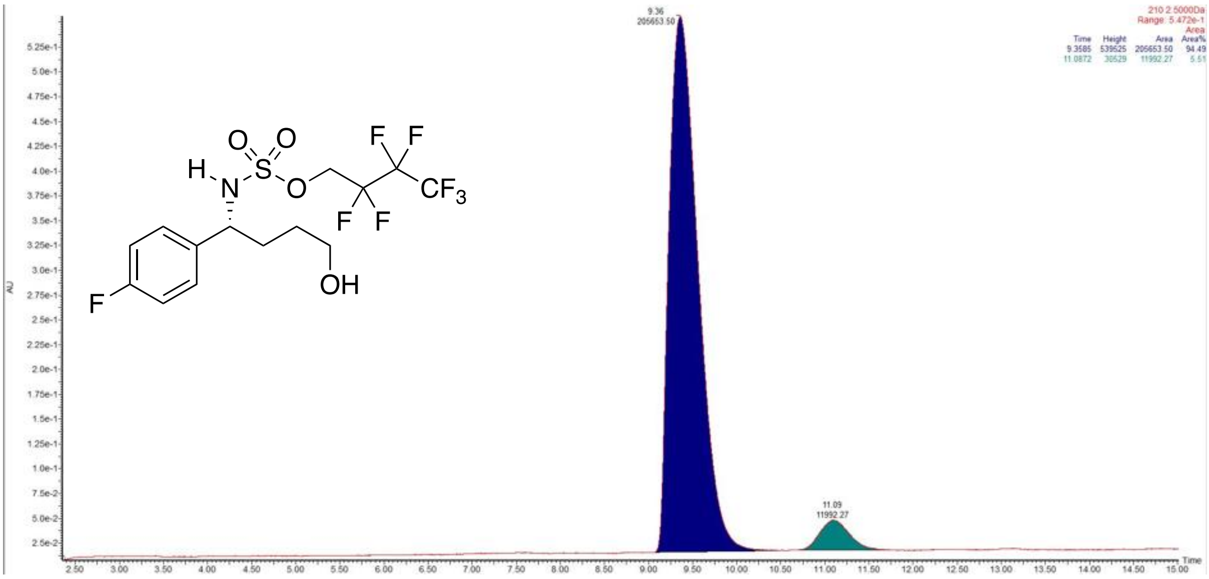

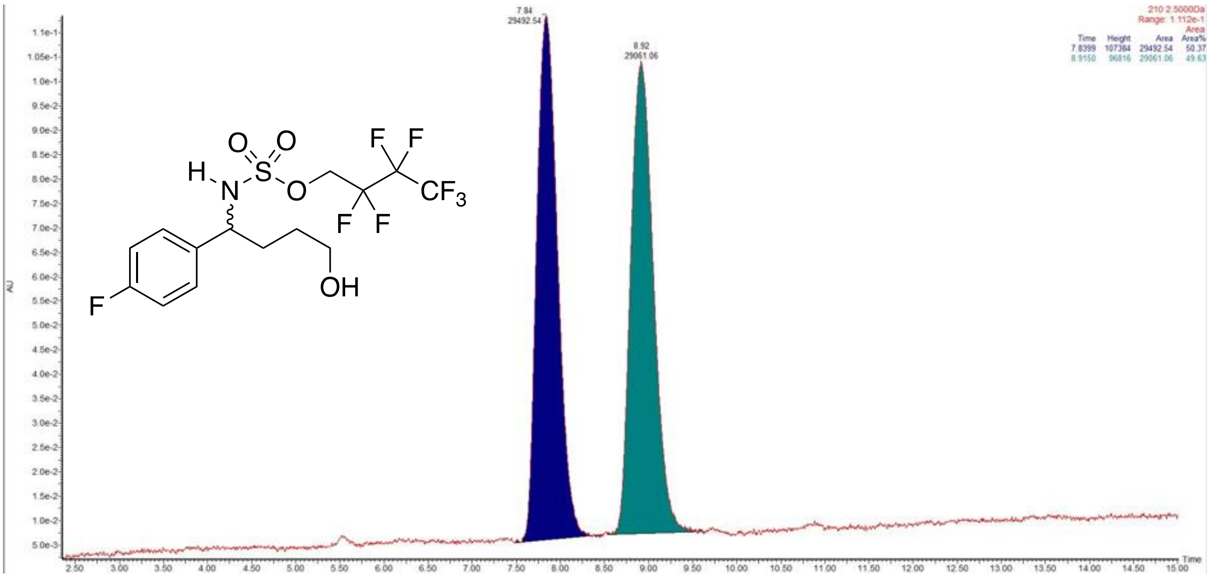

2,2,3,3,4,4,4-Heptafluorobutyl (R)-(1-(3-chlorophenyl)-4-hydroxybutyl)sulfamate (**7h**)

Chiral SFC Analysis CHIRAL ART SB (CO_2: ^{i}PrOH, 95:5, 2.5 mL min^{-1}, 40 °C, 211 nm) indicated 87% *ee*, t_R = 12.5 (major), 14.7 (minor) minutes.

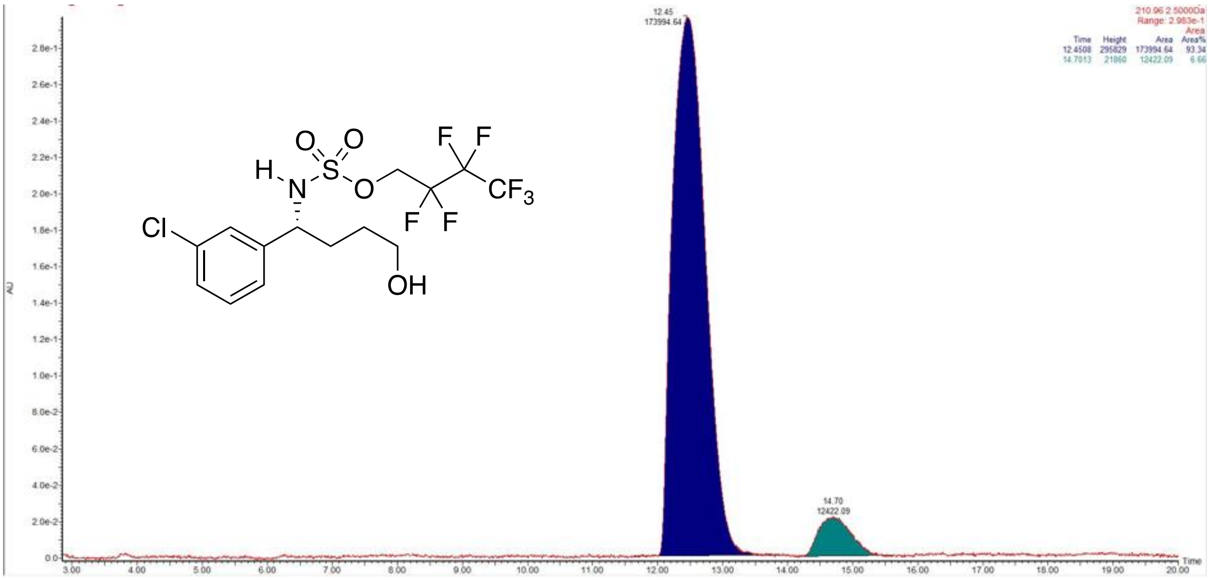

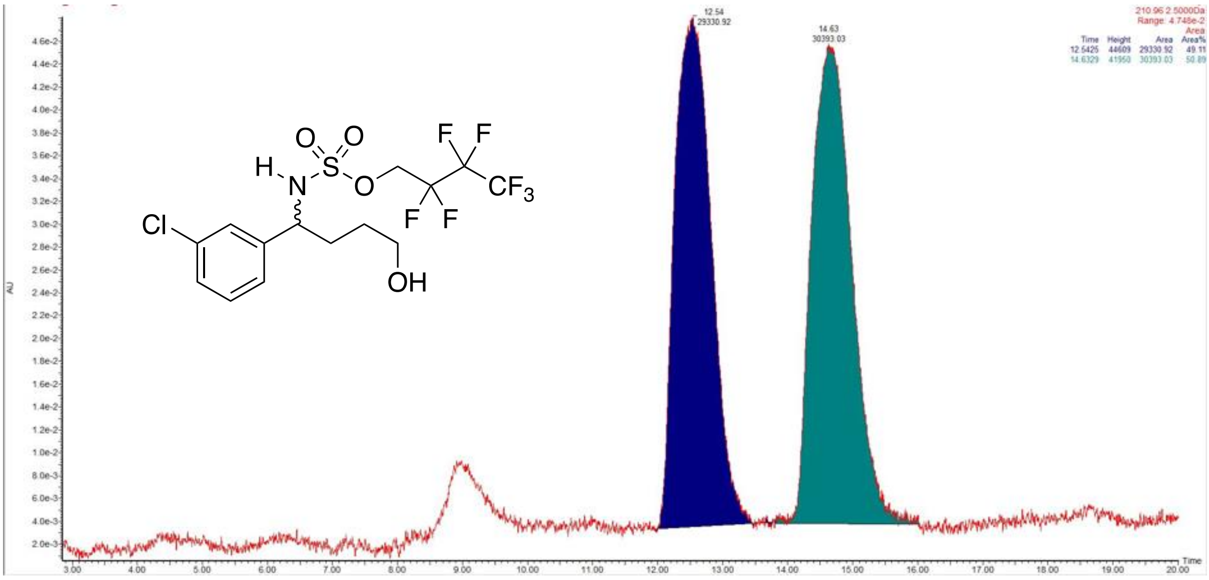

2,2,3,3,4,4,4-Heptafluorobutyl (R)-(1-(3-bromophenyl)-4-hydroxybutyl)sulfamate (**7i**)

Chiral SFC Analysis CHIRAL ART SB (CO_2:MeOH, 96:4, 2.5 mL min^{-1}, 40 °C, 210 nm) indicated 86% *ee*, t_R = 9.8 (major), 11.2 (minor) minutes.

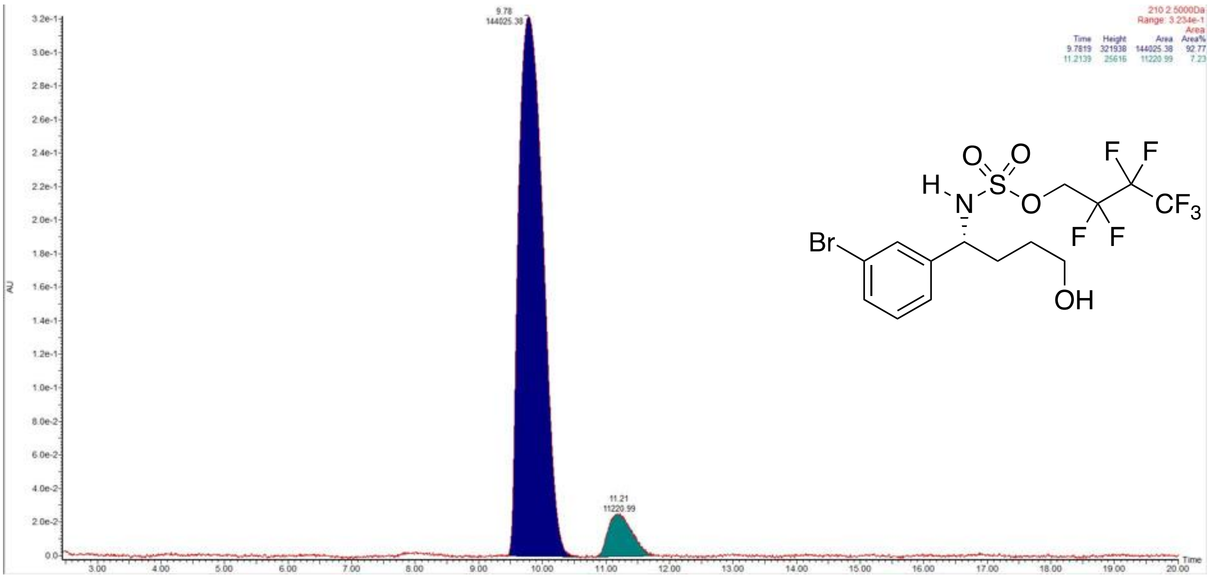

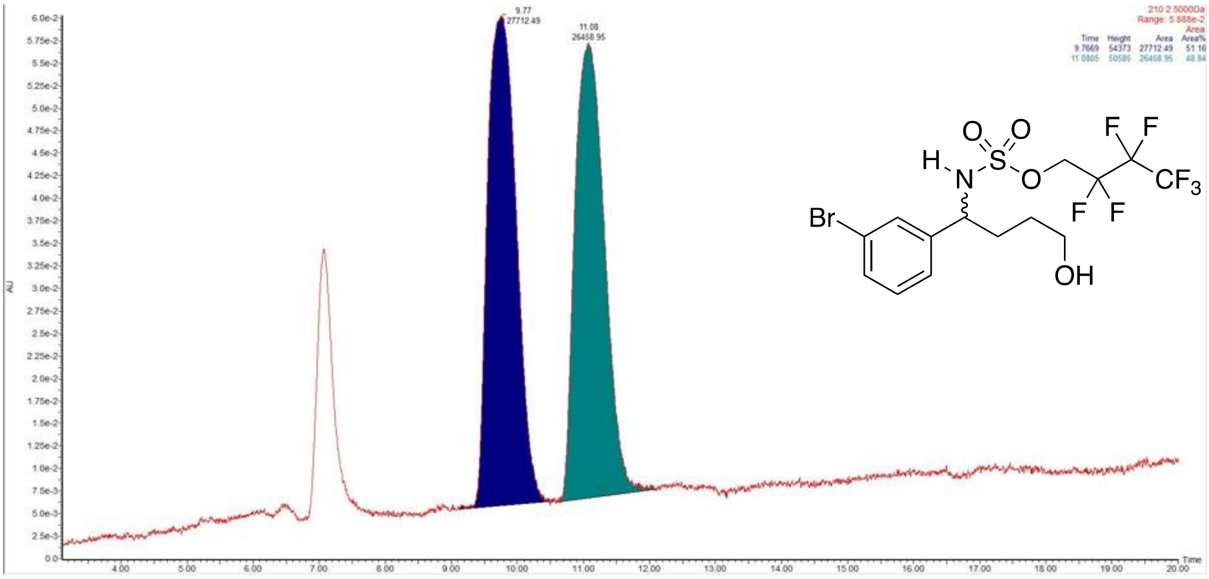

2,2,3,3,4,4,4-Heptafluorobutyl (R)-(4-hydroxy-1-(3-iodophenyl)butyl)sulfamate (**7j**)

Chiral SFC Analysis CHIRAL ART SB (CO_2:^{i}PrOH, 94.5:5.5, 1.25 mL min^{-1}, 40 °C, 227 nm) indicated 88% *ee*, t_R = 45.4 (major), 50.2 (minor) minutes.

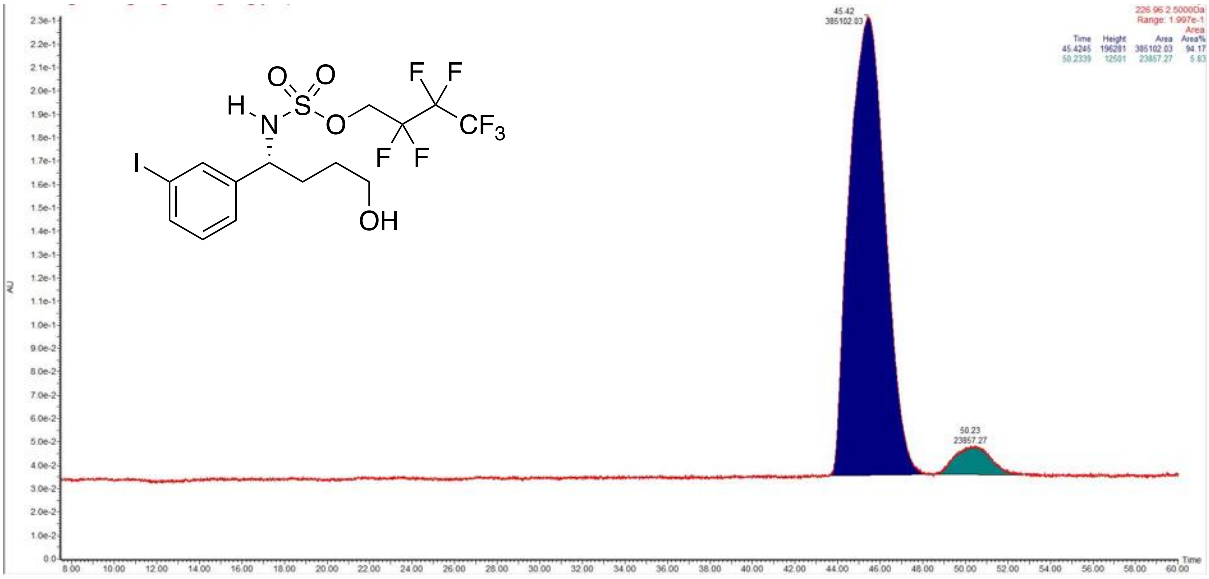

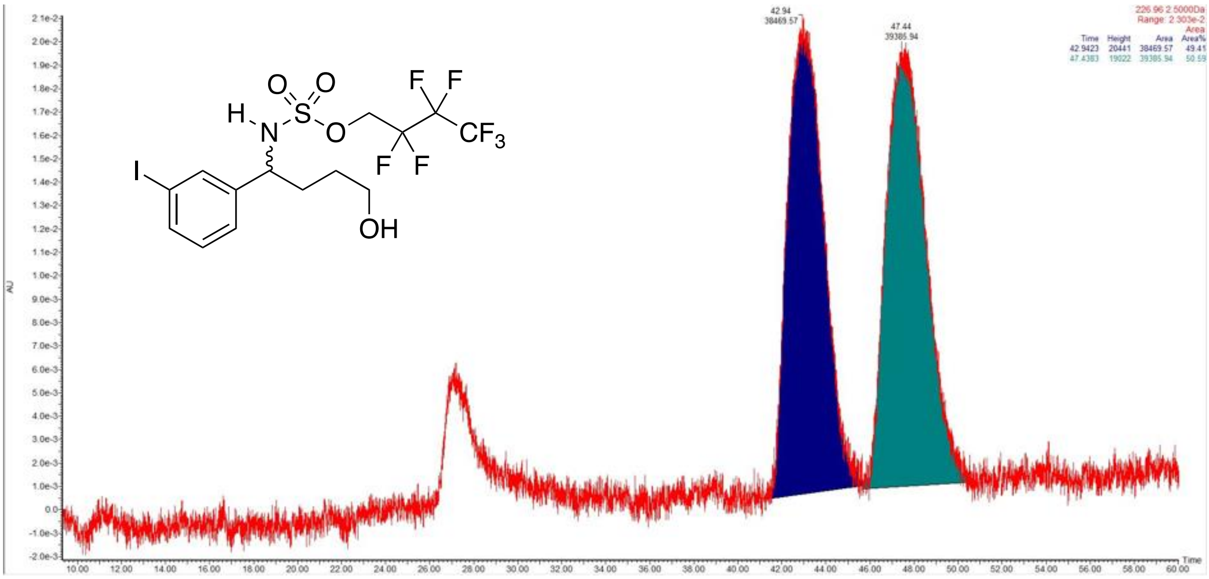

(R)-3-(1-(((2,2,3,3,4,4,4-Heptafluorobutoxy)sulfonyl)amino)-4-hydroxybutyl)phenyl acetate (**7k**)

Chiral SFC Analysis CHIRAL ART SC (CO_2:^{i}PrOH, 96:4, 2.5 mL min^{-1}, 40 °C, 210 nm) indicated 86% *ee*, t_R = 15.6 (major), 17.7 (minor) minutes.

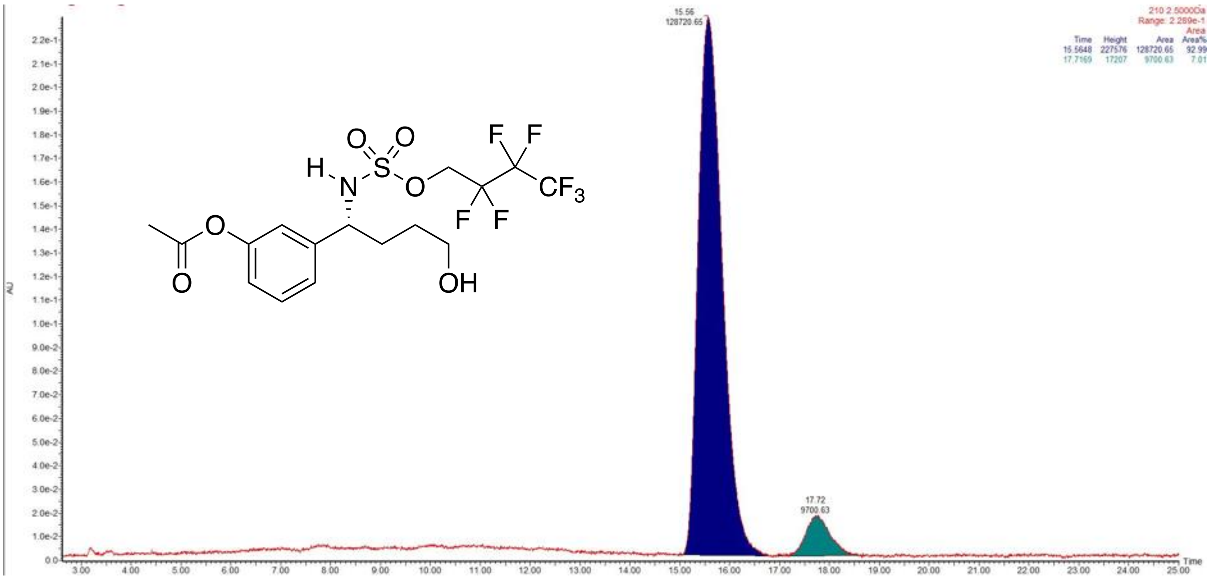

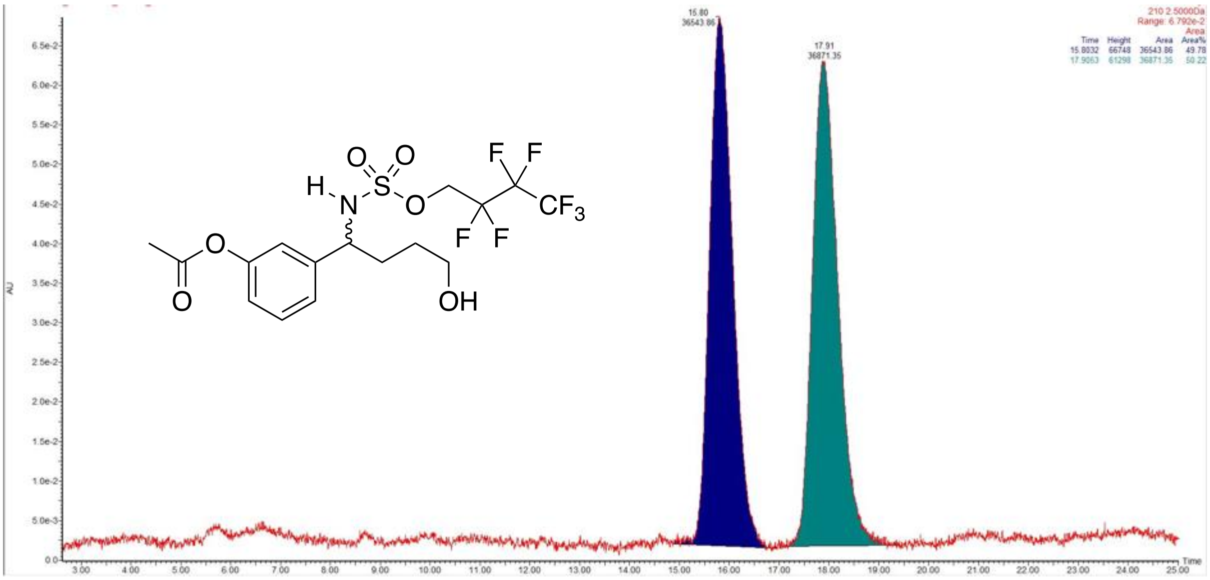

2,2,3,3,4,4,4-Heptafluorobutyl (R)-(4-hydroxy-1-(3-isopropylphenyl)butyl)sulfamate (**7l**)

Chiral SFC Analysis CHIRAL ART SC (CO_2:^{i}PrOH, 95:5, 2.5 mL min^{-1}, 40 °C, 210 nm) indicated 87% *ee*, t_R = 18.0 (major), 20.7 (minor) minutes.

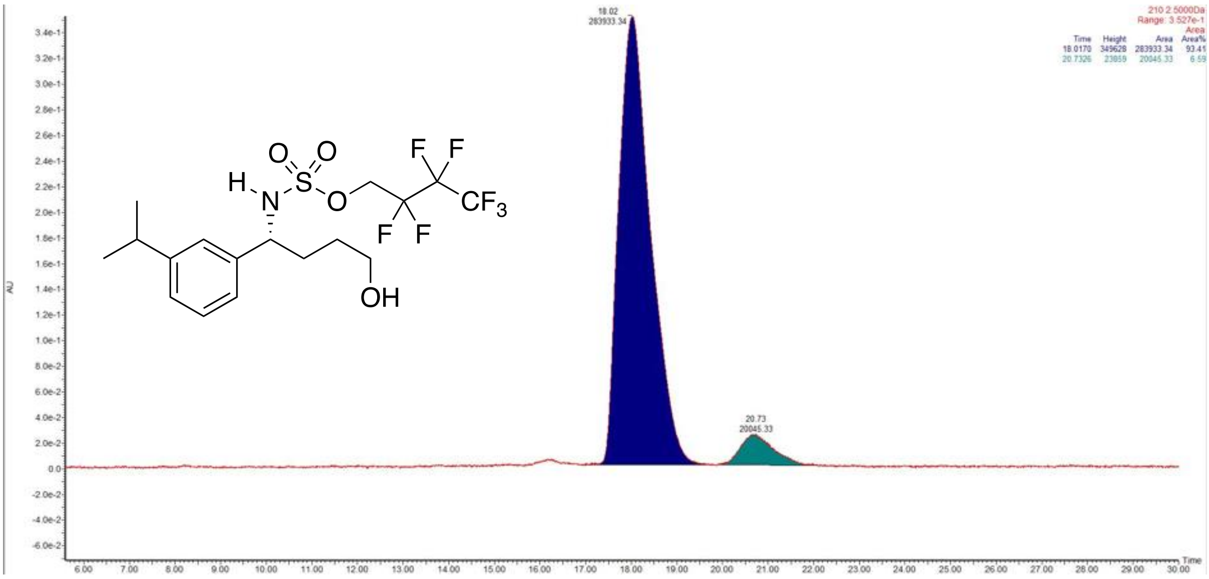

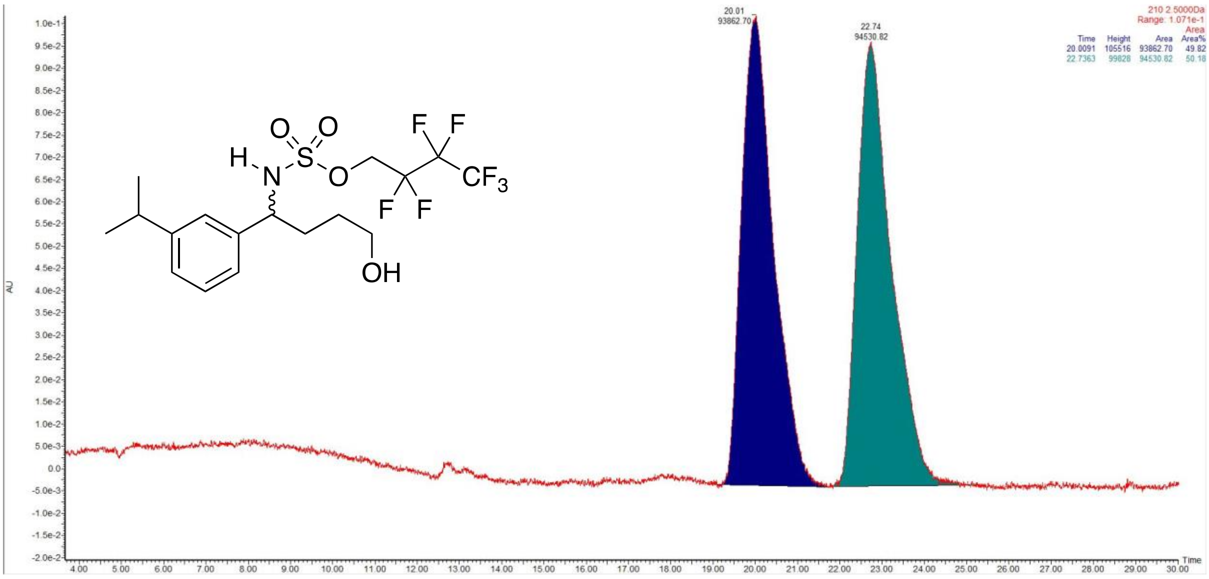

2,2,3,3,4,4,4-Heptafluorobutyl (R)-(1-(2-chlorophenyl)-4-hydroxybutyl)sulfamate (**7m**)

HPLC Analysis CHIRALPAK IC (Hexane:^{i}PrOH, 96:4, 1.0 mL min^{-1}, 30 °C, 190 nm) indicated 85% *ee*, t_R = 15.0 (major), 18.9 (minor) minutes.

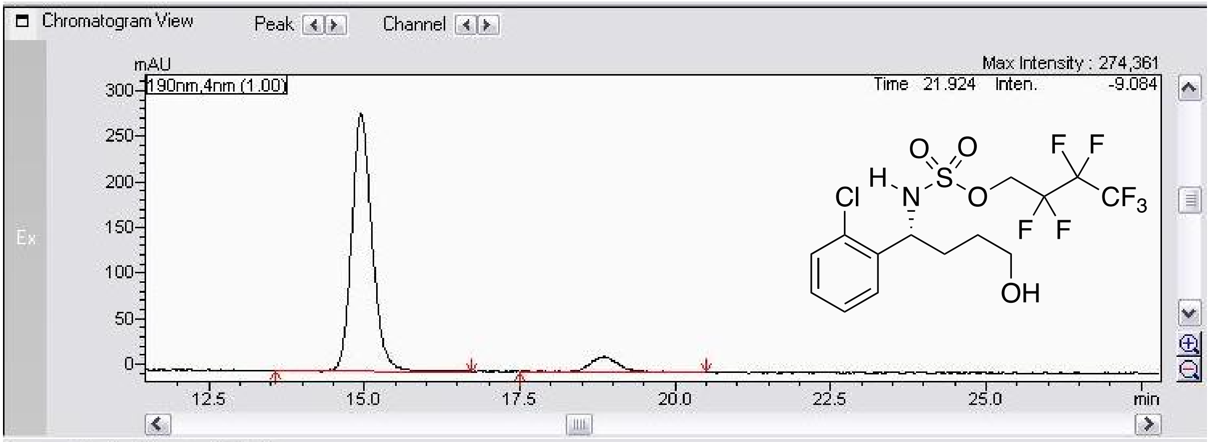

Results View - Peak Table

Peak#	Ret. Time	Area	Height	Area%
1	14.951	6354097	281308	92.668
2	18.858	502729	16790	7.332
Total		6856826	298098	100.000

Extracted 190nm / Ch1 254nm

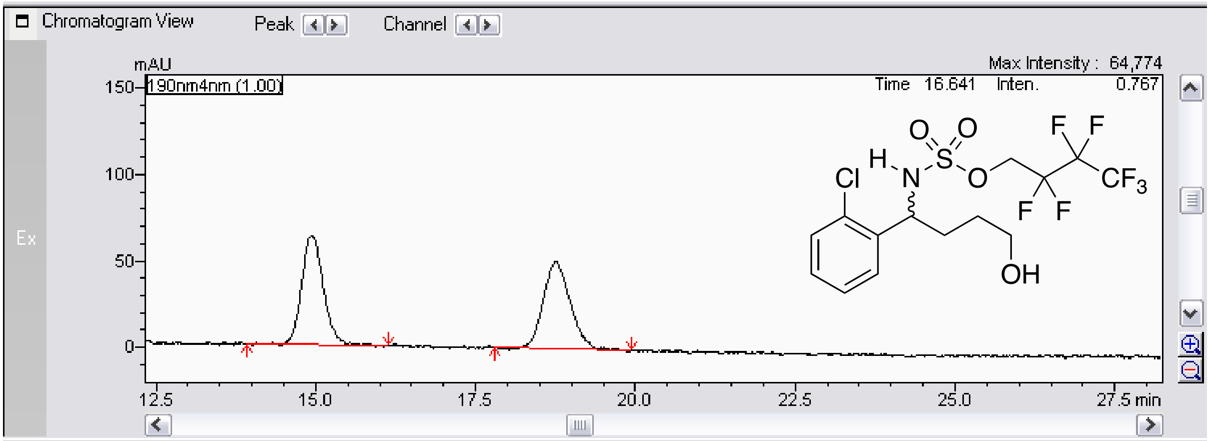

Results View - Peak Table

Peak#	Ret. Time	Area	Height	Area%
1	14.933	1468495	63003	50.254
2	18.759	1453659	50330	49.746
Total		2922154	113334	100.000

Extracted 190nm / Ch1 254nm

2,2,3,3,4,4,4-Heptafluorobutyl (R)-(4-hydroxy-1-(4-methoxyphenyl)butyl)sulfamate (**7n**)

SFC Analysis CHIRALPAK IG (CO_2:^{i}PrOH, 95.5:4.5, 2.5 mL min^{-1}, 40 °C, 223 nm) indicated 75% *ee*, t_R = 11.3 (major), 13.5 (minor) minutes

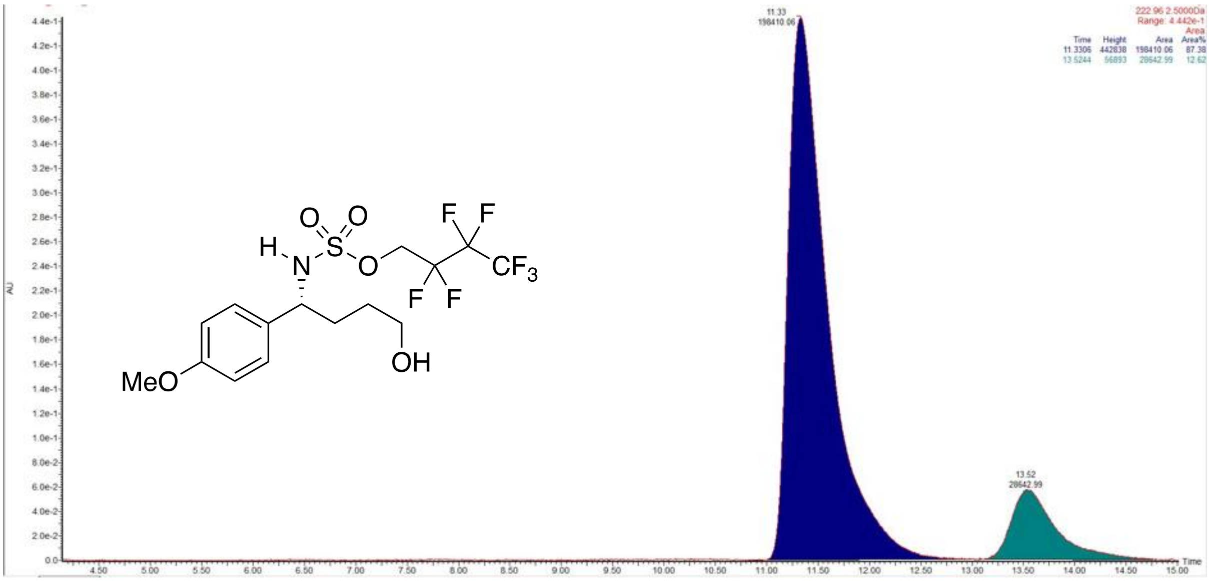

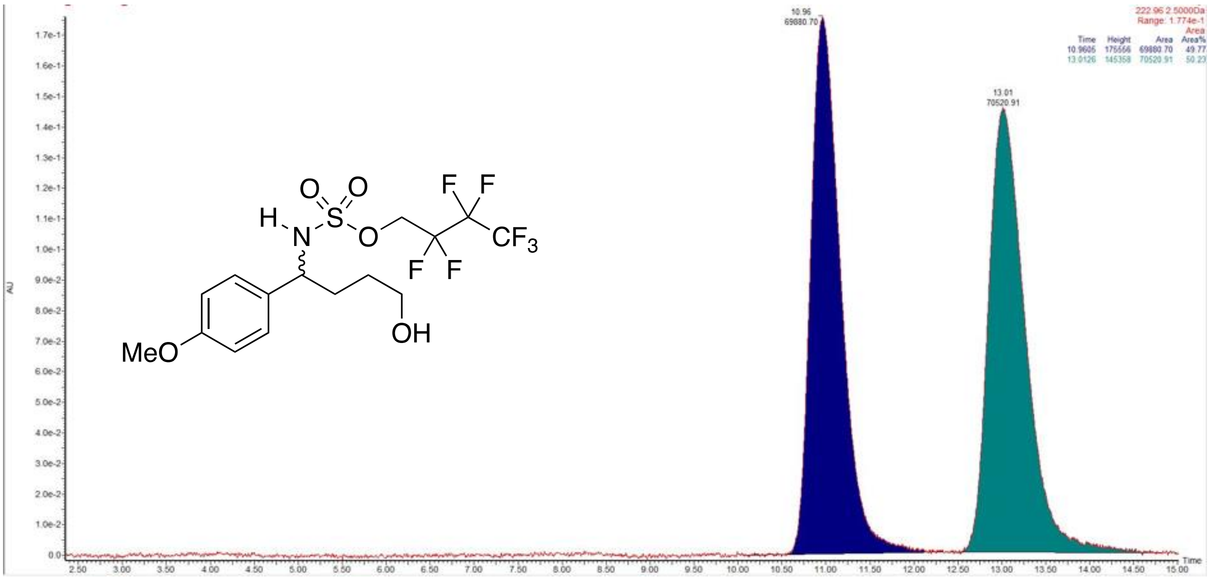

2,2,3,3,4,4,4-Heptafluorobutyl (R)-(1-(4-chlorophenyl)-4-hydroxybutyl)sulfamate (**7o**)

HPLC Analysis CHIRALPAK IC (Hexane:^{i}PrOH, 96:4, 1.0 mL min^{-1}, 30 °C, 196 nm) indicated 86% *ee*, t_R = 12.7 (major), 19.4 (minor) minutes.

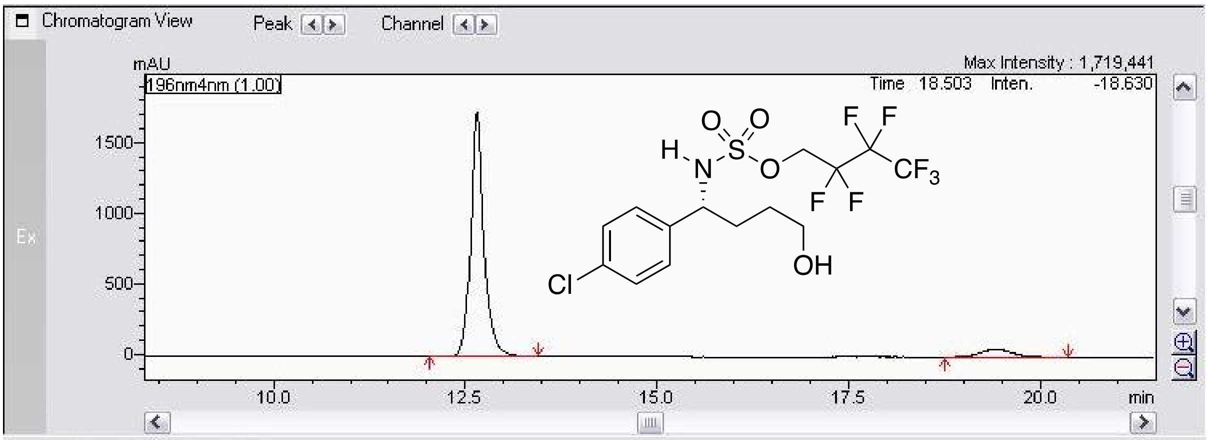

Results View - Peak Table

Peak Table | Compound | Group | Calibration Curve

Peak#	Ret. Time	Area	Height	Area%
1	12.652	20784579	1733970	92.937
2	19.413	1579564	56143	7.063
Total		22364143	1790113	100.000

Extracted 196nm | Ch1 254nm

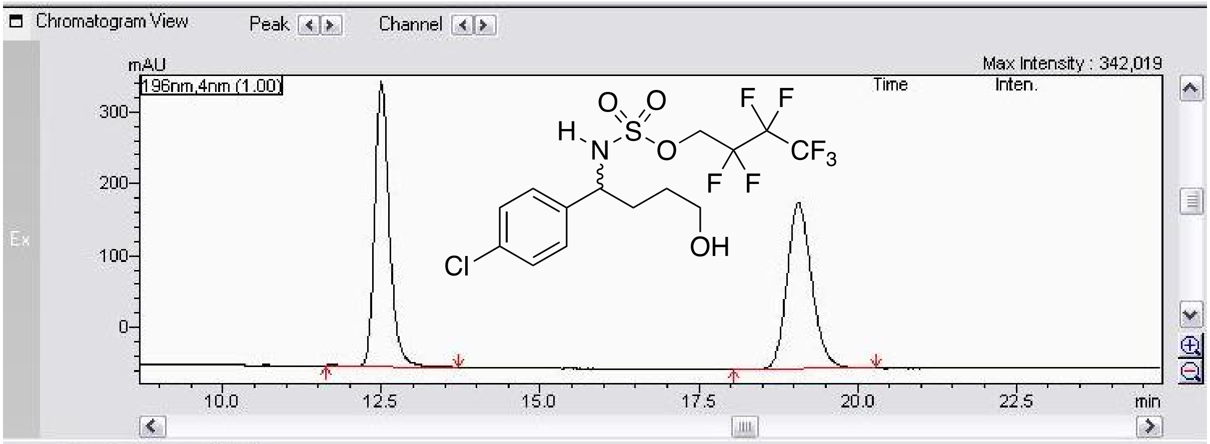

Results View - Peak Table

Peak Table | Compound | Group | Calibration Curve

Peak#	Ret. Time	Area	Height	Area%
1	12.499	6260776	395686	50.089
2	19.070	6238443	230871	49.911
Total		12499219	626557	100.000

Extracted 196nm | Ch1 254nm

2,2,3,3,4,4,4-Heptafluorobutyl (R)-(1-(5-chloro-2-methylphenyl)-4-hydroxybutyl)sulfamate (**7p**)

Chiral SFC Analysis CHIRAL ART SB (CO_2:^{i}PrOH, 95:5, 1.25 mL min^{-1}, 40 °C, 217 nm) indicated 88% *ee*, t_R = 27.0 (major), 29.7 (minor) minutes.

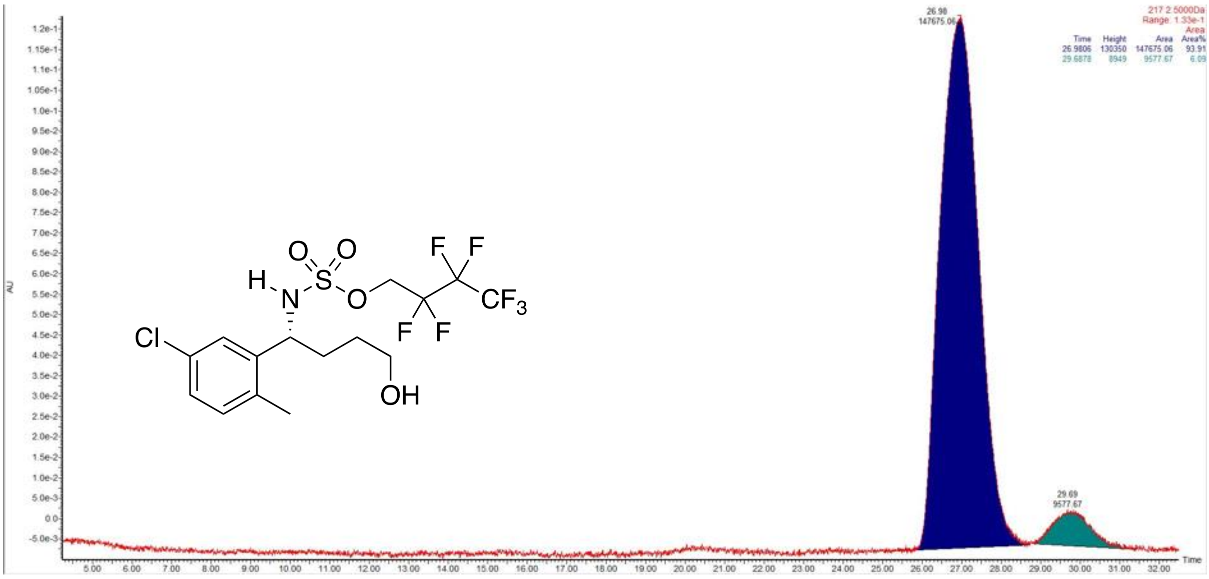

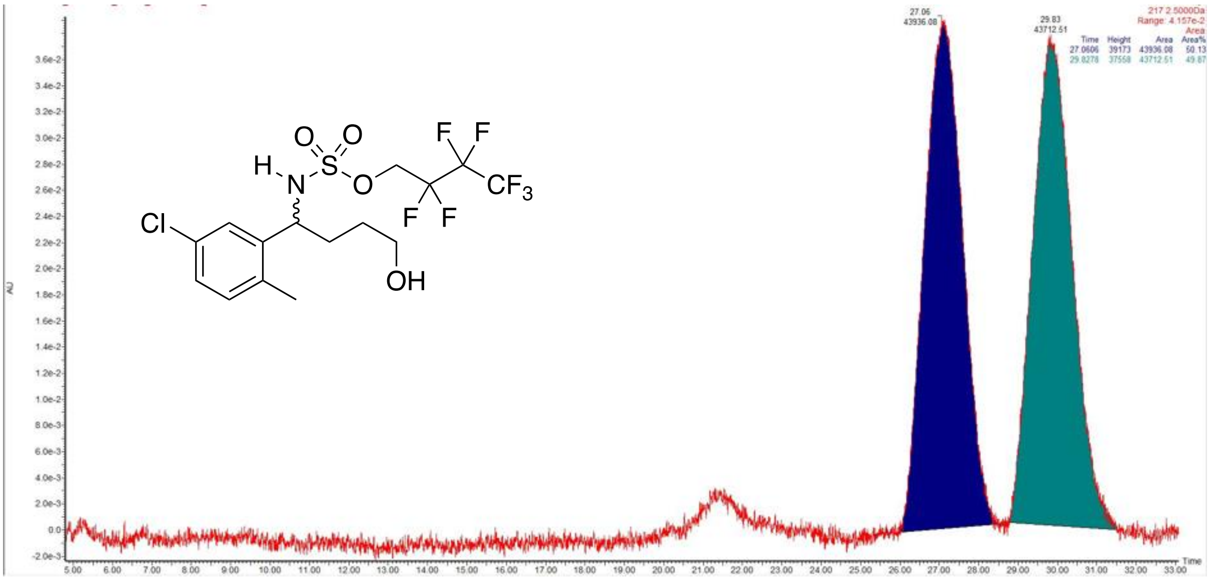

2,2,3,3,4,4,4-Heptafluorobutyl (R)-(1-(2,3-dimethylphenyl)-4-hydroxybutyl)sulfamate (**7q**)

Chiral SFC Analysis CHIRAL ART SC (CO_2:^{i}PrOH, 96.5:3.5, 2.5 mL min^{-1}, 40 °C, 210 nm) indicated 87% *ee*, t_R = 18.5 (major), 23.1 (minor) minutes.

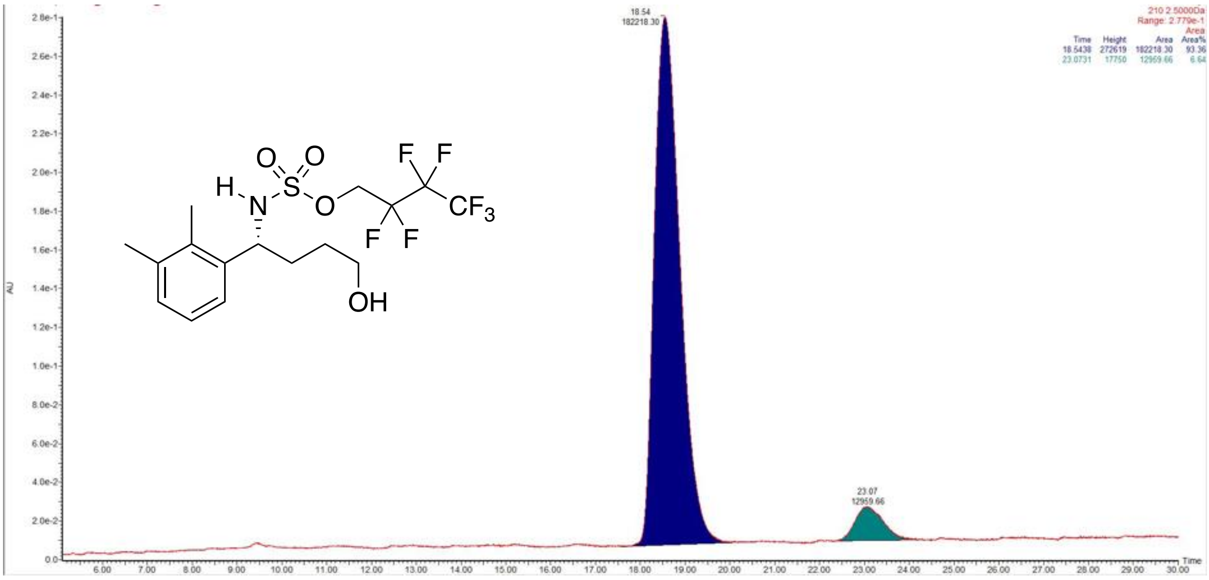

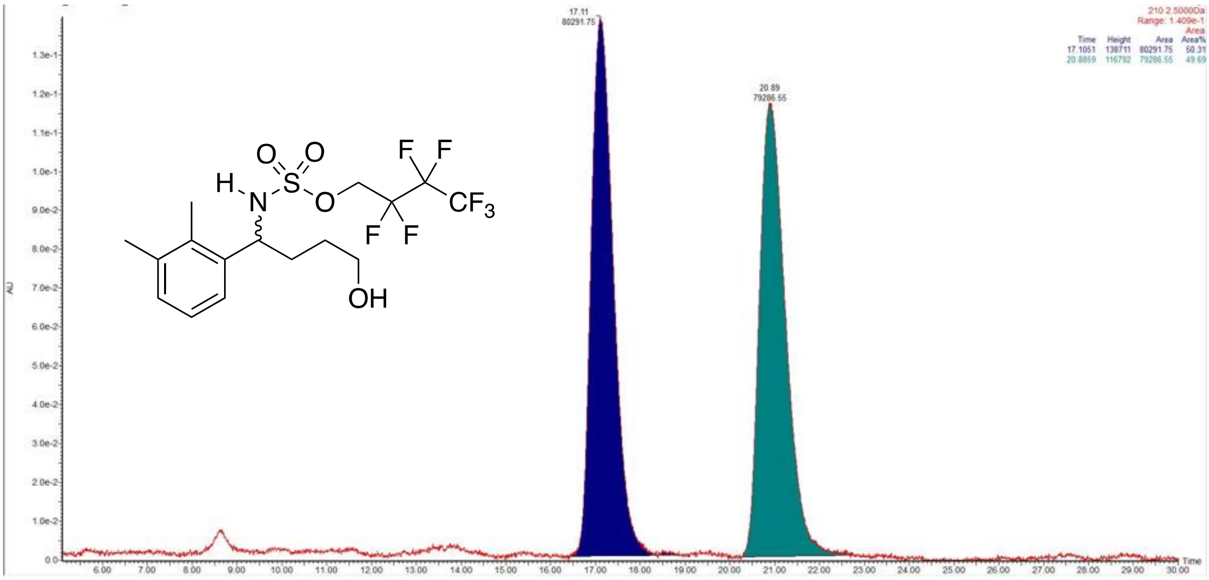

2,2,3,3,4,4,4-Heptafluorobutyl (R)-(1-(4-fluoro-2-methylphenyl)-4-hydroxybutyl)sulfamate (**7r**)

Chiral SFC Analysis CHIRAL ART SC (CO_2:^{i}PrOH, 97:3, 2.5 mL min^{-1}, 40 °C, 210 nm) indicated 85% *ee*, t_R = 14.3 (major), 16.4 (minor) minutes.

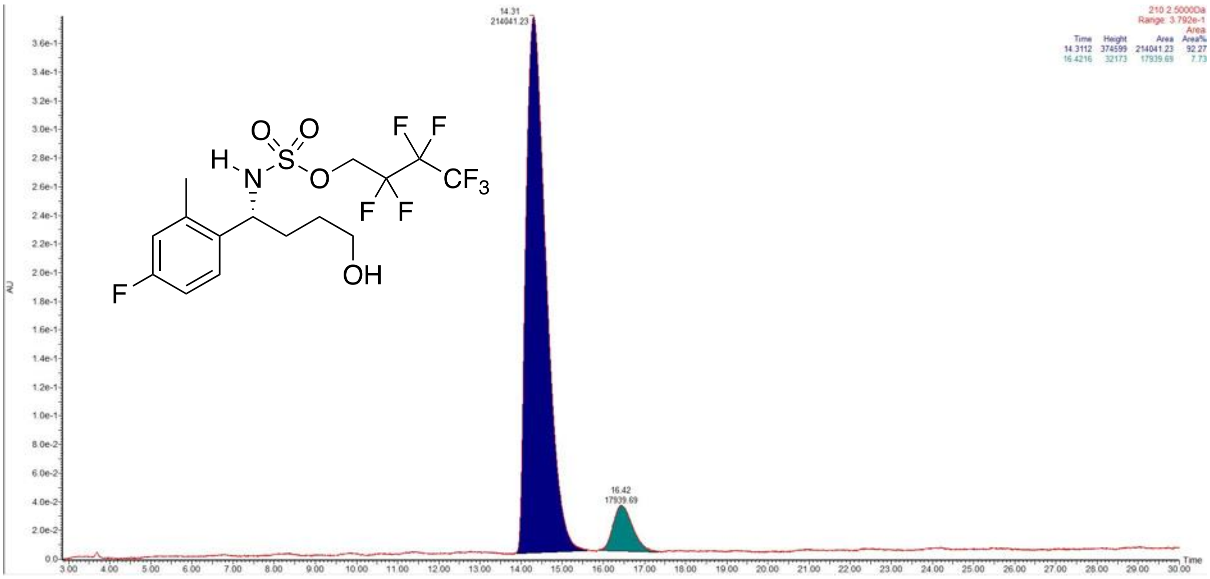

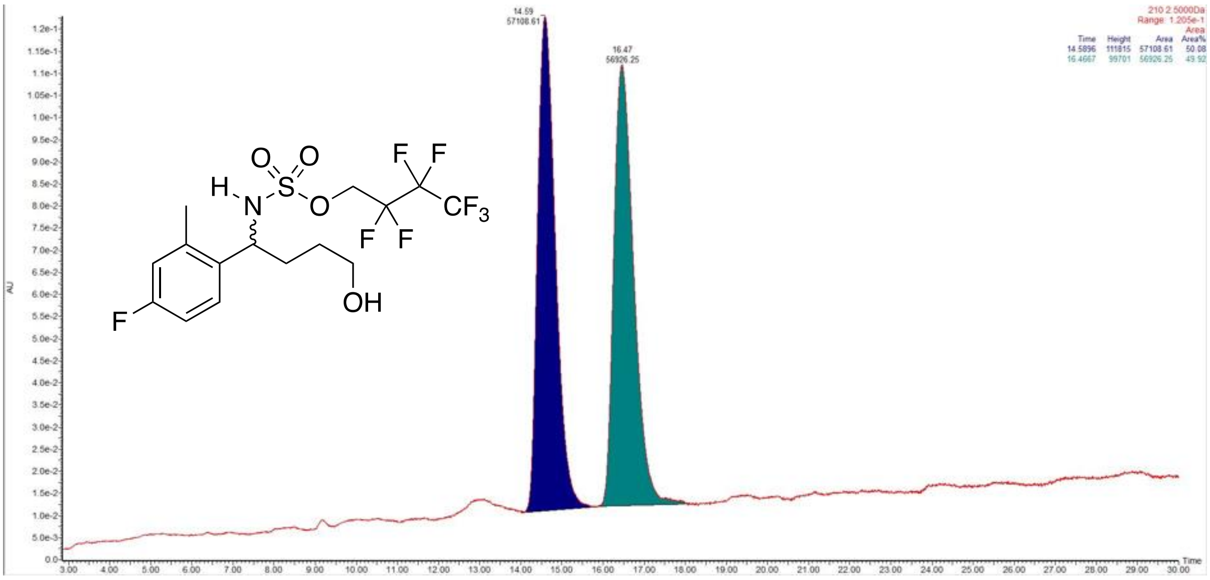

2,2,3,3,4,4,4-Heptafluorobutyl (R)-(1-(2,5-dimethylphenyl)-4-hydroxybutyl)sulfamate (**7s**)

Chiral SFC Analysis CHIRAL ART SC (CO_2:^{i}PrOH, 96.5:3.5, 2.5 mL min^{-1}, 40 °C, 210 nm) indicated 86% *ee*, t_R = 13.1 (major), 14.6 (minor) minutes.

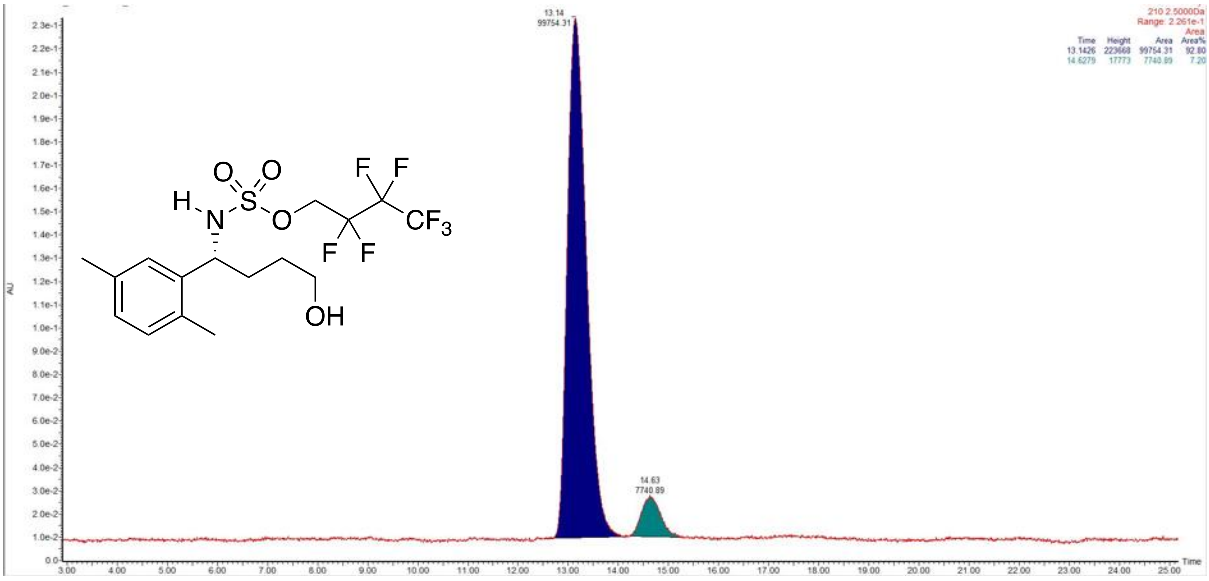

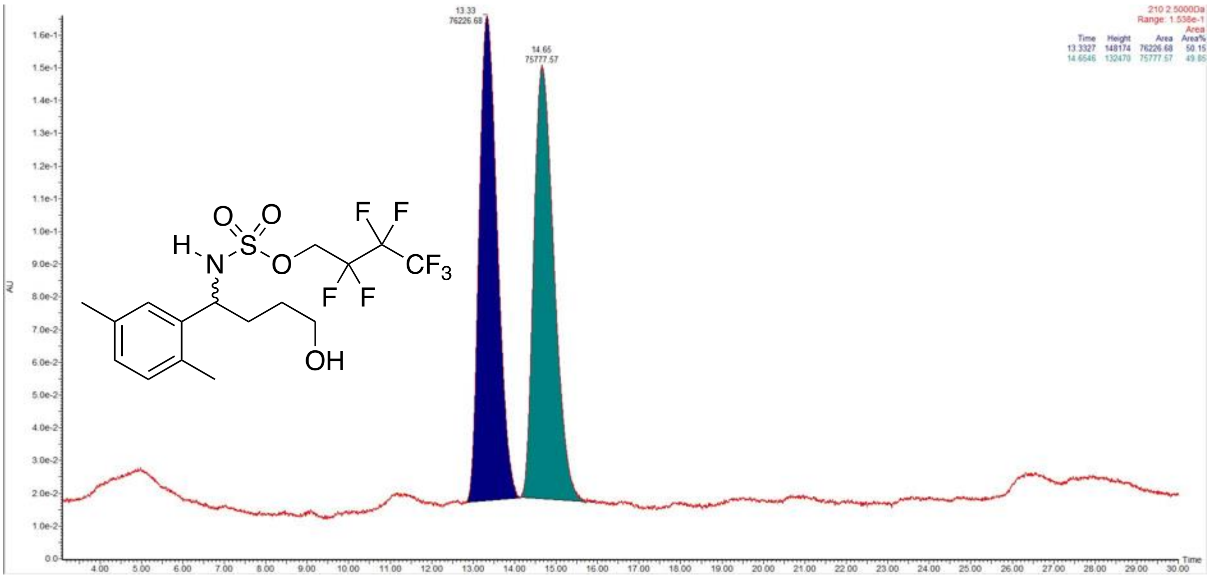

2,2,3,3,4,4,4-Heptafluorobutyl (R)-(1-(2,4-difluorophenyl)-4-hydroxybutyl)sulfamate (**7t**)

HPLC Analysis CHIRALPAK IC (Hexane:^{i}PrOH, 96:4, 1.0 mL min^{-1}, 30 °C, 190 nm) indicated 89% *ee*, t_R = 11.8 (major), 17.3 (minor) minutes.

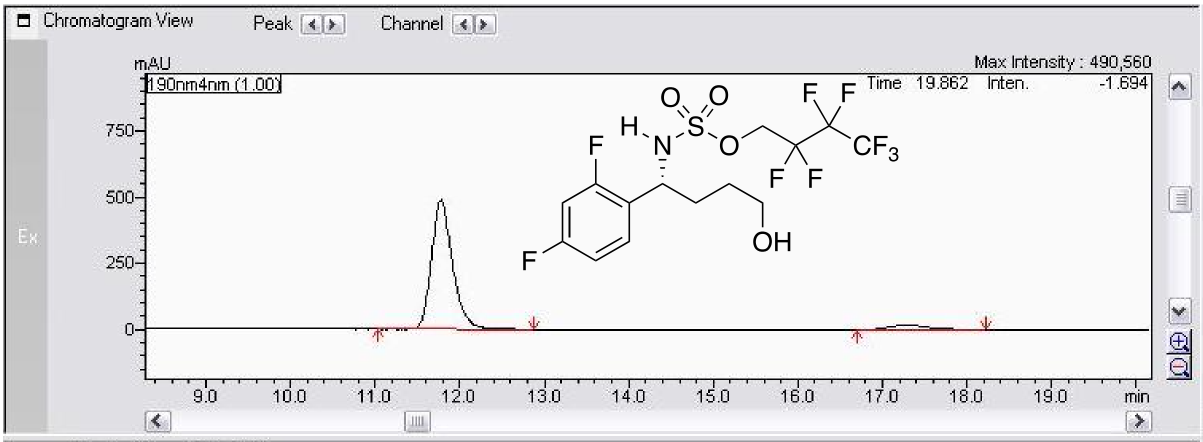

Results View - Peak Table

Peak Table | Compound | Group | Calibration Curve

Peak#	Ret. Time	Area	Height	Area%
1	11.778	8421480	489010	94.342
2	17.283	505092	17985	5.658
Total		8926572	506995	100.000

Extracted 190nm Ch1 254nm

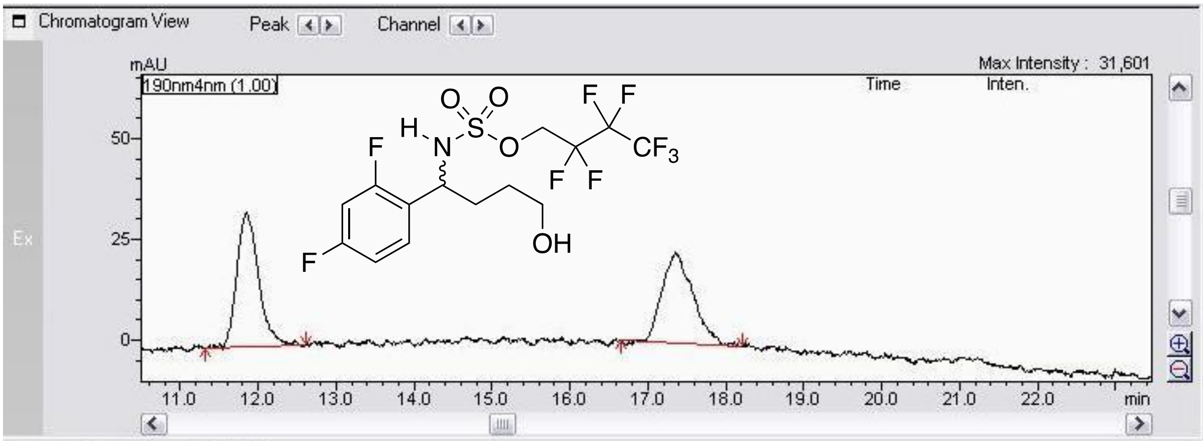

Results View - Peak Table

Peak Table | Compound | Group | Calibration Curve

Peak#	Ret. Time	Area	Height	Area%
1	11.860	651438	33335	51.644
2	17.349	609972	22348	48.356
Total		1261410	55683	100.000

Extracted 190nm Ch1 254nm

2,2,3,3,4,4,4-Heptafluorobutyl (R)-(1-(3-bromo-2-methylphenyl)-4-hydroxybutyl)sulfamate (**7u**)

Chiral SFC Analysis CHIRAL ART SB (CO_2:MeOH, 96:4, 2.5 mL min^{-1}, 40 °C, 210 nm) indicated 84% *ee*, t_R = 10.7 (minor), 13.3 (major) minutes.

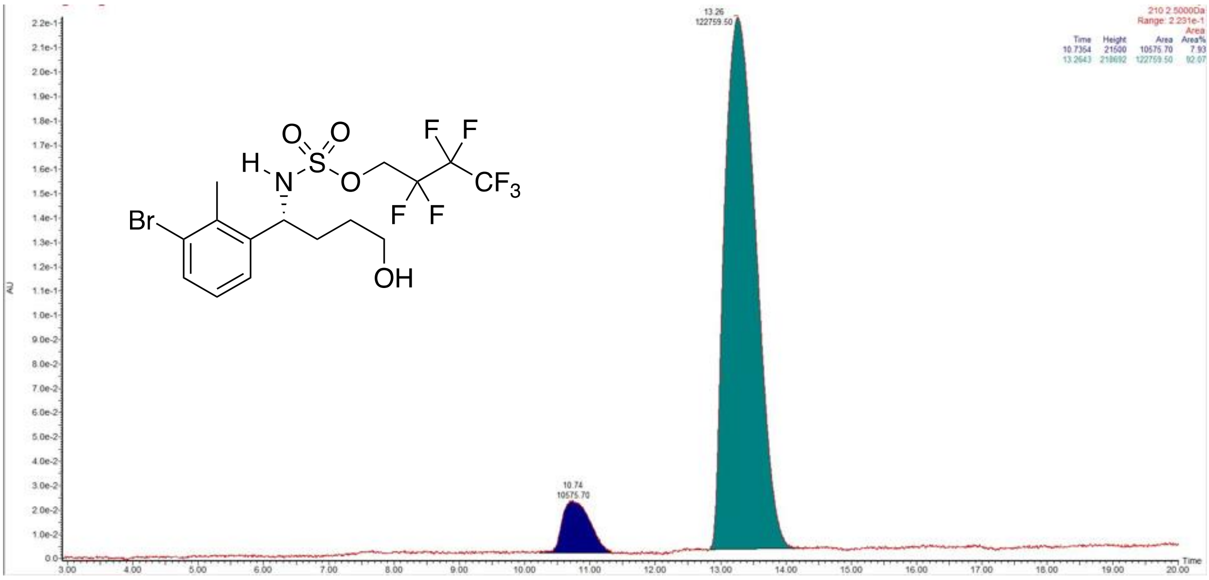

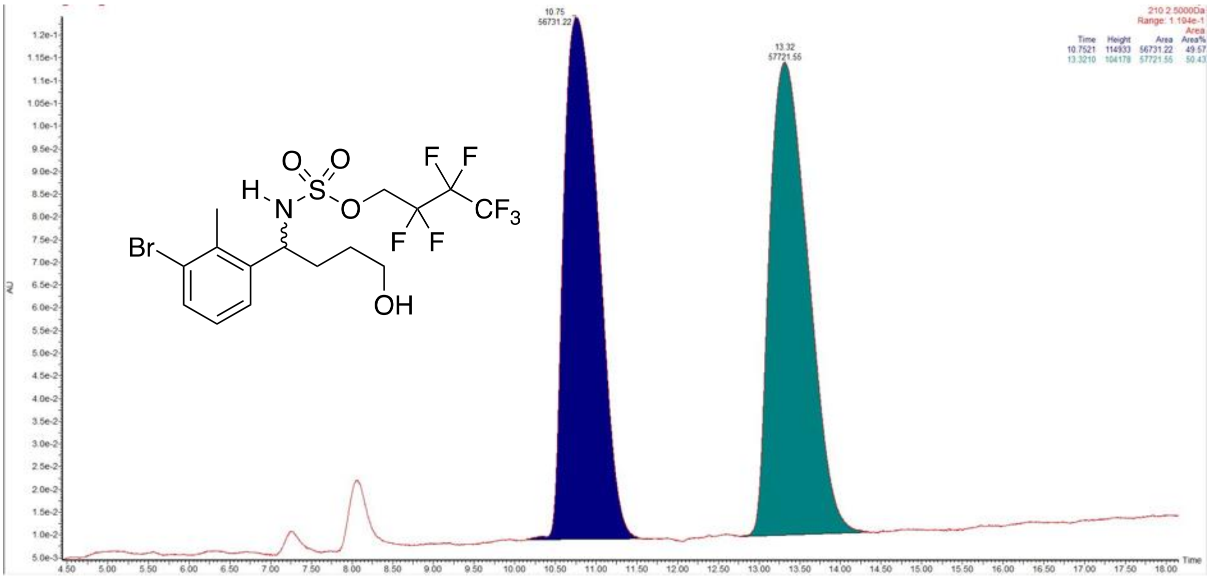

2,2,3,3,4,4,4-Heptafluorobutyl (R)-(1-(3-chloro-2-methylphenyl)-4-hydroxybutyl)sulfamate (**7v**)

Chiral SFC Analysis CHIRAL ART SB (CO_2: ^{i}PrOH, 95:5, 2.5 mL min^{-1}, 40 °C, 214 nm) indicated 86% *ee*, t_R = 14.8 (minor), 16.5 (major) minutes.

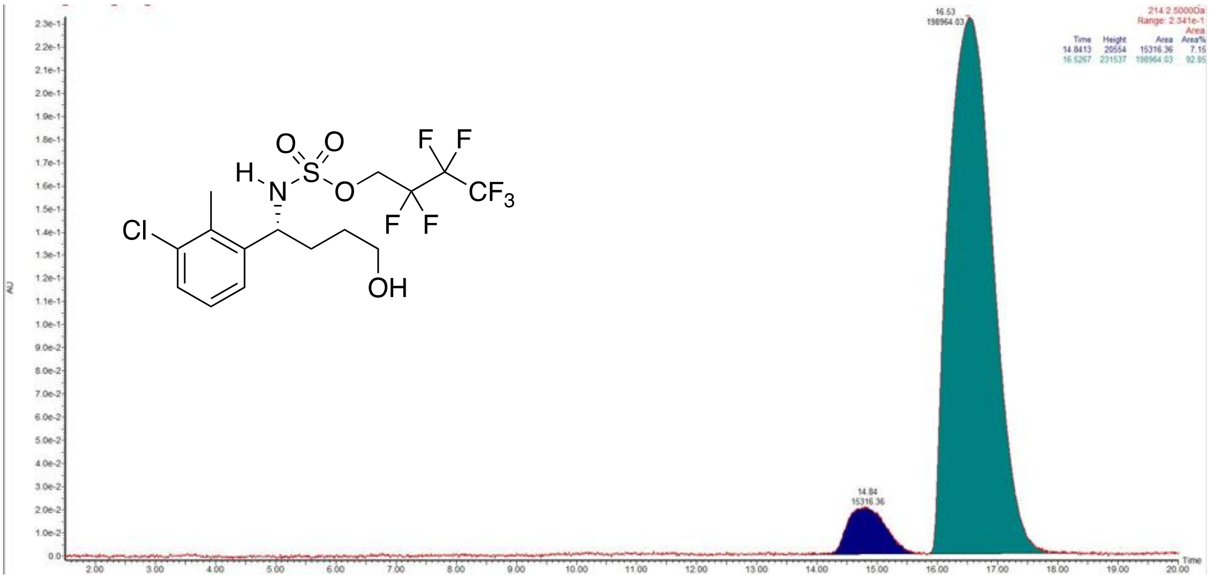

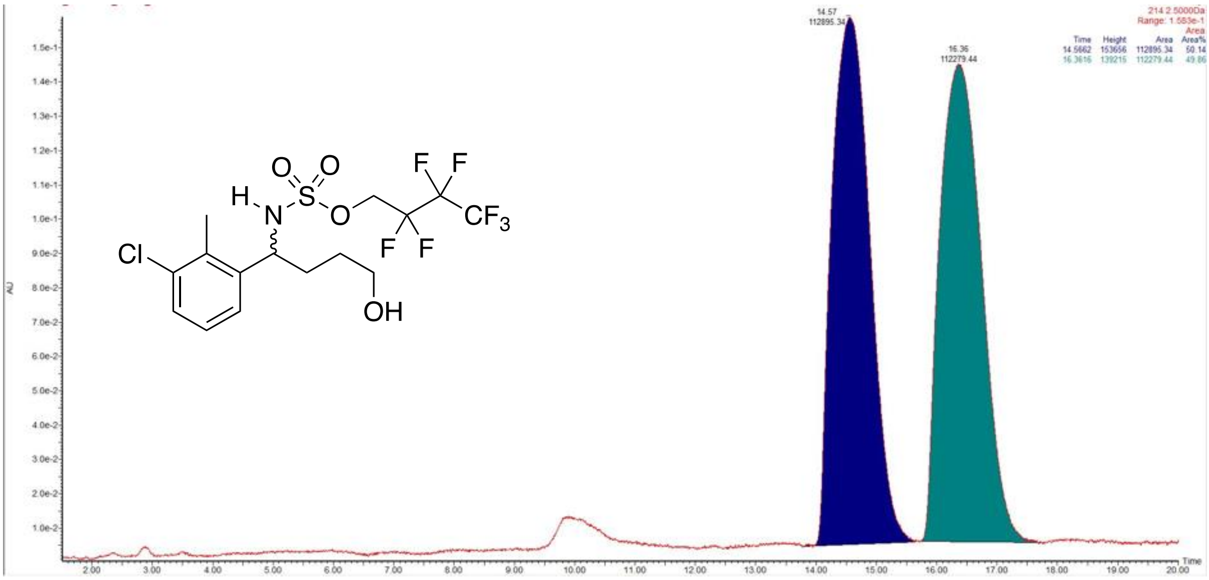

2,2,3,3,4,4,4-Heptafluorobutyl (R)-(4-hydroxy-1-(naphthalen-1-yl)butyl)sulfamate (**7w**)

Chiral SFC Analysis CHIRAL ART SB (CO_2:MeOH, 94:6, 1.25 mL min^{-1}, 40 °C, 220 nm) indicated 80% *ee*, t_R = 22.3 (minor), 25.6 (major) minutes.

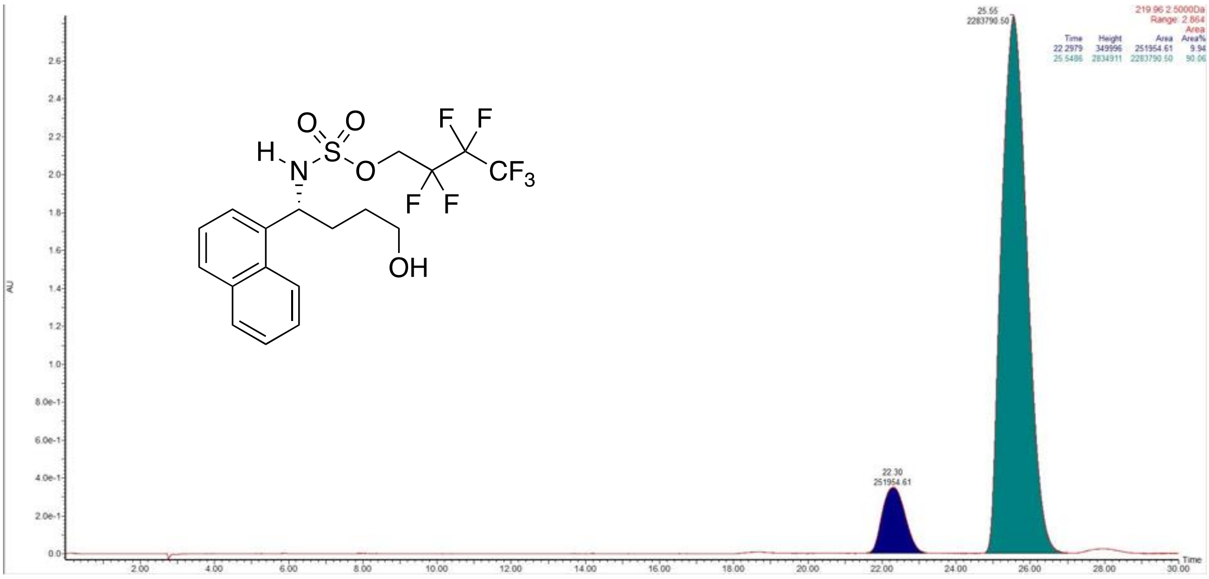

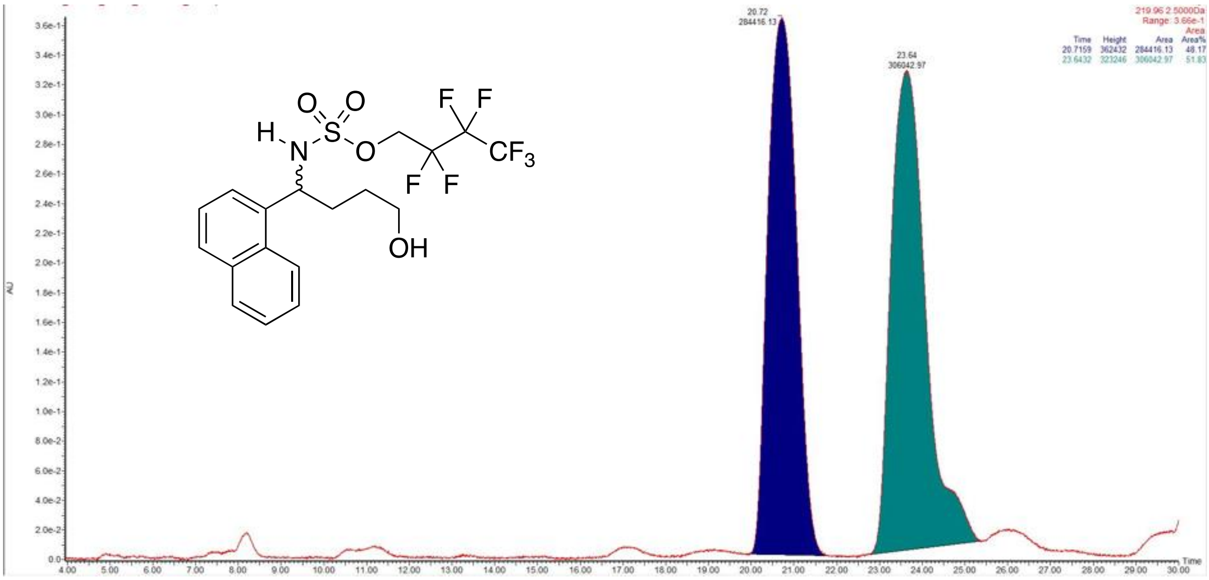

2,2,3,3,4,4,4-Heptafluorobutyl (R)-(4-hydroxy-1-(3-methylthiophen-2-yl)butyl)sulfamate (**7x**)

Chiral SFC Analysis CHIRAL ART SC (CO_2:MeOH, 95.5:4.5, 1.25 mL min^{-1}, 40 °C, 234 nm) indicated 80% *ee*, t_R = 20.0 (major), 23.9 (minor) minutes.

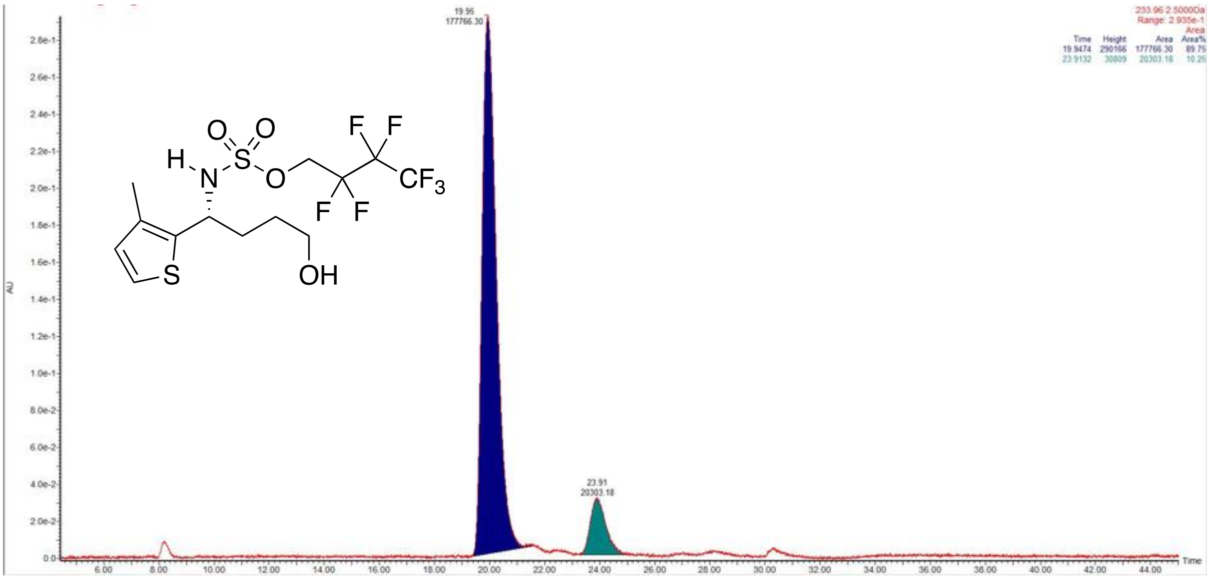

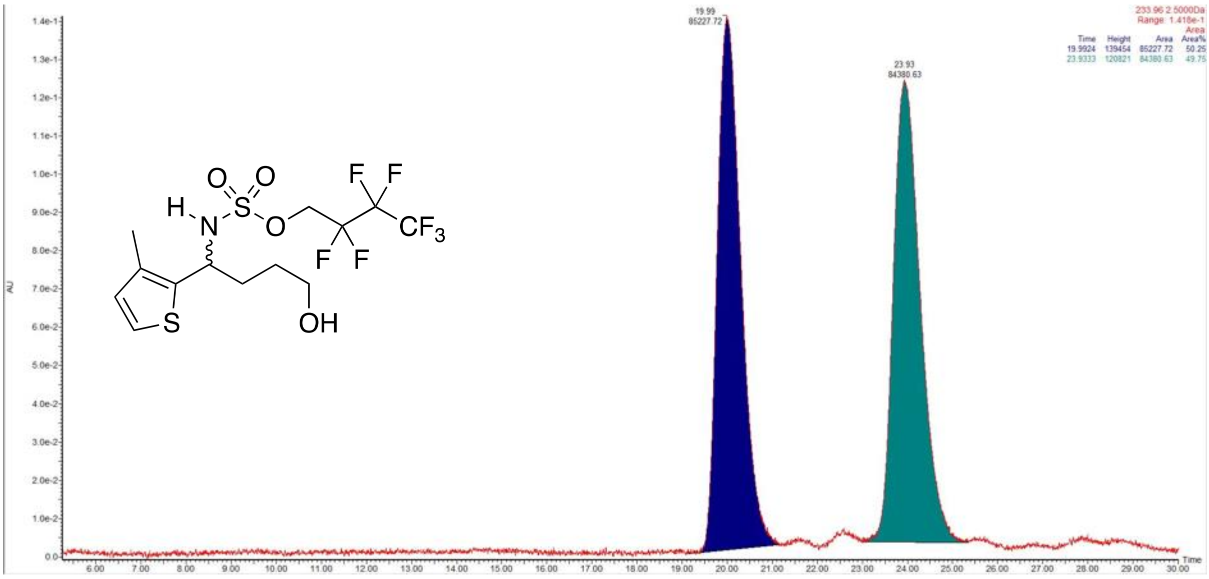

(R)-2-Phenylpyrrolidine (**9**)

Chiral HPLC Analysis CHIRALPAK AD-H (Hexane + 0.1% Et_2NH:$^{i}PrOH$, 98:2, 1.0 mL min^{-1}, 30 °C, 254 nm) indicated 89% *ee*, t_R = 14.8 (major), 17.0 (minor) minutes.

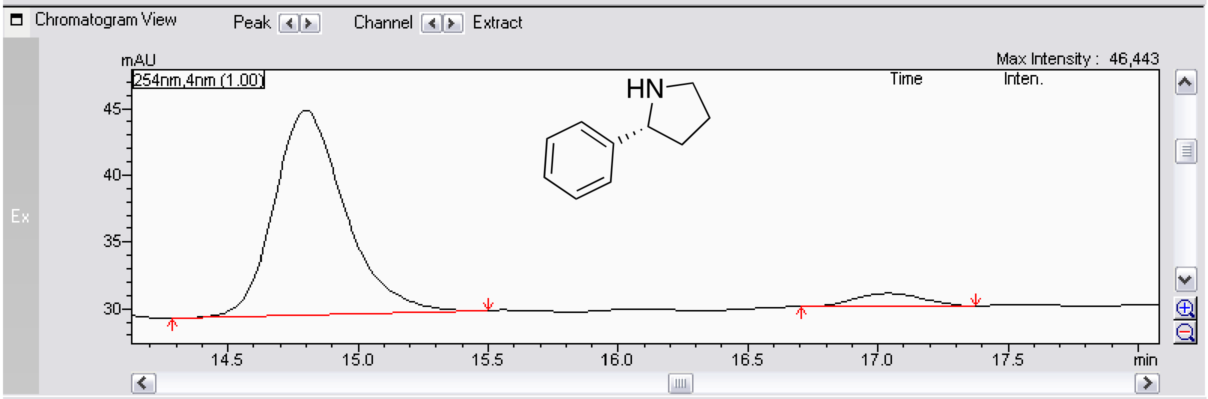

Results View - Peak Table

Peak Table | Compound | Group | Calibration Curve

Peak#	Ret. Time	Area	Height	Area%
1	14.797	289767	15305	94.315
2	17.043	17465	977	5.685
Total		307232	16283	100.000

Extracted 254nm | Ch1 254nm

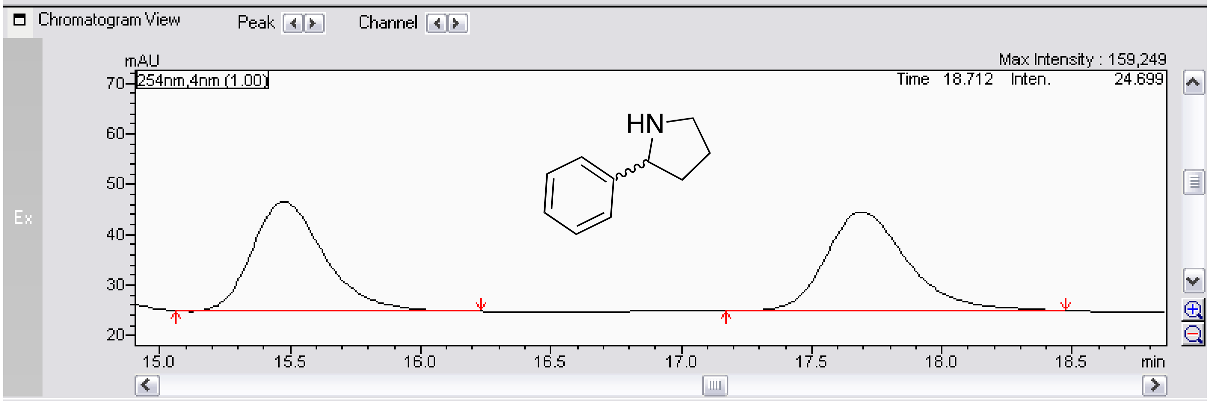

Results View - Peak Table

Peak Table | Compound | Group | Calibration Curve

Peak#	Ret. Time	Area	Height	Area%
1	15.475	416026	21776	49.421
2	17.693	425777	19635	50.579
Total		841803	41411	100.000

Extracted 254nm | Ch1 254nm

2,2,3,3,4,4,4-Heptafluorobutyl (R)-(1-phenylbutyl)sulfamate (**11**)

Chiral SFC Analysis CHIRAL ART SB (CO_2:^{i}PrOH, 99.5:0.5, 1.25 mL min^{-1}, 40 °C, 210 nm) indicated 28% *ee*, t_R = 17.4 (minor), 20.2 (major) minutes.

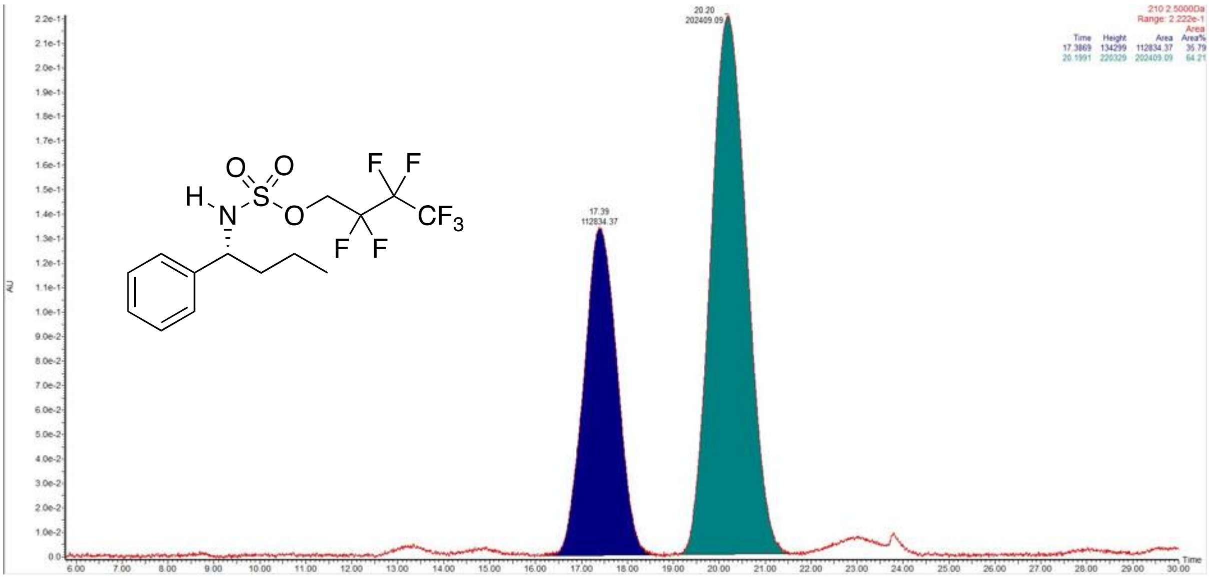

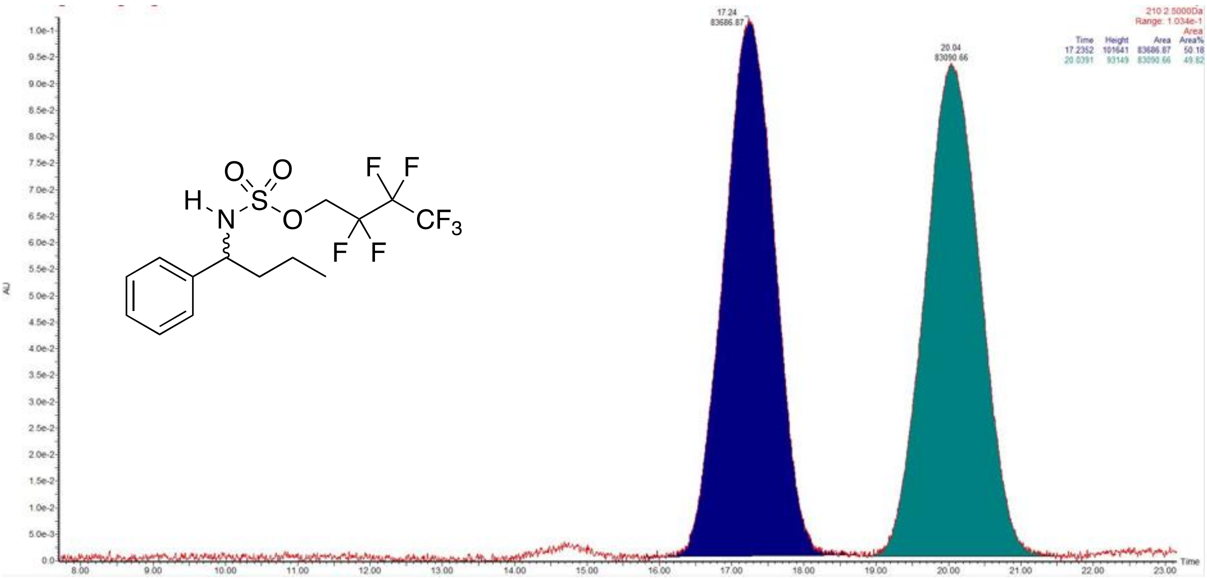

Methyl (R)-4-(((2,2,3,3,4,4,4-heptafluorobutoxy)sulfonyl)amino)-4-phenylbutanoate (**13**)

Chiral SFC Analysis CHIRAL ART SC (CO_2:MeOH, 98.5:1.5, 2.5 mL min^{-1}, 40 °C, 210 nm) indicated 78% *ee*, t_R = 5.6 (major), 6.9 (minor) minutes.

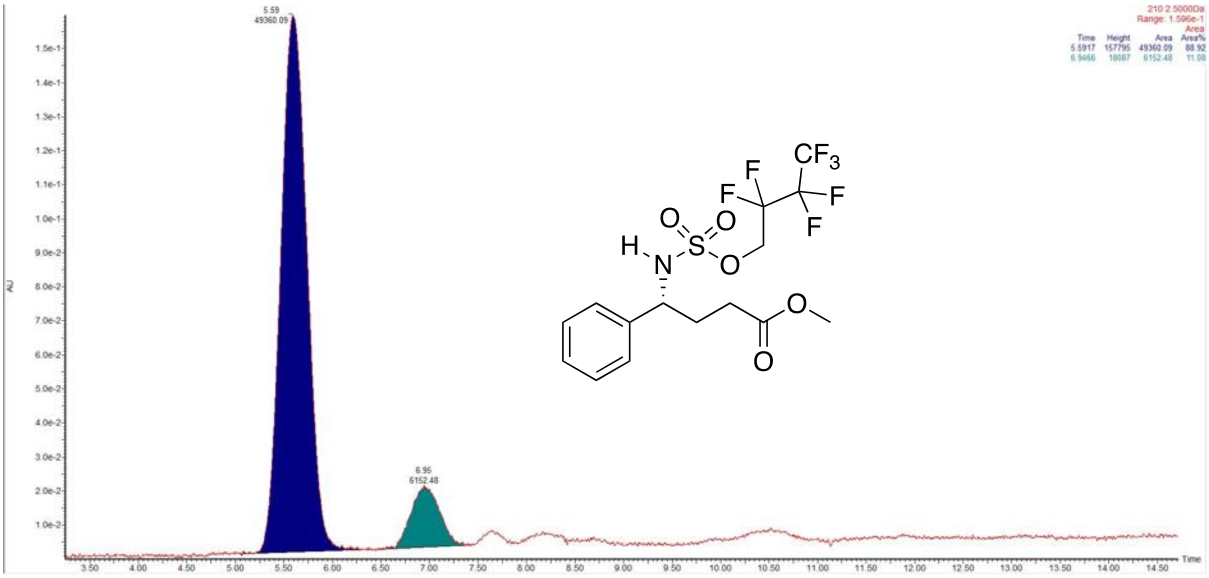

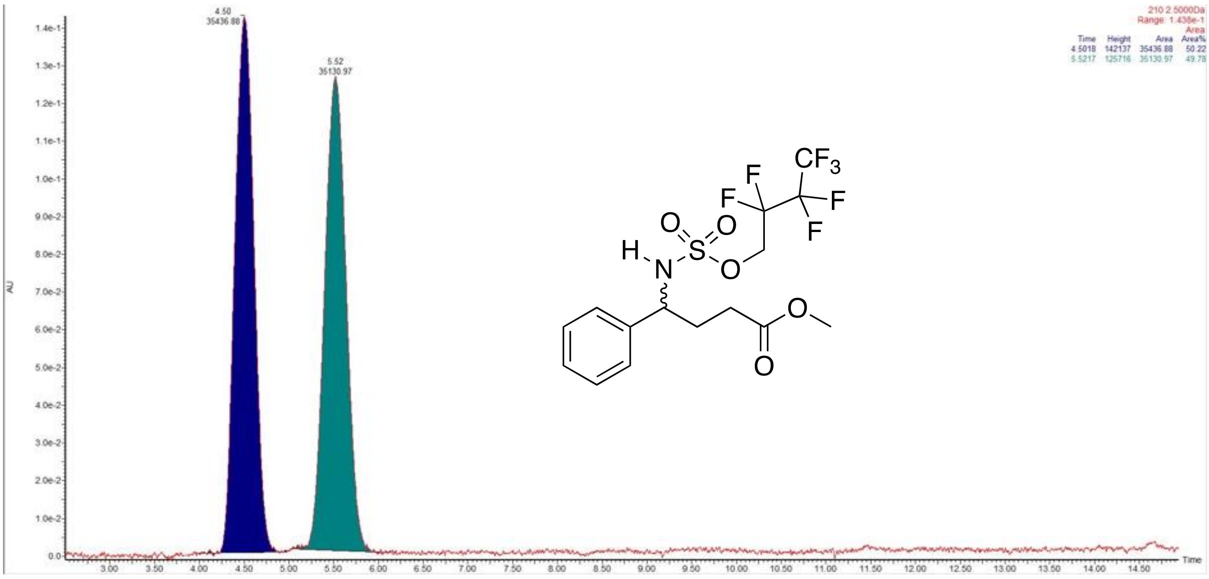

2,2,3,3,4,4,4-Heptafluorobutyl (R)-(3-hydroxy-1-phenylpropyl)sulfamate (**14**)

Chiral SFC Analysis CHIRALPAK IG (CO_2:MeOH, 97.5:2.5, 2.5 mL min^{-1}, 40 °C, 210 nm) indicated 78% *ee*, t_R = 7.4 (minor), 8.1 (major) minutes.

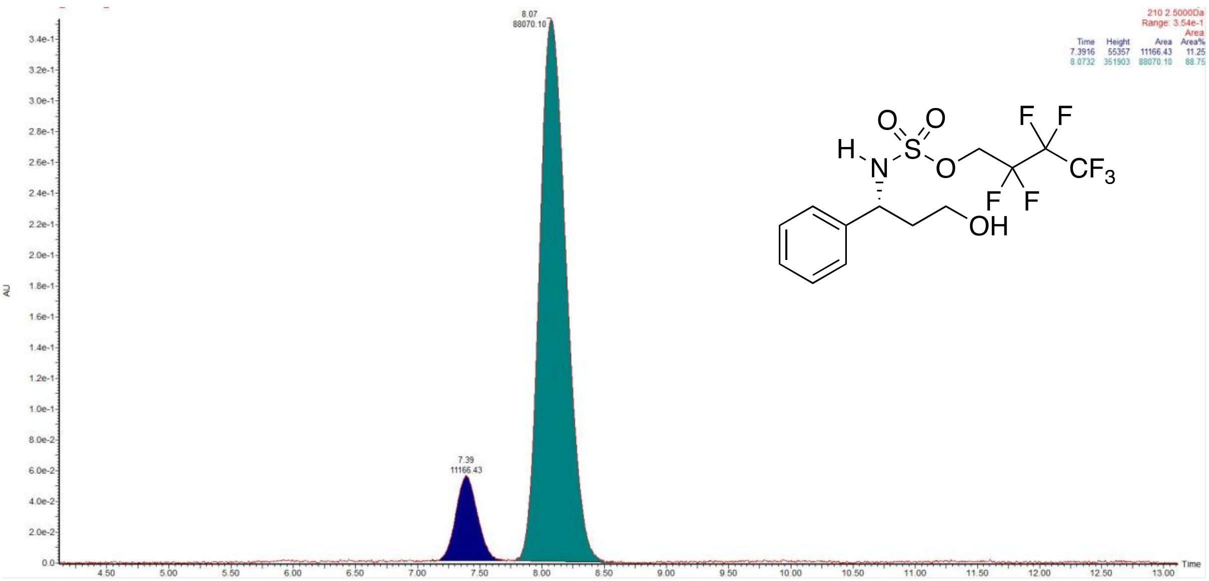

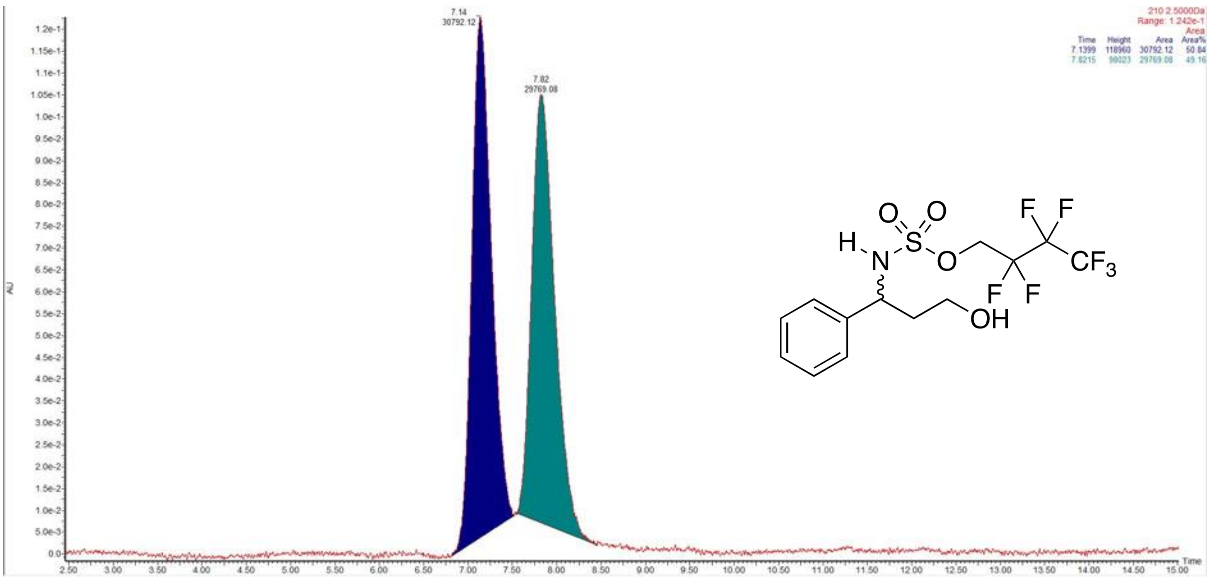

2,2,3,3,4,4,4-Heptafluorobutyl (R)-(5-hydroxy-1-phenylpentyl)sulfamate (**15**)

SFC Analysis CHIRAL ART SC (CO_2:MeOH, 97.5:2.5, 2.5 mL min^{-1}, 40 °C, 210 nm) indicated 76% *ee*, t_R = 11.2 (major), 12.4 (minor) minutes.

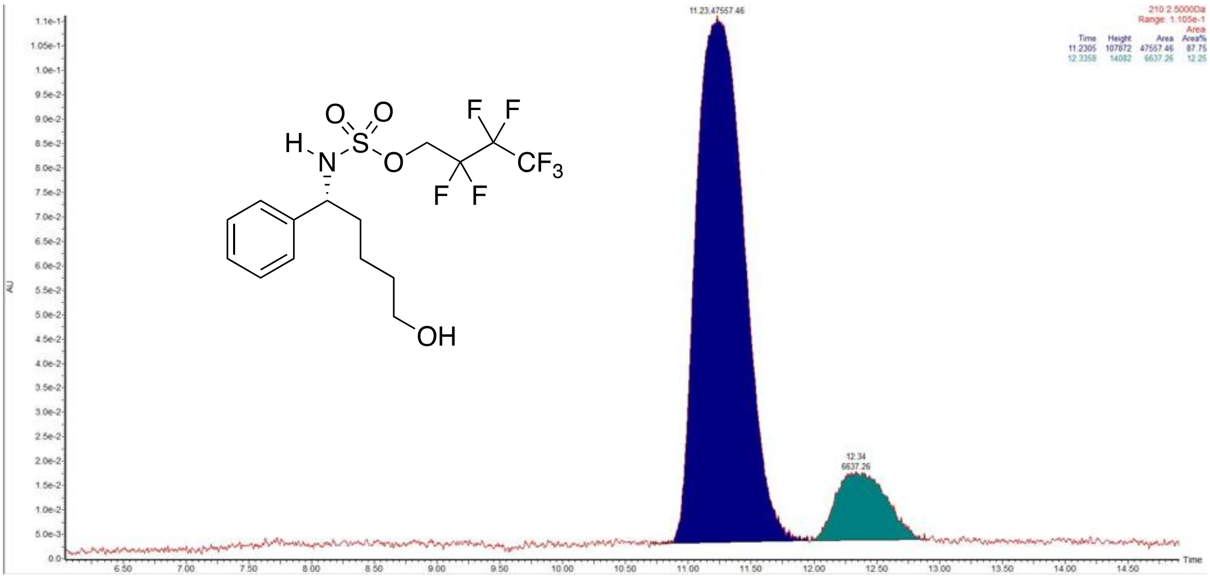

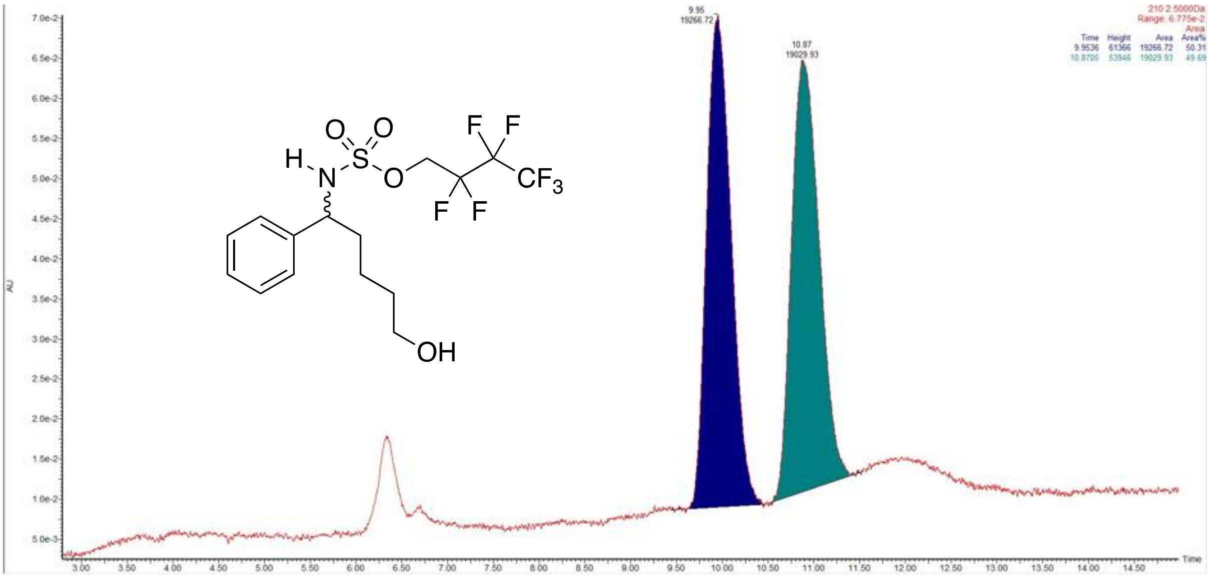

2,2,3,3,4,4,4-Heptafluorobutyl (4-acetamido-1-phenylbutyl)sulfamate

SFC Analysis CHIRALPAK IG (CO_2:MeOH, 96.5:3.5, 2.5 mL min^{-1}, 40 °C, 210 nm) indicated racemic, t_R = 9.5, 10.3 minutes

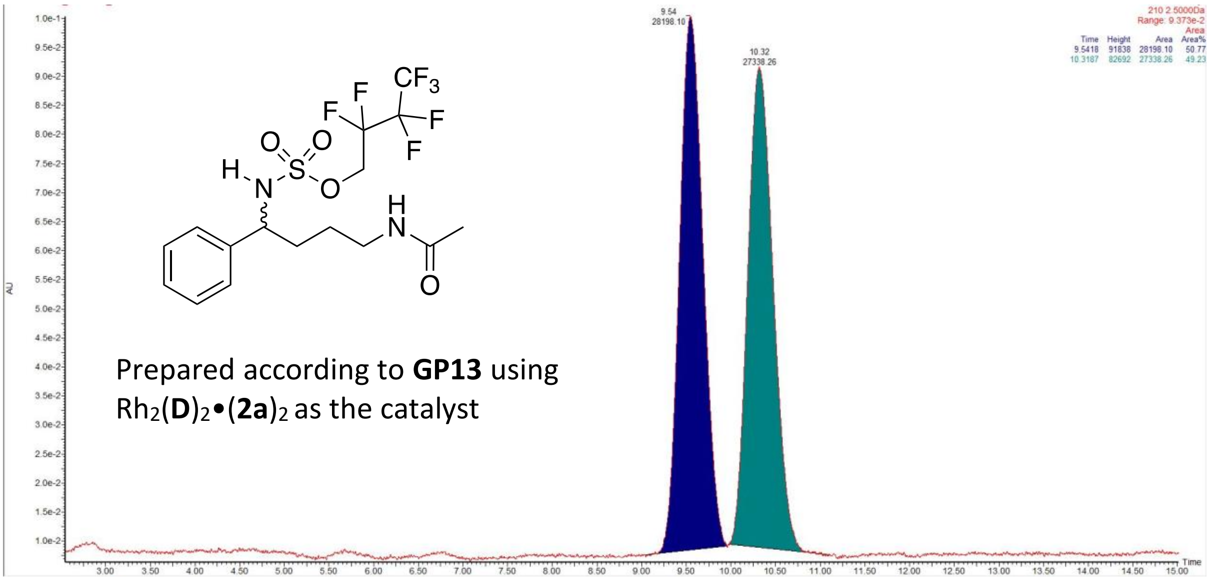

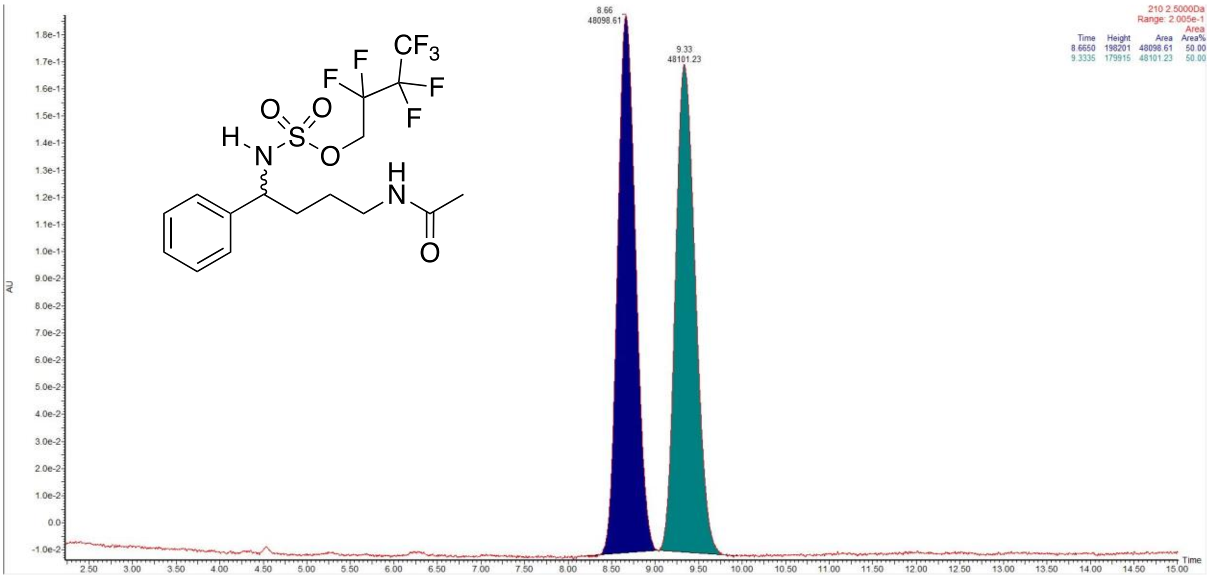

NMR Spectra

¹H NMR (400 MHz, $CDCl_3$) for *dimethyl 3,3'-(5-(bromomethyl)-1,3-phenylene)bis(2,2-dimethylpropanoate)*

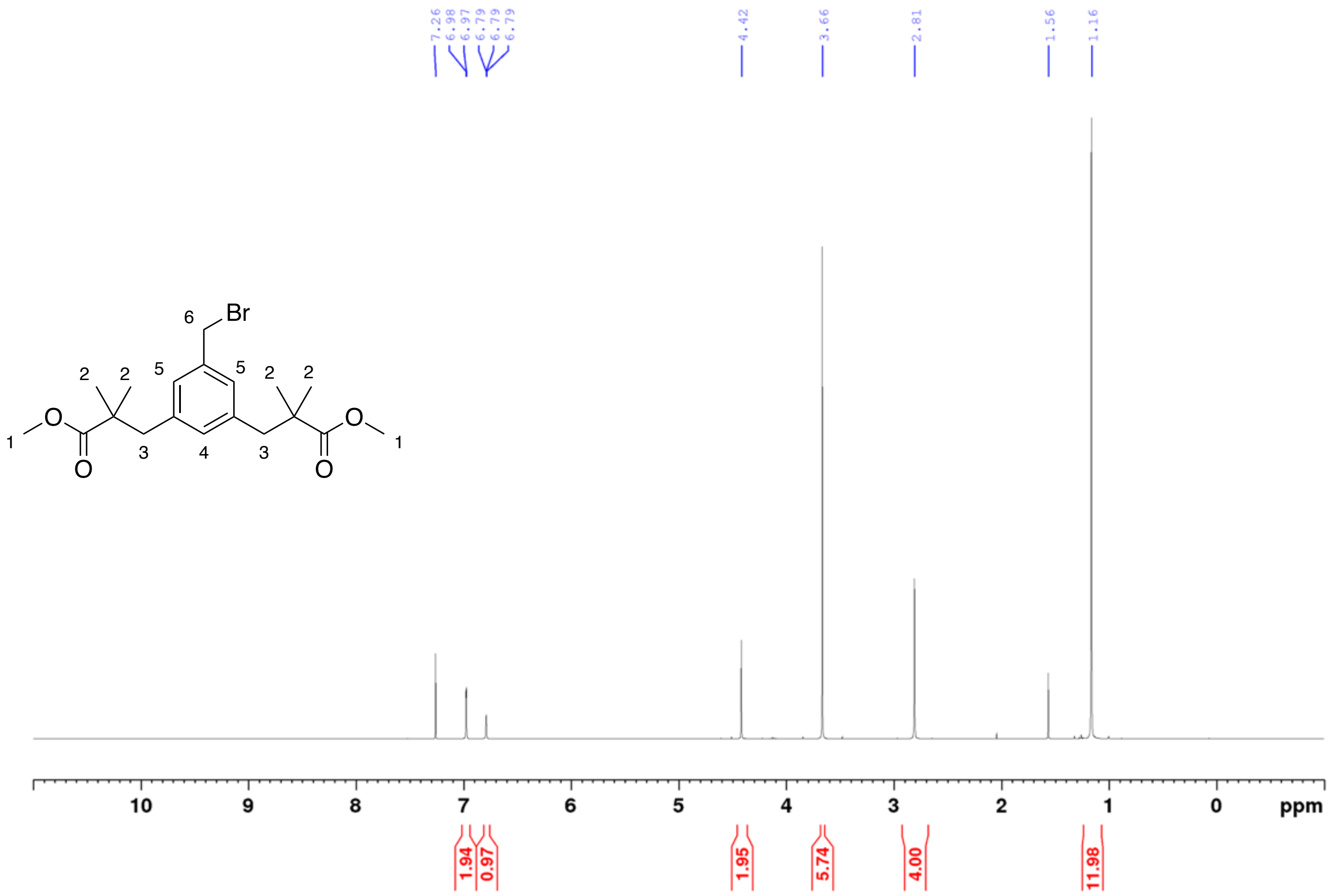

^{13}C NMR (101 MHz, $CDCl_3$) for *dimethyl 3,3'-(5-(bromomethyl)-1,3-phenylene)bis(2,2-dimethylpropanoate)*

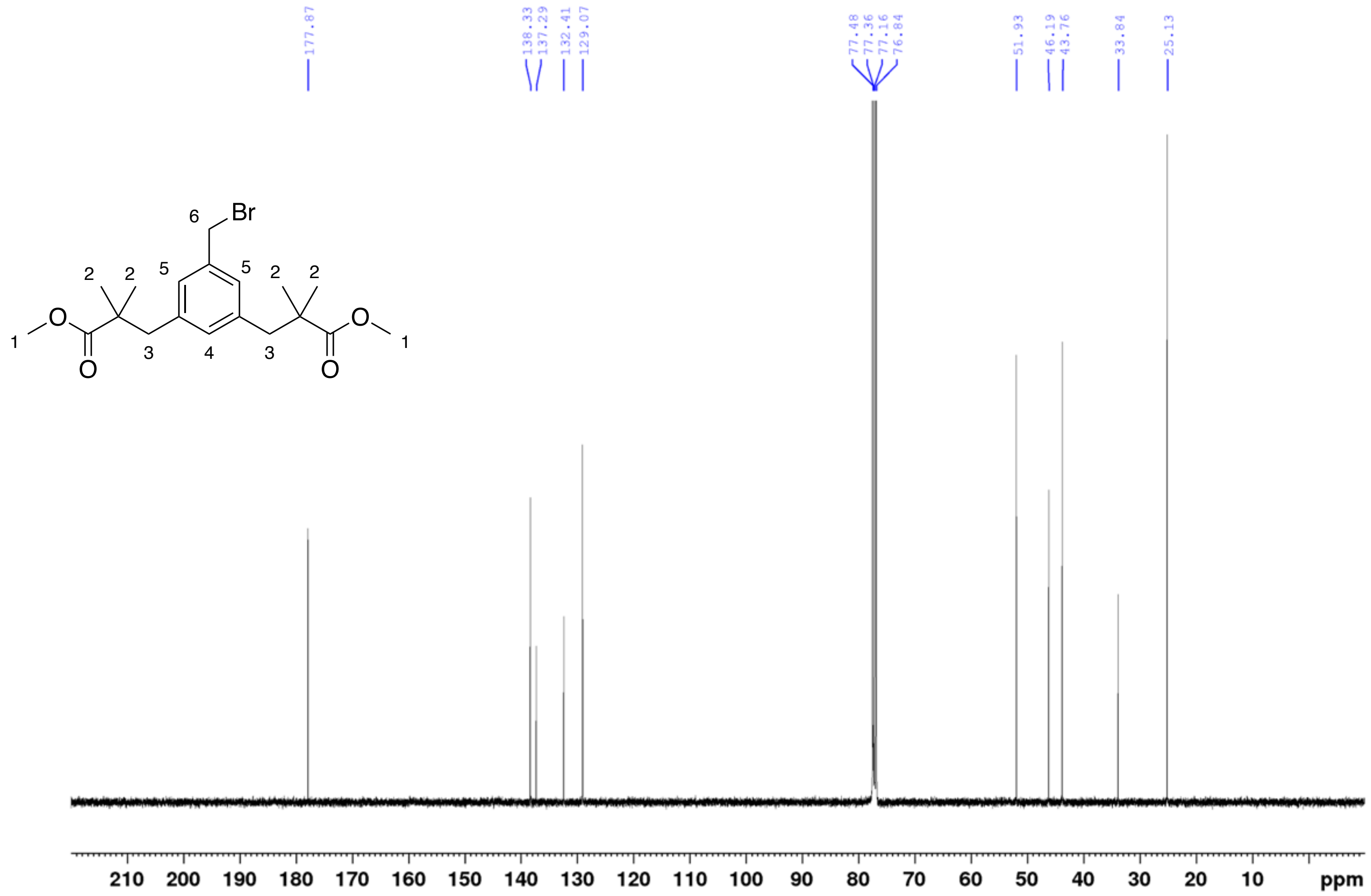

^{1}H NMR (400 MHz, $CDCl_3$) for *Tetrabutylammonium (3,5-bis(3-methoxy-2,2-dimethyl-3-oxopropyl)phenyl)methanesulfonate*

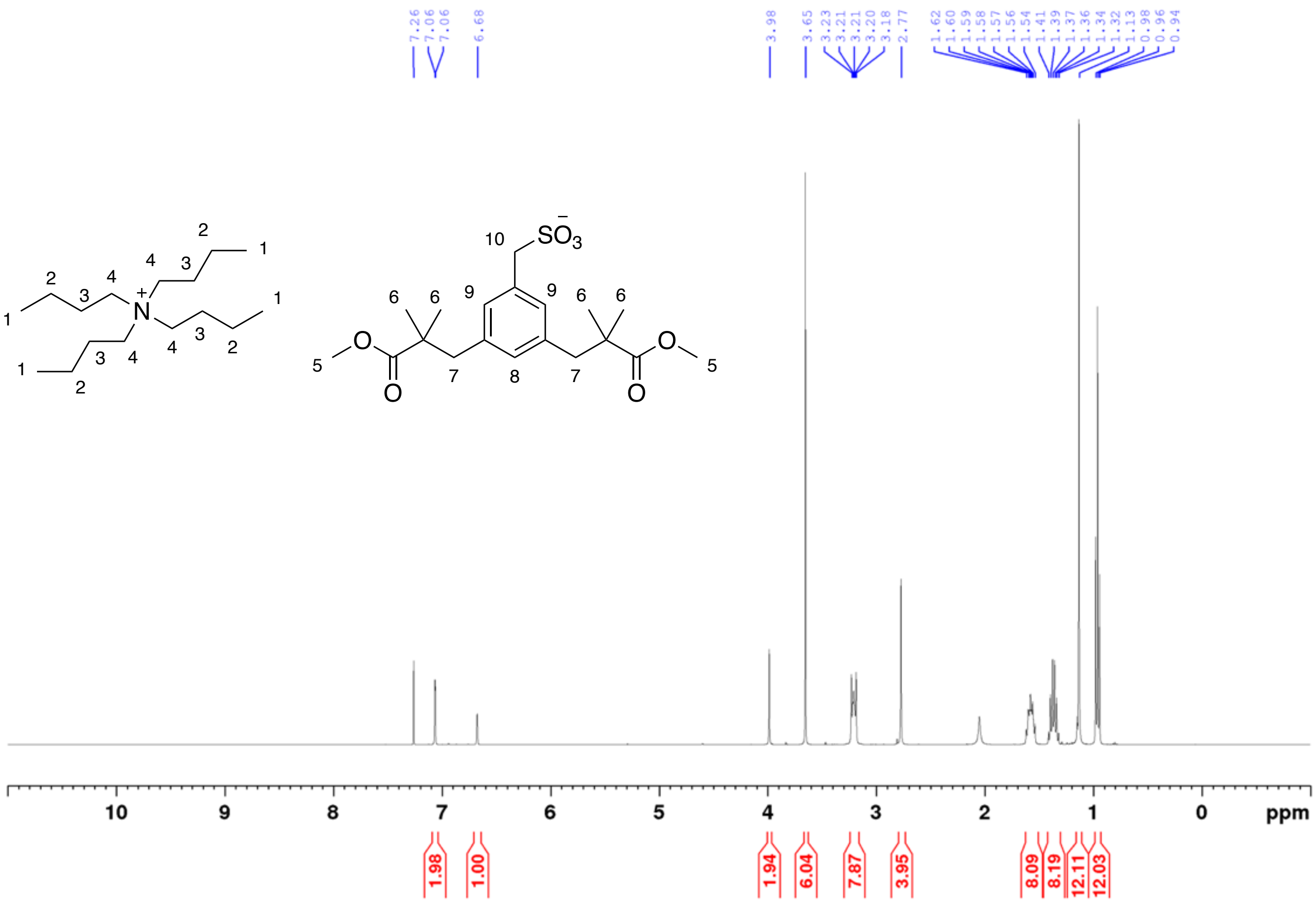

^{13}C NMR (101 MHz, $CDCl_3$) for *Tetrabutylammonium (3,5-bis(3-methoxy-2,2-dimethyl-3-oxopropyl)phenyl)methanesulfonate*

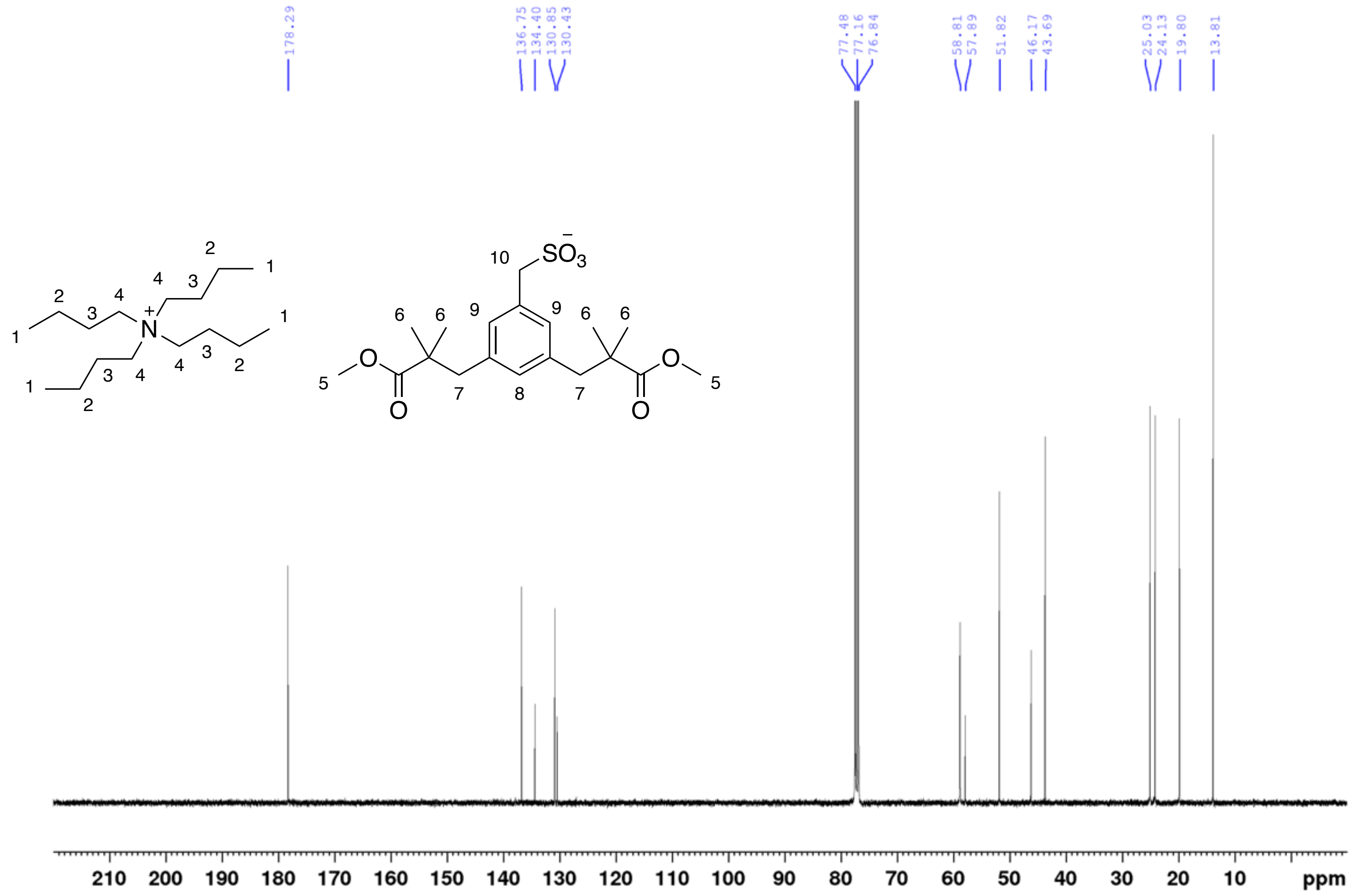

^{1}H NMR (400 MHz, MeOD) for *Tetrabutylammonium (3,5-bis(2-carboxy-2-methylpropyl)phenyl)methanesulfonate* (**A•Bu$_4$N**)

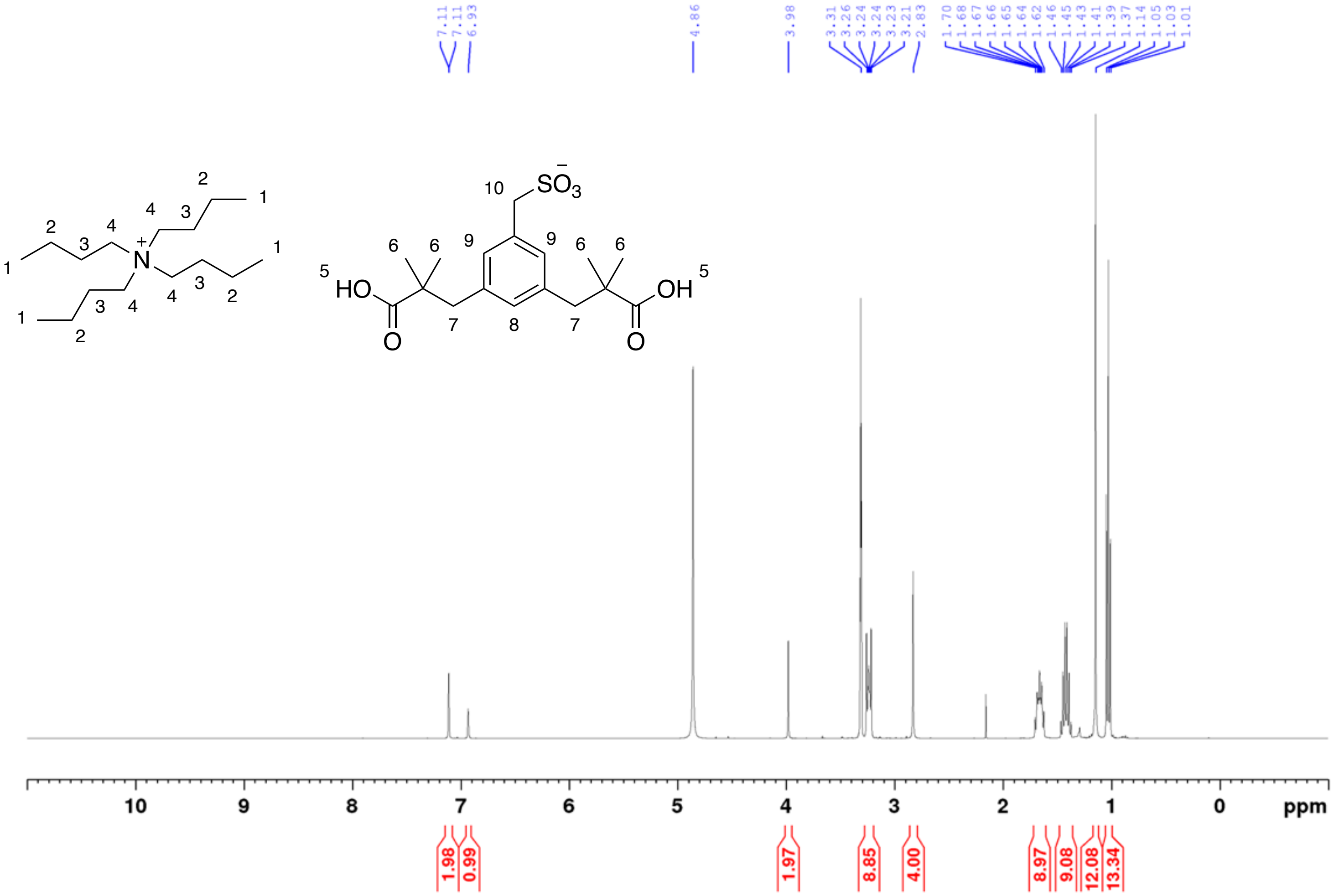

^{13}C NMR (101 MHz, MeOD) for *Tetrabutylammonium (3,5-bis(2-carboxy-2-methylpropyl)phenyl)methanesulfonate* (**A•Bu$_4$N**)

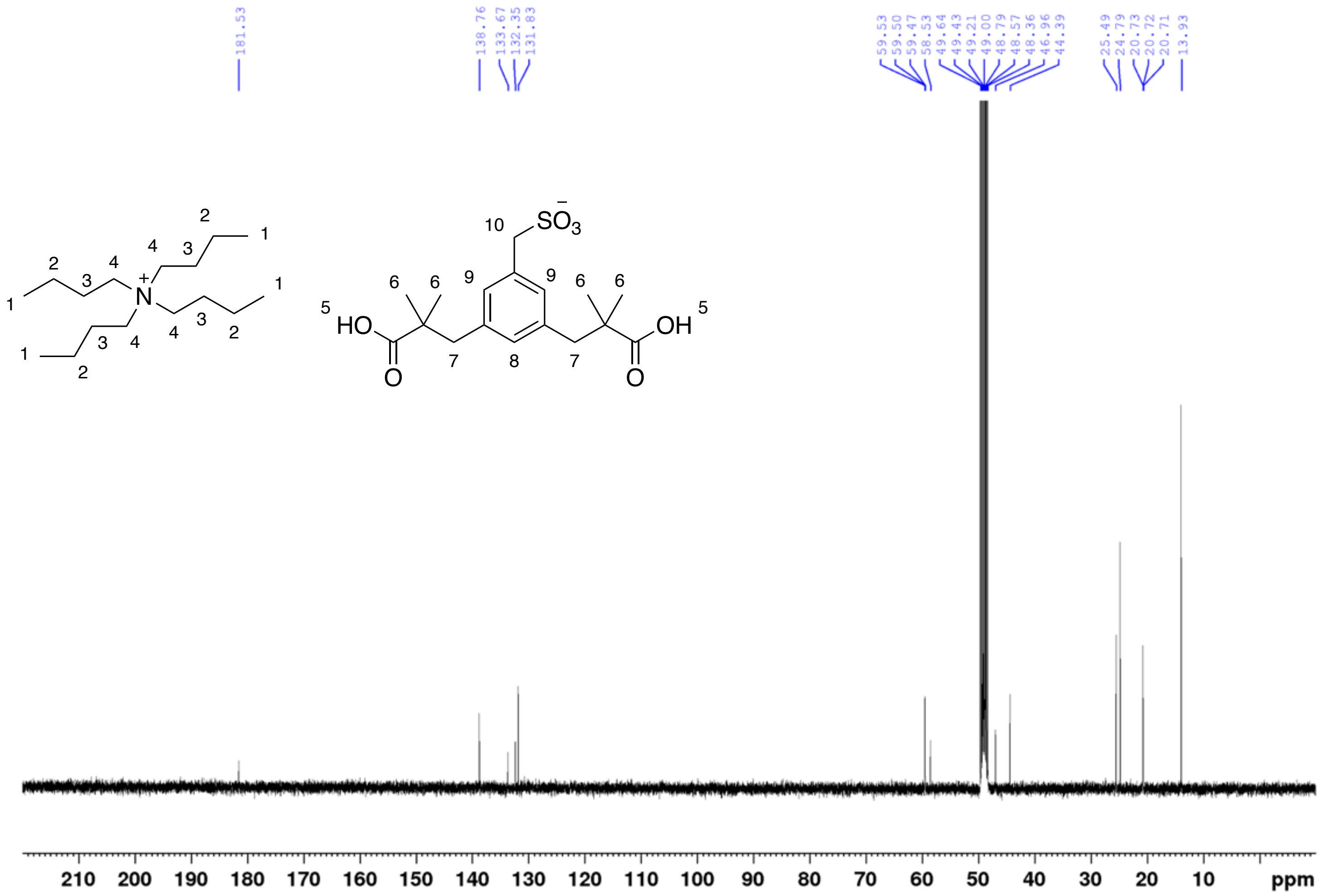

^{1}H NMR (600 MHz, MeOD) for *Bis[rhodium Tetrabutylammonium (3,5-bis(2-carboxy-2-methylpropyl)phenyl)methanesulfonate)]* (Rh$_2$(**A**)$_2$•(**Bu$_4$N**)$_2$)

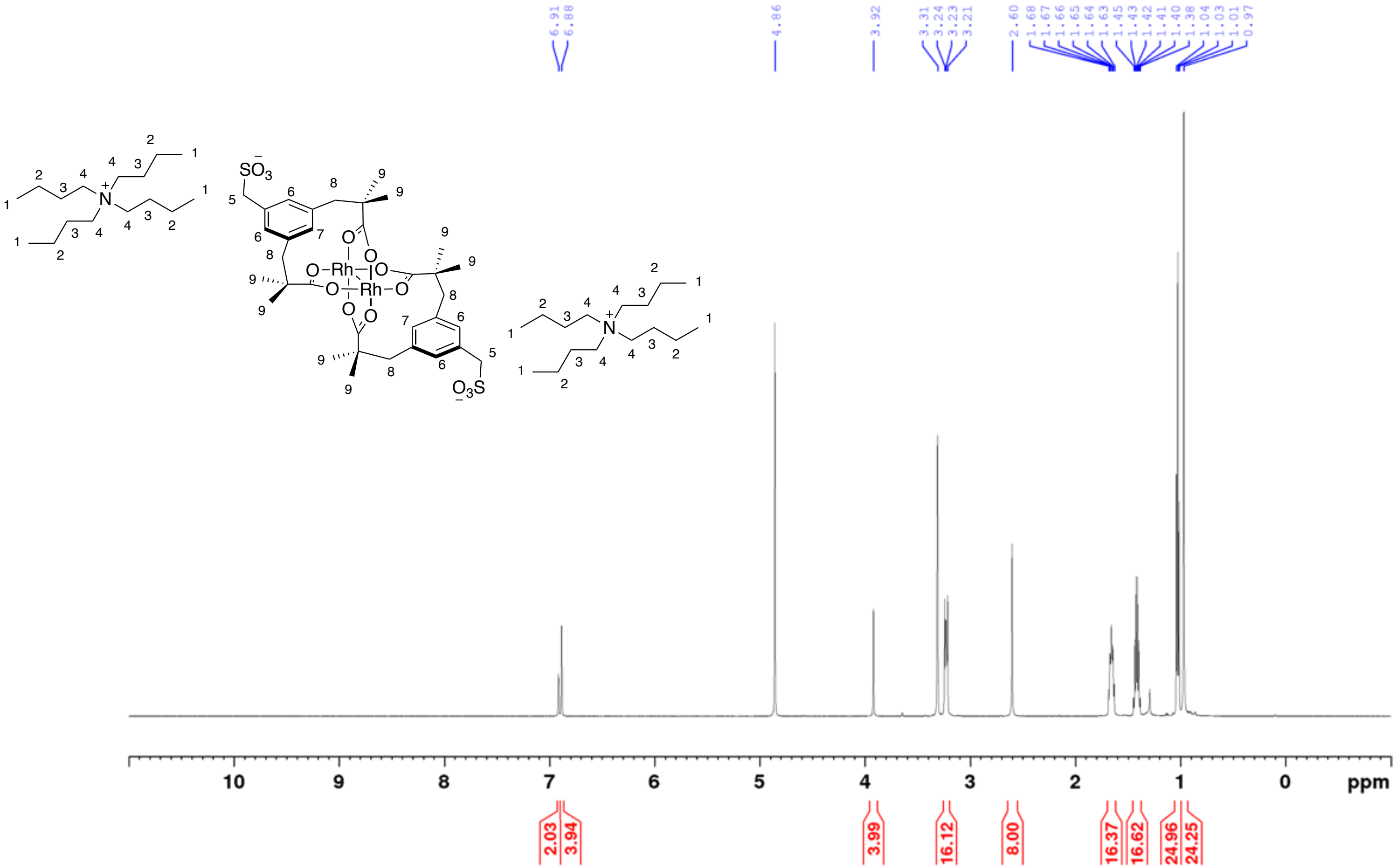

^{12}**C NMR** (101 MHz, MeOD) for *Bis[rhodium Tetrabutylammonium (3,5-bis(2-carboxy-2-methylpropyl)phenyl)methanesulfonate)]* (Rh_2(**A**)$_2$•(**Bu_4N**)$_2$)

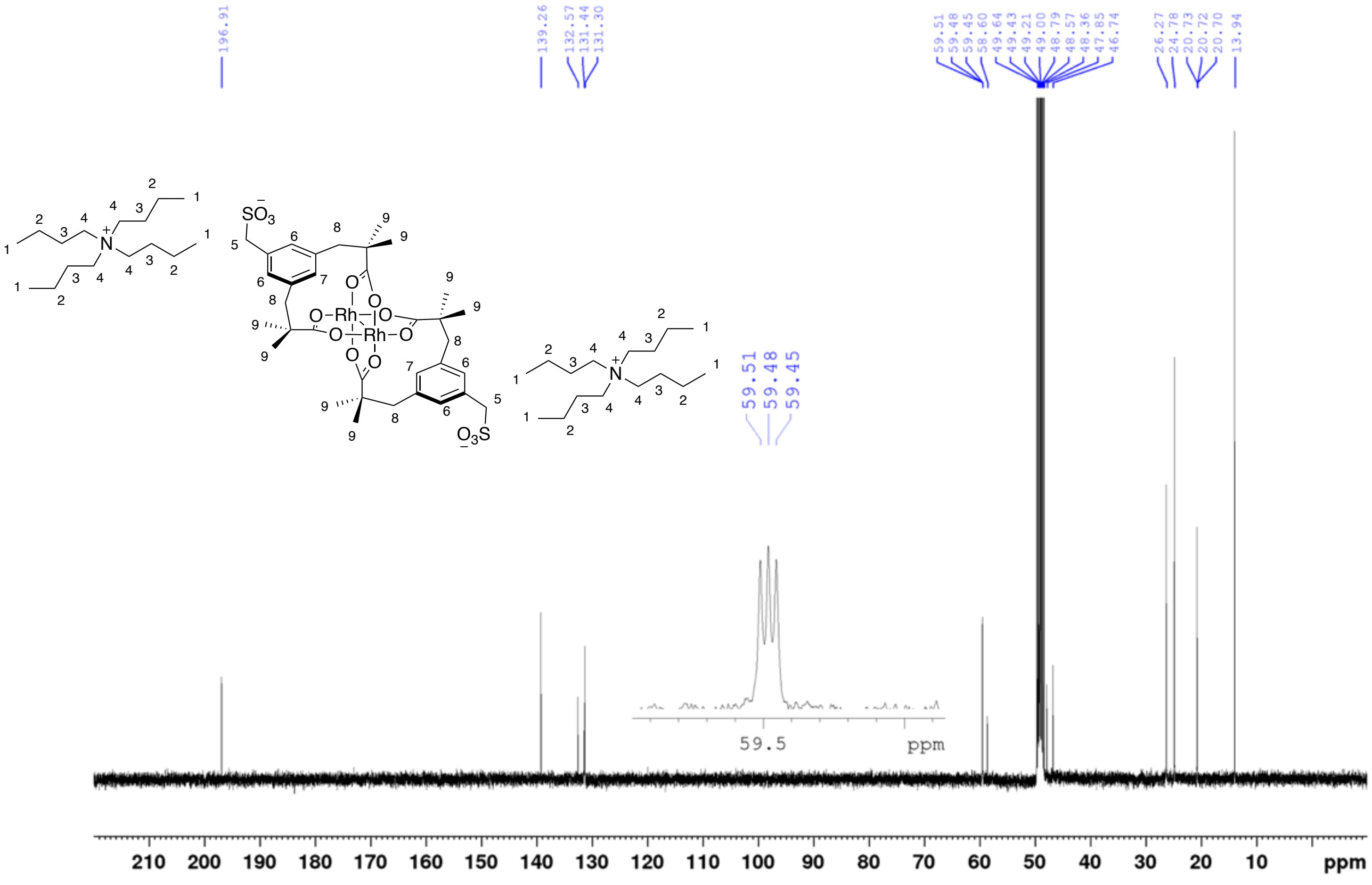

^{1}H NMR (500 MHz, $CDCl_3$) for *diethyl 1,1'-((5-(bromomethyl)-1,3-phenylene)bis(methylene))bis(cyclobutane-1-carboxylate)*

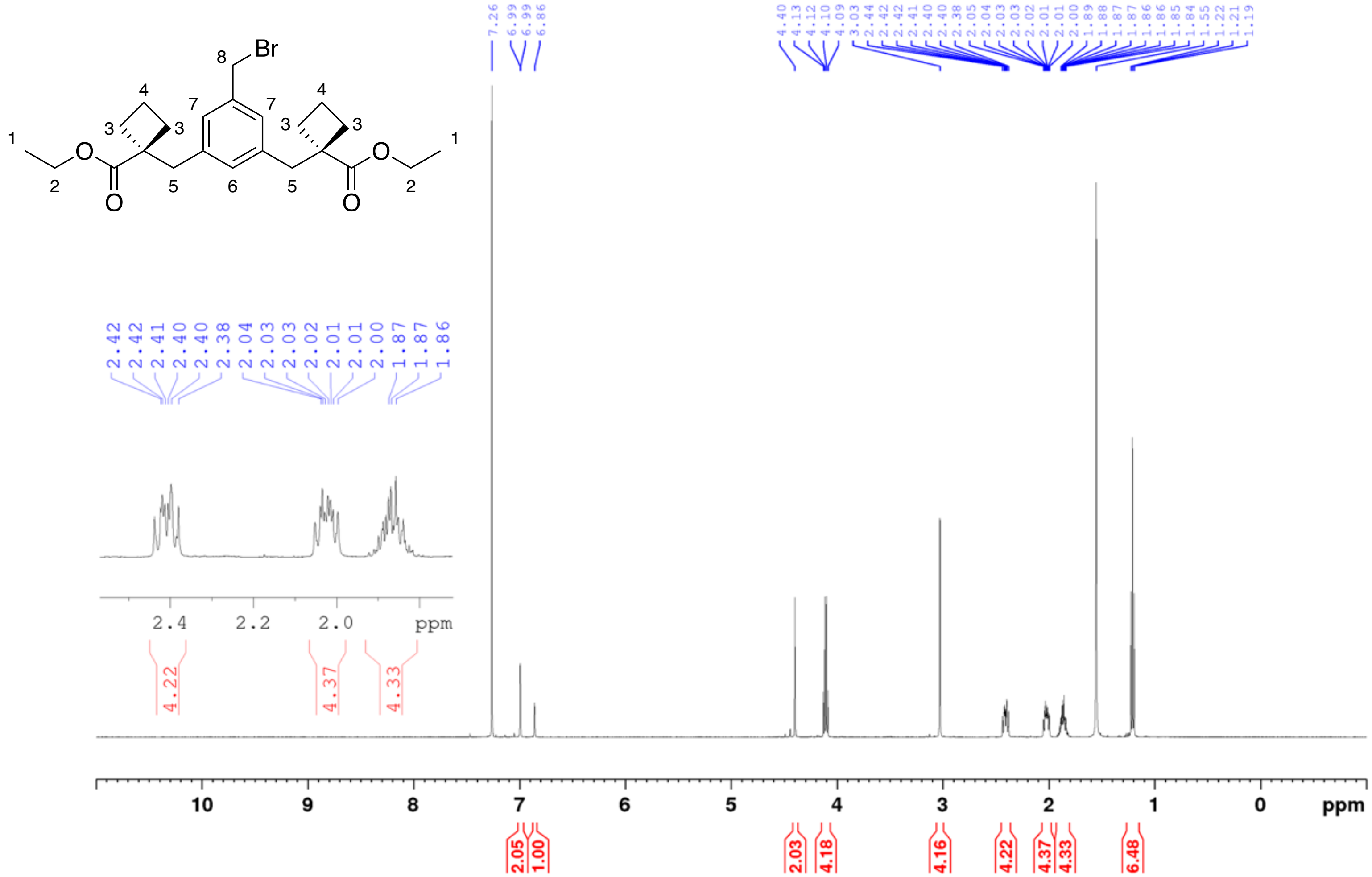

^{13}C NMR (126 MHz, $CDCl_3$) for *diethyl 1,1'-((5-(bromomethyl)-1,3-phenylene)bis(methylene))bis(cyclobutane-1-carboxylate)*

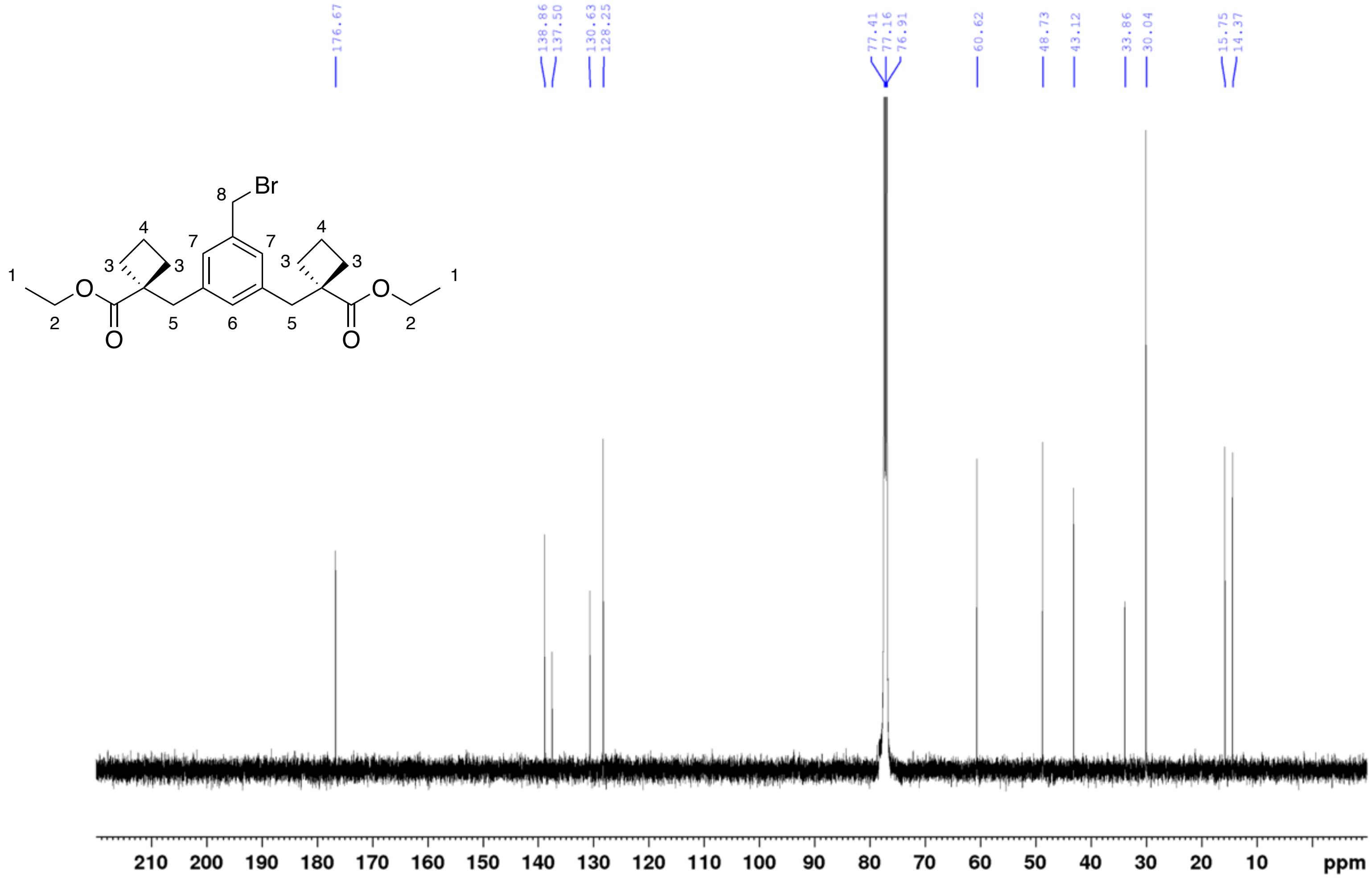

^{1}H NMR (500 MHz, $CDCl_3$) for *Tetrabutylammonium (3,5-bis((1-(ethoxycarbonyl)cyclobutyl)methyl)phenyl)methanesulfonate*

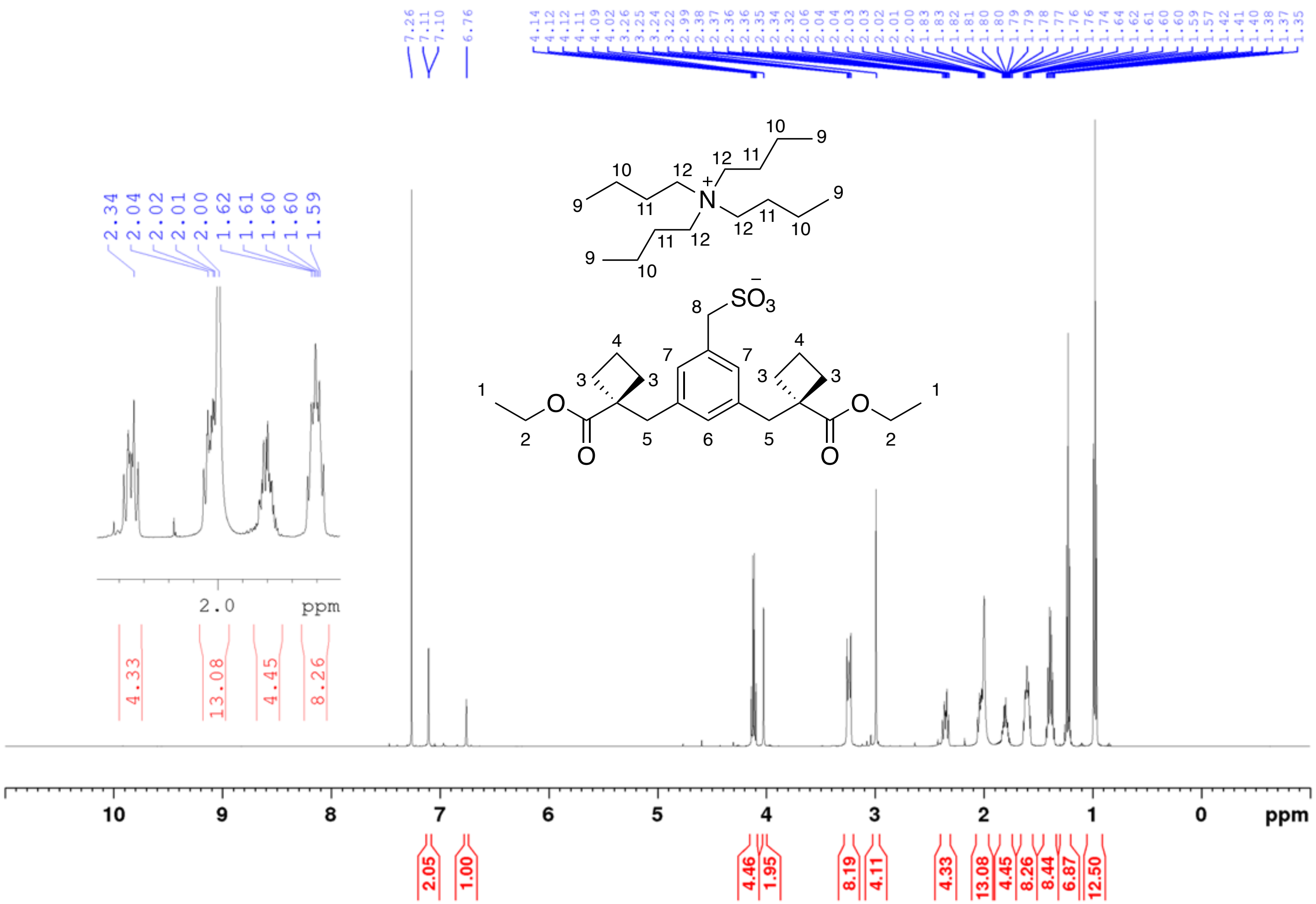

^{13}C NMR (126 MHz, $CDCl_3$) for *Tetrabutylammonium (3,5-bis((1-(ethoxycarbonyl)cyclobutyl)methyl)phenyl)methanesulfonate*

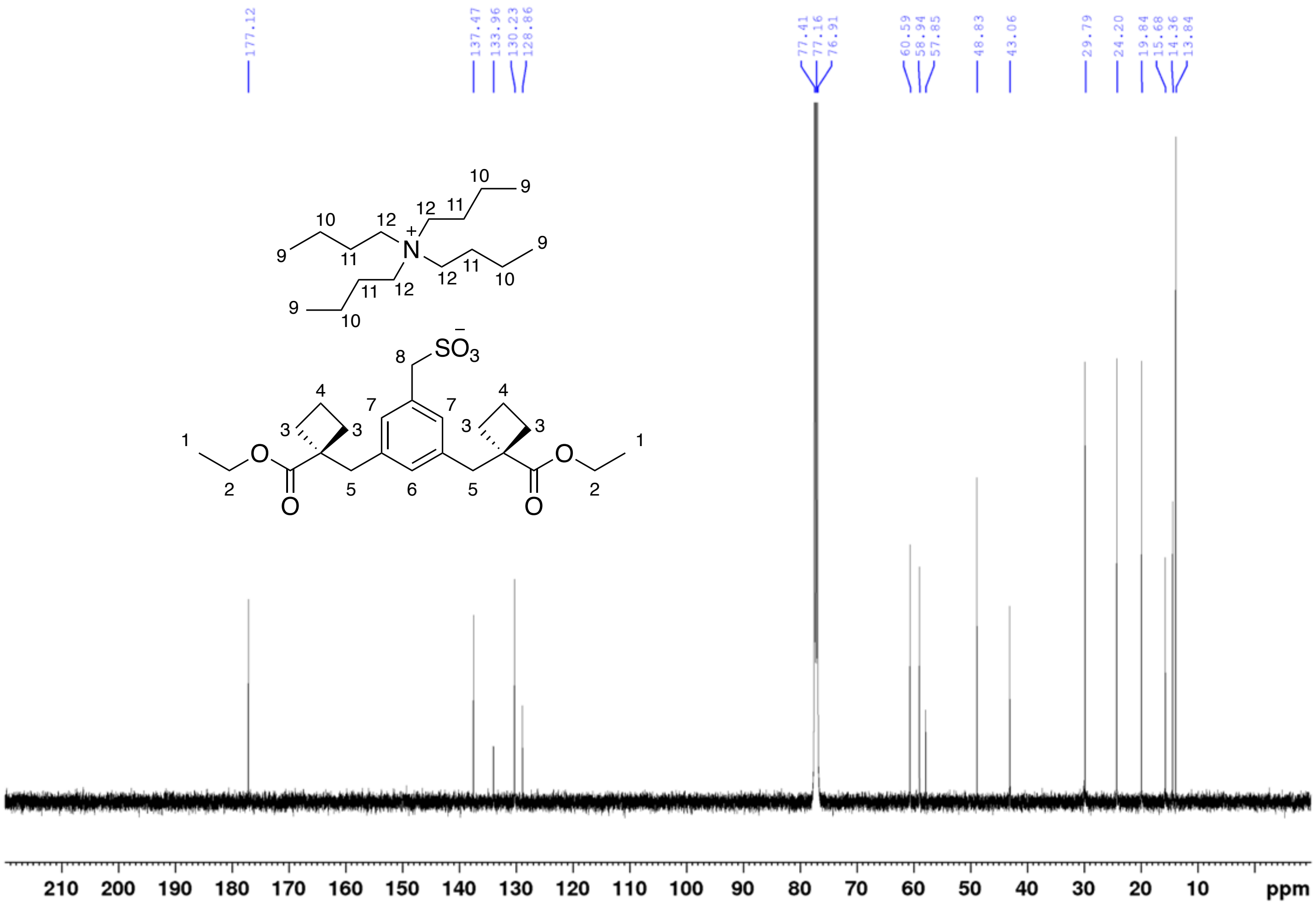

^{1}H NMR (500 MHz, CD_3CN + drops of MeOH) for *Tetrabutylammonium (3,5-bis((1-carboxycyclobutyl)methyl)phenyl)methanesulfonate* (**B•Bu$_4$N**)

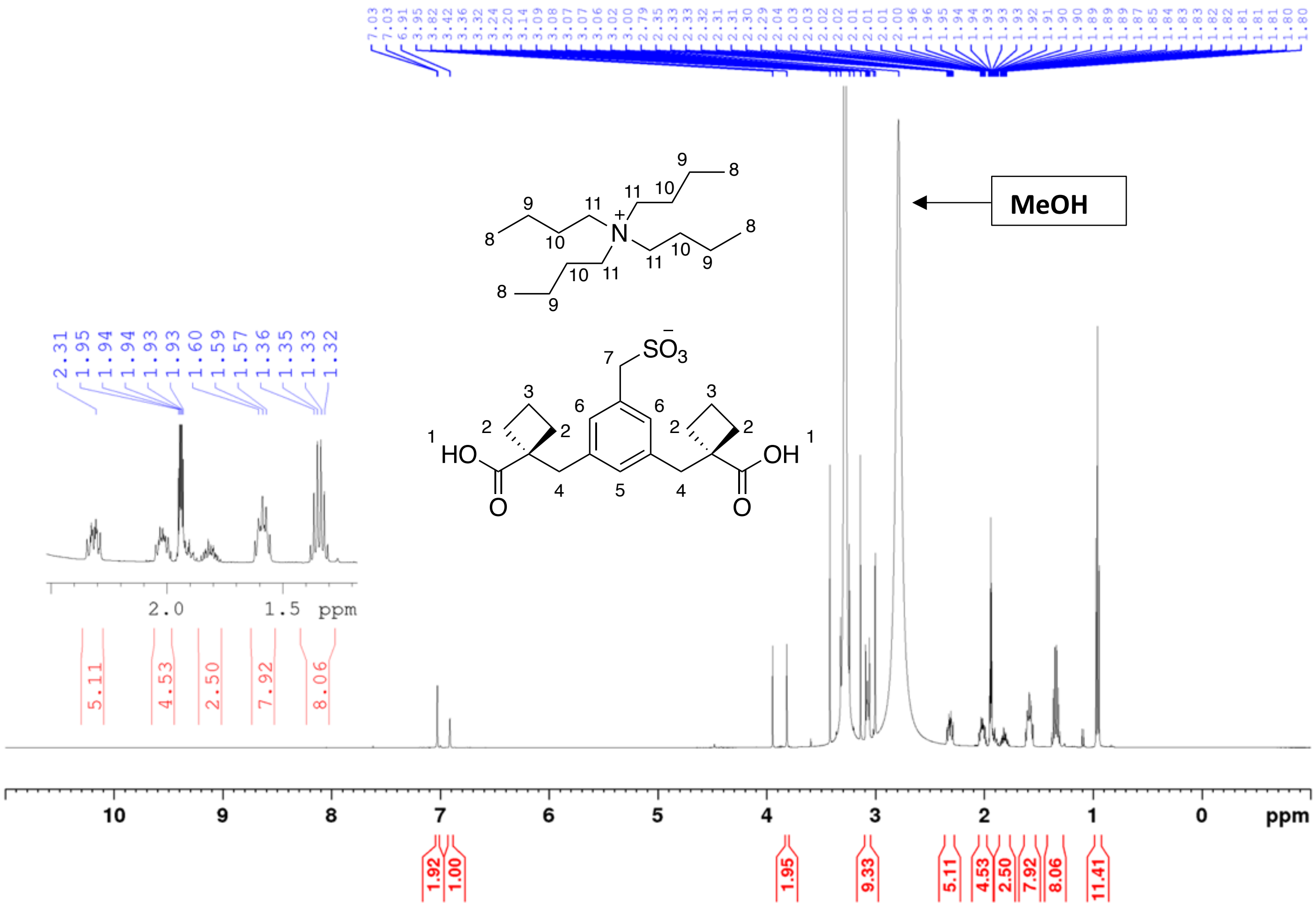

^{13}C NMR (126 MHz, CD_3CN + drops of MeOH) for *Tetrabutylammonium (3,5-bis((1-carboxycyclobutyl)methyl)phenyl)methanesulfonate* (**B•Bu_4N**)

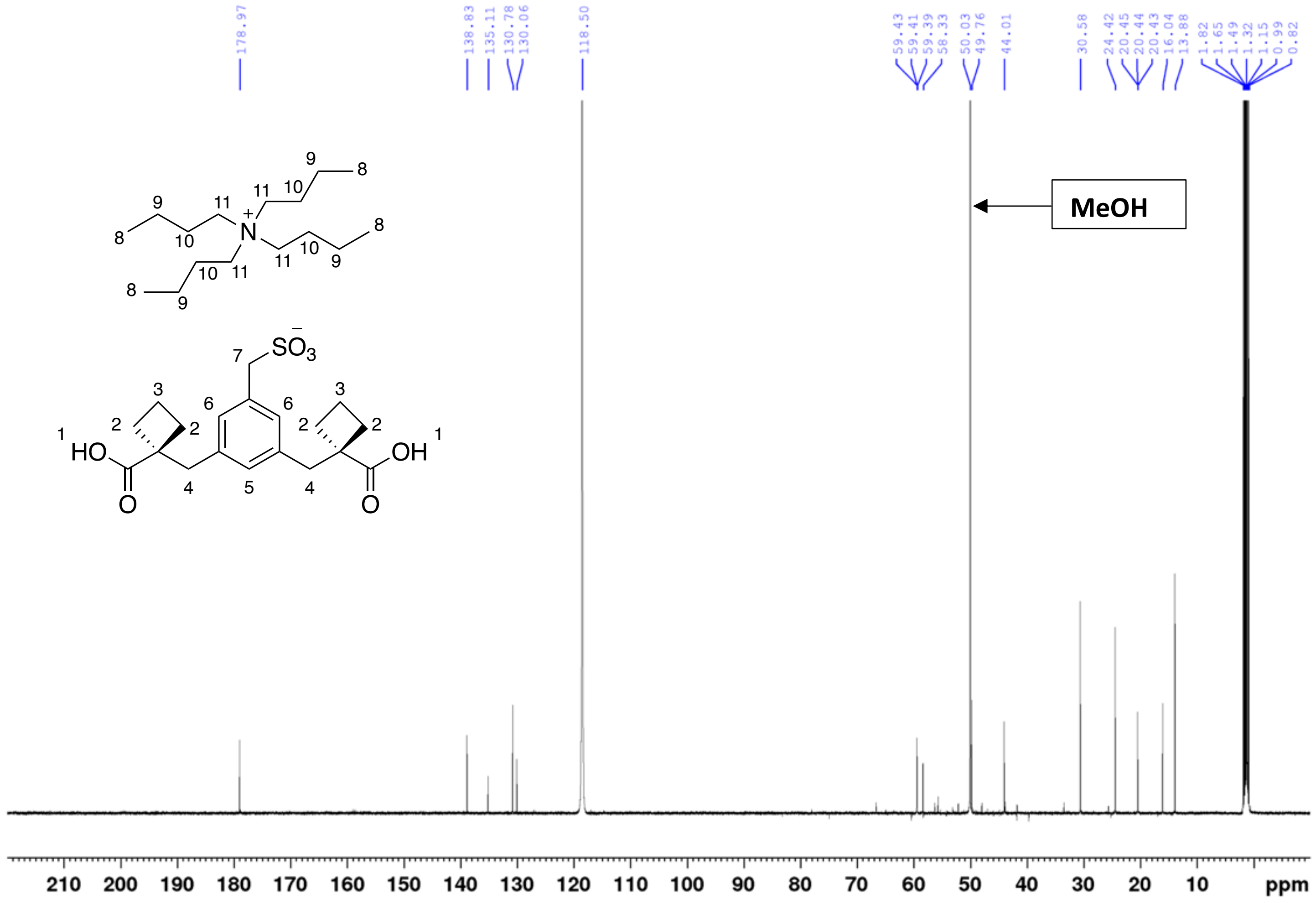

¹H NMR (500 MHz, C_5D_5N) for *Bis[rhodium Tetrabutylammonium (3,5-bis((1-carboxycyclobutyl)methyl)phenyl)methanesulfonate* (Rh_2(**B**)$_2$•(**Bu$_4$N**)$_2$)

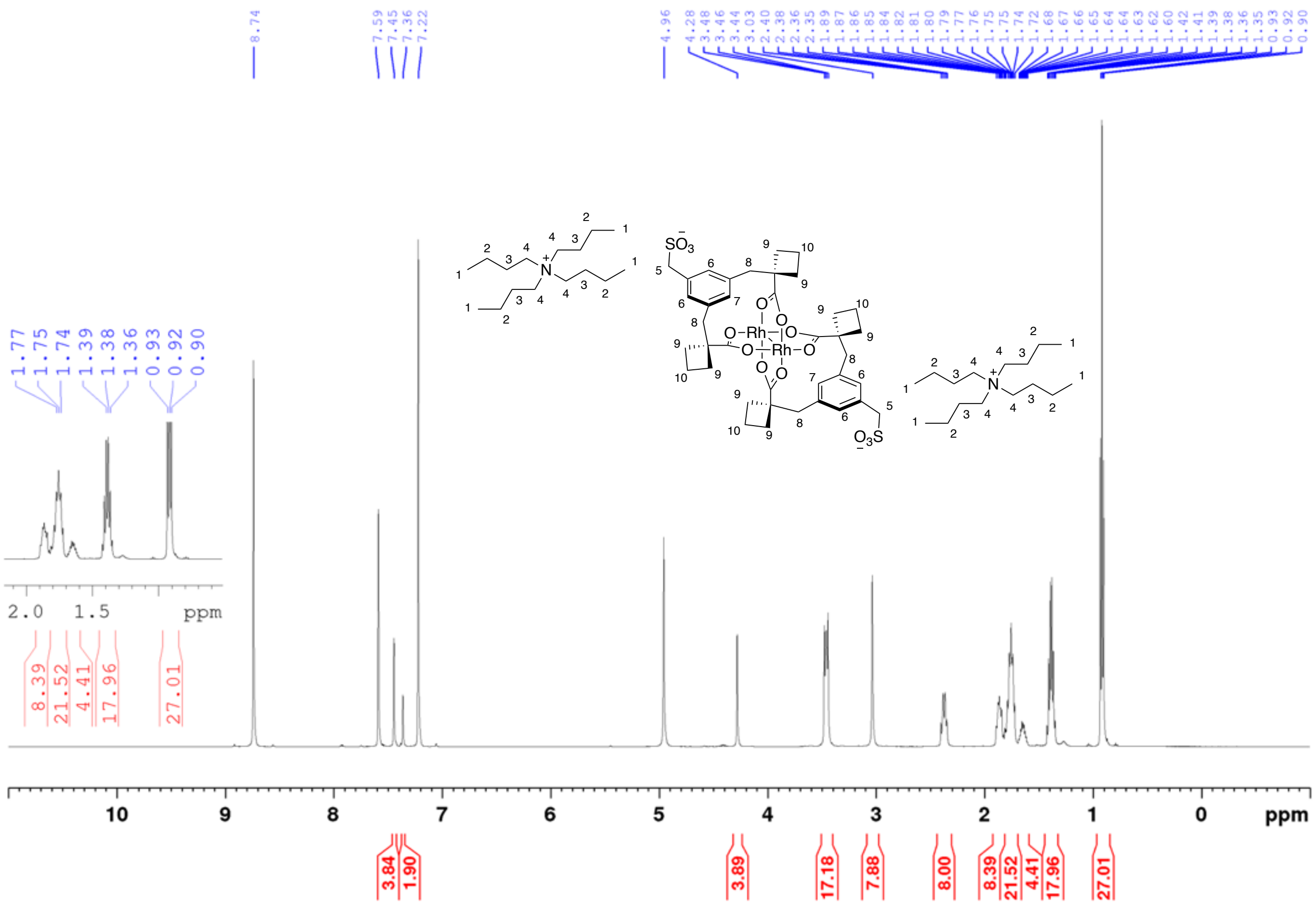

^{13}C NMR (126 MHz, C_5D_5N) for *Bis[rhodium Tetrabutylammonium (3,5-bis((1-carboxycyclobutyl)methyl)phenyl)methanesulfonate* (Rh_2(**B**)$_2$•(**Bu_4N**)$_2$)

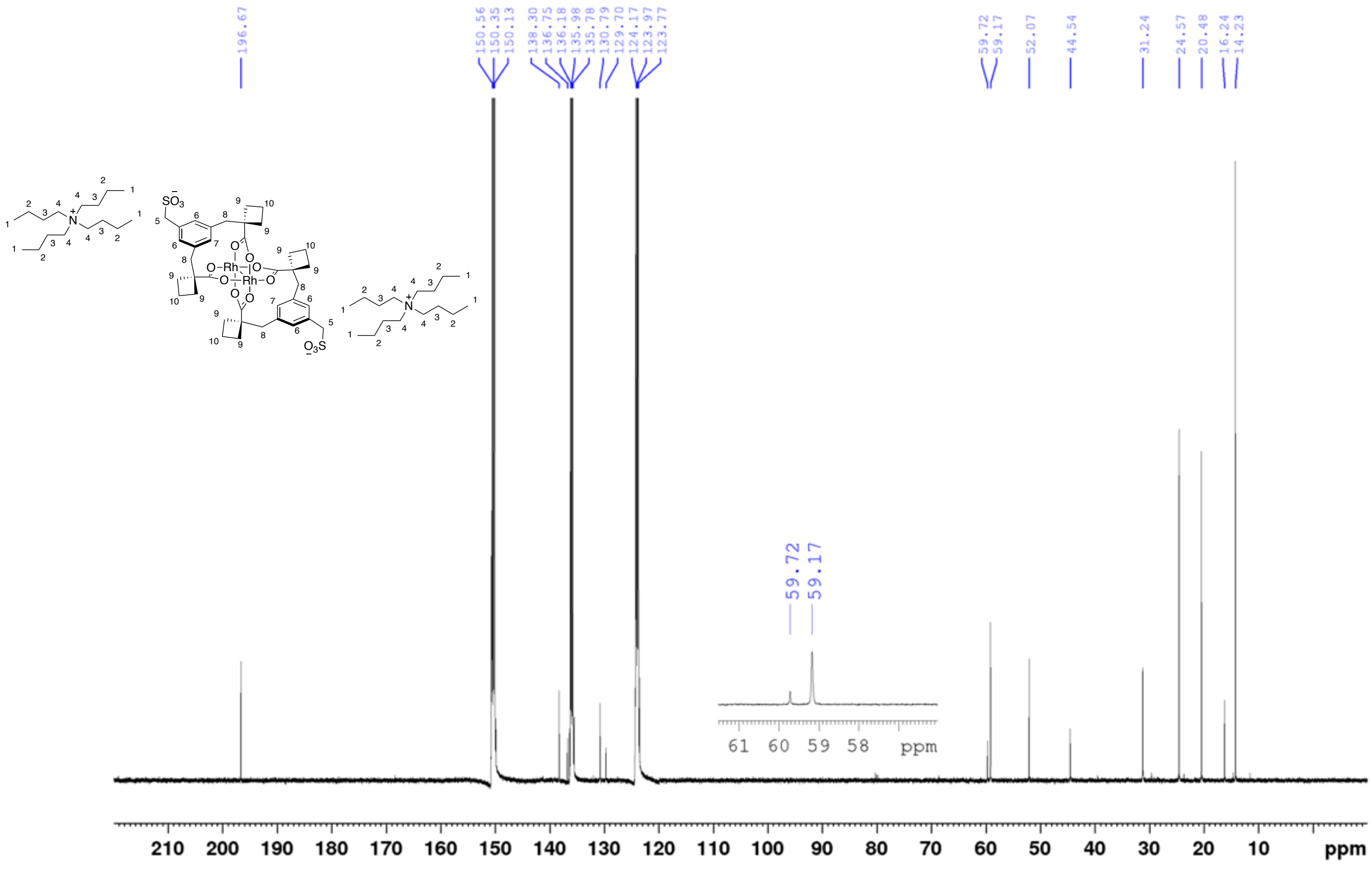

^{1}H NMR (400 MHz, $CDCl_3$) for *dimethyl 1,1'-((5-(bromomethyl)-1,3-phenylene)bis(methylene))bis(cyclopentane-1-carboxylate)*

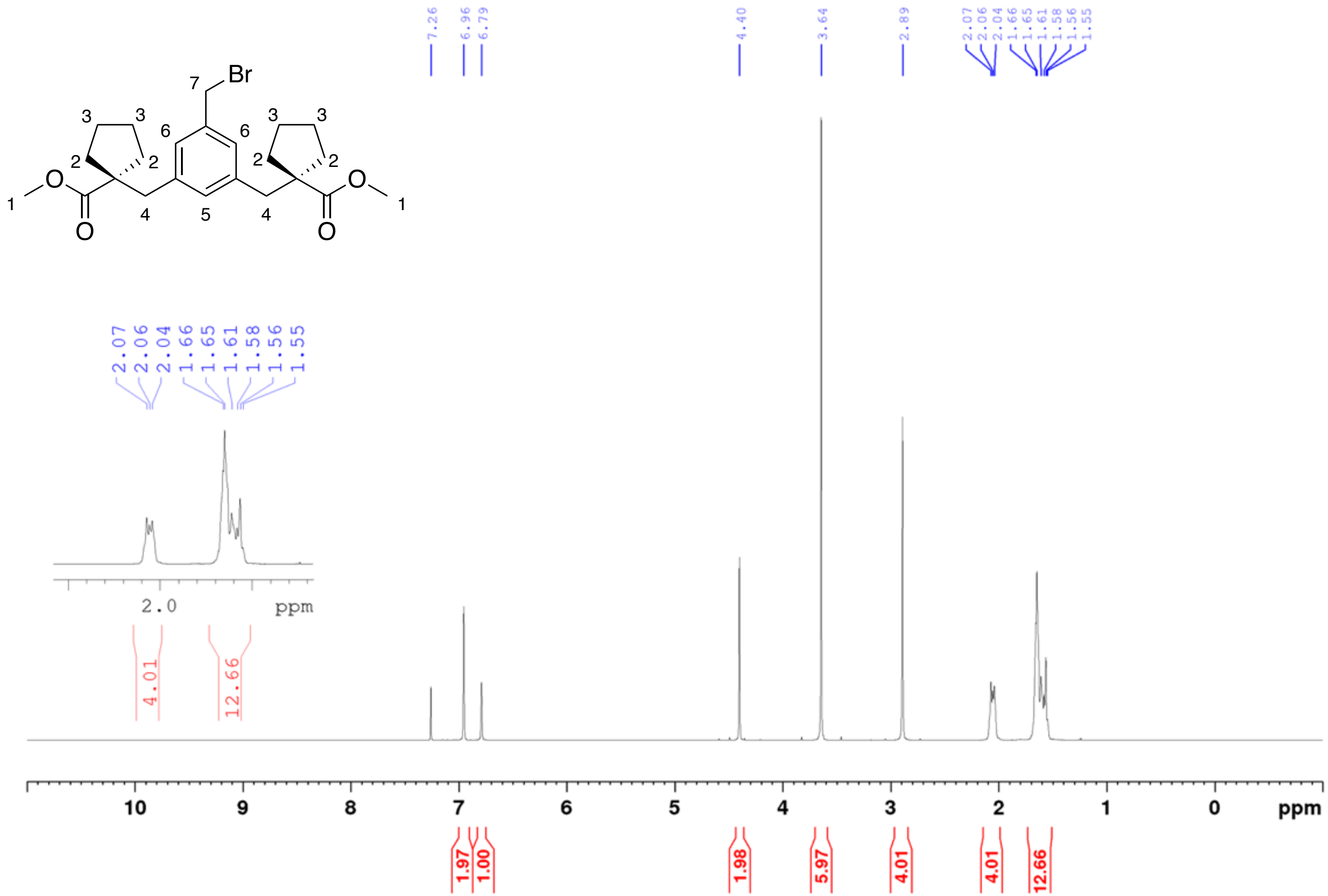

^{13}C NMR (101 MHz, $CDCl_3$) for *dimethyl 1,1'-((5-(bromomethyl)-1,3-phenylene)bis(methylene))bis(cyclopentane-1-carboxylate)*

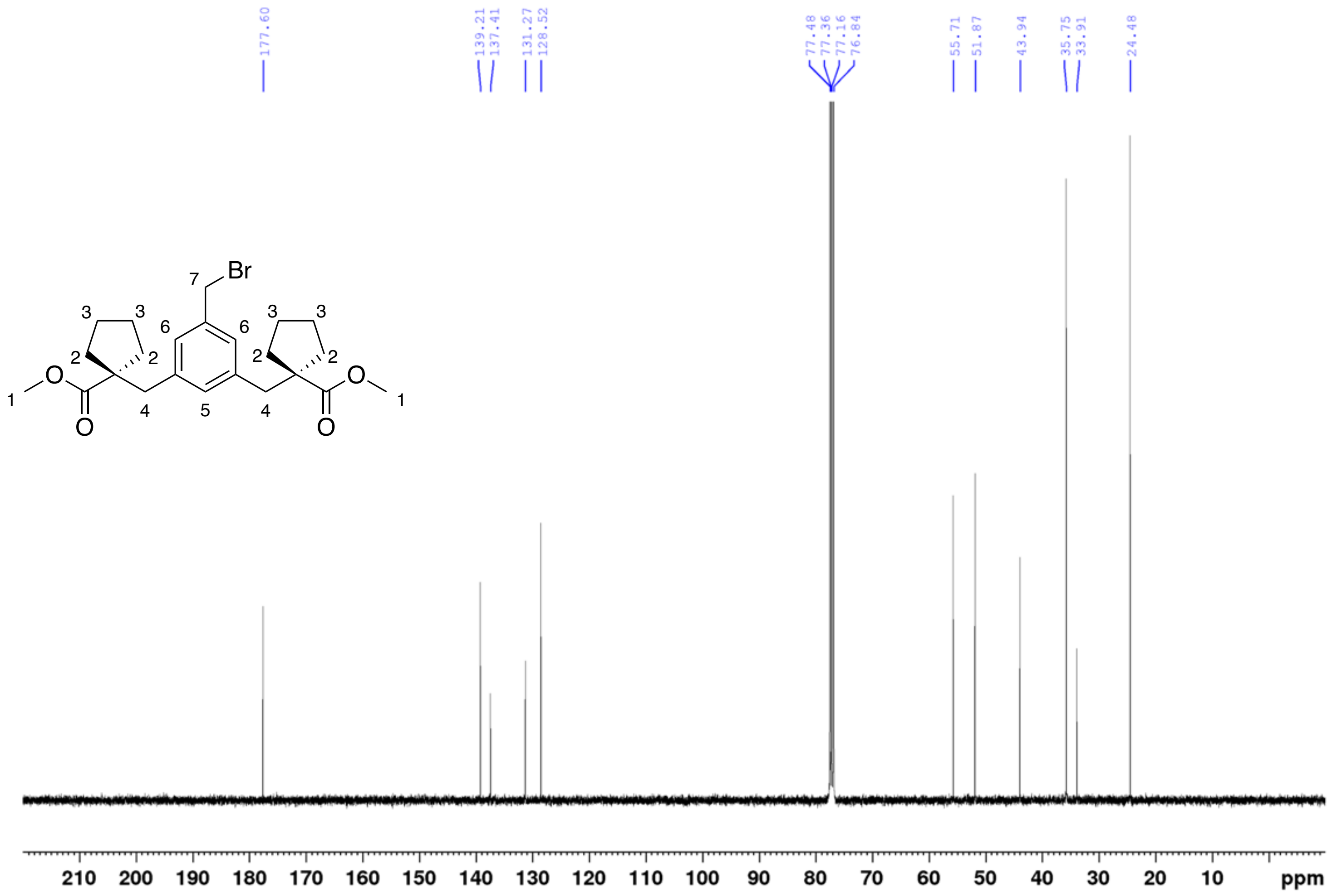

¹H NMR (400 MHz, $CDCl_3$) for *Tetrabutylammonium (3,5-bis((1-(methoxycarbonyl)cyclopentyl)methyl)phenyl)methanesulfonate*

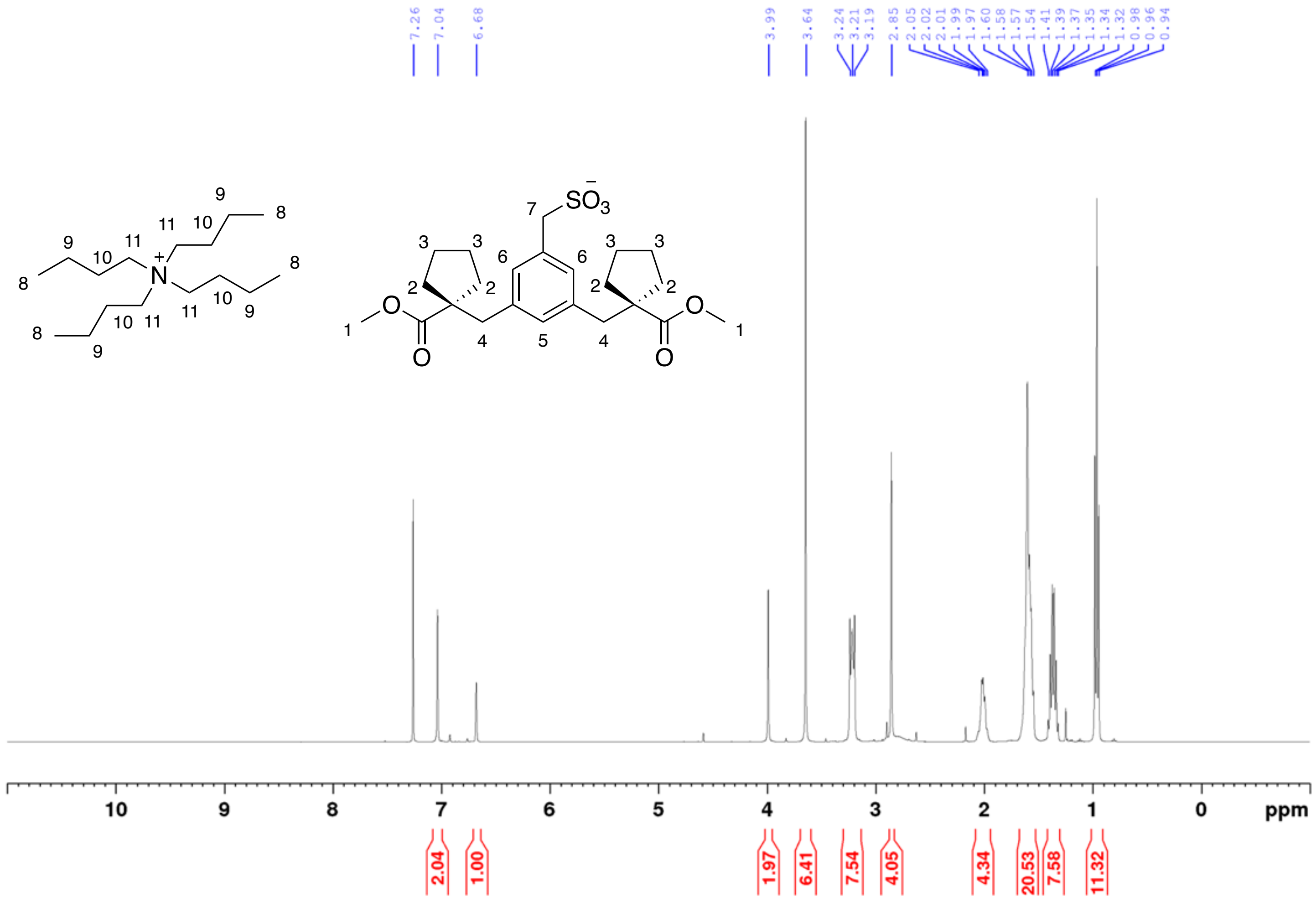

^{13}C NMR (101 MHz, $CDCl_3$) for *Tetrabutylammonium (3,5-bis((1-(methoxycarbonyl)cyclopentyl)methyl)phenyl)methanesulfonate*

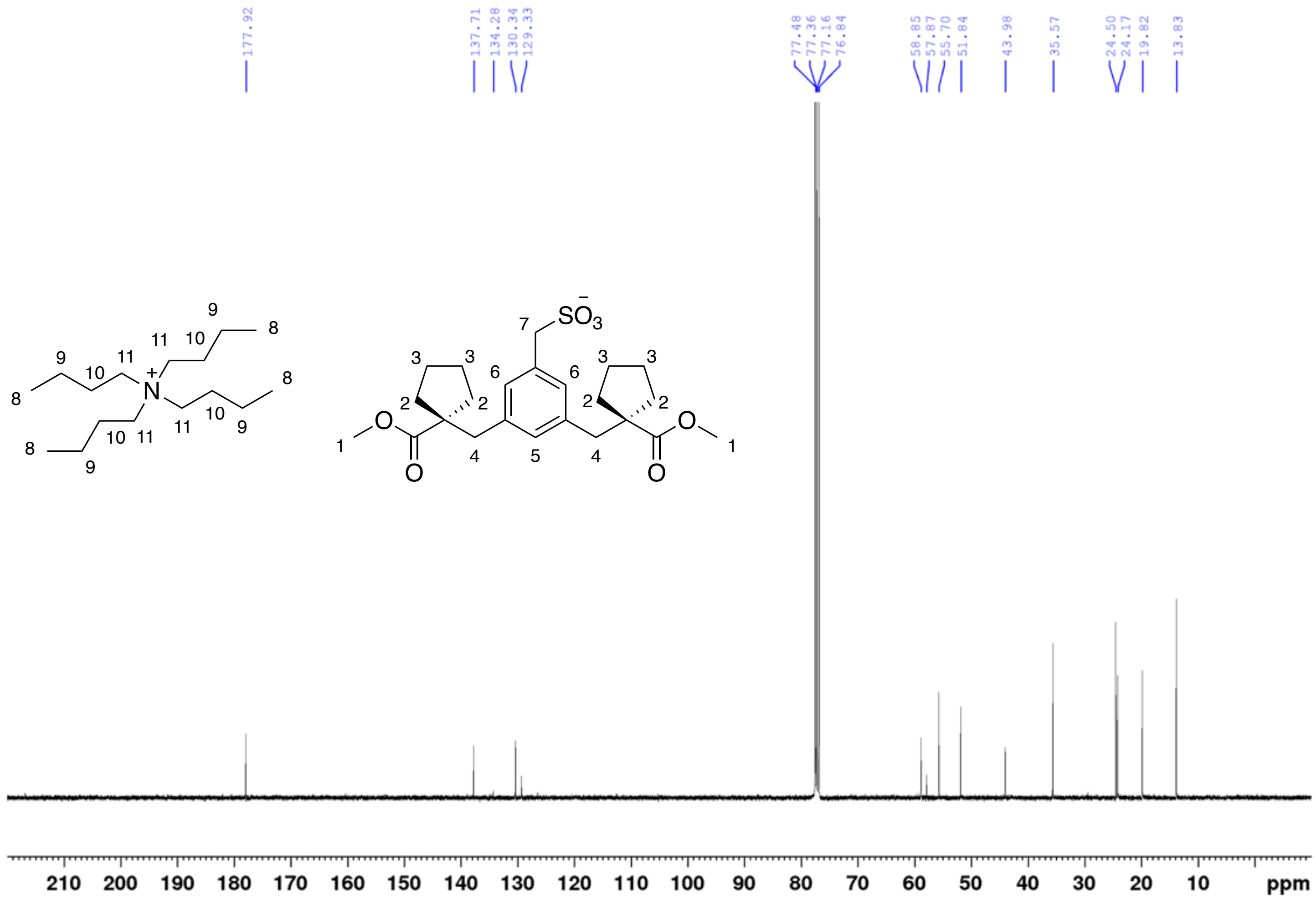

^{1}H NMR (400 MHz, MeOD) for *Tetrabutylammonium (3,5-bis((1-carboxycyclopentyl)methyl)phenyl)methanesulfonate* (**C•Bu$_4$N**)

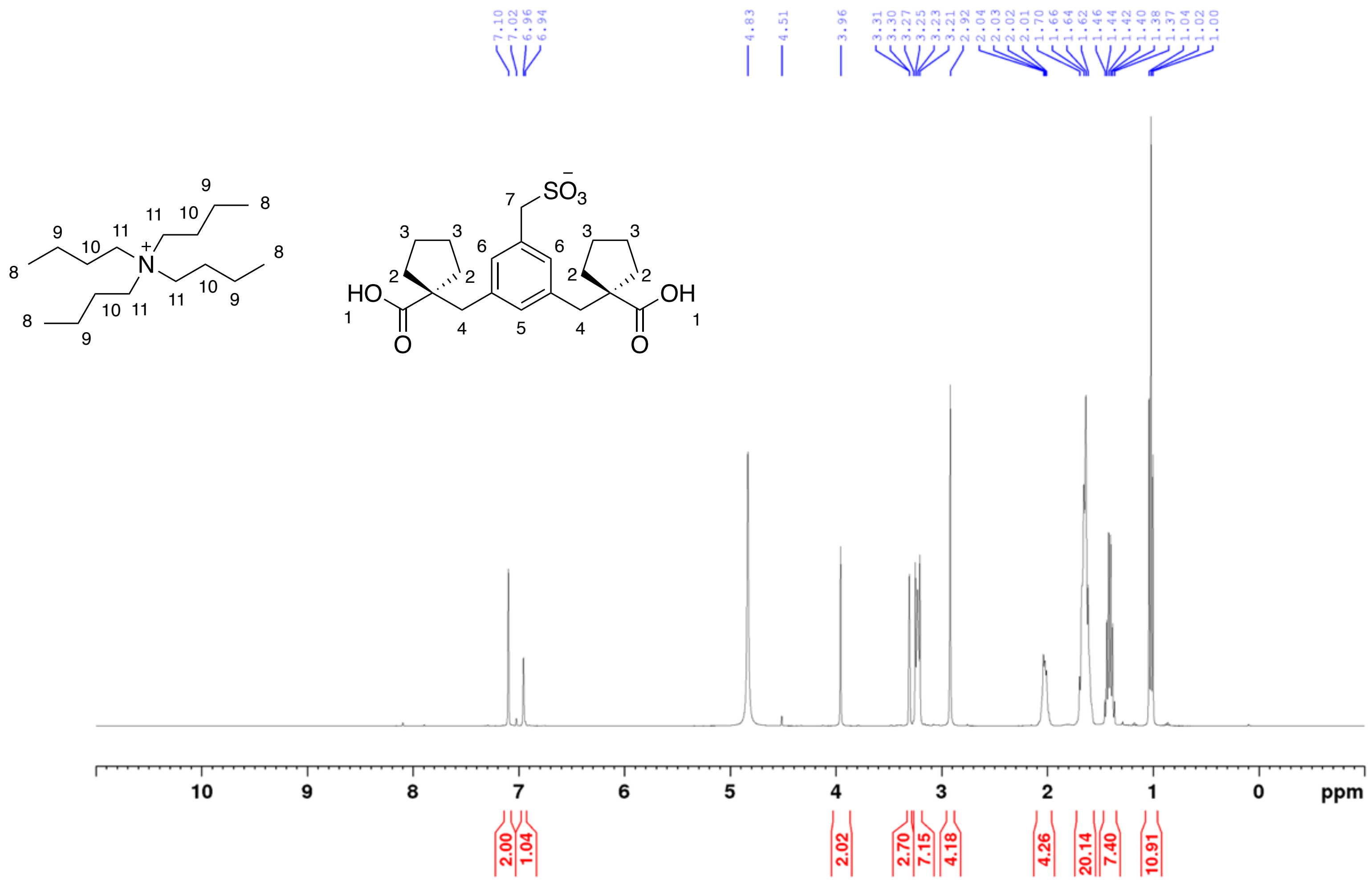

^{13}C NMR (101 MHz, MeOD) for *Tetrabutylammonium (3,5-bis((1-carboxycyclopentyl)methyl)phenyl)methanesulfonate* (**C•Bu$_4$N**)

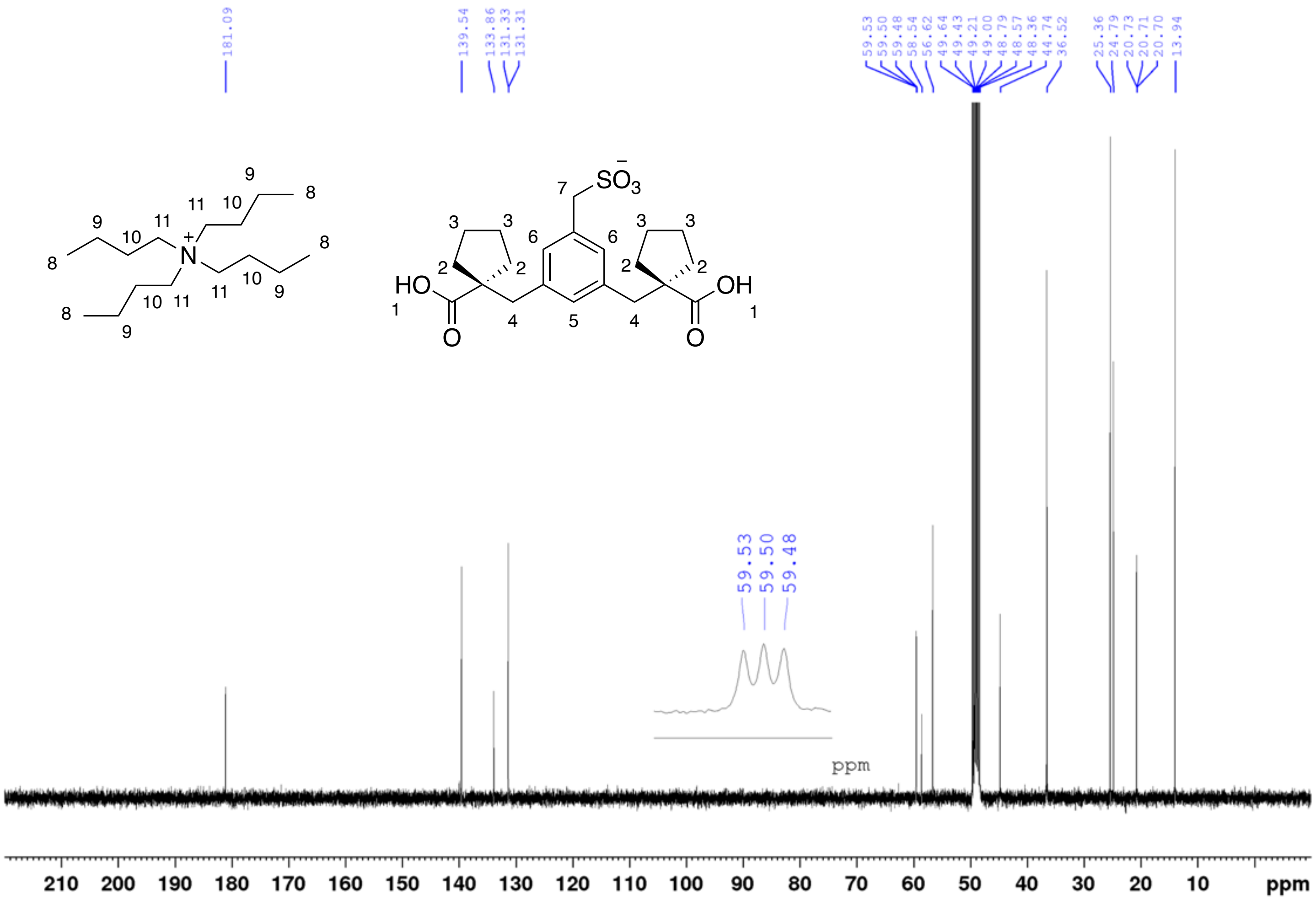

¹H NMR (500 MHz, DMSO-d6) for *Bis[rhodium Tetrabutylammonium (3,5-bis((1-carboxycyclopentyll)methyl)phenyl)methanesulfonate)]* (Rh_2(**C**)$_2$•(**Bu_4N**)$_2$)

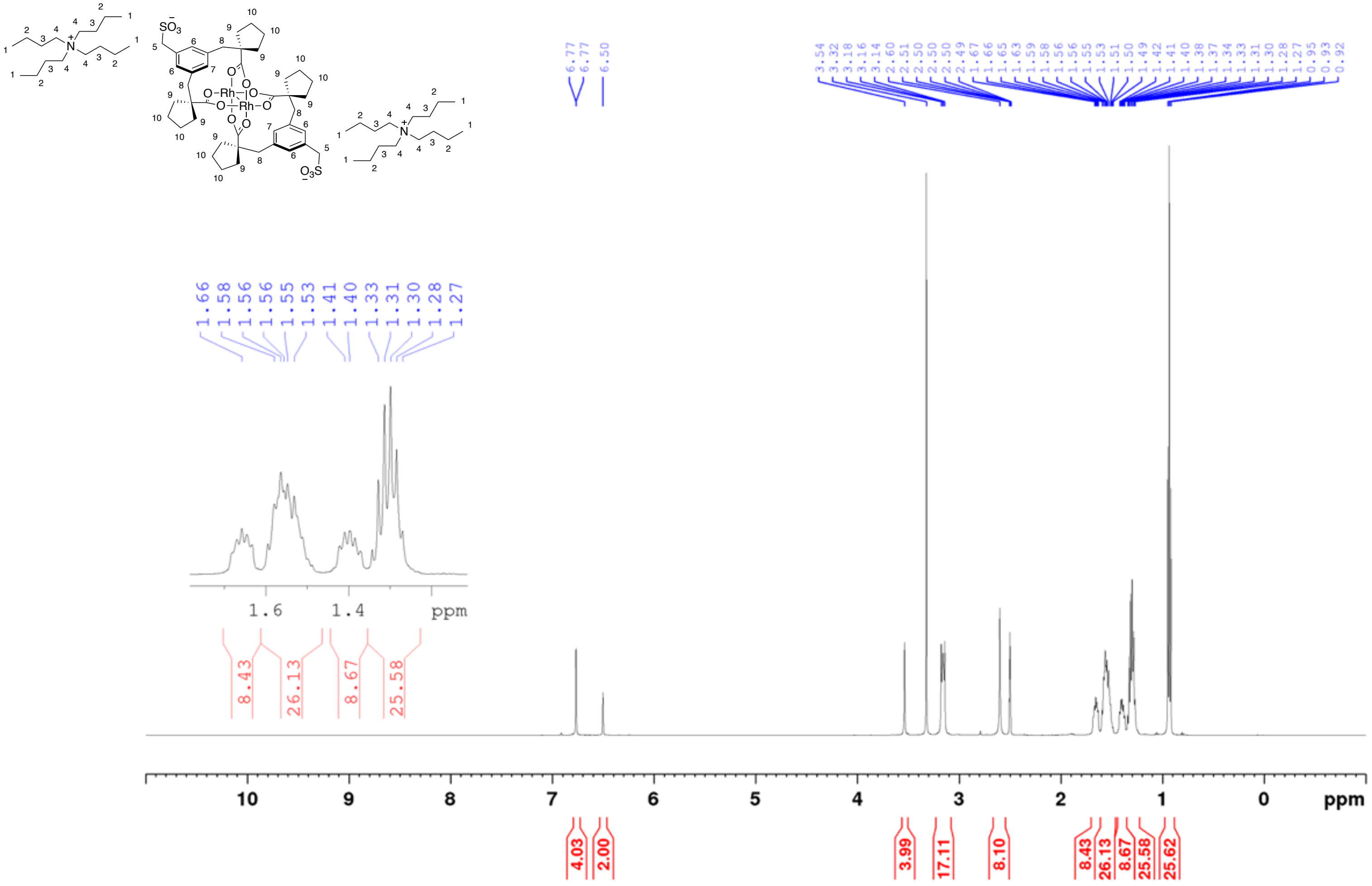

^{13}C NMR (126 MHz, DMSO-d$_6$) for *Bis[rhodium Tetrabutylammonium (3,5-bis((1-carboxycyclopentyll)methyl)phenyl)methanesulfonate)]* (Rh$_2$(**C**)$_2$•(**Bu$_4$N**)$_2$)

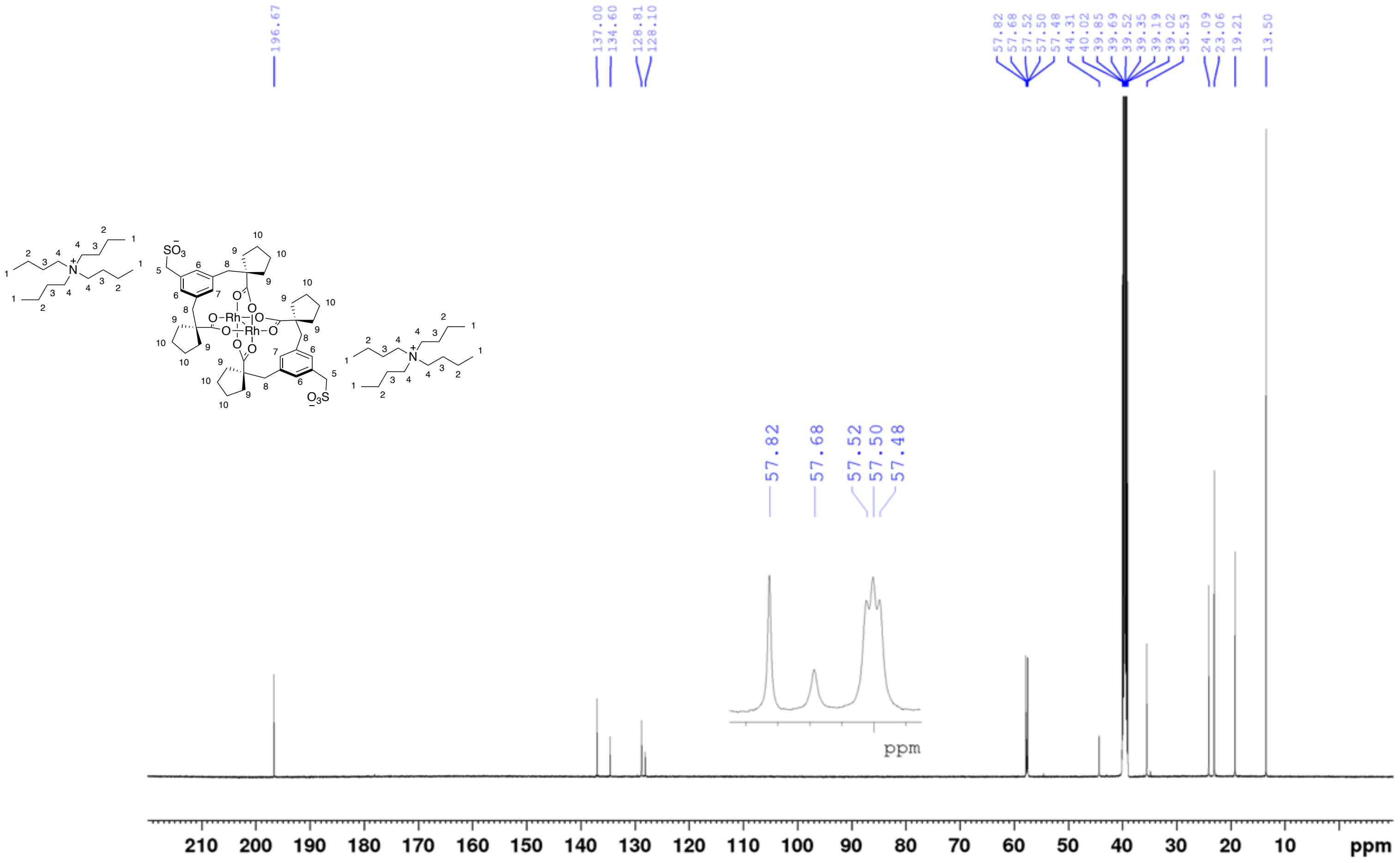

^{1}H NMR (400 MHz, $CDCl_3$) for *methyl cycloheptanecarboxylate*

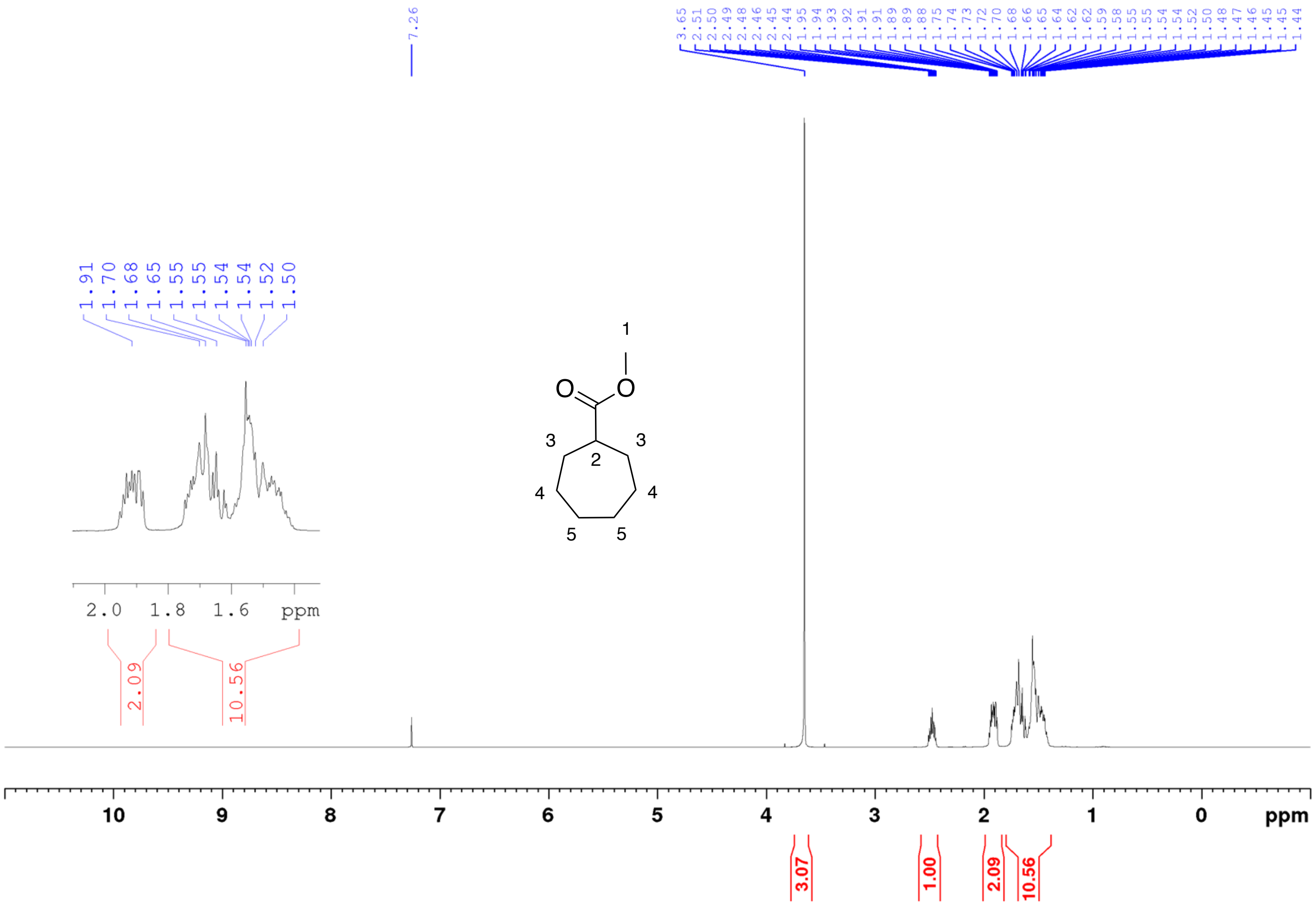

^{13}**C NMR** (101 MHz, $CDCl_3$) for *methyl cycloheptanecarboxylate*

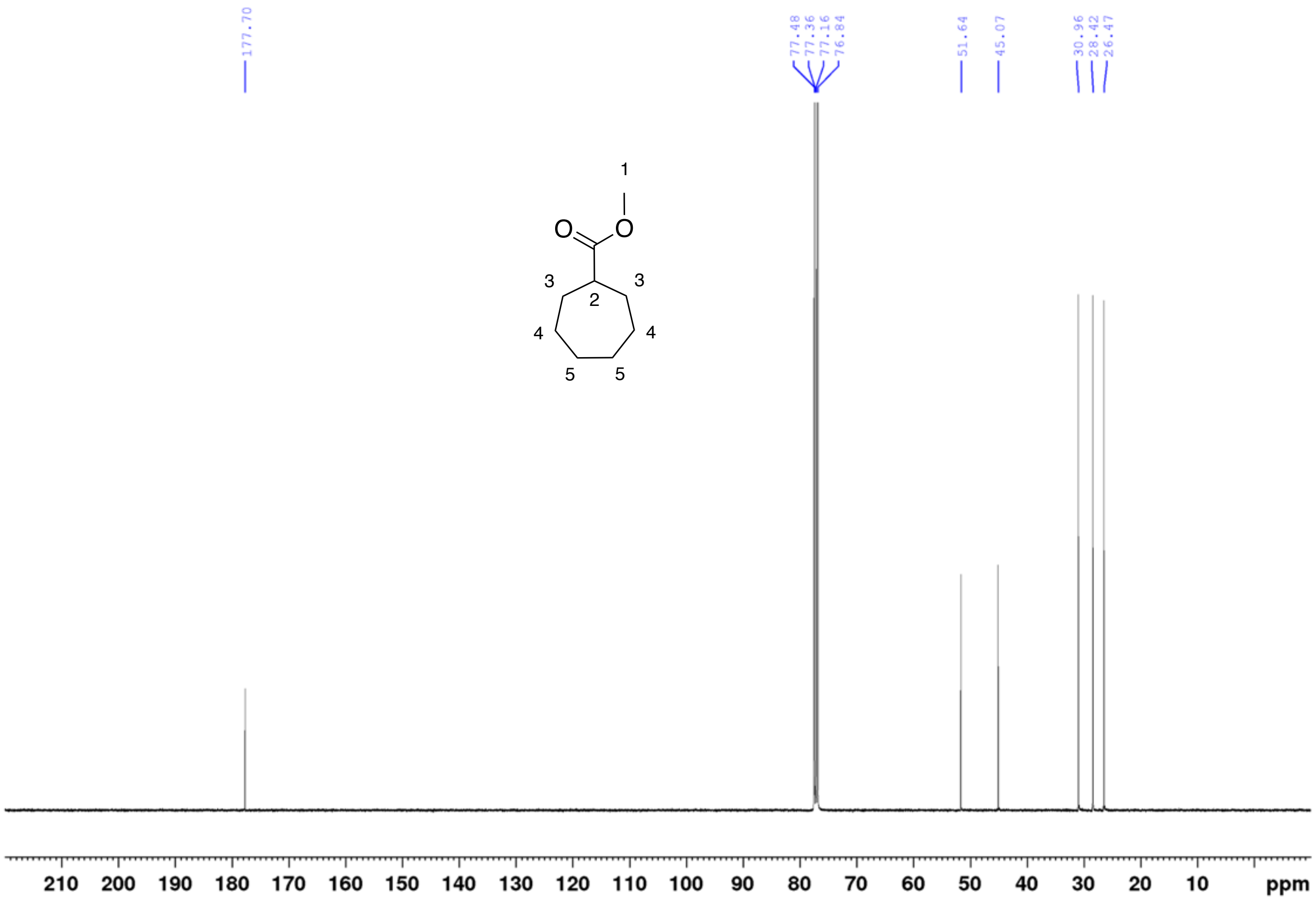

^{1}H NMR (400 MHz, $CDCl_3$) for *dimethyl 1,1'-((5-(bromomethyl)-1,3-phenylene)bis(methylene))bis(cycloheptane-1-carboxylate)*

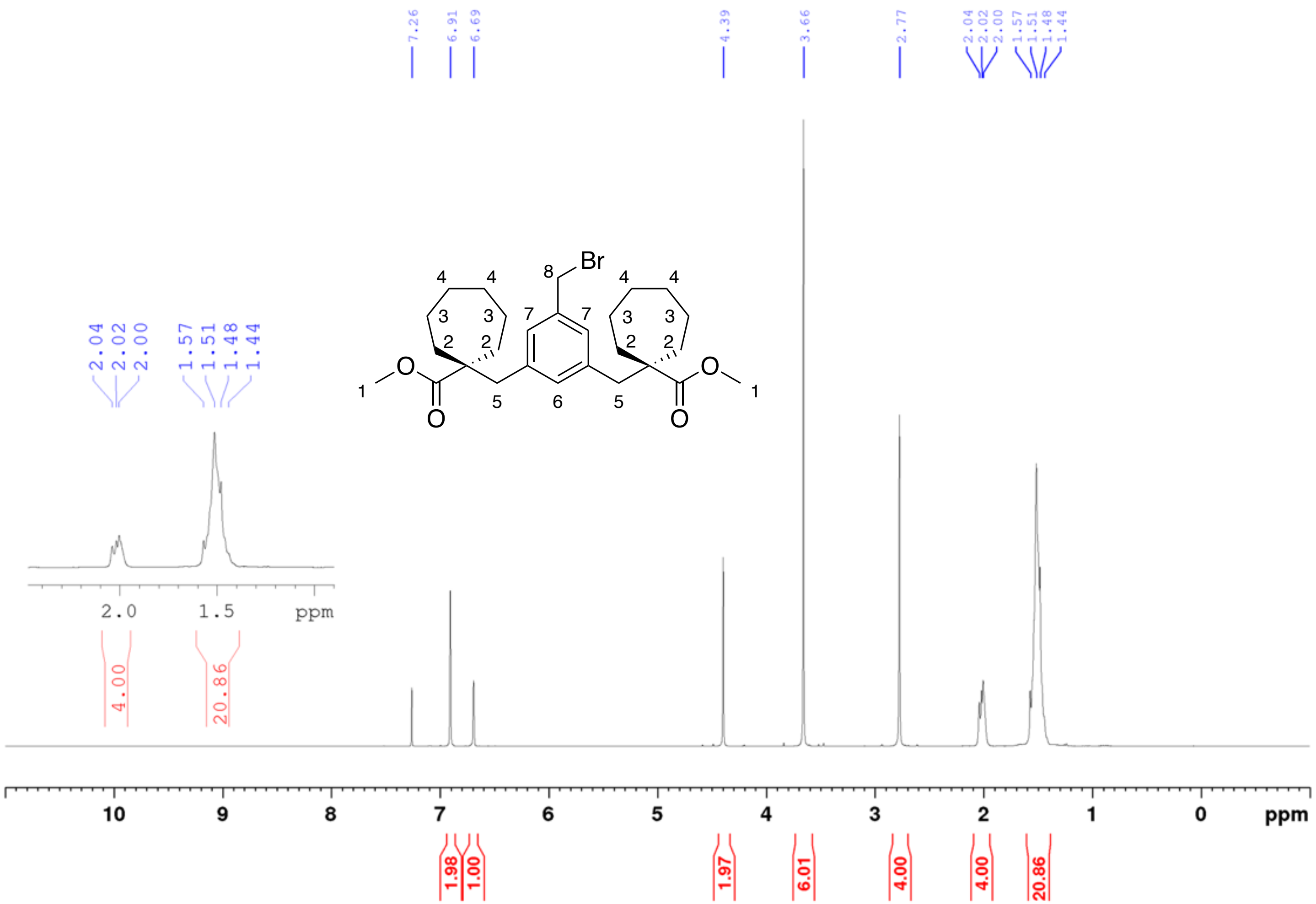

^{13}C NMR (101 MHz, $CDCl_3$) for *dimethyl 1,1'-((5-(bromomethyl)-1,3-phenylene)bis(methylene))bis(cycloheptane-1-carboxylate)*

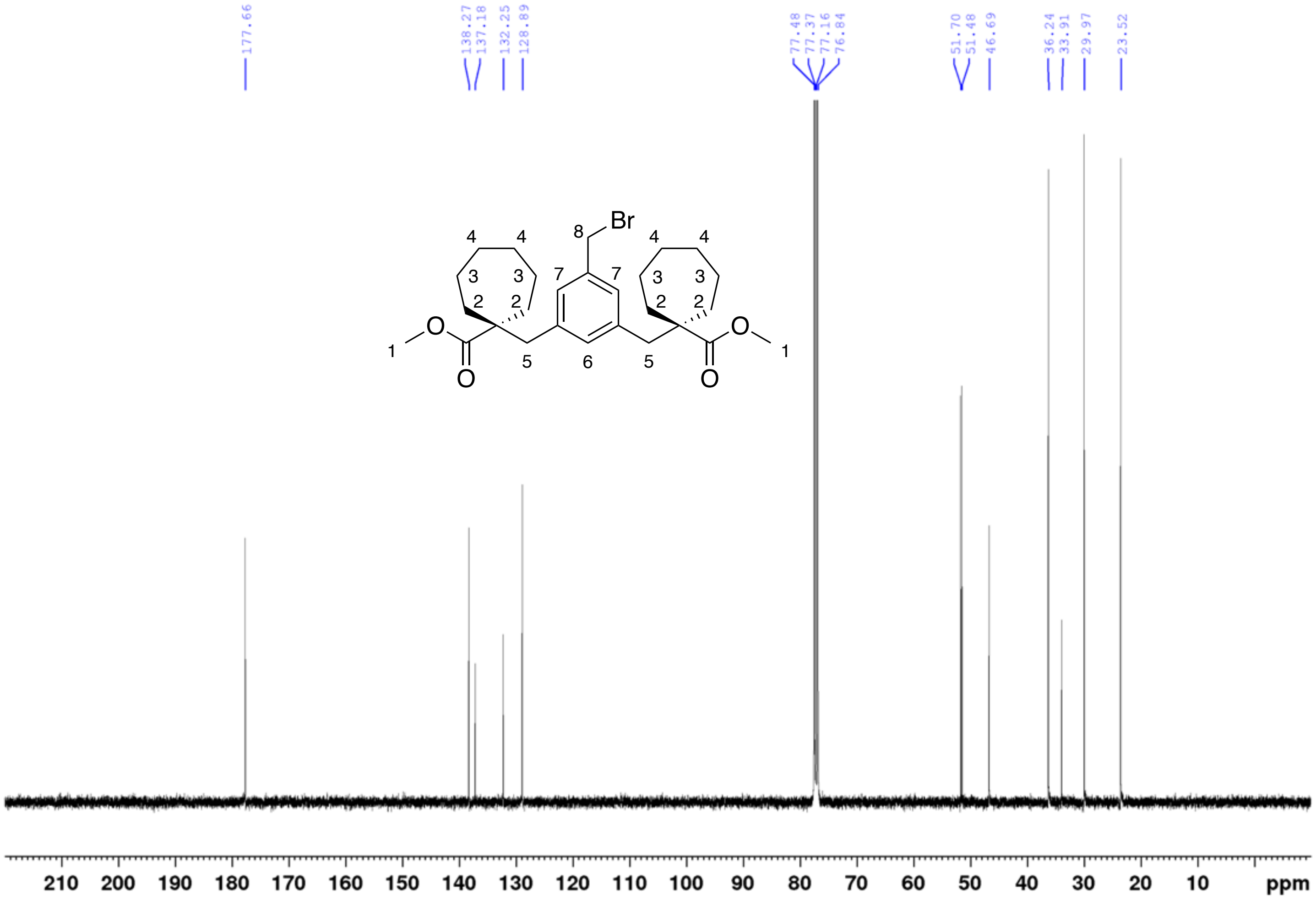

^{1}H NMR (500 MHz, $CDCl_3$) for *Tetrabutylammonium (3,5-bis((1-(methoxycarbonyl)cycloheptyl)methyl)phenyl)methanesulfonate*

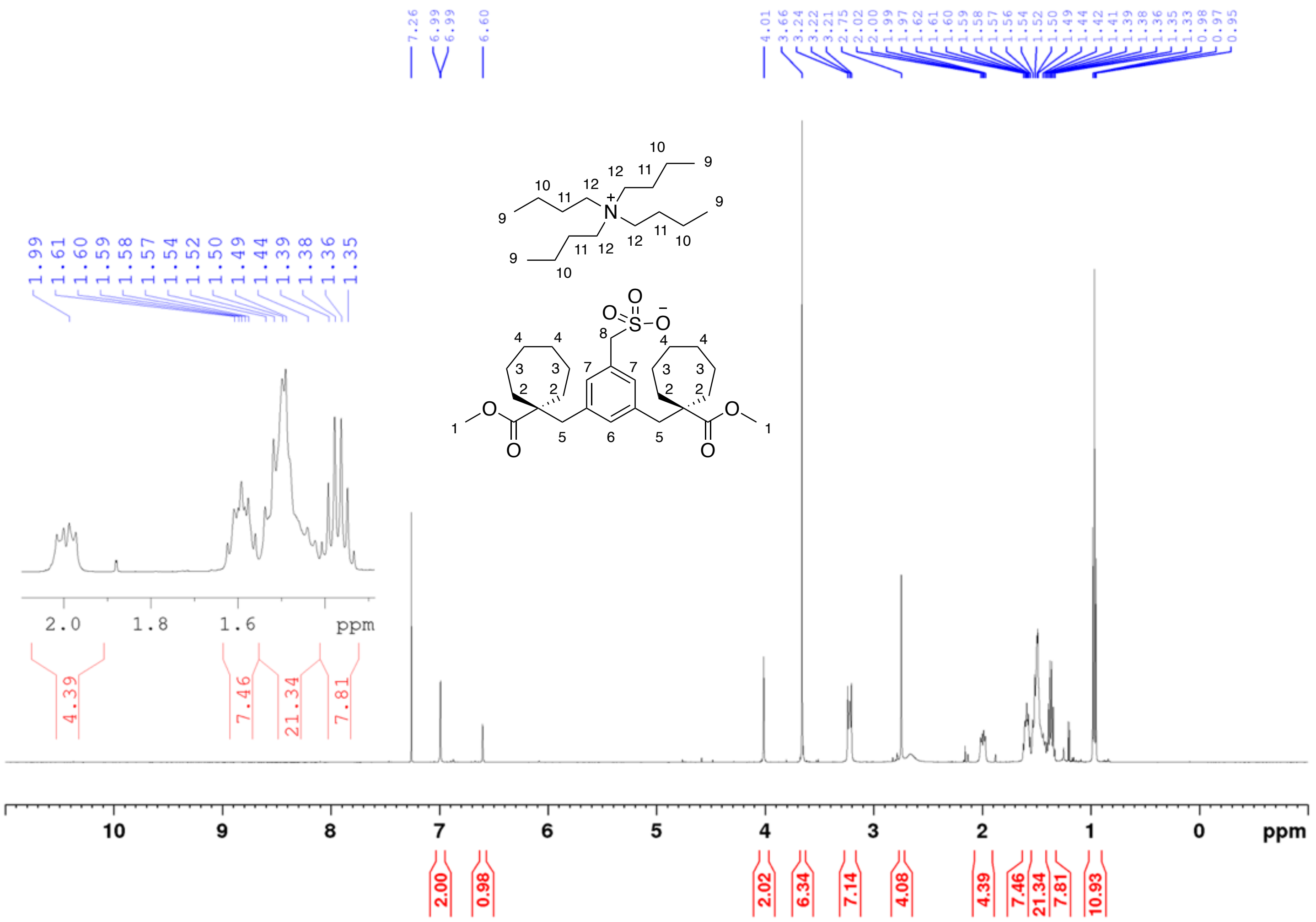

^{13}C NMR (126 MHz, $CDCl_3$) for *Tetrabutylammonium (3,5-bis((1-(methoxycarbonyl)cycloheptyl)methyl)phenyl)methanesulfonate*

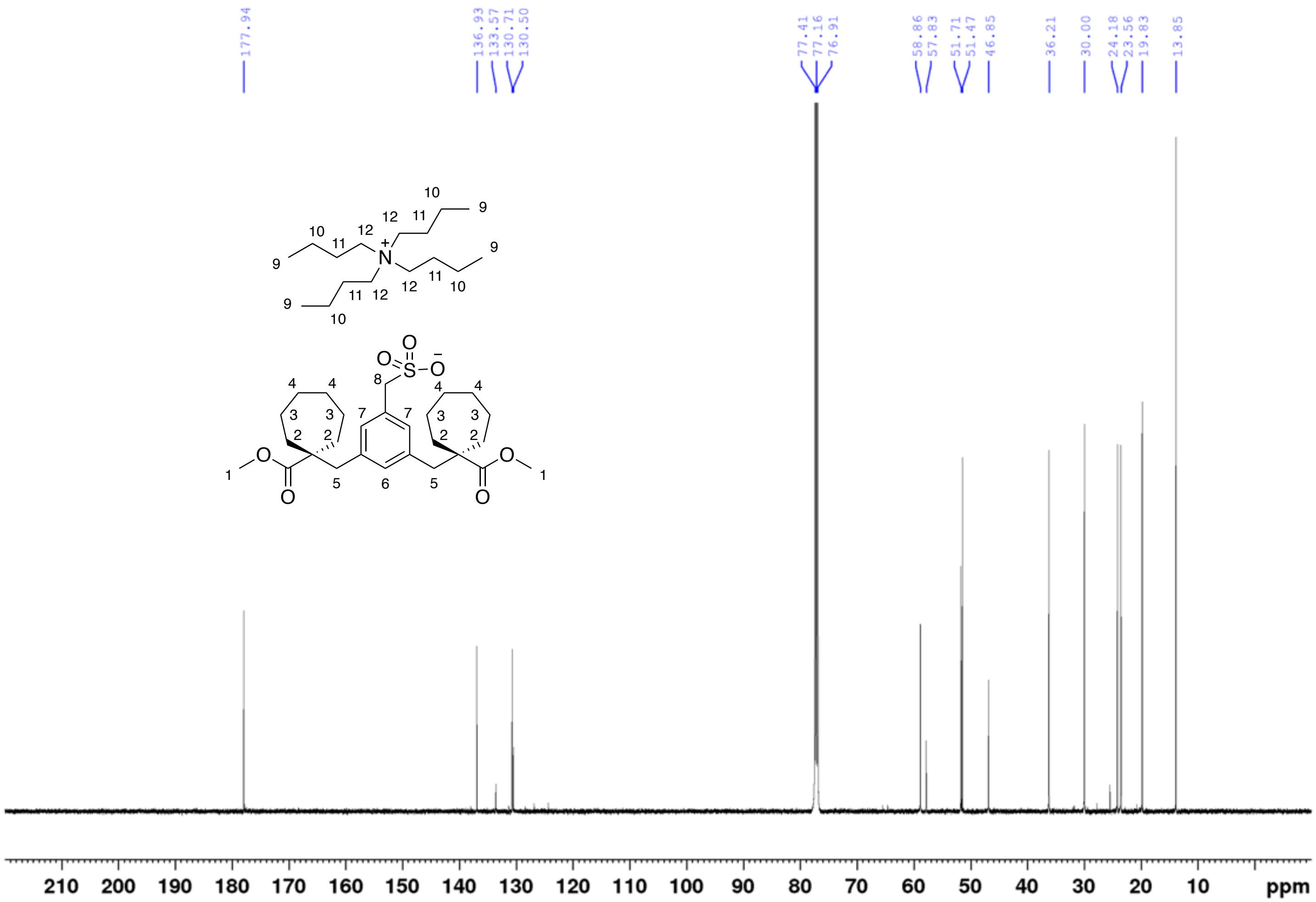

¹H NMR (400 MHz, MeOD) for *Tetrabutylammonium (3,5-bis((1-carboxycycloheptyl)methyl)phenyl)methanesulfonate* (**D•Bu$_4$N**)

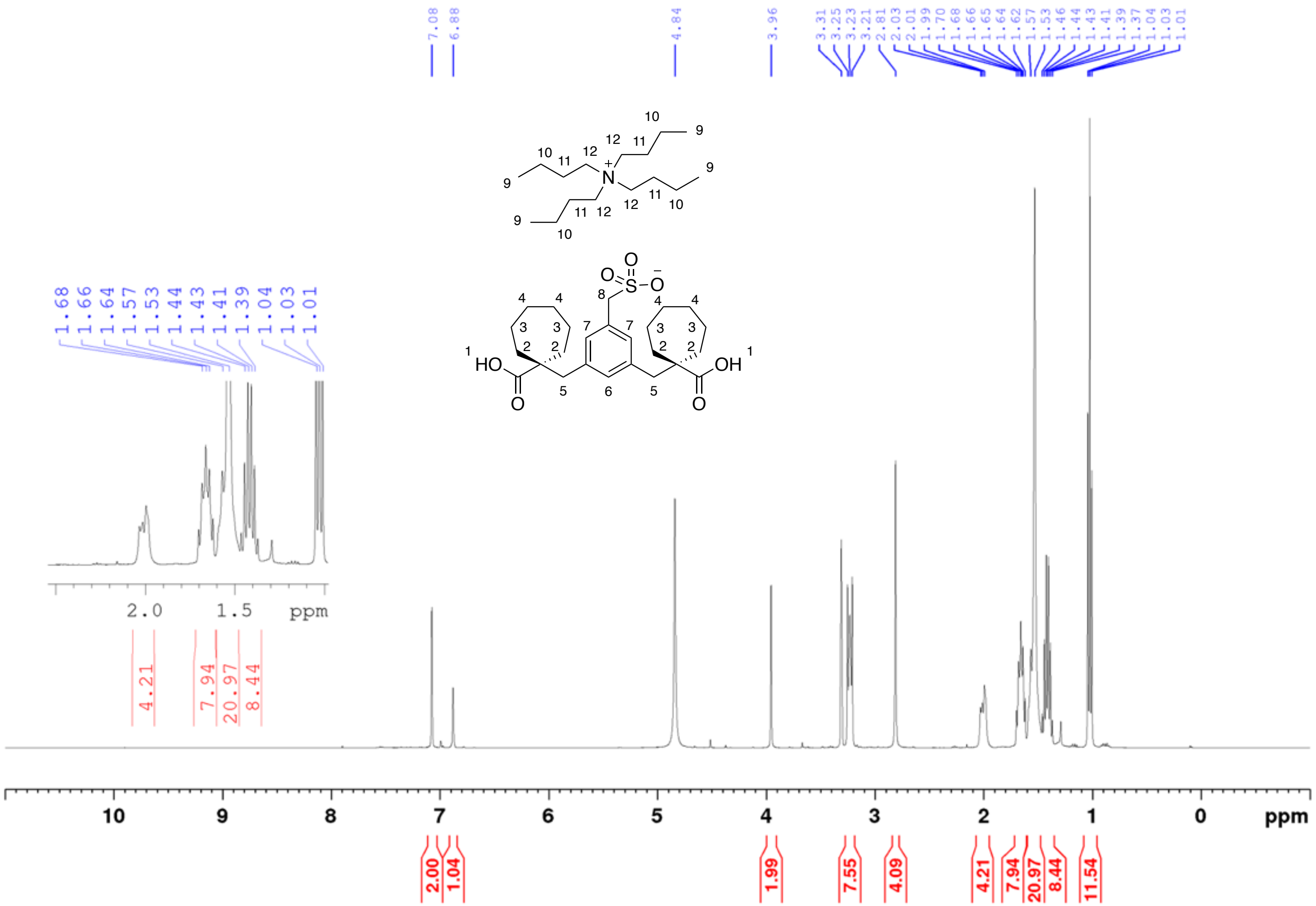

^{13}C NMR (101 MHz, MeOD) for *Tetrabutylammonium (3,5-bis((1-carboxycycloheptyl)methyl)phenyl)methanesulfonate* (**D•Bu$_4$N**)

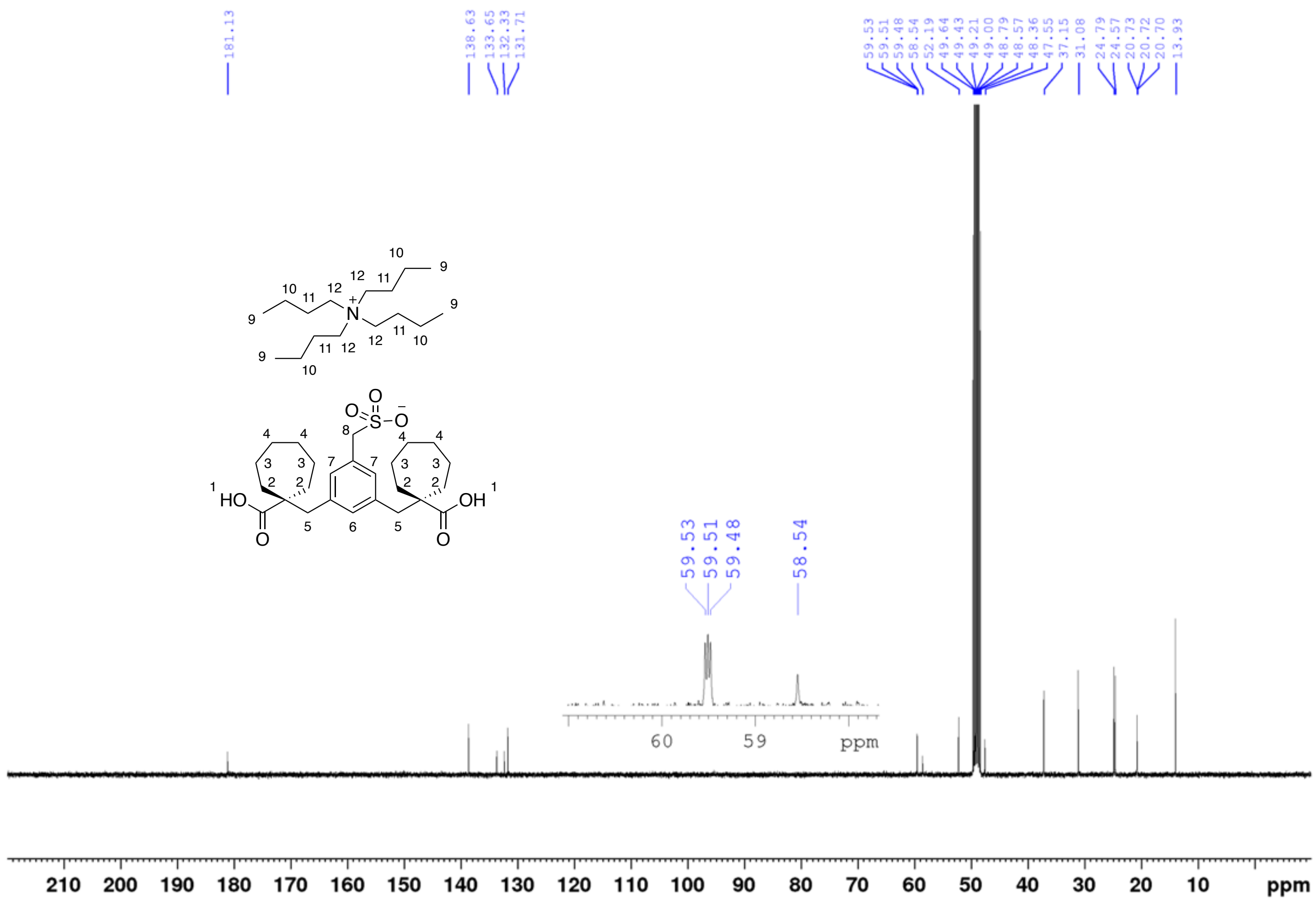

¹H NMR (500 MHz, C_5D_5N) for *Bis[rhodium Tetrabutylammonium (3,5-bis((1-carboxycycloheptyl)methyl)phenyl)methanesulfonate)]* (Rh_2(**D**)$_2$•(**Bu₄N**)$_2$)

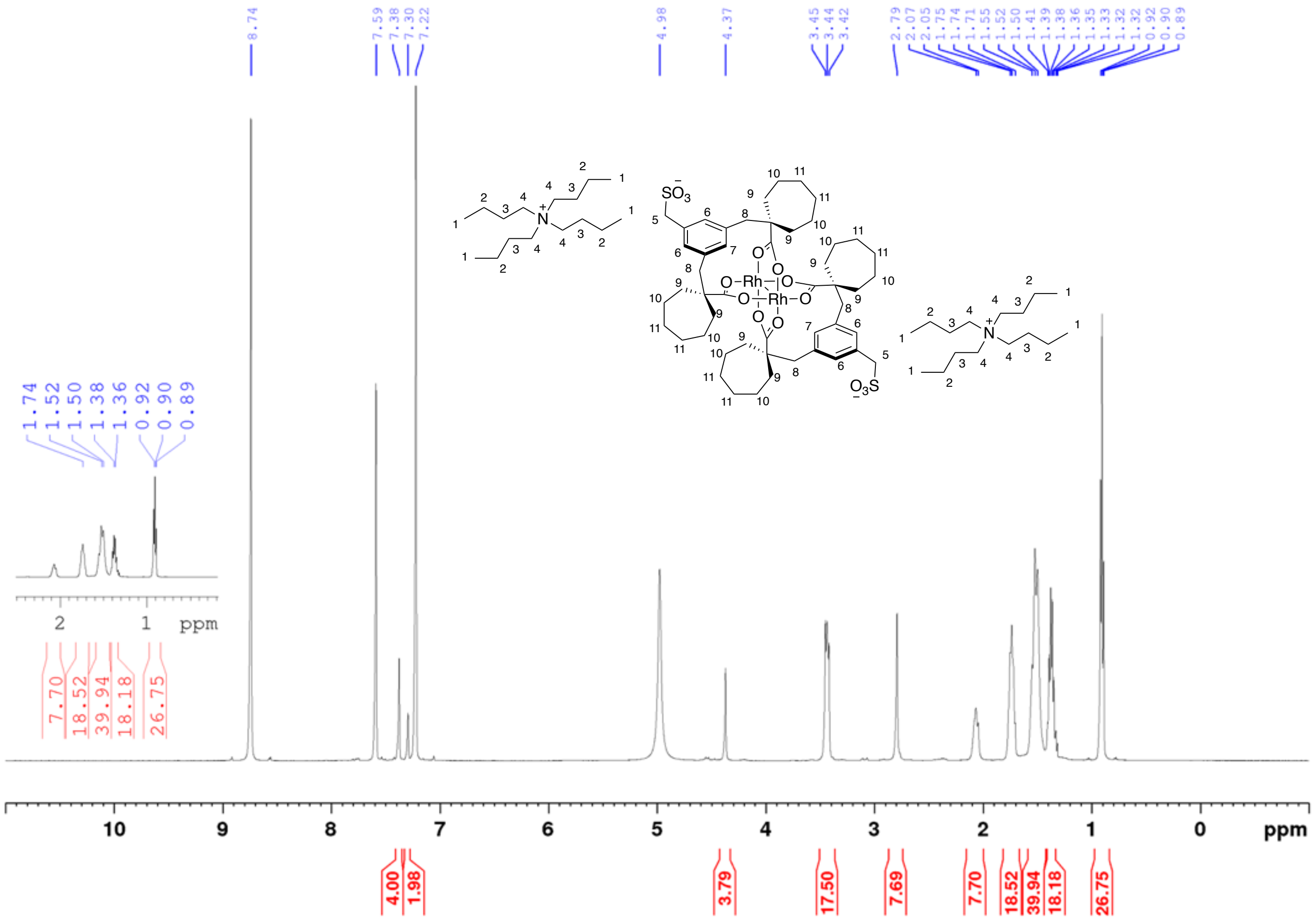

^{13}C NMR (126 MHz, C_5D_5N) for *Bis[rhodium Tetrabutylammonium (3,5-bis((1-carboxycycloheptyl)methyl)phenyl)methanesulfonate)]* (Rh_2(**D**)$_2$•(**Bu_4N**)$_2$)

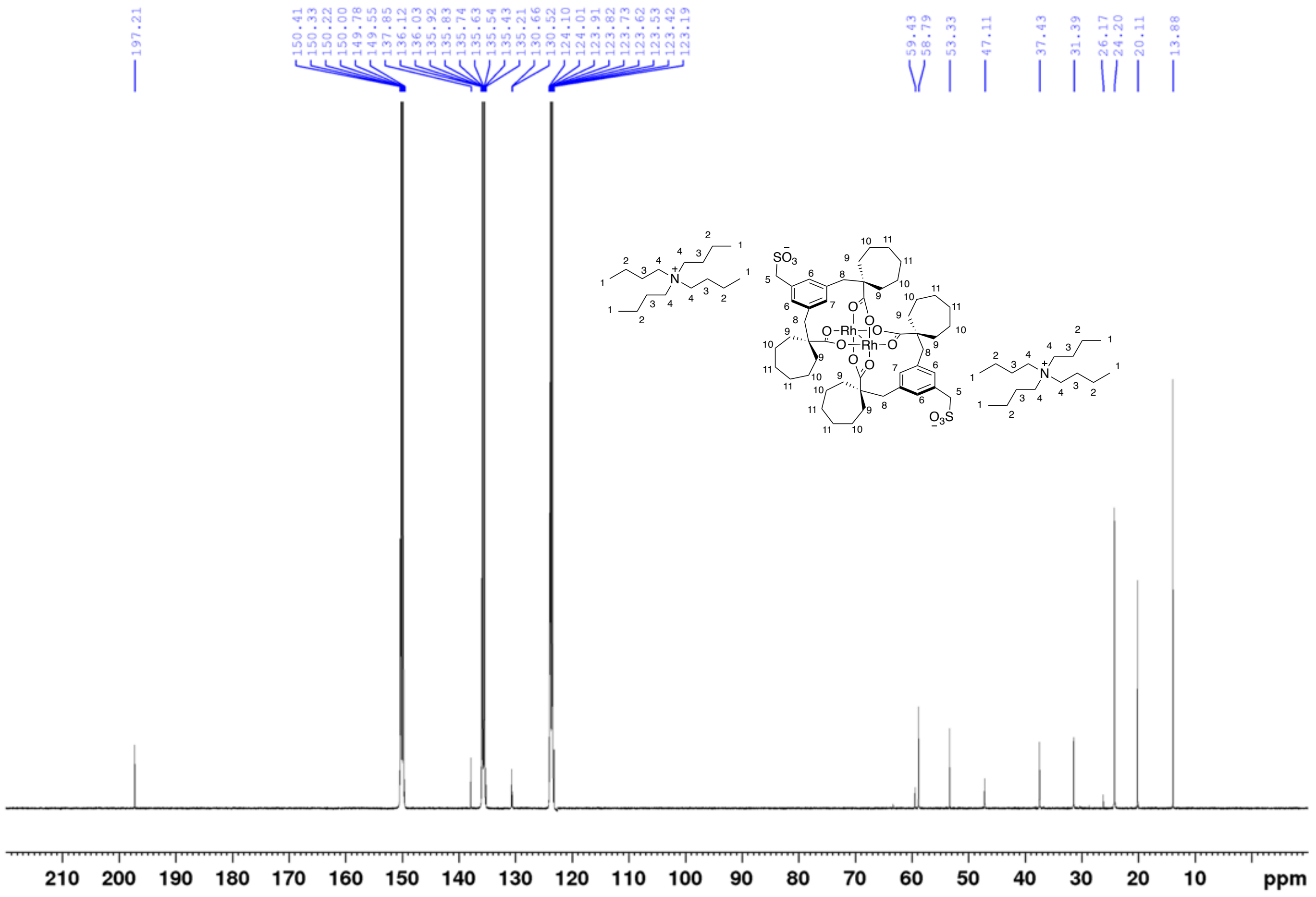

^{1}H NMR (400 MHz, $CDCl_3$) for *methyl cyclooctanecarboxylate*

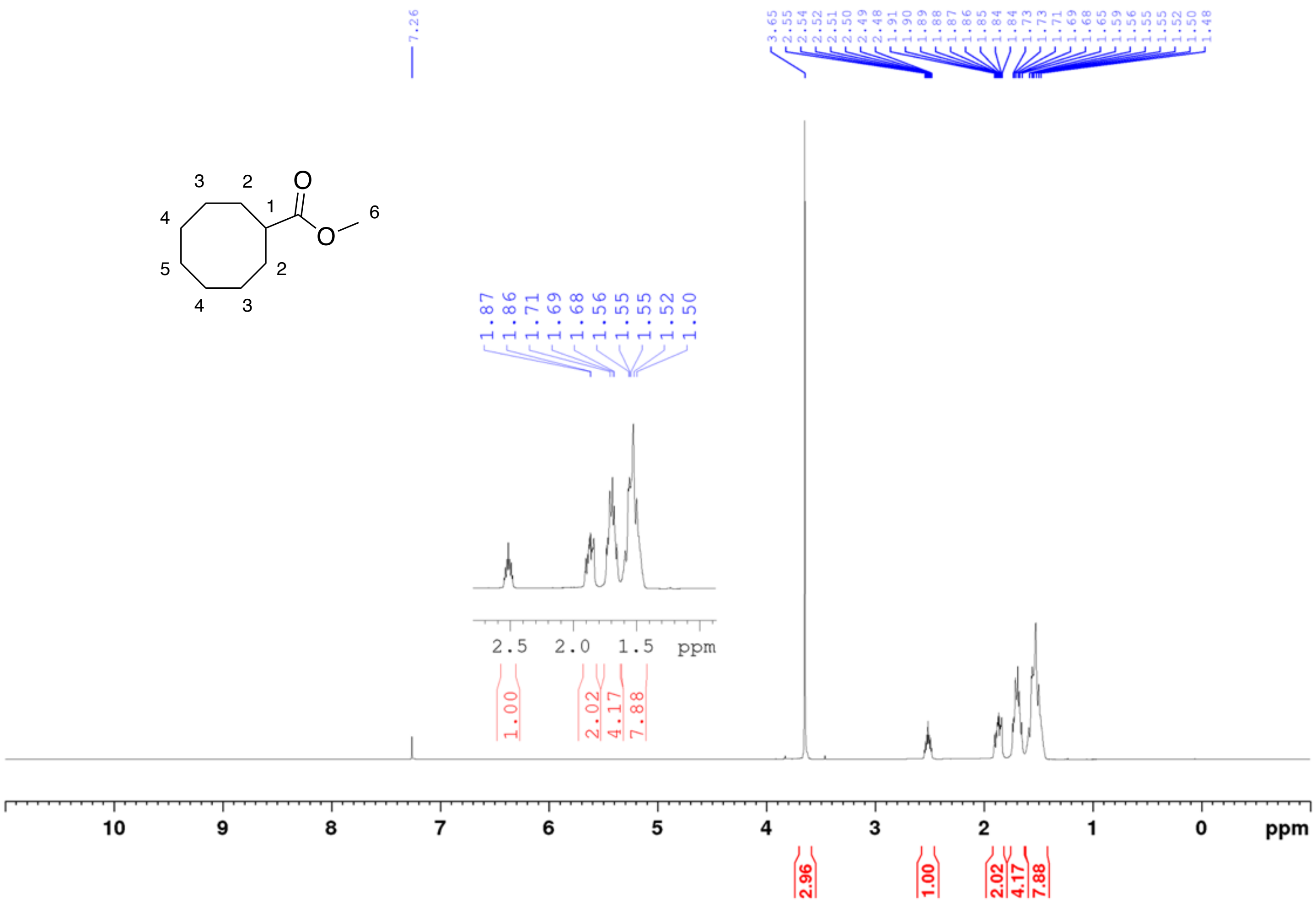

^{13}C NMR (101 MHz, $CDCl_3$) for *methyl cyclooctanecarboxylate*

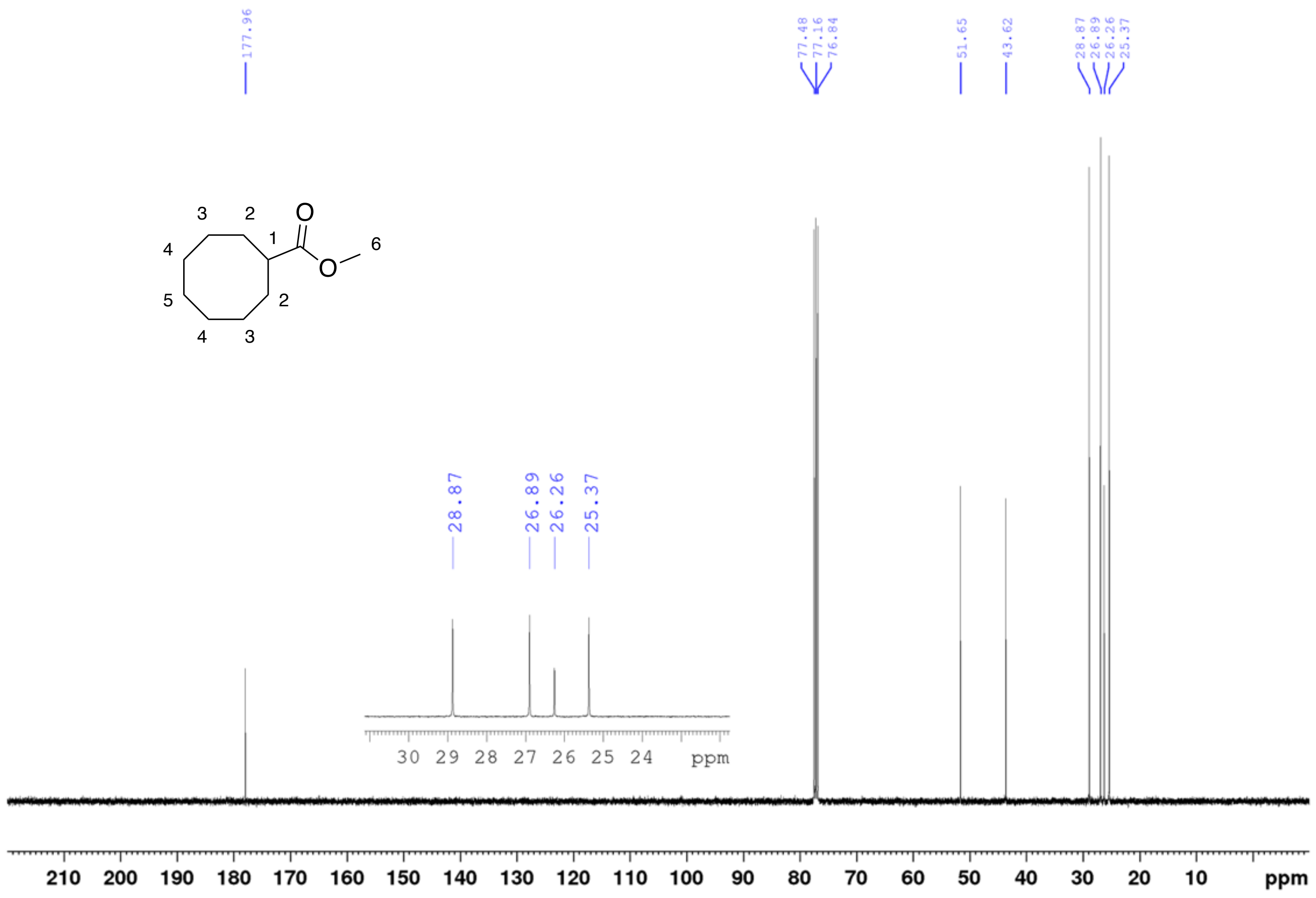

^{1}H NMR (400 MHz, $CDCl_3$) for *dimethyl 1,1'-((5-(bromomethyl)-1,3-phenylene)bis(methylene))bis(cyclooctane-1-carboxylate)*

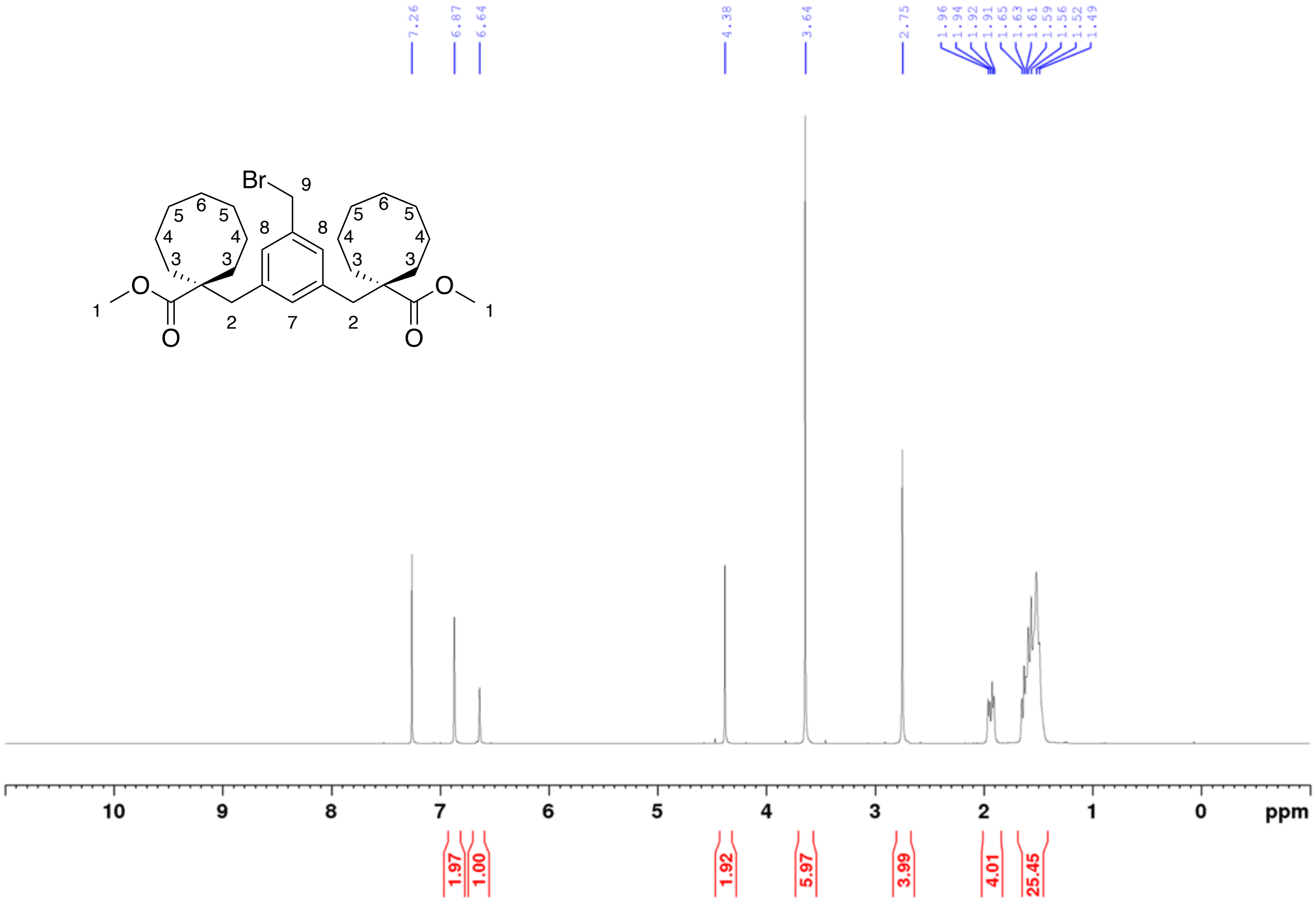

^{13}C NMR (101 MHz, $CDCl_3$) for *dimethyl 1,1'-((5-(bromomethyl)-1,3-phenylene)bis(methylene))bis(cyclooctane-1-carboxylate)*

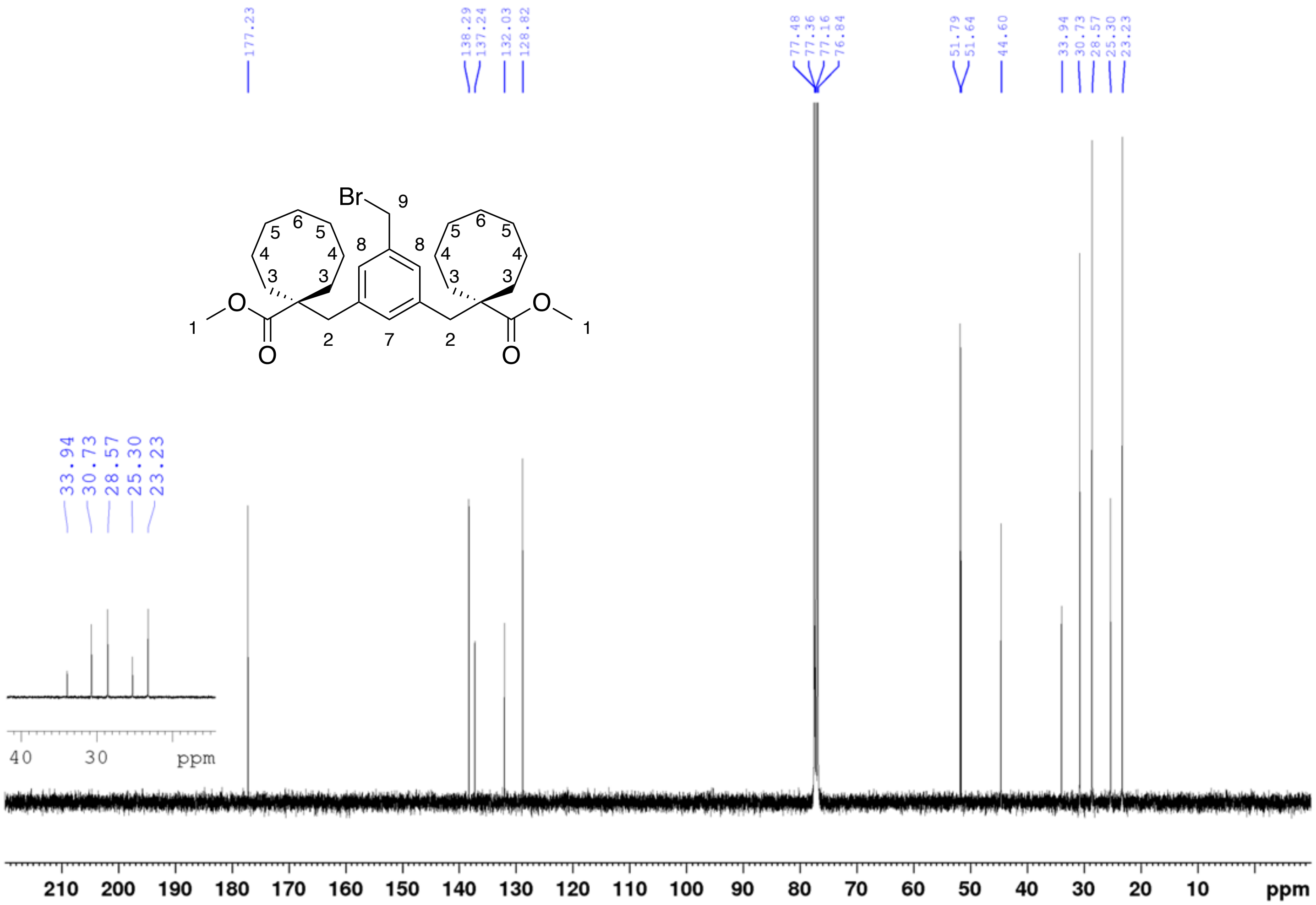

^{1}H NMR (500 MHz, $CDCl_3$) for *dimethyl 1,1'-((5-(((2,2,2-trifluoroethoxy)sulfonyl)methyl)-1,3-phenylene)bis(methylene))bis(cyclooctane-1-carboxylate)*

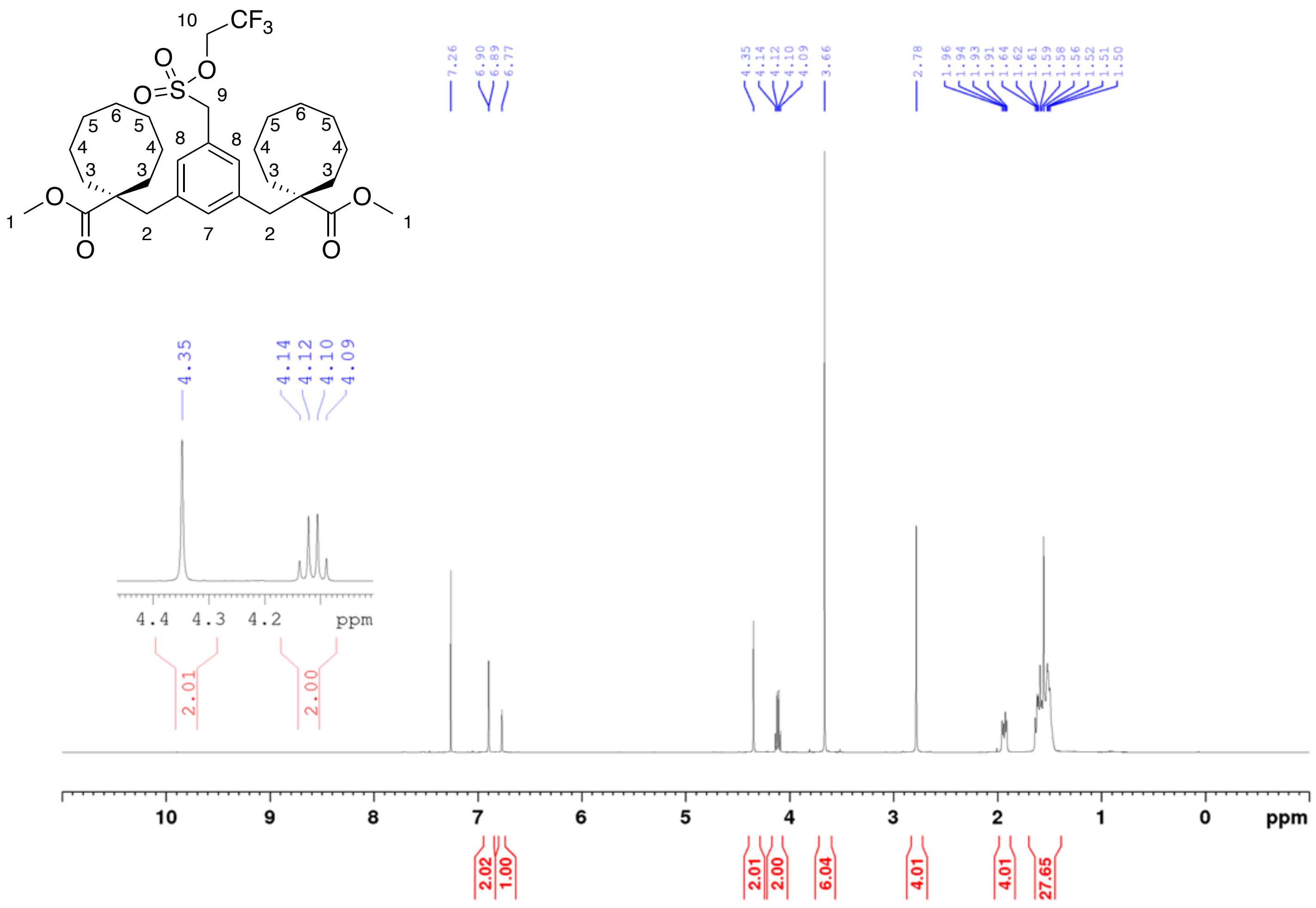

^{13}C NMR (126 MHz, $CDCl_3$) for *dimethyl 1,1'-((5-(((2,2,2-trifluoroethoxy)sulfonyl)methyl)-1,3-phenylene)bis(methylene))bis(cyclooctane-1-carboxylate*

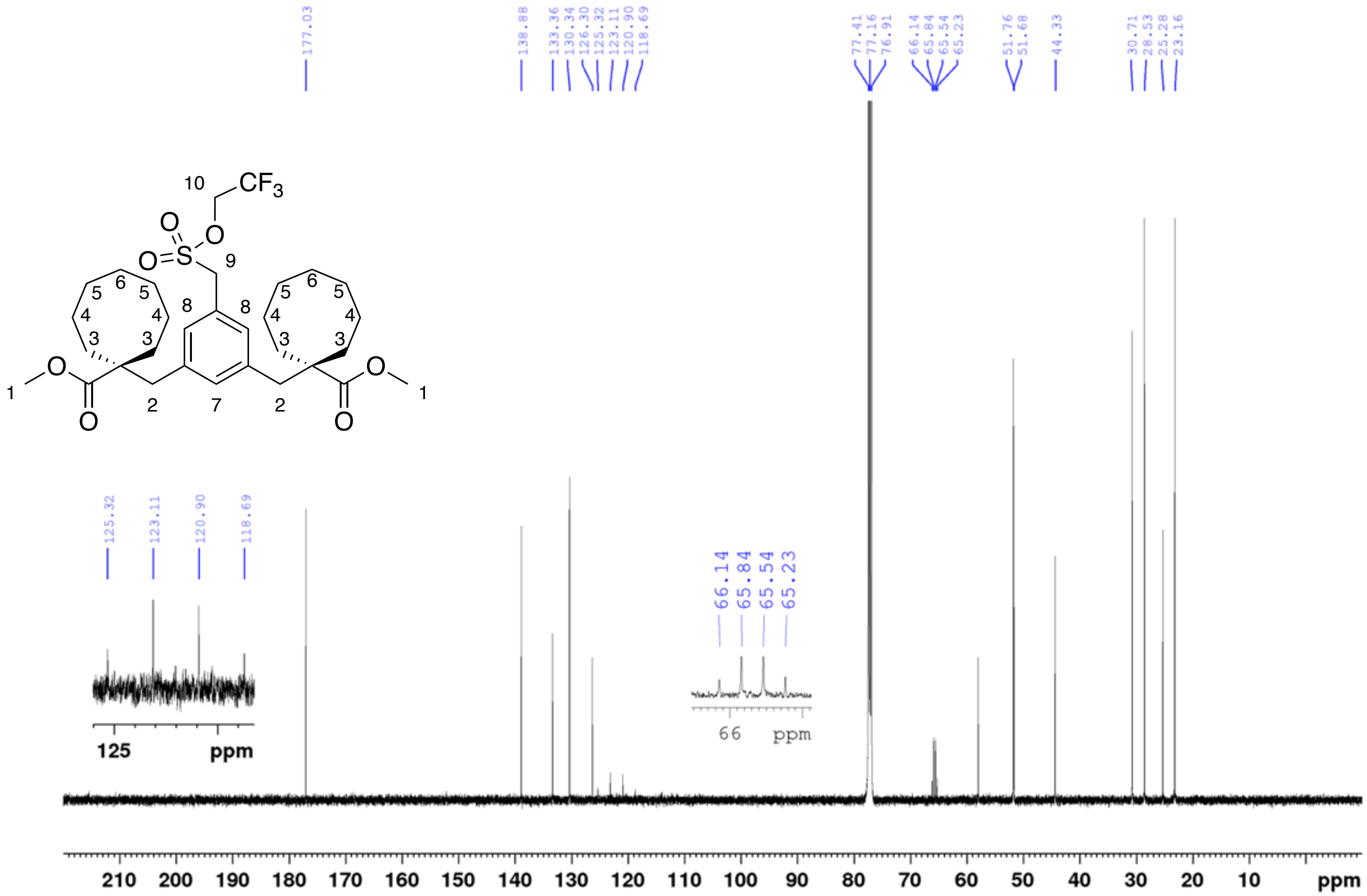

^{19}F NMR (376 MHz, $CDCl_3$) for *dimethyl 1,1'-((5-(((2,2,2-trifluoroethoxy)sulfonyl)methyl)-1,3-phenylene)bis(methylene))bis(cyclooctane-1-carboxylate*

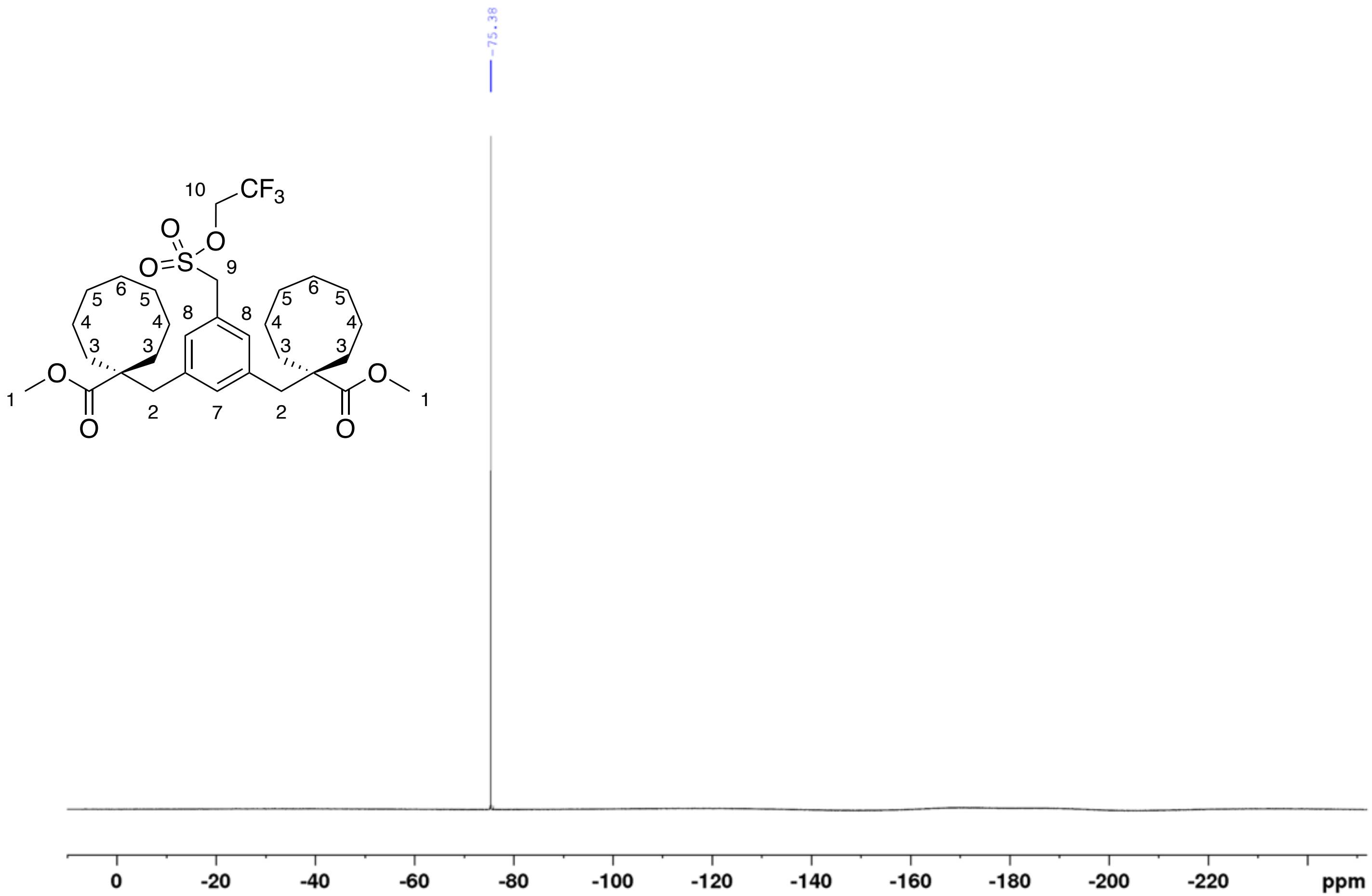

^{1}H NMR (500 MHz, DMSO-d$_6$) for *Tetrabutylammonium (3,5-bis((1-carboxycyclooctyl)methyl)phenyl)methanesulfonate* (**E•Bu$_4$N**)

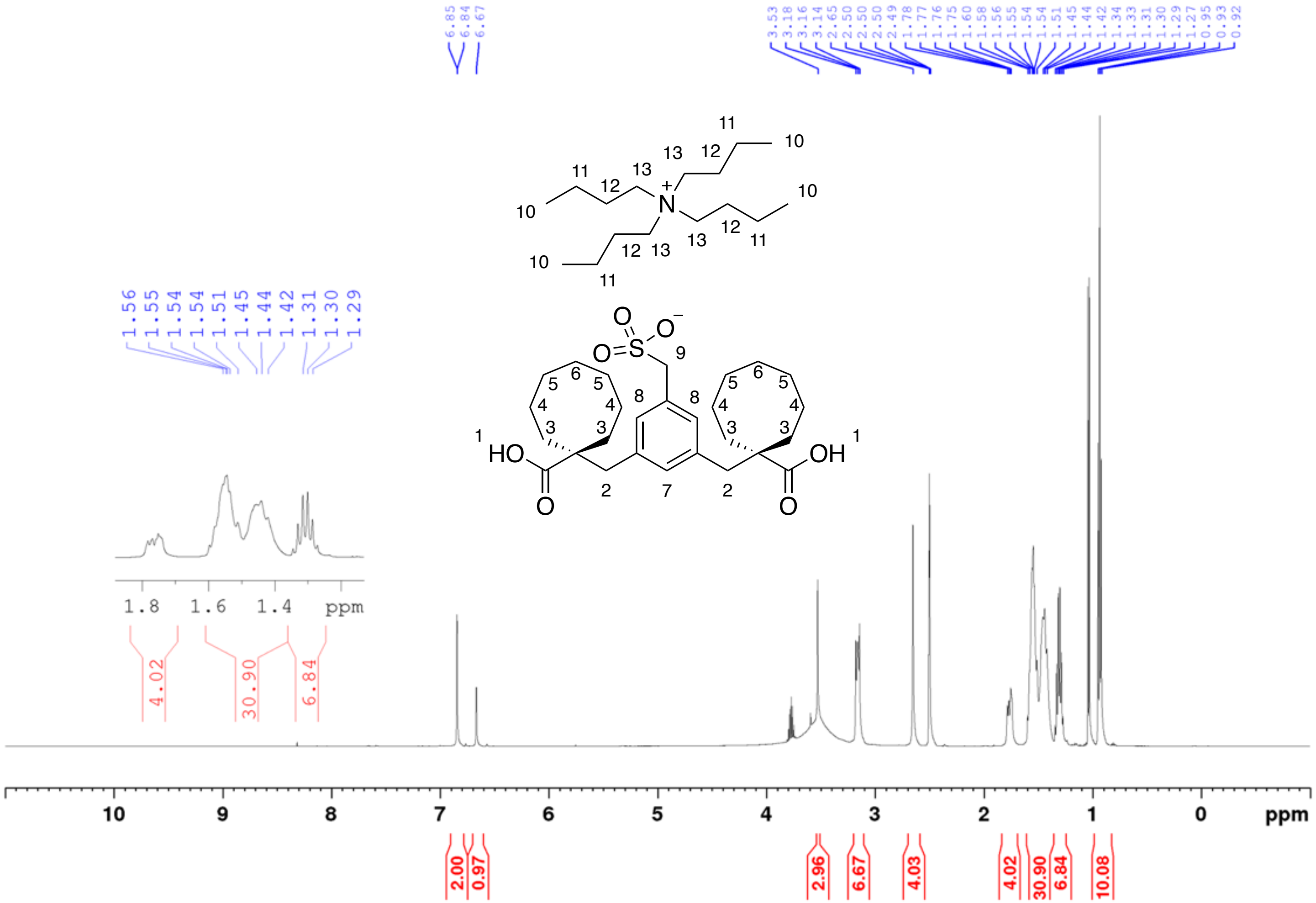

^{13}**C NMR** (126 MHz, DMSO-d$_6$) for *Tetrabutylammonium (3,5-bis((1-carboxycyclooctyl)methyl)phenyl)methanesulfonate* (**E•Bu$_4$N**)

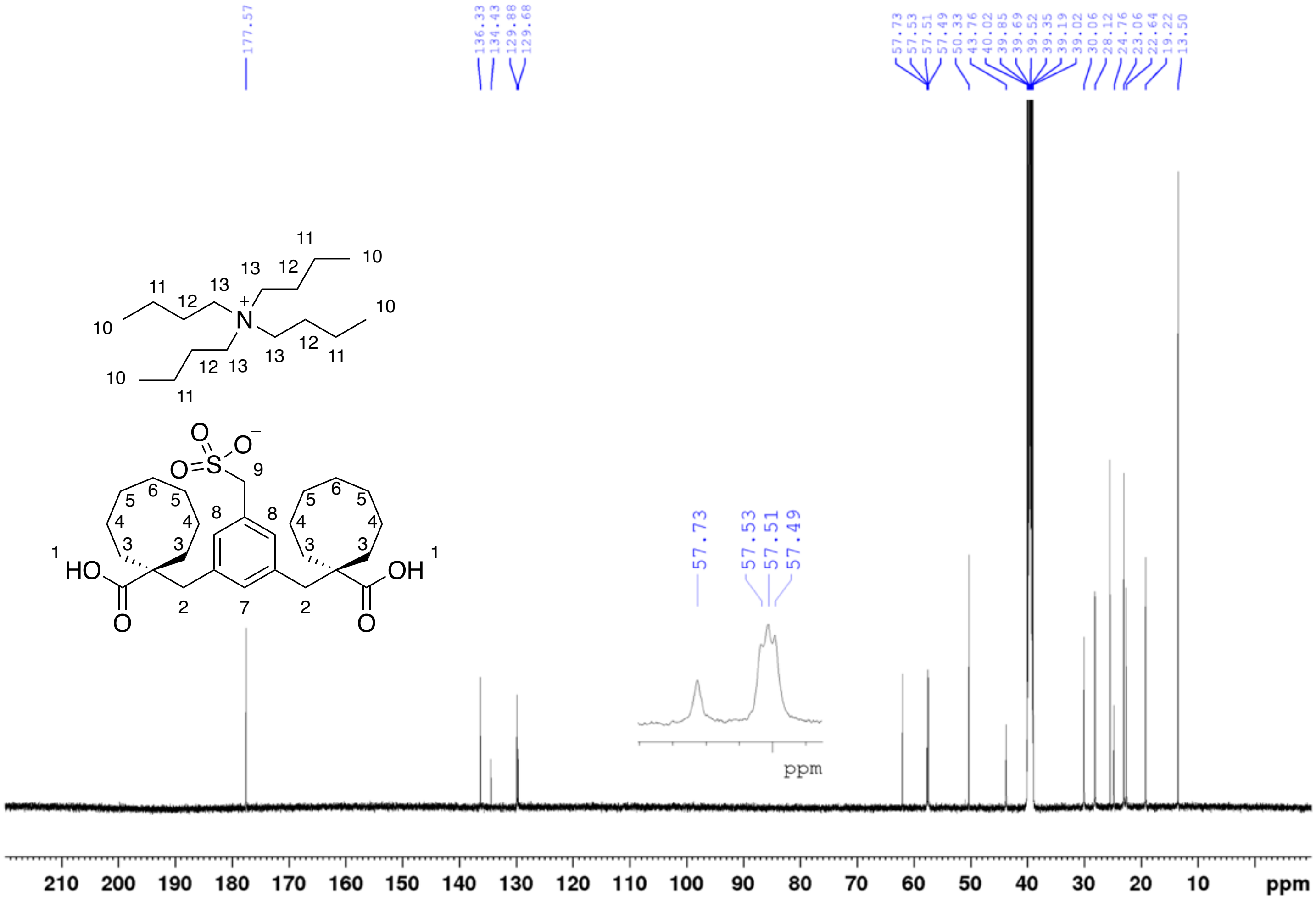

^{1}H NMR (500 MHz, C_5D_5N) for *Bis[rhodium Tetrabutylammonium (3,5-bis((1-carboxycyclooctyl)methyl)phenyl)methanesulfonate* (Rh_2(**E**)$_2$•(**Bu_4N**)$_2$)

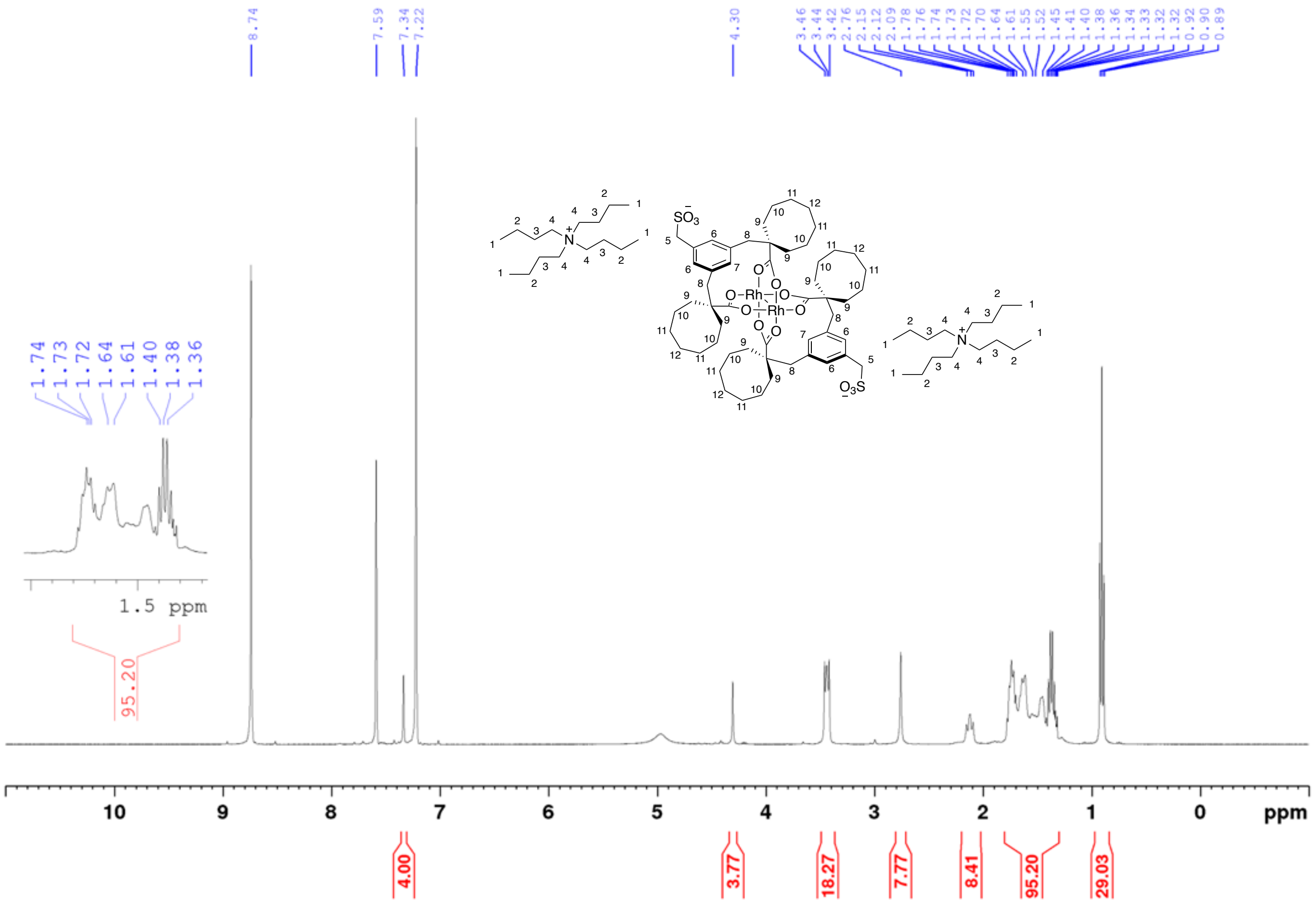

^{13}C NMR (126 MHz, C_5D_5N) for *Bis[rhodium Tetrabutylammonium (3,5-bis((1-carboxycyclooctyl)methyl)phenyl)methanesulfonate* (Rh_2(**E**)$_2$•(**Bu_4N**)$_2$)

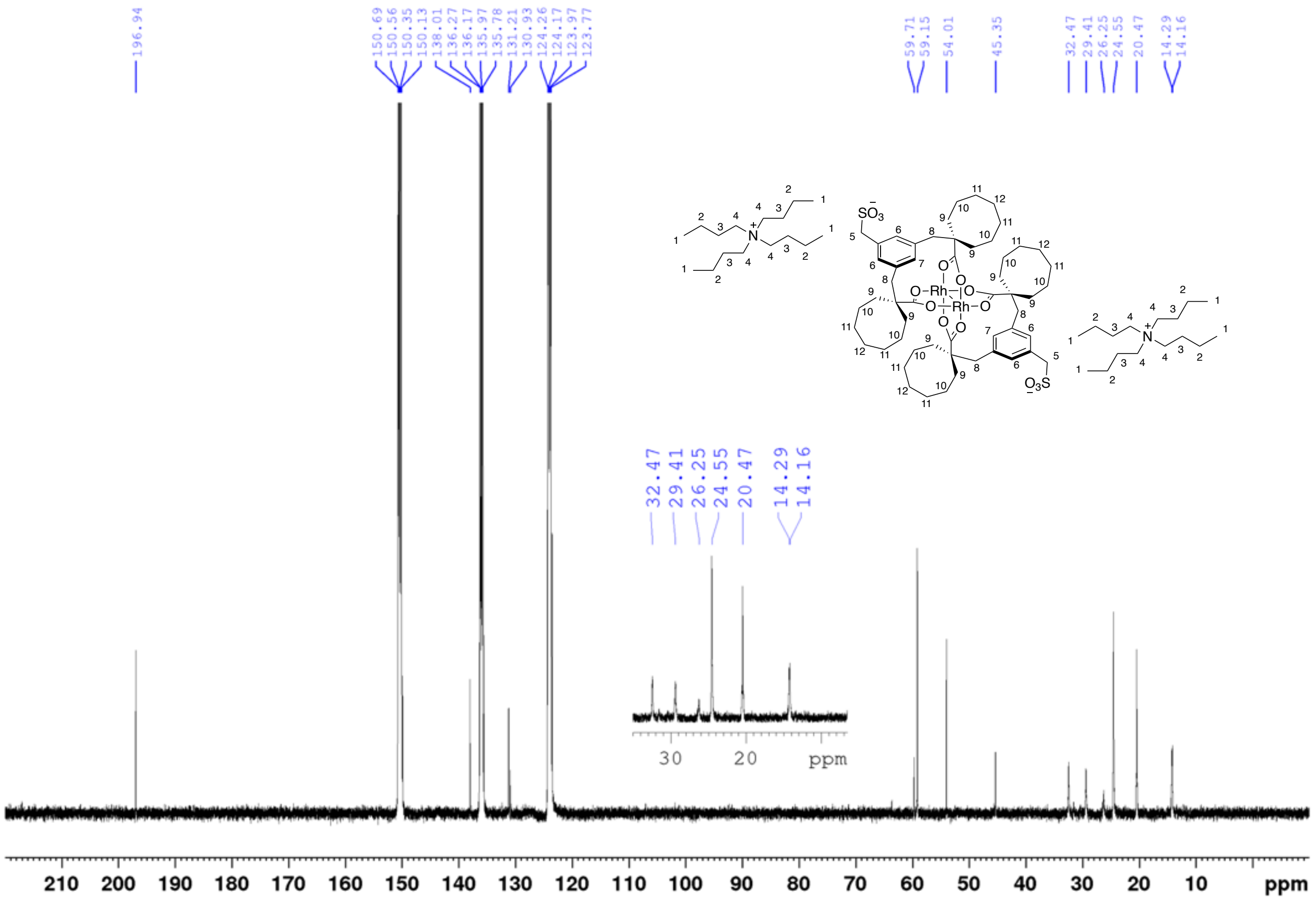

^{1}H NMR (400 MHz, $CDCl_3$) for *(R)-((1S,2S,4S,5R)-5-ethylquinuclidin-2-yl)(6-methoxyquinolin-4-yl)methanol (Dihydroquinine)*

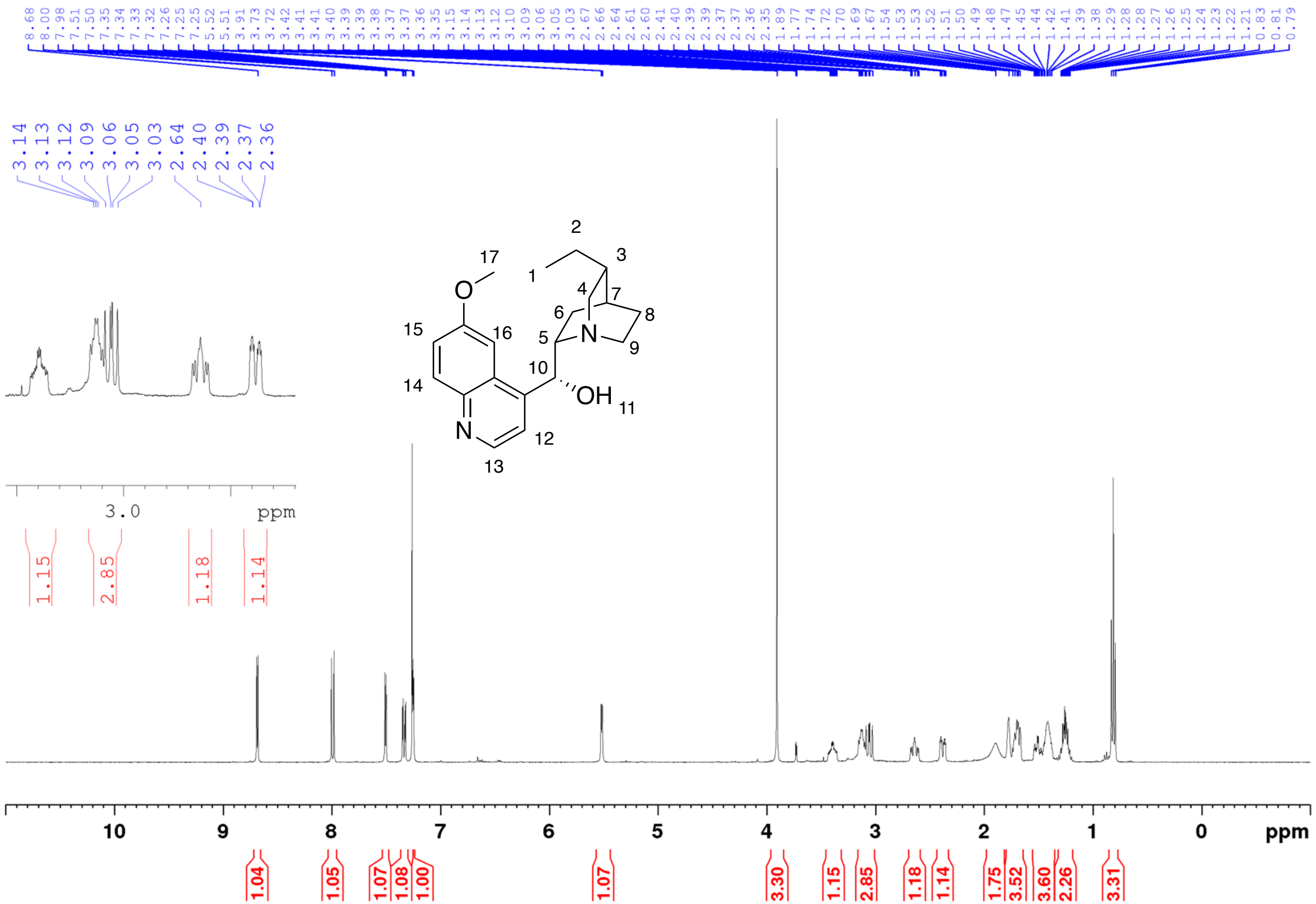

^{13}C NMR (101 MHz, $CDCl_3$) for *(R)-((1S,2S,4S,5R)-5-ethylquinuclidin-2-yl)(6-methoxyquinolin-4-yl)methanol (Dihydroquinine)*

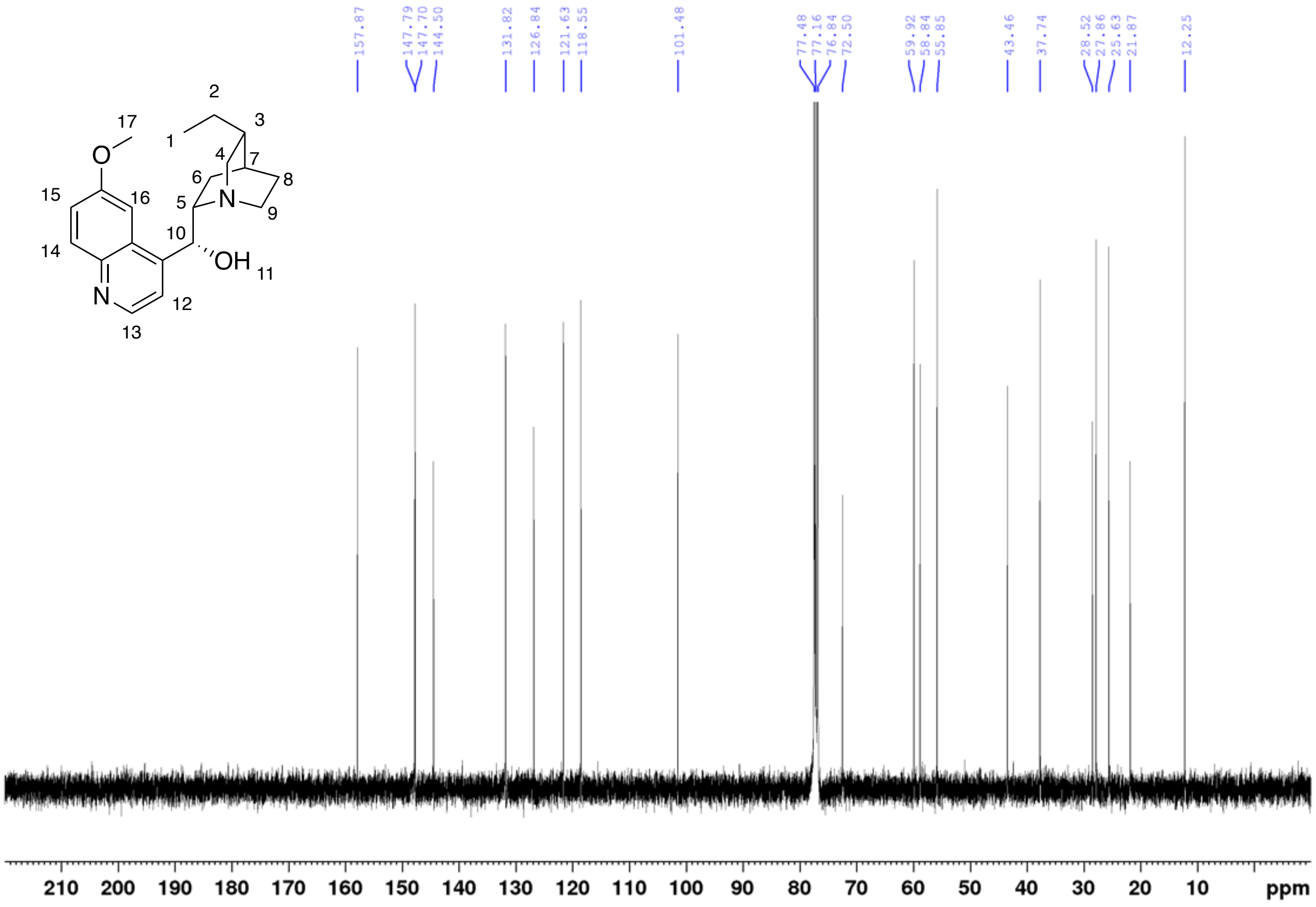

¹H NMR (500 MHz, $CDCl_3$) *(S)-((1S,2R,4S,5R)-5-ethylquinuclidin-2-yl)(6-methoxyquinolin-4-yl)methanol (Dihydroquinidine)*

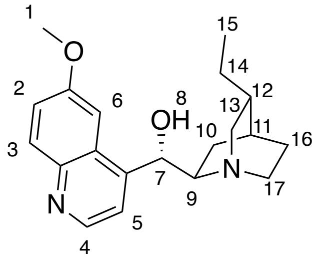

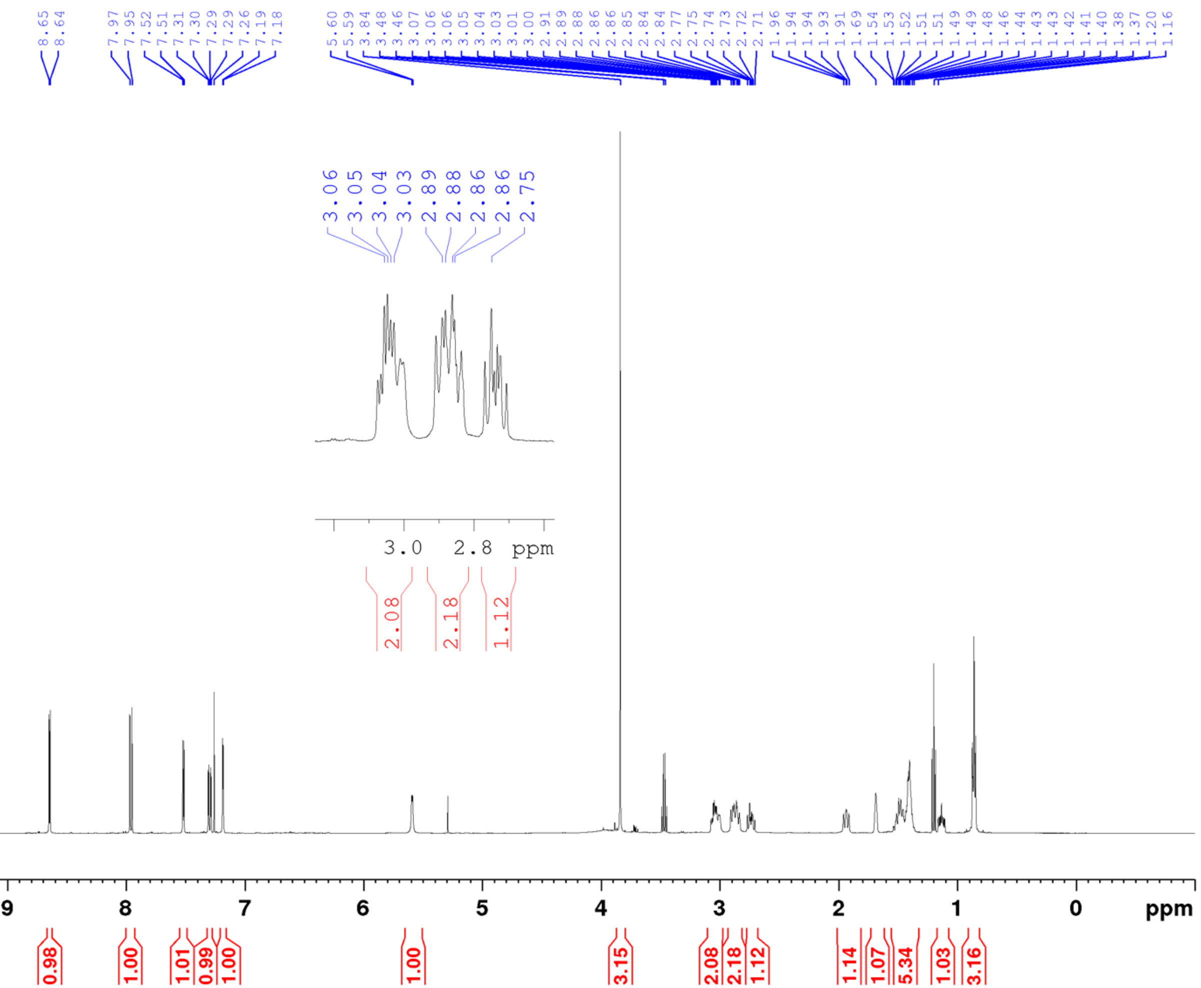

¹³C NMR (126 MHz, $CDCl_3$) *(S)-((1S,2R,4S,5R)-5-ethylquinuclidin-2-yl)(6-methoxyquinolin-4-yl)methanol (Dihydroquinidine)*

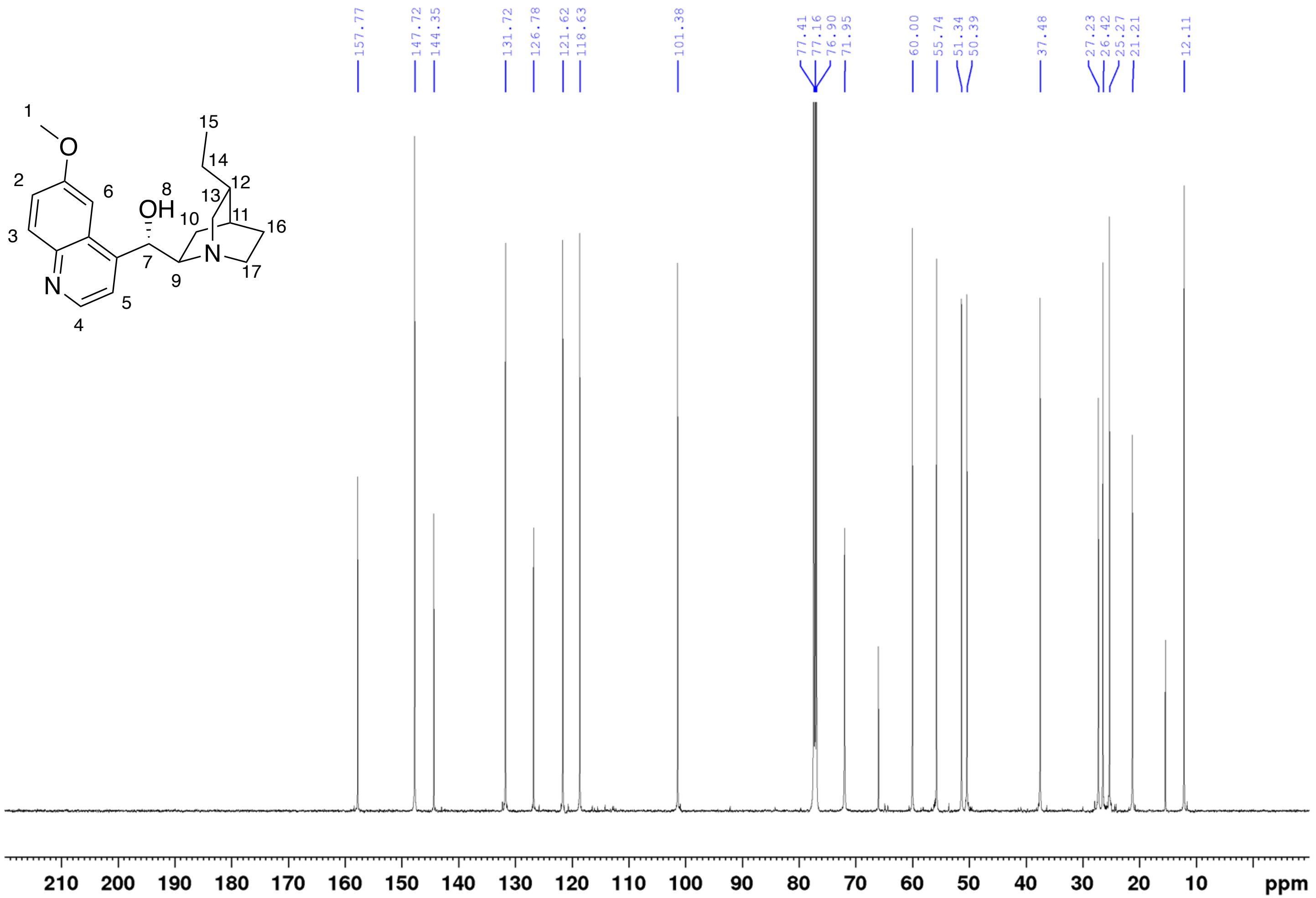

¹H NMR (500 MHz, $CDCl_3$) for *(1S,2S,4S,5R)-5-ethyl-2-((R)-hydroxy(6-methoxyquinolin-4-yl)methyl)-1-((3,3'',5,5''-tetra-tert-butyl-[1,1':3',1''-terphenyl]-5'-yl)methyl)quinuclidin-1-ium bromide* (**1•Br**)

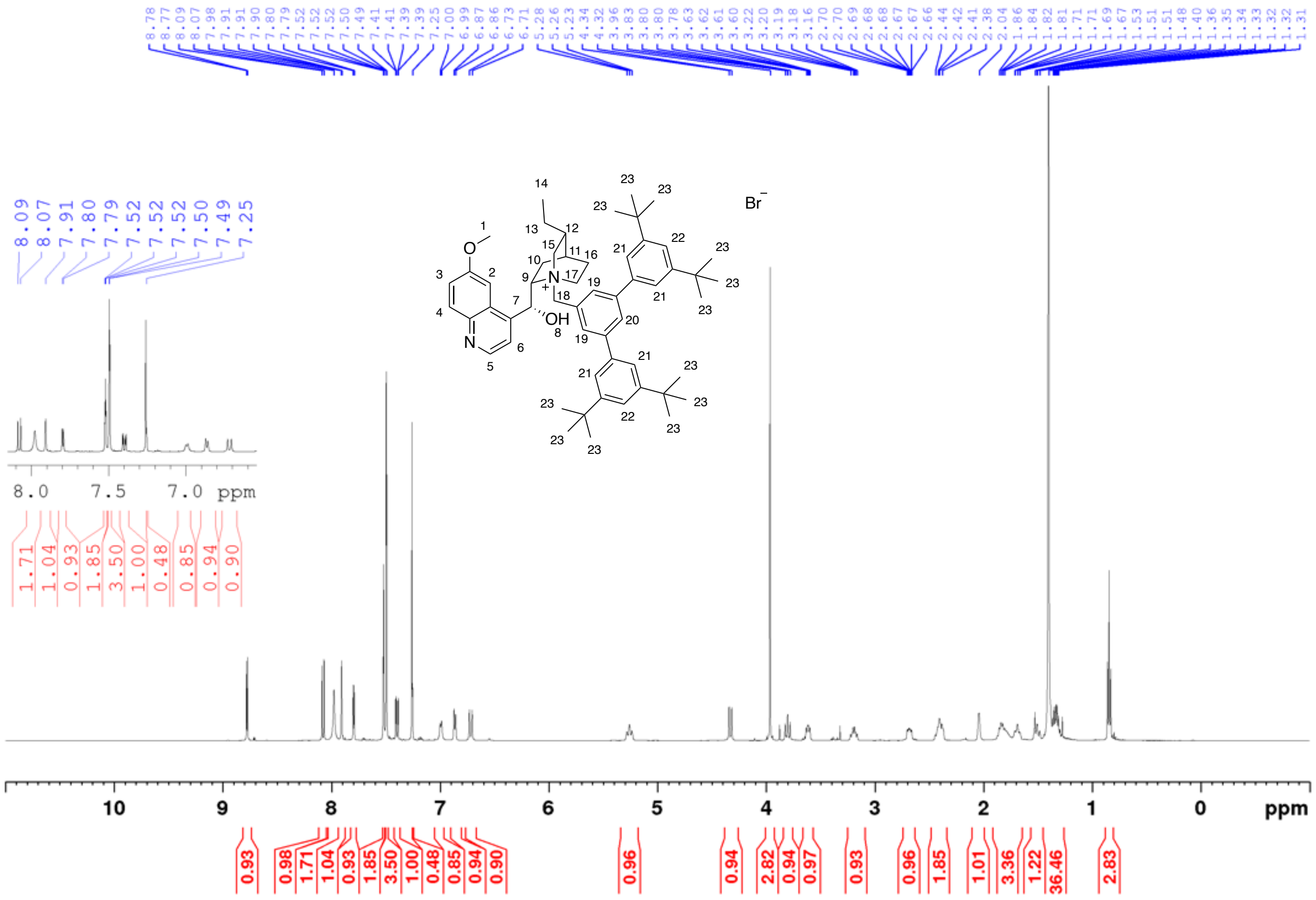

^{13}C NMR (126 MHz, $CDCl_3$) for *(1S,2S,4S,5R)-5-ethyl-2-((R)-hydroxy(6-methoxyquinolin-4-yl)methyl)-1-((3,3'',5,5''-tetra-tert-butyl-[1,1':3',1''-terphenyl]-5'-yl)methyl)quinuclidin-1-ium bromide* (**1•Br**)

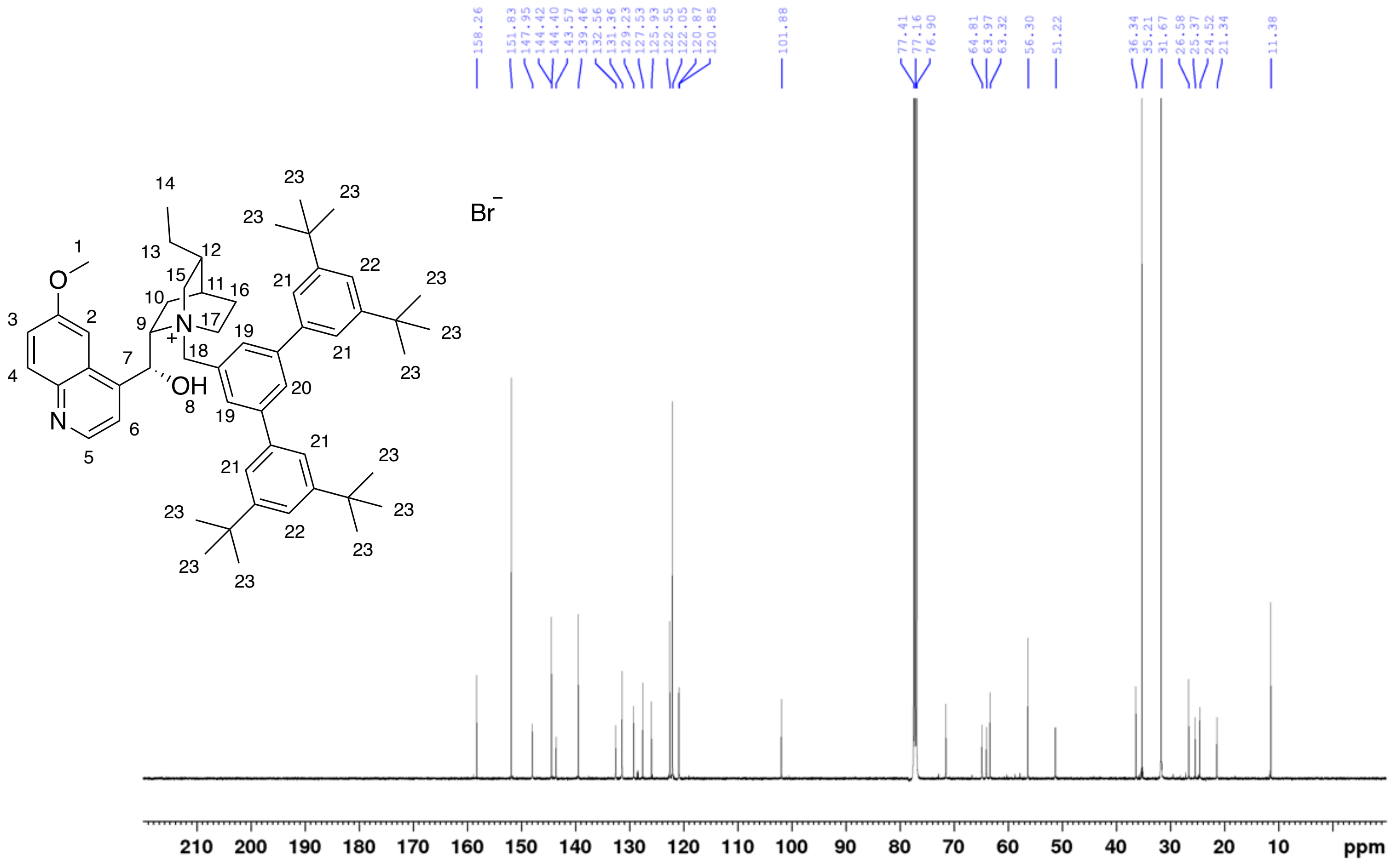

^{1}H NMR (500 MHz, $CDCl_3$) for *(1S,2R,4S,5R)-5-ethyl-2-((S)-hydroxy(6-methoxyquinolin-4-yl)methyl)-1-((3,3'',5,5''-tetra-tert-butyl-[1,1':3',1''-terphenyl]-5'-yl)methyl)quinuclidin-1-ium bromide* (**2a•Br**)

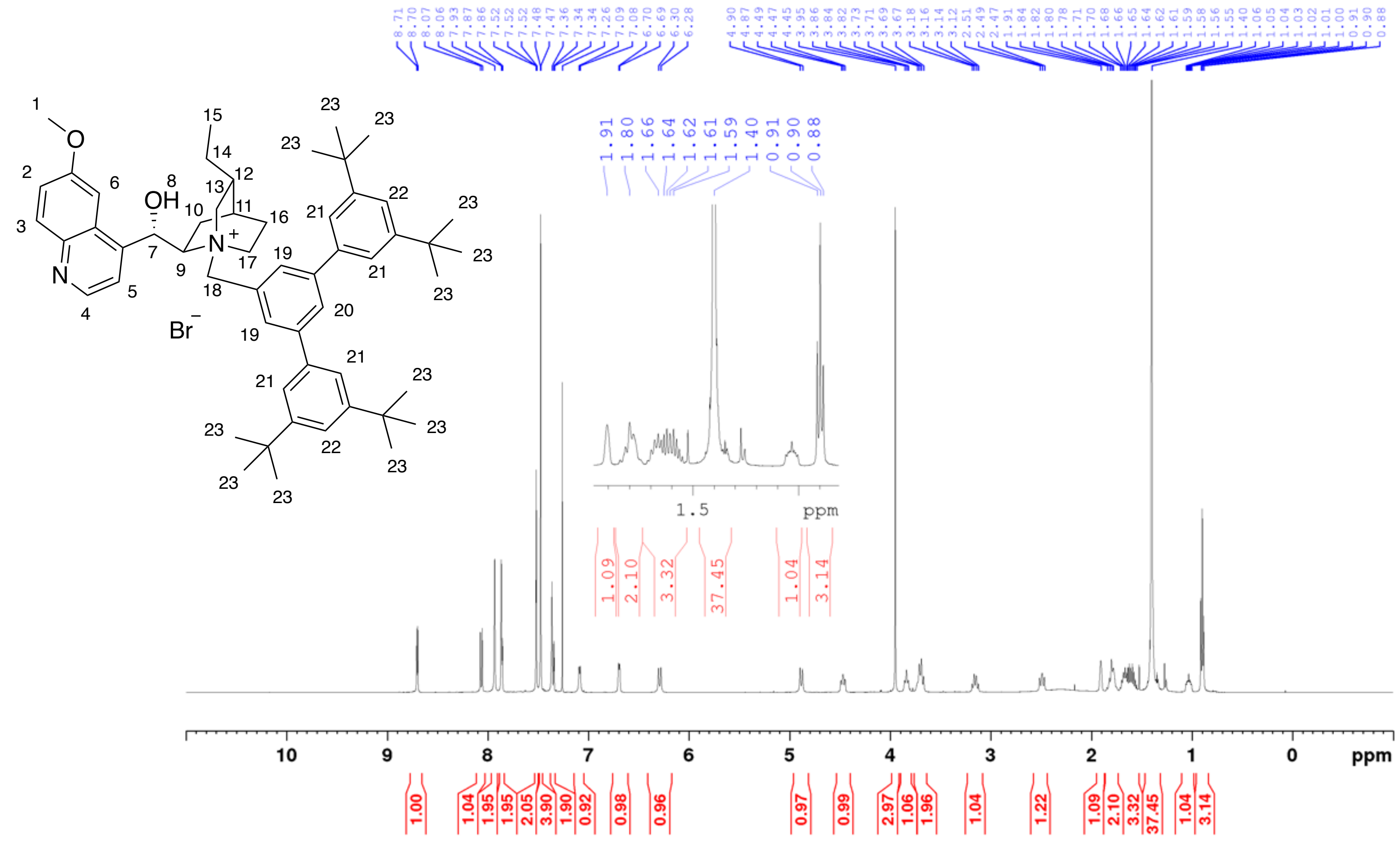

^{13}C NMR (126 MHz, $CDCl_3$) for *(1S,2R,4S,5R)-5-ethyl-2-((S)-hydroxy(6-methoxyquinolin-4-yl)methyl)-1-((3,3'',5,5''-tetra-tert-butyl-[1,1':3',1''-terphenyl]-5'-yl)methyl)quinuclidin-1-ium bromide* (**2a•Br**)

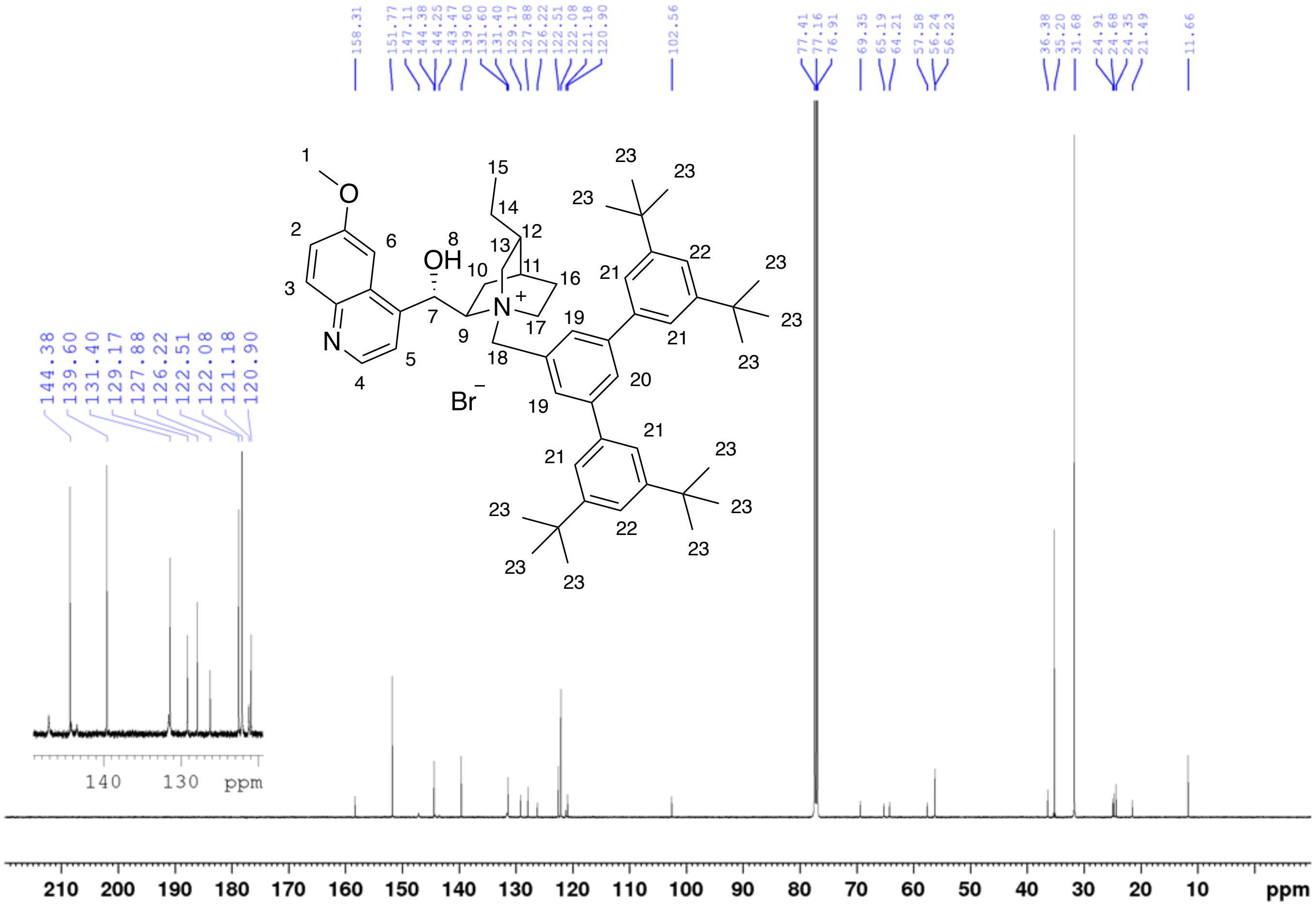

^{1}H NMR (400 MHz, $CDCl_3$) for *(1S,2R,4S,5R)-5-ethyl-2-((S)-hydroxy(6-methoxyquinolin-4-yl)methyl)-1-((3,3'',5,5''-tetrakis(trifluoromethyl)-[1,1':3',1''-terphenyl]-5'-yl)methyl)quinuclidin-1-ium bromide* (**2b•Br**)

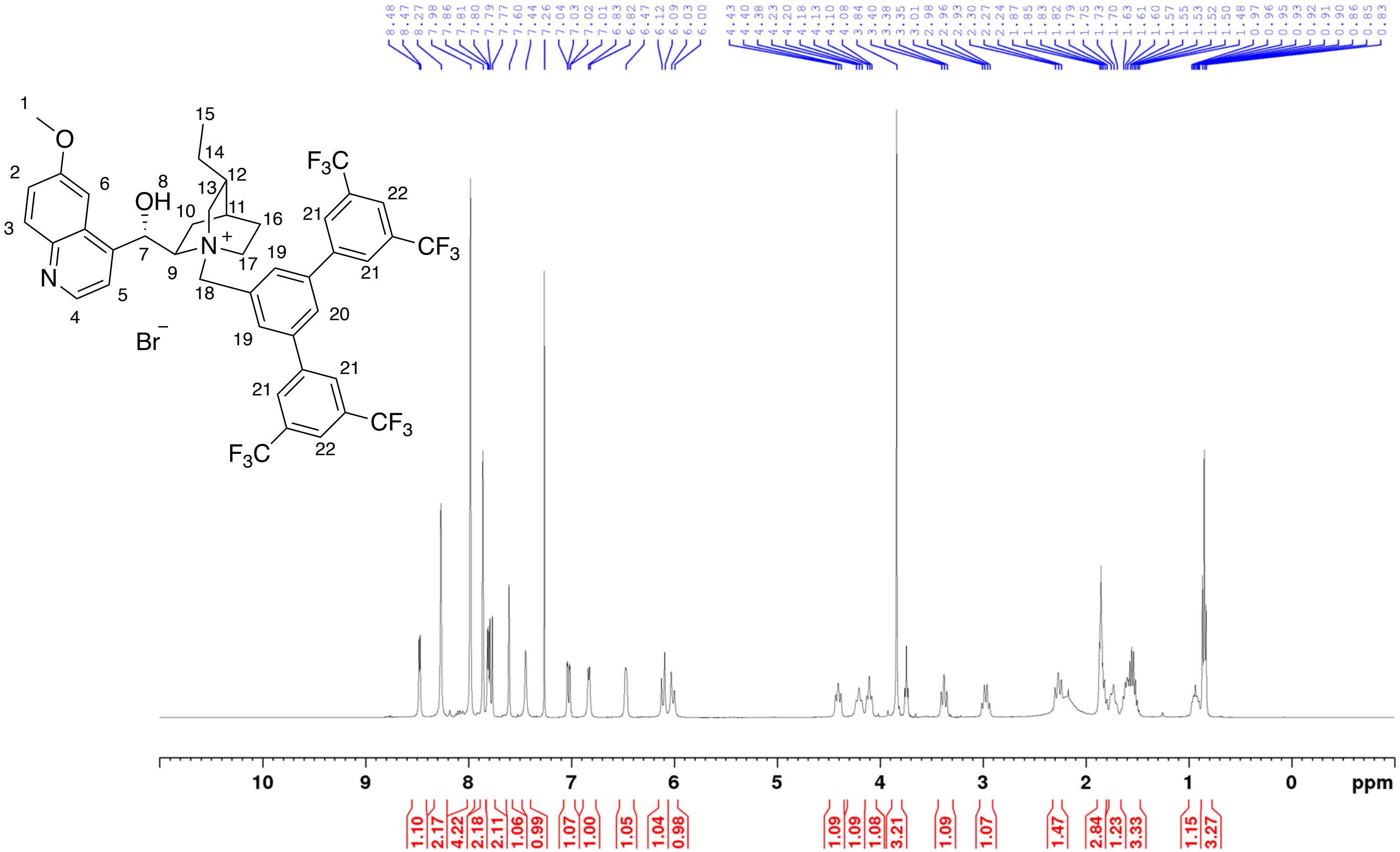

^{13}C NMR (101 MHz, $CDCl_3$) for *(1S,2R,4S,5R)-5-ethyl-2-((S)-hydroxy(6-methoxyquinolin-4-yl)methyl)-1-((3,3'',5,5''-tetrakis(trifluoromethyl)-[1,1':3',1''-terphenyl]-5'-yl)methyl)quinuclidin-1-ium bromide* (**2b•Br**)

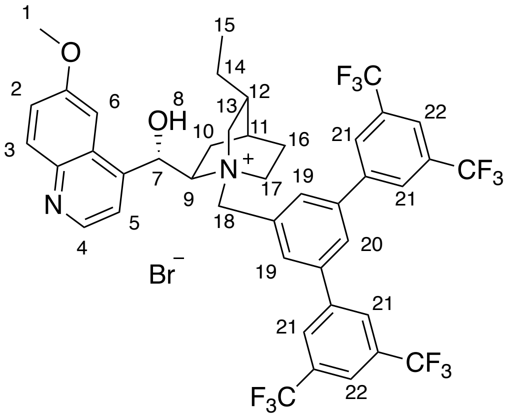

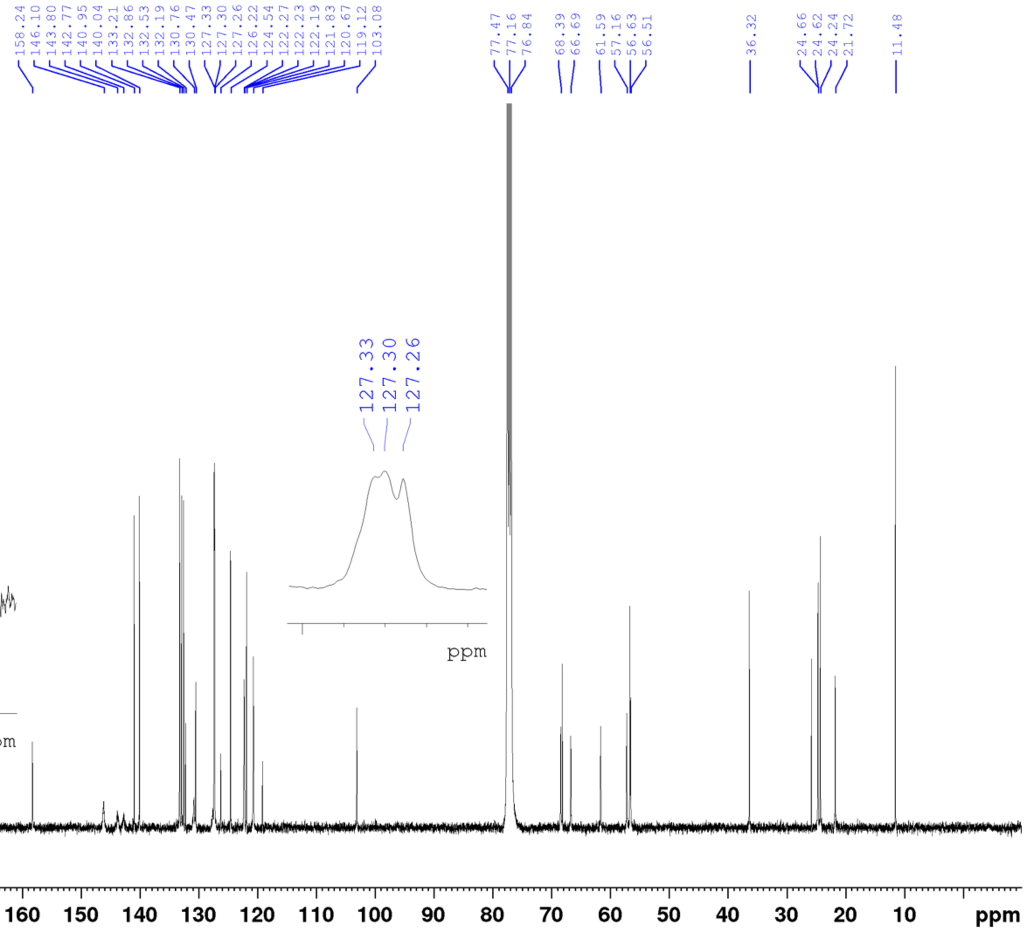

¹⁹F NMR (376 MHz, $CDCl_3$) for *(1S,2R,4S,5R)-5-ethyl-2-((S)-hydroxy(6-methoxyquinolin-4-yl)methyl)-1-((3,3'',5,5''-tetrakis(trifluoromethyl)-[1,1':3',1''-terphenyl]-5'-yl)methyl)quinuclidin-1-ium bromide* (**2b•Br**)

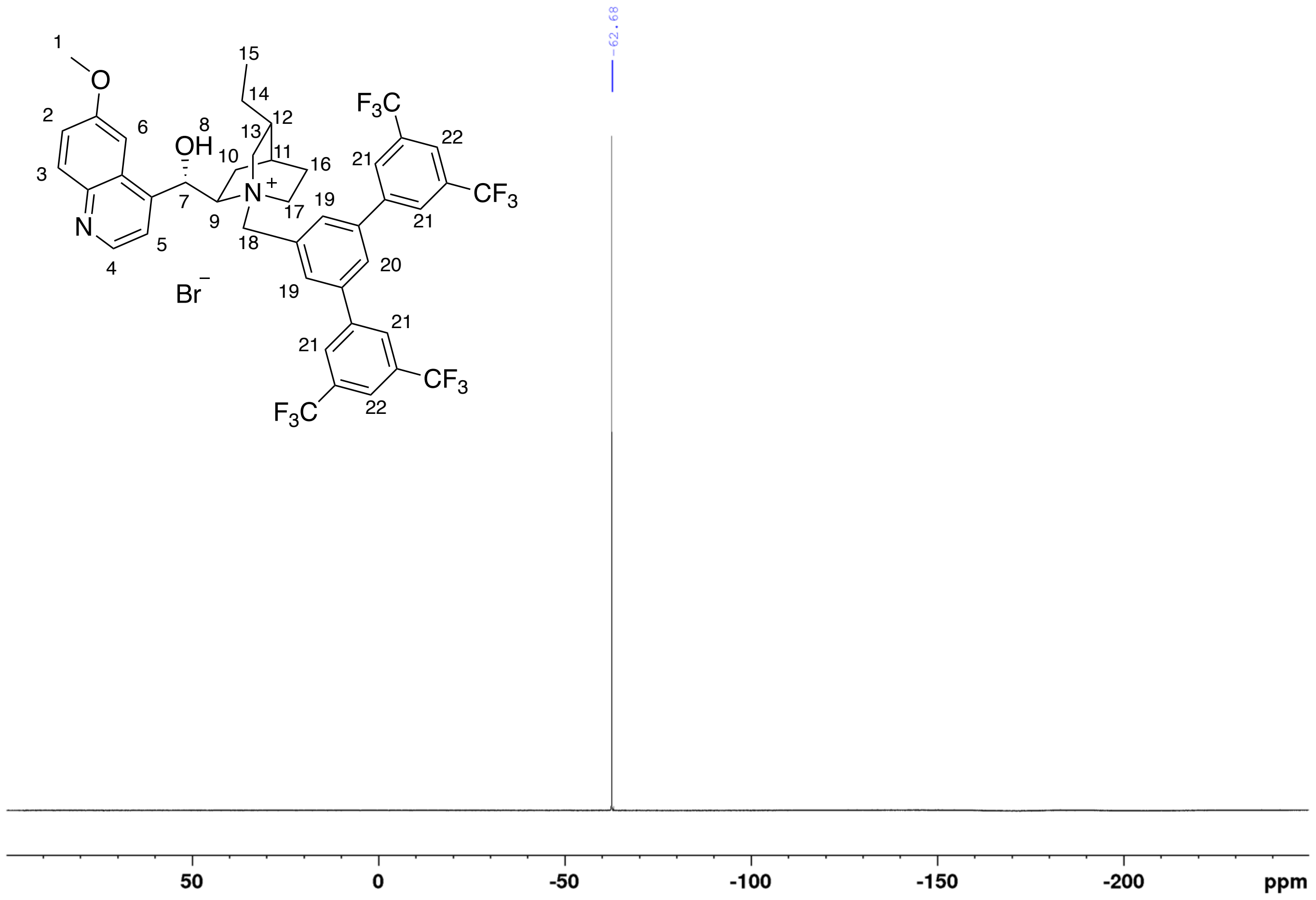

^{1}H NMR (400 MHz, $CDCl_3$) for *[1,1':3',1''-terphenyl]-5'-ylmethanol*

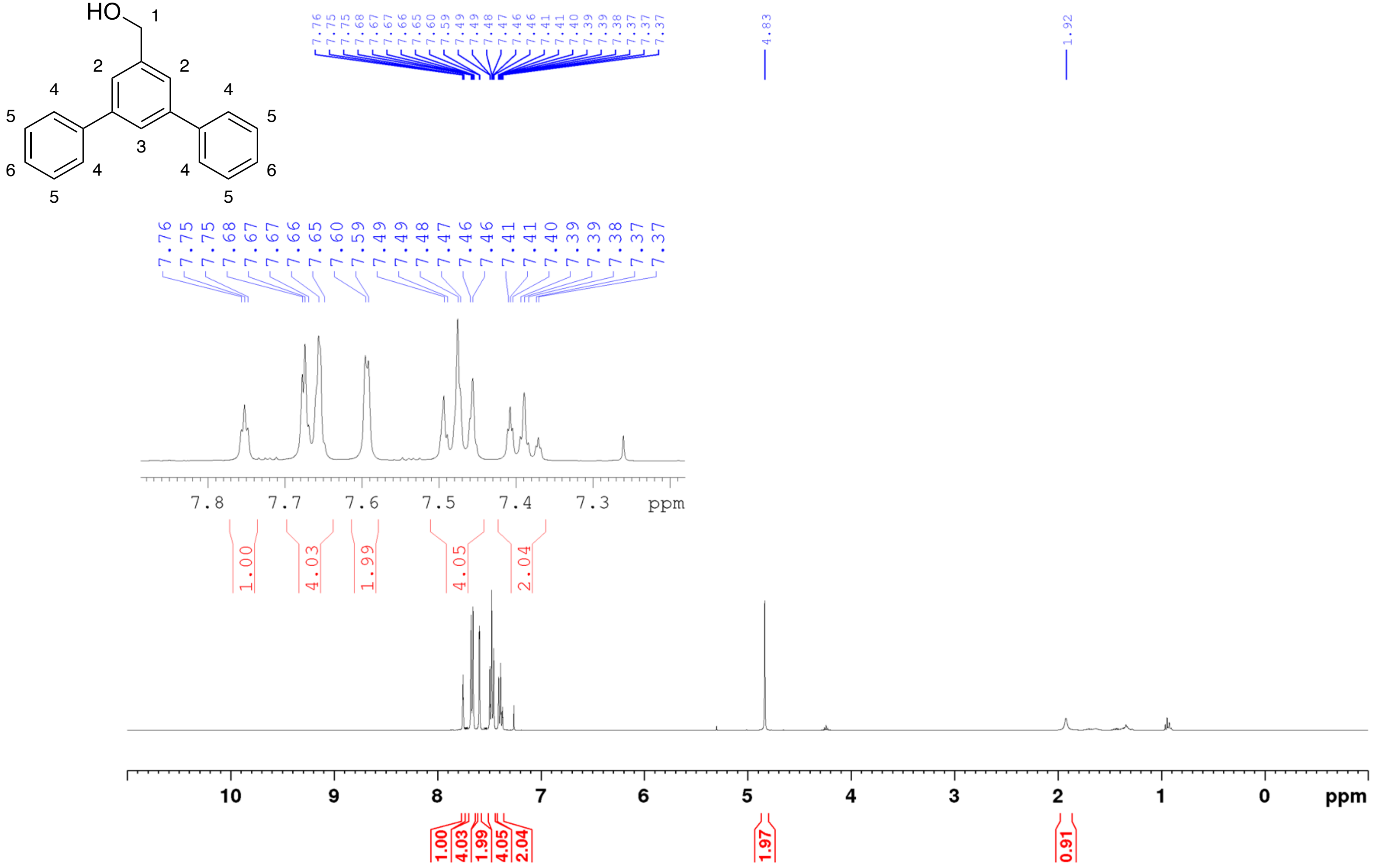

^{13}C NMR (101 MHz, $CDCl_3$) for *[1,1':3',1''-terphenyl]-5'-ylmethanol*

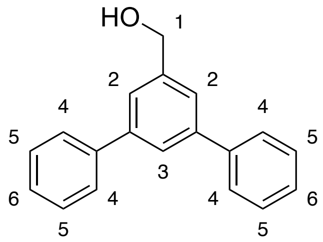

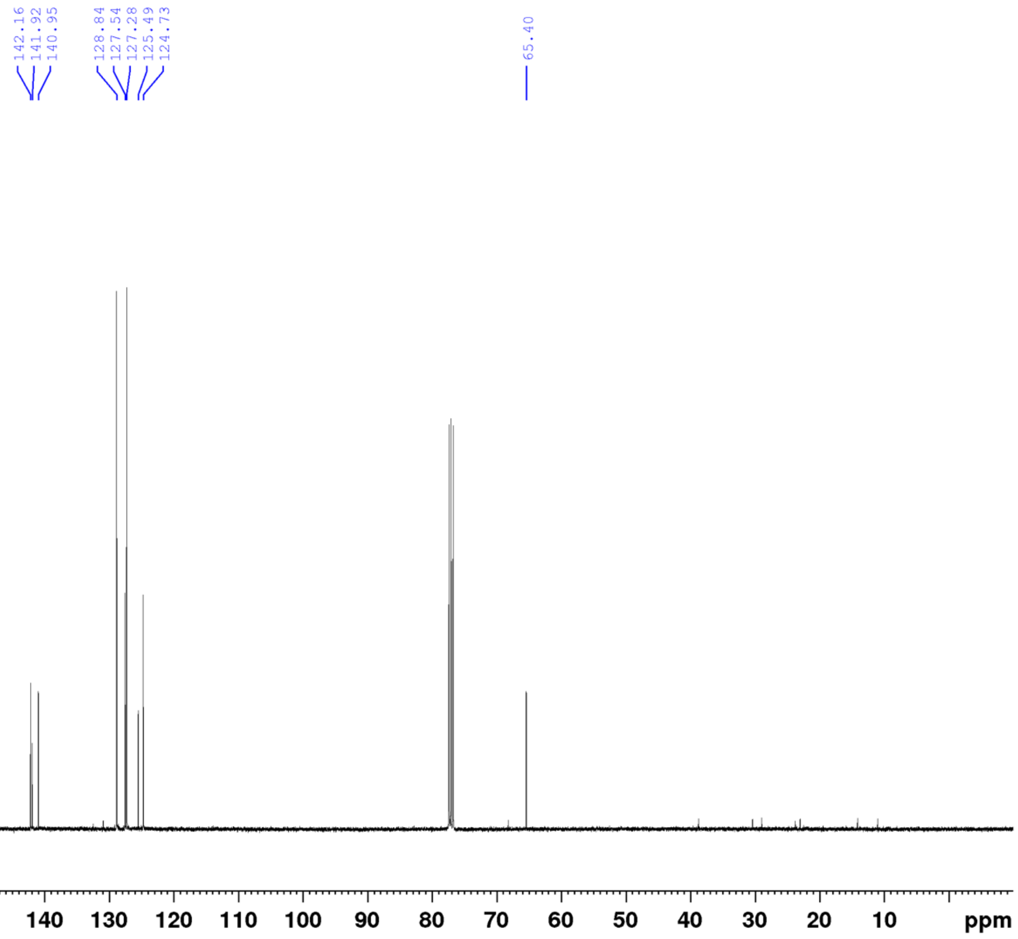

^{1}H NMR (400 MHz, $CDCl_3$) for *5'-(bromomethyl)-1,1':3',1''-terphenyl*

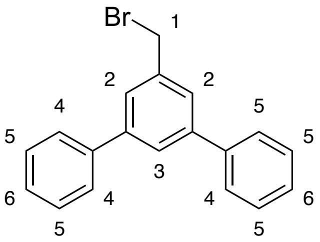

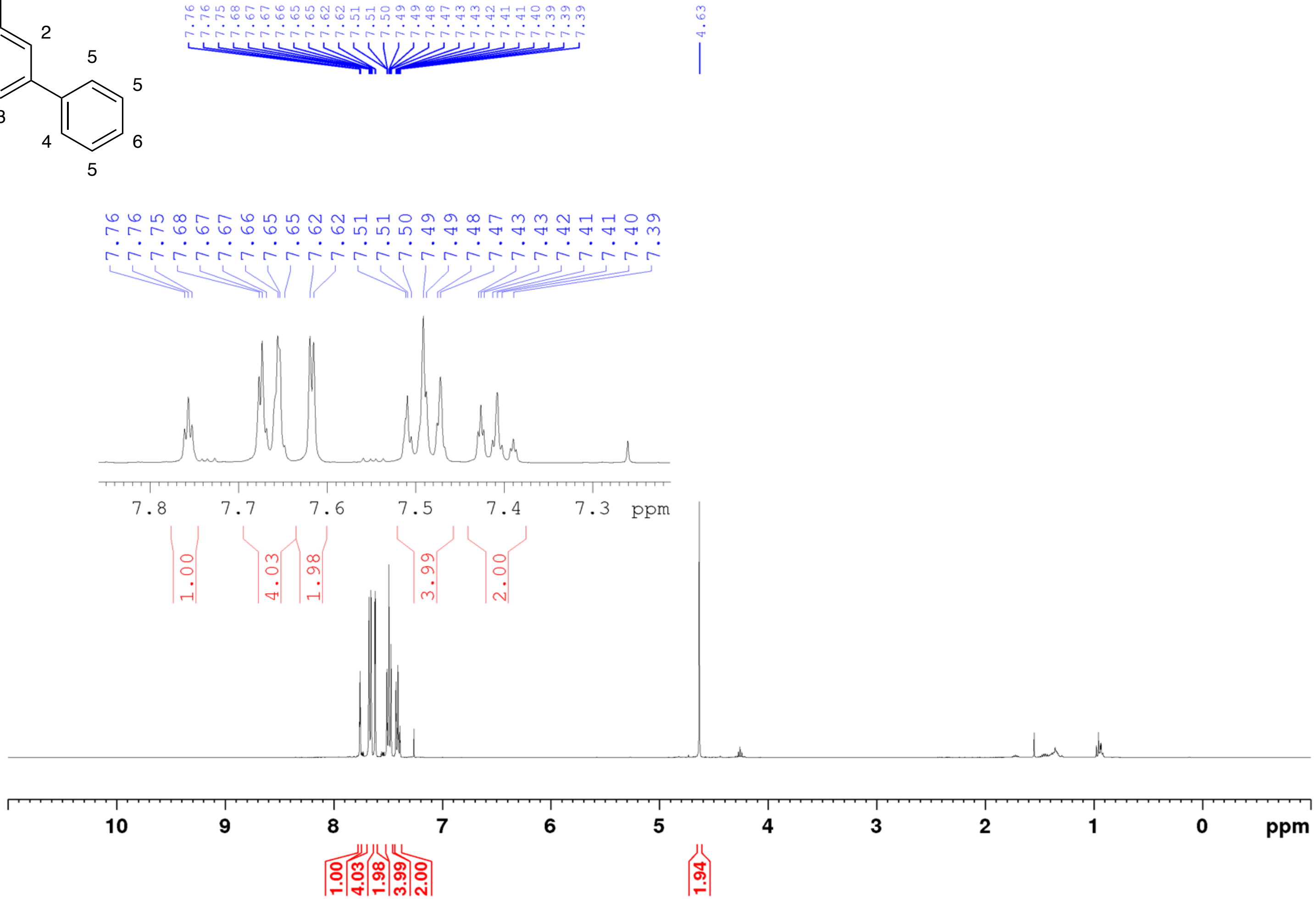

^{13}C NMR (101 MHz, $CDCl_3$) *5'-(bromomethyl)-1,1':3',1''-terphenyl*

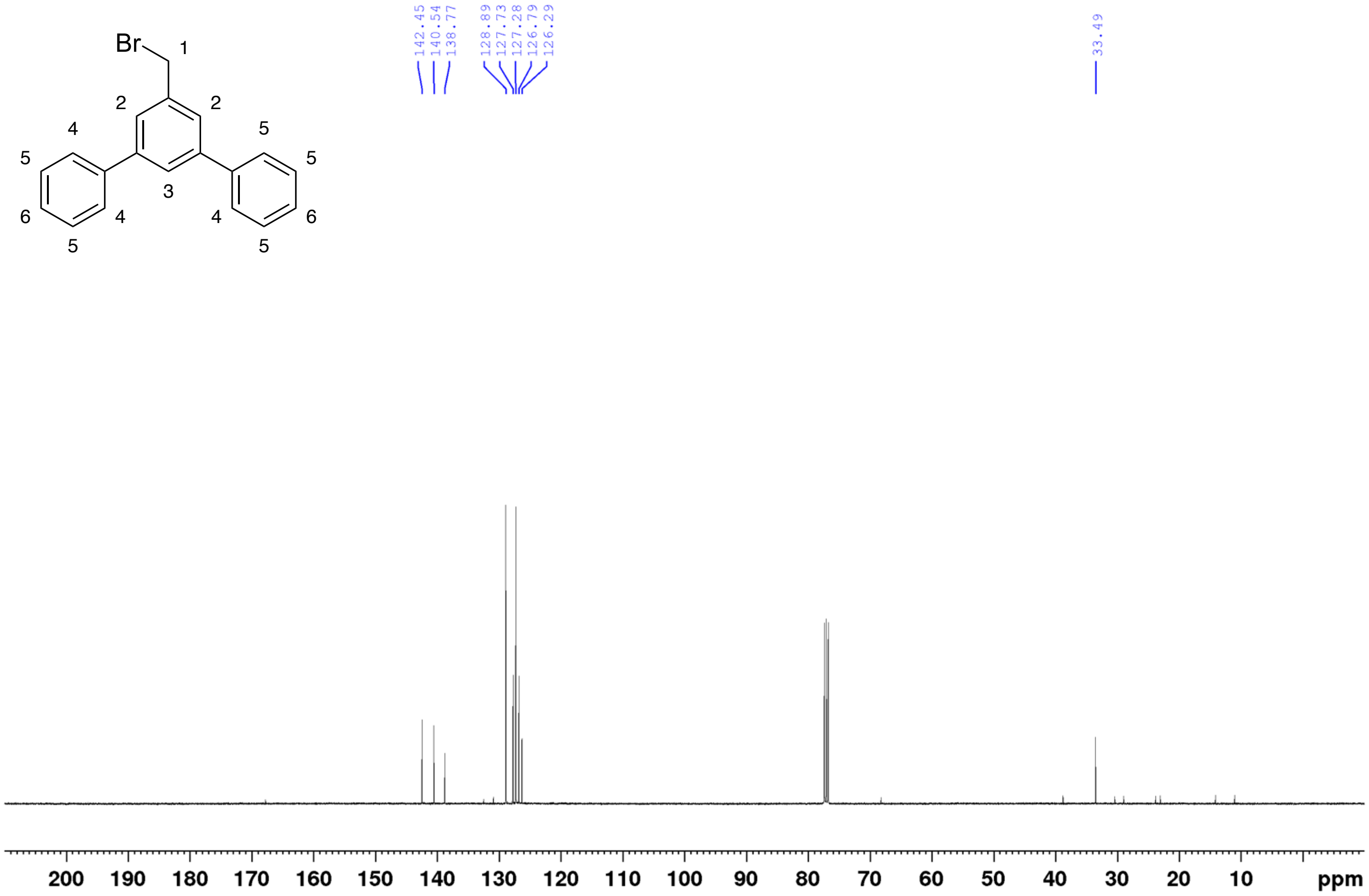

^{1}H NMR (500 MHz, $CDCl_3$) for *(1S,2R,4S,5R)-1-([1,1':3',1''-terphenyl]-5'-ylmethyl)-5-ethyl-2-((S)-hydroxy(6-methoxyquinolin-4-yl)methyl)quinuclidin-1-ium bromide* (**2c•Br**)

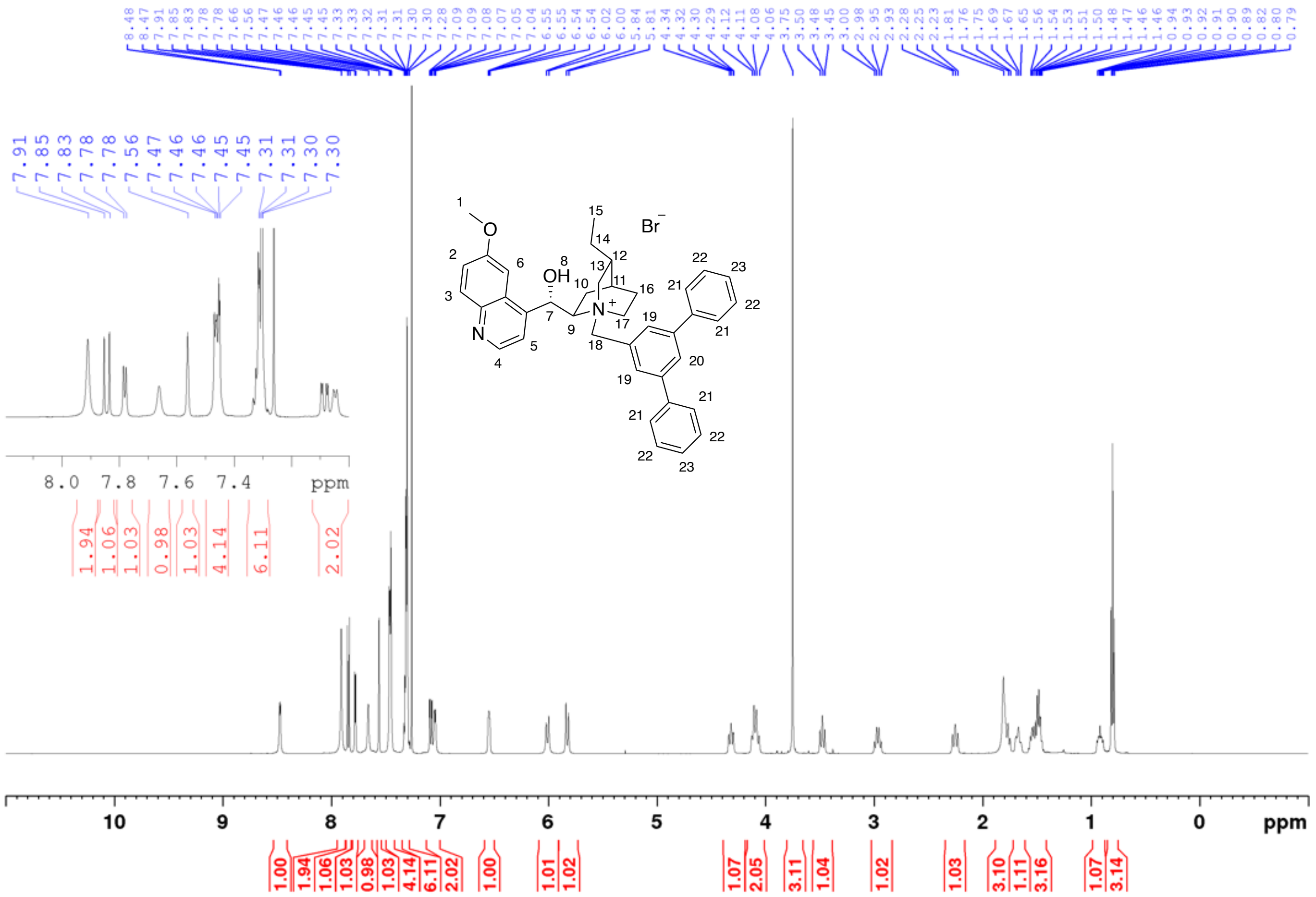

^{13}C NMR (126 MHz, $CDCl_3$) for *(1S,2R,4S,5R)-1-([1,1':3',1''-terphenyl]-5'-ylmethyl)-5-ethyl-2-((S)-hydroxy(6-methoxyquinolin-4-yl)methyl)quinuclidin-1-ium bromide* (**2c•Br**)

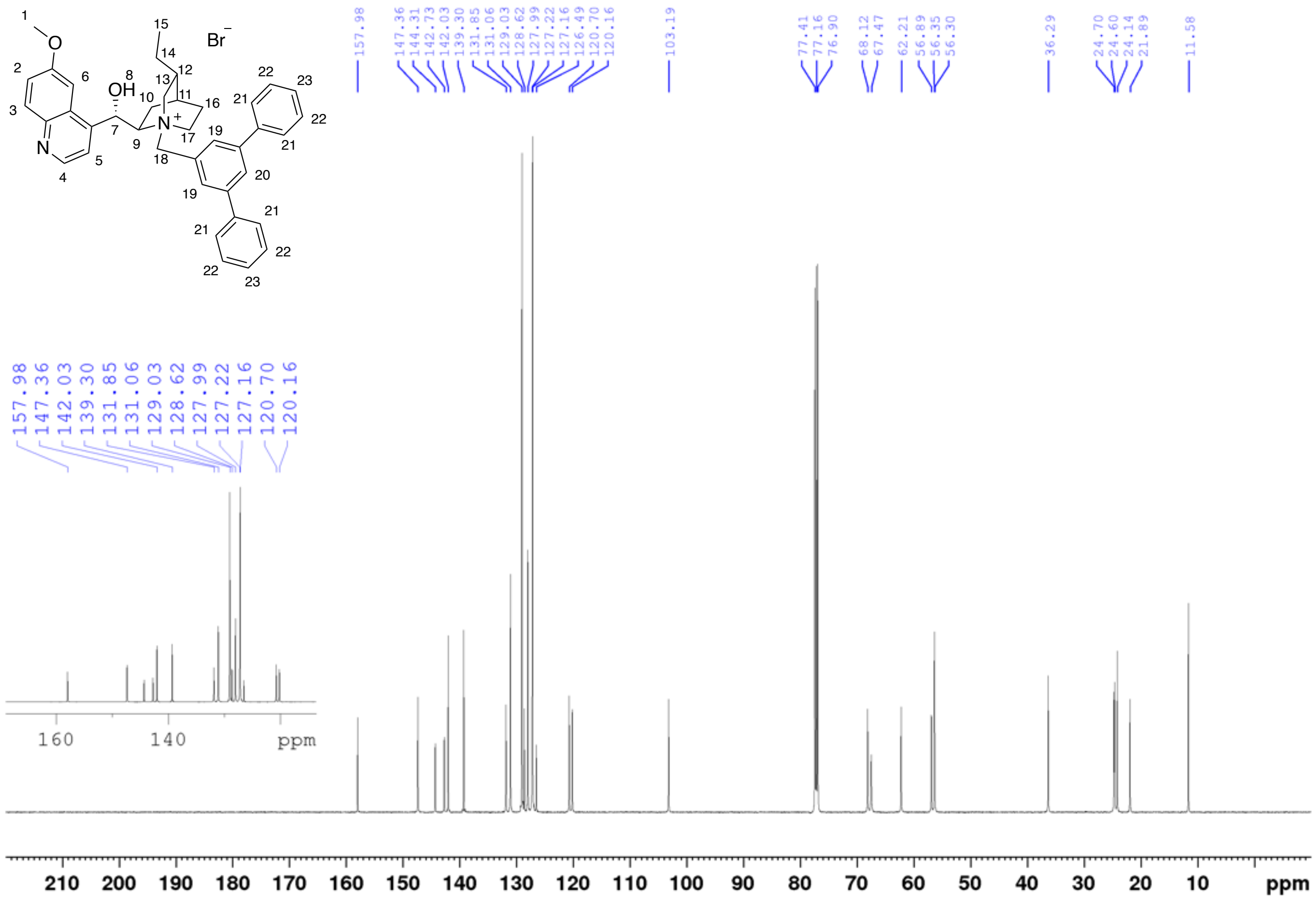

^{1}H NMR (400 MHz, $CDCl_3$) for *(1S,2R,4S,5R)-1-(3,5-di-tert-butylbenzyl)-5-ethyl-2-((S)-hydroxy(6-methoxyquinolin-4-yl)methyl)quinuclidin-1-ium bromide* (**2d•Br**)

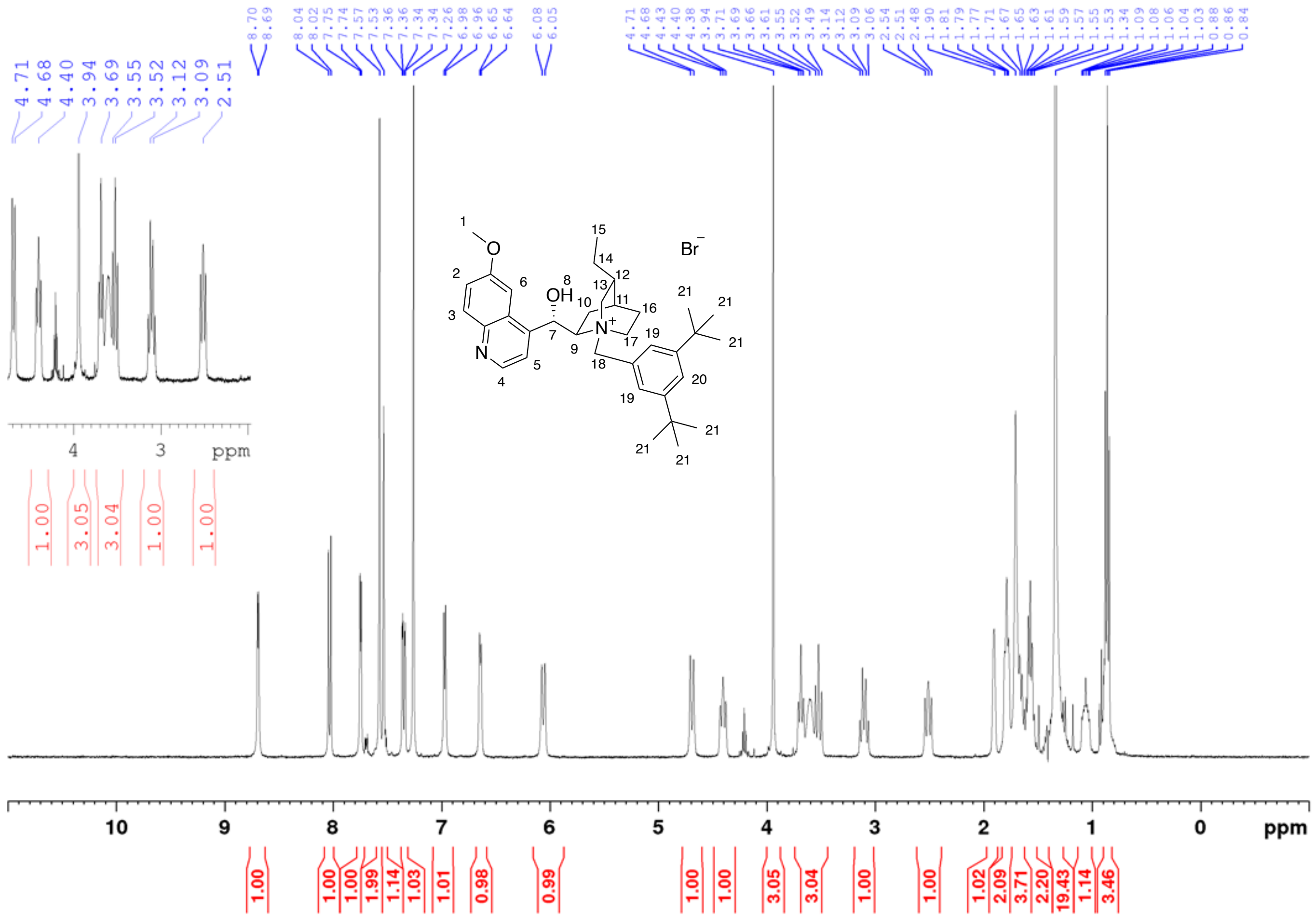

^{13}C NMR (126 MHz, $CDCl_3$) for *(1S,2R,4S,5R)-1-(3,5-di-tert-butylbenzyl)-5-ethyl-2-((S)-hydroxy(6-methoxyquinolin-4-yl)methyl)quinuclidin-1-ium bromide* (**2d•Br**)

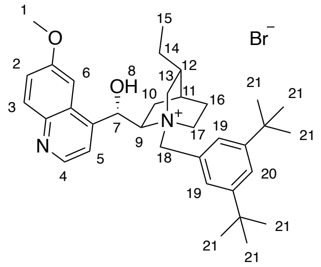

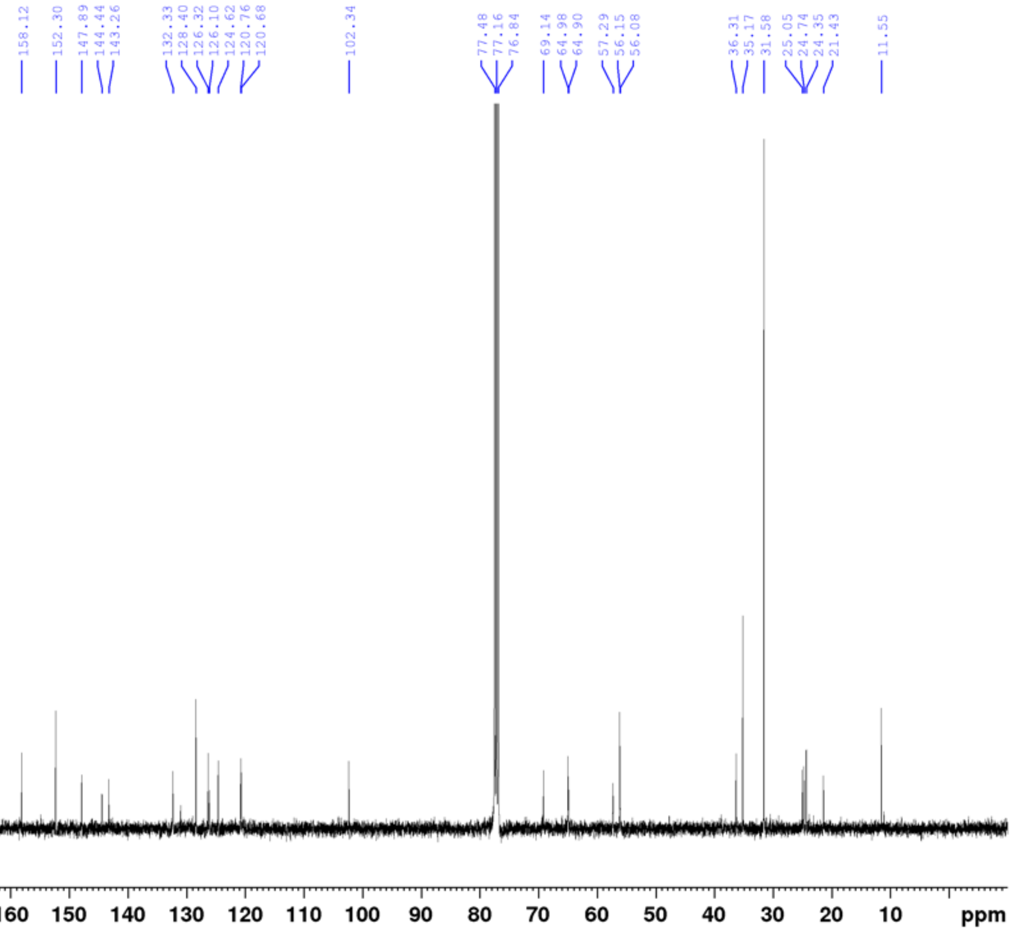

¹H NMR (400 MHz, $CDCl_3$) for *(1S,2R,4S,5R)-1-(3,5-bis(trifluoromethyl)benzyl)-5-ethyl-2-((S)-hydroxy(6-methoxyquinolin-4- yl)methyl)quinuclidin-1-ium bromide* (**2e•Br**)

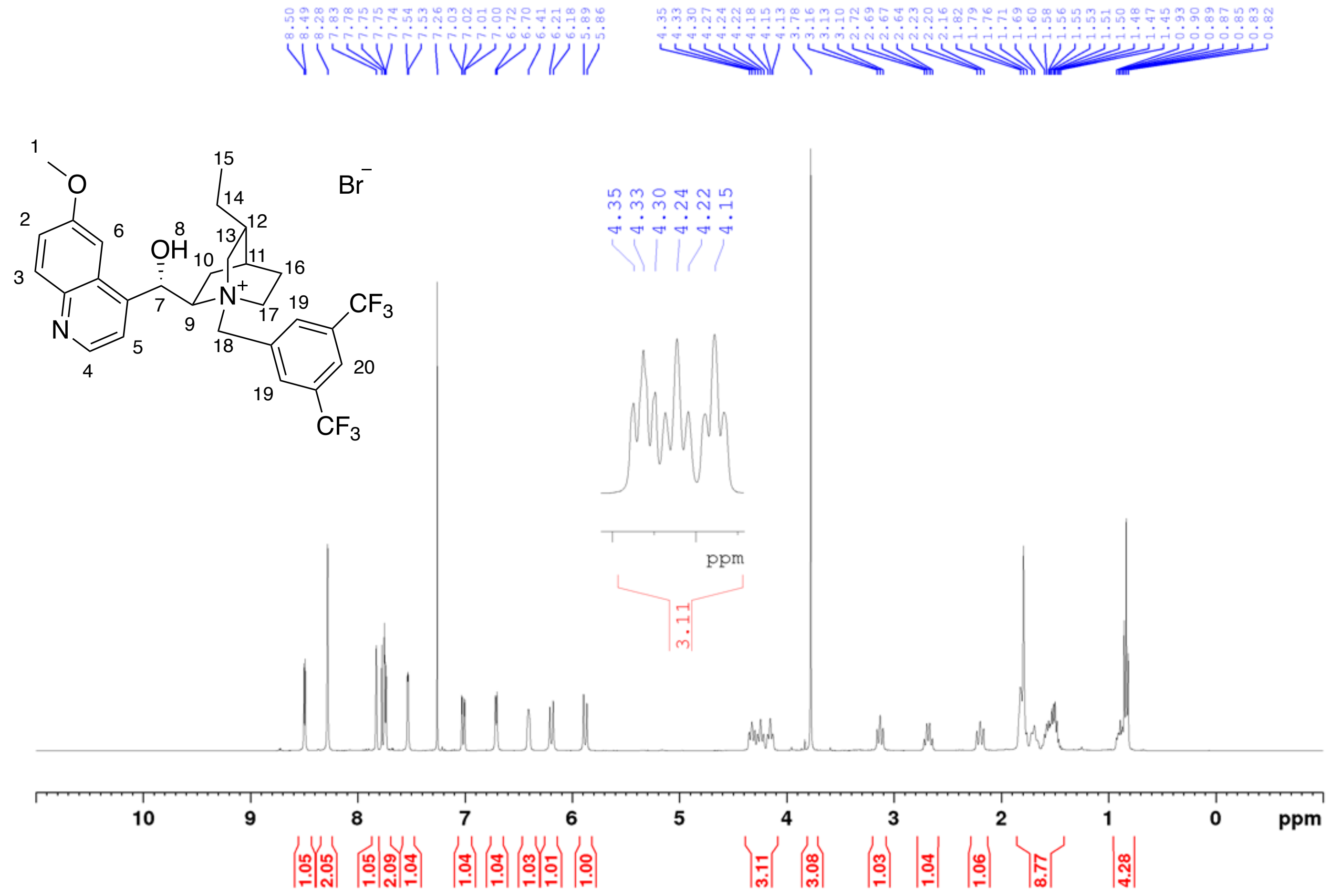

³C NMR (101 MHz, CDCl₃) for *(1S,2R,4S,5R)-1-(3,5-bis(trifluoromethyl)benzyl)-5-ethyl-2-((S)-hydroxy(6-methoxyquinolin-4- yl)methyl)quinuclidin-1-ium bromide* (**2e•Br**)

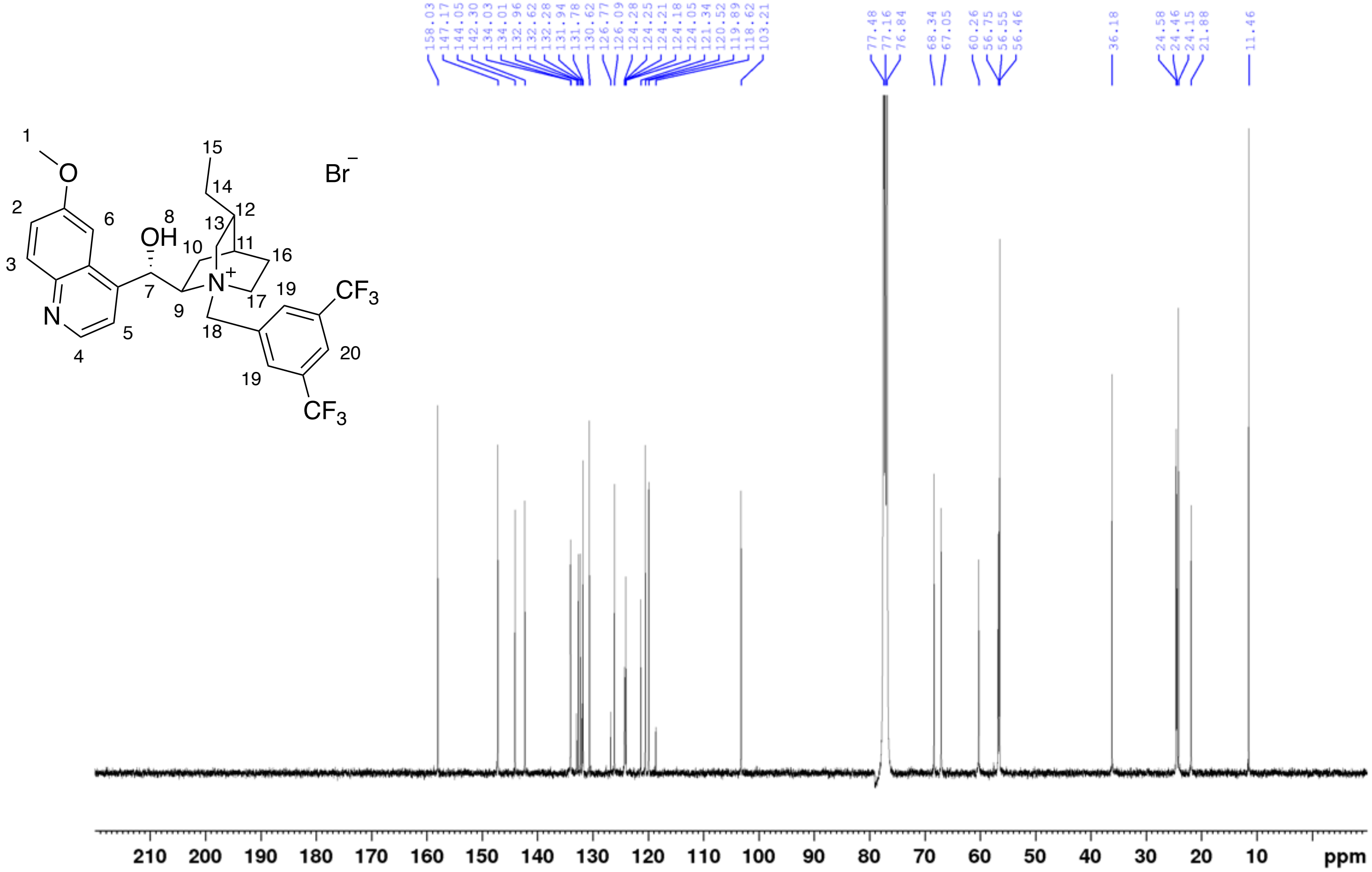

^{19}F NMR (376 MHz, $CDCl_3$) for *(1S,2R,4S,5R)-1-(3,5-bis(trifluoromethyl)benzyl)-5-ethyl-2-((S)-hydroxy(6-methoxyquinolin-4-yl)methyl)quinuclidin-1-ium bromide* (**2e•Br**)

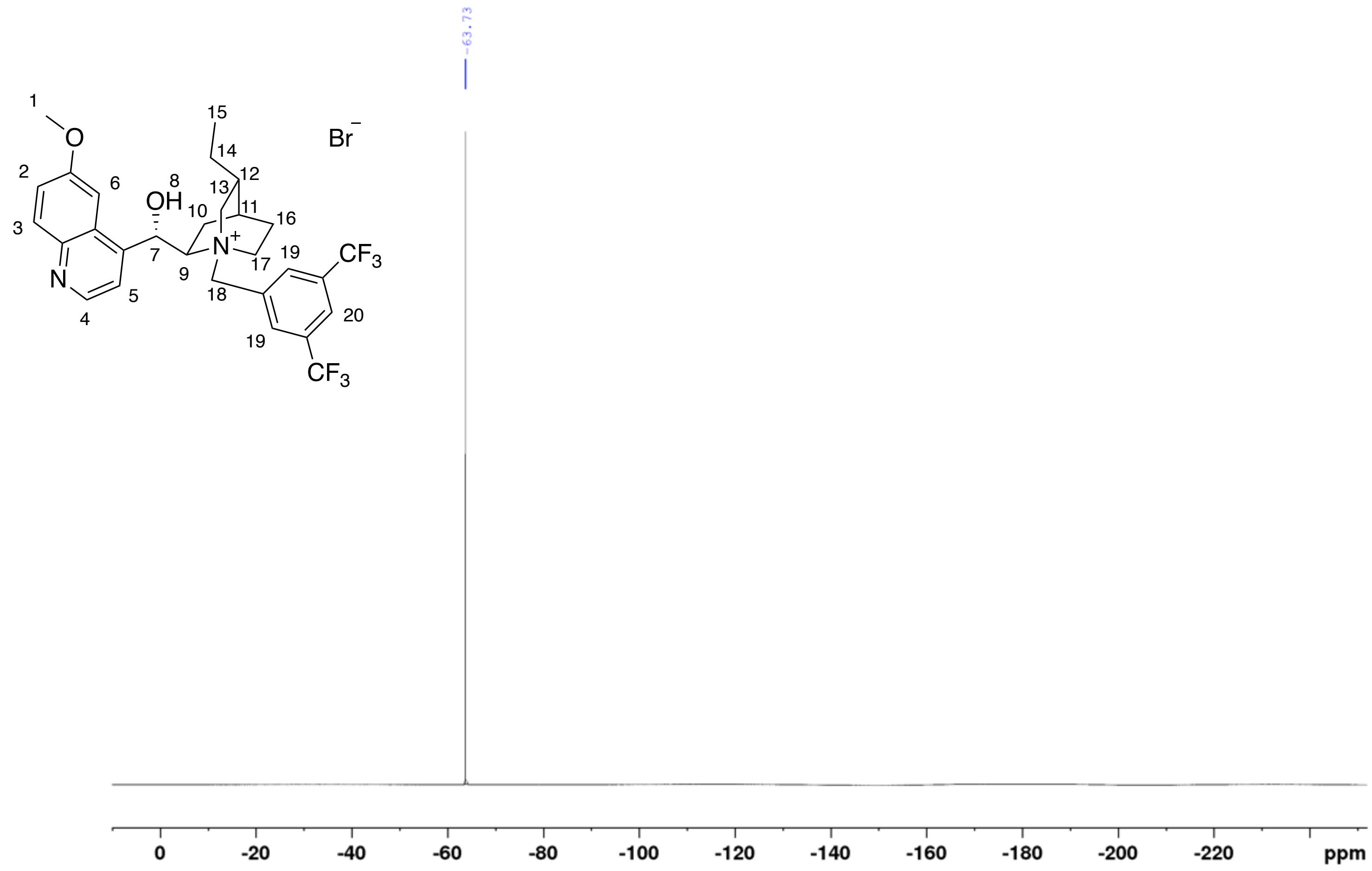

¹H NMR (400 MHz, $CDCl_3$) for *(1S,2R,4S,5R)-5-ethyl-2-((R)-hydroxy(6-methoxyquinolin-4-yl)methyl)-1-((3,3'',5,5''-tetra-tert-butyl-[1,1':3',1''-terphenyl]-5'-yl)methyl)quinuclidin-1-ium bromide* (**4•Br**)

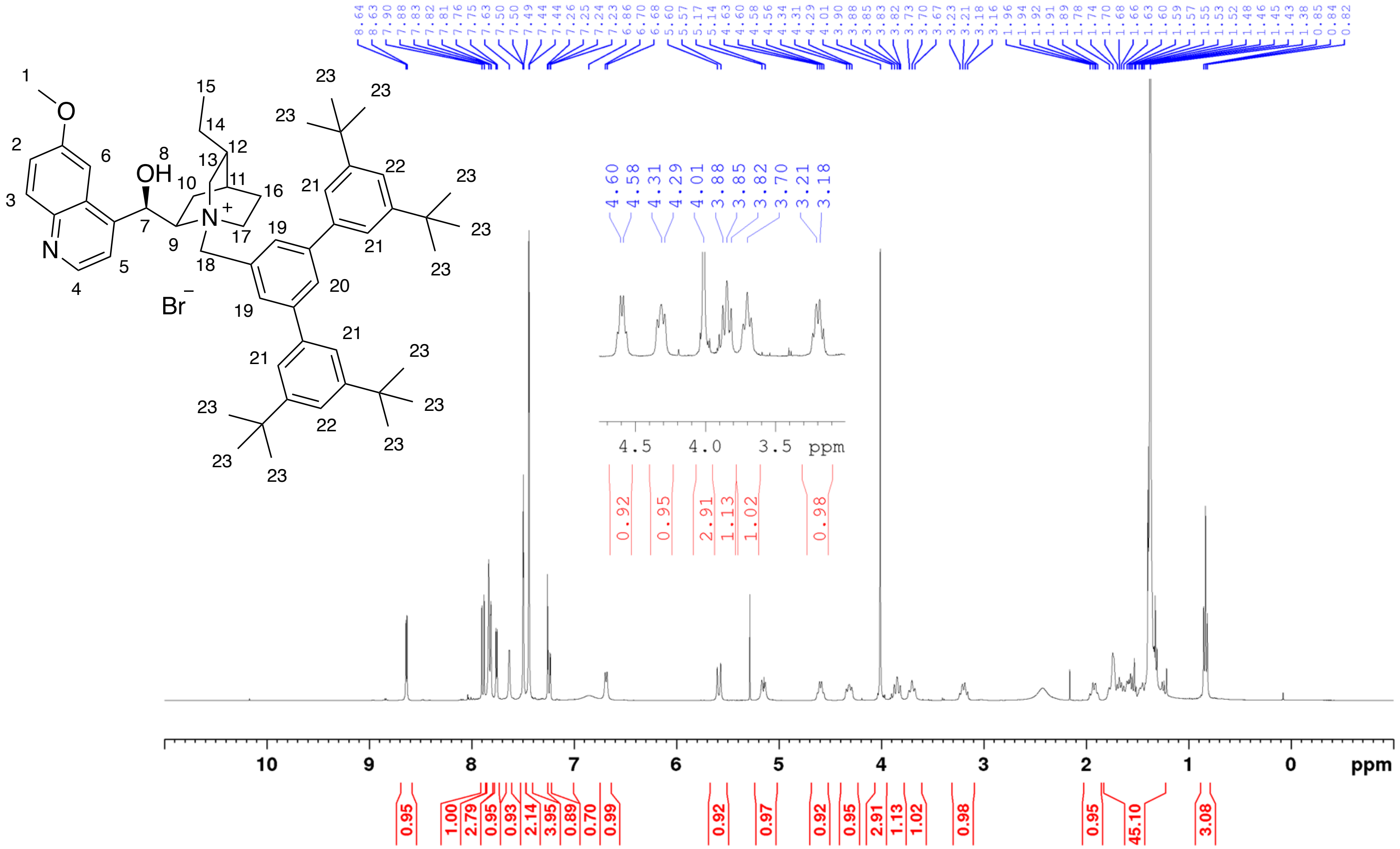

^{13}C NMR (101 MHz, $CDCl_3$) for *(1S,2R,4S,5R)-5-ethyl-2-((R)-hydroxy(6-methoxyquinolin-4-yl)methyl)-1-((3,3'',5,5''-tetra-tert-butyl-[1,1':3',1''-terphenyl]-5'-yl)methyl)quinuclidin-1-ium bromide* (**4•Br**)

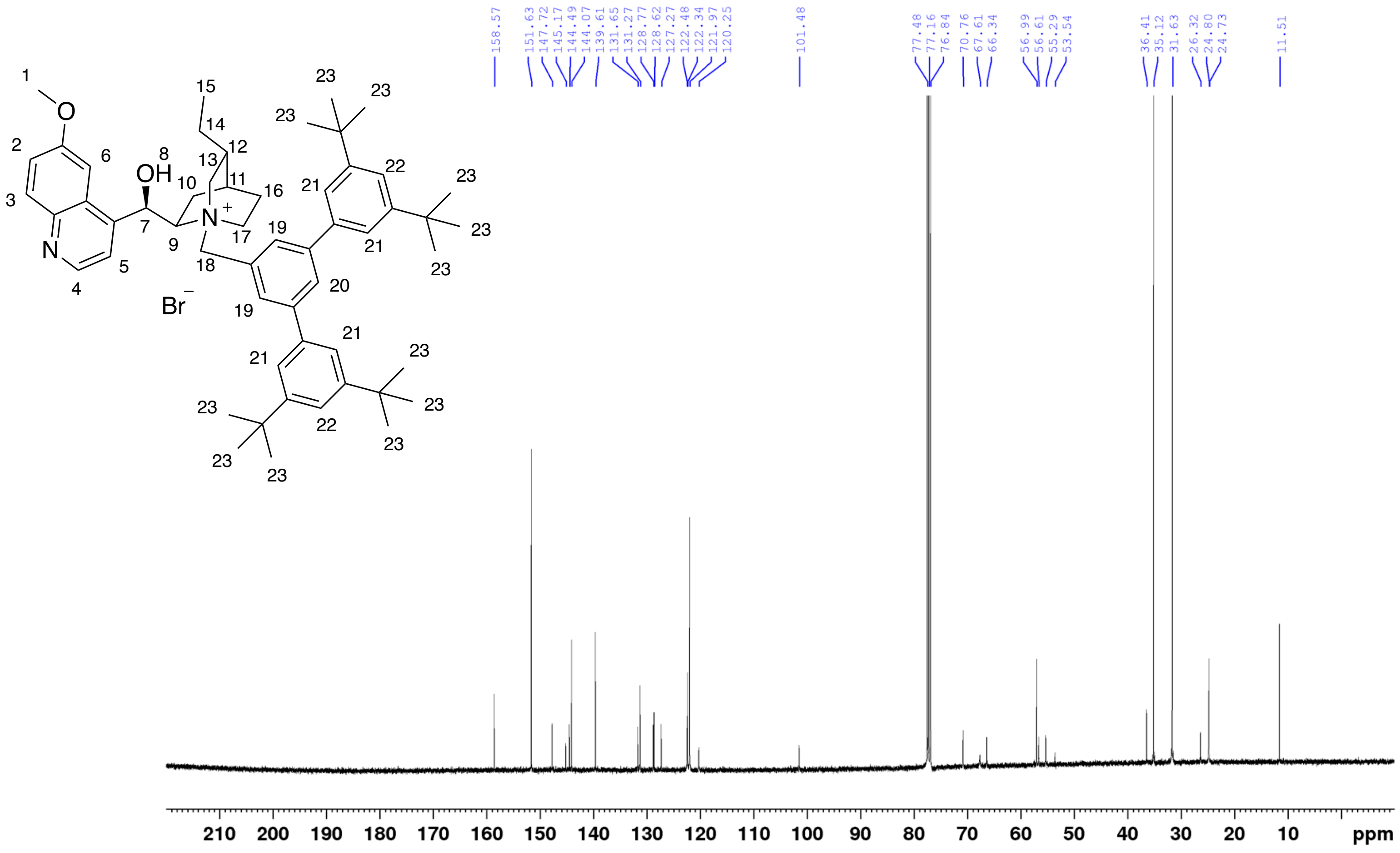

^{1}H NMR (400 MHz, $CDCl_3$) for *(R)-((1S,2S,4S)-5-formylquinuclidin-2-yl)(6-methoxyquinolin-4-yl)methyl acetate*

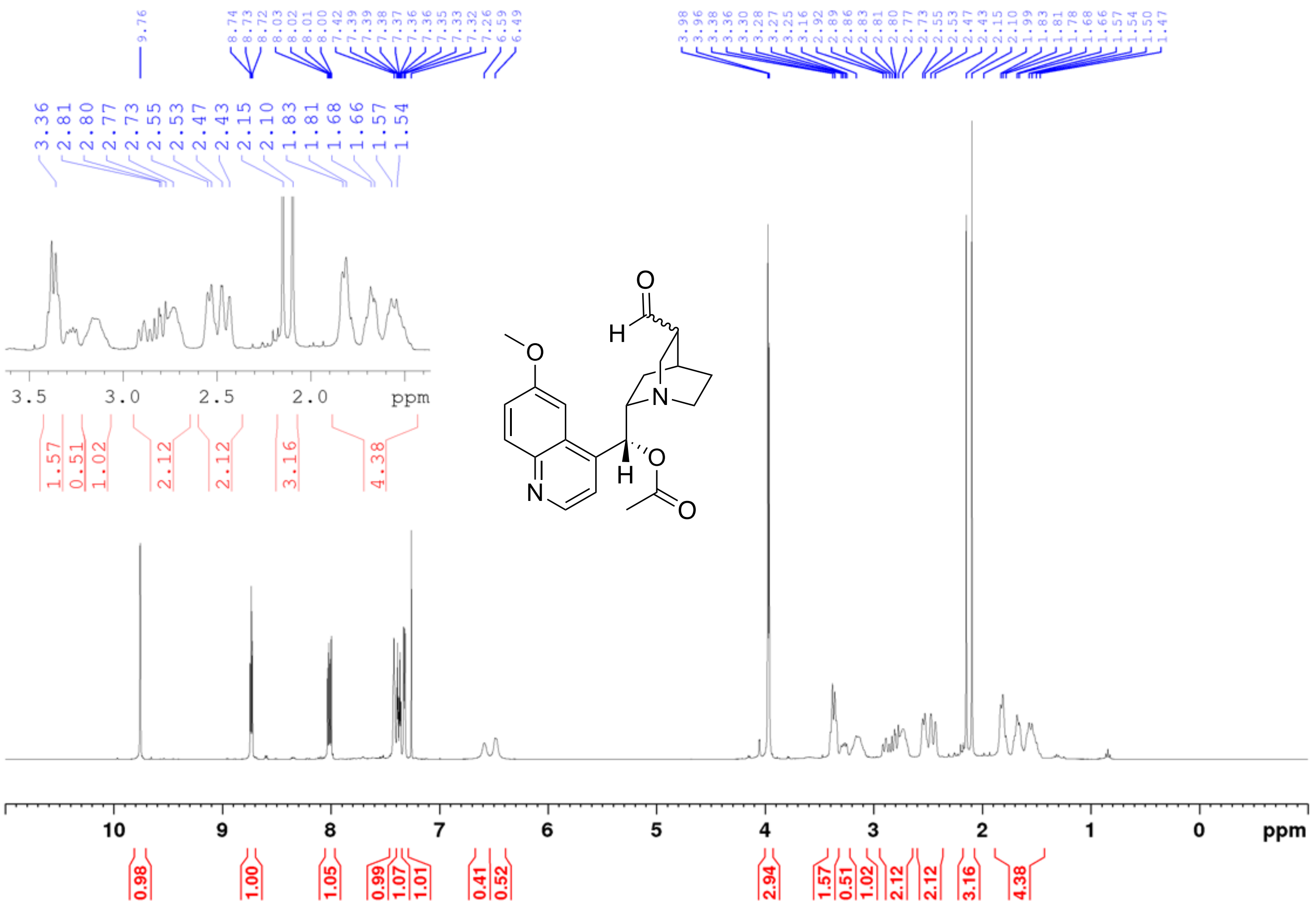

^{13}C NMR (101 MHz, $CDCl_3$) for *(R)-((1S,2S,4S)-5-formylquinuclidin-2-yl)(6-methoxyquinolin-4-yl)methyl acetate*

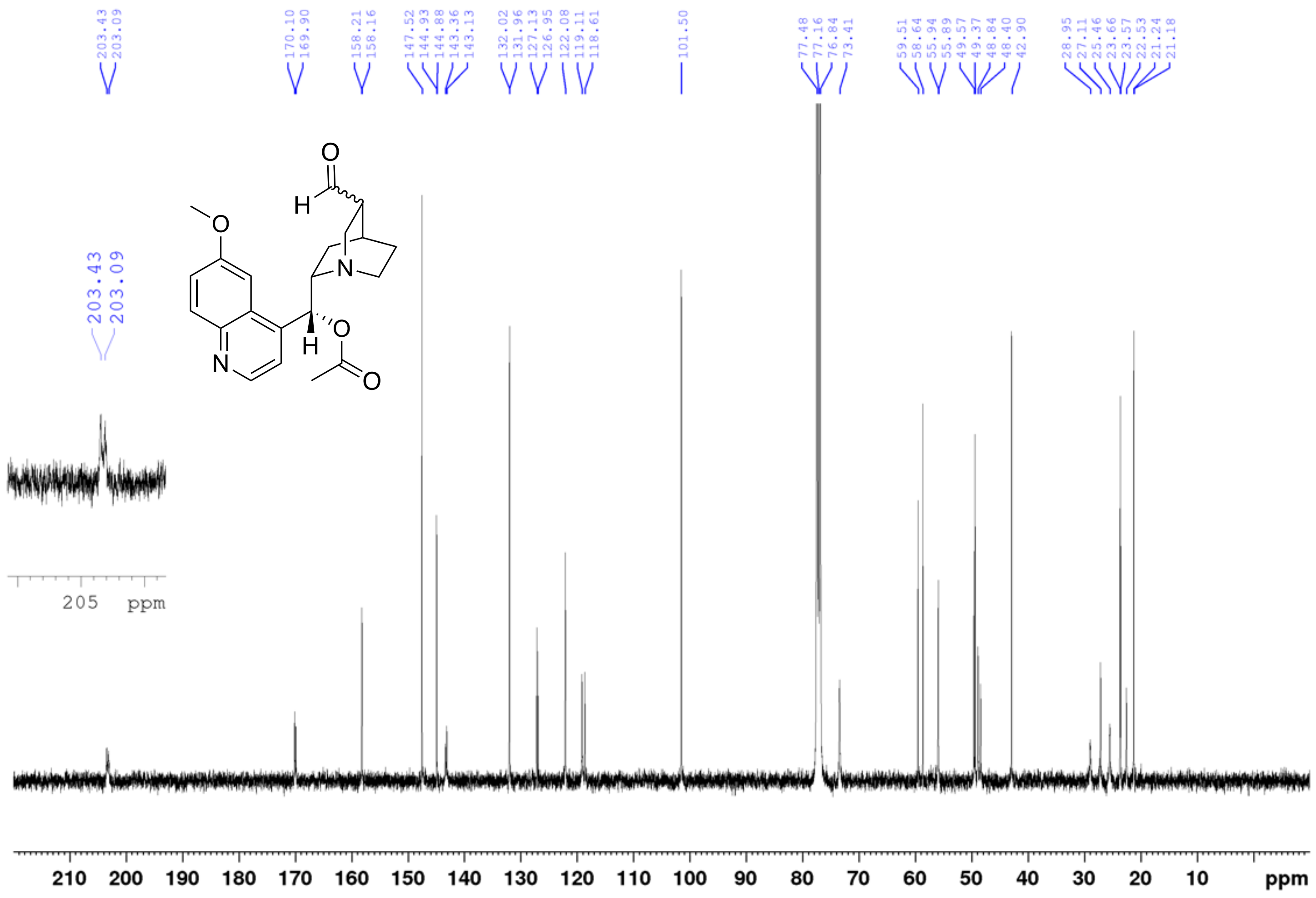

¹H NMR (400 MHz, $CDCl_3$) for *(R)-(6-methoxyquinolin-4-yl)((1R,2S,4R)-quinuclidin-2-yl)methanol (Desvinylquinine)*

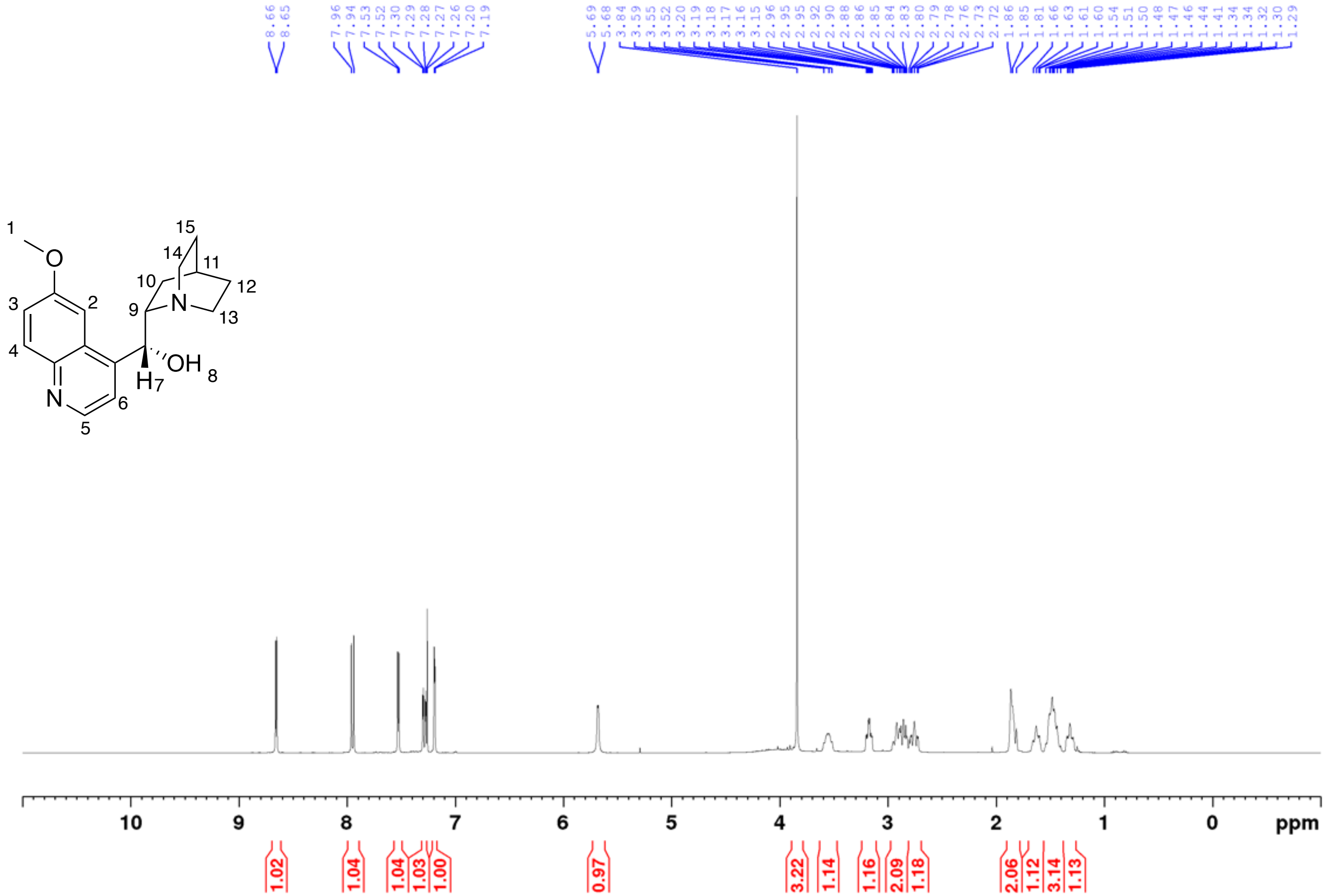

^{13}C NMR (101 MHz, $CDCl_3$) for *(R)-(6-methoxyquinolin-4-yl)((1R,2S,4R)-quinuclidin-2-yl)methanol (Desvinylquinine)*

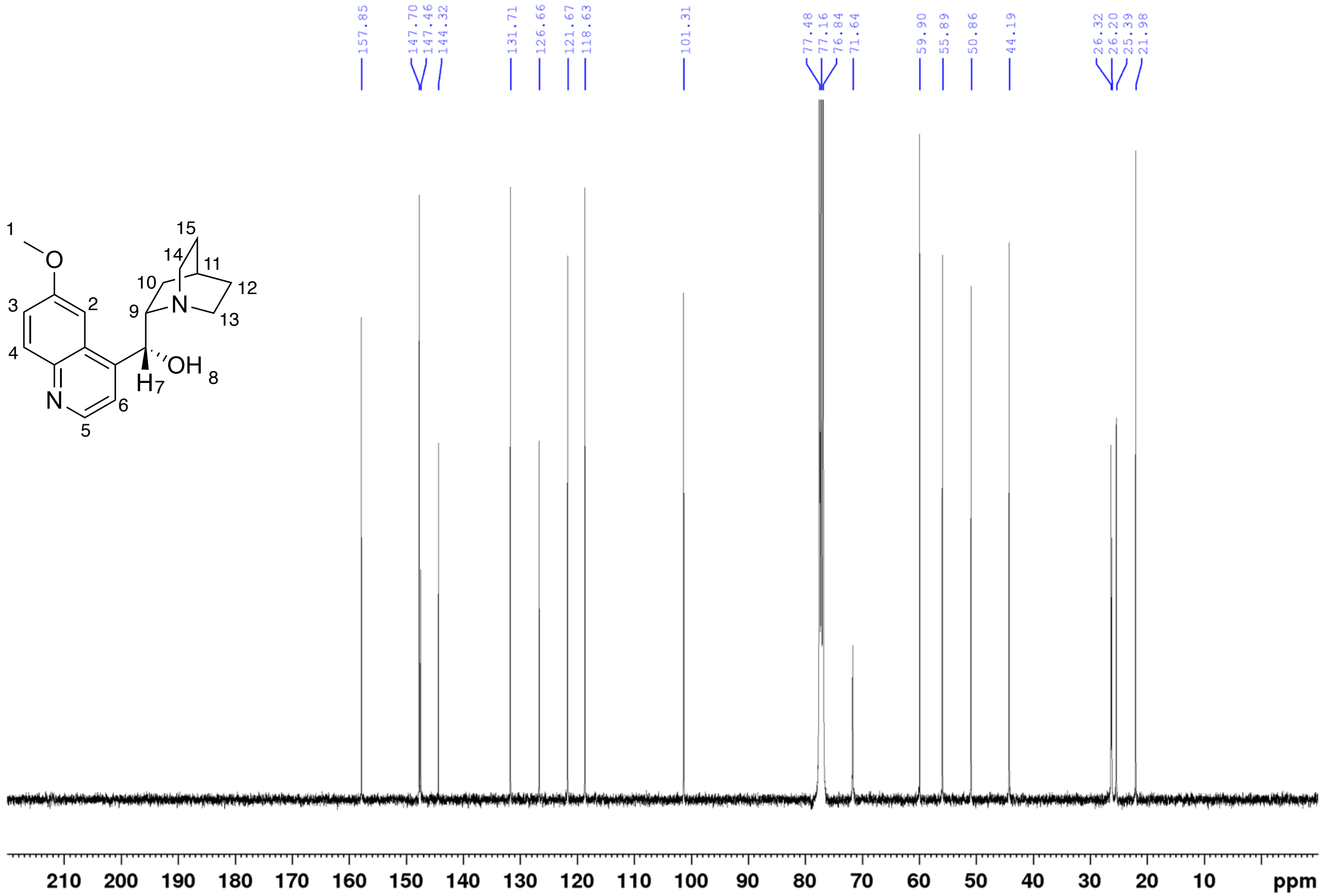

¹H NMR (400 MHz, $CDCl_3$) for *(1R,2S,4R)-2-((R)-hydroxy(6-methoxyquinolin-4-yl)methyl)-1-((3,3'',5,5''-tetra-tert-butyl-[1,1':3',1''-terphenyl]-5'-yl)methyl)quinuclidin-1-ium bromide* (**10•Br**)

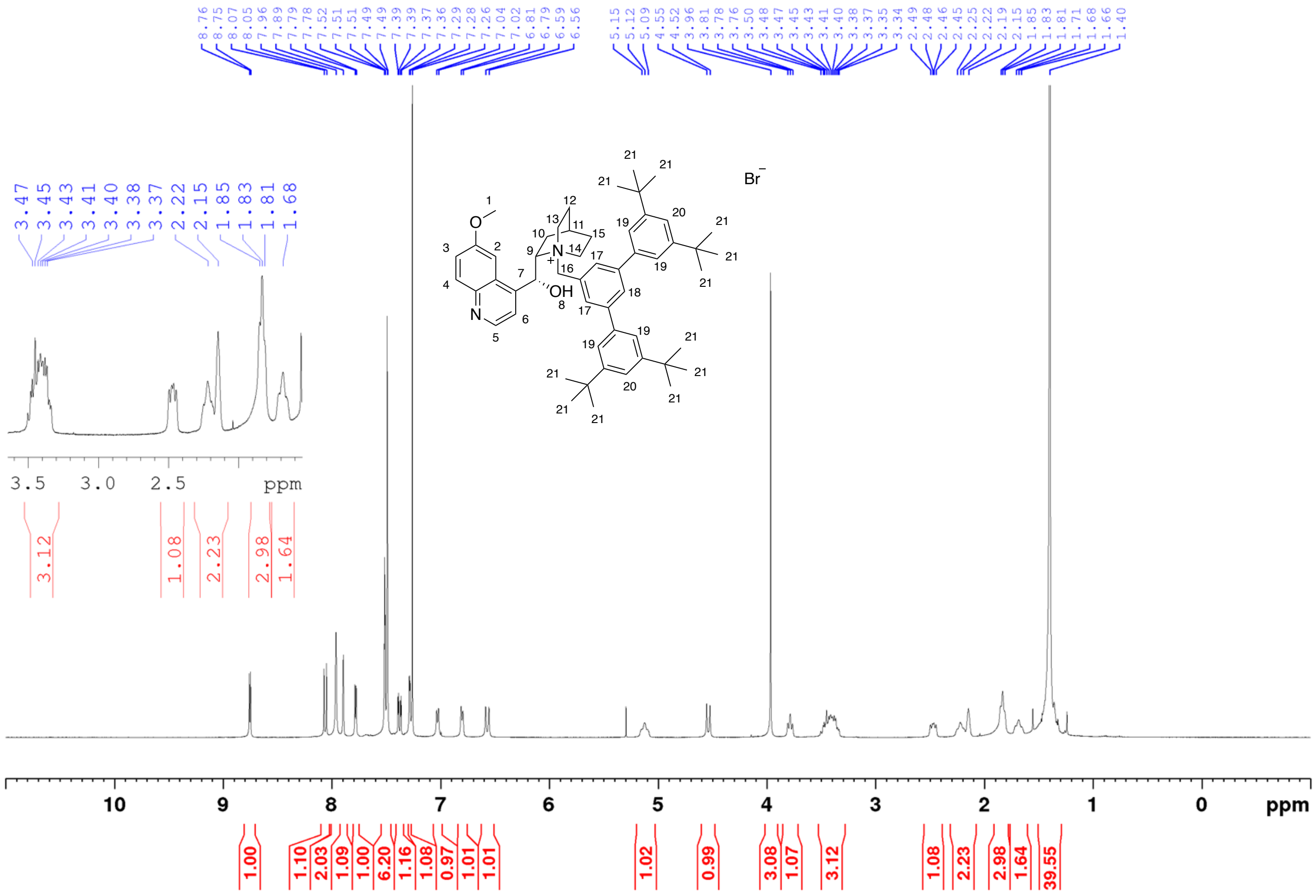

^{13}C NMR (126 MHz, $CDCl_3$) for *(1R,2S,4R)-2-((R)-hydroxy(6-methoxyquinolin-4-yl)methyl)-1-((3,3'',5,5''-tetra-tert-butyl-[1,1':3',1''-terphenyl]-5'-yl)methyl)quinuclidin-1-ium bromide* (**10•Br**)

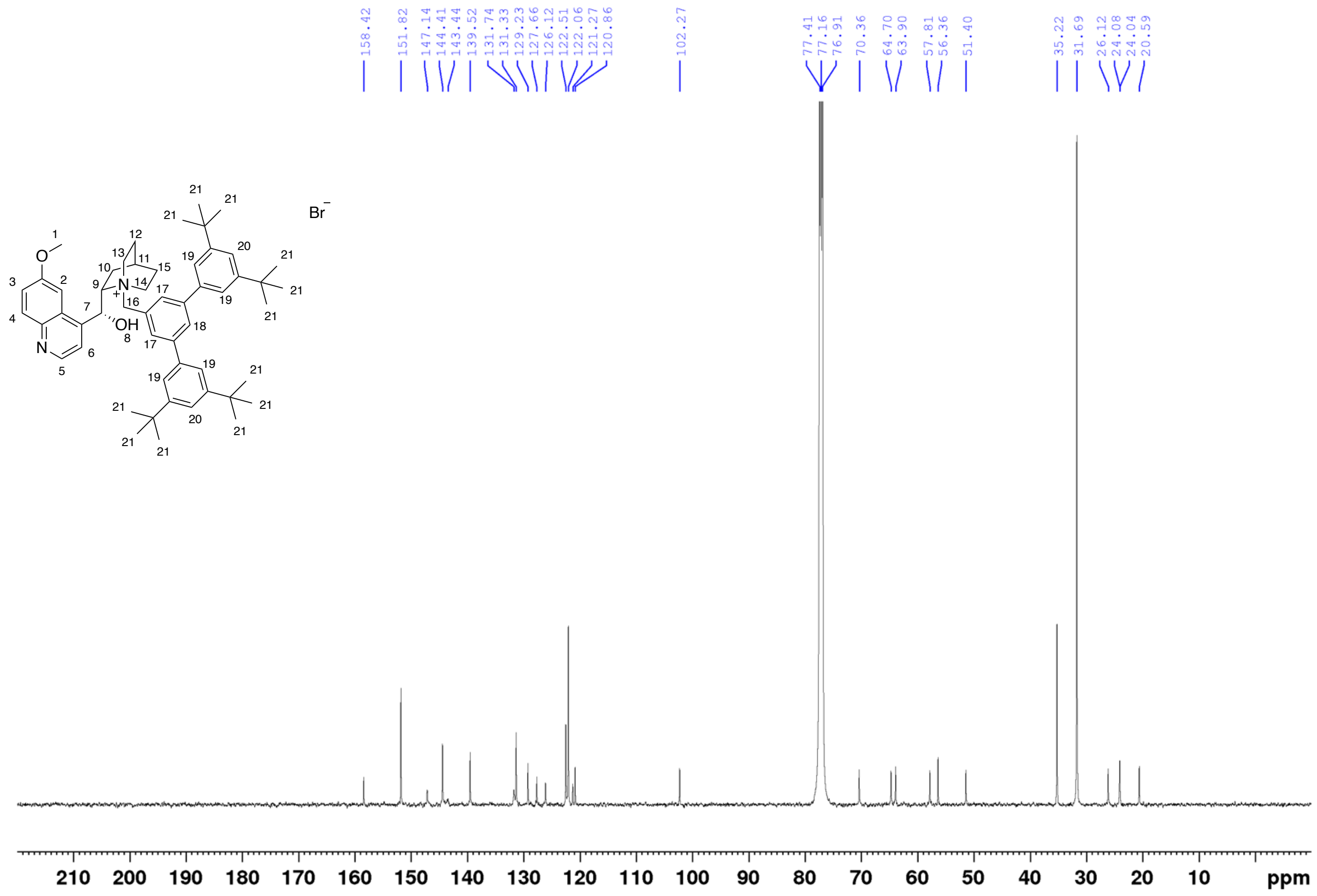

^{1}H NMR (500 MHz, DMSO-d$_6$) for *Bis[rhodium (1S,2S,4S,5R)-5-ethyl-2-((R)-hydroxy(6-methoxyquinolin-4-yl)methyl)-1-((3,3'',5,5''-tetra-tert-butyl-[1,1':3',1''-terphenyl]-5'-yl)methyl)quinuclidin-1-ium (3,5-bis(2-carboxy-2-methylpropyl)benzenesulfonate)]* (Rh$_2$(**A**)$_2$•(**1**)$_2$)

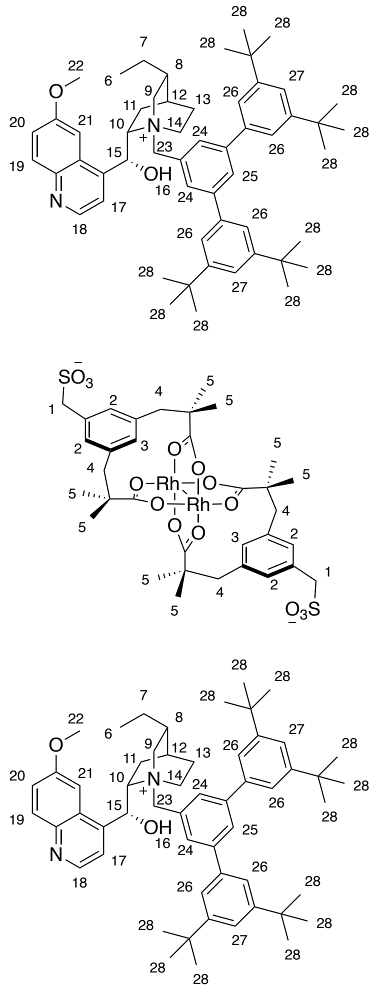

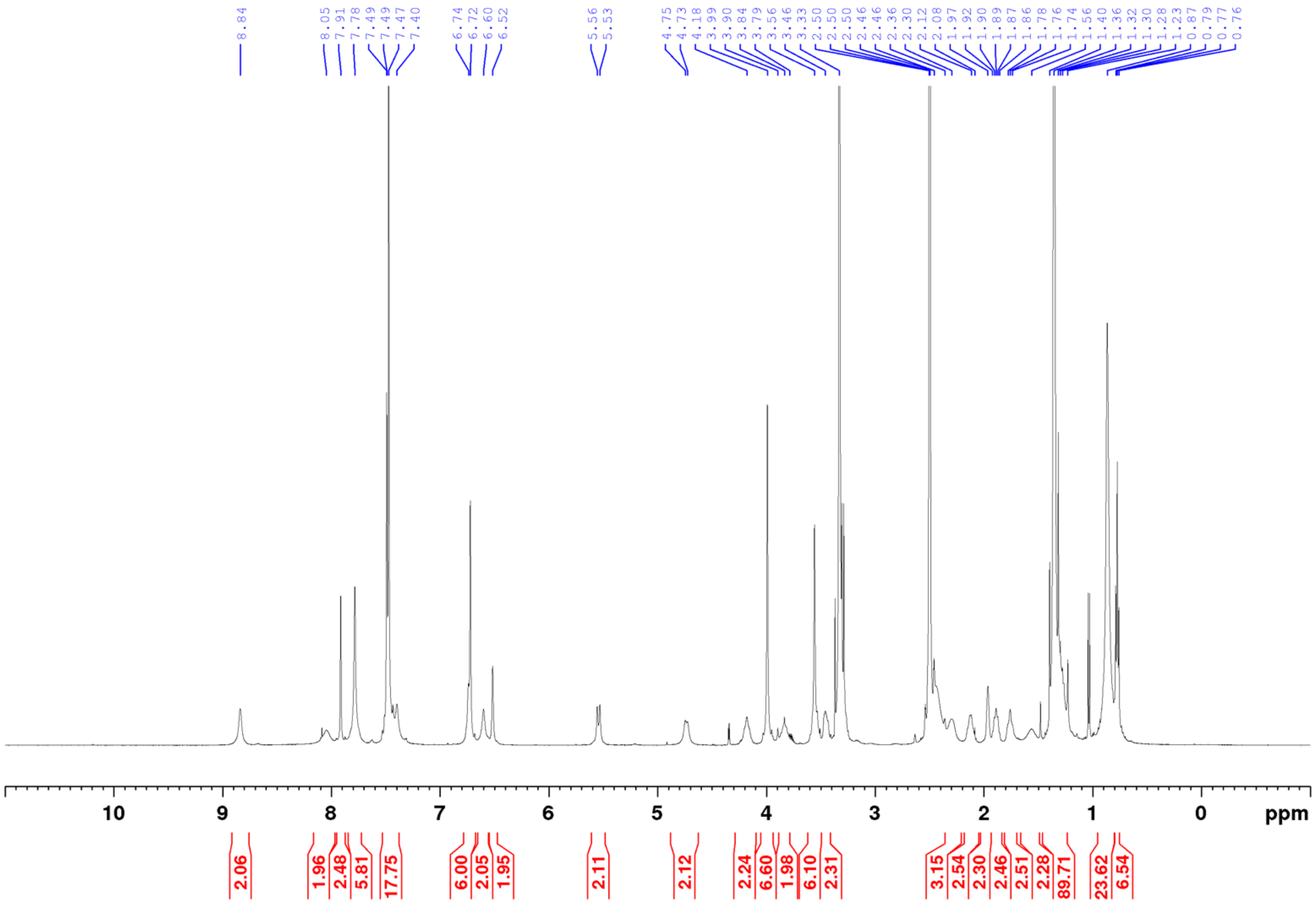

^{13}C NMR (126 MHz, DMSO-d$_6$) for *Bis[rhodium (1S,2S,4S,5R)-5-ethyl-2-((R)-hydroxy(6-methoxyquinolin-4-yl)methyl)-1-((3,3'',5,5''-tetra-tert-butyl-[1,1':3',1''-terphenyl]-5'-yl)methyl)quinuclidin-1-ium (3,5-bis(2-carboxy-2-methylpropyl)benzenesulfonate)]* (Rh$_2$(**A**)$_2$•(**1**)$_2$)

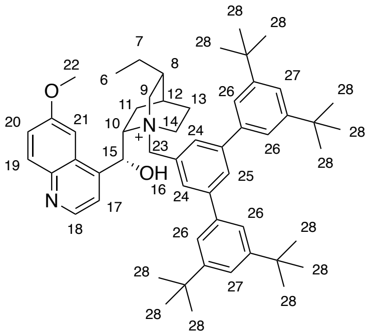

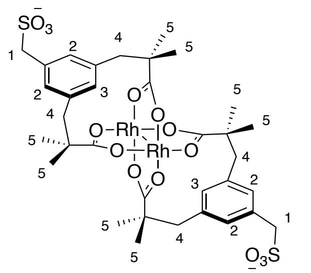

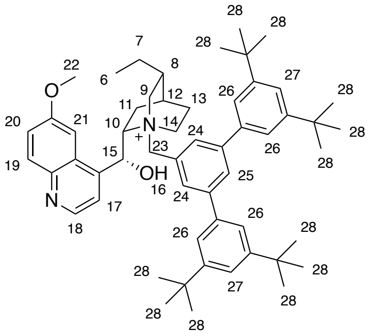

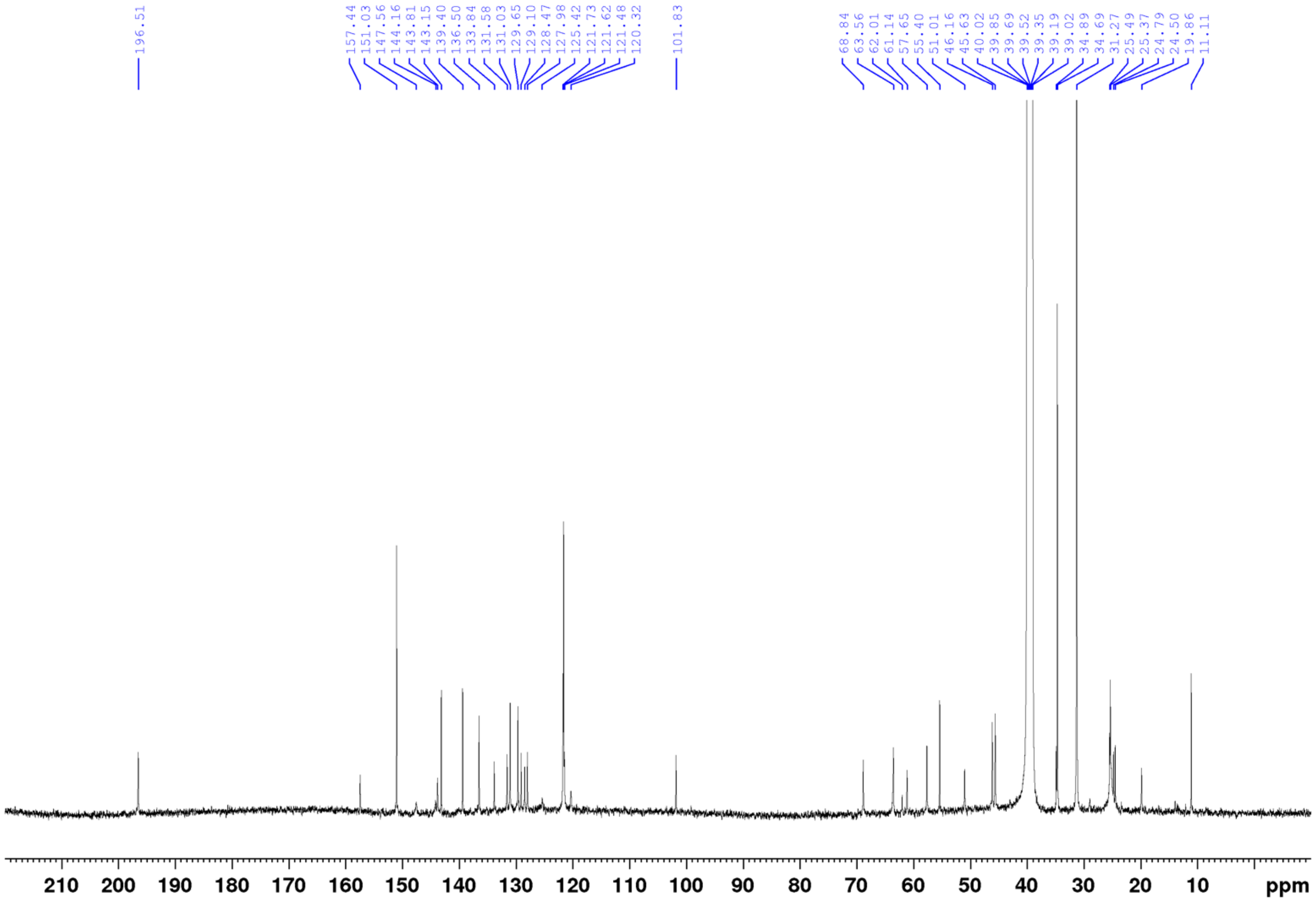

^{1}H NMR (500 MHz, C_5D_5N) for *Bis[rhodium (1S,2R,4S,5R)-5-ethyl-2-((S)-hydroxy(6-methoxyquinolin-4-yl)methyl)-1-((3,3'',5,5''-tetra-tert-butyl-[1,1':3',1''-terphenyl]-5'-yl)methyl)quinuclidin-1-ium (3,5-bis(2-carboxy-2-methylpropyl)benzenesulfonate)]* (Rh_2(**A**)$_2$•(**2a**)$_2$)

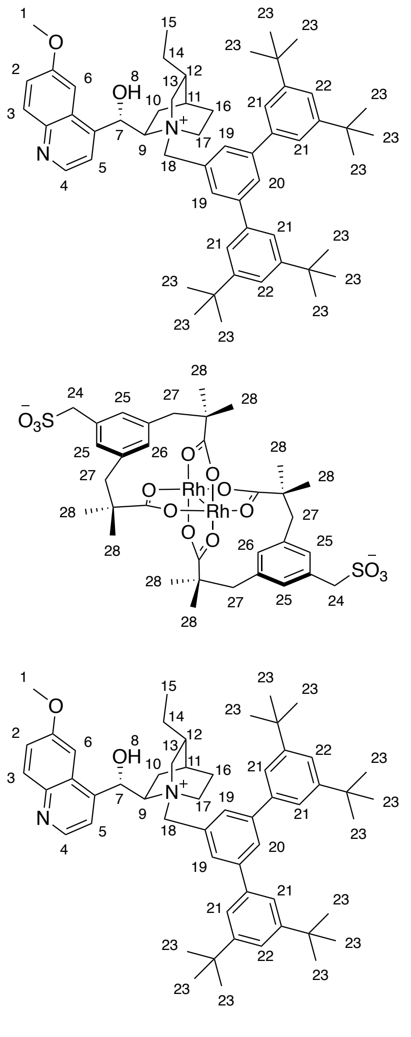

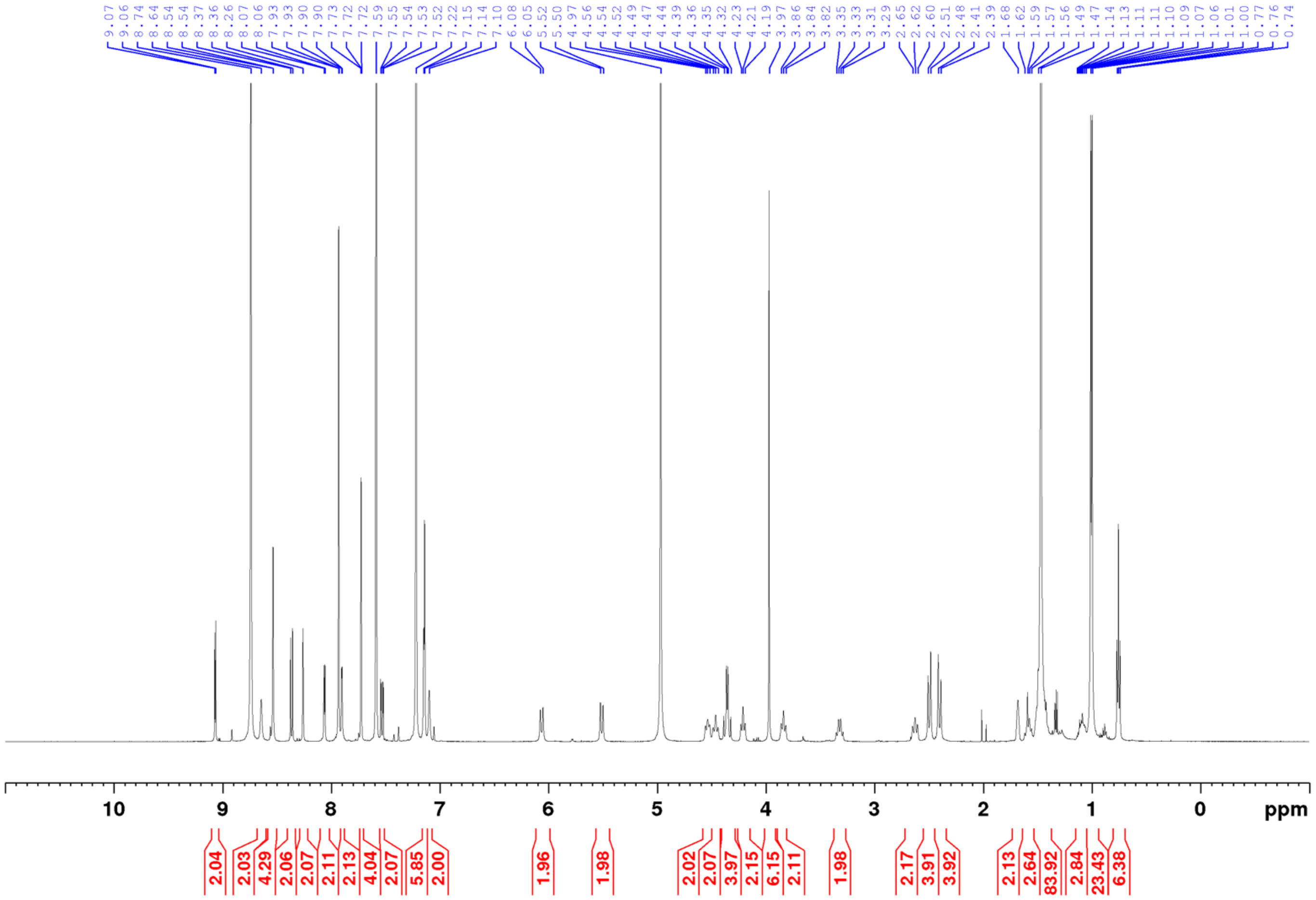

^{13}C NMR (126 MHz, C_5D_5N) for *Bis[rhodium (1S,2R,4S,5R)-5-ethyl-2-((S)-hydroxy(6-methoxyquinolin-4-yl)methyl)-1-((3,3'',5,5''-tetra-tert-butyl-[1,1':3',1''-terphenyl]-5'-yl)methyl)quinuclidin-1-ium (3,5-bis(2-carboxy-2-methylpropyl)benzenesulfonate)]* (Rh_2(**A**)$_2$•(**2a**)$_2$)

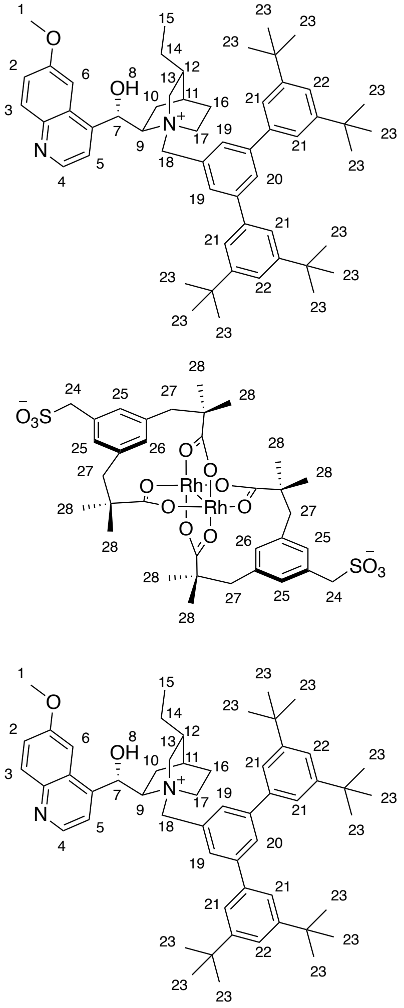

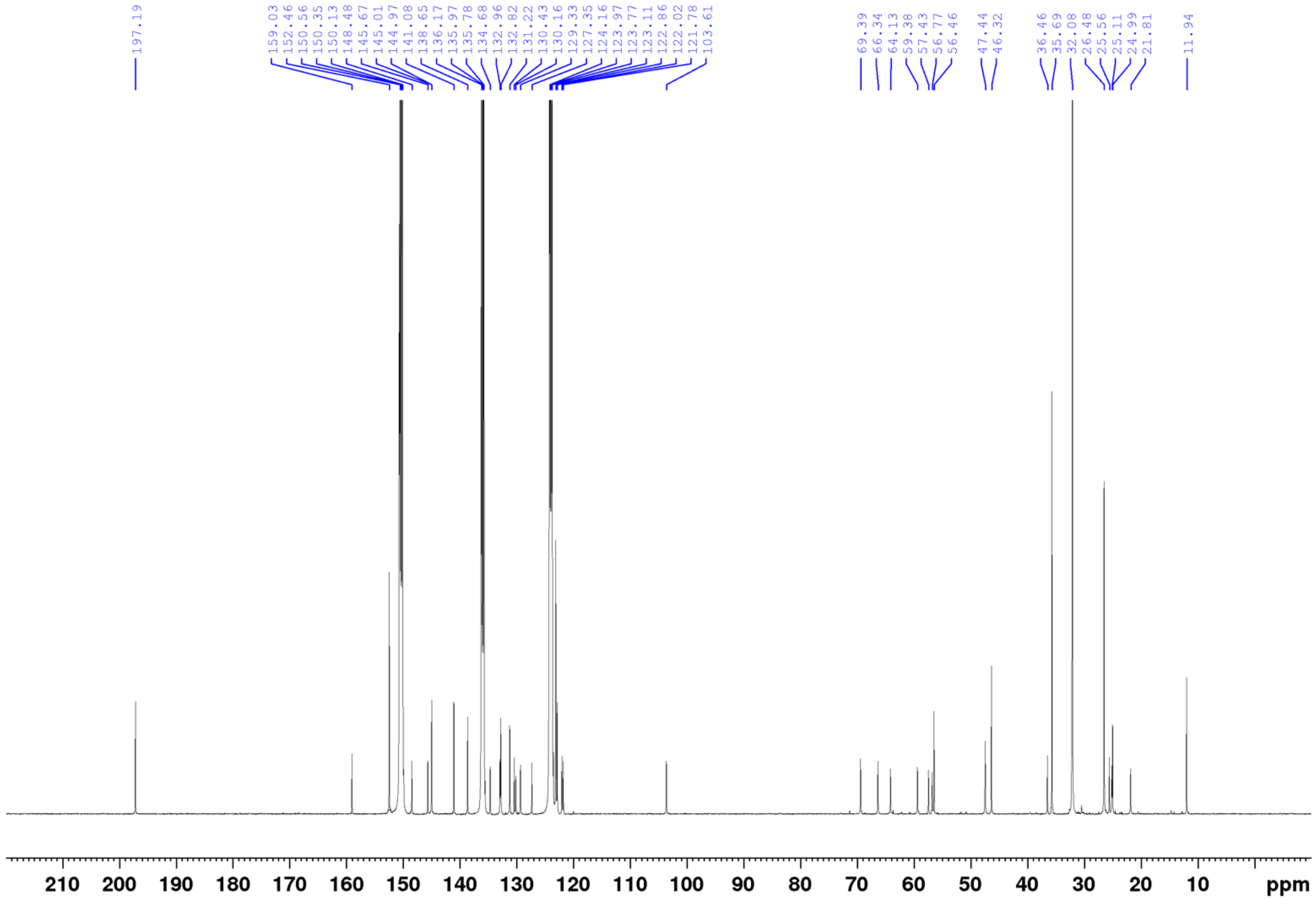

^{1}H NMR (500 MHz, C_5D_5N) for *Bis[rhodium (1S,2R,4S,5R)-5-ethyl-2-((S)-hydroxy(6-methoxyquinolin-4-yl)methyl)-1-((3,3'',5,5''-tetra-tert-butyl-[1,1':3',1''-terphenyl]-5'-yl)methyl)quinuclidin-1-ium (3,5-bis(2-carboxy-2-methylpropyl)benzenesulfonate)]* (Rh_2(**A**)$_2$•(**3**)$_2$)

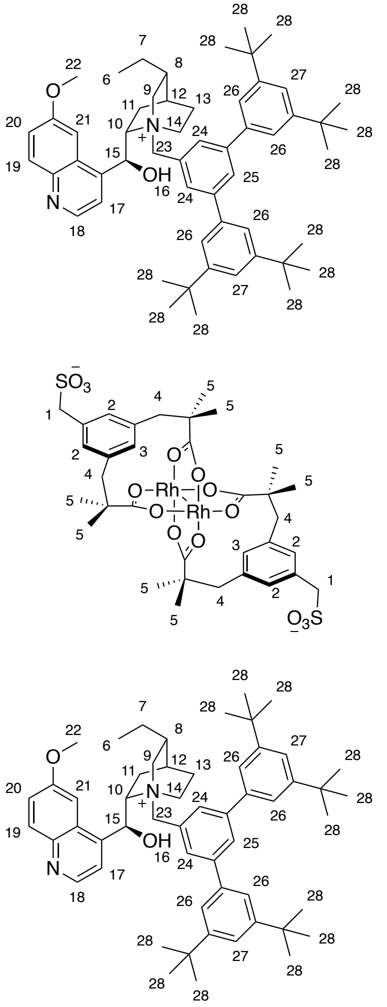

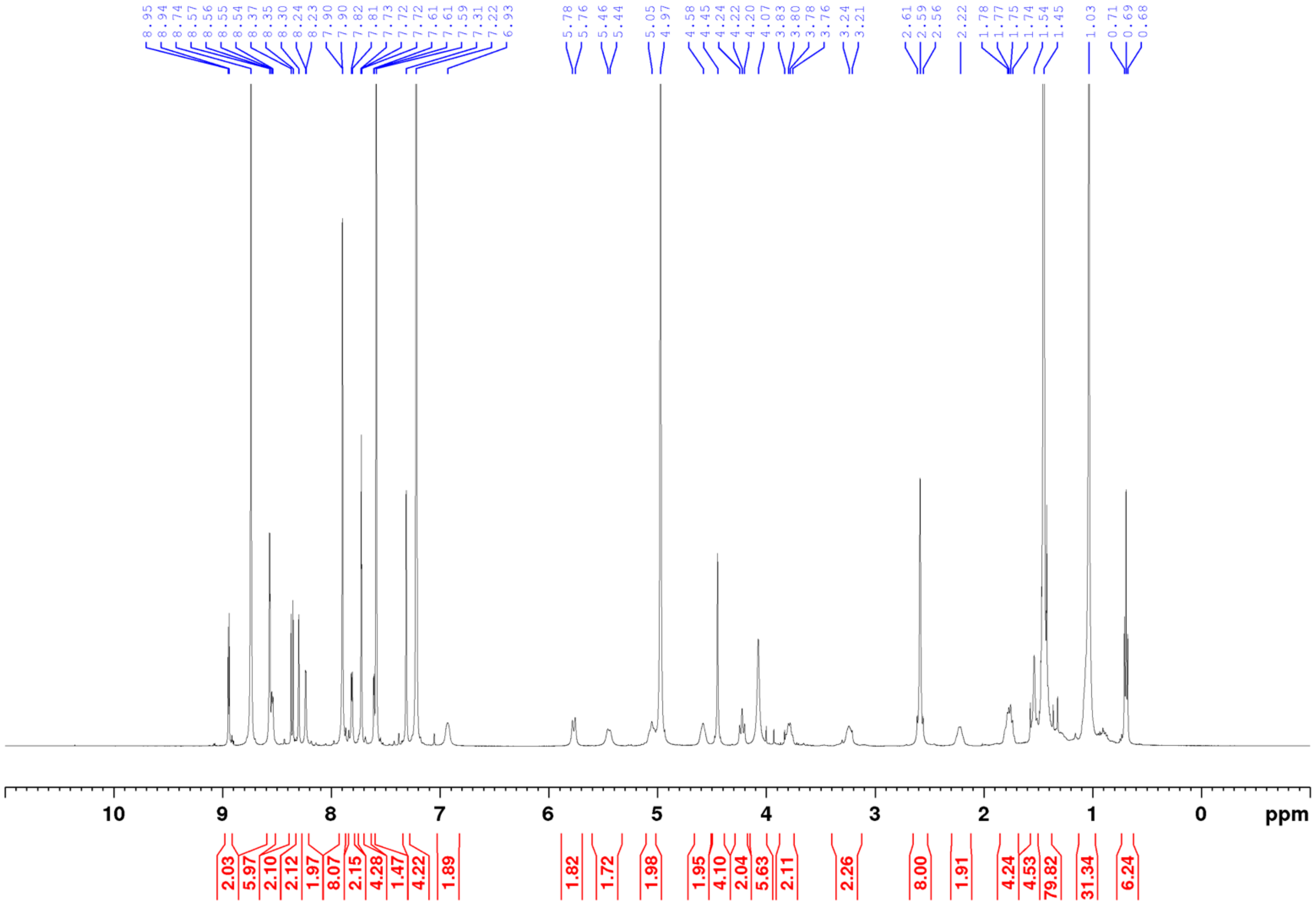

^{13}C NMR (126 MHz, C_5D_5N) for *Bis[rhodium (1S,2R,4S,5R)-5-ethyl-2-((S)-hydroxy(6-methoxyquinolin-4-yl)methyl)-1-((3,3'',5,5''-tetra-tert-butyl-[1,1':3',1''-terphenyl]-5'-yl)methyl)quinuclidin-1-ium (3,5-bis(2-carboxy-2-methylpropyl)benzenesulfonate)]* (Rh_2(**A**)$_2$•(**3**)$_2$)

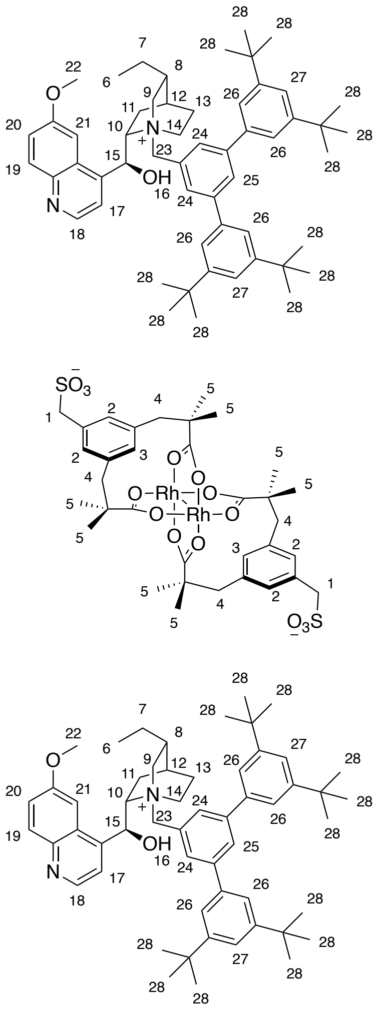

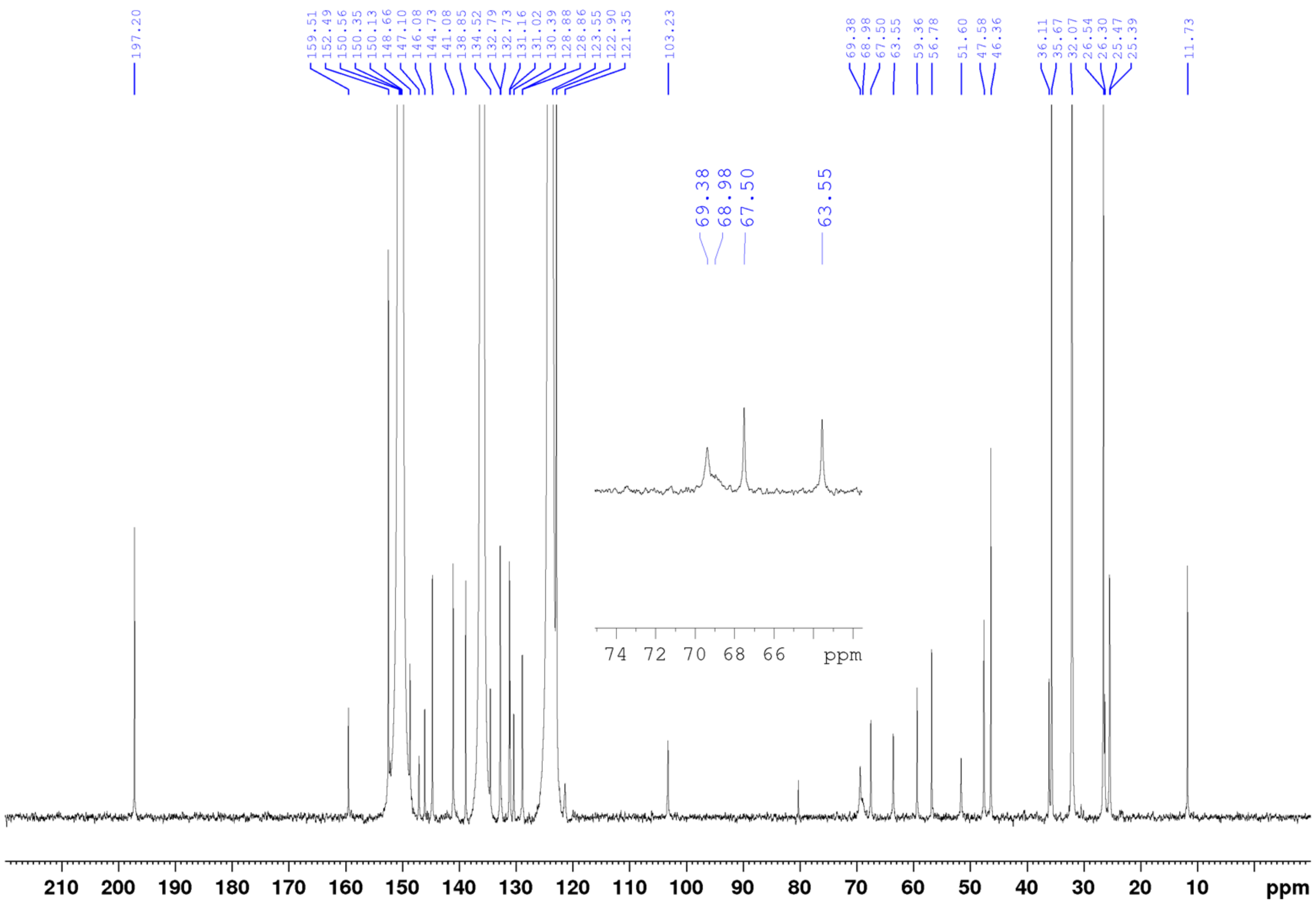

^{1}H NMR (500 MHz, C_5D_5N) for *Bis[rhodium (1S,2R,4S,5R)-5-ethyl-2-((R)-hydroxy(6-methoxyquinolin-4-yl)methyl)-1-((3,3'',5,5''-tetra-tert-butyl-[1,1':3',1''-terphenyl]-5'-yl)methyl)quinuclidin-1-ium (3,5-bis(2-carboxy-2-methylpropyl)benzenesulfonate)]* (Rh_2(**A**)$_2$•(**4**)$_2$)

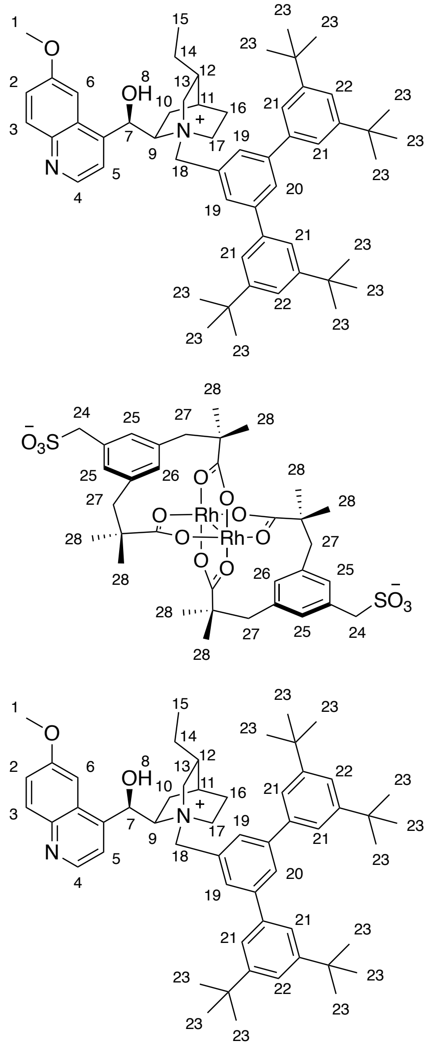

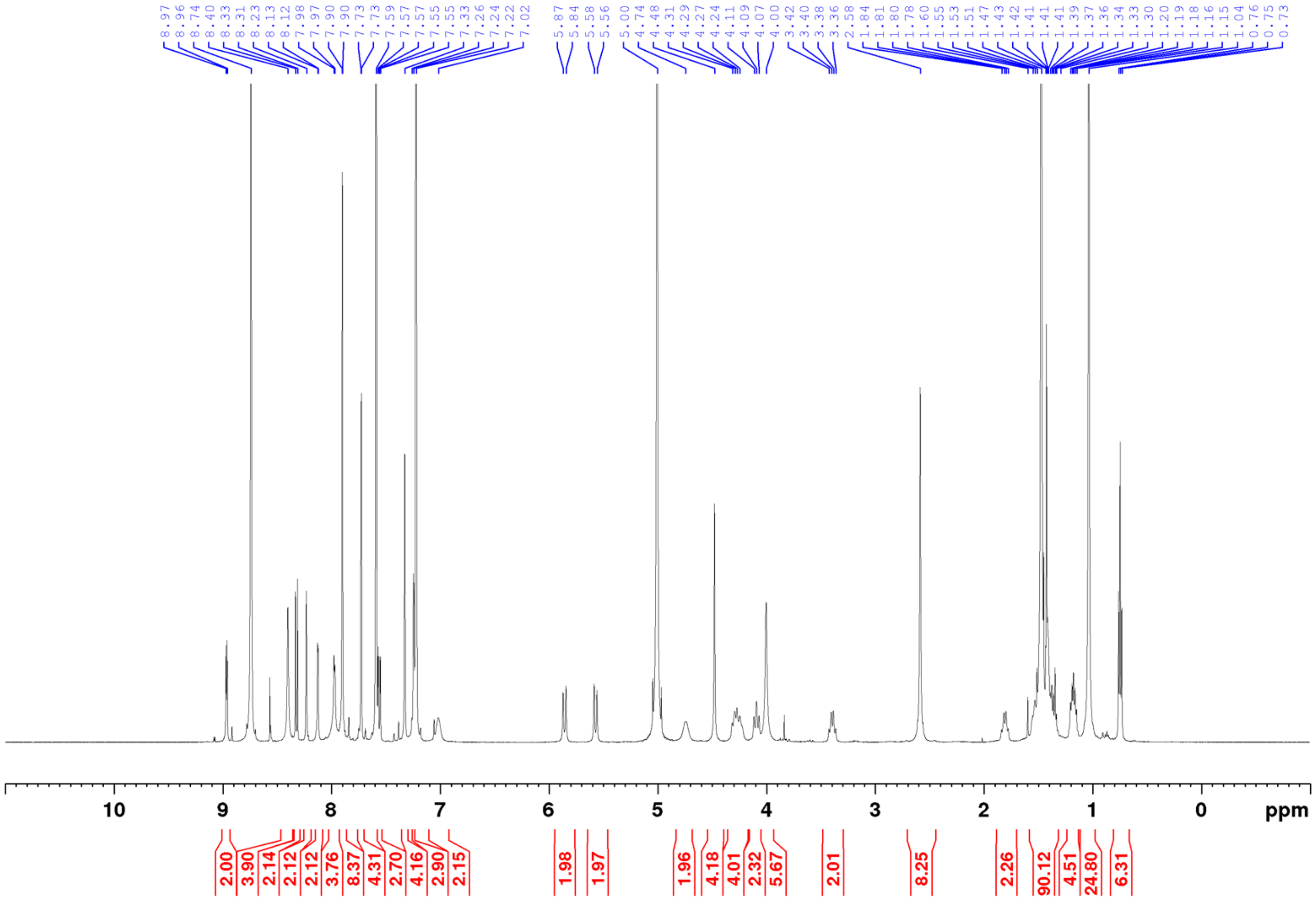

^{13}C NMR (126 MHz, C_5D_5N) for *Bis[rhodium (1S,2R,4S,5R)-5-ethyl-2-((R)-hydroxy(6-methoxyquinolin-4-yl)methyl)-1-((3,3'',5,5''-tetra-tert-butyl-[1,1':3',1''-terphenyl]-5'-yl)methyl)quinuclidin-1-ium (3,5-bis(2-carboxy-2-methylpropyl)benzenesulfonate)]* (Rh_2(**A**)$_2$•(**4**)$_2$)

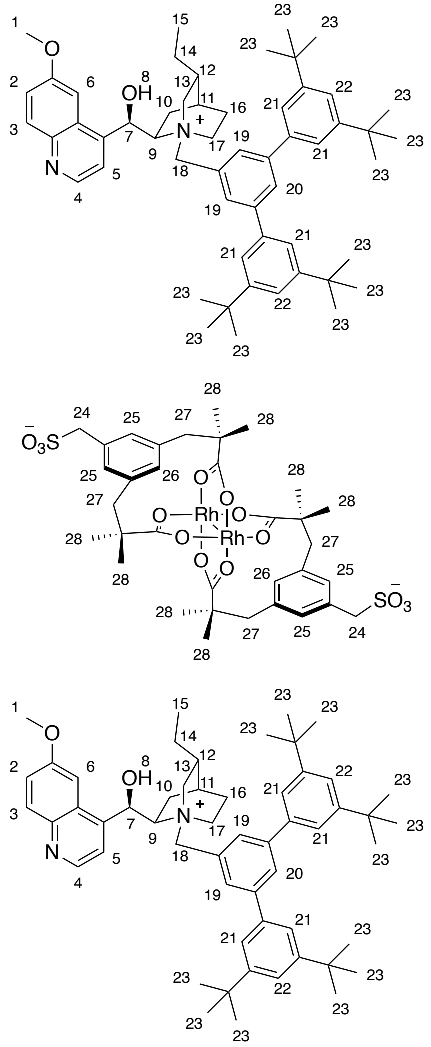

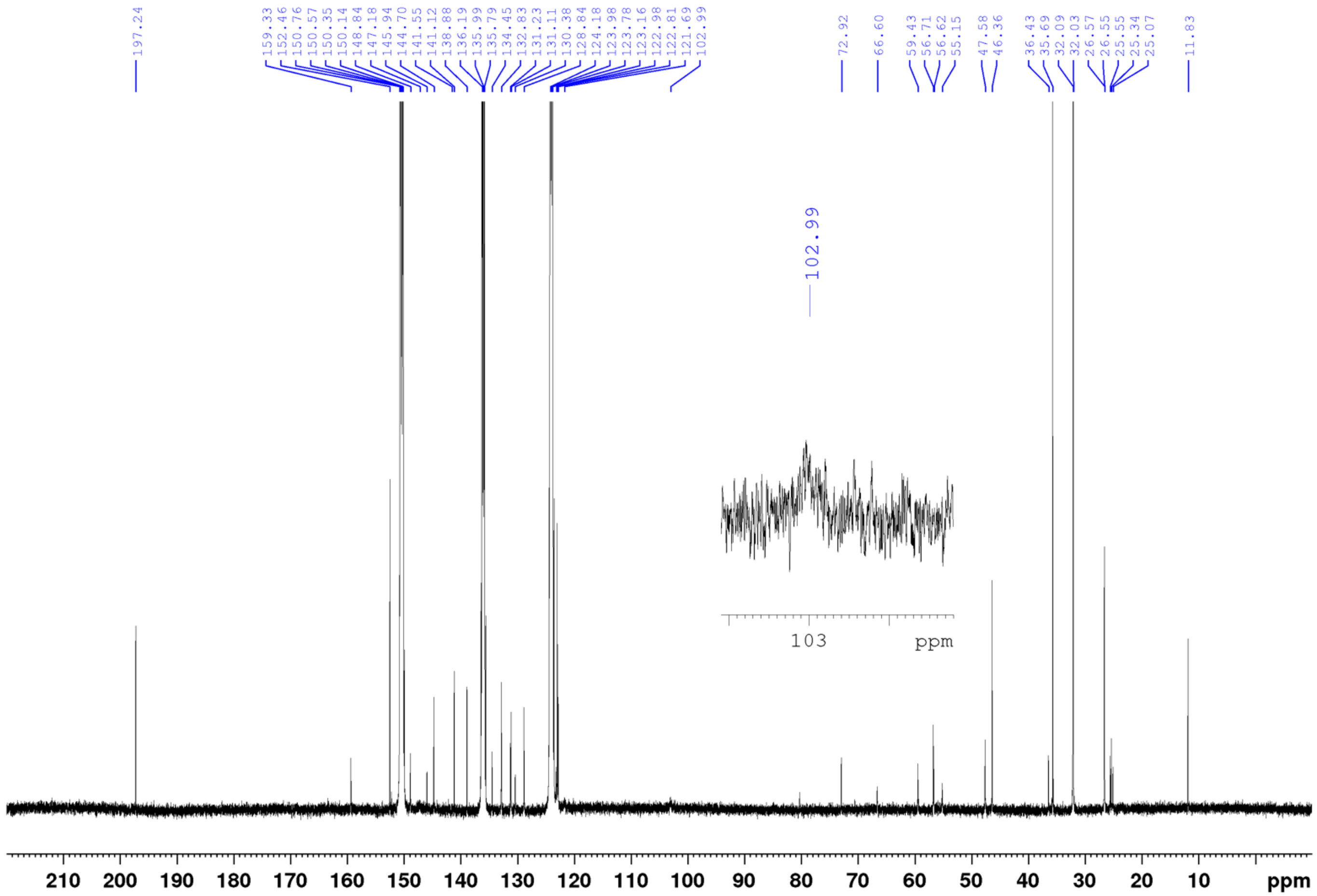

^{1}H NMR (500 MHz, C_5D_5N) for *Bis[rhodium (1S,2R,4S,5R)-5-ethyl-2-((S)-hydroxy(6-methoxyquinolin-4-yl)methyl)-1-((3,3'',5,5''-tetra-tert-butyl-[1,1':3',1''-terphenyl]-5'-yl)methyl)quinuclidin-1-ium (3,5-bis((1-carboxycyclobutyl)methyl)phenyl)methanesulfonate]* (Rh_2(**B**)$_2$•(**2a**)$_2$)

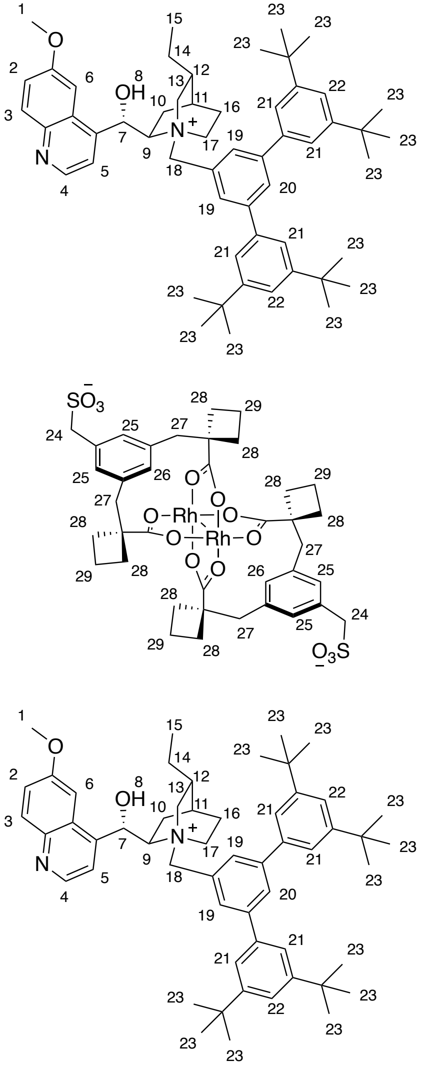

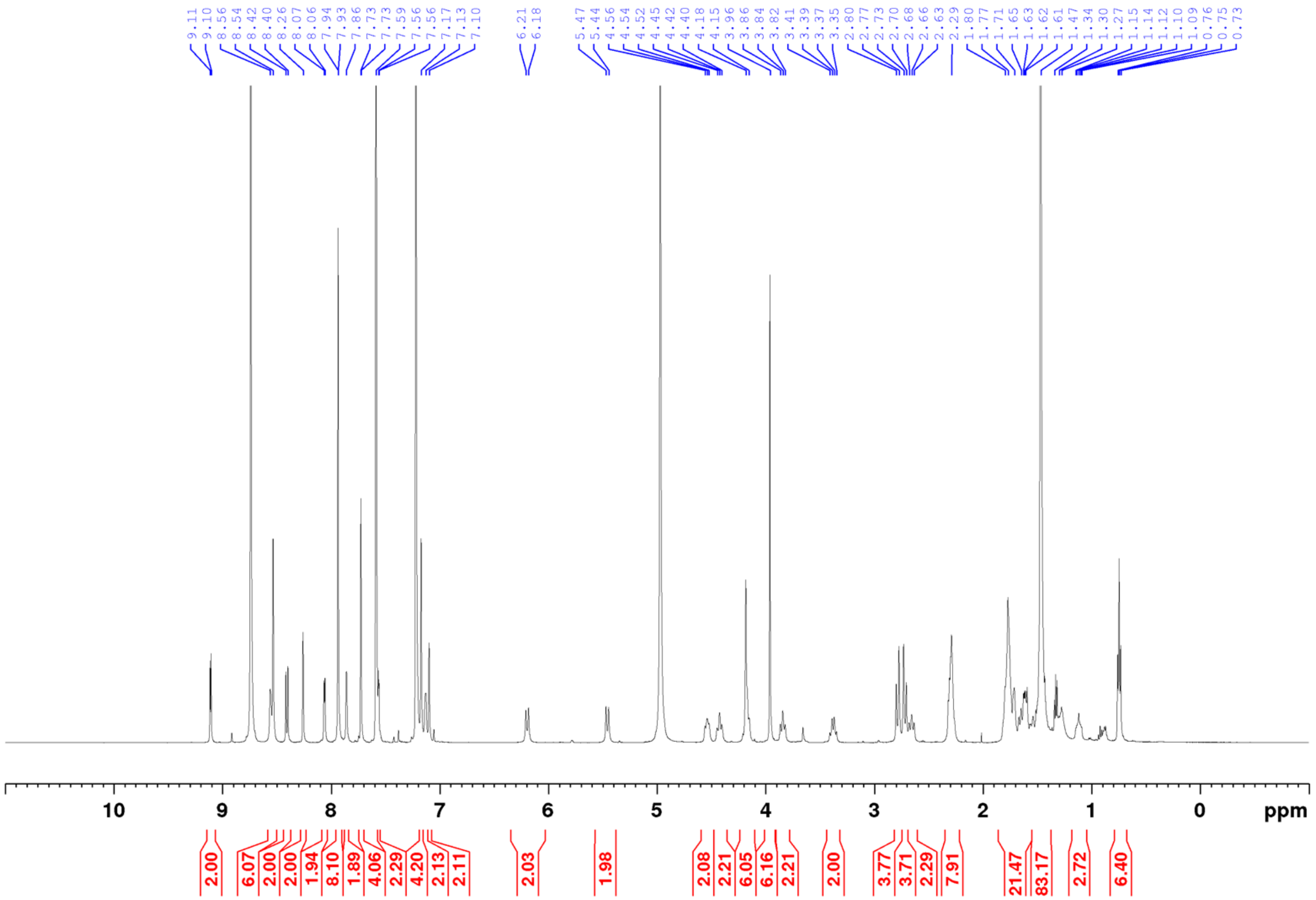

^{13}C NMR (126 MHz, C_5D_5N) for *Bis[rhodium (1S,2R,4S,5R)-5-ethyl-2-((S)-hydroxy(6-methoxyquinolin-4-yl)methyl)-1-((3,3'',5,5''-tetra-tert-butyl-[1,1':3',1''-terphenyl]-5'-yl)methyl)quinuclidin-1-ium (3,5-bis((1-carboxycyclobutyl)methyl)phenyl)methanesulfonate]* (Rh_2(**B**)$_2$•(**2a**)$_2$)

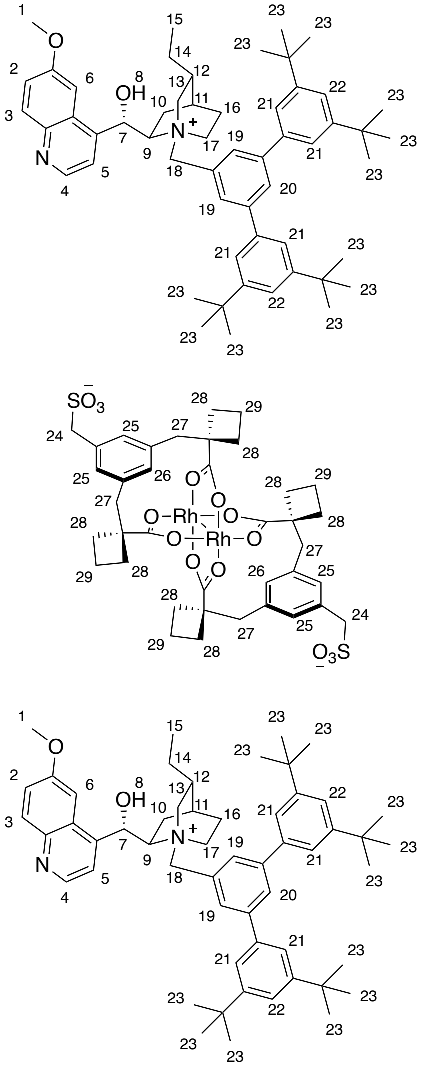

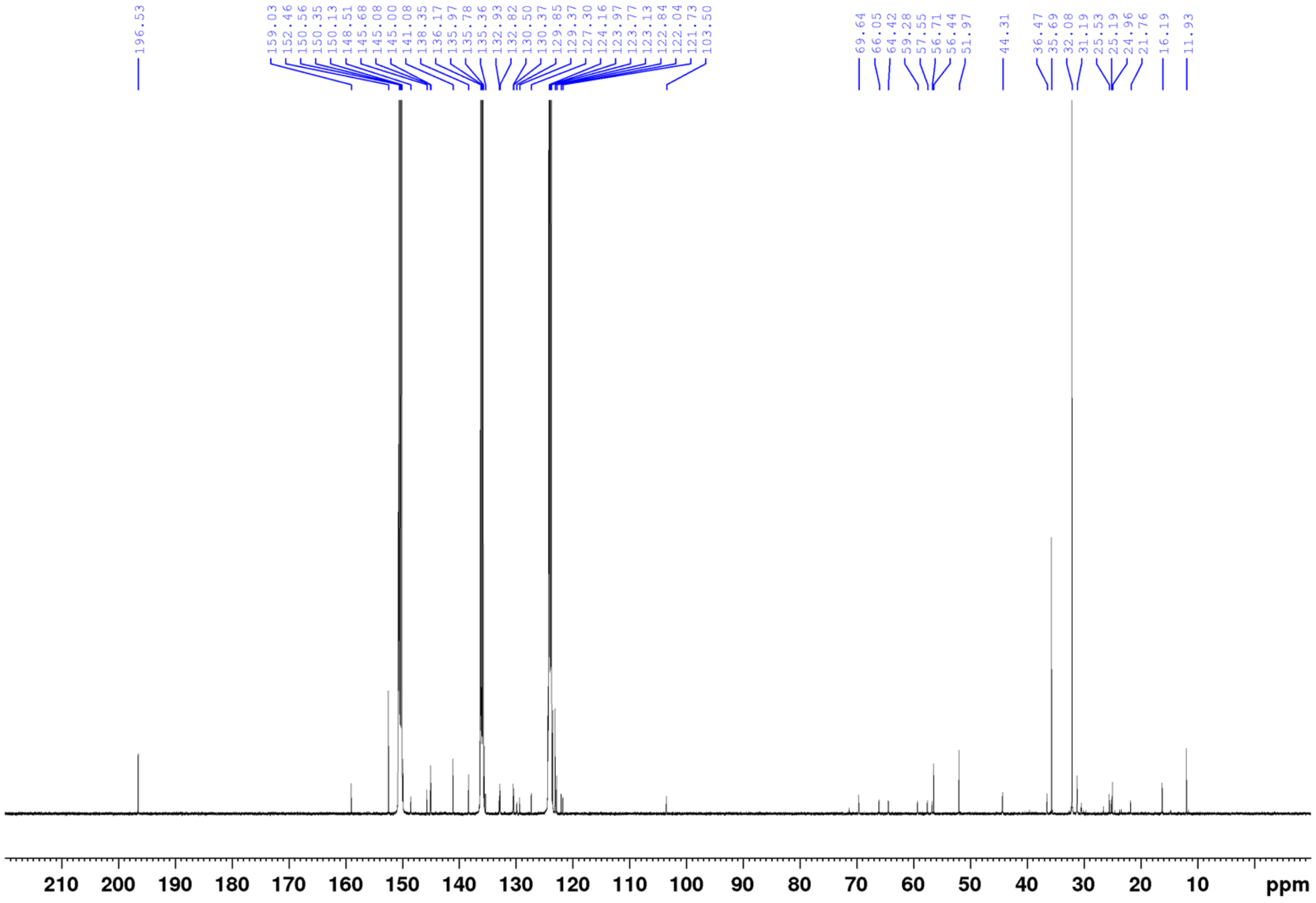

^{1}H NMR (500 MHz, C_5D_5N) for *Bis[rhodium (1S,2R,4S,5R)-5-ethyl-2-((S)-hydroxy(6-methoxyquinolin-4-yl)methyl)-1-((3,3'',5,5''-tetra-tert-butyl-[1,1':3',1''-terphenyl]-5'-yl)methyl)quinuclidin-1-ium (3,5-bis((1-carboxycyclopentyll)methyl)phenyl)methanesulfonate)]* (Rh_2(**C**)$_2$•(**2a**)$_2$)

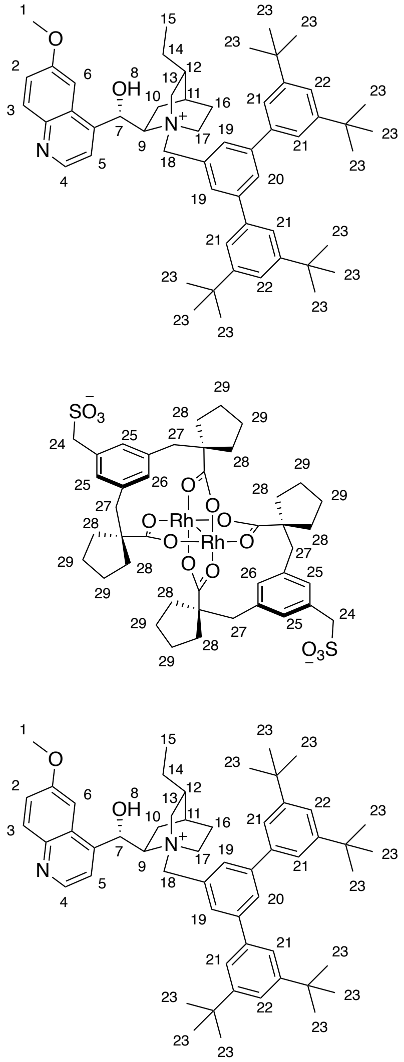

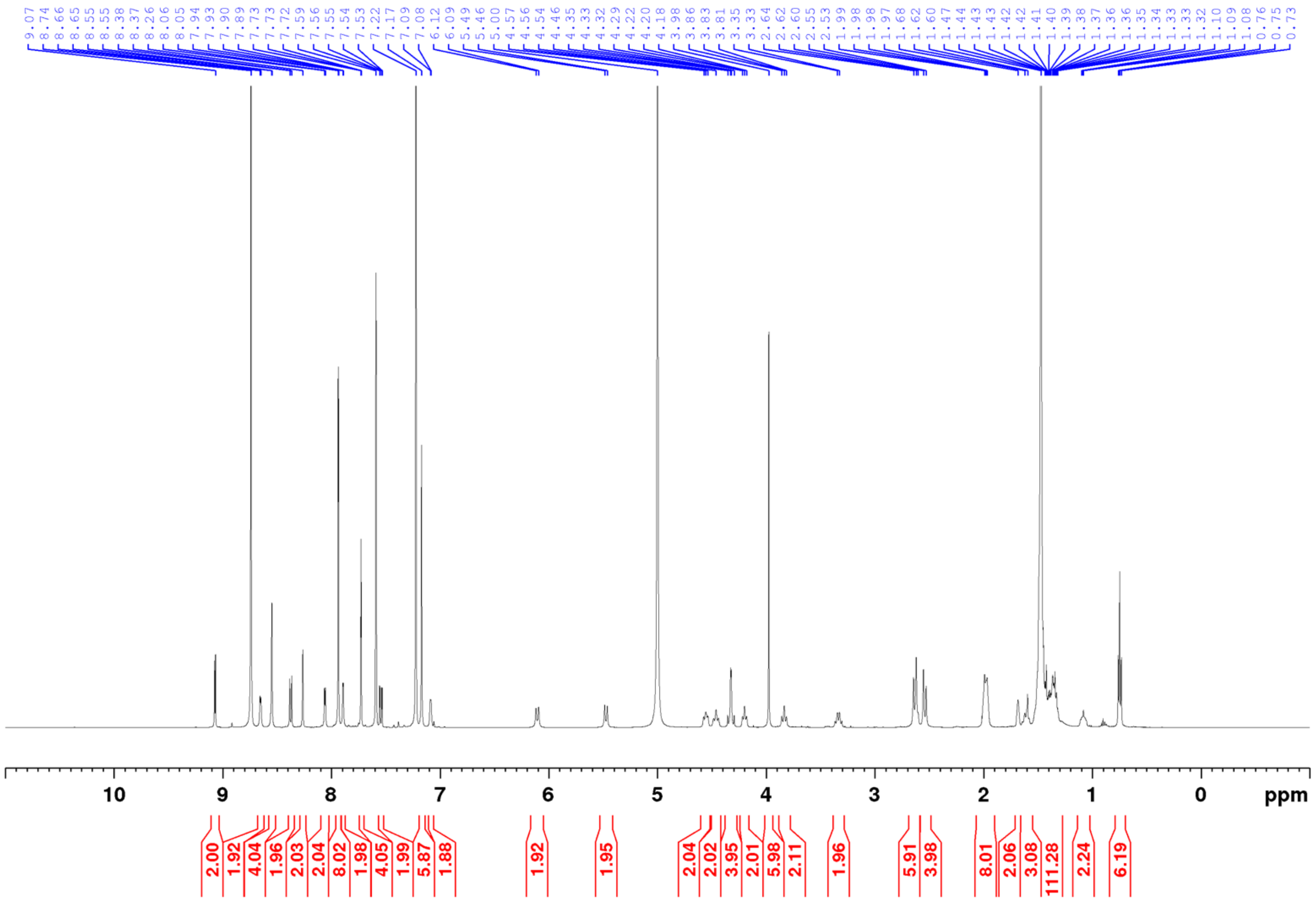

^{13}C NMR (126 MHz, C_5D_5N) for *Bis[rhodium (1S,2R,4S,5R)-5-ethyl-2-((S)-hydroxy(6-methoxyquinolin-4-yl)methyl)-1-((3,3'',5,5''-tetra-tert-butyl-[1,1':3',1''-terphenyl]-5'-yl)methyl)quinuclidin-1-ium (3,5-bis((1-carboxycyclopentyll)methyl)phenyl)methanesulfonate)]* (Rh_2(**C**)$_2$•(**2a**)$_2$)

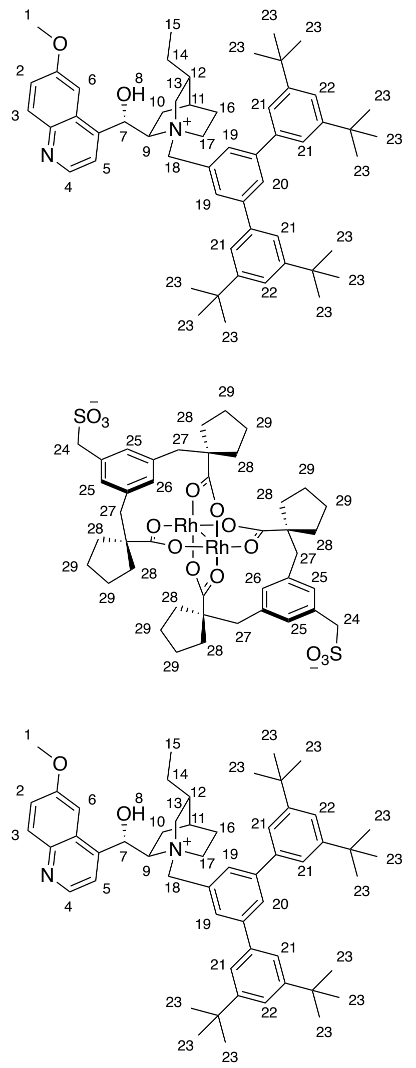

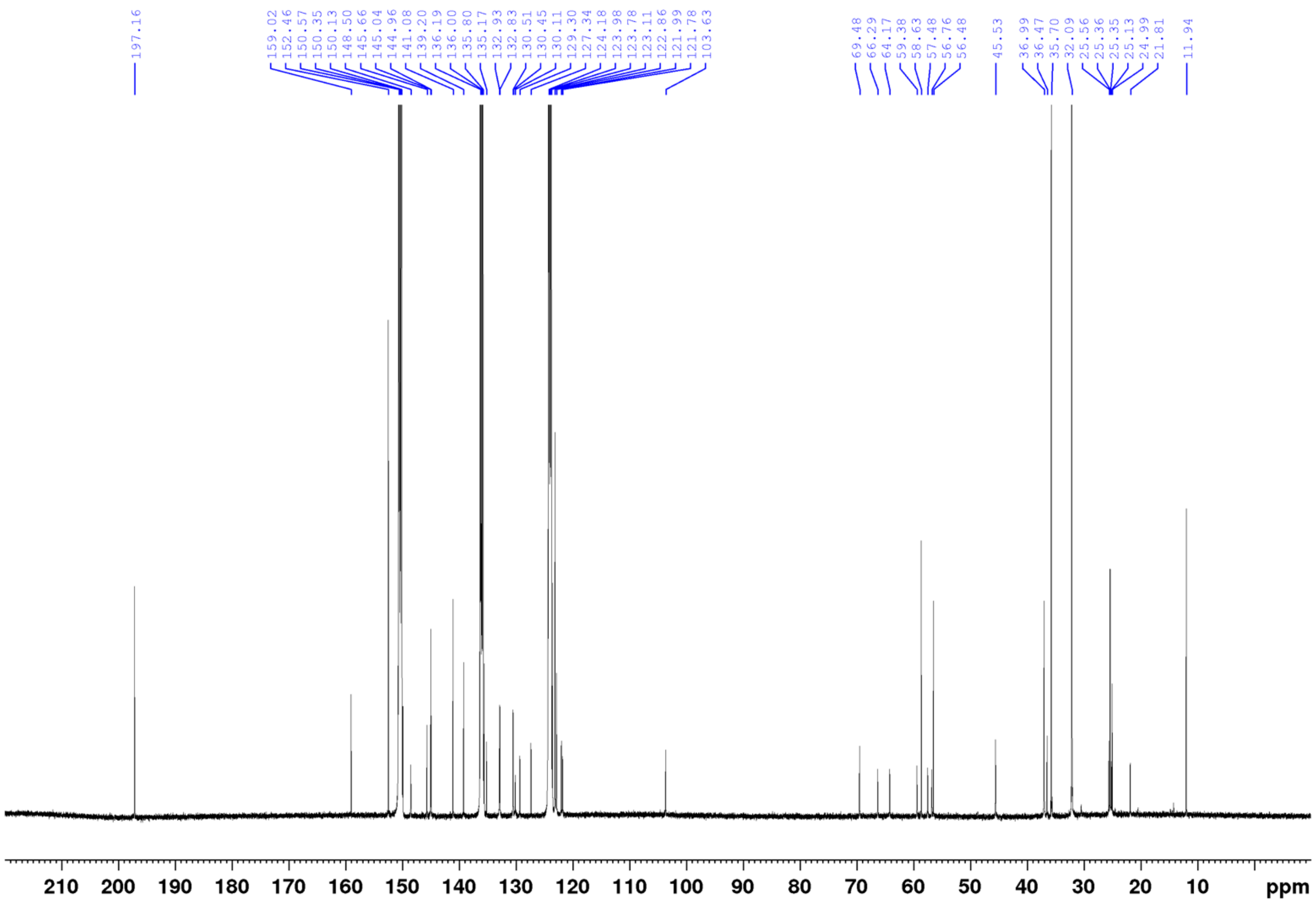

¹H NMR (500 MHz, C_5D_5N) for *Bis[rhodium (1S,2R,4S,5R)-5-ethyl-2-((S)-hydroxy(6-methoxyquinolin-4-yl)methyl)-1-((3,3'',5,5''-tetra-tert-butyl-[1,1':3',1''-terphenyl]-5'-yl)methyl)quinuclidin-1-ium (3,5-bis((1-carboxycycloheptyl)methyl)phenyl)methanesulfonate)]* (Rh_2(**D**)$_2$•(**2a**)$_2$)

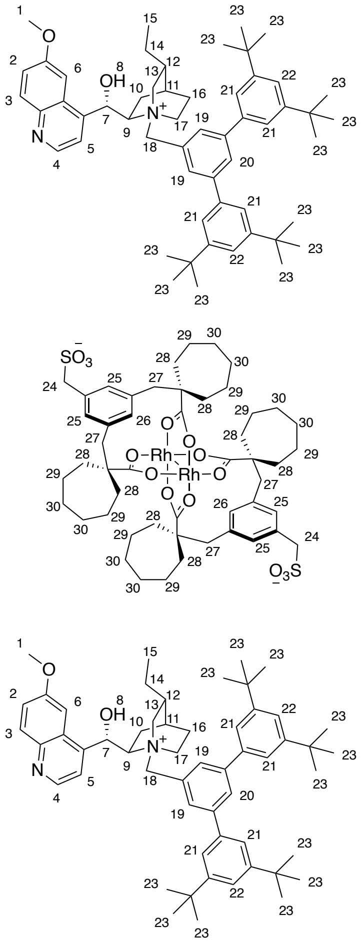

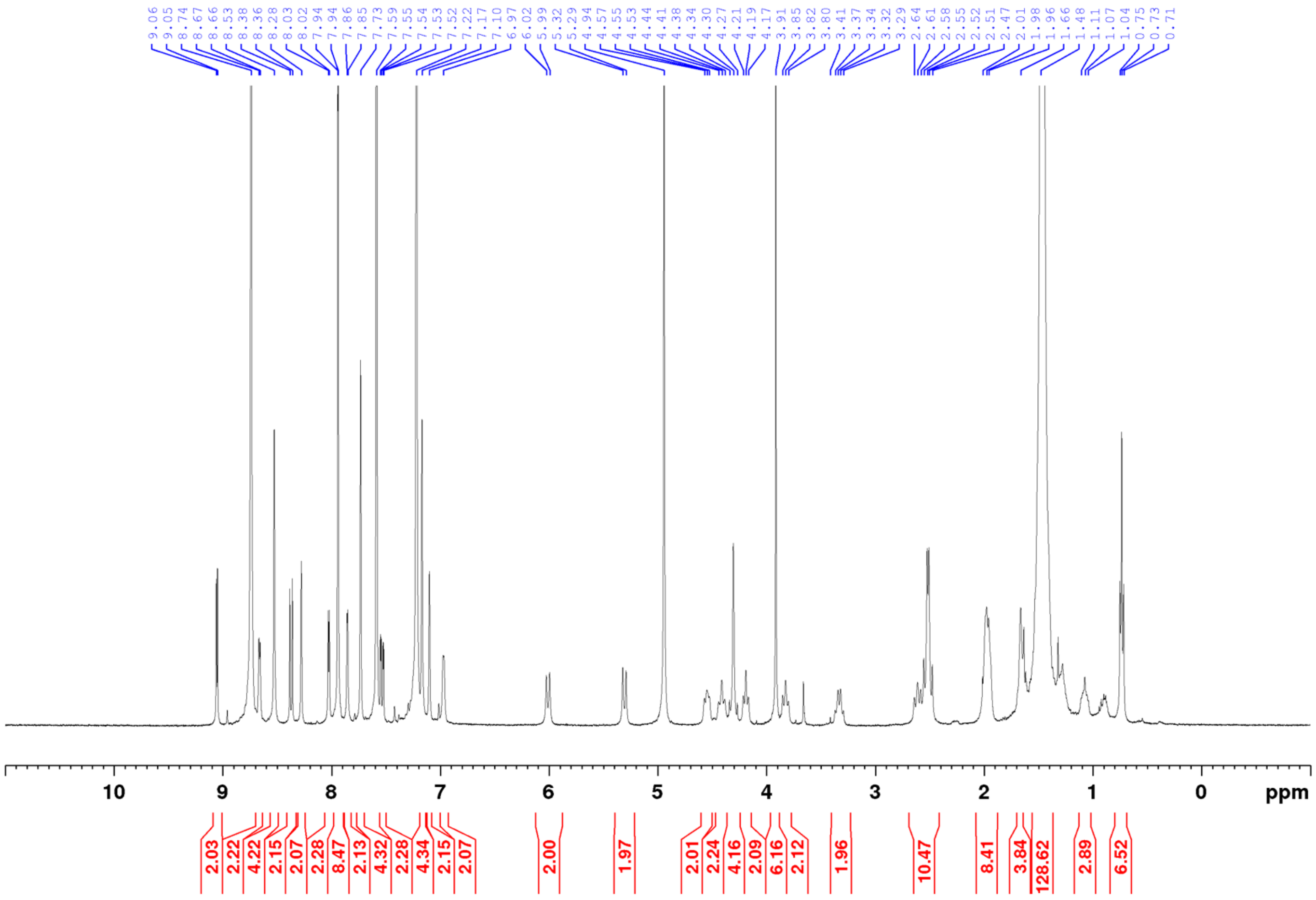

^{13}C NMR (126 MHz, C_5D_5N) for *Bis[rhodium (1S,2R,4S,5R)-5-ethyl-2-((S)-hydroxy(6-methoxyquinolin-4-yl)methyl)-1-((3,3'',5,5''-tetra-tert-butyl-[1,1':3',1''-terphenyl]-5'-yl)methyl)quinuclidin-1-ium (3,5-bis((1-carboxycycloheptyl)methyl)phenyl)methanesulfonate)]* (Rh_2(**D**)$_2$•(**2a**)$_2$)

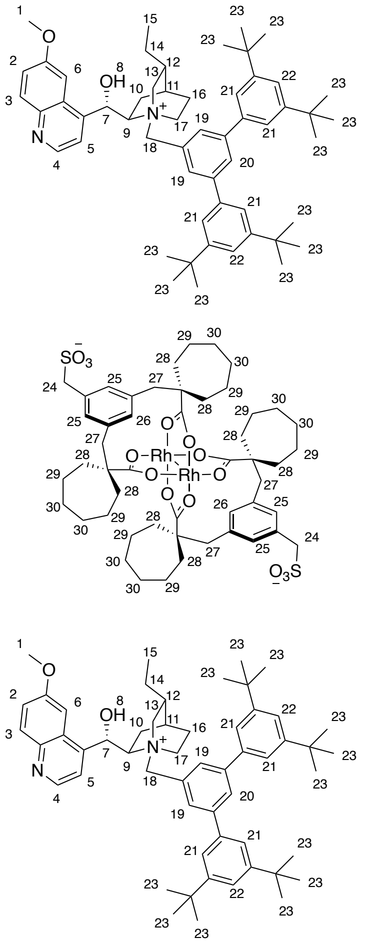

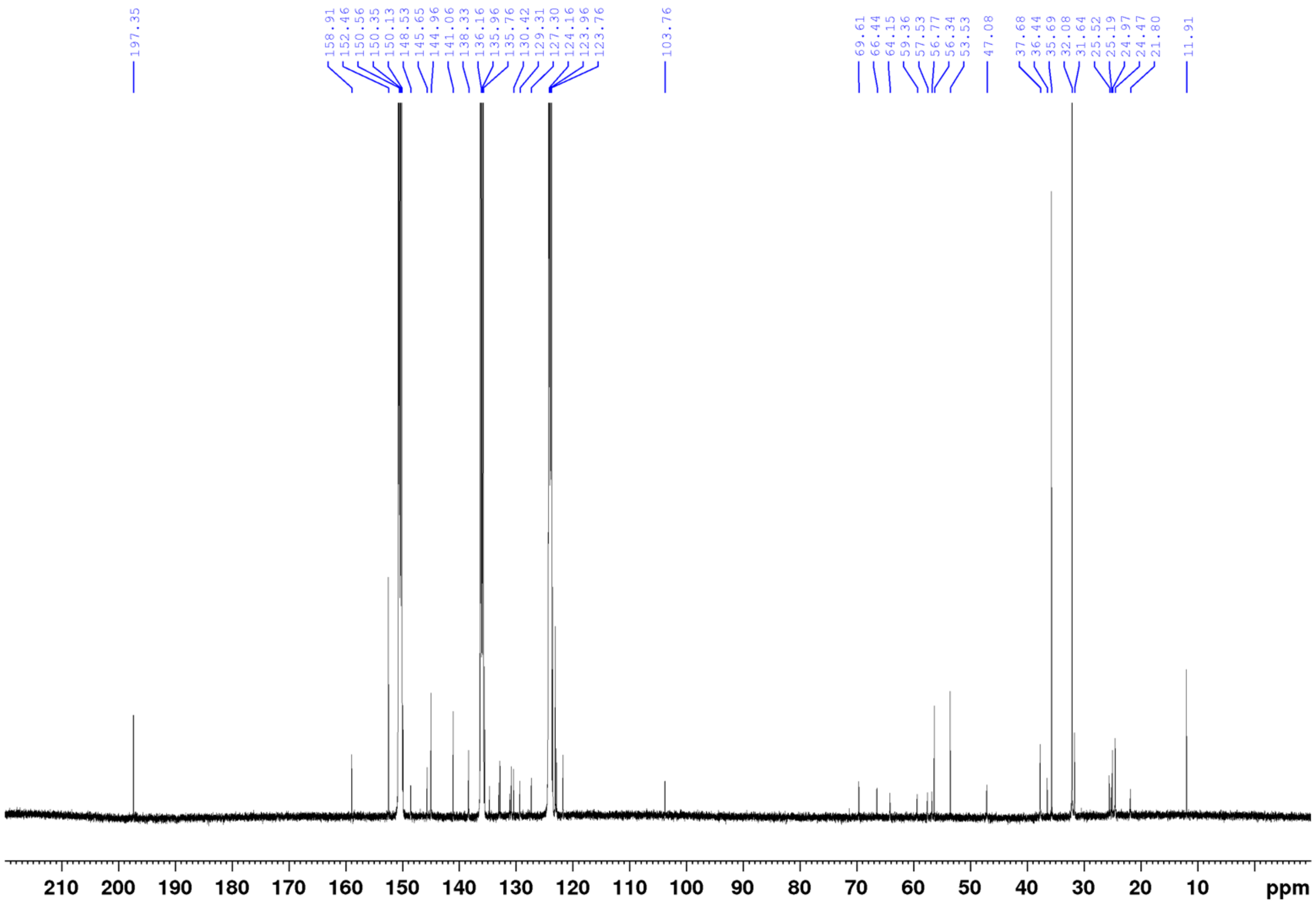

¹H NMR (500 MHz, C_5D_5N) for *Bis[rhodium (1S,2R,4S,5R)-5-ethyl-2-((S)-hydroxy(6-methoxyquinolin-4-yl)methyl)-1-((3,3'',5,5''-tetra-tert-butyl-[1,1':3',1''-terphenyl]-5'-yl)methyl)quinuclidin-1-ium (3,5-bis((1-carboxycyclooctyl)methyl)phenyl)methanesulfonate* (Rh_2(**E**)$_2$•(**2a**)$_2$)

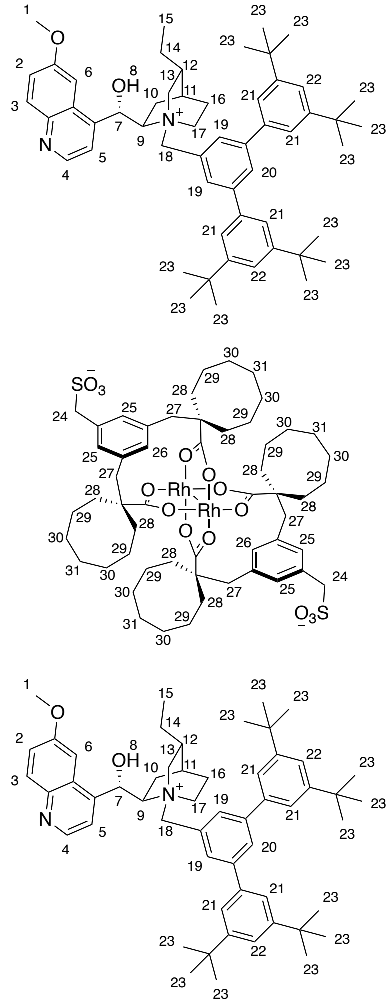

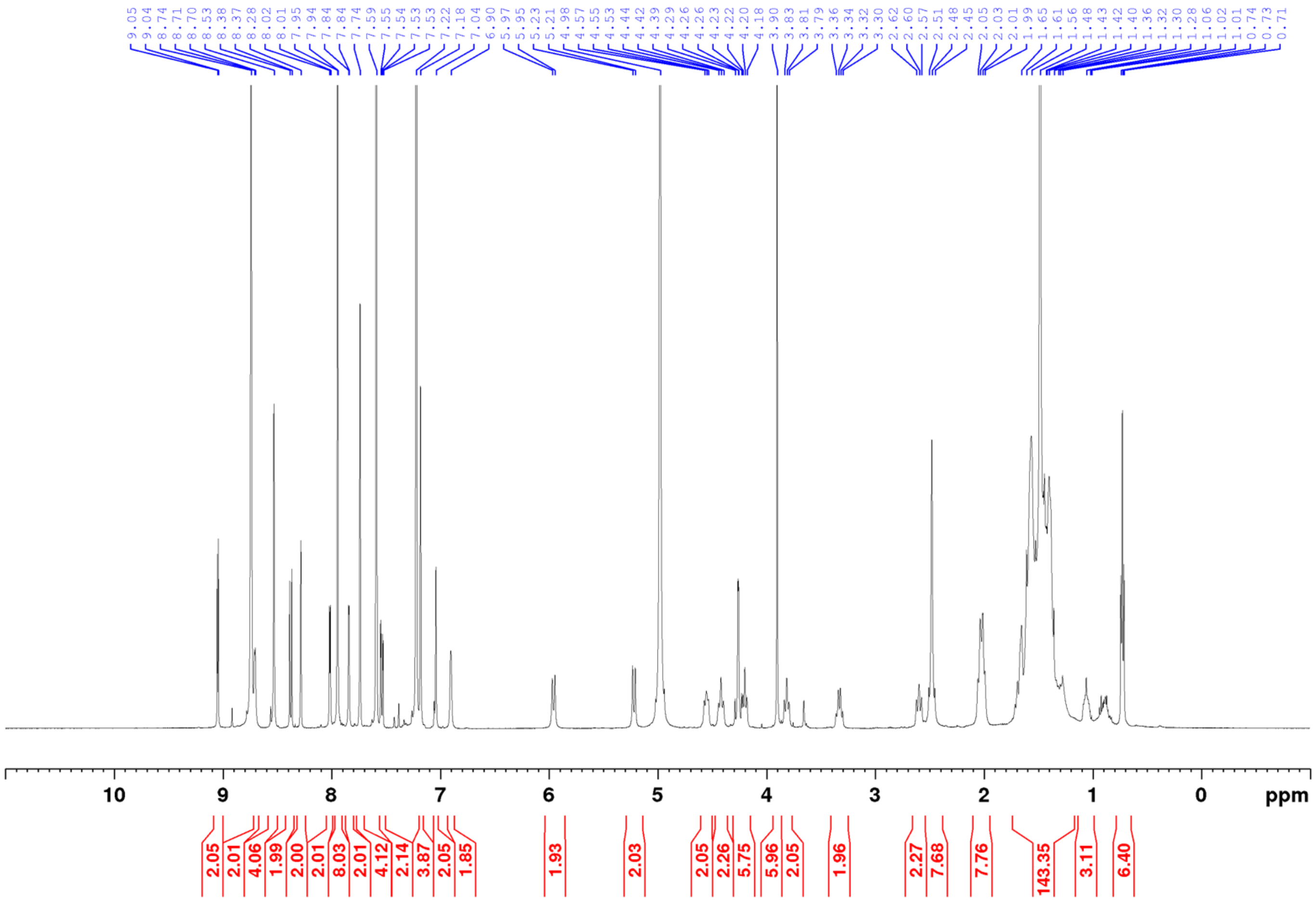

^{13}C NMR (126 MHz, C_5D_5N) for *Bis[rhodium (1S,2R,4S,5R)-5-ethyl-2-((S)-hydroxy(6-methoxyquinolin-4-yl)methyl)-1-((3,3'',5,5''-tetra-tert-butyl-[1,1':3',1''-terphenyl]-5'-yl)methyl)quinuclidin-1-ium (3,5-bis((1-carboxycyclooctyl)methyl)phenyl)methanesulfonate* (Rh_2(**E**)$_2$•(**2a**)$_2$)

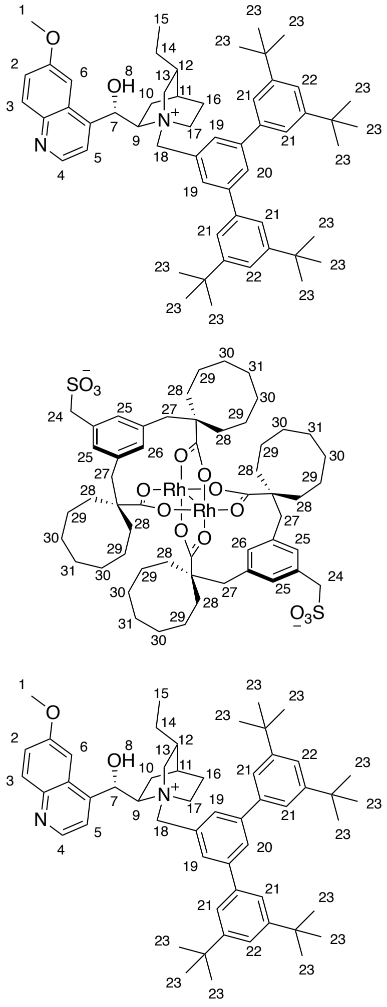

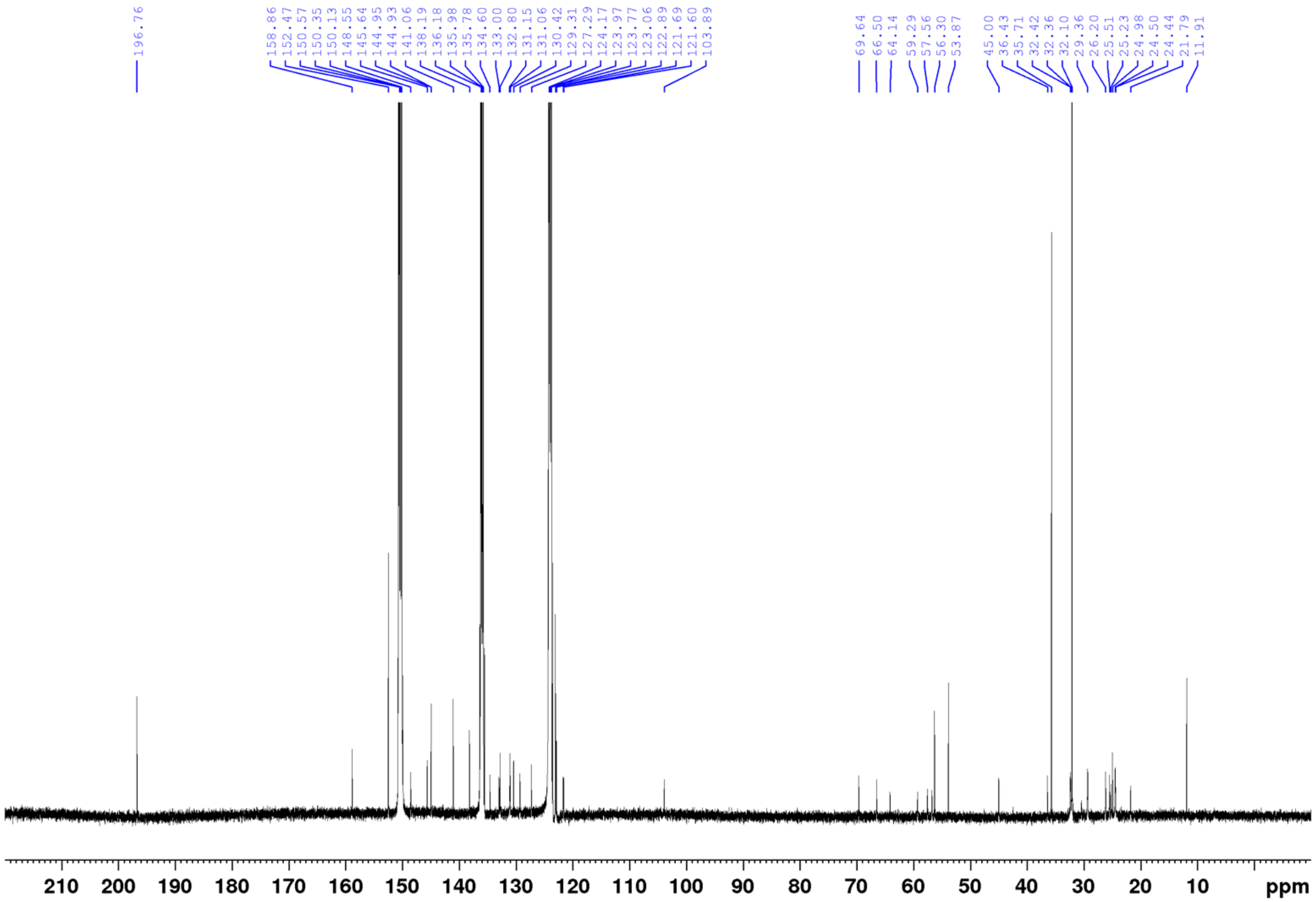

¹H NMR (500 MHz, C_5D_5N) for *Bis[rhodium (1S,2R,4S,5R)-5-ethyl-2-((S)-hydroxy(6-methoxyquinolin-4-yl)methyl)-1-((3,3'',5,5''-tetrakis(trifluoromethyl)-[1,1':3',1''-terphenyl]-5'-yl)methyl)quinuclidin-1-ium (3,5-bis((1-carboxycycloheptyl)methyl)phenyl)methanesulfonate)]* (Rh_2(**D**)$_2$•(**2b**)$_2$)

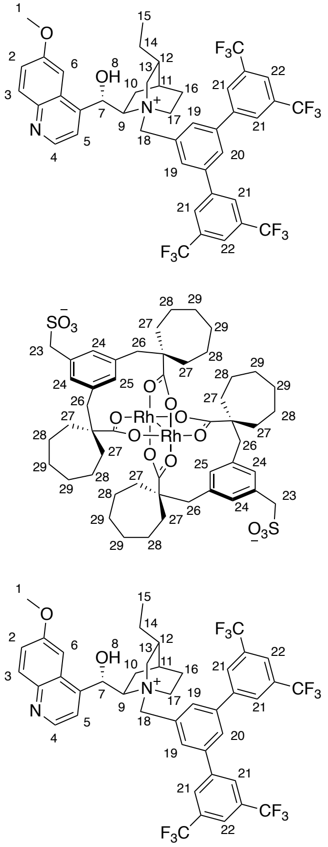

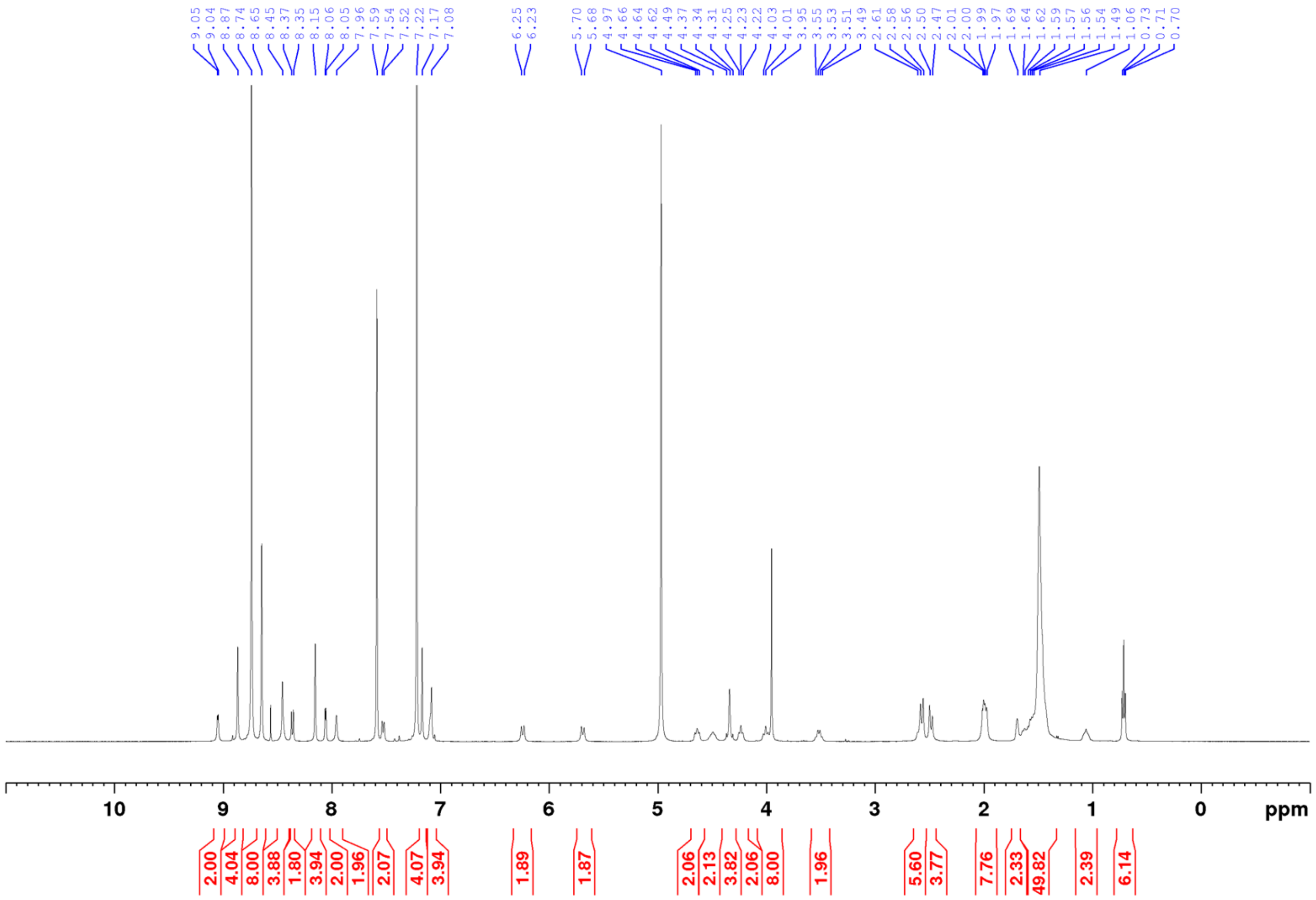

^{13}C NMR (126 MHz, C_5D_5N) for *Bis[rhodium (1S,2R,4S,5R)-5-ethyl-2-((S)-hydroxy(6-methoxyquinolin-4-yl)methyl)-1-((3,3'',5,5''-tetrakis(trifluoromethyl)-[1,1':3',1''-terphenyl]-5'-yl)methyl)quinuclidin-1-ium (3,5-bis((1-carboxycycloheptyl)methyl)phenyl)methanesulfonate)]* (Rh_2(**D**)$_2$•(**2b**)$_2$)

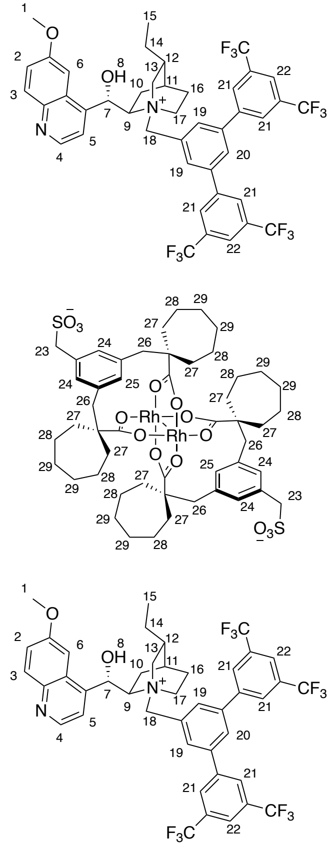

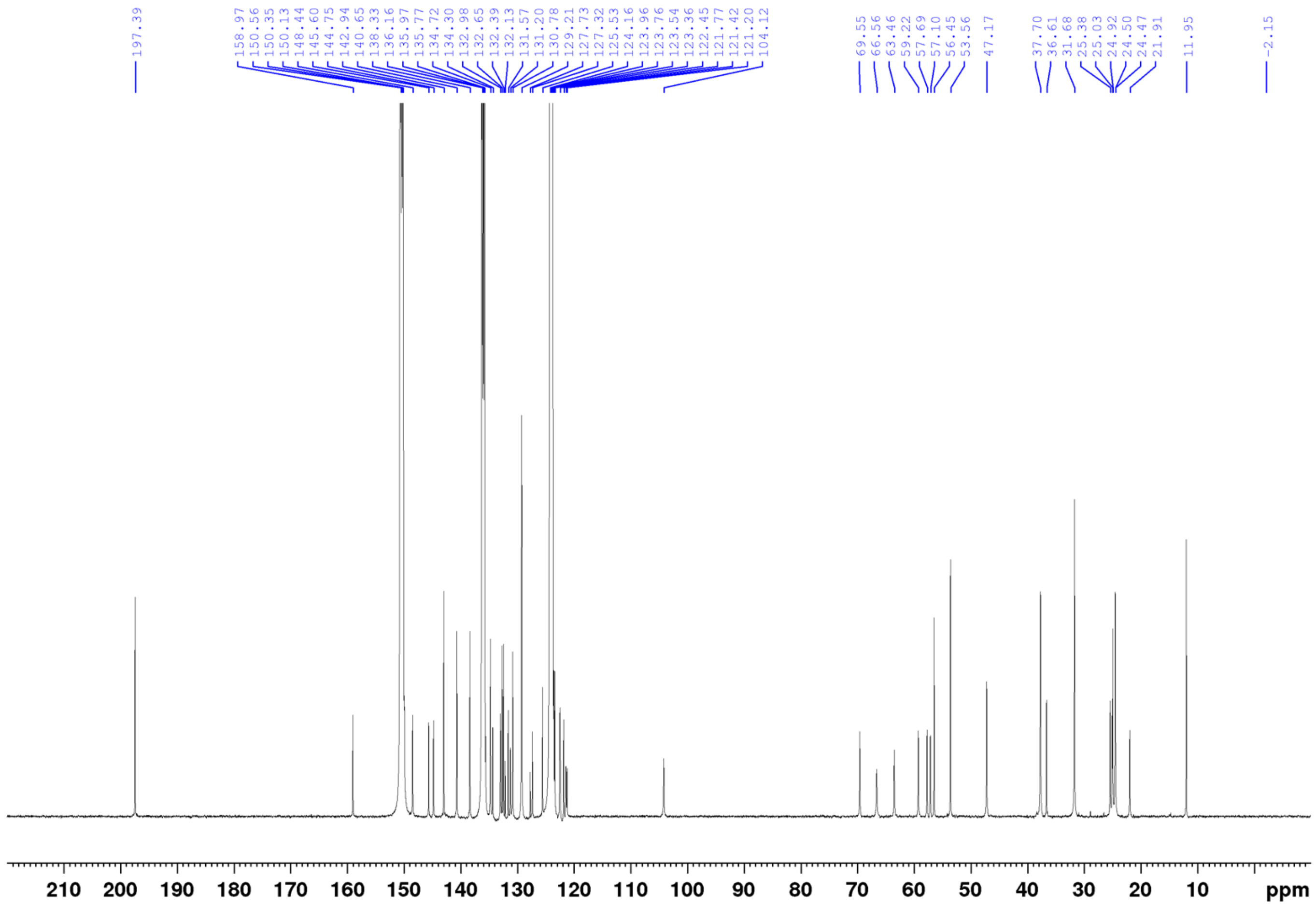

^{19}F NMR (376 MHz, C_5D_5N) for *Bis[rhodium (1S,2R,4S,5R)-5-ethyl-2-((S)-hydroxy(6-methoxyquinolin-4-yl)methyl)-1-((3,3'',5,5''-tetrakis(trifluoromethyl)-[1,1':3',1''-terphenyl]-5'-yl)methyl)quinuclidin-1-ium (3,5-bis((1-carboxycycloheptyl)methyl)phenyl)methanesulfonate)]* (Rh_2(**D**)$_2$•(**2b**)$_2$)

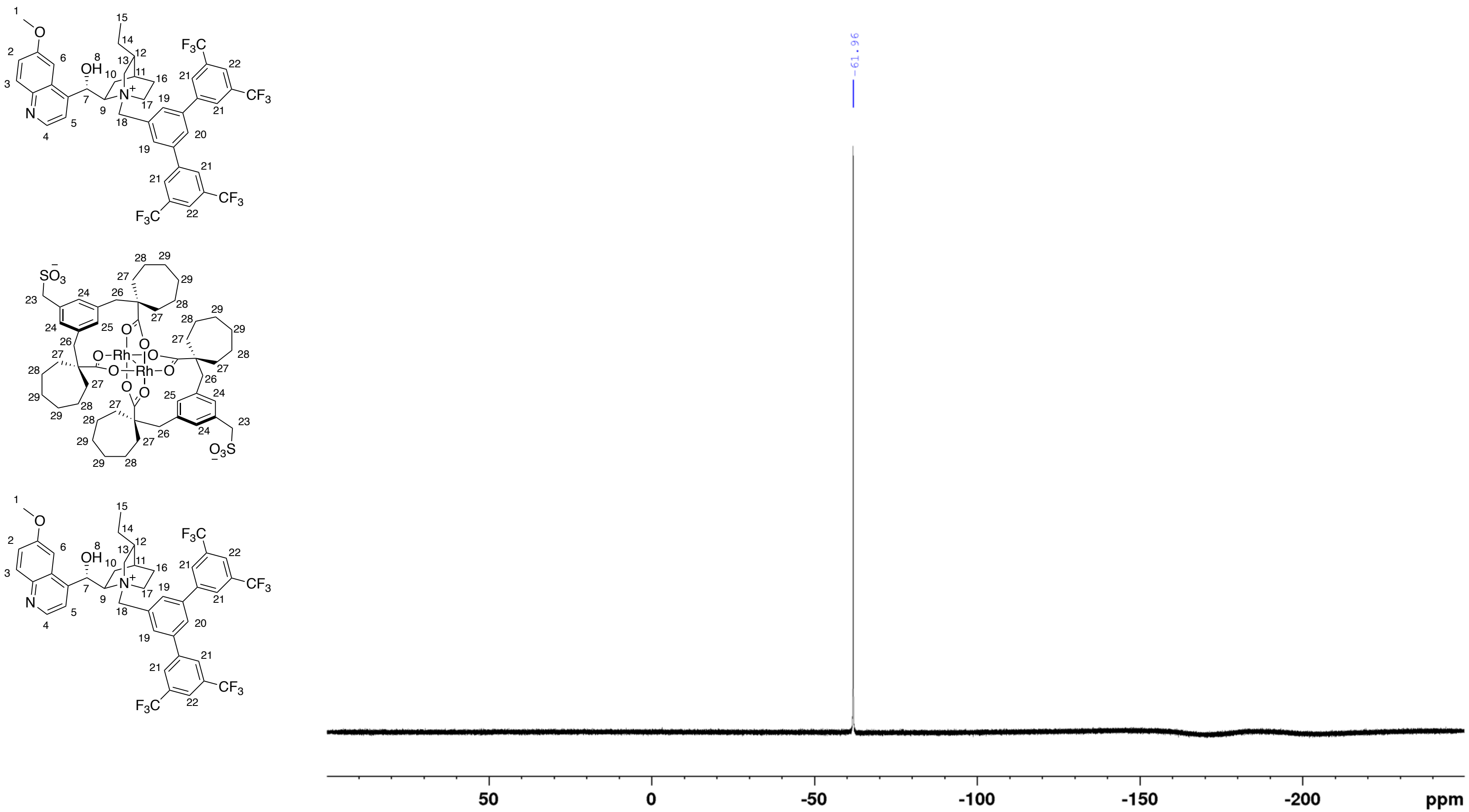

^{1}H NMR (500 MHz, C_5D_5N) for *Bis[rhodium (1S,2R,4S,5R)-1-([1,1':3',1''-terphenyl]-5'-ylmethyl)-5-ethyl-2-((S)-hydroxy(6-methoxyquinolin-4-yl)methyl)quinuclidin-1-ium (3,5-bis((1-carboxycycloheptyl)methyl)phenyl)methanesulfonate)]* (Rh_2(**D**)$_2$•(**2c**)$_2$)

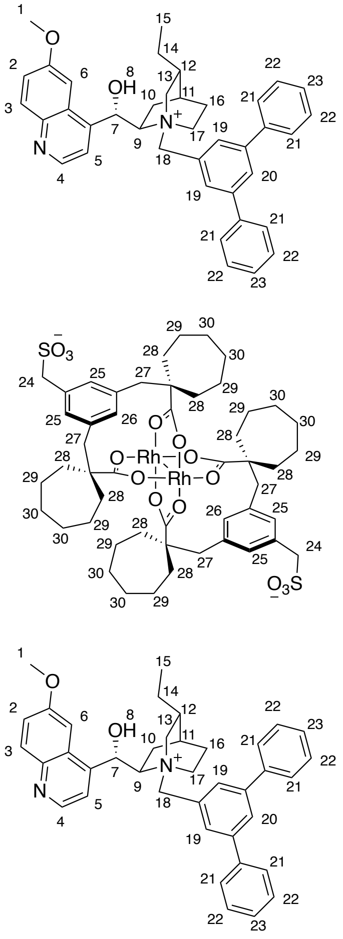

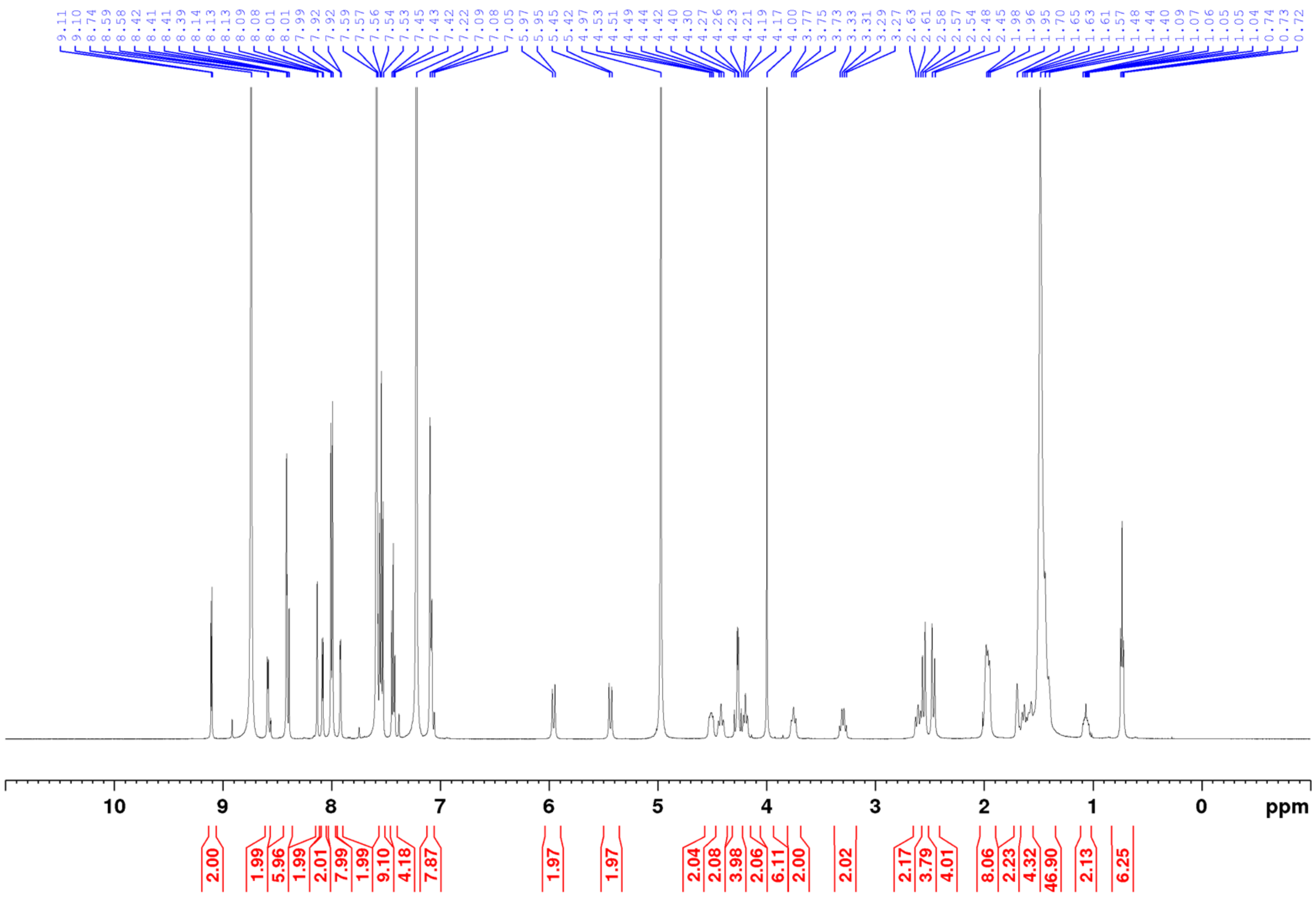

^{13}C NMR (126 MHz, C_5D_5N) for *Bis[rhodium (1S,2R,4S,5R)-1-([1,1':3',1''-terphenyl]-5'-ylmethyl)-5-ethyl-2-((S)-hydroxy(6-methoxyquinolin-4-yl)methyl)quinuclidin-1-ium (3,5-bis((1-carboxycycloheptyl)methyl)phenyl)methanesulfonate)]* (Rh_2(**D**)$_2$•(**2c**)$_2$)

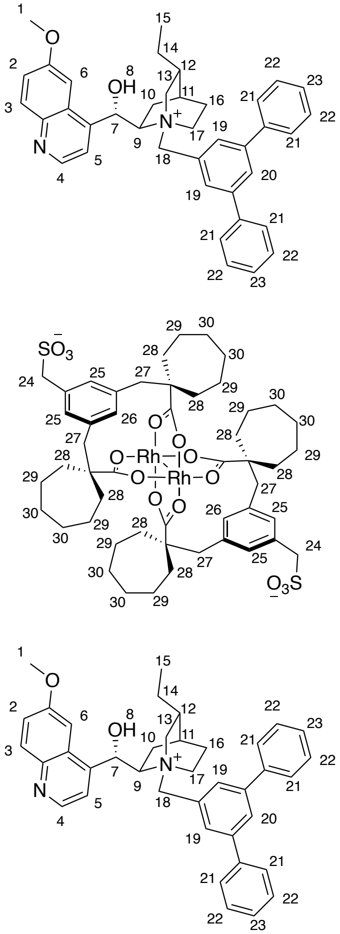

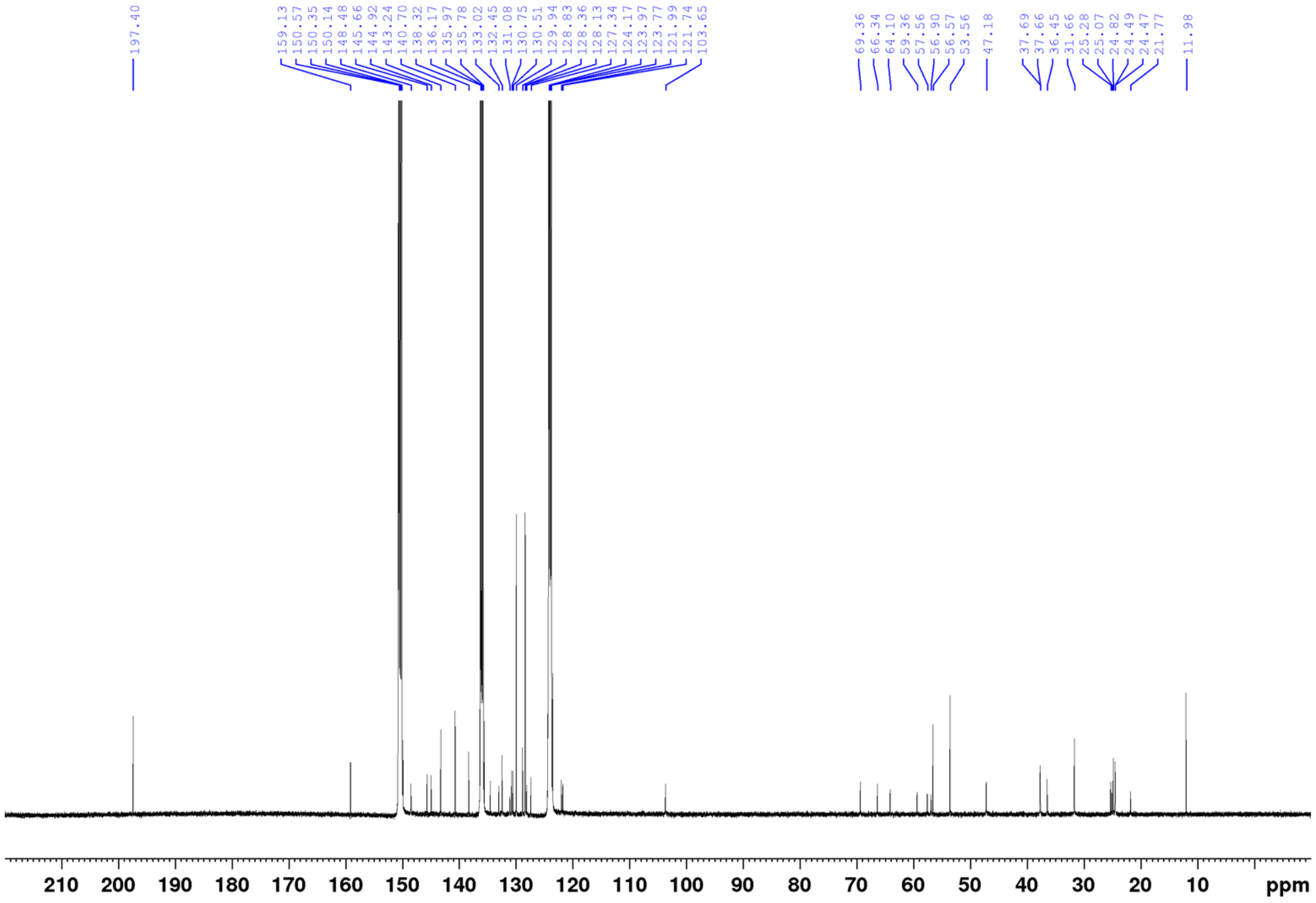

¹H NMR (500 MHz, C_5D_5N) for *Bis[rhodium (1S,2R,4S,5R)-1-(3,5-di-tert-butylbenzyl)-5-ethyl-2-((S)-hydroxy(6-methoxyquinolin-4-yl)methyl)quinuclidin-1-ium (3,5-bis((1-carboxycycloheptyl)methyl)phenyl)methanesulfonate)]* (Rh_2(**D**)$_2$•(**2d**)$_2$)

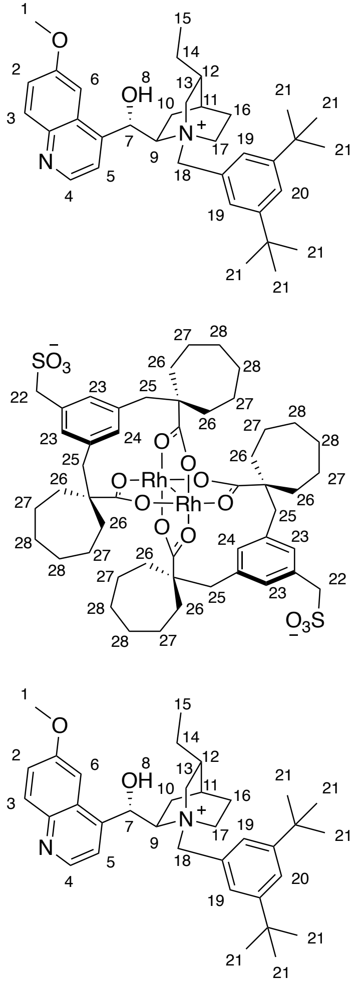

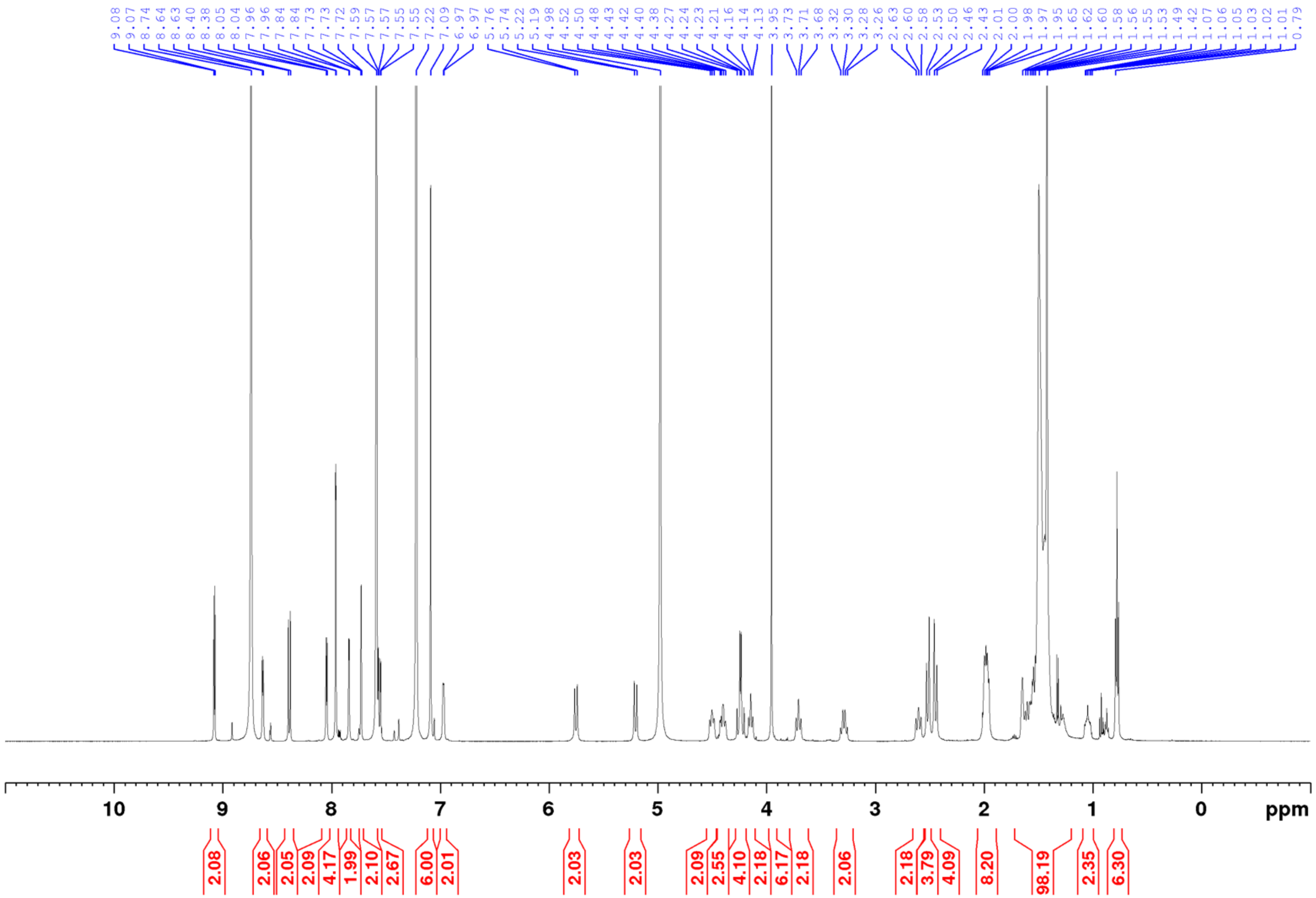

^{13}C NMR (126 MHz, C_5D_5N) for *Bis[rhodium (1S,2R,4S,5R)-1-(3,5-di-tert-butylbenzyl)-5-ethyl-2-((S)-hydroxy(6-methoxyquinolin-4-yl)methyl)quinuclidin-1-ium (3,5-bis((1-carboxycycloheptyl)methyl)phenyl)methanesulfonate)]* (Rh_2(**D**)$_2$•(**2d**)$_2$)

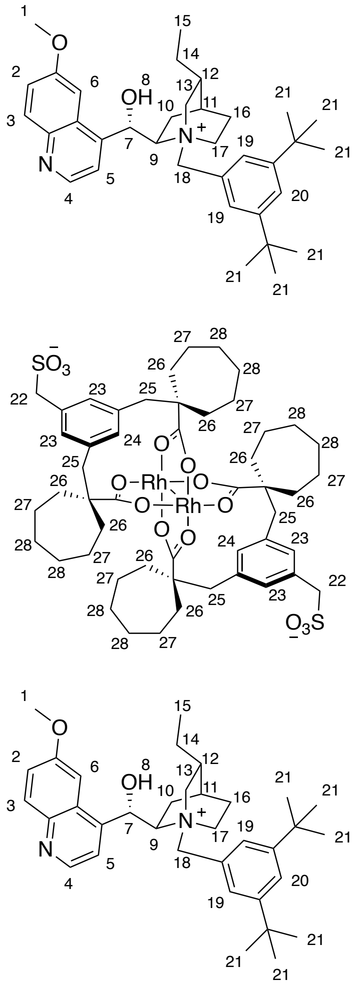

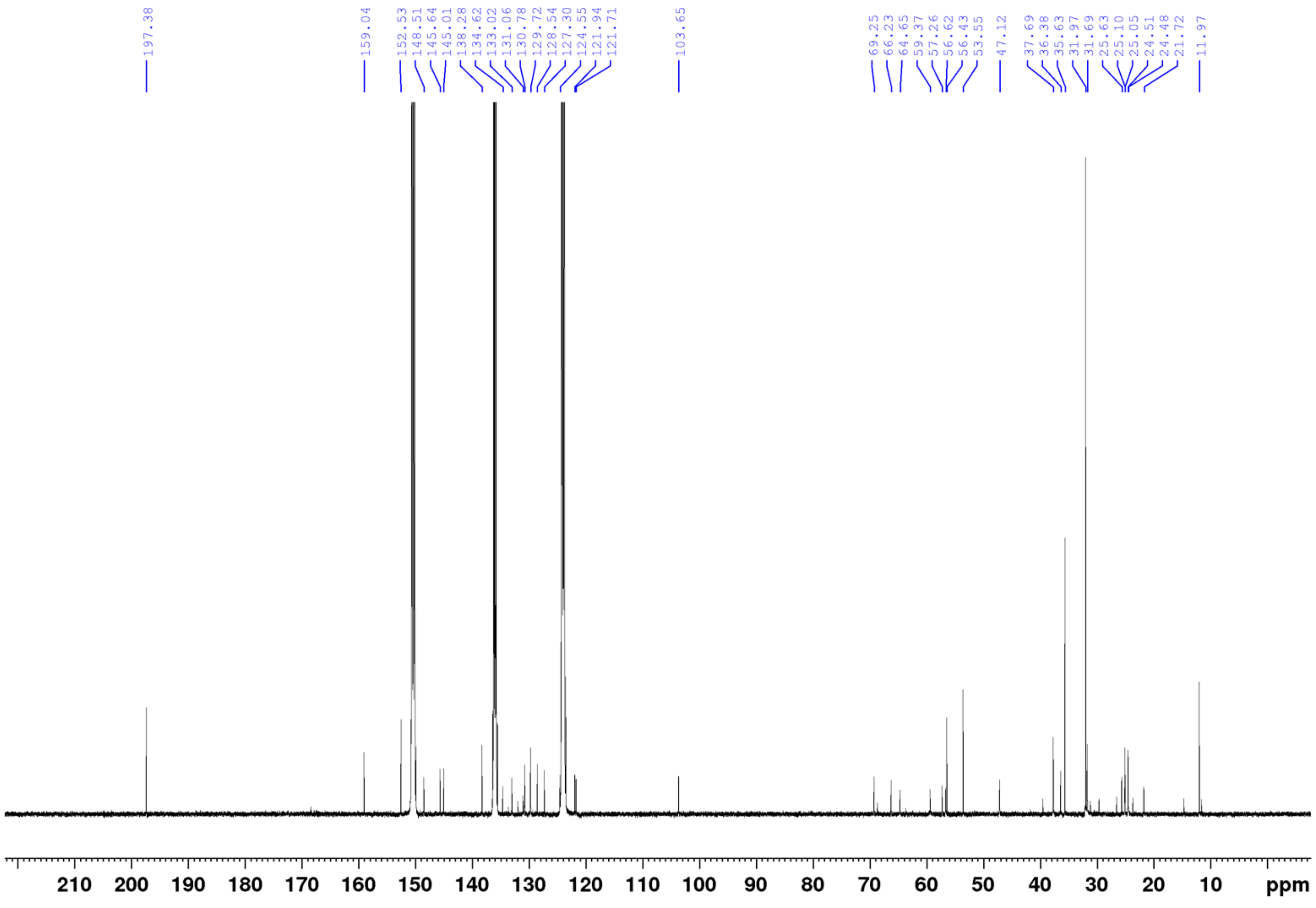

^{1}H NMR (500 MHz, C_5D_5N) for *Bis[rhodium (1S,2R,4S,5R)-1-(3,5-bis(trifluoromethyl)benzyl)-5-ethyl-2-((S)-hydroxy(6-methoxyquinolin-4-yl)methyl)quinuclidin-1-ium (3,5-bis((1-carboxycycloheptyl)methyl)phenyl)methanesulfonate)]* (Rh_2(**D**)$_2$•(**2e**)$_2$)

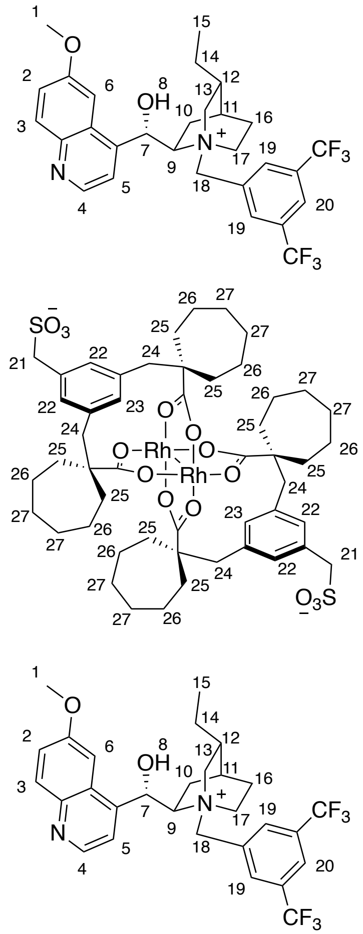

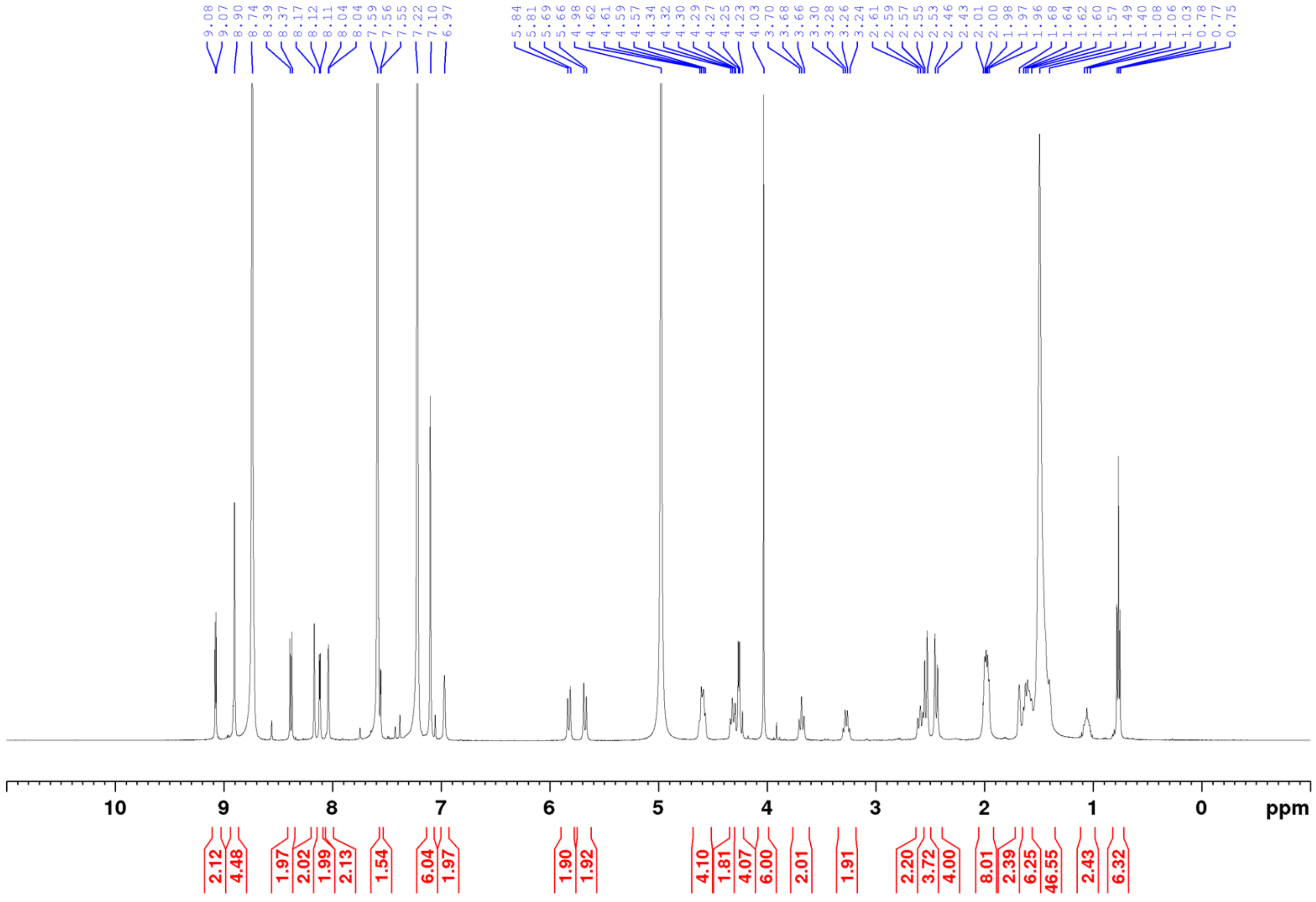

^{13}C NMR (126 MHz, C_5D_5N) for *Bis[rhodium (1S,2R,4S,5R)-1-(3,5-bis(trifluoromethyl)benzyl)-5-ethyl-2-((S)-hydroxy(6-methoxyquinolin-4-yl)methyl)quinuclidin-1-ium (3,5-bis((1-carboxycycloheptyl)methyl)phenyl)methanesulfonate)]* (Rh_2(**D**)$_2$•(**2e**)$_2$)

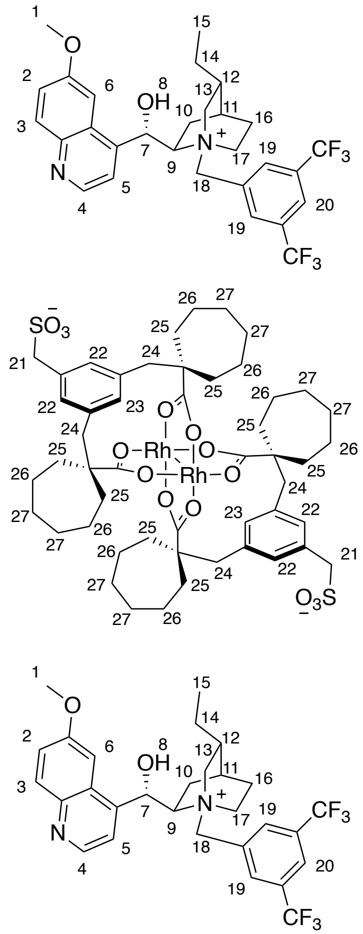

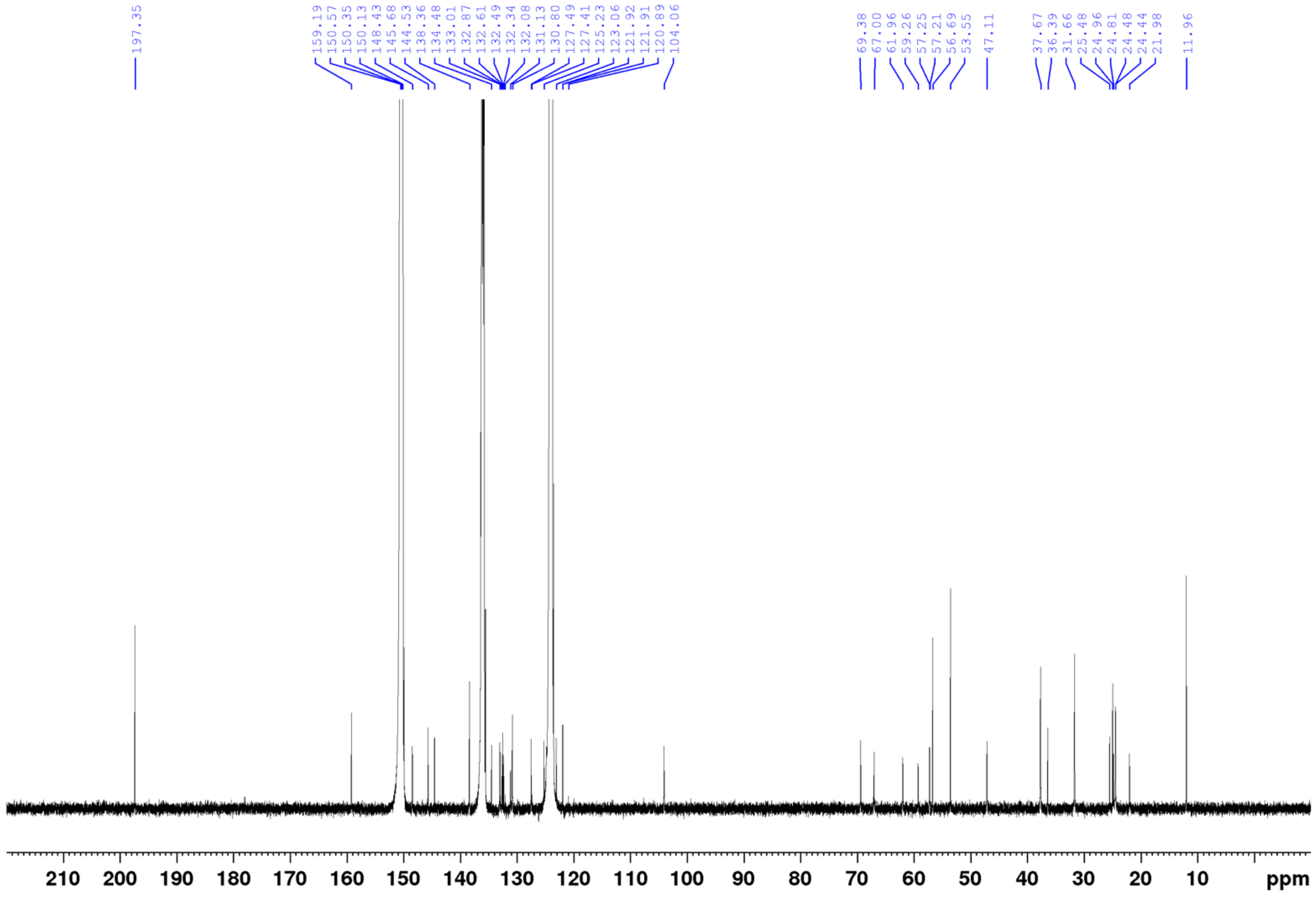

^{19}F NMR (376 MHz, C_5D_5N) for *Bis[rhodium (1S,2R,4S,5R)-1-(3,5-bis(trifluoromethyl)benzyl)-5-ethyl-2-((S)-hydroxy(6-methoxyquinolin-4-yl)methyl)quinuclidin-1-ium (3,5-bis((1-carboxycycloheptyl)methyl)phenyl)methanesulfonate)]* (Rh_2(**D**)$_2$•(**2e**)$_2$)

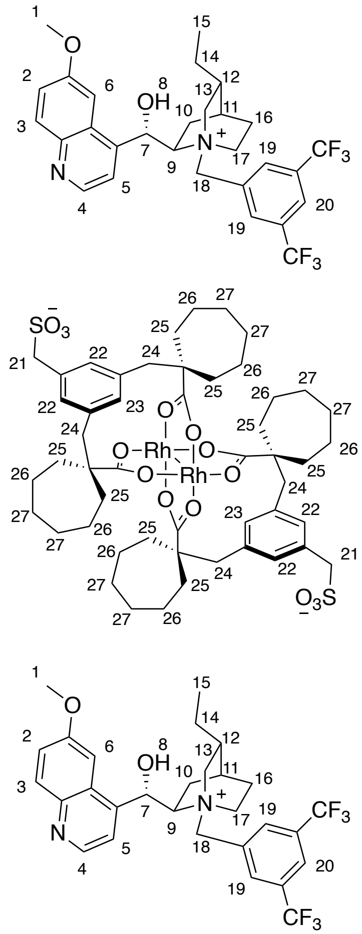

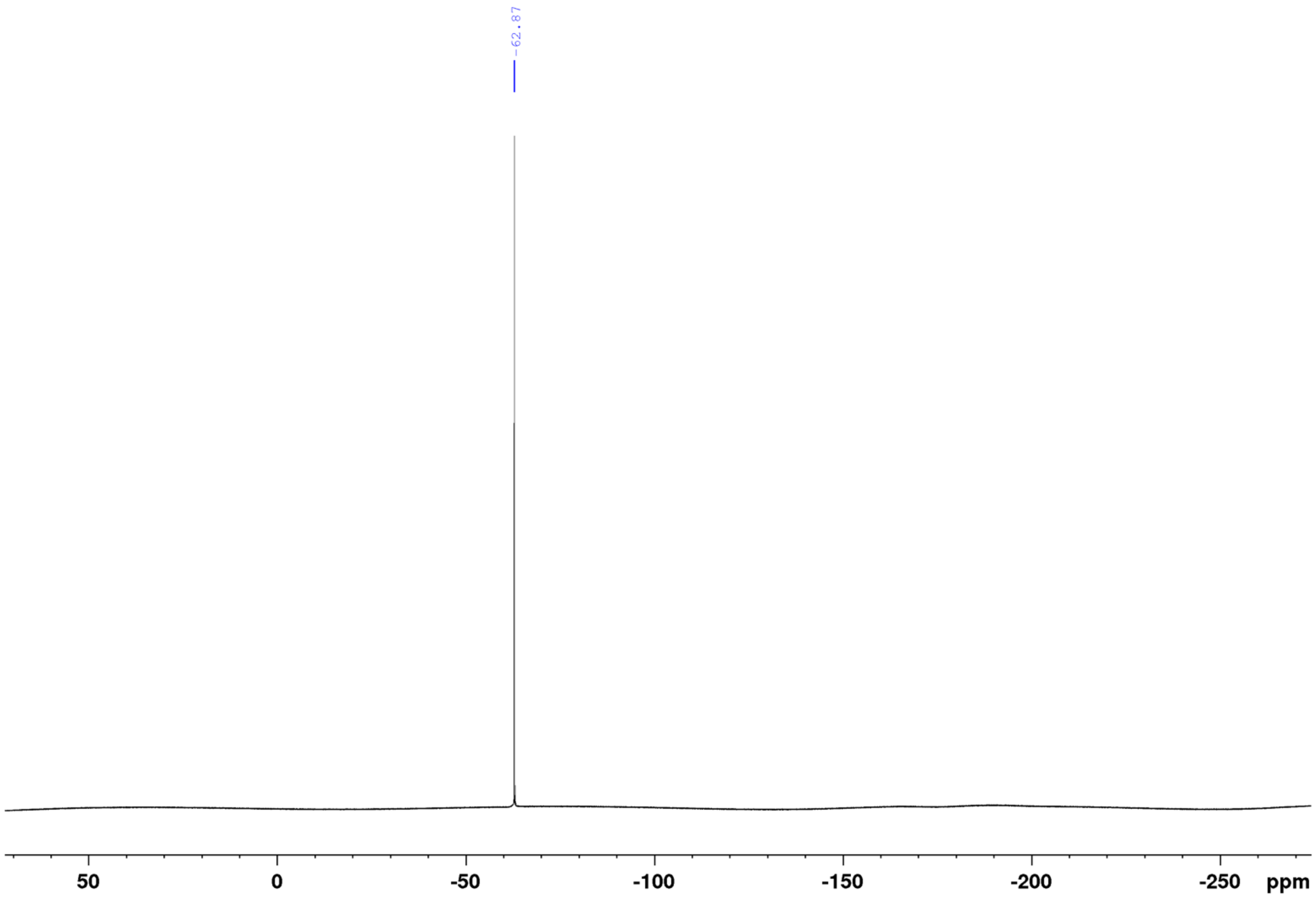

¹H NMR (500 MHz, C_5D_5N) for *Bis[rhodium (1R,2S,4R)-2-((R)-hydroxy(6-methoxyquinolin-4-yl)methyl)-1-((3,3'',5,5''-tetra-tert-butyl-[1,1':3',1''-terphenyl]-5'-yl)methyl)quinuclidin-1-ium (3,5-bis((1-carboxycycloheptyl)methyl)phenyl)methanesulfonate)]* (Rh_2(**D**)$_2$•(**10**)$_2$)

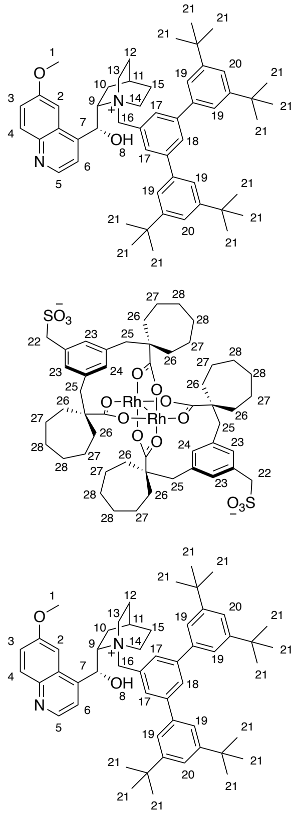

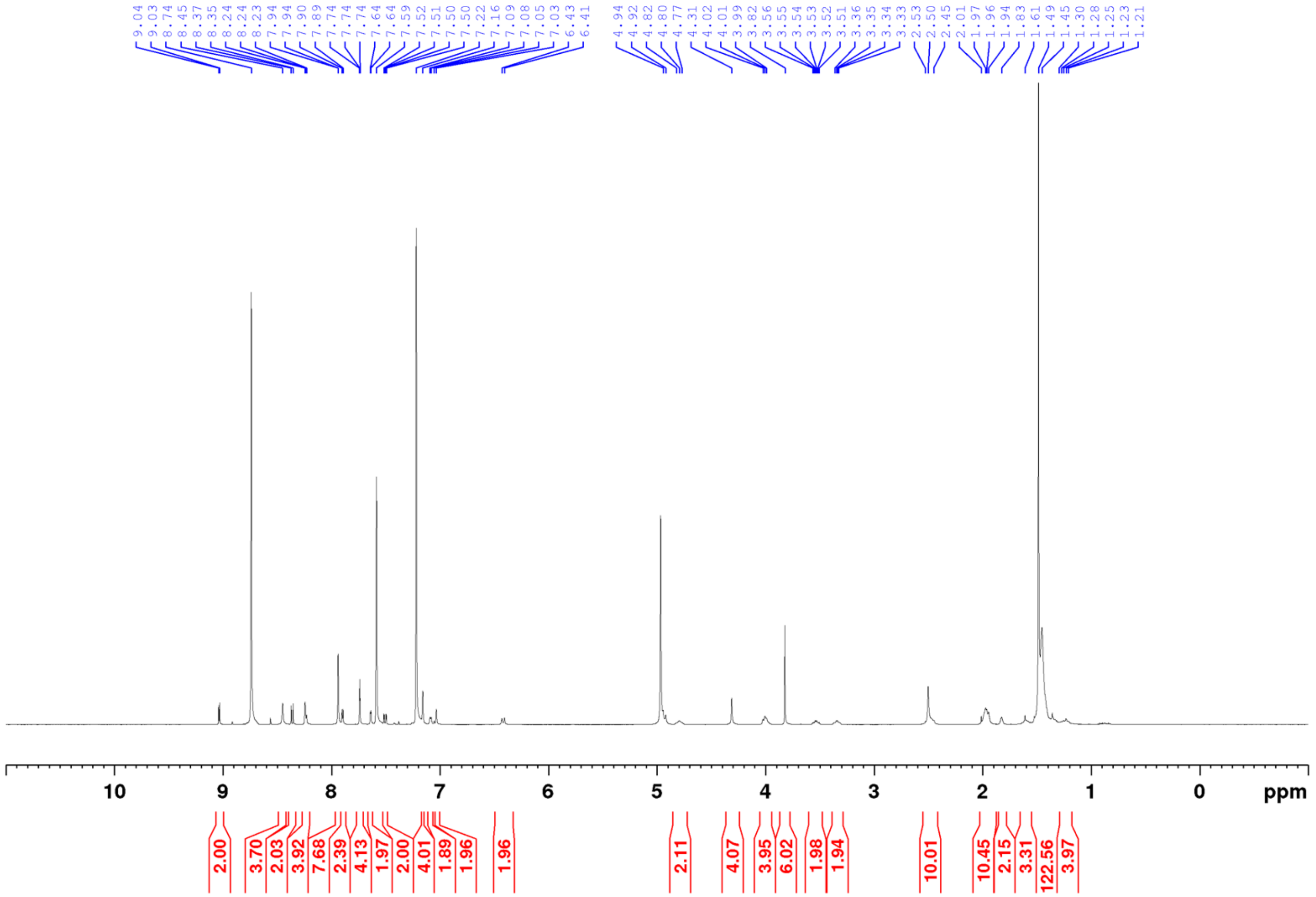

^{13}C NMR (126 MHz, C_5D_5N) for *Bis[rhodium (1R,2S,4R)-2-((R)-hydroxy(6-methoxyquinolin-4-yl)methyl)-1-((3,3'',5,5''-tetra-tert-butyl-[1,1':3',1''-terphenyl]-5'-yl)methyl)quinuclidin-1-ium (3,5-bis((1-carboxycycloheptyl)methyl)phenyl)methanesulfonate)]* (Rh_2(**D**)$_2$•(**10**)$_2$)

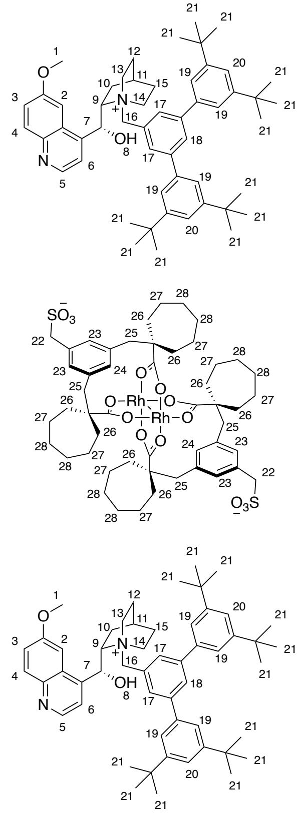

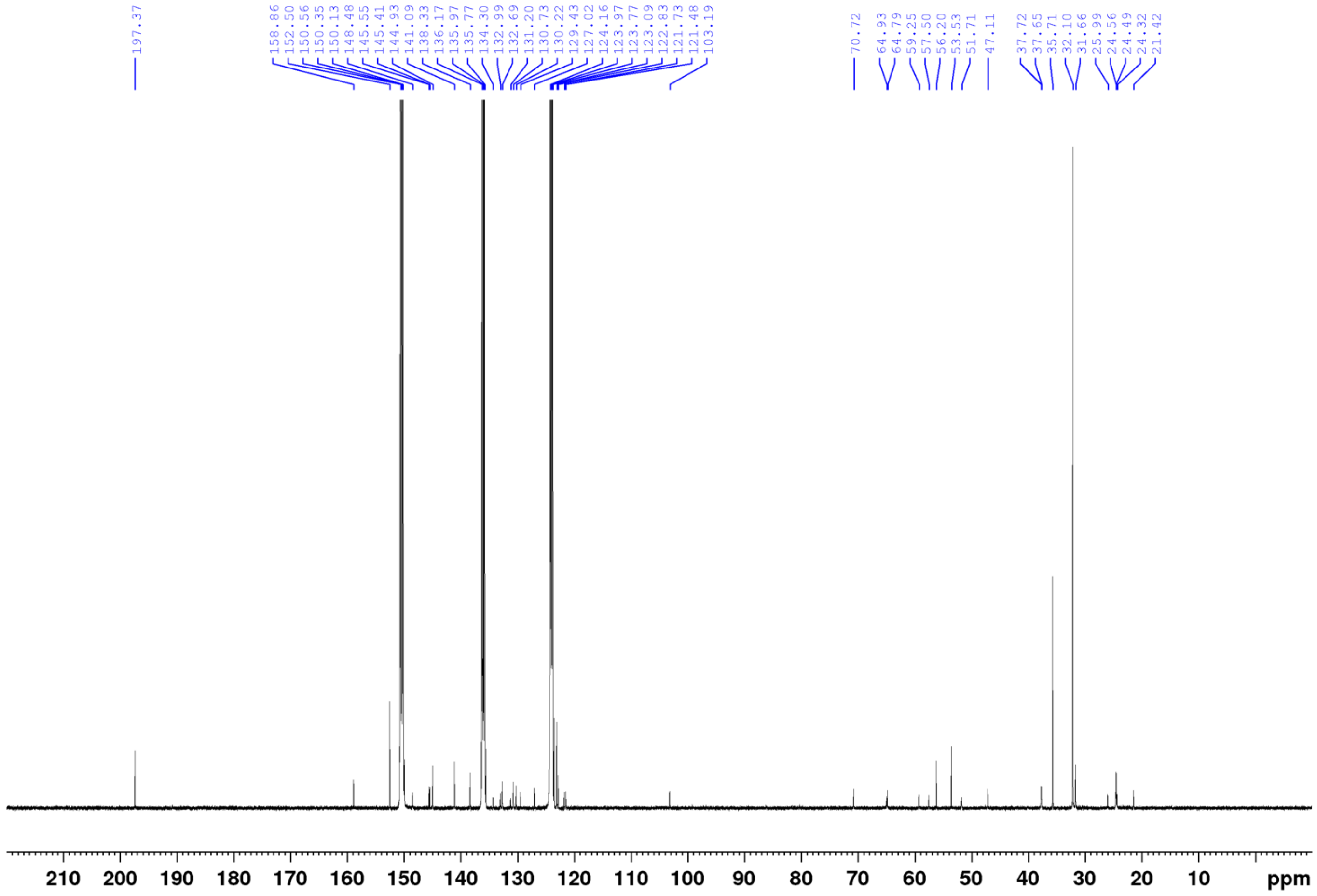

[1]**H NMR** (400 MHz, $(CD_3)_2CO$) for *2,2,3,3,4,4,4-heptafluorobutyl sulfamate* (**5**)

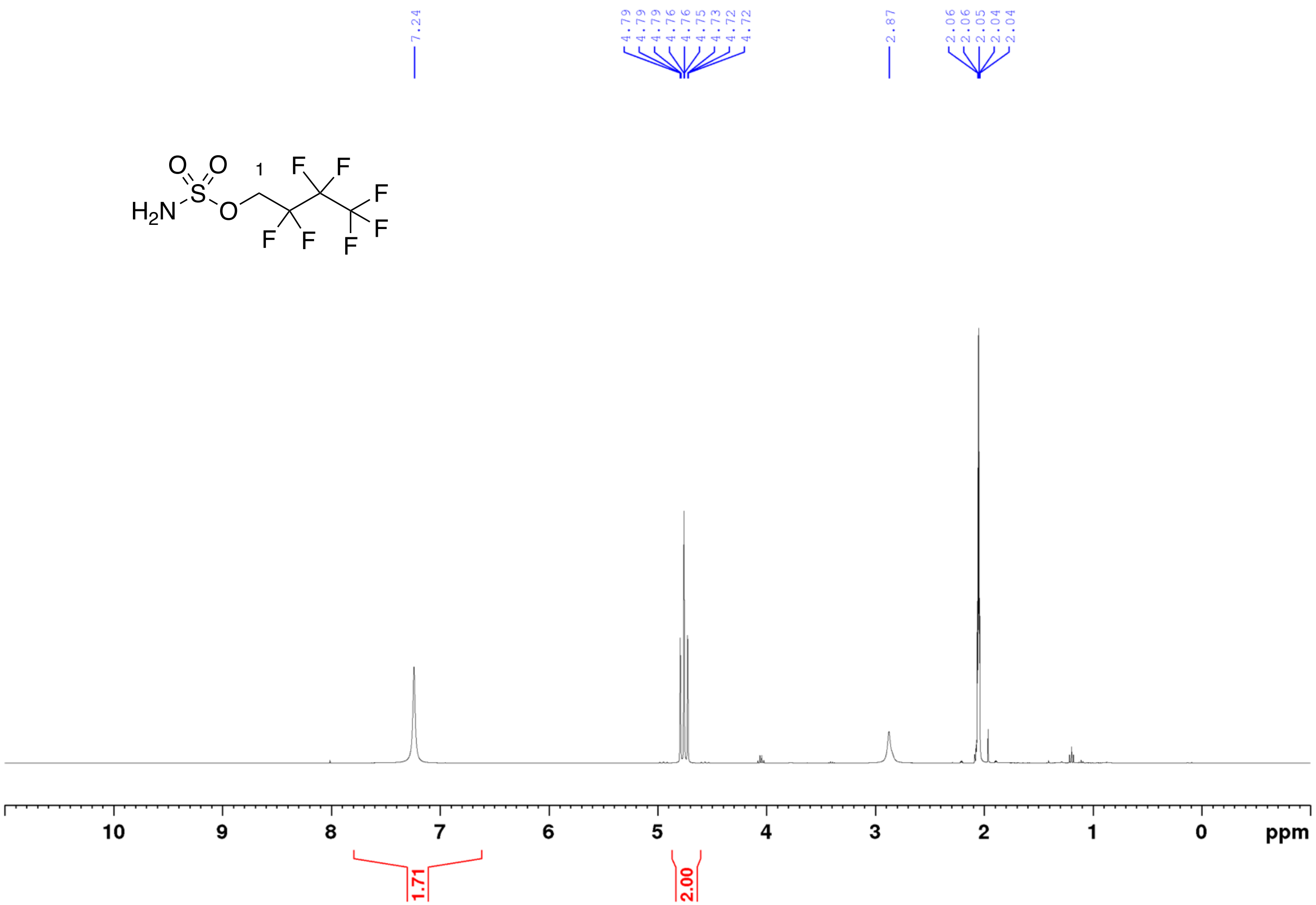

^{13}C NMR (101 MHz, $(CD_3)_2CO$) for *2,2,3,3,4,4,4-heptafluorobutyl sulfamate* (**5**)

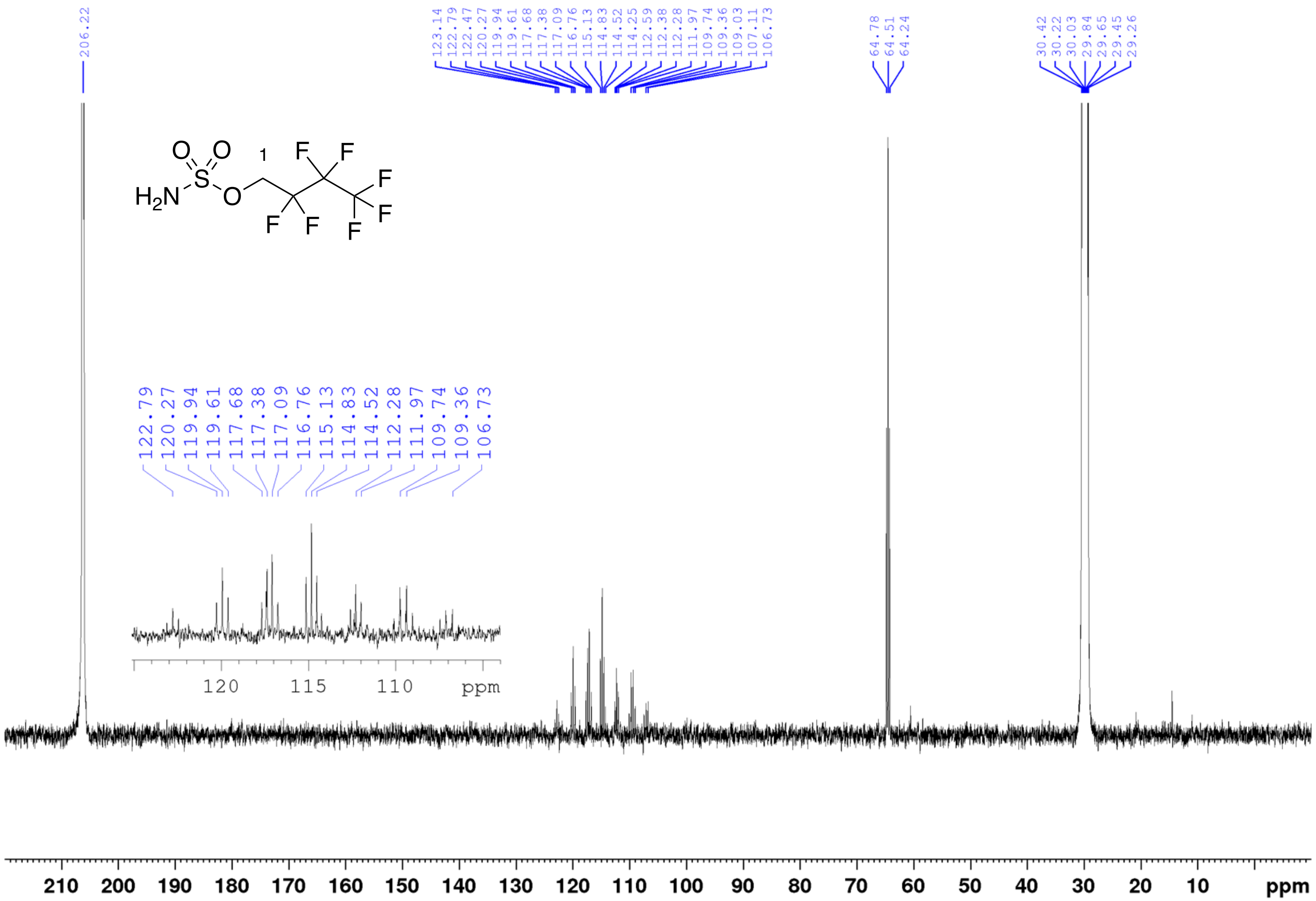

^{19}F NMR (376 MHz, $(CD_3)_2CO$) for *2,2,3,3,4,4,4-heptafluorobutyl sulfamate* (**5**)

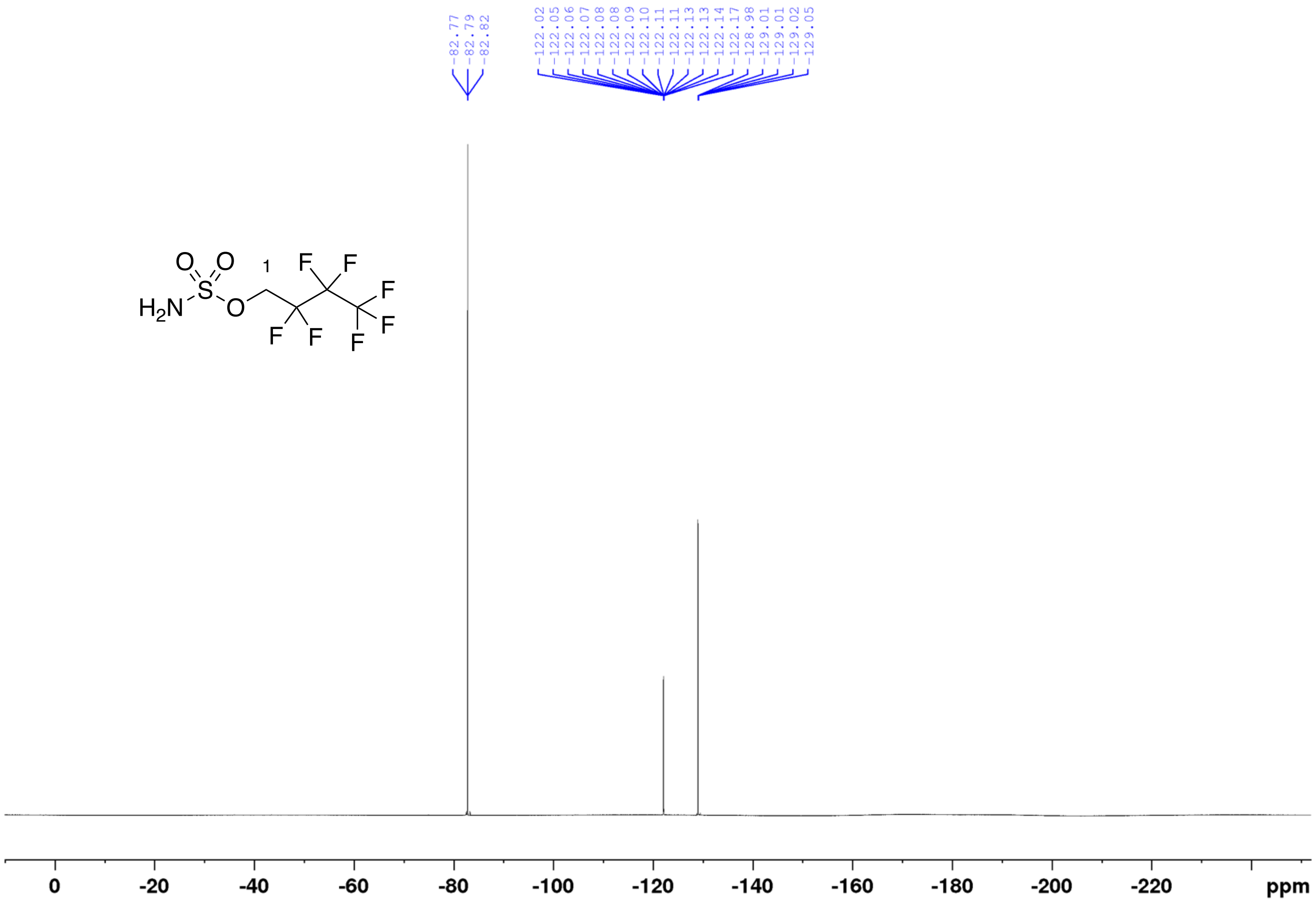

^{1}H NMR (400 MHz, $CDCl_3$) for *ethyl 3-(4-hydroxybut-1-yn-1-yl)benzoate*

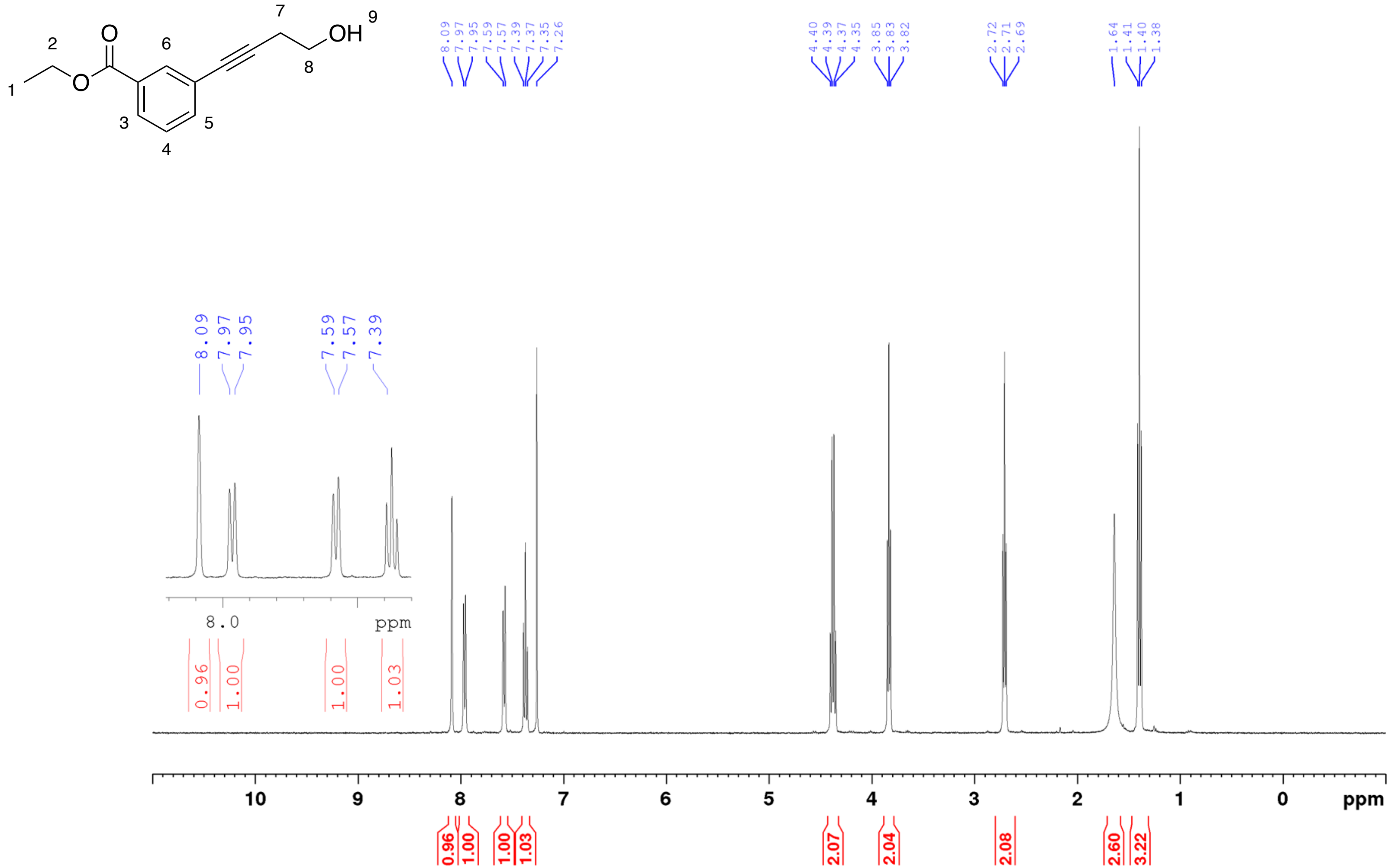

^{13}C NMR (101 MHz, $CDCl_3$) for *ethyl 3-(4-hydroxybut-1-yn-1-yl)benzoate*

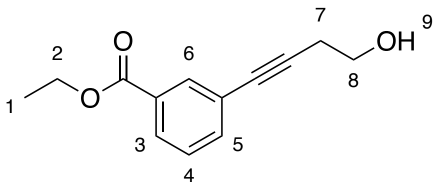

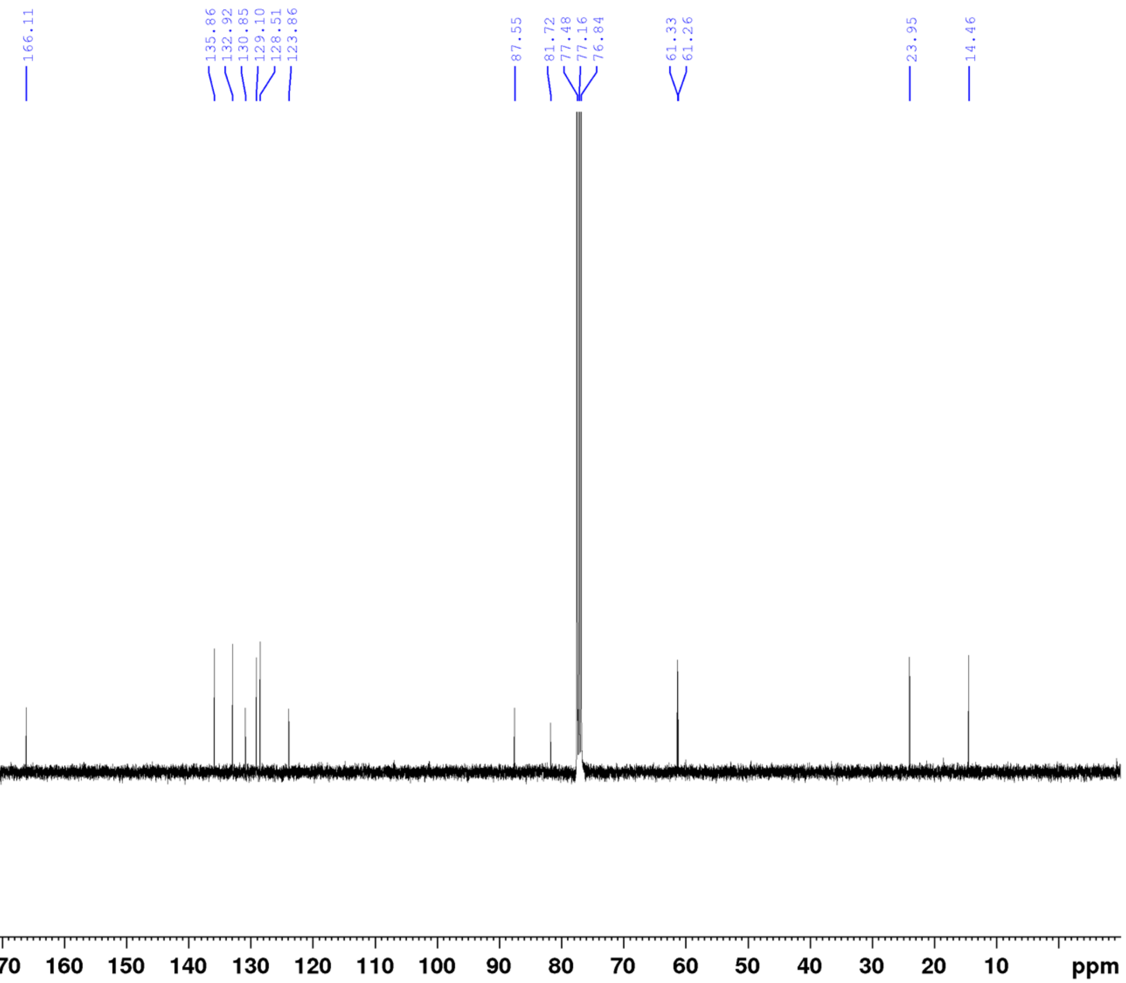

¹H NMR (400 MHz, $CDCl_3$) for *ethyl 3-(4-hydroxybutyl)benzoate* (**6b**)

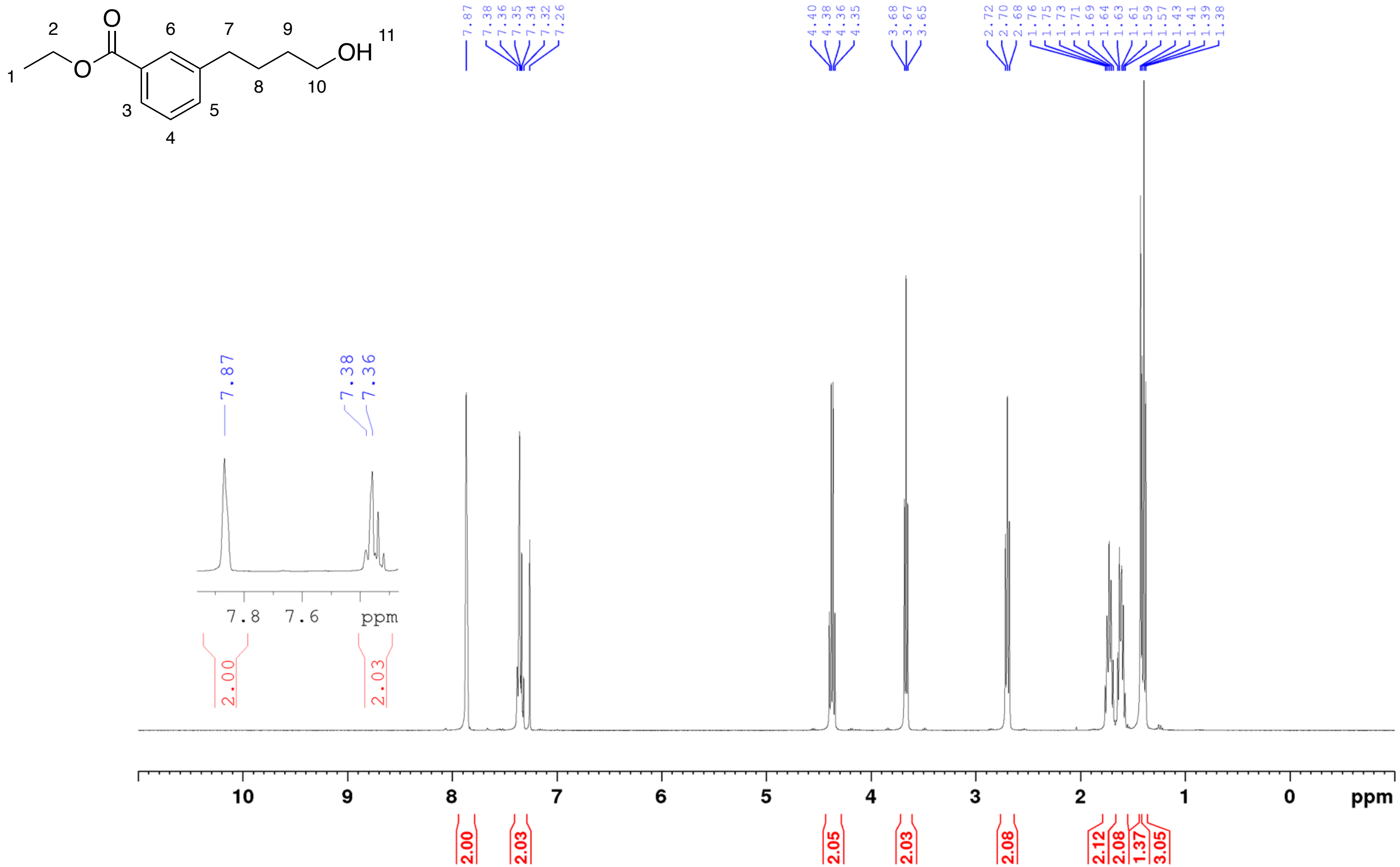

^{13}C NMR (101 MHz, $CDCl_3$) for *ethyl 3-(4-hydroxybutyl)benzoate* (**6b**)

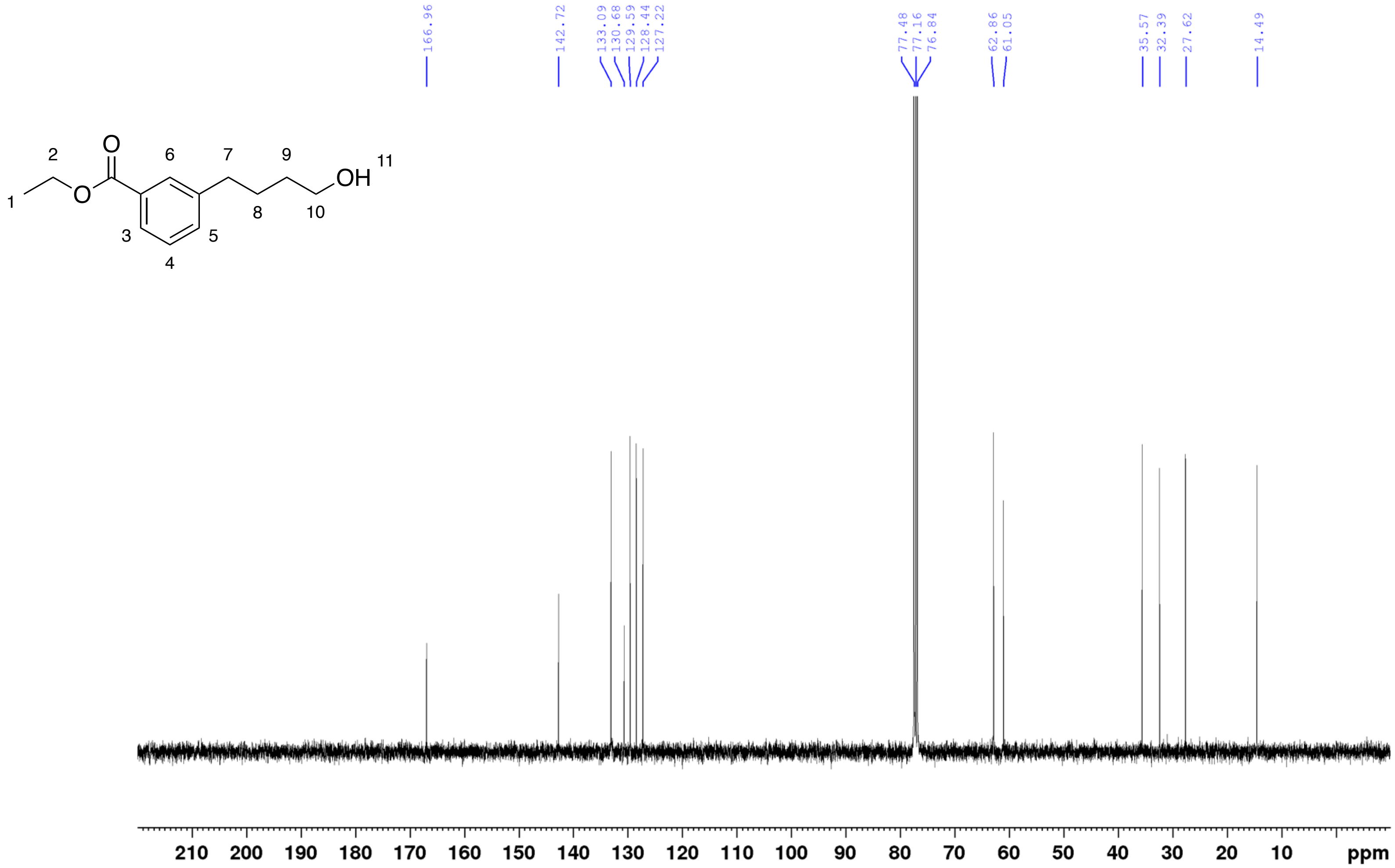

^{1}H NMR (400 MHz, $CDCl_3$) for *4-(o-tolyl)but-3-yn-1-ol*

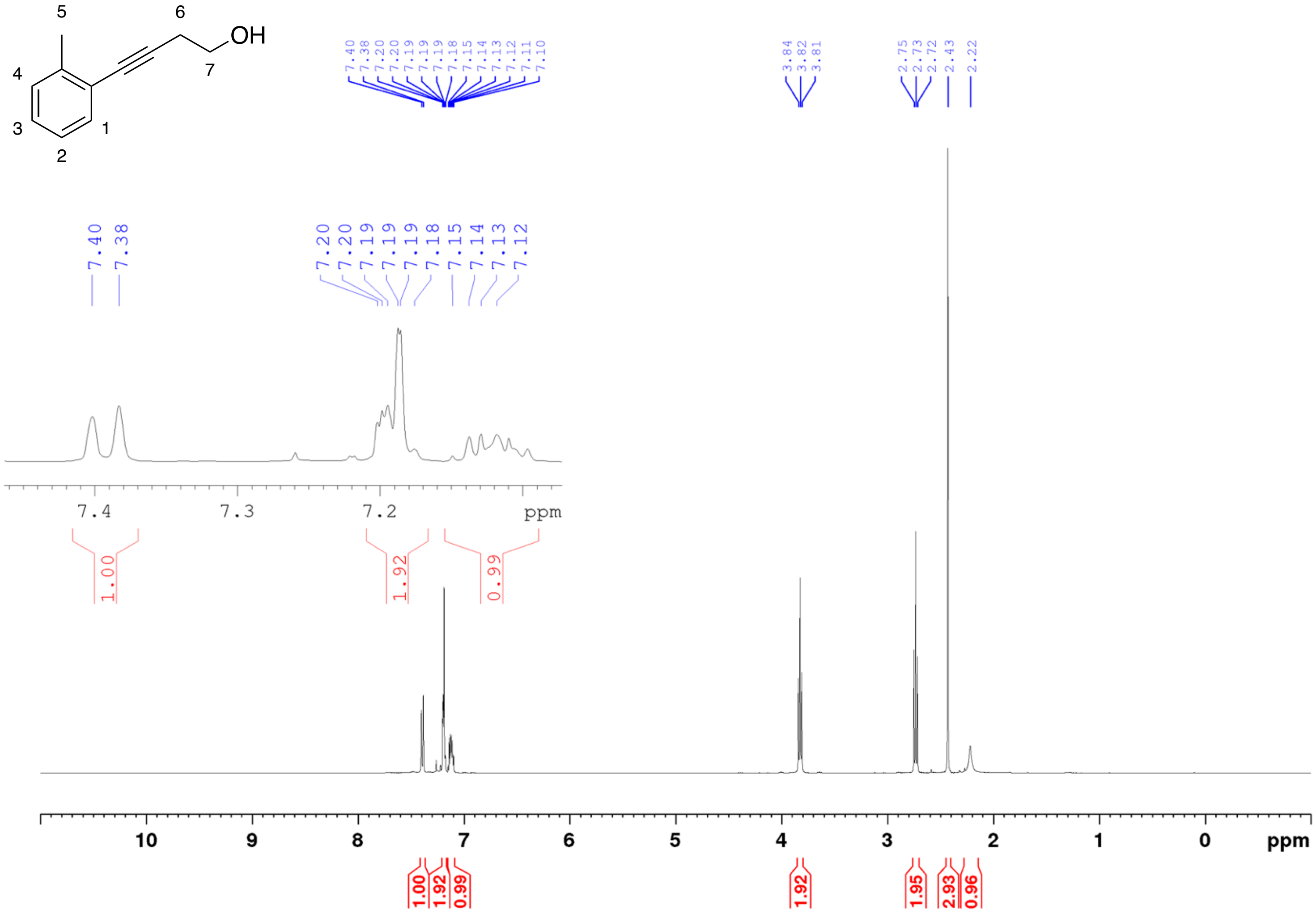

^{13}C NMR (101 MHz, $CDCl_3$) for *4-(o-tolyl)but-3-yn-1-ol*

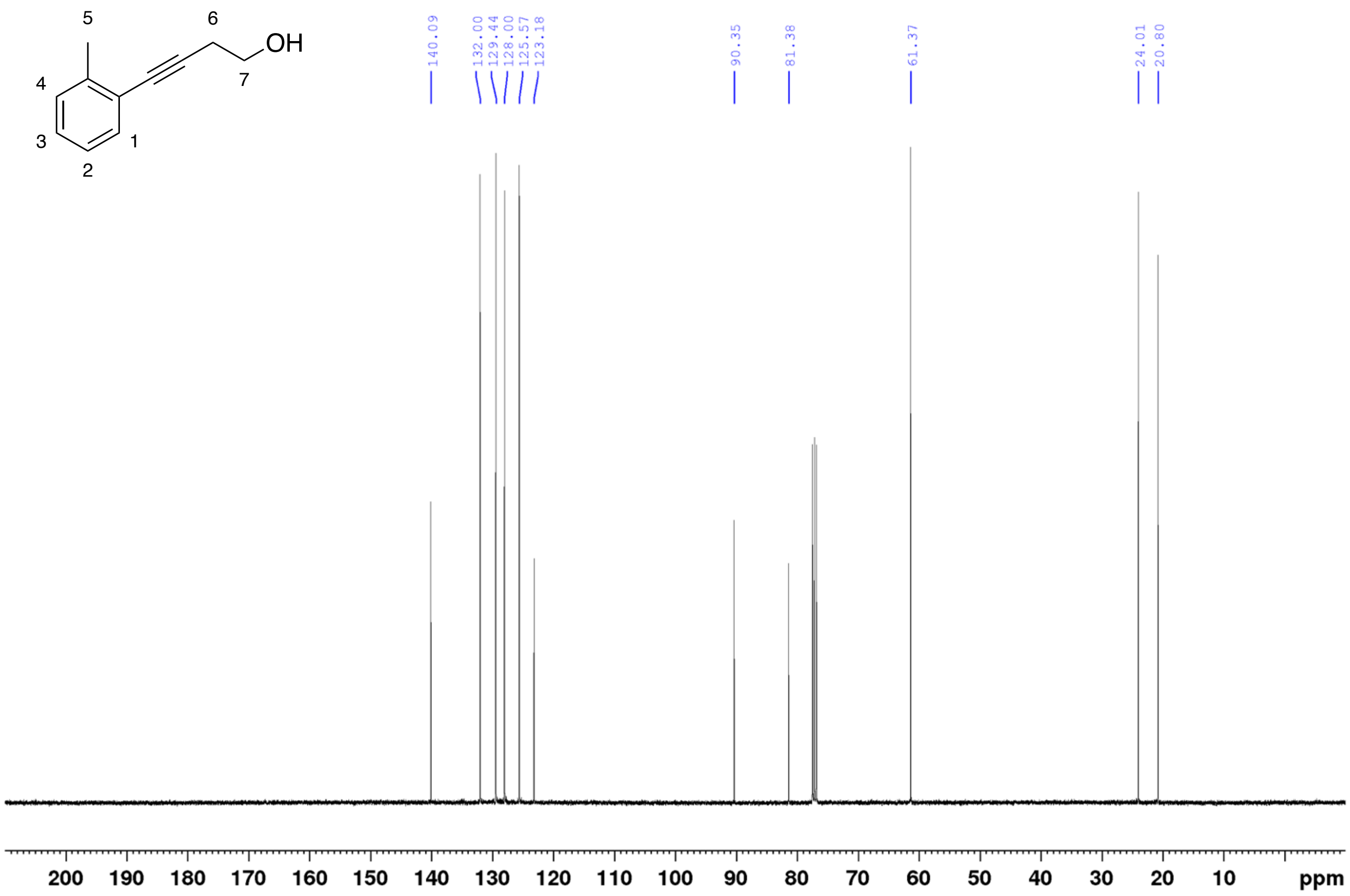

^{1}H NMR (400 MHz, $CDCl_3$) for *4-(o-tolyl)butan-1-ol* (**6c**)

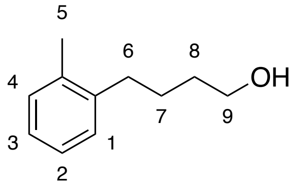

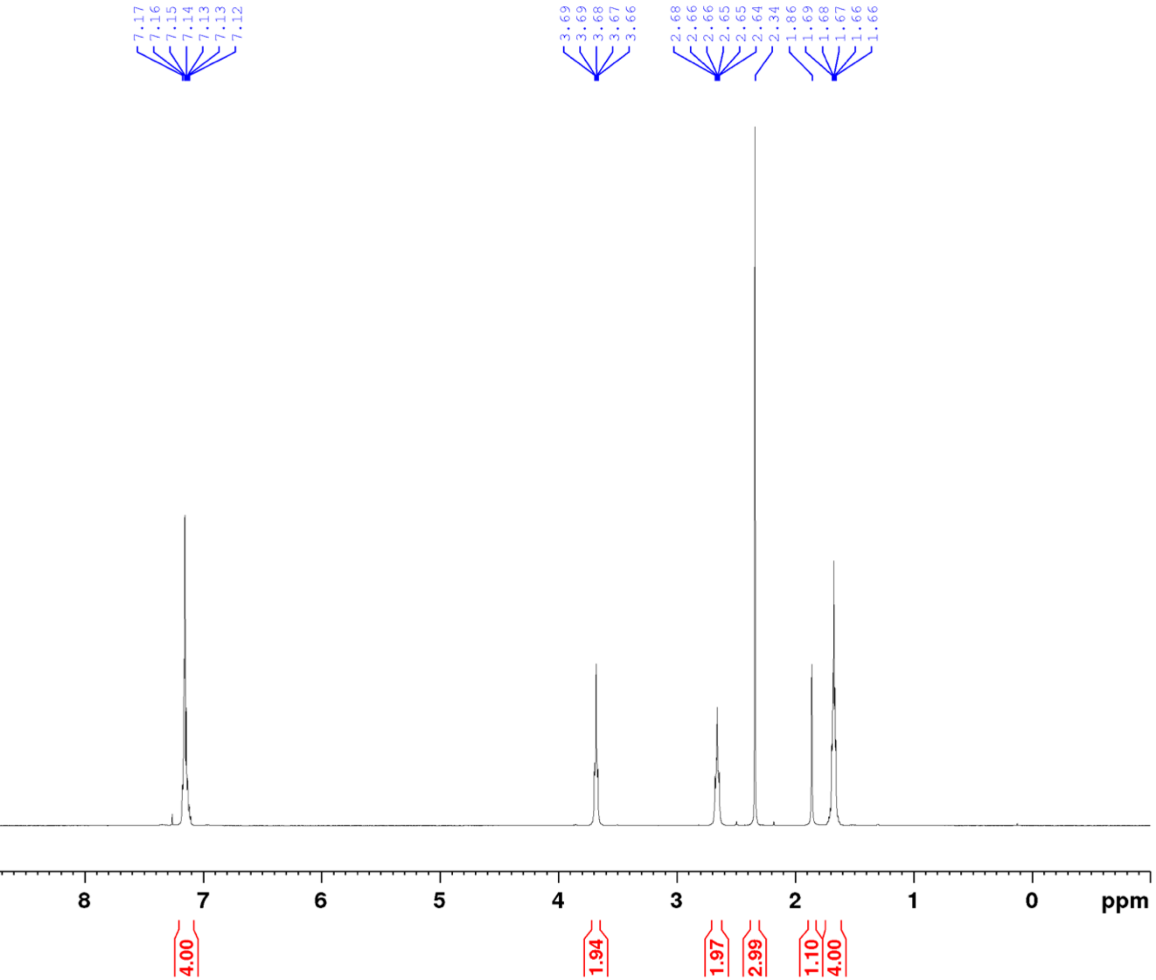

^{13}C NMR (101 MHz, $CDCl_3$) for *4-(o-tolyl)butan-1-ol* (**6c**)

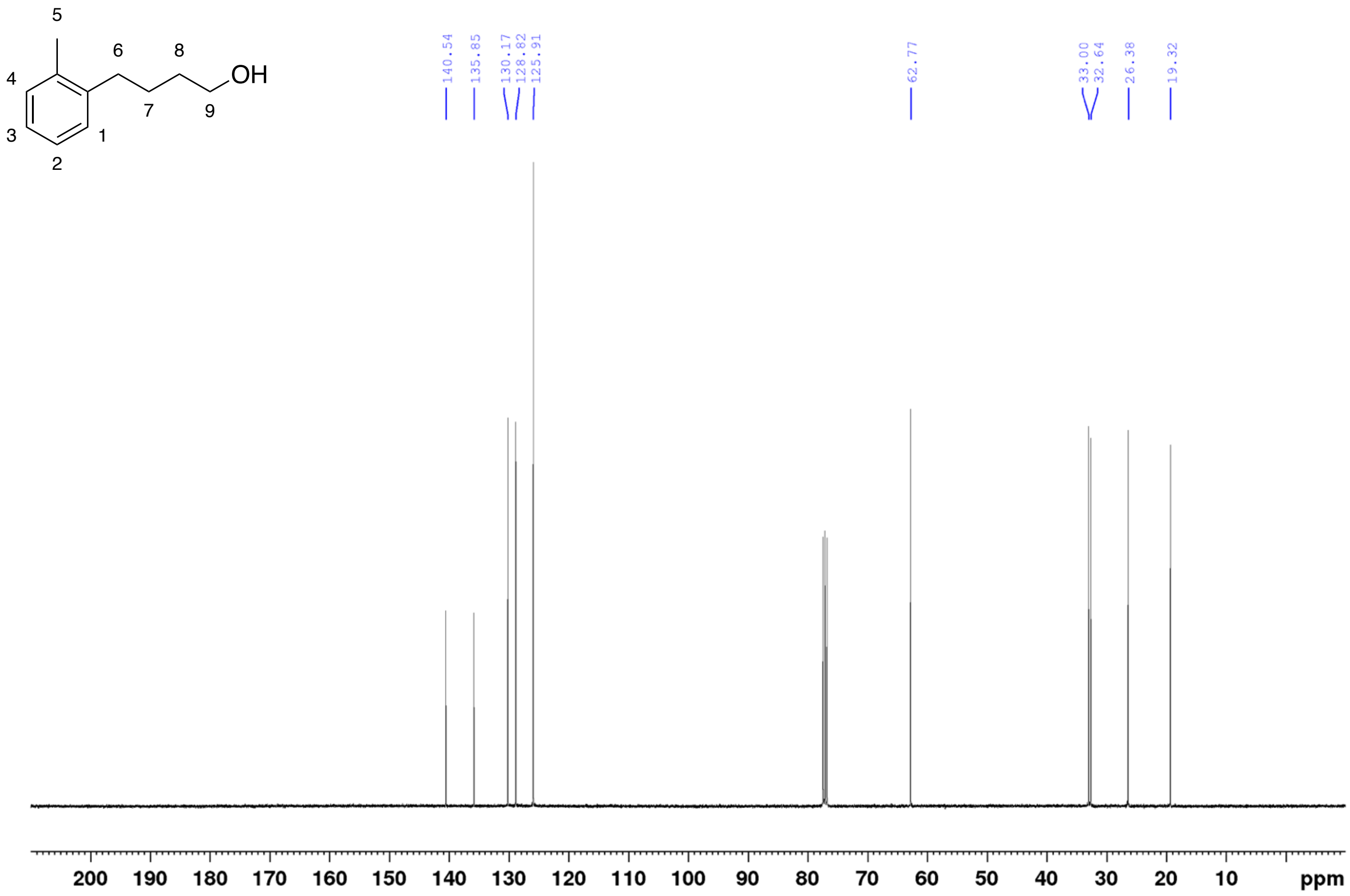

^{1}H NMR (400 MHz, $CDCl_3$) for *4-(m-tolyl)but-3-yn-1-ol*

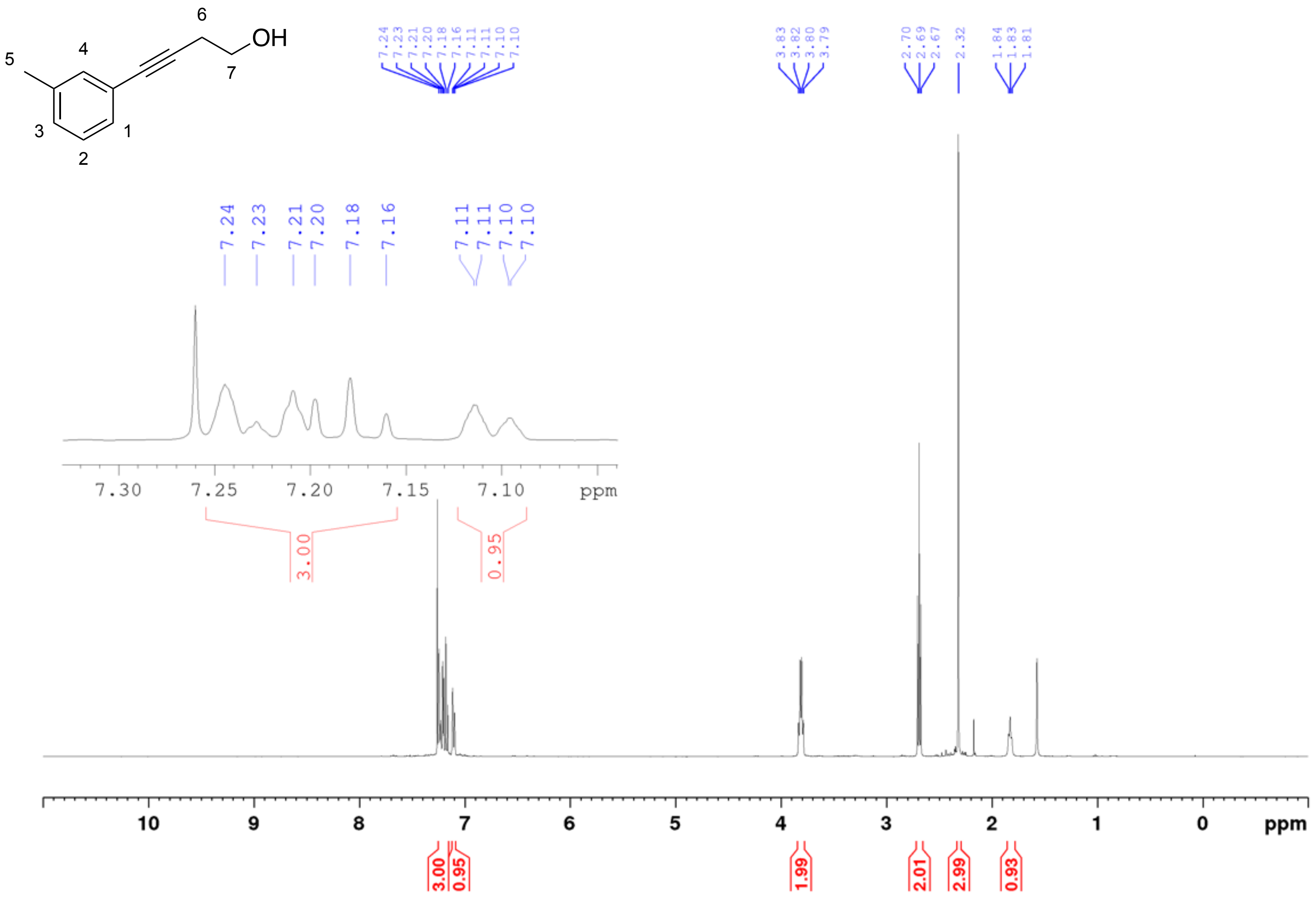

^{13}**C NMR** (101 MHz, $CDCl_3$) for *4-(m-tolyl)but-3-yn-1-ol*

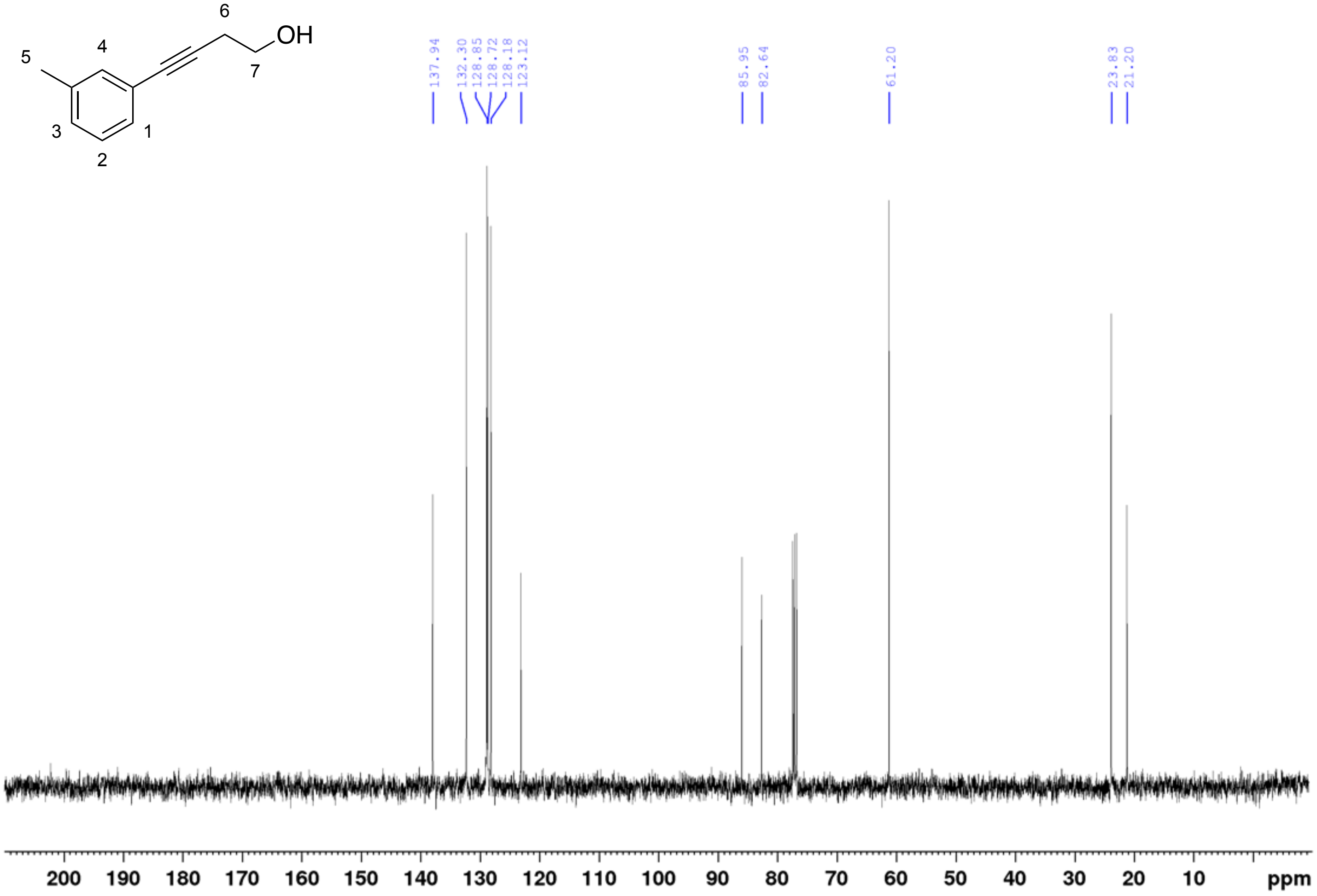

^{1}H NMR (400 MHz, $CDCl_3$) for *4-(m-tolyl)butan-1-ol* (**6d**)

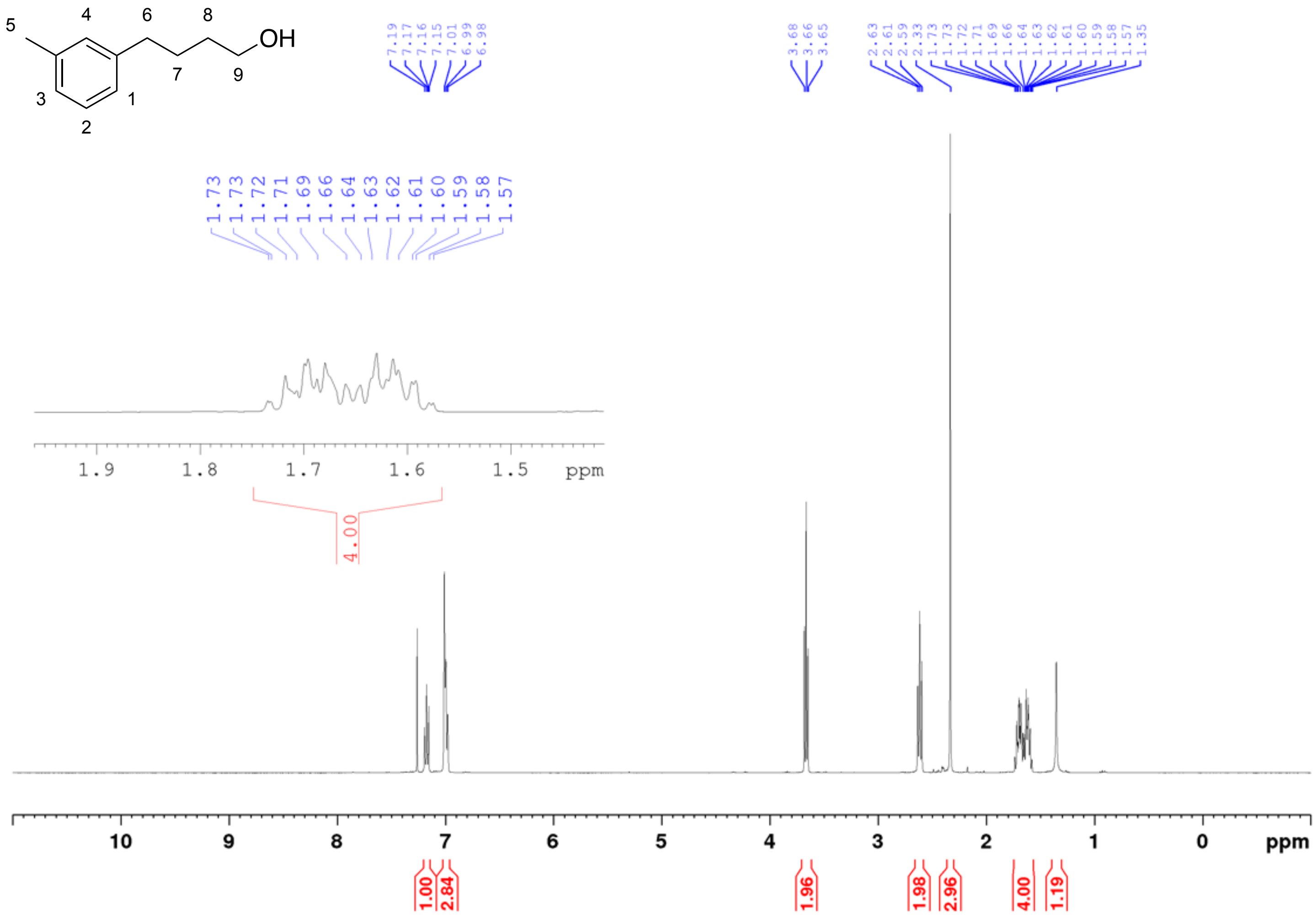

^{13}C NMR (101 MHz, $CDCl_3$) for *4-(m-tolyl)butan-1-ol* (**6d**)

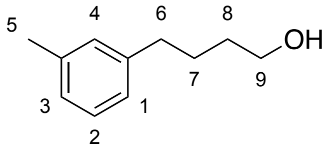

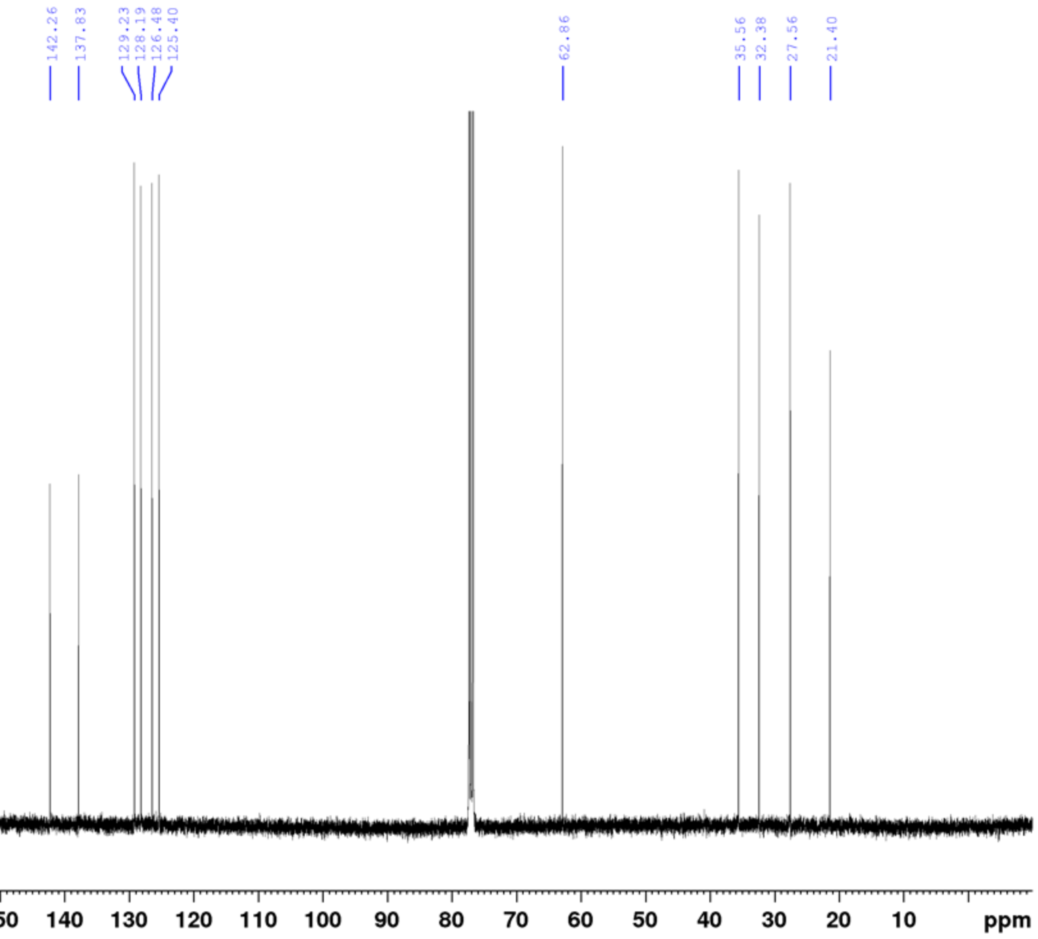

^{1}H NMR (400 MHz, $CDCl_3$) for *4-(3-fluorophenyl)but-3-yn-1-ol*

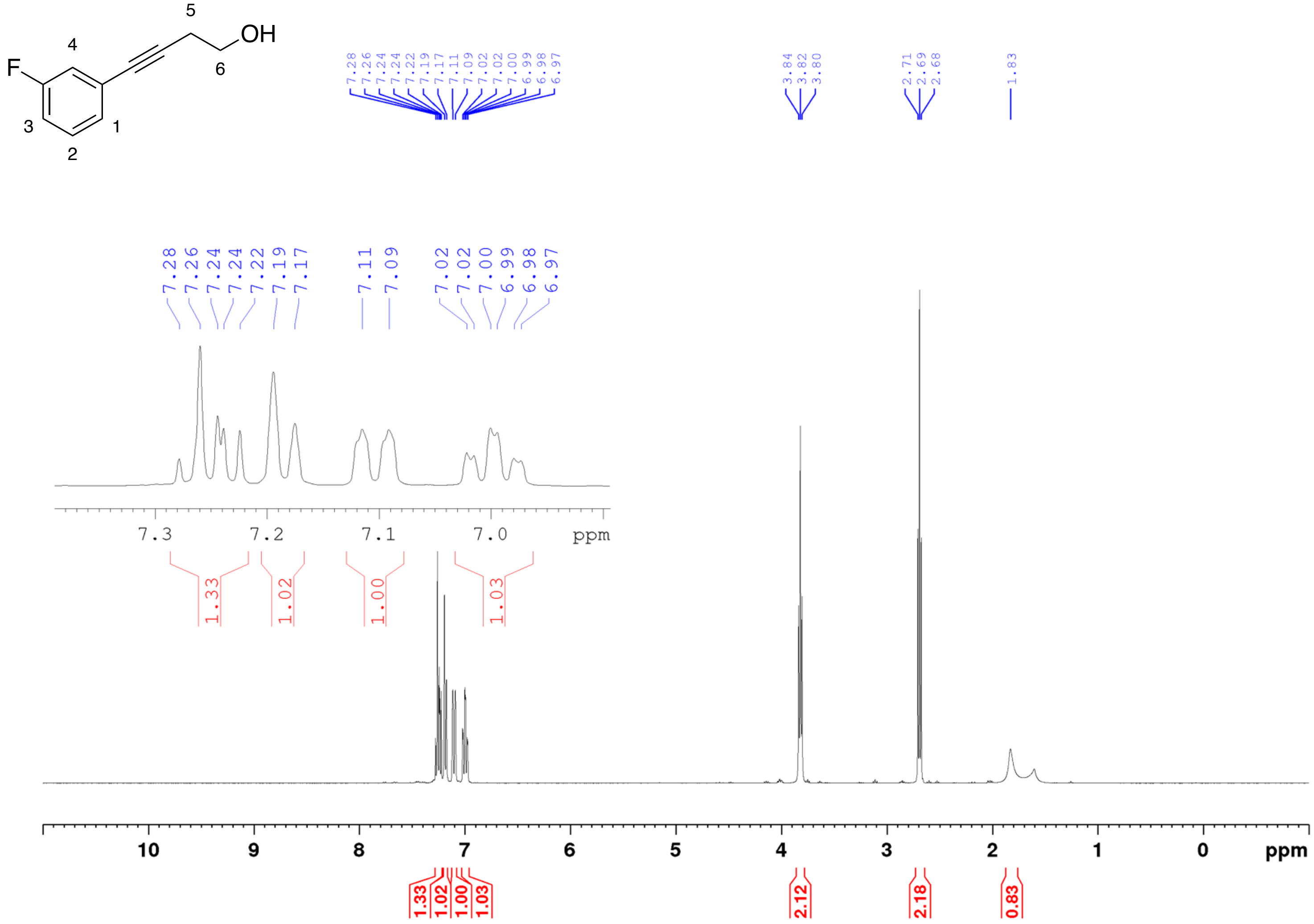

^{13}C NMR (101 MHz, $CDCl_3$) for *4-(3-fluorophenyl)but-3-yn-1-ol*

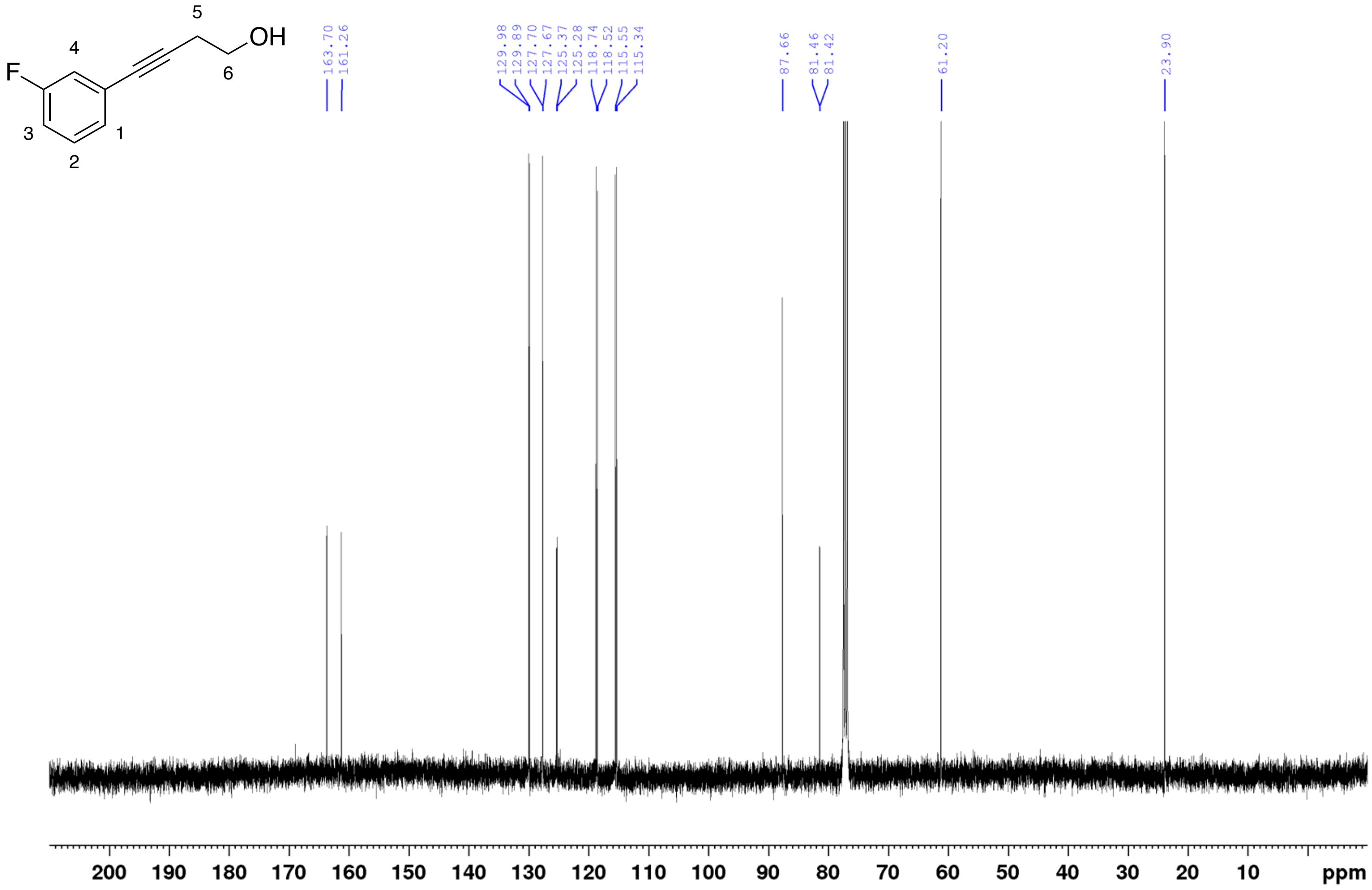

^{19}F NMR (376 MHz, $CDCl_3$) for *4-(3-fluorophenyl)but-3-yn-1-ol*

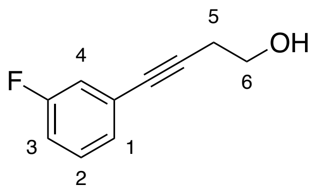

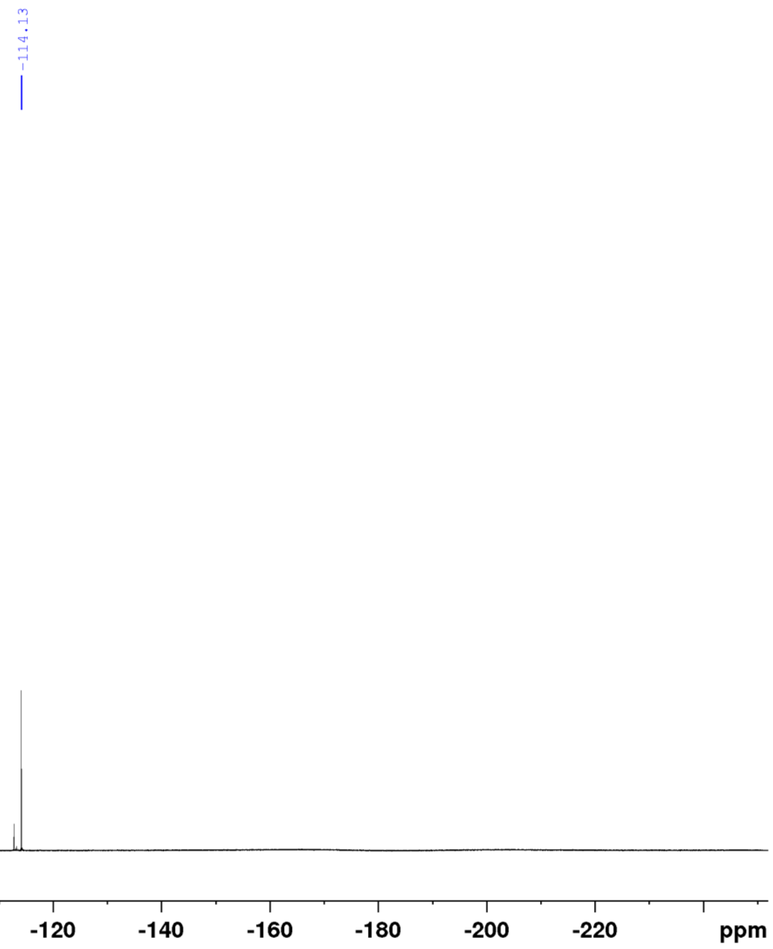

^{1}H NMR (400 MHz, $CDCl_3$) for *4-(3-fluorophenyl)butan-1-ol* (**6e**)

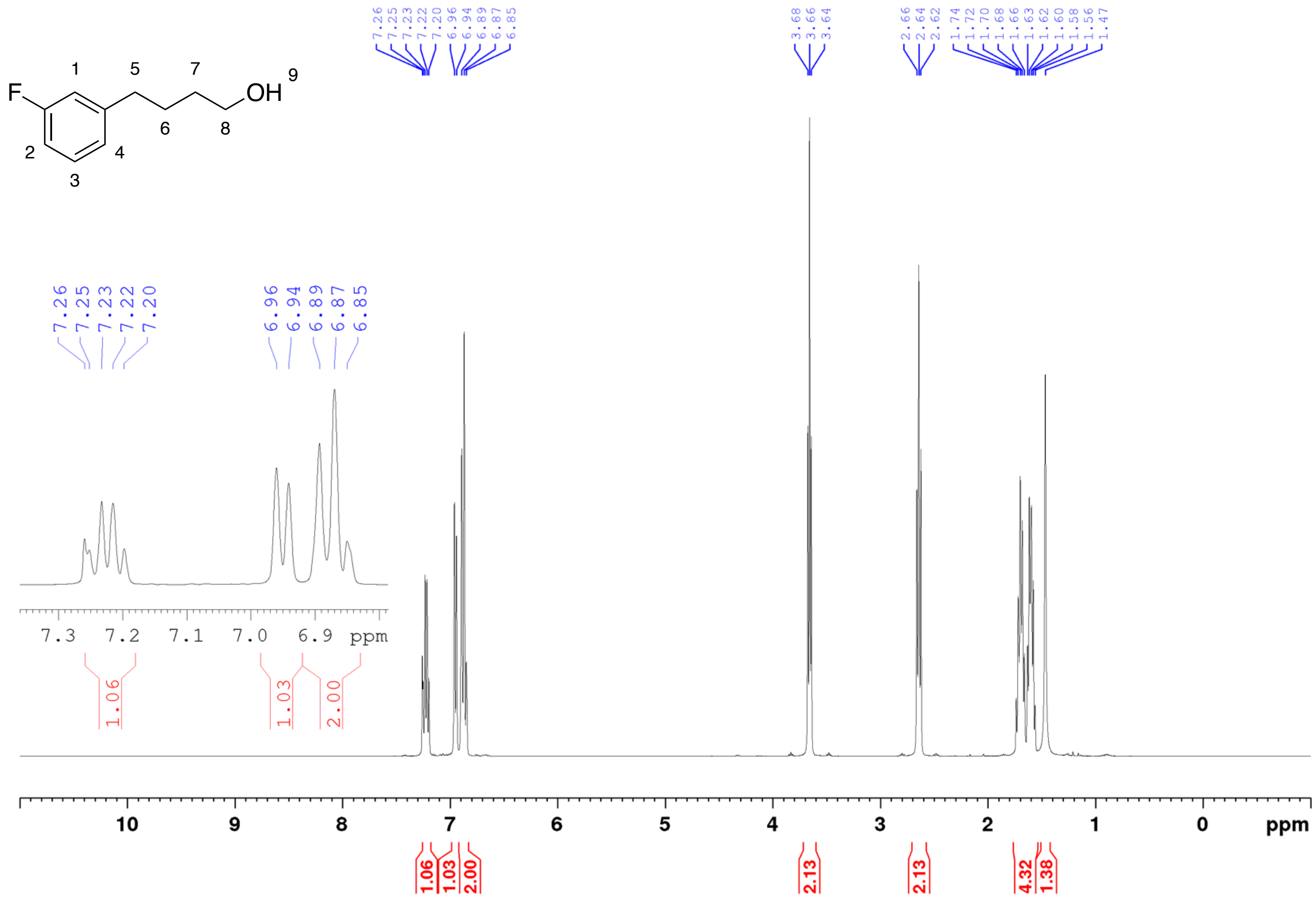

^{13}C NMR (101 MHz, $CDCl_3$) for *4-(3-fluorophenyl)butan-1-ol* (**6e**)

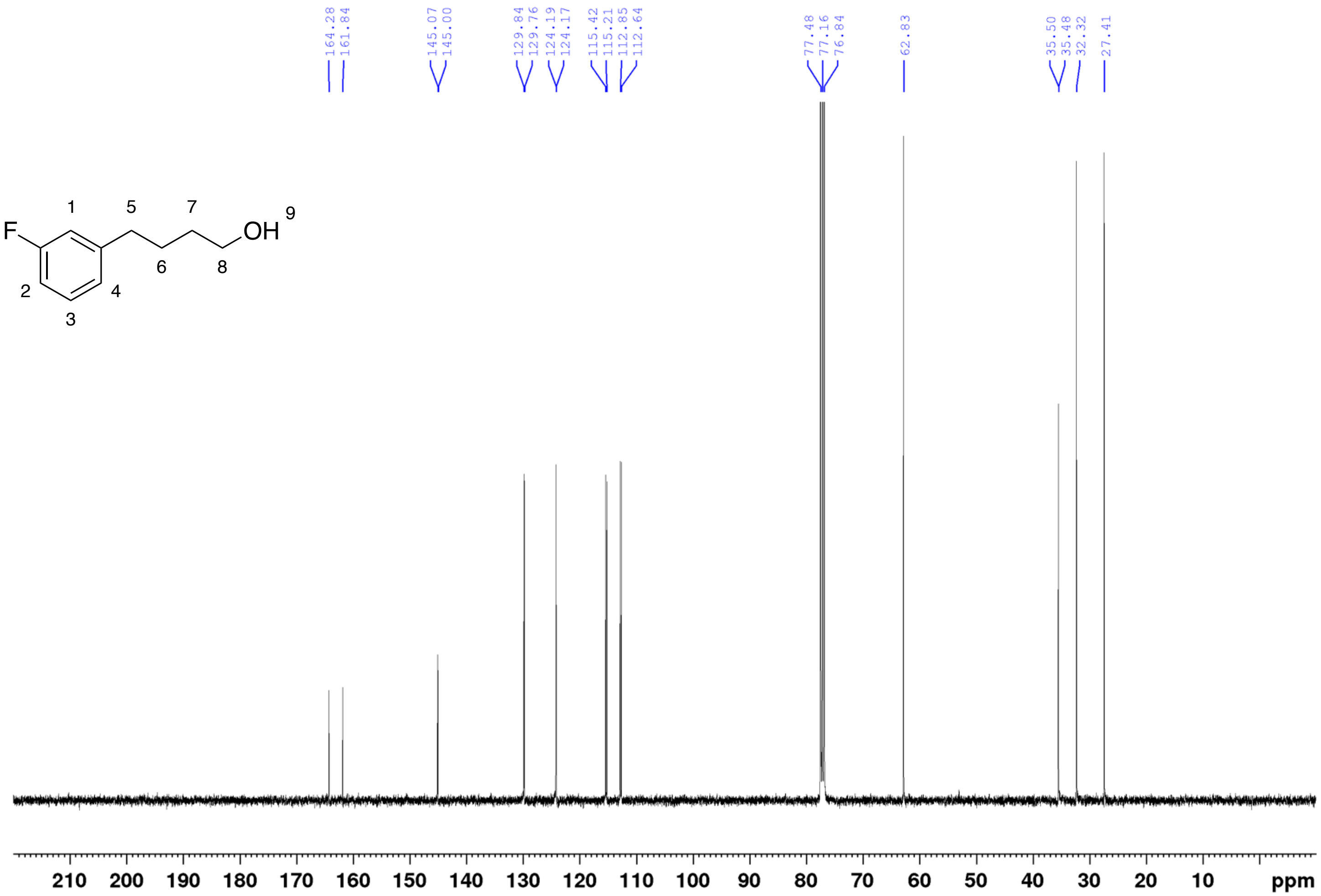

^{19}**F NMR** (376 MHz, $CDCl_3$) for *4-(3-fluorophenyl)butan-1-ol* (**6e**)

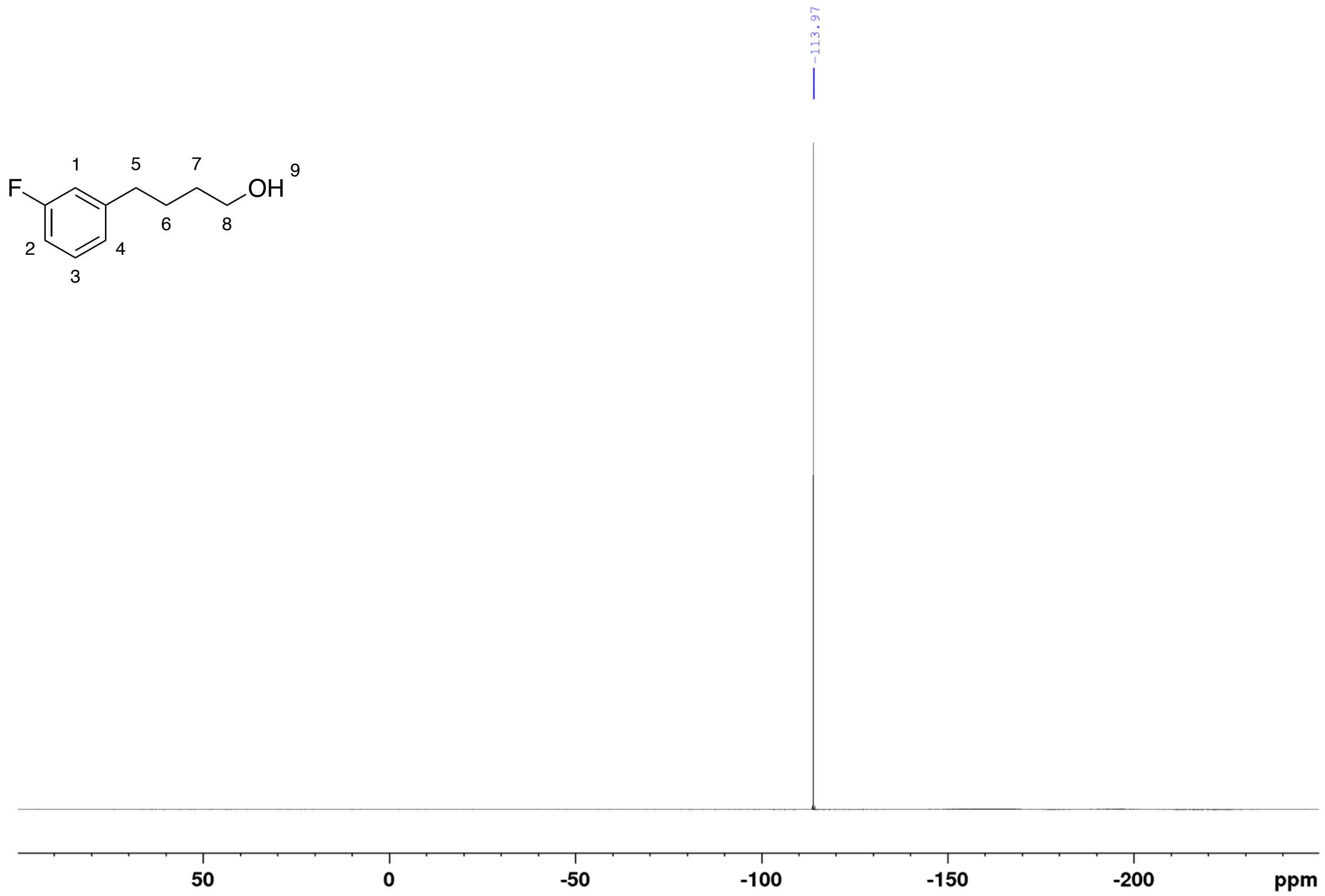

^{1}H NMR (400 MHz, $CDCl_3$) for *4-(2-fluorophenyl)but-3-yn-1-ol*

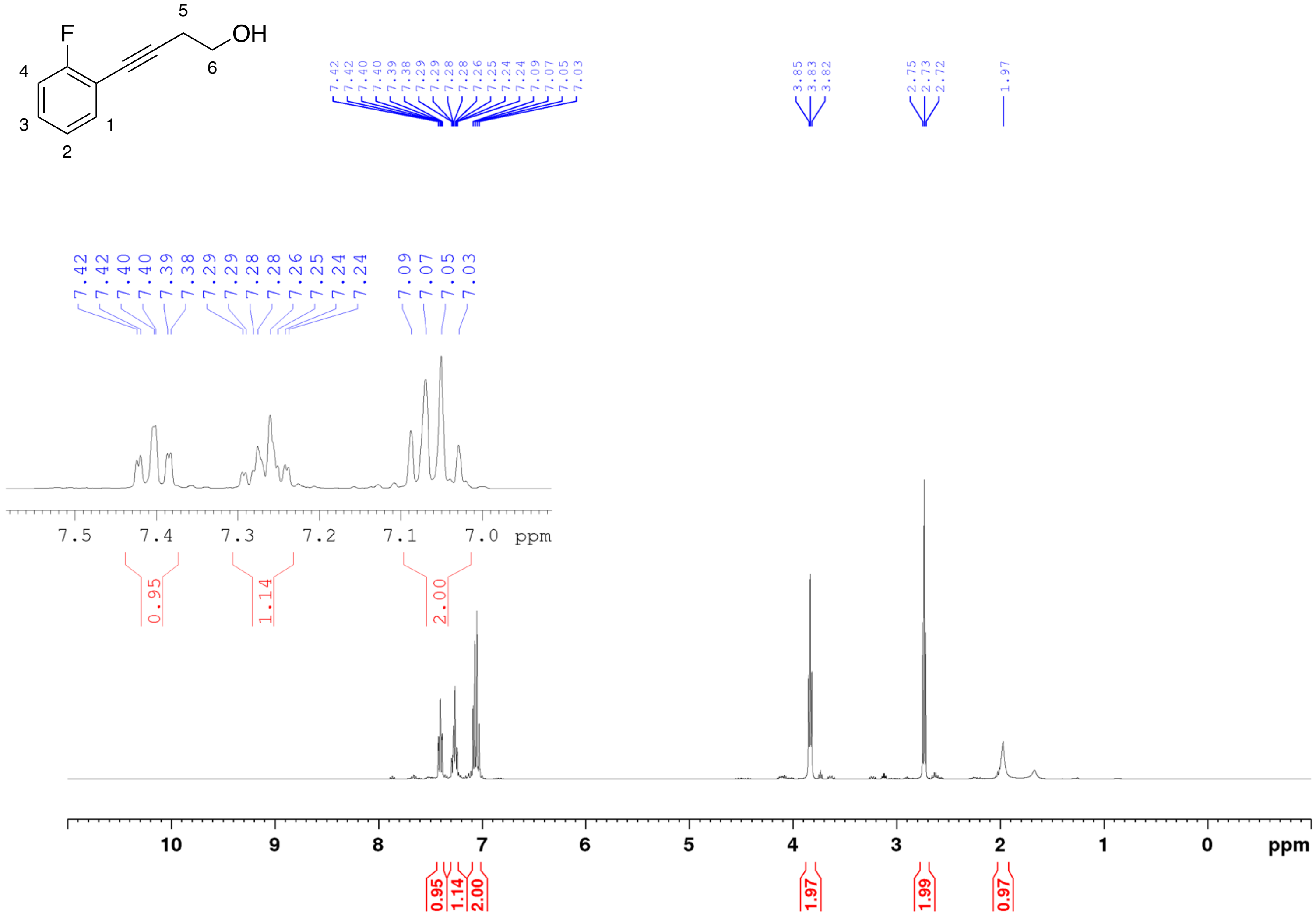

^{13}C NMR (101 MHz, $CDCl_3$) for *4-(2-fluorophenyl)but-3-yn-1-ol*

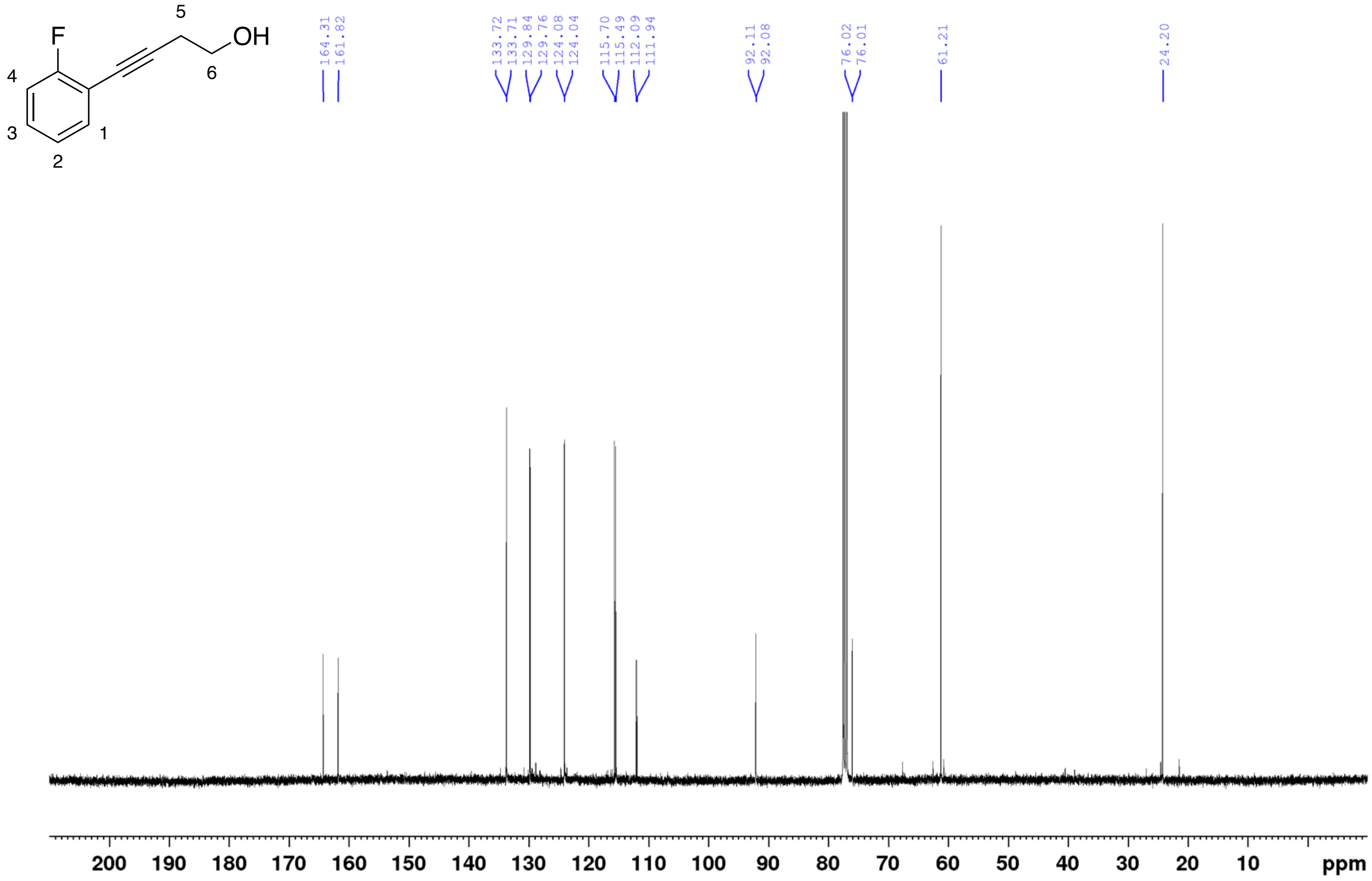

^{19}F NMR (376 MHz, $CDCl_3$) for *4-(2-fluorophenyl)but-3-yn-1-ol*

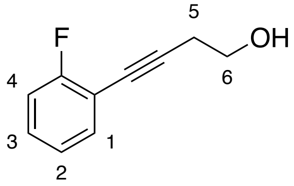

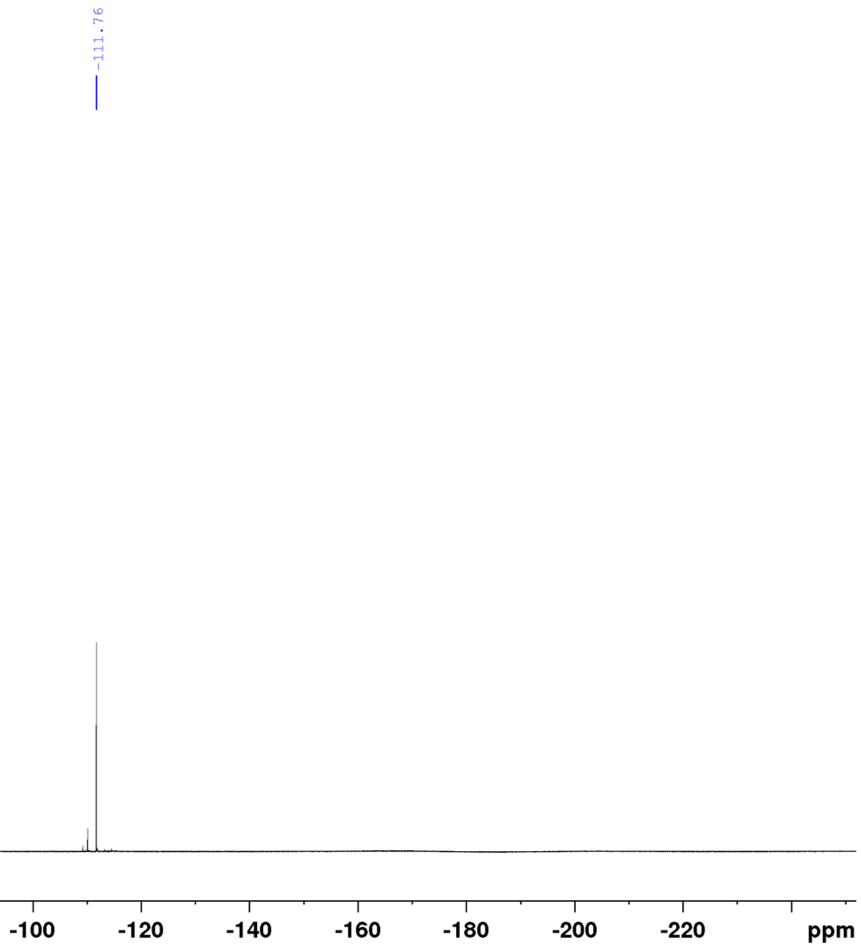

^{1}H NMR (400 MHz, $CDCl_3$) for *4-(2-fluorophenyl)butan-1-ol* (**6f**)

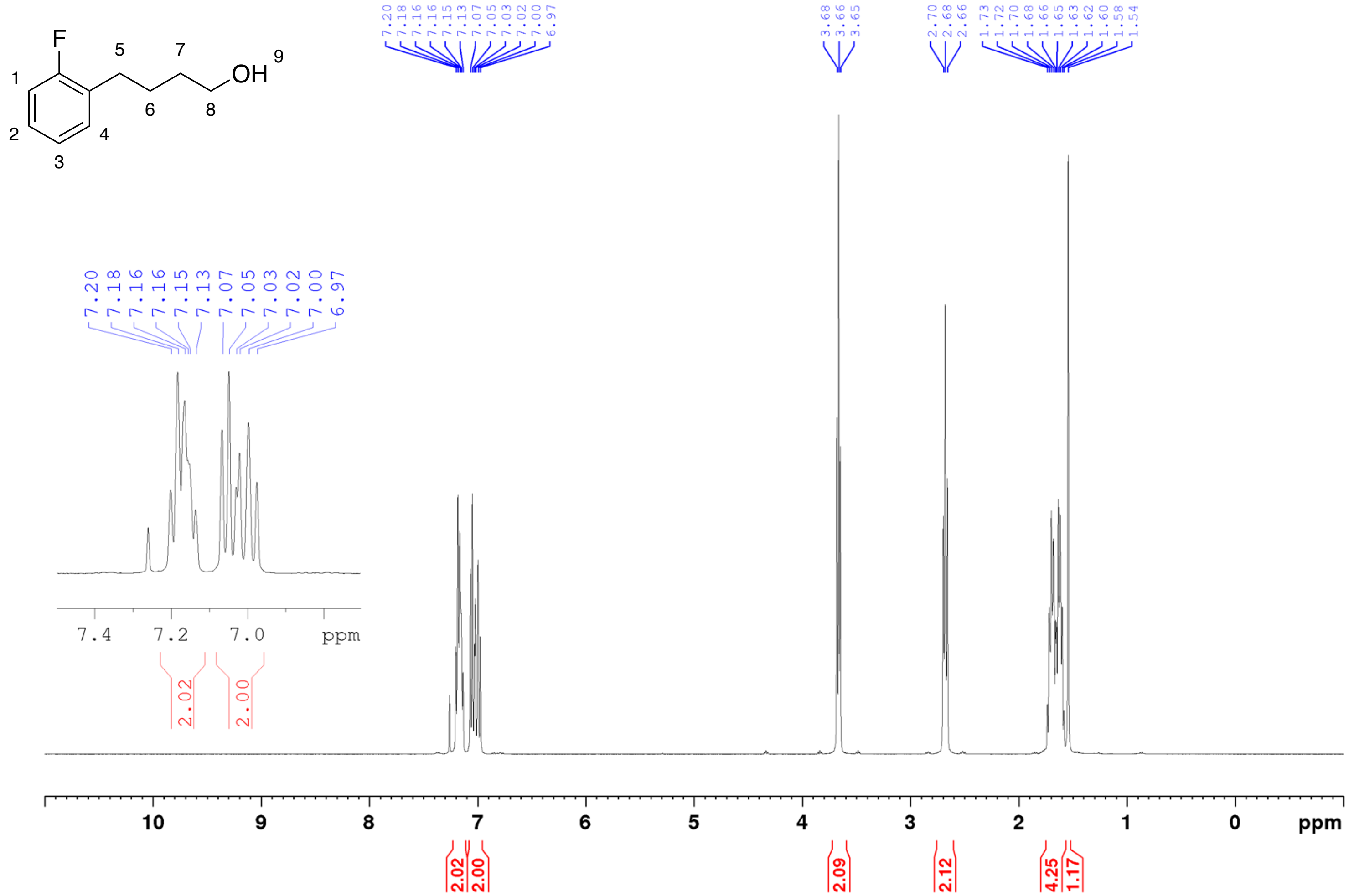

^{13}C NMR (101 MHz, $CDCl_3$) for *4-(2-fluorophenyl)butan-1-ol* (**6f**)

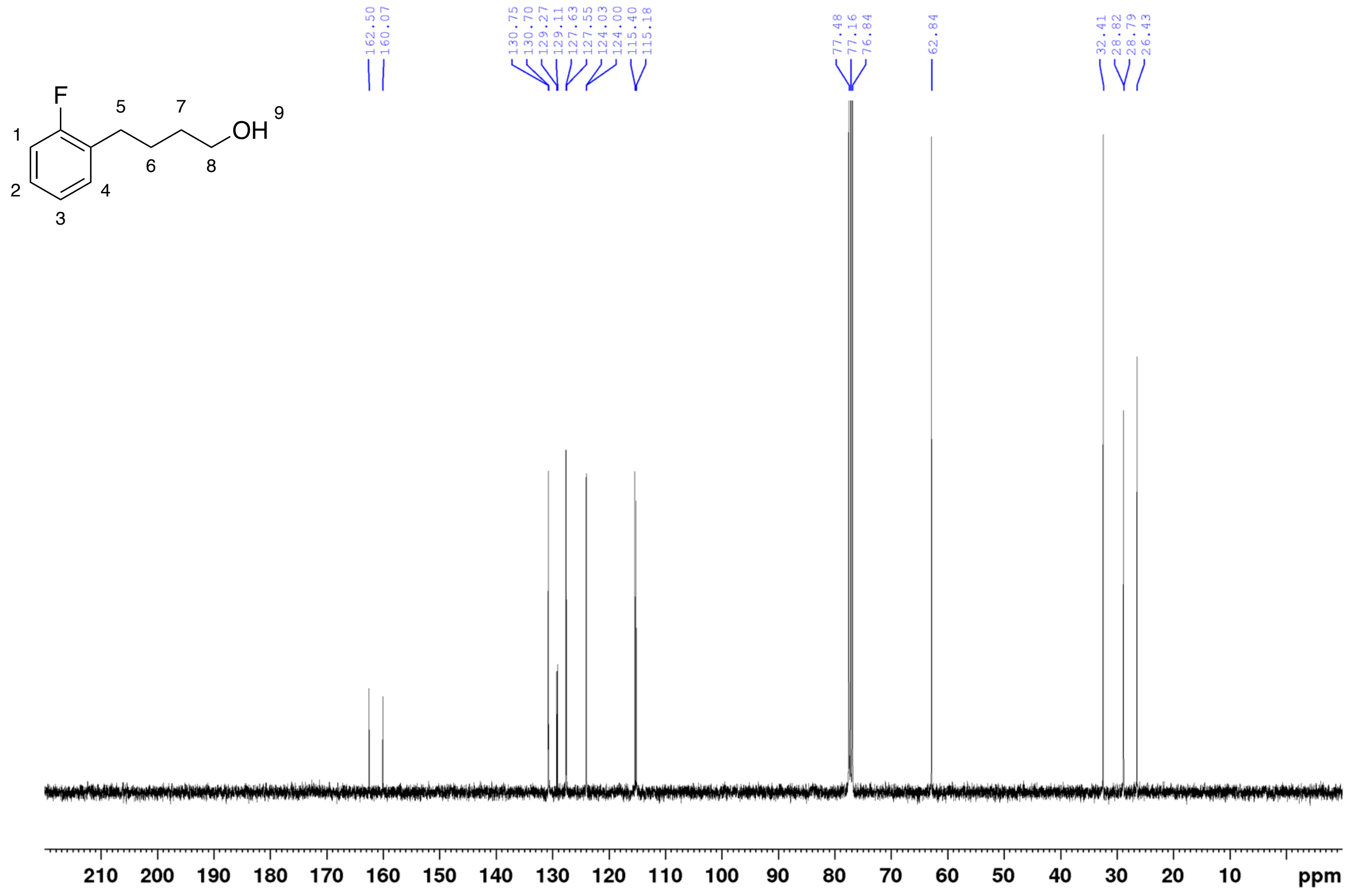

^{19}F NMR (376 MHz, $CDCl_3$) for *4-(2-fluorophenyl)butan-1-ol* (**6f**)

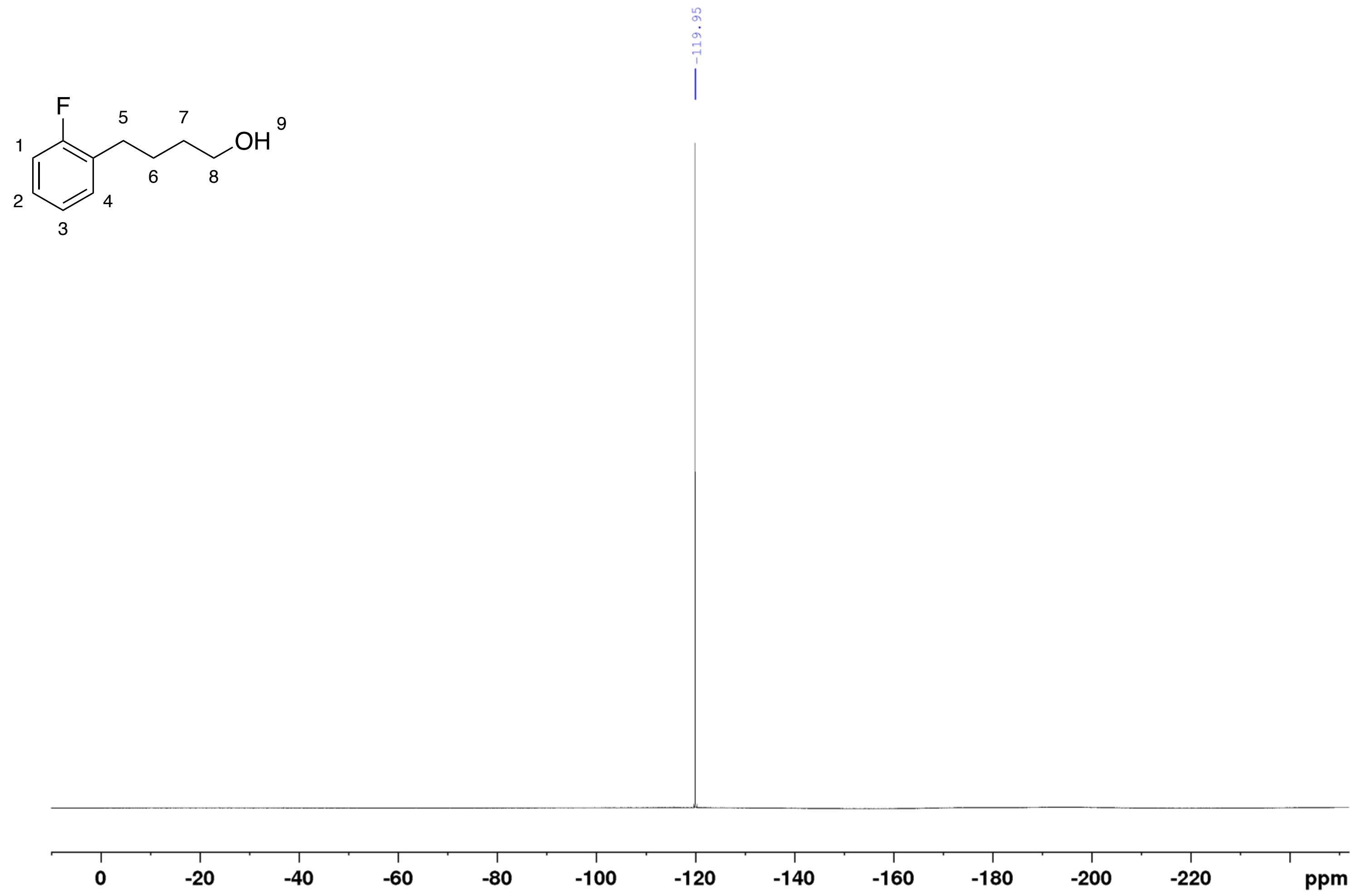

^{1}H NMR (400 MHz, $CDCl_3$) for *4-(4-fluorophenyl)but-3-yn-1-ol*

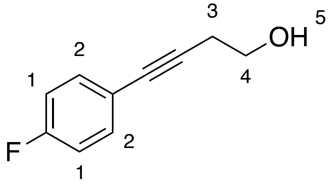

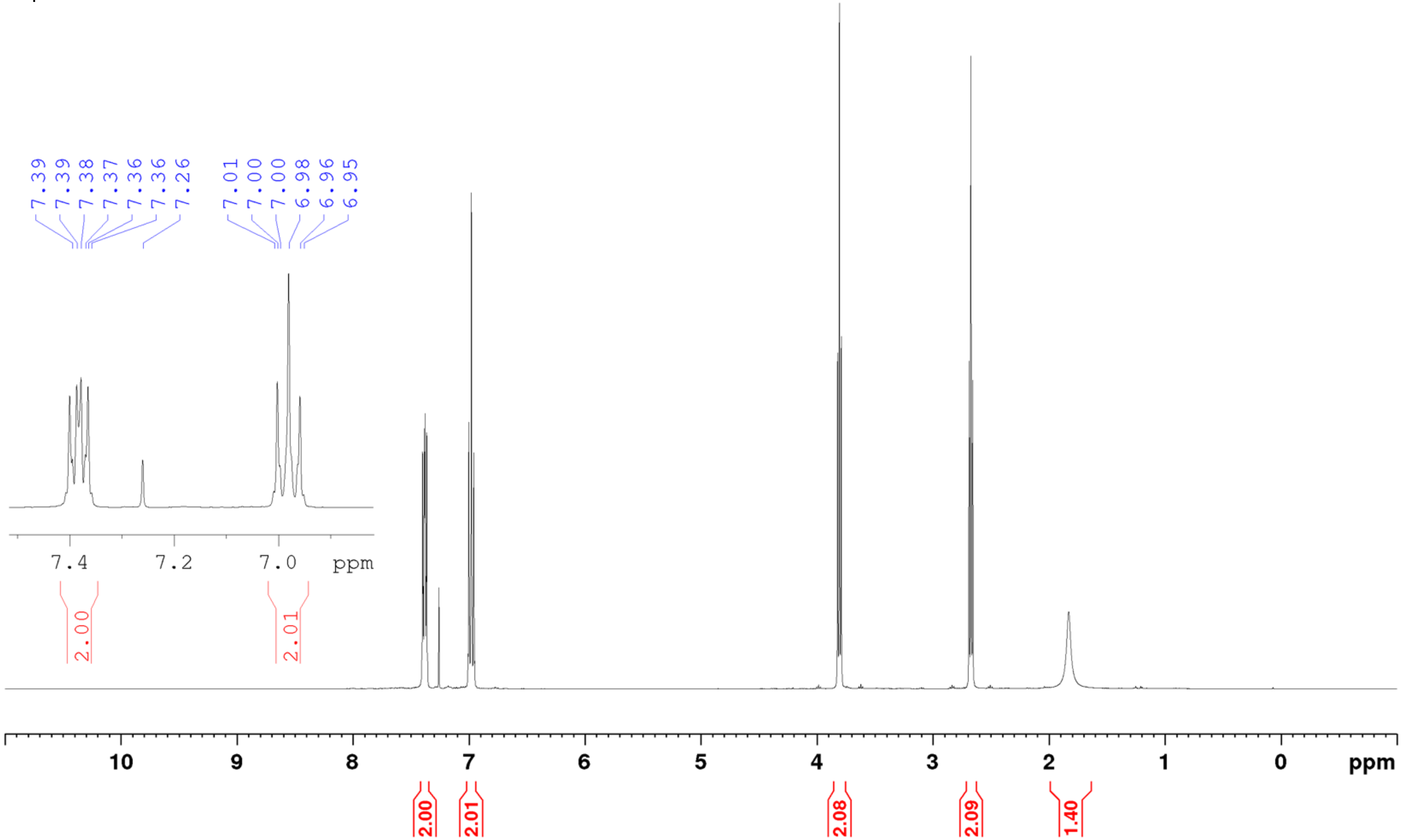

^{13}C NMR (101 MHz, $CDCl_3$) for *4-(4-fluorophenyl)but-3-yn-1-ol*

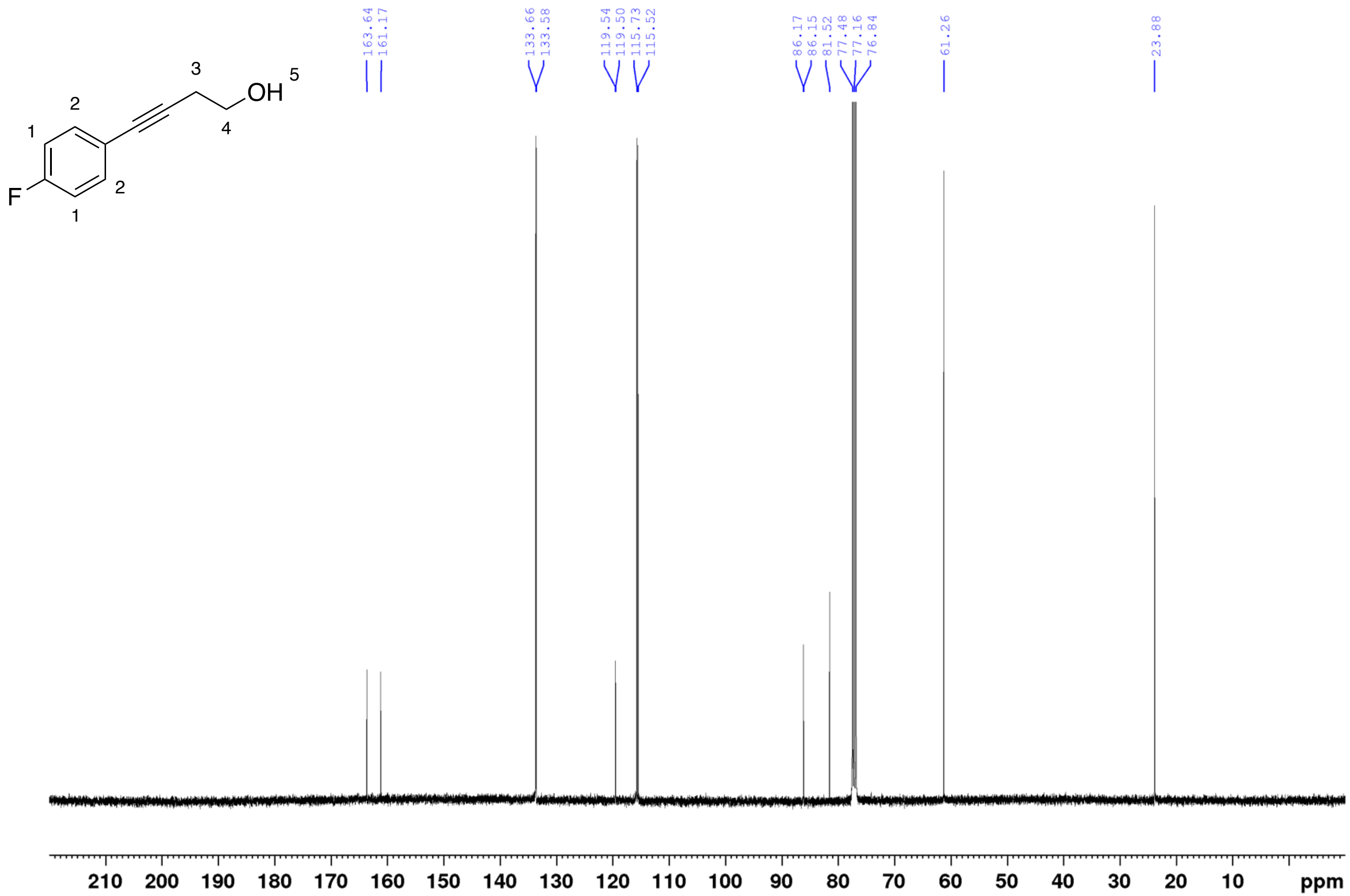

^{19}F NMR (376 MHz, $CDCl_3$) for *4-(4-fluorophenyl)but-3-yn-1-ol*

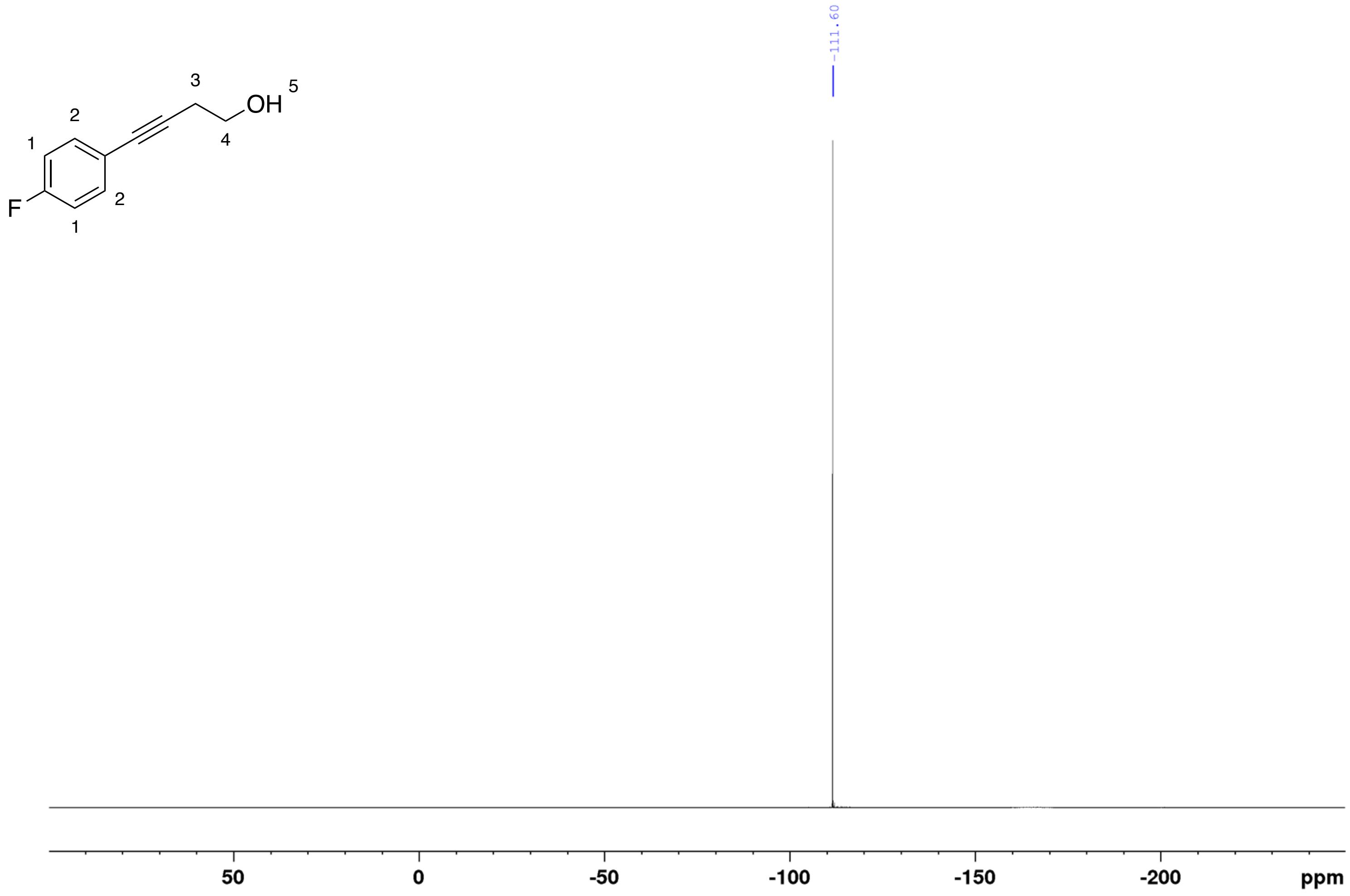

^{1}**H NMR** (400 MHz, $CDCl_3$) for *4-(4-fluorophenyl)butan-1-ol* (**6g**)

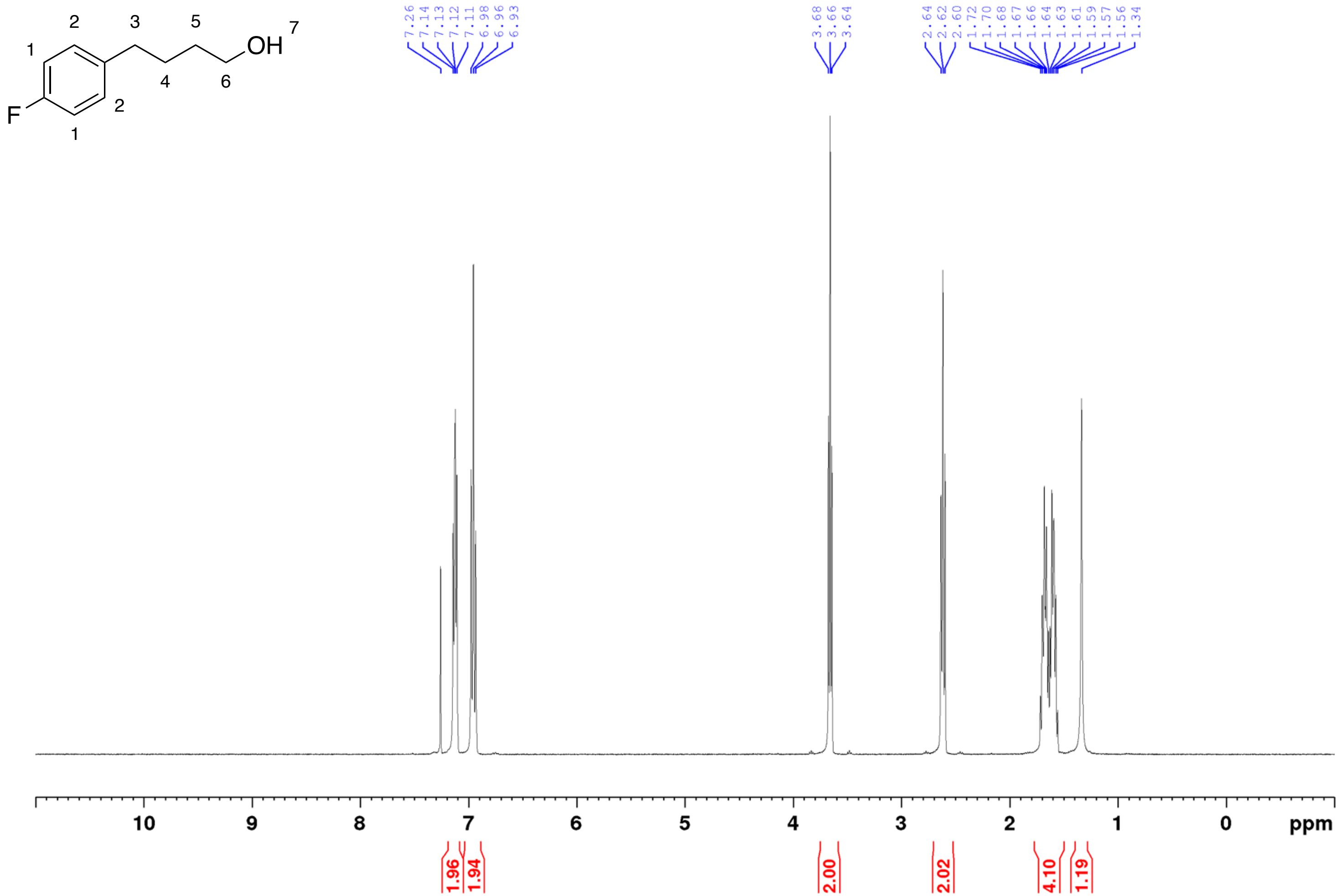

^{13}C NMR (101 MHz, $CDCl_3$) for *4-(4-fluorophenyl)butan-1-ol* (**6g**)

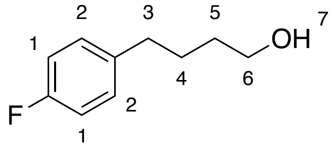

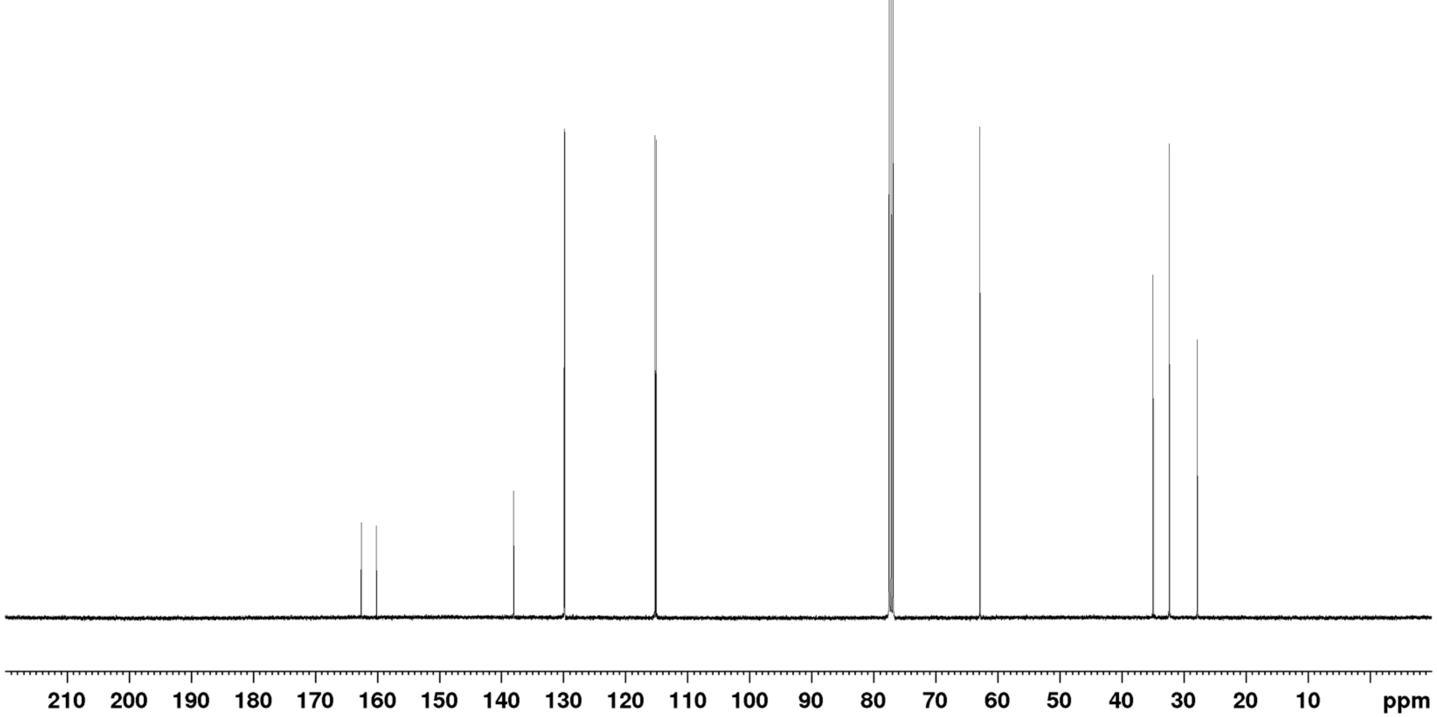

^{19}F NMR (376 MHz, $CDCl_3$) for *4-(4-fluorophenyl)butan-1-ol* (**6g**)

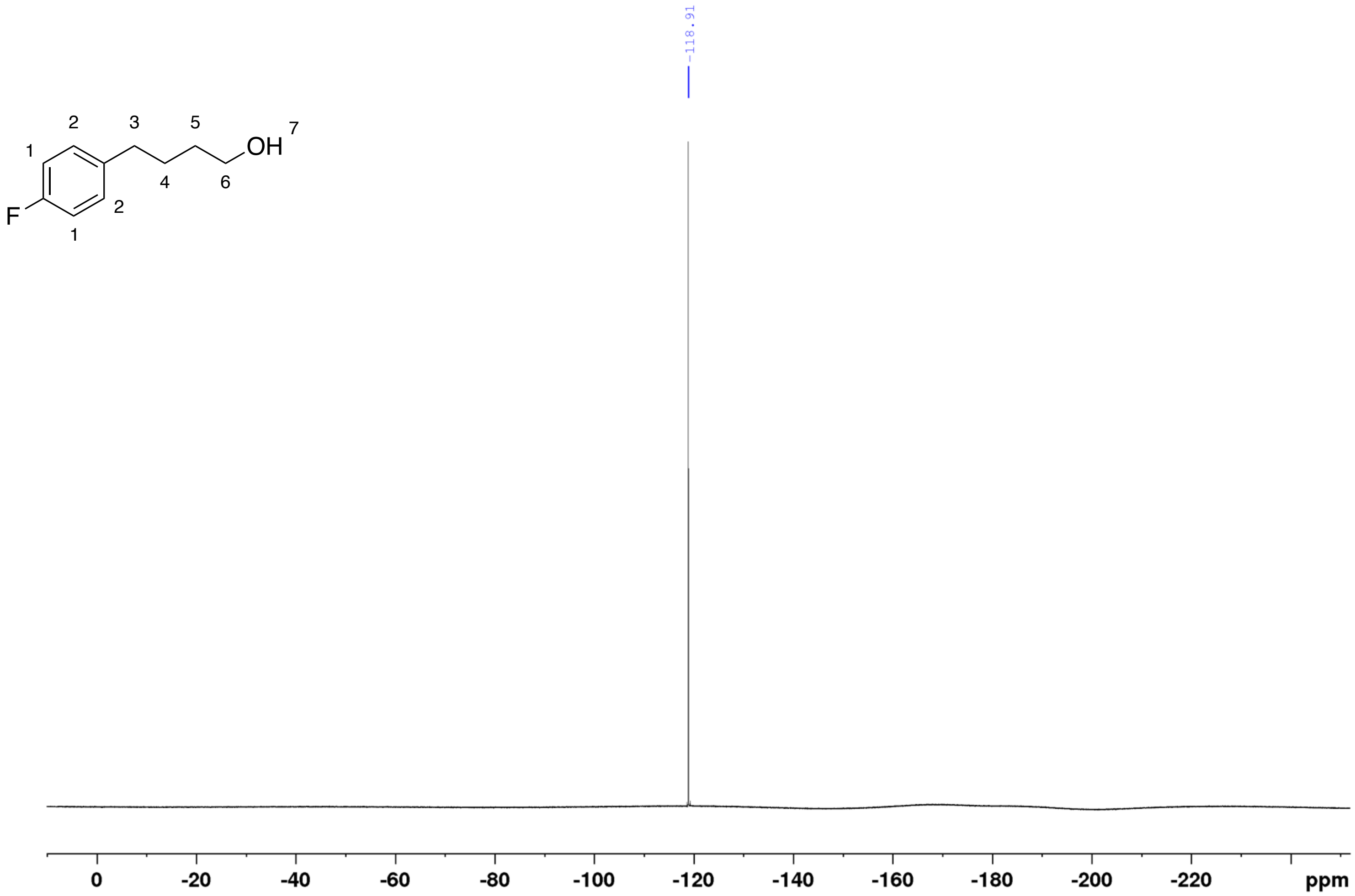

^{1}H NMR (400 MHz, $CDCl_3$) for *4-(3-chlorophenyl)butan-1-ol* (**6h**)

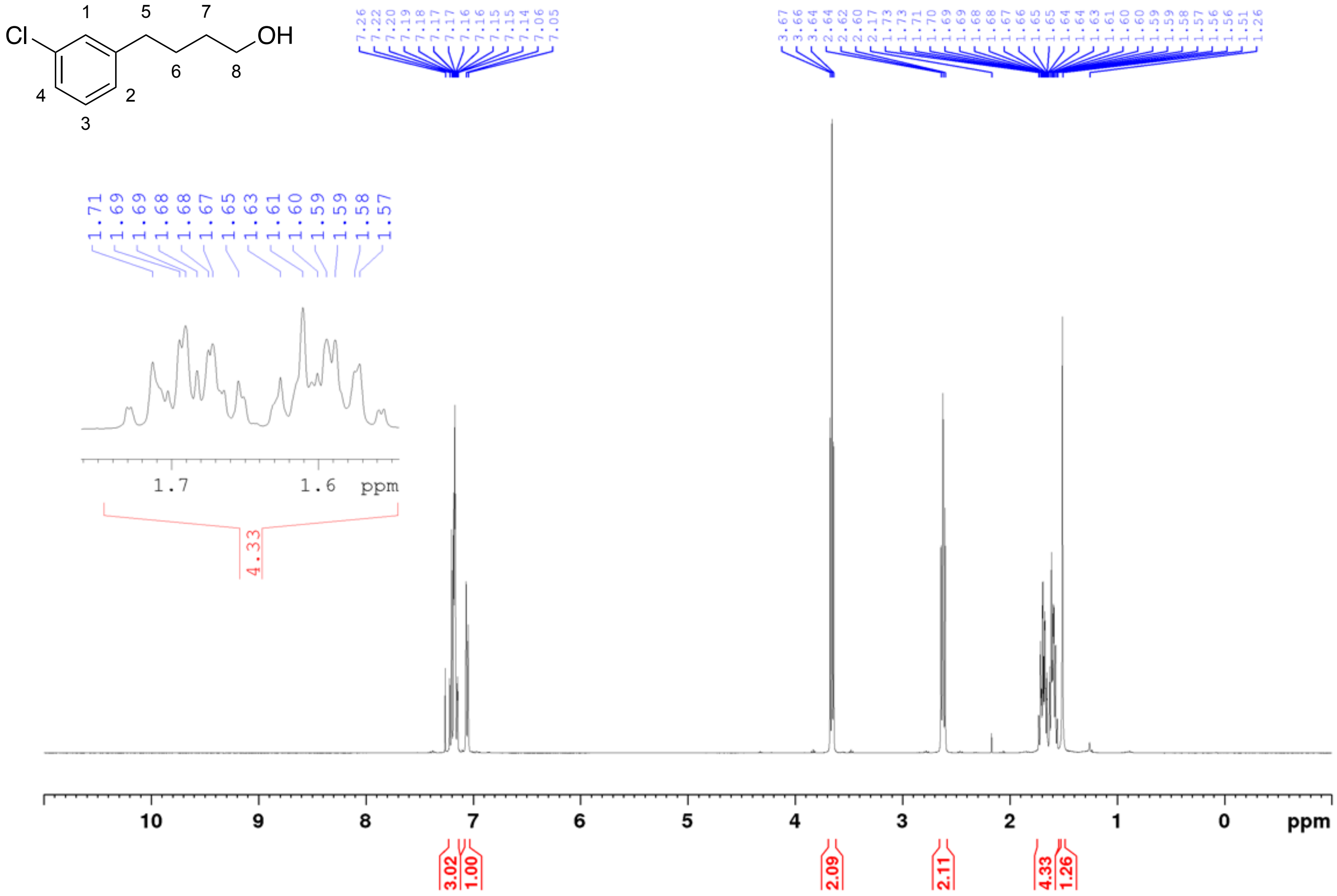

^{13}C NMR (101 MHz, $CDCl_3$) for *4-(3-chlorophenyl)butan-1-ol* (**6h**)

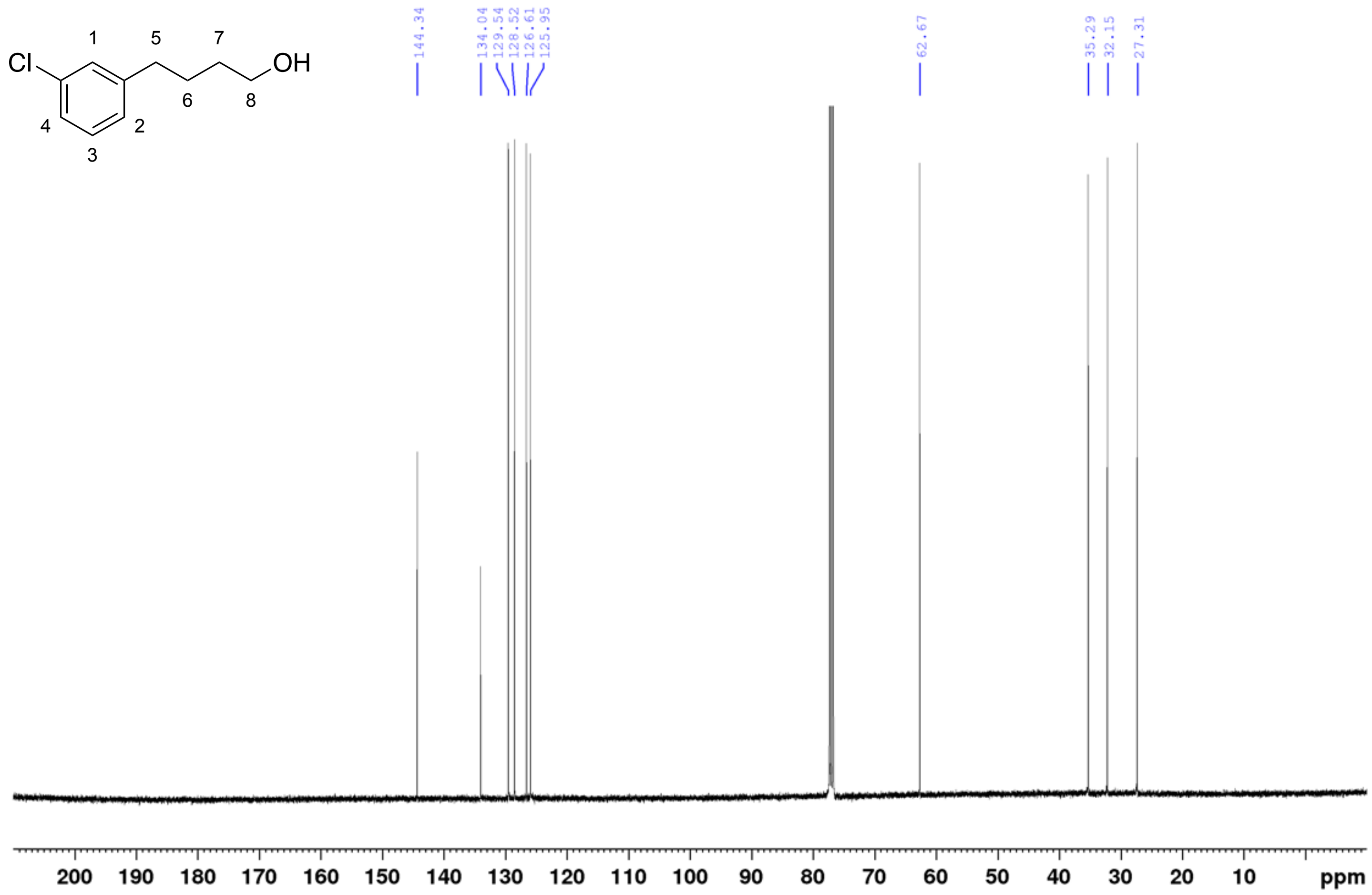

^{1}H NMR (400 MHz, $CDCl_3$) for *4-(3-bromophenyl)butan-1-ol* (**6i**)

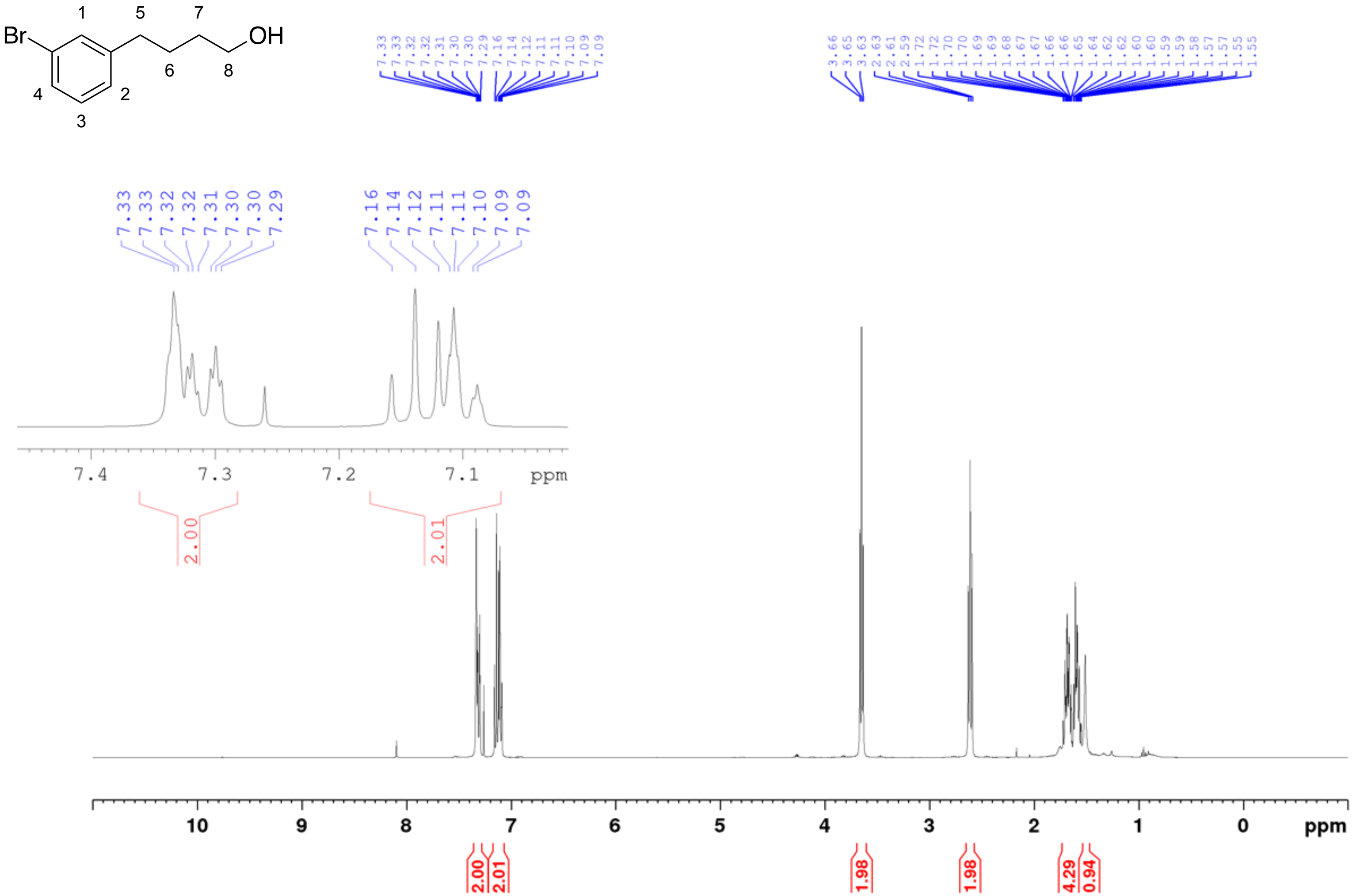

^{13}C NMR (101 MHz, $CDCl_3$) for *4-(3-bromophenyl)butan-1-ol* (**6i**)

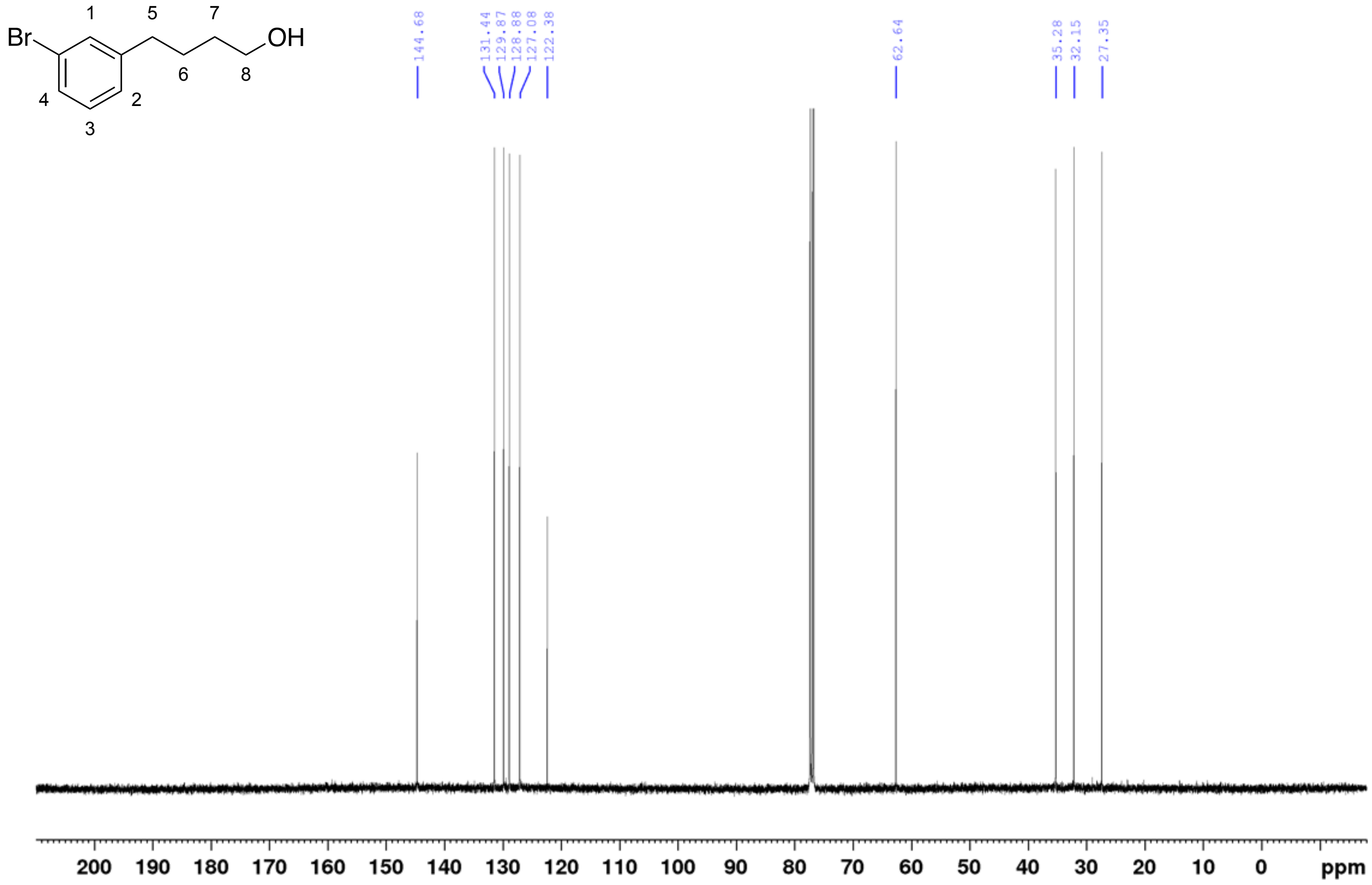

^{1}H NMR (400 MHz, $CDCl_3$) for *(3-iodophenyl)methanol*

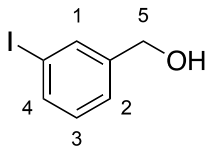

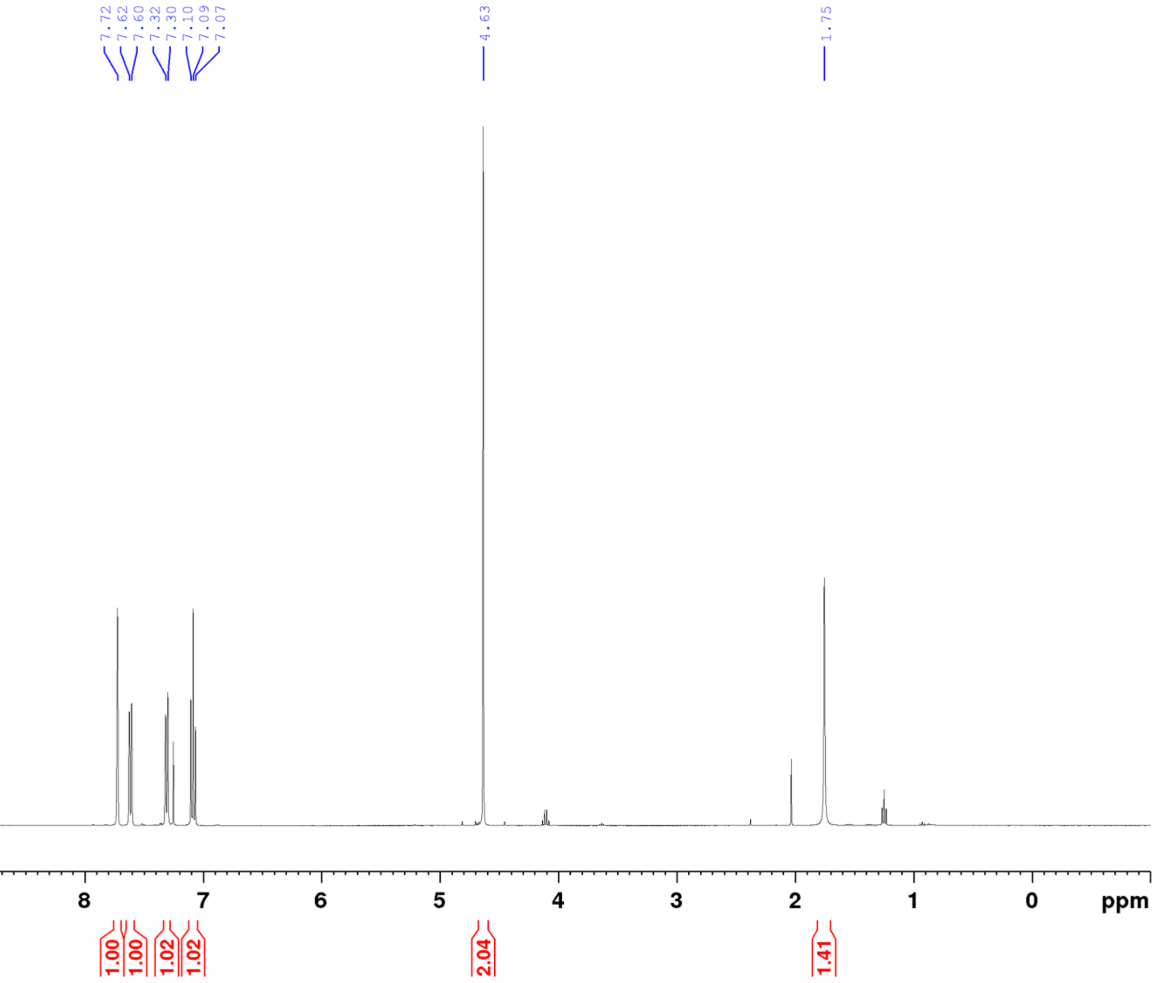

^{13}C NMR (101 MHz, $CDCl_3$) for *(3-iodophenyl)methanol*

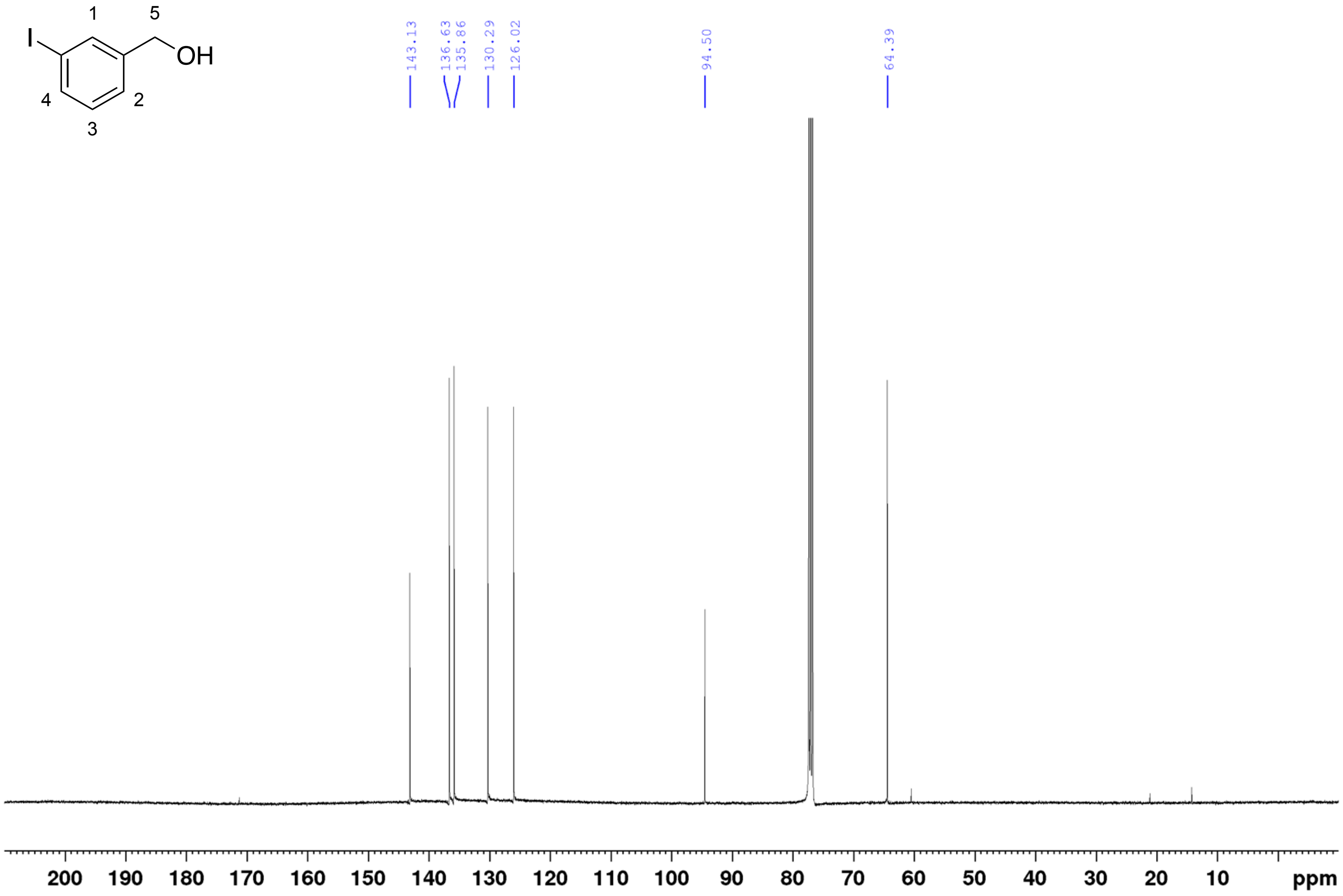

^{1}H NMR (500 MHz, $CDCl_3$) for *4-(3-iodophenyl)butan-1-ol* (**6j**)

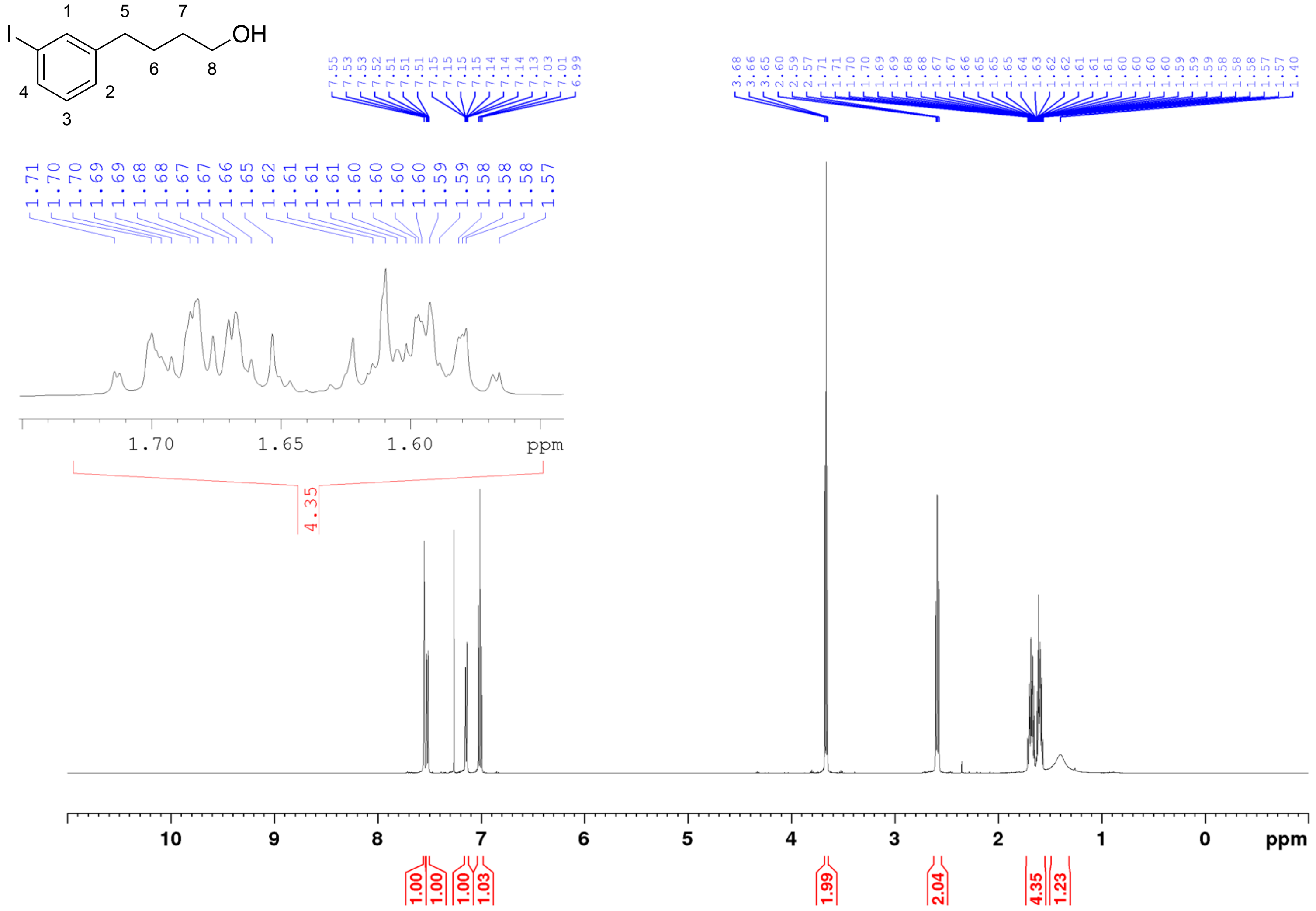

^{13}C NMR (126 MHz, $CDCl_3$) for *4-(3-iodophenyl)butan-1-ol* (**6j**)

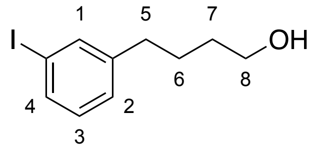

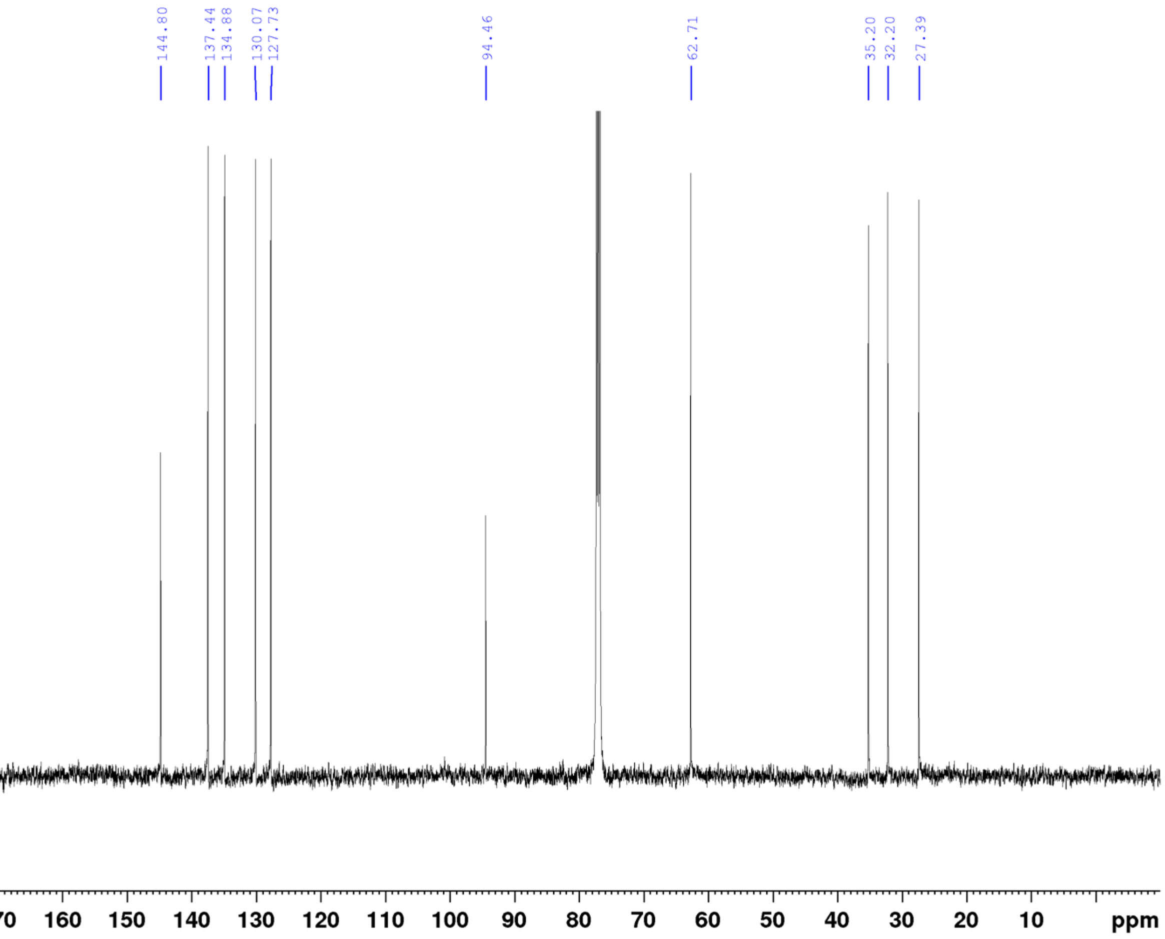

^{1}H NMR (400 MHz, $CDCl_3$) for *3-iodophenyl acetate*

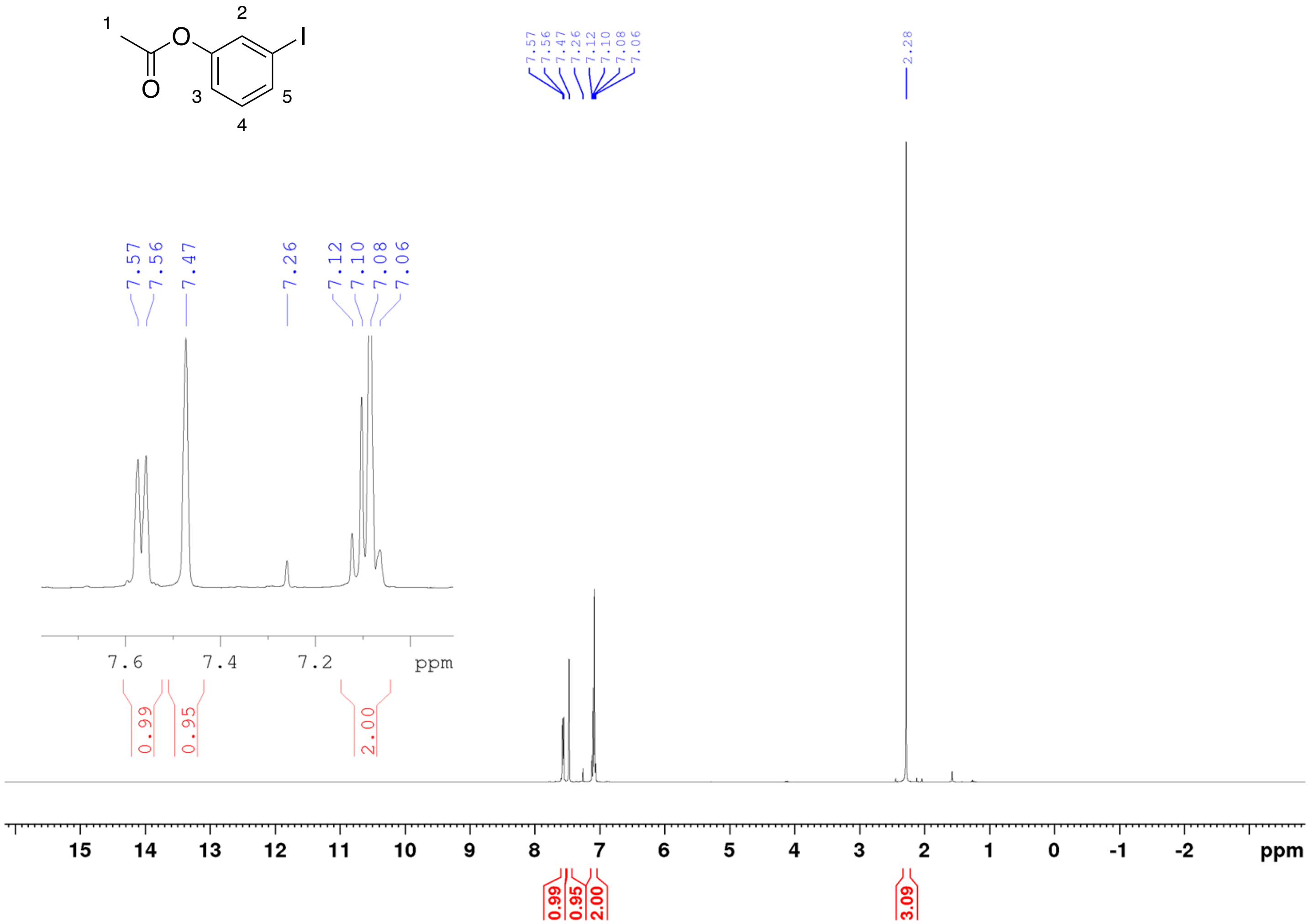

^{13}C NMR (101 MHz, $CDCl_3$) for *3-iodophenyl acetate*

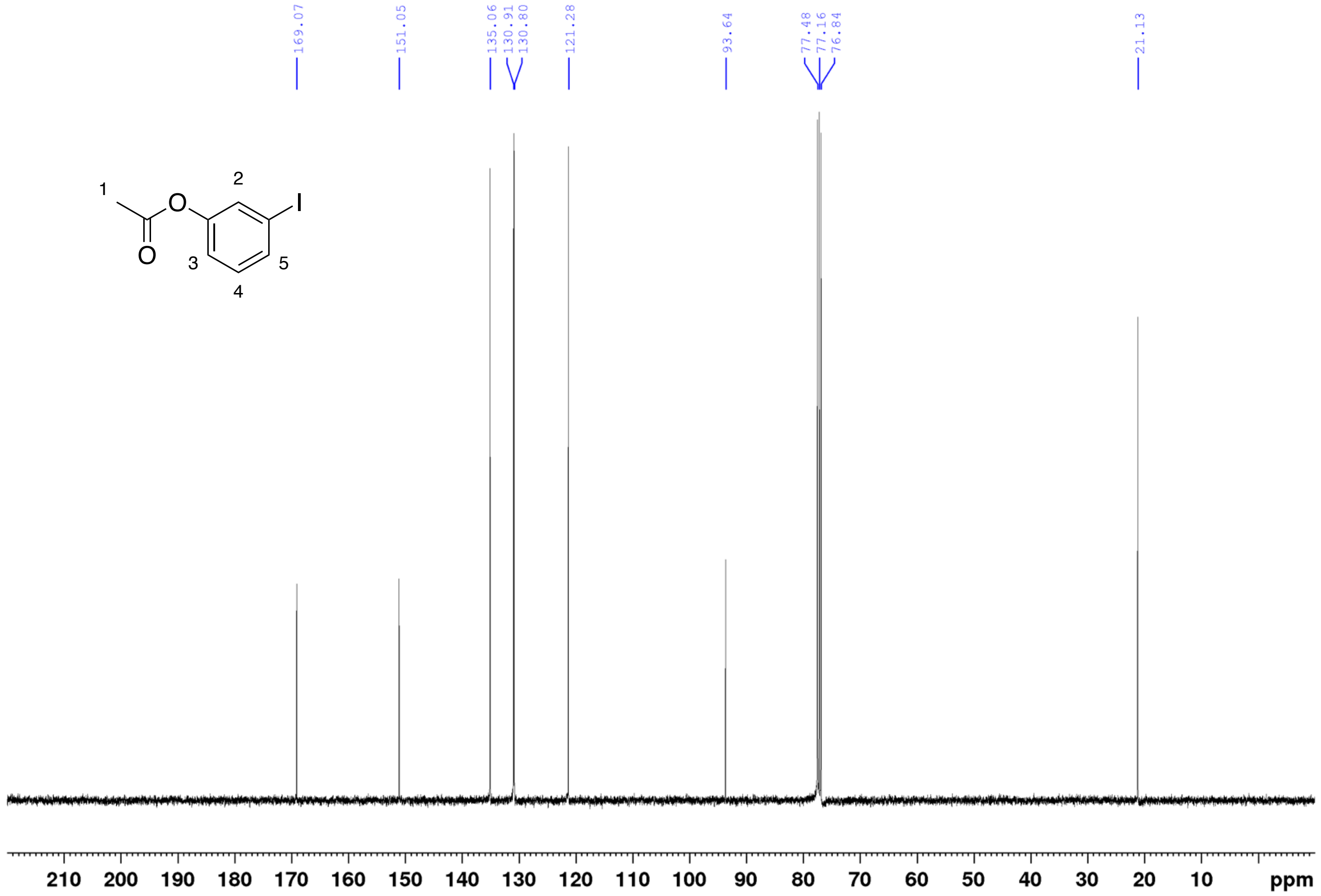

^{1}H NMR (400 MHz, $CDCl_3$) for *3-(4-hydroxybut-1-yn-1-yl)phenyl acetate*

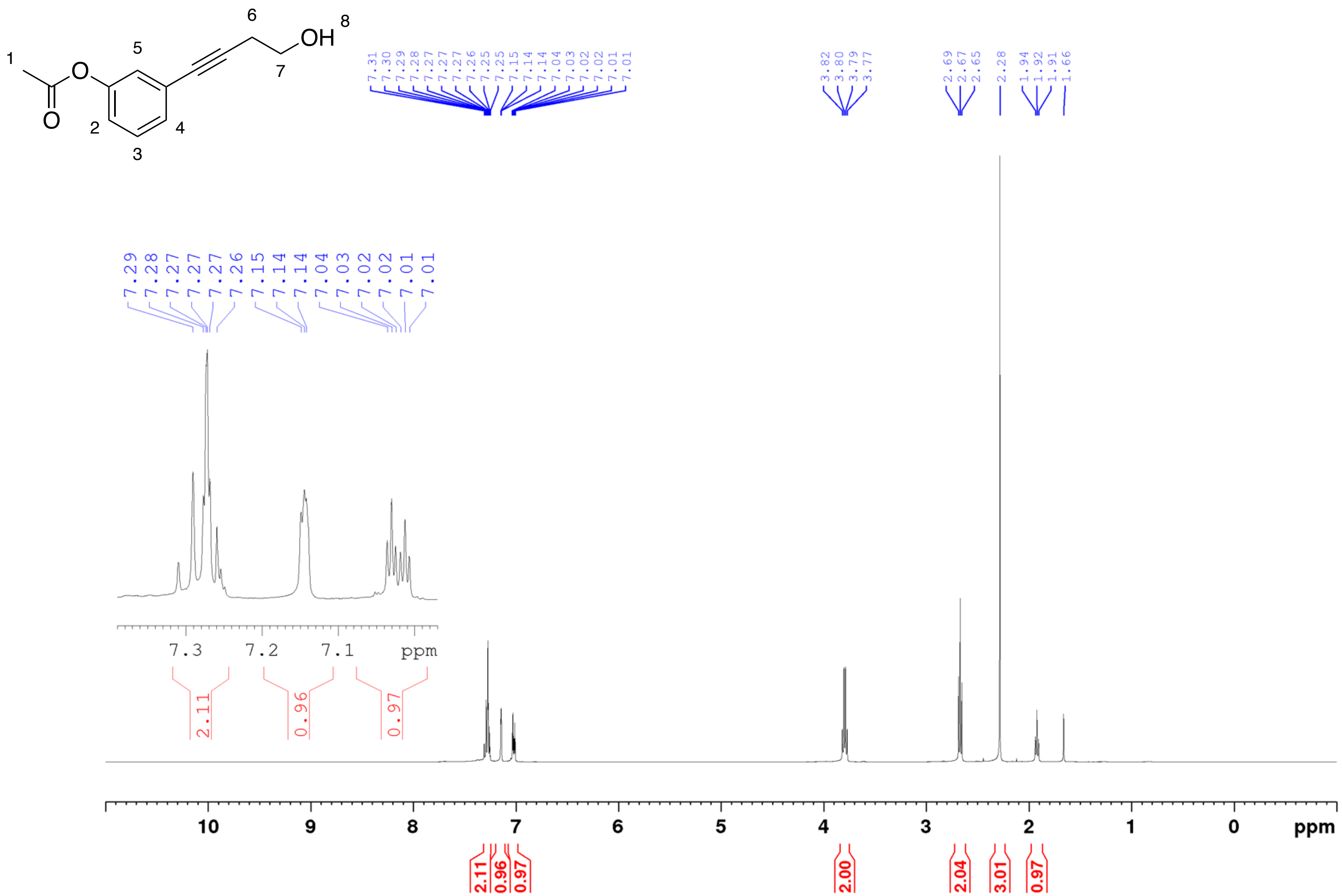

^{13}C NMR (101 MHz, $CDCl_3$) for *3-(4-hydroxybut-1-yn-1-yl)phenyl acetate*

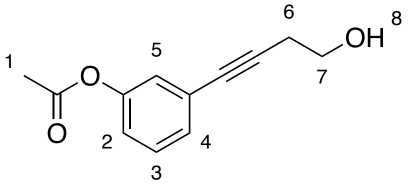

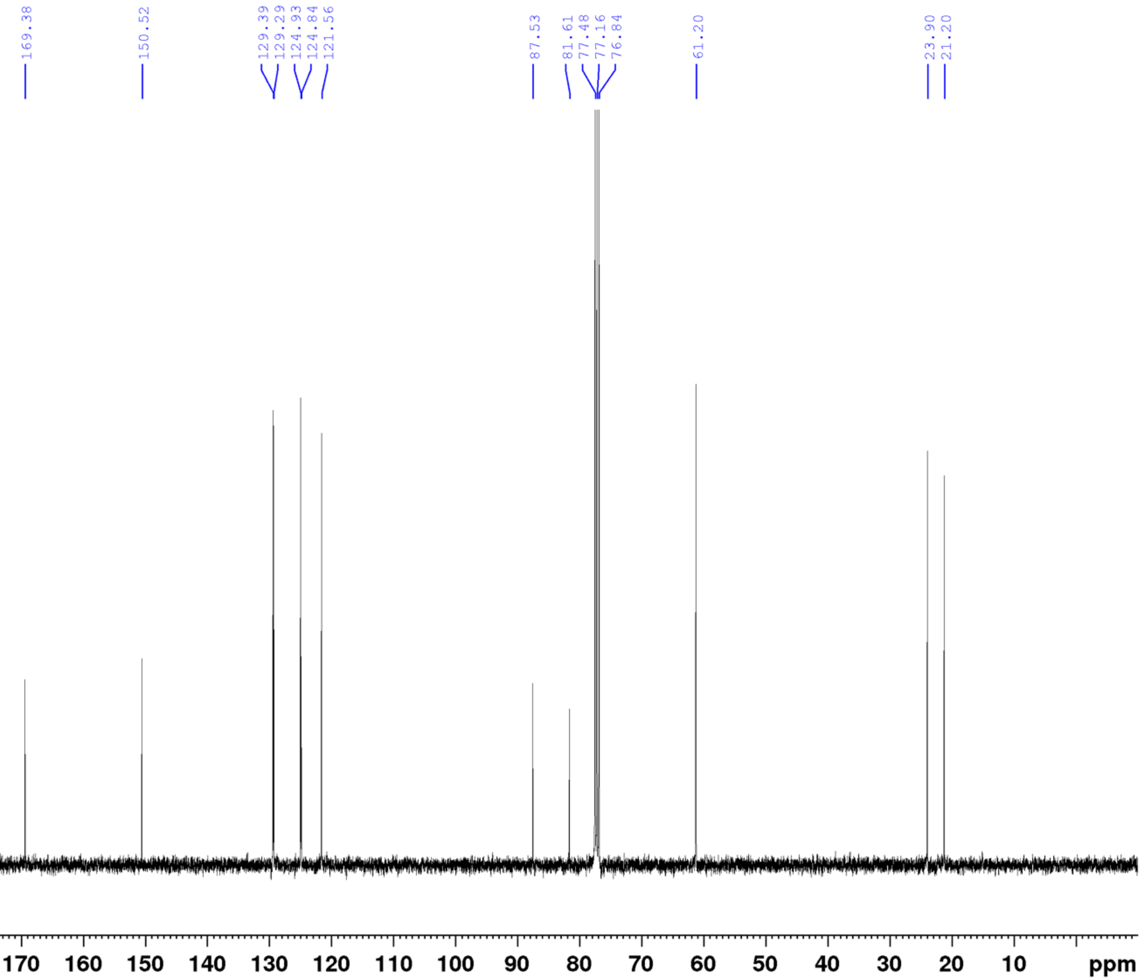

^{1}H NMR (400 MHz, $CDCl_3$) for *3-(4-hydroxybutyl)phenyl acetate* (**6k**)

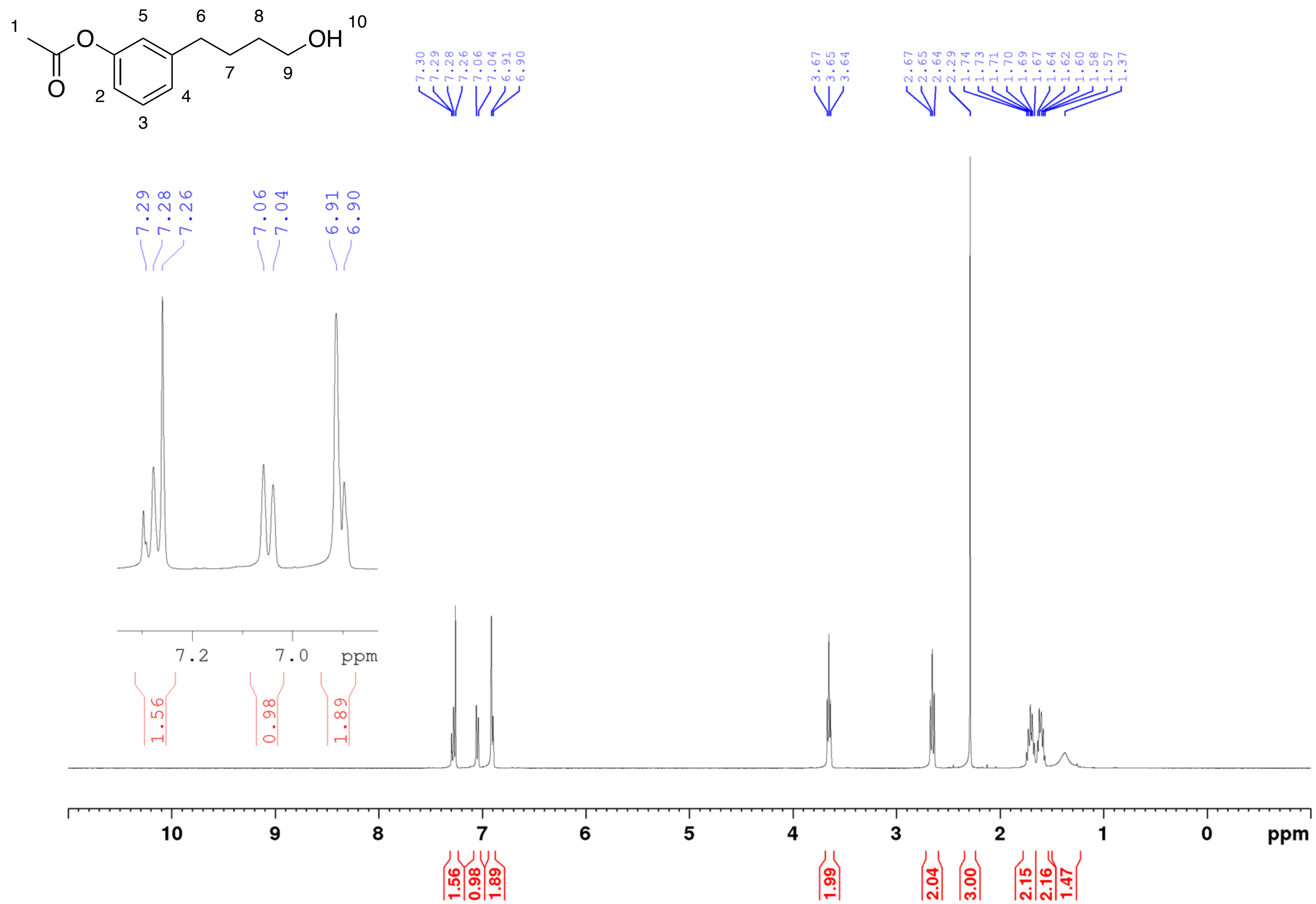

^{13}C NMR (101 MHz, $CDCl_3$) for *3-(4-hydroxybutyl)phenyl acetate* (**6k**)

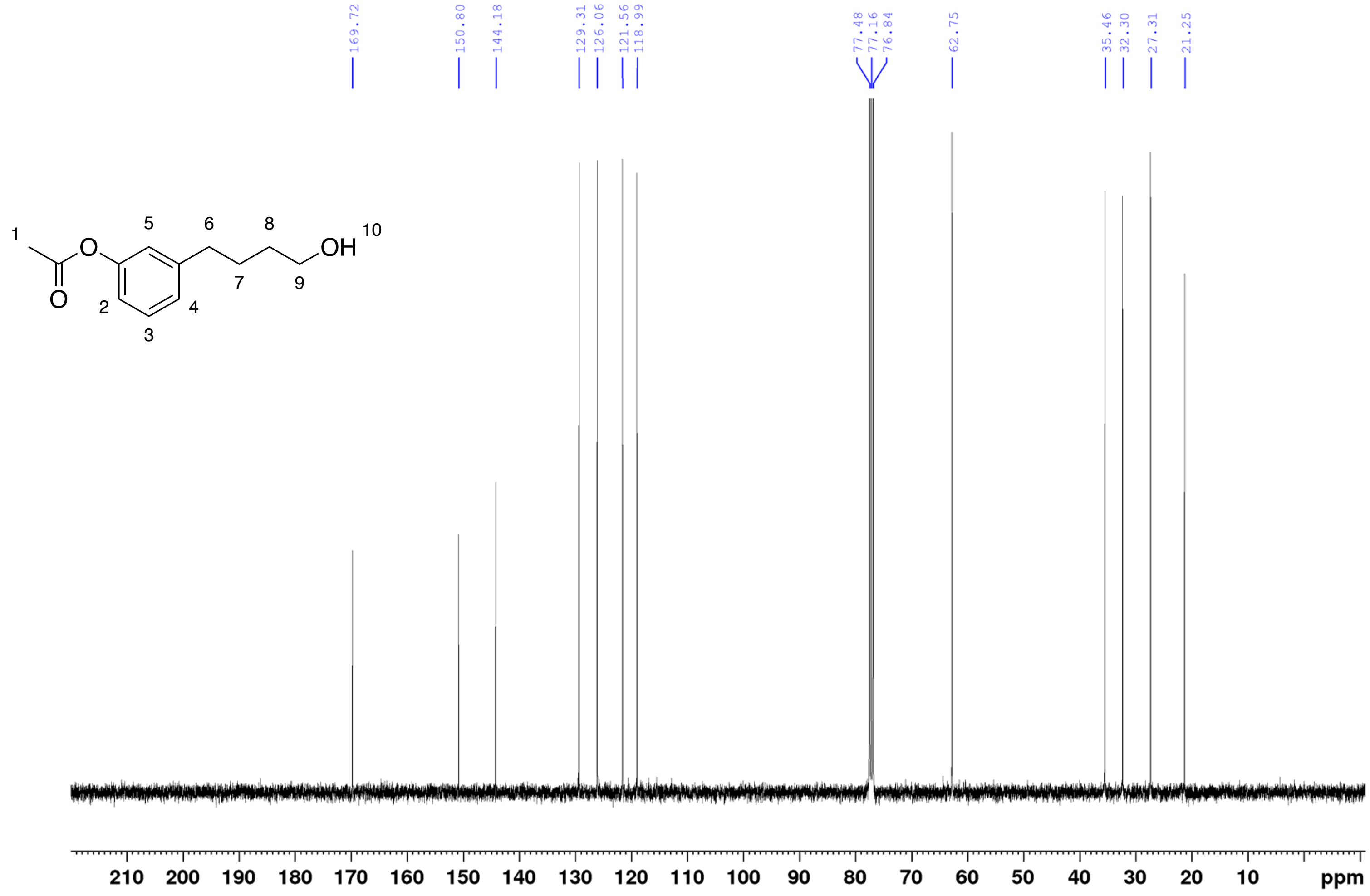

^{1}H NMR (400 MHz, $CDCl_3$) for *4-(3-isopropylphenyl)but-3-yn-1-ol*

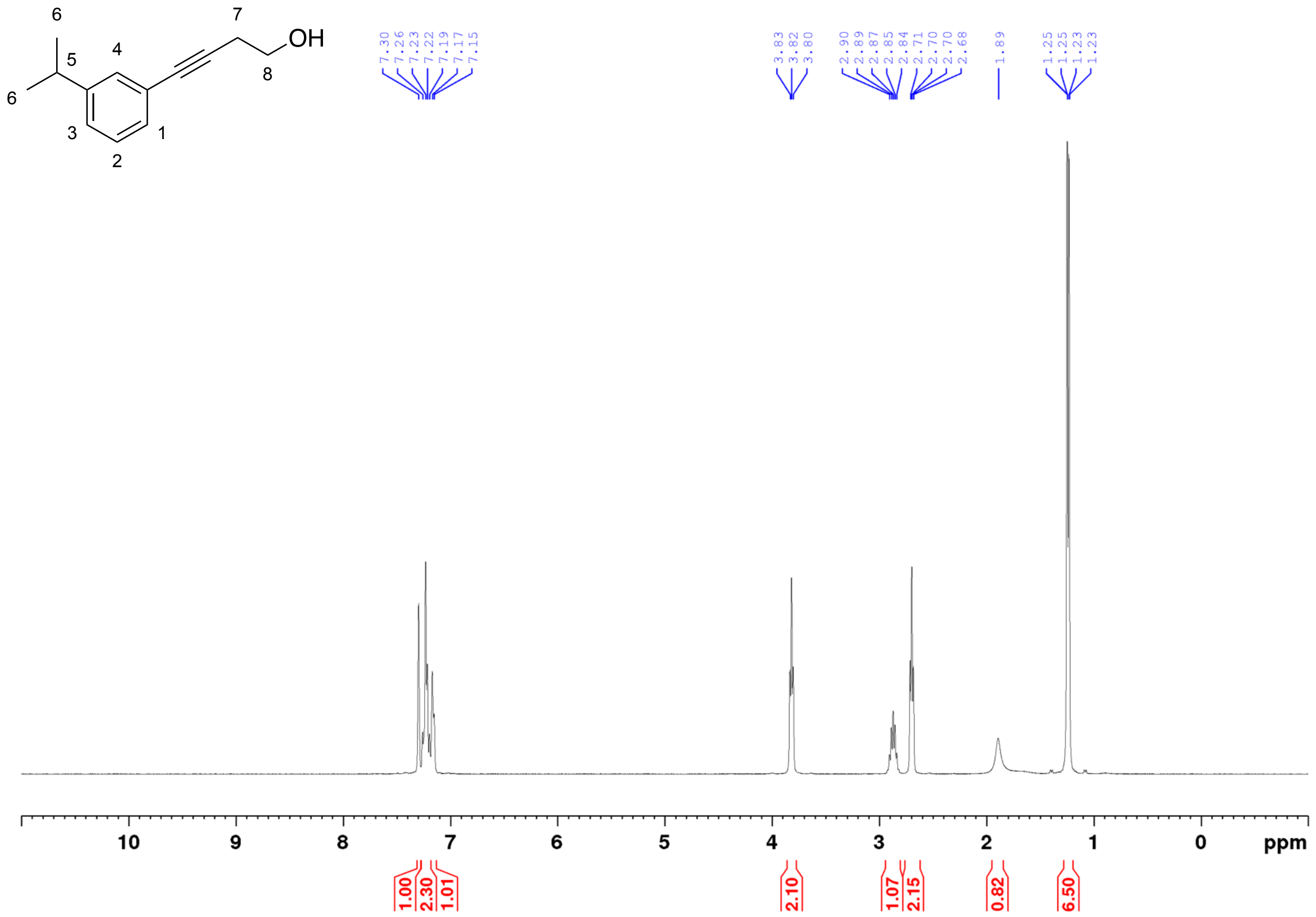

^{13}C NMR (101 MHz, $CDCl_3$) for *4-(3-isopropylphenyl)but-3-yn-1-ol*

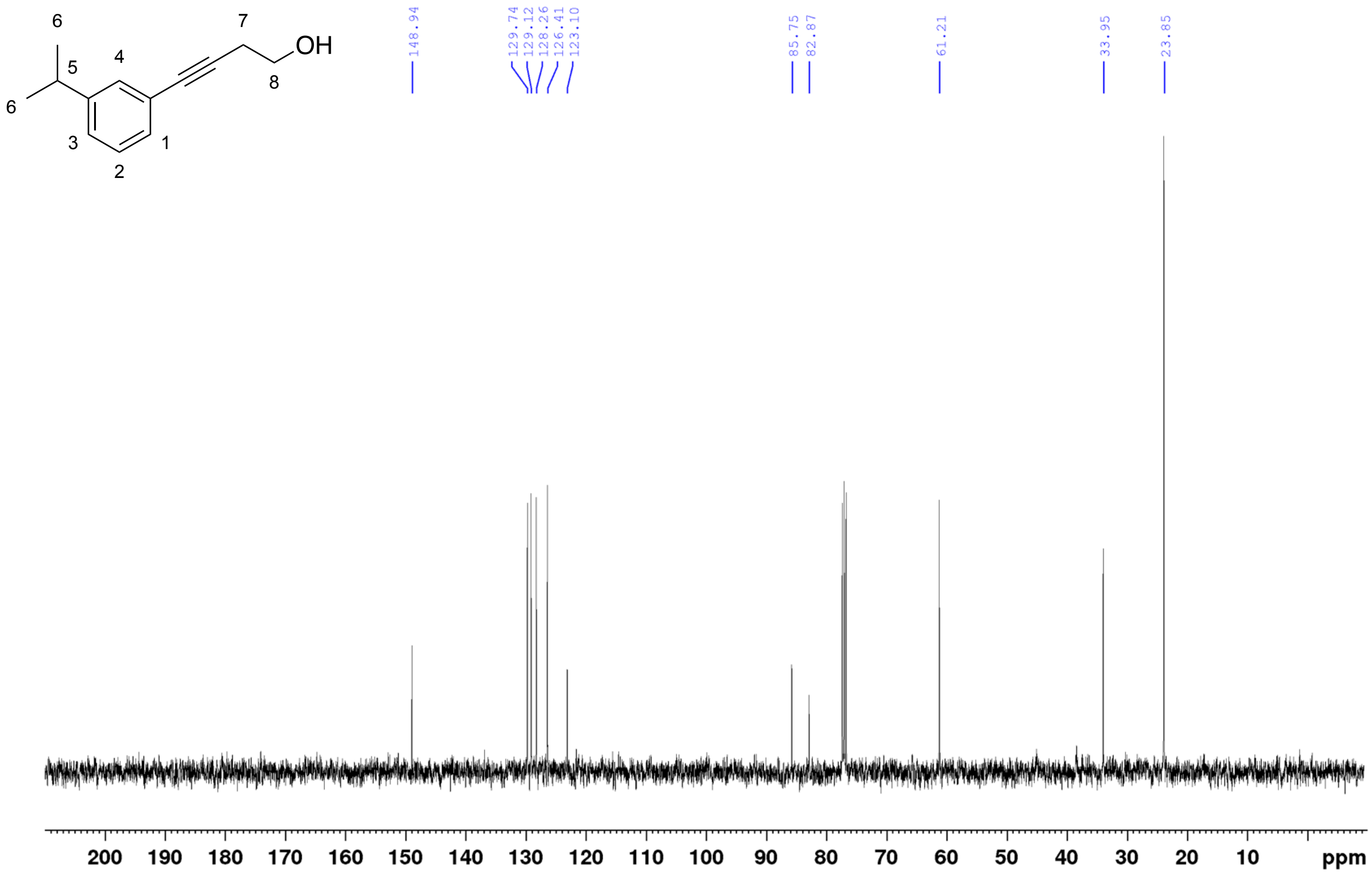

^{1}H NMR (500 MHz, $CDCl_3$) for *4-(3-Isopropylphenyl)butan-1-ol* (**6l**)

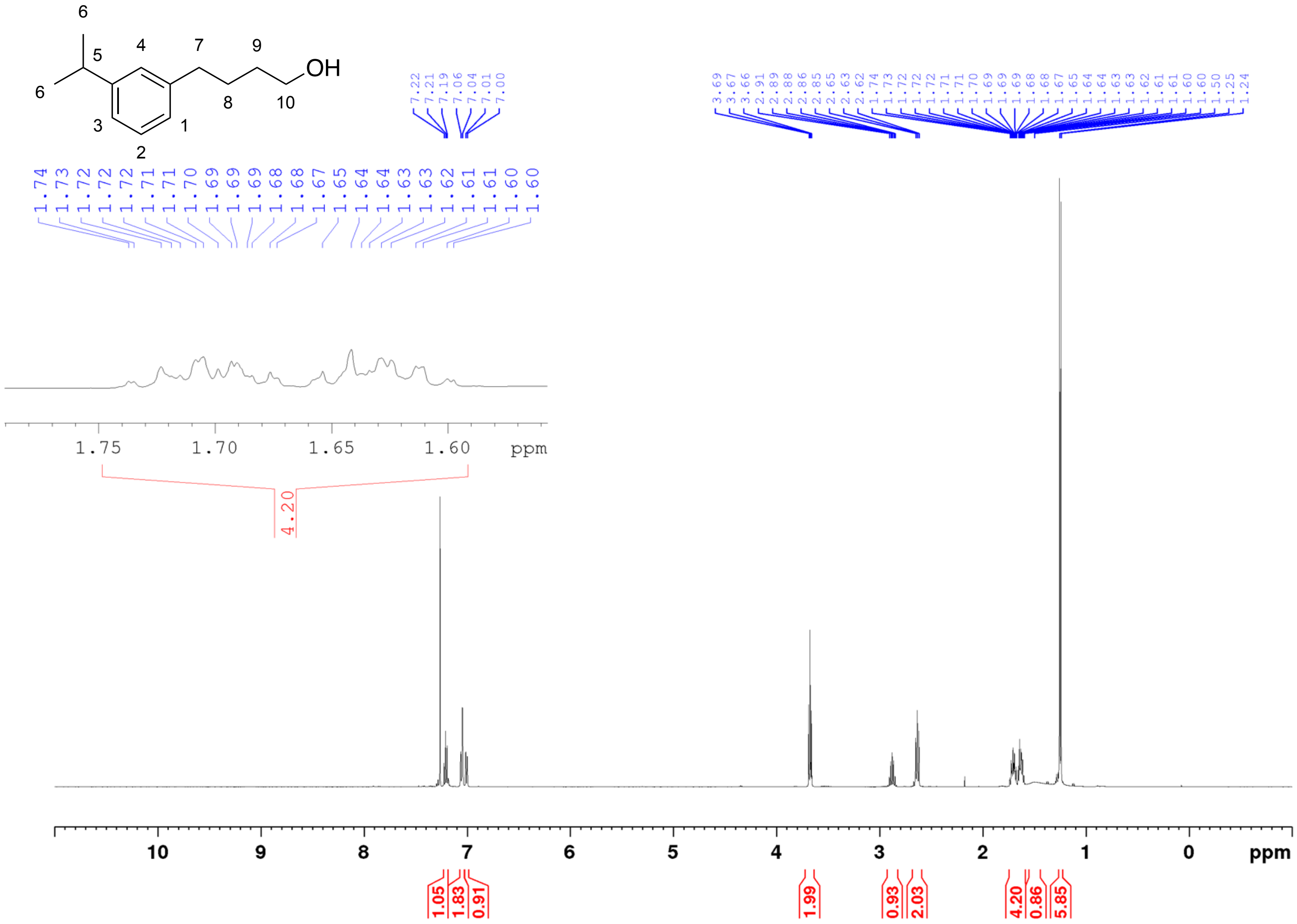

^{13}C NMR (126 MHz, $CDCl_3$) for *4-(3-Isopropylphenyl)butan-1-ol* (**6l**)

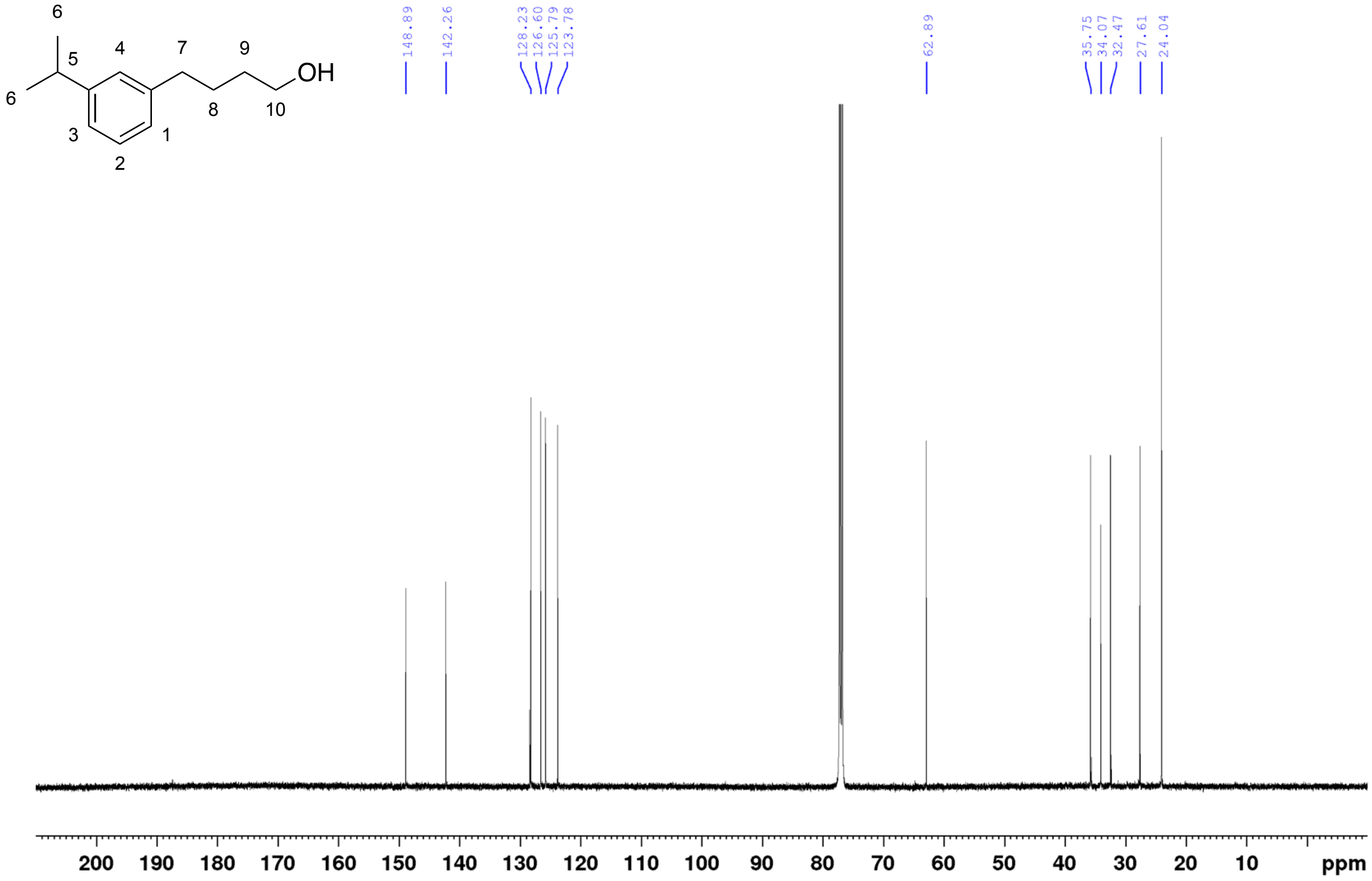

^{1}H NMR (400 MHz, $CDCl_3$) for *4-(2-chlorophenyl)butan-1-ol* (**6m**)

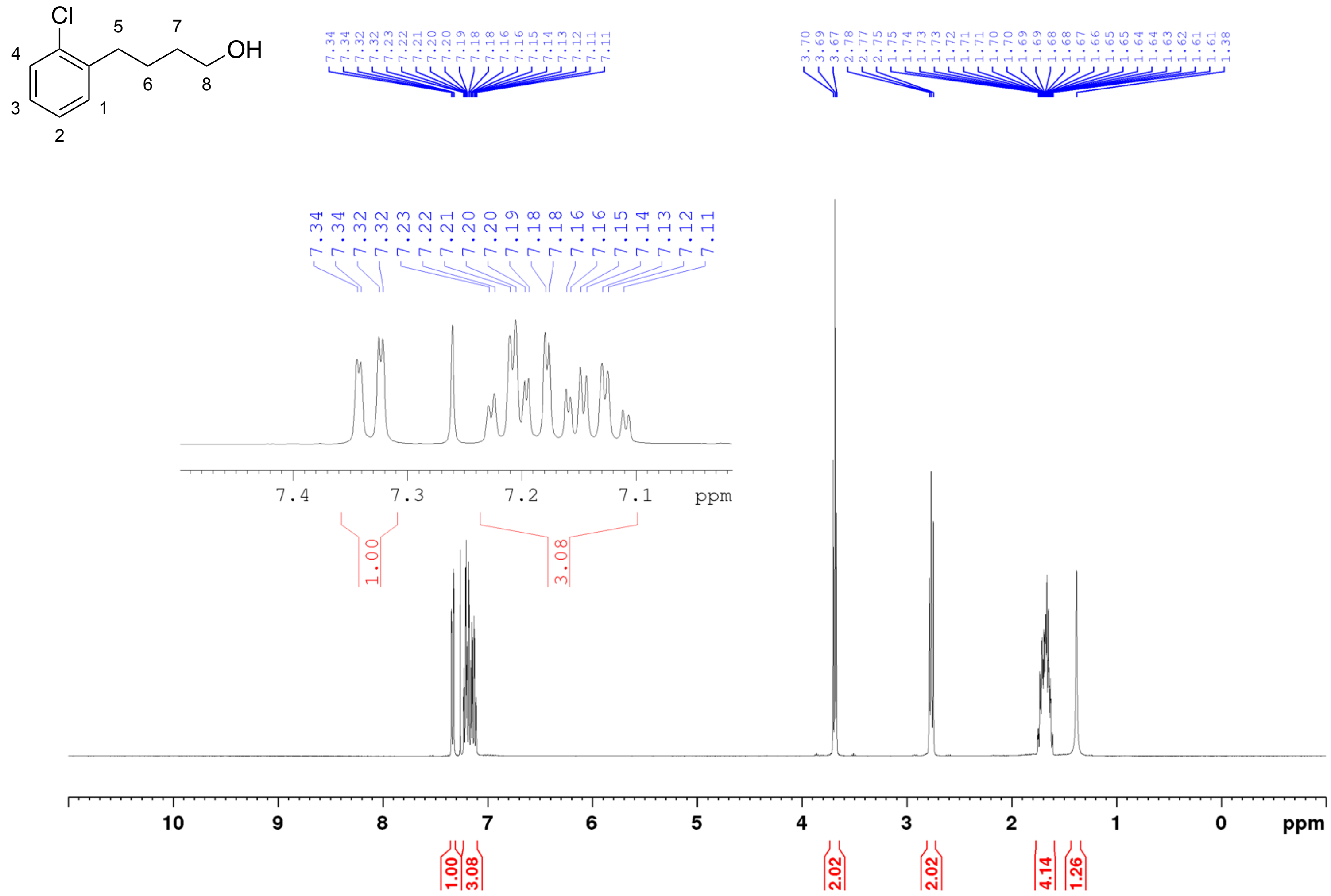

^{13}C NMR (101 MHz, $CDCl_3$) for *4-(2-chlorophenyl)butan-1-ol* (**6m**)

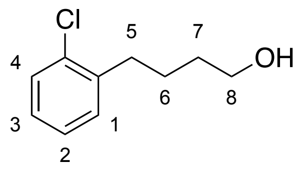

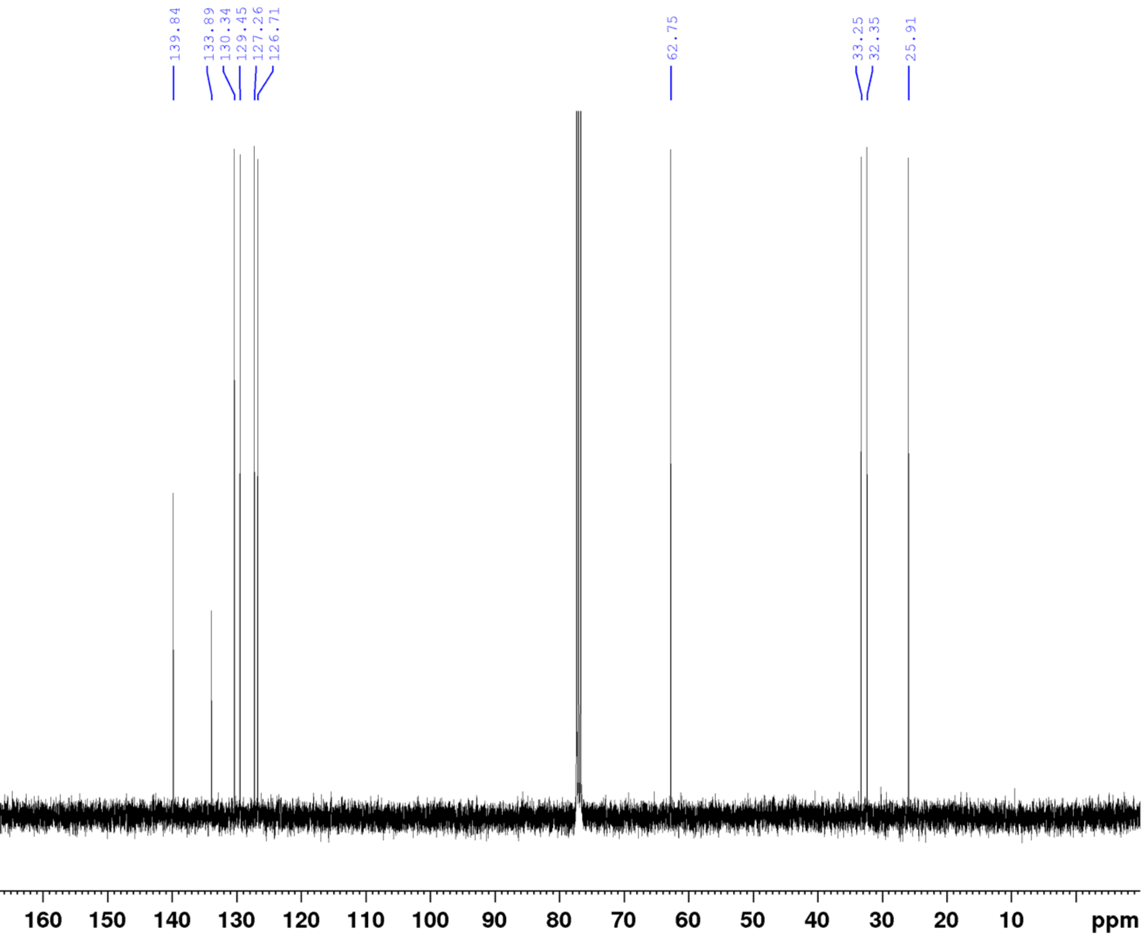

¹H NMR (400 MHz, $CDCl_3$) for *4-(4-chlorophenyl)butan-1-ol* (**6o**)

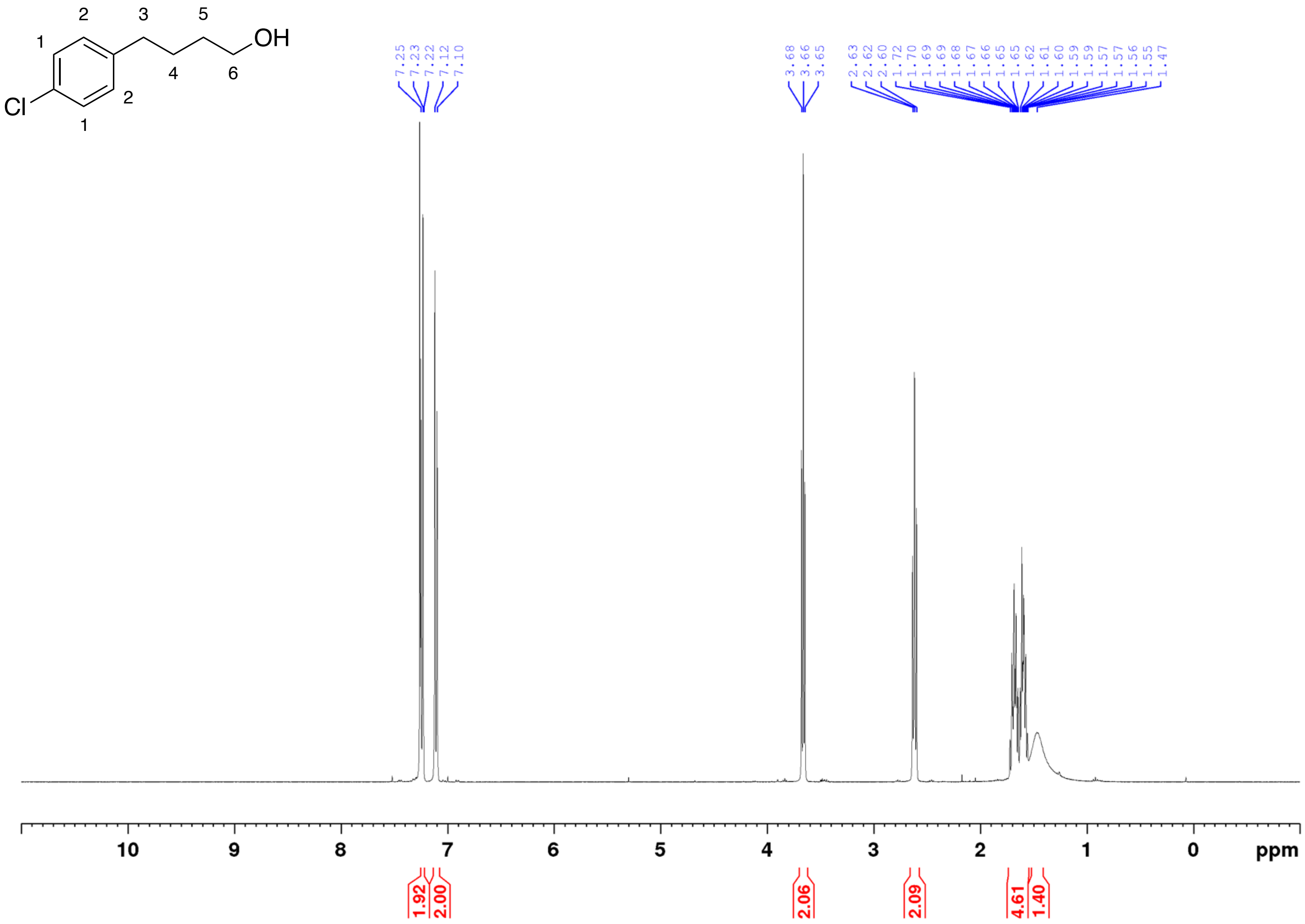

^{13}C NMR (101 MHz, $CDCl_3$) for *4-(4-chlorophenyl)butan-1-ol* (**6o**)

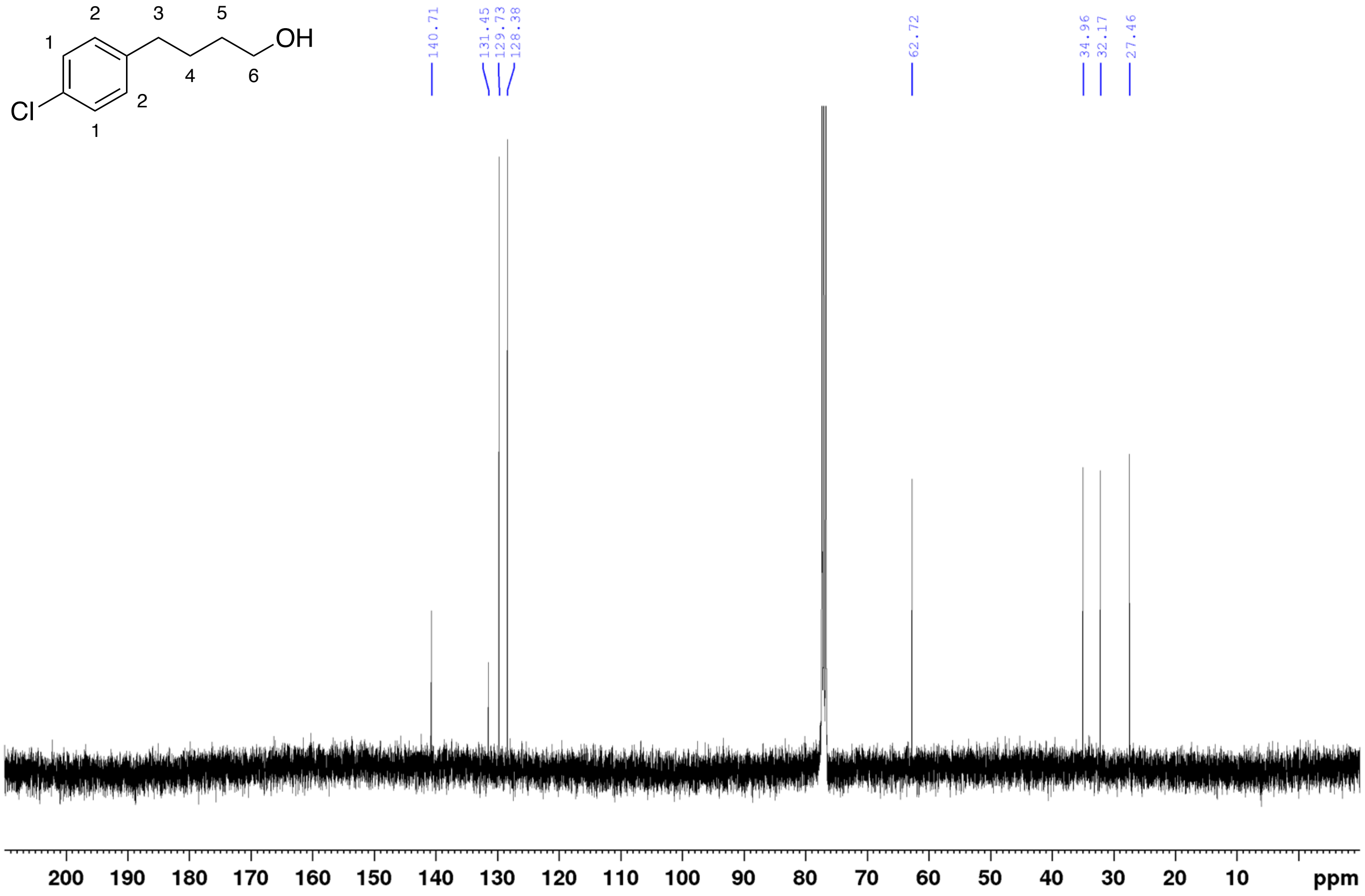

^{1}H NMR (400 MHz, $CDCl_3$) for *(5-chloro-2-methylphenyl)methanol*

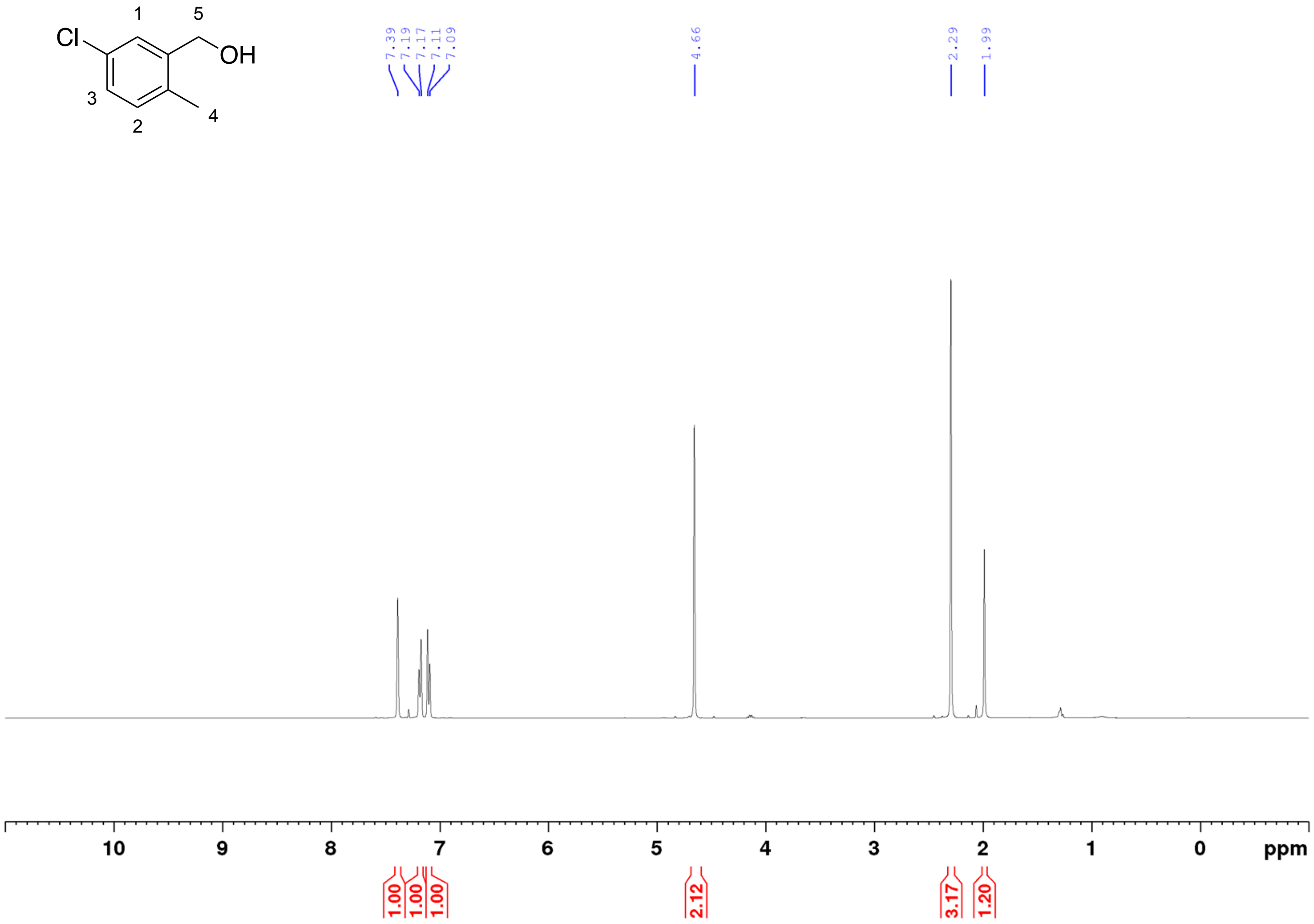

^{13}C NMR (101 MHz, $CDCl_3$) for *(5-chloro-2-methylphenyl)methanol*

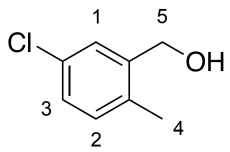

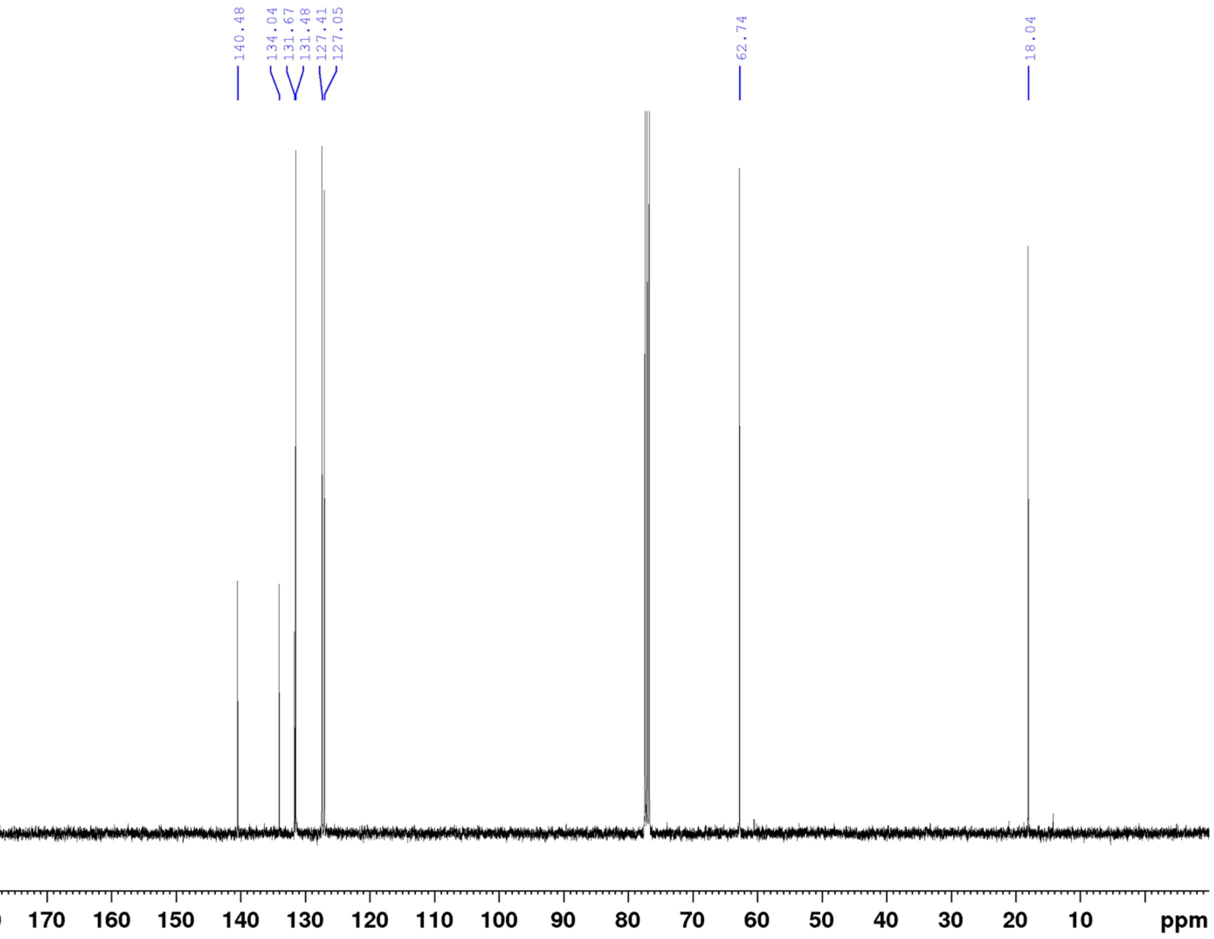

¹H NMR (400 MHz, $CDCl_3$) for *4-(5-chloro-2-methylphenyl)butan-1-ol* (**6p**)

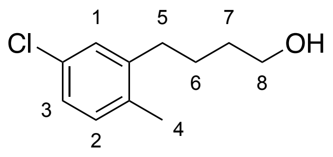

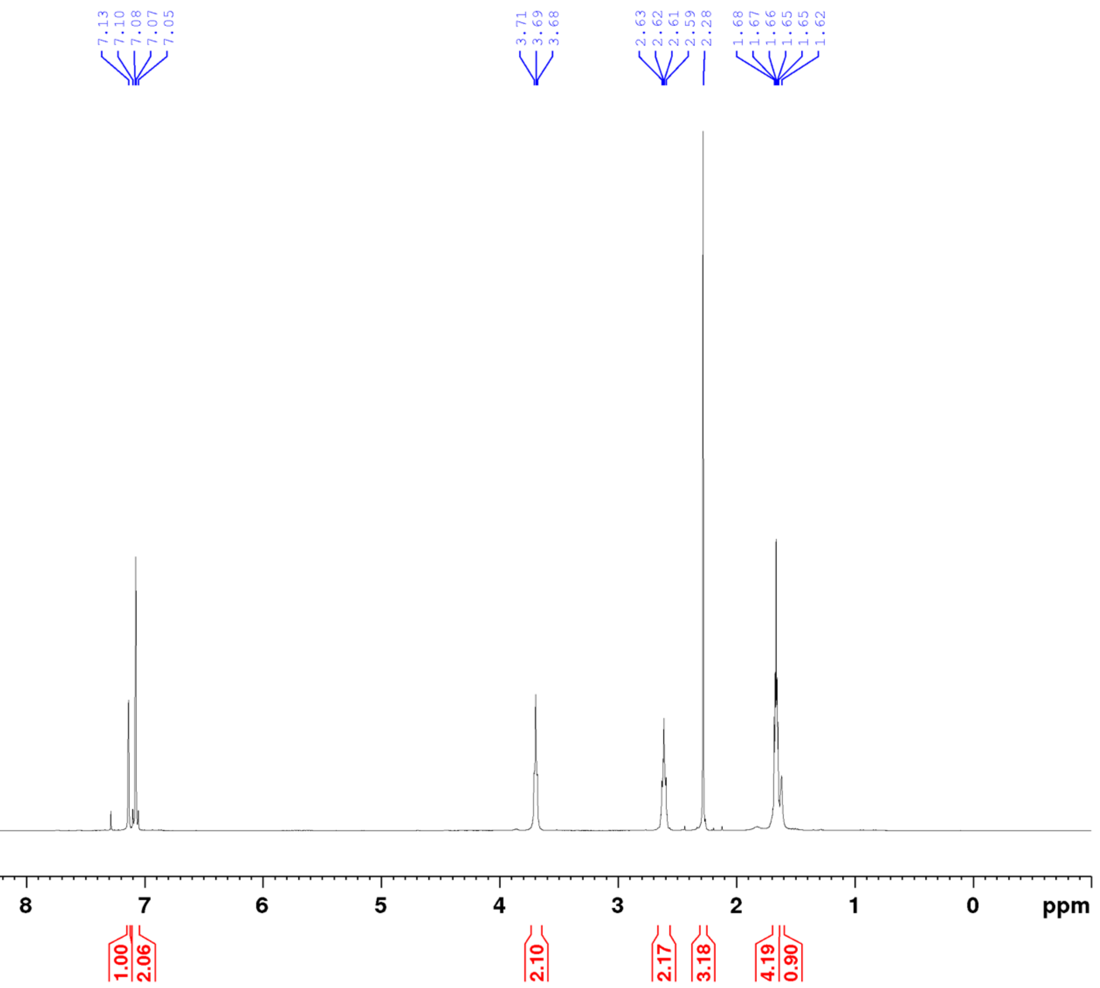

^{13}C NMR (101 MHz, $CDCl_3$) for *4-(5-chloro-2-methylphenyl)butan-1-ol* (**6p**)

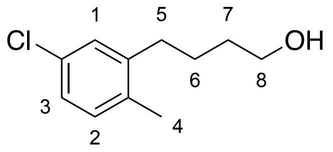

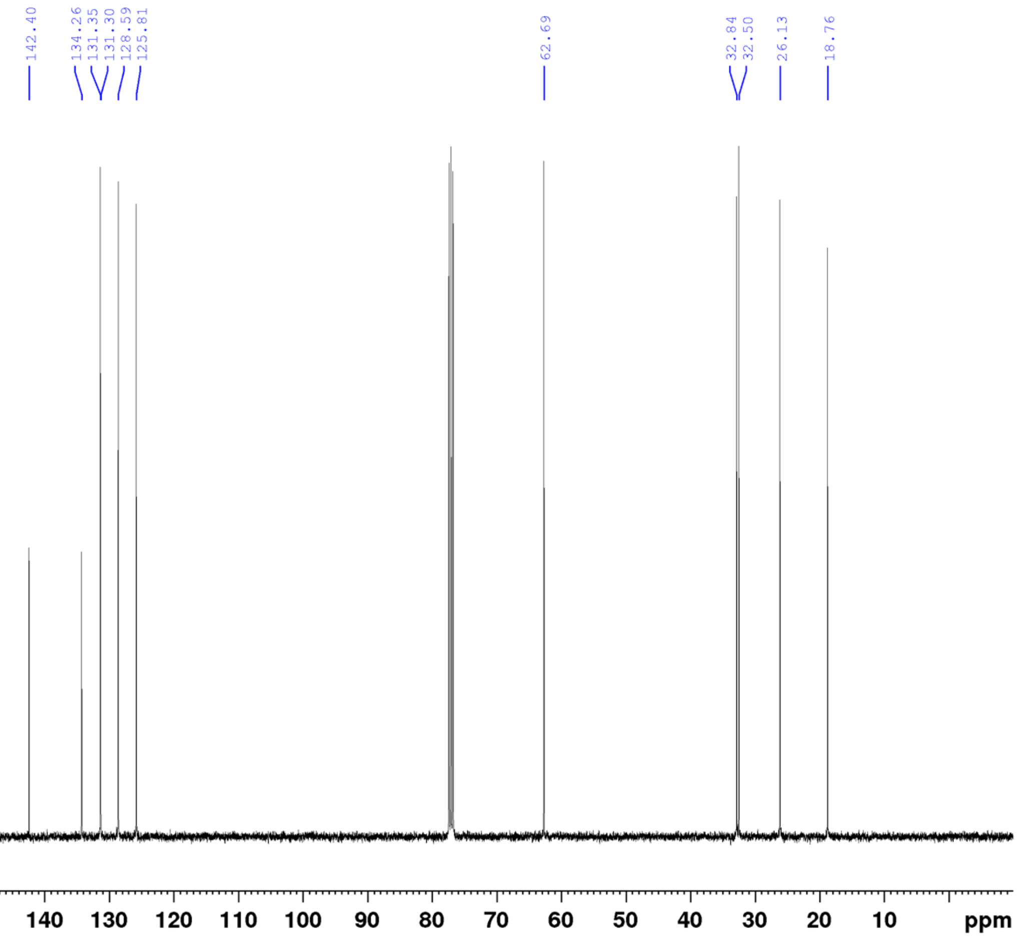

^{1}H NMR (400 MHz, $CDCl_3$) for *4-(2,3-dimethylphenyl)but-3-yn-1-ol*

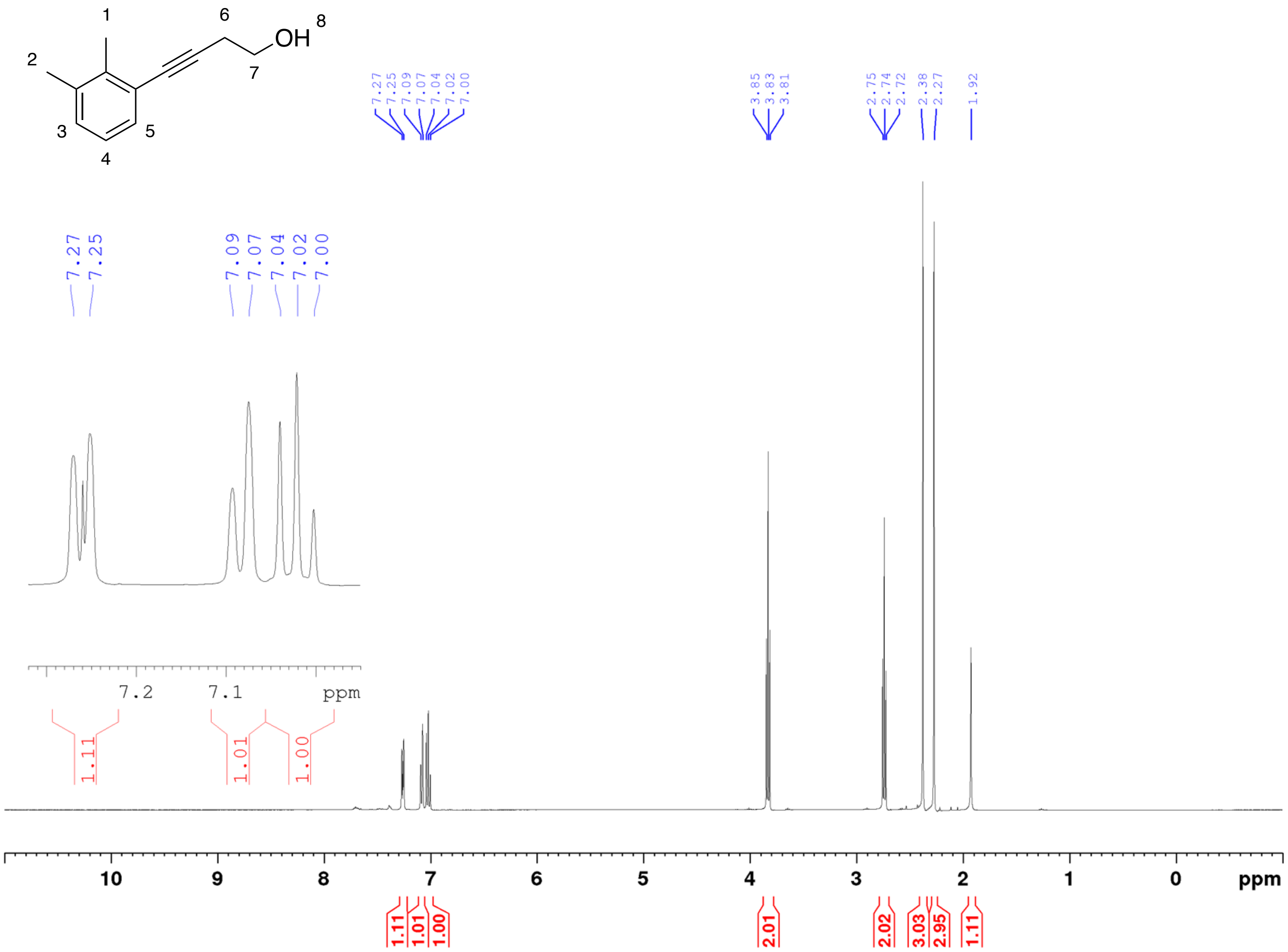

^{13}C NMR (101 MHz, $CDCl_3$) for *4-(2,3-dimethylphenyl)but-3-yn-1-ol*

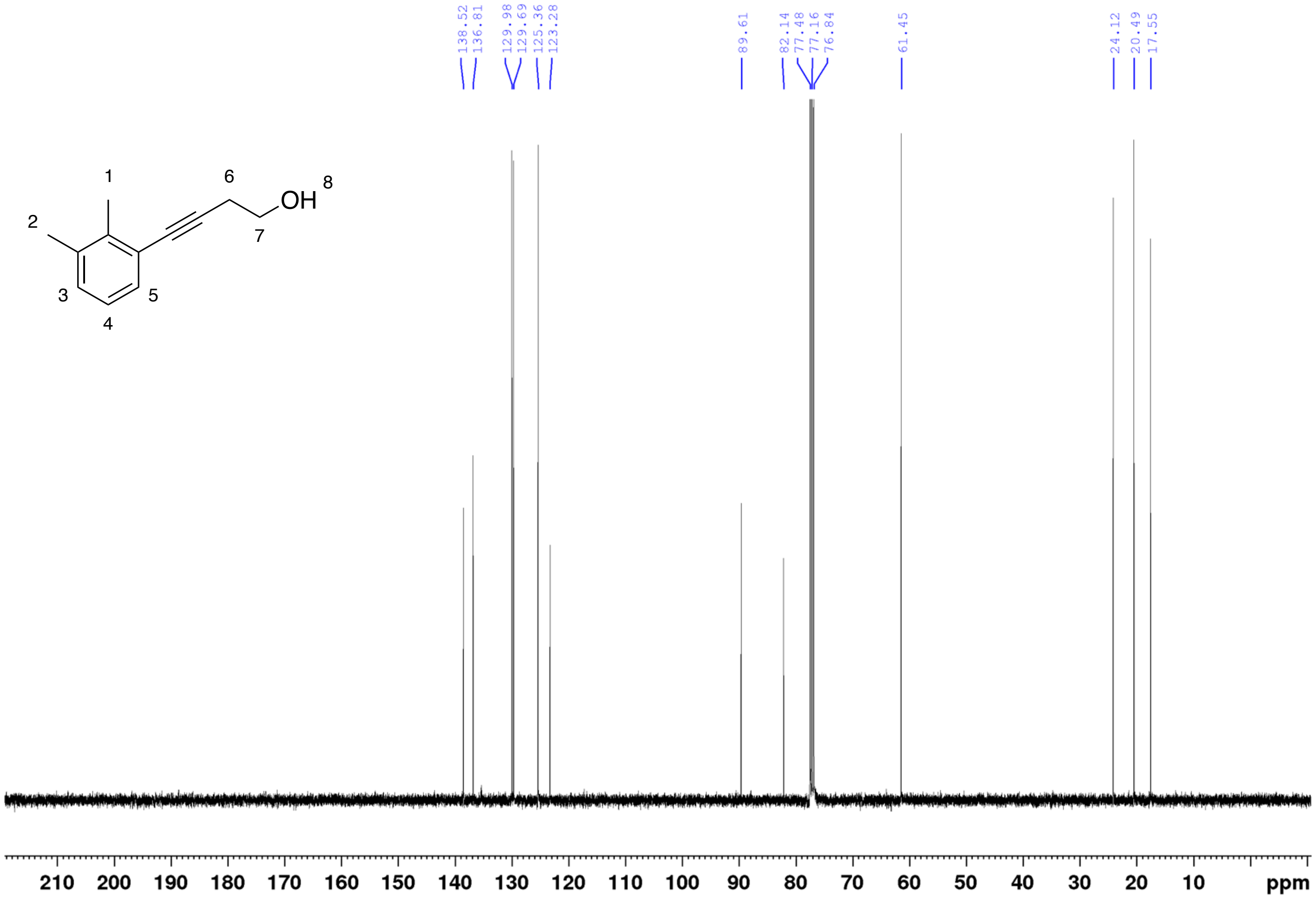

^{1}H NMR (400 MHz, $CDCl_3$) for *4-(2,3-dimethylphenyl)butan-1-ol* (**6q**)

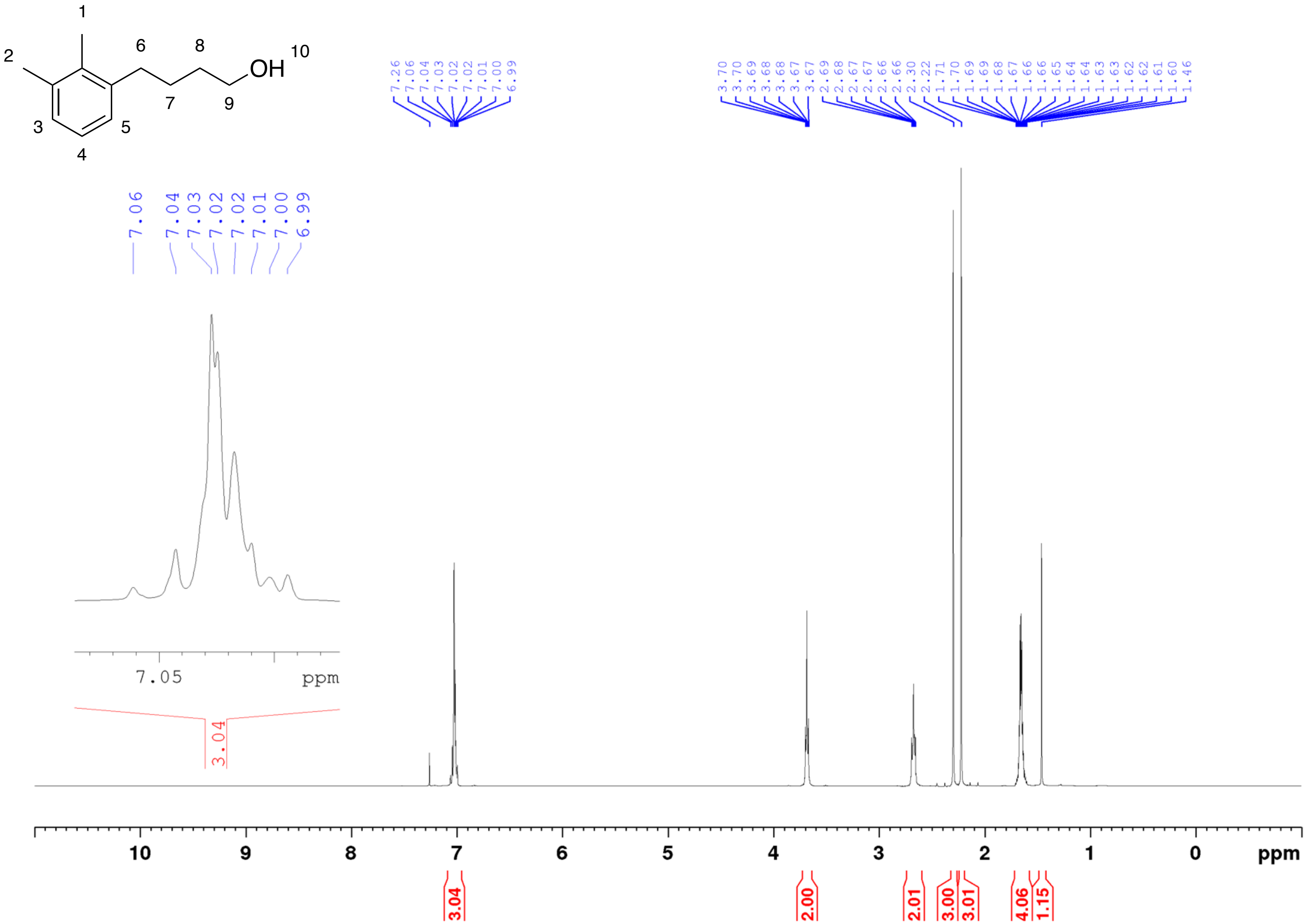

^{13}**C NMR** (101 MHz, $CDCl_3$) for *4-(2,3-dimethylphenyl)butan-1-ol* (**6q**)

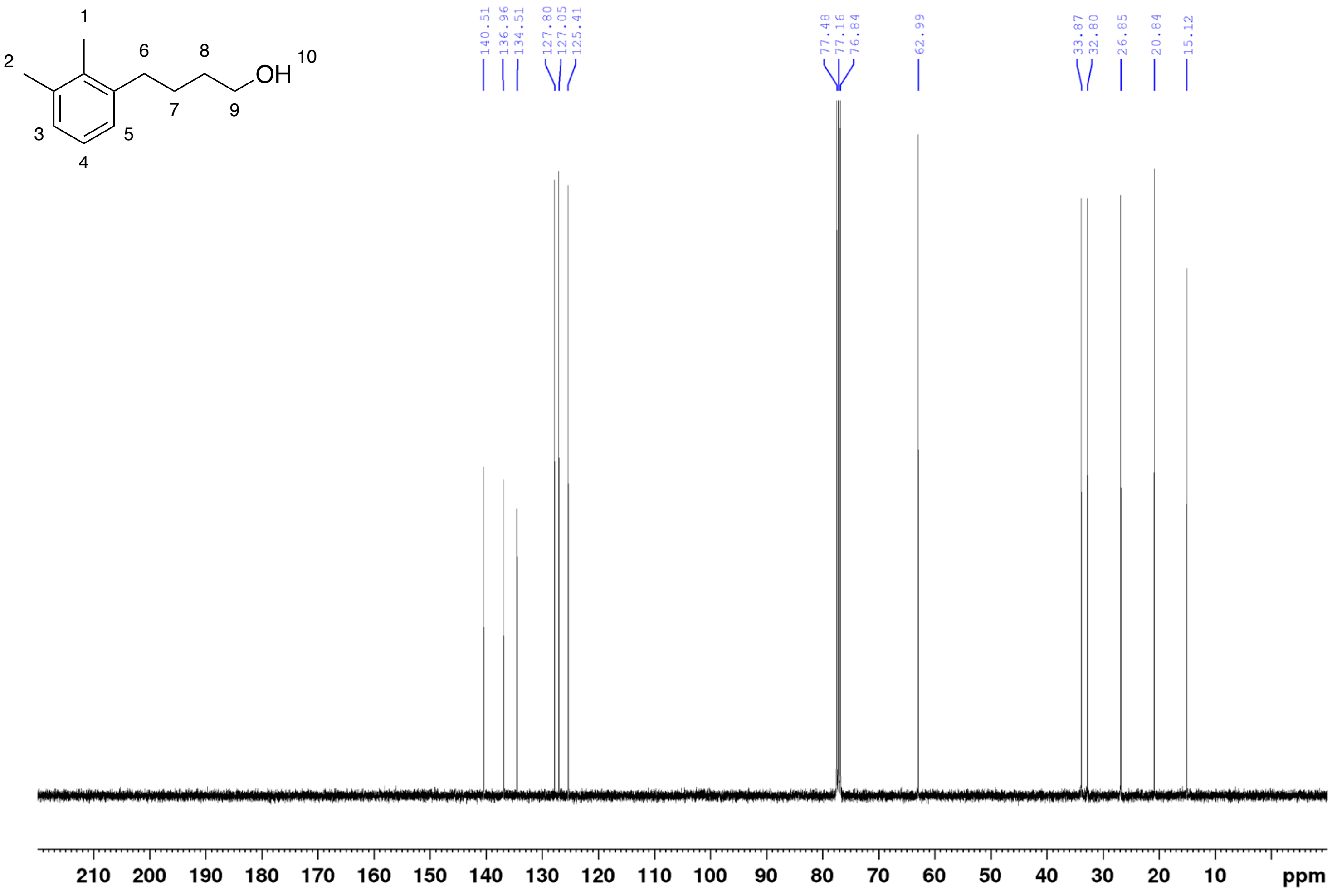

^{1}H NMR (400 MHz, $CDCl_3$) for *4-(4-fluoro-2-methylphenyl)butan-1-ol* (**6r**)

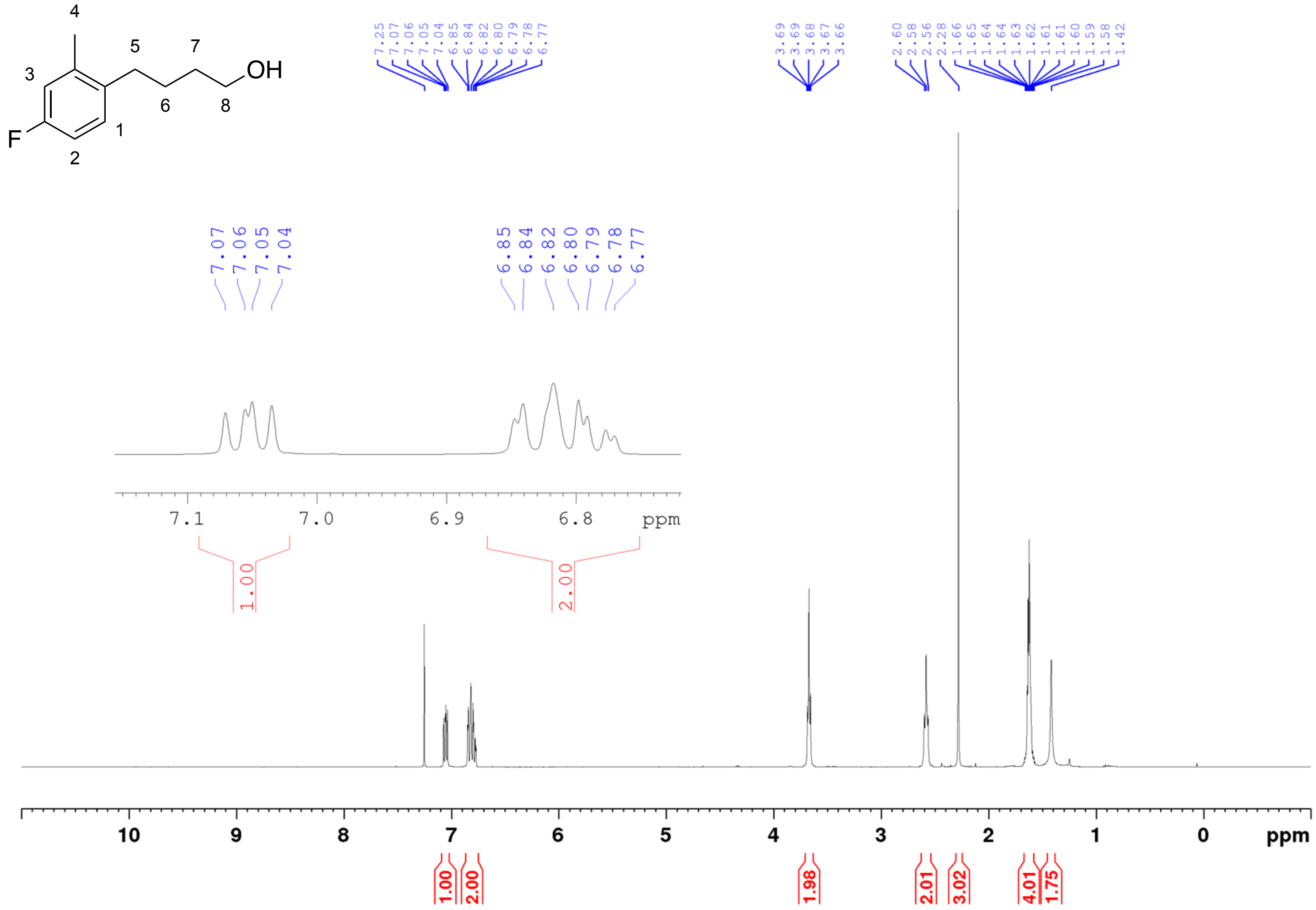

^{13}C NMR (101 MHz, $CDCl_3$) for *4-(4-fluoro-2-methylphenyl)butan-1-ol* (**6r**)

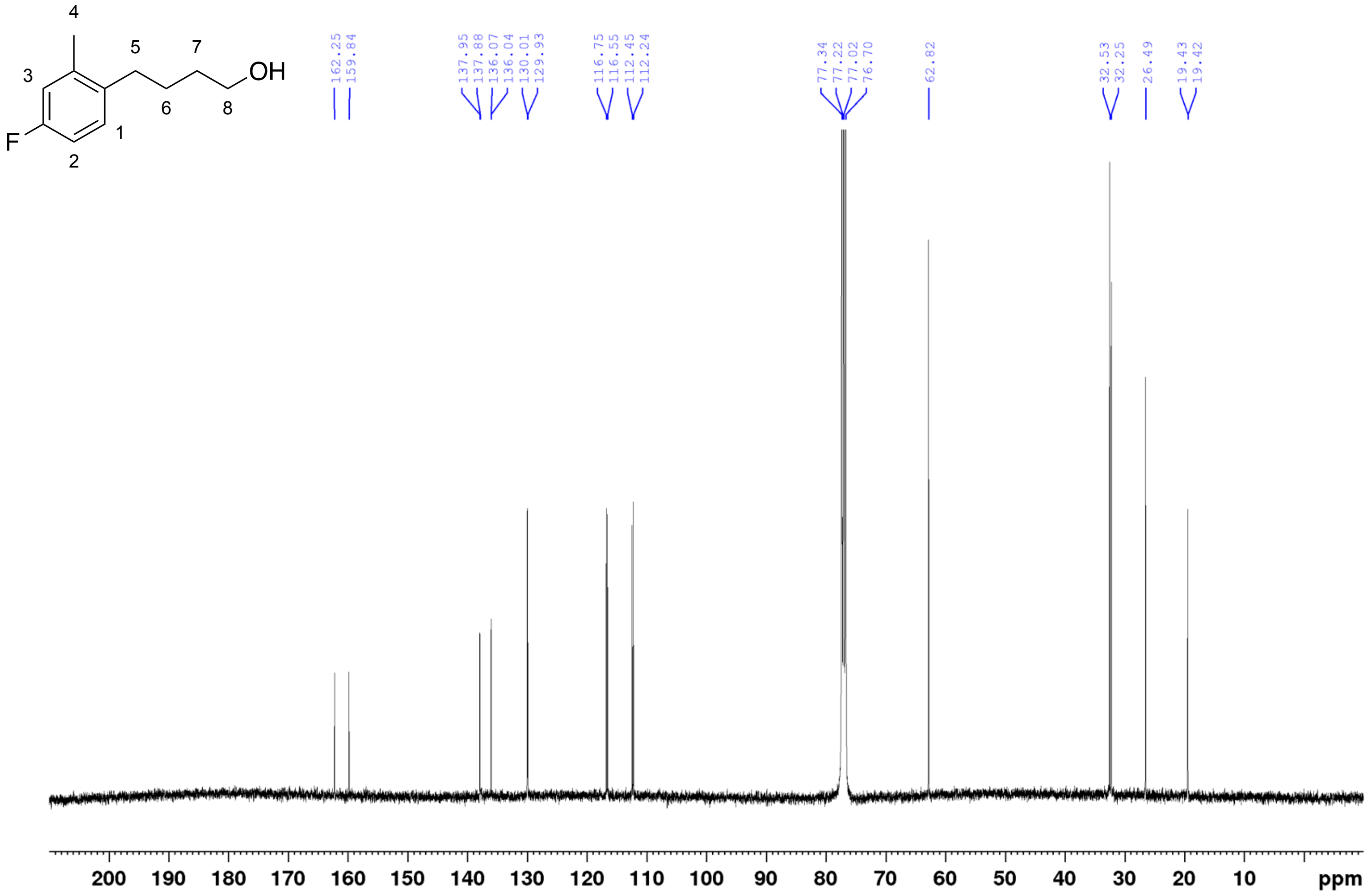

^{19}F NMR (376 MHz, $CDCl_3$) for *4-(4-fluoro-2-methylphenyl)butan-1-ol* (**6r**)

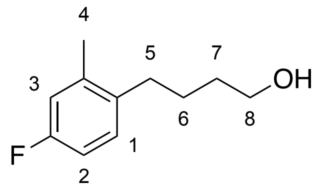

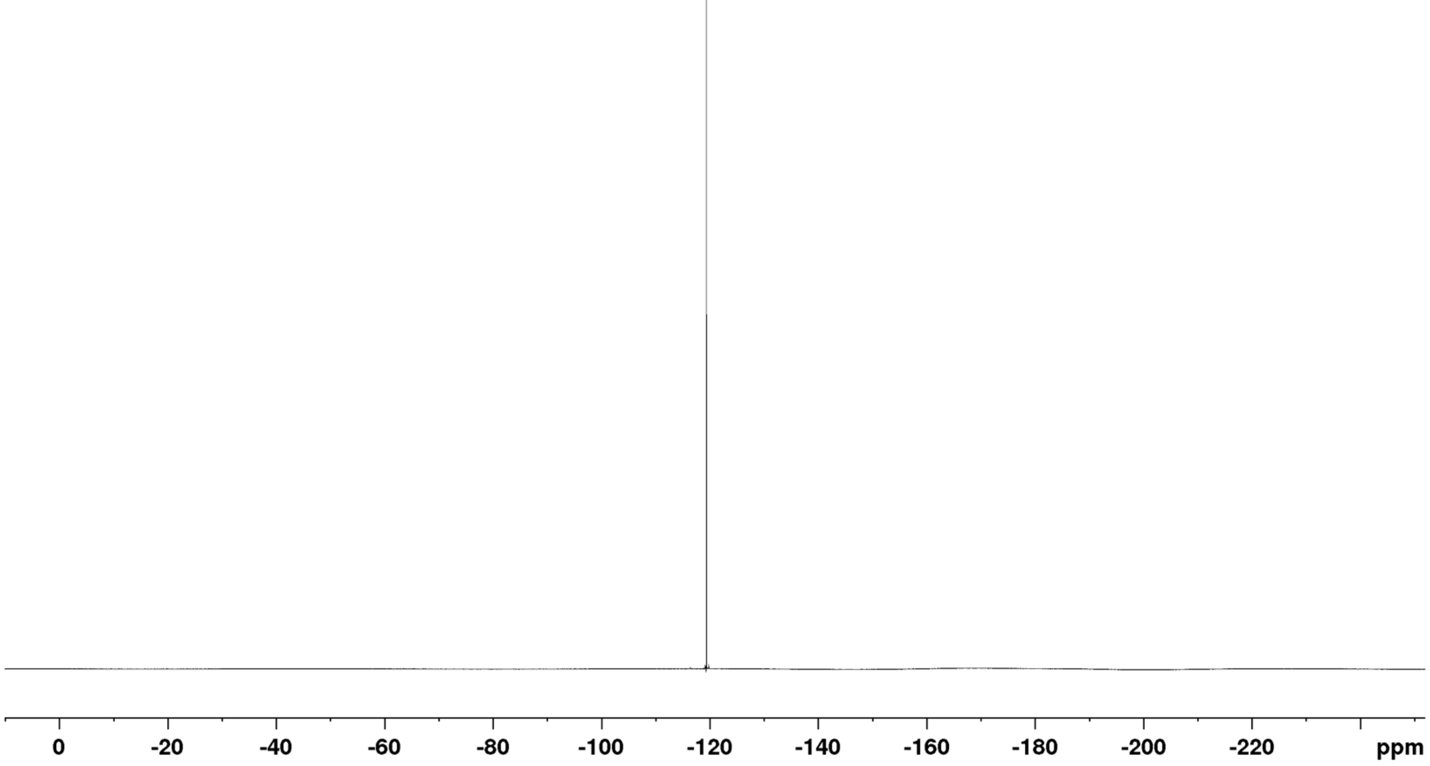

^{1}H NMR (400 MHz, $CDCl_3$) for *4-(2,5-dimethylphenyl)butan-1-ol* (**6s**)

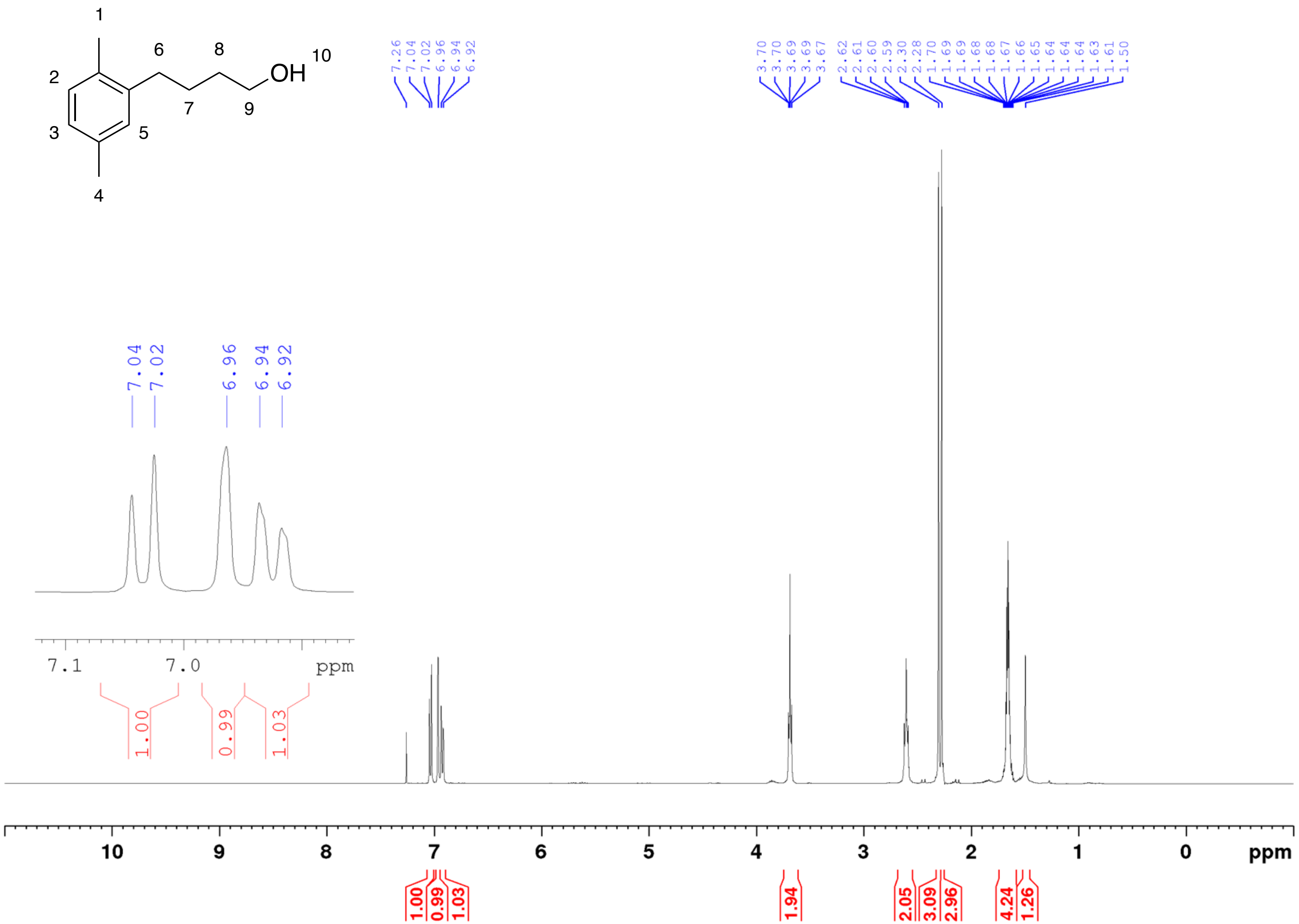

^{13}C NMR (101 MHz, $CDCl_3$) for *4-(2,5-dimethylphenyl)butan-1-ol* (**6s**)

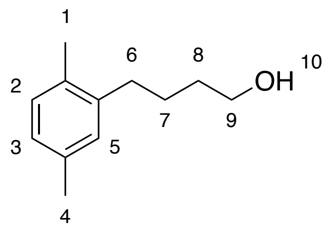

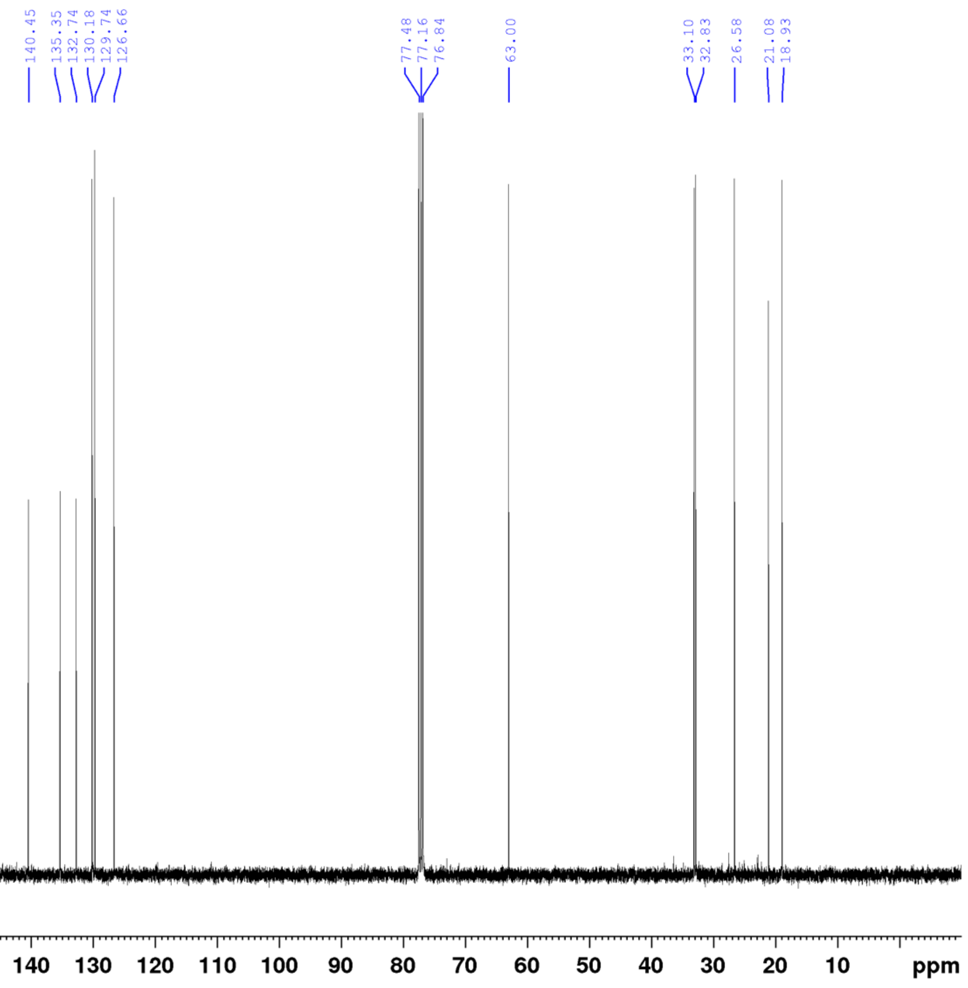

¹H NMR (400 MHz, $CDCl_3$) for *4-(2,4-difluorophenyl)but-3-yn-1-ol*

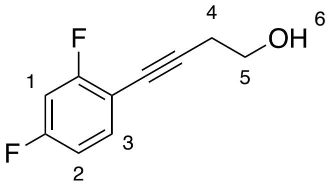

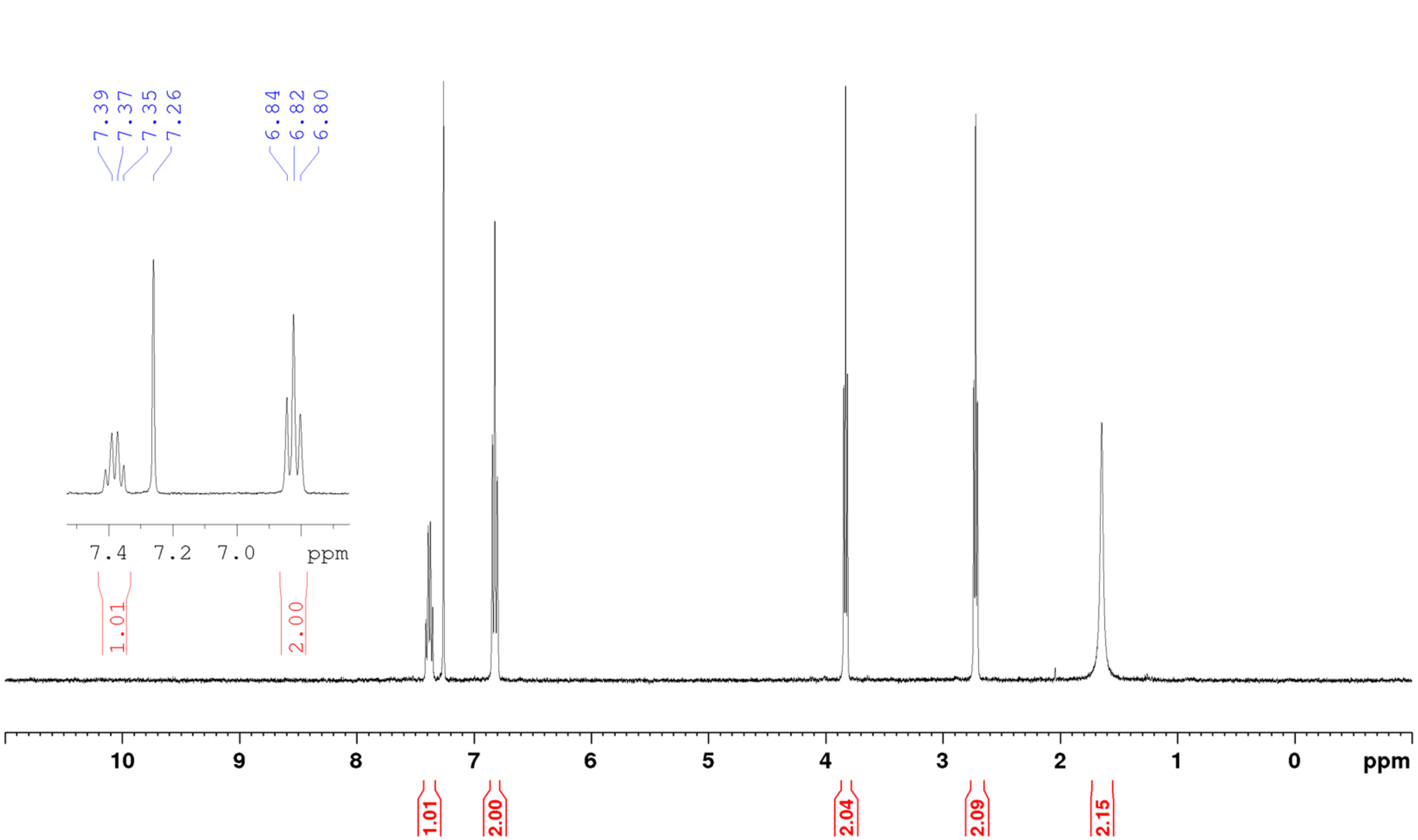

^{13}C NMR (101 MHz, $CDCl_3$) for *4-(2,4-difluorophenyl)but-3-yn-1-ol*

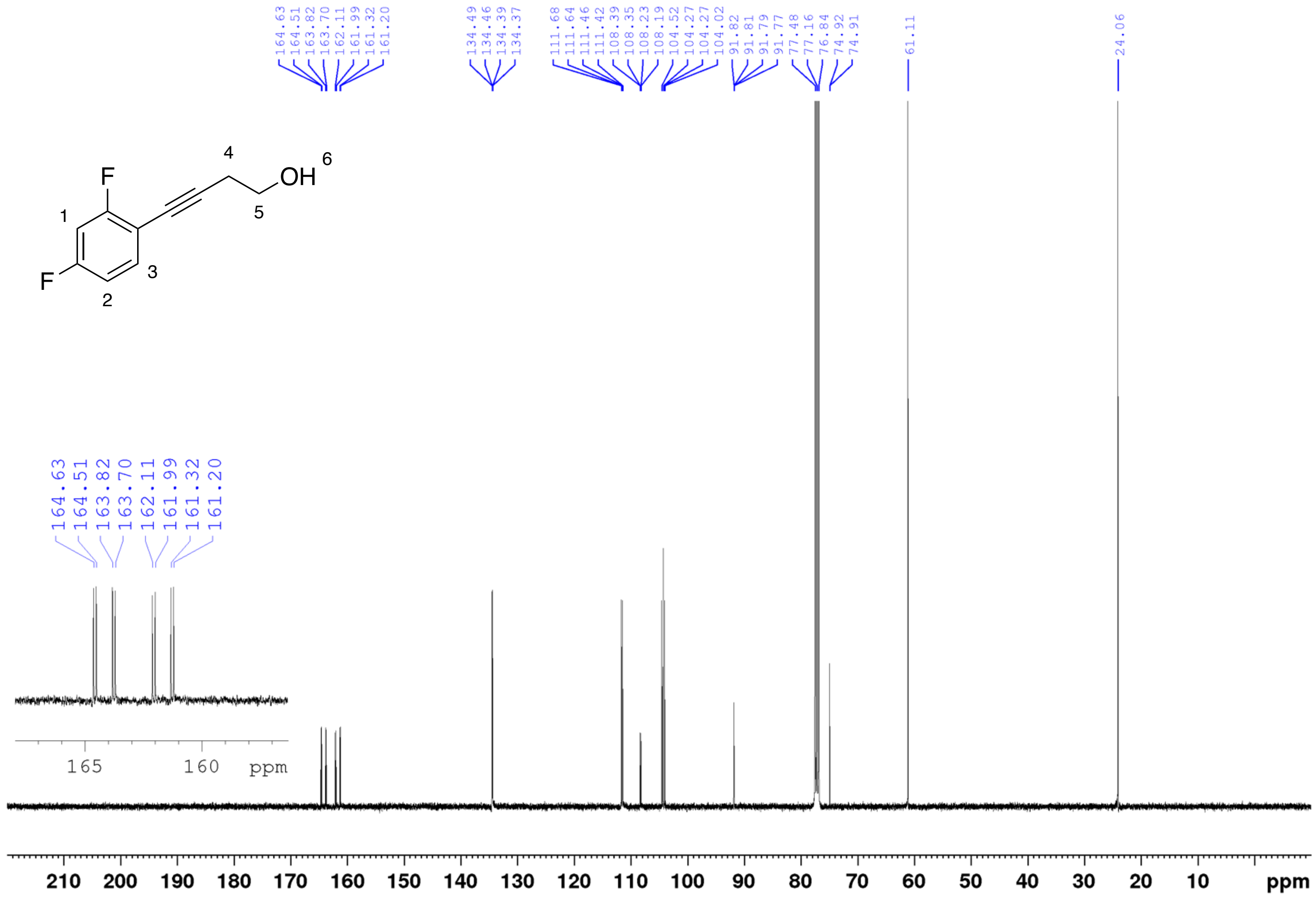

^{19}F NMR (376 MHz, $CDCl_3$) for *4-(2,4-difluorophenyl)but-3-yn-1-ol*

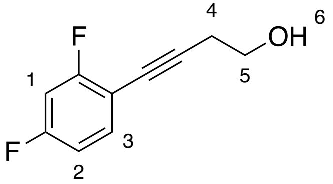

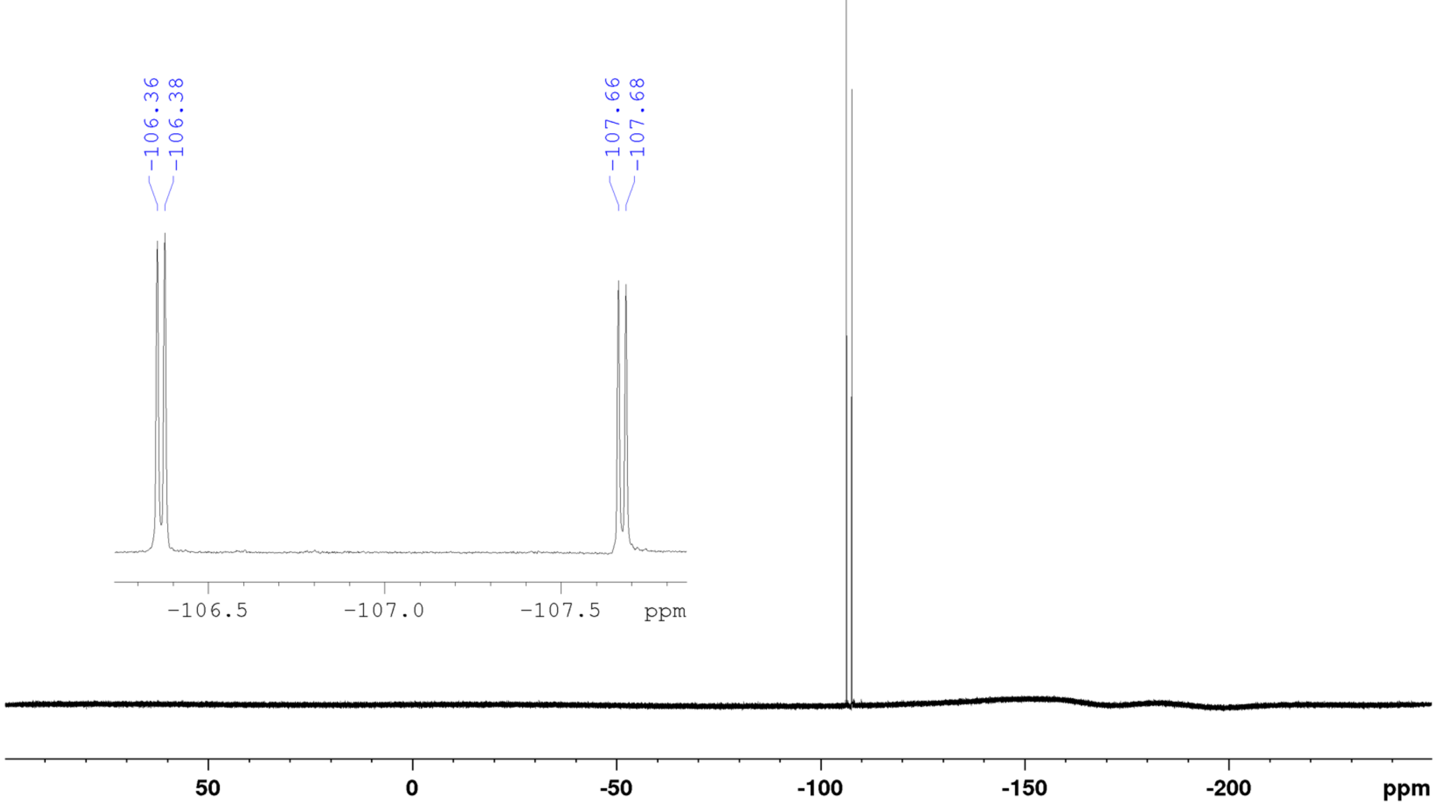

^{1}H NMR (400 MHz, $CDCl_3$) for *4-(2,4-difluorophenyl)butan-1-ol* (**6t**)

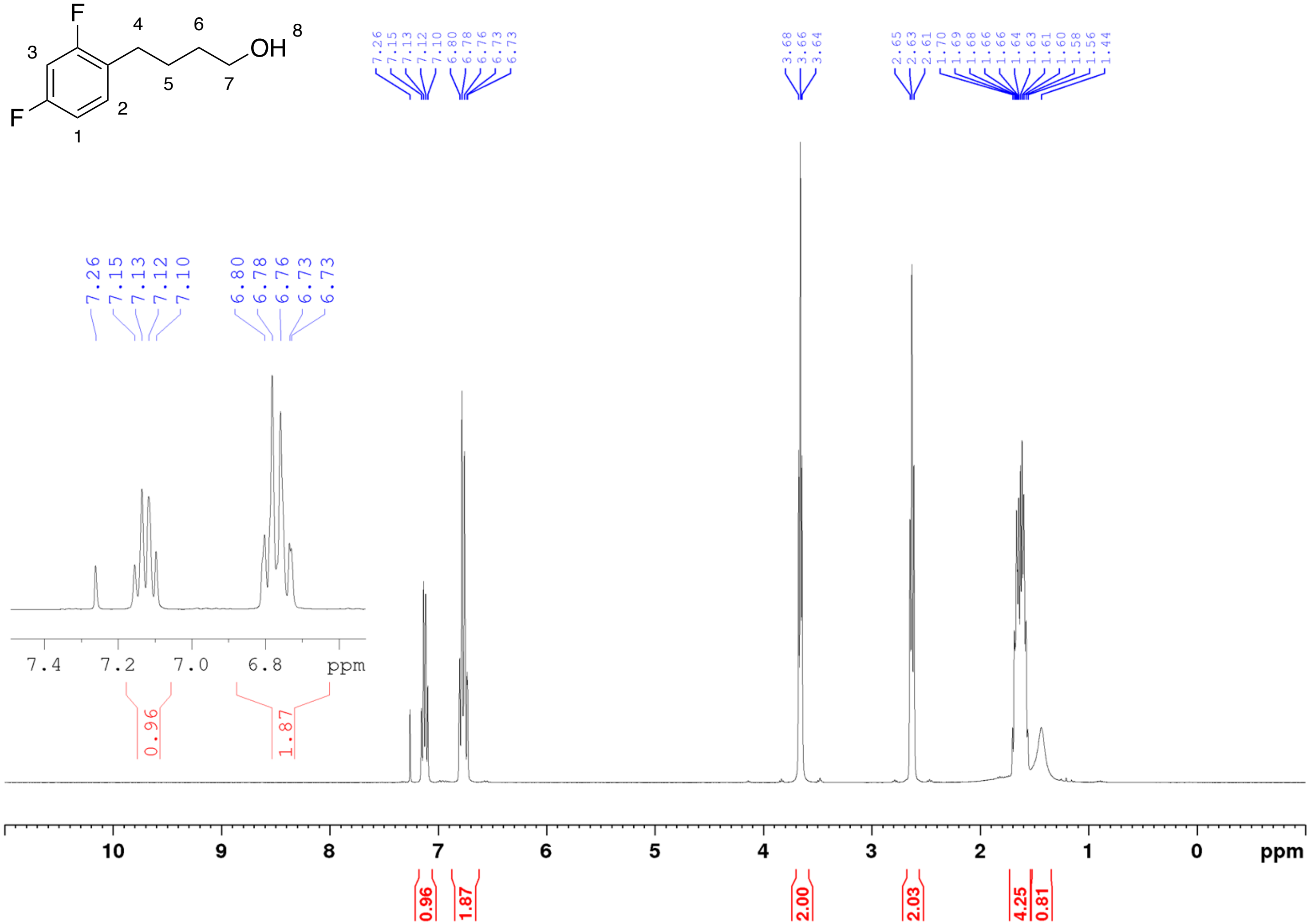

^{13}**C NMR** (101 MHz, $CDCl_3$) for *4-(2,4-difluorophenyl)butan-1-ol* (**6t**)

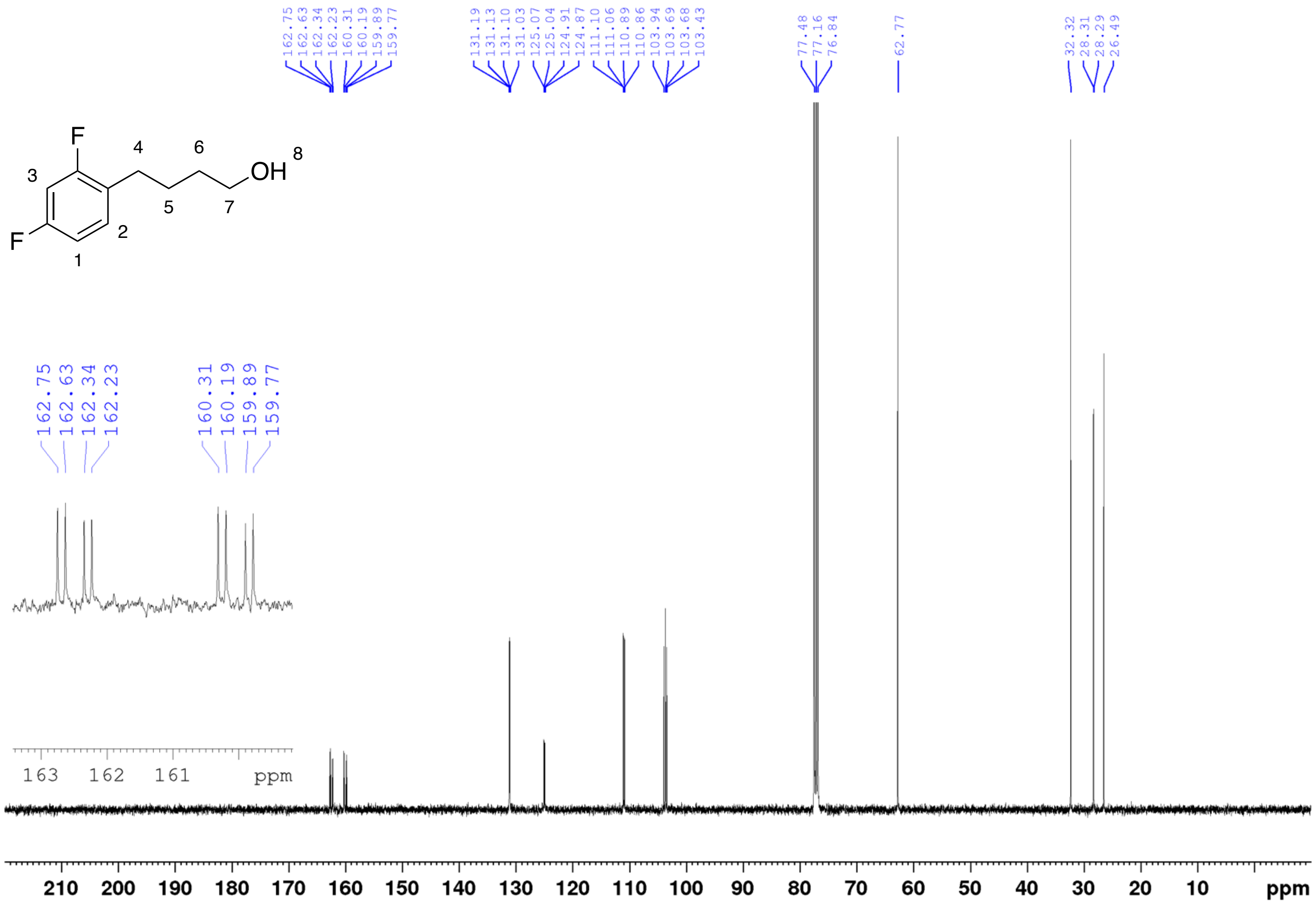

^{19}F NMR (376 MHz, $CDCl_3$) for *4-(2,4-difluorophenyl)butan-1-ol* (**6t**)

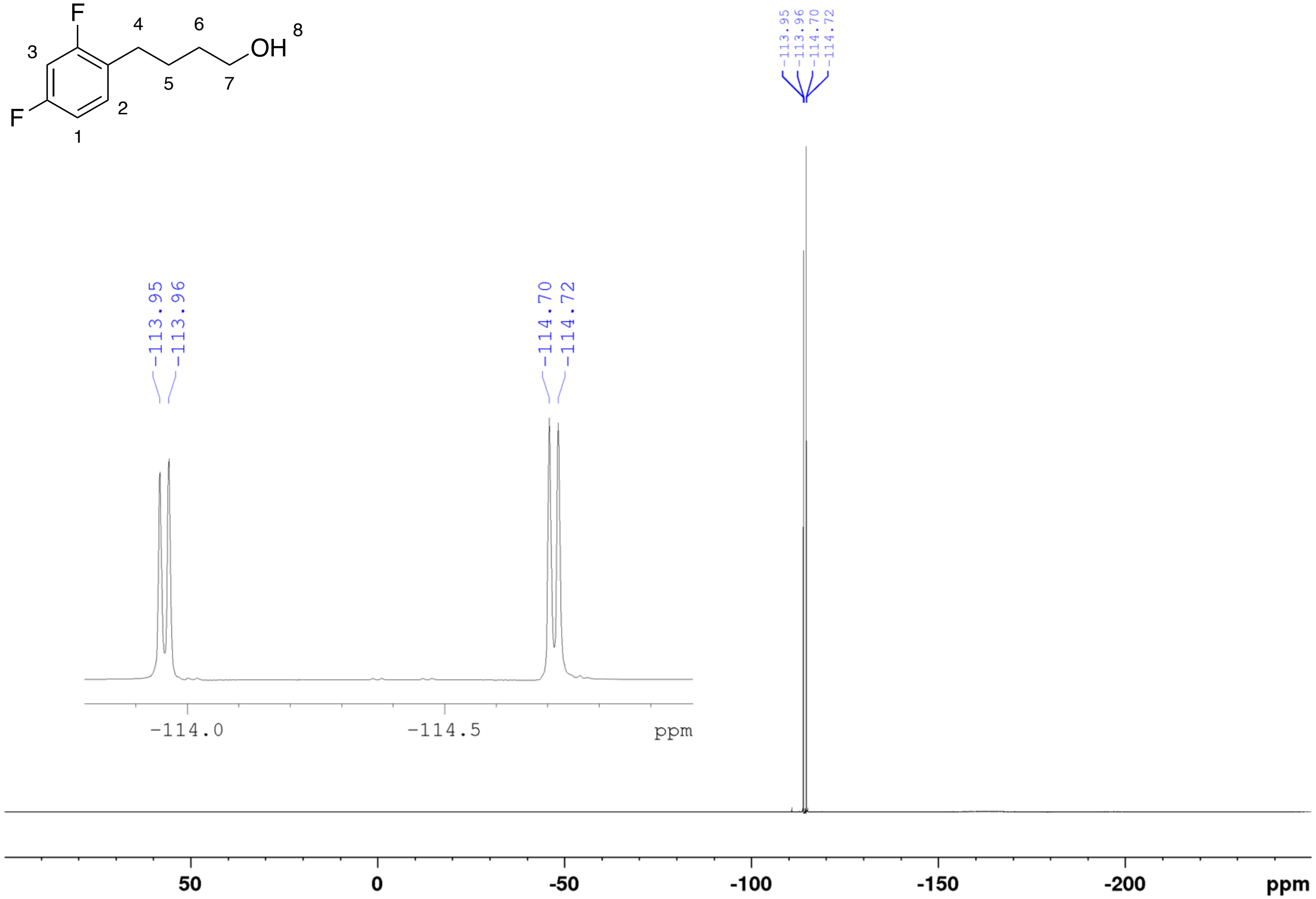

¹H NMR (400 MHz, $CDCl_3$) for *4-(3-bromo-2-methylphenyl)butan-1-ol* (**6u**)

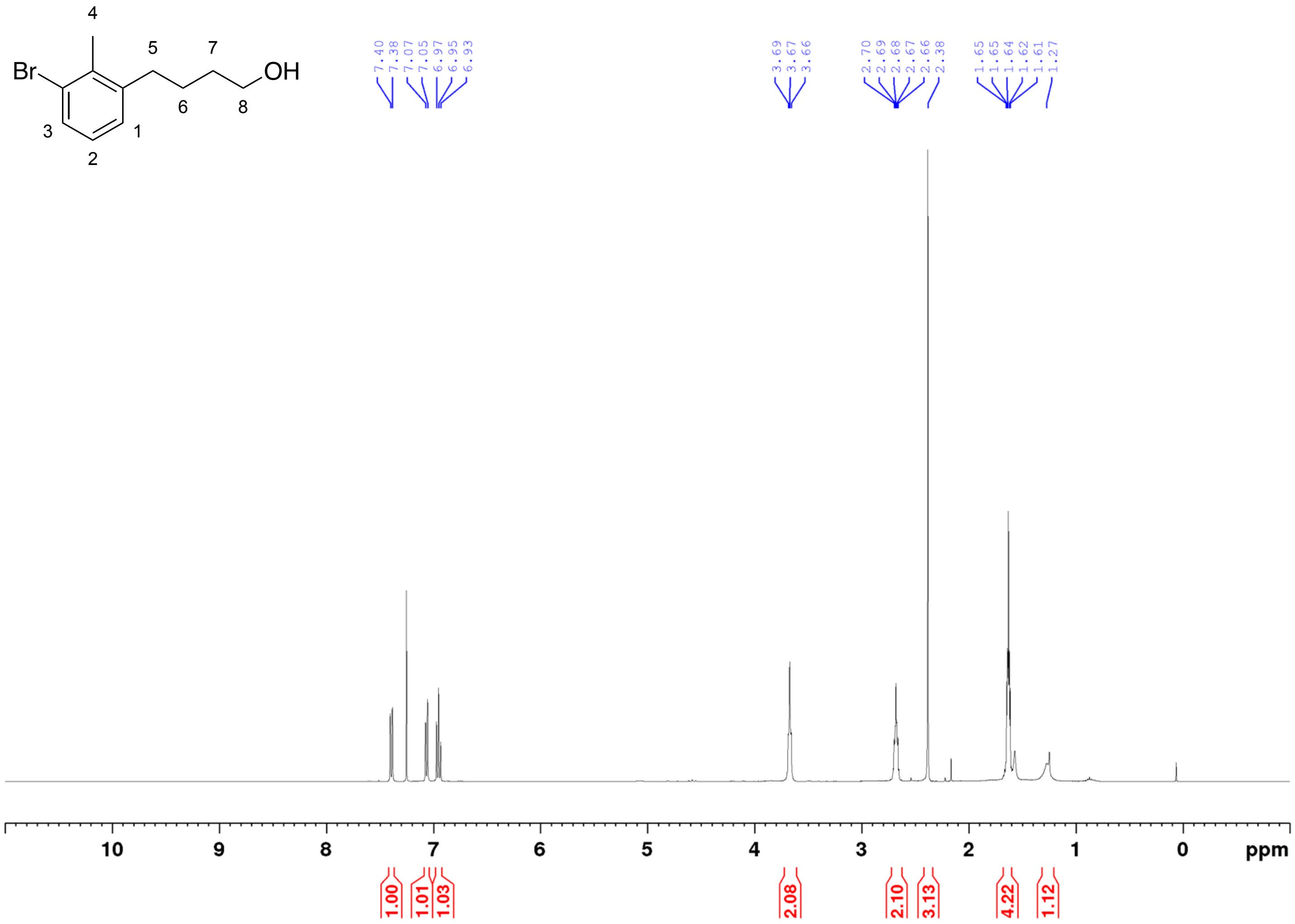

^{13}C NMR (101 MHz, $CDCl_3$) for *4-(3-bromo-2-methylphenyl)butan-1-ol* (**6u**)

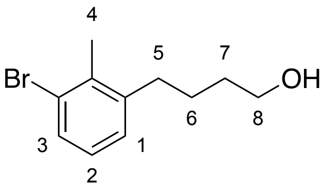

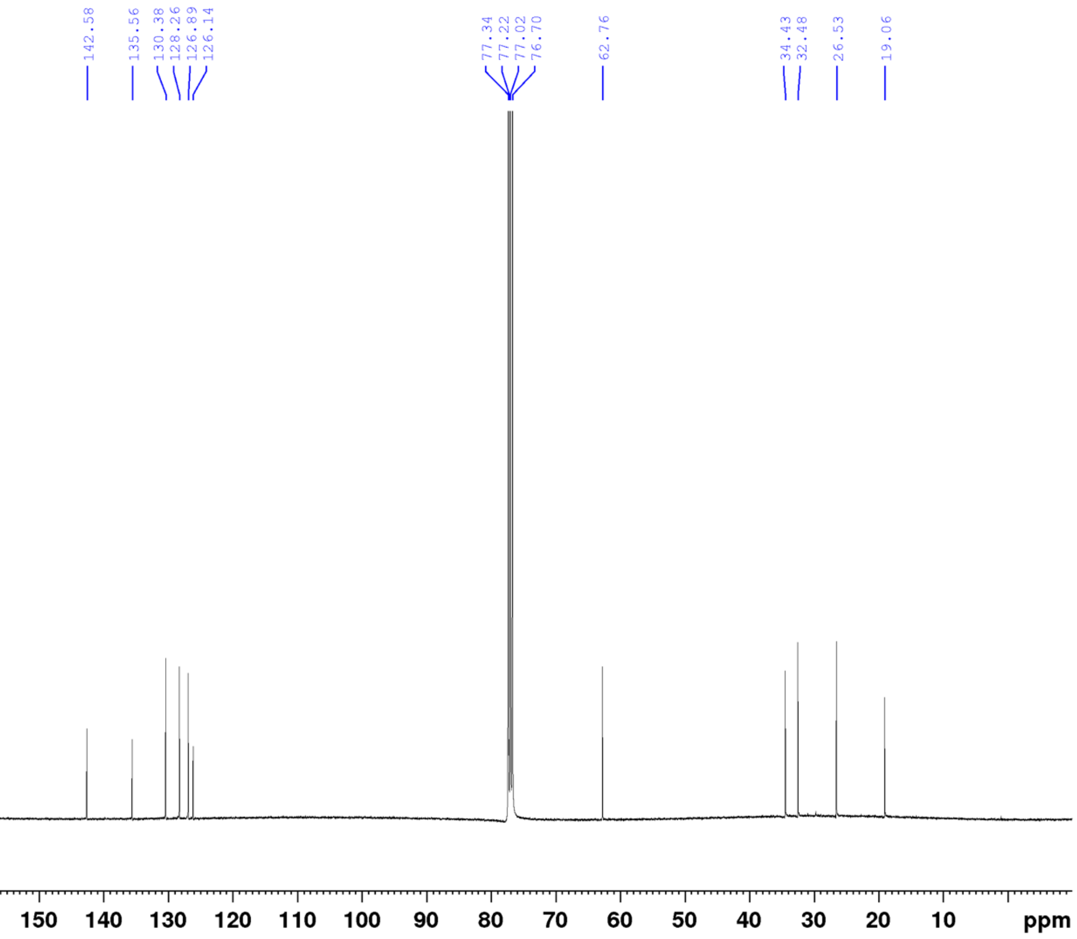

¹H NMR (400 MHz, $CDCl_3$) for *(3-chloro-2-methylphenyl)methanol*

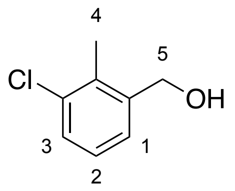

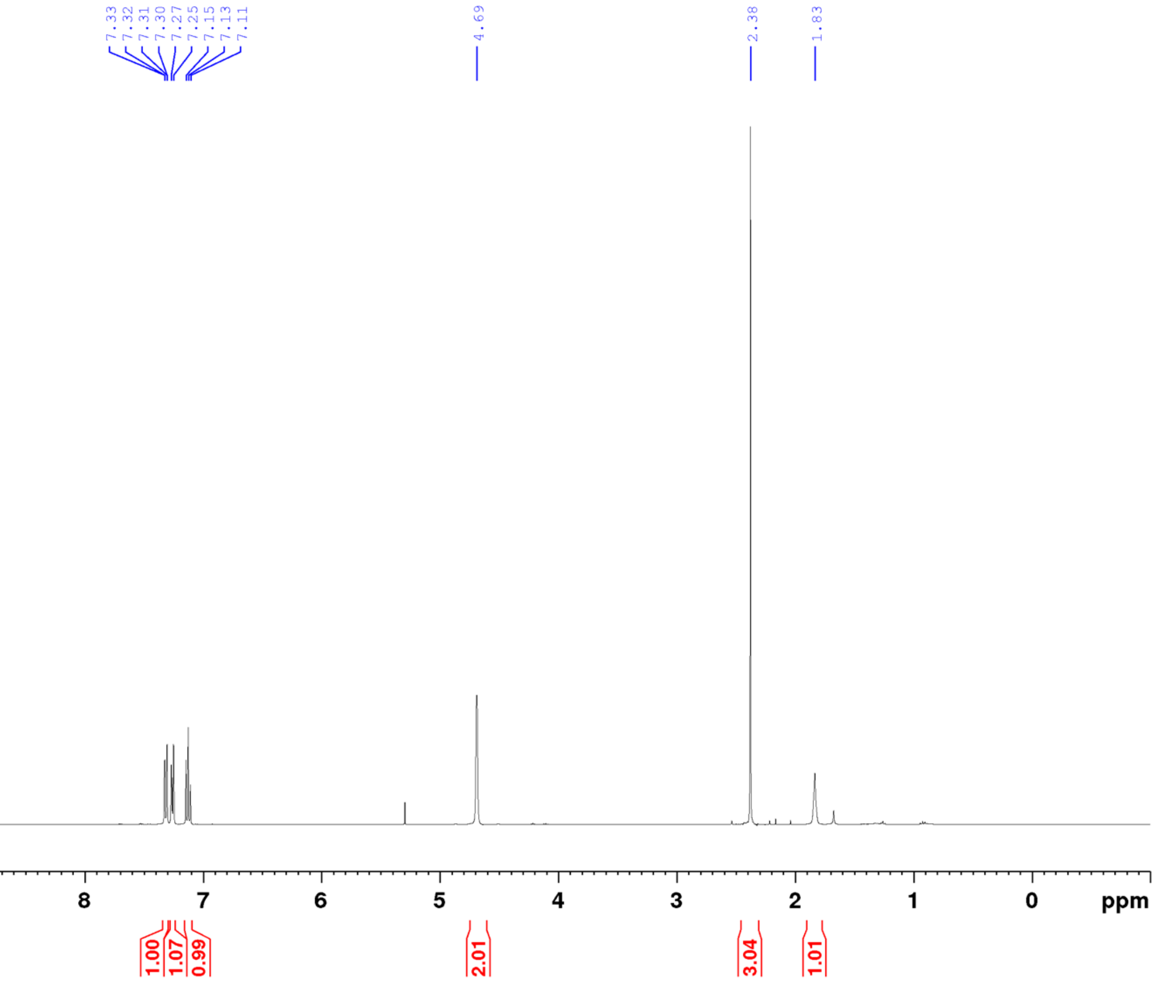

^{13}C NMR (101 MHz, $CDCl_3$) for *(3-chloro-2-methylphenyl)methanol*

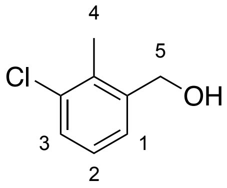

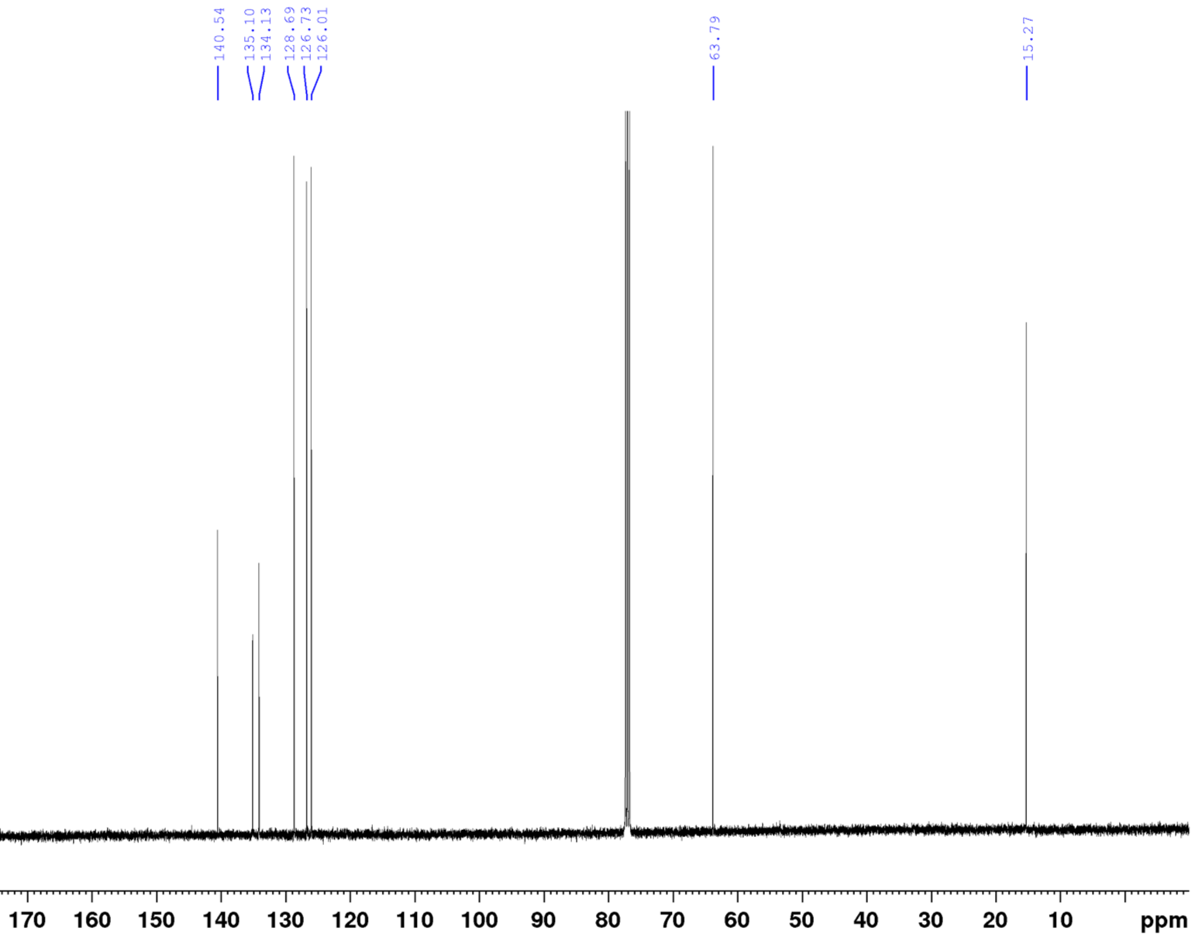

¹H NMR (400 MHz, $CDCl_3$) for *4-(3-chloro-2-methylphenyl)butan-1-ol* (**6v**)

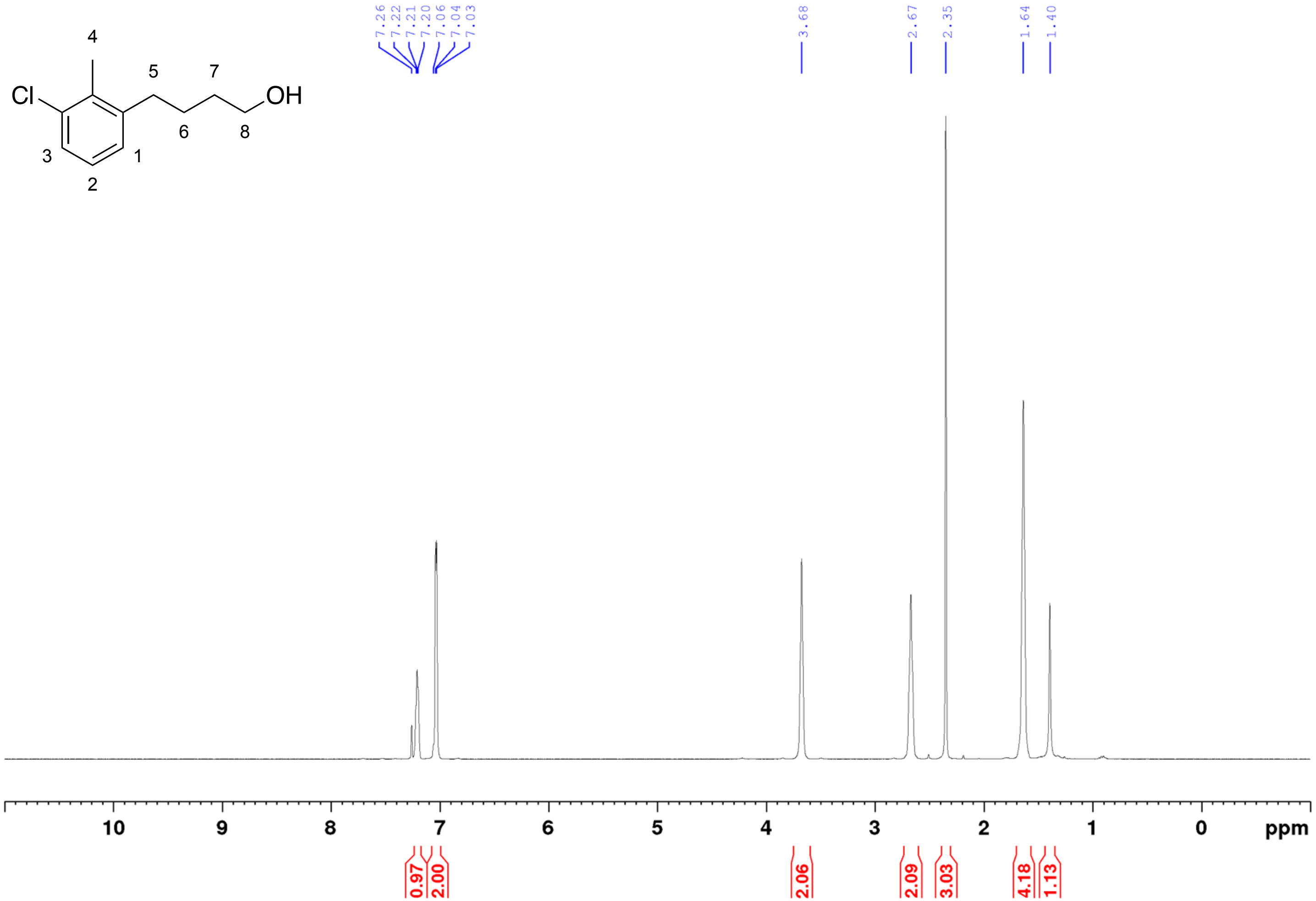

^{13}C NMR (101 MHz, $CDCl_3$) for *4-(3-chloro-2-methylphenyl)butan-1-ol* (**6v**)

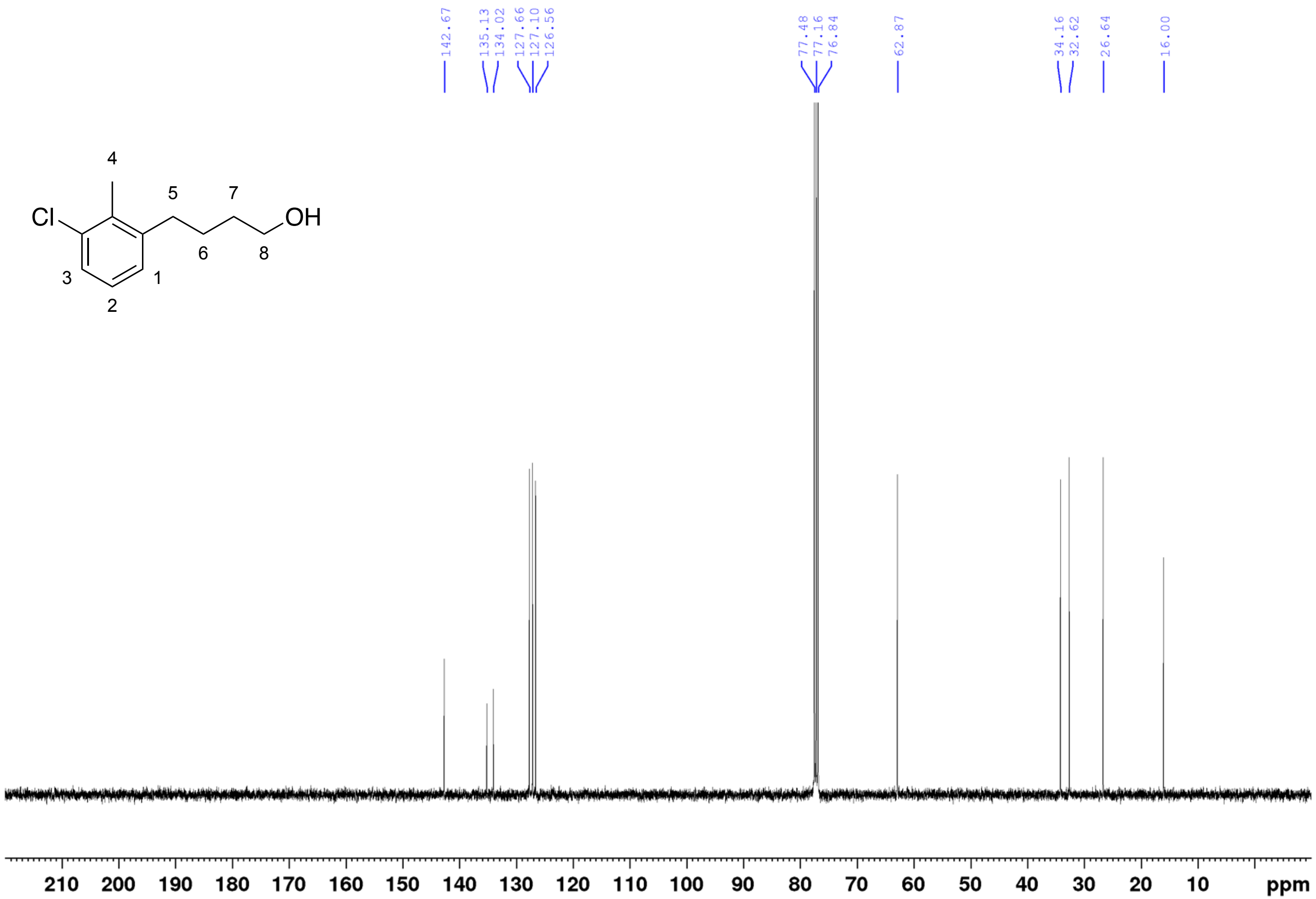

^{1}H NMR (400 MHz, $CDCl_3$) for *4-(naphthalen-1-yl)butan-1-ol* (**6w**)

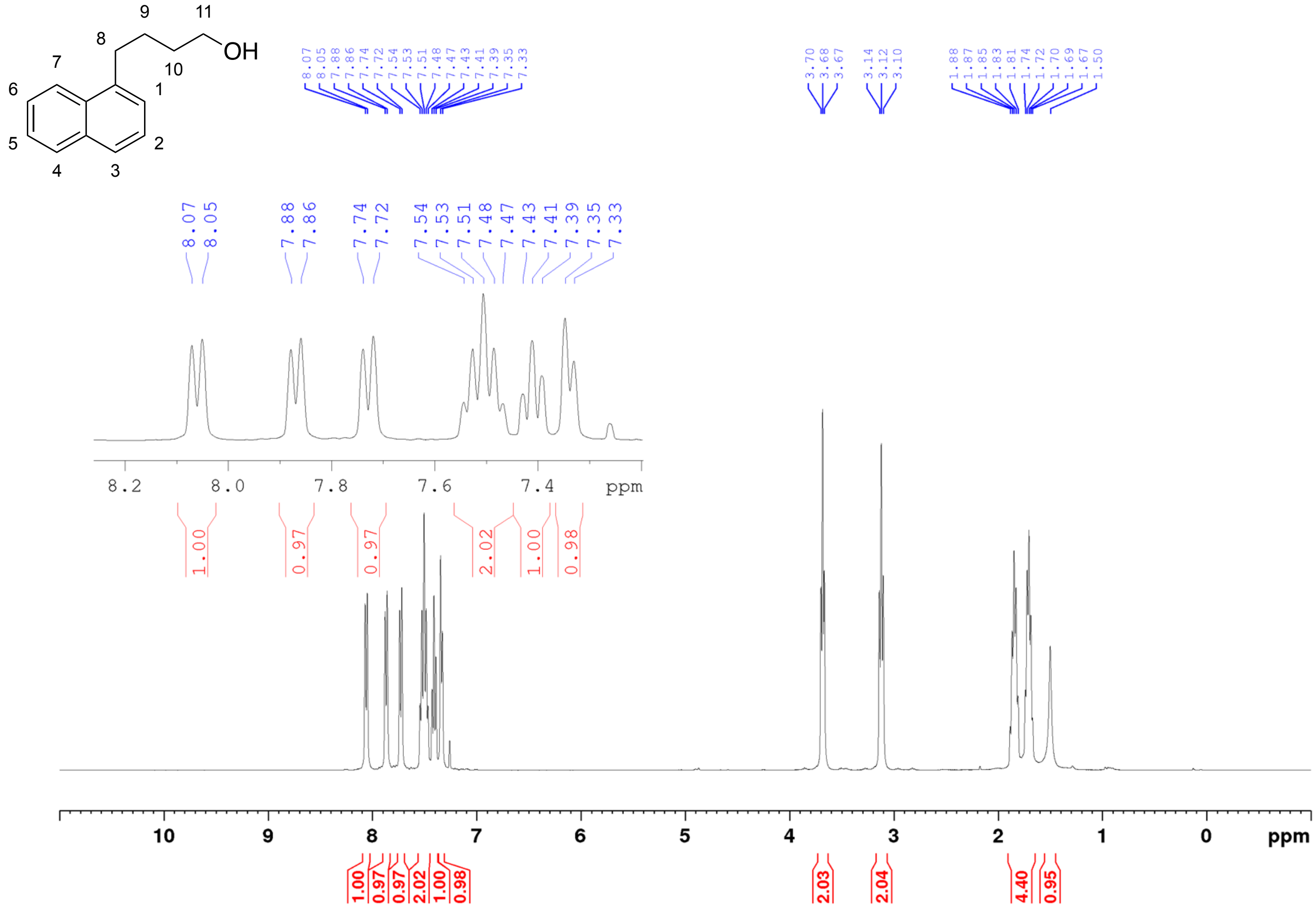

^{13}**C NMR** (101 MHz, $CDCl_3$) for *4-(naphthalen-1-yl)butan-1-ol* (**6w**)

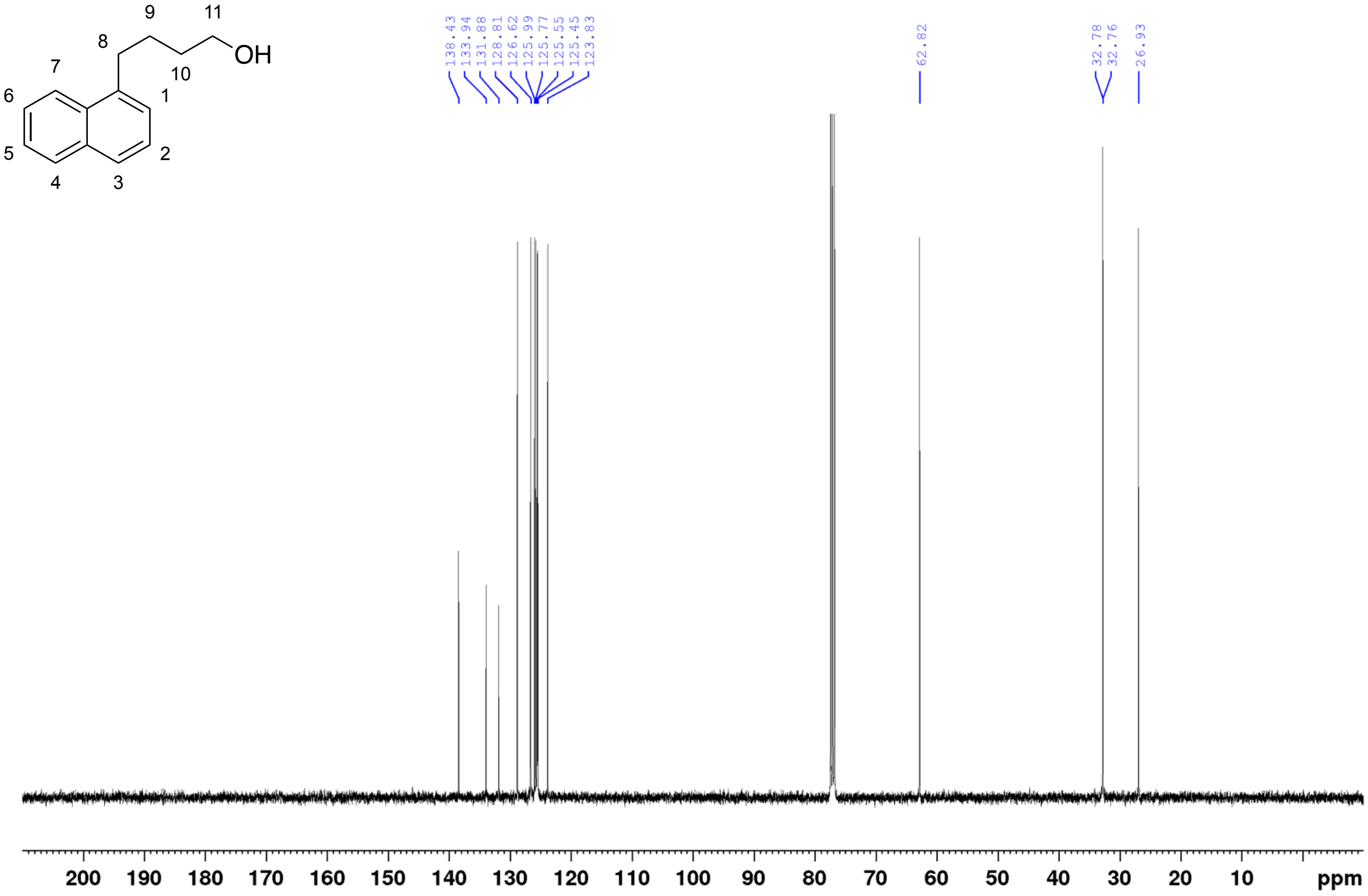

^{1}H NMR (400 MHz, $CDCl_3$) for *4-(3-methylthiophen-2-yl)but-3-yn-1-ol*

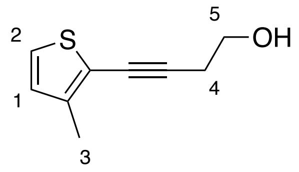

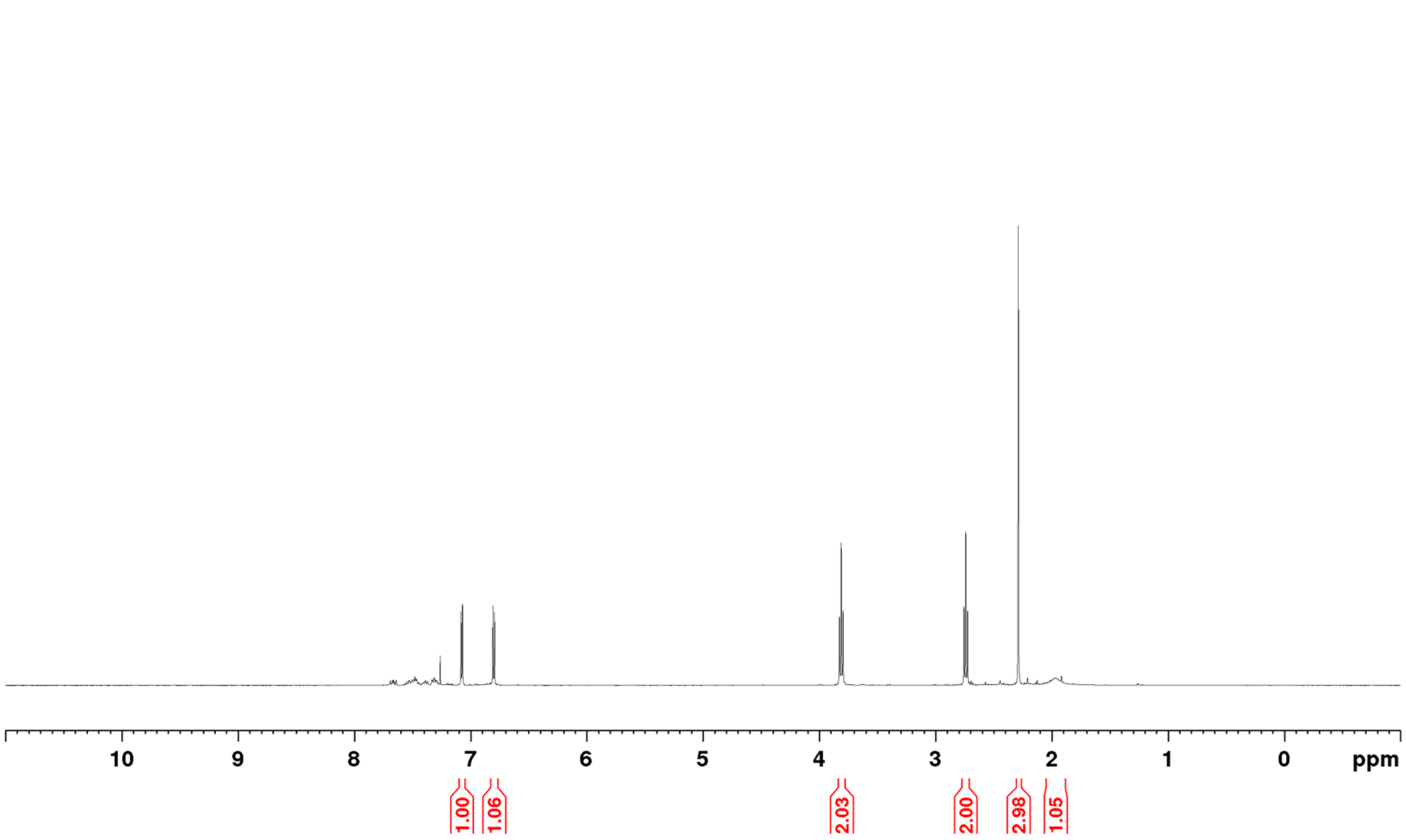

^{13}C NMR (101 MHz, $CDCl_3$) for *4-(3-methylthiophen-2-yl)but-3-yn-1-ol*

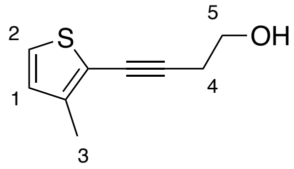

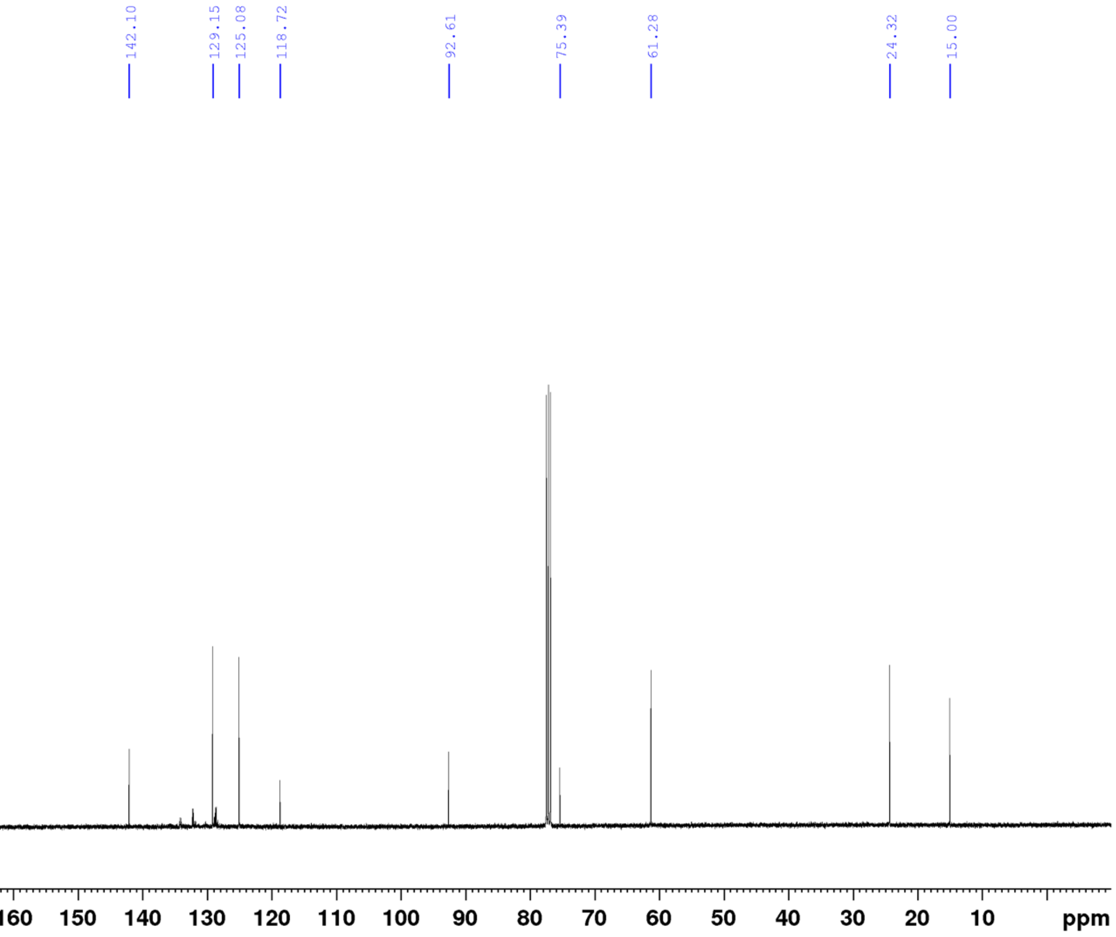

¹H NMR (500 MHz, $CDCl_3$) for *4-(3-methylthiophen-2-yl)butan-1-ol* (**6x**)

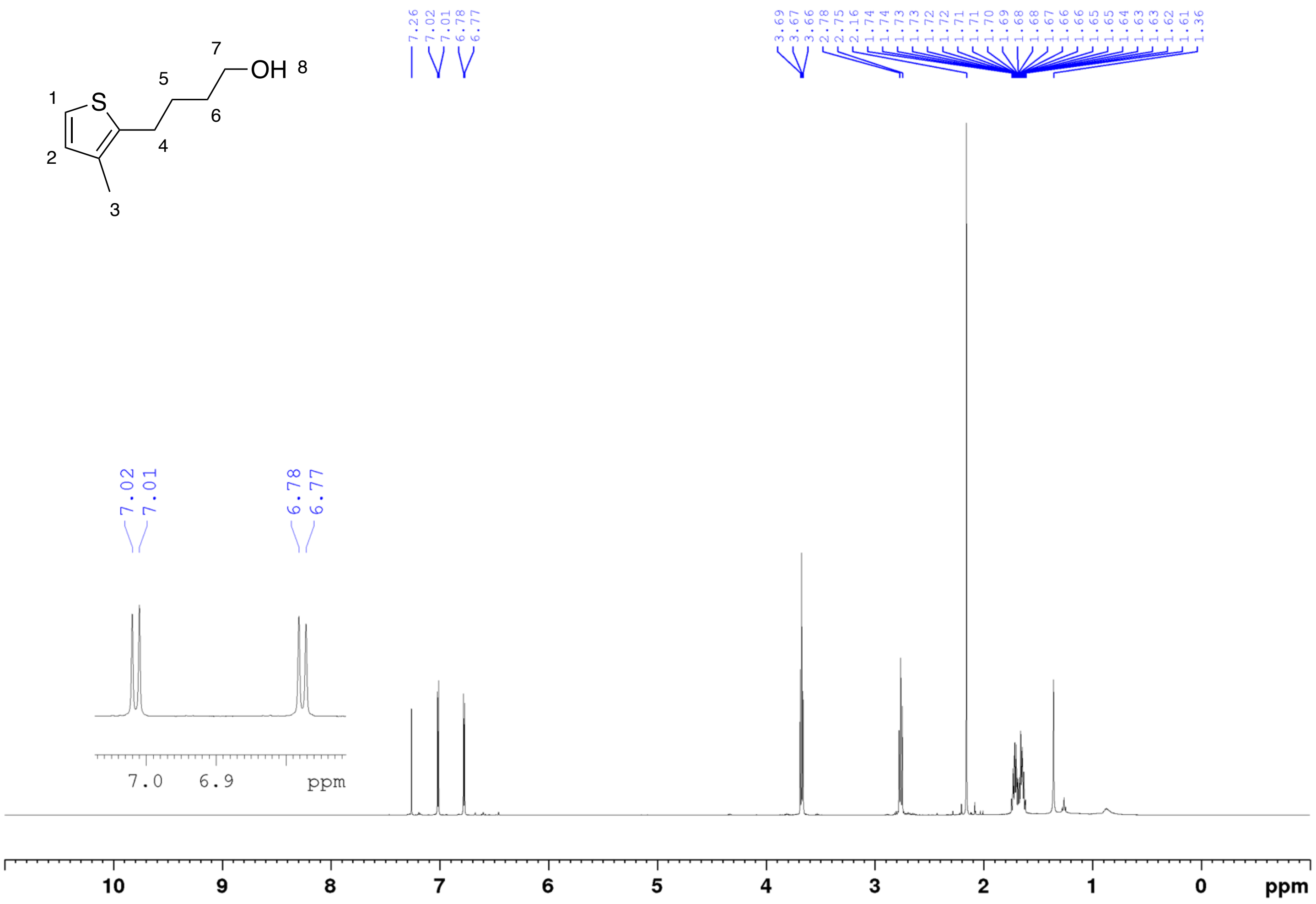

^{13}C NMR (126 MHz, $CDCl_3$) for *4-(3-methylthiophen-2-yl)butan-1-ol* (**6x**)

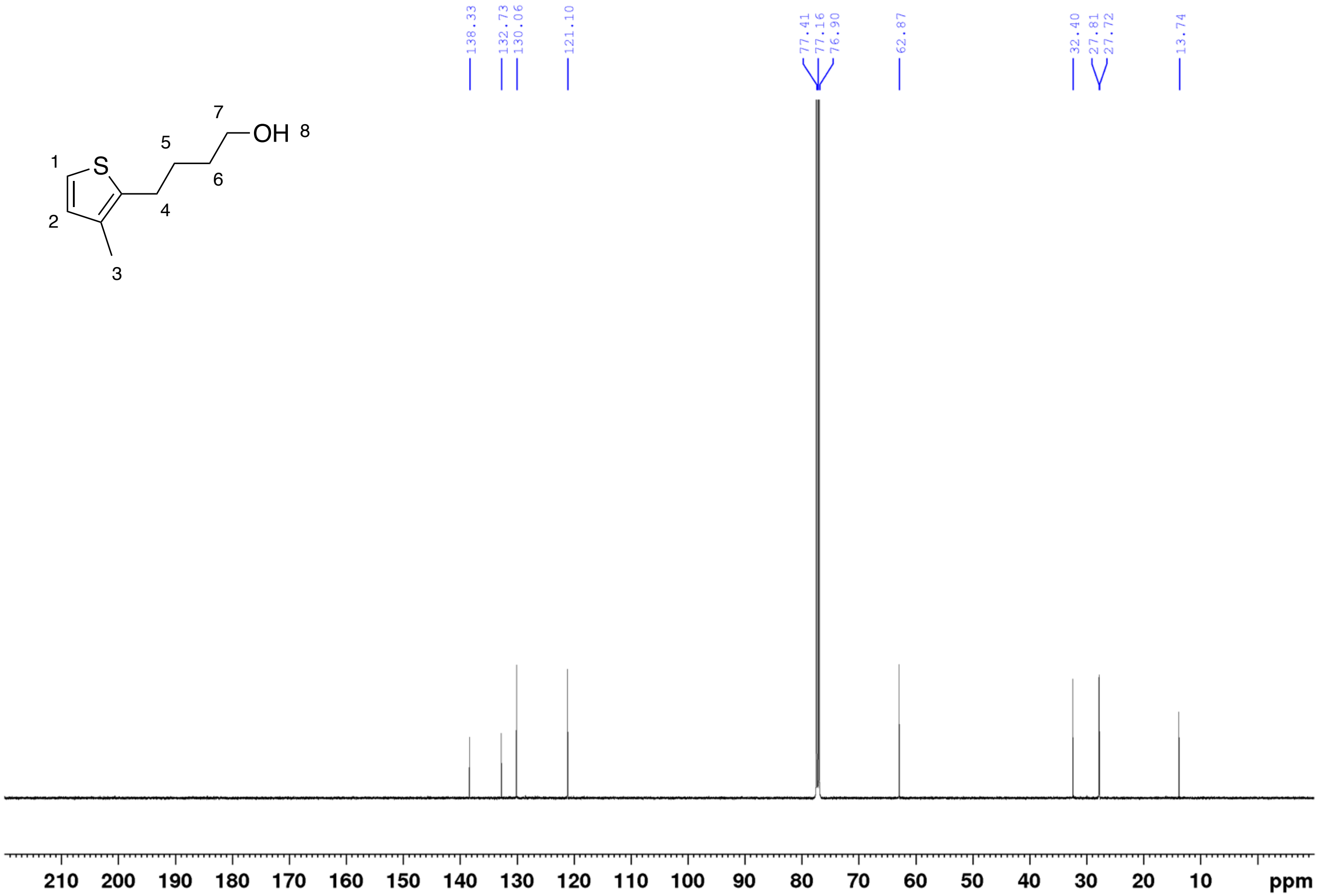

^{1}H NMR (400 MHz, $CDCl_3$) for *N-(4-phenylbutyl)acetamide*

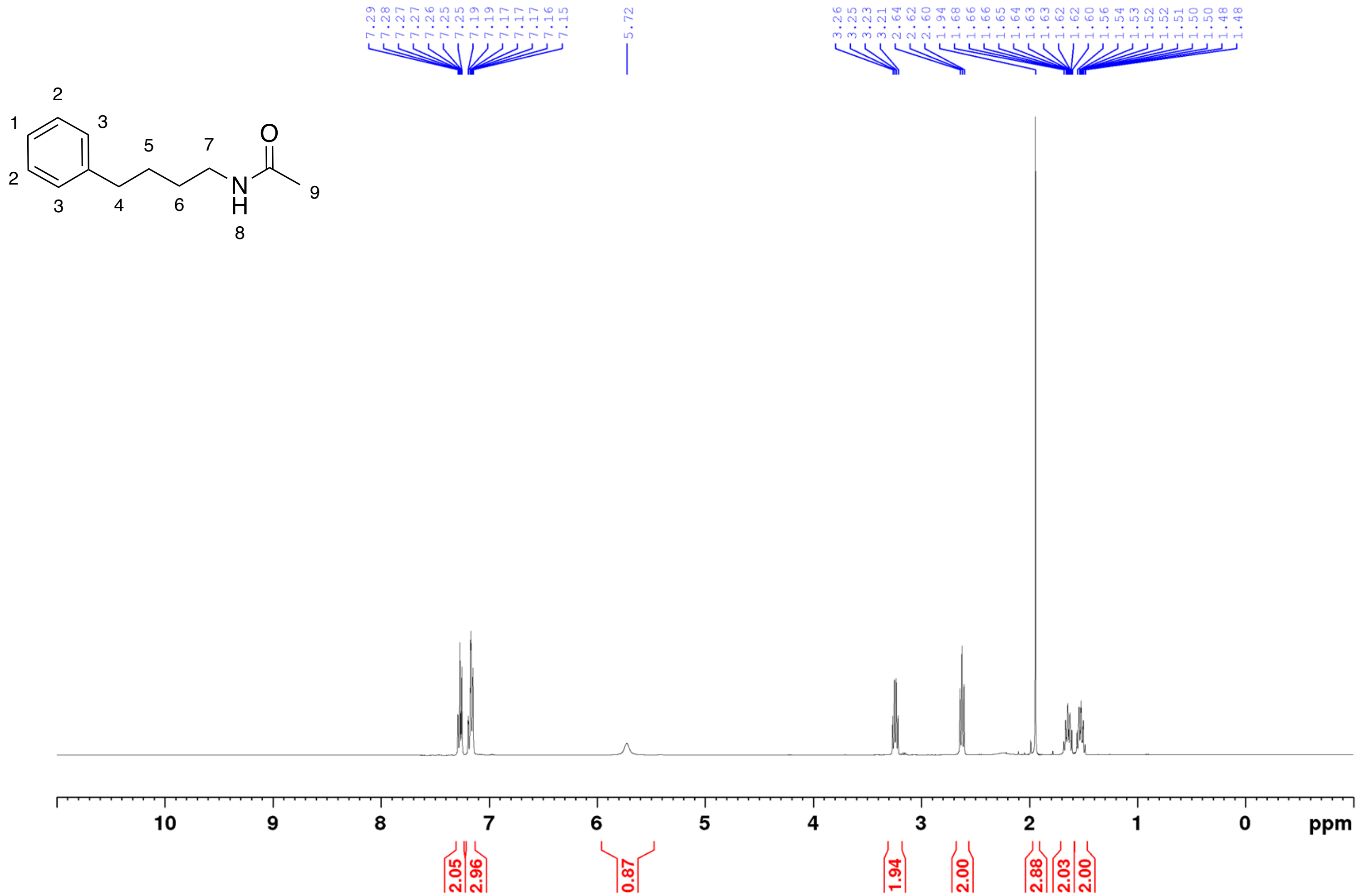

^{13}C NMR (101 MHz, $CDCl_3$) for *N-(4-phenylbutyl)acetamide*

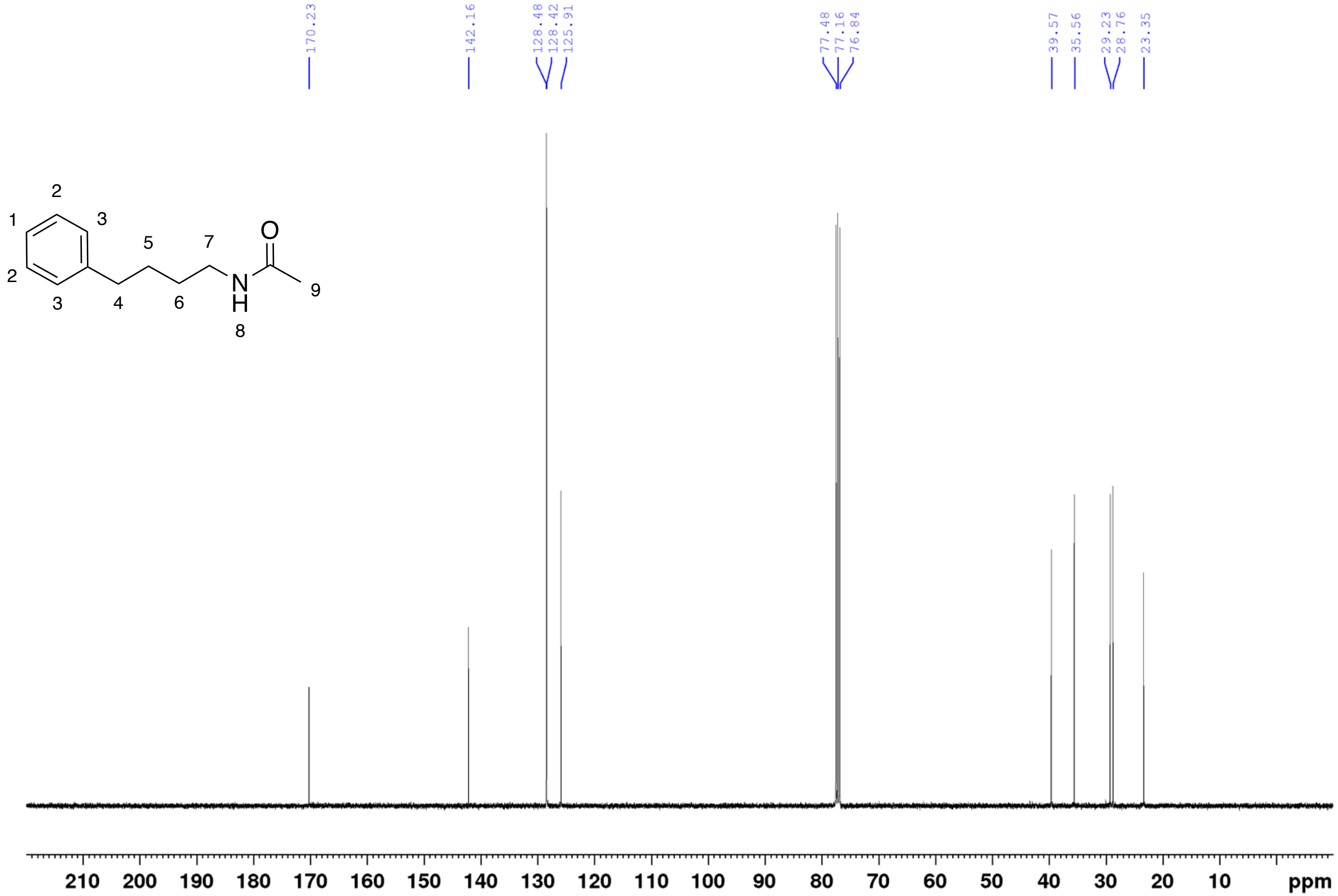

^{1}H NMR (400 MHz, $CDCl_3$) for *4-Phenylbutane-1-thiol*

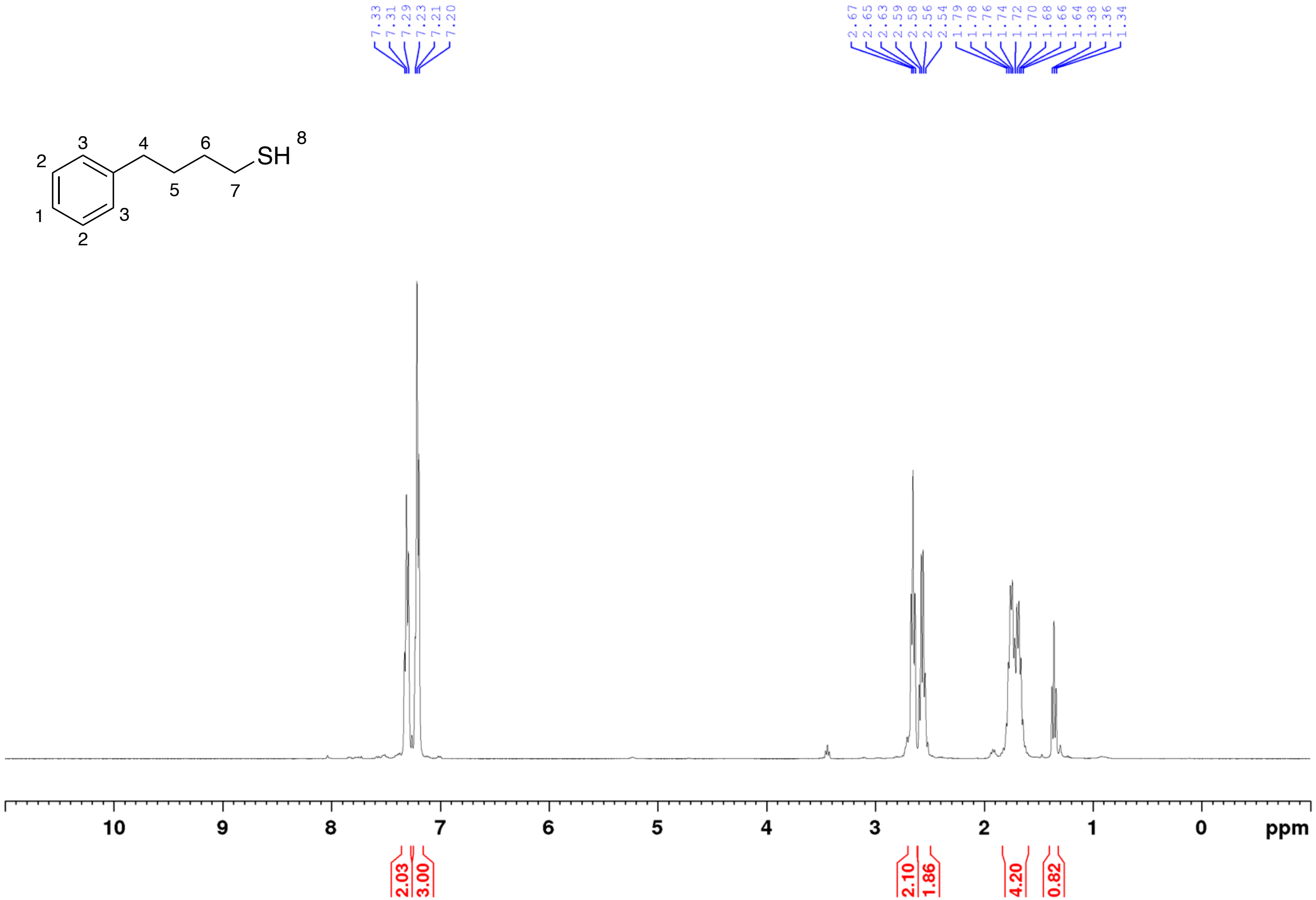

^{13}C NMR (101 MHz, $CDCl_3$) for *4-Phenylbutane-1-thiol*

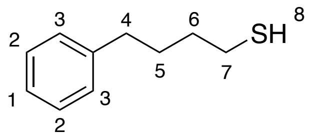

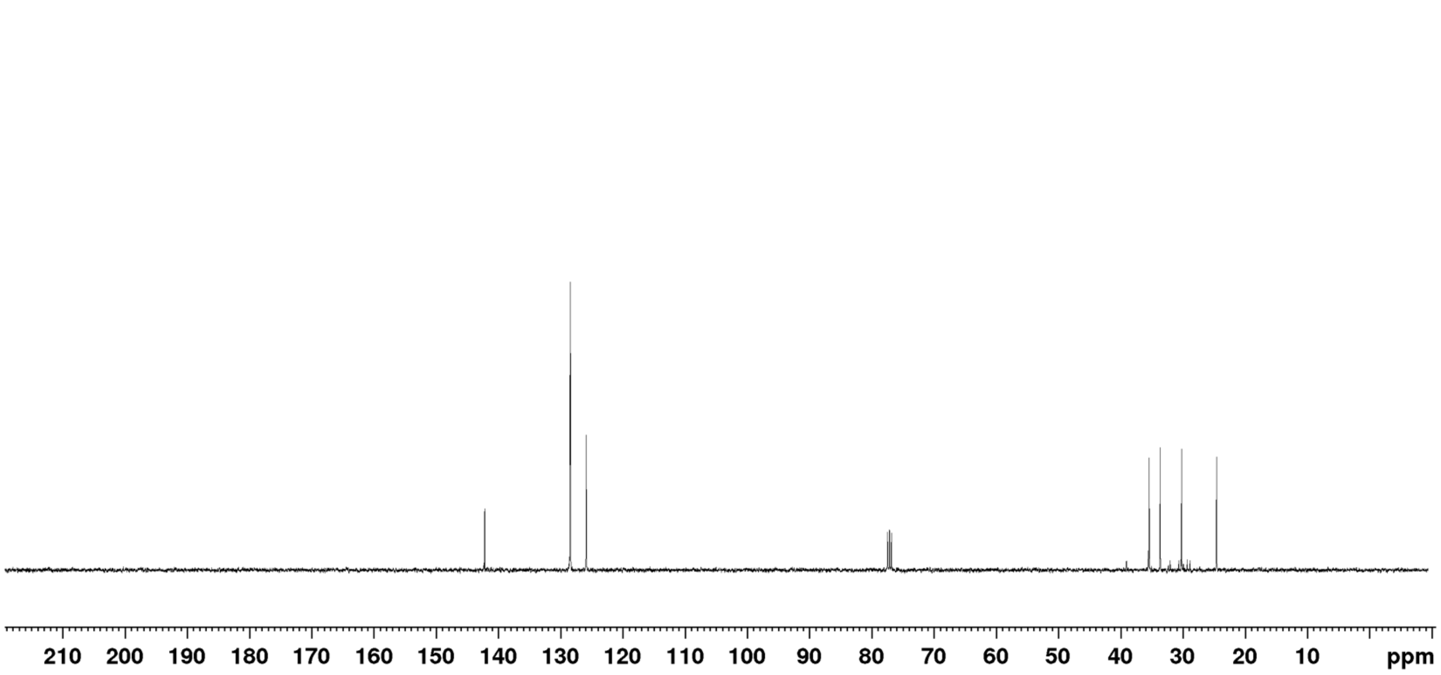

¹H NMR (500 MHz, $CDCl_3$) for *2,2,3,3,4,4,4-heptafluorobutyl (R)-(4-hydroxy-1-phenylbutyl)sulfamate* (**7a**)

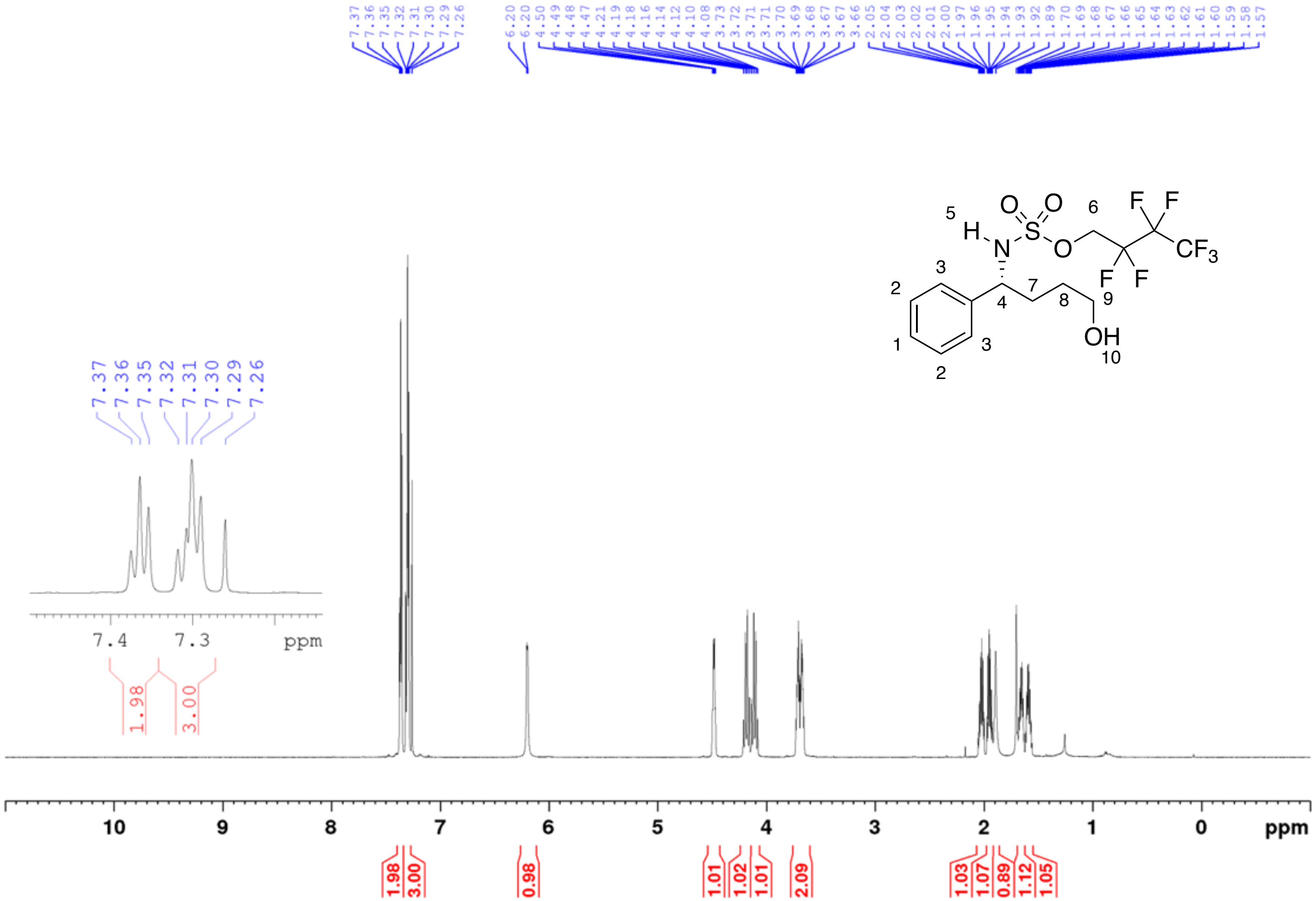

^{13}C NMR (126 MHz, $CDCl_3$) for *2,2,3,3,4,4,4-heptafluorobutyl (R)-(4-hydroxy-1-phenylbutyl)sulfamate* (**7a**)

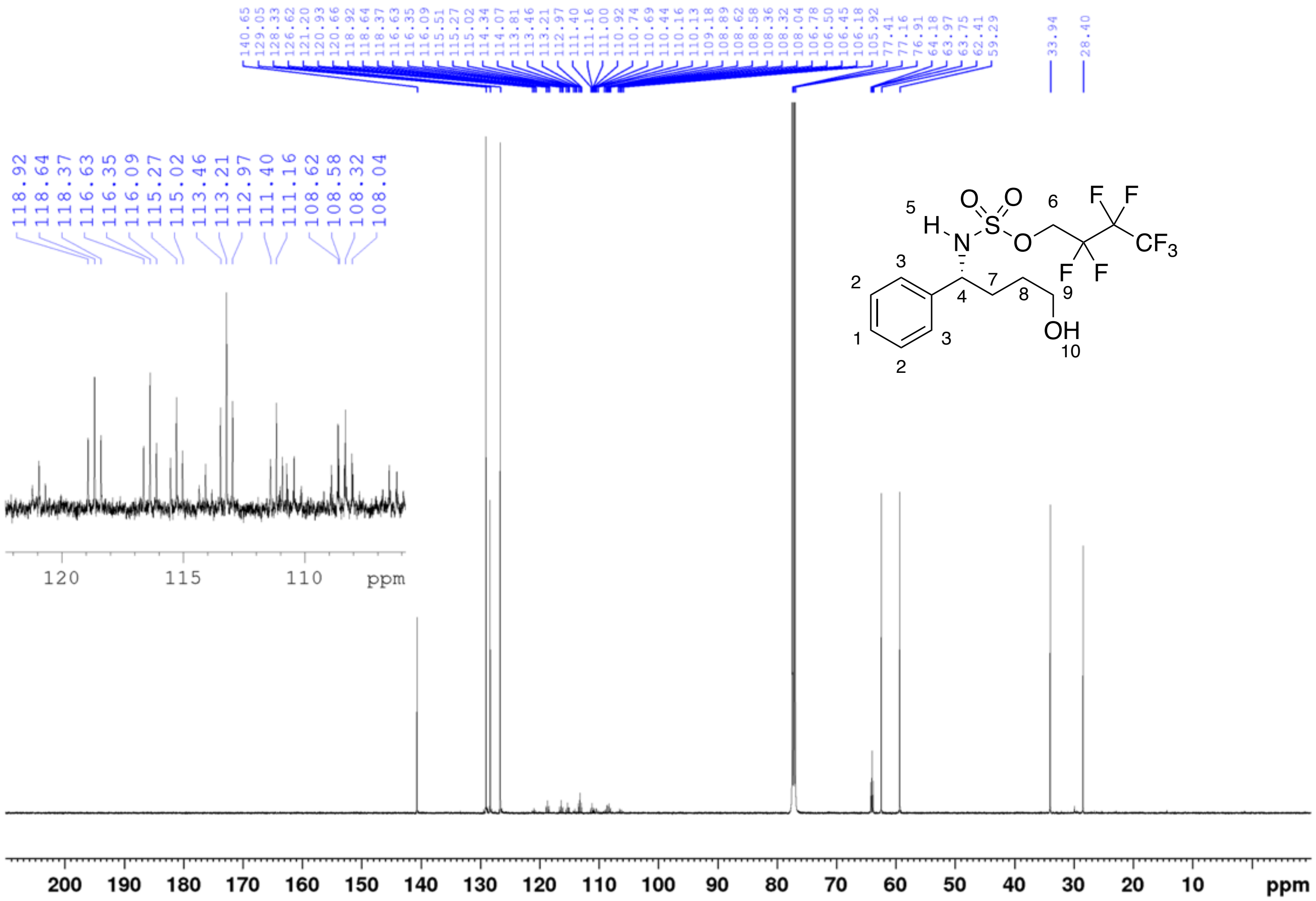

^{19}F NMR (376 MHz, $CDCl_3$) for *2,2,3,3,4,4,4-heptafluorobutyl (R)-(4-hydroxy-1-phenylbutyl)sulfamate* (**7a**)

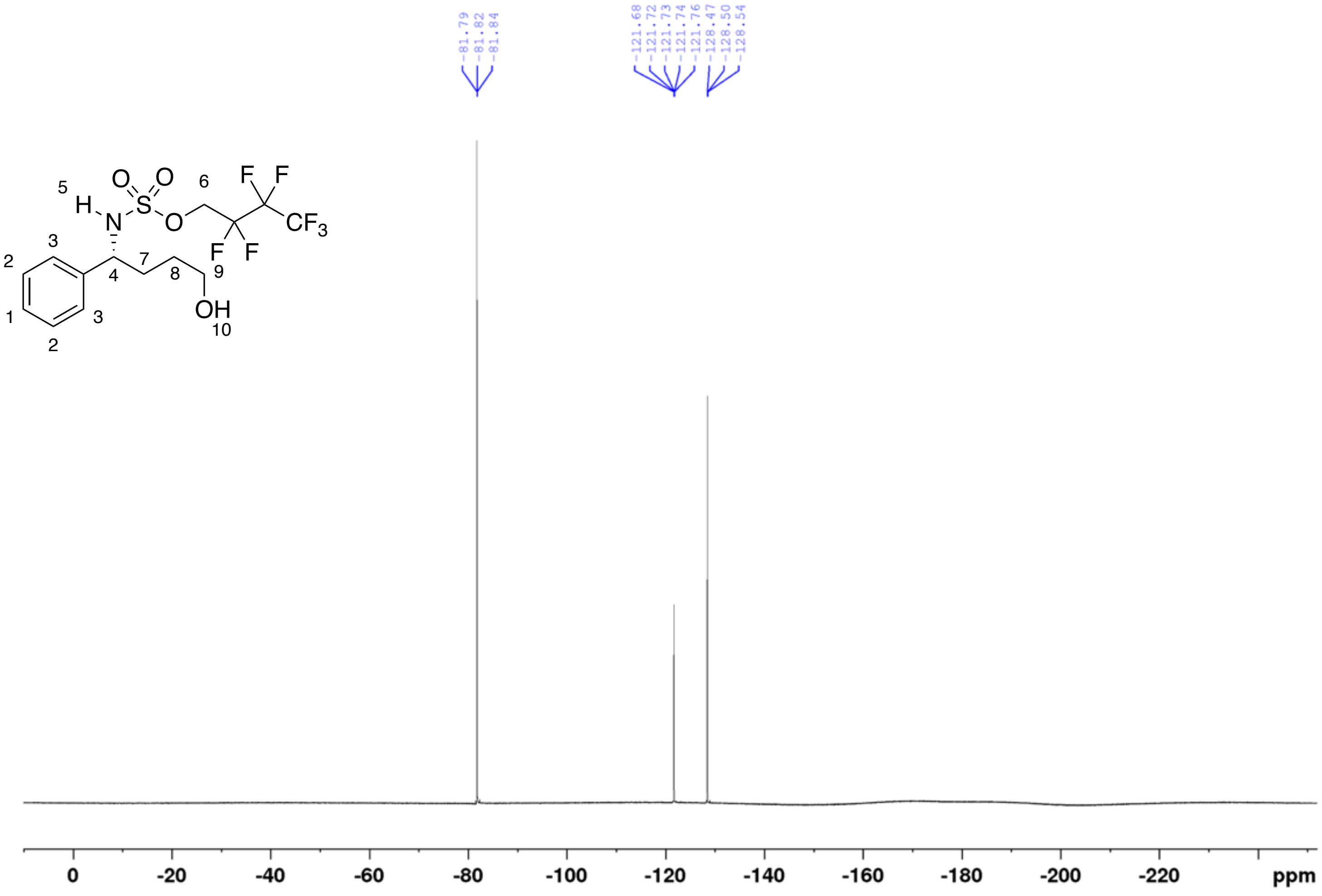

¹H NMR (500 MHz, $CDCl_3$) for *ethyl (R)-3-(1-(((2,2,3,3,4,4,4-heptafluorobutoxy)sulfonyl)amino)-4-hydroxybutyl)benzoate* (**7b**)

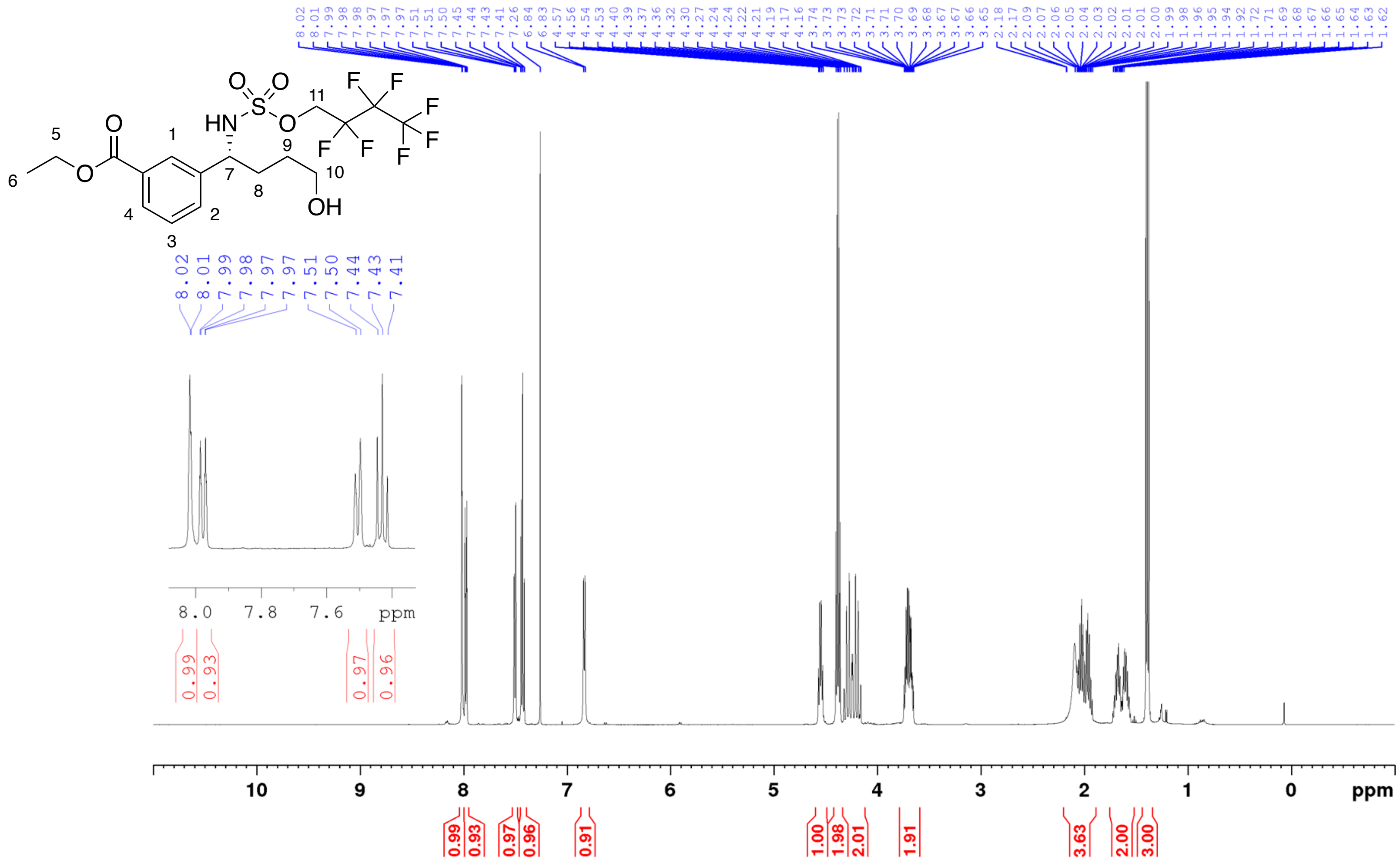

^{13}C NMR (126 MHz, $CDCl_3$) for *ethyl (R)-3-(1-(((2,2,3,3,4,4,4-heptafluorobutoxy)sulfonyl)amino)-4-hydroxybutyl)benzoate* (**7b**)

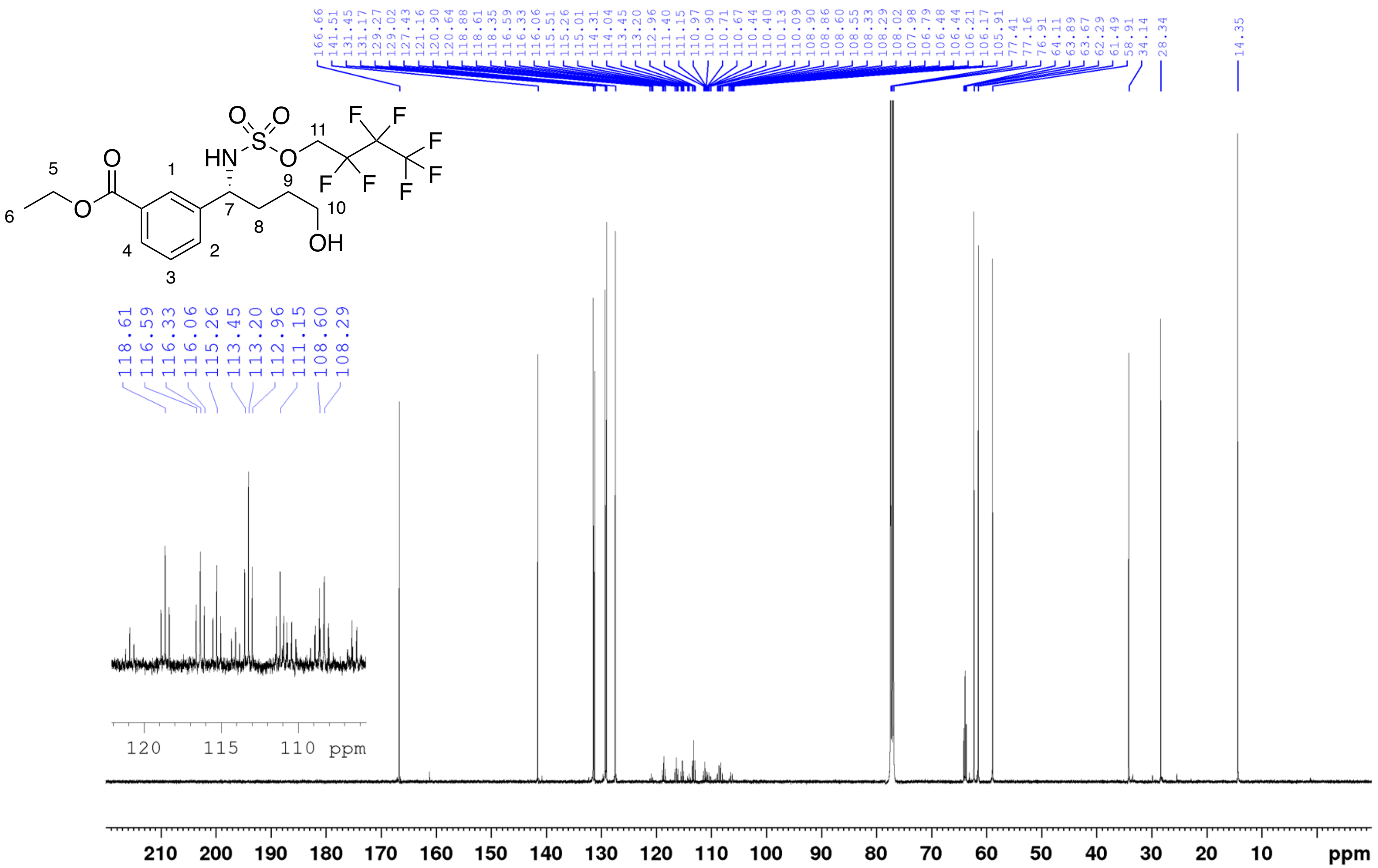

¹⁹F NMR (376 MHz, $CDCl_3$) for *ethyl (R)-3-(1-(((2,2,3,3,4,4,4-heptafluorobutoxy)sulfonyl)amino)-4-hydroxybutyl)benzoate* (**7b**)

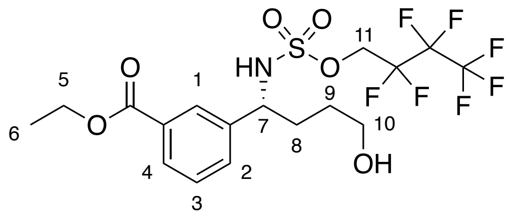

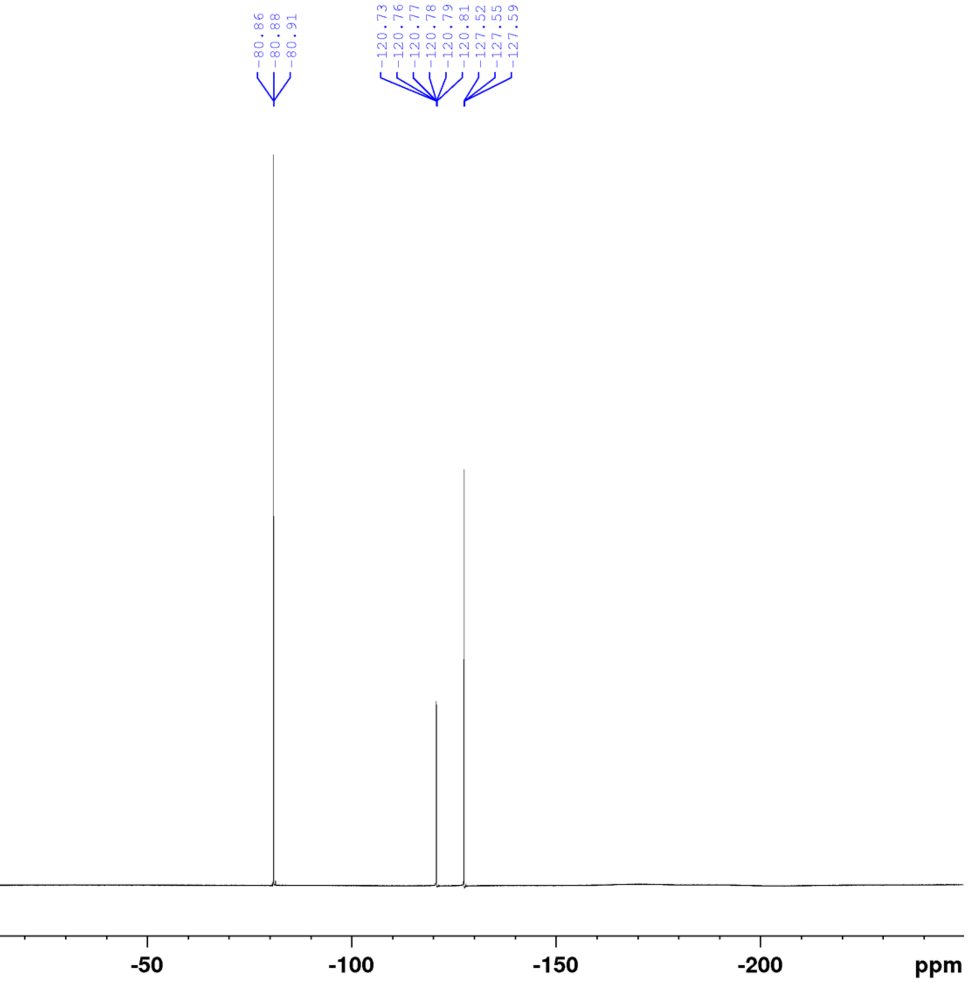

^{1}H NMR (500 MHz, $CDCl_3$) *for 2,2,3,3,4,4,4-heptafluorobutyl (R)-(4-hydroxy-1-(o-tolyl)butyl)sulfamate* (**7c**)

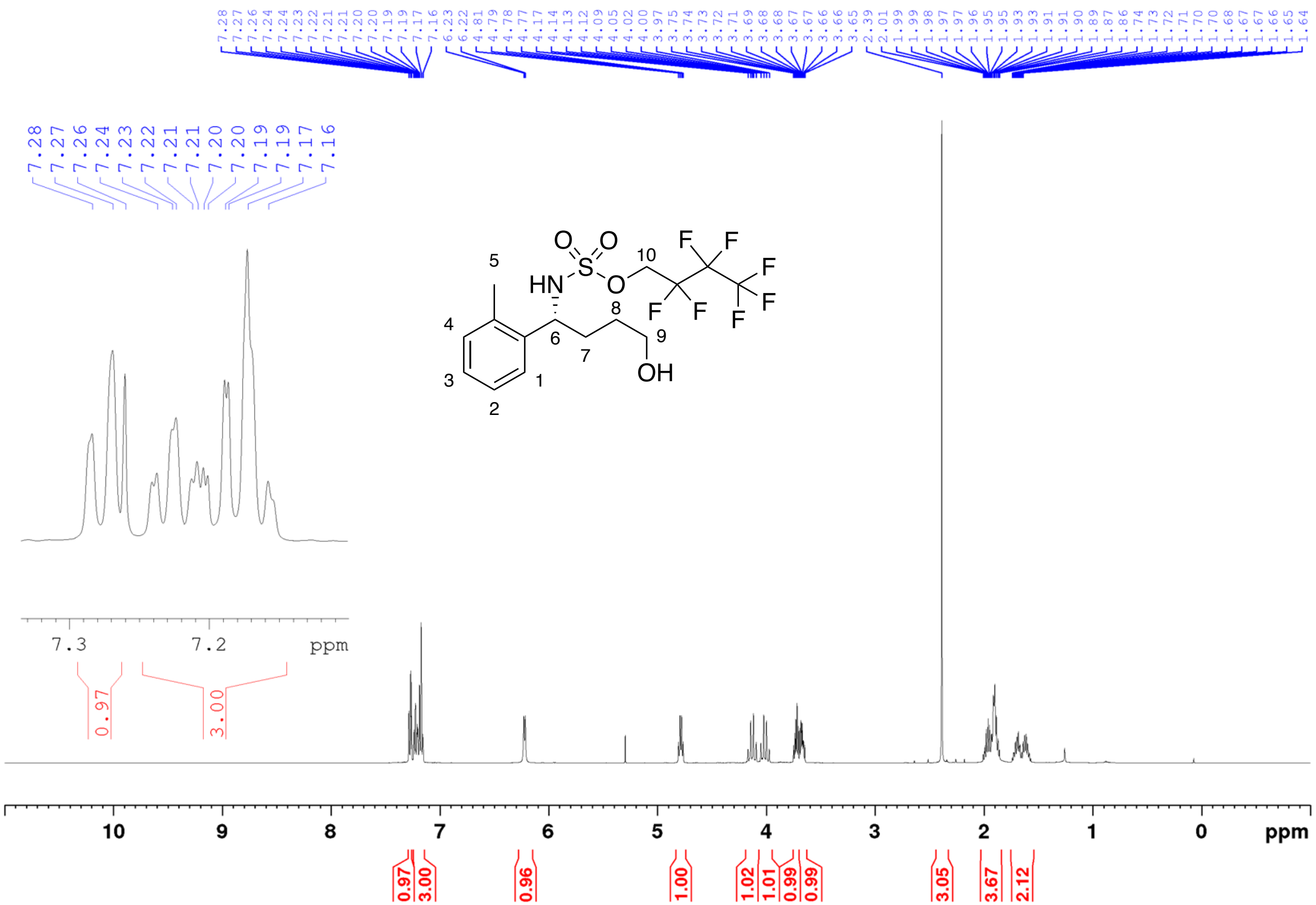

^{13}C NMR (126 MHz, $CDCl_3$) *for 2,2,3,3,4,4,4-heptafluorobutyl (R)-(4-hydroxy-1-(o-tolyl)butyl)sulfamate* (**7c**)

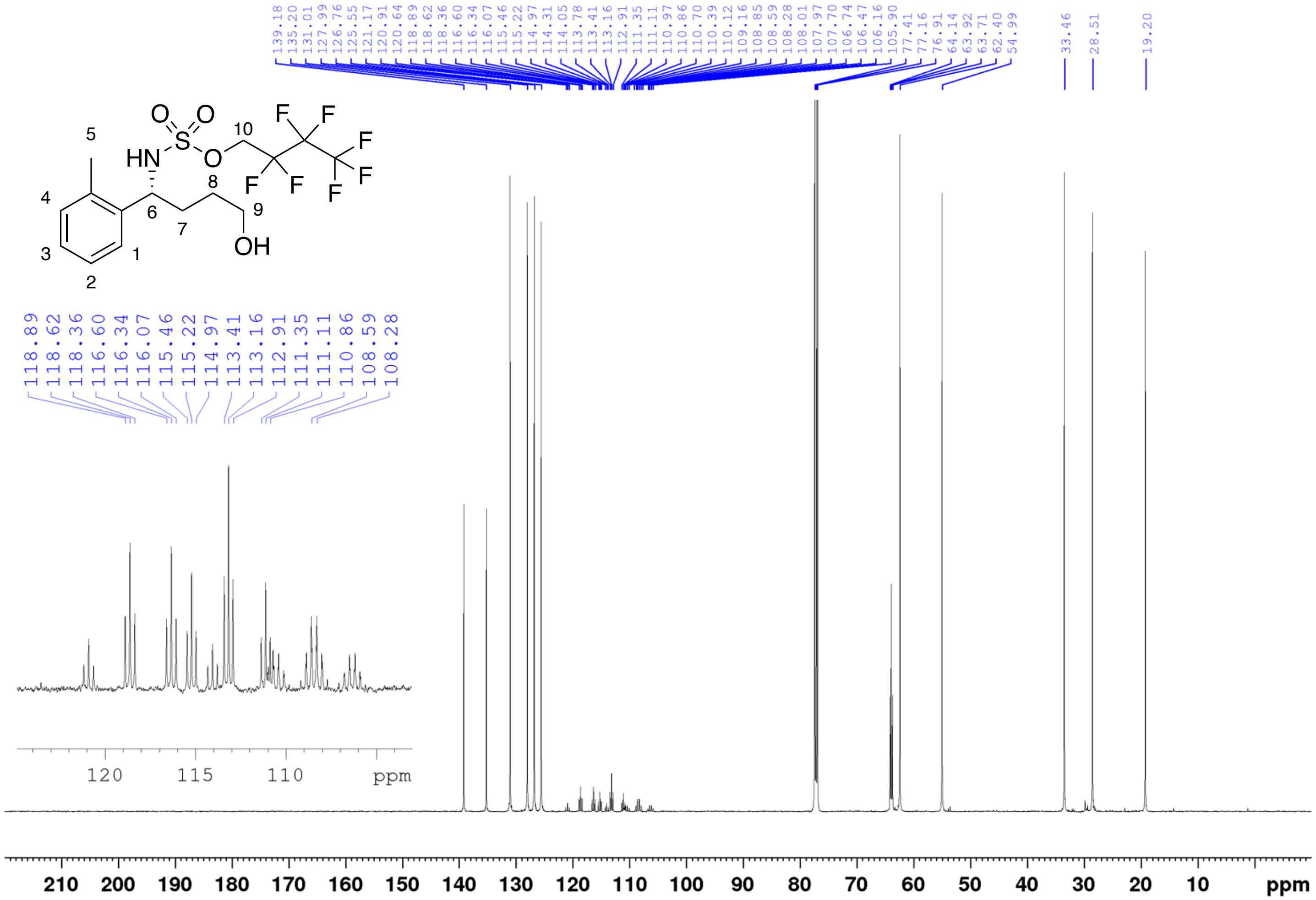

^{19}F NMR (376 MHz, $CDCl_3$) *for 2,2,3,3,4,4,4-heptafluorobutyl (R)-(4-hydroxy-1-(o-tolyl)butyl)sulfamate* (**7c**)

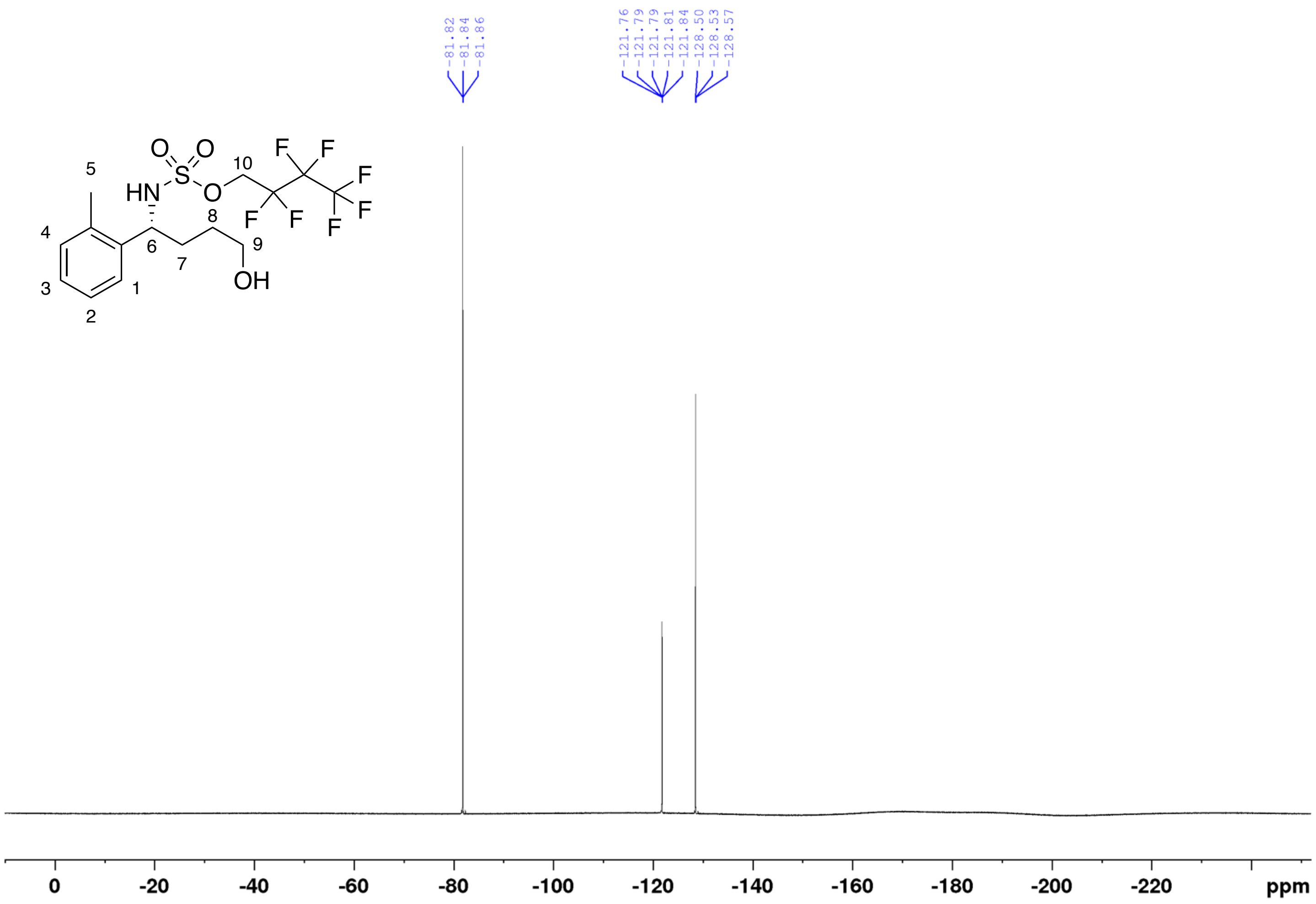

^{1}H NMR (500 MHz, $CDCl_3$) for *2,2,3,3,4,4,4-heptafluorobutyl (R)-(4-hydroxy-1-(m-tolyl)butyl)sulfamate* (**7d**)

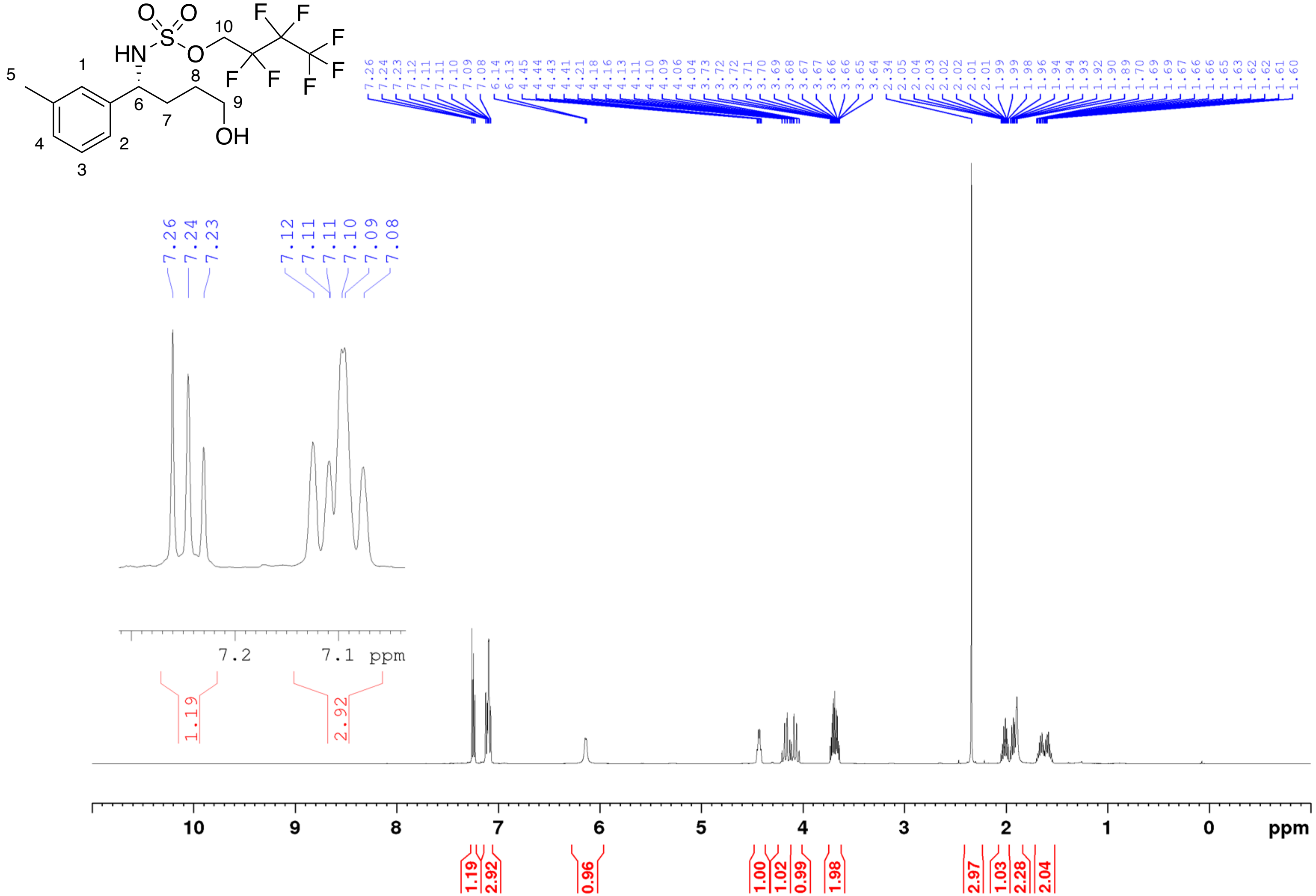

^{13}C NMR (126 MHz, $CDCl_3$) for *2,2,3,3,4,4,4-heptafluorobutyl (R)-(4-hydroxy-1-(m-tolyl)butyl)sulfamate* (**7d**)

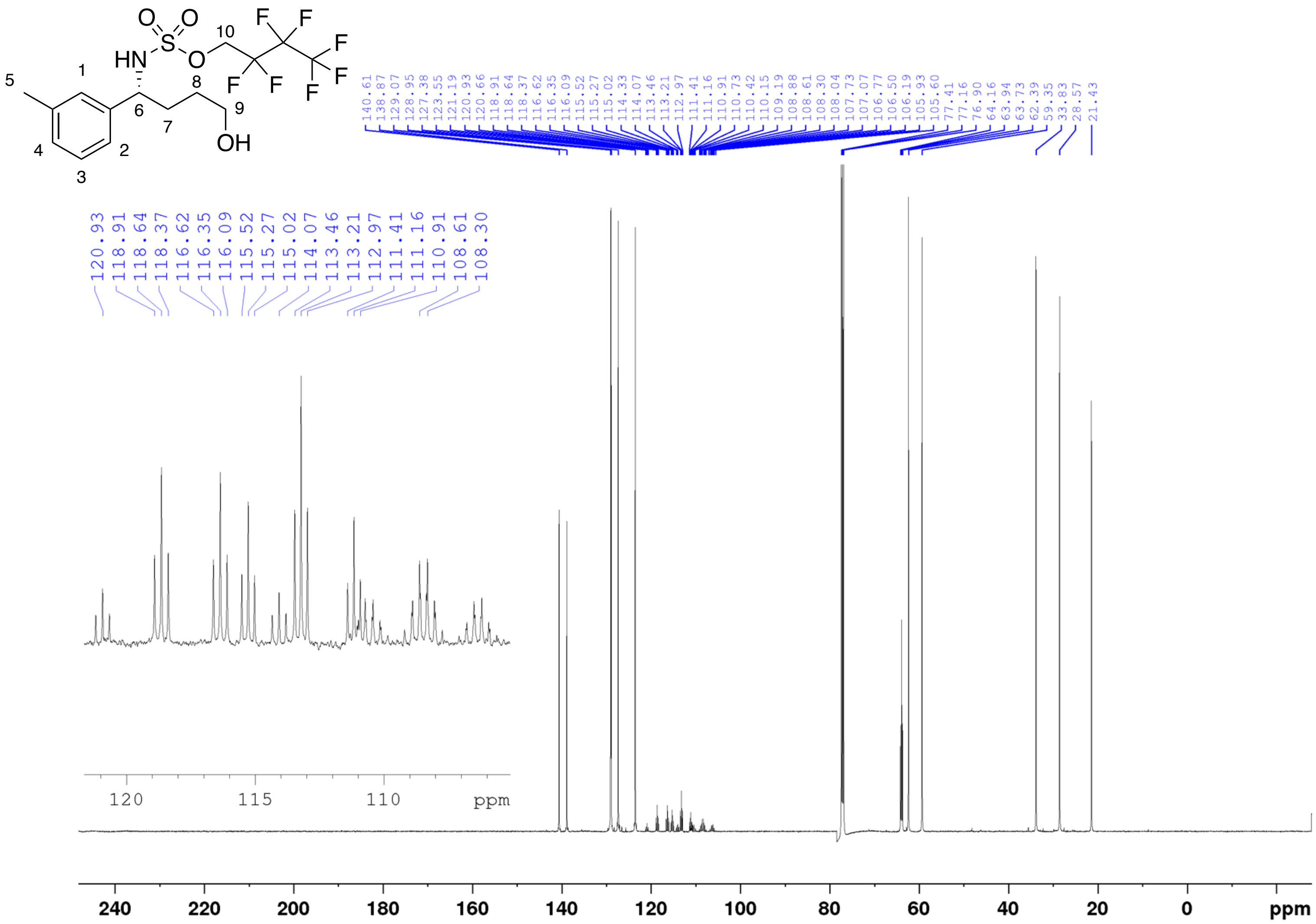

^{19}F NMR (376 MHz, $CDCl_3$) for *2,2,3,3,4,4,4-heptafluorobutyl (R)-(4-hydroxy-1-(m-tolyl)butyl)sulfamate* (**7d**)

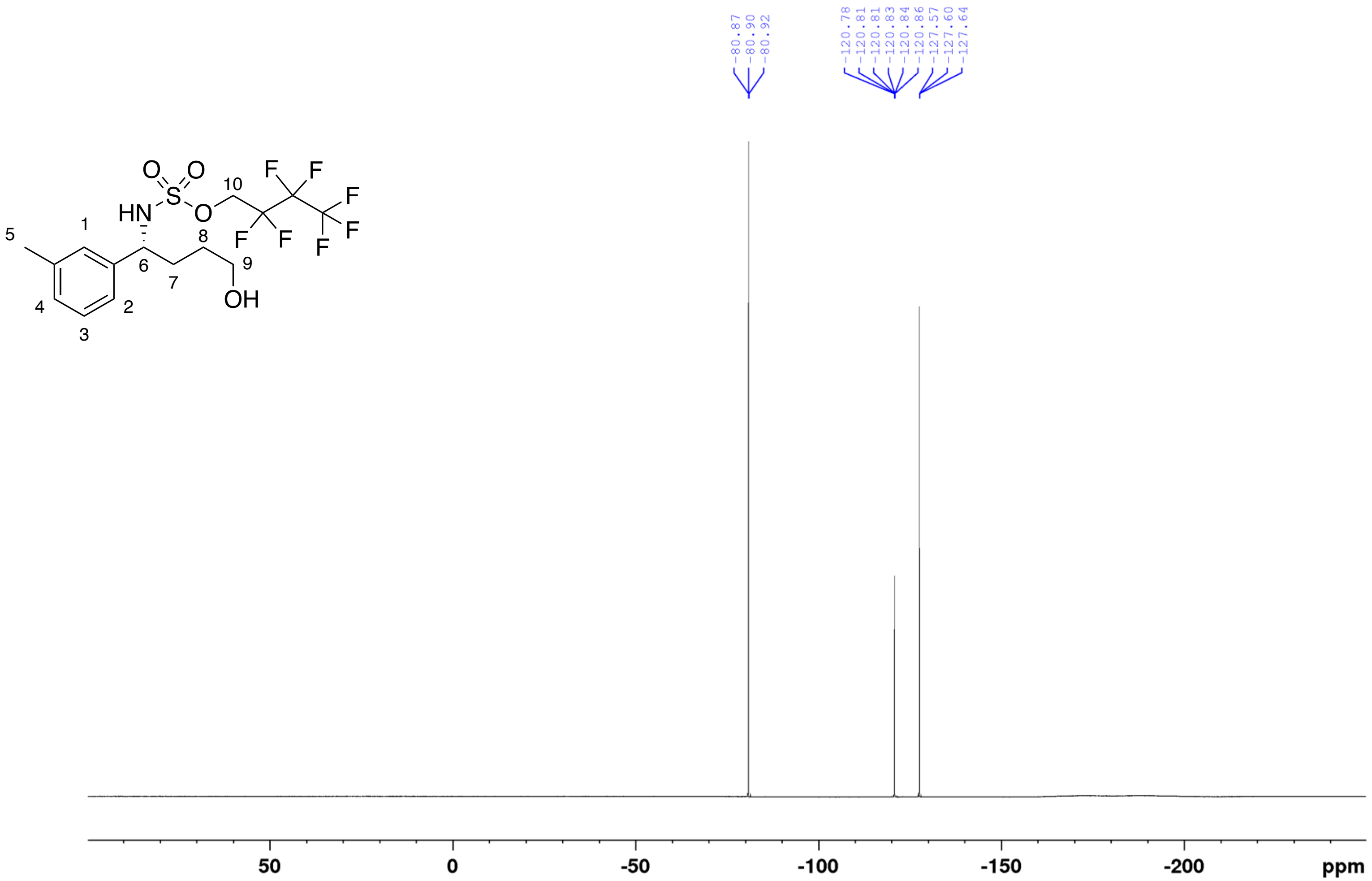

¹H NMR (500 MHz, $CDCl_3$) for *2,2,3,3,4,4,4-heptafluorobutyl (R)-(1-(3-fluorophenyl)-4-hydroxybutyl)sulfamate* (**7e**)

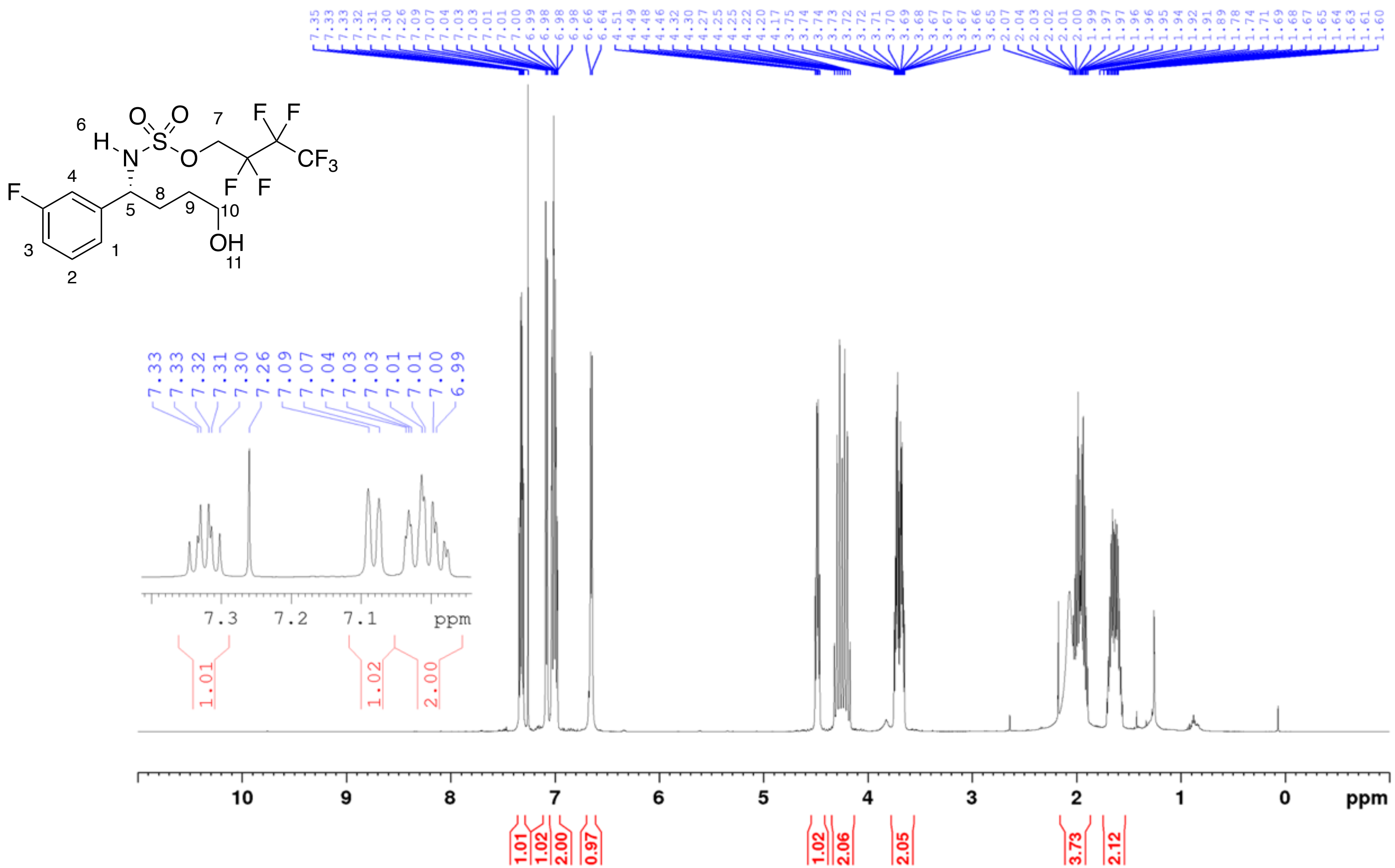

^{13}C NMR (126 MHz, $CDCl_3$) for 2,2,3,3,4,4,4-heptafluorobutyl (R)-(1-(3-fluorophenyl)-4-hydroxybutyl)sulfamate (**7e**)

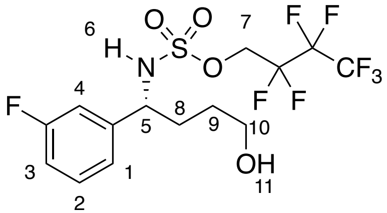

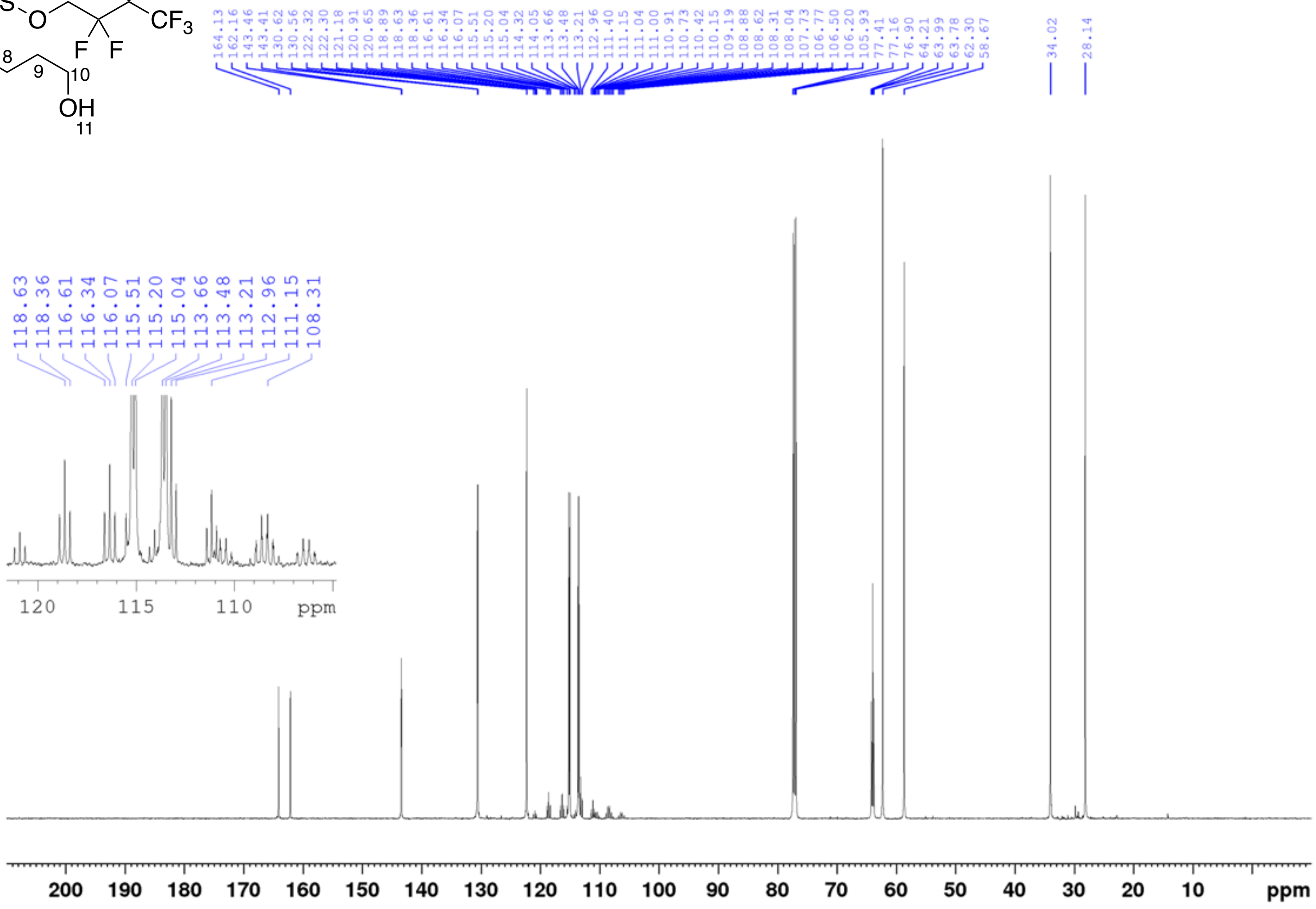

^{19}F NMR (376 MHz, $CDCl_3$) for 2,2,3,3,4,4,4-heptafluorobutyl (R)-(1-(3-fluorophenyl)-4-hydroxybutyl)sulfamate (**7e**)

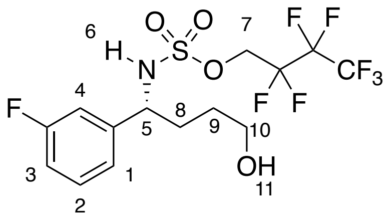

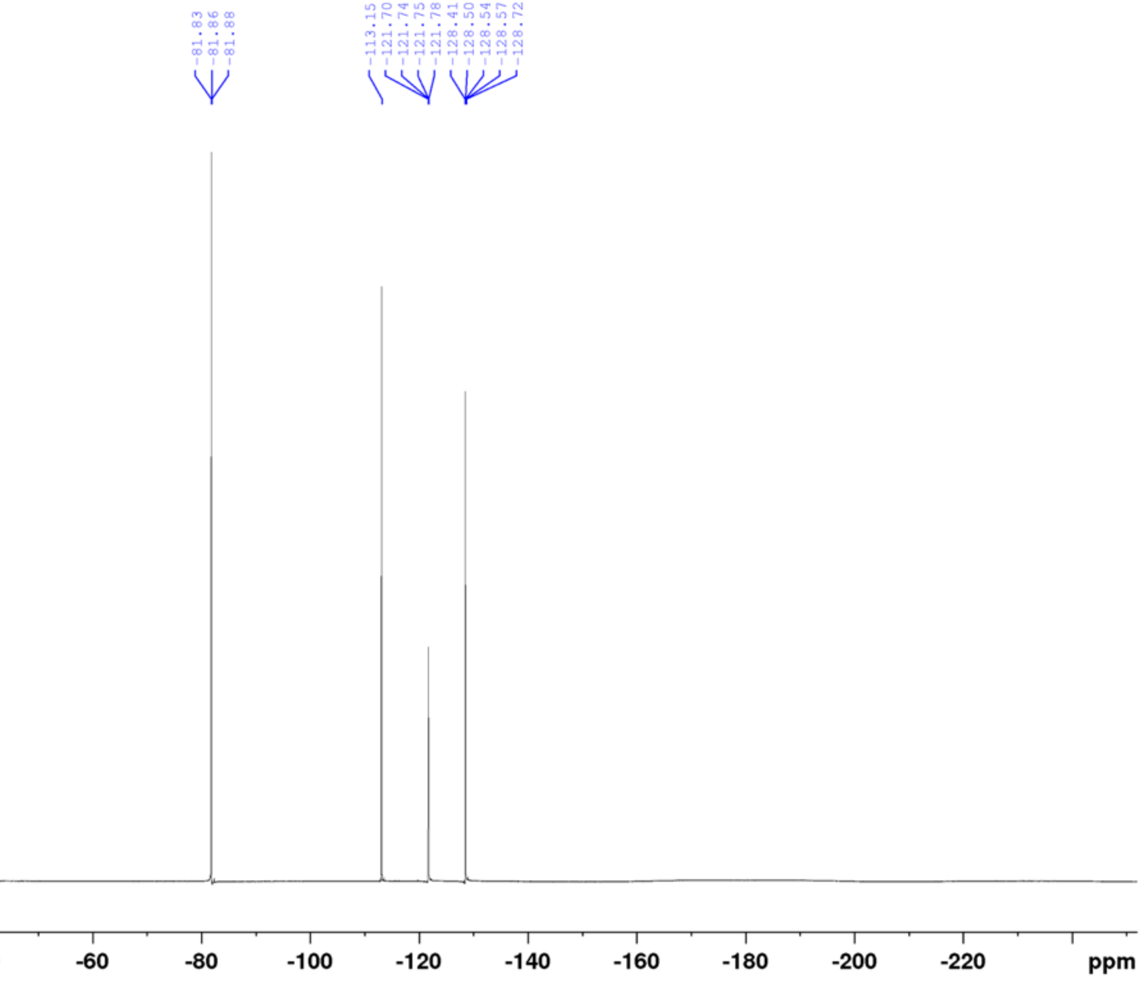

¹H NMR (500 MHz, $CDCl_3$) for *2,2,3,3,4,4,4-heptafluorobutyl (R)-(1-(2-fluorophenyl)-4-hydroxybutyl)sulfamate* (**7f**)

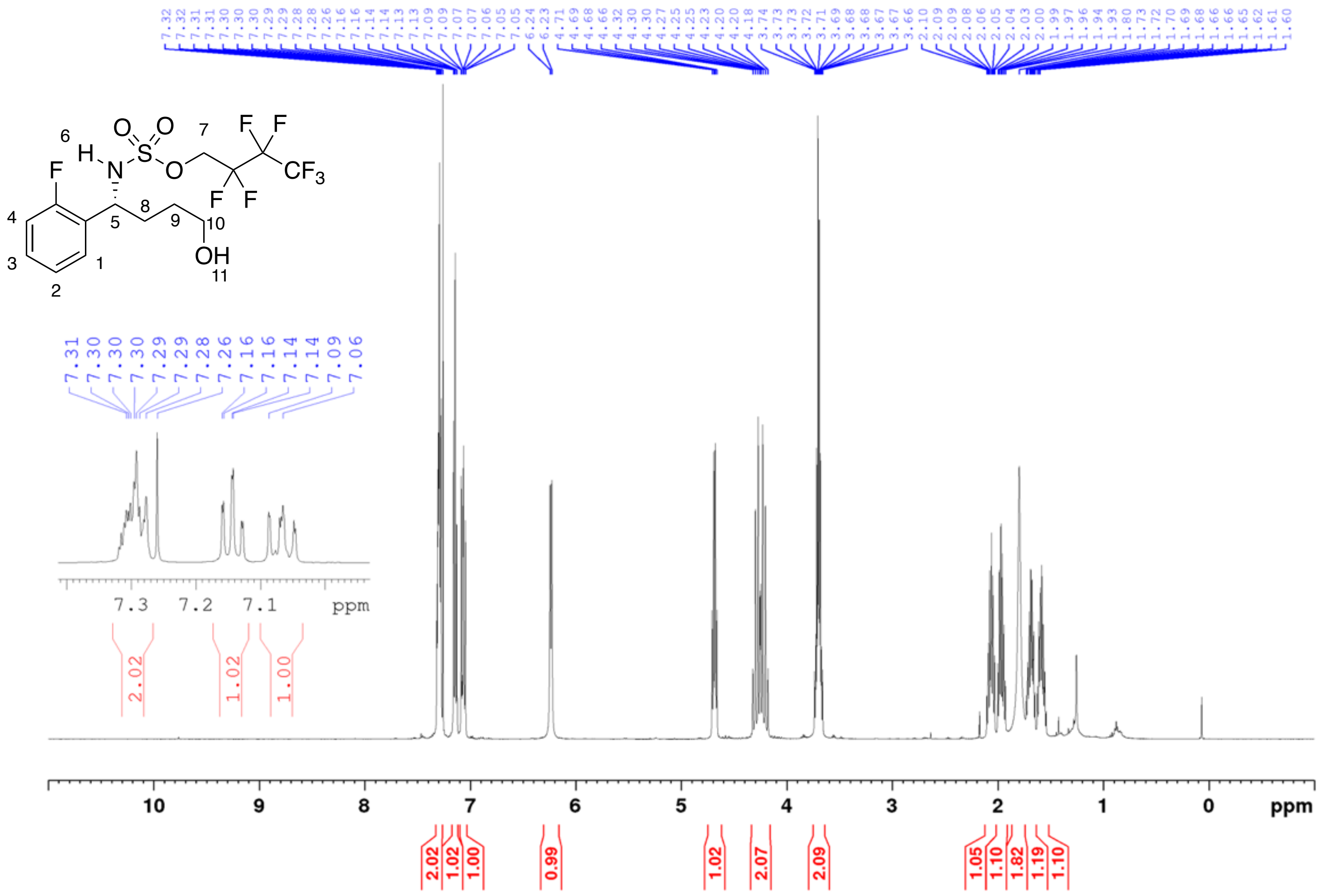

^{13}C NMR (126 MHz, $CDCl_3$) for *2,2,3,3,4,4,4-heptafluorobutyl (R)-(1-(2-fluorophenyl)-4-hydroxybutyl)sulfamate* (**7f**)

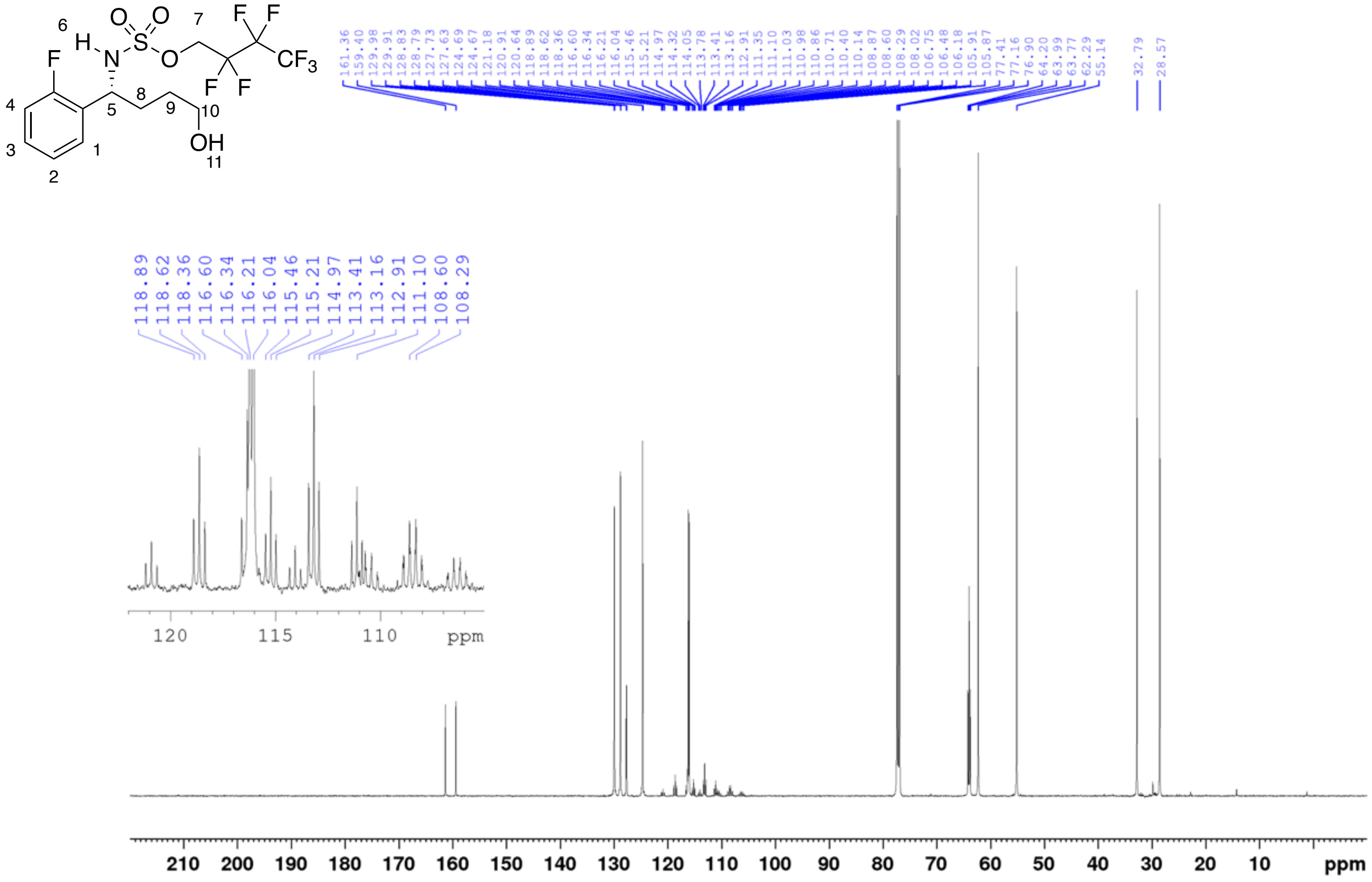

^{19}F NMR (376 MHz, $CDCl_3$) for *2,2,3,3,4,4,4-heptafluorobutyl (R)-(1-(2-fluorophenyl)-4-hydroxybutyl)sulfamate* (**7f**)

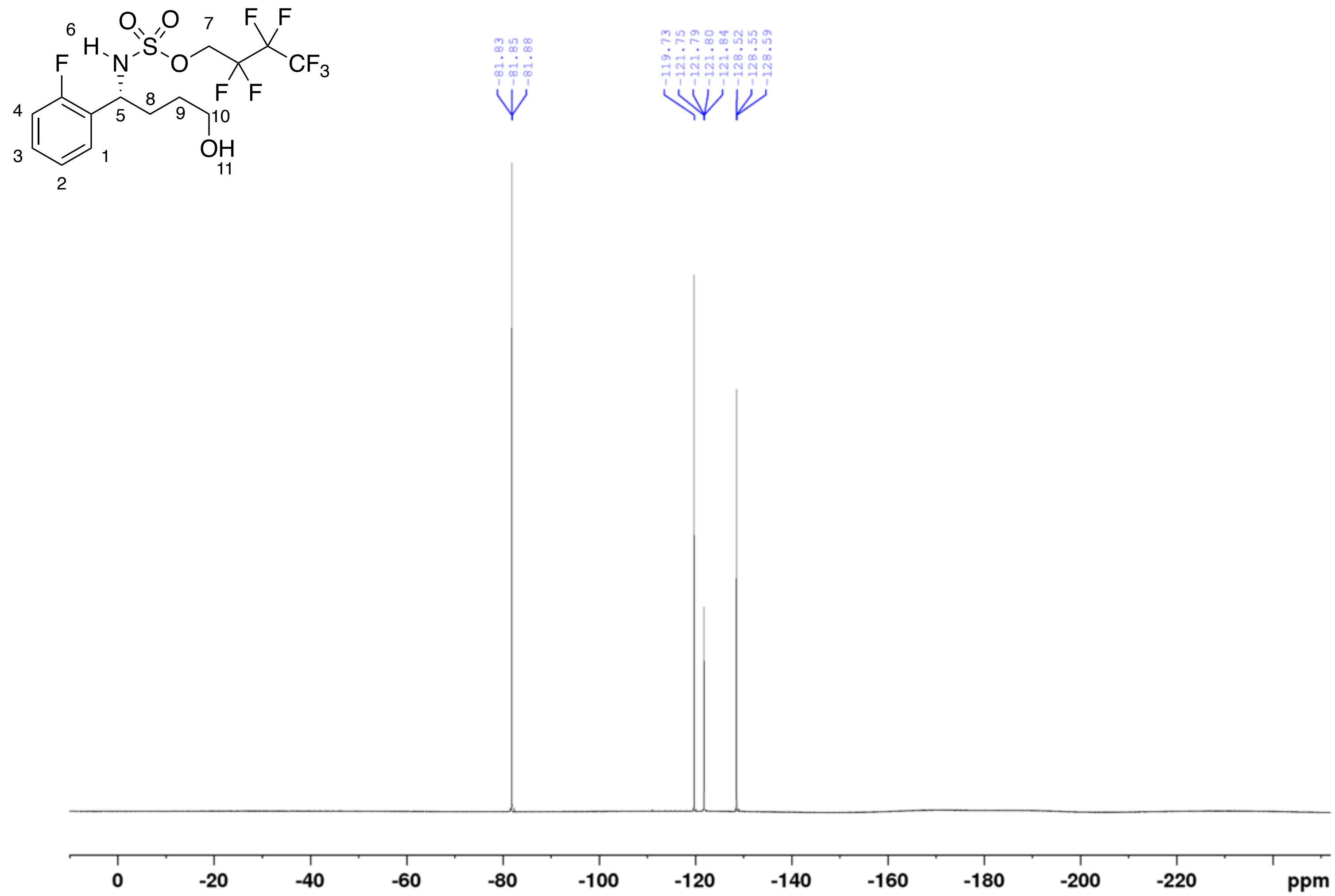

^{1}H NMR (500 MHz, $CDCl_3$) for *2,2,3,3,4,4,4-heptafluorobutyl (R)-(1-(4-fluorophenyl)-4-hydroxybutyl)sulfamate* (**7g**)

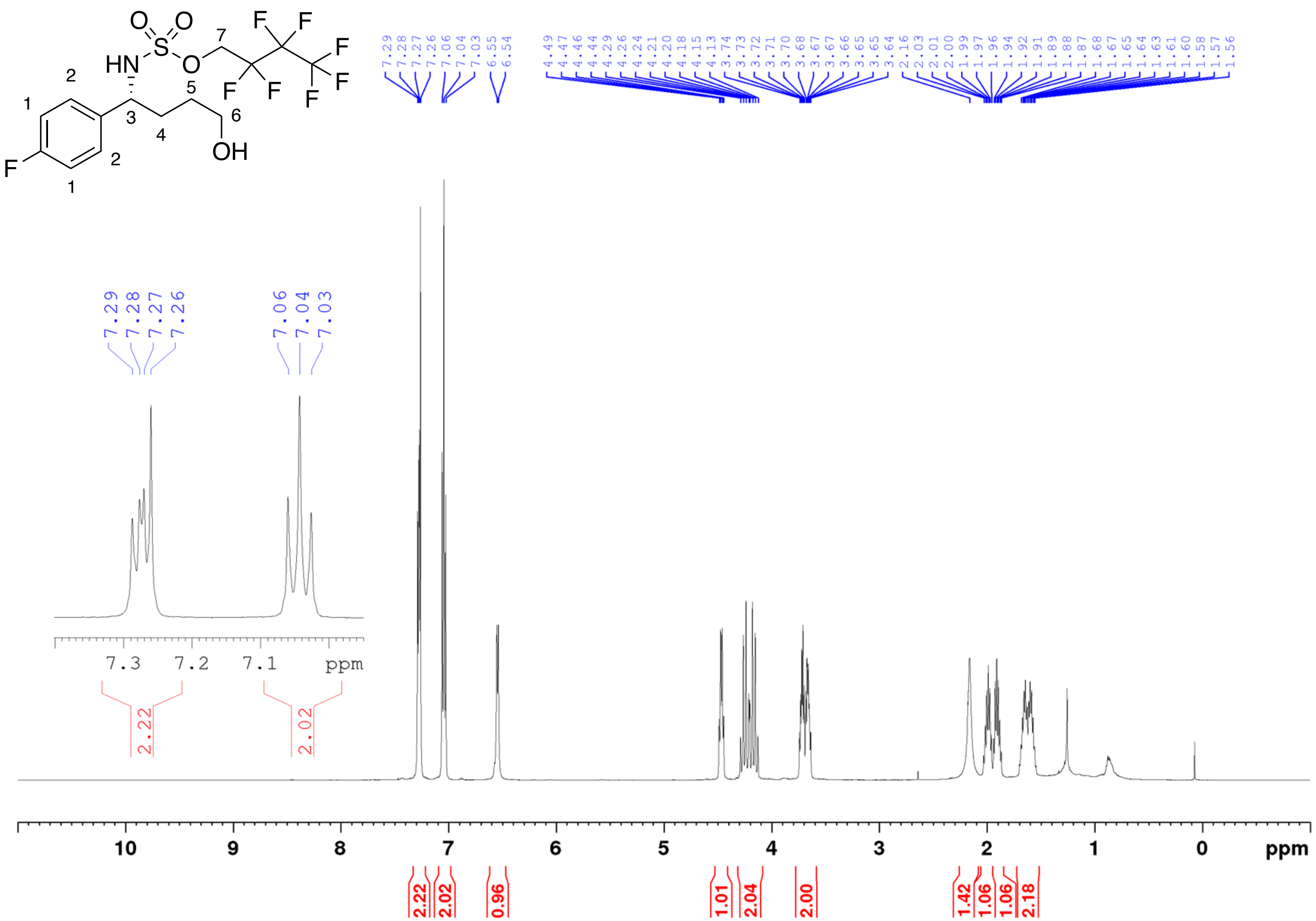

^{13}C NMR (126 MHz, $CDCl_3$) for *2,2,3,3,4,4,4-heptafluorobutyl (R)-(1-(4-fluorophenyl)-4-hydroxybutyl)sulfamate* (**7g**)

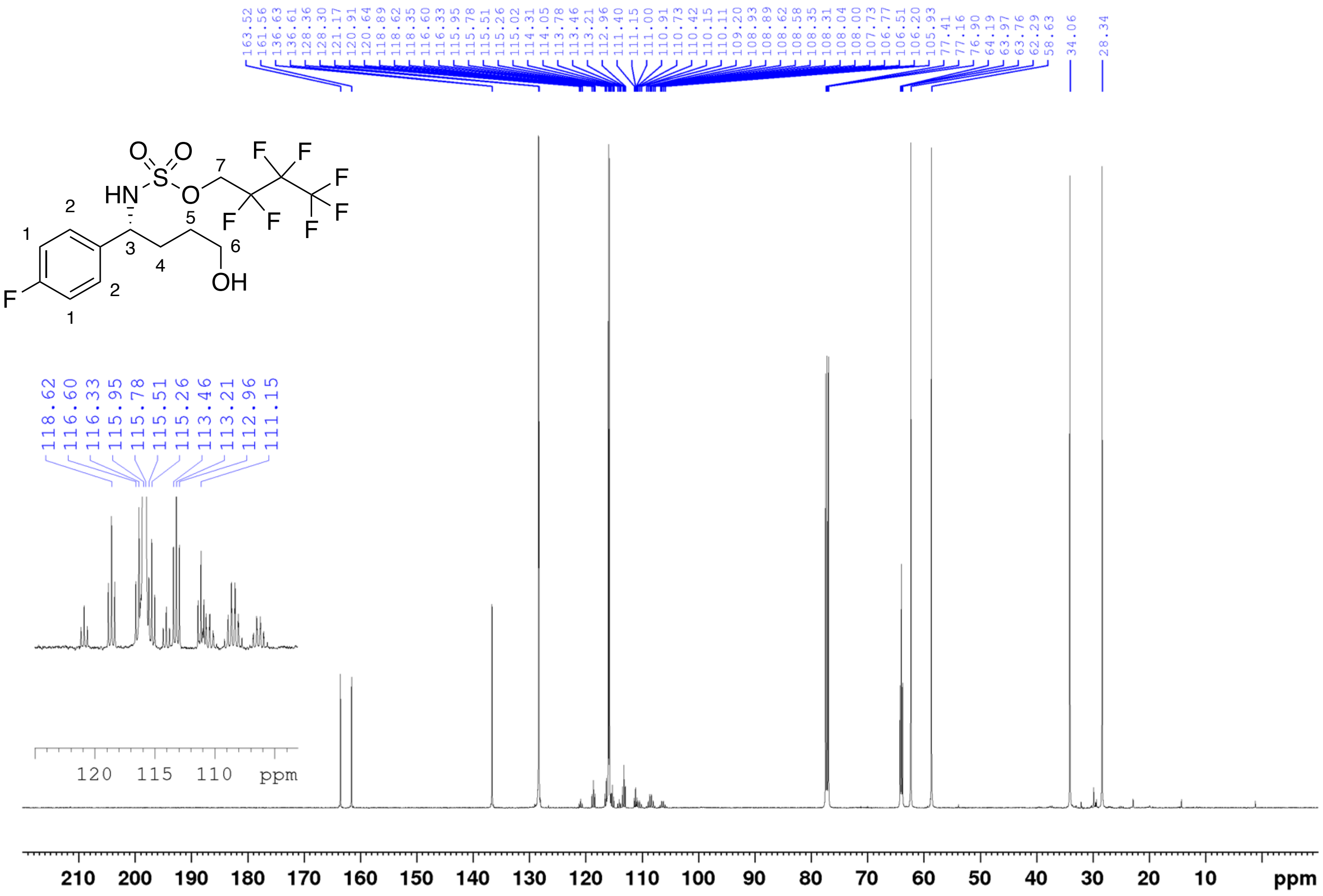

^{19}F NMR (376 MHz, $CDCl_3$) for *2,2,3,3,4,4,4-heptafluorobutyl (R)-(1-(4-fluorophenyl)-4-hydroxybutyl)sulfamate* (**7g**)

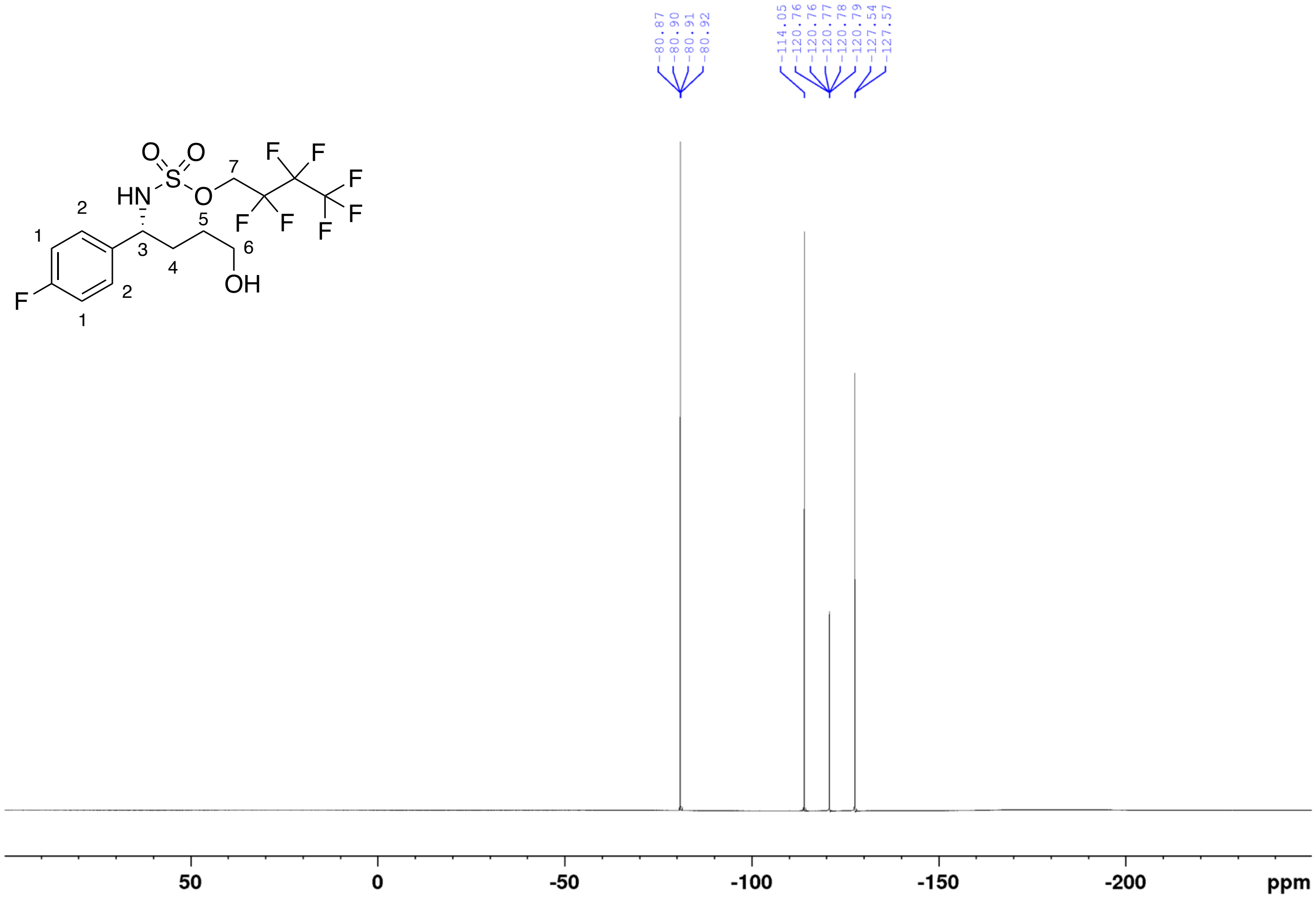

¹H NMR (500 MHz, $CDCl_3$) for *2,2,3,3,4,4,4-heptafluorobutyl (R)-(1-(3-chlorophenyl)-4-hydroxybutyl)sulfamate* (**7h**)

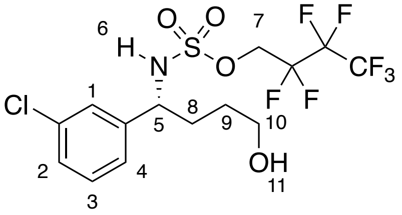

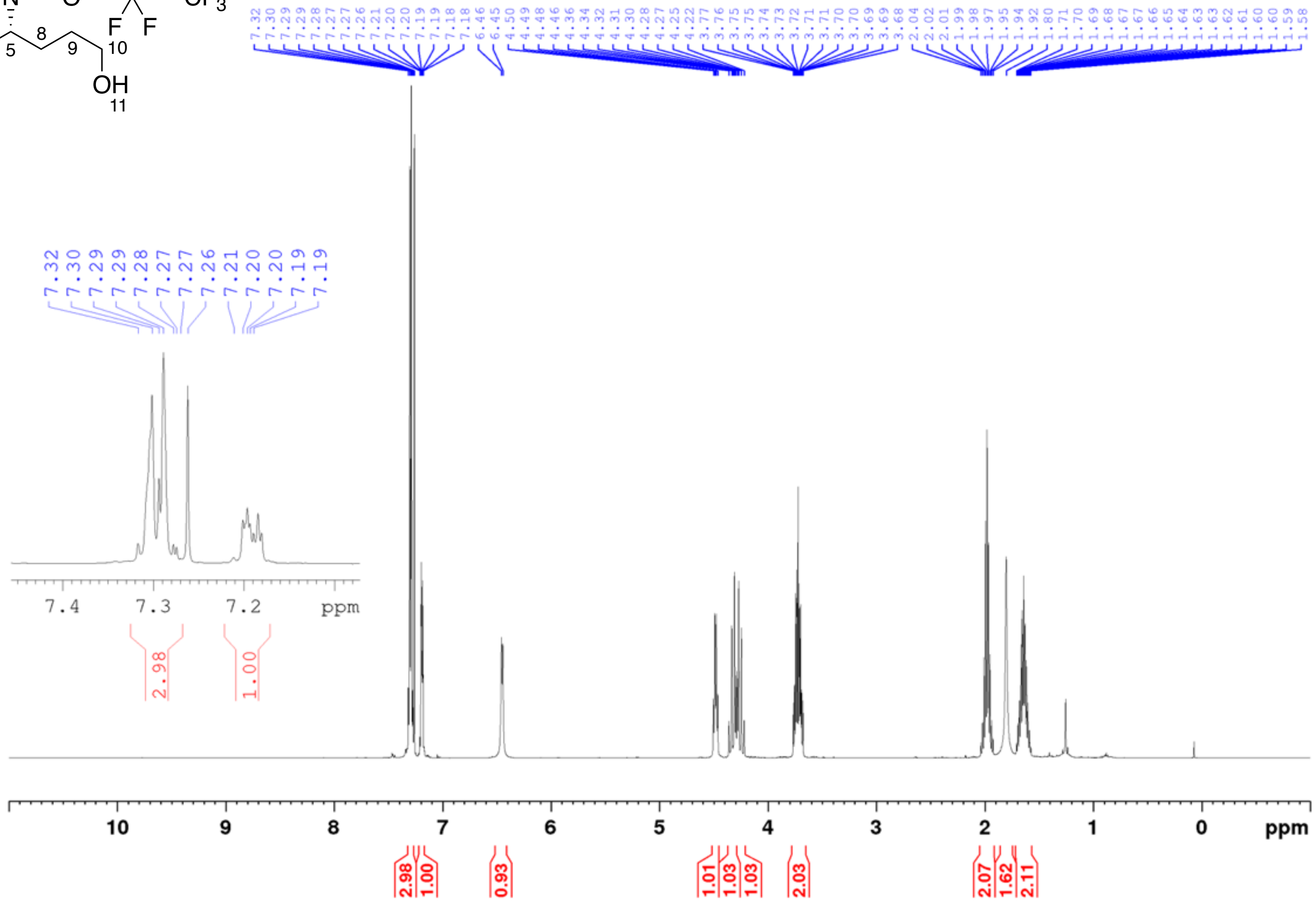

^{13}C NMR (126 MHz, $CDCl_3$) for *2,2,3,3,4,4,4-heptafluorobutyl (R)-(1-(3-chlorophenyl)-4-hydroxybutyl)sulfamate* (**7h**)

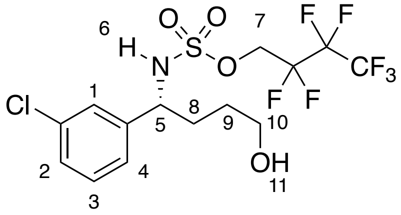

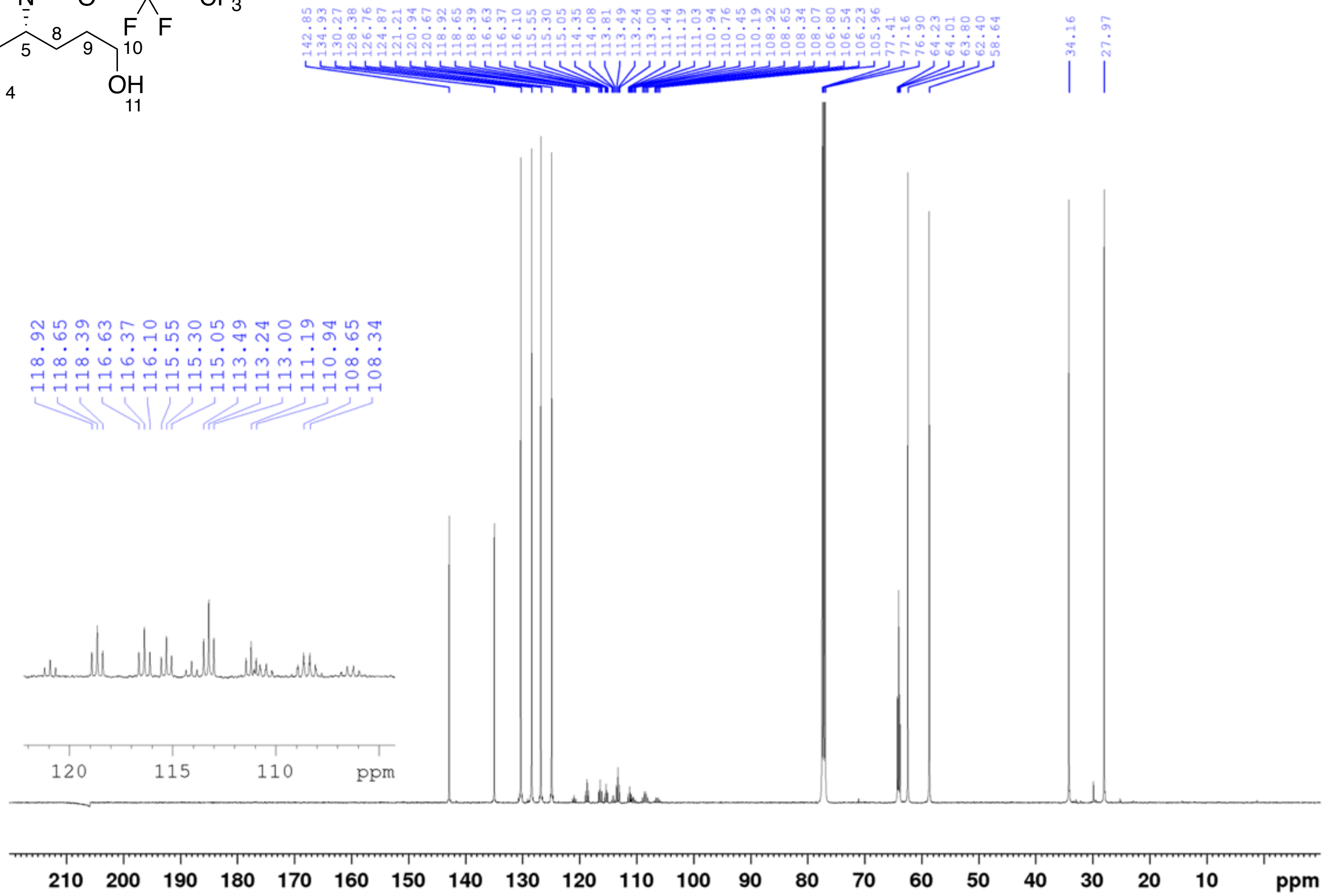

^{19}**F NMR** (376 MHz, $CDCl_3$) for *2,2,3,3,4,4,4-heptafluorobutyl (R)-(1-(3-chlorophenyl)-4-hydroxybutyl)sulfamate* (**7h**)

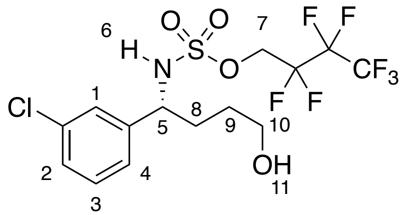

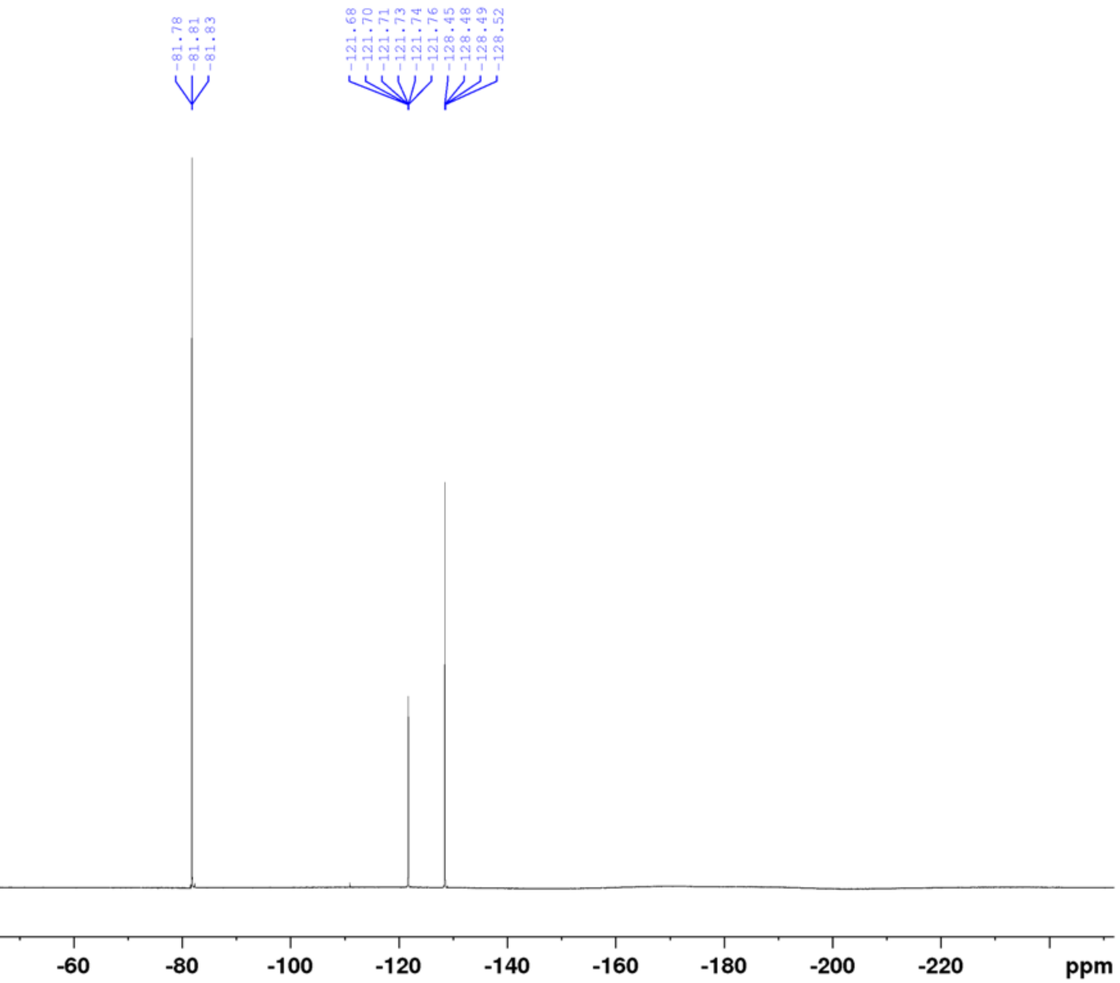

^{1}H NMR (400 MHz, $CDCl_3$) for *2,2,3,3,4,4,4-heptafluorobutyl (R)-(1-(3-bromophenyl)-4-hydroxybutyl)sulfamate* (**7i**)

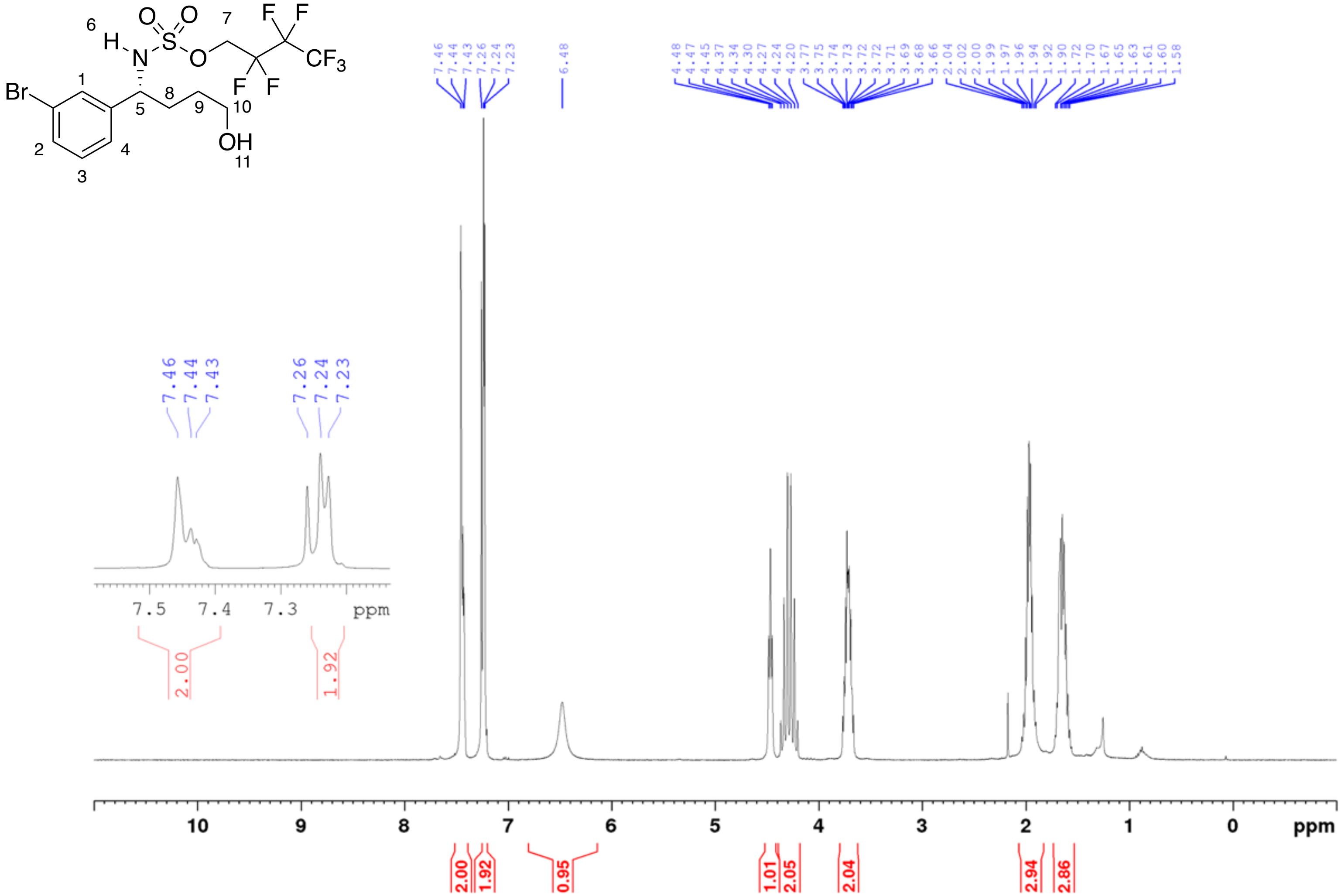

^{13}C NMR (126 MHz, $CDCl_3$) for *2,2,3,3,4,4,4-heptafluorobutyl (R)-(1-(3-bromophenyl)-4-hydroxybutyl)sulfamate* (**7i**)

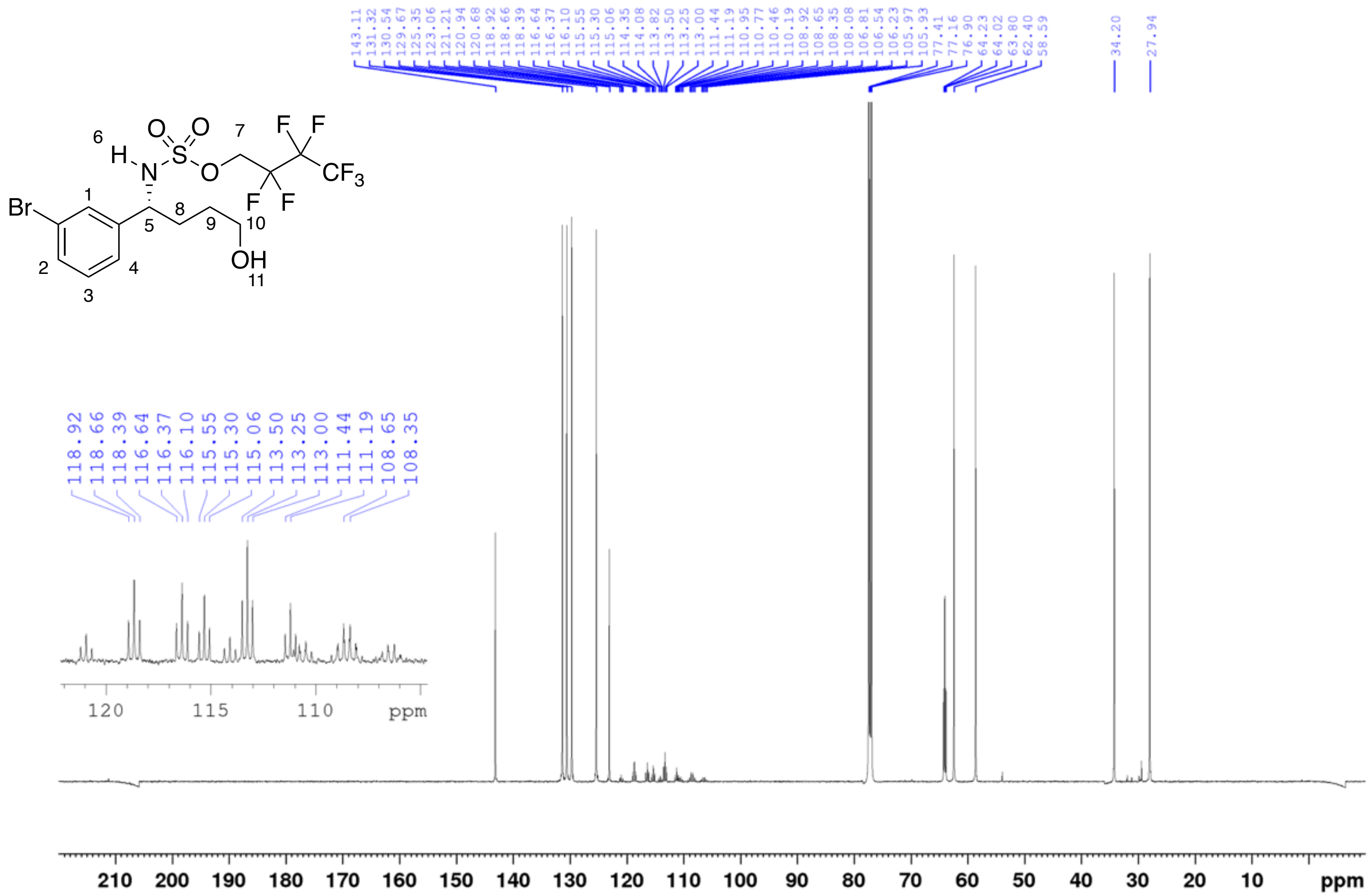

^{19}F NMR (376 MHz, $CDCl_3$) for *2,2,3,3,4,4,4-heptafluorobutyl (R)-(1-(3-bromophenyl)-4-hydroxybutyl)sulfamate* (**7i**)

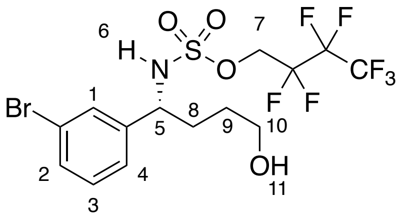

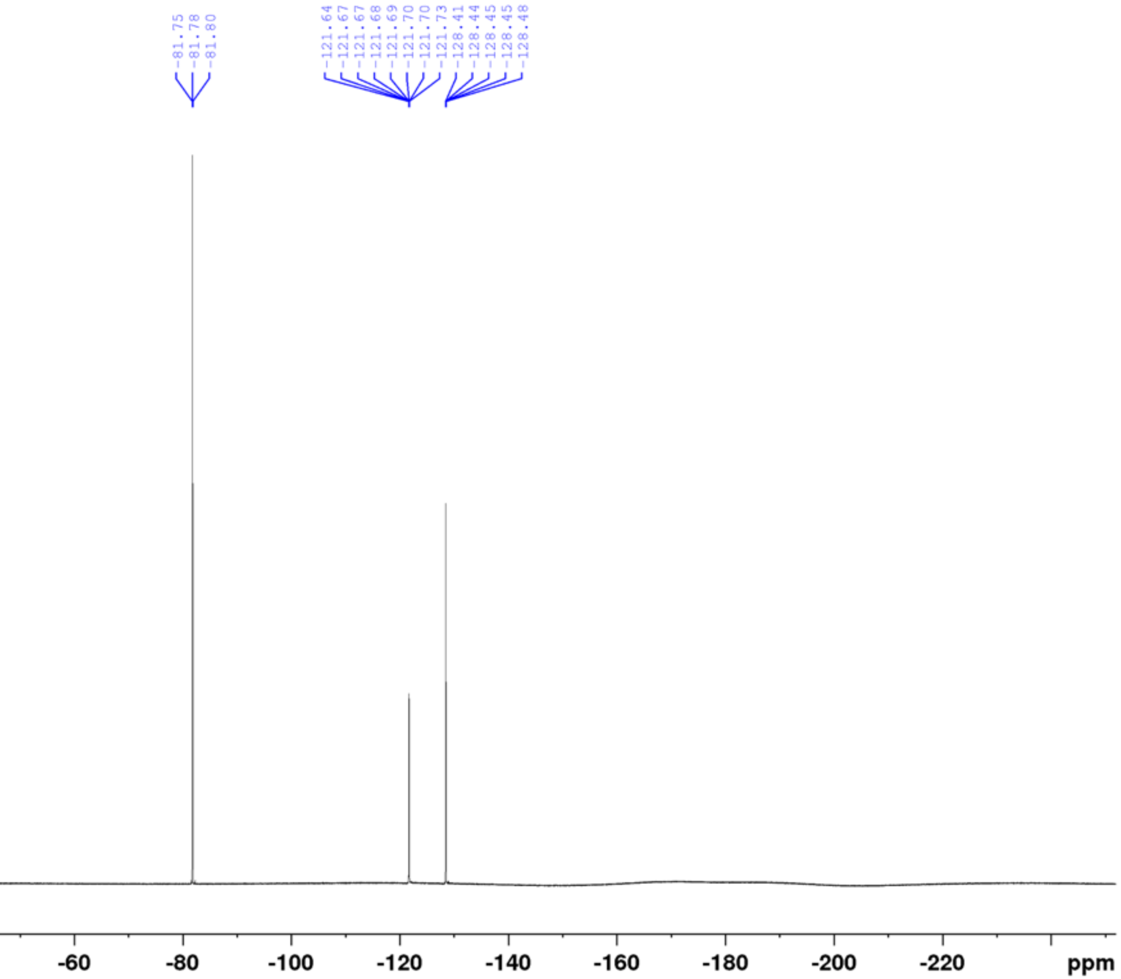

^{1}H NMR (400 MHz, $CDCl_3$) for *2,2,3,3,4,4,4-heptafluorobutyl (R)-(4-hydroxy-1-(3-iodophenyl)butyl)sulfamate* (**7j**)

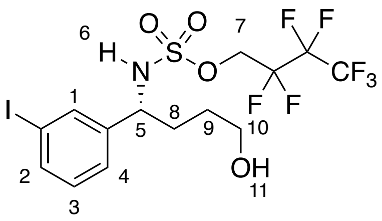

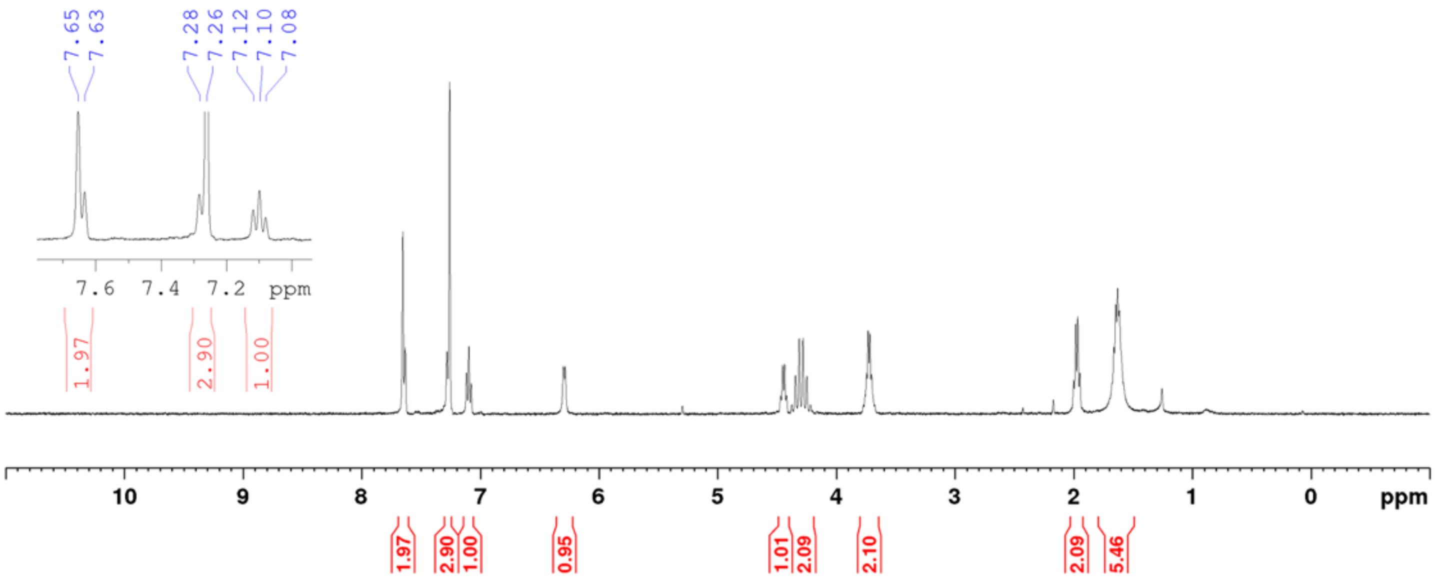

^{13}C NMR (126 MHz, $CDCl_3$) for *2,2,3,3,4,4,4-heptafluorobutyl (R)-(4-hydroxy-1-(3-iodophenyl)butyl)sulfamate* (**7j**)

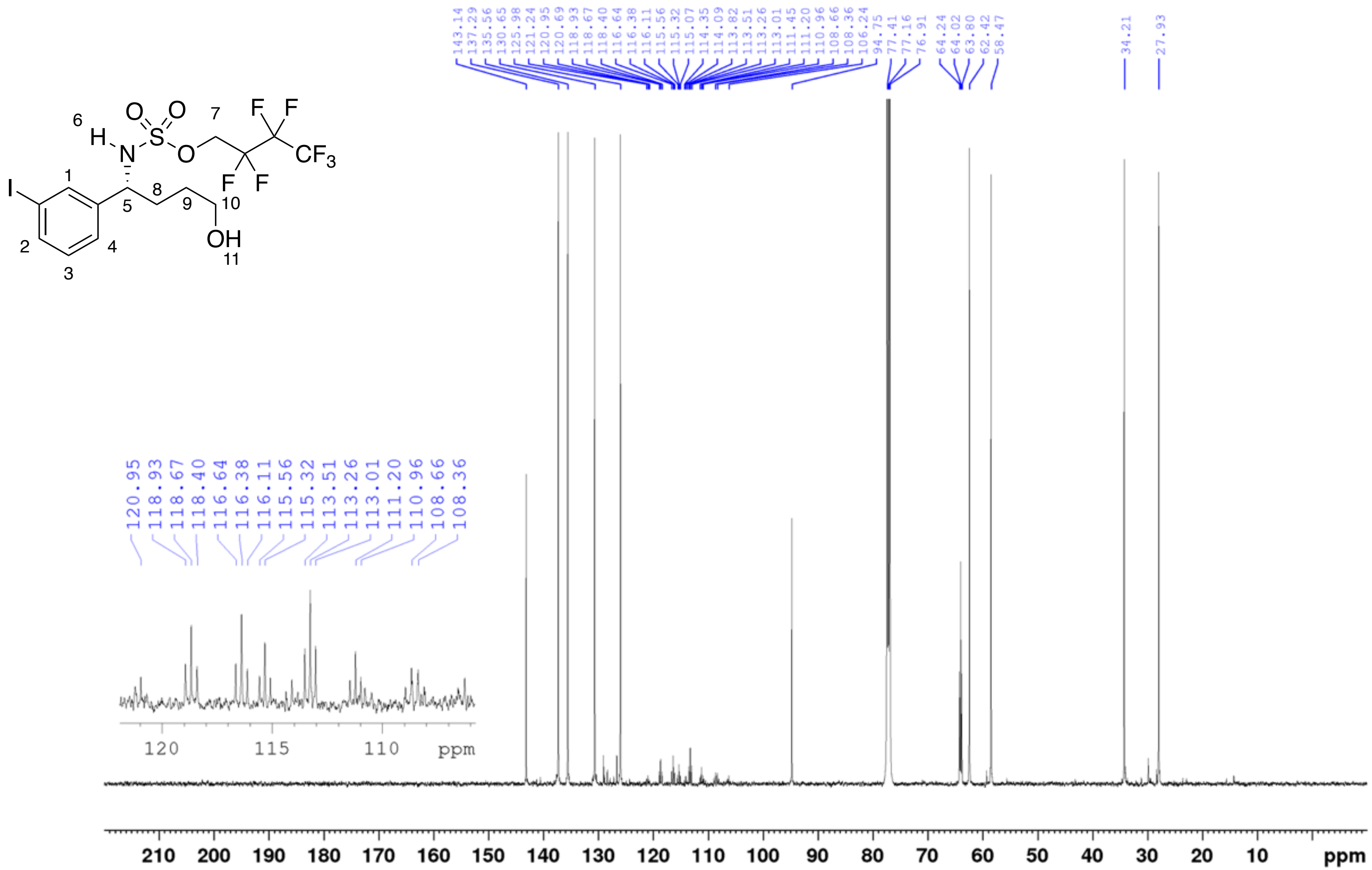

^{19}F NMR (376 MHz, $CDCl_3$) for *2,2,3,3,4,4,4-heptafluorobutyl (R)-(4-hydroxy-1-(3-iodophenyl)butyl)sulfamate* (**7j**)

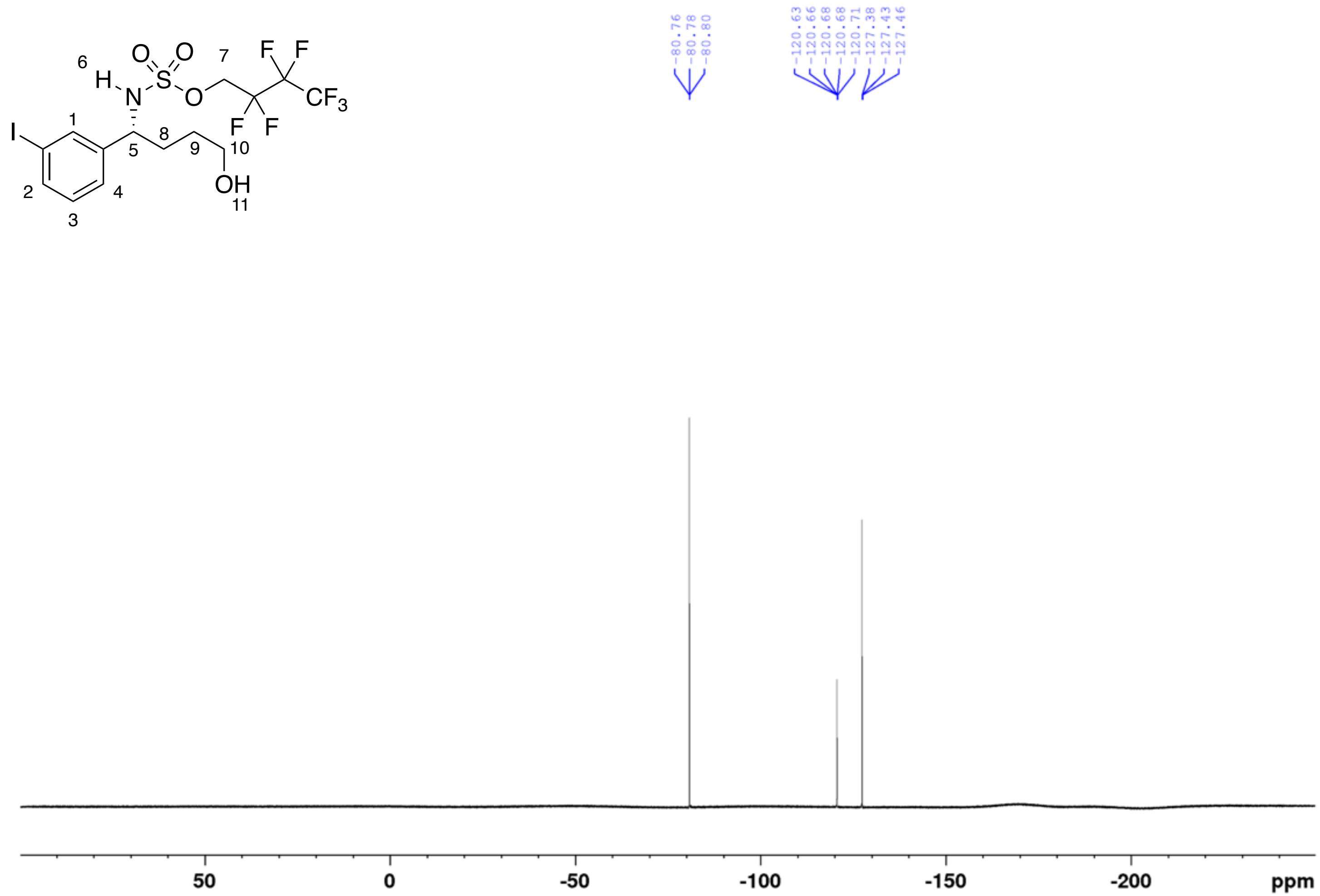

^{1}H NMR (700 MHz, $CDCl_3$) for *(R)-3-(1-(((2,2,3,3,4,4,4-heptafluorobutoxy)sulfonyl)amino)-4-hydroxybutyl)phenyl acetate* (**7k**)

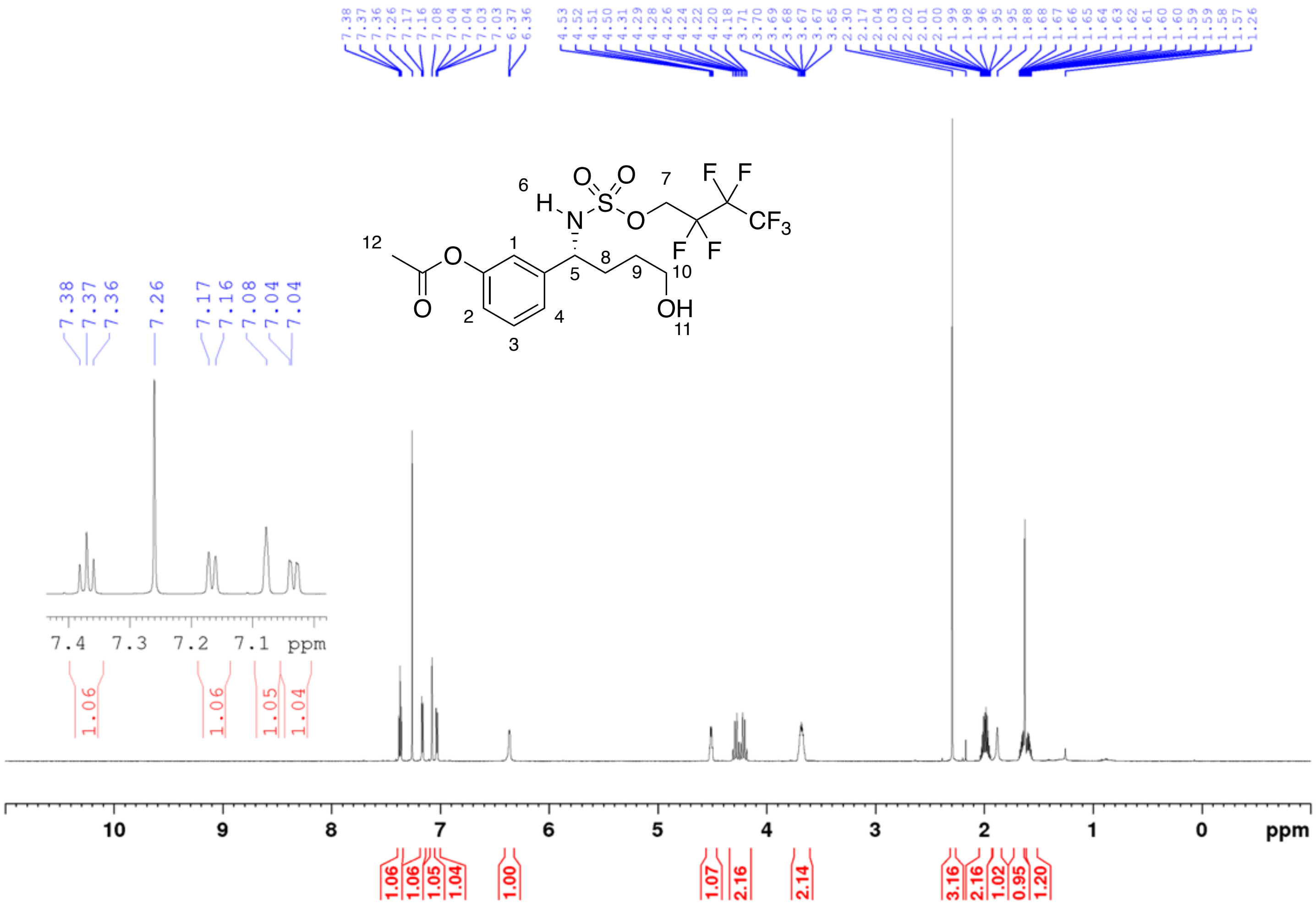

^{13}C NMR (126 MHz, $CDCl_3$) for *(R)-3-(1-(((2,2,3,3,4,4,4-heptafluorobutoxy)sulfonyl)amino)-4-hydroxybutyl)phenyl acetate* (**7k**)

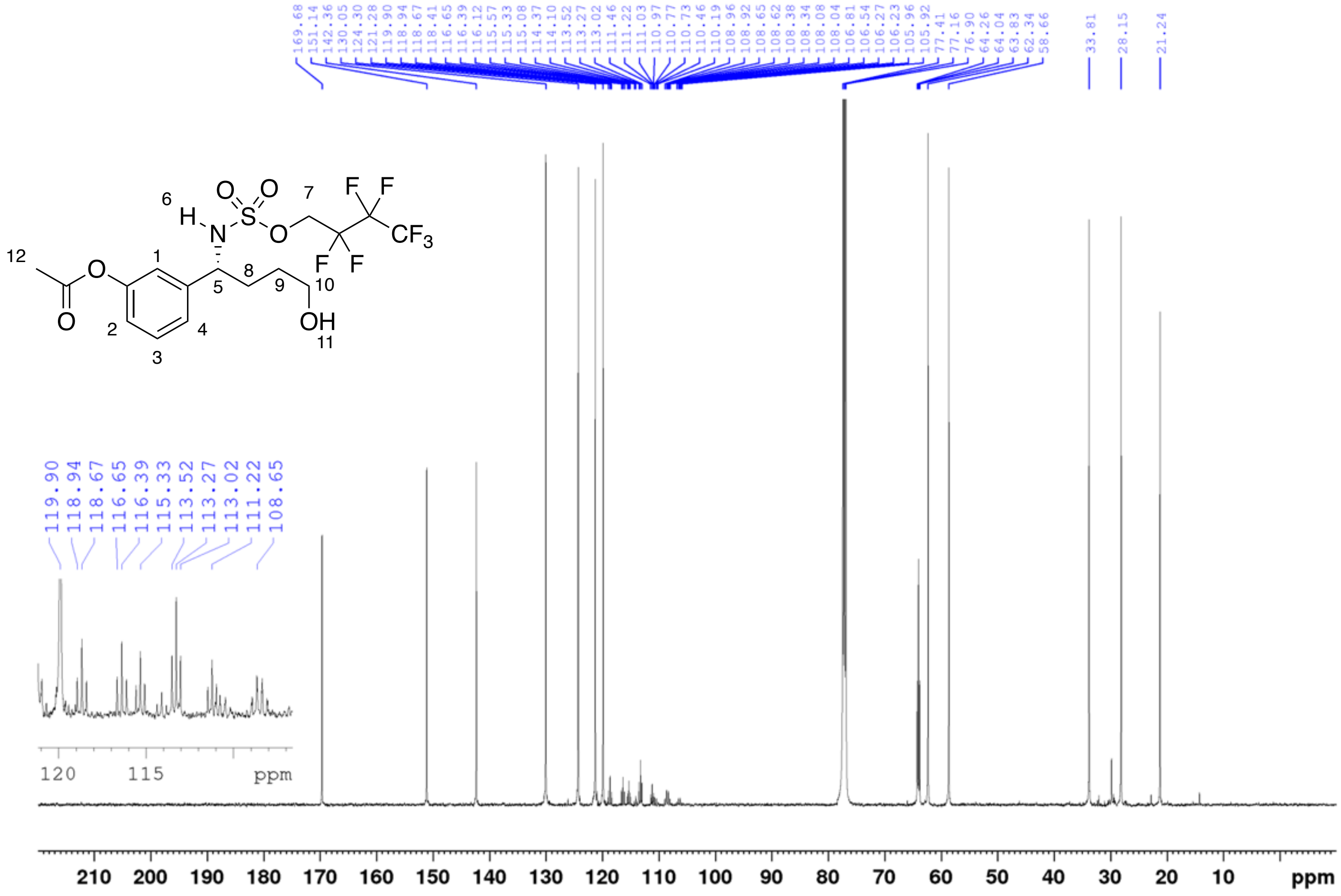

^{19}F NMR (376 MHz, $CDCl_3$) for *(R)-3-(1-(((2,2,3,3,4,4,4-heptafluorobutoxy)sulfonyl)amino)-4-hydroxybutyl)phenyl acetate* (**7k**)

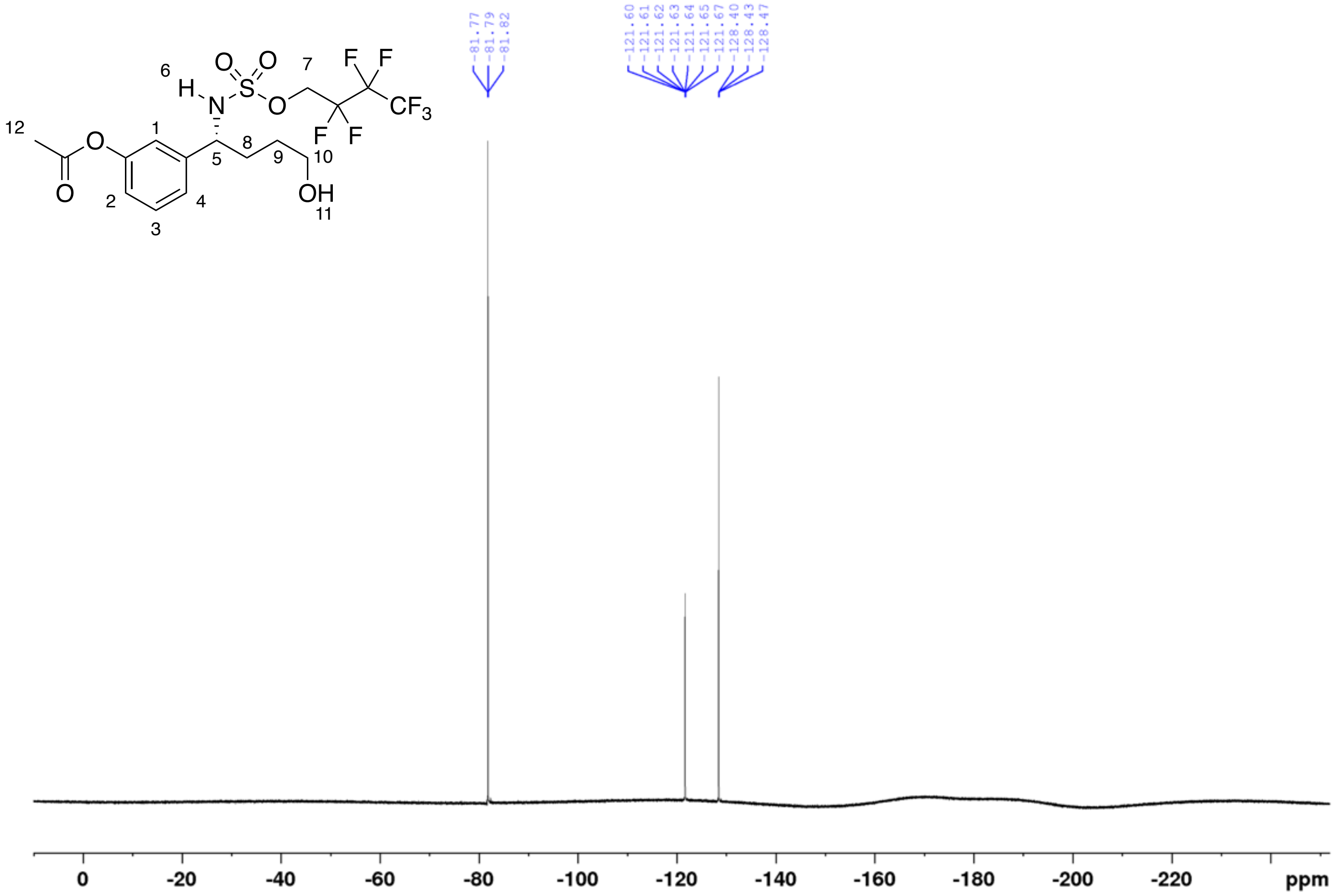

^{1}H NMR (500 MHz, $CDCl_3$) for *2,2,3,3,4,4,4-Heptafluorobutyl (R)-(4-hydroxy-1-(3-isopropylphenyl)butyl)sulfamate* (**7l**)

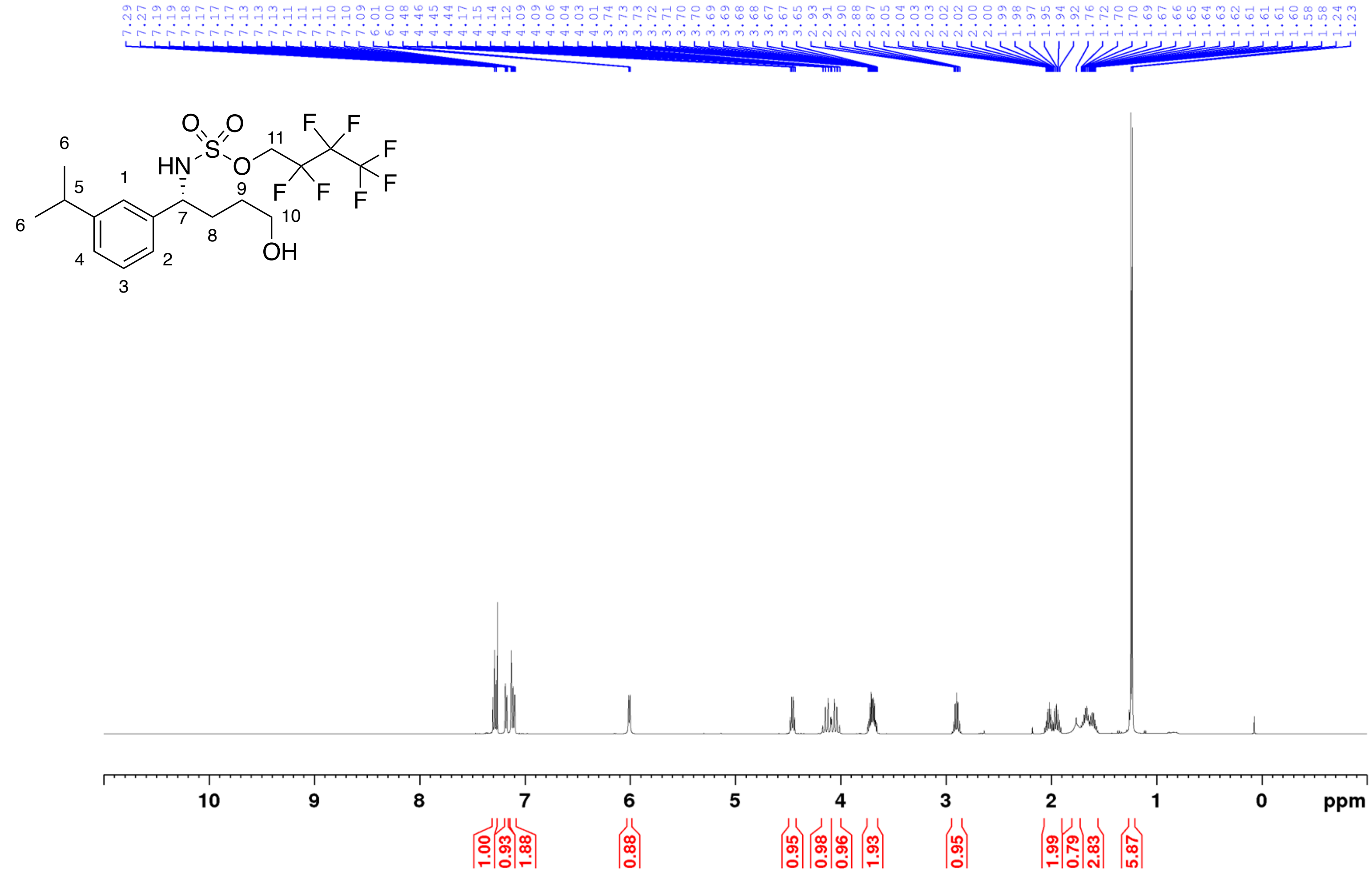

^{13}C NMR (126 MHz, $CDCl_3$) for *2,2,3,3,4,4,4-Heptafluorobutyl (R)-(4-hydroxy-1-(3-isopropylphenyl)butyl)sulfamate* (**7l**)

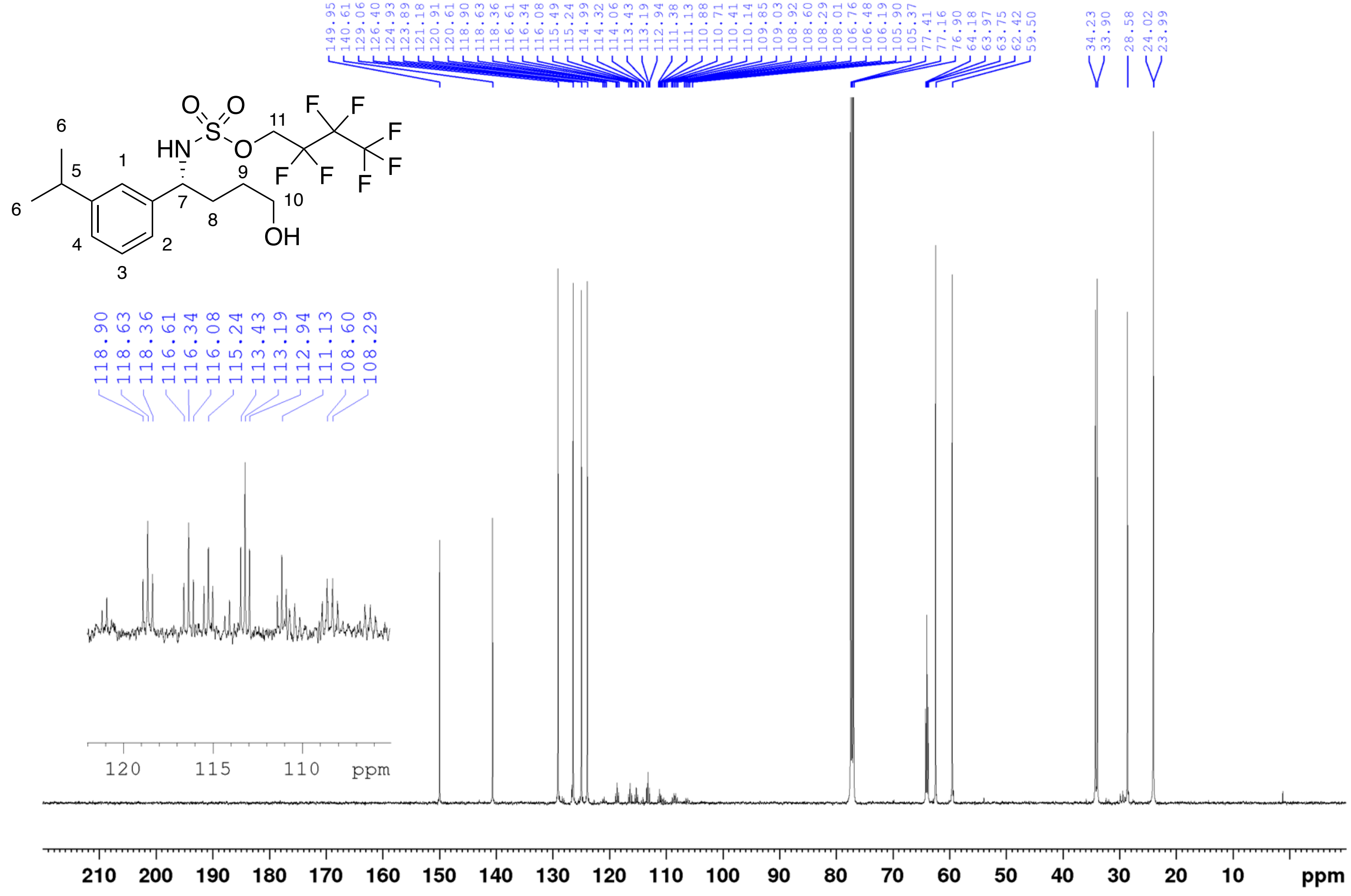

^{19}F NMR (376 MHz, $CDCl_3$) for *2,2,3,3,4,4,4-Heptafluorobutyl (R)-(4-hydroxy-1-(3-isopropylphenyl)butyl)sulfamate* (**7l**)

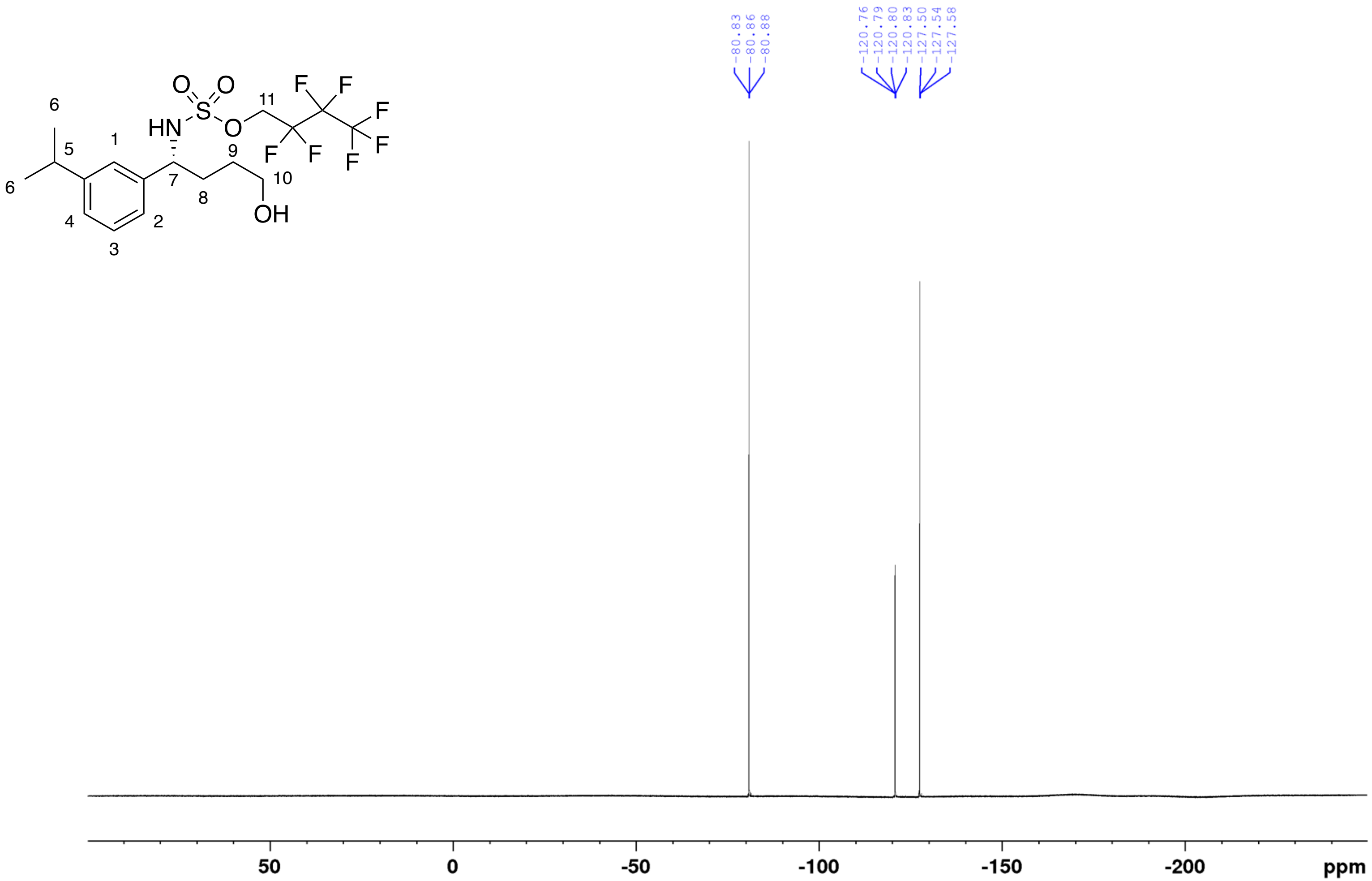

^{1}H NMR (500 MHz, $CDCl_3$) for *2,2,3,3,4,4,4-heptafluorobutyl (R)-(1-(2-chlorophenyl)-4-hydroxybutyl)sulfamate* (**7m**)

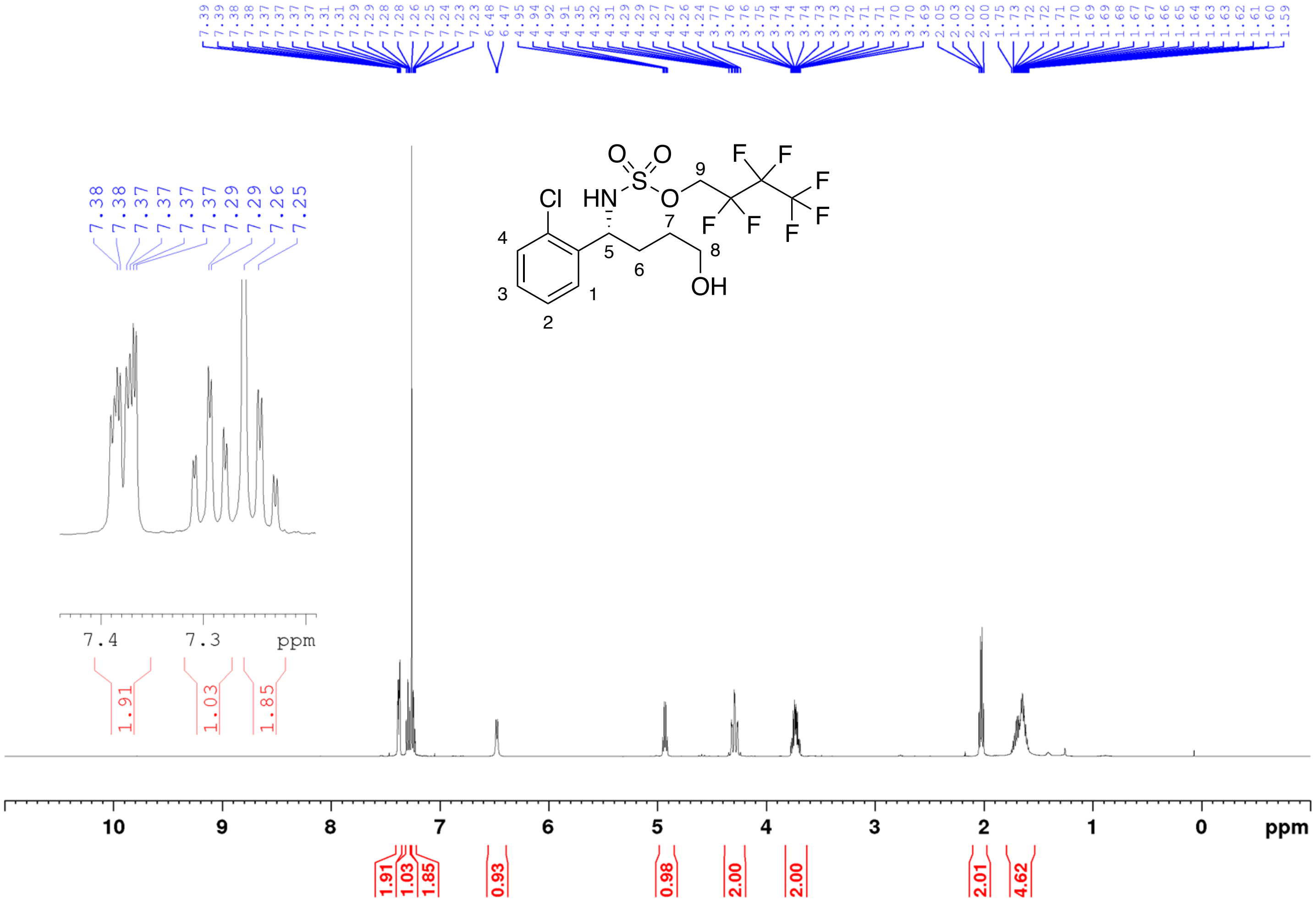

^{13}C NMR (126 MHz, $CDCl_3$) for *2,2,3,3,4,4,4-heptafluorobutyl (R)-(1-(2-chlorophenyl)-4-hydroxybutyl)sulfamate* (**7m**)

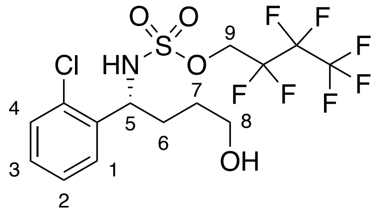

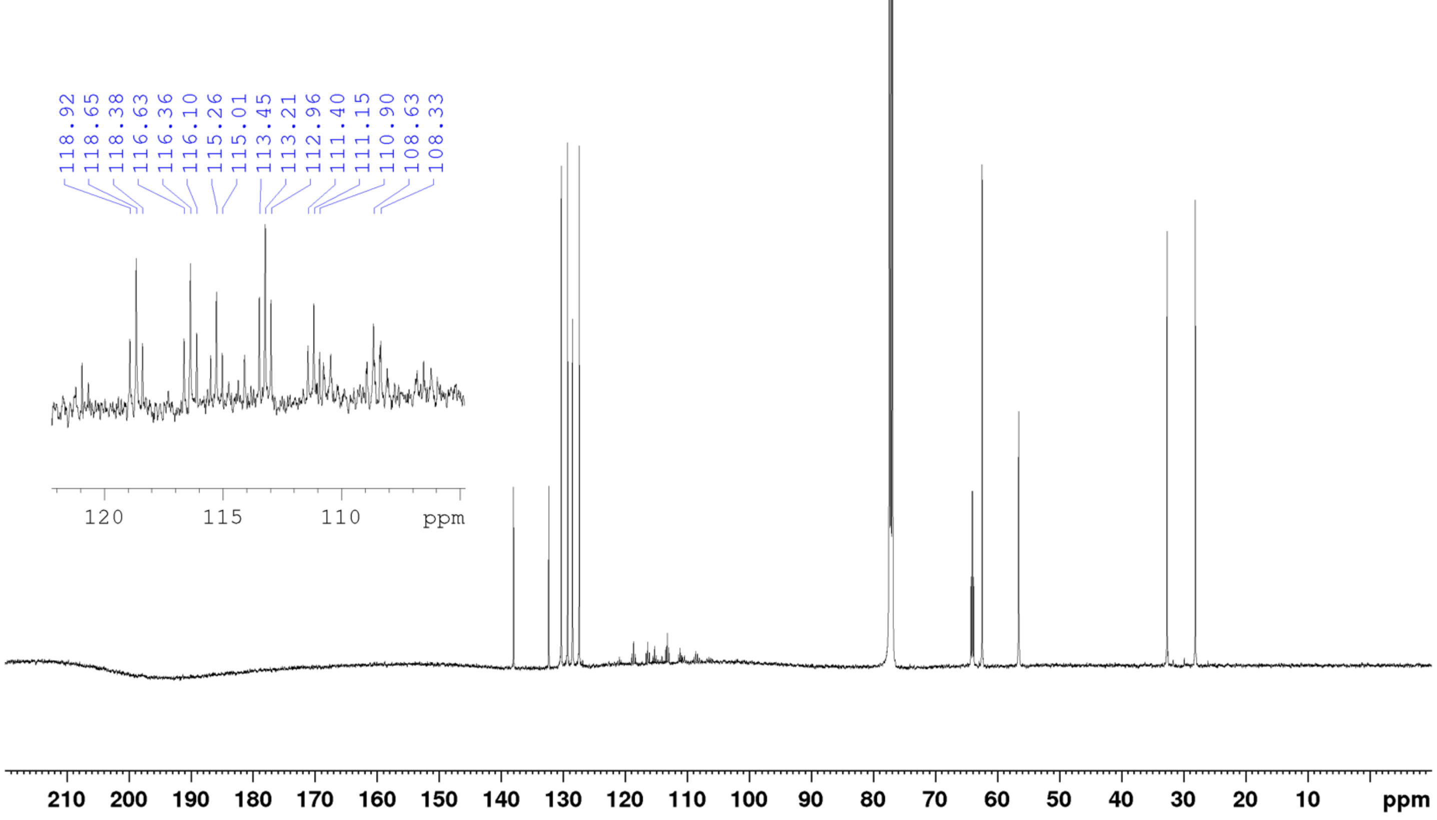

^{19}F NMR (376 MHz, $CDCl_3$) for *2,2,3,3,4,4,4-heptafluorobutyl (R)-(1-(2-chlorophenyl)-4-hydroxybutyl)sulfamate* (**7m**)

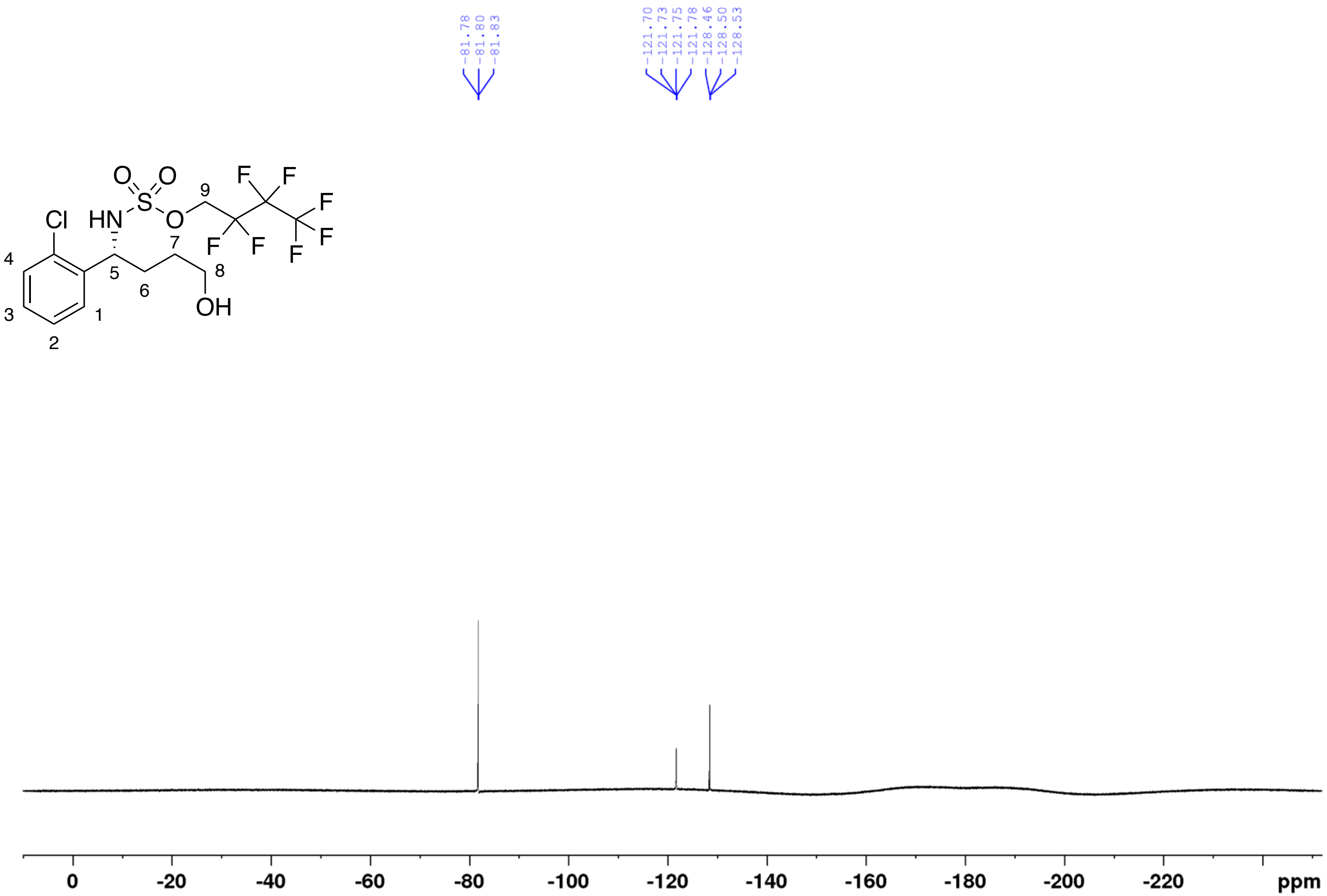

^{1}H NMR (500 MHz, $CDCl_3$) for *2,2,3,3,4,4,4-heptafluorobutyl (R)-(4-hydroxy-1-(4-methoxyphenyl)butyl)sulfamate* (**7n**)

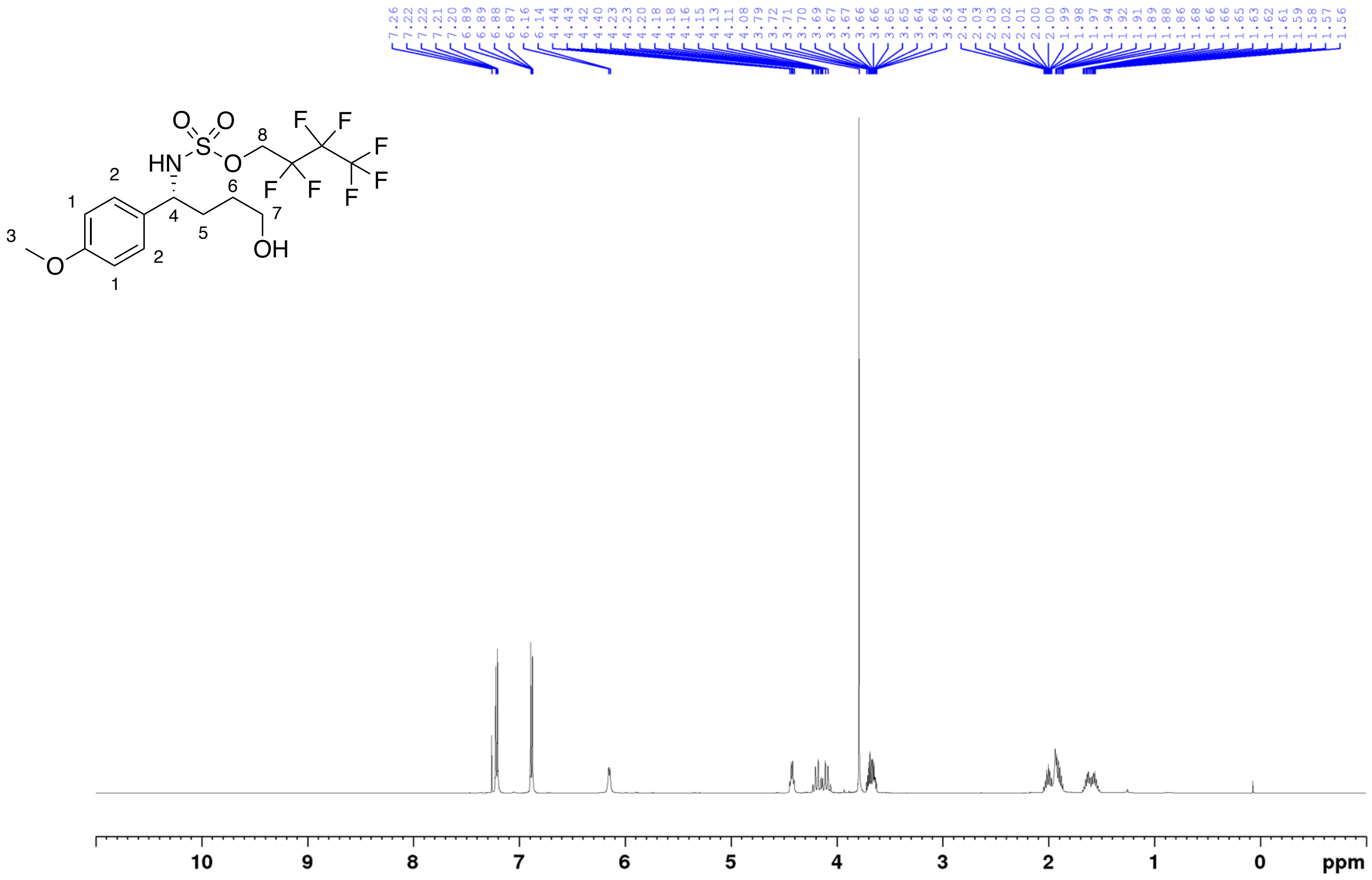

^{13}C NMR (126 MHz, $CDCl_3$) for *2,2,3,3,4,4,4-heptafluorobutyl (R)-(4-hydroxy-1-(4-methoxyphenyl)butyl)sulfamate* (**7n**)

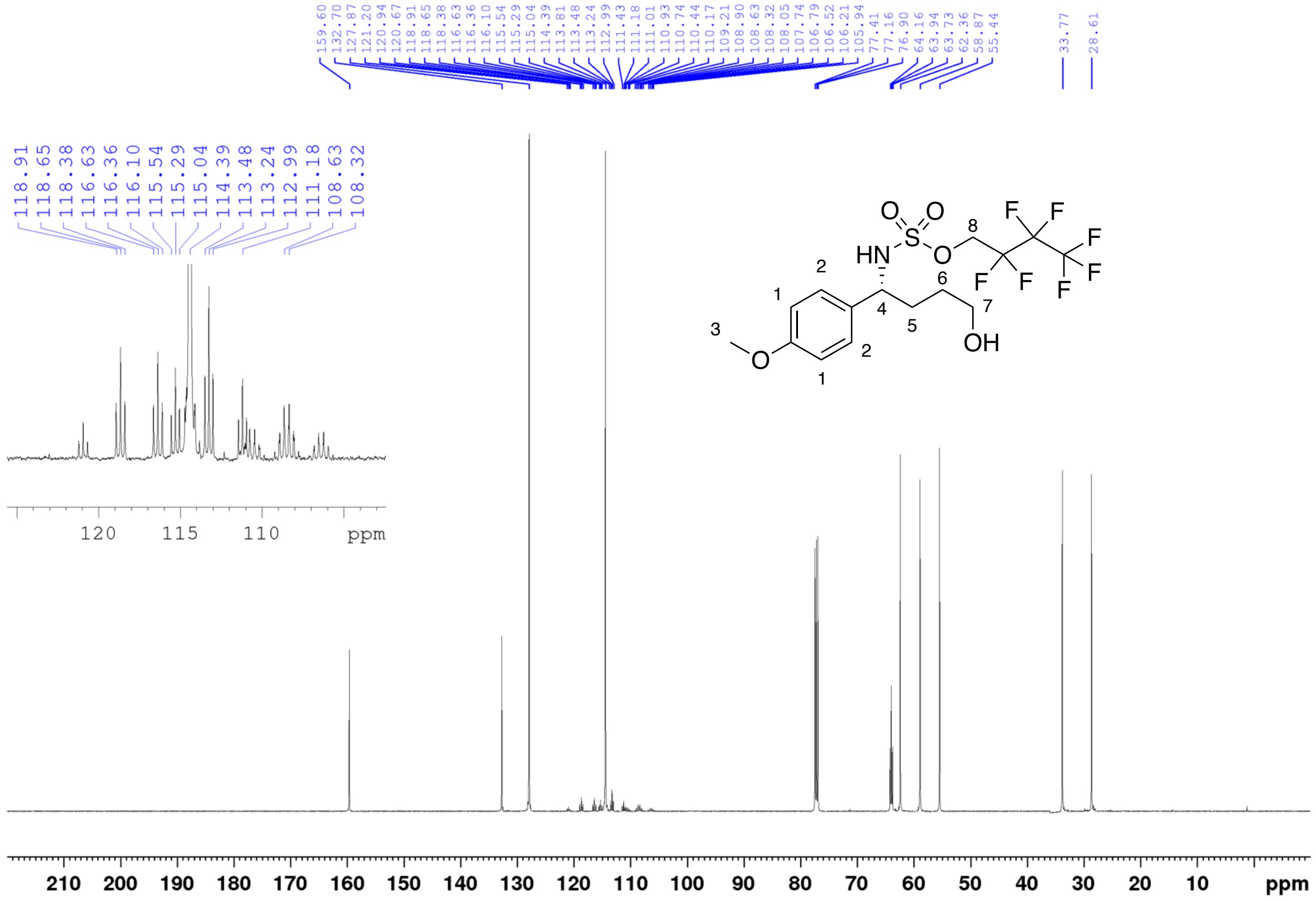

^{19}F NMR (376 MHz, $CDCl_3$) for *2,2,3,3,4,4,4-heptafluorobutyl (R)-(4-hydroxy-1-(4-methoxyphenyl)butyl)sulfamate* (**7n**)

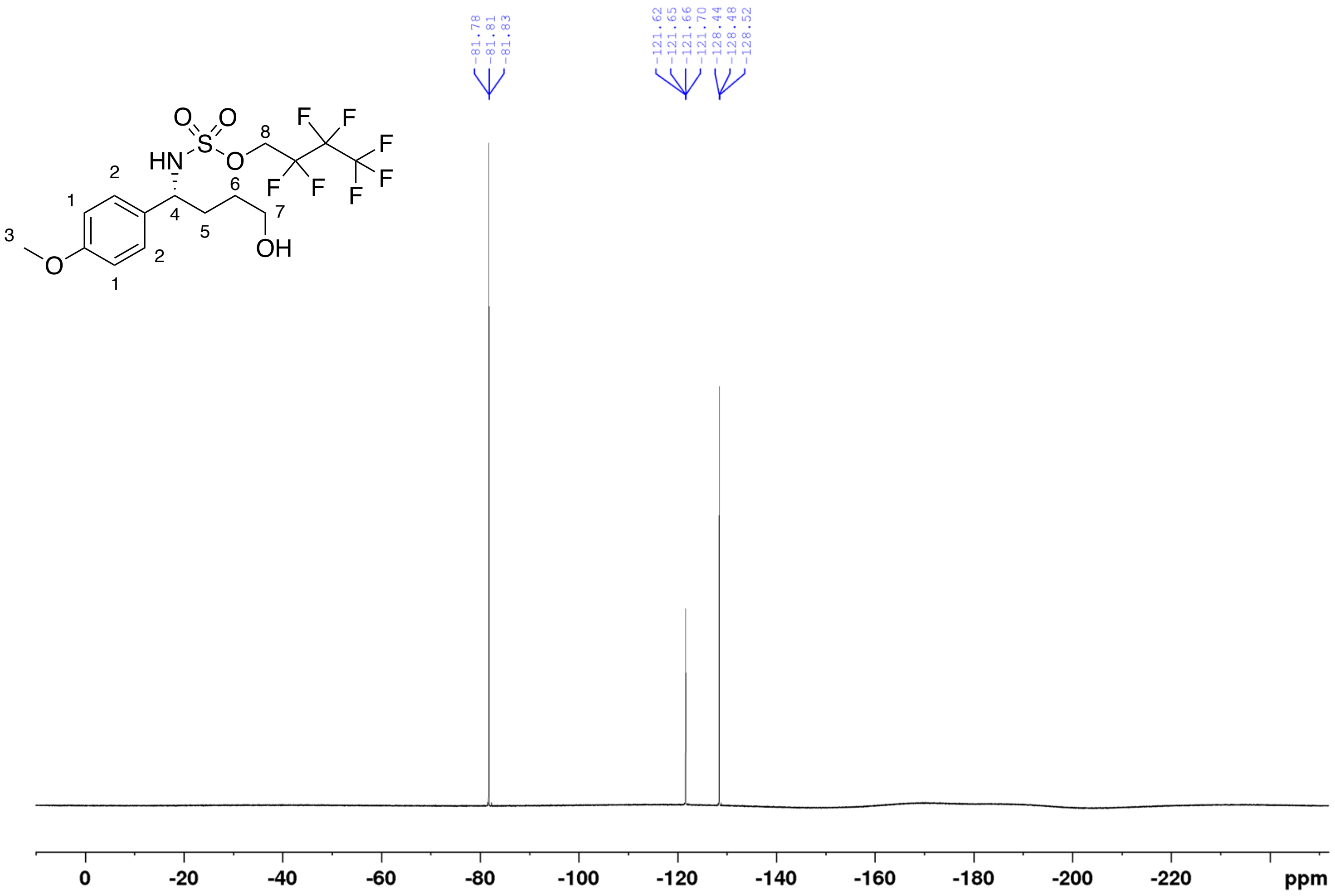

¹H NMR (500 MHz, $CDCl_3$) for *2,2,3,3,4,4,4-heptafluorobutyl (R)-(1-(4-chlorophenyl)-4-hydroxybutyl)sulfamate* (**7o**)

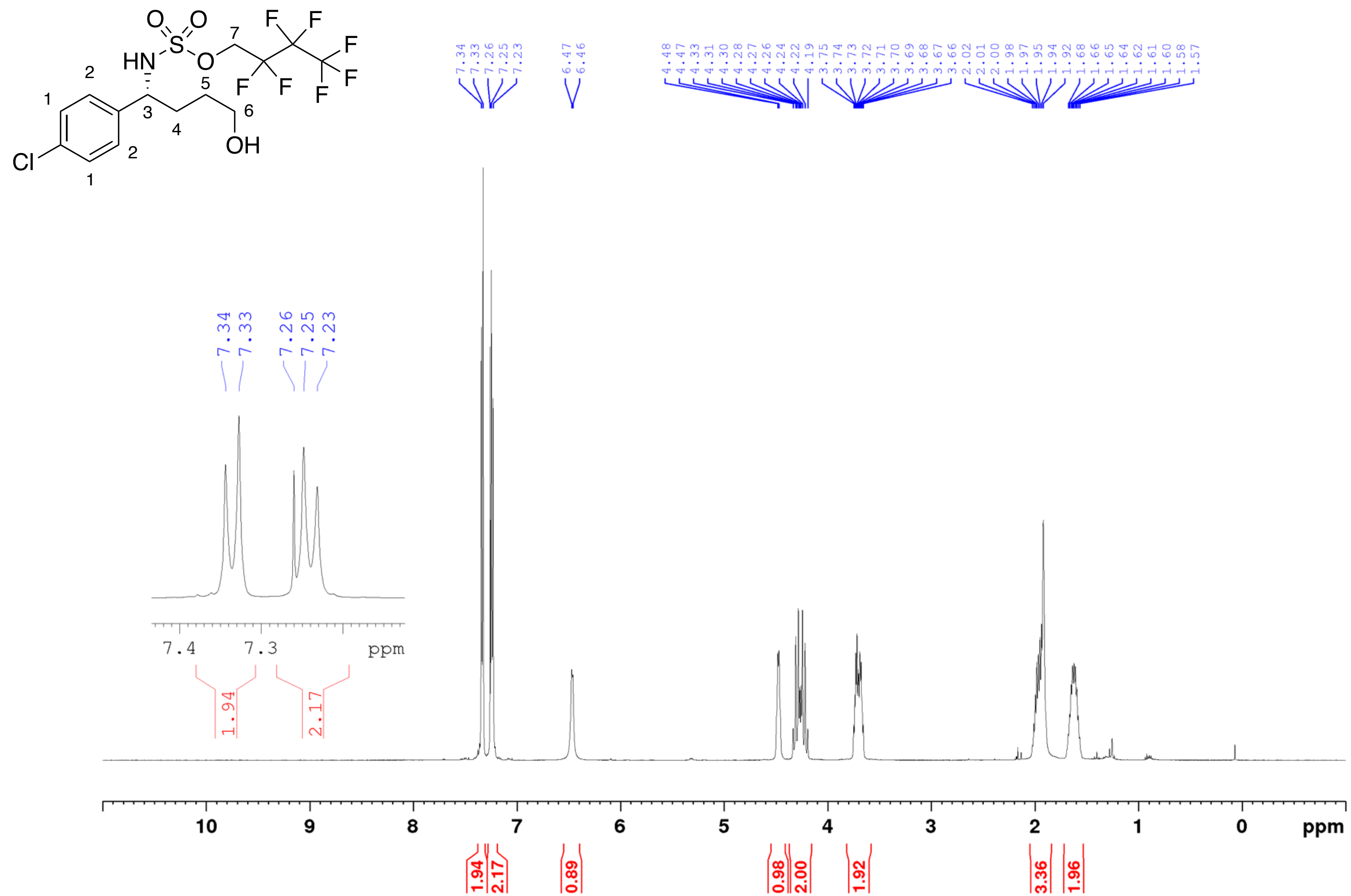

^{13}C NMR (126 MHz, $CDCl_3$) for *2,2,3,3,4,4,4-heptafluorobutyl (R)-(1-(4-chlorophenyl)-4-hydroxybutyl)sulfamate* (**7o**)

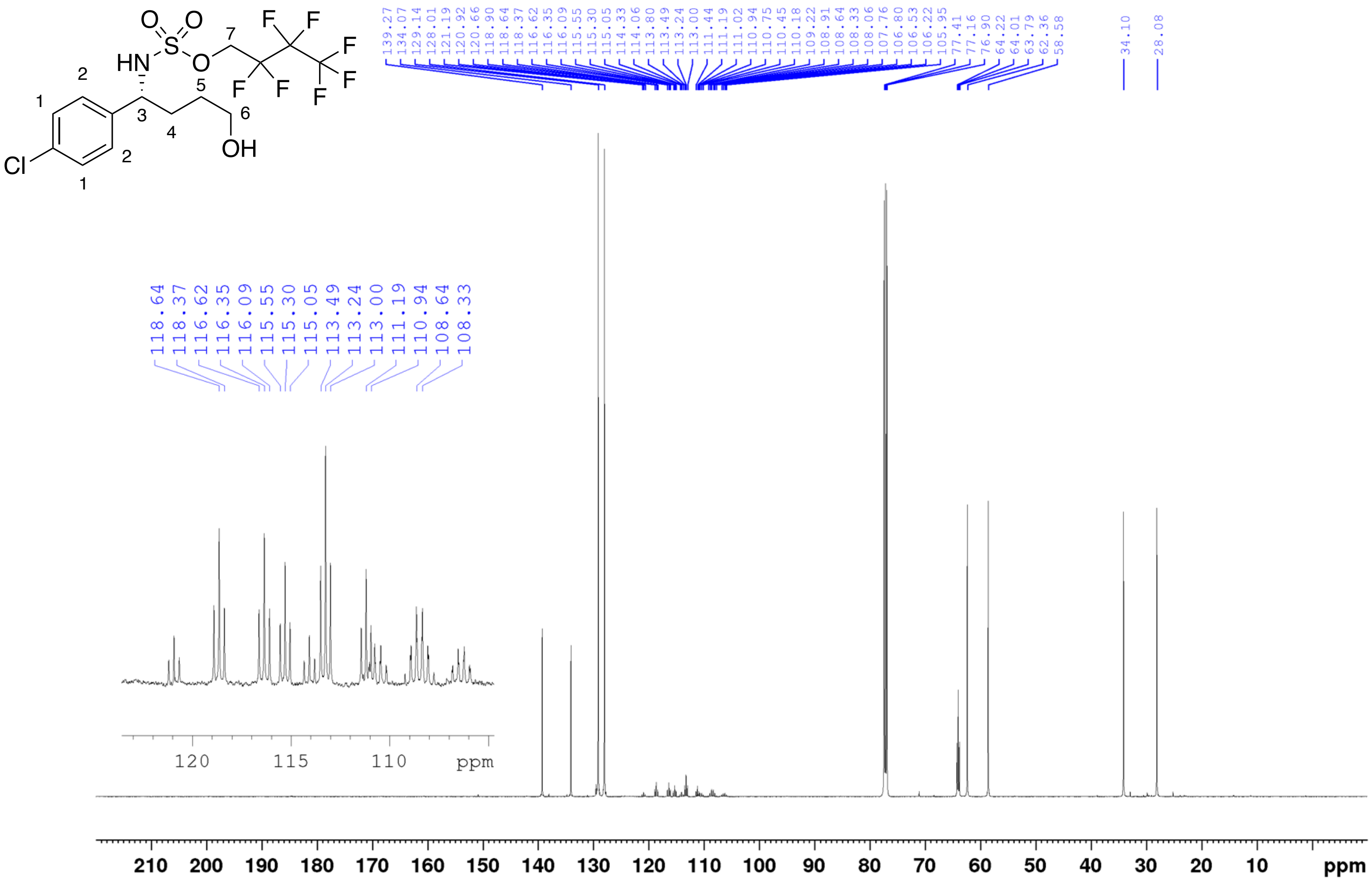

^{19}F NMR (376 MHz, $CDCl_3$) for *2,2,3,3,4,4,4-heptafluorobutyl (R)-(1-(4-chlorophenyl)-4-hydroxybutyl)sulfamate* (**7o**)

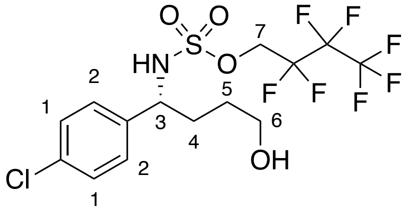

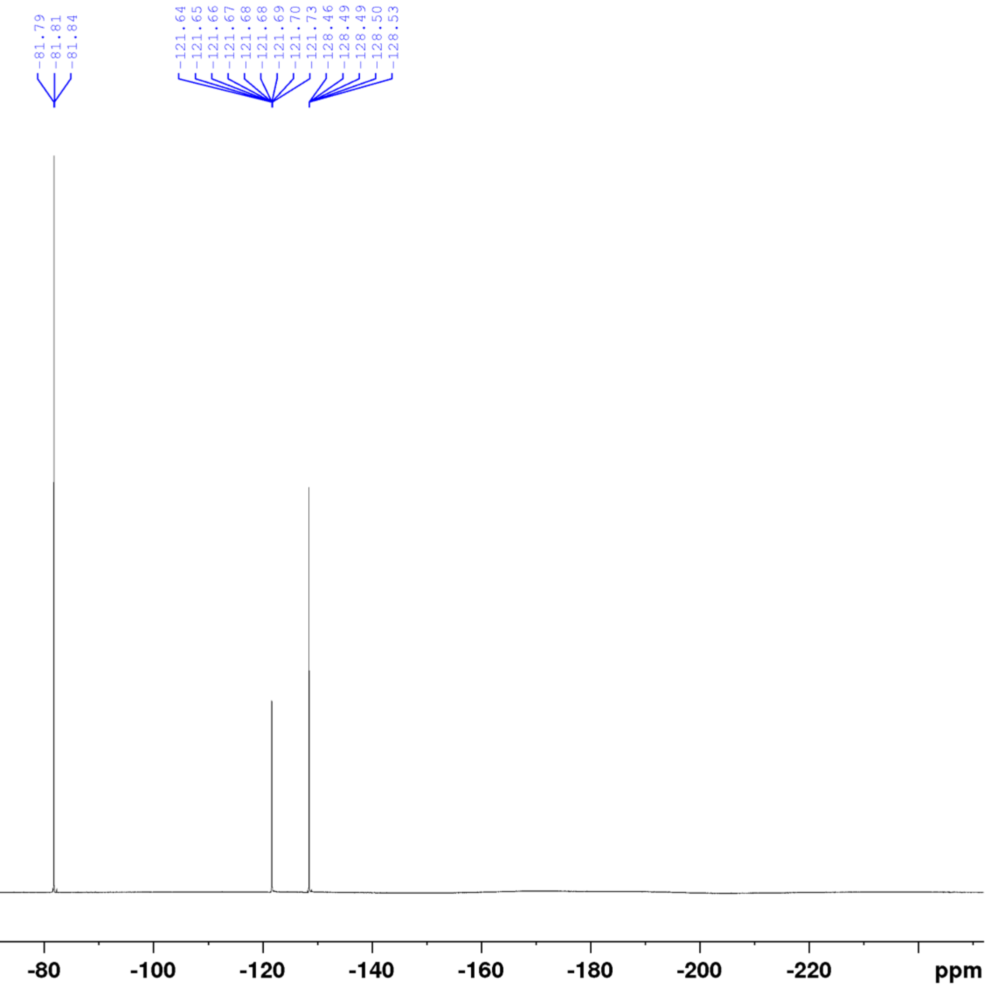

¹H NMR (500 MHz, $CDCl_3$) for *2,2,3,3,4,4,4-heptafluorobutyl (R)-(1-(5-chloro-2-methylphenyl)-4-hydroxybutyl)sulfamate* (**7p**)

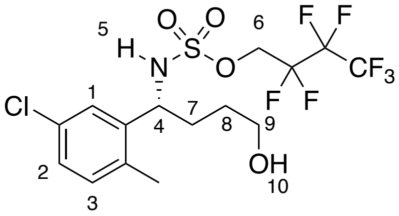

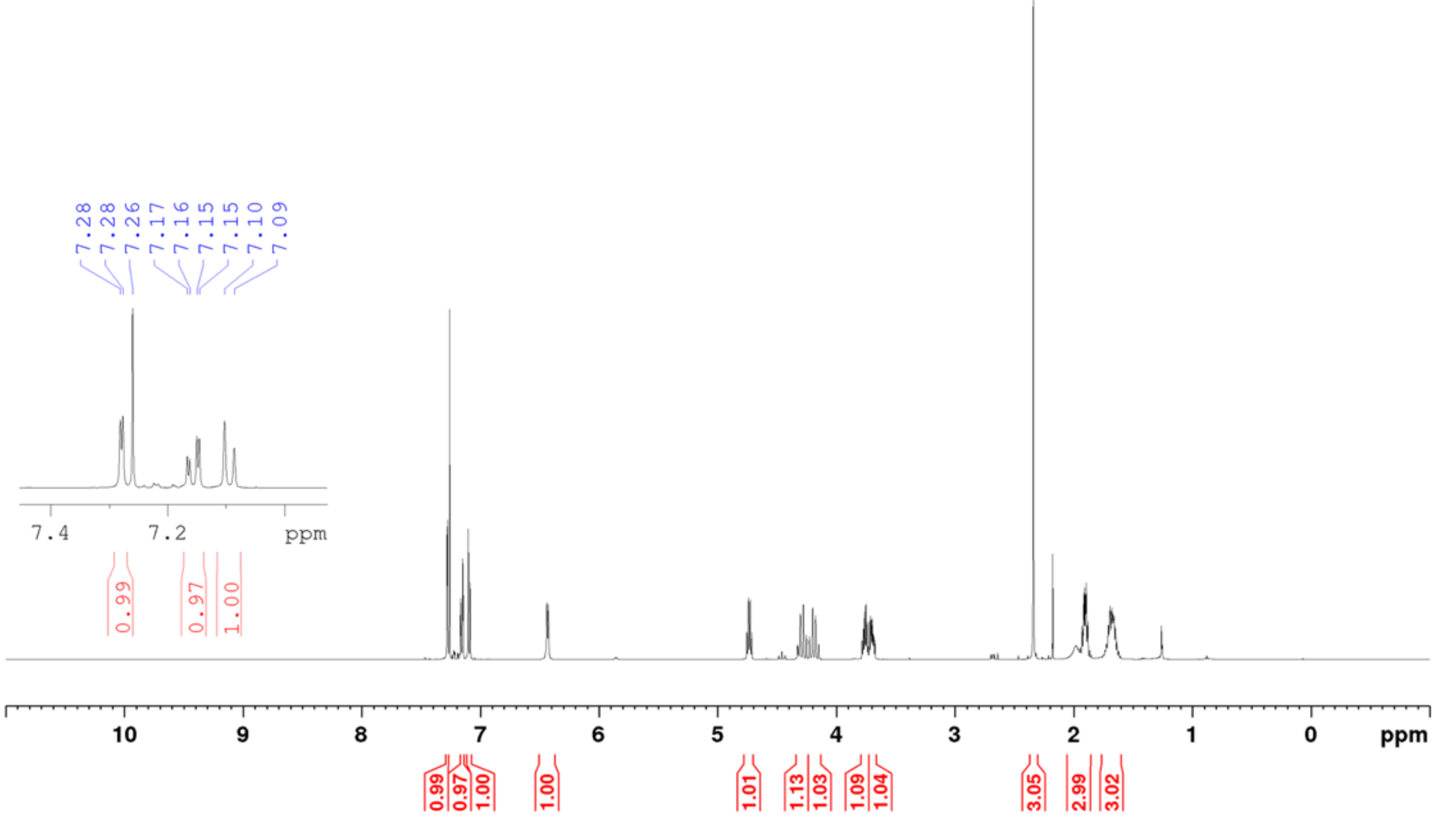

^{13}C NMR (126 MHz, $CDCl_3$) for *2,2,3,3,4,4,4-heptafluorobutyl (R)-(1-(5-chloro-2-methylphenyl)-4-hydroxybutyl)sulfamate* (**7p**)

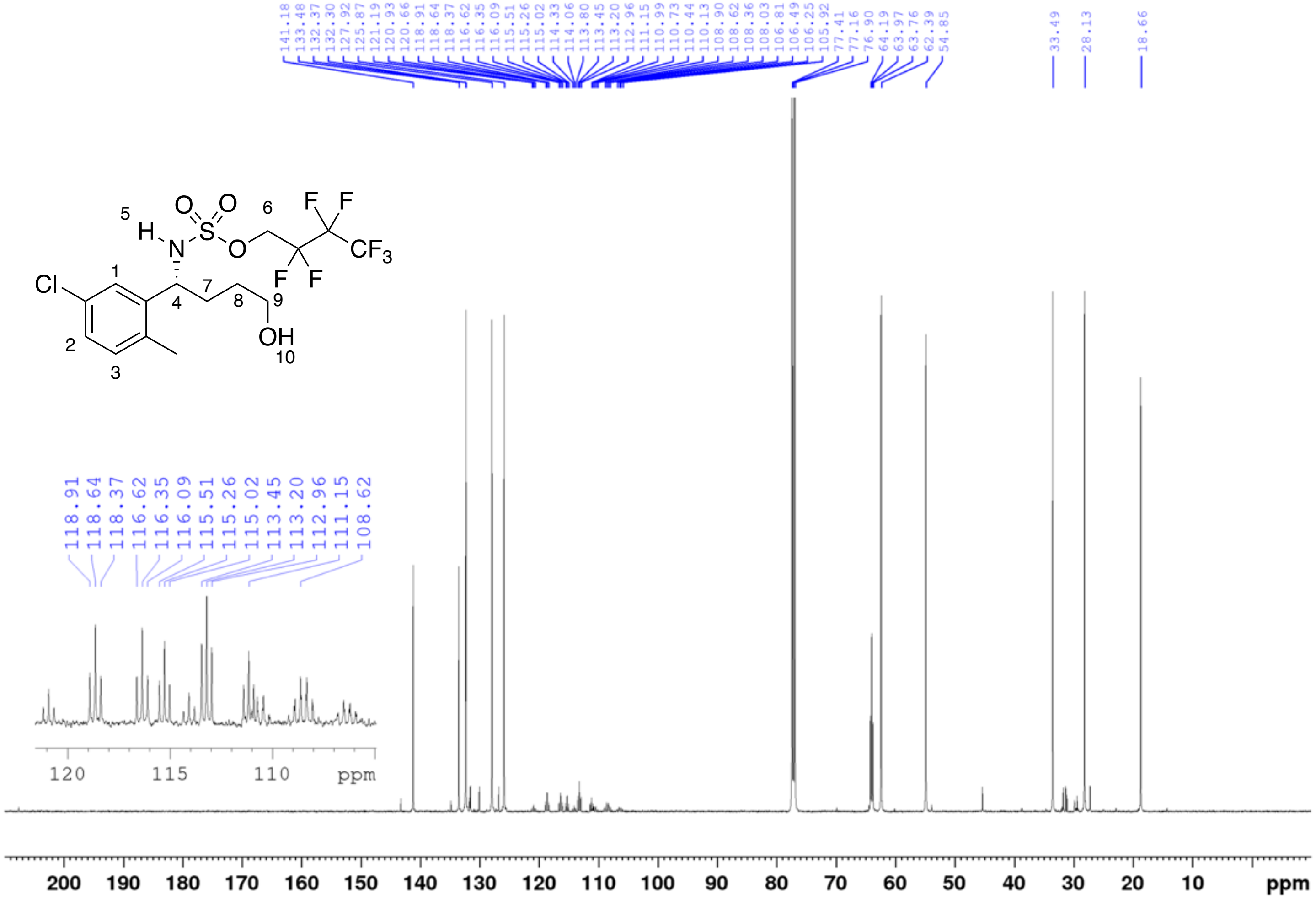

^{19}F NMR (376 MHz, $CDCl_3$) for *2,2,3,3,4,4,4-heptafluorobutyl (R)-(1-(5-chloro-2-methylphenyl)-4-hydroxybutyl)sulfamate* (**7p**)

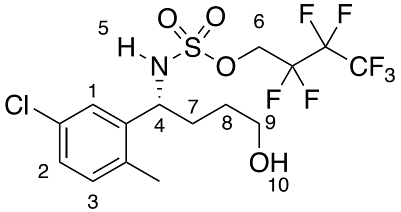

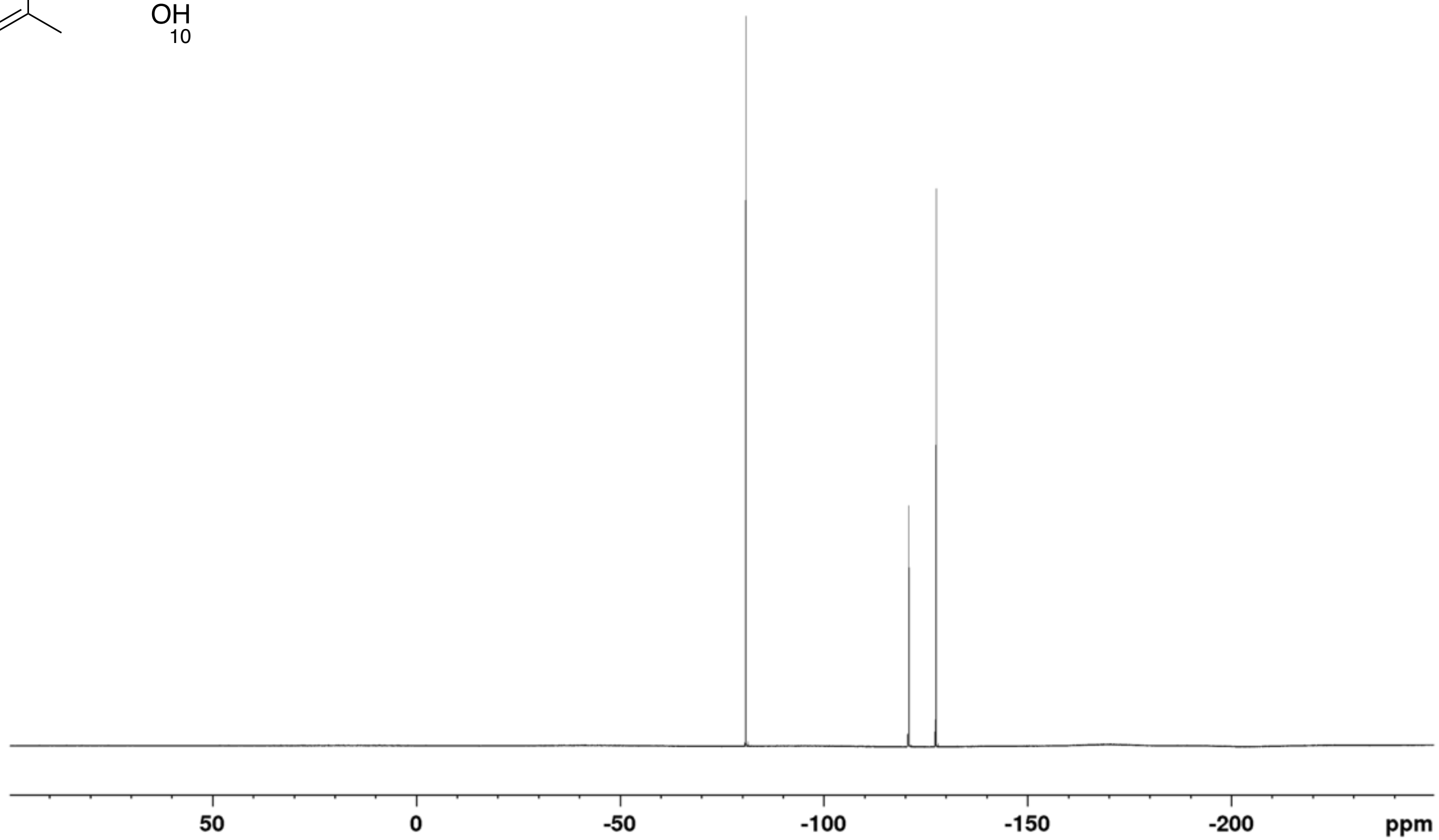

^{1}H NMR (500 MHz, $CDCl_3$) for *2,2,3,3,4,4,4-heptafluorobutyl (R)-(1-(2,3-dimethylphenyl)-4-hydroxybutyl)sulfamate* (**7q**)

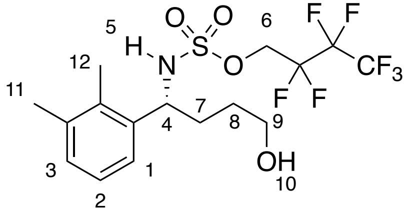

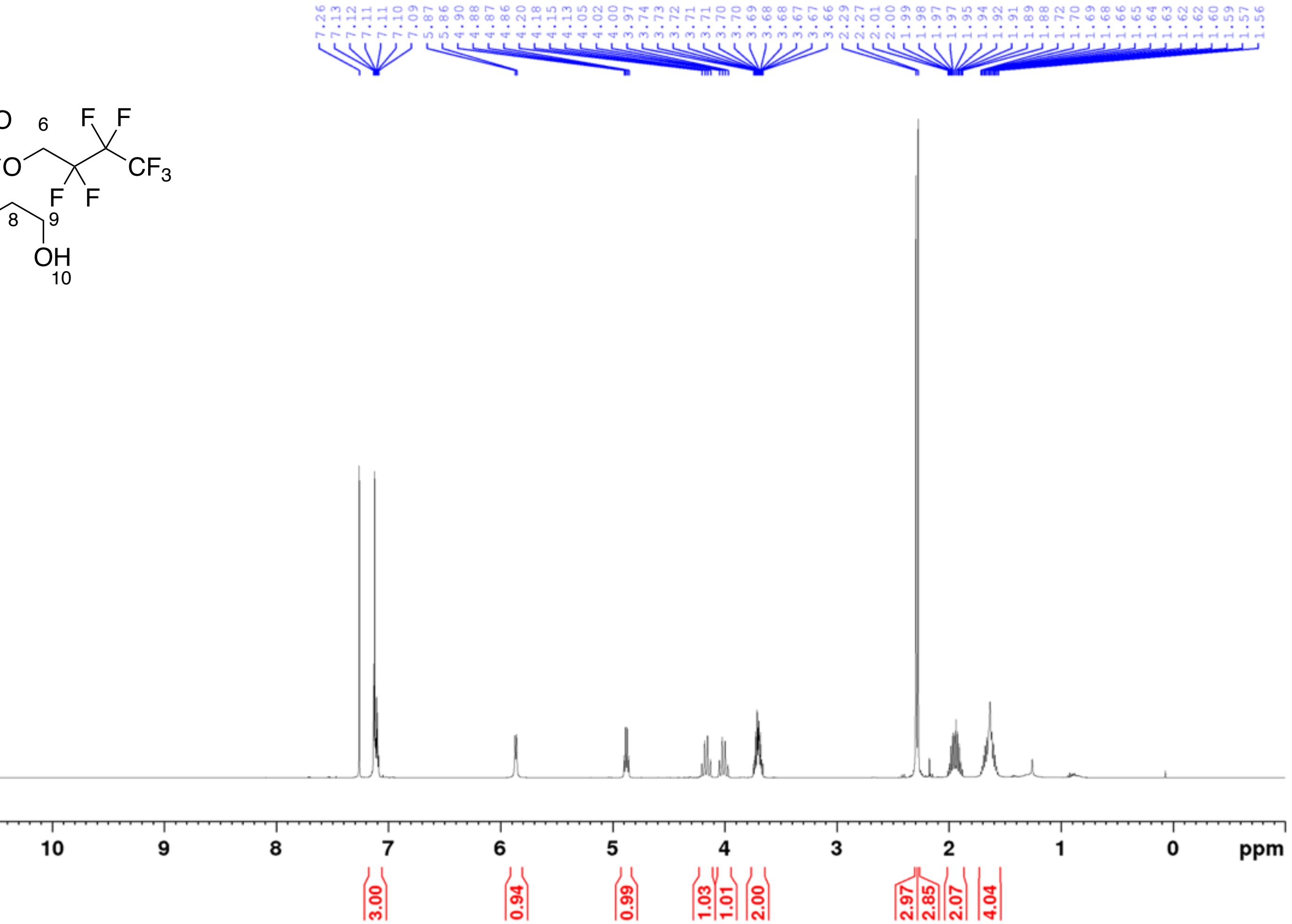

^{13}C NMR (126 MHz, $CDCl_3$) for *2,2,3,3,4,4,4-heptafluorobutyl (R)-(1-(2,3-dimethylphenyl)-4-hydroxybutyl)sulfamate* (**7q**)

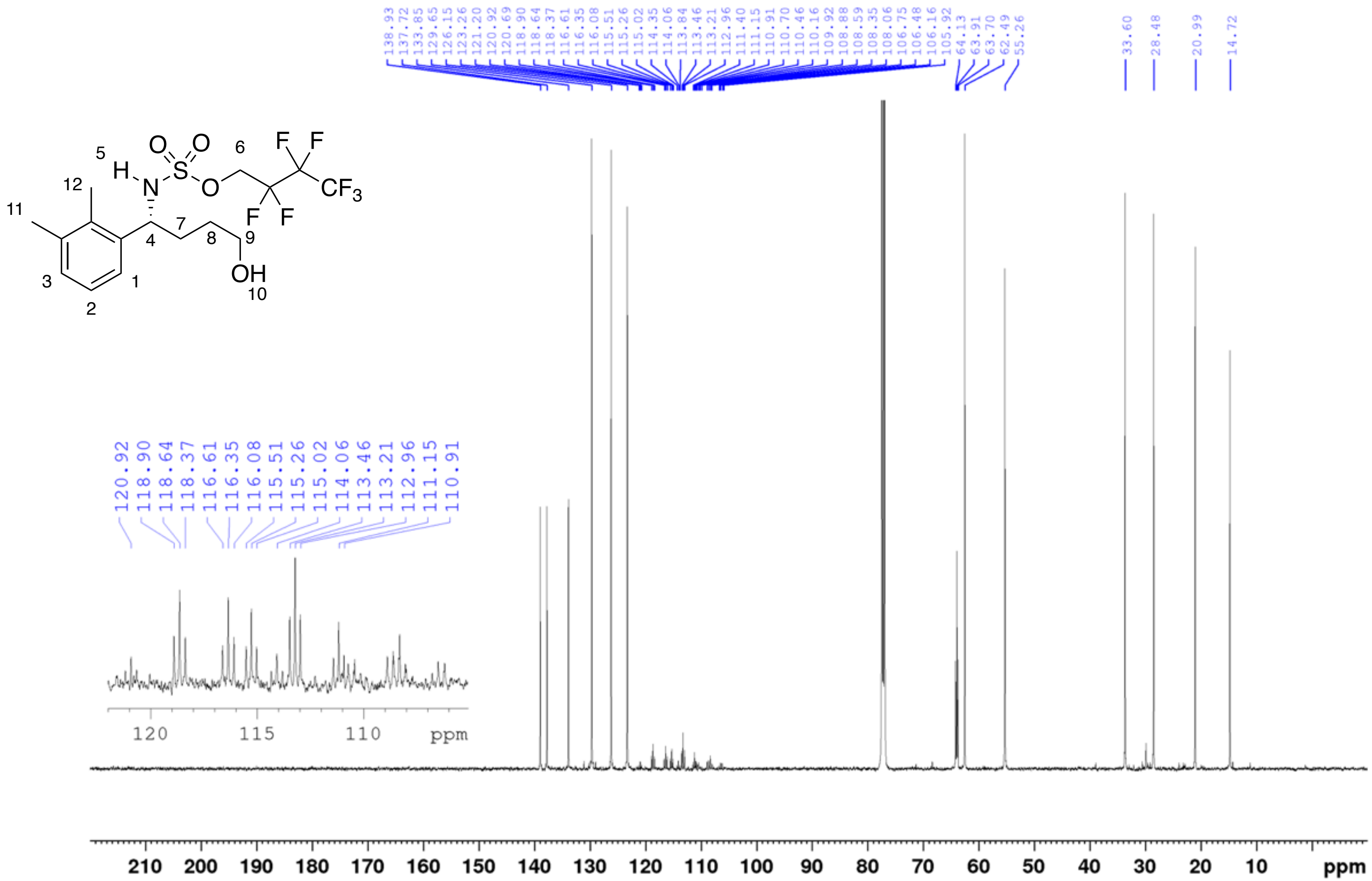

^{19}F NMR (376 MHz, $CDCl_3$) for *2,2,3,3,4,4,4-heptafluorobutyl (R)-(1-(2,3-dimethylphenyl)-4-hydroxybutyl)sulfamate* (**7q**)

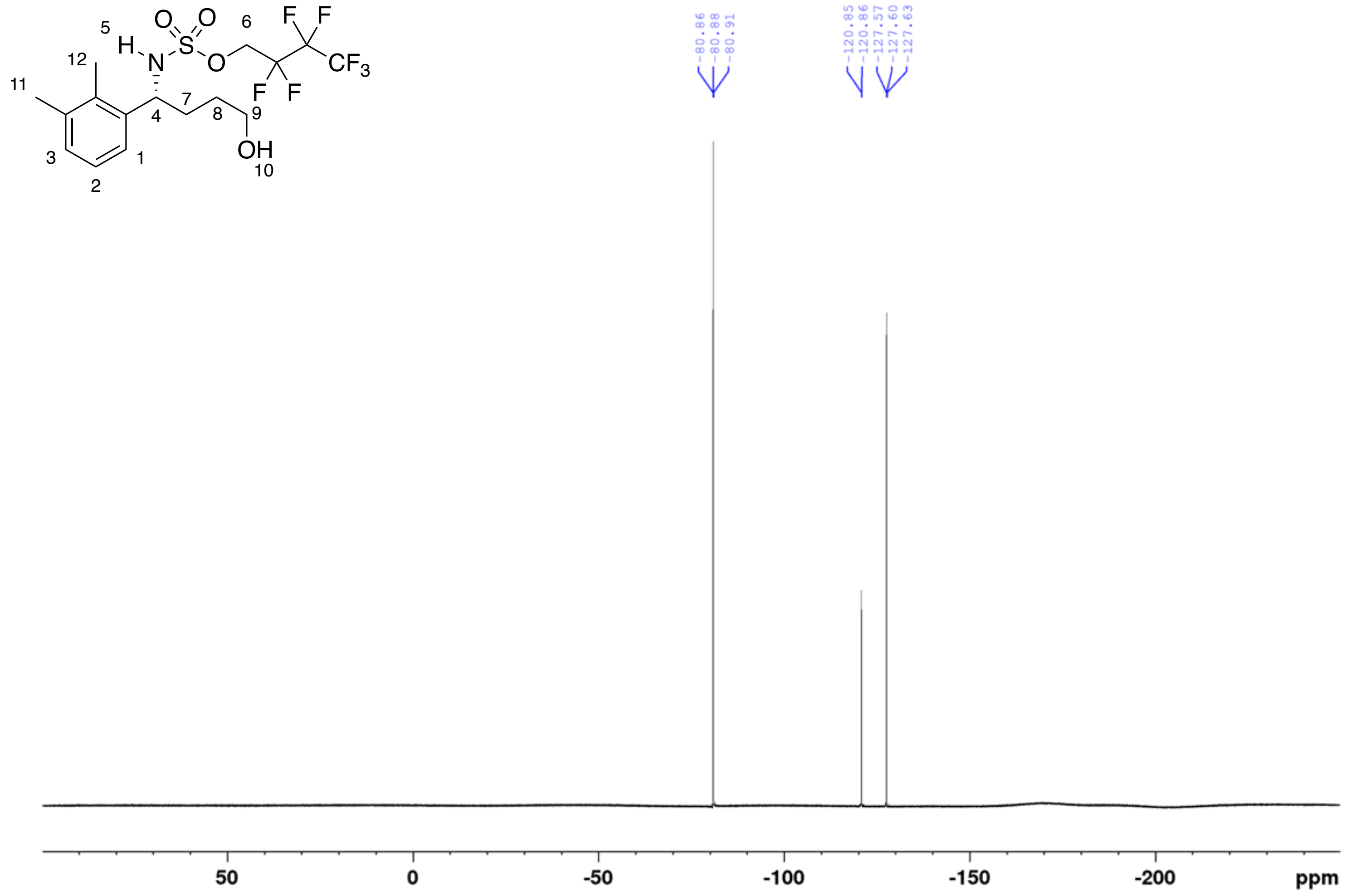

^{1}H NMR (700 MHz, $CDCl_3$) for *2,2,3,3,4,4,4-heptafluorobutyl (R)-(1-(4-fluoro-2-methylphenyl)-4-hydroxybutyl)sulfamate* (**7r**)

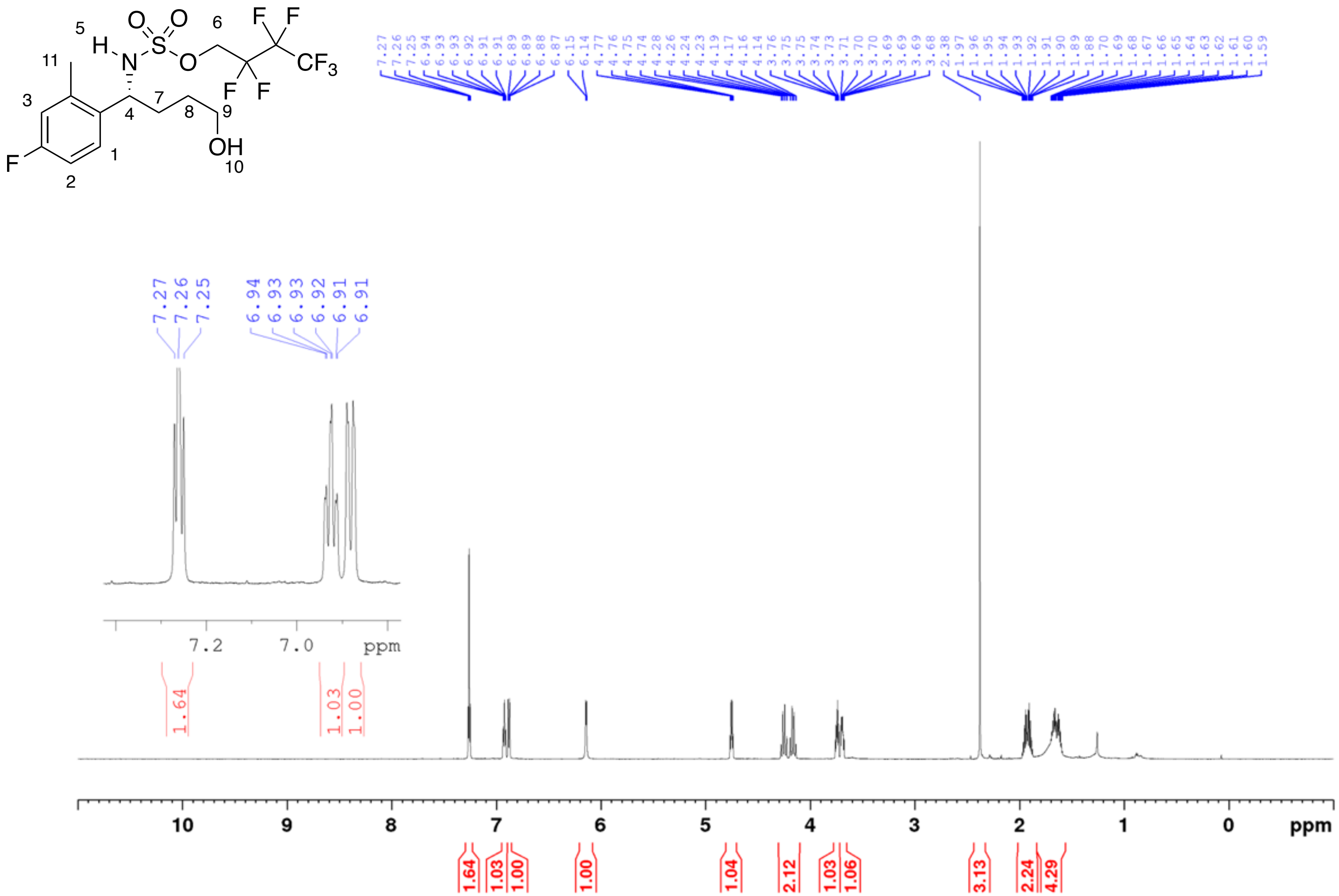

^{13}C NMR (126 MHz, $CDCl_3$) for *2,2,3,3,4,4,4-heptafluorobutyl (R)-(1-(4-fluoro-2-methylphenyl)-4-hydroxybutyl)sulfamate* (**7r**)

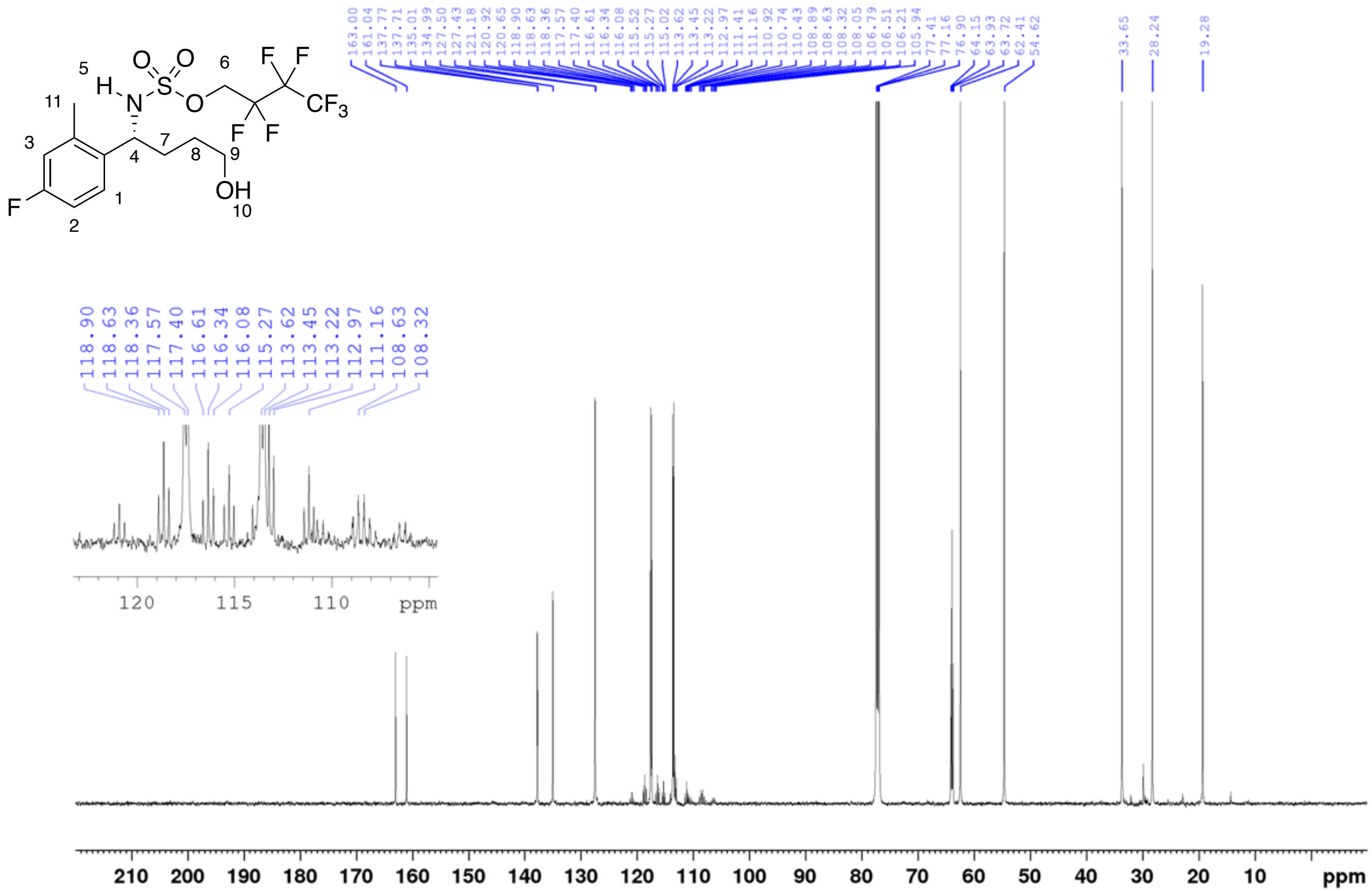

^{19}F NMR (376 MHz, $CDCl_3$) for *2,2,3,3,4,4,4-heptafluorobutyl (R)-(1-(4-fluoro-2-methylphenyl)-4-hydroxybutyl)sulfamate* (**7r**)

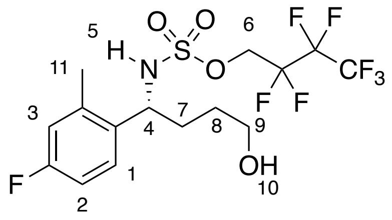

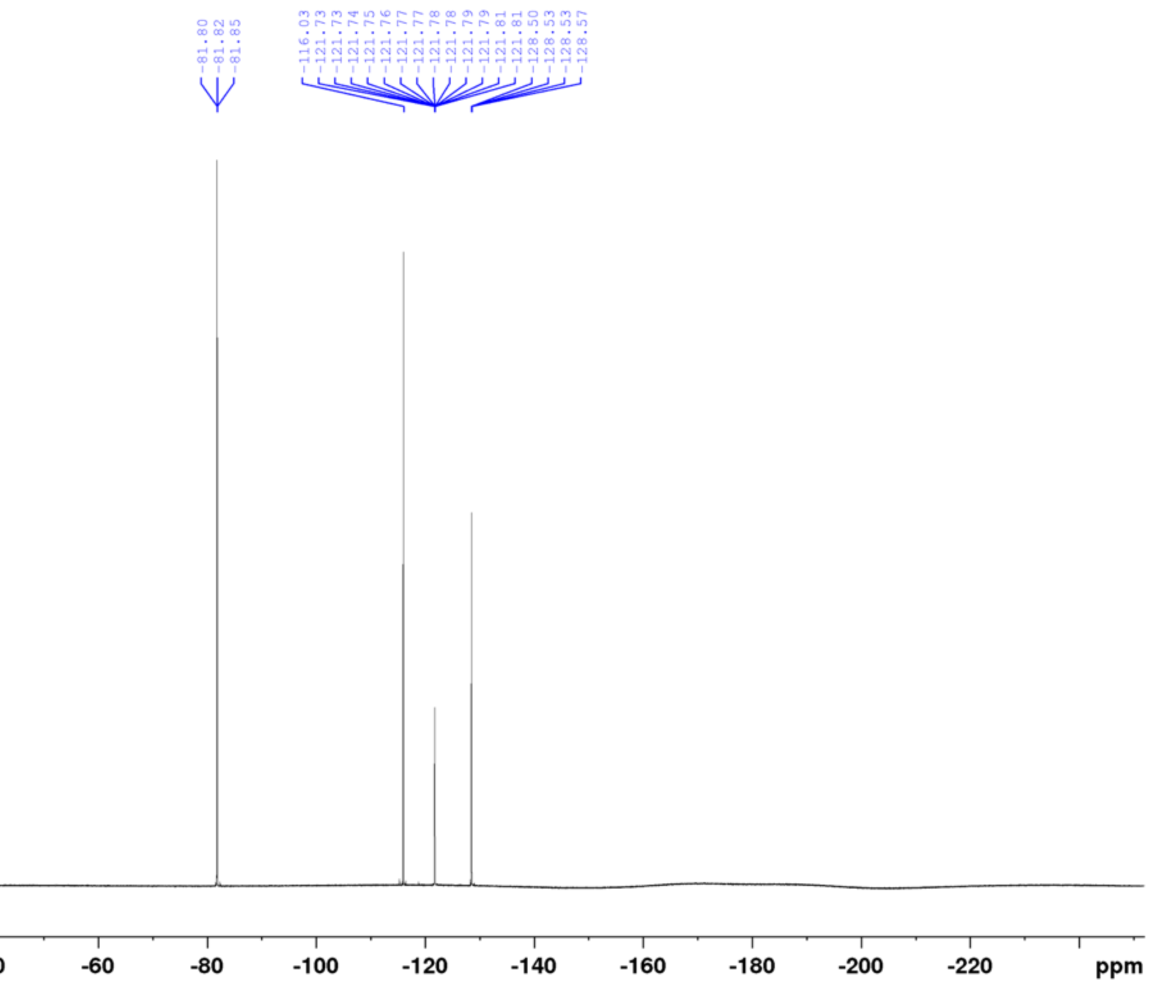

¹H NMR (500 MHz, $CDCl_3$) for *2,2,3,3,4,4,4-heptafluorobutyl (R)-(1-(2,5-dimethylphenyl)-4-hydroxybutyl)sulfamate* (**7s**)

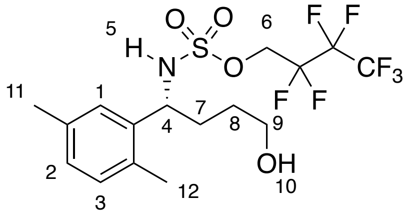

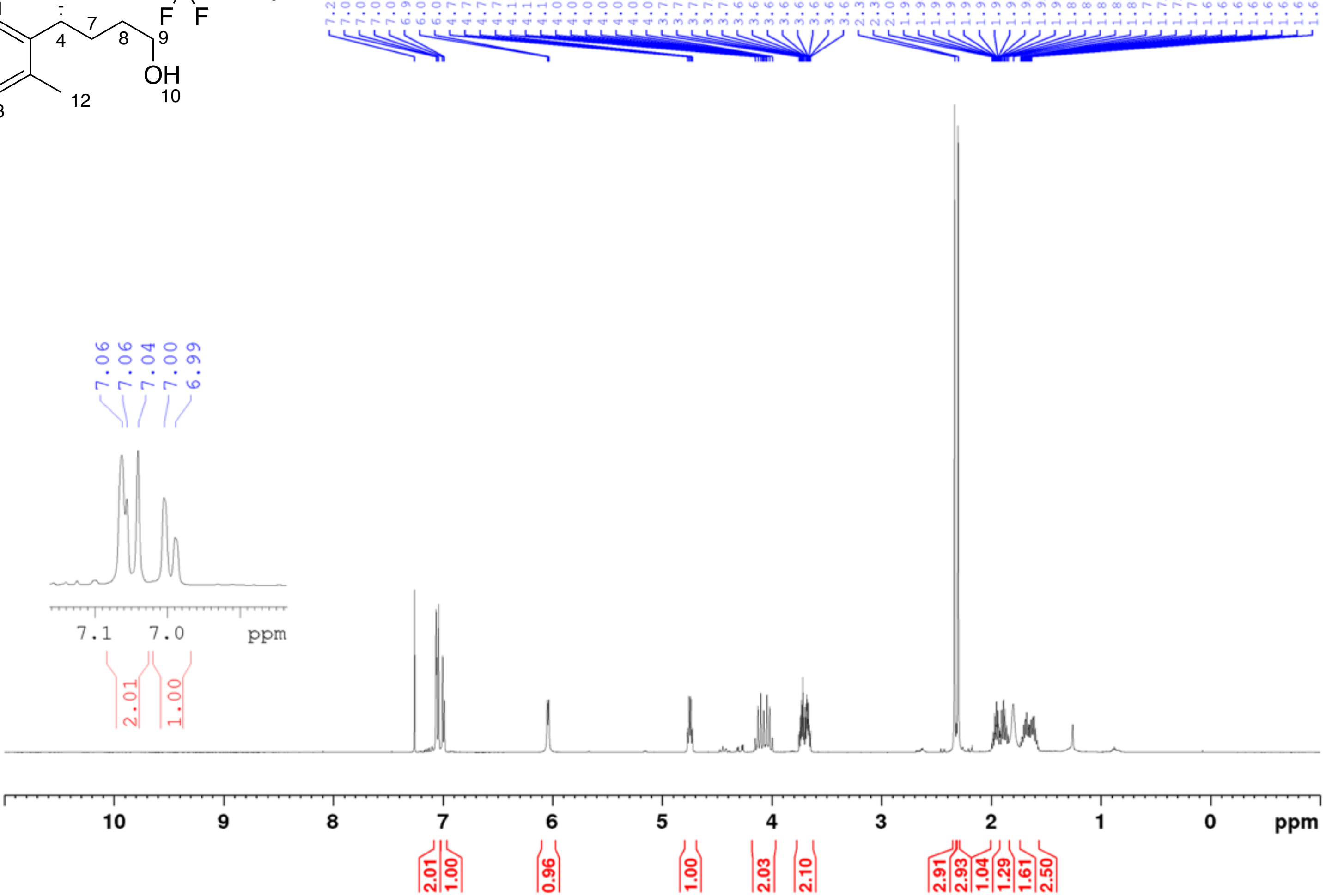

^{13}C NMR (126 MHz, CDCl₃) for *2,2,3,3,4,4,4-heptafluorobutyl (R)-(1-(2,5-dimethylphenyl)-4-hydroxybutyl)sulfamate* (**7s**)

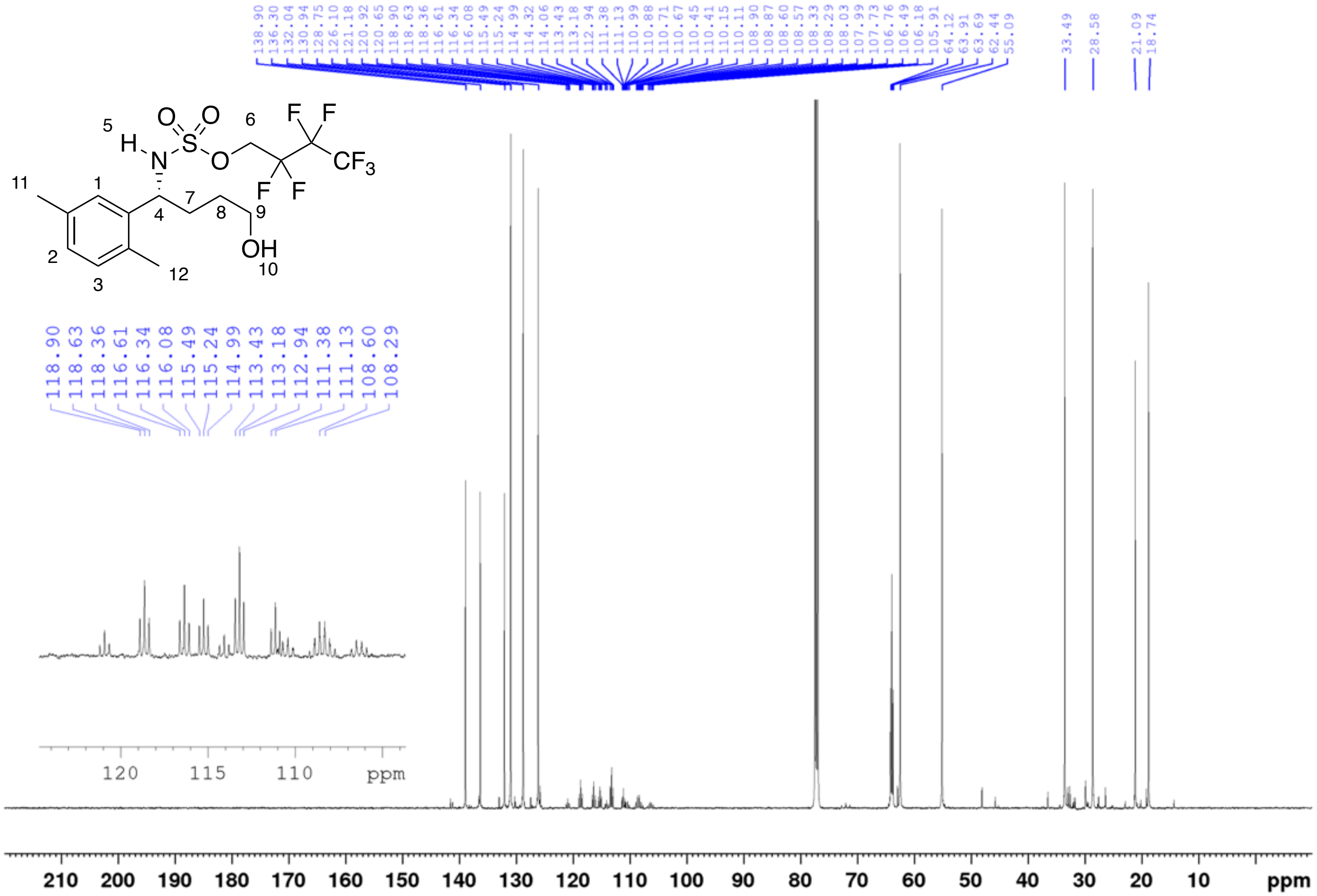

^{19}F NMR (376 MHz, $CDCl_3$) for *2,2,3,3,4,4,4-heptafluorobutyl (R)-(1-(2,5-dimethylphenyl)-4-hydroxybutyl)sulfamate* (**7s**)

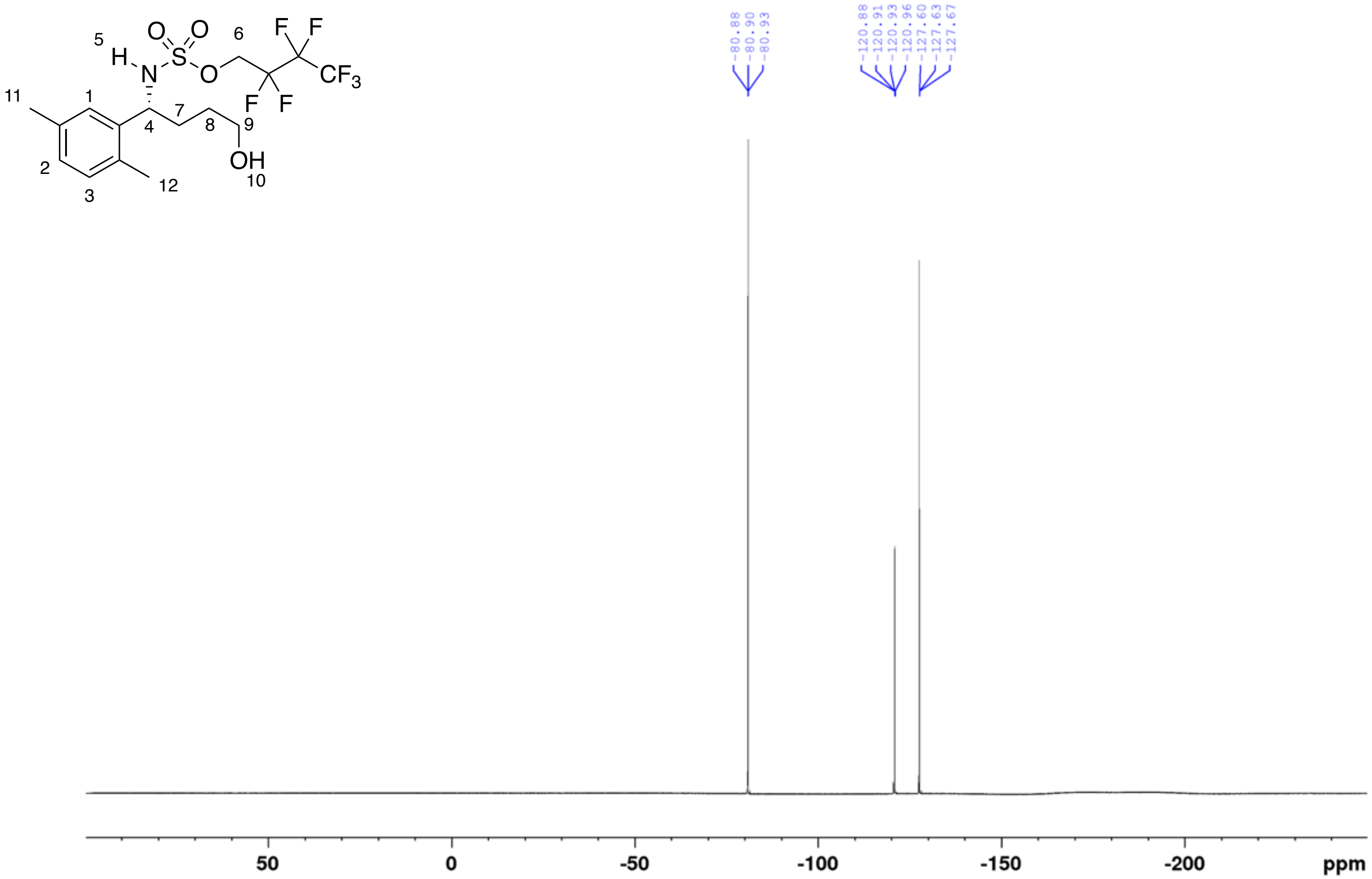

^{1}H NMR (500 MHz, $CDCl_3$) for *2,2,3,3,4,4,4-Heptafluorobutyl (1-(2,4-difluorophenyl)-4-hydroxybutyl)sulfamate* (**7t**)

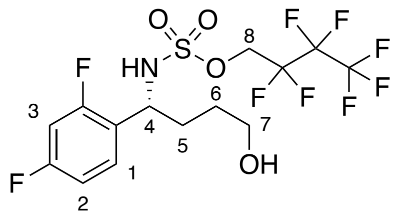

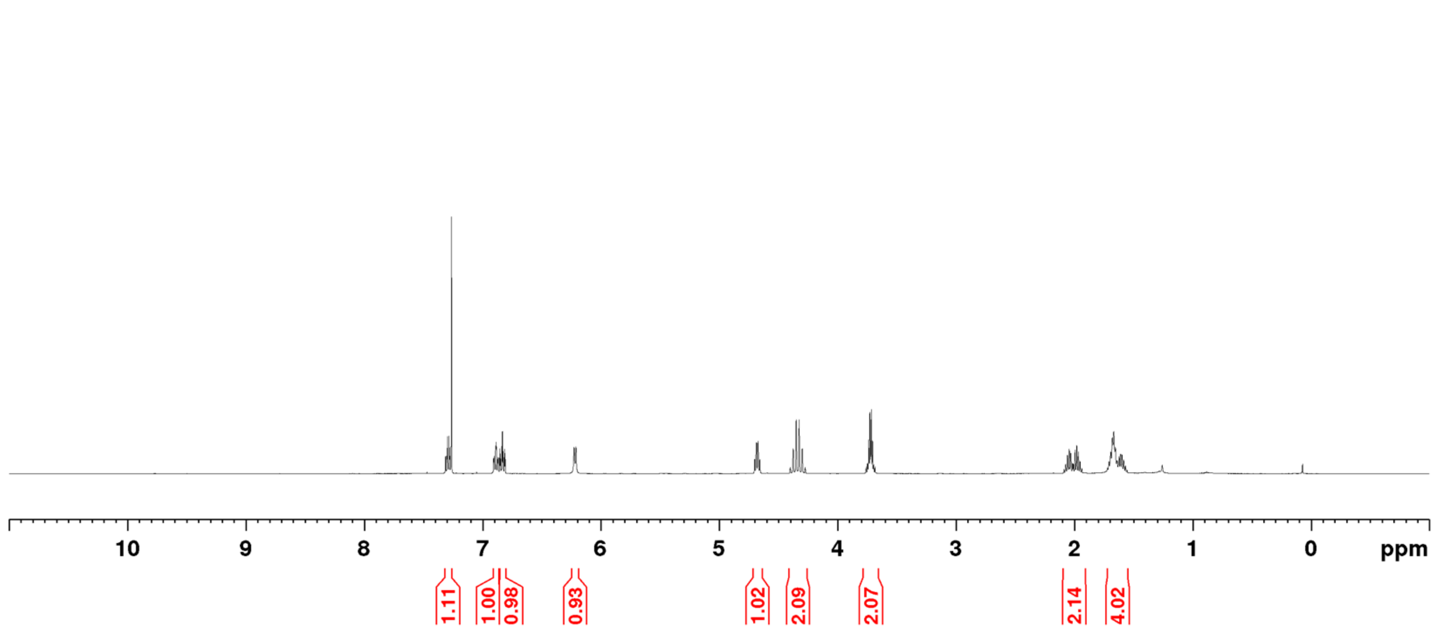

^{13}C NMR (126 MHz, $CDCl_3$) for *2,2,3,3,4,4,4-Heptafluorobutyl (1-(2,4-difluorophenyl)-4-hydroxybutyl)sulfamate* (**7t**)

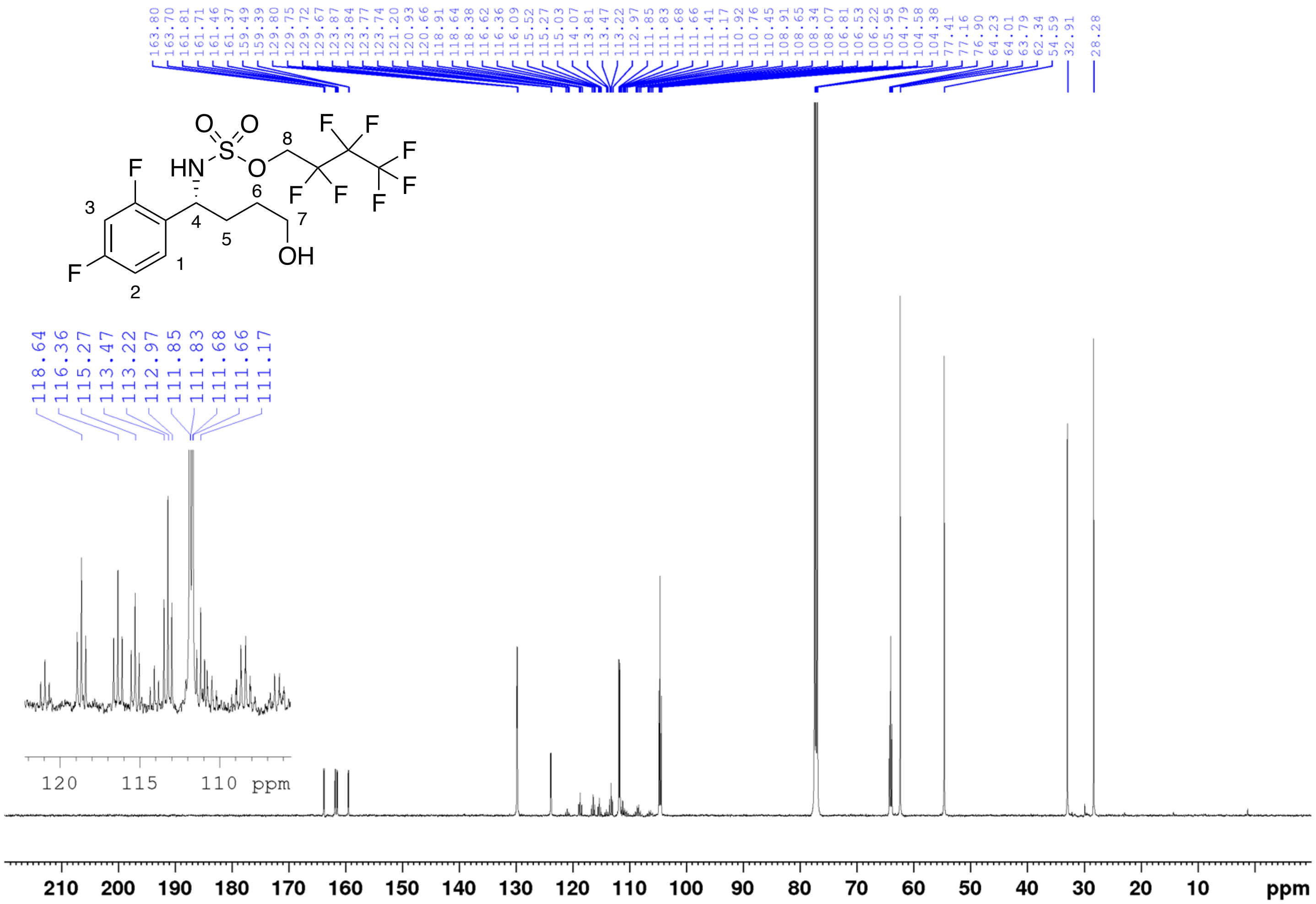

^{19}F NMR (376 MHz, $CDCl_3$) for *2,2,3,3,4,4,4-Heptafluorobutyl (1-(2,4-difluorophenyl)-4-hydroxybutyl)sulfamate* (**7t**)

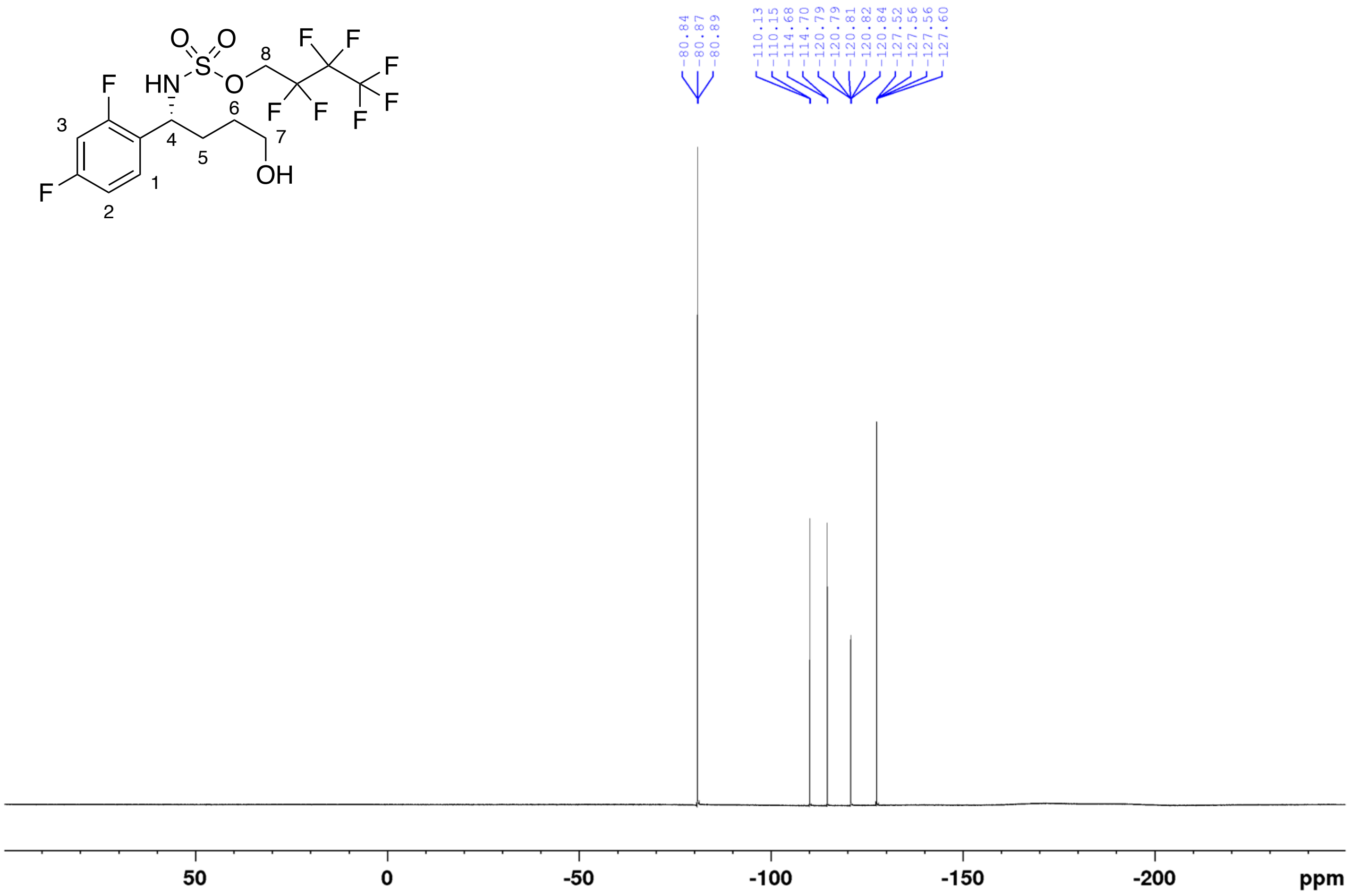

¹H NMR (500 MHz, $CDCl_3$) for *2,2,3,3,4,4,4-heptafluorobutyl (R)-(1-(3-bromo-2-methylphenyl)-4-hydroxybutyl)sulfamate* (**7u**)

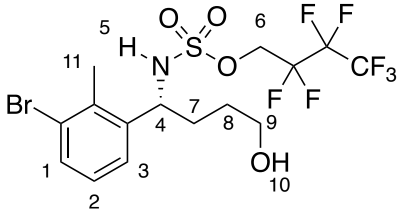

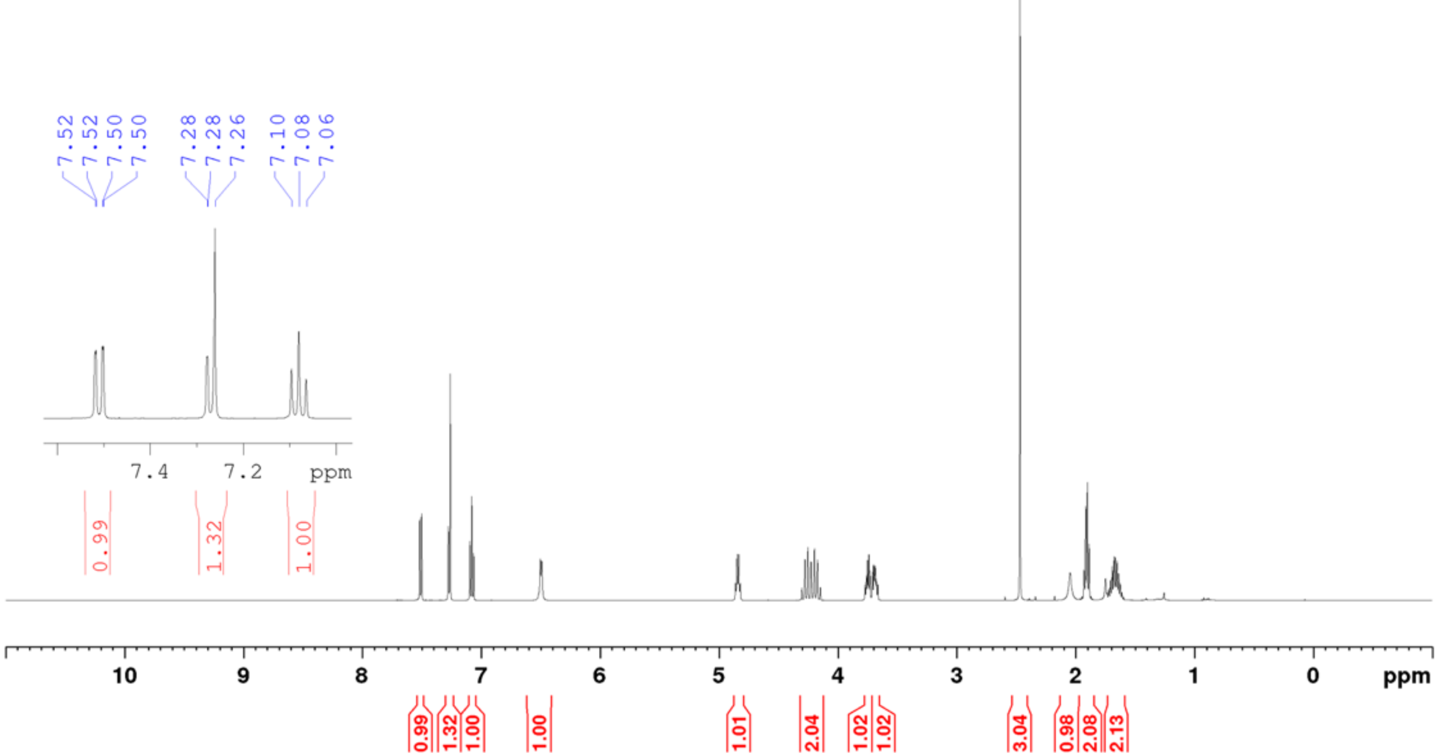

^{13}C NMR (126 MHz, $CDCl_3$) for *2,2,3,3,4,4,4-heptafluorobutyl (R)-(1-(3-bromo-2-methylphenyl)-4-hydroxybutyl)sulfamate* (**7u**)

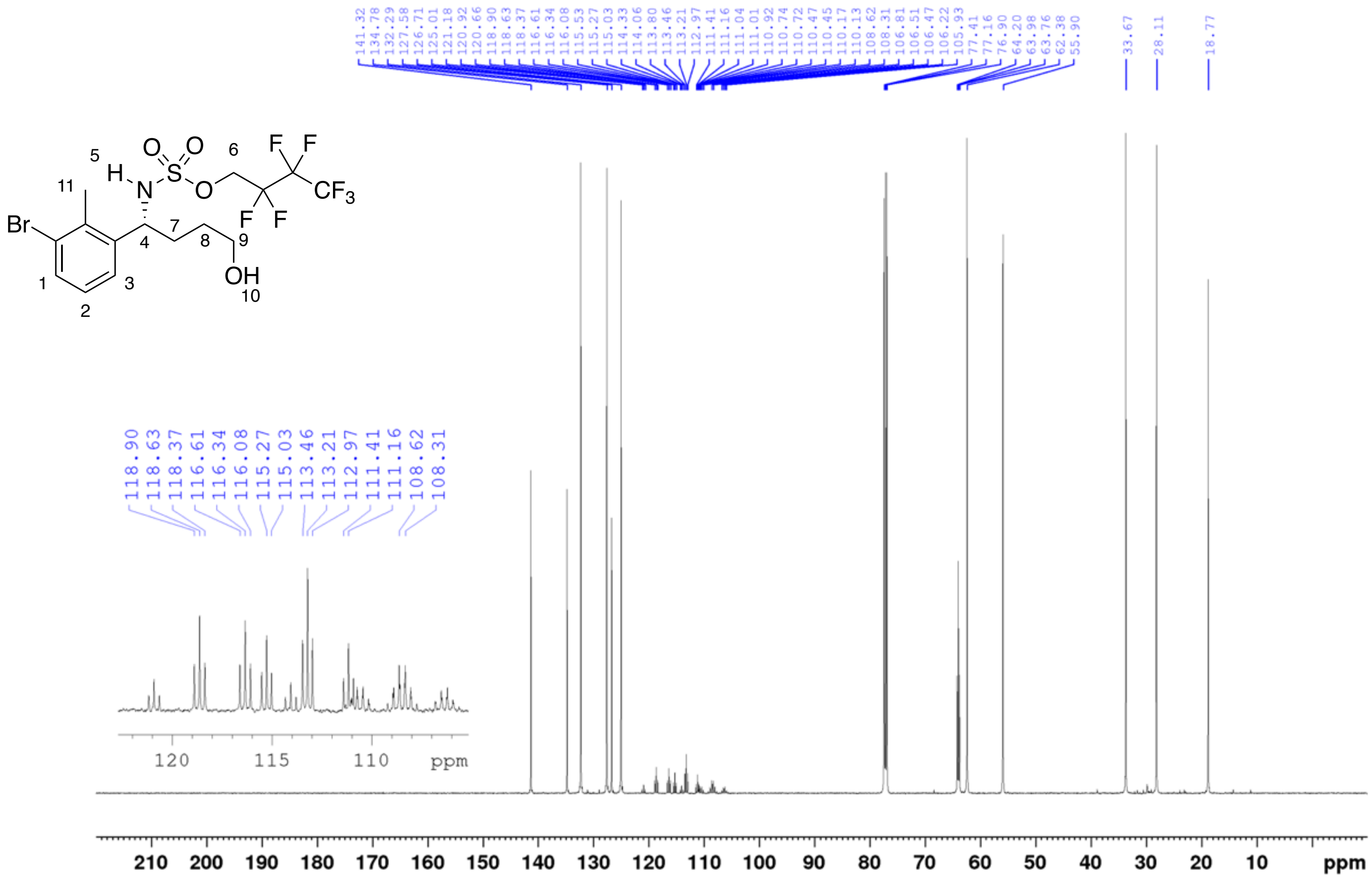

^{19}F NMR (376 MHz, $CDCl_3$) for *2,2,3,3,4,4,4-heptafluorobutyl (R)-(1-(3-bromo-2-methylphenyl)-4-hydroxybutyl)sulfamate* (**7u**)

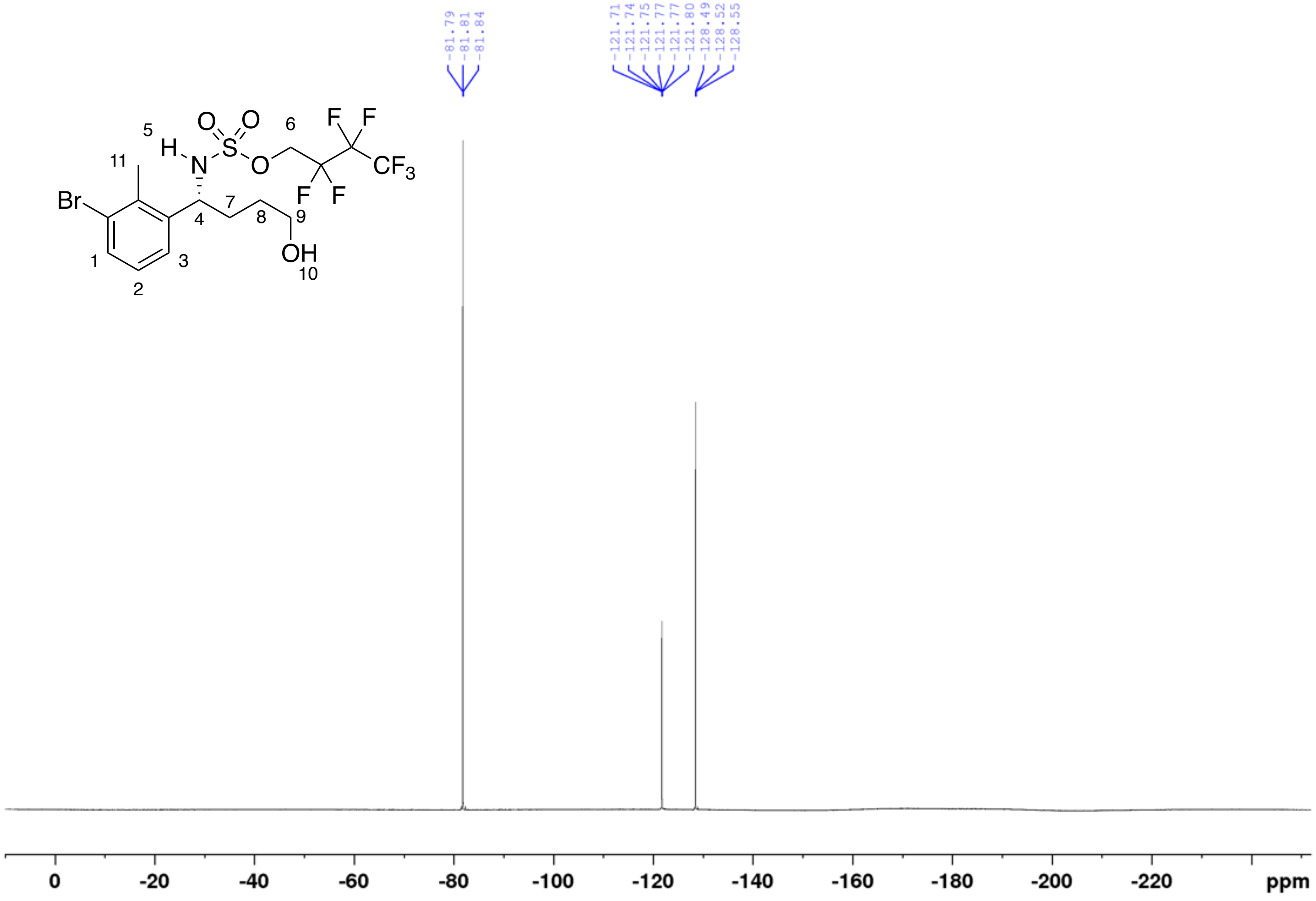

^{1}H NMR (500 MHz, $CDCl_3$) for *2,2,3,3,4,4,4-heptafluorobutyl (R)-(1-(3-chloro-2-methylphenyl)-4-hydroxybutyl)sulfamate* (**7v**)

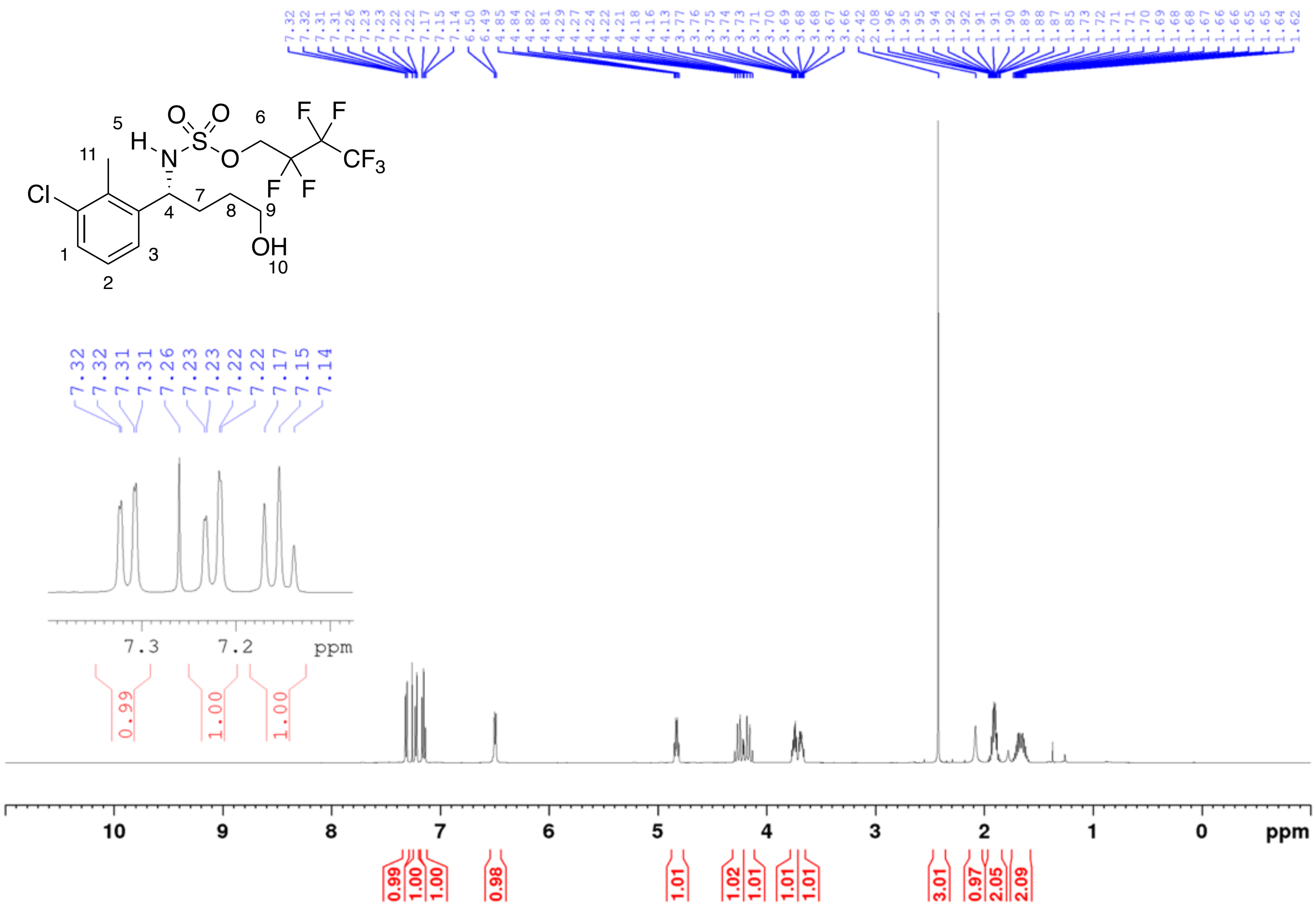

^{13}C NMR (126 MHz, $CDCl_3$) for *2,2,3,3,4,4,4-heptafluorobutyl (R)-(1-(3-chloro-2-methylphenyl)-4-hydroxybutyl)sulfamate* (**7v**)

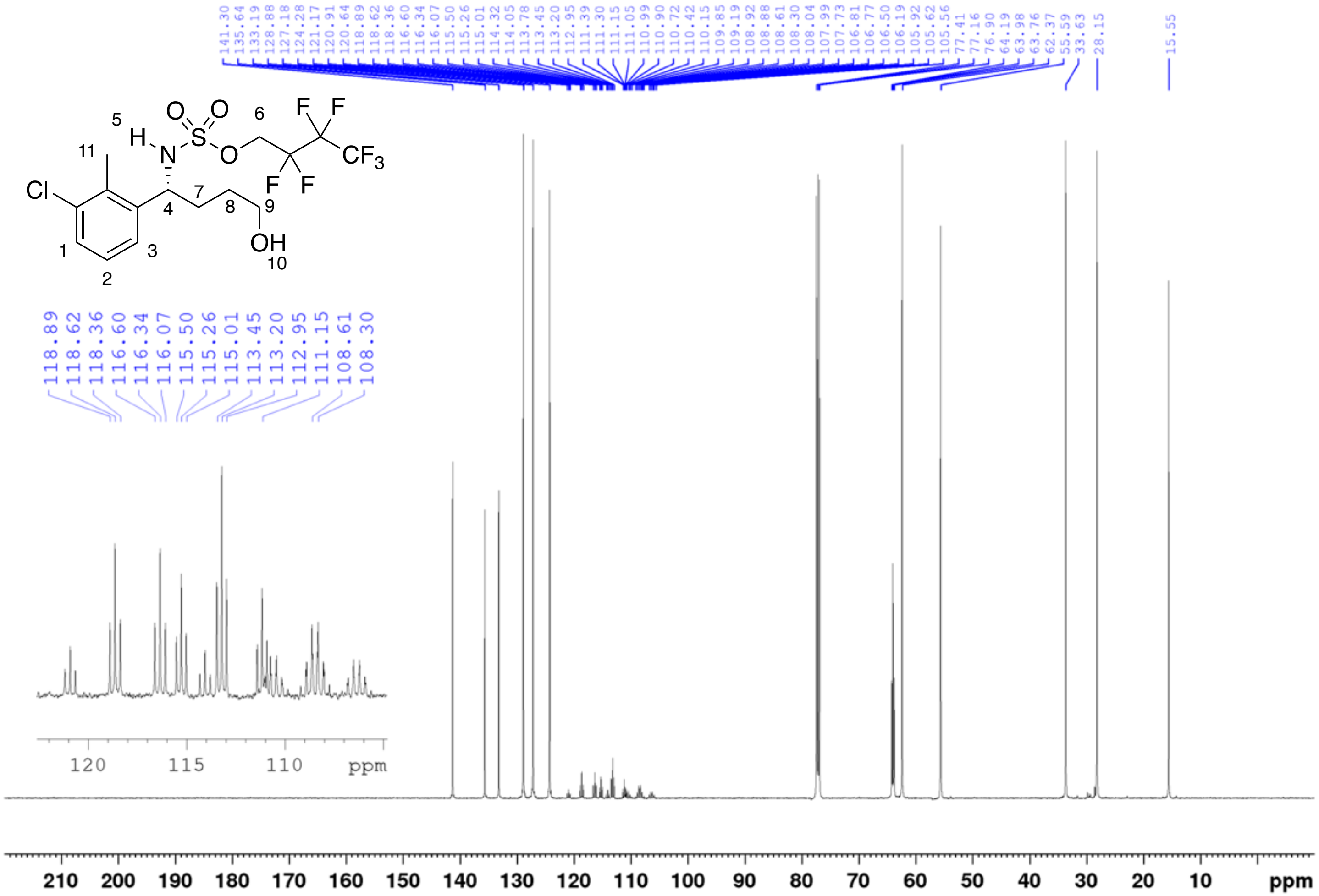

19F NMR (376 MHz, $CDCl_3$) for *2,2,3,3,4,4,4-heptafluorobutyl (R)-(1-(3-chloro-2-methylphenyl)-4-hydroxybutyl)sulfamate* (**7v**)

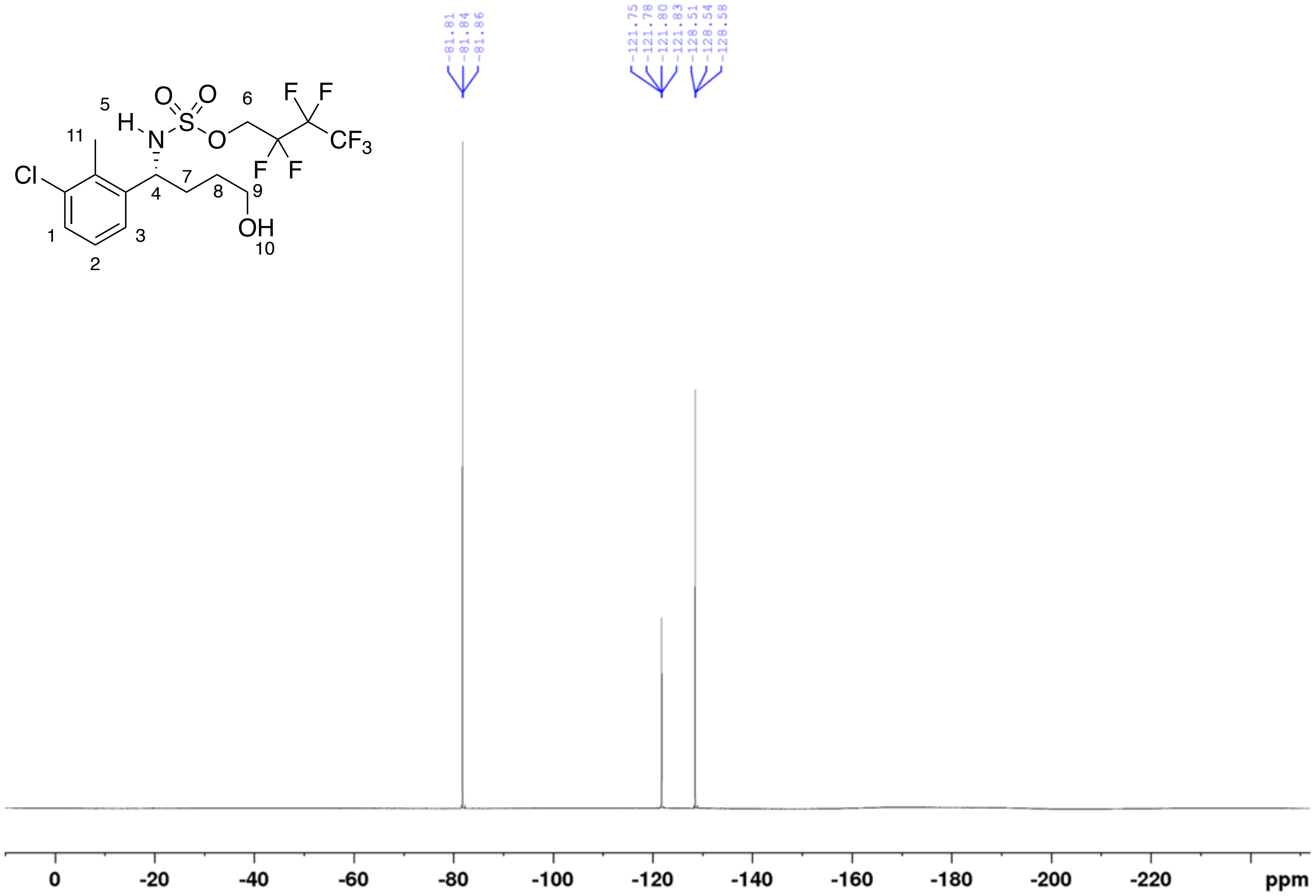

¹H NMR (500 MHz, $CDCl_3$) for *2,2,3,3,4,4,4-heptafluorobutyl (R)-(4-hydroxy-1-(naphthalen-1-yl)butyl)sulfamate* (**7w**)

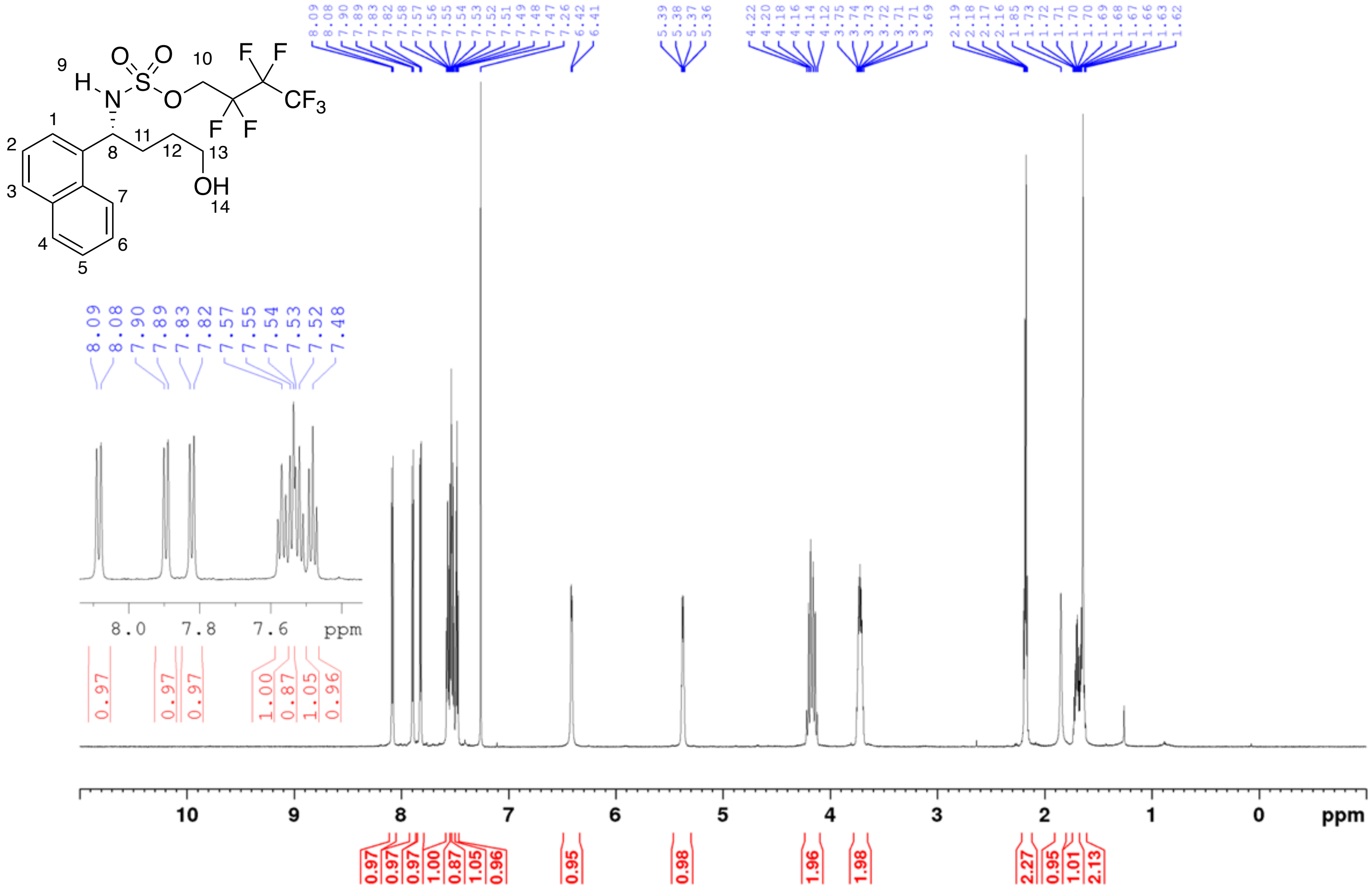

^{13}C NMR (126 MHz, $CDCl_3$) for *2,2,3,3,4,4,4-heptafluorobutyl (R)-(4-hydroxy-1-(naphthalen-1-yl)butyl)sulfamate* (**7w**)

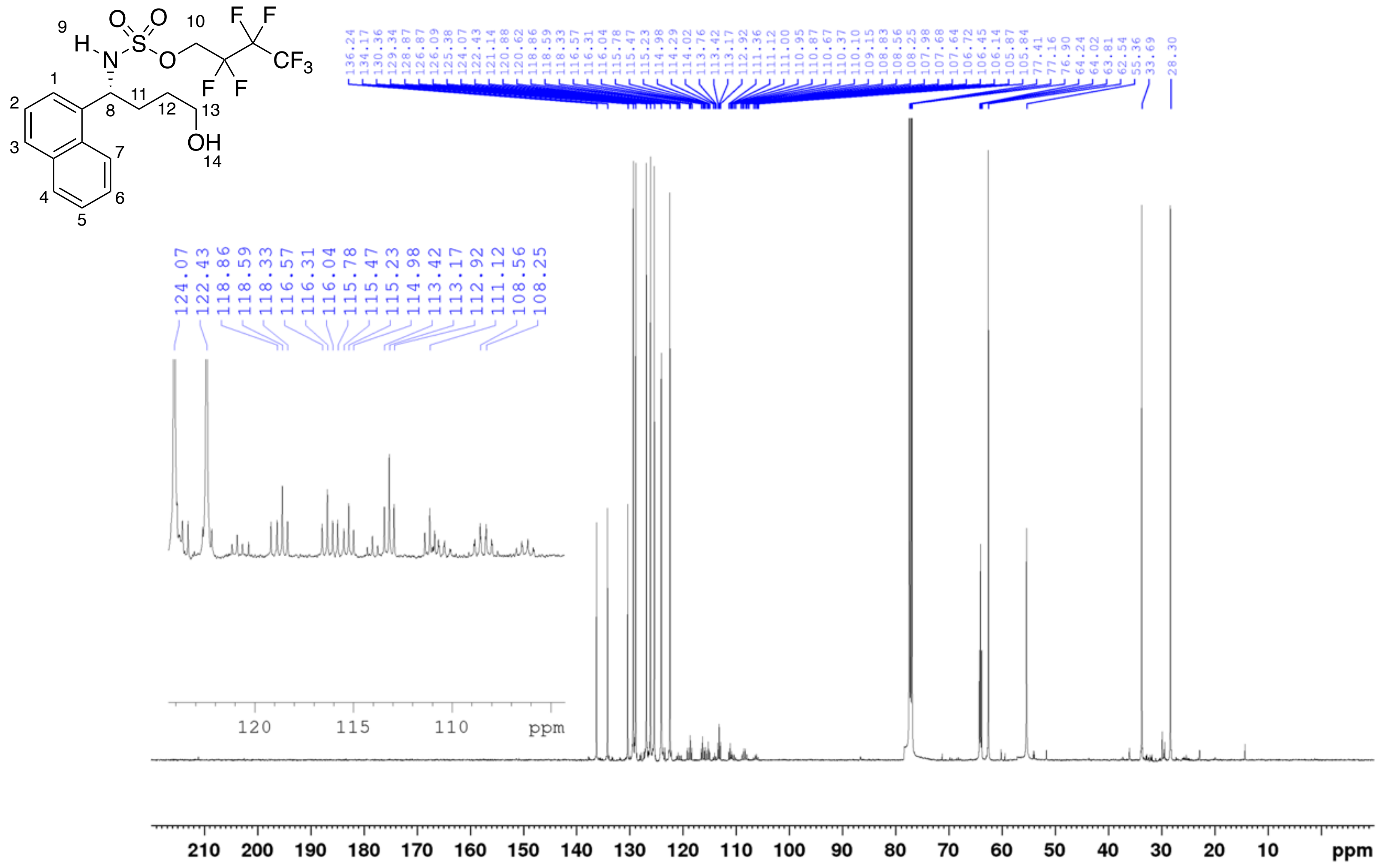

^{19}F NMR (376 MHz, $CDCl_3$) for *2,2,3,3,4,4,4-heptafluorobutyl (R)-(4-hydroxy-1-(naphthalen-1-yl)butyl)sulfamate* (**7w**)

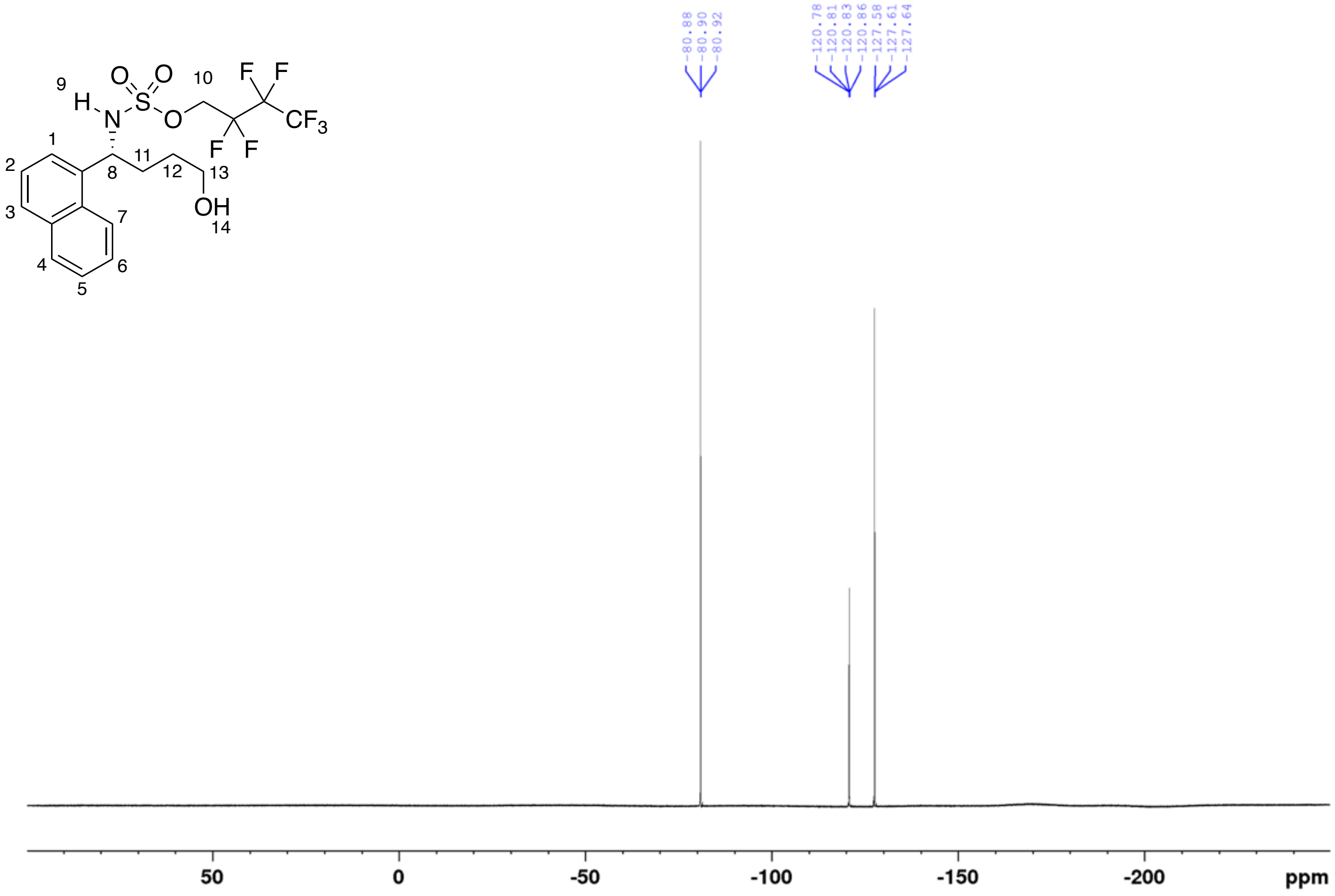

^{1}H NMR (500 MHz, $CDCl_3$) for *2,2,3,3,4,4,4-heptafluorobutyl (R)-(4-hydroxy-1-(3-methylthiophen-2-yl)butyl)sulfamate* (**7x**)

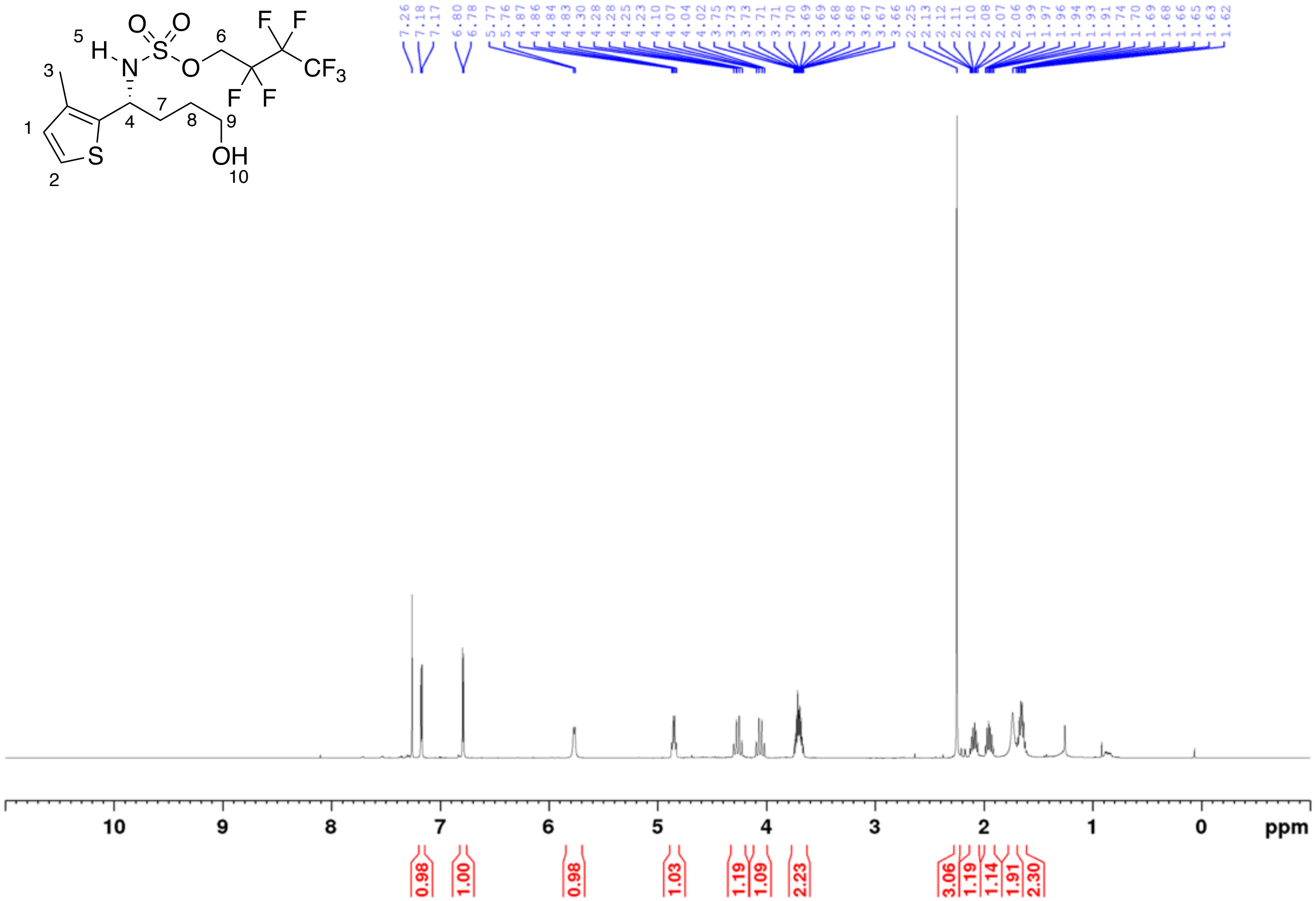

^{13}C NMR (126 MHz, $CDCl_3$) for *2,2,3,3,4,4,4-heptafluorobutyl (R)-(4-hydroxy-1-(3-methylthiophen-2-yl)butyl)sulfamate* (**7x**)

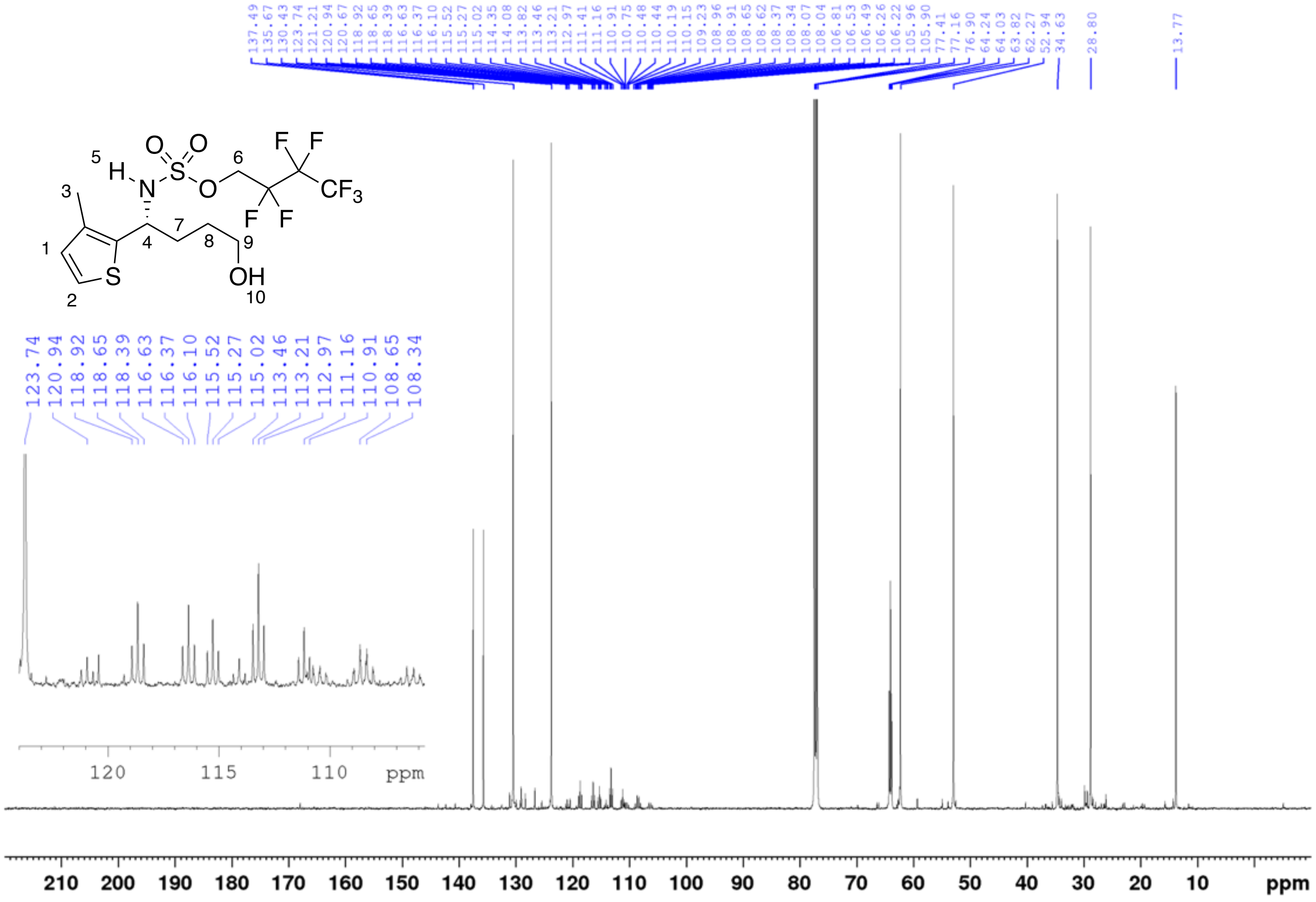

^{19}F NMR (376 MHz, $CDCl_3$) for *2,2,3,3,4,4,4-heptafluorobutyl (R)-(4-hydroxy-1-(3-methylthiophen-2-yl)butyl)sulfamate* (**7x**)

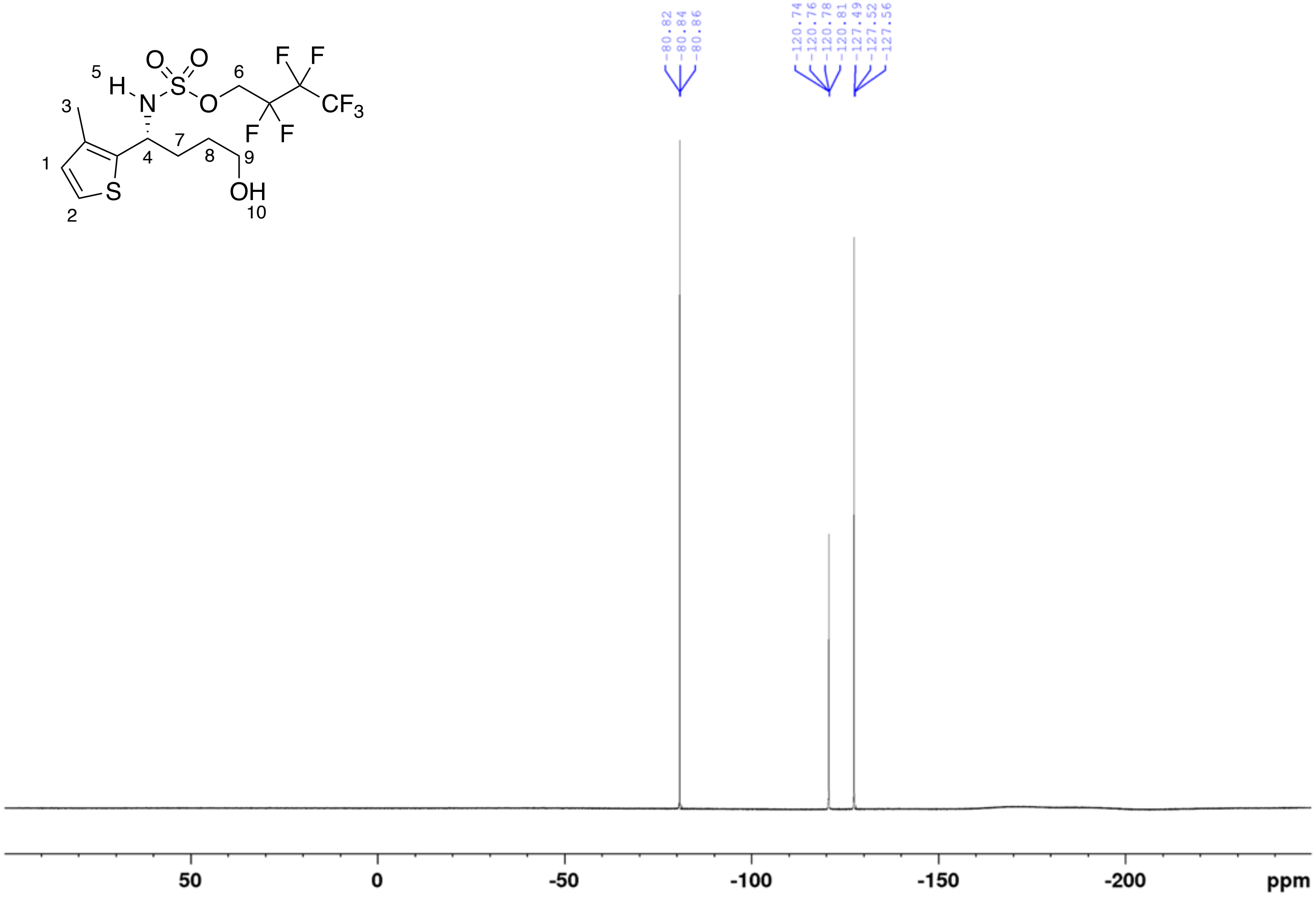

¹H NMR (400 MHz, $CDCl_3$) for *2,2,3,3,4,4,4-heptafluorobutyl (R)-2-phenylpyrrolidine-1-sulfonate* (**8**)

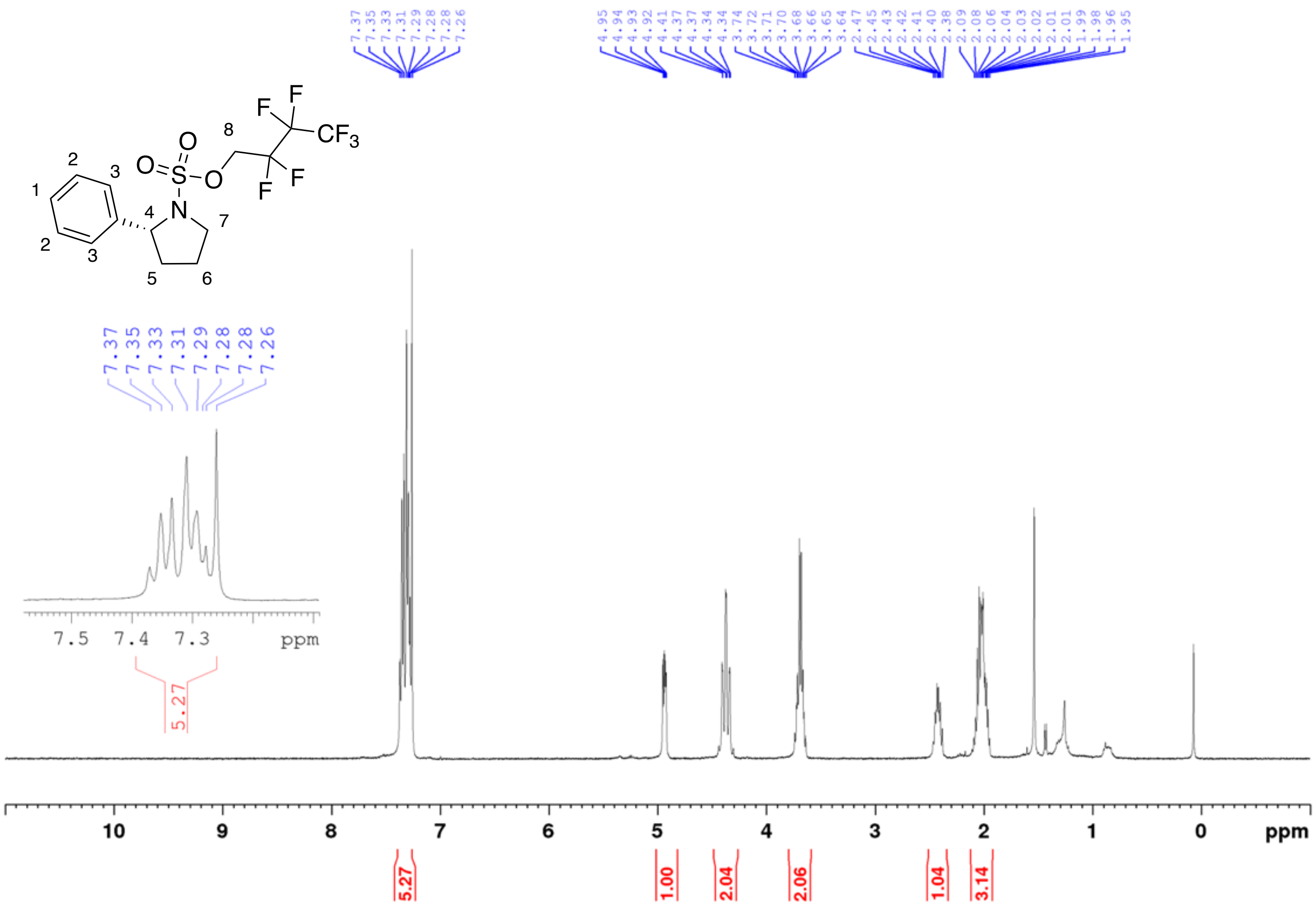

^{13}C NMR (126 MHz, $CDCl_3$) for *2,2,3,3,4,4,4-heptafluorobutyl (R)-2-phenylpyrrolidine-1-sulfonate* (**8**)

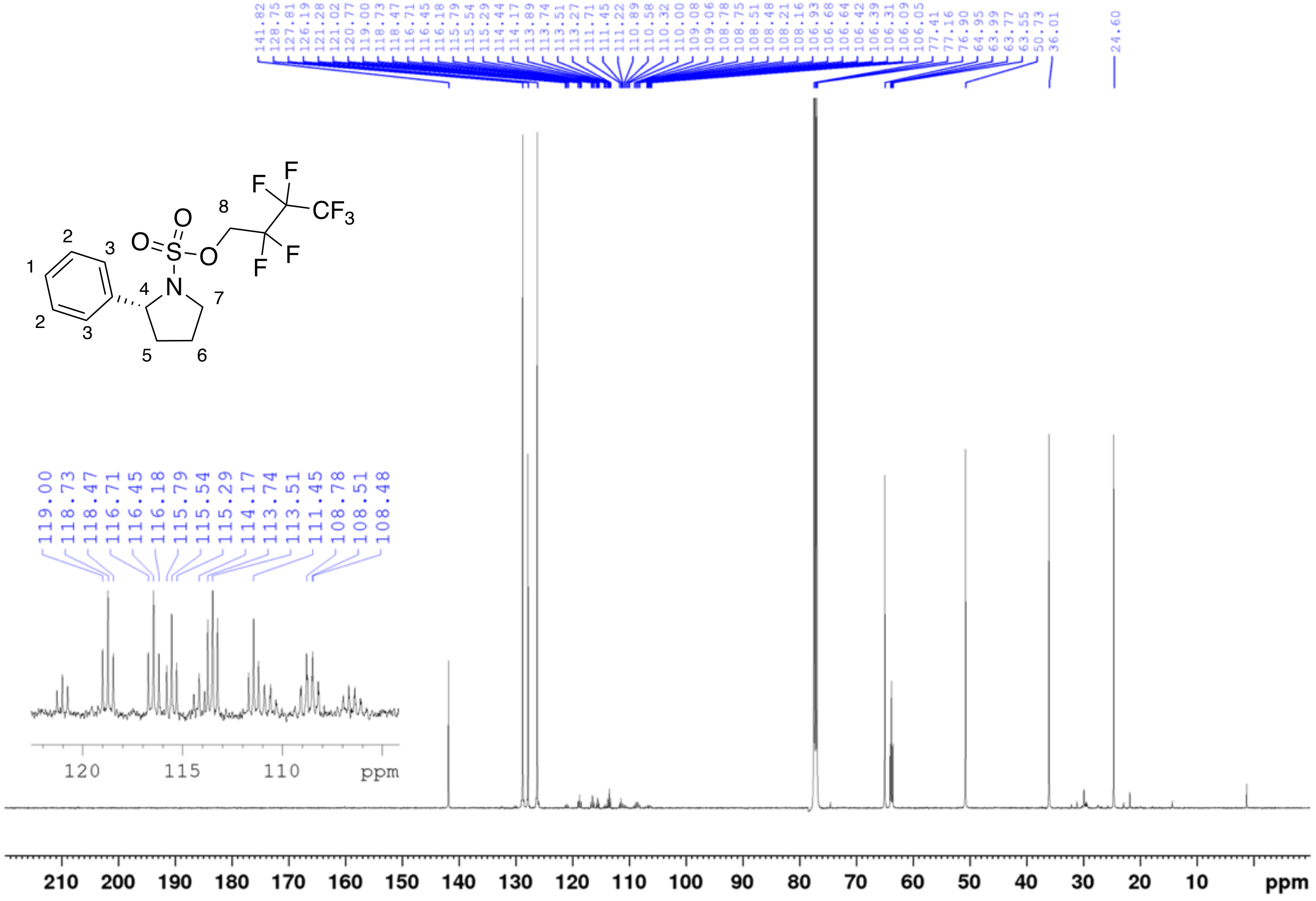

^{19}F NMR (376 MHz, $CDCl_3$) for *2,2,3,3,4,4,4-heptafluorobutyl (R)-2-phenylpyrrolidine-1-sulfonate* (**8**)

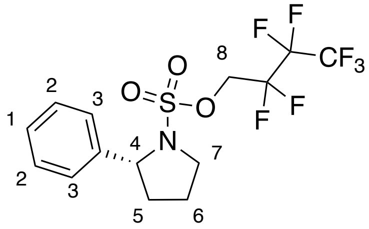

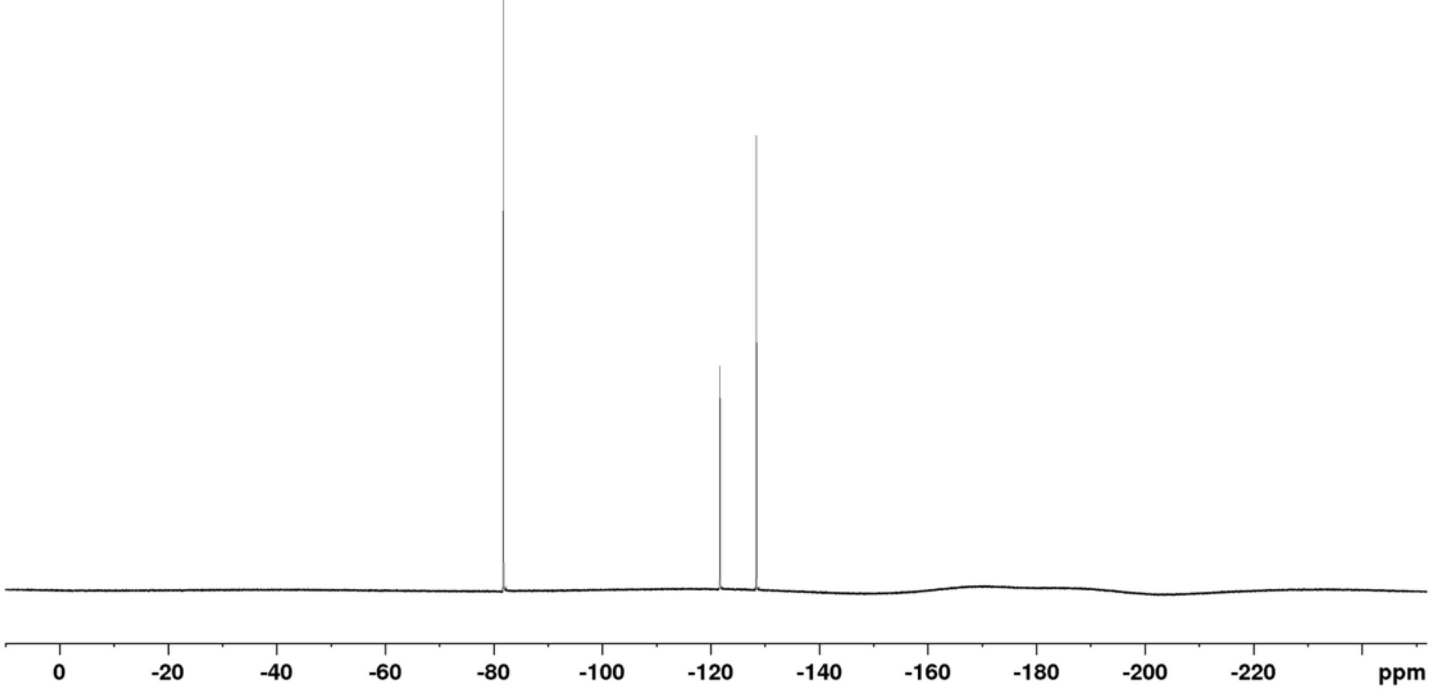

¹H NMR (400 MHz, $CDCl_3$) for *(R)-2-phenylpyrrolidine* (**9**)

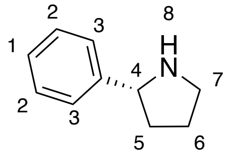

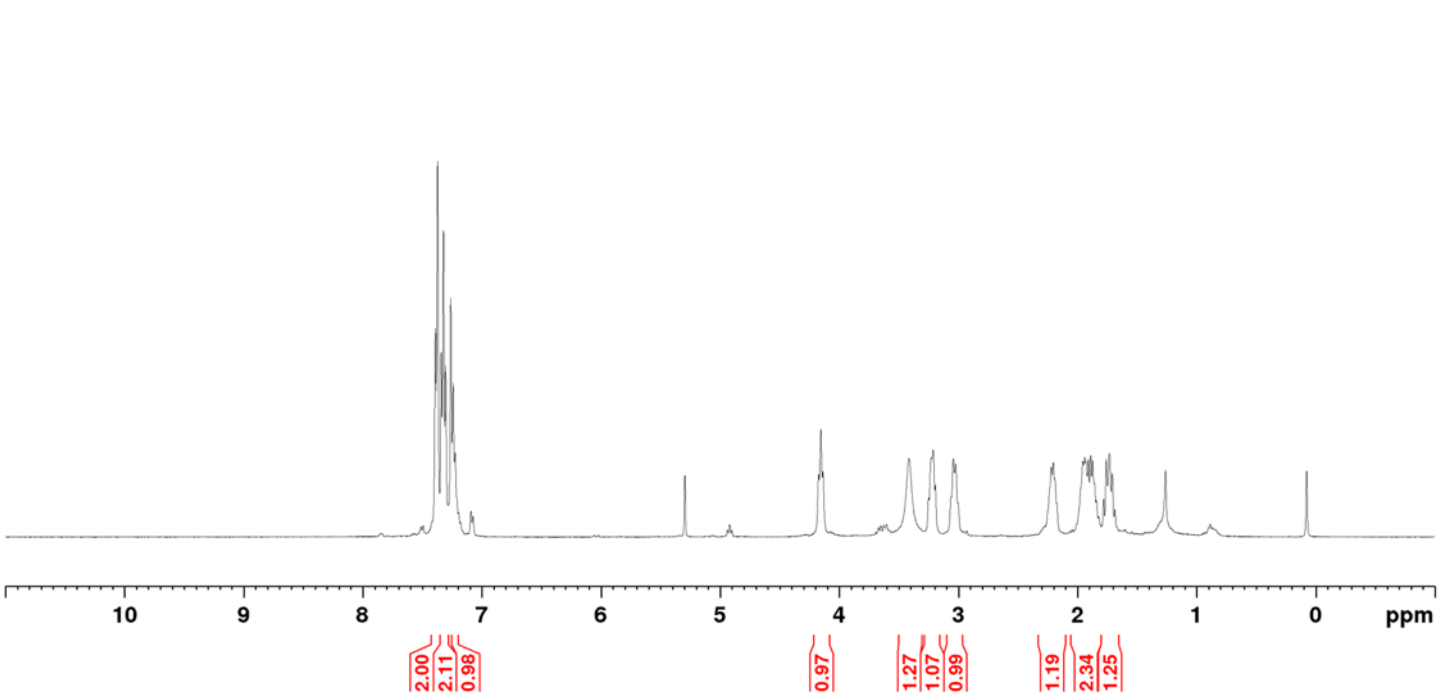

^{13}**C NMR** (101 MHz, $CDCl_3$) for *(R)-2-phenylpyrrolidine* (**9**)

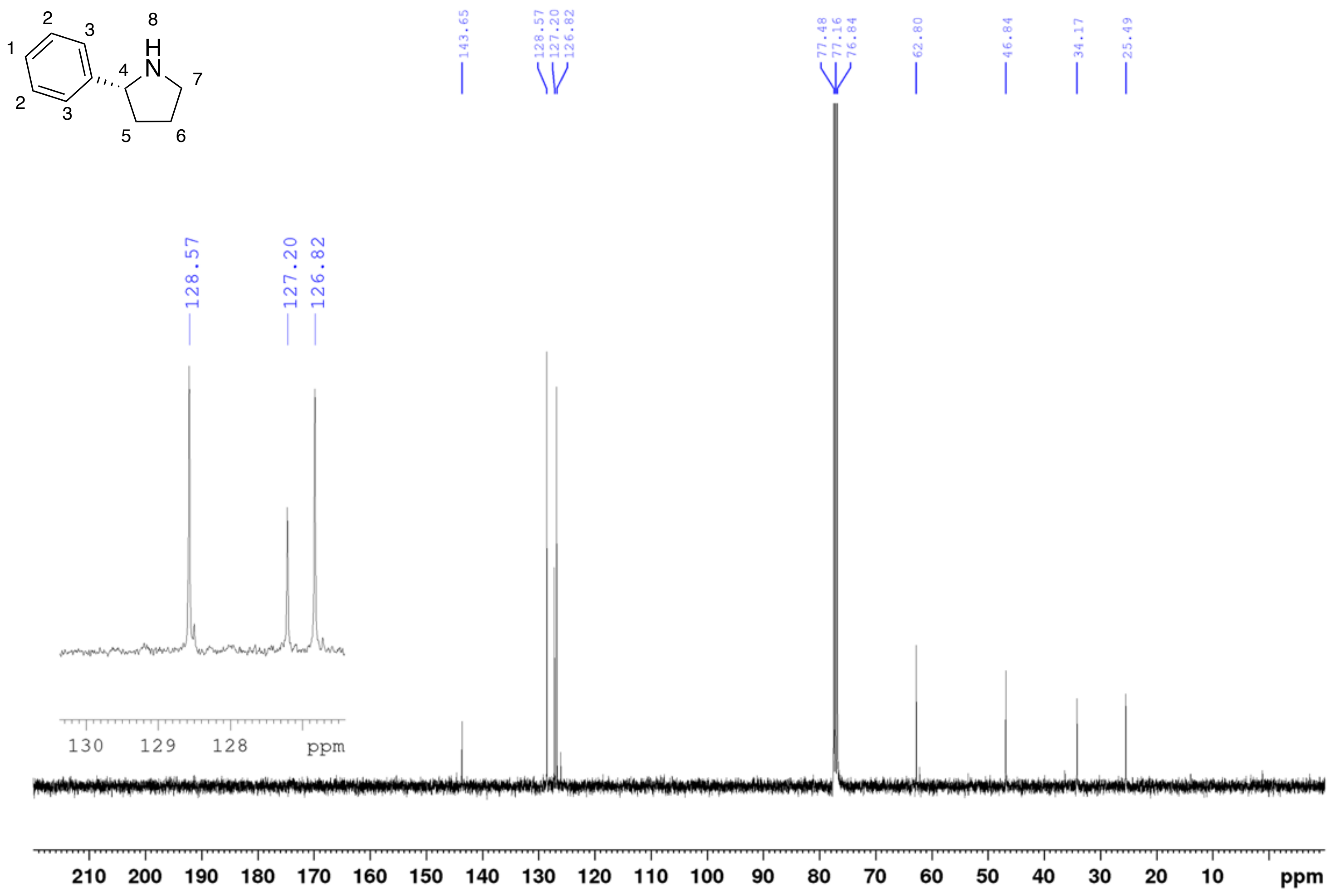

^{1}H NMR (500 MHz, $CDCl_3$) for *2,2,3,3,4,4,4-heptafluorobutyl (R)-(1-phenylbutyl)sulfamate* (**11**)

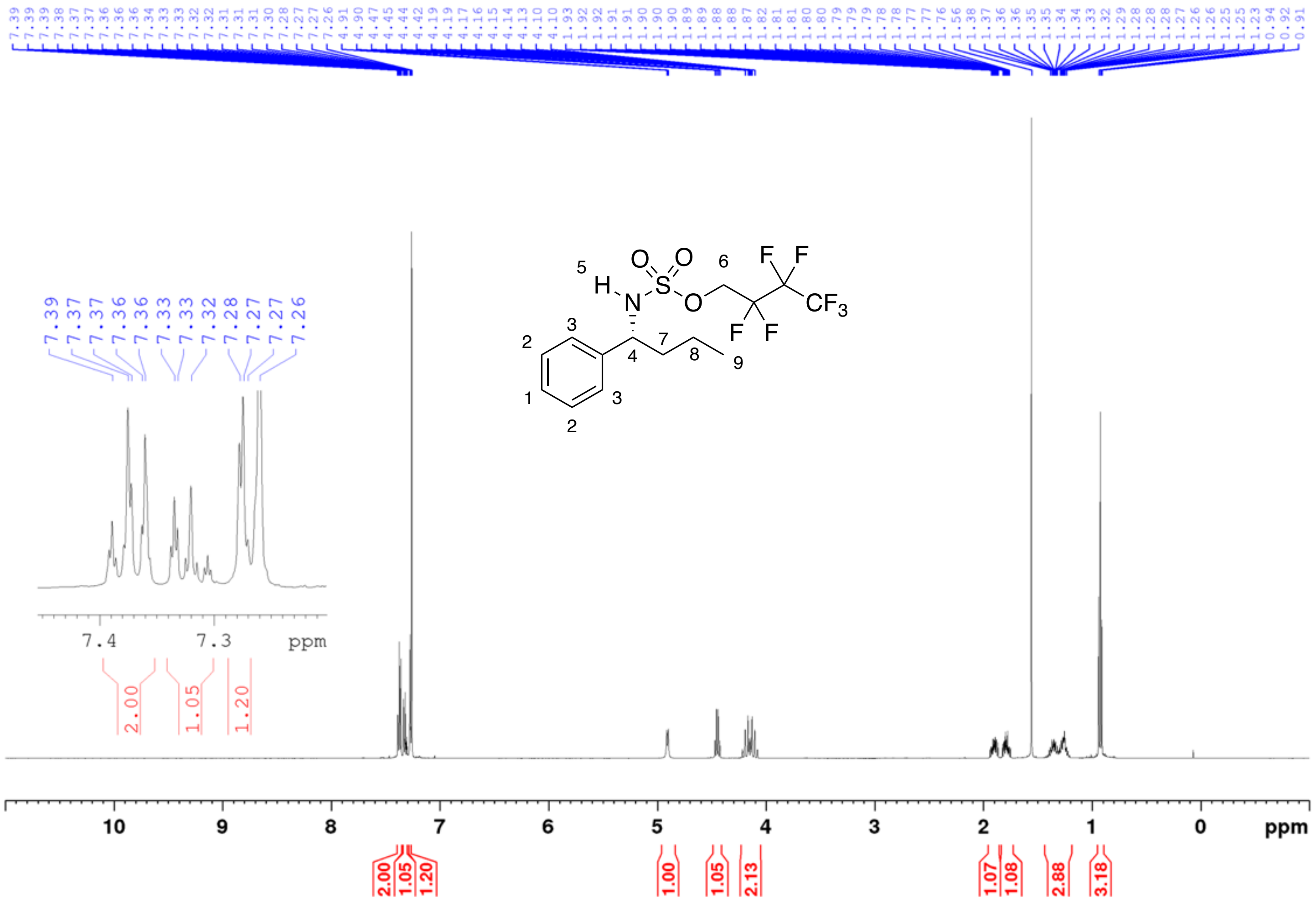

^{13}C NMR (126 MHz, $CDCl_3$) for *2,2,3,3,4,4,4-heptafluorobutyl (R)-(1-phenylbutyl)sulfamate* (**11**)

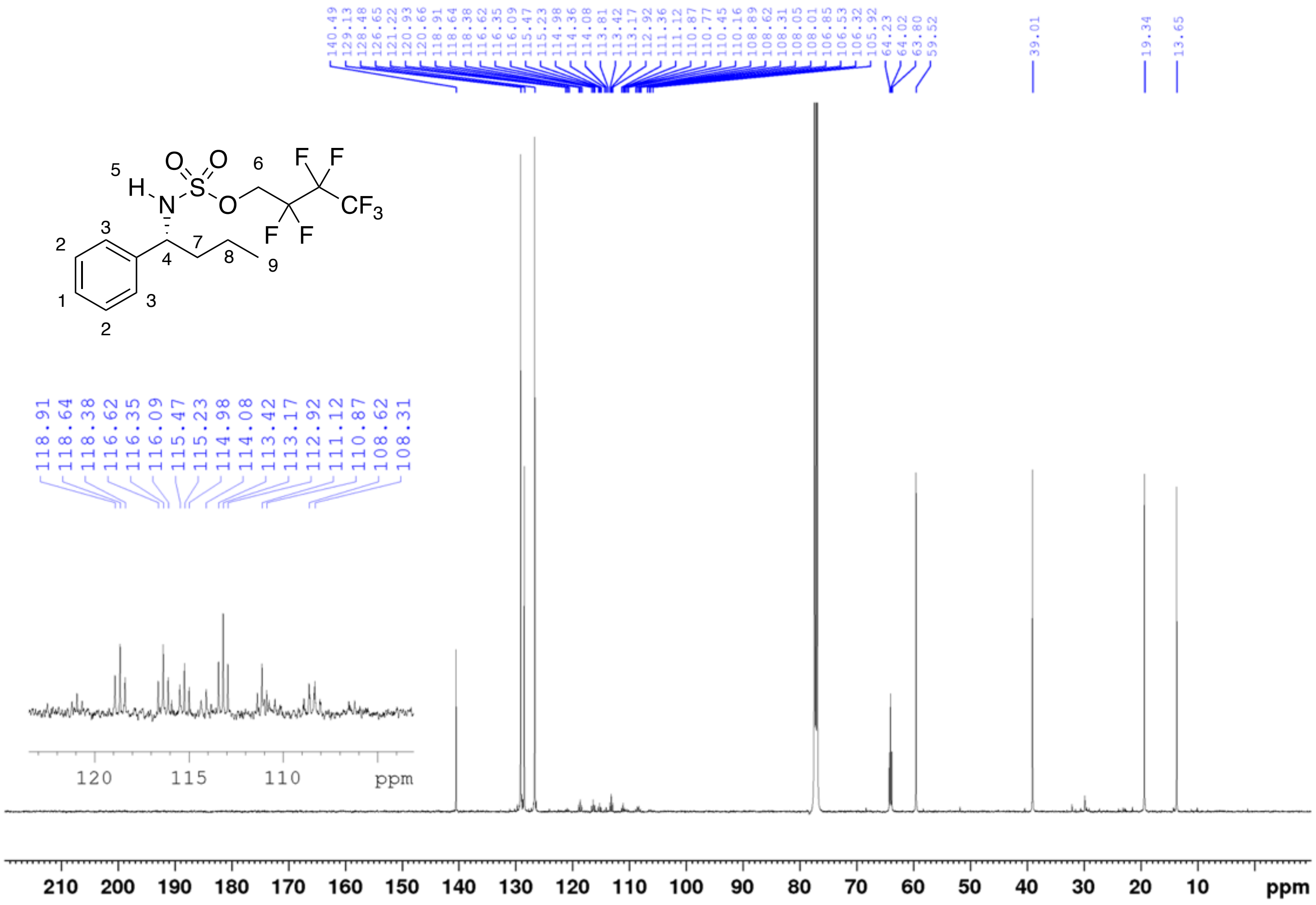

¹⁹F NMR (376 MHz, $CDCl_3$) for *2,2,3,3,4,4,4-heptafluorobutyl (R)-(1-phenylbutyl)sulfamate* (**11**)

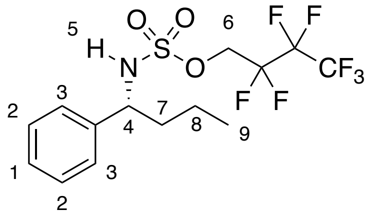

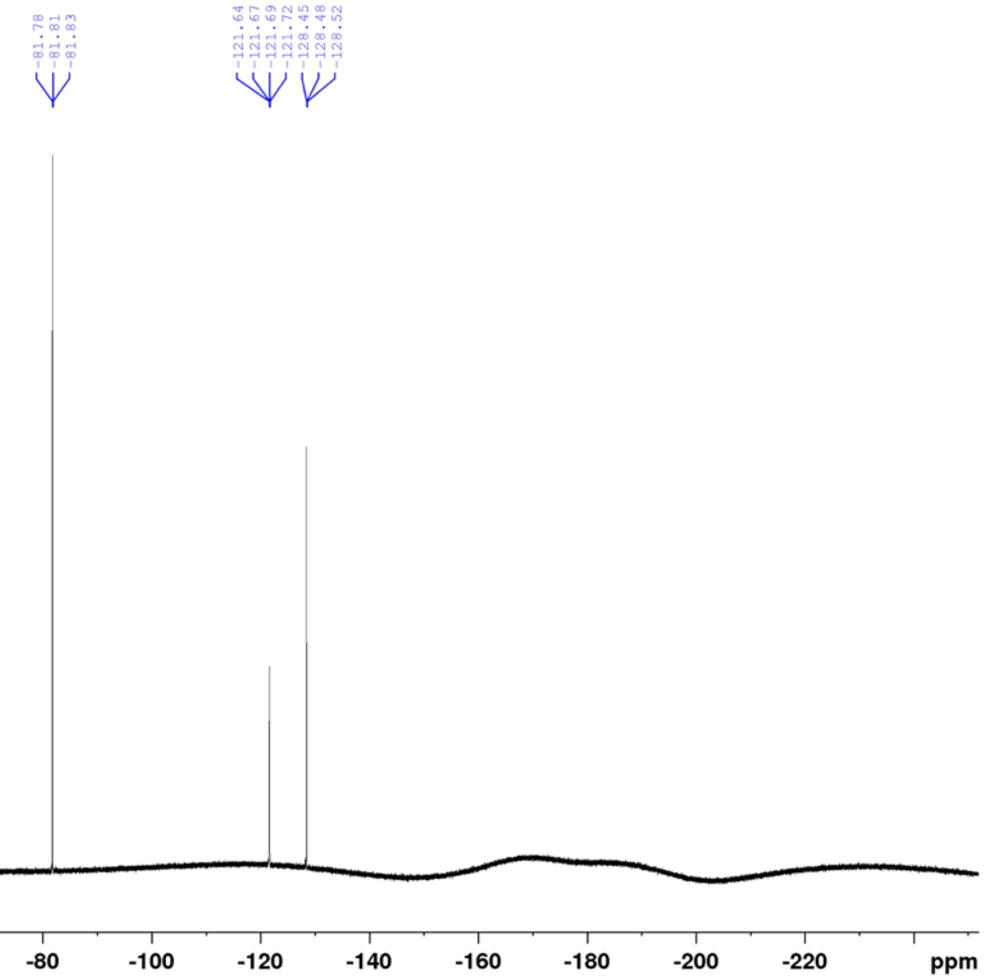

^{1}**H NMR** (500 MHz, $CDCl_3$) for *methyl (R)-4-(((2,2,3,3,4,4,4-heptafluorobutoxy)sulfonyl)amino)-4-phenylbutanoate* (**13**)

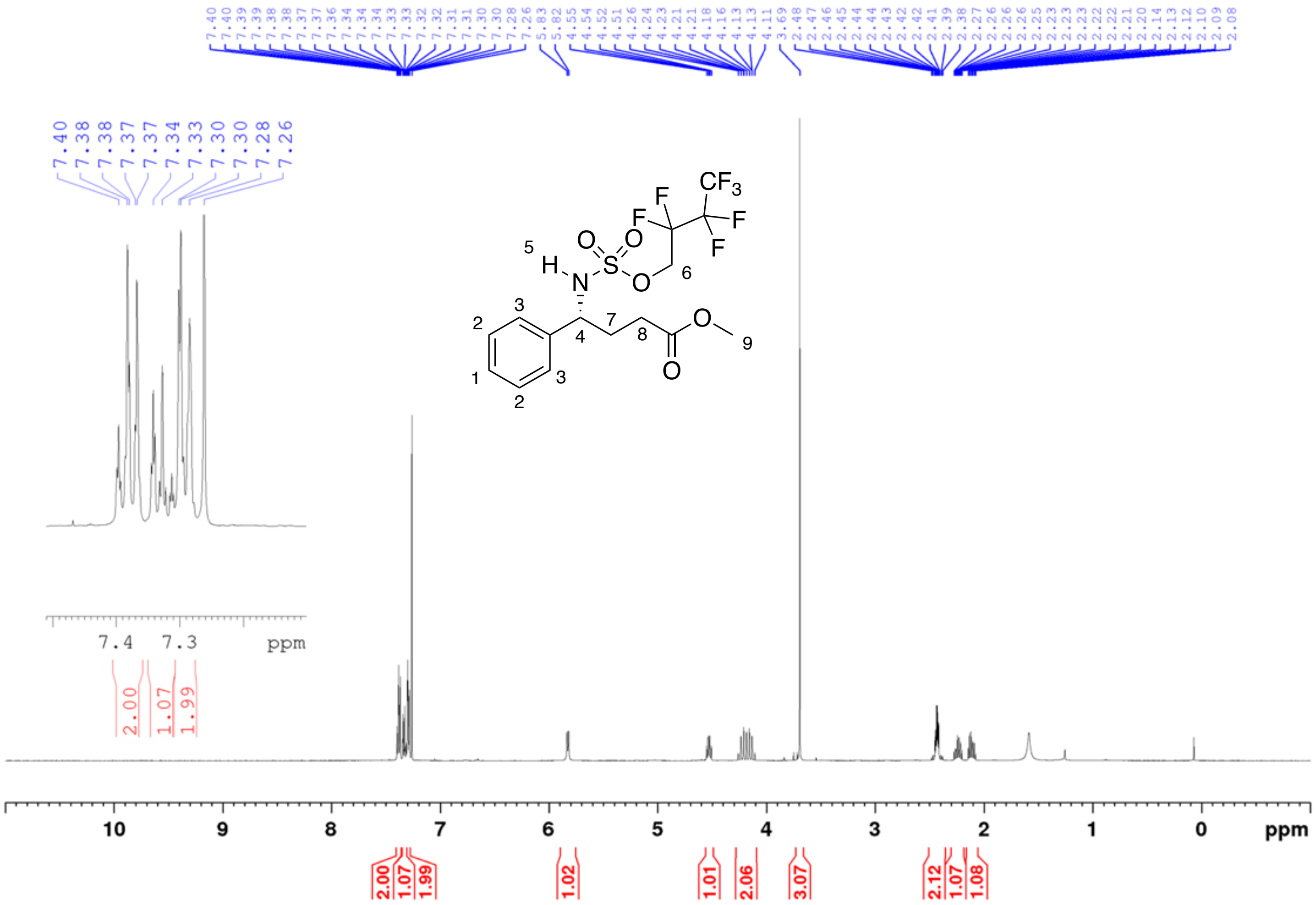

^{13}C NMR (126 MHz, $CDCl_3$) for *methyl (R)-4-(((2,2,3,3,4,4,4-heptafluorobutoxy)sulfonyl)amino)-4-phenylbutanoate* (**13**)

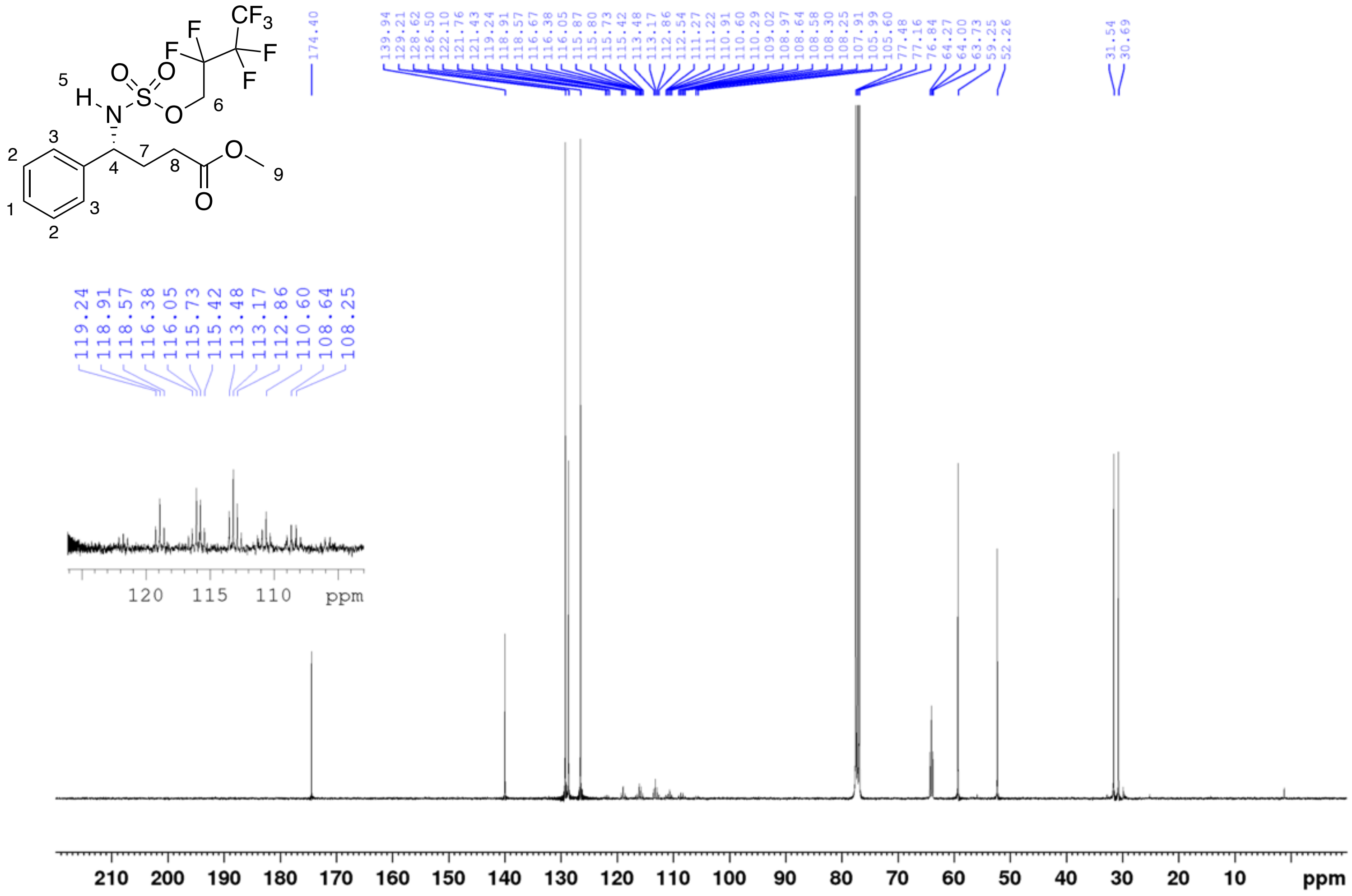

^{19}F NMR (376 MHz, $CDCl_3$) for *methyl (R)-4-(((2,2,3,3,4,4,4-heptafluorobutoxy)sulfonyl)amino)-4-phenylbutanoate* (**13**)

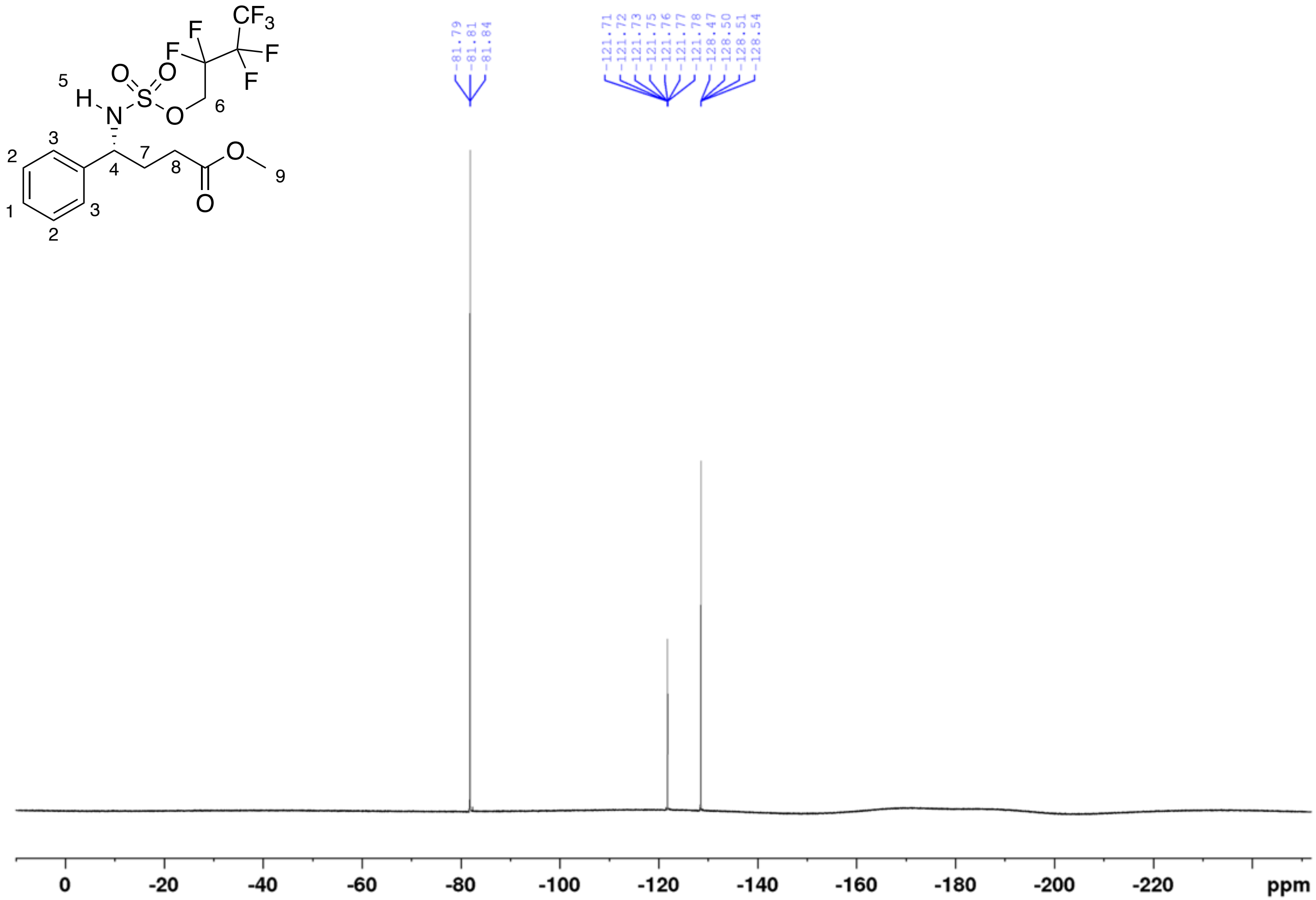

^{1}H NMR (400 MHz, $CDCl_3$) for *2,2,3,3,4,4,4-heptafluorobutyl (R)-(3-hydroxy-1-phenylpropyl)sulfamate* (**14**)

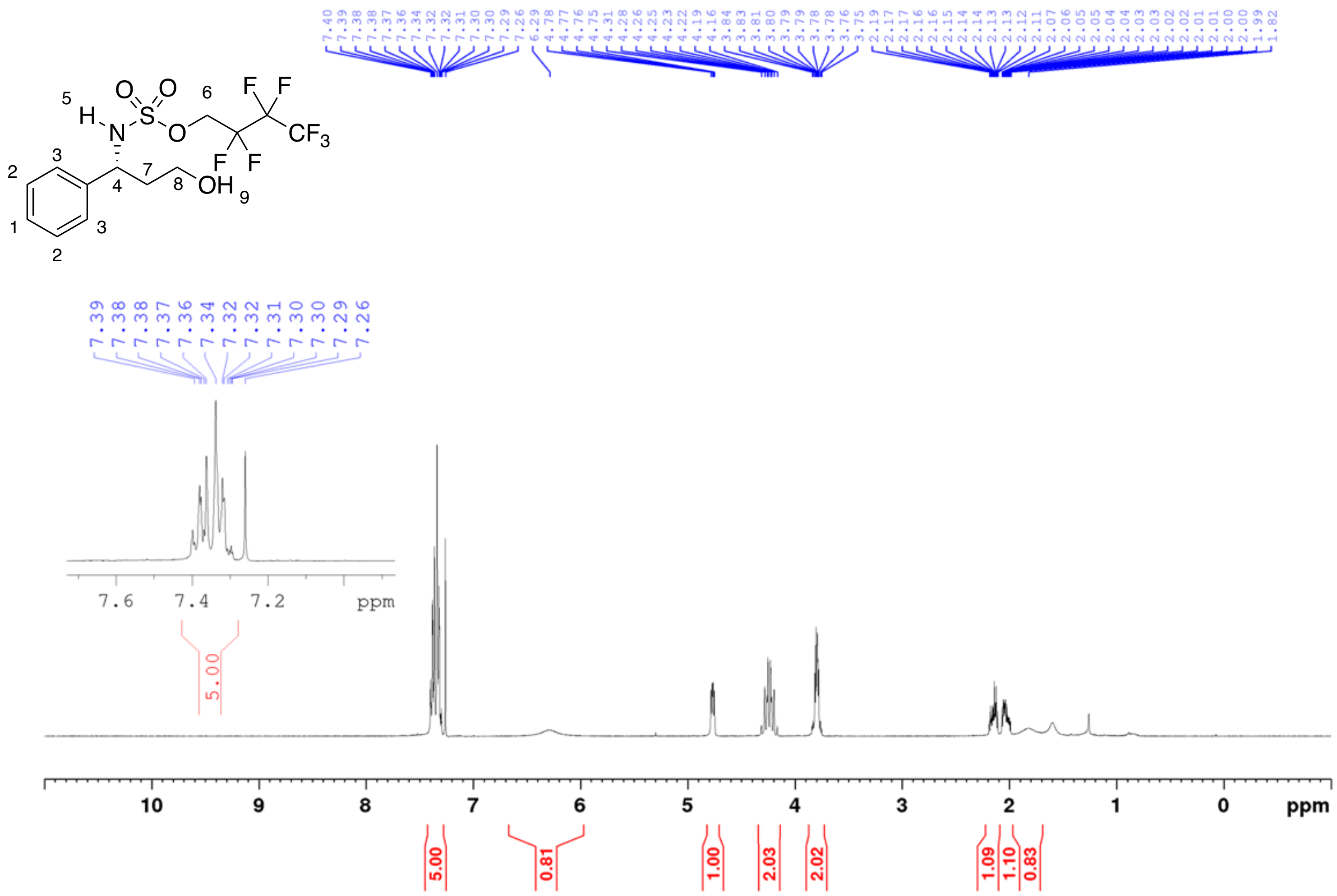

^{13}C NMR (101 MHz, $CDCl_3$) for *2,2,3,3,4,4,4-heptafluorobutyl (R)-(3-hydroxy-1-phenylpropyl)sulfamate* (**14**)

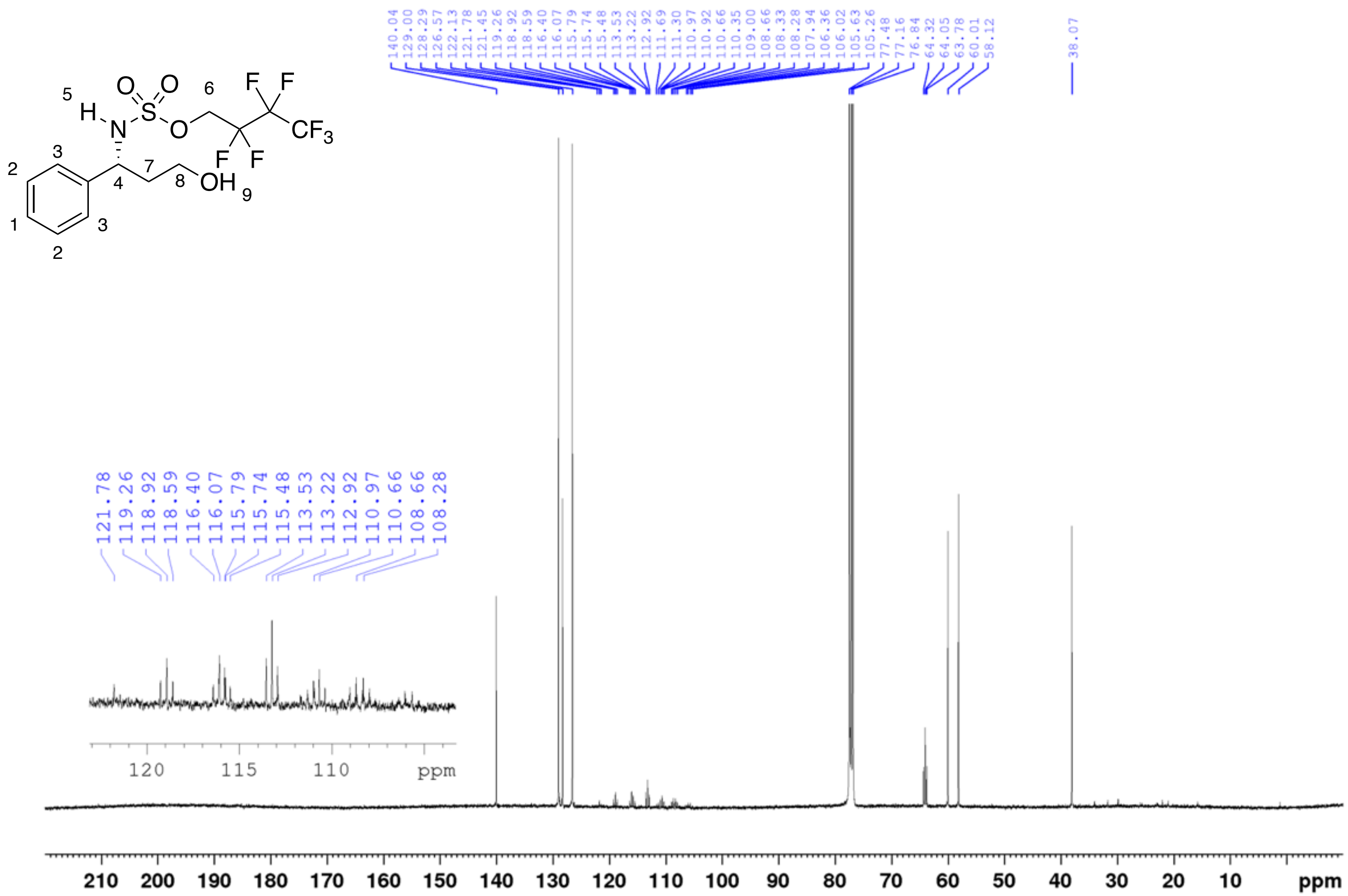

^{19}F NMR (376 MHz, $CDCl_3$) for *2,2,3,3,4,4,4-heptafluorobutyl (R)-(3-hydroxy-1-phenylpropyl)sulfamate* (**14**)

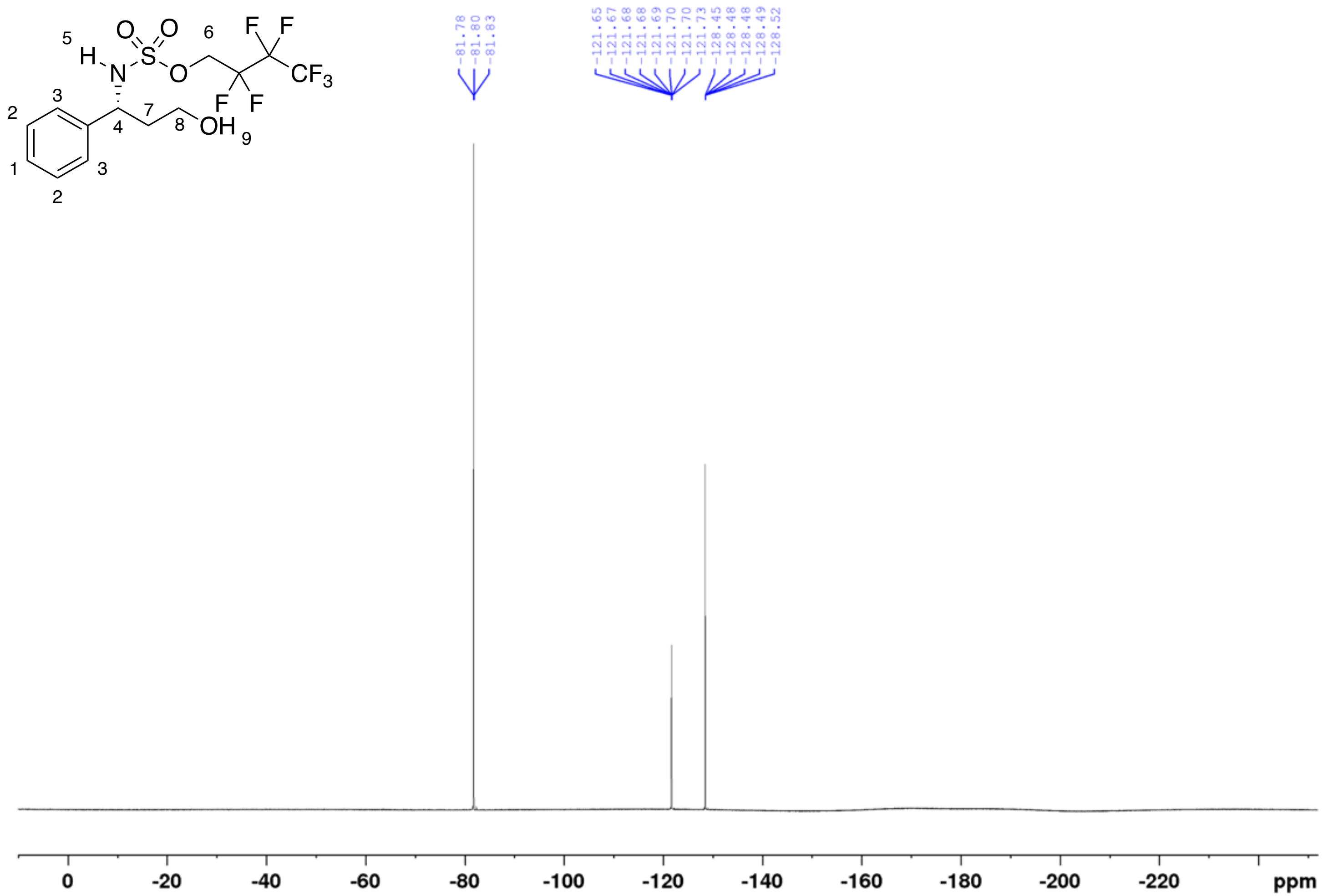

^{1}H NMR (500 MHz, $CDCl_3$) for *2,2,3,3,4,4,4-Heptafluorobutyl (5-hydroxy-1-phenylpentyl)sulfamate* (**15**)

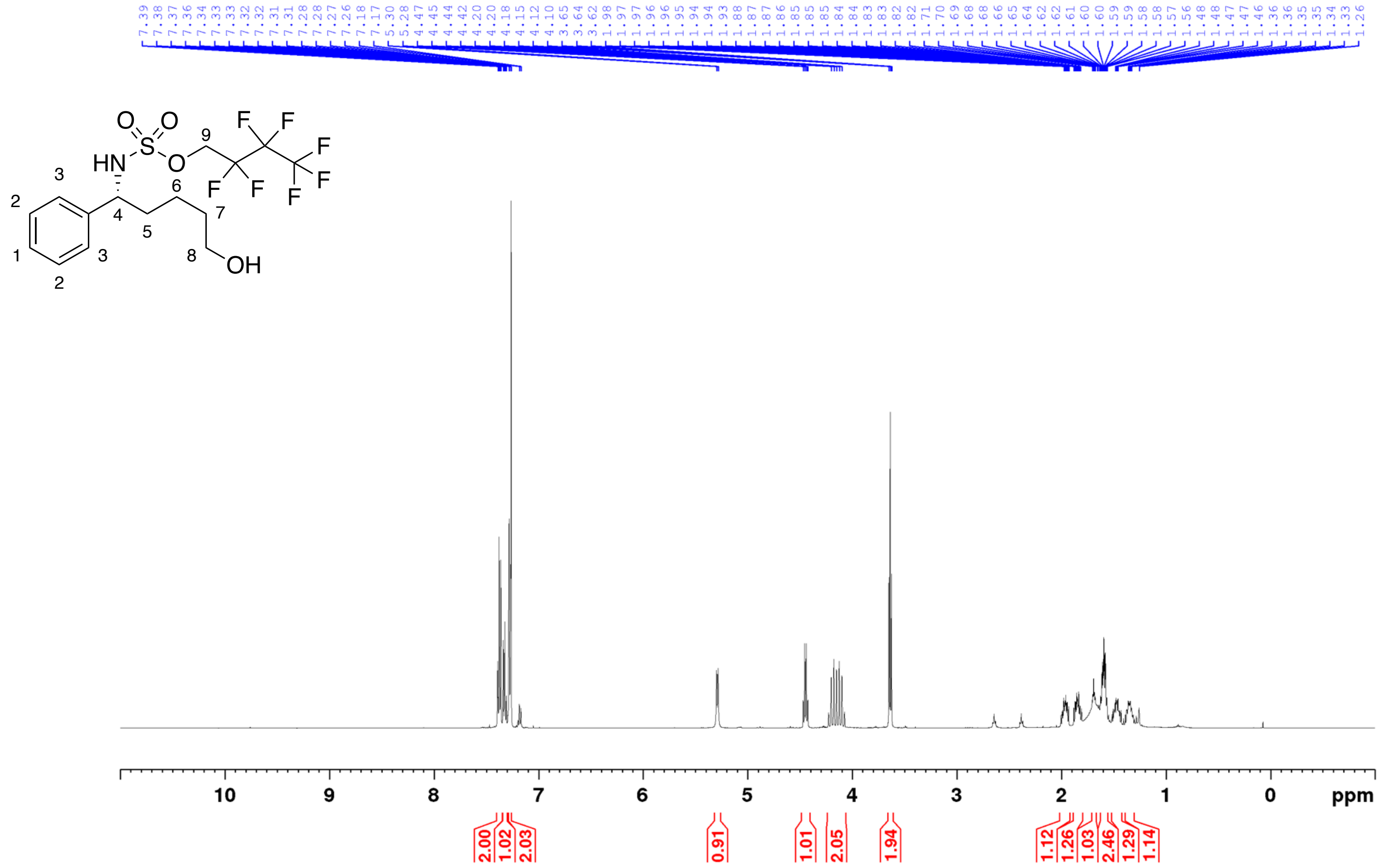

^{13}C NMR (126 MHz, $CDCl_3$) for *2,2,3,3,4,4,4-Heptafluorobutyl (5-hydroxy-1-phenylpentyl)sulfamate* (**15**)

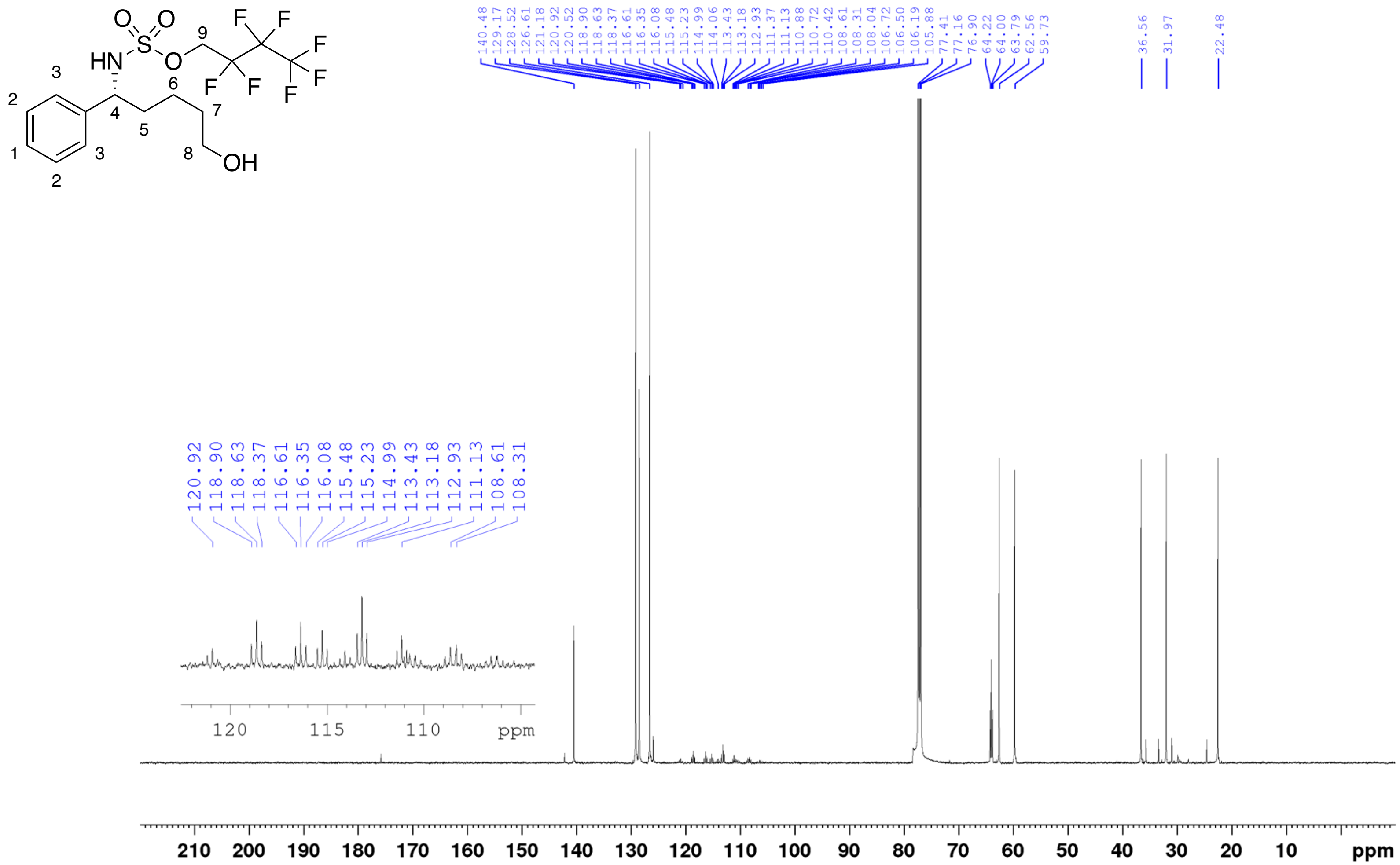

^{19}F NMR (376 MHz, $CDCl_3$) for *2,2,3,3,4,4,4-Heptafluorobutyl (5-hydroxy-1-phenylpentyl)sulfamate* (**15**)

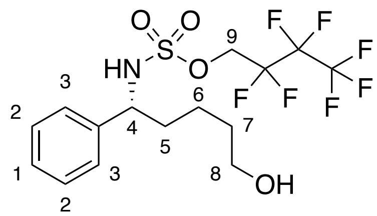

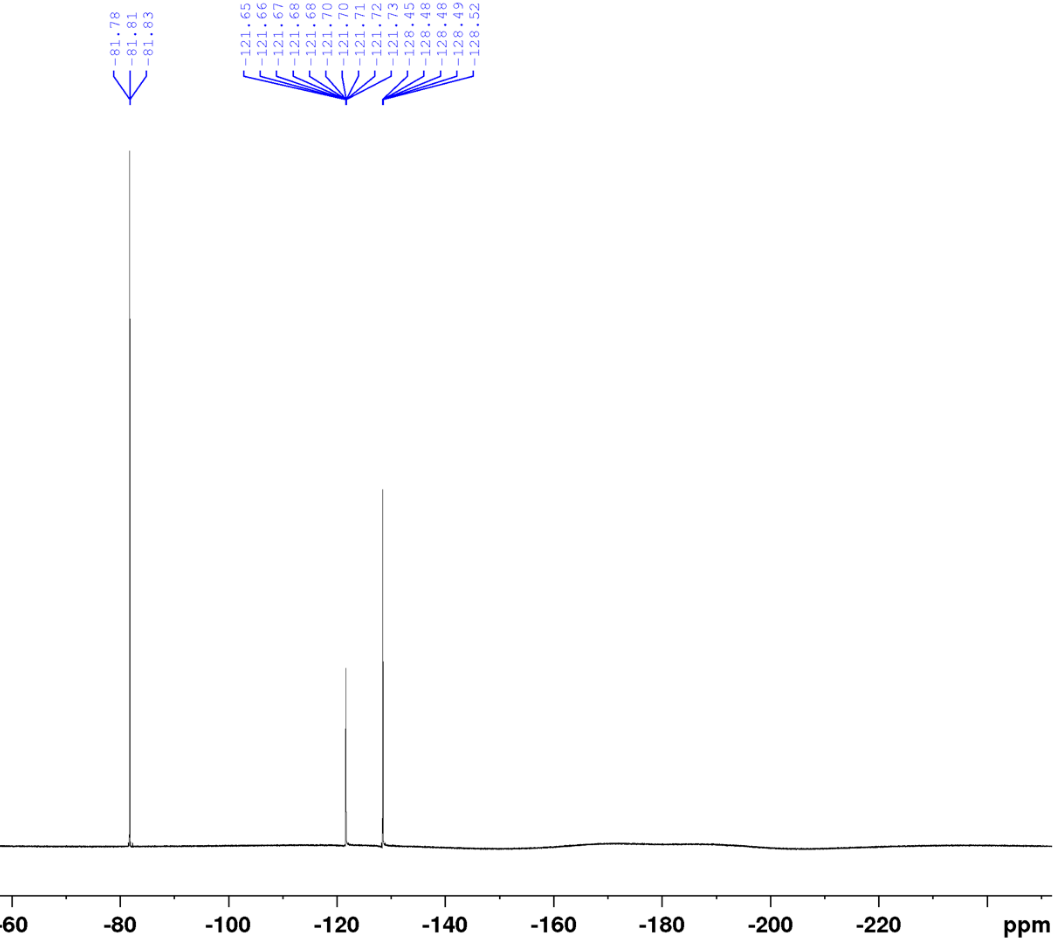

^{1}H NMR (500 MHz, $CDCl_3$) for *2,2,3,3,4,4,4-heptafluorobutyl (4-acetamido-1-phenylbutyl)sulfamate*

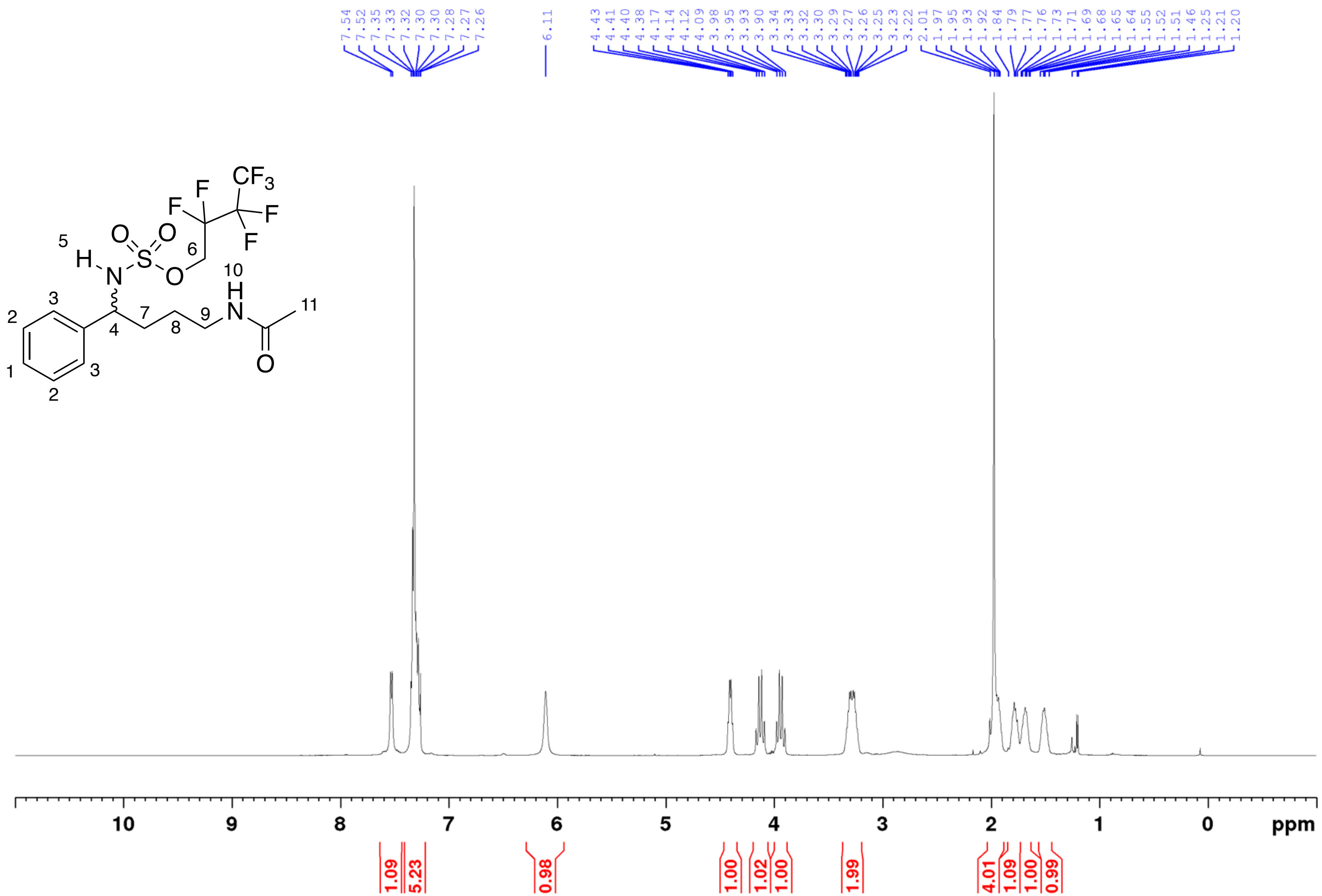

^{13}C NMR (126 MHz, $CDCl_3$) for *2,2,3,3,4,4,4-heptafluorobutyl (4-acetamido-1-phenylbutyl)sulfamate*

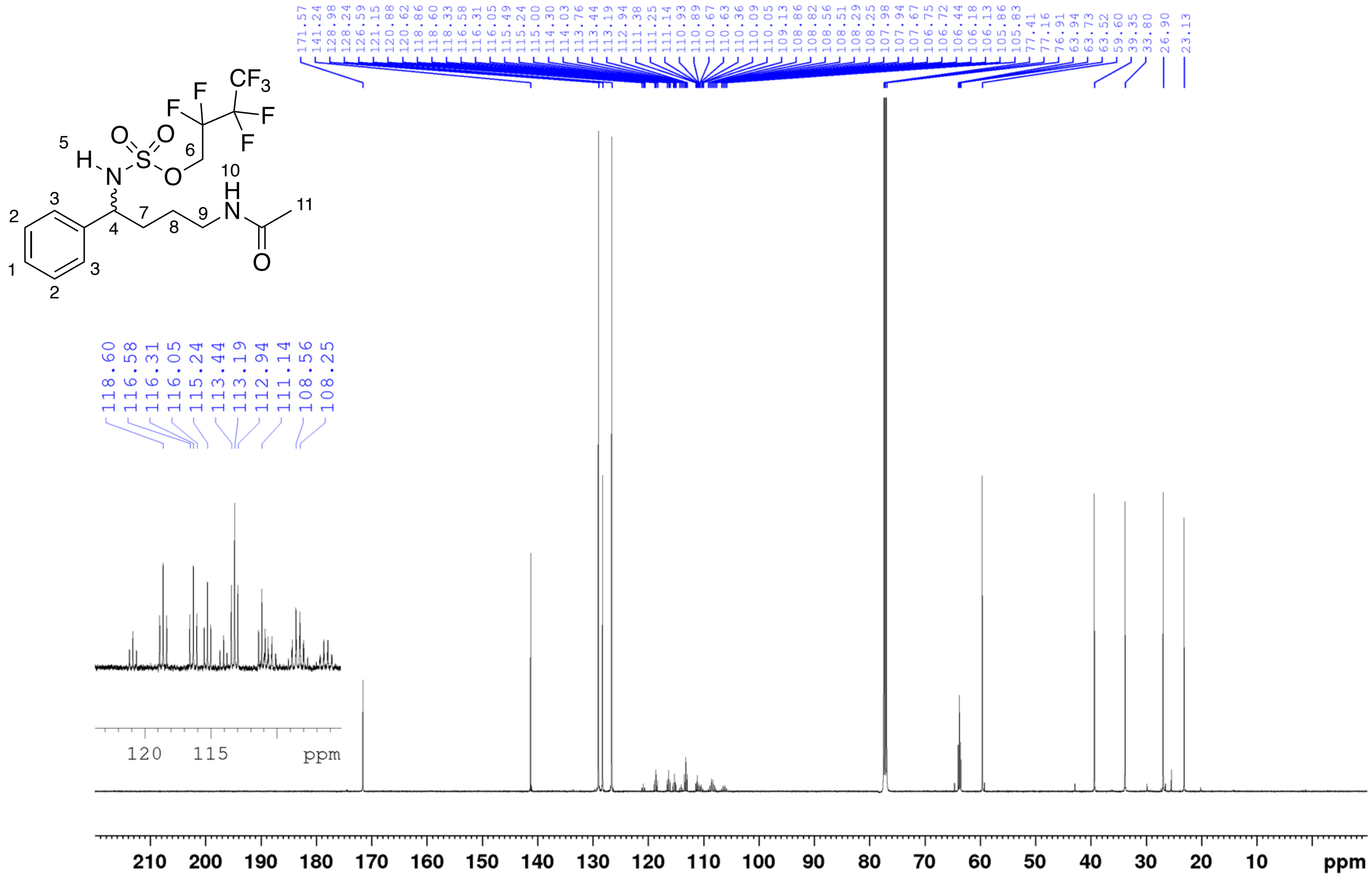

^{19}F NMR (376 MHz, $CDCl_3$) for *2,2,3,3,4,4,4-heptafluorobutyl (4-acetamido-1-phenylbutyl)sulfamate*

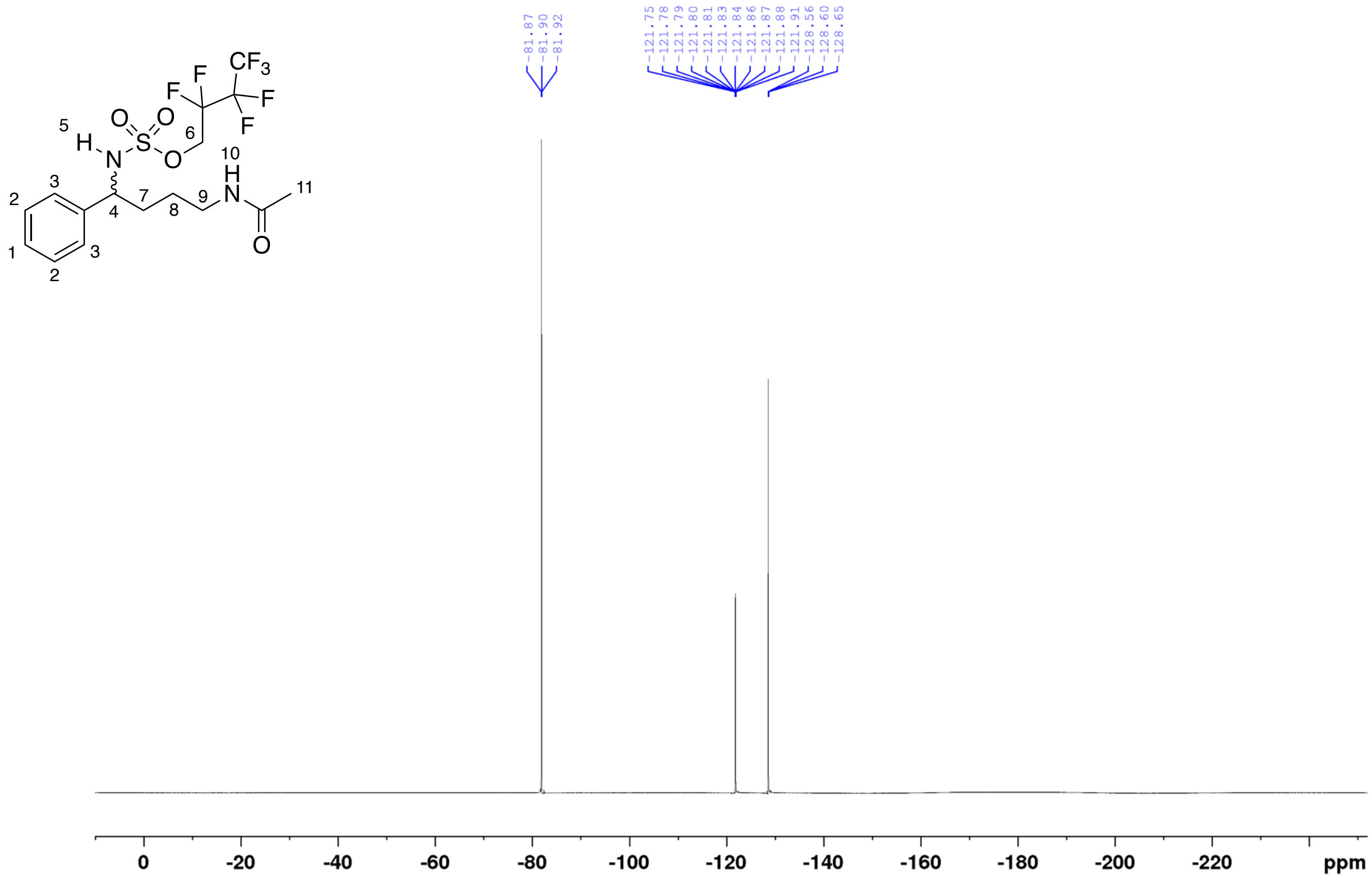

References

(1) Kauffman, G. B.; Fang, L. Y. Transition Metal Complexes and Compounds. In *Inorganic Syntheses*; John Wiley & Sons, Inc.: Toronto, Canada, 1983; Vol. 22, pp 101–103.

(2) Li, K.; Weber, A. E.; Tseng, L.; Malcolmson, S. J. Diastereoselective and Enantiospecific Synthesis of 1,3-Diamines via 2-Azaallyl Anion Benzylic Ring-Opening of Aziridines. *Org. Lett.* **2017**, *19*, 4239–4242.

(3) Williams, C. M.; Mander, L. N. Chromatography with Silver Nitrate. *Tetrahedron* **2001**, *57*, 425–447.

(4) Mander, L. N.; Williams, C. M. Chromatography with Silver Nitrate: Part 2. *Tetrahedron* **2016**, *72*, 1133–1150.

(5) Gottlieb, H. E.; Kotlyar, V.; Nudelman, A. NMR Chemical Shifts of Common Laboratory Solvents as Trace Impurities. *J. Org. Chem.* **1997**, *62*, 7512–7515.

(6) Budvari, S.; O'Neil, M. J.; Smith, A.; Heckelman, P. E. *The Merck Index, an Encyclopedia of Chemicals, Drugs, and Biologicals*, 11th ed.; Merck Co., Inc. Rahway: NJ, 1989.

(7) Piotto, M.; Bourdonneau, M.; Elbayed, K.; Wieruszeski, J.-M.; Lippens, G. New DEFT Sequences for the Acquisition of One-Dimensional Carbon NMR Spectra of Small Unlabelled Molecules. *Magn. Reson. Chem.* **2006**, *44*, 943–947.

(8) Chiappini, N. D.; Mack, J. B. C.; Du Bois, J. Intermolecular C(Sp3)−H Amination of Complex Molecules. *Angew. Chem.* **2018**, *130*, 5050–5053.

(9) Genov, G. R.; Douthwaite, J. L.; Lahdenperä, A. S. K.; Gibson, D. C.; Phipps, R. J. Enantioselective Remote C–H Activation Directed by a Chiral Cation. *Science* **2020**, *367*, 1246–1251.

(10) Lee, S. J.; Terrazas, M. S.; Pippel, D. J.; Beak, P. Mechanism of Electrophilic Chlorination: Experimental Determination of a Geometrical Requirement for Chlorine Transfer by the Endocyclic Restriction Test. *J. Am. Chem. Soc.* **2003**, *125*, 7307–7312.

(11) Liu, R.; Lu, Z.-H.; Hu, X.-H.; Li, J.-L.; Yang, X.-J. Monocarboxylation and Intramolecular Coupling of Butenylated Arenes via Palladium-Catalyzed C–H Activation Process. *Org. Lett.* **2015**, *17*, 1489–1492.

(12) Peters, M.; Trobe, M.; Tan, H.; Kleineweischede, R.; Breinbauer, R. A Modular Synthesis of Teraryl-Based α-Helix Mimetics, Part 1: Synthesis of Core Fragments with

Two Electronically Differentiated Leaving Groups. *Chem. – Eur. J.* **2013**, *19*, 2442–2449.

(13) Wang, L.; Jacobson, O.; Avdic, D.; Rotstein, B. H.; Weiss, I. D.; Collier, L.; Chen, X.; Vasdev, N.; Liang, S. H. Ortho-Stabilized 18F-Azido Click Agents and Their Application in PET Imaging with Single-Stranded DNA Aptamers. *Angew. Chem. Int. Ed.* **2015**, *54*, 12777–12781.

(14) Davis, H. J.; Mihai, M. T.; Phipps, R. J. Ion Pair-Directed Regiocontrol in Transition-Metal Catalysis: A Meta-Selective C–H Borylation of Aromatic Quaternary Ammonium Salts. *J. Am. Chem. Soc.* **2016**, *138*, 12759–12762.

(15) Sang, R.; Kucmierczyk, P.; Dong, K.; Franke, R.; Neumann, H.; Jackstell, R.; Beller, M. Palladium-Catalyzed Selective Generation of CO from Formic Acid for Carbonylation of Alkenes. *J. Am. Chem. Soc.* **2018**, *140*, 5217–5223.

(16) Wang, Y.; Ren, W.; Shi, Y. An Atom-Economic Approach to Carboxylic Acids via Pd-Catalyzed Direct Addition of Formic Acid to Olefins with Acetic Anhydride as a Co-Catalyst. *Org. Biomol. Chem.* **2015**, *13*, 8416–8419.

(17) Dong, K.; Sang, R.; Liu, J.; Razzaq, R.; Franke, R.; Jackstell, R.; Beller, M. Palladium-Catalyzed Carbonylation of Sec- and Tert-Alcohols. *Angew. Chem. Int. Ed.* **2017**, *56*, 6203–6207.

(18) Bag, S.; Jayarajan, R.; Mondal, R.; Maiti, D. Template-Assisted Meta-C−H Alkylation and Alkenylation of Arenes. *Angew. Chem. Int. Ed.* **2017**, *56*, 3182–3186.

(19) Che, Z.; Yang, J.; Sun, D.; Tian, Y.; Liu, S.; Lin, X.; Jiang, J.; Chen, G. Synthesis of Novel (9S)-Acyloxy Derivatives of Quinidine and Dihydroquinidine as Insecticidal Agents. *Chem. Biodivers.* **2020**, *17*, e1900696.

(20) Dötz, F.; Brand, J. D.; Ito, S.; Gherghel, L.; Müllen, K. Synthesis of Large Polycyclic Aromatic Hydrocarbons: Variation of Size and Periphery. *J. Am. Chem. Soc.* **2000**, *122*, 7707–7717.

(21) Zielińska-Błajet, M.; Kucharska, M.; Skarżewski, J. Simple Enantiospecific Synthesis of Sulfides of Cinchona Alkaloids. *Synthesis* **2006**, *2006*, 1176–1182.

(22) Moreland, C. G.; Philip, A.; Carroll, F. I. Carbon-13 Nuclear Magnetic Resonance Spectra of Cinchona Alkaloids. *J. Org. Chem.* **1974**, *39*, 2413–2416.

(23) Dijkstra, G. D. H.; Kellogg, R. M.; Wynberg, H.; Svendsen, J. S.; Marko, I.; Sharpless, K. B. Conformational Study of Cinchona Alkaloids. A Combined NMR, Molecular Mechanics and x-Ray Approach. *J. Am. Chem. Soc.* **1989**, *111*, 8069–8076.

(24) Breman, A. C.; van der Heijden, G.; van Maarseveen, J. H.; Ingemann, S.; Hiemstra, H. Synthetic and Organocatalytic Studies of Quinidine Analogues with Ring-Size Modifications in the Quinuclidine Moiety. *Chem. – Eur. J.* **2016**, *22*, 14247–14256.

(25) Hu, B.; Bezpalko, M. W.; Fei, C.; Dickie, D. A.; Foxman, B. M.; Deng, L. Origin of and a Solution for Uneven Efficiency by Cinchona Alkaloid-Derived, Pseudoenantiomeric Catalysts for Asymmetric Reactions. *J. Am. Chem. Soc.* **2018**, *140*, 13913–13920.

(26) Bess, E. N.; DeLuca, R. J.; Tindall, D. J.; Oderinde, M. S.; Roizen, J. L.; Du Bois, J.; Sigman, M. S. Analyzing Site Selectivity in Rh2(Esp)2-Catalyzed Intermolecular C–H Amination Reactions. *J. Am. Chem. Soc.* **2014**, *136*, 5783–5789.

(27) Kumar, S.; Patel, M.; Saunthwal, R. K.; Verma, A. K. Chemoselective Oxidative Esterification and Iodocyclization of Hydroxyalkynyl Aldehydes. *Asian J. Org. Chem.* **2017**, *6*, 1893–1902.

(28) Denmark, S. E.; Edwards, M. G. On the Mechanism of the Selenolactonization Reaction with Selenenyl Halides. *J. Org. Chem.* **2006**, *71*, 7293–7306.

(29) Novák, Z.; Szabó, A.; Répási, J.; Kotschy, A. Sonogashira Coupling of Aryl Halides Catalyzed by Palladium on Charcoal. *J. Org. Chem.* **2003**, *68*, 3327–3329.

(30) Fyfe, T. J.; Kellam, B.; Sykes, D. A.; Capuano, B.; Scammells, P. J.; Lane, J. R.; Charlton, S. J.; Mistry, S. N. Structure–Kinetic Profiling of Haloperidol Analogues at the Human Dopamine D2 Receptor. *J. Med. Chem.* **2019**, *62*, 9488–9520.

(31) Dutta, S.; Lopez Charcas, O.; Tanner, S.; Gradek, F.; Driffort, V.; Roger, S.; Selander, K.; Velu, S. E.; Brouillette, W. Discovery and Evaluation of NNav1.5 Sodium Channel Blockers with Potent Cell Invasion Inhibitory Activity in Breast Cancer Cells. *Bioorg. Med. Chem.* **2018**, *26*, 2428–2436.

(32) Shaalan, Y.; Boulton, L.; Jamieson, C. Ruthenium-Catalyzed Ester Reductions Applied to Pharmaceutical Intermediates. *Org. Process Res. Dev.* **2020**, *24*, 2745–2751.

(33) Costello, J. P.; Ferreira, E. M. Regioselectivity Influences in Platinum-Catalyzed Intramolecular Alkyne O–H and N–H Additions. *Org. Lett.* **2019**, *21*, 9934–9939.

(34) Das, R.; Chakraborty, D. Silver Triflate Catalyzed Acetylation of Alcohols, Thiols, Phenols, and Amines. *Synthesis* **2011**, *2011*, 1621–1625.

(35) Kuwabe, S.; Torraca, K. E.; Buchwald, S. L. Palladium-Catalyzed Intramolecular C–O Bond Formation. *J. Am. Chem. Soc.* **2001**, *123*, 12202–12206.

(36) Baciocchi, E.; Crescenzi, M. Selectivity and Mechanism in the Side-Chain Halogenation of Methylbenzenes Promoted Photochemically and by Metal Complexes in the Presence of Halide Ions. *Tetrahedron* **1988**, *44*, 6525–6536.

(37) Mitchell, R. H.; Lai, Y. H. Syntheses and Reactions of the First Dithia[3.1.3.1]Metacyclophanes, [2.1.2.1]Metacyclophanes, and [2.1.2.1]Metacyclophanedienes. *J. Org. Chem.* **1984**, *49*, 2534–2540.

(38) Yus, M.; Herrera, R. P.; Guijarro, A. On the Mechanism of Arene-Catalyzed Lithiation: The Role of Arene Dianions—Naphthalene Radical Anion versus Naphthalene Dianion. *Chem. – Eur. J.* **2002**, *8*, 2574–2584.

(39) Bartoli, G.; Cipolletti, R.; Antonio, G. D.; Giovannini, R.; Lanari, S.; Marcolini, M.; Marcantoni, E. A Convergent Approach to (R)-Tiagabine by a Regio- and Stereocontrolled Hydroiodination of Alkynes. *Org. Biomol. Chem.* **2010**, *8*, 3509–3517.

(40) Häring, A. P.; Biallas, P.; Kirsch, S. F. An Unconventional Reaction of 2,2-Diazido Acylacetates with Amines. *Eur. J. Org. Chem.* **2017**, *2017*, 1526–1539.

(41) Hamilton, G. S.; Wu, Y.-Q.; Limburg, D. C.; Wilkinson, D. E.; Vaal, M. J.; Li, J.-H.; Thomas, C.; Huang, W.; Sauer, H.; Ross, D. T.; Soni, R.; Chen, Y.; Guo, H.; Howorth, P.; Valentine, H.; Liang, S.; Spicer, D.; Fuller, M.; Steiner, J. P. Synthesis of N-Glyoxyl Prolyl and Pipecolyl Amides and Thioesters and Evaluation of Their In Vitro and In Vivo Nerve Regenerative Effects. *J. Med. Chem.* **2002**, *45*, 3549–3557.

(42) Schoenauer, S.; Buergy, A.; Kreissl, J.; Schieberle, P. Structure/Odor Activity Studies on Aromatic Mercaptans and Their Cyclohexane Analogues Synthesized by Changing the Structural Motifs of Naturally Occurring Phenyl Alkanethiols. *J. Agric. Food Chem.* **2019**, *67*, 2598–2606.

(43) Tejo, C.; Anders See, Y. F.; Mathiew, M.; Hong Chan, P. W. Synthesis of 1,4-Amino Alcohols by Grignard Reagent Addition to THF and N -Tosyliminobenzyliodinane. *Org. Biomol. Chem.* **2016**, *14*, 844–848.

(44) Węglarz, I.; Michalak, K.; Mlynarski, J. Zinc-Catalyzed Asymmetric Hydrosilylation of Cyclic Imines: Synthesis of Chiral 2-Aryl-Substituted Pyrrolidines as Pharmaceutical Building Blocks. *Adv. Synth. Catal.* **2021**, *363*, 1317–1321.

(45) Berry, J. F. The Role of Three-Center/Four-Electron Bonds in Superelectrophilic Dirhodium Carbene and Nitrene Catalytic Intermediates. *Dalton Trans.* **2011**, *41*, 700–713.

(46) Warzecha, E.; Berto, T. C.; Berry, J. F. Axial Ligand Coordination to the C–H Amination Catalyst Rh2(Esp)2: A Structural and Spectroscopic Study. *Inorg. Chem.* **2015**, *54*, 8817–8824.

(47) Warzecha, E.; Berto, T. C.; Wilkinson, C. C.; Berry, J. F. Rhodium Rainbow: A Colorful Laboratory Experiment Highlighting Ligand Field Effects of Dirhodium Tetraacetate. *J. Chem. Educ.* **2019**, *96*, 571–576.
